# Reversible, interrelated mRNA and miRNA expression patterns in the transcriptome of Rasless fibroblasts: functional and mechanistic implications

**DOI:** 10.1186/1471-2164-14-731

**Published:** 2013-10-25

**Authors:** Sami S Azrak, Alicia Ginel-Picardo, Matthias Drosten, Mariano Barbacid, Eugenio Santos

**Affiliations:** 1 Centro de Investigacion del Cancer, IBMCC (CSIC-USAL), University of Salamanca, Campus Unamuno, 37007, Salamanca, Spain; 2 CNIO, ISCIII, 28029, Madrid, Spain

**Keywords:** Ras, Cell cycle, Transcriptome, mRNA, miRNA, Differential gene expression, Transcriptional responses, HRAS, NRAS, KRAS

## Abstract

**Background:**

4-Hydroxy-tamoxifen (4OHT) triggers Cre-mediated K-Ras removal in [H-Ras^-/-^;N-Ras^-/-^;K-Ras^lox/lox^;RERT^ert/ert^] fibroblasts, generating growth-arrested “Rasless” MEFs which are able to recover their proliferative ability after ectopic expression of Ras oncoproteins or constitutively active BRAF or MEK1.

**Results:**

Comparison of the transcriptional profiles of Rasless fibroblasts with those of MEFs lacking only H-Ras and N-Ras identified a series of differentially expressed mRNAs and microRNAs specifically linked to the disappearance of K-Ras from these cells. The rescue of cell cycle progression in Rasless cells by activated BRAF or MEK1 resulted in the reversal of most such transcriptional mRNA and microRNA alterations.

Functional analysis of the differentially expressed mRNAs uncovered a significant enrichment in the components of pathways regulating cell division, DNA/RNA processing and response to DNA damage. Consistent with G1/S blockade, Rasless cells displayed repression of a series of cell cycle-related genes, including Cyclins, Cyclin-dependent kinases, Myc and E2F transcription targets, and upregulation of Cyclin-dependent kinase inhibitors. The profile of differentially expressed microRNAs included a specific set of oncomiR families and clusters (repressed miR-17 ~ 92, miR-106a ~ 363, miR-106b ~ 25, miR-212 ~ 132, miR-183 ~ 182, and upregulated miR-335) known for their ability to target a specific set of cellular regulators and checkpoint sensors (including Rb, E2F and Cdkns) able to modulate the interplay between the pro- and anti-proliferative or stress-response pathways that are reversibly altered in Rasless cells.

**Conclusions:**

Our data suggest that the reversible proliferation phenotype of Rasless cells is the pleiotropic result of interplay among distinct pro- and anti-proliferative, and stress-response pathways modulated by a regulatory circuitry constituted by a specific set of differentially expressed mRNAs and microRNAs and preferentially targeting two cross-talking signalling axes: Myc-Rb-E2F-dependent and Cdkns-p53-dependent pathways.

## Background

The 3 canonical members of the mammalian Ras gene family (*H-ras*, *N-ras*, and *K-ras*) code for four distinct protein isoforms (H-Ras, N-Ras, K-Ras4A and K-Ras4B) which cycle continuously between active (GTP-bound) and inactive (GDP-bound) conformations in a process regulated by their functional interactions with negative (GAP) and positive (GEF) cellular regulators. Mammalian Ras genes and proteins are widely conserved across species and are almost ubiquitously expressed in different cell lineages and organs, and they play significant roles in many cellular processes, including proliferation, differentiation and cell death [1-6]. The wealth of Ras activators and effectors identified in mammalian cells places the Ras proteins at the center of multiple signaling networks critical for normal cellular development and homeostasis and for pathological processes such as cancer [1-4,7-9].

Despite earlier preconceived views of functional redundancy, most of the experimental evidence supports the notion of functional specificity for each Ras family member. Indeed, the high conservation across mammalian species of the specific amino acid sequence of each Ras isoform at its C-terminal hypervariable (HVR) region, the distinctive patterns of expression, intracellular processing and subcellular location displayed by the fully processed protein products of the different *ras* gene isoforms, and the prevalent presence of specific *ras* oncogenes in particular types of human tumors are indicative of such functional specificity [1,2,7-12].

Genomic disruption of K-*ras* 4B causes embryonic lethality, whereas H-*ras*, N-*ras* and K-*ras*4A single-knockout (KO) mice are perfectly viable and fertile, and simultaneous removal of H-*ras* and N-*ras* also results in viable mice with no evident phenotypic abnormalities [13-17]. Joint analysis of the different Ras KO animal models available indicates that only K-*ras*4B is necessary and sufficient for full embryonic development and suggests that K-Ras performs specific function(s) that cannot be carried out by either H-Ras or N-Ras. An alternative explanation [18] suggests that the mortality of K-Ras KO animals might not derive from the intrinsic inability of the other isoforms to substitute for K-Ras function but rather from their inability to be expressed in the same cell types or developmental stages as K-Ras. Further insight into the functional relationships among the three different Ras isoforms is now possible through the analysis of mouse strains that can be rendered “Rasless” because they harbor constitutive null H-*ras* and N-*ras* alleles together with a conditionally floxed K-*ras* locus [19].

The functional specificity of individual Ras isoforms is also supported by their demonstrated ability to drive specific transcriptional programs and generate distinct genomic expression signatures in the particular cell lineages where they are expressed [19-26]. Thus, our characterization of the transcriptional networks of fibroblasts harboring single or double null mutations in the H-*ras* and/or N-*ras* loci has shown that these two isoforms control different, rather antagonistic transcriptional profiles, supporting the notion of different functional roles for H-Ras and N-Ras in these cells, with a preferential involvement of H-Ras in processes of cell growth and proliferation and N-Ras in control of immune modulation/host defense and apoptotic responses [20,21].

The analysis of Ras KO cell lines has also contributed to a better understanding of the participation of different Ras isoforms in control of the cell cycle [27-29]. Our study of the transcriptional profiles of cells lacking H-*ras* and N-*ras,* either alone or in combination, during the early stages of the cell cycle [21] suggested a preferential involvement of N-Ras in immediate-early cellular responses to serum stimulation, and of H-Ras in cellular responses related to growth and proliferation during mid-G1 progression [20,21]. Also, the characterization of triple KO Rasless MEFs [19] has further confirmed the critical requirement of Ras proteins for cell cycle progression by showing the inability of Rasless cells to inactivate Rb pocket proteins [30], suggesting that in contrast to current hypotheses Ras signaling does not induce proliferation by inducing expression of D-type cyclins [19]. Since the exact mechanisms underlying the participation of Ras proteins in cell cycle activation and progression are still largely undefined, further studies are needed to determine whether the different Ras isoforms play specific or redundant functional roles in those processes.

In this report, we describe a detailed characterization of the transcriptional networks of mRNA and microRNA that are specifically associated with the generation and reversal of the Rasless phenotype. Our analysis shows that the patterns of differential mRNA and miRNA expression in growth-arrested, Rasless cells are clearly interdependent and, in addition, that they can undergo specific reversal after recovery of the proliferative ability of such cells through the introduction of activated BRAF or MEK1 kinases. Functional analysis of the reversible mRNA and miRNA profiles identified a cell cycle regulatory circuitry focused on the preferential targeting of Myc-Rb-E2F-dependent and Cdkns-p53-dependent signalling pathways.

## Results and discussion

### Microarray analysis of transcriptomic profiles in Rasless fibroblasts

**Figure 1:**
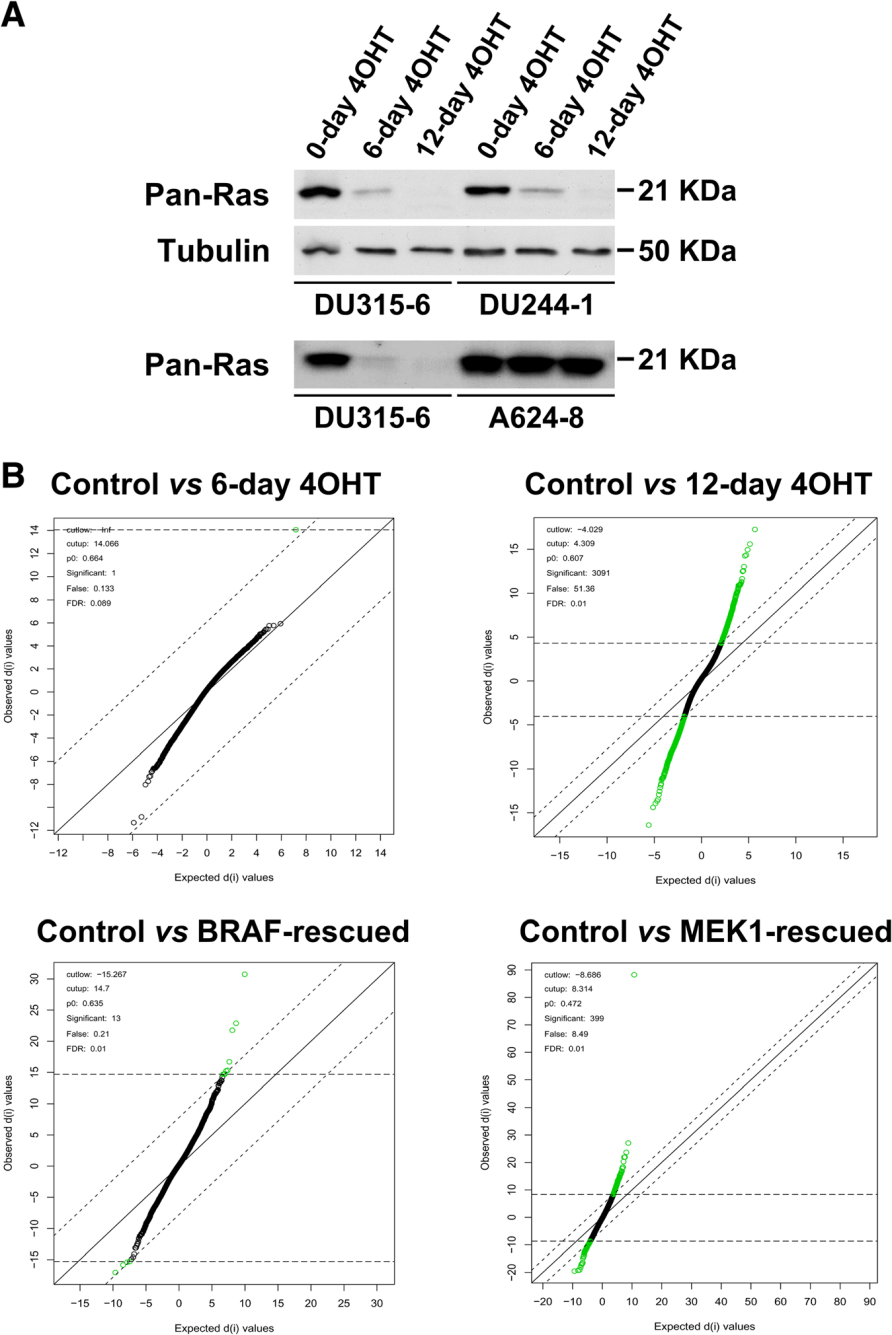
**Characterization and differential gene expression analysis of Rasless MEFs. (A)** Ras protein expression levels in MEFs treated with 4-hydroxy-tamoxifen (4OHT). Western immunoblots showing Ras protein levels after treatment with 4-hydroxy-tamoxifen (4OHT). Pan-Ras immunoblots showing the decrease in K-Ras protein expression after 4OHT treatment for 6 or 12 days of two different cell lines, DU315-6 and DU244-1. As control, 4OHT had no effect on K-Ras protein level in a representative, constitutive double knockout (H-Ras^-/-^;N-Ras^-/-^) MEF cell line A624-8 [21]. **(B)** Differential gene expression in Rasless MEFs as determined by microarray hybridizations. The Statistical Analysis of Microarray (SAM) algorithm [109] was used to identify differentially expressed probesets by comparing the microarray-generated transcriptional profiles of K-Ras^lox^ cell lines treated with 4OHT for 6 days (6-day 4OHT, upper left panel) or for 12 days (12-day 4OHT Rasless, upper right panel) with those of control, untreated K-Ras^lox^ MEFs. Only 1 differentially expressed probeset could be identified in 6-day 4OHT samples using a lax False Discovery Rate (FDR) value of 0.089 (upper left panel). In contrast, the SAM plot for the 12-day 4OHT Rasless samples allowed the identification of 3091 differentially expressed probesets using a highly stringent FDR value of 0.01 (upper right panel). Comparison of the transcriptional profile of control K-Ras^lox^ MEFs with those of the BRAF-rescued (lower left panel) or the MEK1-rescued (lower right panel) cells using the same FDR value of 0.01 showed that the expression of transfected BRAF or MEK1 resulted in reversal of the majority of transcriptional alterations observed in Rasless MEFs generated after 12-day 4OHT treatment since only 13 or 399 differentially expressed probesets could be identified respectively.

“Rasless” cells lacking expression of the three canonical *ras* genes can be generated by 4-hydroxy-tamoxifen (4OHT) treatment of immortalized mouse embryo fibroblasts (MEF) derived from a mouse strain harboring constitutive homozygous null mutations of the H-ras and N-ras loci as well as an inducible null mutation of the K-ras locus (H-ras^-/-^;N-ras^-/-^;K-ras^lox/lox^;RERT^ert/ert^) [19]. Under our experimental conditions, treatment of the MEF cultures with 4OHT for 6 days produced a significant decrease in the amount of detectable K-Ras protein, whereas a 12-day treatment resulted in complete absence of any detectable Ras protein in the cells (Figure 1A). The elimination of K-Ras expression was not a non-specific off-target effect of the 4OHT treatment but rather a specific result of the activation of the resident Cre-ERT2 recombinase by this compound. Thus, 4OHT treatment of K-Ras-expressing, constitutive double KO (H-ras^-/-^;N-ras^-/-^) A624-8 cells [21] did not elicit any changes in the total amount of Ras protein detectable with specific antibodies (Figure 1A). As previously described [19], the Rasless cells were unable to proliferate, but did recover their proliferative ability after ectopic expression of transfected constructs coding for constitutively active downstream kinases of the Ras-MAPK pathway such as BRAF^CAAX^ and MEK1^Q56P^.

To determine whether the Rasless status might be linked to specific gene expression programs, we used commercial oligonucleotide microarrays to compare the transcriptional profiles of control, untreated immortalized fibroblast cultures derived from the KO mice to those of the same cells after 4OHT-induced removal of the conditional K-Ras^lox^ alleles. In addition, the transcriptomes of cells reversed to proliferate after the introduction of either BRAF^CAAX^ or MEK1^Q56P^ constructs [19] were also compared with that of growth-arrested, Rasless cells generated after a 12-day treatment with 4OHT. For this purpose, RNA extracted from pre-confluent cultures of the different sample groups was hybridized with high-density oligonucleotide microarrays. Affymetrix *Mouse Genome 430 2.0* Arrays were used to analyze mRNA expression patterns associated with the different experimental conditions analysed, whereas *Mouse GeneChip miRNA* arrays were used to characterize the patterns of microRNA expression under the same conditions. The different sets of experimental samples analyzed here included RNAs from (i) control proliferating cells (H-Ras^-/-^; N-Ras^-/-^; K-Ras^lox/lox^) expressing only K-Ras (designated K-Ras^lox^ from here on); (ii) the same cells after treatment with 4OHT for 6 days or 12 days to render them non-proliferating, Rasless fibroblasts (henceforth designated 6-day 4OHT and 12-day 4OHT Rasless); and (iii) proliferating Rasless cells harboring transfected, activated MEK1 or BRAF constructs after treatment with 4OHT for 12 days (henceforth designated MEK1-rescued or BRAF-rescued, respectively).

### Differential gene expression patterns in Rasless and BRAF- or MEK1-rescued MEFs

SAM pair-wise contrast analyses provided an initial overall view of the global mRNA transcriptional changes occurring in MEFs devoid of expression of the 3 canonical *ras* genes. Figure 1B shows that a 6-day 4OHT treatment of K-Ras^lox^ MEFs (devoid of H-Ras and N-Ras, but still keeping about 50% of the regular K-Ras dosage; Figure 1A) did not cause any significant modification in the overall transcriptional profile of these cells in comparison to untreated K-Ras^lox^ cells, as determined by a SAM contrast performed at a relatively high False Discovery Rate (FDR) value (0.089) (Figure 1B, upper left panel). In contrast, in Rasless MEFs resulting from treatment with 4OHT for 12 days, and therefore completely devoid of Ras protein (Figure 1A), up to 3091 differentially expressed probesets (corresponding to 2239 distinct, differentially expressed genes) could be identified, even using a much lower (0.01) FDR value (Figure 1B, upper right panel). A complete list of the differentially expressed probesets observed in the 12-day 4OHT Rasless cells is presented in Additional file 1: Table S1, where the parameters of statistical significance for the level of overexpression or repression of each probeset are also shown.

Interestingly, rescue of the proliferative ability of the Rasless cells by expressing activated BRAF or MEK1 [19] also reversed most of the transcriptional alterations previously identified as being associated with the absence of K-Ras. Figure 1B shows that, using a similar 0.01 FDR value for the SAM contrasts, the BRAF-rescued cells and the MEK1-rescued cells only show minor transcriptional changes in comparison with the original untreated proliferating K-Ras^lox^ MEFs controls (Figure 1B, lower panels).

These data indicate that the transcriptional networks integrating the set of differentially expressed genes identified in 12-day 4OHT-treated Rasless cells (Figure 1B) are specifically linked to the absence of K-Ras in those cells, thus representing a specific transcriptional signature of the Rasless status.

### Characterization and functional annotation of transcriptional networks in Rasless cells

A detailed list of differentially expressed loci resulting from a 12-day 4OHT treatment of K-Ras^lox^ MEFs to render them totally Rasless is shown in Additional file 1: Table S1. At a highly stringent FDR value of 0.01, 1101 probesets (861 distinct genes) were overexpressed, whereas 1990 probesets (1381 genes) were repressed. The bulk of overexpressed loci showed amplification levels lower than 5-fold, with about 27% of them included in the 2–5 fold range and fewer than 5% showing amplification levels higher than ten-fold (Additional file 1: Table S1). Among the genes showing high levels of R-fold overexpression, the extracellular matrix-related Prelp locus as well as cytoskeleton-related loci such as Mfap5, Fbn2 or Afap1l2 or loci related to immunity or inflammatory responses such as Wisp2, Vnn1 or Ly6a (= Sca1) and Ly6c1 can be mentioned (Additional file 1: Table S1). On the other hand, the majority of differentially expressed loci of Rasless cells (about 65% of the total number of genes listed in the table) showed reduced expression levels in comparison with control fibroblasts. Notably, the highest level of transcriptional repression was detected in Dusp6 (d-value = -16.4; R-fold = 0.09), a dual-specificity phosphatase acting in Ras signaling pathways. Interestingly, other members of the Dusp family (Dusp5, Dusp9 and Dusp4) were also significantly repressed in Rasless cells (Additional file 1: Table S1). The clear prevalence of transcriptional repression over induction in Rasless cells suggests a predominant functional contribution of the (missing) Ras proteins to mechanisms of positive modulation of transcription. Furthermore, as discussed later, most induced and repressed differentially expressed genes identified in Rasless cells showed an exactly opposite transcriptional behavior when examined in BRAF-rescued or MEK1-rescued cells (see Additional file 1: Table S1, column “*Expression reversed by*”).

Using Genecodis software, we searched for co-occurrence of functional annotations corresponding to GO biological processes or KEGG signaling pathways that could potentially be ascribed to specific subsets of the induced or repressed genes listed in Additional file 1: Table S1. This search yielded the identification of specific groups of downregulated (Additional file 2: Table S2) or upregulated (Additional file 3: Table S3) loci of Rasless MEFs that are related to specific biological processes at significantly high values of statistical significance.

**Figure 2:**
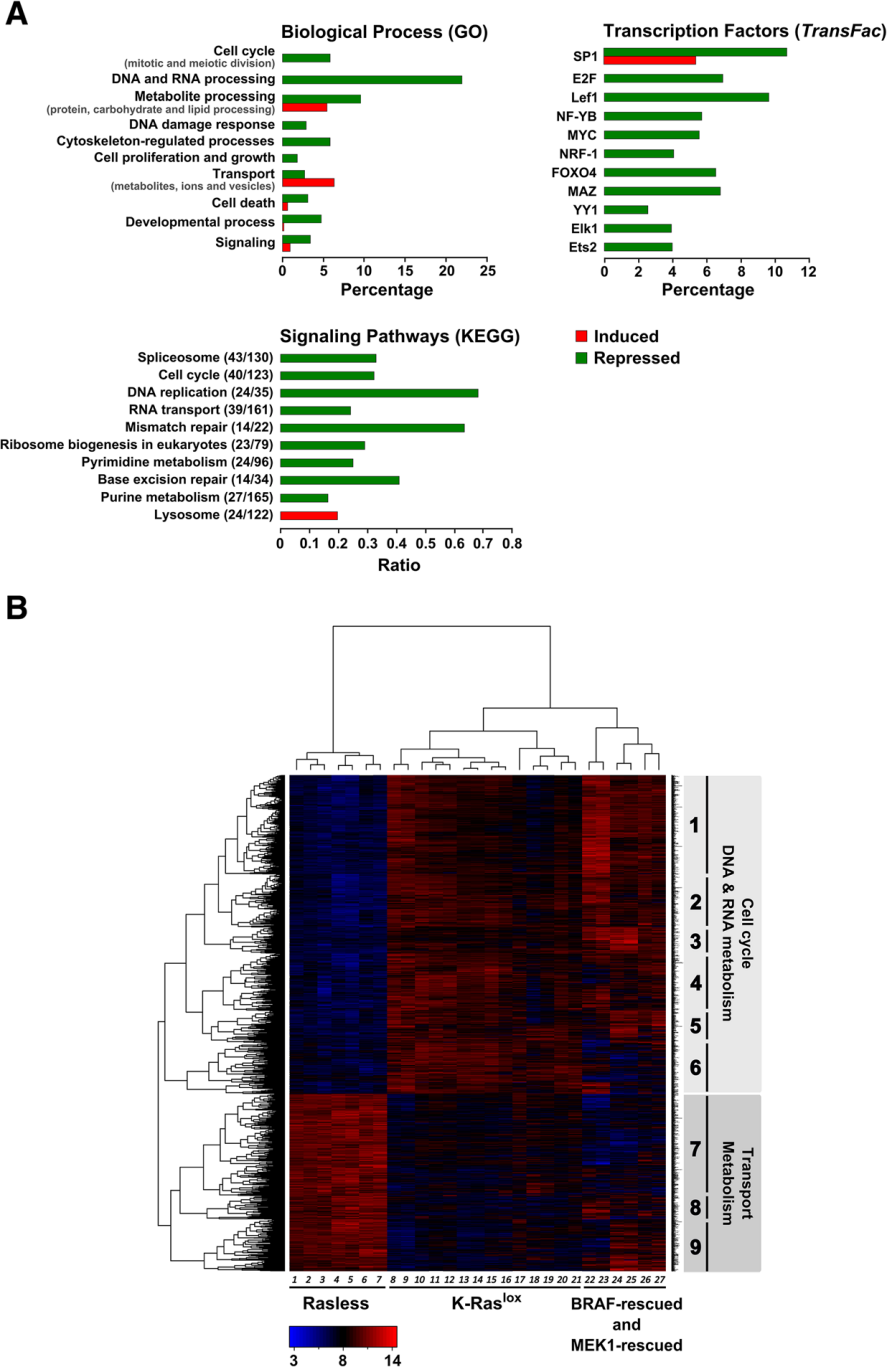
**Global functional annotation and multiclass comparisons of differentially expressed genes of Rasless MEFs*. *(A)** The GeneCodis functional annotation tool was used to identify subsets of the list of differentially expressed genes of Rasless MEFs (FDR=0.01; Additional file 1: Table S1) sharing co-occurrent functional annotations linking them to specific Biological Processes *(Gene Ontology* (GO) database; p-values < 0.005), Transcription Factors *(TransFac* database*;* p-values < 10^-16^) or Signaling Pathways *(KEGG* pathway database; p-values < 10^-10^). Red: induction. Green: repression. The complete functional annotation analyses are described in Additional file 2: Table S2 and Additional file 3: Table S3. **(B)** Hierarchical cluster analysis of the absolute expression values of differentially expressed probesets in Additional file 1: Table S1 (FDR = 0.01). Lanes 1–7: *Rasless cells.* 12-d 4OHT-treated cell lines DU315-6 (1–3, 6, 7) and DU244-1 (4, 5). Lanes 8–21: *K-Ras*^*lox*^*cell lines.* DU315-6 (8, 9, 11, 13, 14, 20), DU244-1 (17, 21), MCL23-1 (15, 16, 18, 19; puromycin-resistant controls of MEK-rescued lines) and JU10-2 (10, 12; hygromycin-resistant controls for BRAF-rescued lines). Lanes 22–27: *BRAF-rescued cell line* LG7-6 (22, 23, 26) and *MEK1-rescued cell line* MCL1-6 (24, 25, 27). Red: overexpression. Blue: repression. Black: unchanged expression. GO categories and associated p-values for horizontal clusters: Clusters 1 and 2: cell-cycle (1.14E-58 and 7.16E-48); cell-division (1.15E-41 and 1.08E-38); mitosis (2.33E-39 and 9.11E-37); DNA replication (6.43E-39 and 2.18E-19). Cluster 1: DNA-dependent transcription (5.1E-26). Cluster 2: chromosome segregation (1.55E-19); DNA damage response (9.06E-19); DNA repair (6.06E-17). Cluster 3: inactivation of MAPK activity (1.91E-04); negative regulation of ERK1/ERK2 cascade (1.79E-03); positive regulation of apoptosis (1.33E-03); negative regulation of cell growth (1.98E-03). Clusters 4 and 6: mRNA processing (1.53E-20 and 1.26E-05); RNA splicing (1.25E-18 and 1.26E-06); transcription, DNA-dependent (9.10E-13 and 1.02E-06). Cluster 7: cellular transport of ions and proteins (5.97E-16); metabolic processes (4.69E-06); small-GTPase-mediated signaling (1.37E-05). Clusters 8 and 9: protein transport (cl.8: 5.99E-09).

Regarding the pool of repressed genes in Rasless cells, Additional file 2: Table S2, section S2-BP identified a series of gene subsets that are functionally linked to several GO categories of Biological Processes (BP) with high statistical significance. Among these, those most significantly affected by the transcriptional repression occurring in Rasless cells were (ranked by p-value): (i) RNA metabolism and processing (p-value 4.19E-80) and DNA metabolism and processing (p-value 1.46E-71); (ii) cellular protein metabolism (p-value 4.24E-18) and modification (p-value 6.05E-11); (iii) mitotic cell cycle progression (p-value 2.52E-68) and associated subcellular processes; (iv) organization of the cytoskeleton and subcellular organelles in relation to chromatin architecture (p-value 1.94E-19); (v) DNA repair (p-value 6.54E-31), and (vi) intracellular transport of RNA (p-value 2.66E-17) and protein (p-value 4.27E-06) (data summarized in Figure 2A-Biological Process (GO). In addition, consistent with the above GO BP categories, Genecodis analysis also identified a series of KEGG signaling pathways that may potentially be disturbed by the transcriptional repression changes occurring in Rasless cells. Among others, the most significant included the following: (i) spliceosome-related signaling (p-value 1.24E-28); cell cycle control (p-value 1.52E-26); (iii) DNA replication (p-value 6.31E-26); (iv) RNA transport (p-value 6.57E-21); (v) mismatch repair (p-value 1.99E-14); and (vi) ribosome biogenesis (p-value 2.62E-14) (Additional file 2: Table S2 section S2-KEGG; data summarized in Figure 2A-Signaling Pathways (KEGG).

On the other hand, specific subsets of the pool of overexpressed loci in Rasless cells were functionally annotated with high statistical significance to a shorter list of GO biological processes such as: (i) cellular protein transport and ion transport (p-value 2.42E-29); (ii) cellular metabolic processes (p-value 3.06E-08) and small GTPase-mediated signal transduction (p-value 1.73E-05) (Additional file 3: Table S3 section S3-BP). GeneCodis also identified a statistically significant accumulation of induced overexpressed loci related to KEGG lysosomal signalling pathways (p-value 1.74E-14) (Additional file 3: Table S3, section S3-KEGG).

The bar plots in Figure 2A summarize and quantitate the percentage distribution of induced or repressed genes from Rasless fibroblasts that can be functionally ascribed to the variety of GO Biological Processes or KEGG signaling pathways identified by Genecodis. As shown, a clear prevalence of repressed loci over induced loci can be seen. Consistent with the phenotypic growth arrest exhibited by Rasless cells in culture, a remarkable over-representation of functional categories relevant to growth arrest, such as metabolic processes, cell cycle progression, cell proliferation and growth, DNA repair, etc., was observed (Figure 2A).

Further support for the notion of a direct link between the absence of the three canonical Ras proteins and cell cycle arrest in Rasless cells was provided by studies aimed at identifying possible transcription factors that could account for the pattern of repressed genes listed in Additional file 1: Table S1 (Figure 2A-Transcription Factors (TransFac); Additional file 2: Table S2 section S2-TF). Interestingly, GeneCodis analysis of the pool of downregulated loci in Rasless cells identified several distinct groups of repressed genes (Additional file 2: Table S2, section S2-TF) that are known targets for transcriptional regulation by E2F or by SP1 at exceptionally high levels of statistical significance (respective p-values 9.6E-50 and 1.80E-49). In addition, several other subsets of repressed loci were also identified as specific targets for the Myc, Fox04 or Egr transcription factors at high levels of significance (p-values: 1.14E-28, 3.54E-24 and 1.67E-12, respectively) (Additional file 2: Table S2 section S2-TF). Consistent with this suggested pattern of negative transcriptional regulation, the mRNA levels for the transcription factors Myc, Fox and Egr were indeed significantly reduced in the transcriptome of Rasless cells (R-fold values in Additional file 1: Table S1: Myc: 0.4; Mycn: 0.17; Foxp1: 0.6; Foxm1: 0.4; Egr1: 0.09; Egr2: 0.16).

### Reversal of the transcriptional signature of Rasless cells by activated BRAF or MEK1

The SAM contrasts depicted in Figure 1B documented that the bulk of differential gene expression changes associated with the growth-arrested Rasless status are absent from the transcriptional profiles of BRAF-rescued and MEK1-rescued MEFs, which are otherwise characterized by their recovered ability to proliferate after expression of either of these two activated downstream components of the Ras signaling pathway [19]. Indeed, the SAM contrasts comparing the transcriptome of untransfected K-Ras^lox^ MEFs with those of either BRAF-rescued or MEK1-rescued fibroblasts recognized only a very short list of transcriptional changes, of which those with the highest R-fold values (i.e., N-Myc) were not significant since they were also detected in the control K-Ras^lox^ MEFs transfected with the empty vectors used to express the exogenous BRAF or MEK1 molecules (not shown). A detailed comparison of the transcriptional profile of Rasless cells with those of either BRAF-rescued or MEK1-rescued MEFs showed that most transcriptional alterations typical of Rasless cells (at FDR = 0.01) were reversed after expression of BRAF or MEK1. Specifically, a total of 938 probesets (735 loci) overexpressed in Rasless cells were repressed in both BRAF- and MEK1-rescued cells, whereas 1679 probesets (1208 loci) repressed in Rasless cells showed overexpression in both the BRAF-and MEK1-rescued cells (Additional file 1: Table S1).

Further visual evidence for the reversibility of the transcriptomic profile of Rasless cells is provided by Figure 2B, depicting a dendrogram generated by hierarchical clustering of microarray hybridization data sets corresponding to the list of differentially expressed probesets in Rasless cells at FDR = 0.01. This dendrogram allowed a clear discrimination of three main vertical branches corresponding to (i) non-proliferating Rasless cells as well as proliferating (ii) control K-Ras^lox^ MEFs and (iii) MEFs reverted to proliferate after transfection of Rasless cells with BRAF or MEK1 (Figure 2B). Interestingly, whereas the proliferating K-Ras^lox^ MEFs showed an almost opposite, antagonistic expression profile to that of the growth-arrested Rasless MEFs, for the most part the transcriptome of the BRAF- and MEK1-rescued MEFs regained an opposite, antagonistic expression profile to that of the Rasless MEFs (Figure 2B). These observations indicate that the transcriptional alterations caused by the absence of the three canonical Ras proteins can be almost completely reversed *in vivo* through the expression of activated components of downstream Ras signaling pathways such as BRAF or MEK1.

Functional annotation analysis of the horizontal gene clusters defined by the dendrogram (Figure 2B, blocks 1–9) highlighted the most significant functional categories accounting for the opposite transcriptional signature patterns displayed by non-proliferating Rasless cells in comparison with proliferating control K-Ras^lox^ or BRAF-rescued or MEK1-rescued MEFs. Clusters 1–6 included genes repressed in arrested Rasless cells and overexpressed in proliferating cells, whereas clusters 7–9 showed completely opposite transcriptional behavior. Interestingly, clusters 1–2 displayed a very marked statistically significant enrichment in genes linked to GO BP categories such as cell cycle, mitosis and DNA replication, DNA-dependent transcription, and response to DNA damage and DNA repair, whereas cluster 3 displayed a significant accumulation of genes related to inactivation of MAPK activity and regulation of apoptosis, and clusters 4–6 showed a special enrichment in genes related to RNA splicing, processing and transcription. On the other hand, clusters 7–9 were significantly enriched in genes involved in cellular transport processes of ions and proteins, metabolic processes or small GTPase-mediated signal transduction (see Figure 2B for details).

In sum, analysis of the functional annotations of the different gene blocks defined by the dendrogram in Figure 2B focused our initial studies mainly on the genes (repressed in the Rasless status) that are involved in regulation of cell cycle progression and the loci (overexpressed in Rasless cells) that are relevant for regulation of growth, in particular in aspects of cellular transport and metabolism.

### Functional gene set enrichment analysis of the reversible transcriptional signature of Rasless fibroblasts. Identification of the most significant components

**Figure 3:**
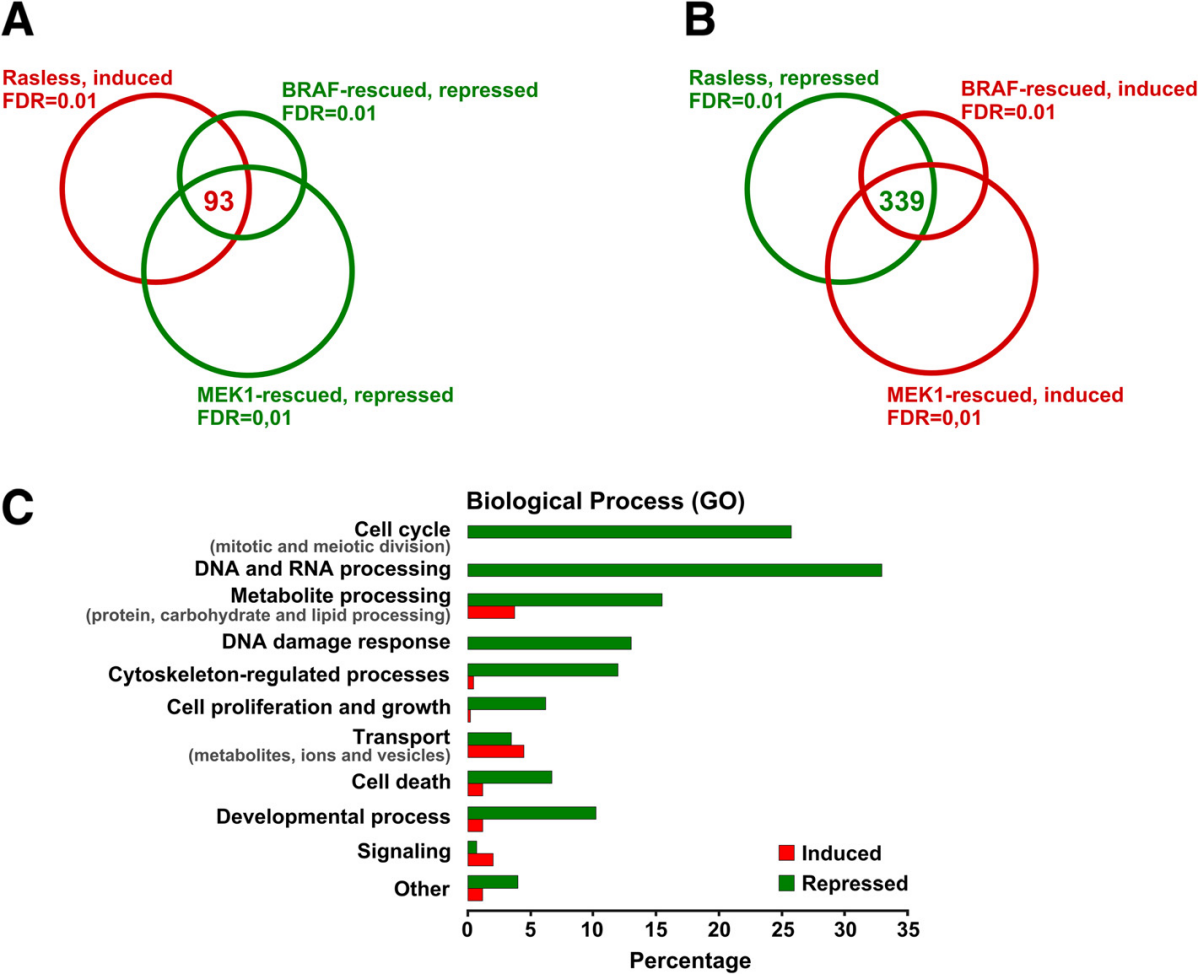
**Differentially expressed genes of Rasless MEFs showing opposite patterns of expression in both BRAF- and MEK1-rescued MEFs. (A)** Venn diagram showing the number of shared differentially expressed genes that were simultaneously detected as upregulated in Rasless MEFs (pair-wise comparison to control MEFs, FDR = 0.01) and as repressed in both BRAF- and MEK1-rescued MEFs (pair-wise comparisons to Rasless MEFs, FDR = 0.01). **(B)** Venn diagram showing the number of shared differentially expressed genes that were simultaneously detected as repressed in Rasless MEFs (pair-wise comparison to control MEFs, FDR = 0.01) and induced in both BRAF- and MEK1-rescued MEFs (pair-wise comparisons to Rasless MEFs, FDR = 0.01). Diagrams **A** and **B** generated using the Venny web-based application as indicated in Methods. Red: transcriptional induction and overexpression. Green: transcriptional repression. **(C)** Functional enrichment of GO Biological Process categories linked to the list of induced and repressed genes identified in panels **A** and **B**. The GeneCodis (Gene Annotation Co-occurrence Discovery) functional annotation tool (http://genecodis.dacya.ucm.es) was used to identify specific gene subsets within the list of 432 differentially expressed induced or repressed genes (691 probesets) (panels 3**A** and 3**B**; Additional file 4: Table S4, FDR = 0.01) that shared co-occurrent functional annotations linking them, with high statistical significance, to particular Biological Processes. Green bars: repressed loci. Red bars: induced loci. Complete GeneCodis functional annotation analyses are described in detail in Additional file 5: Table S5. Specific p-values for the most significant components of the two main categories depicted in this graph are as follows: Cell cycle: various cell division steps (p-value 2.20E-65). DNA/RNA processing: DNA replication (p-value 7.49E-35); regulation of DNA dependent transcription (p-value 3.08E-14); RNA splicing (p-value 4.38E-14); RNA processing (p-value 8.88E-13); DNA damage response (p-value 4.90E-29); DNA repair (p-value 9.92E-23).

As mentioned in previous sections, more than 80% of the transcriptional alterations occurring in Rasless cells are reversed by activated BRAF or MEK1 molecules. Indeed, 735 loci overexpressed in Rasless cells (FDR = 0.01) were repressed in both BRAF- and MEK1-rescued cells (FDR = 0.1), whereas 1208 genes repressed in Rasless cells (FDR = 0.01) showed overexpression in both the BRAF- and MEK1-rescued cells (FDR = 0.1) (Additional file 1: Table S1). However, in order to identify the most relevant transcriptional alterations associated with the Rasless status, we focused our initial analysis on the loci identified by means of Venn diagrams (Figure 3) depicting the intersections occurring among the lists of differential gene expression (identified at very restrictive FDR = 0.01) of non-proliferating Rasless cells and proliferating, BRAF-rescued and MEK1-rescued cells. Figure 3A depicts a Venn diagram identifying 93 induced genes of the transcriptome of Rasless cells that were also simultaneously listed as repressed loci in the tables of differential expression resulting from comparing Rasless cells to the BRAF-rescued or MEK1-rescued MEFs. Similarly, Figure 3B identifies 339 repressed genes of Rasless cells that were simultaneously identified as induced in both the BRAF- and MEK1- rescued MEFs. A detailed description of this restricted pool of induced and repressed loci of Rasless cells showing exactly opposite expression pattern in both the BRAF-rescued and MEK1-rescued MEFs is shown in Additional file 4: Table S4. Although many more differentially expressed genes are actually rescued by BRAF or MEK1 (see Additional file 1: Table S1), this initial report focuses mainly on studying the functional significance of the loci listed in Additional file 4: Table S4, which potentially represent the core of most significant loci regarding the transcriptional changes relevant for the generation and/or reversal of the Rasless status, since their high FDR value (0.01) is indicative of very reproducible and/or high R-fold transcriptional changes.

Additional file 5: Table S5 displays GeneCodis functional analyses of the genes listed in Additional file 4: Table S4. The results show that repressed loci linked to the Rasless status are significantly associated to the regulation of various cell division steps as well as DNA/RNA metabolic processes including -among other categories- DNA replication, regulation of DNA-dependent transcription, RNA splicing and processing and response to DNA damage and DNA repair (Additional file 5: Table S5, section S5A). On the other hand, the Rasless status also appeared to be significantly associated to overexpression of loci related to cellular transport (Additional file 5: Table S5, section S5B). A summary of the most significant GO functional categories affected by the Rasless status is shown in panel 3C. This graph confirms our previous functional analyses (Figure 2) and also indicates that (i) cell cycle progression, (ii) DNA/RNA processing and metabolism related to cellular growth, and (iii) cellular responses to stress and DNA damage are the most prominently targeted cellular biological processes that may be altered under the Rasless status (Figure 3C).

### Diversity of cellular mechanisms responsible for the reversible transcriptional changes of Rasless cells. Sca1 differential expression as a model

The wide variety of statistically significant transcriptional alterations occurring in Rasless cells as regards the expression of components of signaling pathways (including, among others, repression of components of intracellular signaling cascades mediated by p53, MAPK or Jak-STAT, and upregulation of components of small GTPase-mediated signaling; see in Additional file 2: Table S2 and Additional file 3: Table S3, for details) documents the availability of a great diversity of potential biochemical regulatory mechanisms able to contribute, at the molecular level, to the generation of their altered transcriptomic profiles. Thus, it is apparent that the mechanistic details involved in the generation of the transcriptional profile of each differentially expressed gene of Rasless MEFs will ultimately have to be ascertained on an individual basis.

As a representative example, here we report data relevant to the generation of, and possible mechanisms involved in, the patterns of differential expression of Sca1 (Stem cell antigen 1) in Rasless cells (Additional file 6: Figure S1). Sca1 is associated with murine stem cell self-renewal [31], and the modulation of its expression has profound effects on cellular function and tumor development [32]. Our initial microarray-based mRNA expression data showed that Sca1 (= Ly6a1) is one of the most significantly upregulated loci in growth-arrested Rasless cells (R-fold = 10.3) as compared to proliferating K-Ras^lox^ control MEFs, and that its overexpression is reversed in BRAF-rescued and MEK1-rescued MEFs (Additional file 1: Table S1). Interestingly, related loci such as Ly6c1 (R-fold = 7.22), Ly6/neurotoxin (R-fold = 3.03) and Slurp1 (secreted Ly6/Plaur domain containing, R-fold = 12) follow similar patterns of upregulation and reversal in Rasless and rescued MEFs (Additional file 1: Table S1). The mRNA transcriptional data were further confirmed at the level of protein expression by means of FACS analysis using specific antibodies (Additional file 6: Figure S1A). Our data show that treatment of control, K-Ras^lox^ cells with 4OHT for 6d or 12 days to render them Rasless resulted in a significant enhancement (about one order of magnitude) of the Sca1 protein levels detectable in these cells. Of note is that 6-day 4OHT-treated and 12-day-treated Rasless cells showed similar Sca1 protein levels, suggesting that Sca1 upregulation is an early effect linked mechanistically to the process of disappearance of K-Ras from these cells (Additional file 6: Figure S1A). In contrast, our FACS analysis of the BRAF-rescued and MEK1-rescued MEFs also showed a complete recovery of Sca1 protein expression to levels similar to those measured in the control K-Ras^lox^ cells (Additional file 6: Figure S1A). Consistent with previous reports indicating that Sca1 acts downstream from Stat1 [33], a test of the effect of inhibitors of specific signaling molecules on the patterns of expression of Sca1 in our K-Ras^lox^ cells showed that specific Jak inhibitors produced a progressive, time-dependent reversal of the elevated levels of Sca1 expression associated with the disappearance of K-Ras (Additional file 6: Figure S1B). These observations suggest that the Jak-Stat signaling pathway is a significant component of the transcriptional regulatory machinery of Sca1 in these MEFs.

We also tested the feasibility of modulating Sca1 protein expression levels in our MEFs by means of specific shRNA constructs. Thus, using non-targeting shRNA particles as control, we observed that specific shRNA-Sca1 particles produced a very significant reduction in Sca1 protein expression levels in both proliferating K-Ras^lox^ cells and in growth-arrested Rasless cells generated after extended treatment with 4OHT (Additional file 6: Figure S1C). However, the significant reduction in Sca1 expression in Rasless cells was not accompanied by recovery of their proliferative ability, as determined by means of MTT proliferation assays (Additional file 6: Figure S1D) and by WB measurements of the levels of various specific cell progression markers (Additional file 6: Figure S1E)*.* Interestingly, the MTT assays revealed a slight increase of the rate of proliferation of the K-Ras^lox^ cells transduced with shRNA-Sca1 particles in comparison with the controls (Additional file 6: Figure S1A), in agreement with previous reports of hyperproliferation of Sca1 KO cell lineages [34].

These data show that the growth-arrested phenotype of Rasless cells cannot be corrected by reversal of expression levels of Sca1 alone. This would be expected, since the Rasless phenotype is linked to multiple transcriptional alterations (Additional file 1: Table S1) and hence its correction probably requires the reversal of the expression patterns of many more loci than just Sca1, in particular those with pivotal functional roles in signaling networks involved in global pleitropic control of cell cycle progression and arrest.

### Transcriptional changes targeting regulators of early cell cycle progression in Rasless cells

**Figure 4:**
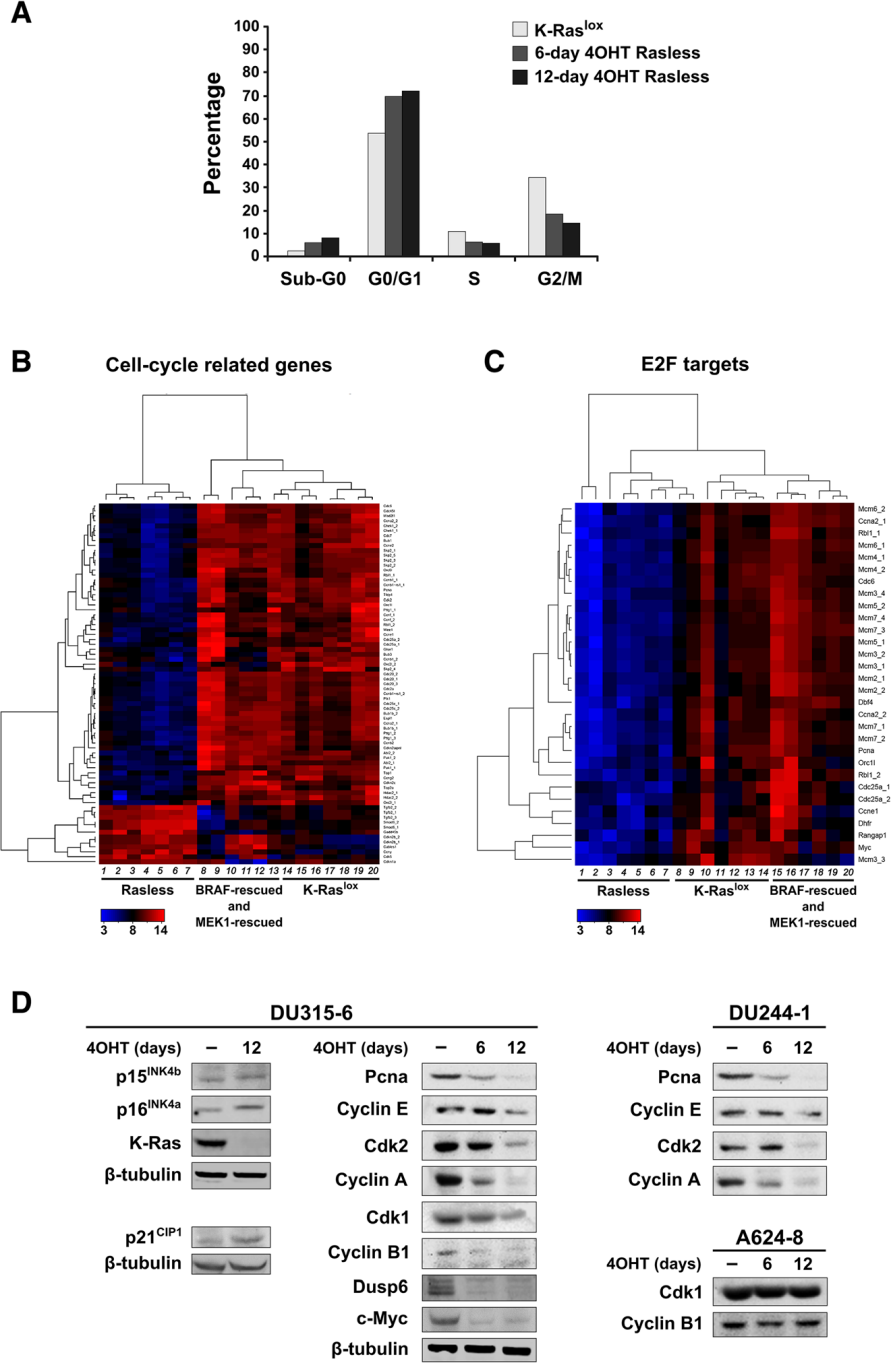
**Characterization of cell cycle-related parameters in Rasless cells. (A)** FACS analysis of cell-cycle stages in cultures of proliferating K-Ras^lox^ MEFs and derived growth-arrested cultures treated with 4OHT for 6-days or 12-days (Rasless). Barplot of a representative experiment including untreated, control DU315-6 cells (K-Ras^lox^) (empty bars) and the same cells after treatment with 4OHT for 6-days (grey bars) or 12-days (black bars). **(B, C)** Hierarchical clustering of cell-cycle-related (panel **B**) and E2F-target (panel **C**) genes differentially expressed in Rasless MEFs. Heatmaps analyzing absolute expression values of a group of 73 probesets relevant for cell cycle regulation (selecting genes annotated to cell-cycle GO term 0007049 in our GeneCodis functional analyses), and a set of 30 probesets for E2F transcription factor targets (identified from available E2F literature [35,36]) that were repressed in Rasless MEFs and rescued by BRAF and MEK1 expression (listed in Additional file 1: Table S1, FDR = 0.01). Red: overexpression. Green: repression. Black: unchanged expression. **(B)** Lanes 1–7: *Rasless cells.* 12-d 4OHT-treated cell lines DU315-6 (1–3, 6,7) and DU244-1 (4, 5). Lanes 14–20: *K-Ras*^*lox*^*cell lines* DU315-6 (14, 16–20) and DU244-1 (15). Lanes 8–13: *BRAF-rescued cell line* LG7-6 (8, 9, 13) and *MEK1-rescued cell line* MCL1-6 (10–12). **(C)** Lanes 1–7: *Rasless cell lines* 12-d 4OHT-treated cell lines DU315-6 (3–7) and DU244-1 (1, 2). Lanes 8–14: *K-Ras*^*lox*^*cell lines* DU315-6 (9, 10, 12–14) and DU244-1 (8, 11). Lanes 15–20: *BRAF-rescued cell line* LG7-6 (15–17) and *MEK1-rescued cell line* MCL1-6 (18–20). **(D)** Immunoblot validation of transcriptional data confirming upregulation of Cdkns (p15^INK4b^, p16^INK4a^ and p21^CIP1^) and downregulation of cell-cycle-related proteins (Myc, Dusp6, Pcna and different cyclins and Cdks) after 6-days or 12-days 4OHT treatments of two representative K-Ras^lox^ cell lines (DU315-6 and DU244-1) and a control double-knockout (H-Ras^-/-^; N-Ras^-/-^) MEF cell line (A624-8).

Our previous functional annotation analyses unveiled a significant enrichment in cellcycle-related genes within the content of several gene clusters defined by the dendrogram comparing the profiles of differential expression of Rasless cells (Figure 2A, B). We also described that expression of activated BRAF or MEK1 is sufficient to reverse the growth arrest of Rasless cells, as well as a large percentage of the associated transcriptional alterations (Additional file 1: Table S1). Searching for mechanistic clues about the phenotypic growth arrest exhibited by Rasless cells, we performed detailed cell cycle FACS analyses of our 4OHT-treated Rasless cell cultures (Figure 4A). Consistent with previous observations [19], our results revealed a predominant blockade in progression through the G1 phase of the cell cycle (Figure 4). This effect was K-Ras-specific because it was not observed in 4OHT-treated cultures of the control constitutive N-Ras/H-Ras double KO cells not harboring the 4OHT-sensitive Cre recombinase and the floxed K-*ras* allele (not shown).

Analysis of the transcriptomic patterns exhibited by Rasless cells offered further clues about their growth-arrest phenotype, since a significant subset of the reversible transcriptomic alterations described in Rasless cells are functionally related to control of early cell cycle progression and cell division (Additional file 1: Table S1, Additional file 2: Table S2 and Additional file 3: Table S3; Figure 4). In particular, panel 4B shows a heatmap describing the transcriptional behavior of a series of positive and negative regulators of cell cycle progression in control, Rasless, and BRAF- or MEK1-rescued fibroblasts. This dendrogram defines two vertically defined branches that discriminate absolutely between the non-proliferating Rasless cells and proliferating, control K-Ras^lox^ and the BRAF- or MEK1-rescued cells. In addition, the horizontal branches identify two clearly distinct sets of repressed and overexpressed genes, thus revealing a largely opposite transcriptional behavior between the growth-arrested, non-proliferating Rasless fibroblasts and the proliferating, K-Ras^lox^ and BRAF- or MEK1-rescued fibroblasts (Figure 4B). Consistent with the phenotypic G1 arrest observed in Rasless cells, Additional file 1: Table S1 and the heatmap in Figure 4B identify in the Rasless clones a large group of significantly repressed genes coding for cyclins and cyclin-dependent kinases (Ccna2, Ccnb1, Ccnb2, Ccne1, Ccne2, Ccnf, Ccng2, Cdk2, Cdk5, etc.), Myc and Myc targets (Myc, Myct1, Mycn, Ndrg4), and other positive regulators of early cell cycle progression (such as Rbl1, Pttg1, Pcna, Top1, Top2a, Skp2, Cdc25a and Cdc25c, Hdac2, Pak1*,* etc.). In addition, a smaller group of overexpressed genes, coding for negative/feedback regulators of cell cycle progression such as Tgfb2, Smad6, Gadd45b, or the cyclin-dependent kinase inhibitors Cdkn1a (p21), Cdkn2b (p15) and Cdkn2a (p16), was also identified (Additional file 1: Table S1; Figure 4B). In contrast, an approximately opposite pattern of induction and repression for all these loci was found in the dendrogram branches corresponding to proliferating fibroblasts, including control K-Ras^lox^ cells as well as BRAF- and MEK1-rescued fibroblasts (Figure 4B, Additional file 1: Table S1). In confirmation of a previous report [19], Cyclin D1 levels did not change in Rasless cells (Additional file 1: Table S1) but were highly overexpressed in the BRAF- and MEK1-rescued cells in comparison to Rasless cells (pair-wise SAM contrasts afforded R-fold values of 4.5 and 3.5 in BRAF- or MEK1-rescued cells, respectively). Also highly consistent with arrest at an early stage of the cell cycle was the observation of a significant downregulation of the expression of multiple E2F-targets [35-37] including cyclins A2 and F, cdc6 and cdc25a, several Mcm (2–7) proteins, and other cycle regulators such as Myc, Rbl1, Dhfr or Dbf4, in the non-proliferating Rasless cells. Such downregulation disappeared, showing the opposite pattern of expression (overexpression), in proliferating control K-Ras^lox^ as well as in BRAF- and MEK1-rescued fibroblasts (Figure 4C; Additional file 1: Table S1).

Confirmation, at the level of protein expression, of some of these transcriptional alterations was obtained by means of Western immunoblots using available specific antibodies, which documented the progressive reduction or disappearance of different cyclins, Cdks and Pcna in two independent representative clones of (K-Ras^lox/lox^; H-Ras^-/-^; N-Ras^-/-^) cells treated with 4OHT to remove K-Ras expression (Figure 4D). As a control, treatment of double KO (H-Ras^-/-^;N-Ras^-/-^) A624-8 cells, which still express K-Ras constitutively [21], did not disclose any change in the expression level of Cyclin B1 or Cdk1 (Figure 4D), indicating that the above changes are not off-target effects of the 4OHT treatment.

The patterns of transcriptional downregulation of Myc, E2F targets, Cyclins and Cdks are consistent with the G1/S blockade observed experimentally by flow cytometry in Rasless cells. Consistent with recent reports demonstrating the essential role of Myc in K-Ras-driven tumorigenesis [38], the strong Myc phenotype displayed by Rasless cells is noteworthy, as seen from the detection of direct transcriptional repression of the Myc proteins (Additional file 1: Table S1) as well as of many recognized Myc transcriptional targets (Additional file 2: Table S2-TF). Furthermore, our additional observations in Rasless cells of roughly unchanged levels of cyclin D1, together with the significant upregulation of Cdk inhibitors such as Cdkn1a (p21), Cdkn2b (p15) and Cdkn2a (p16), are consistent with a previous report challenging the previously accepted notion that Ras signalling initiates the cell cycle by inducing expression of D-type cyclins [30,39] and suggesting that p21 may be mechanistically involved in preventing cell proliferation in the absence of Ras proteins [19]. Indeed, since E2F proteins and targets are controlled by Rb, and since Rb loss is known to override the requirement for downstream ERK signalling for cell proliferation [30,40,41], and p21 is a transcriptional target of p53 [42,43], the previous hypothesis might be tested experimentally by checking whether or not the downregulation of Rb, p53 or Cdkns (p21, p15, p16), individually or in combination, could contribute to bypassing the proliferative defects of Rasless cells and restoring their proliferative ability in a manner similar to that observed with activated BRAF or MEK1 molecules*.* Our analyses of miRNA profiles in Rasless and rescued MEFs (see below) are also consistent with these views.

### Differential expression of microRNAs in Rasless MEFs

**Figure 5:**
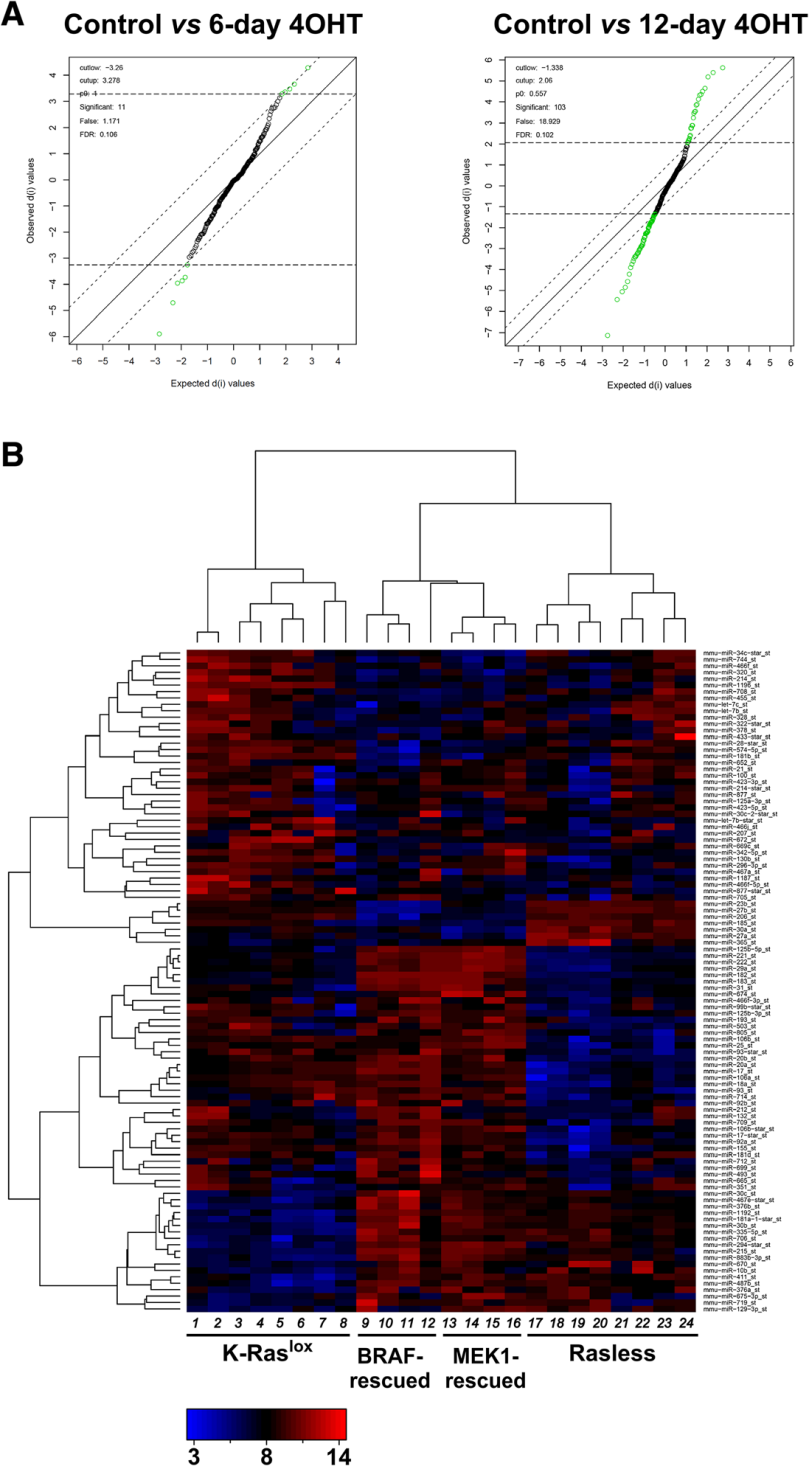
**Differential expression of microRNAs in Rasless cells. (A)** Statistical identification of differentially expressed miRNAs in Rasless MEFs. SAM contrasts [109] comparing the microarray-generated miRNA expression profiles of K-Ras^lox^ cell lines treated with 4OHT for 6-days (left panel) or for 12-days (right panel) with those of control, untreated K-Ras^lox^ MEFs. The plots identified 11 differentially expressed miRNAs after 6-days of 4OHT treatment (left panel), and 103 differentially expressed miRNAs after the 12-day treatment (right panel) using similar FDR = 0.1values. Differential expression for a given miRNA is calculated by the distance of the spot representing its expression value to the no-change diagonal. Green dots depict differentially expressed miRNAs. Black dots remaining close to the diagonal represent miRNAs without significant expression changes relative to the control samples. **(B)** Hierarchical clustering of differentially expressed microRNAs of Rasless MEFs**.** Heatmap generated by cluster analysis of the absolute expression values of the group of 103 differentially expressed miRNAs listed in Table 1 (FDR = 0.1), obtained with expression data from non-proliferating Rasless MEFs (lanes 17–24); proliferating control K-Ras^lox^ (H-Ras^-/-^; N-Ras^-/-^) cells expressing only K-Ras and the same cells transfected with the empty vectors (lanes 1–8), or BRAF-rescued (lanes 9–12) and MEK1-rescued MEFs (lanes 13–16). The intensity of color saturation in each miRNA box (ranging from 3 to 14 on a log_2_ scale) provides a quantitative estimation of its expression level. Red: overexpression. Green: repression. Black:unchanged expression signals relative to controls. Lanes 1–4: *K-Ras*^*lox*^*cell lines* DU315-6 (lanes 1, 2) and DU244-1 (lanes 3, 4). Lanes 5–8: *K-Ras*^*lox*^ *+ empty puromoycin resistance vector cell line* MCL23-1 (5–8). Lanes 9–16**:***BRAF-rescued cell line* LG7-6 (lanes 9–12) and *MEK1-rescued cell line* MCL1-6 (lanes 13–16). Lanes 17–24: *Rasless cell lines* 6-d 4OHT-treated DU315-6 (lanes 21, 22), 12-d 4OHT-treated DU315-6 (lanes 19, 20), 6-d 4OHT-treated DU244-1 (lanes 23, 24) and 12-d 4OHT-treated DU244-1 (lanes 17, 18).

In order to uncover additional cellular mechanisms responsible for the reversible cell cycle arrest and altered transcriptional pattern of Rasless cells, we performed Genecodis analyses that identified –with very high levels of statistical significance- a series of specific miRNAs potentially capable of generating large blocks of the repressed (Additional file 2: Table S2 section S2-miRNA) or induced (Additional file 3: Table S3 section S3-miRNA) mRNAs of Rasless MEFs listed in Additional file 1: Table S1. In order to test these predictions experimentally and to identify specific miRNA alterations linked to the Rasless status, we carried out specific microarray hybridizations using miRNA preparations from defined sample sets, including control, untreated K-Ras^lox^ MEFs as well as 4OHT-treated cultures leading to the Rasless status, or BRAF- and MEK1-rescued cell lines (Figure 5). We observed that partial K-Ras removal achieved after 6 days of 4OHT treatment allowed detection of a reduced number of differentially expressed miRNAs, but that total elimination of Ras proteins after 12 days of 4OHT treatment allowed the identification of at least 103 distinct miRNAs that were differentially expressed in the Rasless cells at the statistically significant FDR value of 0.1 (Figure 5A). A detailed description of the list of 103 miRNAs that were specifically induced or repressed in MEFs after reaching the Rasless status is shown in Table 1. As happened with the differentially expressed mRNAs (Additional file 1: Table S1), the majority (~75%) of the differentially expressed miRNAs of 12-day 4OHT-treated Rasless cells were repressed (Table 1), indicating a predominant functional role of the missing K-Ras as a positive regulator of miRNA transcription. The relevance of this group of differentially expressed miRNAs with regards to the Rasless phenotype is further supported by the observation that the majority (73 out of the 103) of differentially expressed miRNAs listed in Table 1 were predicted (with highly significant p-values ranging between 10E-06 and 10E-21) by Genecodis analyses (Additional file 2: Table S2 section S2-miRNA and Additional file 3: Table S3 section S3-miRNA) of the list of differentially expressed mRNAs in Additional file 1: Table S1 (Table 1, column *Genecodis prediction*). Table 1 also shows that a small group of these differentially expressed miRNAs were already present in 6-day 4OHT-treated cells*.* The rapid response and the sensitivity shown by the differential expression of this subgroup of miRNAs to the partial disappearance of K-Ras in the 6-day 4OHT-treated MEFs (Figure 1) suggests the potential significance of their differential expression in relation to the initial steps of generation of the Rasless phenotype.

**Table 1:** Differential microRNA expression in Rasless MEFs

**12-D 4OHT-TREATED MEFs (RASLESS)**									**6-D 4OHT-TREATED**	**BRAF-RESCUED**	**MEK1-RESCUED**
***Pairwise comparison to control K-Ras***^***lox ***^ ***cells***											
***miRNA probeset ID***	***miRNA name***	***family***	***Chromosome, strand and cluster***	***Genecodis prediction***	***d-value***	***p-value***	***q-value***	***R-fold vs. Control***	***R-fold vs. Control***	***R-fold vs. Rasless***	***R-fold vs. Rasless***
mmu-let-7b_st	mmu-let-7b	let-7	*Chrom* 15 (+): let-7c-2 *|***let-7b**	7.95E-06	-2.2	0.02	0.05	0.68	n.a.	n.a.	n.a.
mmu-let-7c_st	mmu-let-7c	let-7	*Chrom* 16 (+)*:* mir-99a *|***let-7c-1**	2.89E-06	-1.41	0.09	0.17	0.78	n.a.	n.a.	n.a.
mmu-let-7b-star_st	mmu-let-7b*	let-7	*Chrom* 15 (+): let-7c-2 *|***let-7b**	1.86E-21	-1.36	0.1	0.15	0.63	n.a.	n.a.	n.a.
mmu-miR-206_st	mmu-miR-206	mir-1	*Chrom* 1 (+)*:***mir-206***|* mir-133b		2.76	0.01	0.03	1.78	n.a.	0.05	0.13
mmu-miR-10b_st	mmu-miR-10b	mir-10	*Chrom* 2 (+)		2.17	0.02	0.05	2.26	n.a.	n.a.	n.a.
mmu-miR-125b-3p_st	mmu-miR-125b-3p	mir-125	*Chrom* 9 (+)	7.97E-06 (1.13E-07)	-3.91	0.00	0.03	0.32	n.a.	3.8	3.42
mmu-miR-125b-5p_st	mmu-miR-125b-5p	mir-125	*Chrom* 9 (+)		-1.71	0.05	0.09	0.75	n.a.	2.18	2.29
mmu-miR-125a-3p_st	mmu-miR-125a-3p	mir-125	*Chrom* 17 (+): **mir-99b***|* let-7e *|***mir-125a**		-1.67	0.05	0.09	0.45	n.a.	n.a.	n.a.
mmu-miR-129-3p_st	mmu-miR-129-3p	mir-129	*Chrom* 2 (-)	4.56E-06	5.63	0.00	0.02	6.02	n.a.	n.a.	n.a.
mmu-miR-130b_st	mmu-miR-130b	mir-130	*Chrom* 16 (-): mir-301b *|***mir-130b**	6.71E-17	-3.2	0.01	0.03	0.45	n.a.	n.a.	1.96
mmu-miR-132_st	mmu-miR-132	mir-132	*Chrom* 11 (+): **mir-212***|***mir**-**132**	6.39E-17	-2.45	0.01	0.04	0.11	n.a.	17.75	3.61
mmu-miR-212_st	mmu-miR-212	mir-132	*Chrom* 11 (+*):***mir-212***|***mir-132**	1.05E-14	-1.95	0.03	0.07	0.21	n.a.	11.18	1.47
mmu-miR-487b_st	mmu-miR-487b	mir-154	*Chrom* 12 (+): mir-495 *|* mir-667 *|* mir-376c *|* mir-654 *|***mir-376b***|***mir-376a***|* mir-300 *|* mir-381 *|***mir-487b***|* mir-539 *|* mir-544 *|* mir-382 *|* mir-134 *|* mir-668 *|* mir-485 *|* mir-453	8.76E-11	3.25	0.01	0.03	3.45	n.a.	n.a.	n.a.
mmu-miR-155_st	mmu-miR-155	mir-155	*Chrom* 16 (+)	2.44E-08	-3.11	0.01	0.03	0.16 # [0.47]	n.a. # [1.1]	7.5 # [10.68]	4.38 # [6.26]
mmu-miR-17-star_st	mmu-miR-17*	mir-17	*Chrom* 14 (+): **mir-17***|***mir-18a***|* mir-19a *|***mir-20a***|* mir-19b-1 *|***mir-92a-1**		-5.05	0.00	0.02	0.32	n.a.	3.47	2.69
mmu-miR-18a_st	mmu-miR-18a	mir-17	*Chrom* 14 (+): **mir-17***|***mir-18a***|* mir-19a *|***mir-20a***|* mir-19b-1 *|***mir-92a-1**	(5.42E-08)	-4.84	0.00	0.03	0.38	n.a.	4.0	2.46
mmu-miR-20b_st	mmu-miR-20b	mir-17	*Chrom* X (-): **mir-106a***|* mir-18b *|***mir-20b***|* mir-19b-2 *|* mir-92a-2 *|* mir-363	7.42E-17	-4.22	0.00	0.03	0.21	0.29	9.88	5.93
mmu-miR-106a_st	mmu-miR-106a	mir-17	*Chrom* X (-): **mir-106a***|* mir-18b *|***mir-20b***|* mir-19b-2 *|* mir-92a-2 *|* mir-363	8.87E-14 (1.25E-06)	-3.38	0.01	0.03	0.19 # [0.15]	n.a. # [0.86]	6.84 # [7.75]	6.07 # [13.58]
mmu-miR-106b-star_st	mmu-miR-106b*	mir-17	*Chrom* 5 (-): **mir-106b***|***mir-93***|***mir-25**		-2.99	0.01	0.03	0.29	n.a.	4.92	5.28
mmu-miR-17_st	mmu-miR-17	mir-17	*Chrom* 14 (+): **mir-17***|***mir-18a***|* mir-19a *|***mir-20a***|* mir-19b-1 *|***mir-92a-1**	1.08E-15 (3.84E-07)	-2.87	0.01	0.03	0.37 # [0.27]	n.a. # [0.58]	5.74 # [6.1]	3.48 # [9.01]
mmu-miR-20a_st	mmu-miR-20a	mir-17	*Chrom* 14 (+): **mir-17***|***mir-18a***|* mir-19a *|***mir-20a***|* mir-19b-1 *|***mir-92a-1**	(7.38E-07)	-2.58	0.01	0.04	0.37 # [0.24]	n.a. # [0.55]	5.13 # [5.8]	3.57 # [7.85]
mmu-miR-93-star_st	mmu-miR-93*	mir-17	*Chrom* 5 (-): **mir-106b***|***mir-93***|***mir-25**		-2.44	0.01	0.04	0.42	0.33	2.58	2.15
mmu-miR-106b_st	mmu-miR-106b	mir-17	*Chrom* 5 (-): **mir-106b***|***mir-93***|***mir-25**	2.82E-11	-2.25	0.02	0.05	0.46	n.a.	2.17	3.07
mmu-miR-93_st	mmu-miR-93	mir-17	*Chrom* 5 (-): **mir-106b***|***mir-93***|***mir-25**	4.77E-14 (4.35E-06)	-1.84	0.04	0.08	0.64	n.a.	2.19	2.4
mmu-miR-181d_st	mmu-miR-181d	mir-181	*Chrom* 8 (-): mir-181c *|***mir-181d**	7.13E-10	-3.24	0.01	0.03	0.23	n.a.	5.63	4.29
mmu-miR-181b_st	mmu-miR-181b	mir-181	*Chrom* 1 (+): **mir-181a-1***|***mir-181b-1**	1.40E-09	-1.43	0.09	0.13	0.68	n.a.	n.a.	n.a.
mmu-miR-181a-1-star_st	mmu-miR-181a-1*	mir-181	*Chrom* 1 (+): **mir-181a-1***|***mir-181b-1**	1.37E-08	5.4	0.00	0.02	8.55	5.5	n.a.	1.68
mmu-miR-182_st	mmu-miR-182	mir-182	*Chrom* 6 (-): **mir-183***|* mir-96 *|***mir-182**	4.24E-11	-2.21	0.02	0.05	0.52	n.a.	22.16	13.95
mmu-miR-183_st	mmu-miR-183	mir-183	*Chrom* 6 (-): **mir-183***|* mir-96 *|***mir-182**	2.68E-07	-2.26	0.02	0.05	0.62	n.a.	28.07	18.61
mmu-miR-185_st	mmu-miR-185	mir-185	*Chrom* 16 (-)		2.38	0.02	0.04	1.39	n.a.	0.23	0.25
mmu-miR-215_st	mmu-miR-215	mir-192	*Chrom* 1 (+): mir-194-1 *|***mir-215**	5.61E-10	2.89	0.01	0.03	4.3	n.a.	6.23	5.21
mmu-miR-193_st	mmu-miR-193	mir-193	*Chrom* 11 (+)	4.31E-08	-1.73	0.05	0.09	0.47	0.3	2.33	2.36
mmu-miR-207_st	mmu-miR-207	mir-207	*Chrom* 4 (+)		-1.44	0.08	0.13	0.61	n.a.	n.a.	1.69
mmu-miR-21_st	mmu-miR-21	mir-21	*Chrom* 11 (-)	6.39E-09	-1.74	0.05	0.09	0.31	n.a.	n.a.	2.53
mmu-miR-214_st	mmu-miR-214	mir-214	*Chrom* 1 (+): mir-199a-2 *|***mir-214**	1.58E-08 (1.13E-07)	-3.02	0.01	0.03	0.54	n.a.	n.a.	n.a.
mmu-miR-214-star_st	mmu-miR-214*	mir-214	*Chrom* 1 (+): mir-199a-2 *|***mir-214**		-2.59	0.01	0.05	0.26	n.a.	n.a.	3.02
mmu-miR-222_st	mmu-miR-222	mir-221	*Chrom* X (-): **mir-222***|***mir-221**	6.41E-08	-5.43	0.00	0.02	0.47	n.a.	5.76	7.49
mmu-miR-221_st	mmu-miR-221	mir-221	*Chrom* X (-): **mir-222***|***mir-221**	1.64E-15	-3.04	0.01	0.03	0.54	n.a.	5.21	6.11
mmu-miR-23b_st	mmu-miR-23b	mir-23	*Chrom* 13 (+): **mir-23b***|***mir-27b***|* mir-3074-1 *|* mir-24-1	1.92E-07	2.24	0.02	0.05	1.43	n.a.	0.17	0.43
mmu-miR-92a_st	mmu-miR-92a	mir-25	*Chrom* 14 (+): **mir-17***|***mir-18a***|* mir-19a *|***mir-20a***|* mir-19b-1 *|***mir-92a-1**	3.92E-10	-7.12	0.00	0.02	0.24	n.a.	6.59	4.41
mmu-miR-25_st	mmu-miR-25	mir-25	*Chrom*5(-): **mir-106b***|***mir-93***|***mir-25**	1.03E-10	-2.68	0.01	0.03	0.38 # [0.51]	n.a. # [0.62]	2.76 # [2.9]	4.96 # [5.08]
mmu-miR-92b_st	mmu-miR-92b	mir-25	*Chrom* 3 (-)	1.67E-09	-1.69	0.05	0.09	0.61	n.a.	2.2	2.35
mmu-miR-27a_st	mmu-miR-27a	mir-27	*Chrom* 8 (+): mir-23a *|***mir-27a***|* mir-24-2 *|* mir-3074-2	6.34E-07	2.89	0.01	0.03	1.57	n.a.	n.a.	0.54
mmu-miR-27b_st	mmu-miR-27b	mir-27	*Chrom* 13 (+): **mir-23b***|***mir-27b***|* mir-3074-1 *|* mir-24-1	5.98E-07	3.86	0.00	0.03	1.93	n.a.	0.07	0.23
mmu-miR-28-star_st	mmu-miR-28*	mir-28	*Chrom* 16 (+)	6.88E-07	-1.36	0.10	0.15	0.63	n.a.	n.a.	n.a.
mmu-miR-29a_st	mmu-miR-29a	mir-29	*Chrom* 6 (-): mir-29b-1 *|***mir-29a**	8.04E-10 (5.02E-07)	-4.57	0.00	0.03	0.28	n.a.	8.13	12.61
	
mmu-miR-294-star_st	mmu-miR-294*	mir-290	*Chrom* 7 (+): mir-290 *|* mir-291a *|* mir-292 *|* mir-291b *|* mir-293 *|***mir-294***|* mir-295	4.58E-08	2.41	0.01	0.04	3.19	n.a.	n.a.	2.81
mmu-miR-296-3p_st	mmu-miR-296-3p	mir-296	*Chrom* 2 (-): mir-298 ***| *mir-296**		-3.21	0.01	0.03	0.42	n.a.	n.a.	2.1
mmu-miR-30c-2-star_st	mmu-miR-30c-2*	mir-30	*Chrom* 1 (+)		-2.18	0.02	0.05	0.43	n.a.	n.a.	n.a.
mmu-miR-30c_st	mmu-miR-30c	mir-30	*Chrom* 4 (-): mir-30f *|* mir-30e *|***mir-30c-1**	2.40E-15	2.06	0.03	0.06	2.05	n.a.	n.a.	n.a.
mmu-miR-30a_st	mmu-miR-30a	mir-30	*Chrom* 1 (+)	2.41E-14	2.77	0.01	0.03	1.69	n.a.	n.a.	0.39
mmu-miR-30b_st	mmu-miR-30b	mir-30	*Chrom* 15 (-): mir-30d *|***mir-30b**	6.64E-17	3.88	0.00	0.03	2.67	n.a.	n.a.	n.a.
mmu-miR-31_st	mmu-miR-31	mir-31	*Chrom* 4 (-)	(3.84E-07)	-2.87	0.01	0.03	0.64	n.a.	2.12	1.88
mmu-miR-320_st	mmu-miR-320	mir-320	*Chrom* 14 (+)	7.97E-07	-1.39	0.09	0.14	0.73	n.a.	n.a.	n.a.
mmu-miR-322-star_st	mmu-miR-322*	mir-322	*Chrom* X (-): **mir-322***|***mir-503***|***mir-351***|* mir-542 *|* mir-450a-2 *|* mir-450a-1 *|* mir-450b	6.72E-13	-1.72	0.05	0.09	0.37	n.a.	n.a.	1.77
mmu-miR-328_st	mmu-miR-328	mir-328	*Chrom* 8 (-)		-1.98	0.03	0.07	0.39	n.a.	n.a.	n.a.
mmu-miR-335-5p_st	mmu-miR-335-5p	mir-335	*Chrom* 6 (+)	5.76E-06	4.65	0.00	0.03	9.44 # [3.88]	n.a. # [0.47]	n.a. # [0.01]	n.a # [0.16]
mmu-miR-34c-star_st	mmu-miR-34c*	mir-34	*Chrom* 9 (-): mir-34b *|***mir-34c**	9.47E-11	-1.39	0.09	0.14	0.46	n.a.	n.a.	0.28
mmu-miR-342-5p_st	mmu-miR-342-5p	mir-342	*Chrom* 12 (+)		-1.83	0.04	0.08	0.58	0.47	n.a.	n.a.
mmu-miR-351_st	mmu-miR-351	mir-351	*Chrom* X (-): **mir-322***|***mir-503***|***mir-351***|* mir-542 *|* mir-450a-2 *|* mir-450a-1 *|* mir-450b	(2.36E-06)	-2.01	0.03	0.06	0.22	n.a.	n.a.	3.61
mmu-miR-365_st	mmu-miR-365	mir-365	*Chrom* 16 (+): mir-193b *|***mir-365-1**		4.32	0.00	0.03	4.75	n.a.	0.21	0.32
mmu-miR-376a_st	mmu-miR-376a	mir-368	*Chrom* 12 (+): mir-494 *|* mir-679 *|* mir-1193 *|* mir-666 *|* mir-543 *|* mir-495 *|* mir-667 *|* mir-376c *|* mir-654 *|***mir-376b***|***mir-376a***|* mir-300 *|* mir-381 *|***mir-487b***|* mir-539 *|* mir-544 *|* mir-382	1.84E-10	2.17	0.02	0.05	2.31	n.a.	n.a.	n.a.
mmu-miR-376b_st	mmu-miR-376b	mir-368	*Chrom* 12 (+): mir-329 *|* mir-494 *|* mir-679 *|* mir-1193 *|* mir-666 *|* mir-543 *|* mir-495 *|* mir-667 *|* mir-376c *|* mir-654 *|***mir-376b***|***mir-376a***|* mir-300 *|* mir-381 *|***mir-487b***|* mir-539 *|* mir-544	1.89E-09	4.35	0.00	0.03	3.24	2.05	n.a.	n.a.
mmu-miR-378_st	mmu-miR-378	mir-378	*Chrom* 18 (-)		-2.92	0.01	0.03	0.27	n.a.	0.24	1.91
mmu-miR-411_st	mmu-miR-411	mir-379	*Chrom* 12 (+): mir-379 *|***mir-411***|* mir-299a *|* mir-299b *|* mir-380 *|*mir-1197 *|* mir-323 *|* mir-758 *|* mir-329 *|* mir-494 *|* mir-679 *|* mir-1193 *|* mir-666 *|* mir-543 *|* mir-495 *|* mir-667	2.81E-10	2.26	0.02	0.05	2.72	n.a.	n.a.	n.a.
mmu-miR-423-5p_st	mmu-miR-423-5p	mir-423	*Chrom* 11 (-)	(2.68E-09)	-2.41	0.01	0.04	0.41	n.a.	n.a.	n.a.
mmu-miR-423-3p_st	mmu-miR-423-3p	mir-423	*Chrom* 11 (-)	(7.44E-08)	-1.89	0.03	0.07	0.49	n.a.	2.22	1.9
mmu-miR-433-star_st	mmu-miR-433*	mir-433	*Chrom* 12 (+): mir-337 *|* mir-3544 *|* mir-540 *|***mir-665***|* mir-3070a *|* mir-3070b *|* mir-431 *|***mir-433***|* mir-127 *|* mir-434 *|* mir-432 *|* mir-3071 *|* mir-136		-2.25	0.02	0.05	0.59	n.a.	n.a.	n.a.
mmu-miR-455_st	mmu-miR-455	mir-455	*Chrom* 4 (+)	9.31E-07	-2.88	0.01	0.03	0.23	n.a.	n.a.	n.a.
mmu-miR-466f_st	mmu-miR-466f	mir-467	*Chrom* 2 (+): mir-466m *|***mir-466f-1***|* mir-669f *|* mir-669e *|* mir-669b *|* mir-669d *|* mir-466f-2 *|* mir-669l *|* mir-669d-2 *|* mir-466f-3 *|* mir-297a-2 *|* mir-466o *|* mir-467c *|* mir-466b-1 *|* mir-669a-3 *|* mir-669k *|***mir-467a-1***|* mir-466b-8 *|* mir-669a-1		-3.48	0.00	0.03	0.25	n.a.	n.a.	n.a.
mmu-miR-467a_st	mmu-miR-467a	mir-467	*Chrom* 2 (+): mir-466m *|***mir-466f-1***|* mir-669f *|* mir-669e *|* mir-669b *|* mir-669d *|* mir-466f-2 *|* mir-669l *|* mir-669d-2 *|* mir-466f-3 *|* mir-297a-2 *|* mir-466o *|* mir-467c *|* mir-466b-1 *|* mir-669a-3 *|* mir-669k *|***mir-467a-1***|* mir-466b-8 *|* mir-669a-1 *|* mir-669g *|* mir-669j *|* mir-467a-2 *|* mir-466e *|* mir-669a-4 *|* mir-467b *|* mir-466c-1 *|* mir-669a-5 *|* mir-467a-3 *|* mir-466c-2 *|* mir-669a-6 *|* mir-467a-4	7.61E-08	-3.38	0.01	0.03	0.52	0.46	n.a.	n.a.
mmu-miR-466f-5p_st	mmu-miR-466f-5p	mir-467	*Chrom* 2 (+): mir-466m *|***mir-466f-1***|* mir-669f *|* mir-669e *|* mir-669b *|* mir-669d *|* mir-466f-2 *|* mir-669l *|* mir-669d-2 *|* mir-466f-3 *|* mir-297a-2 *|* mir-466o *|* mir-467c *|* mir-466b-1 *|* mir-669a-3 *|* mir-669k *|***mir-467a-1***|* mir-466b-8 *|* mir-669a-1	1.86E-07	-2.8	0.01	0.03	0.32	n.a.	n.a.	n.a.
mmu-miR-466f-3p_st	mmu-miR-466f-3p	mir-467	*Chrom* 2 (+): mir-466m *|***mir-466f-1***|* mir-669f *|* mir-669e *|* mir-669b *|* mir-669d *|* mir-466f-2 *|* mir-669l *|* mir-669d-2 *|* mir-466f-3 *|* mir-297a-2 *|* mir-466o *|* mir-467c *|* mir-466b-1 *|* mir-669a-3 *|* mir-669k *|***mir-467a-1***|* mir-466b-8 *|* mir-669a-1	2.94E-17	-1.99	0.03	0.07	0.54	n.a.	3.54	2.36
mmu-miR-466j_st	mmu-miR-466j	mir-467	*Chrom* 10 (+)		-1.56	0.07	0.11	0.48	n.a.	n.a.	n.a.
mmu-miR-669c_st	mmu-miR-669c	mir-467	*Chrom* 2 (+): mir-669a-11 *|* mir-467a-10 *|* mir-466b-3 *|* mir-669a-12 *|***mir-467e***|* mir-466p *|* mir-467d *|* mir-466a *|* mir-297c *|***mir-669c***|* mir-669a-2 *|* mir-297b *|* mir-466d *|* mir-669m-1 *|* mir-669m-2 *|* mir-466n *|* mir-669o *|* mir-466g *|* mir-466h *|* mir-297a-3 *|* mir-466l *|* mir-297a-4 *|* mir-669i *|* mir-669h	8.81E-11	-1.34	0.11	0.15	0.48	n.a.	n.a.	n.a.
mmu-miR-467e-star_st	mmu-miR-467e*	mir-467	*Chrom* 2 (+): mir-467a-8 *|* mir-466b-7 *|*mir-669p-2 *|* mir-467a-9 *|* mir-466b-2 *|* mir-669a-10 *|* mir-669a-11 *|* mir-467a-10 *|* mir-466b-3 *|* mir-669a-12 *|***mir-467e***|* mir-466p *|* mir-467d *|* mir-466a *|* mir-297c *|***mir-669c***|* mir-669a-2 *|* mir-297b *|* mir-466d *|* mir-669m-1 *|* mir-669m-2 *|* mir-466n *|* mir-669o *|* mmir-466g *|* mir-466h	4.32E-09	3.57	0.00	0.03	3.2	n.a.	3.88	1.57
mmu-miR-493_st	mmu-miR-493	mir-493	*Chrom* 12 (+): mir-673 *|***mir-493***|* mir-337 *|* mir-3544 *|* mir-540 *|***mir-665***|* mir-3070a *|* mir-3070b		-1.59	0.06	0.11	0.52	n.a.	3.02	n.a.
mmu-miR-503_st	mmu-miR-503	mir-503	*Chrom* X (-): **mir-322***|***mir-503***|***mir-351***|* mir-542 *|* mir-450a-2 *|* mir-450a-1 *|* mir-450b	2.84E-06	-2.58	0.01	0.04	0.25	0.23	2.75	2.95
mmu-miR-574-5p_st	mmu-miR-574-5p	mir-574	*Chrom* 5 (+)	(5.07E-06)	-3.32	0.01	0.03	0.43	n.a.	n.a.	n.a.
mmu-miR-652_st	mmu-miR-652	mir-652	*Chrom* X (+)	1.72E-07	-1.59	0.06	0.11	0.67	n.a.	n.a.	1.48
mmu-miR-665_st	mmu-miR-665	mir-665	*Chrom* 12 (+): **mir-493***|* mir-337 *|* mir-3544 *|* mir-540 *|***mir-665***|* mir-3070a *|* mir-3070b *|* mir-431 *|***mir-433***|* mir-127 *|* mir-434 *|* mir-432 *|* mir-3071 *|* mir-136	(2.89E-06)	-1.72	0.05	0.09	0.32	n.a.	4.99	2.88
mmu-miR-670_st	mmu-miR-670	mir-670	*Chrom* 2 (-)		2.11	0.02	0.06	3.48	n.a.	n.a.	n.a.
mmu-miR-672_st	mmu-miR-672	mir-672	*Chrom* X (-):		-1.47	0.08	0.13	0.22	n.a.	n.a.	1.48
mmu-miR-674_st	mmu-miR-674	mir-674	*Chrom* 2 (+)		-2.21	0.02	0.05	0.68	n.a.	n.a.	2.76
mmu-miR-675-3p_st	mmu-miR-675-3p	mir-675	*Chrom* 7 (-)	2.79E-07	3.48	0.00	0.03	3.3	n.a.	n.a.	n.a.
mmu-miR-708_st	mmu-miR-708	mir-708	*Chrom* 7 (+)	6.41E-06	-1.74	0.05	0.09	0.47	n.a.	0.27	0.35
mmu-miR-744_st	mmu-miR-744	mir-744	*Chrom* 11 (-)	(1.89E-07)	-1.69	0.05	0.09	0.68	n.a.	n.a.	n.a.
mmu-miR-877-star_st	mmu-miR-877*	mir-877	*Chrom* 17 (-)		-2.8	0.01	0.03	0.47	n.a.	n.a.	n.a.
mmu-miR-877_st	mmu-miR-877	mir-877	*Chrom* 17 (-)	(2.52E-07)	-1.36	0.1	0.15	0.5	n.a.	n.a.	1.68
mmu-miR-883b-3p_st	mmu-miR-883b-3p	mir-883	*Chrom* X (-): mir-463 *|* mir-741 *|* mir-471 *|***mir-883b***|* mir-883a *|* mir-742	6.72E-11	4.13	0.00	0.03	6.57	n.a.	3.15	1.94
mmu-miR-99b-star_st	mmu-miR-99b*	mir-99	*Chrom* 17 (+): **mir-99b***|* let-7e *|***mir-125a**		-3.6	0.00	0.03	0.38	n.a.	2.33	2.03
mmu-miR-100_st	mmu-miR-100	mir-99	*Chrom* 9 (+): **mir-100***|* let-7a-2	(1.13E-07)	-1.97	0.03	0.07	0.56	n.a.	n.a.	1.68
mmu-miR-1196_st	mmu-miR-1196		*Chrom* 14 (-)		-3.74	0.00	0.03	0.27	n.a.	n.a.	n.a.
mmu-miR-714_st	mmu-miR-714			2.06E-06	-2.29	0.02	0.05	0.35	n.a.	9.4	6.12
mmu-miR-805_st	mmu-miR-805		Death miR entry: maps to the Mt genome and overlaps a Mt tRNA sequence.	2.75E-06	-2.26	0.02	0.05	0.58	n.a.	n.a.	2.04
mmu-miR-709_st	mmu-miR-709		*Chrom* 18 (+)	2.87E-08	-1.85	0.04	0.08	0.58	n.a.	n.a.	1.72
mmu-miR-705_st	mmu-miR-705		*Chrom* 6 (-)		-1.8	0.04	0.08	0.4	n.a.	2.35	n.a.
mmu-miR-1187_st	mmu-miR-1187		*Chrom* 5 (-)		-1.76	0.05	0.09	0.38	n.a.	n.a.	n.a.
mmu-miR-699_st	mmu-miR-699		Death miR entry: appears to be a fragment of RNase MRP RNA (Paul Gardner pers comm)		-1.58	0.07	0.11	0.52	n.a.	2.75	1.57
mmu-miR-712_st	mmu-miR-712		mir-712a and mir-712b sequences map to the same genomic locus in mouse genome assembly NCBI36		-1.38	0.1	0.14	0.41	n.a.	8.61	2.91
mmu-miR-1192_st	mmu-miR-1192		*Chrom* 19 (+)		3.55	0.00	0.03	5.61	4.17	2.85	n.a.
mmu-miR-719_st	mmu-miR-719		*Chrom* 14 (-)	9.32E-08	4.51	0.00	0.03	5.79	n.a.	n.a.	n.a.
mmu-miR-706_st	mmu-miR-706		*Chrom* 6 (-)	1.79E-08	5.19	0.00	0.02	4.98	2.66	n.a.	n.a.

List of 103 differentially expressed microRNAs (77 repressed and 26 induced) identified by means of pair-wise SAM contrasts (FDR = 0.1) comparing the microarray-generated miRNA transcriptional profile of 12-day 4OHT Rasless MEFs with that of control untreated K-Ras^lox^ MEFs. The last three columns on the right show data corresponding to independent pair-wise comparisons between 6-day 4OHT-treated MEFs and untreated K-Ras^lox^ control MEFs, and comparisons between BRAF-rescued or MEK1-rescued cells and Rasless MEFs (all three also at the same FDR = 0.1). The differentially expressed miRNAs are identified by the Affymetrix *miRNA probeset ID*, *miRNA name*, *miRNA family*, *Chromosome location* and *miRNA cluster* to which they belong (using updated data from the miRBase database (http://www.mirbase.org/). All miRNA members of the same cluster that show concomitant differential expression in Rasless cells are written in bold. The *Genecodis prediction* column shows the p-values of statistical significance for predictions of the indicated miRNAs made by Genecodis analysis of the list of repressed or induced (in parenthesis) mRNAs of Rasless cells (Additional file 1: Table S1). *d-value* quantifies the degree of overexpression (positive values) or repression (negative values) and is a parameter measuring the statistical distance separating the calculated expression value of each gene probeset from the null hypothesis (no-change). *p-value* is a statistical measurement indicating the probability of random expression for that probeset. *q-value* is the estimated FDR at the highest p-value for which the probeset would be statistically significant. *R-fold* is a measurement of the fold-change of a probeset in the collection of the microarrays provided by the SAM algorithm. Independent confirmation of the microarray-generated *R-fold* values of differential expression for several randomly selected miRNAs was obtained by means of qRT-PCR using specific primers which generated the data indicated by the # symbols and the actual *fold* values included in the square [parenthesis]. “n.a.”: not applicable at FDR = 0.1. In some cases, the BRAF- and MEK1-rescued samples showed the opposite transcriptional behavior (reversion) in comparison with the Rasless samples but their respective R-fold parameters are still shown here as “n.a.” because the corresponding FDR value of the overall contrasts was higher than 0.1 under the experimental conditions used.

The functional significance of our microarray-based miRNA profile with regards to generation/maintenance of the Rasless status was further supported by the observation of a remarkable accumulation of members of specific miRNA families (sharing seed recognition sequence) and/or clusters (sharing genomic location). In particular, this differential miRNA expression profile identified at least 15 distinct miRNA families, including two or more individual miRNA species (Table 1). In particular, the pool of repressed miRNAs of Rasless cells included at least 10 different members of the mir-17 family, 6 members of the mir-467 family, 3 members of the let-7 and mir-25 families, and 2 members each of a number of distinct miRNA families such as mir-181, mir-125, mir-132, mir-214, mir-221, mir-423, mir-877 and mir-99. Additionally, at least 3 different miRNA families (mir-30, mir-368 and mir-27) were also identified that included 2 or more of their members in this list of upregulated Rasless miRNA species (Table 1).

It is also noteworthy that a large percentage of the differentially expressed miRNAs of Rasless cells were concentrated in specific genomic locations, frequently sharing their physical location within the same miRNA cluster or the same chromosome. Table 1 identifies at least 17 different miRNA clusters holding two or more differentially expressed miRNAs of Rasless cells. The physical proximity shared by a large percentage of differentially expressed miRNAs of Rasless cells, together with the fact that all members of a given cluster often share common regulatory mechanisms, is also supportive of the notion that the miRNA profile identified in Table 1 may be mechanistically and/or functionally relevant as regards the generation or maintenance of the Rasless status.

Analysis of functional annotations available in the scientific literature and miRNA databases indicated that the majority of miRNAs listed in Table 1 can be classified as “oncomirs”, since they have previously been reported to contribute to the development of tumorigenic processes [44-46]. Among the repressed miRNAs, we found 3 members of the let-7 family (involved in control of cell proliferation and regulation of expression of Ras oncogenes [47] and associated with the development of lung tumors [48,49]) and, in particular, 10 different members of the mir-17 family (miR-17, miR-18a, miR-20a, miR-20b, miR-93, miR-106a and miR-106b) and 3 different members of the mir-25 family (miR-92a, miR-25 and miR-92b) which are distributed among three different clusters (miR-17 ~ 92, miR-106a ~ 363 and miR-106b ~ 25) located, respectively, in mouse chromosomes 14, X and 5 (Table 1).

The involvement of the miR-17 ~ 92 cluster in human cancer has been known for a long time [50]. In particular, this cluster was proposed as a diagnostic tool in large B-cell malignancies [51] and different reports have described its overexpression or amplification in various cancer types including B cell lymphomas, rhabdomyosarcomas, lung cancer, and liposarcomas [49,52-56]. The oncogenic potential of the components of the miR-106a ~ 20b ~ 363 cluster and their involvement in T-cell leukemia [57], breast cancer [58] and gastrointestinal tumors [59,60] has also been described. The involvement of members of miR-106b ~ 25 cluster in prostate [61], gastric [62], hepatic [63,64] and glioblastoma multiforme tumors is also documented [65]. The members of the miR-212 ~ 132 cluster are among the most strongly downregulated miRs in Rasless cells (Table 1) and previous reports have described their functional contribution to pancreatic [66] and non-small cell lung cancer [67]. The downregulated miR-155 (Table 1) has also been previously linked to B-cell-related cancers and shown to be upregulated in pediatric Burkitt’s and Hodgkin lymphomas [44,68]. Finally, the downregulated components of the miR-222 ~ 221 cluster (Table 1) are amplified in papillary thyroid carcinomas [69] and the components of the miR-183 ~ 182 cluster (Table 1) have been linked to development of medulloblastomas [70], lung cancer [71] and gliomas [72].

The pool of upregulated miRNAs identified in Rasless cells is less extensive and is limited to components of the mir-27 and mir-30 families and, in particular, the individual miR-355 and miR-181a which show the highest R-fold overexpression values in Rasless cells (Table 1). Different reports have identified miR-335 as a prognostic indicator in gastric cancer [73] and gliomas [74], whereas the overexpressed miR-181a has been reported to modulate T cell sensitivity and selection [75] and to contribute to human myeloid leukemia [76]. Additionally, the members of the mir-27 and mir-30 families have been shown to play pro-angiogenic roles in tumors [77,78] and, in particular, the individual miR-30c has been reported to directly target the 3′UTR region of K-RAS in hereditary breast cancers [79].

All in all, the list of related tumors and canonical targets identified in the scientific literature for each differentially expressed miRNA listed in Table 1 may provide significant functional clues regarding the specific mechanisms and causal relationships linking the miRNA profile of Rasless cells (Table 1) to the generation/maintenance of the transcriptomic mRNA profile and phenotype of Rasless cells.

### Reversibility of the microRNA expression profile of Rasless cells and inferred mechanistic implications

Further confirmation of the functional significance of our microarray-generated profile of differential miRNA expression is provided by the observation that most alterations of miRNA expression identified in the growth-arrested Rasless cells were fully reversed, in exactly the opposite direction of induction or repression, in the proliferating, BRAF-rescued and MEK1-rescued MEFs (Table 1). Interestingly, the differential expression of most of these “reversible” miRs was predicted by Genecodis with very high statistical significance from the profile of induced and repressed mRNAs occurring in Rasless cells (see *“Genecodis prediction”* column*,* Table 1). Indeed, those reversals affected more than 55% of all differentially expressed miRNAs in Rasless cells, and frequently affected all members of specific miRs families or clusters (Table 1). Thus, it was particularly noticeable that all members of the mir-17 and mir-25 families (involving the 3 separate clusters miR-17 ~ 92, miR-106a ~ 363 and miR-106b ~ 25) showed opposite transcriptional behavior between the proliferating, BRAF- or MEK1-rescued cells (upregulated) and the growth-arrested Rasless cells (repressed) (Table 1). A similar reversal was also observed with all miRs located in specific clusters, such as miR-212 ~ 132 (mir-132 family), miR-183 ~ 182 or miR-222 ~ 221 (mir-221 family). Reversal from downregulated (in Rasless cells) to upregulated (in both BRAF- and MEK1-rescued cells) was also observed in a number of individual miRs, including miR-155, miR-29a, miR-31, miR-193, miR-503, miR-714 and miR-712. Quantitatively, the miRNAs of the miR-183 ~ 182, miR-222 ~ 132, miR-17 ~ 92 and miR-106a ~ 363 clusters, as well as in the individual miR-155 and miR-29a, showed the highest rebound from downregulation to upregulation (Table 1). In contrast, miR-23b and miR-27b (belonging to the same cluster) were upregulated in Rasless cells and clearly downregulated in both BRAF- and MEK1-rescued MEFs (Table 1). In particular, miR-335 and miR-365 were the most highly upregulated individual miRs identified in Rasless cells that were also simultaneously detected as being downregulated in both BRAF- and MEK1-rescued MEFs (Table 1). Additionally, miR-27a and the miR-30a, miR-30b and miR-30c (components of the mir-30 family) were also upregulated in Rasless cells but transcriptional reversal was only detectable for miR-27a and miR-30a in MEK1-rescued cells. In other cases, such as the downregulated let-7 family members or the upregulated miR-10b, miR-129, miR-215, miR-487b and miR-883, no reversal of their transcriptional pattern was detected (Table 1).

Direct visual evidence of the reversibility of the miRNA profile of Rasless cells is provided by the heatmap in Figure 5B, depicting a multiclass comparison resulting from hierarchical clustering of the microarray-based miRNA profiles of control, Rasless, and BRAF- or MEK1-rescued MEF clones. This dendrogram shows a clear discrimination among three main vertical branches corresponding to (i) Control, proliferating K-Ras^lox^ MEFs, (ii) the BRAF- or MEK1-rescued MEFs and (iii) the non-proliferating, Rasless cells (Figure 5B). Remarkably, the profiles of the BRAF- and MEK1-rescued MEFs showed an expression pattern that was antagonistic to that of Rasless cells, thus regaining a miRNA profile that approached that of the original proliferating control K-Ras^lox^ MEFs (Figure 5B).

### Mechanistic implications inferred from the reversible miRNA expression patterns of Rasless cells

**Figure 6:**
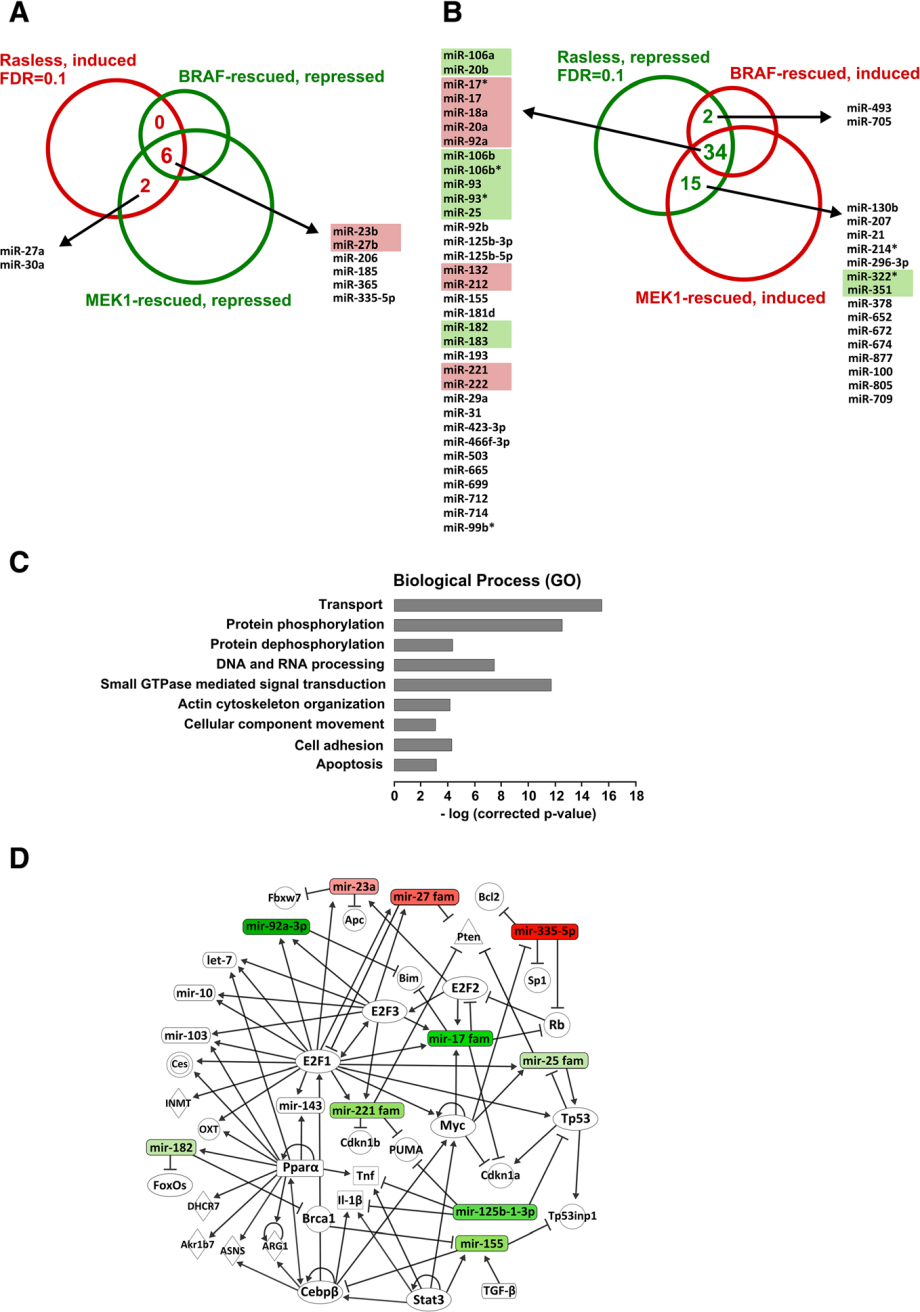
**Reversibility of the microRNA expression profile of Rasless cells and inferred mechanistic implications. (A, B)** Overlap of opposing differential miRNA expression profiles between Rasless MEFs and BRAF- and MEK1-rescued MEFs. **(A)** Venn diagram showing numbers of shared differentially expressed miRNAs (FDR = 0.1) that were simultaneously detected as induced in Rasless MEFs (pair-wise comparison with control MEFs, FDR = 0.1) and as repressed in both BRAF- and MEK1-rescued MEFs (pair-wise comparisons to Rasless MEFs, FDR ≤ 0.19). **(B)** Venn diagram showing numbers of shared differentially expressed miRNAs that were simultaneously repressed in Rasless MEFs (pair-wise comparison with control MEFs, FDR = 0.1) and induced in BRAF- and MEK1-rescued MEFs (pair-wise comparisons with Rasless MEFs, FDR ≤ 0.19). Red: transcriptional induction. Green: transcriptional repression. Different families and clusters encompassing the 6 upregulated and 34 downregulated miRs identified are highlighted in separate colors. **(C)** GO Biological Process categories assigned (p-value < 10^-4^) by the StarBase (sRNA Target Base) functional annotation tool to the panel of 40 differentially expressed miRNAs identified in panels **A** and **B**. Bar length quantifies degree of statistical significance for each functional category. **(D)** Ingenuity Pathways (IPA) software analysis identifying networks of biologically significant functional interactions among the 40 differentially-expressed miRNAs identified in panels A and B and a variety of checkpoint sensors and cell-cycle regulators including Myc, Pten, Rb, Tp53 and Cdkns. Graphic outcome adapted to highlight the most relevant individual miRs or miR families and the key regulators involved in the interactions. **mir-17 fam** (miR-17, miR-18a, miR-20a, miR-20b, miR-106a, miR-106b, miR-93), **mir-27 fam (**miR-27a, miR-27b) and **mir-221 fam** (miR-221, miR-222) designate those cases where multiple members of the same mir family participate in the indicated regulatory interaction. Green: downregulation; Red: upregulation. Color intensity graded according to d-value of each miRNA entry in Table 1.

We attempted to identify the most salient reversible miRNA alterations with regards to the generation and/or maintenance of the Rasless status by means of Venn diagrams identifying miRs from Table 1 that showed an exactly opposite pattern of differential expression (repression and induction, respectively) between Rasless cells and both the BRAF-rescued and MEK1-rescued proliferating MEFs (Figure 6A, B). This approach identified at least 34 distinct repressed miRs and 6 overexpressed miRs of Rasless cells fulfilling that condition (Figure 6A, B; Table 1; Figure 5B). This particular group of 40 miRs potentially represents the core of most functionally relevant miRs with regards to the mechanisms involved in the generation and/or reversal of the Rasless phenotype.

An overall view of the most significant cellular functional categories predicted [80] to be affected by this pool of Rasless miRNAs is represented in Figure 6C. Interestingly, this analysis recognized a set of general GO functional categories that are highly coincident with those previously identified in a similar analysis of the pool of reversible mRNAs of Rasless cells (Figure 3C). Among others, these included the following: Transport (p-value 3.42E-16), Protein phosphorylation (p-value 3.49E-13), Small GTPase-mediated signalling (p-value 1.94E-12) and DNA/RNA processing (in particular, regulation of transcription, DNA-dependent, p-value 3.51E-08) (Figure 6C).

Focusing on the identity of the individual miRs in this group, it was striking to observe a significant enrichment in miRs belonging to a short list of specific miR clusters and families (Figure 6A, B and D) characterized by their shared ability to target several specific cellular regulators participating in modulation of cell cycle progression/arrest checkpoints, response to DNA damage stress and apoptosis*.* It is likely that the pleiotropic sum of all these different, miR-based modulatory interactions (going in opposite directions in Rasless cells as compared to BRAF- or MEK1-rescued cells) may contribute, at least in part, to the growth arrest/proliferation processes involved in generation and/or reversal of the Rasless phenotype. In this regard, the reversal of the expression patterns of all members of the highly related mir-17 and mir-25 families (repressed in Rasless cells and distributed among 3 defined clusters miR-106a ~ 363, miR-17 ~ 92 and miR-106b ~ 25) is particularly striking (Table 1, Figure 6). Although some have been cited as being involved in aging processes [81], most members of the mir-17 and mir-25 families have been implicated in cell cycle control and regulation of tumor development through a variety of mechanisms involving the specific targeting of modulators and checkpoint sensors for processes of cell cycle progression/arrest, DNA damage stress response and apoptosis, including in particular Rb, E2F, p21 and p53. Thus, a defined set of 3 miRs, including miR-17 and the miR-106a ~ 20b cluster components has been identified as a regulatory intermediate for coordinating p63 with MAPK signaling through the targeting of different signaling molecules including Rb, p21 and multiple MAPKs [82]. Overexpressed miR-106a alone has been shown to downregulate RB in colorectal cancer [59] and T cell leukemia [57] as well as to inhibit apoptosis by targeting FAS in gastric cancer [60], whereas miR-20b has been reported to target pro-angiogenic modulators in breast cancer cells [58].

The mechanistic relevance of the miR-17 ~ 92 cluster with regards to cell cycle regulation is also clearly established [44,49,50] since this cluster is recognized as the central element of a complex regulatory network that tightly controls proliferative signals in a variety of biological contexts. Specifically, this polycistronic miR-17 ~ 92 cluster is known to carry out pleiotropic functions modulating proliferation, apoptosis and survival in different cellular contexts *via* its participation in a complex networked Myc-miR-17 ~ 92-E2F genetic circuit in which Myc regulates expression of the miR-17 ~ 92 cluster components and, in turn, these components of the cluster negatively target and regulate expression of E2F family members [50,83,84]. This miR-17 ~ 92-mediated regulatory circuitry [85-88], which targets the Rb pathway [49,89] through modulation of E2F factors [84-87], is highly consistent with our experimental observation of miR-17 ~ 92 downregulation in growth-arrested Rasless cells and upregulation in BRAF or MEK1-rescued MEFs (Table 1, Figure 6A, B), as well as with the detection of disappearance of a number of E2F targets in Rasless cells and their re-appearance in BRAF- and MEK1-rescued cells (Figure 4B, C). Whereas the Rb-E2F pathway appears to be the primary target of miR-17 ~ 92, this cluster has also been reported to modulate other targets capable of modulating cell cycle progression or arrest through other pathways. Of particular interest in this regard is a report showing that synthetic lethality between Rb, p53 and Dicer or miR17 ~ 92 in retinal progenitors suppresses retinoblastoma [90], thus adding another mechanistic connection between Rb-dependent pathways and p53-dependent pathways to the variety of pleiotropic effects of this cluster with respect to the control of cell cycle progression and arrest. Such pleiotropic mode of action is also supported by a report indicating that this cluster acts by upregulating p21^Cip1^ in retinoblastomas [89], and by our experimental detection of enhanced levels of p21 in Rasless cells (Figure 4; Additional file 1: Table S1).

The overlapping members of the miR-106b ~ 25 cluster and the mir-25 family also display opposite patterns of expression in Rasless cells and in BRAF- and MEK1-rescued cells (Table 1, Figure 6A, B), and analysis of their canonical targets and biological effects offers additional mechanistic explanations for the reversible proliferative phenotypes of Rasless MEFs. In particular, the members of the miR-106b ~ 25 cluster have been shown to interfere with cell survival and apoptosis in different tumor systems [61,63,91]*via* targeting of a variety of modulators of cell cycle progression or checkpoint functions, thus providing a mechanistic basis for cross-talk between Rb- and p21- and PTEN-dependent pathways [92]. Thus, the miR-106b ~ 25 cluster has been shown to target PTEN in prostate tumors [61] or E2F1 in hepatocellular carcinoma [63] and gastric tumors, where it impairs TGFβ-dependent cell cycle arrest and apoptosis [62,91]. In particular, the members of this cluster have been reported to target and downregulate p21/Cdkn1a levels in multiple tumour systems [93-95], an observation highly consistent with our experimental observation of increased levels of Cdkns (p21, p15, p16) in Rasless cells (Additional file 1: Table S1, Figure 4B-D). In addition, miR-25 alone has also been reported to target apoptotic modulators in different tumor types [64,65,96]. Of interest in this regard is the recent identification, in glioblastoma multiforme, of a miR/TP53 feedback autoregulatory circuit involving expression of p53, E2F1 and Myc to regulate expression of miR-25, which in turn controls p53 accumulation [65], most likely *via* direct targeting of the 3′UTR region of TP53 [97].

The parallel transcriptional behavior of the components of clusters miR-212 ~ 132, miR-222 ~ 221 and miR-183 ~ 182 (Table 1) adds further support to the notion of a miR-based, coordinated regulatory circuitry involved in cross-talk between pro- and anti-proliferative and apoptotic/survival or DNA damage response pathways that may be responsible, at least in part, for the arrested or proliferative phenotypes of Rasless cells and the BRAF- or MEK1-rescued cells. Accordingly, recent reports have shown the ability of the two miR-212/miR-132 family members to directly target Rb in pancreatic tumors [66] and of miR-221/miR-222 to favor tumor progression through targeting of the pro-apoptotic PUMA [98] or the tumor supressor PTEN, thus activating the Akt pathway [99,100]. The known cellular targets of the miR-183/miR-182 cluster also establish a potential functional connection with DNA damage response pathways in our Rasless/rescued MEFs, since both components of the cluster have been linked to stress-induced premature cellular senescence (SIPS) responses in primary fibroblasts [101] and miR-182 alone has also been described to target BRCA1 in breast cancer cells [102]. Interestingly, miR-181d exhibits a parallel transcriptional pattern to that of the three clusters mentioned above and is known to directly target K-Ras and Blc-2 in gliomas, an observation suggesting additional functional links between the K-Ras-related PI3K/Akt and MAPK/ERK pathways that would be consistent with the disappearance of K-Ras in Rasless cells [103].

miR-335 is the most highly overexpressed miR in Rasless cells and its transcriptional pattern is also completely reversed in both the BRAF- and MEK1-rescued MEFs (Table 1). Recent reports have demonstrated that miR-335 directly targets Rb in meningiomas [104] as well as different genes of the non-canonical TGFβ signalling pathway in neuroblastomas [105]. Interestingly, mechanistic analysis of cancer cell lines has shown that direct targeting of Rb by miR-335 also establishes a proximal connection to the p53-dependent stress response since, by altering the Rb levels, miR-335 activates the p53 pathway to limit cell proliferation after DNA damage [106]. Consistent with this, miR-335 has also been reported to be crucial for the BRCA1 regulatory cascade by targeting upstream components of the BRCA1 regulatory cascade with impact on key cellular functions such as proliferation and apoptosis [107]. These observations strongly suggest that miR-335 may play a significant role in controlling proliferation by balancing the activities of the Rb and p53 tumor suppressor pathways.

Our observations suggest that this defined set of 40 “reversible”, differentially expressed miRNAs (Table 1, Figure 6) is mechanistically relevant for the generation/maintenance and reversal of the Rasless phenotype. It is remarkable in this regard that the functional targets of this particular core of reversible miRs usually include a short list of specific targets such as Rb, E2F, p53, Cdkns (1a, 2a, 2b) or a few other apoptotic or checkpoint modulators (Figure 6D) known to act in a defined group of cross-talking cellular pathways with impact on processes of cell cycle progression/arrest, apoptosis/survival, or DNA damage stress responses. The notion of interdependent mRNA-miRNA transcriptional profiles controlling the Rasless phenotype is also supported by the observation that most transcriptional alterations of these miRs were predicted, with highly significant p-values (Table 1), by Genecodis analysis of the list of differentially expressed mRNAs of Rasless cells (Additional file 1: Table S1). The disappearance of many E2F targets, or the somewhat unexpected upregulation of Cdkns (p21, p16, p15) in Rasless cells [19] (Additional file 1: Table S1; Figure 4), are also highly consistent experimental observations supporting such a notion.

All these considerations raise the interesting hypothesis that the set of transcriptionally reversible miRs identified in this report may constitute the core of a miR-based regulatory circuitry focused around a few specific targets such as Rb, E2F or p53 and Cdkns (p21, p16, p15) capable of modulating interplay among pathways controlling proliferation, survival and DNA damage stress responses that may account for the mechanisms responsible of the growth/arrest phenotype exhibited by Rassles or rescued MEFs. Interestingly, our data uncovered specifically the Myc/Rb/E2F axis and the Cdkns/p53 axis as the two main signaling contributors to this regulatory circuitry. Regarding the first axis, E2F proteins and targets are controlled by Rb, and Rb loss is known to override the requirement for downstream ERK signalling for cell proliferation [30,40,41]. In the second axis, p21 is known to be a transcriptional target of p53 [42,43]. Therefore, a prediction directly derived from such hypothesis would be that reversion of the transcriptional patterns of downregulation or upregulation of mRNA and miRNA identified in Rasless cells may lead to a similar reversal of the growth-arrest phenotype, as observed in BRAF- or MEK1-rescued MEFs. Such a reversal could be tested experimentally in Rasless cells either by the introduction of specific antagomIrs or, more directly, through direct knockout or the knockdown of some of the critical core modulator targets identified in this study, such as Rb, p53 or the Cdkns (p21, p16, p15). Our preliminary analysis of the transcriptome of Rasless MEFs that recovered their proliferative ability after silencing of Rb *via* the introduction of specific shRNA constructs [19] appears to support this hypothesis (Additional file 7: Figure S2). Indeed, the patterns of differential expression of mRNAs and miRNAs in these shRb-rescued cells were highly reminiscent of those of BRAF- and MEK1-rescued cells, with the most significant components of their mRNA and miRNA compartments showing transcriptional behavior opposite to that seen in Rasless cells (compare panels A and B of Additional file 7: Figure S2 to Figure 3 and Figure 6, respectively).

## Conclusions

In this report we characterized the transcriptional profiles of the populations of messenger RNA and microRNA that are differentially expressed in growth-arrested Rasless fibroblasts lacking the three canonical Ras family members. Restoring the proliferative ability of those cells after ectopic expression of activated BRAF or MEK1 resulted in the reversal of a large proportion of the transcriptional mRNA and miRNA alterations identified, indicating that the altered mRNA and miRNA expression patterns are functionally interrelated and specifically associated with the disappearance of the Ras proteins in Rasless cells.

Classification into functional categories of the lists of differentially expressed mRNAs and miRNAs supported the functional relevance of the (absent) canonical *ras* genes for a number of cellular functions, including DNA/RNA processing and metabolism, cellular transport processes, metabolite processing and, in particular, positive and negative control of cell cycle progression, programmed cell death and DNA damage response. Specifically, the list of differentially expressed mRNAs of Rasless cells involved repression of a large number of cell cycle-related genes, including cyclins, cyclin-dependent kinases, and E2F transcription targets, as well as induction of cyclin-dependent kinase inhibitors (Cdkns). Consistent with this, flow cytometric analysis of Rasless cultures identified a predominant blockade at the G1 phase of the cell cycle.

Analysis of the profile of differential miRNA expression in Rasless cells identified the reversible, altered expression of a distinct list of interrelated oncomiR families and clusters including, among others, downregulation of all members of the mir-17 and mir-25 families and upregulation of miR-335. Remarkably, the gene targets for most of those miRs are concentrated around a short list of signaling modulators, including in particular, Rb, E2F, p53, several Cdkns and a few other apoptotic modulators. Since these targets are known modulators of cross-talk signaling pathways regulating cell cycle progression/arrest, apoptosis/survival or response to cellular stress such as DNA damage, our observations suggest that the reversible Rasless phenotype may be a pleiotropic result of the interplay among several, distinct pro-and antiproliferative signaling and stress response pathways regulated by the differentially expressed mRNAs and miRNAs identified. This hypothesis is based on the observation of preferential targeting of Myc-Rb-E2F and Cdkns (p21, p16, p15)-Tp53 dependent pathways by the differentially expressed mRNAs and miRNAs identified in Rasless cells, and it challenges current hypotheses for Ras-driven cell cycle progression based exclusively on induction of CcnD synthesis. This hypothesis would also predict that reversing the transcriptional patterns of mRNA and miRNA differential expression of Rasless cells may lead to a parallel restoration of their proliferative abilities, similar to what happens in BRAF- or MEK1-rescued MEFs. We suggest that the introduction of specific antagomIrs or direct silencing of some or all of the critical miR target protein modulators identified in this study, such as Rb, E2F, Cdkns (p21, p16, p15) or p53, may be an adequate experimental approach to directly test such a possibility. Preliminary work introducing specific shRNA constructs for Rb into Rasless cells has indicated that silencing Rb expression rescues their proliferative ability [19] and significantly restores the normal mRNA and miRNA transcriptional profiles (Additional file 7: Figure S2) in those cells.

## Methods

### Cell culture

All cell lines used here were mouse embryonic fibroblasts (MEFs) harboring the same basic genotype (H-Ras^-/-^;N-Ras^-/-^;K-Ras^lox/lox^;RERT^ert/ert^) [19]. Cell lines designated DU315-6 and DU244-1 were used as K-Ras^lox^ controls for experiments involving the induction of the Rasless phenotype under 4OHT treatment. The cell clones designated LG7-6 had the same genotype and carried a hygromycin-resistance vector expressing a BRAF^CAAX^ construct. The cell lines designated JU10-2 served as control for experiments with LG7-6 lines since they carried the same empty hygromycin resistance vector. The cell lines designated MCL1-6 harbored a puromycin resistance vector expressing an MEK1^Q56P^ construct and cell lines MCL23-1 served as controls since they bore the same puromycin resistance empty vector. Cultures were grown in a humidified CO_2_ (5%) atmosphere at 37°C, in Dulbecco’s modified Eagle’s medium (DMEM; Gibco) supplemented with fetal bovine serum (10% FBS; Hyclone, Logan, Utah, USA), glutamine (2 mM), penicillin (100 U/ml) and streptomycin (100 mg/ml). Hygromycin (200 μg/ml, Sigma-Aldrich) or puromycin (2 g/ml, Sigma-Aldrich) was also added as appropriate to MEF cultures expressing BRAF^CAAX^ or MEK1^Q56P^, respectively. For tamoxifen induction, cultures were treated as appropriate with 4-hydroxy-tamoxifen (4OHT, H7904, Sigma-Aldrich) for 6 or 12 days at final concentration 0.6 μM to promote Cre-induced disruption of the K-Ras locus. Subconfluent cultures of untreated or 4OHT-treated cell lines were used for total RNA, miRNA and protein extractions.

Cell-proliferation assays were performed using MTT [3-(4.5-dimethylthiazol-2-yl)-2.5-diphenyltetrazolium bromide, Sigma-Aldrich, 5 μg/μl]. The absorbance (at 570 nm wavelength) of quadruplicate samples for each experimental condition was measured every 24 hours for 3 days using an Ultra Evolution Microplate Reader (TECAN).

Sca1 downregulation studies were performed by transducing control MEFs with lentiviral particles (MISSION® Lentiviral Transduction Particles, SHCLNV, Sigma-Aldrich) harboring either specific Sca1 shRNA constructs (shRNA-Sca1 cell line), or non-targeting shRNA control constructs (shRNA-NT cell line) to rule out any off-target effects. Puromycin (1.5 μg/ml) was used to select the infected cells and the TCRN0000100120 construct was found to be the most effective Sca1 shRNA. For Sca1 expression studies, cells were incubated with JAK inhibitor I (420099, Millipore) (3 μM) for 6, 24 or 48 hours.

### RNA isolation and microarray hybridization

For mRNA expression analyses, total RNA was isolated using the TRIzol® reagent and protocol as described by the manufacturer (Ambion, Life Technologies). RNA samples were purified using the RNeasy® Mini Kit (Qiagen) and their concentration, purity and integrity were measured on an Agilent 2100 Bioanalyzer (Agilent Technologies). RNA was then used to synthesize complementary RNA (cRNA) probes for hybridization to the Affymetrix GeneChip® Mouse Genome 430 2.0 Array that was carried out as described previously [20,21].

For miRNA studies, total RNA was extracted from two 10-cm culture dishes per individual sample using the mirVana™ miRNA isolation kit (Ambion) according to the manufacturer’s protocol. RNA integrity was assessed using an Agilent 2100 Bioanalyzer (Agilent Technologies). Briefly, 1000 ng of total RNA were labeled using the Flash Tag Biotin HSR Labeling kit (Genisphere, P/N HSR10FTA) according to the manufacturer’s instructions. Hybridizations were performed using the GeneChip miRNA Array (Affymetrix) according to protocols from Affymetrix. Washing and scanning were performed using the Affymetrix *GeneChip* System (GeneChip Hybridization Oven 640, GeneChip Fluidics Station 450 and GeneChip Scanner 7G).

### Microarray data analysis: normalization, differential expression and clustering

To ensure statistical significance, several separate microarray hybridizations and independently extracted mRNA or miRNA samples were used in all cases for the characterization of each genotype and/or experimental condition under study. The sample set used in this report for mRNA expression studies included 27 independent hybridizations corresponding to 14 controls, 7 Rasless, 3 BRAF-rescued and 3 MEK1-rescued samples. The sample set for miRNA expression analysis included 24 independent hybridizations corresponding to 8 controls, 8 Rasless, 4 BRAF-rescued and 4 MEK1-rescued cell lines.

Data analysis was carried out using the RMA [108] and SAM [109] algorithms, as previously described [20,21]. For analyses of mRNA differential expression, a FDR value of 0.01 was applied, whereas in the studies of differential expression of miRNA, generally an FDR value of 0.1 was used. Following the identification of the differentially expressed probesets (corresponding to mRNAs or miRNAs), the corresponding matrix of expression values for all the microarray hybridizations performed were analysed using the *hclust* clustering algorithm, implemented in R [110]. This algorithm performs hierarchical cluster analysis with complete linkage to find similarities between probesets based on their expression values in the different chip microarrays analyzed. The algorithm classifies the probesets in correlated groups showing similar expression profiles or expression signatures.

### Functional analysis of microarray data

For functional analysis of the lists of differentially expressed genes identified in our studies, we used the GeneCodis (Gene Annotation Co-occurrence Discovery) software tools (http://genecodis.dacya.ucm.es) to find combinations of co-occurrent functional annotations within the components of a given gene list with respect to a reference list [111,112]. The significance of the annotations was calculated using a hypergeometric statistical test with FDR p-value correction [113], using the mouse genome as reference. Functional annotations were obtained, as indicated in each case, by referral to either the Gene Ontology (GO) (http://www.geneontology.org), KEGG pathways (http://www.genome.ad.jp/kegg/pathway.html), TRANSFAC® (vers. 7.4, http://www.gene-regulation.com/), or miRBase (http://www.mirbase.org/, Source-version miRanda 3.0) databases. Redundancies in the lists of GO categories generated by the software were submitted to further manual curation in order to focus on the most general biological functions and cellular processes, as seen in Additional file 2: Table S2, Additional file 3: Table S3 and Additional file 5: Table S5).

Functional analysis of the lists of differentially expressed miRNAs was performed using the StarBase public platform (http://starbase.sysu.edu.cn/) and web-based functional annotation tools such as miRGO or miRPathway, which respectively identify enriched GO terms and KEGG pathways associated with the predicted miRNA target genes by overlapping with the experimental CLIP-Seq data [80]. Statistical significance of the enrichment data was estimated by means of confidence p-values calculated by applying the hypergeometric test and Bonferroni correction. Only corrected p-values < 10^-4^ were taken into consideration in this work. The Ingenuity Pathway Analysis (IPA) commercial software (Ingenuity® System, http://www.ingenuity.com) was also used to explore miRNA regulatory connections and identify potential networks of genes and miRNAs (targets and regulators) in a context of biological significance within the set of differentially expressed miRNAs shared by both the BRAF and MEK1-rescued cells.

When required, overlapping among the various sets of differentially expressed elements identified in our studies was characterized by means of Venn diagrams generated with the *Venny* web-based application (http://bioinfogp.cnb.csic.es/tools/venny/index.html) [114].

### Real-time PCR

Total RNA was extracted from either untreated or 4OHT-treated (6 and 12 days) K-Ras^lox^ cells, as well as BRAF- and MEK1-rescued cell lines using the mirVana™ miRNA isolation kit (Ambion) according to the manufacturer’s protocol. RNA integrity was also evaluated with an Agilent 2100 Bioanalyzer (Agilent Technologies). Quantitative RT-PCR (qRT-PCR) analyses were performed using the miRCURY LNA™ Universal RT microRNA PCR System (Exiqon) following the supplier’s intrstructions. Briefly, 5.5 ng of total RNA was reverse-transcribed with miRNA specific primers and Transcriptor Reverse Transcriptase. Then, cDNA from each sample was used as a template for the qPCR reaction (in triplicate, per sample and miRNA) using SYBR Green master mix, miRNA specific LNA™ PCR primer, and Universal PCR primer (Exiqon). The primer sequences are available at http://www.exiqon.com/mirna-pcr. miRNA expression levels were measured using the iCycler termociclator (Bio-Rad) and analyzed with the iQ5 2.1 Standard Edition Optical System Software (Bio-Rad). miR-103 was chosen for reference miRNA. Relative expression was calculated using the comparative Ct (Cycle threshold) method [115].

### Flow cytometry

Cell cycle distribution and Sca1 protein expression in cell cultures were analyzed by means of flow cytometry. Briefly, subconfluent cultures of untreated or 4OHT-treated (for 6 and 12 days) cell cultures were trypsinized and fixed in 70% cold ethanol for 2 hours. After washing with cold PBS, the cells were incubated with propidium iodide (PI) (1 μg/μl, Sigma-Aldrich) and DNase-free Ribonuclease A (25 μg/μl, Sigma-Aldrich) in the dark at room temperature with shaking for 1 hour. Fluorescence from PI-stained DNA was analyzed with a FACSCalibur Flow Cytometer (Becton Dickinson). The proportions of cells in the different cell cycle phases were quantified using the WinMDI® software (version 2.9). For Sca1 protein expression, the cells were collected by trypsinization, washed with PBS and then blocked for 10 minutes with 0.5% bovine serum albumin (BSA). Subsequently, Sca1 antibody (1:200) Sca1/Ly6A/E (PE/Cy5) (ab24880, Abcam) was added to the cell suspension and maintained in the dark for 20 minutes on ice before quantitation of the specific Sca1 fluorescence.

### Western immunoblots

Protein lysates (30–40 μg/lane) obtained and quantified as previously described [20,21] were loaded onto SDS polyacrylamide gels and the electrophoresed proteins transferred to polyvinylidene difluoride membranes (Millipore Immobilon-P) by electroblotting. Membranes blocked in Tween 20-tris-buffered saline (TTBS) (10 mM Tris–HCl (pH 8.0), 150 mM NaCl, 0.05% Tween 20) plus 2% (BSA) were incubated, as appropriate, with commercial primary antibodies diluted in 2% BSA. Antibodies from Santa Cruz Biotechnologies recognized: K-Ras (sc-30; 1:1000), Cdk1 (sc-054; 1:500), Cyclin A (sc-596; 1:1000), Cyclin E (sc-481; 1:1000), Cyclin B1 (sc-752; 1:1000), p16^INK4a^ (sc-1207; 1:1000), p21^CIP1^ (sc-397; 1:250). Antibodies from other companies reacted with: Pan-Ras (05–516, Upstate, Millipore; 1:1000 in 5% milk), β-tubulin (T5293, Sigma; 1:2000), Pcna (1170–406, Boehring; 1:500), Cdk2 (ms-459-po, NeoMarkers; 1:500), Dusp6 (ab76310, abcam; 1:500), c-Myc (#5605, Cell Signaling; 1:1000) and p15^INK4b^ (#05-430, Upstate, Millipore; 1:500). Secondary horseradish peroxidase-conjugated antibodies (Amersham Bioscience) were used throughout. Immunoblots were developed using the Enhanced chemiluminescence (ECL) and ECL plus commercial kits (Amershan Pharmacia Biotech, Piscataway) following the supplier’s recommendations.

## Availability of supporting data

All microarray hybridization data have been deposited and are available at the NCBI, Gene Expression Omnibus database (GEO accession series GSE45222, http://www.ncbi.nlm.nih.gov/geo/query/acc.cgi?acc=GSE45222).

## Abbreviations

WT: Wild type; KO: Knockout; qRT-PCR: Quantitative real time PCR; DMEM: Dulbecco’s modified Eagle’s medium; MEF: Mouse embryo fibroblast; GAP: GTPase-activating protein; GEF: Guanosine nucleotide exchange factor.

## Competing interests

The authors declare that they have no competing interests.

## Authors’ contributions

SSA and AGP carried out the experiments and data acquisition. MD and MB generated and characterized the MEF cell lines. ES, AGP and SSA carried out the bioinformatics analyses of the transcriptional data. ES coordinated the study and wrote the manuscript. All authors read and approved the final manuscript.

## Supplementary Material

Additional file 1: Table S1 Differential gene expression in Rasless MEFs. List of 3091 differentially expressed probesets (2239 different genes) identified by means of SAM contrasts (FDR = 0.01) comparing the microarray-generated transcriptional profile of Rasless MEFs to that of control, K-Ras^lox^ MEFs (already H-Ras/N-Ras double KO). Click here for file

Additional file 2: Table S2 Functional annotation of the downregulated differentially expressed genes of Rasless MEFs. The GeneCodis functional annotation tool was used on the list of downregulated genes included in Additional file 1: Table S1. Statistically significant associations of particular gene subsets to specific *Gene Ontology* (GO) functional categories designated as Biological Processes (section S2-BP), KEGG signaling pathways (section S2-KEGG), transcription factors (section S2-TF) and miRNAs prediction (section S2-miRNAs) are presented in this table. Click here for file

Additional file 3: Table S3 Functional annotation of the upregulated differentially expressed genes of Rasless MEFs. The GeneCodis functional annotation tool was used on the list of upregulated genes included in Additional file 1: Table S1. Statistical associations of particular gene subsets to specific *Gene Ontology* (GO) functional categories designated as Biological Processes (section S3-BP), KEGG signaling pathways (section S3-KEGG), transcription factors (section S3-TF) and miRNAs prediction (section S3-miRNAs) are presented in this table. Click here for file

Additional file 4: Table S4 Differentially expressed genes of Rasless cells showing reversed, opposite transcriptional pattern in both BRAF- and MEK1-rescued MEFs. List of differentially expressed genes in Rasless MEFs (93 induced and 339 repressed) that show opposite expression pattern in the transcriptional profiles of both BRAF-rescued and MEK1-rescued MEFs (generated by SAM comparison to Rasless cells at FDR = 0.01). Click here for file

Additional file 5: Table S5 Functional annotation of differentially expressed repressed and induced genes of Rasless MEFs whose transcriptional pattern is reversed in both BRAF- and MEK1-rescued MEFs. The GeneCodis functional annotation tool was used on the list of genes included in Additional file 4: Table S4. Section S5A shows the results for the repressed genes while Section S5B shows the results from the induced genes. Click here for file

Additional file 6: Figure S1 Alterations of Sca1 expression in Rasless fibroblasts. (A) Flow cytometric analysis of Sca1 (Ly6A) protein expression using specific antibodies in K-Ras^lox^ MEFs before (solid grey profile) and after 6 days or 12 days of 4OHT treatment to render them Rasless, as well as in BRAF-rescued and MEK1-rescued MEFs. As a control, Sca1 protein expression in two constitutive double-knockout (H-Ras^-/-^; N-Ras^-/-^) MEF cell lines (A624-6 and A624-8) did not show any change after similar treatment with 4OHT for 9 or 16 days, indicating that increased Sca1 expression is not an off-target effect of 4OHT treatment (not shown). (B) Reduced Sca1 protein expression as a result of incubating 6-day 4OHT-treated K-Ras^lox^ MEFs with Jak inhibitor I (420099, Millipore) for the times indicated (6, 24 and 48 hours). K-Ras^lox^ MEFs treated with either DMSO or Jak inhibitor I showed a similar Sca1 expression to the control untreated K-Ras^lox^ MEFs (not shown). (C) Stable knockdown of Sca1 expression by specific *shRNA-Sca1* constructs introduced into K-Ras^lox^ MEFs and Rasless cells (generated after 16- and 22-day 4OHT–treatment). As a control, stable integration of a non-targeting shRNA construct (*shRNA-NT*) did not cause any detectable changes in Sca1 expression in the same cell lines. (D) MTT proliferation assays of cultures of control K-Ras^lox^ and Rasless MEFs transduced with shRNA-NT and shRNA-Sca1 constructs. * p < 0.05 (shRNA-Sca1 *vs* K-Ras^lox^). (E) Immunoblot assays of several cell cycle-related proteins in control, untreated K-Ras^lox^ MEFs and the same K-Ras^lox^ cells knocked down by means of a shRNA-Sca1 construct, before or after a 12-day 4OHT treatment to render them Rasless. Click here for file

Additional file 7: Figure S2 Reversal of the mRNA and microRNA expression profiles of Rasless cells by RB silencing. (A) Differentially expressed mRNAs in Rasless MEFs showing the opposite pattern of expression in shRB-rescued cells. Venn diagrams showing numbers of shared, differentially expressed mRNAs that were simultaneously detected as induced (54 genes, left panel) or repressed (215 genes, right panel) in Rasless MEFs (pair-wise comparison with control MEFs, FDR = 0.01) and as repressed (left panel) or induced (right panel), respectively, in shRB-rescued MEFs (pair-wise comparisons with Rasless MEFs, FDR = 0.03); Diagrams generated using the Venny application. Red: transcriptional induction. Green: transcriptional repression. Histogram bars represent the functional enrichment of GO Biological Process categories linked to the list of induced (54) and repressed (215) genes identified in the upper Venn diagrams. The GeneCodis (Gene Annotation Co-occurrence Discovery) functional annotation tool was used to identify specific gene subsets within the list of 269 differentially expressed, induced or repressed genes that shared co-occurrent functional annotations linking them, with high statistical significance, to particular Biological Procesess. Green bars: repressed loci. Red bars: induced loci. (B) Differentially expressed microRNAs in Rasless MEFs showing the opposite pattern of expression in shRB-rescued cells. Venn diagrams showing the numbers of shared, differentially expressed miRNAs that were simultaneously detected as induced (12 miRNAs, left panel) or repressed (28 miRNAs, right panel) in Rasless MEFs (pair-wise comparison with control K-Ras^lox^ MEFs, FDR = 0.1) and as repressed (left panel) or induced (right panel), respectively, in shRB-rescued MEFs (pair-wise comparisons with Rasless MEFs, FDR ≤ 0,17); Diagrams generated using the Venny application software as indicated. Red: transcriptional induction. Green: transcriptional repression. Functional enrichment analysis of the list of 40 differentially expressed miRNAs identified in the Venn diagrams showing the opposite transcriptional behaviour between Rasless and shRB-rescued MEFs. The StarBase (sRNA Target Base) functional annotation tool was used to detect enriched GO Biological Process terms identified with high statistical significance (p-value < 10^-4^). Bars depict the degree of statistical significance for each functional category, represented as the -log of the corrected p-value of significance. Click here for file
