# Supplementary material for: Reversible, interrelated mRNA and miRNA expression patterns in the transcriptome of Rasless fibroblasts: functional and mechanistic implications
Source: BMC Genomics. 2013 Oct 25;14:731. doi: 10.1186/1471-2164-14-731 (PMC4007593; doi:10.1186/1471-2164-14-731)
Supplement: Additional file 1: Table S1 — Differential gene expression in Rasless MEFs. List of 3091 differentially expressed probesets (2239 different genes) identified by means of SAM contrasts (FDR = 0.01) comparing the microarray-generated transcriptional profile of Rasless MEFs to that of control, K-Raslox MEFs (already H-Ras/N-Ras double KO). [file 1471-2164-14-731-S1.pdf]

Table S1. Differential gene expression in Rasless MEFs.

List of 3091 differentially expressed probesets (2239 different genes) identified by means of SAM contrasts (FDR=0.01) comparing the microarray-generated transcriptional profile of 12-day 4OHT Rasless MEFs to that of control, untreated K-Ras^{lox} MEFs. The differentially expressed loci are identified by *Affymetrix Probeset ID*, *Gene Name and Symbol*, and listed according to degree of overexpression or repression, quantitated by *d-value*. The ***** symbols in the *R-fold* column denote independent validation of the transcriptional data obtained by means of Western immunoblot or FACS analysis. *d-value* is a parameter measuring the statistical distance separating the calculated expression value of each gene probeset from the null hypothesis (no-change). *q-value* is the estimated FDR at the largest p-value for which the probe set would be statistically significant. *R-fold* is a measure of the fold change of a probeset in the collection of microarrays provided by the SAM algorithm. Values in red denote overexpression. Values in green denote transcriptional repression. The column *Expression reversed by* indicates whether or not the exogenous expression of activated BRAF or MEK1 is able to reverse the transcriptional direction shown by that particular probeset under the Rasless status (BRAF: Reversed expression profile resulting from exogenous expression of activated BRAF at FDR=0.1; MEK: Reversed expression from exogenous expression of activated MEK1 at FDR=0.1; n.r.= not rescued, same transcriptional direction in Rasless and BRAF- or MEK1-rescued MEFs. Empty space: no data available in BRAF- or MEK1-rescued cells for this probeset at FDR=0.1.

Probeset ID	*Symbol*	*Gene Name*	*d-value*	*q-value*	*R-fold*	*Expression reversed by*
1444026_at	AI593442	expressed sequence AI593442	17.25	0.00	5.88	BRAF
1449815_a_at	Ssbp2	single-stranded DNA binding protein 2	15.58	0.00	2.65	BRAF; MEK
1435950_at	Hr	hairless	14.94	0.00	3.20	BRAF; MEK
1436978_at	Wnt9a	wingless-type MMTV integration site 9A	14.33	0.00	4.05	BRAF; MEK
1438266_at	Adamtsl5	ADAMTS-like 5	14.25	0.00	4.88	BRAF; MEK
1420986_s_at	Kif3b	kinesin family member 3B	13.01	0.00	1.67	BRAF; MEK
1449082_at	Mfap5	microfibrillar associated protein 5	12.54	0.00	15.90	BRAF; MEK
1420562_at	Slurp1	secreted Ly6/Plaur domain containing 1	12.47	0.00	12.27	BRAF; MEK
1426672_at	Tmem16k	transmembrane protein 16K	11.63	0.00	1.66	; MEK
1434052_at	AI593442	expressed sequence AI593442	11.30	0.00	3.41	BRAF
1417304_at	Chrd	chordin	11.17	0.00	1.56	; MEK
1418962_at	Necap2	NECAP endocytosis associated 2	11.03	0.00	1.73	BRAF; MEK
1460561_x_at	Sepw1	selenoprotein W. muscle 1	10.95	0.00	2.76	BRAF
1435738_x_at	Serf2	small EDRK-rich factor 2	10.93	0.00	1.35	BRAF; MEK
1455236_x_at	Serf2	small EDRK-rich factor 2	10.92	0.00	1.36	BRAF; MEK
1432558_a_at	Mal	myelin and lymphocyte protein. T-cell differentiation protein	10.89	0.00	3.66	BRAF; MEK
1426811_at	Ppp2r5b	protein phosphatase 2. regulatory subunit B (B56). beta isoform	10.88	0.00	2.70	BRAF; MEK
1453419_at	Mras	muscle and microspikes RAS	10.88	0.00	1.82	BRAF; MEK
1418195_at	Galnt10	UDP-N-acetyl-alpha-D-galactosamine:polypeptide N-acetylgalactosaminyltransferase 10	10.88	0.00	2.30	BRAF; MEK
1419619_at	Krt80	keratin 80	10.69	0.00	2.83	
1417439_at	Cd248	CD248 antigen. endosialin	10.50	0.00	2.07	BRAF; n.r.
1454881_s_at	Upk3b	uroplakin 3B	10.40	0.00	5.81	BRAF; MEK
1434237_at	Upk3b	uroplakin 3B	10.28	0.00	3.41	BRAF; MEK
1417034_at	Trappc6a	trafficking protein particle complex 6A	10.19	0.00	1.61	; MEK
1449711_at	Atp6v1e1	ATPase. H+ transporting. lysosomal V1 subunit E1	10.16	0.00	1.49	BRAF; MEK
1417275_at	Mal	myelin and lymphocyte protein. T-cell differentiation protein	10.14	0.00	21.04	BRAF; MEK
1417133_at	Pmp22	peripheral myelin protein 22	10.12	0.00	5.06	BRAF; MEK
1459871_x_at	March2	membrane-associated ring finger (C3HC4) 2	10.08	0.00	2.01	BRAF; MEK
1416411_at	Gstm2	glutathione S-transferase. mu 2	9.95	0.00	3.88	BRAF; MEK
1416017_at	Copg	coatomer protein complex. subunit gamma	9.91	0.00	1.53	
1450512_at	Ntn4	netrin 4	9.90	0.00	4.04	BRAF; MEK

Probeset ID	Symbol	Gene Name	d-value	q-value	R-fold	Expression reversed by
1424365_at	1810037I17Rik	RIKEN cDNA 1810037I17 gene	9.89	0.00	1.70	BRAF; MEK
1418454_at	Mfap5	microfibrillar associated protein 5	9.76	0.00	21.53	BRAF; MEK
1418671_at	Capn5	calpain 5	9.70	0.00	2.06	
1415677_at	Dhrs1	dehydrogenase/reductase (SDR family) member 1	9.68	0.00	1.45	BRAF; MEK
1452173_at	Hadha	hydroxyacyl-Coenzyme A dehydrogenase/3-ketoacyl-Coenzyme A thiolase/enoyl-Coenzyme A hydratase (trifunctional protein). alpha subunit	9.62	0.00	1.37	
1428750_at	Cdc42ep2	CDC42 effector protein (Rho GTPase binding) 2	9.62	0.00	3.55	BRAF
1430421_a_at	Tmem205	transmembrane protein 205	9.50	0.00	2.47	BRAF; MEK
1424057_at	Gdap2	ganglioside-induced differentiation-associated-protein 2	9.48	0.00	1.50	BRAF; MEK
1451207_at	Cbara1	calcium binding atopy-related autoantigen 1	9.45	0.00	1.69	
1423047_at	Tollip	toll interacting protein	9.26	0.00	1.72	BRAF; MEK
1422347_at	Npy6r	neuropeptide Y receptor Y6	9.25	0.00	9.01	BRAF; MEK
1433786_x_at	Serf2	small EDRK-rich factor 2	9.20	0.00	1.34	BRAF; MEK
1448429_at	Gyg	glycogenin	9.14	0.00	1.91	BRAF; MEK
1437893_at	Plb1	phospholipase B1	9.11	0.00	4.83	BRAF; MEK
1431359_a_at	1110007C09Rik	RIKEN cDNA 1110007C09 gene	9.08	0.00	1.80	BRAF; MEK
1428717_at	Scrn1	secernin 1	9.05	0.00	3.52	BRAF; MEK
1417604_at	Camk1	calcium/calmodulin-dependent protein kinase I	9.05	0.00	2.66	BRAF; MEK
1453008_at	2300002D11Rik	RIKEN cDNA 2300002D11 gene	9.03	0.00	5.06	BRAF; MEK
1423239_at	Impdh1	inosine 5'-phosphate dehydrogenase 1	9.01	0.00	2.46	BRAF; MEK
1428718_at	Scrn1	secernin 1	9.00	0.00	3.96	BRAF; MEK
1449259_at	Rab3d	RAB3D. member RAS oncogene family	9.00	0.00	1.87	BRAF; MEK
1449712_s_at	Atp6v1e1	ATPase. H+ transporting. lysosomal V1 subunit E1	8.92	0.00	1.48	BRAF; MEK
1459807_x_at	4933406E20Rik	RIKEN cDNA 4933406E20 gene	8.85	0.00	1.76	BRAF
1432158_a_at	Trappc2	trafficking protein particle complex 2	8.81	0.00	1.75	BRAF; MEK
1435716_x_at	Snrpn	small nuclear ribonucleoprotein N	8.80	0.00	1.63	BRAF; MEK
1438086_at	Npy6r	neuropeptide Y receptor Y6	8.77	0.00	17.26	BRAF; MEK
1416513_at	Lamb2	laminin. beta 2	8.76	0.00	2.82	BRAF; MEK
1429212_a_at	Lrrc51	leucine rich repeat containing 51	8.76	0.00	1.77	BRAF; MEK
1433916_at	Vamp3	vesicle-associated membrane protein 3	8.74	0.00	1.44	BRAF; MEK
1429119_at	Iah1	isoamyl acetate-hydrolyzing esterase 1 homolog (S. cerevisiae)	8.73	0.00	2.62	BRAF; MEK
1417842_at	Caml	calcium modulating ligand	8.67	0.00	1.53	BRAF; MEK
1436865_at	Slc26a11	solute carrier family 26. member 11	8.66	0.00	1.44	BRAF; MEK
1439728_at	D330027H18Rik	RIKEN cDNA D330027H18 gene	8.63	0.00	4.50	BRAF
1424762_at	C1qtnf5	C1q and tumor necrosis factor related protein 5	8.57	0.00	2.30	BRAF; MEK
1421217_a_at	Lgals9	lectin. galactose binding. soluble 9	8.56	0.00	3.44	BRAF; MEK
1442076_at	Cobl	cordon-bleu	8.52	0.00	2.55	BRAF; MEK
1423856_at	Rpl17	ribosomal protein L17	8.51	0.00	4.37	
1416635_at	Smpdl3a	sphingomyelin phosphodiesterase. acid-like 3A	8.39	0.00	7.85	BRAF; MEK
1418891_a_at	Rab3d	RAB3D. member RAS oncogene family	8.37	0.00	2.76	BRAF; MEK
1422157_a_at	Itgb1bp1	integrin beta 1 binding protein 1	8.31	0.00	1.69	BRAF; MEK
1433449_at	Snx32	sorting nexin 32	8.29	0.00	1.90	BRAF; MEK
1435510_at	Ppm1h	protein phosphatase 1H (PP2C domain containing)	8.27	0.00	1.75	BRAF; MEK
1433891_at	Lgr4	leucine-rich repeat-containing G protein-coupled receptor 4	8.26	0.00	3.12	BRAF
1451496_at	Mtss1	metastasis suppressor 1	8.16	0.00	1.51	BRAF; MEK
1433566_at	Rasl10b	RAS-like. family 10. member B	8.16	0.00	2.14	BRAF; MEK
1439500_at	Scrn1	secernin 1	8.14	0.00	4.75	BRAF; MEK
1434558_at	Wdr47	WD repeat domain 47	8.13	0.00	1.79	BRAF; MEK
1437498_at	ENSMUSG00000055440	predicted gene. ENSMUSG00000055440	8.12	0.00	1.71	BRAF; MEK
1418714_at	Dusp8	dual specificity phosphatase 8	8.09	0.00	2.38	BRAF; MEK
1450409_a_at	4930570C03Rik	RIKEN cDNA 4930570C03 gene	8.08	0.00	2.53	BRAF; MEK
1422596_at	Nkain4	Na+/K+ transporting ATPase interacting 4	8.07	0.00	11.90	BRAF; MEK
1418595_at	S3-12	plasma membrane associated protein. S3-12	8.06	0.00	2.86	BRAF; MEK
1416321_s_at	Prelp	proline arginine-rich end leucine-rich repeat	8.05	0.00	41.78	BRAF; MEK
1424256_at	Rdh12	retinol dehydrogenase 12	8.02	0.00	1.47	

Probeset ID	Symbol	Gene Name	d-value	q-value	R-fold	Expression reversed by
1427864_at	Hist1h3d	histone cluster 1. H3d	8.00	0.00	1.88	BRAF; MEK
1451978_at	Loxl1	lysyl oxidase-like 1	7.98	0.00	1.60	BRAF; MEK
1416727_a_at	Cyb5	cytochrome b-5	7.97	0.00	1.78	BRAF; MEK
1418586_at	Adcy9	adenylate cyclase 9	7.97	0.00	1.97	BRAF
1418702_a_at	2810428I15Rik	RIKEN cDNA 2810428I15 gene	7.95	0.00	3.56	BRAF; MEK
1451217_a_at	Immp1l	IMP1 inner mitochondrial membrane peptidase-like (S. cerevisiae)	7.95	0.00	1.57	BRAF; MEK
1434387_at	Itfg3	integrin alpha FG-GAP repeat containing 3	7.94	0.00	1.91	BRAF
1417185_at	Ly6a	lymphocyte antigen 6 complex. locus A	7.90	0.00	10.26	; n.r.
1418890_a_at	Rab3d	RAB3D. member RAS oncogene family	7.87	0.00	2.67	BRAF; MEK
1419979_s_at	Creb3	cAMP responsive element binding protein 3	7.86	0.00	1.98	BRAF; MEK
1416521_at	Sepw1	selenoprotein W. muscle 1	7.84	0.00	2.74	BRAF; MEK
1422780_at	Pxmp4	peroxisomal membrane protein 4	7.83	0.00	2.02	; MEK
1421045_at	Mrc2	mannose receptor. C type 2	7.82	0.00	3.15	BRAF
1423507_a_at	Sirt2	sirtuin 2 (silent mating type information regulation 2. homolog) 2 (S. cerevisiae)	7.82	0.00	1.77	BRAF; MEK
1452745_at	Trappc9	trafficking protein particle complex 9	7.81	0.00	1.52	; n.r.
1439256_x_at	Gpr137b-ps	G protein-coupled receptor 137B. pseudogene	7.79	0.00	1.76	BRAF; MEK
1418089_at	Stx8	syntaxin 8	7.78	0.00	1.55	BRAF; MEK
1429348_at	Sema3c	sema domain. immunoglobulin domain (Ig). short basic domain. secreted. (semaphorin) 3C	7.77	0.00	6.82	BRAF; MEK
1433979_at	Rbms2	RNA binding motif. single stranded interacting protein 2	7.73	0.00	1.51	BRAF; MEK
1454898_s_at	Iah1	isoamyl acetate-hydrolyzing esterase 1 homolog (S. cerevisiae)	7.68	0.00	2.66	BRAF; MEK
1427773_a_at	Rabac1	Rab acceptor 1 (prenylated)	7.68	0.00	1.75	BRAF; MEK
1423365_at	Cacna1g	calcium channel. voltage-dependent. T type. alpha 1G subunit	7.67	0.00	2.11	BRAF; MEK
1438550_x_at	Srr	serine racemase	7.67	0.00	1.54	BRAF; MEK
1435446_a_at	Chpt1	choline phosphotransferase 1	7.66	0.00	1.66	BRAF; MEK
1450435_at	L1cam	L1 cell adhesion molecule	7.66	0.00	3.91	
1430539_at	Mxra7	matrix-remodelling associated 7	7.61	0.00	2.13	BRAF; MEK
1416328_a_at	Atp6v0e	ATPase. H+ transporting. lysosomal V0 subunit E	7.58	0.00	1.48	BRAF; MEK
1450881_s_at	Gpr137b-ps	G protein-coupled receptor 137B. pseudogene	7.56	0.00	2.05	; MEK
1424826_s_at	Mtss1	metastasis suppressor 1	7.55	0.00	6.33	BRAF; MEK
1425125_at	Oit3	oncoprotein induced transcript 3	7.52	0.00	1.50	BRAF; MEK
1456026_at	Nhlrc3	NHL repeat containing 3	7.52	0.00	2.14	BRAF; MEK
1437900_at	4930523C07Rik	RIKEN cDNA 4930523C07 gene	7.51	0.00	1.99	BRAF
1443502_at	Birc7	baculoviral IAP repeat-containing 7 (livin)	7.51	0.00	1.66	BRAF; MEK
1449024_a_at	Hexa	hexosaminidase A	7.48	0.00	1.69	BRAF; MEK
1417565_at	Abhd5	abhydrolase domain containing 5	7.45	0.00	2.22	BRAF; MEK
1455768_at	Npc2	Niemann Pick type C2	7.45	0.00	2.18	BRAF
1435055_a_at	Tom1	target of myb1 homolog (chicken)	7.43	0.00	2.04	BRAF; MEK
1448744_at	Galns	galactosamine (N-acetyl)-6-sulfate sulfatase	7.41	0.00	2.16	BRAF; MEK
1425439_a_at	Slc41a3	solute carrier family 41. member 3	7.41	0.00	1.66	BRAF; MEK
1451932_a_at	Adamtsl4	ADAMTS-like 4	7.41	0.00	3.62	BRAF; MEK
1439794_at	NA	NA	7.40	0.00	9.45	BRAF; MEK
1436870_s_at	Afap1l2	actin filament associated protein 1-like 2	7.39	0.00	5.71	; n.r.
1419668_at	Sgcb	sarcoglycan. beta (dystrophin-associated glycoprotein)	7.39	0.00	2.15	BRAF; MEK
1423794_at	D2Ertd391e	DNA segment. Chr 2. ERATO Doi 391. expressed	7.37	0.00	1.33	BRAF
1456805_a_at	LOC100043911	hypothetical protein LOC100043911	7.36	0.00	1.54	BRAF; MEK
1435785_at	Ehd2	EH-domain containing 2	7.35	0.00	2.15	BRAF; MEK
1452646_at	Trp53inp2	transformation related protein 53 inducible nuclear protein 2	7.34	0.00	1.93	BRAF; MEK
1416330_at	Cd81	CD81 antigen	7.33	0.00	1.30	BRAF; MEK
1417605_s_at	Camk1	calcium/calmodulin-dependent protein kinase I	7.32	0.00	2.66	BRAF; MEK
1454830_at	Fbn2	fibrillin 2	7.30	0.00	13.15	BRAF; MEK
1440617_at	Cpa6	carboxypeptidase A6	7.26	0.00	6.11	BRAF; MEK
1435264_at	Emilin2	elastin microfibril interfacer 2	7.26	0.00	2.39	n.r. ; n.r.
1419015_at	Wisp2	WNT1 inducible signaling pathway protein 2	7.25	0.00	26.74	BRAF; MEK
1459522_s_at	Gyg	glycogenin	7.24	0.00	1.80	BRAF; MEK
1419315_at	Slamf9	SLAM family member 9	7.24	0.00	1.92	BRAF; MEK

Probeset ID	*Symbol*	*Gene Name*	*d-value*	*q-value*	*R-fold*	*Expression reversed by*
1452879_at	Synpo2	synaptopodin 2	7.23	0.00	2.57	BRAF; MEK
1452929_at	Clip1	CAP-GLY domain containing linker protein 1	7.22	0.00	1.56	BRAF; MEK
1444086_at	E030049G20Rik	RIKEN cDNA E030049G20 gene	7.22	0.00	1.66	BRAF; MEK
1416360_at	Snx18	sorting nexin 18	7.22	0.00	2.29	BRAF; MEK
1416567_s_at	Atp5e	ATP synthase. H+ transporting. mitochondrial F1 complex. epsilon subunit	7.20	0.00	1.48	BRAF
1416359_at	Snx18	sorting nexin 18	7.18	0.00	2.15	BRAF; MEK
1439409_x_at	Tyrp1	tyrosinase-related protein 1	7.16	0.00	1.42	BRAF; MEK
1417384_at	Entpd5	ectonucleoside triphosphate diphosphohydrolase 5	7.15	0.00	2.36	BRAF; MEK
1420540_a_at	Rit1	Ras-like without CAAX 1	7.14	0.00	1.68	BRAF; MEK
1427943_at	Acyp2	acylphosphatase 2. muscle type	7.12	0.00	3.30	BRAF; MEK
1422977_at	Gp1bb	glycoprotein Ib. beta polypeptide	7.12	0.00	2.87	BRAF; MEK
1428597_at	Tbc1d9b	TBC1 domain family. member 9B	7.12	0.00	1.43	; MEK
1452330_a_at	Mxra8	matrix-remodelling associated 8	7.11	0.00	1.85	
1435351_at	2310026E23Rik	RIKEN cDNA 2310026E23 gene	7.09	0.00	6.19	
1424408_at	Lims2	LIM and senescent cell antigen like domains 2	7.07	0.00	2.12	BRAF; MEK
1439255_s_at	LOC100044979	similar to Gpr137b protein	7.07	0.00	1.80	BRAF; MEK
1423298_at	Add3	adducin 3 (gamma)	7.06	0.00	1.86	; n.r.
1460702_at	Triap1	TP53 regulated inhibitor of apoptosis 1	7.02	0.00	1.61	BRAF; MEK
1418927_a_at	Habp4	hyaluronic acid binding protein 4	7.02	0.00	1.79	BRAF; MEK
1432260_at	Gpr39	G protein-coupled receptor 39	7.02	0.00	2.83	BRAF; MEK
1427287_s_at	Itpr2	inositol 1.4.5-triphosphate receptor 2	7.01	0.00	2.76	; MEK
1416322_at	Prelp	proline arginine-rich end leucine-rich repeat	7.01	0.00	10.07	BRAF; MEK
1418895_at	Skap2	src family associated phosphoprotein 2	7.01	0.00	1.63	; MEK
1438011_at	Pcyt1a	phosphate cytidylyltransferase 1. choline. alpha isoform	7.00	0.00	1.65	BRAF; MEK
1455566_s_at	2810022L02Rik	RIKEN cDNA 2810022L02 gene	6.99	0.00	2.21	BRAF; MEK
1436520_at	Ahnak2	AHNAK nucleoprotein 2	6.99	0.00	2.60	
1423939_a_at	Yif1a	Yip1 interacting factor homolog A (S. cerevisiae)	6.98	0.00	1.63	BRAF; MEK
1452445_at	Slc41a2	solute carrier family 41. member 2	6.97	0.00	2.12	BRAF; MEK
1428891_at	9130213B05Rik	RIKEN cDNA 9130213B05 gene	6.96	0.00	4.97	BRAF; MEK
1434930_at	Tpcn1	two pore channel 1	6.95	0.00	2.34	
1417073_a_at	Qk	quaking	6.94	0.00	2.14	BRAF
1434823_x_at	Myeov2	myeloma overexpressed 2	6.94	0.00	1.65	BRAF; MEK
1416924_at	Bri3	brain protein I3	6.93	0.00	1.77	BRAF; MEK
1439593_s_at	LOC100043911	hypothetical protein LOC100043911	6.93	0.00	1.40	BRAF; MEK
1428864_at	5530400B01Rik	RIKEN cDNA 5530400B01 gene	6.92	0.00	2.26	BRAF; MEK
1431597_a_at	Nrip3	nuclear receptor interacting protein 3	6.92	0.00	2.83	BRAF; MEK
1449331_a_at	Rapsn	receptor-associated protein of the synapse	6.90	0.00	1.93	BRAF; MEK
1448390_a_at	Dhrs3	dehydrogenase/reductase (SDR family) member 3	6.90	0.00	5.47	BRAF; MEK
1431198_x_at	9430038I01Rik	RIKEN cDNA 9430038I01 gene	6.88	0.00	1.31	; MEK
1455652_at	Kif3a	kinesin family member 3A	6.87	0.00	1.84	BRAF; MEK
1438026_at	Zfp560	zinc finger protein 560	6.85	0.00	1.79	BRAF; MEK
1453278_a_at	Clip4	CAP-GLY domain containing linker protein family. member 4	6.85	0.00	1.35	
1423940_at	Yif1a	Yip1 interacting factor homolog A (S. cerevisiae)	6.84	0.00	1.71	BRAF; MEK
1424154_a_at	Isca2	iron-sulfur cluster assembly 2 homolog (S. cerevisiae)	6.83	0.00	1.42	BRAF; MEK
1455833_at	Afap1l2	actin filament associated protein 1-like 2	6.83	0.00	1.78	; n.r.
1421022_x_at	Acyp1	acylphosphatase 1. erythrocyte (common) type	6.83	0.00	1.58	BRAF; n.r.
1424628_a_at	1500032D16Rik	RIKEN cDNA 1500032D16 gene	6.82	0.00	1.59	BRAF
1450410_a_at	4930570C03Rik	RIKEN cDNA 4930570C03 gene	6.82	0.00	2.25	BRAF
1416408_at	Acox1	acyl-Coenzyme A oxidase 1. palmitoyl	6.81	0.00	1.93	BRAF; MEK
1448233_at	Prnp	prion protein	6.80	0.00	2.46	BRAF; MEK
1456245_x_at	Vamp3	vesicle-associated membrane protein 3	6.80	0.00	1.45	BRAF; MEK
1425869_a_at	Psen2	presenilin 2	6.79	0.00	1.70	BRAF; MEK
1417527_at	Ap3m2	adaptor-related protein complex 3. mu 2 subunit	6.77	0.00	2.03	BRAF; MEK
1426997_at	Thra	thyroid hormone receptor alpha	6.77	0.00	1.80	BRAF; MEK
1451854_a_at	Shroom3	shroom family member 3	6.77	0.00	1.72	BRAF; MEK

Probeset ID	Symbol	Gene Name	d-value	q-value	R-fold	Expression reversed by
1447845_s_at	Vnn1	vanin 1	6.76	0.00	21.50	BRAF; MEK
1426743_at	Appl2	adaptor protein. phosphotyrosine interaction. PH domain and leucine zipper containing 2	6.74	0.00	2.67	BRAF; MEK
1433628_at	Coq10a	coenzyme Q10 homolog A (yeast)	6.73	0.00	1.79	BRAF; MEK
1424833_at	Itpr2	inositol 1.4.5-triphosphate receptor 2	6.73	0.00	2.68	; MEK
1455596_a_at	BC029214	cDNA sequence BC029214	6.73	0.00	1.84	BRAF; MEK
1435085_at	NA	NA	6.71	0.00	2.76	BRAF; MEK
1425141_at	Lactb2	lactamase. beta 2	6.71	0.00	2.16	BRAF; MEK
1435028_at	Wdr7	WD repeat domain 7	6.71	0.00	1.53	BRAF; MEK
1423205_at	Tm9sf4	transmembrane 9 superfamily protein member 4	6.70	0.00	1.38	
1454810_s_at	Vps8	vacuolar protein sorting 8 homolog (S. cerevisiae)	6.68	0.00	1.50	BRAF; MEK
1452385_at	Usp53	ubiquitin specific peptidase 53	6.68	0.00	2.55	BRAF; MEK
1417934_at	Dnajc4	DnaJ (Hsp40) homolog. subfamily C. member 4	6.68	0.00	1.42	; MEK
1432419_a_at	2700078K21Rik	RIKEN cDNA 2700078K21 gene	6.67	0.00	2.13	BRAF; MEK
1426435_at	Tmem135	transmembrane protein 135	6.66	0.00	1.54	BRAF; MEK
1458359_at	NA	NA	6.65	0.00	1.32	BRAF; MEK
1452309_at	Cgnl1	cingulin-like 1	6.65	0.00	2.14	BRAF; MEK
1454677_at	Timp2	tissue inhibitor of metalloproteinase 2	6.64	0.00	2.11	BRAF; MEK
1417236_at	Ehd3	EH-domain containing 3	6.62	0.00	1.97	BRAF; MEK
1426285_at	Lama2	laminin. alpha 2	6.61	0.00	2.27	BRAF; MEK
1417639_at	Slc22a4	solute carrier family 22 (organic cation transporter). member 4	6.61	0.00	2.69	BRAF; MEK
1449267_at	Chid1	chitinase domain containing 1	6.61	0.00	1.60	BRAF; MEK
1415756_a_at	Snapin	SNAP-associated protein	6.59	0.00	1.62	BRAF; MEK
1427244_at	Ttc15	tetratricopeptide repeat domain 15	6.58	0.00	1.48	
1428332_at	Pik3ip1	phosphoinositide-3-kinase interacting protein 1	6.55	0.00	2.40	; n.r.
1450222_x_at	Klk1b4	kallikrein 1-related pepidase b4	6.55	0.00	1.12	BRAF; MEK
1451172_at	Tprgl	transformation related protein 63 regulated like	6.55	0.00	2.09	BRAF; MEK
1429176_at	Lrsam1	leucine rich repeat and sterile alpha motif containing 1	6.55	0.00	1.82	
1424713_at	Calml4	calmodulin-like 4	6.55	0.00	4.52	; n.r.
1437683_x_at	Serf2	small EDRK-rich factor 2	6.54	0.00	1.33	BRAF; MEK
1434385_at	Tom1l2	target of myb1-like 2 (chicken)	6.54	0.00	1.61	
1418486_at	Vnn1	vanin 1	6.53	0.00	23.38	BRAF; MEK
1445896_at	2810433D01Rik	RIKEN cDNA 2810433D01 gene	6.52	0.00	3.28	BRAF; MEK
1431856_a_at	C1qtnf6	C1q and tumor necrosis factor related protein 6	6.52	0.00	4.14	BRAF; MEK
1451490_at	Lyplal1	lysophospholipase-like 1	6.50	0.00	2.01	BRAF; MEK
1422668_at	Serpinb9b	serine (or cysteine) peptidase inhibitor. clade B. member 9b	6.49	0.00	4.35	; MEK
1435874_at	Prkab2	protein kinase. AMP-activated. beta 2 non-catalytic subunit	6.48	0.00	1.73	BRAF; MEK
1423376_a_at	Dok4	docking protein 4	6.48	0.00	1.78	BRAF; MEK
1443583_at	LOC100043911	hypothetical protein LOC100043911	6.48	0.00	1.36	BRAF; MEK
1455282_x_at	Alas1	aminolevulinic acid synthase 1	6.48	0.00	1.52	BRAF; MEK
1425819_at	Zbtb7c	zinc finger and BTB domain containing 7C	6.47	0.00	1.50	; MEK
1444786_at	Nol3	nucleolar protein 3 (apoptosis repressor with CARD domain)	6.45	0.00	2.06	BRAF; MEK
1456176_x_at	Slc25a39	solute carrier family 25. member 39	6.44	0.00	1.50	
1434036_at	Mtss1	metastasis suppressor 1	6.44	0.00	5.90	BRAF
1418436_at	Stx7	syntaxin 7	6.43	0.00	1.36	BRAF; MEK
1428375_at	4932415G12Rik	RIKEN cDNA 4932415G12 gene	6.42	0.00	1.72	
1452621_at	Pcbd2	pterin 4 alpha carbinolamine dehydratase/dimerization cofactor of hepatocyte nuclear factor 1 alpha (TCF1) 2	6.42	0.00	1.62	BRAF; MEK
1426801_at	Sept8	septin 8	6.42	0.00	2.05	BRAF; MEK
1454615_x_at	Srp14	signal recognition particle 14	6.40	0.00	1.35	BRAF; MEK
1436580_at	Hgsnat	heparan-alpha-glucosaminide N-acetyltransferase	6.39	0.00	1.45	; MEK
1427347_s_at	Tubb2a	tubulin. beta 2a	6.39	0.00	1.97	BRAF; MEK
1435875_at	Prkab2	protein kinase. AMP-activated. beta 2 non-catalytic subunit	6.39	0.00	1.65	BRAF; MEK
1455469_at	Slc6a7	solute carrier family 6 (neurotransmitter transporter. L-proline). member 7	6.38	0.00	1.24	BRAF; MEK
1449964_a_at	Mlycd	malonyl-CoA decarboxylase	6.38	0.00	1.57	; MEK
1422477_at	Cables1	Cdk5 and Abl enzyme substrate 1	6.37	0.00	2.52	BRAF; n.r.

Probeset ID	Symbol	Gene Name	d-value	q-value	R-fold	Expression reversed by
1455390_at	Alkbh6	alkB. alkylation repair homolog 6 (E. coli)	6.37	0.00	1.57	; MEK
1449117_at	Jund	Jun proto-oncogene related gene d	6.37	0.00	1.58	
1417545_at	Trpv4	transient receptor potential cation channel. subfamily V. member 4	6.35	0.00	1.66	; n.r.
1451196_at	Ypel5	yippee-like 5 (Drosophila)	6.34	0.00	2.07	BRAF; MEK
1424214_at	9130213B05Rik	RIKEN cDNA 9130213B05 gene	6.34	0.00	4.98	BRAF; MEK
1437112_at	Pld1	phospholipase D1	6.33	0.00	1.60	BRAF; MEK
1420336_at	Adamtsl5	ADAMTS-like 5	6.33	0.00	2.55	BRAF; MEK
1428621_a_at	Vps25	vacuolar protein sorting 25 (yeast)	6.32	0.00	1.58	; MEK
1424177_at	Tmem38a	transmembrane protein 38A	6.32	0.00	1.88	BRAF; MEK
1424539_at	Ubl4	ubiquitin-like 4	6.30	0.00	1.98	BRAF; MEK
1424416_at	Nkiras2	NFKB inhibitor interacting Ras-like protein 2	6.30	0.00	1.70	BRAF; MEK
1433516_a_at	Myeov2	myeloma overexpressed 2	6.30	0.00	1.62	BRAF; MEK
1436365_at	Zbtb7c	zinc finger and BTB domain containing 7C	6.29	0.00	2.33	; MEK
1454470_at	4930404F17Rik	RIKEN cDNA 4930404F17 gene	6.29	0.00	1.19	BRAF; MEK
1455594_at	Exoc3	exocyst complex component 3	6.28	0.00	2.06	BRAF; MEK
1455671_at	Commd8	COMM domain containing 8	6.26	0.00	1.35	
1448415_a_at	Sema3b	sema domain. immunoglobulin domain (Ig). short basic domain. secreted. (semaphorin) 3B	6.26	0.00	2.78	BRAF; MEK
1436109_at	AI317395	expressed sequence AI317395	6.24	0.00	1.50	BRAF; MEK
1438549_a_at	Srr	serine racemase	6.24	0.00	1.72	BRAF
1426317_at	Stard6	StAR-related lipid transfer (START) domain containing 6	6.24	0.00	1.85	BRAF; MEK
1460235_at	Scarb2	scavenger receptor class B. member 2	6.24	0.00	1.40	BRAF; MEK
1416825_at	Snta1	syntrophin. acidic 1	6.23	0.00	2.93	BRAF; MEK
1450040_at	Timp2	tissue inhibitor of metalloproteinase 2	6.23	0.00	2.79	BRAF; MEK
1452702_at	Clcn7	chloride channel 7	6.22	0.00	1.66	BRAF; MEK
1439542_at	Zfp651	zinc finger protein 651	6.22	0.00	1.76	BRAF; MEK
1426622_a_at	Qpct	glutaminyl-peptide cyclotransferase (glutaminyl cyclase)	6.22	0.00	2.37	BRAF
1429961_at	1700021C14Rik	RIKEN cDNA 1700021C14 gene	6.20	0.00	1.63	n.r. ; n.r.
1454654_at	Dirc2	disrupted in renal carcinoma 2 (human)	6.20	0.00	1.75	BRAF; MEK
1422986_at	Esrrb	estrogen related receptor. beta	6.20	0.00	1.29	BRAF; MEK
1417751_at	Stk10	serine/threonine kinase 10	6.20	0.00	1.54	n.r. ; n.r.
1426624_a_at	Ypel3	yippee-like 3 (Drosophila)	6.19	0.00	1.94	BRAF; MEK
1456267_at	Mef2b	myocyte enhancer factor 2B	6.19	0.00	1.84	; MEK
1447850_x_at	Zfand3	zinc finger. AN1-type domain 3	6.18	0.00	1.37	; MEK
1429164_at	Prss36	protease. serine. 36	6.18	0.00	1.44	BRAF; MEK
1435910_at	Fads3	fatty acid desaturase 3	6.18	0.00	1.96	BRAF; MEK
1456134_x_at	Yif1a	Yip1 interacting factor homolog A (S. cerevisiae)	6.17	0.00	1.42	BRAF; MEK
1424359_at	Oplah	5-oxoprolinase (ATP-hydrolysing)	6.17	0.00	1.43	
1455045_at	Srr	serine racemase	6.17	0.00	2.18	BRAF; MEK
1416154_at	Srp54a	signal recognition particle 54a	6.16	0.00	1.16	n.r. ; MEK
1417421_at	S100a1	S100 calcium binding protein A1	6.14	0.00	1.86	BRAF
1427050_at	Txndc16	thioredoxin domain containing 16	6.14	0.00	2.16	BRAF; MEK
1451074_at	Rnf13	ring finger protein 13	6.13	0.00	1.91	BRAF; MEK
1420859_at	Pkia	protein kinase inhibitor. alpha	6.13	0.00	2.86	BRAF; MEK
1417392_a_at	Slc7a7	solute carrier family 7 (cationic amino acid transporter. y+ system). member 7	6.13	0.00	1.85	BRAF; MEK
1416327_at	Ufc1	ubiquitin-fold modifier conjugating enzyme 1	6.13	0.00	1.42	BRAF
1436678_at	Sgcb	sarcoglycan. beta (dystrophin-associated glycoprotein)	6.12	0.00	2.50	BRAF; MEK
1424979_at	Aph1a	anterior pharynx defective 1a homolog (C. elegans)	6.12	0.00	1.62	BRAF; MEK
1448954_at	Nrip3	nuclear receptor interacting protein 3	6.11	0.00	5.06	BRAF; MEK
1454675_at	Thra	thyroid hormone receptor alpha	6.11	0.00	2.42	BRAF
1420772_a_at	Tsc22d3	TSC22 domain family. member 3	6.11	0.00	3.80	BRAF; MEK
1459850_x_at	Glrb	glycine receptor. beta subunit	6.09	0.00	4.33	BRAF
1419954_s_at	Zfand3	zinc finger. AN1-type domain 3	6.08	0.00	1.80	BRAF; MEK
1460695_a_at	2010111I01Rik	RIKEN cDNA 2010111I01 gene	6.08	0.00	1.87	BRAF; MEK
1429083_at	Agl	amylo-1.6-glucosidase. 4-alpha-glucanotransferase	6.07	0.00	1.52	BRAF; MEK
1435928_at	6430548M08Rik	RIKEN cDNA 6430548M08 gene	6.07	0.00	1.54	BRAF; MEK

Probeset ID	*Symbol*	*Gene Name*	*d-value*	*q-value*	*R-fold*	*Expression reversed by*
1417970_at	Atp5s	ATP synthase. H+ transporting. mitochondrial F0 complex. subunit s	6.06	0.00	1.74	BRAF; MEK
1416735_at	Asah1	N-acylsphingosine amidohydrolase 1	6.06	0.00	1.95	BRAF; MEK
1437038_x_at	1700029I08Rik	RIKEN cDNA 1700029I08 gene	6.06	0.00	1.32	BRAF; MEK
1434510_at	Papss2	3'-phosphoadenosine 5'-phosphosulfate synthase 2	6.05	0.00	3.26	BRAF; MEK
1422831_at	Fbn2	fibrillin 2	6.05	0.00	13.40	BRAF
1428202_at	1810037C20Rik	RIKEN cDNA 1810037C20 gene	6.05	0.00	1.49	BRAF
1436272_at	Rab3gap2	RAB3 GTPase activating protein subunit 2	6.04	0.00	1.59	BRAF; MEK
1451362_at	Rab7l1	RAB7. member RAS oncogene family-like 1	6.04	0.00	1.88	BRAF; MEK
1420641_a_at	Sqrdl	sulfide quinone reductase-like (yeast)	6.04	0.00	2.28	BRAF
1441081_a_at	1110038B12Rik	RIKEN cDNA 1110038B12 gene	6.04	0.00	1.46	; MEK
1434944_at	Dmpk	dystrophia myotonica-protein kinase	6.04	0.00	5.29	BRAF; MEK
1417590_at	Cyp27a1	cytochrome P450. family 27. subfamily a. polypeptide 1	6.03	0.00	2.28	BRAF; MEK
1422568_at	Ndel1	nuclear distribution gene E-like homolog 1 (A. nidulans)	6.03	0.00	1.37	BRAF; MEK
1416880_at	Mcl1	myeloid cell leukemia sequence 1	6.03	0.00	1.67	BRAF
1453767_a_at	Nt5m	5'.3'-nucleotidase. mitochondrial	6.02	0.00	1.50	; MEK
1421088_at	Gpc4	glypican 4	6.01	0.00	1.76	BRAF; MEK
1435189_at	Frmpd1	FERM and PDZ domain containing 1	6.01	0.00	1.62	BRAF; MEK
1423348_at	Fzd8	frizzled homolog 8 (Drosophila)	6.01	0.00	1.96	BRAF; MEK
1451814_a_at	Htatip2	HIV-1 tat interactive protein 2. homolog (human)	6.01	0.00	1.73	; MEK
1427927_at	Hscb	HscB iron-sulfur cluster co-chaperone homolog (E. coli)	6.01	0.00	1.63	BRAF; MEK
1417149_at	P4ha2	procollagen-proline. 2-oxoglutarate 4-dioxygenase (proline 4-hydroxylase). alpha II polypeptide	6.00	0.00	1.95	BRAF; MEK
1436560_at	Kif3a	kinesin family member 3A	6.00	0.00	2.41	BRAF; MEK
1448912_at	C1qtnf1	C1q and tumor necrosis factor related protein 1	5.99	0.00	1.43	
1416373_at	Nfs1	nitrogen fixation gene 1 (S. cerevisiae)	5.99	0.00	1.33	BRAF; MEK
1430985_at	1810027O10Rik	RIKEN cDNA 1810027O10 gene	5.99	0.00	1.67	BRAF; MEK
1450922_a_at	Tgfb2	transforming growth factor. beta 2	5.98	0.00	3.82	BRAF; MEK
1417272_at	9130005N14Rik	RIKEN cDNA 9130005N14 gene	5.98	0.00	1.81	BRAF; MEK
1423086_at	Npc1	Niemann Pick type C1	5.98	0.00	1.61	BRAF; MEK
1443916_at	2900026A02Rik	RIKEN cDNA 2900026A02 gene	5.98	0.00	1.55	BRAF; MEK
1437245_at	Tmem154	transmembrane protein 154	5.96	0.00	1.52	BRAF; MEK
1449043_at	Naga	N-acetyl galactosaminidase. alpha	5.96	0.00	1.82	BRAF
1433833_at	Fndc3b	fibronectin type III domain containing 3B	5.96	0.00	1.70	BRAF; MEK
1452134_at	Tmem175	transmembrane protein 175	5.95	0.00	1.89	BRAF; MEK
1452590_a_at	Plac9	placenta specific 9	5.95	0.00	5.33	BRAF; MEK
1418164_at	Stx2	syntaxin 2	5.94	0.00	1.51	
1428445_at	Yif1b	Yip1 interacting factor homolog B (S. cerevisiae)	5.94	0.00	1.42	BRAF; MEK
1424242_at	Bphl	biphenyl hydrolase-like (serine hydrolase. breast epithelial mucin-associated antigen)	5.93	0.00	1.63	BRAF
1424834_s_at	Itpr2	inositol 1.4.5-triphosphate receptor 2	5.93	0.00	2.48	
1423425_at	1300012G16Rik	RIKEN cDNA 1300012G16 gene	5.92	0.00	1.55	; MEK
1450628_at	Slc2a8	solute carrier family 2. (facilitated glucose transporter). member 8	5.92	0.00	1.44	BRAF; MEK
1432136_s_at	Zdhhc4	zinc finger. DHHC domain containing 4	5.92	0.00	1.54	
1416130_at	Prnp	prion protein	5.91	0.00	3.46	BRAF; MEK
1452847_at	2410008K03Rik	RIKEN cDNA 2410008K03 gene	5.91	0.00	1.79	BRAF
1417881_at	Slc39a3	solute carrier family 39 (zinc transporter). member 3	5.91	0.00	1.85	BRAF; MEK
1422771_at	Smad6	MAD homolog 6 (Drosophila)	5.89	0.00	2.92	BRAF; MEK
1426974_at	Os9	amplified in osteosarcoma	5.88	0.00	1.35	BRAF
1419664_at	Srr	serine racemase	5.88	0.00	2.30	BRAF; MEK
1460180_at	Hexb	hexosaminidase B	5.88	0.00	1.76	BRAF; MEK
1426167_a_at	Camk4	calcium/calmodulin-dependent protein kinase IV	5.88	0.00	1.82	BRAF; MEK
1436070_at	Glo1	glyoxalase 1	5.87	0.00	2.00	BRAF
1449263_at	Ufm1	ubiquitin-fold modifier 1	5.87	0.00	1.82	BRAF; MEK
1421120_at	Myo6	myosin VI	5.87	0.00	1.30	BRAF; MEK
1417283_at	Lynx1	Ly6/neurotoxin 1	5.86	0.00	3.03	BRAF; MEK
1449455_at	Hck	hemopoietic cell kinase	5.86	0.00	2.67	BRAF; MEK
1428851_at	1300014I06Rik	RIKEN cDNA 1300014I06 gene	5.84	0.00	2.19	BRAF; MEK

Probeset ID	Symbol	Gene Name	d-value	q-value	R-fold	Expression reversed by
1424726_at	Tmem150	transmembrane protein 150	5.83	0.00	1.88	BRAF; MEK
1458412_at	Zfp78	zinc finger protein 78	5.83	0.00	1.65	BRAF; MEK
1440975_at	Mxra7	matrix-remodelling associated 7	5.82	0.00	2.48	BRAF; MEK
1415896_x_at	Snrpn	small nuclear ribonucleoprotein N	5.82	0.00	1.33	BRAF; MEK
1448808_a_at	Nme2	non-metastatic cells 2. protein (NM23B) expressed in	5.81	0.00	1.08	; MEK
1453153_at	Lins2	lines homolog 2 (Drosophila)	5.81	0.00	1.34	; MEK
1430668_a_at	Ankra2	ankyrin repeat. family A (RFXANK-like). 2	5.80	0.00	1.28	BRAF; MEK
1451381_at	1810020D17Rik	RIKEN cDNA 1810020D17 gene	5.80	0.00	1.83	BRAF; MEK
1419666_x_at	Nupr1	nuclear protein 1	5.80	0.00	3.48	BRAF; MEK
1423913_at	Hectd3	HECT domain containing 3	5.80	0.00	1.74	BRAF; MEK
1460608_at	Cacna1b	calcium channel. voltage-dependent. N type. alpha 1B subunit	5.79	0.00	1.15	BRAF
1422504_at	Glrb	glycine receptor. beta subunit	5.79	0.00	4.76	BRAF
1453004_at	Slc22a23	solute carrier family 22. member 23	5.78	0.00	3.59	BRAF; MEK
1455781_at	BC027231	cDNA sequence BC027231	5.77	0.00	2.31	BRAF; MEK
1426461_at	Ugp2	UDP-glucose pyrophosphorylase 2	5.77	0.00	2.05	BRAF; MEK
1434454_at	Adcy9	adenylate cyclase 9	5.77	0.00	1.50	BRAF; MEK
1460623_at	Skap2	src family associated phosphoprotein 2	5.76	0.00	2.52	
1418068_at	Ndufa10	NADH dehydrogenase (ubiquinone) 1 alpha subcomplex 10	5.76	0.00	1.30	; MEK
1435360_at	Zfp651	zinc finger protein 651	5.76	0.00	1.95	BRAF; MEK
1428667_at	Maoa	monoamine oxidase A	5.76	0.00	1.57	BRAF; MEK
1433722_at	Akap13	A kinase (PRKA) anchor protein 13	5.75	0.00	1.67	BRAF; MEK
1434715_at	1600014C10Rik	RIKEN cDNA 1600014C10 gene	5.74	0.00	1.40	BRAF; MEK
1454690_at	Ikbkg	inhibitor of kappaB kinase gamma	5.74	0.00	1.95	
1421571_a_at	Ly6c1	lymphocyte antigen 6 complex. locus C1	5.74	0.00	7.22	; n.r.
1448858_at	Ulk2	Unc-51 like kinase 2 (C. elegans)	5.74	0.00	1.77	BRAF; MEK
1436502_at	Mtus1	mitochondrial tumor suppressor 1	5.74	0.00	1.95	BRAF; MEK
1434265_s_at	Ank2	ankyrin 2. brain	5.73	0.00	3.80	BRAF
1439843_at	Camk4	calcium/calmodulin-dependent protein kinase IV	5.73	0.00	4.84	BRAF; MEK
1419667_at	Sgcb	sarcoglycan. beta (dystrophin-associated glycoprotein)	5.73	0.00	1.66	BRAF; MEK
1418088_a_at	Stx8	syntaxin 8	5.72	0.00	1.54	BRAF; MEK
1418961_at	Necap2	NECAP endocytosis associated 2	5.72	0.00	1.67	BRAF; MEK
1422593_at	Ap3s1	adaptor-related protein complex 3. sigma 1 subunit	5.72	0.00	1.63	; MEK
1416195_at	RP23-136K12.4	putative phosphatase	5.72	0.00	1.58	BRAF; MEK
1443620_at	NA	NA	5.71	0.00	1.99	BRAF; MEK
1451486_at	Slc46a3	solute carrier family 46. member 3	5.71	0.00	1.54	BRAF; MEK
1420696_at	Sema3c	sema domain. immunoglobulin domain (Ig). short basic domain. secreted. (semaphorin) 3C	5.71	0.00	6.20	BRAF; MEK
1436063_at	Loxl1	lysyl oxidase-like 1	5.71	0.00	1.98	BRAF; MEK
1433662_s_at	Timp2	tissue inhibitor of metalloproteinase 2	5.71	0.00	2.13	BRAF; MEK
1449023_a_at	Ezh1	enhancer of zeste homolog 1 (Drosophila)	5.70	0.00	1.73	
1459900_at	C79468	expressed sequence C79468	5.70	0.00	2.07	BRAF; MEK
1450658_at	Adamts5	a disintegrin-like and metallopeptidase (reprolysin type) with thrombospondin type 1 motif. 5 (aggrecanase-2)	5.70	0.00	2.77	; MEK
1427955_a_at	Deb1	differentially expressed in B16F10 1	5.70	0.00	1.62	BRAF; MEK
1436102_at	Sec22c	SEC22 vesicle trafficking protein homolog C (S. cerevisiae)	5.69	0.00	1.49	n.r. ;
1442351_a_at	BC029214	cDNA sequence BC029214	5.69	0.00	1.36	BRAF; MEK
1427324_at	Tmem120b	transmembrane protein 120B	5.68	0.00	1.78	BRAF; MEK
1433693_x_at	Vamp3	vesicle-associated membrane protein 3	5.68	0.00	1.31	BRAF; MEK
1416843_at	Pde6d	phosphodiesterase 6D. cGMP-specific. rod. delta	5.68	0.00	1.38	
1448260_at	Uchl1	ubiquitin carboxy-terminal hydrolase L1	5.68	0.00	3.27	BRAF; MEK
1427377_x_at	Hsd3b3	hydroxy-delta-5-steroid dehydrogenase. 3 beta- and steroid delta-isomerase 3	5.67	0.00	1.29	; MEK
1419509_a_at	Nagk	N-acetylglucosamine kinase	5.67	0.00	1.46	BRAF; MEK
1426697_a_at	Lrpap1	low density lipoprotein receptor-related protein associated protein 1	5.66	0.00	1.98	BRAF; MEK
1417566_at	Abhd5	abhydrolase domain containing 5	5.66	0.00	1.93	BRAF; MEK
1436224_at	Kif1c	kinesin family member 1C	5.65	0.00	1.84	; MEK
1427918_a_at	Rhoq	ras homolog gene family. member Q	5.65	0.00	1.64	BRAF; MEK

Probeset ID	*Symbol*	*Gene Name*	*d-value*	*q-value*	*R-fold*	*Expression reversed by*
1433529_at	E430002G05Rik	RIKEN cDNA E430002G05 gene	5.65	0.00	22.81	BRAF; MEK
1453303_at	4833417J20Rik	RIKEN cDNA 4833417J20 gene	5.65	0.00	2.20	BRAF; MEK
1427196_at	Wnk4	WNK lysine deficient protein kinase 4	5.65	0.00	1.53	n.r. ; n.r.
1420382_at	Apob48r	apolipoprotein B48 receptor	5.64	0.00	2.64	BRAF; MEK
1424354_at	Tmem140	transmembrane protein 140	5.64	0.00	2.91	BRAF; MEK
1421170_a_at	Plcb1	phospholipase C. beta 1	5.63	0.00	1.31	; MEK
1417168_a_at	Usp2	ubiquitin specific peptidase 2	5.62	0.00	1.82	; MEK
1438568_at	Mrgpre	MAS-related GPR. member E	5.61	0.00	1.85	BRAF; MEK
1438967_x_at	Amhr2	anti-Mullerian hormone type 2 receptor	5.61	0.00	2.14	; MEK
1433852_at	C330002I19Rik	RIKEN cDNA C330002I19 gene	5.61	0.00	1.56	BRAF; MEK
1425333_at	Rab43	RAB43. member RAS oncogene family	5.60	0.00	1.76	; MEK
1431188_a_at	Tom1	target of myb1 homolog (chicken)	5.60	0.00	2.09	BRAF; MEK
1456424_s_at	Pltp	phospholipid transfer protein	5.60	0.00	3.45	
1448547_at	Rassf3	Ras association (RalGDS/AF-6) domain family member 3	5.59	0.00	2.00	BRAF; MEK
1416331_a_at	Nfe2l1	nuclear factor. erythroid derived 2.-like 1	5.59	0.00	1.78	BRAF; MEK
1452907_at	Galc	galactosylceramidase	5.59	0.00	1.25	n.r. ;
1439153_at	Rnf144b	ring finger protein 144B	5.58	0.00	2.52	; MEK
1448104_at	Aldh6a1	aldehyde dehydrogenase family 6. subfamily A1	5.58	0.00	3.04	BRAF
1435009_at	Slc9a6	solute carrier family 9 (sodium/hydrogen exchanger). member 6	5.58	0.00	1.61	BRAF; MEK
1435367_at	Mapk4	mitogen-activated protein kinase 4	5.58	0.00	1.53	
1452182_at	Galnt2	UDP-N-acetyl-alpha-D-galactosamine:polypeptide N-acetylgalactosaminyltransferase 2	5.58	0.00	1.40	; MEK
1435213_at	Nhlrc1	NHL repeat containing 1	5.57	0.00	1.48	; MEK
1416395_at	Guk1	guanylate kinase 1	5.57	0.00	1.63	; MEK
1455377_at	Ttll7	tubulin tyrosine ligase-like family. member 7	5.57	0.00	2.21	BRAF
1419617_at	Kcnn1	potassium intermediate/small conductance calcium-activated channel. subfamily N. member 1	5.57	0.00	1.40	BRAF; MEK
1429987_at	9930013L23Rik	RIKEN cDNA 9930013L23 gene	5.56	0.00	9.53	BRAF
1416272_at	Map2k1ip1	mitogen-activated protein kinase kinase 1 interacting protein 1	5.56	0.00	1.62	BRAF; MEK
1436513_at	Tanc2	tetratricopeptide repeat. ankyrin repeat and coiled-coil containing 2	5.55	0.00	1.56	; n.r.
1452888_at	1110034G24Rik	RIKEN cDNA 1110034G24 gene	5.55	0.00	2.04	BRAF; MEK
1424362_at	Ppapdc3	phosphatidic acid phosphatase type 2 domain containing 3	5.55	0.00	1.88	BRAF; MEK
1417301_at	Fzd6	frizzled homolog 6 (Drosophila)	5.54	0.00	1.81	BRAF
1453573_at	Hist2h3c1	histone cluster 2. H3c1	5.53	0.00	2.13	BRAF; MEK
1418174_at	Dbp	D site albumin promoter binding protein	5.53	0.00	1.38	BRAF
1434537_at	Slco3a1	solute carrier organic anion transporter family. member 3a1	5.53	0.00	2.45	BRAF; MEK
1449929_at	Dynlt3	dynein light chain Tctex-type 3	5.53	0.00	1.65	BRAF; MEK
1455438_at	NA	NA	5.52	0.00	1.77	BRAF; MEK
1434004_at	Dhps	deoxyhypusine synthase	5.52	0.00	1.49	; MEK
1448867_at	Tmem9b	TMEM9 domain family. member B	5.52	0.00	1.59	BRAF; MEK
1433763_at	Entpd5	ectonucleoside triphosphate diphosphohydrolase 5	5.51	0.00	1.60	BRAF; MEK
1415759_a_at	Hbxip	hepatitis B virus x interacting protein	5.51	0.00	1.23	BRAF; MEK
1437149_at	Slc6a6	solute carrier family 6 (neurotransmitter transporter. taurine). member 6	5.51	0.00	1.81	BRAF; MEK
1455793_at	BC035537	cDNA sequence BC035537	5.51	0.00	1.36	; MEK
1424614_at	Frag1	FGF receptor activating protein 1	5.50	0.00	1.46	BRAF; MEK
1426615_s_at	Ndrg4	N-myc downstream regulated gene 4	5.50	0.00	2.72	BRAF; MEK
1439389_s_at	Myadm	myeloid-associated differentiation marker	5.49	0.00	1.24	BRAF; MEK
1433942_at	Myo6	myosin VI	5.49	0.00	1.79	BRAF
1417700_at	Rab38	Rab38. member of RAS oncogene family	5.49	0.00	2.69	BRAF; MEK
1452984_at	Ccny	cyclin Y	5.49	0.00	1.61	BRAF
1423250_a_at	Tgfb2	transforming growth factor. beta 2	5.48	0.00	3.72	BRAF; MEK
1434042_s_at	Mtmr3	myotubularin related protein 3	5.48	0.00	1.48	BRAF
1435796_at	Wscd2	WSC domain containing 2	5.48	0.00	9.48	; MEK
1419298_at	Pon3	paraoxonase 3	5.47	0.00	1.88	BRAF; MEK
1418699_s_at	Fech	ferrochelatase	5.47	0.00	1.21	; MEK
1451390_s_at	Zfand2b	zinc finger. AN1 type domain 2B	5.47	0.00	1.51	BRAF; MEK

Probeset ID	Symbol	Gene Name	d-value	q-value	R-fold	Expression reversed by
1451554_a_at	Aph1a	anterior pharynx defective 1a homolog (C. elegans)	5.47	0.00	1.49	BRAF; MEK
1448769_at	Slc35b1	solute carrier family 35. member B1	5.47	0.00	1.30	BRAF; MEK
1436254_at	NA	NA	5.46	0.00	1.42	BRAF; MEK
1460287_at	Timp2	tissue inhibitor of metalloproteinase 2	5.46	0.00	1.57	BRAF; MEK
1418399_at	Kctd9	potassium channel tetramerisation domain containing 9	5.46	0.00	1.75	BRAF; MEK
1435387_at	Slc2a13	solute carrier family 2 (facilitated glucose transporter). member 13	5.46	0.00	3.29	BRAF; MEK
1422118_at	Sync	syncoilin	5.46	0.00	1.98	BRAF; MEK
1424394_at	Selm	selenoprotein M	5.45	0.00	2.10	BRAF; MEK
1417226_at	Fbxw4	F-box and WD-40 domain protein 4	5.45	0.00	1.44	n.r. ; MEK
1423745_at	1110031B06Rik	RIKEN cDNA 1110031B06 gene	5.45	0.00	1.81	BRAF; MEK
1439013_x_at	Acyp1	acylphosphatase 1. erythrocyte (common) type	5.44	0.00	1.33	BRAF
1450350_a_at	Jdp2	Jun dimerization protein 2	5.44	0.00	1.90	BRAF; MEK
1455697_at	NA	NA	5.43	0.00	4.02	BRAF; MEK
1429181_at	1700009P17Rik	RIKEN cDNA 1700009P17 gene	5.42	0.00	1.43	BRAF
1440215_at	OTTMUSG00000004461	predicted gene. OTTMUSG00000004461	5.42	0.00	1.75	BRAF; MEK
1455376_at	1300010F03Rik	RIKEN cDNA 1300010F03 gene	5.42	0.00	1.26	BRAF
1452194_at	Tbcd	tubulin-specific chaperone d	5.41	0.00	1.26	n.r. ; MEK
1455718_at	Spata21	spermatogenesis associated 21	5.41	0.00	1.18	; MEK
1417092_at	Pthr1	parathyroid hormone receptor 1	5.40	0.00	2.79	BRAF
1418954_at	Camkk1	calcium/calmodulin-dependent protein kinase kinase 1. alpha	5.40	0.00	1.57	; MEK
1460566_at	Mtap1a	microtubule-associated protein 1 A	5.40	0.00	1.89	BRAF; MEK
1416644_a_at	Sema3b	sema domain. immunoglobulin domain (Ig). short basic domain. secreted. (semaphorin) 3B	5.40	0.00	1.48	BRAF; MEK
1419329_at	Sorbs3	sorbin and SH3 domain containing 3	5.40	0.00	2.06	
1423341_at	Cspg4	chondroitin sulfate proteoglycan 4	5.40	0.00	2.39	BRAF; MEK
1434548_at	Serinc3	serine incorporator 3	5.39	0.00	1.94	BRAF; MEK
1454935_at	D930001I22Rik	RIKEN cDNA D930001I22 gene	5.39	0.00	1.41	; MEK
1428289_at	2310051E17Rik	RIKEN cDNA 2310051E17 gene	5.39	0.00	1.98	BRAF; MEK
1417537_at	Zmat2	zinc finger. matrin type 2	5.39	0.00	1.35	; MEK
1433553_at	Garnl3	GTPase activating RANGAP domain-like 3	5.38	0.00	1.46	
1425557_x_at	Tsc22d3	TSC22 domain family. member 3	5.38	0.00	1.49	BRAF; MEK
1455258_at	Kcnc2	potassium voltage gated channel. Shaw-related subfamily. member 2	5.37	0.00	2.09	; MEK
1429115_at	2010003O02Rik	RIKEN cDNA 2010003O02 gene	5.37	0.00	1.60	; n.r.
1434828_at	B430201A12Rik	RIKEN cDNA B430201A12 gene	5.37	0.00	1.59	BRAF; MEK
1452703_at	Ahcyl2	S-adenosylhomocysteine hydrolase-like 2	5.37	0.00	1.66	; MEK
1454211_a_at	Shroom3	shroom family member 3	5.37	0.00	1.89	; MEK
1438534_x_at	BC004004	cDNA sequence BC004004	5.37	0.00	1.30	BRAF; MEK
1449536_at	Kcnn1	potassium intermediate/small conductance calcium-activated channel. subfamily N. member 1	5.36	0.00	1.39	; MEK
1448392_at	Sparc	secreted acidic cysteine rich glycoprotein	5.36	0.00	1.57	BRAF; MEK
1433808_at	D330001F17Rik	RIKEN cDNA D330001F17 gene	5.36	0.00	1.46	BRAF; MEK
1439429_x_at	Dtx2	deltex 2 homolog (Drosophila)	5.36	0.00	1.37	n.r. ;
1429365_at	1700016M24Rik	RIKEN cDNA 1700016M24 gene	5.36	0.00	1.94	BRAF; MEK
1448100_at	4833439L19Rik	RIKEN cDNA 4833439L19 gene	5.36	0.00	1.29	BRAF; MEK
1424191_a_at	Tmem41a	transmembrane protein 41a	5.36	0.00	1.36	; MEK
1425068_a_at	Tex264	testis expressed gene 264	5.36	0.00	2.09	BRAF; MEK
1422654_at	Sgca	sarcoglycan. alpha (dystrophin-associated glycoprotein)	5.36	0.00	1.64	BRAF; MEK
1424068_at	Tcta	T-cell leukemia translocation altered gene	5.35	0.00	2.93	BRAF; MEK
1434051_s_at	Hspa12a	heat shock protein 12A	5.35	0.00	1.32	; n.r.
1453522_at	6530401N04Rik	RIKEN cDNA 6530401N04 gene	5.35	0.00	1.26	; MEK
1436056_at	Kif13b	kinesin family member 13B	5.35	0.00	1.74	BRAF; MEK
1420430_a_at	2510048L02Rik	RIKEN cDNA 2510048L02 gene	5.35	0.00	1.44	; MEK
1426272_at	Lmbr1	limb region 1	5.34	0.00	1.95	BRAF; MEK
1433558_at	Dab2ip	disabled homolog 2 (Drosophila) interacting protein	5.34	0.00	2.00	BRAF; MEK
1418567_a_at	Srp14	signal recognition particle 14	5.33	0.00	1.50	BRAF; MEK
1450828_at	Synpo2	synaptopodin 2	5.33	0.00	1.52	BRAF; MEK

Probeset ID	*Symbol*	*Gene Name*	*d-value*	*q-value*	*R-fold*	*Expression reversed by*
1433723_s_at	Serf2	small EDRK-rich factor 2	5.32	0.00	2.31	BRAF; MEK
1419376_at	1110018M03Rik	RIKEN cDNA 1110018M03 gene	5.32	0.00	3.57	BRAF; MEK
1435603_at	Sned1	sushi. nidogen and EGF-like domains 1	5.32	0.00	1.35	n.r. ; n.r.
1451707_s_at	Slc41a3	solute carrier family 41. member 3	5.32	0.00	2.41	BRAF; MEK
1445565_at	Hist1h1e	histone cluster 1. H1e	5.32	0.00	1.73	BRAF; MEK
1428620_at	Ensa	endosulfine alpha	5.31	0.00	1.52	
1439113_at	LOC77114	similar to development and differentiation enhancing factor 2	5.31	0.00	1.45	BRAF; MEK
1435461_at	Magi3	membrane associated guanylate kinase. WW and PDZ domain containing 3	5.31	0.00	1.91	BRAF; n.r.
1458562_at	NA	NA	5.31	0.00	1.73	BRAF; MEK
1456404_at	Adamts5	a disintegrin-like and metallopeptidase (reprolysin type) with thrombospondin type 1 motif. 5 (aggrecanase-2)	5.31	0.00	2.63	
1428165_at	Vps24	vacuolar protein sorting 24 (yeast)	5.30	0.00	1.62	; MEK
1460559_at	Kank2	KN motif and ankyrin repeat domains 2	5.30	0.00	1.57	BRAF
1448063_at	Iqsec2	IQ motif and Sec7 domain 2	5.30	0.00	1.60	BRAF
1443952_at	NA	NA	5.29	0.00	1.70	BRAF; MEK
1426802_at	Sept8	septin 8	5.29	0.00	1.64	BRAF; MEK
1435522_a_at	2310016E02Rik	RIKEN cDNA 2310016E02 gene	5.29	0.00	1.48	BRAF
1426816_at	Ccdc64	coiled-coil domain containing 64	5.29	0.00	1.65	BRAF; MEK
1427012_at	Lancl1	LanC (bacterial lantibiotic synthetase component C)-like 1	5.29	0.00	1.47	
1429722_at	Zbtb4	zinc finger and BTB domain containing 4	5.29	0.00	2.21	BRAF; MEK
1434263_at	F630110N24Rik	RIKEN cDNA F630110N24 gene	5.28	0.00	1.64	BRAF
1428427_at	Fbxl2	F-box and leucine-rich repeat protein 2	5.28	0.00	3.19	BRAF; MEK
1418455_at	Copz2	coatomer protein complex. subunit zeta 2	5.28	0.00	1.92	BRAF; MEK
1452667_at	Rab2b	RAB2B. member RAS oncogene family	5.27	0.00	1.98	BRAF; MEK
1427405_s_at	Rab11fip5	RAB11 family interacting protein 5 (class I)	5.27	0.00	1.72	
1419814_s_at	S100a1	S100 calcium binding protein A1	5.27	0.00	2.05	BRAF
1419869_s_at	Hdlbp	high density lipoprotein (HDL) binding protein	5.26	0.00	1.40	; n.r.
1417105_at	Trappc2l	trafficking protein particle complex 2-like	5.26	0.00	1.66	BRAF; MEK
1449412_at	1810046J19Rik	RIKEN cDNA 1810046J19 gene	5.26	0.00	1.95	BRAF; MEK
1434354_at	Maob	monoamine oxidase B	5.26	0.00	3.76	BRAF; MEK
1427155_at	Rell2	RELT-like 2	5.26	0.00	1.43	BRAF
1418838_at	Abcd1	ATP-binding cassette. sub-family D (ALD). member 1	5.26	0.00	1.35	BRAF
1434050_at	Vps8	vacuolar protein sorting 8 homolog (S. cerevisiae)	5.25	0.00	1.57	BRAF; MEK
1455385_at	Sh3glb1	SH3-domain GRB2-like B1 (endophilin)	5.25	0.00	1.61	; MEK
1424411_at	Tmem189	transmembrane protein 189	5.25	0.00	1.74	BRAF; MEK
1428067_at	Rasl12	RAS-like. family 12	5.25	0.00	3.12	BRAF; MEK
1421989_s_at	Papss2	3'-phosphoadenosine 5'-phosphosulfate synthase 2	5.25	0.00	3.45	BRAF; MEK
1422550_a_at	Mtap6	microtubule-associated protein 6	5.23	0.00	1.58	BRAF; MEK
1422644_at	Sh3bgr	SH3-binding domain glutamic acid-rich protein	5.23	0.00	2.36	BRAF; MEK
1455295_at	Slc38a7	solute carrier family 38. member 7	5.23	0.00	1.55	; MEK
1427442_a_at	App	amyloid beta (A4) precursor protein	5.23	0.00	1.31	BRAF; MEK
1431167_at	Dgkg	diacylglycerol kinase. gamma	5.23	0.00	2.09	BRAF; MEK
1441263_a_at	A930005H10Rik	RIKEN cDNA A930005H10 gene	5.22	0.00	1.42	; n.r.
1433740_at	Tmem87b	transmembrane protein 87B	5.22	0.00	1.45	BRAF; MEK
1457097_at	Skap2	src family associated phosphoprotein 2	5.22	0.00	1.72	BRAF; MEK
1435109_at	Tmem175	transmembrane protein 175	5.22	0.00	1.66	BRAF; MEK
1418448_at	Rras	Harvey rat sarcoma oncogene. subgroup R	5.21	0.00	1.57	BRAF; MEK
1458306_at	Pigz	phosphatidylinositol glycan anchor biosynthesis. class Z	5.21	0.00	1.26	; MEK
1415721_a_at	1200013P24Rik	RIKEN cDNA 1200013P24 gene	5.21	0.00	1.57	BRAF; MEK
1424434_at	BC024814	cDNA sequence BC024814	5.21	0.00	1.55	; MEK
1428014_at	Hist1h4h	histone cluster 1. H4h	5.21	0.00	3.59	BRAF; MEK
1427168_a_at	Col14a1	collagen. type XIV. alpha 1	5.20	0.00	13.58	BRAF; MEK
1423164_at	Gpr137b-ps	G protein-coupled receptor 137B. pseudogene	5.20	0.00	1.88	; MEK
1428357_at	2610019F03Rik	RIKEN cDNA 2610019F03 gene	5.20	0.00	2.60	BRAF; MEK
1434952_at	Cox4nb	COX4 neighbor	5.20	0.00	1.61	
1425980_at	Wdr54	WD repeat domain 54	5.20	0.00	1.26	BRAF; MEK

Probeset ID	*Symbol*	*Gene Name*	*d-value*	*q-value*	*R-fold*	*Expression reversed by*
1448613_at	Ecm1	extracellular matrix protein 1	5.19	0.00	2.69	BRAF; MEK
1437902_s_at	Rarres2	retinoic acid receptor responder (tazarotene induced) 2	5.19	0.00	1.27	BRAF; MEK
1425281_a_at	Tsc22d3	TSC22 domain family. member 3	5.19	0.00	3.24	BRAF; MEK
1452615_s_at	Trpt1	tRNA phosphotransferase 1	5.19	0.00	1.64	BRAF; MEK
1416937_at	Gabarap	gamma-aminobutyric acid receptor associated protein	5.19	0.00	1.66	BRAF; MEK
1451594_s_at	Serpinb6c	serine (or cysteine) peptidase inhibitor. clade B. member 6c	5.19	0.00	1.35	BRAF; MEK
1435714_x_at	Il17d	interleukin 17D	5.18	0.00	2.22	BRAF; MEK
1453377_at	Sh2d4a	SH2 domain containing 4A	5.17	0.00	7.17	; MEK
1434307_at	Tmem64	transmembrane protein 64	5.17	0.00	1.61	BRAF; MEK
1444270_at	Rshl3	radial spokehead-like 3	5.17	0.00	1.24	; MEK
1417963_at	Pltp	phospholipid transfer protein	5.17	0.00	4.06	
1443872_at	NA	NA	5.16	0.00	1.76	BRAF; MEK
1441952_x_at	Lynx1	Ly6/neurotoxin 1	5.16	0.00	2.71	BRAF; MEK
1416768_at	1110003E01Rik	RIKEN cDNA 1110003E01 gene	5.15	0.00	1.84	BRAF; MEK
1417383_at	Entpd5	ectonucleoside triphosphate diphosphohydrolase 5	5.14	0.00	1.45	BRAF; MEK
1425201_a_at	Hyi	hydroxypyruvate isomerase homolog (E. coli)	5.14	0.00	1.58	
1441948_x_at	Zfand3	zinc finger. AN1-type domain 3	5.14	0.00	1.72	BRAF; MEK
1441376_at	Gabarapl2	gamma-aminobutyric acid (GABA-A) receptor-associated protein-like 2	5.14	0.00	1.63	n.r. ; MEK
1451747_a_at	Atg12	autophagy-related 12 (yeast)	5.13	0.00	1.86	BRAF; MEK
1420124_s_at	Tcta	T-cell leukemia translocation altered gene	5.13	0.00	3.21	BRAF; MEK
1438006_at	4933439F18Rik	RIKEN cDNA 4933439F18 gene	5.12	0.00	1.57	BRAF; MEK
1423707_at	Tmem50b	transmembrane protein 50B	5.12	0.00	1.54	BRAF; MEK
1451970_at	Daglb	diacylglycerol lipase. beta	5.12	0.00	1.97	BRAF; MEK
1424746_at	Kif1c	kinesin family member 1C	5.12	0.00	1.84	; MEK
1420123_at	Tcta	T-cell leukemia translocation altered gene	5.12	0.00	3.20	BRAF; MEK
1417857_at	Mmaa	methylmalonic aciduria (cobalamin deficiency) type A	5.12	0.00	1.64	BRAF; MEK
1431774_a_at	Lyrm1	LYR motif containing 1	5.12	0.00	1.66	BRAF; MEK
1426288_at	Lrp4	low density lipoprotein receptor-related protein 4	5.12	0.00	1.56	; n.r.
1435089_at	2010111I01Rik	RIKEN cDNA 2010111I01 gene	5.11	0.00	1.93	BRAF; MEK
1460343_at	Neurl	neuralized-like homolog (Drosophila)	5.11	0.00	1.67	BRAF
1418568_x_at	Srp14	signal recognition particle 14	5.11	0.00	1.44	BRAF; MEK
1434169_at	9030409G11Rik	RIKEN cDNA 9030409G11 gene	5.11	0.00	1.67	BRAF; MEK
1417626_at	Pde4dip	phosphodiesterase 4D interacting protein (myomegalin)	5.11	0.00	3.02	BRAF; MEK
1416688_at	Snap91	synaptosomal-associated protein 91	5.10	0.00	2.54	
1426711_at	Tmco3	transmembrane and coiled-coil domains 3	5.10	0.00	1.80	BRAF; MEK
1428115_a_at	Rab2b	RAB2B. member RAS oncogene family	5.10	0.00	2.01	BRAF; MEK
1436752_at	Tbccd1	TBCC domain containing 1	5.10	0.00	1.78	BRAF; MEK
1433605_at	Inpp5a	inositol polyphosphate-5-phosphatase A	5.10	0.00	1.85	BRAF; MEK
1452668_x_at	Rab2b	RAB2B. member RAS oncogene family	5.10	0.00	1.96	BRAF; MEK
1421998_at	Tor3a	torsin family 3. member A	5.09	0.00	2.25	BRAF; MEK
1451132_at	Pbxip1	pre-B-cell leukemia transcription factor interacting protein 1	5.09	0.00	1.66	BRAF; MEK
1424309_a_at	Mocs2	molybdenum cofactor synthesis 2	5.09	0.00	1.46	BRAF; MEK
1436609_a_at	Lrpap1	low density lipoprotein receptor-related protein associated protein 1	5.09	0.00	1.73	BRAF
1424927_at	Glipr1	GLI pathogenesis-related 1 (glioma)	5.08	0.00	3.09	; MEK
1423868_at	Txnrd3	thioredoxin reductase 3	5.08	0.00	1.44	n.r. ; MEK
1454075_s_at	Nudt13	nudix (nucleoside diphosphate linked moiety X)-type motif 13	5.08	0.00	1.30	BRAF; n.r.
1418704_at	S100a13	S100 calcium binding protein A13	5.07	0.00	2.35	BRAF
1429583_at	2900053A13Rik	RIKEN cDNA 2900053A13 gene	5.07	0.00	1.89	BRAF; MEK
1416164_at	Fbln5	fibulin 5	5.07	0.00	2.32	BRAF; MEK
1417235_at	Ehd3	EH-domain containing 3	5.07	0.00	2.08	BRAF; MEK
1420858_at	Pkia	protein kinase inhibitor. alpha	5.07	0.00	3.05	BRAF; MEK
1417174_at	Tmem218	transmembrane protein 218	5.07	0.00	1.75	BRAF
1437113_s_at	Pld1	phospholipase D1	5.06	0.00	1.41	BRAF; MEK
1449590_a_at	Mras	muscle and microspikes RAS	5.06	0.00	1.65	BRAF; MEK
1437874_s_at	Hexb	hexosaminidase B	5.06	0.00	1.67	BRAF; MEK
1429018_at	3830408D24Rik	RIKEN cDNA 3830408D24 gene	5.06	0.00	1.68	BRAF; MEK

Probeset ID	Symbol	Gene Name	d-value	q-value	R-fold	Expression reversed by
1415919_at	Npdc1	neural proliferation. differentiation and control gene 1	5.06	0.00	1.82	BRAF; MEK
1427011_a_at	Lancl1	LanC (bacterial lantibiotic synthetase component C)-like 1	5.06	0.00	1.47	; MEK
1452283_at	Rassf8	Ras association (RalGDS/AF-6) domain family (N-terminal) member 8	5.05	0.00	1.78	BRAF; MEK
1421861_at	Clstn1	calsyntenin 1	5.05	0.00	1.62	; n.r.
1448181_at	Klf15	Kruppel-like factor 15	5.05	0.00	1.37	BRAF; MEK
1436325_at	Rora	RAR-related orphan receptor alpha	5.05	0.00	2.71	BRAF; MEK
1415749_a_at	Rragc	Ras-related GTP binding C	5.05	0.00	1.43	BRAF; MEK
1435646_at	Ikbkg	inhibitor of kappaB kinase gamma	5.05	0.00	1.68	; MEK
1428327_at	Trak1	trafficking protein. kinesin binding 1	5.05	0.00	1.30	n.r. ;
1435902_at	Nudt18	nudix (nucleoside diphosphate linked moiety X)-type motif 18	5.05	0.00	2.45	BRAF; MEK
1424219_at	ENSMUSG00000054212	predicted gene. ENSMUSG00000054212	5.04	0.00	1.69	; MEK
1454824_s_at	Mtus1	mitochondrial tumor suppressor 1	5.04	0.00	2.15	BRAF; MEK
1424126_at	Alas1	aminolevulinic acid synthase 1	5.04	0.00	1.78	BRAF; MEK
1417169_at	Usp2	ubiquitin specific peptidase 2	5.04	0.00	1.38	
1416430_at	Cat	catalase	5.04	0.00	1.39	BRAF; MEK
1434864_at	Nipa1	non imprinted in Prader-Willi/Angelman syndrome 1 homolog (human)	5.03	0.00	1.37	BRAF; MEK
1418104_at	Nrip3	nuclear receptor interacting protein 3	5.03	0.00	3.29	BRAF; MEK
1428057_a_at	Ahnak	AHNAK nucleoprotein (desmoyokin)	5.03	0.00	2.41	; MEK
1426522_at	Hadhb	hydroxyacyl-Coenzyme A dehydrogenase/3-ketoacyl-Coenzyme A thiolase/enoyl-Coenzyme A hydratase (trifunctional protein). beta subunit	5.03	0.00	1.46	BRAF; MEK
1428854_at	Tmed8	transmembrane emp24 domain containing 8	5.02	0.00	1.38	BRAF; MEK
1424477_at	Tmem184a	transmembrane protein 184a	5.02	0.00	2.98	BRAF; MEK
1433670_at	Emp2	epithelial membrane protein 2	5.02	0.00	1.67	; MEK
1423865_at	Slc44a1	solute carrier family 44. member 1	5.02	0.00	1.53	n.r. ;
1417707_at	N4bp2l1	NEDD4 binding protein 2-like 1	5.02	0.00	1.36	BRAF; n.r.
1417316_at	Them2	thioesterase superfamily member 2	5.02	0.00	1.62	BRAF; MEK
1451339_at	Suox	sulfite oxidase	5.02	0.00	1.63	BRAF; MEK
1419208_at	Map3k8	mitogen-activated protein kinase kinase kinase 8	5.01	0.00	1.43	
1453855_at	Mxra7	matrix-remodelling associated 7	5.01	0.00	1.92	BRAF; MEK
1459895_at	Ppp4r1l	protein phosphatase 4. regulatory subunit 1-like	5.01	0.00	1.45	; n.r.
1430538_at	2210013O21Rik	RIKEN cDNA 2210013O21 gene	5.01	0.00	1.80	BRAF; MEK
1456500_at	Aph1b	anterior pharynx defective 1b homolog (C. elegans)	5.01	0.00	1.81	BRAF; MEK
1417614_at	Ckm	creatine kinase. muscle	5.00	0.00	1.26	BRAF; MEK
1455126_x_at	2310028O11Rik	RIKEN cDNA 2310028O11 gene	5.00	0.00	1.35	BRAF
1426937_at	6330406I15Rik	RIKEN cDNA 6330406I15 gene	5.00	0.00	9.58	; n.r.
1417809_at	Slc22a18	solute carrier family 22 (organic cation transporter). member 18	5.00	0.00	1.56	BRAF; MEK
1418302_at	Ppt2	palmitoyl-protein thioesterase 2	4.99	0.00	1.46	; MEK
1441937_s_at	LOC100047214	similar to PTEN induced putative kinase 1	4.99	0.00	2.96	BRAF
1448137_at	Aldh7a1	aldehyde dehydrogenase family 7. member A1	4.99	0.00	1.41	; MEK
1416409_at	Acox1	acyl-Coenzyme A oxidase 1. palmitoyl	4.99	0.00	1.77	BRAF; MEK
1422549_at	Arl2	ADP-ribosylation factor-like 2	4.99	0.00	1.59	BRAF; MEK
1436339_at	1810058I24Rik	RIKEN cDNA 1810058I24 gene	4.99	0.00	1.54	BRAF; MEK
1421987_at	Papss2	3'-phosphoadenosine 5'-phosphosulfate synthase 2	4.98	0.00	2.45	BRAF; MEK
1449152_at	Cdkn2b	cyclin-dependent kinase inhibitor 2B (p15. inhibits CDK4)	4.98	0.00	4.03 *	BRAF; n.r.
1418294_at	Epb4.1l4b	erythrocyte protein band 4.1-like 4b	4.98	0.00	1.63	
1449751_at	Slc6a6	solute carrier family 6 (neurotransmitter transporter. taurine). member 6	4.98	0.00	1.62	BRAF; MEK
1415745_a_at	Dscr3	Down syndrome critical region gene 3	4.98	0.00	1.42	BRAF; MEK
1424888_at	March2	membrane-associated ring finger (C3HC4) 2	4.98	0.00	1.72	BRAF; MEK
1420508_at	Sema3f	sema domain. immunoglobulin domain (Ig). short basic domain. secreted. (semaphorin) 3F	4.98	0.00	1.53	BRAF; MEK
1439490_at	Nr2c1	nuclear receptor subfamily 2. group C. member 1	4.98	0.00	1.36	
1434945_at	Lpcat2	lysophosphatidylcholine acyltransferase 2	4.97	0.00	2.36	; MEK
1448418_s_at	Wdr23	WD repeat domain 23	4.97	0.00	1.58	; MEK
1457373_at	NA	NA	4.97	0.00	1.16	BRAF; MEK
1455779_at	Mtap1a	microtubule-associated protein 1 A	4.97	0.00	2.49	BRAF; MEK

Probeset ID	*Symbol*	*Gene Name*	*d-value*	*q-value*	*R-fold*	*Expression reversed by*
1423736_a_at	Dym	dymeclin	4.97	0.00	1.58	BRAF; MEK
1423173_at	Napb	N-ethylmaleimide sensitive fusion protein attachment protein beta	4.96	0.00	1.42	BRAF
1457021_x_at	Amhr2	anti-Mullerian hormone type 2 receptor	4.96	0.00	2.33	; MEK
1421209_s_at	Ikbkg	inhibitor of kappaB kinase gamma	4.96	0.00	1.50	; MEK
1416934_at	Mtm1	X-linked myotubular myopathy gene 1	4.96	0.00	1.75	BRAF; MEK
1434439_at	Gsk3b	glycogen synthase kinase 3 beta	4.95	0.00	2.06	BRAF; MEK
1426981_at	Pcsk6	proprotein convertase subtilisin/kexin type 6	4.95	0.00	2.51	BRAF; MEK
1425327_at	BC008163	cDNA sequence BC008163	4.95	0.00	1.49	BRAF; MEK
1453324_at	6330509M23Rik	RIKEN cDNA 6330509M23 gene	4.95	0.00	1.71	n.r. ;
1450454_at	Tor3a	torsin family 3. member A	4.95	0.00	2.31	BRAF; MEK
1422561_at	Adamts5	a disintegrin-like and metallopeptidase (reprolysin type) with thrombospondin type 1 motif. 5 (aggrecanase-2)	4.95	0.00	2.61	; MEK
1444507_at	Usp53	ubiquitin specific peptidase 53	4.95	0.00	2.73	BRAF; MEK
1434313_at	Ccdc126	coiled-coil domain containing 126	4.94	0.00	2.21	BRAF; MEK
1436514_at	Gpc4	glypican 4	4.94	0.00	1.70	BRAF; MEK
1415911_at	Impact	imprinted and ancient	4.94	0.00	2.10	BRAF; MEK
1422107_at	2410066E13Rik	RIKEN cDNA 2410066E13 gene	4.94	0.00	1.55	BRAF; MEK
1419999_at	Igbp1	immunoglobulin (CD79A) binding protein 1	4.93	0.00	1.79	BRAF; MEK
1450474_at	Serpinb9c	serine (or cysteine) peptidase inhibitor. clade B. member 9c	4.93	0.00	1.43	BRAF; MEK
1434593_at	Eif5a2	eukaryotic translation initiation factor 5A2	4.93	0.00	1.48	BRAF; MEK
1446466_at	NA	NA	4.93	0.00	1.22	
1428736_at	Gramd3	GRAM domain containing 3	4.93	0.00	1.93	BRAF; MEK
1433454_at	Abtb2	ankyrin repeat and BTB (POZ) domain containing 2	4.93	0.00	1.72	BRAF
1423776_s_at	Tbc1d22a	TBC1 domain family. member 22a	4.93	0.00	1.33	; n.r.
1449324_at	Ero1l	ERO1-like (S. cerevisiae)	4.93	0.00	1.84	BRAF; MEK
1448875_at	Zhx1	zinc fingers and homeoboxes 1	4.93	0.00	1.58	; n.r.
1447346_s_at	5530400B01Rik	RIKEN cDNA 5530400B01 gene	4.93	0.00	1.51	BRAF; MEK
1451550_at	Ephb3	Eph receptor B3	4.93	0.00	2.00	BRAF; MEK
1416023_at	Fabp3	fatty acid binding protein 3. muscle and heart	4.93	0.00	5.05	BRAF; MEK
1417607_at	Cox6a2	cytochrome c oxidase. subunit VI a. polypeptide 2	4.93	0.00	1.79	BRAF; MEK
1417116_at	Slc6a8	solute carrier family 6 (neurotransmitter transporter. creatine). member 8	4.93	0.00	1.67	BRAF; MEK
1434089_at	Synpo	synaptopodin	4.92	0.00	4.13	BRAF
1456867_x_at	Ergic3	ERGIC and golgi 3	4.92	0.00	1.26	
1416769_s_at	Atp6v0b	ATPase. H+ transporting. lysosomal V0 subunit B	4.91	0.00	1.91	BRAF; MEK
1434581_at	2410066E13Rik	RIKEN cDNA 2410066E13 gene	4.91	0.00	2.23	
1415853_at	Def8	differentially expressed in FDCP 8	4.90	0.00	1.47	; MEK
1449351_s_at	Pdgfc	platelet-derived growth factor. C polypeptide	4.90	0.00	1.57	BRAF; MEK
1418194_at	Galnt10	UDP-N-acetyl-alpha-D-galactosamine:polypeptide N-acetylgalactosaminyltransferase 10	4.90	0.00	1.84	BRAF; MEK
1457435_x_at	Myom2	myomesin 2	4.90	0.00	2.46	BRAF; MEK
1427040_at	Mdfic	MyoD family inhibitor domain containing	4.90	0.00	1.79	BRAF; MEK
1426647_at	9130011J15Rik	RIKEN cDNA 9130011J15 gene	4.90	0.00	1.71	BRAF; MEK
1419665_a_at	Nupr1	nuclear protein 1	4.90	0.00	3.13	BRAF; MEK
1449575_a_at	Gstp1	glutathione S-transferase. pi 1	4.90	0.00	1.25	; n.r.
1453266_at	Zbtb4	zinc finger and BTB domain containing 4	4.90	0.00	2.07	BRAF; MEK
1450107_a_at	Renbp	renin binding protein	4.89	0.00	1.91	; MEK
1431190_x_at	Fahd2a	fumarylacetoacetate hydrolase domain containing 2A	4.89	0.00	1.24	; MEK
1422989_a_at	Dmpk	dystrophia myotonica-protein kinase	4.89	0.00	2.07	BRAF; MEK
1449772_at	Tbc1d14	TBC1 domain family. member 14	4.88	0.00	1.18	
1424186_at	Ccdc80	coiled-coil domain containing 80	4.88	0.00	3.80	BRAF; MEK
1416340_a_at	Man2b1	mannosidase 2. alpha B1	4.88	0.00	1.45	; MEK
1448546_at	Rassf3	Ras association (RalGDS/AF-6) domain family member 3	4.88	0.00	2.67	BRAF; MEK
1449181_at	Fech	ferrochelatase	4.87	0.00	1.59	BRAF; MEK
1459151_x_at	Ifi35	interferon-induced protein 35	4.87	0.00	1.44	BRAF; MEK
1425790_a_at	Grik2	glutamate receptor. ionotropic. kainate 2 (beta 2)	4.85	0.00	1.21	
1417879_at	Nenf	neuron derived neurotrophic factor	4.85	0.00	1.58	BRAF; MEK

Probeset ID	Symbol	Gene Name	d-value	q-value	R-fold	Expression reversed by
1426460_a_at	Ugp2	UDP-glucose pyrophosphorylase 2	4.85	0.00	2.23	BRAF; MEK
1452212_at	Lmna	lamin A	4.84	0.00	1.20	; MEK
1423210_a_at	Nola3	nucleolar protein family A. member 3	4.84	0.00	1.29	n.r. ;
1426696_at	Lrpap1	low density lipoprotein receptor-related protein associated protein 1	4.84	0.00	1.74	BRAF
1434961_at	Asb1	ankyrin repeat and SOCS box-containing 1	4.84	0.00	1.90	BRAF; MEK
1451810_at	ORF63	open reading frame 63	4.84	0.00	2.36	BRAF; MEK
1427201_at	Mustn1	musculoskeletal. embryonic nuclear protein 1	4.84	0.00	3.17	BRAF; MEK
1435697_a_at	Cytip	cytohesin 1 interacting protein	4.83	0.00	12.01	
1435472_at	Kremen1	kringle containing transmembrane protein 1	4.83	0.00	1.58	BRAF; MEK
1429453_a_at	Mrpl55	mitochondrial ribosomal protein L55	4.83	0.00	1.24	; MEK
1436011_at	Elmo2	engulfment and cell motility 2. ced-12 homolog (C. elegans)	4.83	0.00	1.42	BRAF; MEK
1424503_at	Rab22a	RAB22A. member RAS oncogene family	4.82	0.00	1.38	BRAF
1416654_at	Slc31a2	solute carrier family 31. member 2	4.82	0.00	1.99	BRAF; MEK
1436040_at	LOC100039864	hypothetical protein LOC100039864	4.82	0.00	2.42	BRAF; MEK
1452783_at	Fndc3b	fibronectin type III domain containing 3B	4.82	0.00	1.76	BRAF; MEK
1455466_at	Gpr133	G protein-coupled receptor 133	4.82	0.00	1.65	
1419019_a_at	Akap4	A kinase (PRKA) anchor protein 4	4.82	0.00	1.22	
1459931_a_at	NA	NA	4.82	0.00	1.27	BRAF
1428739_at	2310040A07Rik	RIKEN cDNA 2310040A07 gene	4.82	0.00	2.44	BRAF; MEK
1460285_at	Itga9	integrin alpha 9	4.82	0.00	1.54	BRAF
1415982_at	Herpud2	HERPUD family member 2	4.81	0.00	1.55	BRAF; MEK
1417071_s_at	Cyp4v3	cytochrome P450. family 4. subfamily v. polypeptide 3	4.81	0.00	1.91	
1417846_at	Ulk2	Unc-51 like kinase 2 (C. elegans)	4.81	0.00	1.61	BRAF; MEK
1442371_at	Lmbr1	limb region 1	4.81	0.00	1.98	BRAF; MEK
1417480_at	Fbxo9	f-box protein 9	4.81	0.00	1.66	BRAF; MEK
1429419_at	2310007A19Rik	RIKEN cDNA 2310007A19 gene	4.81	0.00	1.45	BRAF; MEK
1422186_s_at	Cyb5r3	cytochrome b5 reductase 3	4.81	0.00	1.57	BRAF
1428076_s_at	Ndufb4	NADH dehydrogenase (ubiquinone) 1 beta subcomplex 4	4.81	0.00	1.40	; MEK
1433501_at	Ctso	cathepsin O	4.80	0.00	2.00	BRAF; MEK
1420197_at	Gadd45b	growth arrest and DNA-damage-inducible 45 beta	4.80	0.00	1.49	BRAF; MEK
1423328_at	Gdap1	ganglioside-induced differentiation-associated-protein 1	4.80	0.00	1.35	; MEK
1435935_at	NA	NA	4.79	0.00	1.72	BRAF; MEK
1431996_at	NA	NA	4.79	0.00	1.12	
1417492_at	Ctsb	cathepsin B	4.79	0.00	1.72	
1434264_at	Ank2	ankyrin 2. brain	4.79	0.00	4.04	BRAF
1424654_at	Acp2	acid phosphatase 2. lysosomal	4.79	0.00	1.36	BRAF; MEK
1419811_at	Adcy9	adenylate cyclase 9	4.79	0.00	2.55	BRAF
1450967_at	Ptplad2	protein tyrosine phosphatase-like A domain containing 2	4.79	0.00	1.77	; n.r.
1439529_at	A430110N23Rik	RIKEN cDNA A430110N23 gene	4.78	0.01	1.28	
1452763_at	Nipa1	non imprinted in Prader-Willi/Angelman syndrome 1 homolog (human)	4.78	0.01	1.63	BRAF; MEK
1415895_at	Snrpn	small nuclear ribonucleoprotein N	4.78	0.01	1.29	BRAF; MEK
1439874_at	9330102E08Rik	RIKEN cDNA 9330102E08 gene	4.78	0.01	1.50	BRAF; MEK
1418081_at	Dnajc30	DnaJ (Hsp40) homolog. subfamily C. member 30	4.78	0.01	1.39	; MEK
1435008_at	Slc9a6	solute carrier family 9 (sodium/hydrogen exchanger). member 6	4.77	0.01	1.43	BRAF
1436188_a_at	Ndrg4	N-myc downstream regulated gene 4	4.77	0.01	2.78	BRAF; MEK
1455509_at	AB182283	cDNA sequence AB182283	4.77	0.01	1.44	BRAF; MEK
1457440_at	Sstr4	somatostatin receptor 4	4.77	0.01	1.22	; MEK
1431302_a_at	Nudt7	nudix (nucleoside diphosphate linked moiety X)-type motif 7	4.76	0.01	1.63	BRAF; MEK
1418583_at	Hint3	histidine triad nucleotide binding protein 3	4.76	0.01	1.40	BRAF; MEK
1434043_a_at	Repin1	replication initiator 1	4.76	0.01	1.67	BRAF; n.r.
1420614_at	Dynlt3	dynein light chain Tctex-type 3	4.76	0.01	1.82	BRAF; MEK
1457779_at	1110046J04Rik	RIKEN cDNA 1110046J04 gene	4.76	0.01	1.55	; MEK
1419115_at	Alg14	asparagine-linked glycosylation 14 homolog (yeast)	4.75	0.01	1.62	BRAF; MEK
1455291_s_at	Znrf2	zinc and ring finger 2	4.75	0.01	1.86	BRAF; MEK
1452244_at	6330406I15Rik	RIKEN cDNA 6330406I15 gene	4.75	0.01	9.45	; n.r.
1447556_x_at	1700094D03Rik	RIKEN cDNA 1700094D03 gene	4.75	0.01	1.26	BRAF

Probeset ID	Symbol	Gene Name	d-value	q-value	R-fold	Expression reversed by
1417632_at	Atp6v0a1	ATPase. H+ transporting. lysosomal V0 subunit A1	4.75	0.01	2.02	BRAF; MEK
1445870_at	NA	NA	4.75	0.01	1.66	
1421129_a_at	Atp2a3	ATPase. Ca++ transporting. ubiquitous	4.75	0.01	1.66	BRAF; MEK
1422912_at	Bmp4	bone morphogenetic protein 4	4.75	0.01	3.18	BRAF; MEK
1436195_at	BC046404	cDNA sequence BC046404	4.75	0.01	2.87	
1428962_at	1700013F07Rik	RIKEN cDNA 1700013F07 gene	4.74	0.01	1.28	; MEK
1456020_at	Sh3tc2	SH3 domain and tetratricopeptide repeats 2	4.74	0.01	1.80	BRAF; MEK
1436810_x_at	2900010M23Rik	RIKEN cDNA 2900010M23 gene	4.74	0.01	1.44	n.r. ;
1436297_a_at	Grina	glutamate receptor. ionotropic. N-methyl D-aspartate-associated protein 1 (glutamate binding)	4.74	0.01	1.92	BRAF
1437617_x_at	1110034G24Rik	RIKEN cDNA 1110034G24 gene	4.73	0.01	1.39	BRAF; MEK
1433976_at	Reep3	receptor accessory protein 3	4.73	0.01	1.50	
1429185_at	4631416L12Rik	RIKEN cDNA 4631416L12 gene	4.73	0.01	1.60	BRAF; MEK
1428312_at	Lrrc57	leucine rich repeat containing 57	4.72	0.01	1.55	BRAF; MEK
1448900_at	D16H22S680E	DNA segment. Chr 16. human D22S680E. expressed	4.72	0.01	1.30	
1436622_at	Iqsec2	IQ motif and Sec7 domain 2	4.72	0.01	1.72	BRAF
1425560_a_at	S100a16	S100 calcium binding protein A16	4.72	0.01	4.06	; MEK
1423540_at	Rbms2	RNA binding motif. single stranded interacting protein 2	4.71	0.01	1.33	BRAF; MEK
1434268_at	Adar	adenosine deaminase. RNA-specific	4.71	0.01	1.18	
1418434_at	Mkrn1	makorin. ring finger protein. 1	4.70	0.01	1.61	; MEK
1428270_at	Glt8d1	glycosyltransferase 8 domain containing 1	4.70	0.01	1.63	BRAF; MEK
1439981_at	5830405N20Rik	RIKEN cDNA 5830405N20 gene	4.70	0.01	1.20	; MEK
1423061_at	Arvcf	armadillo repeat gene deleted in velo-cardio-facial syndrome	4.70	0.01	1.46	
1416481_s_at	Higd1a	HIG1 domain family. member 1A	4.70	0.01	3.04	BRAF; MEK
1417042_at	Slc37a4	solute carrier family 37 (glucose-6-phosphate transporter). member 4	4.70	0.01	1.55	BRAF; MEK
1424741_s_at	Creb3	cAMP responsive element binding protein 3	4.70	0.01	1.90	BRAF; MEK
1455630_at	Dnalc1	dynein. axonemal. light chain 1	4.69	0.01	1.55	BRAF; MEK
1435504_at	Clip4	CAP-GLY domain containing linker protein family. member 4	4.69	0.01	2.01	; n.r.
1448751_at	Ap3m2	adaptor-related protein complex 3. mu 2 subunit	4.69	0.01	1.89	BRAF; MEK
1418802_at	R74862	expressed sequence R74862	4.69	0.01	1.63	BRAF; MEK
1423613_at	Ssfa2	sperm specific antigen 2	4.69	0.01	1.54	
1422524_at	Abcb6	ATP-binding cassette. sub-family B (MDR/TAP). member 6	4.69	0.01	1.93	BRAF; MEK
1420488_at	Mrps14	mitochondrial ribosomal protein S14	4.69	0.01	1.45	
1438303_at	Tgfb2	transforming growth factor. beta 2	4.69	0.01	2.86	BRAF; MEK
1431627_at	Kif27	kinesin family member 27	4.68	0.01	1.20	BRAF
1451379_at	Rab22a	RAB22A. member RAS oncogene family	4.68	0.01	1.52	BRAF; MEK
1418474_at	1500005A01Rik	RIKEN cDNA 1500005A01 gene	4.68	0.01	1.63	
1436132_at	9830001H06Rik	RIKEN cDNA 9830001H06 gene	4.68	0.01	1.48	BRAF; MEK
1435464_at	1110003E01Rik	RIKEN cDNA 1110003E01 gene	4.68	0.01	3.04	BRAF
1415776_at	Aldh3a2	aldehyde dehydrogenase family 3. subfamily A2	4.68	0.01	1.45	BRAF; MEK
1447936_at	2410006H16Rik	RIKEN cDNA 2410006H16 gene	4.67	0.01	1.94	; n.r.
1448259_at	Fstl1	follistatin-like 1	4.67	0.01	1.64	BRAF; MEK
1423671_at	Dner	delta/notch-like EGF-related receptor	4.67	0.01	8.17	BRAF; MEK
1451643_a_at	Rab4b	RAB4B. member RAS oncogene family	4.67	0.01	1.38	BRAF; MEK
1417659_at	Vps29	vacuolar protein sorting 29 (S. pombe)	4.67	0.01	1.44	BRAF; MEK
1427156_s_at	Ascc2	activating signal cointegrator 1 complex subunit 2	4.67	0.01	1.58	
1428257_s_at	Dynlrb1	dynein light chain roadblock-type 1	4.67	0.01	1.35	BRAF
1419548_at	Kpna1	karyopherin (importin) alpha 1	4.66	0.01	1.28	BRAF; MEK
1460690_at	BC003940	cDNA sequence BC003940	4.66	0.01	1.40	BRAF; MEK
1436468_at	Zdhhc8	zinc finger. DHHC domain containing 8	4.66	0.01	1.30	BRAF; MEK
1455369_at	Apba1	amyloid beta (A4) precursor protein binding. family A. member 1	4.66	0.01	1.43	
1452823_at	Gstk1	glutathione S-transferase kappa 1	4.66	0.01	1.98	n.r. ;
1456193_x_at	Gpx4	glutathione peroxidase 4	4.66	0.01	1.33	BRAF; MEK
1419551_s_at	Stk39	serine/threonine kinase 39. STE20/SPS1 homolog (yeast)	4.65	0.01	1.35	BRAF; MEK
1458236_at	NA	NA	4.65	0.01	3.36	BRAF; MEK
1416372_at	Ptdss1	phosphatidylserine synthase 1	4.65	0.01	1.27	BRAF; MEK

Probeset ID	Symbol	Gene Name	d-value	q-value	R-fold	Expression reversed by
1430292_a_at	1810030N24Rik	RIKEN cDNA 1810030N24 gene	4.65	0.01	1.78	BRAF
1415675_at	Dpm2	dolichol-phosphate (beta-D) mannosyltransferase 2	4.65	0.01	1.34	BRAF
1417066_at	Cabc1	chaperone. ABC1 activity of bc1 complex like (S. pombe)	4.65	0.01	1.39	; MEK
1434162_at	2700078E11Rik	RIKEN cDNA 2700078E11 gene	4.65	0.01	1.44	BRAF; MEK
1423549_at	Slc1a4	solute carrier family 1 (glutamate/neutral amino acid transporter). member 4	4.65	0.01	2.14	BRAF; MEK
1429292_a_at	2310046K01Rik	RIKEN cDNA 2310046K01 gene	4.64	0.01	1.32	BRAF; MEK
1435990_at	Adamts2	a disintegrin-like and metallopeptidase (reprolysin type) with thrombospondin type 1 motif. 2	4.64	0.01	3.42	BRAF; MEK
1428710_at	Rit1	Ras-like without CAAX 1	4.64	0.01	1.61	BRAF; MEK
1452148_at	Lrpap1	low density lipoprotein receptor-related protein associated protein 1	4.64	0.01	2.14	BRAF; MEK
1424781_at	Reep3	receptor accessory protein 3	4.64	0.01	1.58	
1436501_at	Mtus1	mitochondrial tumor suppressor 1	4.64	0.01	2.31	BRAF; MEK
1441478_at	9930013L23Rik	RIKEN cDNA 9930013L23 gene	4.64	0.01	1.76	BRAF
1447842_x_at	NA	NA	4.63	0.01	1.46	
1418949_at	Gdf15	growth differentiation factor 15	4.63	0.01	3.04	; MEK
1452719_at	Zdhhc24	zinc finger. DHHC domain containing 24	4.63	0.01	2.29	BRAF; MEK
1435230_at	Ankrd12	ankyrin repeat domain 12	4.63	0.01	1.60	; MEK
1451720_at	Vps39	vacuolar protein sorting 39 (yeast)	4.62	0.01	1.46	; MEK
1435692_at	Kctd21	potassium channel tetramerisation domain containing 21	4.62	0.01	1.59	BRAF; MEK
1421063_s_at	Snurf	SNRPN upstream reading frame	4.62	0.01	1.83	BRAF; MEK
1425894_at	Mrgprf	MAS-related GPR. member F	4.62	0.01	3.60	BRAF
1420505_a_at	Stxbp1	syntaxin binding protein 1	4.61	0.01	1.54	
1417162_at	Tmbim1	transmembrane BAX inhibitor motif containing 1	4.61	0.01	2.13	BRAF; MEK
1454159_a_at	Igfbp2	insulin-like growth factor binding protein 2	4.61	0.01	1.28	; MEK
1435097_at	Mmab	methylmalonic aciduria (cobalamin deficiency) type B homolog (human)	4.61	0.01	1.63	BRAF
1425610_s_at	Galnt2	UDP-N-acetyl-alpha-D-galactosamine:polypeptide N-acetylgalactosaminyltransferase 2	4.61	0.01	1.49	
1436788_at	Acp2	acid phosphatase 2. lysosomal	4.61	0.01	1.57	BRAF
1446658_at	NA	NA	4.61	0.01	1.16	
1459886_at	NA	NA	4.61	0.01	2.05	
1444108_at	Dnajc25	DnaJ (Hsp40) homolog. subfamily C . member 25	4.61	0.01	1.42	BRAF
1457860_at	NA	NA	4.60	0.01	1.36	; MEK
1449907_at	Bcmo1	beta-carotene 15.15'-monooxygenase	4.60	0.01	1.14	
1426468_at	0610037L13Rik	RIKEN cDNA 0610037L13 gene	4.60	0.01	1.41	
1434662_at	Atg4a	autophagy-related 4A (yeast)	4.60	0.01	1.66	BRAF; MEK
1460731_at	Slc35c2	solute carrier family 35. member C2	4.60	0.01	1.29	; MEK
1421426_at	Hhip	Hedgehog-interacting protein	4.59	0.01	3.03	
1442124_at	NA	NA	4.59	0.01	1.68	BRAF; MEK
1450784_at	Reck	reversion-inducing-cysteine-rich protein with kazal motifs	4.59	0.01	3.98	BRAF; MEK
1416419_s_at	Gabarapl1	gamma-aminobutyric acid (GABA?) receptor-associated protein-like 1	4.59	0.01	1.59	BRAF; MEK
1434379_at	NA	NA	4.59	0.01	1.94	BRAF
1417625_s_at	Cxcr7	chemokine (C-X-C motif) receptor 7	4.59	0.01	7.00	BRAF; MEK
1419577_at	Fig4	FIG4 homolog (S. cerevisiae)	4.58	0.01	1.58	
1423985_at	Gng5	guanine nucleotide binding protein (G protein). gamma 5	4.58	0.01	1.31	BRAF; MEK
1428503_a_at	Nkiras1	NFKB inhibitor interacting Ras-like protein 1	4.58	0.01	1.73	BRAF; MEK
1436566_at	Rab40b	Rab40b. member RAS oncogene family	4.58	0.01	2.54	BRAF; MEK
1435113_x_at	Stmn3	stathmin-like 3	4.57	0.01	1.40	BRAF; MEK
1424747_at	Kif1c	kinesin family member 1C	4.57	0.01	1.66	BRAF; MEK
1416831_at	Neu1	neuraminidase 1	4.57	0.01	1.97	BRAF; MEK
1421352_at	Tlr6	toll-like receptor 6	4.57	0.01	1.26	; MEK
1420375_at	Kif3a	kinesin family member 3A	4.57	0.01	1.79	BRAF; MEK
1418738_at	Scn1b	sodium channel. voltage-gated. type I. beta	4.56	0.01	1.46	BRAF; MEK
1426383_at	Cry2	cryptochrome 2 (photolyase-like)	4.56	0.01	1.63	BRAF; MEK
1423918_at	Rhbdd1	rhomboid domain containing 1	4.56	0.01	1.84	BRAF; MEK
1447544_at	NA	NA	4.56	0.01	2.38	BRAF; MEK

Probeset ID	Symbol	Gene Name	d-value	q-value	R-fold	Expression reversed by
1418892_at	Rhoj	ras homolog gene family. member J	4.56	0.01	2.01	BRAF; MEK
1453312_at	Iqwd1	IQ motif and WD repeats 1	4.56	0.01	1.46	BRAF; MEK
1452766_at	Tppp	tubulin polymerization promoting protein	4.56	0.01	3.27	BRAF; MEK
1420565_at	Hoxa1	homeo box A1	4.56	0.01	1.72	BRAF; MEK
1416565_at	Cox6b1	cytochrome c oxidase. subunit VIb polypeptide 1	4.55	0.01	1.19	; MEK
1419996_s_at	Arnt	aryl hydrocarbon receptor nuclear translocator	4.55	0.01	1.28	
1430726_at	Rassf8	Ras association (RalGDS/AF-6) domain family (N-terminal) member 8	4.55	0.01	1.48	; MEK
1451310_a_at	Ctsl	cathepsin L	4.55	0.01	1.37	BRAF; MEK
1435106_at	Limch1	LIM and calponin homology domains 1	4.55	0.01	4.50	BRAF; MEK
1423176_at	Tob1	transducer of ErbB-2.1	4.55	0.01	1.83	; MEK
1427661_a_at	Tssc4	tumor-suppressing subchromosomal transferable fragment 4	4.54	0.01	1.72	BRAF; MEK
1420561_at	Trpc7	transient receptor potential cation channel. subfamily C. member 7	4.54	0.01	1.19	; MEK
1434402_at	Samd8	sterile alpha motif domain containing 8	4.54	0.01	1.53	BRAF; MEK
1430510_at	Evi5l	ecotropic viral integration site 5 like	4.54	0.01	1.30	; MEK
1434015_at	Slc2a6	solute carrier family 2 (facilitated glucose transporter). member 6	4.54	0.01	1.58	BRAF; MEK
1437527_x_at	Mcl1	myeloid cell leukemia sequence 1	4.54	0.01	1.83	
1422827_x_at	Slc35a2	solute carrier family 35 (UDP-galactose transporter). member A2	4.54	0.01	1.72	BRAF; MEK
1416010_a_at	Ehd1	EH-domain containing 1	4.54	0.01	1.52	BRAF; MEK
1442537_at	NA	NA	4.53	0.01	1.80	BRAF; MEK
1448685_at	2900010M23Rik	RIKEN cDNA 2900010M23 gene	4.53	0.01	1.47	; MEK
1455890_x_at	Snrpn	small nuclear ribonucleoprotein N	4.53	0.01	1.34	BRAF; MEK
1438477_a_at	Mcee	methylmalonyl CoA epimerase	4.53	0.01	1.32	BRAF; MEK
1425756_at	Rab40b	Rab40b. member RAS oncogene family	4.53	0.01	1.24	BRAF; MEK
1452579_at	Iscu	IscU iron-sulfur cluster scaffold homolog (E. coli)	4.52	0.01	1.80	BRAF; MEK
1416976_at	Stam2	signal transducing adaptor molecule (SH3 domain and ITAM motif) 2	4.52	0.01	1.27	
1425140_at	Lactb2	lactamase. beta 2	4.52	0.01	1.50	BRAF; MEK
1421824_at	Bace1	beta-site APP cleaving enzyme 1	4.51	0.01	1.66	BRAF
1426216_at	Cog6	component of oligomeric golgi complex 6	4.51	0.01	1.41	BRAF; MEK
1434472_at	Dusp3	dual specificity phosphatase 3 (vaccinia virus phosphatase VH1-related)	4.51	0.01	2.13	BRAF; MEK
1438032_at	Lrch1	leucine-rich repeats and calponin homology (CH) domain containing 1	4.51	0.01	1.56	BRAF; MEK
1447676_x_at	S100a16	S100 calcium binding protein A16	4.51	0.01	4.58	; MEK
1420731_a_at	Csrp2	cysteine and glycine-rich protein 2	4.51	0.01	1.64	BRAF
1448985_at	Dusp22	dual specificity phosphatase 22	4.51	0.01	1.48	; MEK
1418261_at	Syk	spleen tyrosine kinase	4.51	0.01	1.83	; MEK
1422541_at	Ptprm	protein tyrosine phosphatase. receptor type. M	4.50	0.01	1.64	BRAF; MEK
1433840_a_at	C330006K01Rik	RIKEN cDNA C330006K01 gene	4.50	0.01	1.45	BRAF; MEK
1454152_a_at	Grk4	G protein-coupled receptor kinase 4	4.50	0.01	1.52	BRAF; MEK
1422183_a_at	Adra1b	adrenergic receptor. alpha 1b	4.50	0.01	1.63	BRAF; MEK
1439628_x_at	Rab38	Rab38. member of RAS oncogene family	4.50	0.01	2.17	BRAF; MEK
1421200_at	Dlg2	discs. large homolog 2 (Drosophila)	4.49	0.01	1.21	; MEK
1436992_x_at	Vdac1	voltage-dependent anion channel 1	4.49	0.01	1.09	
1442098_at	AU022434	expressed sequence AU022434	4.49	0.01	1.24	
1421957_a_at	Pcyt1a	phosphate cytidylyltransferase 1. choline. alpha isoform	4.49	0.01	1.58	
1431055_a_at	Snx10	sorting nexin 10	4.49	0.01	1.63	
1448890_at	Klf2	Kruppel-like factor 2 (lung)	4.49	0.01	3.18	
1429726_at	Slc16a9	solute carrier family 16 (monocarboxylic acid transporters). member 9	4.49	0.01	1.23	BRAF
1447418_at	NA	NA	4.48	0.01	1.15	; MEK
1436603_at	Tbl2	transducin (beta)-like 2	4.48	0.01	1.22	
1445667_at	Tbc1d10a	TBC1 domain family. member 10a	4.48	0.01	2.01	BRAF; MEK
1417382_at	Entpd5	ectonucleoside triphosphate diphosphohydrolase 5	4.48	0.01	1.45	BRAF; MEK
1455428_at	A930008G19Rik	RIKEN cDNA A930008G19 gene	4.47	0.01	1.95	
1460260_s_at	Kpna1	karyopherin (importin) alpha 1	4.47	0.01	1.27	BRAF; MEK
1436526_at	Gnptg	N-acetylglucosamine-1-phosphotransferase. gamma subunit	4.47	0.01	1.57	BRAF; MEK
1418899_at	Ufm1	ubiquitin-fold modifier 1	4.47	0.01	1.36	BRAF; MEK
1442093_at	Adc	arginine decarboxylase	4.47	0.01	1.73	BRAF; MEK

Probeset ID	Symbol	Gene Name	d-value	q-value	R-fold	Expression reversed by
1450674_at	Cdk5	cyclin-dependent kinase 5	4.47	0.01	1.36	BRAF
1418957_at	Stac	src homology three (SH3) and cysteine rich domain	4.47	0.01	1.31	
1417423_at	Grina	glutamate receptor. ionotropic. N-methyl D-aspartate-associated protein 1 (glutamate binding)	4.47	0.01	2.07	BRAF; MEK
1437350_at	Dph1	DPH1 homolog (S. cerevisiae)	4.47	0.01	1.37	; MEK
1419170_at	Fam174a	family with sequence similarity 174. member A	4.46	0.01	1.74	BRAF; MEK
1426093_at	Trim34	tripartite motif-containing 34	4.46	0.01	1.23	
1436841_at	B230380D07Rik	RIKEN cDNA B230380D07 gene	4.46	0.01	2.08	BRAF
1447550_at	EG666892	predicted gene. EG666892	4.46	0.01	1.45	
1434017_at	Znrf2	zinc and ring finger 2	4.46	0.01	1.65	BRAF; MEK
1441969_at	Trim36	tripartite motif-containing 36	4.46	0.01	1.56	; MEK
1441774_at	NA	NA	4.46	0.01	1.28	
1442101_at	Elfn1	leucine rich repeat and fibronectin type III. extracellular 1	4.46	0.01	2.28	BRAF; MEK
1439858_at	NA	NA	4.46	0.01	1.67	n.r. ; MEK
1431517_at	4933417C20Rik	RIKEN cDNA 4933417C20 gene	4.45	0.01	1.16	
1458302_at	NA	NA	4.45	0.01	1.44	n.r. ;
1441747_at	NA	NA	4.45	0.01	1.23	
1448213_at	Anxa1	annexin A1	4.45	0.01	1.28	BRAF; MEK
1428288_at	2310051E17Rik	RIKEN cDNA 2310051E17 gene	4.45	0.01	1.95	BRAF; MEK
1449620_s_at	Adcy9	adenylate cyclase 9	4.44	0.01	1.98	BRAF
1423583_at	Fem1a	feminization 1 homolog a (C. elegans)	4.44	0.01	1.45	BRAF; MEK
1416318_at	Serpinb1a	serine (or cysteine) peptidase inhibitor. clade B. member 1a	4.44	0.01	11.00	
1426998_at	Zfand3	zinc finger. AN1-type domain 3	4.44	0.01	2.03	BRAF; MEK
1429073_at	2210015D19Rik	RIKEN cDNA 2210015D19 gene	4.44	0.01	1.40	
1448644_at	Pef1	penta-EF hand domain containing 1	4.44	0.01	1.55	BRAF
1417660_s_at	Vps29	vacuolar protein sorting 29 (S. pombe)	4.44	0.01	1.37	BRAF; MEK
1434856_at	Ankrd44	ankyrin repeat domain 44	4.44	0.01	1.59	BRAF; MEK
1436542_at	NA	NA	4.43	0.01	1.54	; MEK
1439021_at	Centb5	centaurin. beta 5	4.43	0.01	1.48	; MEK
1450923_at	Tgfb2	transforming growth factor. beta 2	4.43	0.01	3.34	BRAF; MEK
1455582_at	NA	NA	4.43	0.01	2.32	
1441264_x_at	A930005H10Rik	RIKEN cDNA A930005H10 gene	4.42	0.01	1.31	
1448146_at	Wwp2	WW domain containing E3 ubiquitin protein ligase 2	4.42	0.01	1.49	
1438442_at	5730470L24Rik	RIKEN cDNA 5730470L24 gene	4.42	0.01	1.74	
1459751_s_at	Ppp1r16a	protein phosphatase 1. regulatory (inhibitor) subunit 16A	4.42	0.01	1.26	n.r. ; n.r.
1417981_at	Insig2	insulin induced gene 2	4.42	0.01	1.54	BRAF; MEK
1440799_s_at	Farp2	FERM. RhoGEF and pleckstrin domain protein 2	4.42	0.01	2.24	BRAF
1428791_at	Ube2h	ubiquitin-conjugating enzyme E2H	4.42	0.01	1.52	BRAF
1416046_a_at	Fuca2	fucosidase. alpha-L- 2. plasma	4.42	0.01	3.05	
1440474_at	Crot	carnitine O-octanoyltransferase	4.42	0.01	1.23	; MEK
1416091_at	Mtap4	microtubule-associated protein 4	4.41	0.01	1.72	n.r. ; n.r.
1429943_at	Ctbs	chitobiase. di-N-acetyl-	4.41	0.01	2.02	
1442051_at	Hist2h3c1	histone cluster 2. H3c1	4.41	0.01	5.78	BRAF; MEK
1417008_at	Crat	carnitine acetyltransferase	4.41	0.01	1.50	BRAF
1452955_at	Gtf2h5	general transcription factor IIH. polypeptide 5	4.41	0.01	1.66	BRAF
1451489_at	Slc25a35	solute carrier family 25. member 35	4.40	0.01	1.49	BRAF; MEK
1452446_a_at	Tmub2	transmembrane and ubiquitin-like domain containing 2	4.40	0.01	1.52	BRAF; MEK
1441076_at	Mpv17	Mpv17 transgene. kidney disease mutant	4.40	0.01	1.40	BRAF; MEK
1457368_at	2310033K02Rik	RIKEN cDNA 2310033K02 gene	4.40	0.01	1.21	BRAF; MEK
1425868_at	Hist2h2bb	histone cluster 2. H2bb	4.40	0.01	2.30	BRAF; MEK
1440330_at	Hist1h1e	histone cluster 1. H1e	4.40	0.01	1.43	BRAF; MEK
1448477_at	Chst12	carbohydrate sulfotransferase 12	4.40	0.01	1.51	BRAF; MEK
1456716_s_at	3110002H16Rik	RIKEN cDNA 3110002H16 gene	4.40	0.01	1.59	
1429551_at	4930579G22Rik	RIKEN cDNA 4930579G22 gene	4.40	0.01	1.36	; MEK
1419005_at	Crybb3	crystallin. beta B3	4.40	0.01	1.31	BRAF; MEK
1451309_at	Arhgap1	Rho GTPase activating protein 1	4.39	0.01	1.34	

Probeset ID	Symbol	Gene Name	d-value	q-value	R-fold	Expression reversed by
1431176_at	Chmp4c	chromatin modifying protein 4C	4.39	0.01	1.79	n.r. ;
1423574_s_at	Srd5a3	steroid 5 alpha-reductase 3	4.39	0.01	1.28	; MEK
1438678_at	1500011K16Rik	RIKEN cDNA 1500011K16 gene	4.39	0.01	1.49	BRAF; MEK
1434129_s_at	Lhfpl2	lipoma HMGIC fusion partner-like 2	4.39	0.01	2.18	BRAF; MEK
1438532_at	Hmcn1	hemicentin 1	4.39	0.01	5.22	BRAF; MEK
1457825_x_at	Tcn2	transcobalamin 2	4.39	0.01	2.47	BRAF; MEK
1456737_x_at	Acaa1a	acetyl-Coenzyme A acyltransferase 1A	4.39	0.01	1.34	
1419292_at	Htra3	HtrA serine peptidase 3	4.39	0.01	1.39	
1424444_a_at	1600014C10Rik	RIKEN cDNA 1600014C10 gene	4.39	0.01	1.50	BRAF; MEK
1420282_s_at	Prss29	protease. serine. 29	4.38	0.01	1.20	BRAF; MEK
1455939_x_at	Srp14	signal recognition particle 14	4.38	0.01	1.38	
1436101_at	Pank2	pantothenate kinase 2 (Hallervorden-Spatz syndrome)	4.38	0.01	1.87	BRAF
1424650_at	Pdia5	protein disulfide isomerase associated 5	4.38	0.01	1.34	; MEK
1424638_at	Cdkn1a	cyclin-dependent kinase inhibitor 1A (P21)	4.38	0.01	1.50 *	; MEK
1417848_at	Zfp704	zinc finger protein 704	4.38	0.01	1.89	
1454215_at	2410007B07Rik	RIKEN cDNA 2410007B07 gene	4.38	0.01	1.18	
1426642_at	Fn1	fibronectin 1	4.38	0.01	1.17	BRAF; MEK
1418049_at	Ltbp3	latent transforming growth factor beta binding protein 3	4.38	0.01	1.70	
1424691_at	5930434B04Rik	RIKEN cDNA 5930434B04 gene	4.37	0.01	1.40	BRAF
1426646_at	9130011J15Rik	RIKEN cDNA 9130011J15 gene	4.37	0.01	1.49	; MEK
1427035_at	Slc39a14	solute carrier family 39 (zinc transporter). member 14	4.37	0.01	1.25	
1455113_at	Armc8	armadillo repeat containing 8	4.37	0.01	1.89	BRAF
1460728_s_at	Ing4	inhibitor of growth family. member 4	4.37	0.01	1.40	BRAF; MEK
1419981_at	Riok3	RIO kinase 3 (yeast)	4.37	0.01	1.19	
1418419_at	Fbxl20	F-box and leucine-rich repeat protein 20	4.37	0.01	1.40	; MEK
1455595_at	Ugt2b36	UDP glucuronosyltransferase 2 family. polypeptide B36	4.37	0.01	1.26	BRAF; MEK
1417399_at	Gas6	growth arrest specific 6	4.37	0.01	3.40	; MEK
1424521_at	Zfand2b	zinc finger. AN1 type domain 2B	4.37	0.01	1.69	BRAF; MEK
1439860_at	Eef2k	eukaryotic elongation factor-2 kinase	4.37	0.01	1.44	
1449437_at	D6Wsu163e	DNA segment. Chr 6. Wayne State University 163. expressed	4.36	0.01	1.88	BRAF; MEK
1445170_at	2700012I20Rik	RIKEN cDNA 2700012I20 gene	4.36	0.01	1.27	; MEK
1435345_at	Cercam	cerebral endothelial cell adhesion molecule	4.36	0.01	1.53	BRAF
1415727_at	Apoa1bp	apolipoprotein A-I binding protein	4.36	0.01	1.56	BRAF
1415732_at	Bat5	HLA-B associated transcript 5	4.36	0.01	1.43	BRAF
1438061_at	4930523C07Rik	RIKEN cDNA 4930523C07 gene	4.36	0.01	1.62	BRAF; MEK
1455543_at	Klhl18	kelch-like 18 (Drosophila)	4.36	0.01	1.41	
1416011_x_at	Ehd1	EH-domain containing 1	4.36	0.01	1.52	BRAF; MEK
1416947_s_at	Acaa1a	acetyl-Coenzyme A acyltransferase 1A	4.36	0.01	1.55	BRAF
1436294_at	Ankrd29	ankyrin repeat domain 29	4.35	0.01	2.44	BRAF
1460330_at	Anxa3	annexin A3	4.35	0.01	1.21	
1436208_at	Asb1	ankyrin repeat and SOCS box-containing 1	4.35	0.01	1.35	BRAF; MEK
1441208_at	NA	NA	4.35	0.01	1.44	; MEK
1448301_s_at	Serpinb1a	serine (or cysteine) peptidase inhibitor. clade B. member 1a	4.35	0.01	3.30	
1429153_at	6530406A20Rik	RIKEN cDNA 6530406A20 gene	4.35	0.01	1.41	
1423204_at	Tm9sf4	transmembrane 9 superfamily protein member 4	4.35	0.01	1.24	BRAF
1424074_at	Btf3l4	basic transcription factor 3-like 4	4.35	0.01	1.43	; n.r.
1455710_x_at	Mtcp1	mature T-cell proliferation 1	4.35	0.01	1.44	; MEK
1432377_x_at	Shroom3	shroom family member 3	4.35	0.01	1.40	BRAF; MEK
1449099_at	Lrba	LPS-responsive beige-like anchor	4.35	0.01	1.58	n.r. ;
1417960_at	Cpeb1	cytoplasmic polyadenylation element binding protein 1	4.35	0.01	2.70	BRAF; MEK
1434369_a_at	Cryab	crystallin. alpha B	4.35	0.01	2.02	
1444736_at	Cdh7	cadherin 7. type 2	4.35	0.01	1.14	
1460703_at	Ascc1	activating signal cointegrator 1 complex subunit 1	4.34	0.01	1.27	n.r. ; n.r.
1430326_s_at	Uqcrq	ubiquinol-cytochrome c reductase. complex III subunit VII	4.34	0.01	1.46	BRAF; MEK
1428612_at	Atg7	autophagy-related 7 (yeast)	4.34	0.01	1.35	
1458790_at	NA	NA	4.34	0.01	1.17	

Probeset ID	*Symbol*	*Gene Name*	*d-value*	*q-value*	*R-fold*	*Expression reversed by*
1455825_s_at	Lnx1	ligand of numb-protein X 1	4.34	0.01	1.25	
1423779_at	Chchd6	coiled-coil-helix-coiled-coil-helix domain containing 6	4.34	0.01	1.43	n.r. ; MEK
1456243_x_at	Mcl1	myeloid cell leukemia sequence 1	4.34	0.01	1.79	
1448648_at	9130005N14Rik	RIKEN cDNA 9130005N14 gene	4.34	0.01	1.46	BRAF; MEK
1428058_at	Ahnak	AHNAK nucleoprotein (desmoyokin)	4.34	0.01	2.10	
1418793_at	Idua	iduronidase. alpha-L-	4.34	0.01	1.26	
1428075_at	Ndufb4	NADH dehydrogenase (ubiquinone) 1 beta subcomplex 4	4.34	0.01	1.40	
1451159_at	Arhgef12	Rho guanine nucleotide exchange factor (GEF) 12	4.34	0.01	1.25	BRAF
1416012_at	Ehd1	EH-domain containing 1	4.33	0.01	1.75	
1448200_at	Tcn2	transcobalamin 2	4.33	0.01	2.50	BRAF; MEK
1448483_a_at	Ndufb2	NADH dehydrogenase (ubiquinone) 1 beta subcomplex. 2	4.33	0.01	1.31	
1460241_a_at	St3gal5	ST3 beta-galactoside alpha-2.3-sialyltransferase 5	4.33	0.01	1.82	BRAF; MEK
1439592_at	LOC100043911	hypothetical protein LOC100043911	4.33	0.01	1.70	BRAF; MEK
1429762_a_at	Bbs5	Bardet-Biedl syndrome 5 (human)	4.33	0.01	1.54	BRAF; MEK
1455712_at	Hist3h2a	histone cluster 3. H2a	4.33	0.01	2.30	
1459809_x_at	1700063D05Rik	RIKEN cDNA 1700063D05 gene	4.33	0.01	1.12	
1435559_at	Myo6	myosin VI	4.33	0.01	1.28	
1441206_at	Synpo2	synaptopodin 2	4.32	0.01	1.72	BRAF; MEK
1427410_at	Dleu2	deleted in lymphocytic leukemia. 2	4.32	0.01	1.94	BRAF; n.r.
1436388_a_at	3830406C13Rik	RIKEN cDNA 3830406C13 gene	4.32	0.01	1.81	
1434768_at	Tpp1	tripeptidyl peptidase I	4.32	0.01	2.12	BRAF; MEK
1436544_at	Atp10d	ATPase. class V. type 10D	4.32	0.01	1.72	
1446771_at	Tuba8	tubulin. alpha 8	4.32	0.01	1.16	
1443729_at	Mtss1	metastasis suppressor 1	4.32	0.01	1.33	BRAF
1419137_at	Shank3	SH3/ankyrin domain gene 3	4.32	0.01	1.88	
1450879_at	Atp9b	ATPase. class II. type 9B	4.32	0.01	1.35	BRAF; MEK
1422594_at	5730470L24Rik	RIKEN cDNA 5730470L24 gene	4.32	0.01	1.45	BRAF; MEK
1437058_at	Megf6	multiple EGF-like-domains 6	4.32	0.01	1.26	
1428330_at	Dopey2	dopey family member 2	4.31	0.01	2.01	; MEK
1416007_at	Satb1	special AT-rich sequence binding protein 1	4.31	0.01	3.28	
1456100_at	BC089491	cDNA sequence BC089491	4.31	0.01	1.22	
1428211_at	4933406E20Rik	RIKEN cDNA 4933406E20 gene	4.31	0.01	1.57	BRAF
1450140_a_at	Cdkn2a	cyclin-dependent kinase inhibitor 2A	3.89	0.01	3.97 *	n.r. ; n.r.
1424229_at	Dyrk3	dual-specificity tyrosine-?-phosphorylation regulated kinase 3	-4.03	0.01	0.41	
1454929_s_at	Safb	scaffold attachment factor B	-4.03	0.01	0.72	BRAF; MEK
1417951_at	Eno3	enolase 3. beta muscle	-4.03	0.01	0.48	BRAF; MEK
1452192_at	Naf1	nuclear assembly factor 1 homolog (S. cerevisiae)	-4.03	0.01	0.71	BRAF
1440715_s_at	Cdkn2aipnl	CDKN2A interacting protein N-terminal like	-4.03	0.01	0.59	; MEK
1448230_at	Usp10	ubiquitin specific peptidase 10	-4.03	0.01	0.68	
1455524_at	2810046L04Rik	RIKEN cDNA 2810046L04 gene	-4.03	0.01	0.77	BRAF; MEK
1424412_at	Ogfrl1	opioid growth factor receptor-like 1	-4.03	0.01	0.69	BRAF
1435730_at	D930026N18Rik	RIKEN cDNA D930026N18 gene	-4.03	0.01	0.68	; MEK
1441910_x_at	Ccne1	cyclin E1	-4.03	0.01	0.58 *	
1417886_at	Ints5	integrator complex subunit 5	-4.04	0.01	0.75	
1438735_at	Rsf1	remodeling and spacing factor 1	-4.04	0.01	0.71	
1427403_at	Slco1a5	solute carrier organic anion transporter family. member 1a5	-4.04	0.01	0.83	
1424938_at	Steap1	six transmembrane epithelial antigen of the prostate 1	-4.04	0.01	0.37	; MEK
1435174_at	Rsbn1	rosbin. round spermatid basic protein 1	-4.04	0.01	0.61	; MEK
1454875_a_at	Rbbp4	retinoblastoma binding protein 4	-4.04	0.01	0.62	
1416152_a_at	Sfrs3	splicing factor. arginine/serine-rich 3 (SRp20)	-4.04	0.01	0.67	
1438354_x_at	Cnn3	calponin 3. acidic	-4.04	0.01	0.79	; MEK
1419266_at	Nfyb	nuclear transcription factor-Y beta	-4.04	0.01	0.73	
1423882_at	Rfwd3	ring finger and WD repeat domain 3	-4.04	0.01	0.59	BRAF; MEK
1424173_at	Tmem48	transmembrane protein 48	-4.04	0.01	0.53	BRAF
1435439_at	Dgcr8	DiGeorge syndrome critical region gene 8	-4.04	0.01	0.74	
1417082_at	Anp32b	acidic (leucine-rich) nuclear phosphoprotein 32 family. member B	-4.04	0.01	0.65	; MEK

Probeset ID	Symbol	Gene Name	d-value	q-value	R-fold	Expression reversed by
1423539_at	Pms2	postmeiotic segregation increased 2 (S. cerevisiae)	-4.04	0.01	0.68	; MEK
1419940_at	C030018P15Rik	RIKEN cDNA C030018P15 gene	-4.05	0.01	0.74	
1429404_at	2010317E24Rik	RIKEN cDNA 2010317E24 gene	-4.05	0.01	0.60	
1428407_at	Hnrnpa0	heterogeneous nuclear ribonucleoprotein A0	-4.05	0.01	0.77	
1449044_at	Eef1e1	eukaryotic translation elongation factor 1 epsilon 1	-4.05	0.01	0.78	
1456433_at	H2afy2	H2A histone family. member Y2	-4.05	0.01	0.80	
1426755_at	Ckap4	cytoskeleton-associated protein 4	-4.05	0.01	0.69	
1417238_at	Ewsr1	Ewing sarcoma breakpoint region 1	-4.05	0.01	0.64	
1423829_at	0910001A06Rik	RIKEN cDNA 0910001A06 gene	-4.05	0.01	0.72	BRAF; MEK
1459805_x_at	Dus3l	dihydrouridine synthase 3-like (S. cerevisiae)	-4.05	0.01	0.75	BRAF; MEK
1454764_s_at	Slc38a1	solute carrier family 38. member 1	-4.05	0.01	0.45	n.r. ;
1449090_a_at	Yes1	Yamaguchi sarcoma viral (v-yes) oncogene homolog 1	-4.05	0.01	0.72	
1443466_s_at	Polr3b	polymerase (RNA) III (DNA directed) polypeptide B	-4.05	0.01	0.79	; MEK
1422801_at	G3bp1	Ras-GTPase-activating protein SH3-domain binding protein 1	-4.05	0.01	0.67	BRAF; MEK
1452107_s_at	NA	NA	-4.05	0.01	0.17	BRAF; MEK
1454064_a_at	Rnf138	ring finger protein 138	-4.05	0.01	0.68	
1429242_at	1110054O05Rik	RIKEN cDNA 1110054O05 gene	-4.06	0.01	0.68	
1428533_at	D1Bwg0212e	DNA segment. Chr 1. Brigham & Women's Genetics 0212 expressed	-4.06	0.01	0.75	BRAF; MEK
1452857_at	Crebzf	CREB/ATF bZIP transcription factor	-4.06	0.01	0.56	; MEK
1419152_at	2810417H13Rik	RIKEN cDNA 2810417H13 gene	-4.06	0.01	0.61	BRAF; MEK
1433606_at	Dcp1a	DCP1 decapping enzyme homolog A (S. cerevisiae)	-4.06	0.01	0.74	
1455317_at	Epc2	enhancer of polycomb homolog 2 (Drosophila)	-4.06	0.01	0.70	
1453293_a_at	2810408A11Rik	RIKEN cDNA 2810408A11 gene	-4.06	0.01	0.66	BRAF; n.r.
1428301_at	ENSMUSG00000068790	predicted gene. ENSMUSG00000068790	-4.06	0.01	0.48	BRAF
1419469_at	Gnb4	guanine nucleotide binding protein (G protein). beta 4	-4.06	0.01	0.42	
1418634_at	Notch1	Notch gene homolog 1 (Drosophila)	-4.07	0.01	0.49	BRAF; MEK
1451532_s_at	Steap1	six transmembrane epithelial antigen of the prostate 1	-4.07	0.01	0.46	BRAF; MEK
1426369_at	Far1	fatty acyl CoA reductase 1	-4.07	0.01	0.78	
1452350_at	Brd8	bromodomain containing 8	-4.07	0.01	0.51	
1434753_at	Nfrkb	nuclear factor related to kappa B binding protein	-4.07	0.01	0.79	BRAF; MEK
1431075_a_at	Eif4enif1	eukaryotic translation initiation factor 4E nuclear import factor 1	-4.07	0.01	0.74	BRAF; MEK
1434336_s_at	Rcor1	REST corepressor 1	-4.08	0.01	0.63	BRAF; MEK
1460716_a_at	Cbfb	core binding factor beta	-4.08	0.01	0.67	
1426964_at	3110003A17Rik	RIKEN cDNA 3110003A17 gene	-4.08	0.01	0.72	
1455048_at	Igsf3	immunoglobulin superfamily. member 3	-4.08	0.01	0.56	
1438955_x_at	Ppif	peptidylprolyl isomerase F (cyclophilin F)	-4.08	0.01	0.67	BRAF; MEK
1437682_x_at	1110004E09Rik	RIKEN cDNA 1110004E09 gene	-4.08	0.01	0.76	
1437634_at	Thoc2	THO complex 2	-4.08	0.01	0.74	
1419402_at	Mns1	meiosis-specific nuclear structural protein 1	-4.08	0.01	0.69	; MEK
1416595_at	Mrps22	mitochondrial ribosomal protein S22	-4.08	0.01	0.69	BRAF; MEK
1437286_x_at	1110020G09Rik	RIKEN cDNA 1110020G09 gene	-4.08	0.01	0.82	
1424147_at	Ahsa1	AHA1. activator of heat shock protein ATPase homolog 1 (yeast)	-4.08	0.01	0.76	BRAF; MEK
1417183_at	Dnaja2	DnaJ (Hsp40) homolog. subfamily A. member 2	-4.08	0.01	0.78	BRAF
1436585_at	BB182297	expressed sequence BB182297	-4.08	0.01	0.63	; MEK
1442618_at	NA	NA	-4.08	0.01	0.74	
1433398_at	Fgd3	FYVE. RhoGEF and PH domain containing 3	-4.08	0.01	0.45	; n.r.
1418772_at	BC016423	cDNA sequence BC016423	-4.08	0.01	0.63	; MEK
1424166_at	Msh3	mutS homolog 3 (E. coli)	-4.09	0.01	0.53	BRAF; MEK
1457830_at	NA	NA	-4.09	0.01	0.65	BRAF; MEK
1454817_at	Utp18	UTP18. small subunit (SSU) processome component. homolog (yeast)	-4.09	0.01	0.65	BRAF
1426683_at	Cnot6	CCR4-NOT transcription complex. subunit 6	-4.09	0.01	0.68	; MEK
1416650_at	Rfpl4	ret finger protein-like 4	-4.09	0.01	0.89	
1452552_at	Npn2	neoplastic progression 2	-4.09	0.01	0.84	BRAF
1428251_at	Smchd1	SMC hinge domain containing 1	-4.09	0.01	0.45	BRAF; MEK
1441062_at	Ddx11	DEAD/H (Asp-Glu-Ala-Asp/His) box polypeptide 11 (CHL1-like helicase homolog. S. cerevisiae)	-4.09	0.01	0.52	BRAF; MEK

Probeset ID	Symbol	Gene Name	d-value	q-value	R-fold	Expression reversed by
1456058_at	Rbm27	RNA binding motif protein 27	-4.09	0.01	0.66	; MEK
1451144_at	Bxdc2	brix domain containing 2	-4.09	0.01	0.62	; MEK
1418835_at	Phlda1	pleckstrin homology-like domain. family A. member 1	-4.09	0.01	0.33	BRAF; MEK
1423480_at	Nol11	nucleolar protein 11	-4.09	0.01	0.74	BRAF; MEK
1424314_at	Prpf3	PRP3 pre-mRNA processing factor 3 homolog (yeast)	-4.10	0.01	0.70	BRAF; MEK
1436499_at	Sgms1	sphingomyelin synthase 1	-4.10	0.01	0.59	n.r. ; n.r.
1423586_at	Axl	AXL receptor tyrosine kinase	-4.10	0.01	0.49	BRAF
1438404_at	Rnf144a	ring finger protein 144A	-4.10	0.01	0.52	; MEK
1427504_s_at	Sfrs2	splicing factor. arginine/serine-rich 2 (SC-35)	-4.10	0.01	0.67	BRAF
1426012_a_at	2610301G19Rik	RIKEN cDNA 2610301G19 gene	-4.10	0.01	0.74	; MEK
1433706_a_at	Ptplad1	protein tyrosine phosphatase-like A domain containing 1	-4.10	0.01	0.76	
1436654_at	Gen1	Gen homolog 1. endonuclease (Drosophila)	-4.11	0.01	0.59	BRAF; MEK
1454703_x_at	Snhg1	small nucleolar RNA host gene (non-protein coding) 1	-4.11	0.01	0.54	
1439435_x_at	Pgk1	phosphoglycerate kinase 1	-4.11	0.01	0.82	; MEK
1416536_at	Mum1	melanoma associated antigen (mutated) 1	-4.11	0.01	0.64	BRAF; MEK
1426803_at	Rbm26	RNA binding motif protein 26	-4.11	0.01	0.66	n.r. ; MEK
1418443_at	Xpo1	exportin 1. CRM1 homolog (yeast)	-4.11	0.01	0.51	BRAF; MEK
1433650_at	Narfl	nuclear prelamin A recognition factor-like	-4.11	0.01	0.84	
1428145_at	Acaa2	acetyl-Coenzyme A acyltransferase 2 (mitochondrial 3-oxoacyl-Coenzyme A thiolase)	-4.11	0.01	0.68	BRAF; MEK
1415869_a_at	Trim28	tripartite motif-containing 28	-4.11	0.01	0.61	BRAF; MEK
1419912_s_at	Strap	serine/threonine kinase receptor associated protein	-4.11	0.01	0.71	BRAF
1416746_at	H2afx	H2A histone family. member X	-4.11	0.01	0.55	BRAF; MEK
1435655_at	Rpl12	ribosomal protein L12	-4.11	0.01	0.45	BRAF; MEK
1460357_at	Ythdf2	YTH domain family 2	-4.12	0.01	0.78	BRAF; MEK
1424413_at	Ogfrl1	opioid growth factor receptor-like 1	-4.12	0.01	0.69	BRAF; MEK
1448358_s_at	Snrpg	small nuclear ribonucleoprotein polypeptide G	-4.12	0.01	0.63	BRAF; MEK
1426433_at	Myct1	myc target 1	-4.12	0.01	0.21	; n.r.
1437497_a_at	Hsp90aa1	heat shock protein 90. alpha (cytosolic). class A member 1	-4.12	0.01	0.69	
1458172_at	Me2	malic enzyme 2. NAD?-dependent. mitochondrial	-4.12	0.01	0.82	
1433928_a_at	Rpl13a	ribosomal protein L13a	-4.12	0.01	0.73	; MEK
1433830_at	Hnrnpa2b1	heterogeneous nuclear ribonucleoprotein A2/B1	-4.12	0.01	0.42	; MEK
1456340_at	Mettl11a	methyltransferase like 11A	-4.12	0.01	0.76	
1438212_at	Vil1	villin 1	-4.12	0.01	0.69	BRAF; MEK
1423489_at	Mmd	monocyte to macrophage differentiation-associated	-4.12	0.01	0.33	BRAF; MEK
1434836_at	Nfatc2ip	nuclear factor of activated T-cells. cytoplasmic. calcineurin-dependent 2 interacting protein	-4.12	0.01	0.67	
1433656_a_at	Gnl3	guanine nucleotide binding protein-like 3 (nucleolar)	-4.12	0.01	0.73	
1429871_at	Hmmr	hyaluronan mediated motility receptor (RHAMM)	-4.12	0.01	0.59	BRAF; MEK
1419819_s_at	Sec63	SEC63-like (S. cerevisiae)	-4.12	0.01	0.66	n.r. ; n.r.
1442454_at	Top2a	topoisomerase (DNA) II alpha	-4.12	0.01	0.44	BRAF; MEK
1460011_at	Cyp26b1	cytochrome P450. family 26. subfamily b. polypeptide 1	-4.12	0.01	0.70	BRAF; MEK
1438076_at	Rpl30	ribosomal protein L30	-4.12	0.01	0.39	; MEK
1435077_at	Asxl1	additional sex combs like 1 (Drosophila)	-4.13	0.01	0.61	BRAF; MEK
1453928_a_at	Ssb	Sjogren syndrome antigen B	-4.13	0.01	0.60	
1448802_at	Nufip1	nuclear fragile X mental retardation protein interacting protein 1	-4.13	0.01	0.73	
1428223_at	Mfsd2	major facilitator superfamily domain containing 2	-4.13	0.01	0.43	
1427764_a_at	Tcfe2a	transcription factor E2a	-4.13	0.01	0.58	BRAF; MEK
1457374_at	Nedd4l	neural precursor cell expressed. developmentally down-regulated gene 4-like	-4.13	0.01	0.80	; MEK
1417319_at	Pvrl3	poliovirus receptor-related 3	-4.13	0.01	0.60	BRAF; MEK
1420793_at	Mup4	major urinary protein 4	-4.13	0.01	0.88	
1421177_at	Thap2	THAP domain containing. apoptosis associated protein 2	-4.13	0.01	0.77	; MEK
1436572_at	Ccdc45	coiled-coil domain containing 45	-4.13	0.01	0.69	BRAF; MEK
1419741_at	Supt16h	suppressor of Ty 16 homolog (S. cerevisiae)	-4.13	0.01	0.65	BRAF; MEK
1451241_at	Lamb1-1	laminin B1 subunit 1	-4.14	0.01	0.39	
1426862_at	Aftph	aftiphilin	-4.14	0.01	0.77	n.r. ;

Probeset ID	*Symbol*	*Gene Name*	*d-value*	*q-value*	*R-fold*	*Expression reversed by*
1452098_at	Chtf18	CTF18. chromosome transmission fidelity factor 18 homolog (S. cerevisiae)	-4.14	0.01	0.34	BRAF; MEK
1448650_a_at	Pole	polymerase (DNA directed). epsilon	-4.14	0.01	0.29	BRAF; MEK
1455725_a_at	LOC100045490	similar to H3 histone. family 3A	-4.15	0.01	0.76	BRAF; MEK
1427982_s_at	Syne2	synaptic nuclear envelope 2	-4.15	0.01	0.28	BRAF; MEK
1425974_a_at	Trim25	tripartite motif-containing 25	-4.15	0.01	0.61	
1426495_at	2410042D21Rik	RIKEN cDNA 2410042D21 gene	-4.15	0.01	0.76	n.r. ; n.r.
1455675_a_at	Tial1	Tia1 cytotoxic granule-associated RNA binding protein-like 1	-4.15	0.01	0.72	
1451972_at	LOC100046012	similar to glucocorticoid induced transcript 1	-4.15	0.01	0.50	
1459358_at	NA	NA	-4.15	0.01	0.62	n.r. ; n.r.
1448103_s_at	Nono	non-POU-domain-containing. octamer binding protein	-4.15	0.01	0.76	BRAF; MEK
1415913_at	Rps13	ribosomal protein S13	-4.16	0.01	0.75	
1437516_at	NA	NA	-4.16	0.01	0.74	; n.r.
1429832_at	Ppih	peptidyl prolyl isomerase H	-4.16	0.01	0.66	BRAF; MEK
1416393_at	Emg1	EMG1 nucleolar protein homolog (S. cerevisiae)	-4.16	0.01	0.67	BRAF; MEK
1423161_s_at	Spred1	sprouty protein with EVH-1 domain 1. related sequence	-4.16	0.01	0.64	
1424089_a_at	Tcf4	transcription factor 4	-4.16	0.01	0.59	; MEK
1415899_at	Junb	Jun-B oncogene	-4.16	0.01	0.38	BRAF; MEK
1448029_at	Tbx3	T-box 3	-4.16	0.01	0.56	BRAF; MEK
1434419_s_at	Tardbp	TAR DNA binding protein	-4.16	0.01	0.72	
1437032_x_at	Rbm14	RNA binding motif protein 14	-4.16	0.01	0.67	BRAF; MEK
1428073_a_at	Nup88	nucleoporin 88	-4.16	0.01	0.79	BRAF; MEK
1433697_at	Patl1	protein associated with topoisomerase II homolog 1 (yeast)	-4.16	0.01	0.78	BRAF; MEK
1455915_at	Galnt4	UDP-N-acetyl-alpha-D-galactosamine:polypeptide N-acetylgalactosaminyltransferase 4	-4.17	0.01	0.65	BRAF
1423460_at	Gigyf1	GRB10 interacting GYF protein 1	-4.17	0.01	0.79	BRAF; MEK
1433543_at	Anln	anillin. actin binding protein	-4.17	0.01	0.35	BRAF; MEK
1455206_at	NA	NA	-4.17	0.01	0.62	BRAF; MEK
1444342_at	Nup155	nucleoporin 155	-4.17	0.01	0.63	BRAF; MEK
1426613_a_at	Snrpb2	U2 small nuclear ribonucleoprotein B	-4.17	0.01	0.73	BRAF; MEK
1424469_a_at	Cpsf4	cleavage and polyadenylation specific factor 4	-4.17	0.01	0.68	BRAF; MEK
1427685_a_at	Synj2	synaptojanin 2	-4.17	0.01	0.38	BRAF
1417166_at	Psip1	PC4 and SFRS1 interacting protein 1	-4.17	0.01	0.50	BRAF; MEK
1450077_at	Chd1	chromodomain helicase DNA binding protein 1	-4.17	0.01	0.60	
1437389_x_at	Khdrbs1	KH domain containing. RNA binding. signal transduction associated 1	-4.17	0.01	0.61	BRAF; MEK
1452999_at	Smndc1	survival motor neuron domain containing 1	-4.17	0.01	0.80	BRAF
1423517_at	LOC100038887	hypothetical protein LOC100038887	-4.17	0.01	0.80	BRAF; MEK
1437051_at	Dffb	DNA fragmentation factor. beta subunit	-4.17	0.01	0.81	
1428529_at	2810026P18Rik	RIKEN cDNA 2810026P18 gene	-4.17	0.01	0.69	; MEK
1420530_at	Dpf1	D4. zinc and double PHD fingers family 1	-4.18	0.01	0.72	BRAF; MEK
1452046_a_at	Ppp1cc	protein phosphatase 1. catalytic subunit. gamma isoform	-4.18	0.01	0.71	
1425306_at	BC027072	cDNA sequence BC027072	-4.18	0.01	0.86	; MEK
1452606_at	Mnd1	meiotic nuclear divisions 1 homolog (S. cerevisiae)	-4.18	0.01	0.51	BRAF; MEK
1451273_x_at	Ftsjd1	FtsJ methyltransferase domain containing 1	-4.18	0.01	0.72	BRAF
1418274_at	Nutf2	nuclear transport factor 2	-4.18	0.01	0.73	BRAF
1426086_a_at	Fmr1	fragile X mental retardation syndrome 1 homolog	-4.18	0.01	0.36	n.r. ; n.r.
1431873_a_at	Tube1	epsilon-tubulin 1	-4.18	0.01	0.48	BRAF
1416559_at	1500003O22Rik	RIKEN cDNA 1500003O22 gene	-4.18	0.01	0.61	BRAF
1423269_a_at	Nedd4l	neural precursor cell expressed. developmentally down-regulated gene 4-like	-4.18	0.01	0.63	; MEK
1425747_at	Dock5	dedicator of cytokinesis 5	-4.18	0.01	0.44	; MEK
1444443_at	NA	NA	-4.18	0.01	0.76	; MEK
1434966_at	Sfrs8	splicing factor. arginine/serine-rich 8	-4.18	0.01	0.74	
1416798_a_at	Nme4	non-metastatic cells 4. protein expressed in	-4.18	0.01	0.62	BRAF
1459740_s_at	Ucp2	uncoupling protein 2 (mitochondrial. proton carrier)	-4.19	0.01	0.72	; MEK
1423192_at	Pspc1	paraspeckle protein 1	-4.19	0.01	0.60	; MEK
1434182_at	Ddx31	DEAD/H (Asp-Glu-Ala-Asp/His) box polypeptide 31	-4.19	0.01	0.78	

Probeset ID	Symbol	Gene Name	d-value	q-value	R-fold	Expression reversed by
1435645_at	Mmd	monocyte to macrophage differentiation-associated	-4.19	0.01	0.52	; MEK
1456005_a_at	Bcl2l11	BCL2-like 11 (apoptosis facilitator)	-4.19	0.01	0.45	BRAF
1443814_x_at	Ctsh	cathepsin H	-4.19	0.01	0.59	BRAF
1429508_at	2310057M21Rik	RIKEN cDNA 2310057M21 gene	-4.19	0.01	0.81	
1435923_at	Ado	2-aminoethanethiol (cysteamine) dioxygenase	-4.19	0.01	0.74	BRAF; MEK
1426799_at	Rab8b	RAB8B. member RAS oncogene family	-4.19	0.01	0.73	; MEK
1438447_at	Ddx11	DEAD/H (Asp-Glu-Ala-Asp/His) box polypeptide 11 (CHL1-like helicase homolog. S. cerevisiae)	-4.19	0.01	0.55	
1419239_at	Zfp54	zinc finger protein 54	-4.19	0.01	0.64	
1439648_at	Anln	anillin. actin binding protein	-4.19	0.01	0.51	BRAF; MEK
1452816_at	Mlf1ip	myeloid leukemia factor 1 interacting protein	-4.20	0.01	0.72	; MEK
1426897_at	Rcc2	regulator of chromosome condensation 2	-4.20	0.01	0.62	BRAF; MEK
1455988_a_at	LOC100038887	hypothetical protein LOC100038887	-4.20	0.01	0.77	BRAF; MEK
1427258_at	Trim24	tripartite motif-containing 24	-4.20	0.01	0.61	BRAF; MEK
1456325_at	Cep68	centrosomal protein 68	-4.20	0.01	0.66	BRAF; MEK
1448361_at	Ttc3	tetratricopeptide repeat domain 3	-4.20	0.01	0.57	; MEK
1444646_at	8430420F16Rik	RIKEN cDNA 8430420F16 gene	-4.20	0.01	0.57	BRAF; MEK
1455899_x_at	Socs3	suppressor of cytokine signaling 3	-4.20	0.01	0.40	BRAF; MEK
1417434_at	Gpd2	glycerol phosphate dehydrogenase 2. mitochondrial	-4.20	0.01	0.65	BRAF; MEK
1428827_at	Whsc1	Wolf-Hirschhorn syndrome candidate 1 (human)	-4.20	0.01	0.36	BRAF; MEK
1440263_at	Nrp	neural regeneration protein	-4.20	0.01	0.62	
1418424_at	Tnfaip6	tumor necrosis factor alpha induced protein 6	-4.20	0.01	0.51	BRAF; MEK
1433631_at	Eif5	eukaryotic translation initiation factor 5	-4.20	0.01	0.76	
1442519_at	NA	NA	-4.21	0.01	0.73	
1417268_at	Cd14	CD14 antigen	-4.21	0.01	0.41	BRAF; MEK
1416150_a_at	Sfrs3	splicing factor. arginine/serine-rich 3 (SRp20)	-4.21	0.01	0.65	BRAF; MEK
1429485_a_at	Utp11l	UTP11-like. U3 small nucleolar ribonucleoprotein. (yeast)	-4.21	0.01	0.76	; MEK
1416724_x_at	Tcf4	transcription factor 4	-4.21	0.01	0.58	; MEK
1428677_at	Wdr73	WD repeat domain 73	-4.22	0.01	0.81	BRAF; MEK
1421750_a_at	Vbp1	von Hippel-Lindau binding protein 1	-4.22	0.01	0.82	n.r. ; MEK
1423470_at	Ptbp2	polypyrimidine tract binding protein 2	-4.22	0.01	0.50	BRAF; MEK
1448389_at	Wdr5	WD repeat domain 5	-4.22	0.01	0.63	BRAF
1417560_at	Sfxn1	sideroflexin 1	-4.22	0.01	0.73	BRAF; MEK
1420406_at	Peg12	paternally expressed 12	-4.22	0.01	0.57	BRAF; n.r.
1434120_a_at	Metap2	methionine aminopeptidase 2	-4.22	0.01	0.84	; MEK
1417190_at	Nampt	nicotinamide phosphoribosyltransferase	-4.22	0.01	0.59	BRAF; MEK
1434242_at	Usp37	ubiquitin specific peptidase 37	-4.22	0.01	0.58	BRAF; MEK
1438115_a_at	Slc9a3r1	solute carrier family 9 (sodium/hydrogen exchanger). member 3 regulator 1	-4.22	0.01	0.53	BRAF; MEK
1435627_x_at	Marcksl1	MARCKS-like 1	-4.23	0.01	0.54	BRAF; MEK
1423596_at	Nek6	NIMA (never in mitosis gene a)-related expressed kinase 6	-4.23	0.01	0.69	; MEK
1416621_at	Llgl1	lethal giant larvae homolog 1 (Drosophila)	-4.23	0.01	0.57	BRAF; MEK
1455820_x_at	Scarb1	scavenger receptor class B. member 1	-4.23	0.01	0.51	BRAF; MEK
1425268_a_at	Pear1	platelet endothelial aggregation receptor 1	-4.23	0.01	0.61	; MEK
1454957_at	Nob1	NIN1/RPN12 binding protein 1 homolog (S. cerevisiae)	-4.23	0.01	0.80	BRAF; MEK
1417736_at	Smc6	structural maintenance of chromosomes 6	-4.23	0.01	0.55	n.r. ; n.r.
1460420_a_at	Egfr	epidermal growth factor receptor	-4.23	0.01	0.71	
1453600_at	Ccdc18	coiled-coil domain containing 18	-4.23	0.01	0.67	BRAF; MEK
1448778_at	Sfrs4	splicing factor. arginine/serine-rich 4 (SRp75)	-4.24	0.01	0.52	BRAF; MEK
1452833_at	Rapgef2	Rap guanine nucleotide exchange factor (GEF) 2	-4.24	0.01	0.68	BRAF; MEK
1449158_at	Kcnk2	potassium channel. subfamily K. member 2	-4.24	0.01	0.37	BRAF; MEK
1416461_at	Caprin1	cell cycle associated protein 1	-4.24	0.01	0.74	; MEK
1446760_at	Trip13	thyroid hormone receptor interactor 13	-4.24	0.01	0.79	BRAF; MEK
1437375_at	NA	NA	-4.24	0.01	0.73	; MEK
1425336_x_at	H2-K1	histocompatibility 2. K1. K region	-4.24	0.01	0.38	; MEK
1454984_at	Lifr	leukemia inhibitory factor receptor	-4.24	0.01	0.42	BRAF; MEK
1456261_at	Sh3kbp1	SH3-domain kinase binding protein 1	-4.24	0.01	0.40	BRAF; MEK

Probeset ID	Symbol	Gene Name	d-value	q-value	R-fold	Expression reversed by
1439403_x_at	Rnf12	ring finger protein 12	-4.24	0.01	0.69	; MEK
1452128_a_at	Brcc3	BRCA1/BRCA2-containing complex. subunit 3	-4.25	0.01	0.77	n.r. ; MEK
1450333_a_at	Gata2	GATA binding protein 2	-4.25	0.01	0.62	BRAF; MEK
1426838_at	Pold3	polymerase (DNA-directed). delta 3. accessory subunit	-4.25	0.01	0.64	BRAF; MEK
1451266_at	Mrpl50	mitochondrial ribosomal protein L50	-4.25	0.01	0.75	
1431287_at	Pcm1	pericentriolar material 1	-4.25	0.01	0.67	; MEK
1439459_x_at	Acly	ATP citrate lyase	-4.25	0.01	0.81	BRAF; MEK
1449254_at	Spp1	secreted phosphoprotein 1	-4.25	0.01	0.27	BRAF; MEK
1454914_at	2610101N10Rik	RIKEN cDNA 2610101N10 gene	-4.25	0.01	0.71	BRAF; MEK
1424020_at	Arl6ip6	ADP-ribosylation factor-like 6 interacting protein 6	-4.25	0.01	0.67	BRAF; MEK
1452856_at	Crebzf	CREB/ATF bZIP transcription factor	-4.26	0.01	0.73	
1418330_at	Ctcf	CCCTC-binding factor	-4.26	0.01	0.68	BRAF; MEK
1460643_at	Ell	elongation factor RNA polymerase II	-4.26	0.01	0.63	BRAF; MEK
1415909_at	Stip1	stress-induced phosphoprotein 1	-4.26	0.01	0.69	BRAF; MEK
1434512_x_at	Sfrs3	splicing factor. arginine/serine-rich 3 (SRp20)	-4.26	0.01	0.69	BRAF; MEK
1438089_a_at	Bclaf1	BCL2-associated transcription factor 1	-4.26	0.01	0.56	n.r. ;
1422823_at	Eps8	epidermal growth factor receptor pathway substrate 8	-4.26	0.01	0.41	BRAF; MEK
1433474_at	Edil3	EGF-like repeats and discoidin I-like domains 3	-4.26	0.01	0.56	BRAF; MEK
1456359_at	Ppwd1	peptidylprolyl isomerase domain and WD repeat containing 1	-4.26	0.01	0.66	
1456700_x_at	Marcks	myristoylated alanine rich protein kinase C substrate	-4.26	0.01	0.59	BRAF; MEK
1442911_at	Riok2	RIO kinase 2 (yeast)	-4.27	0.01	0.62	; MEK
1454993_a_at	Sfrs3	splicing factor. arginine/serine-rich 3 (SRp20)	-4.27	0.01	0.66	BRAF; MEK
1423511_at	Asf1a	ASF1 anti-silencing function 1 homolog A (S. cerevisiae)	-4.27	0.01	0.74	BRAF; MEK
1417140_a_at	Ptpn2	protein tyrosine phosphatase. non-receptor type 2	-4.27	0.01	0.62	; MEK
1452253_at	Crim1	cysteine rich transmembrane BMP regulator 1 (chordin like)	-4.27	0.01	0.58	
1450420_at	Stag1	stromal antigen 1	-4.27	0.01	0.70	BRAF; MEK
1450744_at	Ell2	elongation factor RNA polymerase II 2	-4.27	0.01	0.68	n.r. ; n.r.
1442135_at	Ado	2-aminoethanethiol (cysteamine) dioxygenase	-4.27	0.01	0.66	BRAF; MEK
1454042_a_at	Srpk1	serine/arginine-rich protein specific kinase 1	-4.27	0.01	0.67	BRAF; MEK
1448834_at	Foxm1	forkhead box M1	-4.27	0.01	0.36	BRAF; MEK
1460667_at	U90926	cDNA sequence U90926	-4.27	0.01	0.17	BRAF; MEK
1421274_at	Socs4	suppressor of cytokine signaling 4	-4.27	0.01	0.77	
1418308_at	Hus1	Hus1 homolog (S. pombe)	-4.27	0.01	0.67	
1424572_a_at	H2afy	H2A histone family. member Y	-4.28	0.01	0.70	BRAF
1417250_at	Rnf12	ring finger protein 12	-4.28	0.01	0.70	; MEK
1424787_a_at	Nrf1	nuclear respiratory factor 1	-4.28	0.01	0.66	; n.r.
1416442_at	Ier2	immediate early response 2	-4.28	0.01	0.55	BRAF
1433482_a_at	Fubp1	far upstream element (FUSE) binding protein 1	-4.28	0.01	0.53	BRAF; MEK
1431182_at	Hspa8	heat shock protein 8	-4.28	0.01	0.52	BRAF; MEK
1422613_a_at	Rpl7a	ribosomal protein L7a	-4.28	0.01	0.91	BRAF
1443171_at	NA	NA	-4.28	0.01	0.61	; MEK
1448774_at	Stoml2	stomatin (Epb7.2)-like 2	-4.28	0.01	0.56	BRAF; MEK
1448129_at	Arpc5	actin related protein 2/3 complex. subunit 5	-4.28	0.01	0.74	n.r. ; n.r.
1418017_at	Pum2	pumilio 2 (Drosophila)	-4.28	0.01	0.72	; MEK
1419613_at	Col7a1	collagen. type VII. alpha 1	-4.29	0.01	0.41	BRAF
1423186_at	Tiam2	T-cell lymphoma invasion and metastasis 2	-4.29	0.01	0.47	BRAF; MEK
1427253_s_at	Suz12	suppressor of zeste 12 homolog (Drosophila)	-4.29	0.01	0.55	BRAF; MEK
1419690_at	2610002M06Rik	RIKEN cDNA 2610002M06 gene	-4.29	0.01	0.79	BRAF
1416918_at	Dlg3	discs. large homolog 3 (Drosophila)	-4.29	0.01	0.64	BRAF; MEK
1427260_a_at	Tpm3	tropomyosin 3. gamma	-4.29	0.01	0.82	; MEK
1437626_at	Zfp36l2	zinc finger protein 36. C3H type-like 2	-4.29	0.01	0.60	BRAF; MEK
1448703_at	Lsm8	LSM8 homolog. U6 small nuclear RNA associated (S. cerevisiae)	-4.29	0.01	0.60	BRAF; MEK
1426931_s_at	D19Bwg1357e	DNA segment. Chr 19. Brigham & Women's Genetics 1357 expressed	-4.29	0.01	0.62	BRAF
1431226_a_at	Fndc4	fibronectin type III domain containing 4	-4.29	0.01	0.39	BRAF; MEK
1418372_at	Adsl	adenylosuccinate lyase	-4.29	0.01	0.63	BRAF; MEK
1428210_s_at	Chuk	conserved helix-loop-helix ubiquitous kinase	-4.29	0.01	0.69	

Probeset ID	Symbol	Gene Name	d-value	q-value	R-fold	Expression reversed by
1415785_a_at	Cct8	chaperonin containing Tcp1. subunit 8 (theta)	-4.29	0.01	0.72	; n.r.
1423050_s_at	Hnrnpu	heterogeneous nuclear ribonucleoprotein U	-4.30	0.01	0.75	BRAF
1460544_at	Mak10	MAK10 homolog. amino-acid N-acetyltransferase subunit. (S. cerevisiae)	-4.30	0.01	0.75	BRAF
1455732_at	2610510E02Rik	RIKEN cDNA 2610510E02 gene	-4.30	0.01	0.53	BRAF; MEK
1460062_at	Plekhh2	pleckstrin homology domain containing. family H (with MyTH4 domain) member 2	-4.30	0.01	0.62	; MEK
1429942_at	Gmeb1	glucocorticoid modulatory element binding protein 1	-4.30	0.01	0.73	BRAF; MEK
1433998_at	4933427D14Rik	RIKEN cDNA 4933427D14 gene	-4.31	0.01	0.69	BRAF; MEK
1425617_at	Dhx9	DEAH (Asp-Glu-Ala-His) box polypeptide 9	-4.31	0.01	0.45	BRAF; MEK
1437244_at	Gas2l3	growth arrest-specific 2 like 3	-4.31	0.01	0.42	BRAF; MEK
1419522_at	Zmynd19	zinc finger. MYND domain containing 19	-4.31	0.01	0.68	
1415917_at	Mthfd1	methylenetetrahydrofolate dehydrogenase (NADP+ dependent). methenyltetrahydrofolate cyclohydrolase. formyltetrahydrofolate synthase	-4.31	0.01	0.57	BRAF; MEK
1434525_at	Pkn3	protein kinase N3	-4.31	0.01	0.74	BRAF; MEK
1430286_s_at	LOC100043766	Ppp1r14c pseudogene	-4.32	0.01	0.67	; MEK
1451244_a_at	Zfp422	zinc finger protein 422	-4.32	0.01	0.62	BRAF; MEK
1418822_a_at	Arf6	ADP-ribosylation factor 6	-4.32	0.01	0.75	BRAF; MEK
1457118_at	Eid1	EP300 interacting inhibitor of differentiation 1	-4.32	0.01	0.71	BRAF; MEK
1426462_at	Gphn	gephyrin	-4.32	0.01	0.68	BRAF; MEK
1435390_at	Exod1	exonuclease domain containing 1	-4.32	0.01	0.51	BRAF; MEK
1427165_at	Il13ra1	interleukin 13 receptor. alpha 1	-4.32	0.01	0.35	; MEK
1454750_a_at	BC057552	cDNA sequence BC057552	-4.33	0.01	0.63	BRAF; MEK
1425733_a_at	Eps8	epidermal growth factor receptor pathway substrate 8	-4.33	0.01	0.52	BRAF; MEK
1422955_at	Syt17	synaptotagmin XVII	-4.33	0.01	0.62	
1439010_at	Larp4	La ribonucleoprotein domain family. member 4	-4.33	0.01	0.68	; MEK
1434899_s_at	Tnrc6a	trinucleotide repeat containing 6a	-4.33	0.01	0.74	n.r. ; n.r.
1453465_x_at	LOC100043766	Ppp1r14c pseudogene	-4.33	0.01	0.58	BRAF
1451483_s_at	1700054N08Rik	RIKEN cDNA 1700054N08 gene	-4.33	0.01	0.70	BRAF; MEK
1422632_at	Ctsw	cathepsin W	-4.33	0.01	0.56	; n.r.
1456566_x_at	Rbm14	RNA binding motif protein 14	-4.34	0.01	0.59	BRAF; MEK
1435768_at	Arid4b	AT rich interactive domain 4B (RBP1-like)	-4.34	0.01	0.64	
1427064_a_at	Scrib	scribbled homolog (Drosophila)	-4.34	0.01	0.61	BRAF; MEK
1423773_at	Gpbp1	GC-rich promoter binding protein 1	-4.34	0.01	0.70	; MEK
1433898_at	NA	NA	-4.34	0.01	0.62	BRAF; MEK
1455980_a_at	Gas2l3	growth arrest-specific 2 like 3	-4.34	0.01	0.36	BRAF; MEK
1451356_at	Anp32e	acidic (leucine-rich) nuclear phosphoprotein 32 family. member E	-4.35	0.01	0.67	BRAF; MEK
1424620_at	D13Wsu177e	DNA segment. Chr 13. Wayne State University 177. expressed	-4.35	0.01	0.67	BRAF; n.r.
1426837_at	Metap1	methionyl aminopeptidase 1	-4.35	0.01	0.71	
1453769_at	Ckap2l	cytoskeleton associated protein 2-like	-4.35	0.01	0.47	BRAF
1460037_at	2610510H03Rik	RIKEN cDNA 2610510H03 gene	-4.35	0.01	0.73	BRAF; MEK
1435139_at	Narg1	NMDA receptor-regulated gene 1	-4.35	0.01	0.70	; MEK
1416415_a_at	H2afz	H2A histone family. member Z	-4.35	0.01	0.63	BRAF; MEK
1455609_at	Cit	citron	-4.35	0.01	0.40	BRAF; MEK
1422922_at	Recql4	RecQ protein-like 4	-4.35	0.01	0.63	BRAF; MEK
1435826_at	Rad18	RAD18 homolog (S. cerevisiae)	-4.35	0.01	0.41	BRAF; MEK
1451039_at	2610027L16Rik	RIKEN cDNA 2610027L16 gene	-4.35	0.01	0.68	; n.r.
1449633_s_at	Nt5c3l	5'-nucleotidase. cytosolic III-like	-4.36	0.01	0.60	BRAF; MEK
1425543_s_at	Plekha5	pleckstrin homology domain containing. family A member 5	-4.36	0.01	0.46	BRAF; MEK
1449343_s_at	Sin3a	transcriptional regulator. SIN3A (yeast)	-4.36	0.01	0.65	BRAF; MEK
1418022_at	Narg1	NMDA receptor-regulated gene 1	-4.36	0.01	0.65	; MEK
1424322_at	Apex2	apurinic/apyrimidinic endonuclease 2	-4.36	0.01	0.62	
1453416_at	Gas2l3	growth arrest-specific 2 like 3	-4.36	0.01	0.41	BRAF; MEK
1429476_s_at	Dnaja2	DnaJ (Hsp40) homolog. subfamily A. member 2	-4.36	0.01	0.84	BRAF
1416345_at	Timm8a1	translocase of inner mitochondrial membrane 8 homolog a1 (yeast)	-4.36	0.01	0.58	BRAF; MEK
1439421_x_at	Cbx3	chromobox homolog 3 (Drosophila HP1 gamma)	-4.37	0.01	0.59	BRAF; MEK

Probeset ID	Symbol	Gene Name	d-value	q-value	R-fold	Expression reversed by
1416585_at	Ruvbl1	RuvB-like protein 1	-4.37	0.01	0.62	BRAF; MEK
1460220_a_at	Csf1	colony stimulating factor 1 (macrophage)	-4.37	0.01	0.66	n.r. ;
1434650_at	Pogz	pogo transposable element with ZNF domain	-4.37	0.01	0.67	BRAF; MEK
1457292_at	Josd3	Josephin domain containing 3	-4.37	0.01	0.31	; MEK
1452691_at	Rbm17	RNA binding motif protein 17	-4.37	0.01	0.68	; MEK
1442753_at	Tnfaip8	tumor necrosis factor. alpha-induced protein 8	-4.37	0.01	0.72	BRAF; MEK
1448531_at	Lmnb2	lamin B2	-4.37	0.01	0.62	BRAF; MEK
1419260_a_at	Snrpb	small nuclear ribonucleoprotein B	-4.37	0.01	0.75	BRAF; MEK
1451358_a_at	Racgap1	Rac GTPase-activating protein 1	-4.37	0.01	0.33	BRAF; MEK
1457708_at	Mbd4	methyl-CpG binding domain protein 4	-4.38	0.01	0.65	BRAF; MEK
1428546_at	Syncrip	synaptotagmin binding. cytoplasmic RNA interacting protein	-4.38	0.01	0.58	BRAF; MEK
1417697_at	Soat1	sterol O-acyltransferase 1	-4.38	0.01	0.55	
1423177_a_at	Abi1	abl-interactor 1	-4.38	0.01	0.78	n.r. ; n.r.
1436543_at	Gtpbp10	GTP-binding protein 10 (putative)	-4.38	0.01	0.55	
1452681_at	Dtymk	deoxythymidylate kinase	-4.38	0.01	0.63	BRAF; MEK
1423969_at	Nup37	nucleoporin 37	-4.38	0.01	0.54	BRAF; MEK
1438601_at	Pkmyt1	protein kinase. membrane associated tyrosine/threonine 1	-4.39	0.01	0.86	BRAF
1449210_at	Igf2bp1	insulin-like growth factor 2 mRNA binding protein 1	-4.39	0.01	0.64	BRAF; MEK
1437278_a_at	Uba2	ubiquitin-like modifier activating enzyme 2	-4.39	0.01	0.66	BRAF; MEK
1426682_at	Cnot6	CCR4-NOT transcription complex. subunit 6	-4.39	0.01	0.65	BRAF; MEK
1421323_a_at	G3bp2	GTPase activating protein (SH3 domain) binding protein 2	-4.39	0.01	0.63	BRAF; MEK
1415817_s_at	Cct7	chaperonin containing Tcp1. subunit 7 (eta)	-4.39	0.01	0.69	BRAF
1432022_at	Cdgap	Cdc42 GTPase-activating protein	-4.39	0.01	0.81	
1455260_at	Lcorl	ligand dependent nuclear receptor corepressor-like	-4.39	0.01	0.68	n.r. ;
1416815_s_at	Bub3	budding uninhibited by benzimidazoles 3 homolog (S. cerevisiae)	-4.40	0.01	0.74	BRAF
1428970_at	Nat13	N-acetyltransferase 13	-4.40	0.01	0.62	; n.r.
1418036_at	Prim2	DNA primase. p58 subunit	-4.40	0.01	0.53	BRAF; MEK
1437280_s_at	Serbp1	Serpine1 mRNA binding protein 1	-4.40	0.01	0.72	BRAF; MEK
1446272_at	Pctk2	PCTAIRE-motif protein kinase 2	-4.40	0.01	0.71	
1454737_at	Dusp9	dual specificity phosphatase 9	-4.40	0.01	0.67	; MEK
1426783_at	Kat2a	K(lysine) acetyltransferase 2A	-4.40	0.01	0.68	BRAF; MEK
1424753_at	Nudt14	nudix (nucleoside diphosphate linked moiety X)-type motif 14	-4.40	0.01	0.77	BRAF
1431349_at	Hnrnpab	heterogeneous nuclear ribonucleoprotein A/B	-4.40	0.01	0.64	
1423234_at	Psmd5	proteasome (prosome. macropain) 26S subunit. non-ATPase. 5	-4.40	0.01	0.73	
1440882_at	Lrp8	low density lipoprotein receptor-related protein 8. apolipoprotein e receptor	-4.41	0.01	0.63	; MEK
1429490_at	Rif1	Rap1 interacting factor 1 homolog (yeast)	-4.41	0.01	0.43	BRAF; MEK
1445866_at	Mast4	microtubule associated serine/threonine kinase family member 4	-4.41	0.01	0.32	BRAF; MEK
1421237_at	Tmpo	thymopoietin	-4.41	0.01	0.50	BRAF
1419357_at	Isy1	ISY1 splicing factor homolog (S. cerevisiae)	-4.41	0.01	0.62	BRAF; MEK
1417742_a_at	Dmap1	DNA methyltransferase 1-associated protein 1	-4.41	0.01	0.83	BRAF; MEK
1427397_at	2810046L04Rik	RIKEN cDNA 2810046L04 gene	-4.41	0.01	0.67	; MEK
1440650_at	NA	NA	-4.41	0.01	0.79	
1452760_at	2510012J08Rik	RIKEN cDNA 2510012J08 gene	-4.41	0.01	0.74	BRAF
1424895_at	Gpsm2	G-protein signalling modulator 2 (AGS3-like. C. elegans)	-4.41	0.01	0.52	BRAF; MEK
1419102_at	Sin3a	transcriptional regulator. SIN3A (yeast)	-4.42	0.01	0.57	BRAF; MEK
1435415_x_at	Marcksl1	MARCKS-like 1	-4.42	0.01	0.58	BRAF; MEK
1433569_x_at	LOC100045999	similar to RAN. member RAS oncogene family	-4.42	0.01	0.80	BRAF; MEK
1443196_at	NA	NA	-4.42	0.01	0.70	; n.r.
1418872_at	Abcb1b	ATP-binding cassette. sub-family B (MDR/TAP). member 1B	-4.42	0.01	0.42	BRAF; MEK
1431028_a_at	Pank1	pantothenate kinase 1	-4.42	0.01	0.66	BRAF; MEK
1437632_at	Med14	mediator complex subunit 14	-4.42	0.01	0.61	BRAF; MEK
1449293_a_at	Skp2	S-phase kinase-associated protein 2 (p45)	-4.42	0.01	0.44	BRAF
1438545_at	Slc25a5	solute carrier family 25 (mitochondrial carrier. adenine nucleotide translocator). member 5	-4.42	0.01	0.73	BRAF
1449176_a_at	Dck	deoxycytidine kinase	-4.42	0.01	0.46	BRAF; MEK
1448896_at	Pigf	phosphatidylinositol glycan anchor biosynthesis. class F	-4.43	0.01	0.58	BRAF; MEK

Probeset ID	Symbol	Gene Name	d-value	q-value	R-fold	Expression reversed by
1449012_s_at	Fndc4	fibronectin type III domain containing 4	-4.43	0.01	0.47	BRAF; MEK
1448484_at	Amd1	S-adenosylmethionine decarboxylase 1	-4.43	0.01	0.69	BRAF
1431233_at	Cnnm4	cyclin M4	-4.43	0.01	0.56	BRAF
1460391_at	Ola1	Obg-like ATPase 1	-4.43	0.01	0.77	BRAF; MEK
1417364_at	Eef1g	eukaryotic translation elongation factor 1 gamma	-4.43	0.01	0.88	BRAF; MEK
1417258_at	Cct5	chaperonin containing Tcp1. subunit 5 (epsilon)	-4.43	0.01	0.76	BRAF; MEK
1428727_at	Cep192	centrosomal protein 192	-4.43	0.01	0.50	BRAF; MEK
1417662_at	Elk3	ELK3. member of ETS oncogene family	-4.43	0.01	0.59	; n.r.
1429383_at	Csnk1g3	casein kinase 1. gamma 3	-4.43	0.01	0.71	; MEK
1419493_a_at	Tpd52	tumor protein D52	-4.43	0.01	0.56	
1434608_at	Ddx52	DEAD (Asp-Glu-Ala-Asp) box polypeptide 52	-4.44	0.01	0.77	
1451884_a_at	Lsm2	LSM2 homolog. U6 small nuclear RNA associated (S. cerevisiae)	-4.44	0.01	0.49	BRAF; MEK
1437378_x_at	Scarb1	scavenger receptor class B. member 1	-4.44	0.01	0.54	BRAF; MEK
1448016_at	Sass6	spindle assembly 6 homolog (C. elegans)	-4.44	0.01	0.54	BRAF; MEK
1418629_a_at	Khdrbs1	KH domain containing. RNA binding. signal transduction associated 1	-4.45	0.01	0.63	BRAF; MEK
1437395_at	Zcchc11	zinc finger. CCHC domain containing 11	-4.45	0.01	0.50	BRAF; MEK
1451391_at	2700050L05Rik	RIKEN cDNA 2700050L05 gene	-4.45	0.01	0.82	; MEK
1455330_at	Nol9	nucleolar protein 9	-4.45	0.01	0.66	BRAF; MEK
1455760_at	Slc9a5	solute carrier family 9 (sodium/hydrogen exchanger). member 5	-4.45	0.01	0.53	BRAF; MEK
1437193_s_at	Snrpb	small nuclear ribonucleoprotein B	-4.45	0.01	0.75	BRAF; MEK
1448151_at	Elavl1	ELAV (embryonic lethal. abnormal vision. Drosophila)-like 1 (Hu antigen R)	-4.45	0.01	0.66	BRAF; MEK
1421141_a_at	Foxp1	forkhead box P1	-4.45	0.01	0.60	; n.r.
1417962_s_at	Ghr	growth hormone receptor	-4.45	0.01	0.59	BRAF; MEK
1420368_at	Denr	density-regulated protein	-4.45	0.01	0.59	; MEK
1437226_x_at	Marcksl1	MARCKS-like 1	-4.46	0.01	0.43	BRAF; MEK
1460378_a_at	Tes	testis derived transcript	-4.46	0.01	0.35	BRAF; MEK
1431166_at	Chd1	chromodomain helicase DNA binding protein 1	-4.46	0.01	0.50	BRAF; MEK
1452047_at	Cacybp	calcyclin binding protein	-4.46	0.01	0.76	
1423303_at	Paxip1	PAX interacting (with transcription-activation domain) protein 1	-4.46	0.01	0.58	BRAF; MEK
1449323_a_at	Rpl3	ribosomal protein L3	-4.46	0.01	0.84	BRAF; MEK
1438096_a_at	Dtymk	deoxythymidylate kinase	-4.46	0.01	0.59	BRAF; MEK
1428146_s_at	Acaa2	acetyl-Coenzyme A acyltransferase 2 (mitochondrial 3-oxoacyl-Coenzyme A thiolase)	-4.46	0.01	0.64	BRAF; MEK
1450414_at	Pdgfb	platelet derived growth factor. B polypeptide	-4.47	0.01	0.33	n.r. ; MEK
1438951_x_at	Nup54	nucleoporin 54	-4.47	0.01	0.73	BRAF; MEK
1428834_at	Dusp4	dual specificity phosphatase 4	-4.47	0.01	0.41	BRAF
1417403_at	Elovl6	ELOVL family member 6. elongation of long chain fatty acids (yeast)	-4.47	0.01	0.50	; MEK
1429002_at	Snw1	SNW domain containing 1	-4.47	0.01	0.79	BRAF; MEK
1421546_a_at	Racgap1	Rac GTPase-activating protein 1	-4.47	0.01	0.33	BRAF; MEK
1460573_at	AI848100	expressed sequence AI848100	-4.47	0.01	0.72	n.r. ; MEK
1419543_a_at	Sfrs10	splicing factor. arginine/serine-rich 10 (transformer 2 homolog. Drosophila)	-4.48	0.01	0.60	
1428224_at	Hnrpdl	heterogeneous nuclear ribonucleoprotein D-like	-4.48	0.01	0.57	BRAF; MEK
1427441_a_at	Suclg2	succinate-Coenzyme A ligase. GDP-forming. beta subunit	-4.48	0.01	0.58	BRAF; MEK
1416439_at	2410015N17Rik	RIKEN cDNA 2410015N17 gene	-4.48	0.01	0.54	BRAF; MEK
1418225_at	Orc2l	origin recognition complex. subunit 2-like (S. cerevisiae)	-4.48	0.01	0.72	BRAF; MEK
1435737_a_at	Nde1	nuclear distribution gene E homolog 1 (A nidulans)	-4.48	0.01	0.75	BRAF; MEK
1436813_x_at	Khsrp	KH-type splicing regulatory protein	-4.48	0.01	0.72	BRAF; MEK
1423827_s_at	Noc4l	nucleolar complex associated 4 homolog (S. cerevisiae)	-4.48	0.01	0.63	BRAF; MEK
1436816_at	Nup133	nucleoporin 133	-4.49	0.01	0.56	BRAF; MEK
1430536_a_at	Erh	enhancer of rudimentary homolog (Drosophila)	-4.49	0.01	0.69	BRAF; MEK
1434876_at	Glt8d3	glycosyltransferase 8 domain containing 3	-4.49	0.01	0.69	; MEK
1429538_a_at	Sfrs18	splicing factor. arginine/serine-rich 18	-4.49	0.01	0.55	; MEK
1434278_at	Mtm1	X-linked myotubular myopathy gene 1	-4.49	0.01	0.56	BRAF; MEK
1458130_at	NA	NA	-4.49	0.01	0.57	; MEK
1437130_at	EG432879	predicted gene. EG432879	-4.49	0.01	0.80	BRAF; MEK

Probeset ID	*Symbol*	*Gene Name*	*d-value*	*q-value*	*R-fold*	*Expression reversed by*
1416933_at	Por	P450 (cytochrome) oxidoreductase	-4.49	0.01	0.67	BRAF; MEK
AFFX-TransRecMur/X57349_3_at	Tfrc	transferrin receptor	-4.49	0.01	0.30	; n.r.
1450948_a_at	Mrpl1	mitochondrial ribosomal protein L1	-4.49	0.01	0.73	; MEK
1435349_at	Nrp2	neuropilin 2	-4.49	0.01	0.25	BRAF; MEK
1434938_at	Rbm9	RNA binding motif protein 9	-4.49	0.01	0.68	BRAF; MEK
1435474_at	Taf5	TAF5 RNA polymerase II. TATA box binding protein (TBP)-associated factor	-4.50	0.01	0.61	BRAF; MEK
1449056_at	E330009J07Rik	RIKEN cDNA E330009J07 gene	-4.50	0.01	0.48	BRAF; MEK
1423479_at	Nol11	nucleolar protein 11	-4.50	0.01	0.61	BRAF
1446226_at	Fancb	Fanconi anemia. complementation group B	-4.50	0.01	0.72	BRAF
1420609_at	07-mar	membrane-associated ring finger (C3HC4) 7	-4.50	0.01	0.71	n.r. ; MEK
1420441_at	Cenpc1	centromere protein C1	-4.50	0.01	0.62	BRAF; MEK
1428691_at	Chd2	chromodomain helicase DNA binding protein 2	-4.50	0.01	0.79	BRAF; MEK
1450738_at	Kif21a	kinesin family member 21A	-4.51	0.01	0.62	BRAF; MEK
1456865_x_at	Rrs1	RRS1 ribosome biogenesis regulator homolog (S. cerevisiae)	-4.51	0.01	0.57	BRAF; MEK
1455102_at	Larp4	La ribonucleoprotein domain family. member 4	-4.51	0.01	0.62	n.r. ;
1436475_at	Nr2f2	nuclear receptor subfamily 2. group F. member 2	-4.51	0.01	0.65	
1423761_at	5630401D24Rik	RIKEN cDNA 5630401D24 gene	-4.51	0.01	0.69	
1455562_at	Sox12	SRY-box containing gene 12	-4.52	0.01	0.83	
1452124_at	Ank3	ankyrin 3. epithelial	-4.52	0.01	0.41	BRAF; MEK
1454094_at	4930471E19Rik	RIKEN cDNA 4930471E19 gene	-4.52	0.01	0.63	; MEK
1427413_a_at	Cugbp1	CUG triplet repeat. RNA binding protein 1	-4.52	0.01	0.66	
1415682_at	Xpo7	exportin 7	-4.52	0.01	0.74	; MEK
1433622_at	Gemin4	gem (nuclear organelle) associated protein 4	-4.52	0.01	0.65	BRAF; MEK
1423809_at	Tcf19	transcription factor 19	-4.52	0.01	0.40	BRAF; MEK
1421751_a_at	Psmd14	proteasome (prosome. macropain) 26S subunit. non-ATPase. 14	-4.52	0.01	0.82	; MEK
1452337_at	4930427A07Rik	RIKEN cDNA 4930427A07 gene	-4.53	0.01	0.60	BRAF; MEK
1423617_at	Pdf	peptide deformylase (mitochondrial)	-4.53	0.01	0.74	BRAF; MEK
1447924_at	Nucks1	nuclear casein kinase and cyclin-dependent kinase substrate 1	-4.53	0.01	0.57	
1452454_at	Sdad1	SDA1 domain containing 1	-4.53	0.01	0.60	BRAF; MEK
1433585_at	Tnpo1	transportin 1	-4.53	0.01	0.68	BRAF
1452180_at	Phf17	PHD finger protein 17	-4.53	0.01	0.46	
1418564_s_at	Serbp1	Serpine1 mRNA binding protein 1	-4.53	0.01	0.54	BRAF; MEK
1453187_at	Ociad2	OCIA domain containing 2	-4.53	0.01	0.54	; MEK
1436069_at	Ing5	inhibitor of growth family. member 5	-4.53	0.01	0.69	BRAF; MEK
1417406_at	Sertad1	SERTA domain containing 1	-4.54	0.01	0.71	
1417864_at	Pgk1	phosphoglycerate kinase 1	-4.54	0.01	0.80	; MEK
1428754_at	Trmt6	tRNA methyltransferase 6 homolog (S. cerevisiae)	-4.54	0.01	0.74	BRAF; MEK
1427496_at	Cep152	centrosomal protein 152	-4.54	0.01	0.66	BRAF; MEK
1447749_at	Smyd5	SET and MYND domain containing 5	-4.54	0.01	0.80	BRAF; MEK
1449333_at	Sf3a1	splicing factor 3a. subunit 1	-4.54	0.01	0.64	BRAF; MEK
1452869_at	Prpf38b	PRP38 pre-mRNA processing factor 38 (yeast) domain containing B	-4.55	0.01	0.62	; MEK
1417353_x_at	Snrpa1	small nuclear ribonucleoprotein polypeptide A'	-4.55	0.01	0.49	BRAF; MEK
1421344_a_at	Jub	ajuba	-4.55	0.01	0.71	; n.r.
1455434_a_at	Ktn1	kinectin 1	-4.55	0.01	0.70	n.r. ;
1421142_s_at	Foxp1	forkhead box P1	-4.55	0.01	0.58	BRAF; MEK
1455133_s_at	AI848100	expressed sequence AI848100	-4.55	0.01	0.71	n.r. ; MEK
1453050_at	Gins3	GINS complex subunit 3 (Psf3 homolog)	-4.55	0.01	0.66	BRAF
1438902_a_at	Hsp90aa1	heat shock protein 90. alpha (cytosolic). class A member 1	-4.55	0.01	0.82	n.r. ; MEK
1425166_at	Rbl1	retinoblastoma-like 1 (p107)	-4.55	0.01	0.43	BRAF; MEK
1433565_at	Prpf38a	PRP38 pre-mRNA processing factor 38 (yeast) domain containing A	-4.55	0.01	0.73	BRAF; MEK
1424033_at	Sfrs7	splicing factor. arginine/serine-rich 7	-4.55	0.01	0.47	BRAF; MEK
1452713_a_at	Wdr57	WD repeat domain 57 (U5 snRNP specific)	-4.56	0.01	0.52	BRAF; MEK
1454806_at	D12Ertd553e	DNA segment. Chr 12. ERATO Doi 553. expressed	-4.56	0.01	0.50	; n.r.
1434125_at	Utp15	UTP15. U3 small nucleolar ribonucleoprotein. homolog (yeast)	-4.56	0.01	0.63	BRAF; MEK

Probeset ID	*Symbol*	*Gene Name*	*d-value*	*q-value*	*R-fold*	*Expression reversed by*
1426653_at	Mcm3	minichromosome maintenance deficient 3 (S. cerevisiae)	-4.56	0.01	0.31	BRAF
1438139_at	Arhgap28	Rho GTPase activating protein 28	-4.56	0.01	0.63	BRAF
1455735_at	Ap1s3	adaptor-related protein complex AP-1. sigma 3	-4.56	0.01	0.36	BRAF; MEK
1446399_at	Cdh10	cadherin 10	-4.56	0.01	0.62	; n.r.
1452179_at	Phf17	PHD finger protein 17	-4.57	0.01	0.39	; MEK
1448195_at	Taf5l	TAF5-like RNA polymerase II. p300/CBP-associated factor (PCAF)-associated factor	-4.57	0.01	0.65	BRAF; MEK
1435057_x_at	Polr1e	polymerase (RNA) I polypeptide E	-4.57	0.01	0.64	BRAF; MEK
1418004_a_at	Tmem176b	transmembrane protein 176B	-4.57	0.01	0.23	; MEK
1426392_a_at	Actr3	ARP3 actin-related protein 3 homolog (yeast)	-4.57	0.01	0.79	
1434285_at	Frmd4a	FERM domain containing 4A	-4.57	0.01	0.52	BRAF; MEK
1439562_at	F730047E07Rik	RIKEN cDNA F730047E07 gene	-4.57	0.01	0.48	BRAF; MEK
1433954_at	4632419I22Rik	RIKEN cDNA 4632419I22 gene	-4.57	0.01	0.59	BRAF; MEK
1437492_at	Mkx	mohawk homeobox	-4.57	0.01	0.53	; MEK
1452737_at	2810008M24Rik	RIKEN cDNA 2810008M24 gene	-4.57	0.01	0.75	n.r. ; n.r.
1454952_s_at	Ncapd3	non-SMC condensin II complex. subunit D3	-4.57	0.01	0.38	BRAF; MEK
1442939_at	Rif1	Rap1 interacting factor 1 homolog (yeast)	-4.57	0.01	0.56	; MEK
1452676_a_at	Pnpt1	polyribonucleotide nucleotidyltransferase 1	-4.58	0.01	0.63	; MEK
1424692_at	2810055F11Rik	RIKEN cDNA 2810055F11 gene	-4.58	0.01	0.55	BRAF; MEK
1439597_at	NA	NA	-4.58	0.01	0.81	
1433647_s_at	Rhobtb3	Rho-related BTB domain containing 3	-4.58	0.01	0.58	BRAF; MEK
1429056_at	Narg1l	NMDA receptor regulated 1-like	-4.58	0.01	0.56	; MEK
1448324_at	Rnps1	ribonucleic acid binding protein S1	-4.58	0.01	0.62	BRAF; MEK
1417656_at	Mybl2	myeloblastosis oncogene-like 2	-4.58	0.01	0.36	BRAF; MEK
1435728_at	Tyw3	tRNA-yW synthesizing protein 3 homolog (S. cerevisiae)	-4.59	0.01	0.70	BRAF; MEK
1425022_at	Usp3	ubiquitin specific peptidase 3	-4.59	0.01	0.73	BRAF; MEK
1453255_at	Slc43a1	solute carrier family 43. member 1	-4.59	0.01	0.58	; n.r.
1454612_at	Mex3c	mex3 homolog C (C. elegans)	-4.59	0.01	0.70	; MEK
1416484_at	Ttc3	tetratricopeptide repeat domain 3	-4.59	0.01	0.51	; MEK
1456144_at	Nav3	neuron navigator 3	-4.59	0.01	0.62	BRAF; MEK
1433530_at	Rpl41	ribosomal protein L41	-4.59	0.01	0.54	
1435555_at	NA	NA	-4.59	0.01	0.70	BRAF; MEK
1428388_at	Tnks2	tankyrase. TRF1-interacting ankyrin-related ADP-ribose polymerase 2	-4.59	0.01	0.67	BRAF; MEK
1428390_at	Wdr43	WD repeat domain 43	-4.59	0.01	0.59	BRAF; MEK
1460227_at	Timp1	tissue inhibitor of metalloproteinase 1	-4.59	0.01	0.45	; MEK
1427887_at	Rprd1b	regulation of nuclear pre-mRNA domain containing 1B	-4.60	0.01	0.78	BRAF; MEK
1415829_at	Lbr	lamin B receptor	-4.60	0.01	0.50	BRAF; MEK
1422675_at	Smarce1	SWI/SNF related. matrix associated. actin dependent regulator of chromatin. subfamily e. member 1	-4.60	0.01	0.61	BRAF; MEK
1453849_s_at	Hnrnpab	heterogeneous nuclear ribonucleoprotein A/B	-4.60	0.01	0.60	BRAF; MEK
1452784_at	Itgav	integrin alpha V	-4.60	0.01	0.66	
1432202_a_at	Wdr51a	WD repeat domain 51A	-4.60	0.01	0.62	BRAF; MEK
1436120_at	Setdb2	SET domain. bifurcated 2	-4.60	0.01	0.69	
1434047_x_at	Hnrnpa2b1	heterogeneous nuclear ribonucleoprotein A2/B1	-4.60	0.01	0.65	
1424207_at	Smarca5	SWI/SNF related. matrix associated. actin dependent regulator of chromatin. subfamily a. member 5	-4.60	0.01	0.49	
1416748_a_at	Mre11a	meiotic recombination 11 homolog A (S. cerevisiae)	-4.60	0.01	0.45	BRAF; MEK
1436738_at	Pif1	PIF1 5'-to-3' DNA helicase homolog (S. cerevisiae)	-4.60	0.01	0.49	BRAF; MEK
1422653_at	Cep70	centrosomal protein 70	-4.61	0.01	0.59	; MEK
1416070_a_at	Ddx18	DEAD (Asp-Glu-Ala-Asp) box polypeptide 18	-4.61	0.01	0.71	BRAF; MEK
1423025_a_at	Schip1	schwannomin interacting protein 1	-4.61	0.01	0.64	
1452371_at	Sfrs11	splicing factor. arginine/serine-rich 11	-4.61	0.01	0.59	BRAF; MEK
1444235_at	1700025G04Rik	RIKEN cDNA 1700025G04 gene	-4.61	0.01	0.67	BRAF; MEK
1433893_s_at	Spag5	sperm associated antigen 5	-4.61	0.01	0.33	BRAF; MEK
1456163_at	2700049P18Rik	RIKEN cDNA 2700049P18 gene	-4.61	0.01	0.67	BRAF; MEK
1434296_at	BC049349	cDNA sequence BC049349	-4.61	0.01	0.56	BRAF; MEK
1425362_at	Hrbl	HIV-1 Rev binding protein-like	-4.61	0.01	0.76	; MEK

Probeset ID	*Symbol*	*Gene Name*	*d-value*	*q-value*	*R-fold*	*Expression reversed by*
1423400_at	Kl	klotho	-4.61	0.01	0.86	BRAF
1450701_a_at	Gtf2h2	general transcription factor II H. polypeptide 2	-4.62	0.01	0.77	BRAF
1435891_x_at	NA	NA	-4.62	0.01	0.59	
1434020_at	Pdap1	PDGFA associated protein 1	-4.62	0.01	0.58	BRAF; MEK
1419101_at	Sin3a	transcriptional regulator. SIN3A (yeast)	-4.62	0.01	0.64	BRAF; MEK
1423524_at	Mastl	microtubule associated serine/threonine kinase-like	-4.62	0.01	0.35	BRAF; MEK
1422693_a_at	Sub1	SUB1 homolog (S. cerevisiae)	-4.62	0.01	0.72	; MEK
1439012_a_at	Dck	deoxycytidine kinase	-4.62	0.01	0.37	BRAF; MEK
1452731_x_at	ENSMUSG0000 0068790	predicted gene. ENSMUSG00000068790	-4.62	0.01	0.44	BRAF
1424705_at	Rbmx2	RNA binding motif protein. X-linked 2	-4.63	0.01	0.52	BRAF; MEK
1430983_at	Pdia6	protein disulfide isomerase associated 6	-4.63	0.01	0.80	; MEK
1453727_at	Esf1	ESF1. nucleolar pre-rRNA processing protein. homolog (S. cerevisiae)	-4.63	0.01	0.45	n.r. ;
1416941_s_at	Eif4h	eukaryotic translation initiation factor 4H	-4.63	0.01	0.85	BRAF; MEK
1416268_at	Ets2	E26 avian leukemia oncogene 2. 3' domain	-4.63	0.01	0.52	; MEK
1439027_at	C330023M02Rik	RIKEN cDNA C330023M02 gene	-4.63	0.01	0.61	BRAF; MEK
1449675_at	Ccnb1	cyclin B1	-4.63	0.01	0.51	BRAF
1419417_at	Vegfc	vascular endothelial growth factor C	-4.63	0.01	0.34	BRAF; MEK
1417829_a_at	Rab15	RAB15. member RAS oncogene family	-4.63	0.01	0.79	; MEK
1418856_a_at	Fanca	Fanconi anemia. complementation group A	-4.64	0.01	0.53	BRAF; MEK
1434322_at	Micall2	MICAL-like 2	-4.64	0.01	0.50	BRAF; MEK
1455678_at	Sema4b	sema domain. immunoglobulin domain (Ig). transmembrane domain (TM) and short cytoplasmic domain. (semaphorin) 4B	-4.64	0.01	0.72	BRAF; MEK
1436460_at	Tmem194	transmembrane protein 194	-4.64	0.01	0.56	BRAF; MEK
1416773_at	Wee1	WEE 1 homolog (S. pombe)	-4.64	0.01	0.53	BRAF; MEK
1423071_x_at	6720475J19Rik	RIKEN cDNA 6720475J19 gene	-4.64	0.01	0.51	
1426236_a_at	Glul	glutamate-ammonia ligase (glutamine synthetase)	-4.64	0.01	0.53	BRAF; MEK
1428389_s_at	Wdr43	WD repeat domain 43	-4.64	0.01	0.50	BRAF; MEK
1437822_at	Yme1l1	YME1-like 1 (S. cerevisiae)	-4.65	0.01	0.83	n.r. ;
1428255_at	Luc7l	Luc7 homolog (S. cerevisiae)-like	-4.65	0.01	0.70	BRAF; MEK
1428448_a_at	Gtf3c2	general transcription factor IIIC. polypeptide 2. beta	-4.65	0.01	0.71	BRAF; MEK
1439753_x_at	Six4	sine oculis-related homeobox 4 homolog (Drosophila)	-4.65	0.01	0.47	BRAF; MEK
1435709_at	Ssr4	signal sequence receptor. delta	-4.65	0.01	0.76	; MEK
1419693_at	Colec12	collectin sub-family member 12	-4.65	0.01	0.35	; MEK
1437034_x_at	Marcks	myristoylated alanine rich protein kinase C substrate	-4.65	0.01	0.83	; MEK
1416198_at	Th1l	TH1-like homolog (Drosophila)	-4.65	0.01	0.61	BRAF; MEK
1418943_at	B230120H23Rik	RIKEN cDNA B230120H23 gene	-4.65	0.01	0.39	BRAF
1419470_at	Gnb4	guanine nucleotide binding protein (G protein). beta 4	-4.65	0.01	0.38	
1416210_at	Imp3	IMP3. U3 small nucleolar ribonucleoprotein. homolog (yeast)	-4.66	0.01	0.72	BRAF; MEK
1433746_at	Wdr3	WD repeat domain 3	-4.66	0.01	0.59	BRAF; MEK
1434116_at	Cbx2	chromobox homolog 2 (Drosophila Pc class)	-4.66	0.01	0.52	BRAF; MEK
1448235_s_at	Hmgb1	high mobility group box 1	-4.66	0.01	0.41	; MEK
1452987_at	Josd3	Josephin domain containing 3	-4.66	0.01	0.55	; MEK
1422966_a_at	Tfrc	transferrin receptor	-4.66	0.01	0.27	n.r. ;
1449080_at	Hdac2	histone deacetylase 2	-4.66	0.01	0.53	BRAF; MEK
1426271_at	Smc5	structural maintenance of chromosomes 5	-4.66	0.01	0.55	; MEK
1451208_at	Etf1	eukaryotic translation termination factor 1	-4.66	0.01	0.72	n.r. ;
1423123_at	Rad54l	RAD54 like (S. cerevisiae)	-4.66	0.01	0.66	BRAF; MEK
1419620_at	Pttg1	pituitary tumor-transforming 1	-4.66	0.01	0.71	; MEK
1420937_at	Cpsf2	cleavage and polyadenylation specific factor 2	-4.66	0.01	0.71	BRAF
1433718_a_at	Cbx1	chromobox homolog 1 (Drosophila HP1 beta)	-4.66	0.01	0.70	BRAF; MEK
1448828_at	Smc6	structural maintenance of chromosomes 6	-4.66	0.01	0.58	; MEK
1416962_at	Rcc1	regulator of chromosome condensation 1	-4.67	0.01	0.54	BRAF; MEK
1437856_at	Ipmk	inositol polyphosphate multikinase	-4.67	0.01	0.71	BRAF; MEK
1448504_a_at	Cbx3	chromobox homolog 3 (Drosophila HP1 gamma)	-4.67	0.01	0.57	BRAF; MEK
1452049_at	Rpl7l1	ribosomal protein L7-like 1	-4.67	0.01	0.69	BRAF; MEK
1416184_s_at	Hmga1	high mobility group AT-hook 1	-4.67	0.01	0.15	BRAF; MEK

Probeset ID	*Symbol*	*Gene Name*	*d-value*	*q-value*	*R-fold*	*Expression reversed by*
1421354_at	Prkg2	protein kinase. cGMP-dependent. type II	-4.67	0.01	0.68	BRAF; MEK
1417131_at	Cdc25a	cell division cycle 25 homolog A (S. pombe)	-4.68	0.01	0.59	BRAF; MEK
1430165_at	Stk17b	serine/threonine kinase 17b (apoptosis-inducing)	-4.68	0.01	0.78	; MEK
1454609_x_at	6430527G18Rik	RIKEN cDNA 6430527G18 gene	-4.68	0.01	0.54	BRAF; MEK
1436747_at	1110014K08Rik	RIKEN cDNA 1110014K08 gene	-4.68	0.01	0.60	BRAF; MEK
1436030_at	Cachd1	cache domain containing 1	-4.68	0.01	0.73	BRAF; MEK
1458667_at	4930519N13Rik	RIKEN cDNA 4930519N13 gene	-4.68	0.01	0.49	BRAF; MEK
1429183_at	Pkp2	plakophilin 2	-4.68	0.01	0.46	n.r. ; n.r.
1440254_at	100041277	predicted gene. 100041277	-4.68	0.01	0.68	; MEK
1418442_at	Xpo1	exportin 1. CRM1 homolog (yeast)	-4.68	0.01	0.53	BRAF; MEK
1434602_at	Med13l	mediator complex subunit 13-like	-4.68	0.01	0.63	BRAF; MEK
1420753_at	Tll1	tolloid-like	-4.69	0.01	0.44	n.r. ; n.r.
1438339_at	Fancd2	Fanconi anemia. complementation group D2	-4.69	0.01	0.51	BRAF; MEK
1449291_a_at	Dcbld1	discoidin. CUB and LCCL domain containing 1	-4.69	0.01	0.63	BRAF
1433872_at	2410042D21Rik	RIKEN cDNA 2410042D21 gene	-4.69	0.01	0.73	; MEK
1438833_at	Casc5	cancer susceptibility candidate 5	-4.69	0.01	0.55	BRAF; MEK
1438630_x_at	Mat2a	methionine adenosyltransferase II. alpha	-4.69	0.01	0.70	BRAF; MEK
1438429_at	2610319H10Rik	RIKEN cDNA 2610319H10 gene	-4.69	0.01	0.41	; MEK
1433937_at	Trp53bp2	transformation related protein 53 binding protein 2	-4.70	0.01	0.74	BRAF; MEK
1416126_at	Rpo1-2	RNA polymerase 1-2	-4.70	0.01	0.61	BRAF; MEK
1416144_a_at	Dhx15	DEAH (Asp-Glu-Ala-His) box polypeptide 15	-4.70	0.01	0.63	BRAF; MEK
1415916_a_at	Mthfd1	methylenetetrahydrofolate dehydrogenase (NADP+ dependent). methenyltetrahydrofolate cyclohydrolase. formyltetrahydrofolate synthase	-4.70	0.01	0.58	BRAF; MEK
1437073_x_at	NA	NA	-4.70	0.01	0.63	BRAF; MEK
1453038_at	4930422G04Rik	RIKEN cDNA 4930422G04 gene	-4.70	0.01	0.61	BRAF; MEK
1449328_at	Ly75	lymphocyte antigen 75	-4.70	0.01	0.48	BRAF; MEK
1425753_a_at	Ung	uracil DNA glycosylase	-4.70	0.01	0.45	BRAF; MEK
1423306_at	2010002N04Rik	RIKEN cDNA 2010002N04 gene	-4.71	0.01	0.55	BRAF; MEK
1451346_at	Mtap	methylthioadenosine phosphorylase	-4.71	0.01	0.66	; MEK
1417522_at	Fbxo32	F-box protein 32	-4.71	0.01	0.49	
1456730_x_at	Actl6a	actin-like 6A	-4.71	0.01	0.65	BRAF; MEK
1433762_at	C630043F03Rik	RIKEN cDNA C630043F03 gene	-4.72	0.01	0.75	BRAF; MEK
1415851_a_at	Impdh2	inosine 5'-phosphate dehydrogenase 2	-4.72	0.01	0.57	BRAF; MEK
1425007_at	Zfp566	zinc finger protein 566	-4.72	0.01	0.69	BRAF; MEK
1455029_at	Kif21a	kinesin family member 21A	-4.72	0.01	0.63	BRAF; MEK
1451521_x_at	Eif4h	eukaryotic translation initiation factor 4H	-4.72	0.01	0.81	BRAF; MEK
1429364_at	4930579G24Rik	RIKEN cDNA 4930579G24 gene	-4.72	0.01	0.62	BRAF; MEK
1438168_x_at	Ddx39	DEAD (Asp-Glu-Ala-Asp) box polypeptide 39	-4.73	0.01	0.67	BRAF; MEK
1428615_at	P2ry5	purinergic receptor P2Y. G-protein coupled. 5	-4.73	0.01	0.59	
1437179_at	Rif1	Rap1 interacting factor 1 homolog (yeast)	-4.73	0.01	0.37	BRAF; MEK
1448231_at	Fkbp5	FK506 binding protein 5	-4.73	0.01	0.45	
1424571_at	Ddx46	DEAD (Asp-Glu-Ala-Asp) box polypeptide 46	-4.73	0.01	0.74	; n.r.
1430028_at	2210018M11Rik	RIKEN cDNA 2210018M11 gene	-4.73	0.01	0.71	
1416569_at	Actl6a	actin-like 6A	-4.74	0.01	0.63	BRAF; MEK
1417404_at	Elovl6	ELOVL family member 6. elongation of long chain fatty acids (yeast)	-4.74	0.01	0.56	; MEK
1452197_at	Smc4	structural maintenance of chromosomes 4	-4.74	0.01	0.40	BRAF; MEK
1426297_at	Tcfe2a	transcription factor E2a	-4.74	0.01	0.61	BRAF; MEK
1431181_a_at	Luc7l	Luc7 homolog (S. cerevisiae)-like	-4.74	0.01	0.66	BRAF
1419156_at	Sox4	SRY-box containing gene 4	-4.74	0.01	0.54	BRAF; MEK
1426381_at	Pprc1	peroxisome proliferative activated receptor. gamma. coactivator-related 1	-4.74	0.01	0.60	BRAF; MEK
1417488_at	Fosl1	fos-like antigen 1	-4.74	0.01	0.32	BRAF; MEK
1430542_a_at	Slc25a5	solute carrier family 25 (mitochondrial carrier. adenine nucleotide translocator). member 5	-4.74	0.01	0.69	BRAF; MEK
1455740_at	Hnrnpa1	heterogeneous nuclear ribonucleoprotein A1	-4.74	0.01	0.34	; MEK
1448953_at	Blm	Bloom syndrome homolog (human)	-4.75	0.01	0.44	BRAF; MEK

Probeset ID	Symbol	Gene Name	d-value	q-value	R-fold	Expression reversed by
1454968_at	1110034A24Rik	RIKEN cDNA 1110034A24 gene	-4.75	0.01	0.58	BRAF; MEK
1436186_at	E2f8	E2F transcription factor 8	-4.75	0.01	0.46	BRAF; MEK
1438287_x_at	Ddx39	DEAD (Asp-Glu-Ala-Asp) box polypeptide 39	-4.75	0.01	0.76	BRAF
1451740_at	Paip1	polyadenylate binding protein-interacting protein 1	-4.75	0.01	0.57	BRAF; MEK
1427746_x_at	H2-K1	histocompatibility 2. K1. K region	-4.75	0.01	0.36	; MEK
1433535_x_at	Cct2	chaperonin containing Tcp1. subunit 2 (beta)	-4.75	0.01	0.77	BRAF; MEK
1459765_s_at	Sf1	splicing factor 1	-4.76	0.01	0.61	BRAF; MEK
1454795_at	Cobll1	Cobl-like 1	-4.76	0.01	0.40	BRAF; MEK
1416161_at	Rad21	RAD21 homolog (S. pombe)	-4.76	0.01	0.53	BRAF; MEK
1419568_at	Mapk1	mitogen-activated protein kinase 1	-4.76	0.01	0.74	BRAF; n.r.
1421033_a_at	Tcerg1	transcription elongation regulator 1 (CA150)	-4.76	0.01	0.58	BRAF; MEK
1449470_at	Dlx1	distal-less homeobox 1	-4.76	0.01	0.50	BRAF; MEK
1436026_at	Zfp703	zinc finger protein 703	-4.77	0.01	0.77	BRAF; MEK
1423431_a_at	Mybbp1a	MYB binding protein (P160) 1a	-4.77	0.01	0.67	BRAF; MEK
1418476_at	Crlf1	cytokine receptor-like factor 1	-4.77	0.01	0.44	BRAF; MEK
1426343_at	Stt3b	STT3. subunit of the oligosaccharyltransferase complex. homolog B (S. cerevisiae)	-4.77	0.01	0.75	
1418023_at	Narg1	NMDA receptor-regulated gene 1	-4.77	0.01	0.66	; MEK
1421934_at	Cbx5	chromobox homolog 5 (Drosophila HP1a)	-4.77	0.01	0.63	BRAF; MEK
1451428_x_at	Egfl7	EGF-like domain 7	-4.77	0.01	0.70	; MEK
1434981_at	E130303B06Rik	RIKEN cDNA E130303B06 gene	-4.77	0.01	0.65	BRAF; MEK
1454928_at	Safb	scaffold attachment factor B	-4.77	0.01	0.65	BRAF; MEK
1424161_at	Ddx27	DEAD (Asp-Glu-Ala-Asp) box polypeptide 27	-4.78	0.01	0.59	BRAF; MEK
1435575_at	Kntc1	kinetochore associated 1	-4.78	0.01	0.32	BRAF; MEK
1423674_at	Usp1	ubiquitin specific peptdiase 1	-4.78	0.01	0.45	BRAF; MEK
1424891_a_at	Zw10	ZW10 homolog (Drosophila). centromere/kinetochore protein	-4.78	0.01	0.66	BRAF; MEK
1428919_at	Fgfr1op	Fgfr1 oncogene partner	-4.78	0.01	0.63	; MEK
1428728_at	Ddx51	DEAD (Asp-Glu-Ala-Asp) box polypeptide 51	-4.78	0.01	0.74	BRAF; MEK
1434699_at	6030408C04Rik	RIKEN cDNA 6030408C04 gene	-4.78	0.01	0.36	BRAF; MEK
1448132_at	Slc19a1	solute carrier family 19 (sodium/hydrogen exchanger). member 1	-4.78	0.01	0.64	BRAF
1427506_at	Ppil5	peptidylprolyl isomerase (cyclophilin) like 5	-4.79	0.00	0.66	BRAF; MEK
1418127_a_at	Aifm1	apoptosis-inducing factor. mitochondrion-associated 1	-4.79	0.00	0.53	BRAF; MEK
1426463_at	Gphn	gephyrin	-4.79	0.00	0.59	BRAF; MEK
1454657_s_at	Mak10	MAK10 homolog. amino-acid N-acetyltransferase subunit. (S. cerevisiae)	-4.80	0.00	0.79	BRAF; MEK
1426839_at	Pold3	polymerase (DNA-directed). delta 3. accessory subunit	-4.80	0.00	0.55	BRAF
1426576_at	Sgms1	sphingomyelin synthase 1	-4.80	0.00	0.66	n.r. ; n.r.
1452061_s_at	Strbp	spermatid perinuclear RNA binding protein	-4.81	0.00	0.54	; MEK
1459874_s_at	Mtmr4	myotubularin related protein 4	-4.81	0.00	0.61	BRAF; MEK
1450668_s_at	Hspe1	heat shock protein 1 (chaperonin 10)	-4.81	0.00	0.78	BRAF; MEK
1437979_at	Zcchc2	zinc finger. CCHC domain containing 2	-4.81	0.00	0.69	
1435448_at	Bcl2l11	BCL2-like 11 (apoptosis facilitator)	-4.81	0.00	0.58	BRAF; MEK
1426580_at	Plk4	polo-like kinase 4 (Drosophila)	-4.81	0.00	0.36	BRAF; MEK
1428264_at	Wdr57	WD repeat domain 57 (U5 snRNP specific)	-4.81	0.00	0.63	BRAF; MEK
1456055_x_at	Pold1	polymerase (DNA directed). delta 1. catalytic subunit	-4.81	0.00	0.41	BRAF; MEK
1458165_at	Ipo7	importin 7	-4.81	0.00	0.66	BRAF
1421894_a_at	Tpp2	tripeptidyl peptidase II	-4.82	0.00	0.76	
1439091_at	Fancd2	Fanconi anemia. complementation group D2	-4.82	0.00	0.36	BRAF; MEK
1423440_at	1110001A07Rik	RIKEN cDNA 1110001A07 gene	-4.82	0.00	0.58	BRAF; MEK
1454978_at	Ttyh3	tweety homolog 3 (Drosophila)	-4.82	0.00	0.59	BRAF; MEK
1415781_a_at	Sumo2	SMT3 suppressor of mif two 3 homolog 2 (yeast)	-4.82	0.00	0.82	BRAF; MEK
1434850_at	Iqgap3	IQ motif containing GTPase activating protein 3	-4.82	0.00	0.43	BRAF; MEK
1429104_at	Limd2	LIM domain containing 2	-4.82	0.00	0.49	BRAF; MEK
1428310_at	Larp7	La ribonucleoprotein domain family. member 7	-4.82	0.00	0.54	BRAF; MEK
1427886_at	Pom121	nuclear pore membrane protein 121	-4.83	0.00	0.64	BRAF; MEK
1449227_at	Ch25h	cholesterol 25-hydroxylase	-4.83	0.00	0.74	; MEK
1440226_at	Zfp760	zinc finger protein 760	-4.83	0.00	0.33	; MEK

Probeset ID	Symbol	Gene Name	d-value	q-value	R-fold	Expression reversed by
1416024_x_at	Cct3	chaperonin containing Tcp1. subunit 3 (gamma)	-4.83	0.00	0.78	BRAF; MEK
1436036_at	Whsc2	Wolf-Hirschhorn syndrome candidate 2 (human)	-4.83	0.00	0.52	BRAF; MEK
1428532_at	Ints7	integrator complex subunit 7	-4.83	0.00	0.54	BRAF; MEK
1453107_s_at	Pebp1	phosphatidylethanolamine binding protein 1	-4.83	0.00	0.31	BRAF; MEK
1433789_at	Snhg3	small nucleolar RNA host gene (non-protein coding) 3	-4.84	0.00	0.43	BRAF; MEK
1451111_at	Nup133	nucleoporin 133	-4.84	0.00	0.55	BRAF; MEK
1427276_at	Smc4	structural maintenance of chromosomes 4	-4.84	0.00	0.42	BRAF; MEK
1456067_at	Gli3	GLI-Kruppel family member GLI3	-4.84	0.00	0.62	BRAF; MEK
1421498_a_at	2010204K13Rik	RIKEN cDNA 2010204K13 gene	-4.84	0.00	0.56	BRAF; MEK
1437285_at	1110020G09Rik	RIKEN cDNA 1110020G09 gene	-4.84	0.00	0.76	BRAF; MEK
1422824_s_at	Eps8	epidermal growth factor receptor pathway substrate 8	-4.84	0.00	0.37	BRAF; MEK
1448187_at	Pold1	polymerase (DNA directed). delta 1. catalytic subunit	-4.84	0.00	0.45	BRAF; MEK
1443688_at	BC050092	cDNA sequence BC050092	-4.84	0.00	0.71	BRAF; MEK
1427876_at	Zc3h15	zinc finger CCCH-type containing 15	-4.85	0.00	0.71	; MEK
1454679_at	Zfp828	zinc finger protein 828	-4.85	0.00	0.76	BRAF; MEK
1416593_at	Glrx	glutaredoxin	-4.85	0.00	0.45	
1427439_s_at	Prmt5	protein arginine N-methyltransferase 5	-4.85	0.00	0.70	
1433552_a_at	Polr2b	polymerase (RNA) II (DNA directed) polypeptide B	-4.85	0.00	0.68	BRAF; MEK
1448149_at	Ctnna1	catenin (cadherin associated protein). alpha 1	-4.85	0.00	0.74	BRAF; MEK
1422848_a_at	Pabpn1	poly? binding protein. nuclear 1	-4.85	0.00	0.66	BRAF; MEK
1451530_at	Egfr	epidermal growth factor receptor	-4.85	0.00	0.42	n.r. ; n.r.
1433669_at	Akap8	A kinase (PRKA) anchor protein 8	-4.85	0.00	0.65	BRAF; MEK
1451994_s_at	3930401K13Rik	RIKEN cDNA 3930401K13 gene	-4.85	0.00	0.77	BRAF
1440739_at	Vegfc	vascular endothelial growth factor C	-4.85	0.00	0.29	BRAF; MEK
1452344_at	Synj2	synaptojanin 2	-4.85	0.00	0.39	BRAF; MEK
1456028_x_at	Marcks	myristoylated alanine rich protein kinase C substrate	-4.86	0.00	0.68	BRAF; MEK
1447930_at	Baz1a	bromodomain adjacent to zinc finger domain 1A	-4.86	0.00	0.65	BRAF
1417940_s_at	Rad51ap1	RAD51 associated protein 1	-4.86	0.00	0.56	BRAF
1451782_a_at	Slc29a1	solute carrier family 29 (nucleoside transporters). member 1	-4.86	0.00	0.45	BRAF; MEK
1447880_x_at	S1pr2	sphingosine-1-phosphate receptor 2	-4.86	0.00	0.70	BRAF; MEK
1448144_at	Hnrnpab	heterogeneous nuclear ribonucleoprotein A/B	-4.86	0.00	0.68	BRAF
1429057_at	Narg1l	NMDA receptor regulated 1-like	-4.86	0.00	0.51	BRAF; MEK
1424193_at	Pwp2	PWP2 periodic tryptophan protein homolog (yeast)	-4.87	0.00	0.74	BRAF; MEK
1423181_s_at	Clns1a	chloride channel. nucleotide-sensitive. 1A	-4.87	0.00	0.78	BRAF
1460403_at	Psip1	PC4 and SFRS1 interacting protein 1	-4.87	0.00	0.49	BRAF; MEK
1429503_at	2900024C23Rik	RIKEN cDNA 2900024C23 gene	-4.87	0.00	0.46	BRAF; MEK
1453053_at	2610036L11Rik	RIKEN cDNA 2610036L11 gene	-4.87	0.00	0.63	BRAF; MEK
1453283_at	Pgm1	phosphoglucomutase 1	-4.88	0.00	0.77	; MEK
1460429_at	Cdc5l	cell division cycle 5-like (S. pombe)	-4.88	0.00	0.62	BRAF; MEK
1454074_a_at	Rsrc2	arginine/serine-rich coiled-coil 2	-4.88	0.00	0.66	; MEK
1425498_at	Prpf4b	PRP4 pre-mRNA processing factor 4 homolog B (yeast)	-4.88	0.00	0.68	
1418640_at	Sirt1	sirtuin 1 (silent mating type information regulation 2. homolog) 1 (S. cerevisiae)	-4.88	0.00	0.64	BRAF; MEK
1416208_at	Usp14	ubiquitin specific peptidase 14	-4.88	0.00	0.69	
1417352_s_at	Snrpa1	small nuclear ribonucleoprotein polypeptide A'	-4.88	0.00	0.53	BRAF; MEK
1455897_x_at	Hmgn1	high mobility group nucleosomal binding domain 1	-4.88	0.00	0.72	BRAF; MEK
1443807_x_at	Ccnf	cyclin F	-4.88	0.00	0.37	BRAF; MEK
1427062_at	Rbbp8	retinoblastoma binding protein 8	-4.89	0.00	0.61	n.r. ;
1417132_at	Cdc25a	cell division cycle 25 homolog A (S. pombe)	-4.89	0.00	0.56	BRAF; MEK
1431037_a_at	Elavl1	ELAV (embryonic lethal. abnormal vision. Drosophila)-like 1 (Hu antigen R)	-4.89	0.00	0.66	BRAF; MEK
1449893_a_at	Lrig1	leucine-rich repeats and immunoglobulin-like domains 1	-4.89	0.00	0.41	BRAF; MEK
1437593_x_at	Api5	apoptosis inhibitor 5	-4.89	0.00	0.78	BRAF; MEK
1434842_s_at	Upf3b	UPF3 regulator of nonsense transcripts homolog B (yeast)	-4.89	0.00	0.69	BRAF; MEK
1434434_s_at	Tcerg1	transcription elongation regulator 1 (CA150)	-4.89	0.00	0.60	BRAF; MEK
1429588_at	2810474O19Rik	RIKEN cDNA 2810474O19 gene	-4.89	0.00	0.60	; MEK
1456862_at	Six4	sine oculis-related homeobox 4 homolog (Drosophila)	-4.90	0.00	0.44	BRAF; MEK

Probeset ID	Symbol	Gene Name	d-value	q-value	R-fold	Expression reversed by
1436872_at	Tacc3	transforming. acidic coiled-coil containing protein 3	-4.90	0.00	0.59	BRAF; MEK
1415965_at	Scd1	stearoyl-Coenzyme A desaturase 1	-4.90	0.00	0.46	; MEK
1430805_s_at	Rmi1	RMI1. RecQ mediated genome instability 1. homolog (S. cerevisiae)	-4.90	0.00	0.60	
1425986_a_at	Dcun1d1	DCN1. defective in cullin neddylation 1. domain containing 1 (S. cerevisiae)	-4.90	0.00	0.65	; MEK
1440635_at	Palld	palladin. cytoskeletal associated protein	-4.90	0.00	0.52	; MEK
1436454_x_at	Fen1	flap structure specific endonuclease 1	-4.90	0.00	0.62	BRAF; MEK
1427141_at	2700099C18Rik	RIKEN cDNA 2700099C18 gene	-4.90	0.00	0.44	BRAF; MEK
1417263_at	Ptgs2	prostaglandin-endoperoxide synthase 2	-4.91	0.00	0.17	n.r. ; n.r.
1451103_at	D14Ertd500e	DNA segment. Chr 14. ERATO Doi 500. expressed	-4.91	0.00	0.46	BRAF; MEK
1438462_x_at	Khdrbs1	KH domain containing. RNA binding. signal transduction associated 1	-4.91	0.00	0.72	BRAF; MEK
1439510_at	Sgol1	shugoshin-like 1 (S. pombe)	-4.91	0.00	0.29	BRAF; MEK
1433794_at	Setx	senataxin	-4.91	0.00	0.65	; MEK
1451092_a_at	Rangap1	RAN GTPase activating protein 1	-4.91	0.00	0.61	BRAF; MEK
1450920_at	Ccnb2	cyclin B2	-4.92	0.00	0.34	BRAF; MEK
1429080_at	Mphosph10	M-phase phosphoprotein 10 (U3 small nucleolar ribonucleoprotein)	-4.92	0.00	0.65	n.r. ;
1423699_at	Ncaph2	non-SMC condensin II complex. subunit H2	-4.92	0.00	0.77	BRAF; MEK
1451901_at	Hltf	helicase-like transcription factor	-4.92	0.00	0.81	
1438988_x_at	Hn1	hematological and neurological expressed sequence 1	-4.92	0.00	0.69	BRAF; MEK
1456321_at	Npal1	NIPA-like domain containing 1	-4.92	0.00	0.58	; MEK
1432016_a_at	Idh3a	isocitrate dehydrogenase 3 (NAD+) alpha	-4.92	0.00	0.70	; n.r.
1429156_at	2610036L11Rik	RIKEN cDNA 2610036L11 gene	-4.92	0.00	0.48	BRAF; MEK
1424880_at	Trib1	tribbles homolog 1 (Drosophila)	-4.92	0.00	0.56	BRAF; MEK
1455814_x_at	Ddx39	DEAD (Asp-Glu-Ala-Asp) box polypeptide 39	-4.93	0.00	0.66	BRAF; MEK
1448165_at	Casp2	caspase 2	-4.93	0.00	0.58	BRAF; MEK
1415810_at	Uhrf1	ubiquitin-like. containing PHD and RING finger domains. 1	-4.94	0.00	0.21	BRAF; MEK
1423787_at	Nup133	nucleoporin 133	-4.94	0.00	0.57	BRAF; MEK
1449522_at	Unc5c	unc-5 homolog C (C. elegans)	-4.94	0.00	0.38	BRAF; MEK
1424569_at	Ddx46	DEAD (Asp-Glu-Ala-Asp) box polypeptide 46	-4.94	0.00	0.57	BRAF
1439556_at	Ncam1	neural cell adhesion molecule 1	-4.94	0.00	0.29	; MEK
1420936_s_at	Cpsf2	cleavage and polyadenylation specific factor 2	-4.94	0.00	0.67	BRAF; MEK
1436118_at	Vangl2	vang-like 2 (van gogh. Drosophila)	-4.94	0.00	0.52	BRAF; MEK
1428634_at	Twistnb	TWIST neighbor	-4.94	0.00	0.78	BRAF; MEK
1454648_s_at	D10Wsu102e	DNA segment. Chr 10. Wayne State University 102. expressed	-4.94	0.00	0.61	BRAF
1455220_at	Frat2	frequently rearranged in advanced T-cell lymphomas 2	-4.94	0.00	0.62	BRAF; MEK
1447623_s_at	Prkd1	protein kinase D1	-4.94	0.00	0.49	BRAF; MEK
1417503_at	Rfc2	replication factor C (activator 1) 2	-4.94	0.00	0.69	BRAF; MEK
1434173_s_at	D19Bwg1357e	DNA segment. Chr 19. Brigham & Women's Genetics 1357 expressed	-4.95	0.00	0.63	BRAF; MEK
1434859_at	Umps	uridine monophosphate synthetase	-4.95	0.00	0.57	BRAF; MEK
1422503_s_at	Parp1	poly (ADP-ribose) polymerase family. member 1	-4.95	0.00	0.63	BRAF; MEK
1443794_x_at	Noc4l	nucleolar complex associated 4 homolog (S. cerevisiae)	-4.95	0.00	0.58	BRAF; MEK
1452099_at	AA408296	expressed sequence AA408296	-4.95	0.00	0.67	BRAF; MEK
1449730_s_at	Fzd3	frizzled homolog 3 (Drosophila)	-4.95	0.00	0.72	
1428674_at	Prpf38b	PRP38 pre-mRNA processing factor 38 (yeast) domain containing B	-4.95	0.00	0.63	; MEK
1422628_at	4632417K18Rik	RIKEN cDNA 4632417K18 gene	-4.96	0.00	0.23	BRAF; MEK
1433813_at	NA	NA	-4.96	0.00	0.61	BRAF; MEK
1450874_at	Matr3	matrin 3	-4.96	0.00	0.75	n.r. ;
1439041_at	Slc39a10	solute carrier family 39 (zinc transporter). member 10	-4.96	0.00	0.42	
1426270_at	Smc5	structural maintenance of chromosomes 5	-4.96	0.00	0.56	; MEK
1451044_at	Sip1	survival of motor neuron protein interacting protein 1	-4.97	0.00	0.50	BRAF; MEK
1429446_at	Sdccag1	serologically defined colon cancer antigen 1	-4.97	0.00	0.65	; MEK
1416940_at	Ppif	peptidylprolyl isomerase F (cyclophilin F)	-4.98	0.00	0.58	BRAF; MEK
1428207_at	Bcl7a	B-cell CLL/lymphoma 7A	-4.98	0.00	0.61	BRAF; MEK
1456212_x_at	Socs3	suppressor of cytokine signaling 3	-4.98	0.00	0.38	BRAF; MEK
1435449_at	Bcl2l11	BCL2-like 11 (apoptosis facilitator)	-4.98	0.00	0.52	BRAF; MEK
1456652_at	Dtl	denticleless homolog (Drosophila)	-4.98	0.00	0.46	BRAF; MEK

Probeset ID	Symbol	Gene Name	d-value	q-value	R-fold	Expression reversed by
1415820_x_at	Nono	non-POU-domain-containing. octamer binding protein	-4.98	0.00	0.70	BRAF; MEK
1439766_x_at	Vegfc	vascular endothelial growth factor C	-4.98	0.00	0.28	BRAF; MEK
1456698_s_at	Hnrpdl	heterogeneous nuclear ribonucleoprotein D-like	-4.99	0.00	0.58	BRAF; MEK
1434801_x_at	Slc25a5	solute carrier family 25 (mitochondrial carrier. adenine nucleotide translocator). member 5	-4.99	0.00	0.73	BRAF
1448679_at	Hyal2	hyaluronoglucosaminidase 2	-4.99	0.00	0.72	BRAF; MEK
1416791_a_at	Nxf1	nuclear RNA export factor 1 homolog (S. cerevisiae)	-4.99	0.00	0.65	BRAF; MEK
1435222_at	Foxp1	forkhead box P1	-4.99	0.00	0.67	
1426958_at	Rps9	ribosomal protein S9	-4.99	0.00	0.45	BRAF; MEK
1448283_a_at	Uba2	ubiquitin-like modifier activating enzyme 2	-4.99	0.00	0.64	BRAF; MEK
1451402_at	Ecd	ecdysoneless homolog (Drosophila)	-4.99	0.00	0.60	BRAF; MEK
1427159_at	Pcf11	cleavage and polyadenylation factor subunit homolog (S. cerevisiae)	-4.99	0.00	0.66	BRAF; MEK
1451190_a_at	Sbk1	SH3-binding kinase 1	-5.00	0.00	0.41	BRAF; MEK
1417041_at	Rpo1-1	RNA polymerase 1-1	-5.00	0.00	0.66	BRAF; MEK
1452852_at	Twistnb	TWIST neighbor	-5.00	0.00	0.74	BRAF
1416778_at	Sdpr	serum deprivation response	-5.00	0.00	0.72	; n.r.
1437743_at	Aebp2	AE binding protein 2	-5.00	0.00	0.61	BRAF; n.r.
1427317_at	Kin	antigenic determinant of rec-A protein	-5.00	0.00	0.76	
1416256_a_at	Tubb5	tubulin. beta 5	-5.00	0.00	0.78	BRAF; MEK
1432179_x_at	2810433K01Rik	RIKEN cDNA 2810433K01 gene	-5.00	0.00	0.57	BRAF; MEK
1427083_a_at	Map4k5	mitogen-activated protein kinase kinase kinase kinase 5	-5.01	0.00	0.71	
1417057_a_at	Ppid	peptidylprolyl isomerase D (cyclophilin D)	-5.01	0.00	0.57	BRAF; MEK
1443394_at	NA	NA	-5.01	0.00	0.27	BRAF; MEK
1452315_at	Kif11	kinesin family member 11	-5.01	0.00	0.32	; n.r.
1431893_a_at	Pdss1	prenyl (solanesyl) diphosphate synthase. subunit 1	-5.01	0.00	0.41	BRAF; MEK
1460221_at	Ptges3	prostaglandin E synthase 3 (cytosolic)	-5.01	0.00	0.74	BRAF; MEK
1455943_at	Zfp451	zinc finger protein 451	-5.01	0.00	0.68	; MEK
1426609_at	Dis3	DIS3 mitotic control homolog (S. cerevisiae)	-5.02	0.00	0.46	BRAF; MEK
1426323_x_at	Siva1	SIVA1. apoptosis-inducing factor	-5.02	0.00	0.61	BRAF; MEK
1425029_a_at	Mboat2	membrane bound O-acyltransferase domain containing 2	-5.02	0.00	0.60	BRAF; MEK
1436018_at	Mex3a	mex3 homolog A (C. elegans)	-5.02	0.00	0.65	BRAF; MEK
1428105_at	Tpx2	TPX2. microtubule-associated protein homolog (Xenopus laevis)	-5.02	0.00	0.45	BRAF; MEK
1447766_x_at	Limd2	LIM domain containing 2	-5.02	0.00	0.69	BRAF; MEK
1435669_at	Zfp532	zinc finger protein 532	-5.02	0.00	0.67	BRAF; MEK
1422844_a_at	Wdr77	WD repeat domain 77	-5.02	0.00	0.67	BRAF; MEK
1426290_at	Dimt1	DIM1 dimethyladenosine transferase 1-like (S. cerevisiae)	-5.02	0.00	0.71	; n.r.
1422495_a_at	Hmgn1	high mobility group nucleosomal binding domain 1	-5.03	0.00	0.76	BRAF; MEK
1440924_at	Kif20b	kinesin family member 20B	-5.03	0.00	0.45	
1450929_at	Zfp57	zinc finger protein 57	-5.03	0.00	0.57	BRAF; MEK
1454801_at	Ankrd28	ankyrin repeat domain 28	-5.03	0.00	0.51	BRAF; MEK
1439758_at	Als2cr12	amyotrophic lateral sclerosis 2 (juvenile) chromosome region. candidate 12 (human)	-5.03	0.00	0.29	BRAF; MEK
1437414_at	Zfp217	zinc finger protein 217	-5.03	0.00	0.52	BRAF; MEK
1452838_at	Ddx10	DEAD (Asp-Glu-Ala-Asp) box polypeptide 10	-5.03	0.00	0.67	; MEK
1417242_at	Eif4a3	eukaryotic translation initiation factor 4A. isoform 3	-5.03	0.00	0.74	BRAF; MEK
1452877_at	2700029M09Rik	RIKEN cDNA 2700029M09 gene	-5.04	0.00	0.58	BRAF; MEK
1454032_at	Neto2	neuropilin (NRP) and tolloid (TLL)-like 2	-5.04	0.00	0.57	; MEK
1422442_at	Smu1	smu-1 suppressor of mec-8 and unc-52 homolog (C. elegans)	-5.04	0.00	0.83	BRAF; MEK
1436854_at	Trpc2	transient receptor potential cation channel. subfamily C. member 2	-5.04	0.00	0.72	; MEK
1455127_at	Xkr5	X Kell blood group precursor-related family. member 5	-5.04	0.00	0.64	BRAF; MEK
1438761_a_at	EG666231	predicted gene. EG666231	-5.04	0.00	0.66	BRAF; MEK
1423620_at	Cenpq	centromere protein Q	-5.05	0.00	0.37	BRAF; MEK
1427164_at	Il13ra1	interleukin 13 receptor. alpha 1	-5.05	0.00	0.38	; MEK
1437700_at	Schip1	schwannomin interacting protein 1	-5.05	0.00	0.49	
1422663_at	Orc1l	origin recognition complex. subunit 1-like (S.cereviaiae)	-5.05	0.00	0.40	BRAF; MEK
1448373_at	Mrpl18	mitochondrial ribosomal protein L18	-5.05	0.00	0.61	BRAF
1417453_at	Cul4b	cullin 4B	-5.06	0.00	0.67	; MEK

Probeset ID	Symbol	Gene Name	d-value	q-value	R-fold	Expression reversed by
1424136_a_at	Ppih	peptidyl prolyl isomerase H	-5.06	0.00	0.60	BRAF; MEK
1448838_at	Topors	topoisomerase I binding. arginine/serine-rich	-5.06	0.00	0.64	BRAF; MEK
1415772_at	Ncl	nucleolin	-5.06	0.00	0.53	BRAF; MEK
1434911_s_at	Arhgap19	Rho GTPase activating protein 19	-5.07	0.00	0.53	BRAF; MEK
1456721_at	Thsd7a	thrombospondin. type I. domain containing 7A	-5.07	0.00	0.60	BRAF; MEK
1417192_at	Tomm70a	translocase of outer mitochondrial membrane 70 homolog A (yeast)	-5.07	0.00	0.58	BRAF
1443999_at	NA	NA	-5.08	0.00	0.71	BRAF; MEK
1416557_a_at	Eftud2	elongation factor Tu GTP binding domain containing 2	-5.08	0.00	0.65	BRAF; MEK
1451343_at	Vps36	vacuolar protein sorting 36 (yeast)	-5.08	0.00	0.70	BRAF
1416942_at	Erap1	endoplasmic reticulum aminopeptidase 1	-5.08	0.00	0.64	BRAF; MEK
1455154_at	Gli3	GLI-Kruppel family member GLI3	-5.08	0.00	0.56	BRAF; MEK
1451430_at	Slit2	slit homolog 2 (Drosophila)	-5.08	0.00	0.74	; MEK
1448720_at	Lrrc40	leucine rich repeat containing 40	-5.09	0.00	0.60	BRAF; MEK
1453067_at	Apitd1	apoptosis-inducing. TAF9-like domain 1	-5.09	0.00	0.53	BRAF; MEK
1434119_at	D2Wsu81e	DNA segment. Chr 2. Wayne State University 81. expressed	-5.09	0.00	0.81	BRAF; MEK
1449349_at	Nudt1	nudix (nucleoside diphosphate linked moiety X)-type motif 1	-5.09	0.00	0.65	BRAF; MEK
1452231_x_at	Ifi205	interferon activated gene 205	-5.09	0.00	0.57	; MEK
1424110_a_at	Nme1	non-metastatic cells 1. protein (NM23A) expressed in	-5.09	0.00	0.66	BRAF; MEK
1415803_at	Cx3cl1	chemokine (C-X3-C motif) ligand 1	-5.09	0.00	0.39	BRAF
1451775_s_at	Il13ra1	interleukin 13 receptor. alpha 1	-5.09	0.00	0.34	; MEK
1437480_at	1110001A07Rik	RIKEN cDNA 1110001A07 gene	-5.09	0.00	0.55	BRAF; MEK
1418726_a_at	Tnnt2	troponin T2. cardiac	-5.09	0.00	0.16	BRAF; MEK
1424092_at	Epb4.1	erythrocyte protein band 4.1	-5.09	0.00	0.45	BRAF; MEK
1424766_at	Ercc6l	excision repair cross-complementing rodent repair deficiency complementation group 6 - like	-5.10	0.00	0.34	BRAF; MEK
1442322_at	NA	NA	-5.10	0.00	0.77	; MEK
1425177_at	Shmt1	serine hydroxymethyltransferase 1 (soluble)	-5.10	0.00	0.69	BRAF; MEK
1418942_at	Ift74	intraflagellar transport 74 homolog (Chlamydomonas)	-5.10	0.00	0.63	; MEK
1448236_at	Rdx	radixin	-5.10	0.00	0.68	BRAF; MEK
1423456_at	Bzw2	basic leucine zipper and W2 domains 2	-5.11	0.00	0.58	BRAF; MEK
1415995_at	Casp6	caspase 6	-5.11	0.00	0.72	BRAF; MEK
1419452_at	Uchl5	ubiquitin carboxyl-terminal esterase L5	-5.11	0.00	0.55	BRAF; MEK
1457832_at	NA	NA	-5.11	0.00	0.68	n.r. ; n.r.
1417139_at	Dsn1	DSN1. MIND kinetochore complex component. homolog (S. cerevisiae)	-5.11	0.00	0.56	BRAF; MEK
1421023_at	Pik3c2a	phosphatidylinositol 3-kinase. C2 domain containing. alpha polypeptide	-5.11	0.00	0.66	n.r. ; n.r.
1451400_at	Gemin8	gem (nuclear organelle) associated protein 8	-5.11	0.00	0.60	BRAF; MEK
1422502_at	Parp1	poly (ADP-ribose) polymerase family. member 1	-5.11	0.00	0.62	BRAF; MEK
1435497_at	5730590G19Rik	RIKEN cDNA 5730590G19 gene	-5.11	0.00	0.48	BRAF; MEK
1430147_a_at	Josd3	Josephin domain containing 3	-5.11	0.00	0.61	
1423755_at	Zcchc8	zinc finger. CCHC domain containing 8	-5.12	0.00	0.59	BRAF; MEK
1416037_a_at	Cct2	chaperonin containing Tcp1. subunit 2 (beta)	-5.12	0.00	0.74	BRAF; MEK
1438922_x_at	Slc25a5	solute carrier family 25 (mitochondrial carrier. adenine nucleotide translocator). member 5	-5.12	0.00	0.60	BRAF; MEK
1418035_a_at	Prim2	DNA primase. p58 subunit	-5.12	0.00	0.53	BRAF; MEK
1451306_at	Cdca7l	cell division cycle associated 7 like	-5.13	0.00	0.41	BRAF; MEK
1436000_a_at	Skp2	S-phase kinase-associated protein 2 (p45)	-5.13	0.00	0.36	BRAF; MEK
1429461_at	Ints2	integrator complex subunit 2	-5.13	0.00	0.72	BRAF; MEK
1424300_at	Gemin6	gem (nuclear organelle) associated protein 6	-5.13	0.00	0.48	BRAF; MEK
1456308_x_at	Trim28	tripartite motif-containing 28	-5.13	0.00	0.62	BRAF; MEK
1430271_x_at	Josd3	Josephin domain containing 3	-5.13	0.00	0.60	
1422788_at	Slc43a3	solute carrier family 43. member 3	-5.13	0.00	0.48	BRAF; n.r.
1449147_at	Chst1	carbohydrate (keratan sulfate Gal-6) sulfotransferase 1	-5.13	0.00	0.35	BRAF; MEK
1438161_s_at	Rfc4	replication factor C (activator 1) 4	-5.13	0.00	0.35	BRAF; MEK
1457944_at	NA	NA	-5.14	0.00	0.49	BRAF; MEK
1434401_at	Zcchc2	zinc finger. CCHC domain containing 2	-5.14	0.00	0.64	BRAF

Probeset ID	*Symbol*	*Gene Name*	*d-value*	*q-value*	*R-fold*	*Expression reversed by*
1418761_at	Igf2bp1	insulin-like growth factor 2 mRNA binding protein 1	-5.14	0.00	0.49	BRAF; MEK
1420497_a_at	Cebpz	CCAAT/enhancer binding protein zeta	-5.14	0.00	0.59	BRAF; MEK
1419786_at	Ltbp1	latent transforming growth factor beta binding protein 1	-5.14	0.00	0.69	; MEK
1420498_a_at	Dab2	disabled homolog 2 (Drosophila)	-5.14	0.00	0.72	BRAF; MEK
1435324_x_at	Hmgb1	high mobility group box 1	-5.14	0.00	0.49	BRAF; MEK
1437187_at	E2f7	E2F transcription factor 7	-5.14	0.00	0.30	BRAF; MEK
1449414_at	Zfp53	zinc finger protein 53	-5.14	0.00	0.55	; MEK
1415771_at	Ncl	nucleolin	-5.15	0.00	0.57	BRAF; MEK
1436935_x_at	ENSMUSG00000056003	predicted gene. ENSMUSG00000056003	-5.15	0.00	0.76	BRAF; MEK
1433892_at	Spag5	sperm associated antigen 5	-5.15	0.00	0.31	BRAF; MEK
1454951_at	Zfp606	zinc finger protein 606	-5.16	0.00	0.58	; MEK
1443978_at	Ankle1	ankyrin repeat and LEM domain containing 1	-5.16	0.00	0.64	; MEK
1435595_at	1810011O10Rik	RIKEN cDNA 1810011O10 gene	-5.16	0.00	0.42	BRAF; MEK
1420932_at	Mapk8	mitogen-activated protein kinase 8	-5.16	0.00	0.78	
1428648_at	Cand1	cullin associated and neddylation disassociated 1	-5.17	0.00	0.77	BRAF; MEK
1438095_x_at	Noc4l	nucleolar complex associated 4 homolog (S. cerevisiae)	-5.17	0.00	0.56	BRAF; MEK
1434942_at	Esf1	ESF1. nucleolar pre-rRNA processing protein. homolog (S. cerevisiae)	-5.17	0.00	0.53	; MEK
1416796_at	Nck2	non-catalytic region of tyrosine kinase adaptor protein 2	-5.17	0.00	0.63	BRAF; MEK
1436412_at	NA	NA	-5.17	0.00	0.71	
1426015_s_at	Asph	aspartate-beta-hydroxylase	-5.17	0.00	0.51	BRAF; MEK
1454896_at	Rbpj	recombination signal binding protein for immunoglobulin kappa J region	-5.17	0.00	0.53	BRAF; MEK
1438390_s_at	Pttg1	pituitary tumor-transforming 1	-5.18	0.00	0.42	BRAF; MEK
1436796_at	Matr3	matrin 3	-5.18	0.00	0.53	; MEK
1455592_at	Vangl2	vang-like 2 (van gogh. Drosophila)	-5.18	0.00	0.50	BRAF; MEK
1431225_at	NA	NA	-5.18	0.00	0.47	BRAF; MEK
1460247_a_at	Skp2	S-phase kinase-associated protein 2 (p45)	-5.19	0.00	0.38	BRAF; MEK
1453186_at	Sfrs18	splicing factor. arginine/serine-rich 18	-5.19	0.00	0.56	; MEK
1437408_at	Gpr126	G protein-coupled receptor 126	-5.20	0.00	0.61	BRAF
1445226_at	BC023969	cDNA sequence BC023969	-5.20	0.00	0.46	BRAF; MEK
1418675_at	Osmr	oncostatin M receptor	-5.20	0.00	0.32	; MEK
1423026_at	Rad51c	Rad51 homolog c (S. cerevisiae)	-5.20	0.00	0.51	BRAF
1447787_x_at	Gjc1	gap junction protein. gamma 1	-5.20	0.00	0.46	BRAF
1416928_at	Rbm12	RNA binding motif protein 12	-5.21	0.00	0.70	BRAF; MEK
1433960_at	Isg20l2	interferon stimulated exonuclease gene 20-like 2	-5.21	0.00	0.65	BRAF; MEK
1428315_at	Ebna1bp2	EBNA1 binding protein 2	-5.21	0.00	0.60	BRAF; MEK
1427061_at	Rbbp8	retinoblastoma binding protein 8	-5.21	0.00	0.56	BRAF; MEK
1416167_at	Prdx4	peroxiredoxin 4	-5.21	0.00	0.69	BRAF; MEK
1433996_at	Suv39h2	suppressor of variegation 3-9 homolog 2 (Drosophila)	-5.21	0.00	0.52	BRAF; MEK
1436892_at	Spred2	sprouty-related. EVH1 domain containing 2	-5.21	0.00	0.46	BRAF; MEK
1425006_a_at	Vrk1	vaccinia related kinase 1	-5.22	0.00	0.48	BRAF; MEK
1428227_at	Rest	RE1-silencing transcription factor	-5.22	0.00	0.72	BRAF; MEK
1422488_at	Nxt1	NTF2-related export protein 1	-5.22	0.00	0.64	BRAF; MEK
1425767_a_at	Six4	sine oculis-related homeobox 4 homolog (Drosophila)	-5.22	0.00	0.53	BRAF; MEK
1456528_x_at	Ncl	nucleolin	-5.23	0.00	0.64	BRAF; MEK
1448232_x_at	Tuba1c	tubulin. alpha 1C	-5.23	0.00	0.60	BRAF; MEK
1442821_at	NA	NA	-5.23	0.00	0.70	
1424348_at	1110007A13Rik	RIKEN cDNA 1110007A13 gene	-5.23	0.00	0.56	BRAF
1448413_at	2410016O06Rik	RIKEN cDNA 2410016O06 gene	-5.23	0.00	0.66	BRAF; MEK
1438453_at	Rad51c	Rad51 homolog c (S. cerevisiae)	-5.23	0.00	0.65	BRAF; MEK
1424613_at	Gprc5b	G protein-coupled receptor. family C. group 5. member B	-5.24	0.00	0.53	BRAF; MEK
1424948_x_at	H2-K1	histocompatibility 2. K1. K region	-5.24	0.00	0.28	; MEK
1453370_at	Troap	trophinin associated protein	-5.24	0.00	0.40	BRAF; MEK
1437549_at	2810408I11Rik	RIKEN cDNA 2810408I11 gene	-5.24	0.00	0.57	BRAF; MEK
1426684_at	Cnot6	CCR4-NOT transcription complex. subunit 6	-5.24	0.00	0.64	; MEK
1426235_a_at	Glul	glutamate-ammonia ligase (glutamine synthetase)	-5.24	0.00	0.50	BRAF; MEK

Probeset ID	Symbol	Gene Name	d-value	q-value	R-fold	Expression reversed by
1429087_at	1110054O05Rik	RIKEN cDNA 1110054O05 gene	-5.25	0.00	0.71	BRAF
1423525_at	Mastl	microtubule associated serine/threonine kinase-like	-5.25	0.00	0.50	BRAF; MEK
1435108_at	Arhgap22	Rho GTPase activating protein 22	-5.25	0.00	0.67	BRAF; MEK
1424500_at	Utp6	UTP6. small subunit (SSU) processome component. homolog (yeast)	-5.26	0.00	0.59	
1420876_a_at	Sept6	septin 6	-5.26	0.00	0.48	; MEK
1418736_at	B3galnt1	UDP-GalNAc:betaGlcNAc beta 1.3-galactosaminyltransferase. polypeptide 1	-5.26	0.00	0.42	; MEK
1438554_x_at	Eif4h	eukaryotic translation initiation factor 4H	-5.26	0.00	0.79	BRAF; MEK
1456079_x_at	Apex1	apurinic/apyrimidinic endonuclease 1	-5.26	0.00	0.64	BRAF; MEK
1419397_at	Pola1	polymerase (DNA directed). alpha 1	-5.27	0.00	0.25	BRAF; MEK
1424156_at	Rbl1	retinoblastoma-like 1 (p107)	-5.27	0.00	0.30	BRAF; MEK
1448633_at	Prpf31	PRP31 pre-mRNA processing factor 31 homolog (yeast)	-5.27	0.00	0.72	BRAF; MEK
1424105_a_at	Pttg1	pituitary tumor-transforming 1	-5.28	0.00	0.36	BRAF; MEK
1433935_at	AU020206	expressed sequence AU020206	-5.28	0.00	0.31	BRAF; MEK
1416095_x_at	1110005A23Rik	RIKEN cDNA 1110005A23 gene	-5.28	0.00	0.80	; MEK
1416034_at	Cd24a	CD24a antigen	-5.28	0.00	0.26	BRAF; MEK
1424208_at	Ptger4	prostaglandin E receptor 4 (subtype EP4)	-5.28	0.00	0.48	BRAF; MEK
1426930_at	Brunol4	bruno-like 4. RNA binding protein (Drosophila)	-5.29	0.00	0.40	BRAF; MEK
1452339_at	Adamts7	a disintegrin-like and metallopeptidase (reprolysin type) with thrombospondin type 1 motif. 7	-5.30	0.00	0.61	BRAF; MEK
1448706_at	Ttrap	Traf and Tnf receptor associated protein	-5.30	0.00	0.59	BRAF; MEK
1452115_a_at	Plk4	polo-like kinase 4 (Drosophila)	-5.30	0.00	0.31	BRAF
1426929_at	Brunol4	bruno-like 4. RNA binding protein (Drosophila)	-5.30	0.00	0.47	BRAF; MEK
1417037_at	Orc6l	origin recognition complex. subunit 6-like (S. cerevisiae)	-5.30	0.00	0.56	BRAF; MEK
1448224_at	Tfam	transcription factor A. mitochondrial	-5.30	0.00	0.60	BRAF; MEK
1456748_a_at	Nipsnap1	4-nitrophenylphosphatase domain and non-neuronal SNAP25-like protein homolog 1 (C. elegans)	-5.30	0.00	0.58	BRAF; MEK
1452831_s_at	Ppat	phosphoribosyl pyrophosphate amidotransferase	-5.30	0.00	0.50	BRAF; MEK
1452662_a_at	Eif2s1	eukaryotic translation initiation factor 2. subunit 1 alpha	-5.30	0.00	0.69	BRAF; MEK
1424704_at	Runx2	runt related transcription factor 2	-5.30	0.00	0.37	; MEK
1454739_at	Cdc27	cell division cycle 27 homolog (S. cerevisiae)	-5.30	0.00	0.77	n.r. ; MEK
1455141_at	Tnrc6a	trinucleotide repeat containing 6a	-5.31	0.00	0.69	n.r. ;
1442280_at	D2Ertd750e	DNA segment. Chr 2. ERATO Doi 750. expressed	-5.31	0.00	0.40	BRAF; MEK
1442134_at	Prr11	proline rich 11	-5.31	0.00	0.50	BRAF; MEK
1429184_at	100042856	predicted gene. 100042856	-5.31	0.00	0.13	; MEK
1439553_s_at	Nutf2	nuclear transport factor 2	-5.31	0.00	0.70	BRAF; MEK
1434079_s_at	Mcm2	minichromosome maintenance deficient 2 mitotin (S. cerevisiae)	-5.31	0.00	0.35	BRAF; MEK
1449115_at	Mtf2	metal response element binding transcription factor 2	-5.31	0.00	0.55	BRAF; MEK
1452459_at	Aspm	asp (abnormal spindle)-like. microcephaly associated (Drosophila)	-5.32	0.00	0.43	BRAF; MEK
1434668_at	Nucks1	nuclear casein kinase and cyclin-dependent kinase substrate 1	-5.32	0.00	0.66	
1433739_at	Nol10	nucleolar protein 10	-5.32	0.00	0.65	BRAF; MEK
1451845_a_at	Ptrh2	peptidyl-tRNA hydrolase 2	-5.32	0.00	0.66	BRAF; MEK
1435086_s_at	Klhdc2	kelch domain containing 2	-5.32	0.00	0.61	BRAF; MEK
1451459_at	Ahctf1	AT hook containing transcription factor 1	-5.32	0.00	0.58	BRAF; MEK
1456375_x_at	Trim27	tripartite motif-containing 27	-5.32	0.00	0.69	BRAF
1423614_at	Lrrc8c	leucine rich repeat containing 8 family. member C	-5.33	0.00	0.44	; n.r.
1418016_at	Pum2	pumilio 2 (Drosophila)	-5.33	0.00	0.72	BRAF; MEK
1423712_a_at	Qars	glutaminyl-tRNA synthetase	-5.33	0.00	0.80	BRAF; MEK
1441340_at	Cep68	centrosomal protein 68	-5.33	0.00	0.60	BRAF; MEK
1453077_a_at	Snapc3	small nuclear RNA activating complex. polypeptide 3	-5.33	0.00	0.62	n.r. ; n.r.
1433795_at	Tgfbr3	transforming growth factor. beta receptor III	-5.33	0.00	0.41	BRAF; MEK
1456415_at	Zfp451	zinc finger protein 451	-5.33	0.00	0.56	; MEK
1452917_at	Rfc5	replication factor C (activator 1) 5	-5.34	0.00	0.40	BRAF; MEK
1447935_at	Fancm	Fanconi anemia. complementation group M	-5.34	0.00	0.68	BRAF; MEK
1447815_x_at	6430527G18Rik	RIKEN cDNA 6430527G18 gene	-5.34	0.00	0.82	BRAF; MEK
1421937_at	Dapp1	dual adaptor for phosphotyrosine and 3-phosphoinositides 1	-5.34	0.00	0.64	BRAF; MEK
1419716_a_at	Pou2f1	POU domain. class 2. transcription factor 1	-5.34	0.00	0.67	BRAF; MEK

Probeset ID	***Symbol***	***Gene Name***	***d-value***	***q-value***	***R-fold***	***Expression reversed by***
1438307_at	NA	NA	-5.34	0.00	0.49	BRAF; MEK
1435773_at	4930547N16Rik	RIKEN cDNA 4930547N16 gene	-5.34	0.00	0.36	BRAF; MEK
1438835_a_at	Eftud2	elongation factor Tu GTP binding domain containing 2	-5.35	0.00	0.66	BRAF; MEK
1424827_a_at	Csnk1a1	casein kinase 1. alpha 1	-5.35	0.00	0.75	; MEK
1437319_at	Unc13c	unc-13 homolog C (C. elegans)	-5.35	0.00	0.63	BRAF; MEK
1438376_s_at	Trim27	tripartite motif-containing 27	-5.35	0.00	0.66	BRAF
1451641_at	Dbr1	debranching enzyme homolog 1 (S. cerevisiae)	-5.35	0.00	0.62	BRAF; MEK
1443733_x_at	Pold3	polymerase (DNA-directed). delta 3. accessory subunit	-5.35	0.00	0.60	BRAF; MEK
1434936_at	Hirip3	HIRA interacting protein 3	-5.36	0.00	0.47	BRAF; MEK
1456240_x_at	Cdca4	cell division cycle associated 4	-5.36	0.00	0.53	BRAF; MEK
1460726_at	Adss	adenylosuccinate synthetase. non muscle	-5.36	0.00	0.71	BRAF; MEK
1434700_at	6030408C04Rik	RIKEN cDNA 6030408C04 gene	-5.36	0.00	0.39	BRAF; MEK
1437780_at	Fancb	Fanconi anemia. complementation group B	-5.37	0.00	0.38	BRAF; MEK
1428512_at	Bhlhb9	basic helix-loop-helix domain containing. class B9	-5.37	0.00	0.46	BRAF; MEK
1422802_at	Defcr3	defensin related cryptdin 3	-5.37	0.00	0.56	BRAF
1426218_at	Glcci1	glucocorticoid induced transcript 1	-5.37	0.00	0.47	BRAF
1452912_at	Dscc1	defective in sister chromatid cohesion 1 homolog (S. cerevisiae)	-5.37	0.00	0.23	BRAF; MEK
1428661_at	Nfkbil2	nuclear factor of kappa light polypeptide gene enhancer in B-cells inhibitor-like 2	-5.37	0.00	0.64	BRAF; MEK
1434705_at	Ctbp2	C-terminal binding protein 2	-5.38	0.00	0.73	BRAF
1438757_at	C130069I09Rik	RIKEN cDNA C130069I09 gene	-5.38	0.00	0.45	BRAF; MEK
1433829_a_at	Hnrnpa2b1	heterogeneous nuclear ribonucleoprotein A2/B1	-5.38	0.00	0.69	
1451119_a_at	Fbln1	fibulin 1	-5.38	0.00	0.38	BRAF; MEK
1436723_at	Cenpi	centromere protein I	-5.39	0.00	0.31	BRAF; MEK
1418495_at	Zc3h8	zinc finger CCCH type containing 8	-5.39	0.00	0.72	BRAF; MEK
1429243_at	1110054O05Rik	RIKEN cDNA 1110054O05 gene	-5.39	0.00	0.73	
1428167_a_at	Mpzl1	myelin protein zero-like 1	-5.39	0.00	0.55	BRAF; MEK
1424202_at	Seh1l	SEH1-like (S. cerevisiae	-5.39	0.00	0.68	BRAF
1434682_at	Zfp770	zinc finger protein 770	-5.39	0.00	0.58	BRAF; MEK
1416128_at	Tuba1c	tubulin. alpha 1C	-5.39	0.00	0.64	BRAF; MEK
1423292_a_at	Prx	periaxin	-5.39	0.00	0.69	BRAF; MEK
1447877_x_at	Dnmt1	DNA methyltransferase (cytosine-5) 1	-5.39	0.00	0.31	BRAF; MEK
1424883_s_at	Sfrs7	splicing factor. arginine/serine-rich 7	-5.39	0.00	0.45	BRAF; MEK
1433879_a_at	Mapk1ip1l	mitogen-activated protein kinase 1 interacting protein 1-like	-5.40	0.00	0.74	BRAF
1426853_at	Set	SET translocation	-5.40	0.00	0.62	BRAF; MEK
1427017_at	Satb2	special AT-rich sequence binding protein 2	-5.40	0.00	0.62	BRAF; MEK
1436979_x_at	Rbm14	RNA binding motif protein 14	-5.40	0.00	0.56	BRAF; MEK
1423877_at	Chaf1b	chromatin assembly factor 1. subunit B (p60)	-5.40	0.00	0.36	BRAF; MEK
1437471_at	Lrrc45	leucine rich repeat containing 45	-5.41	0.00	0.67	BRAF; MEK
1437279_x_at	Sdc1	syndecan 1	-5.41	0.00	0.59	BRAF; MEK
1448192_s_at	Prps1	phosphoribosyl pyrophosphate synthetase 1	-5.41	0.00	0.57	; n.r.
1438546_x_at	Slc25a5	solute carrier family 25 (mitochondrial carrier. adenine nucleotide translocator). member 5	-5.41	0.00	0.71	BRAF
1436266_x_at	Cbx1	chromobox homolog 1 (Drosophila HP1 beta)	-5.41	0.00	0.64	BRAF; MEK
1434469_at	Otud4	OTU domain containing 4	-5.42	0.00	0.47	BRAF
1424928_at	2210018M11Rik	RIKEN cDNA 2210018M11 gene	-5.42	0.00	0.67	
1425350_a_at	Myef2	myelin basic protein expression factor 2. repressor	-5.42	0.00	0.68	BRAF; MEK
1417938_at	Rad51ap1	RAD51 associated protein 1	-5.42	0.00	0.32	BRAF; MEK
1428386_at	Acsl3	acyl-CoA synthetase long-chain family member 3	-5.42	0.00	0.49	BRAF; MEK
1425048_a_at	Hmgb1	high mobility group box 1	-5.42	0.00	0.57	BRAF; MEK
1452020_a_at	Siva1	SIVA1. apoptosis-inducing factor	-5.43	0.00	0.57	BRAF
1430811_a_at	Nuf2	NUF2. NDC80 kinetochore complex component. homolog (S. cerevisiae)	-5.43	0.00	0.28	BRAF; MEK
1416125_at	Fkbp5	FK506 binding protein 5	-5.43	0.00	0.32	BRAF; MEK
1426835_at	Metap1	methionyl aminopeptidase 1	-5.43	0.00	0.79	BRAF
1443832_s_at	Sdpr	serum deprivation response	-5.43	0.00	0.65	n.r. ; n.r.
1417511_at	Lyar	Ly1 antibody reactive clone	-5.43	0.00	0.47	BRAF; MEK

Probeset ID	*Symbol*	*Gene Name*	*d-value*	*q-value*	*R-fold*	*Expression reversed by*
1436174_at	Atad2	ATPase family. AAA domain containing 2	-5.43	0.00	0.30	BRAF; MEK
1426496_at	Wdr55	WD repeat domain 55	-5.43	0.00	0.64	BRAF
1418542_s_at	Cenpo	centromere protein O	-5.44	0.00	0.72	; MEK
1427382_a_at	Suv39h1	suppressor of variegation 3-9 homolog 1 (Drosophila)	-5.44	0.00	0.50	BRAF; MEK
1424724_a_at	D16Ertd472e	DNA segment. Chr 16. ERATO Doi 472. expressed	-5.44	0.00	0.47	; n.r.
1418275_a_at	Elf2	E74-like factor 2	-5.44	0.00	0.74	BRAF; MEK
1424452_at	Sltm	SAFB-like. transcription modulator	-5.44	0.00	0.72	; MEK
1435122_x_at	Dnmt1	DNA methyltransferase (cytosine-5) 1	-5.44	0.00	0.30	BRAF; MEK
1442148_at	Psip1	PC4 and SFRS1 interacting protein 1	-5.45	0.00	0.60	BRAF; MEK
1421321_a_at	Net1	neuroepithelial cell transforming gene 1	-5.45	0.00	0.37	BRAF; MEK
1418514_at	Mtf2	metal response element binding transcription factor 2	-5.45	0.00	0.61	BRAF; MEK
1426790_at	Ssrp1	structure specific recognition protein 1	-5.45	0.00	0.50	BRAF; MEK
1428892_at	Ppil1	peptidylprolyl isomerase (cyclophilin)-like 1	-5.45	0.00	0.56	BRAF; MEK
1456320_at	D1Ertd53e	DNA segment. Chr 1. ERATO Doi 53. expressed	-5.45	0.00	0.64	n.r. ;
1439463_x_at	2810416G20Rik	RIKEN cDNA 2810416G20 gene	-5.46	0.00	0.45	BRAF; MEK
1418121_at	Vrk3	vaccinia related kinase 3	-5.46	0.00	0.77	BRAF; MEK
1427707_a_at	Stil	Scl/Tal1 interrupting locus	-5.46	0.00	0.36	BRAF; MEK
1433408_a_at	Mcm10	minichromosome maintenance deficient 10 (S. cerevisiae)	-5.46	0.00	0.30	BRAF; MEK
1436490_x_at	Ran	RAN. member RAS oncogene family	-5.46	0.00	0.89	BRAF; MEK
1436248_at	NA	NA	-5.46	0.00	0.63	n.r. ; n.r.
1429491_s_at	Rif1	Rap1 interacting factor 1 homolog (yeast)	-5.46	0.00	0.31	BRAF; MEK
1435254_at	Plxnb1	plexin B1	-5.46	0.00	0.45	BRAF; MEK
1453198_at	Zfp422-rs1	zinc finger protein 422. related sequence 1	-5.47	0.00	0.59	n.r. ; MEK
1440050_at	Hbs1l	Hbs1-like (S. cerevisiae)	-5.47	0.00	0.67	BRAF; MEK
1427959_at	Abhd10	abhydrolase domain containing 10	-5.47	0.00	0.52	BRAF; MEK
1450983_at	Akap8	A kinase (PRKA) anchor protein 8	-5.47	0.00	0.48	BRAF; MEK
1460240_a_at	Hnrnpc	heterogeneous nuclear ribonucleoprotein C	-5.47	0.00	0.77	BRAF; MEK
1458373_at	Gen1	Gen homolog 1. endonuclease (Drosophila)	-5.47	0.00	0.59	BRAF; MEK
1417647_at	Snx5	sorting nexin 5	-5.48	0.00	0.73	BRAF; MEK
1436431_at	1700025G04Rik	RIKEN cDNA 1700025G04 gene	-5.48	0.00	0.51	BRAF; MEK
1428052_a_at	Zmym1	zinc finger. MYM domain containing 1	-5.48	0.00	0.55	BRAF; MEK
1450479_x_at	Ptpn12	protein tyrosine phosphatase. non-receptor type 12	-5.48	0.00	0.75	; MEK
1418971_x_at	Bcl10	B-cell leukemia/lymphoma 10	-5.48	0.00	0.73	
1460538_at	Cdh10	cadherin 10	-5.48	0.00	0.38	n.r. ; n.r.
1416751_a_at	Ddx20	DEAD (Asp-Glu-Ala-Asp) box polypeptide 20	-5.49	0.00	0.61	BRAF; MEK
1426725_s_at	Ets1	E26 avian leukemia oncogene 1. 5' domain	-5.49	0.00	0.51	BRAF; MEK
1452589_at	Ptk7	PTK7 protein tyrosine kinase 7	-5.49	0.00	0.44	BRAF; MEK
1438179_s_at	Elp2	elongation protein 2 homolog (S. cerevisiae)	-5.49	0.00	0.78	BRAF; MEK
1426533_at	Nol5a	nucleolar protein 5A	-5.49	0.00	0.47	BRAF; MEK
1425349_a_at	Myef2	myelin basic protein expression factor 2. repressor	-5.50	0.00	0.47	
1426789_s_at	Ssrp1	structure specific recognition protein 1	-5.50	0.00	0.55	BRAF; MEK
1442465_s_at	Strbp	spermatid perinuclear RNA binding protein	-5.50	0.00	0.61	; MEK
1420081_s_at	D2Ertd750e	DNA segment. Chr 2. ERATO Doi 750. expressed	-5.50	0.00	0.35	BRAF; MEK
1428291_at	Exosc8	exosome component 8	-5.50	0.00	0.46	BRAF; MEK
1438725_at	Med13	mediator complex subunit 13	-5.50	0.00	0.58	; MEK
1433575_at	Sox4	SRY-box containing gene 4	-5.51	0.00	0.56	BRAF; MEK
1417487_at	Fosl1	fos-like antigen 1	-5.51	0.00	0.28	BRAF; MEK
1448134_at	X99384	cDNA sequence X99384	-5.51	0.00	0.49	BRAF; MEK
1442058_s_at	Psmc3ip	proteasome (prosome. macropain) 26S subunit. ATPase 3. interacting protein	-5.52	0.00	0.34	BRAF; MEK
1452226_at	Rcc2	regulator of chromosome condensation 2	-5.52	0.00	0.43	BRAF; MEK
1433440_x_at	Uba2	ubiquitin-like modifier activating enzyme 2	-5.52	0.00	0.64	BRAF; MEK
1419838_s_at	Plk4	polo-like kinase 4 (Drosophila)	-5.52	0.00	0.28	BRAF; MEK
1429109_at	Msl2l1	male-specific lethal 2-like 1 (Drosophila)	-5.53	0.00	0.62	BRAF; MEK
1418516_at	Mtf2	metal response element binding transcription factor 2	-5.53	0.00	0.59	BRAF; MEK
1450173_at	Ripk2	receptor (TNFRSF)-interacting serine-threonine kinase 2	-5.53	0.00	0.53	n.r. ; n.r.

Probeset ID	Symbol	Gene Name	d-value	q-value	R-fold	Expression reversed by
1416483_at	Ttc3	tetratricopeptide repeat domain 3	-5.53	0.00	0.54	; MEK
1429053_at	1110012J17Rik	RIKEN cDNA 1110012J17 gene	-5.53	0.00	0.52	BRAF; MEK
1416544_at	Ezh2	enhancer of zeste homolog 2 (Drosophila)	-5.53	0.00	0.29	BRAF; MEK
1434695_at	Dtl	denticleless homolog (Drosophila)	-5.53	0.00	0.25	BRAF; MEK
1435597_at	Atad5	ATPase family. AAA domain containing 5	-5.53	0.00	0.29	BRAF; MEK
1455495_at	Abl2	v-abl Abelson murine leukemia viral oncogene homolog 2 (arg. Abelson-related gene)	-5.53	0.00	0.53	BRAF; MEK
1438852_x_at	Mcm6	minichromosome maintenance deficient 6 (MIS5 homolog. S. pombe) (S. cerevisiae)	-5.54	0.00	0.20	BRAF; MEK
1455983_at	Cdca2	cell division cycle associated 2	-5.54	0.00	0.42	BRAF; MEK
1416641_at	Lig1	ligase I. DNA. ATP-dependent	-5.54	0.00	0.31	BRAF; MEK
1428328_at	Nup50	nucleoporin 50	-5.54	0.00	0.70	BRAF; MEK
1455228_at	Whsc1	Wolf-Hirschhorn syndrome candidate 1 (human)	-5.54	0.00	0.58	BRAF; MEK
1427094_at	Pole2	polymerase (DNA directed). epsilon 2 (p59 subunit)	-5.54	0.00	0.26	BRAF; MEK
1452266_at	Las1l	LAS1-like (S. cerevisiae)	-5.54	0.00	0.60	BRAF; MEK
1438861_at	Bnc2	basonuclin 2	-5.54	0.00	0.38	BRAF; MEK
1426441_at	Slc11a2	solute carrier family 11 (proton-coupled divalent metal ion transporters). member 2	-5.55	0.00	0.68	BRAF; MEK
1450307_x_at	H2afy3	H2A histone family. member Y3	-5.55	0.00	0.79	BRAF; MEK
1419592_at	Unc5c	unc-5 homolog C (C. elegans)	-5.55	0.00	0.26	
1452422_a_at	Snrpb2	U2 small nuclear ribonucleoprotein B	-5.55	0.00	0.64	BRAF; MEK
1425178_s_at	Shmt1	serine hydroxymethyltransferase 1 (soluble)	-5.55	0.00	0.67	BRAF; MEK
1417587_at	Timeless	timeless homolog (Drosophila)	-5.55	0.00	0.32	BRAF; MEK
1428409_at	Nat13	N-acetyltransferase 13	-5.56	0.00	0.65	
1445684_s_at	Hdac2	histone deacetylase 2	-5.56	0.00	0.59	BRAF; MEK
1422535_at	Ccne2	cyclin E2	-5.56	0.00	0.31	BRAF; MEK
1421236_at	Ripk2	receptor (TNFRSF)-interacting serine-threonine kinase 2	-5.56	0.00	0.57	n.r. ;
1428452_at	2810025M15Rik	RIKEN cDNA 2810025M15 gene	-5.56	0.00	0.49	BRAF
1448777_at	Mcm2	minichromosome maintenance deficient 2 mitotin (S. cerevisiae)	-5.56	0.00	0.29	BRAF; MEK
1426817_at	Mki67	antigen identified by monoclonal antibody Ki 67	-5.56	0.00	0.20	BRAF; MEK
1451436_at	Sbno1	sno. strawberry notch homolog 1 (Drosophila)	-5.56	0.00	0.59	; MEK
1456377_x_at	Limd2	LIM domain containing 2	-5.56	0.00	0.42	BRAF; MEK
1435743_at	Klhl23	kelch-like 23 (Drosophila)	-5.57	0.00	0.40	BRAF; MEK
1448369_at	Pola2	polymerase (DNA directed). alpha 2	-5.57	0.00	0.53	BRAF; MEK
1458140_at	Slit2	slit homolog 2 (Drosophila)	-5.57	0.00	0.46	; MEK
1449965_at	Mcpt8	mast cell protease 8	-5.57	0.00	0.16	BRAF
1420093_s_at	Hnrpdl	heterogeneous nuclear ribonucleoprotein D-like	-5.57	0.00	0.62	BRAF; MEK
1436345_at	5730559C18Rik	RIKEN cDNA 5730559C18 gene	-5.57	0.00	0.26	BRAF; MEK
1428029_a_at	H2afv	H2A histone family. member V	-5.58	0.00	0.60	BRAF; MEK
1416303_at	Litaf	LPS-induced TN factor	-5.58	0.00	0.55	BRAF; MEK
1438750_at	Atrx	alpha thalassemia/mental retardation syndrome X-linked homolog (human)	-5.58	0.00	0.56	; MEK
1424321_at	Rfc4	replication factor C (activator 1) 4	-5.58	0.00	0.40	BRAF; MEK
1452073_at	6720460F02Rik	RIKEN cDNA 6720460F02 gene	-5.58	0.00	0.33	BRAF; MEK
1430617_at	Oip5	Opa interacting protein 5	-5.58	0.00	0.38	BRAF; MEK
1423382_a_at	Hnrnpf	heterogeneous nuclear ribonucleoprotein F	-5.58	0.00	0.57	BRAF
1427282_a_at	Fxn	frataxin	-5.58	0.00	0.53	BRAF; MEK
1424390_at	Nupl1	nucleoporin like 1	-5.58	0.00	0.70	
1418966_a_at	Dcbld1	discoidin. CUB and LCCL domain containing 1	-5.58	0.00	0.53	; n.r.
1417948_s_at	Ilf2	interleukin enhancer binding factor 2	-5.59	0.00	0.63	BRAF; MEK
1436511_at	BC031781	cDNA sequence BC031781	-5.59	0.00	0.81	; n.r.
1426825_at	Fmnl3	formin-like 3	-5.59	0.00	0.65	BRAF; MEK
1426402_at	Syncrip	synaptotagmin binding. cytoplasmic RNA interacting protein	-5.59	0.00	0.65	BRAF; MEK
1449877_s_at	LOC100044746	similar to Kifc1 protein	-5.59	0.00	0.47	BRAF; MEK
1431089_at	Cpsf2	cleavage and polyadenylation specific factor 2	-5.60	0.00	0.48	BRAF; MEK
1435004_at	Pank4	pantothenate kinase 4	-5.60	0.00	0.69	BRAF; MEK
1426411_a_at	Strbp	spermatid perinuclear RNA binding protein	-5.61	0.00	0.53	n.r. ; n.r.

Probeset ID	*Symbol*	*Gene Name*	*d-value*	*q-value*	*R-fold*	*Expression reversed by*
1437409_s_at	Gpr126	G protein-coupled receptor 126	-5.61	0.00	0.47	BRAF; MEK
1447275_at	Bbs12	Bardet-Biedl syndrome 12 (human)	-5.61	0.00	0.52	BRAF; MEK
1426751_s_at	Nup107	nucleoporin 107	-5.61	0.00	0.53	BRAF; MEK
1430019_a_at	Hnrnpa1	heterogeneous nuclear ribonucleoprotein A1	-5.61	0.00	0.43	BRAF; MEK
1420110_s_at	Zfp334	zinc finger protein 334	-5.61	0.00	0.71	BRAF; MEK
1428458_at	Pop1	processing of precursor 1. ribonuclease P/MRP family. (S. cerevisiae)	-5.61	0.00	0.53	BRAF; MEK
1417696_at	Soat1	sterol O-acyltransferase 1	-5.61	0.00	0.61	; MEK
1420979_at	Pak1	p21 (CDKN1A)-activated kinase 1	-5.61	0.00	0.42	BRAF; MEK
1455430_at	Prr8	proline rich 8	-5.62	0.00	0.73	BRAF; MEK
1437370_at	Sgol2	shugoshin-like 2 (S. pombe)	-5.62	0.00	0.28	BRAF; MEK
1421939_a_at	Stag1	stromal antigen 1	-5.62	0.00	0.65	BRAF; MEK
1428099_a_at	Sfrs1	splicing factor. arginine/serine-rich 1 (ASF/SF2)	-5.62	0.00	0.54	BRAF; MEK
1436874_x_at	Slc25a5	solute carrier family 25 (mitochondrial carrier. adenine nucleotide translocator). member 5	-5.62	0.00	0.70	BRAF; MEK
1435880_at	Ankrd50	ankrin repeat domain 50	-5.62	0.00	0.52	; MEK
1428527_at	Snx7	sorting nexin 7	-5.62	0.00	0.71	n.r. ; n.r.
1424873_at	Rnf2	ring finger protein 2	-5.62	0.00	0.71	; MEK
1428694_at	5033413D16Rik	RIKEN cDNA 5033413D16 gene	-5.63	0.00	0.30	BRAF; MEK
1428537_at	Csnk1a1	casein kinase 1. alpha 1	-5.63	0.00	0.74	; MEK
1416071_at	Ddx18	DEAD (Asp-Glu-Ala-Asp) box polypeptide 18	-5.63	0.00	0.71	BRAF; MEK
1417457_at	Cks2	CDC28 protein kinase regulatory subunit 2	-5.63	0.00	0.47	BRAF; MEK
1437404_at	Mast4	microtubule associated serine/threonine kinase family member 4	-5.64	0.00	0.28	BRAF; MEK
1434365_a_at	BC055324	cDNA sequence BC055324	-5.64	0.00	0.35	BRAF; MEK
1455398_at	Lrrc8c	leucine rich repeat containing 8 family. member C	-5.64	0.00	0.51	BRAF
1434317_s_at	Tex10	testis expressed gene 10	-5.64	0.00	0.65	BRAF; MEK
1459987_s_at	Cct3	chaperonin containing Tcp1. subunit 3 (gamma)	-5.65	0.00	0.74	BRAF; MEK
1424882_a_at	Nt5dc2	5'-nucleotidase domain containing 2	-5.65	0.00	0.41	BRAF; MEK
1435742_at	Smek1	SMEK homolog 1. suppressor of mek1 (Dictyostelium)	-5.66	0.00	0.61	BRAF; MEK
1450861_at	Fancc	Fanconi anemia. complementation group C	-5.66	0.00	0.66	BRAF
1424060_at	Neil3	nei like 3 (E. coli)	-5.66	0.00	0.64	BRAF; MEK
1453010_at	Iws1	IWS1 homolog (S. cerevisiae)	-5.66	0.00	0.61	BRAF; MEK
1456077_x_at	Cdc25c	cell division cycle 25 homolog C (S. pombe)	-5.66	0.00	0.41	BRAF; MEK
1426612_at	Tipin	timeless interacting protein	-5.66	0.00	0.49	BRAF; MEK
1428844_a_at	Bclaf1	BCL2-associated transcription factor 1	-5.66	0.00	0.53	; MEK
1437199_at	Dusp5	dual specificity phosphatase 5	-5.66	0.00	0.21	BRAF; MEK
1423735_a_at	Wdr36	WD repeat domain 36	-5.66	0.00	0.61	BRAF; MEK
1451986_s_at	Lrrk1	leucine-rich repeat kinase 1	-5.67	0.00	0.71	BRAF
1434495_at	Patz1	POZ (BTB) and AT hook containing zinc finger 1	-5.67	0.00	0.49	BRAF; MEK
1429658_a_at	Smc2	structural maintenance of chromosomes 2	-5.67	0.00	0.33	BRAF; MEK
1450842_a_at	Cenpa	centromere protein A	-5.67	0.00	0.31	BRAF; MEK
1437385_at	Ccbe1	collagen and calcium binding EGF domains 1	-5.67	0.00	0.29	; MEK
1424397_at	Dhx36	DEAH (Asp-Glu-Ala-His) box polypeptide 36	-5.67	0.00	0.76	n.r. ;
1428281_at	Trub1	TruB pseudouridine (psi) synthase homolog 1 (E. coli)	-5.67	0.00	0.71	BRAF; MEK
1437655_at	D430020J02Rik	RIKEN cDNA D430020J02 gene	-5.68	0.00	0.51	BRAF; MEK
1436910_at	Rasal2	RAS protein activator like 2	-5.68	0.00	0.59	n.r. ; n.r.
1455523_at	Cstf2	cleavage stimulation factor. 3' pre-RNA subunit 2	-5.69	0.00	0.64	BRAF; MEK
1418377_a_at	Siva1	SIVA1. apoptosis-inducing factor	-5.69	0.00	0.54	BRAF
1450157_a_at	Hmmr	hyaluronan mediated motility receptor (RHAMM)	-5.69	0.00	0.28	BRAF; MEK
1452305_s_at	Cenpn	centromere protein N	-5.70	0.00	0.39	BRAF; MEK
1422198_a_at	Shmt1	serine hydroxymethyltransferase 1 (soluble)	-5.70	0.00	0.42	BRAF; MEK
1453683_a_at	Cep55	centrosomal protein 55	-5.70	0.00	0.26	; n.r.
1454783_at	Il13ra1	interleukin 13 receptor. alpha 1	-5.70	0.00	0.34	BRAF; MEK
1417582_s_at	Dhodh	dihydroorotate dehydrogenase	-5.71	0.00	0.61	BRAF
1424489_a_at	Trit1	tRNA isopentenyltransferase 1	-5.71	0.00	0.73	; MEK
1421140_a_at	Foxp1	forkhead box P1	-5.71	0.00	0.64	n.r. ;
1437239_x_at	Phc2	polyhomeotic-like 2 (Drosophila)	-5.72	0.00	0.55	BRAF; MEK

Probeset ID	*Symbol*	*Gene Name*	*d-value*	*q-value*	*R-fold*	*Expression reversed by*
1424291_at	Nup93	nucleoporin 93	-5.72	0.00	0.68	BRAF; MEK
1459651_s_at	OTTMUSG00000026223	predicted gene. OTTMUSG00000026223	-5.72	0.00	0.68	BRAF; MEK
1450082_s_at	Etv5	ets variant gene 5	-5.72	0.00	0.49	BRAF; MEK
1421207_at	Lif	leukemia inhibitory factor	-5.72	0.00	0.50	BRAF; MEK
1454736_at	Ankrd57	ankyrin repeat domain 57	-5.72	0.00	0.67	BRAF; MEK
1420907_at	Cd2ap	CD2-associated protein	-5.72	0.00	0.64	; MEK
1455355_at	6030408C04Rik	RIKEN cDNA 6030408C04 gene	-5.72	0.00	0.36	BRAF; MEK
1427147_at	F730047E07Rik	RIKEN cDNA F730047E07 gene	-5.73	0.00	0.41	BRAF; MEK
1420877_at	Sept6	septin 6	-5.73	0.00	0.65	BRAF; MEK
1424440_at	Mrps6	mitochondrial ribosomal protein S6	-5.73	0.00	0.43	BRAF; MEK
1416135_at	Apex1	apurinic/apyrimidinic endonuclease 1	-5.74	0.00	0.65	BRAF; MEK
1459211_at	Gli2	GLI-Kruppel family member GLI2	-5.74	0.00	0.28	BRAF; MEK
1450938_at	Pnn	pinin	-5.74	0.00	0.64	BRAF; MEK
1426676_s_at	Tomm70a	translocase of outer mitochondrial membrane 70 homolog A (yeast)	-5.74	0.00	0.64	BRAF; MEK
1416757_at	Zwilch	Zwilch. kinetochore associated. homolog (Drosophila)	-5.75	0.00	0.33	BRAF; MEK
1428911_at	Ttll4	tubulin tyrosine ligase-like family. member 4	-5.75	0.00	0.41	BRAF; MEK
1426827_at	Ythdc1	YTH domain containing 1	-5.75	0.00	0.64	BRAF; MEK
1425126_at	Ncam1	neural cell adhesion molecule 1	-5.76	0.00	0.39	; MEK
1456227_x_at	Rbbp7	retinoblastoma binding protein 7	-5.76	0.00	0.65	BRAF; MEK
1429043_at	Smndc1	survival motor neuron domain containing 1	-5.76	0.00	0.71	BRAF; MEK
1451087_at	Wdr36	WD repeat domain 36	-5.77	0.00	0.64	BRAF; MEK
1450405_at	Mrpl19	mitochondrial ribosomal protein L19	-5.77	0.00	0.65	BRAF
1448519_at	Tead2	TEA domain family member 2	-5.77	0.00	0.40	BRAF; MEK
1452045_at	Zfp281	zinc finger protein 281	-5.77	0.00	0.62	n.r. ; MEK
1428213_at	Nsmce4a	non-SMC element 4 homolog A (S. cerevisiae)	-5.78	0.00	0.62	BRAF; MEK
1423318_at	Rad18	RAD18 homolog (S. cerevisiae)	-5.78	0.00	0.39	BRAF; MEK
1426675_at	Tomm70a	translocase of outer mitochondrial membrane 70 homolog A (yeast)	-5.78	0.00	0.67	BRAF; MEK
1438009_at	Hist1h2ae	histone cluster 1. H2ae	-5.78	0.00	0.27	BRAF; MEK
1424659_at	Slit2	slit homolog 2 (Drosophila)	-5.79	0.00	0.27	BRAF; MEK
1422946_a_at	Dnmt1	DNA methyltransferase (cytosine-5) 1	-5.79	0.00	0.30	BRAF; MEK
1430291_at	Dock5	dedicator of cytokinesis 5	-5.79	0.00	0.36	BRAF; MEK
1459470_at	NA	NA	-5.80	0.00	0.75	BRAF; MEK
1416915_at	Msh6	mutS homolog 6 (E. coli)	-5.80	0.00	0.28	BRAF; MEK
1423775_s_at	Prc1	protein regulator of cytokinesis 1	-5.81	0.00	0.30	BRAF; MEK
1434972_x_at	Sfrs1	splicing factor. arginine/serine-rich 1 (ASF/SF2)	-5.81	0.00	0.58	BRAF; MEK
1424682_at	Gpn3	GPN-loop GTPase 3	-5.81	0.00	0.68	BRAF; MEK
1434190_at	Sms	spermine synthase	-5.81	0.00	0.57	BRAF; MEK
1449705_x_at	Mcm3	minichromosome maintenance deficient 3 (S. cerevisiae)	-5.82	0.00	0.33	BRAF; MEK
1422045_a_at	Ptpn12	protein tyrosine phosphatase. non-receptor type 12	-5.82	0.00	0.69	; MEK
1422462_at	Ube2t	ubiquitin-conjugating enzyme E2T (putative)	-5.82	0.00	0.37	BRAF; MEK
1416868_at	Cdkn2c	cyclin-dependent kinase inhibitor 2C (p18. inhibits CDK4)	-5.82	0.00	0.56	BRAF; MEK
1424629_at	Brca1	breast cancer 1	-5.82	0.00	0.22	BRAF; MEK
1417681_at	Nudt21	nudix (nucleoside diphosphate linked moiety X)-type motif 21	-5.82	0.00	0.64	BRAF; MEK
1452499_a_at	Kif2a	kinesin family member 2A	-5.83	0.00	0.65	BRAF; MEK
1426774_at	Parp12	poly (ADP-ribose) polymerase family. member 12	-5.83	0.00	0.66	n.r. ; MEK
1424967_x_at	Tnnt2	troponin T2. cardiac	-5.83	0.00	0.18	BRAF; MEK
1422252_a_at	Cdc25c	cell division cycle 25 homolog C (S. pombe)	-5.83	0.00	0.52	BRAF; MEK
1443524_x_at	Bcl10	B-cell leukemia/lymphoma 10	-5.83	0.00	0.76	
1426836_s_at	Metap1	methionyl aminopeptidase 1	-5.83	0.00	0.73	BRAF; MEK
1440299_at	E330016A19Rik	RIKEN cDNA E330016A19 gene	-5.83	0.00	0.54	BRAF; MEK
1431087_at	Spc24	SPC24. NDC80 kinetochore complex component. homolog (S. cerevisiae)	-5.83	0.00	0.32	BRAF; MEK
1416120_at	Rrm2	ribonucleotide reductase M2	-5.84	0.00	0.28	BRAF
1429268_at	2610318N02Rik	RIKEN cDNA 2610318N02 gene	-5.84	0.00	0.50	BRAF; MEK
1426630_at	Exosc2	exosome component 2	-5.84	0.00	0.62	BRAF; MEK
1426477_at	Rasa1	RAS p21 protein activator 1	-5.84	0.00	0.56	

Probeset ID	Symbol	Gene Name	d-value	q-value	R-fold	Expression reversed by
1437658_a_at	Snord22	small nucleolar RNA. C/D box 22	-5.84	0.00	0.41	; MEK
1421529_a_at	Txnrd1	thioredoxin reductase 1	-5.84	0.00	0.63	BRAF; MEK
1445882_at	Cd300lb	CD300 antigen like family member B	-5.85	0.00	0.66	BRAF; MEK
1423577_at	Ankrd32	ankyrin repeat domain 32	-5.85	0.00	0.51	BRAF
1434949_at	Armc8	armadillo repeat containing 8	-5.85	0.00	0.55	BRAF; MEK
1450070_s_at	Pak1	p21 (CDKN1A)-activated kinase 1	-5.86	0.00	0.46	BRAF; MEK
1423349_at	Socs5	suppressor of cytokine signaling 5	-5.86	0.00	0.60	BRAF; MEK
1423795_at	Sfpq	splicing factor proline/glutamine rich (polypyrimidine tract binding protein associated)	-5.86	0.00	0.55	BRAF; MEK
1453183_at	1110034A24Rik	RIKEN cDNA 1110034A24 gene	-5.86	0.00	0.49	BRAF; MEK
1417125_at	Ahcy	S-adenosylhomocysteine hydrolase	-5.86	0.00	0.55	BRAF; MEK
1448627_s_at	Pbk	PDZ binding kinase	-5.86	0.00	0.28	BRAF; MEK
1450052_at	Kif2a	kinesin family member 2A	-5.86	0.00	0.56	BRAF; MEK
1418084_at	Nrp1	neuropilin 1	-5.86	0.00	0.29	BRAF; MEK
1429106_at	4921509J17Rik	RIKEN cDNA 4921509J17 gene	-5.87	0.00	0.37	BRAF; MEK
1422513_at	Ccnf	cyclin F	-5.87	0.00	0.26	BRAF; MEK
1453342_at	Cdc40	cell division cycle 40 homolog (yeast)	-5.87	0.00	0.72	BRAF; MEK
1454744_at	F630043A04Rik	RIKEN cDNA F630043A04 gene	-5.88	0.00	0.39	BRAF; MEK
1415698_at	Golm1	golgi membrane protein 1	-5.88	0.00	0.49	BRAF; MEK
1437611_x_at	Kif2c	kinesin family member 2C	-5.88	0.00	0.29	BRAF; MEK
1457687_at	Bcl2	B-cell leukemia/lymphoma 2	-5.89	0.00	0.50	BRAF; MEK
1455852_at	Nsl1	NSL1. MIND kinetochore complex component. homolog (S. cerevisiae)	-5.89	0.00	0.49	BRAF; MEK
1459861_s_at	OTTMUSG00000006609	predicted gene. OTTMUSG00000006609	-5.90	0.00	0.59	BRAF; MEK
1428410_at	Nat13	N-acetyltransferase 13	-5.90	0.00	0.66	n.r. ; MEK
1451417_at	Brca1	breast cancer 1	-5.90	0.00	0.38	BRAF; MEK
1416076_at	Ccnb1-rs1	cyclin B1. related sequence 1	-5.90	0.00	0.24	BRAF; MEK
1423543_at	Swap70	SWA-70 protein	-5.90	0.00	0.59	n.r. ; n.r.
1448899_s_at	Rad51ap1	RAD51 associated protein 1	-5.91	0.00	0.30	BRAF; MEK
1426349_s_at	Tmpo	thymopoietin	-5.91	0.00	0.32	BRAF; MEK
1448870_at	Ltbp1	latent transforming growth factor beta binding protein 1	-5.91	0.00	0.40	BRAF; MEK
1436922_at	Ppil5	peptidylprolyl isomerase (cyclophilin) like 5	-5.91	0.00	0.45	BRAF; MEK
1455832_a_at	Umps	uridine monophosphate synthetase	-5.91	0.00	0.54	BRAF; MEK
1423416_at	Smarcc1	SWI/SNF related. matrix associated. actin dependent regulator of chromatin. subfamily c. member 1	-5.92	0.00	0.52	BRAF; MEK
1430193_at	Casc5	cancer susceptibility candidate 5	-5.92	0.00	0.42	BRAF; MEK
1442289_at	Josd3	Josephin domain containing 3	-5.92	0.00	0.41	BRAF; MEK
1415811_at	Uhrf1	ubiquitin-like. containing PHD and RING finger domains. 1	-5.92	0.00	0.23	BRAF; MEK
1449207_a_at	Kif20a	kinesin family member 20A	-5.92	0.00	0.26	BRAF; MEK
1416336_s_at	Snrpd1	small nuclear ribonucleoprotein D1	-5.92	0.00	0.55	BRAF; MEK
1437033_a_at	Skp2	S-phase kinase-associated protein 2 (p45)	-5.92	0.00	0.37	BRAF; MEK
1423754_at	Ifitm3	interferon induced transmembrane protein 3	-5.92	0.00	0.38	; MEK
1455818_at	4930427A07Rik	RIKEN cDNA 4930427A07 gene	-5.92	0.00	0.53	BRAF; MEK
1417911_at	Ccna2	cyclin A2	-5.93	0.00	0.25	BRAF; MEK
1452040_a_at	Cdca3	cell division cycle associated 3	-5.93	0.00	0.31	BRAF; MEK
1449348_at	Mpp6	membrane protein. palmitoylated 6 (MAGUK p55 subfamily member 6)	-5.94	0.00	0.34	BRAF; MEK
1453581_at	Cep170	centrosomal protein 170	-5.95	0.00	0.62	; MEK
1416052_at	Prps1	phosphoribosyl pyrophosphate synthetase 1	-5.95	0.00	0.61	BRAF; MEK
1423722_at	Tmem49	transmembrane protein 49	-5.95	0.00	0.71	; MEK
1436308_at	Zfp292	zinc finger protein 292	-5.96	0.00	0.52	; MEK
1425092_at	Cdh10	cadherin 10	-5.96	0.00	0.27	n.r. ; n.r.
1436382_at	Zbtb12	zinc finger and BTB domain containing 12	-5.96	0.00	0.58	BRAF; MEK
1437580_s_at	Nek2	NIMA (never in mitosis gene a)-related expressed kinase 2	-5.96	0.00	0.31	BRAF; MEK
1433862_at	Espl1	extra spindle poles-like 1 (S. cerevisiae)	-5.96	0.00	0.25	BRAF; MEK
1449101_at	Ebf2	early B-cell factor 2	-5.97	0.00	0.79	; MEK
1422547_at	Ranbp1	RAN binding protein 1	-5.97	0.00	0.59	BRAF; MEK
1420998_at	Etv5	ets variant gene 5	-5.97	0.00	0.47	BRAF

Probeset ID	*Symbol*	*Gene Name*	*d-value*	*q-value*	*R-fold*	*Expression reversed by*
1428090_at	Ptcd3	pentatricopeptide repeat domain 3	-5.97	0.00	0.60	BRAF; MEK
1426410_at	Pdk3	pyruvate dehydrogenase kinase. isoenzyme 3	-5.98	0.00	0.66	; MEK
1418397_at	Zfp275	zinc finger protein 275	-5.98	0.00	0.76	BRAF; MEK
1417559_at	Sfxn1	sideroflexin 1	-5.98	0.00	0.67	BRAF; MEK
1422979_at	Suv39h2	suppressor of variegation 3-9 homolog 2 (Drosophila)	-5.99	0.00	0.33	BRAF; MEK
1417913_at	Gins4	GINS complex subunit 4 (Sld5 homolog)	-5.99	0.00	0.62	BRAF; MEK
1415872_at	Hnrnph1	heterogeneous nuclear ribonucleoprotein H1	-5.99	0.00	0.55	n.r. ; MEK
1418970_a_at	Bcl10	B-cell leukemia/lymphoma 10	-5.99	0.00	0.68	
1434424_at	Flvcr1	feline leukemia virus subgroup C cellular receptor 1	-5.99	0.00	0.51	BRAF; MEK
1433507_a_at	LOC100039489	similar to Hmgn2 protein	-5.99	0.00	0.58	BRAF; MEK
1426002_a_at	Cdc7	cell division cycle 7 (S. cerevisiae)	-5.99	0.00	0.28	BRAF; MEK
1433903_at	AU021838	expressed sequence AU021838	-6.00	0.00	0.51	BRAF; MEK
1454625_at	Phf6	PHD finger protein 6	-6.00	0.00	0.51	BRAF; MEK
1423051_at	Hnrnpu	heterogeneous nuclear ribonucleoprotein U	-6.00	0.00	0.52	BRAF; MEK
1452458_s_at	Ppil5	peptidylprolyl isomerase (cyclophilin) like 5	-6.01	0.00	0.27	BRAF; MEK
1415813_at	Api5	apoptosis inhibitor 5	-6.01	0.00	0.76	BRAF; MEK
1429172_a_at	Ncapg	on-SMC condensin I complex. subunit G	-6.02	0.00	0.26	BRAF; MEK
1439129_at	Dock5	dedicator of cytokinesis 5	-6.02	0.00	0.37	; MEK
1458447_at	Cenpf	centromere protein F	-6.02	0.00	0.65	
1425271_at	Psmc3ip	proteasome (prosome. macropain) 26S subunit. ATPase 3. interacting protein	-6.02	0.00	0.41	BRAF; MEK
1431358_at	4930547N16Rik	RIKEN cDNA 4930547N16 gene	-6.02	0.00	0.37	BRAF; MEK
1418919_at	Sgol1	shugoshin-like 1 (S. pombe)	-6.02	0.00	0.27	BRAF; MEK
1452954_at	Ube2c	ubiquitin-conjugating enzyme E2C	-6.02	0.00	0.34	BRAF; MEK
1417926_at	Ncapg2	non-SMC condensin II complex. subunit G2	-6.03	0.00	0.20	BRAF; MEK
1423850_at	Nsun2	NOL1/NOP2/Sun domain family member 2	-6.03	0.00	0.61	BRAF; MEK
1424118_a_at	Spc25	SPC25. NDC80 kinetochore complex component. homolog (S. cerevisiae)	-6.04	0.00	0.39	BRAF; MEK
1449708_s_at	Chek1	checkpoint kinase 1 homolog (S. pombe)	-6.04	0.00	0.30	BRAF; MEK
1438459_x_at	Sfpq	splicing factor proline/glutamine rich (polypyrimidine tract binding protein associated)	-6.04	0.00	0.50	BRAF; MEK
1419153_at	2810417H13Rik	RIKEN cDNA 2810417H13 gene	-6.04	0.00	0.23	BRAF; MEK
1423481_at	Riok2	RIO kinase 2 (yeast)	-6.04	0.00	0.64	
1450692_at	Kif4	kinesin family member 4	-6.05	0.00	0.32	BRAF; MEK
1450862_at	Rad54l	RAD54 like (S. cerevisiae)	-6.05	0.00	0.27	BRAF; MEK
1453562_a_at	Nmral1	NmrA-like family domain containing 1	-6.05	0.00	0.48	BRAF; MEK
1438811_at	Dlgap5	discs. large (Drosophila) homolog-associated protein 5	-6.05	0.00	0.53	BRAF; MEK
1429812_at	2610002D18Rik	RIKEN cDNA 2610002D18 gene	-6.05	0.00	0.43	BRAF; MEK
1422944_a_at	Diap3	diaphanous homolog 3 (Drosophila)	-6.06	0.00	0.30	BRAF; MEK
1417091_at	Chuk	conserved helix-loop-helix ubiquitous kinase	-6.06	0.00	0.74	n.r. ; n.r.
1448191_at	Plk1	polo-like kinase 1 (Drosophila)	-6.06	0.00	0.28	BRAF; MEK
1452983_at	Cep57	centrosomal protein 57	-6.07	0.00	0.65	; MEK
1435029_at	NA	NA	-6.07	0.00	0.49	n.r. ; n.r.
1434630_at	Ankrd28	ankyrin repeat domain 28	-6.07	0.00	0.46	BRAF; MEK
1418264_at	Cenpk	centromere protein K	-6.07	0.00	0.39	BRAF; MEK
1416176_at	Hmgb1	high mobility group box 1	-6.07	0.00	0.56	BRAF; MEK
1455049_at	Igsf3	immunoglobulin superfamily. member 3	-6.07	0.00	0.66	BRAF; MEK
1431506_s_at	Ppih	peptidyl prolyl isomerase H	-6.08	0.00	0.60	BRAF; MEK
1416730_at	Rcl1	RNA terminal phosphate cyclase-like 1	-6.08	0.00	0.62	BRAF; MEK
1455855_x_at	Agxt2l2	alanine-glyoxylate aminotransferase 2-like 2	-6.08	0.00	0.69	BRAF; MEK
1434892_x_at	Rbbp4	retinoblastoma binding protein 4	-6.08	0.00	0.64	BRAF; MEK
1452210_at	Dna2	DNA replication helicase 2 homolog (yeast)	-6.09	0.00	0.27	BRAF; MEK
1418665_at	Impa2	inositol (myo)-1(or 4)-monophosphatase 2	-6.09	0.00	0.59	BRAF; MEK
1424226_at	9030617O03Rik	RIKEN cDNA 9030617O03 gene	-6.09	0.00	0.70	BRAF; n.r.
1424511_at	Aurka	aurora kinase A	-6.09	0.00	0.32	BRAF; MEK
1415878_at	Rrm1	ribonucleotide reductase M1	-6.09	0.00	0.40	BRAF; MEK
1423311_s_at	Tpbg	trophoblast glycoprotein	-6.09	0.00	0.34	BRAF; MEK

Probeset ID	Symbol	Gene Name	d-value	q-value	R-fold	Expression reversed by
1418226_at	Orc2l	origin recognition complex. subunit 2-like (S. cerevisiae)	-6.10	0.00	0.63	
1432026_a_at	Herc5	hect domain and RLD 5	-6.10	0.00	0.52	; n.r.
1433909_at	Syt17	synaptotagmin XVII	-6.10	0.00	0.47	n.r. ; MEK
1451286_s_at	Fus	fusion. derived from t(12;16) malignant liposarcoma (human)	-6.10	0.00	0.43	BRAF; MEK
1425255_s_at	Hnrpll	heterogeneous nuclear ribonucleoprotein L-like	-6.11	0.00	0.58	BRAF; MEK
1423092_at	Incenp	inner centromere protein	-6.11	0.00	0.33	BRAF; MEK
1416998_at	Rrs1	RRS1 ribosome biogenesis regulator homolog (S. cerevisiae)	-6.11	0.00	0.57	BRAF; MEK
1426421_s_at	Rbm26	RNA binding motif protein 26	-6.11	0.00	0.76	n.r. ;
1436584_at	Spry2	sprouty homolog 2 (Drosophila)	-6.11	0.00	0.43	BRAF; MEK
1451649_a_at	Wdr75	WD repeat domain 75	-6.12	0.00	0.63	BRAF; MEK
1418305_s_at	Nola1	nucleolar protein family A. member 1 (H/ACA small nucleolar RNPs)	-6.12	0.00	0.53	BRAF; MEK
1451317_at	Ythdf2	YTH domain family 2	-6.12	0.00	0.69	BRAF; MEK
1448586_at	Hspa14	heat shock protein 14	-6.13	0.00	0.69	BRAF; MEK
1416410_at	Pafah1b3	platelet-activating factor acetylhydrolase. isoform 1b. alpha1 subunit	-6.14	0.00	0.36	BRAF; MEK
1430982_at	LOC100048559	similar to splicing factor. arginine/serine-rich 1 (splicing factor 2. alternate splicing factor)	-6.14	0.00	0.47	BRAF; MEK
1456280_at	Clspn	claspin homolog (Xenopus laevis)	-6.14	0.00	0.25	BRAF; MEK
1422865_at	Runx1	runt related transcription factor 1	-6.15	0.00	0.40	BRAF; MEK
1426788_a_at	Ssrp1	structure specific recognition protein 1	-6.15	0.00	0.54	BRAF; MEK
1451180_a_at	Nt5c3l	5'-nucleotidase. cytosolic III-like	-6.15	0.00	0.70	BRAF; MEK
1428518_at	Mlf1ip	myeloid leukemia factor 1 interacting protein	-6.15	0.00	0.36	BRAF; MEK
1455727_at	Zrsr2	zinc finger (CCCH type). RNA binding motif and serine/arginine rich 2	-6.16	0.00	0.64	BRAF; MEK
1452778_x_at	Nap1l1	nucleosome assembly protein 1-like 1	-6.16	0.00	0.65	BRAF; MEK
1417300_at	Smpdl3b	sphingomyelin phosphodiesterase. acid-like 3B	-6.16	0.00	0.63	BRAF; MEK
1451163_at	Tinf2	Terf1 (TRF1)-interacting nuclear factor 2	-6.17	0.00	0.49	BRAF; MEK
1453181_x_at	Plscr1	phospholipid scramblase 1	-6.17	0.00	0.30	BRAF; MEK
1415782_at	Sumo2	SMT3 suppressor of mif two 3 homolog 2 (yeast)	-6.17	0.00	0.77	BRAF; MEK
1435114_at	Wdhd1	WD repeat and HMG-box DNA binding protein 1	-6.17	0.00	0.32	BRAF; MEK
1430020_x_at	Hnrnpa1	heterogeneous nuclear ribonucleoprotein A1	-6.18	0.00	0.44	BRAF; MEK
1429558_a_at	Larp7	La ribonucleoprotein domain family. member 7	-6.18	0.00	0.56	BRAF; MEK
1439464_s_at	Tex10	testis expressed gene 10	-6.18	0.00	0.61	BRAF; MEK
1448944_at	Nrp1	neuropilin 1	-6.18	0.00	0.29	BRAF; MEK
1419471_a_at	Nudc	nuclear distribution gene C homolog (Aspergillus)	-6.18	0.00	0.60	BRAF; MEK
1434941_s_at	Esf1	ESF1. nucleolar pre-rRNA processing protein. homolog (S. cerevisiae)	-6.18	0.00	0.64	; MEK
1418969_at	Skp2	S-phase kinase-associated protein 2 (p45)	-6.19	0.00	0.39	BRAF; MEK
1428498_at	Rnf219	ring finger protein 219	-6.19	0.00	0.52	BRAF; MEK
1420707_a_at	Traip	TRAF-interacting protein	-6.19	0.00	0.42	BRAF; MEK
1416283_at	Gart	phosphoribosylglycinamide formyltransferase	-6.20	0.00	0.56	BRAF; MEK
1415914_at	Hnrnpab	heterogeneous nuclear ribonucleoprotein A/B	-6.20	0.00	0.64	BRAF; MEK
1452717_at	Slc25a24	solute carrier family 25 (mitochondrial carrier. phosphate carrier). member 24	-6.20	0.00	0.74	BRAF; MEK
1420365_a_at	Hnrnpa2b1	heterogeneous nuclear ribonucleoprotein A2/B1	-6.20	0.00	0.47	BRAF; MEK
1455035_s_at	Nol5a	nucleolar protein 5A	-6.21	0.00	0.45	BRAF; MEK
1418380_at	Terf1	telomeric repeat binding factor 1	-6.21	0.00	0.39	BRAF; MEK
1417450_a_at	Tacc3	transforming. acidic coiled-coil containing protein 3	-6.21	0.00	0.29	BRAF; MEK
1415730_at	5730453I16Rik	RIKEN cDNA 5730453I16 gene	-6.21	0.00	0.75	BRAF; MEK
1417260_at	U2af2	U2 small nuclear ribonucleoprotein auxiliary factor (U2AF) 2	-6.21	0.00	0.66	BRAF; MEK
1416988_at	Msh2	mutS homolog 2 (E. coli)	-6.22	0.00	0.35	BRAF; MEK
1448670_at	Ube2e3	ubiquitin-conjugating enzyme E2E 3. UBC4/5 homolog (yeast)	-6.22	0.00	0.57	BRAF; MEK
1452725_a_at	Rnaseh2a	ribonuclease H2. large subunit	-6.22	0.00	0.49	BRAF; MEK
1416592_at	Glrx	glutaredoxin	-6.22	0.00	0.48	
1417844_at	Med4	mediator of RNA polymerase II transcription. subunit 4 homolog (yeast)	-6.23	0.00	0.64	; MEK
1455218_at	6330503K22Rik	RIKEN cDNA 6330503K22 gene	-6.23	0.00	0.56	BRAF; MEK
1428869_at	Nolc1	nucleolar and coiled-body phosphoprotein 1	-6.23	0.00	0.47	BRAF; MEK
1425023_at	Usp3	ubiquitin specific peptidase 3	-6.24	0.00	0.67	BRAF; MEK
1421914_s_at	Mrpl19	mitochondrial ribosomal protein L19	-6.24	0.00	0.67	BRAF; MEK

Probeset ID	Symbol	Gene Name	d-value	q-value	R-fold	Expression reversed by
1437251_at	Cdca2	cell division cycle associated 2	-6.24	0.00	0.30	BRAF; MEK
1426864_a_at	Ncam1	neural cell adhesion molecule 1	-6.24	0.00	0.31	BRAF; MEK
1433946_at	Zik1	zinc finger protein interacting with K protein 1	-6.24	0.00	0.40	BRAF; MEK
1460669_at	Ilf3	interleukin enhancer binding factor 3	-6.24	0.00	0.64	BRAF; MEK
1450084_s_at	Ivns1abp	influenza virus NS1A binding protein	-6.25	0.00	0.42	; MEK
1426419_at	Rbm26	RNA binding motif protein 26	-6.26	0.00	0.52	; MEK
1448176_a_at	Hnrnpk	heterogeneous nuclear ribonucleoprotein K	-6.26	0.00	0.78	BRAF; MEK
1451080_at	Usp1	ubiquitin specific peptdiase 1	-6.28	0.00	0.38	BRAF; MEK
1430530_s_at	Nmral1	NmrA-like family domain containing 1	-6.28	0.00	0.39	BRAF; MEK
1423463_a_at	D2Ertd750e	DNA segment. Chr 2. ERATO Doi 750. expressed	-6.28	0.00	0.34	BRAF; MEK
1451069_at	Pim3	proviral integration site 3	-6.28	0.00	0.45	BRAF; MEK
1422509_at	U2af1	U2 small nuclear ribonucleoprotein auxiliary factor (U2AF) 1	-6.28	0.00	0.41	BRAF; MEK
1416873_a_at	Cdk2	cyclin-dependent kinase 2	-6.29	0.00	0.54 *	BRAF; MEK
1449094_at	Gjc1	gap junction protein. gamma 1	-6.29	0.00	0.43	BRAF; MEK
1417299_at	Nek2	NIMA (never in mitosis gene a)-related expressed kinase 2	-6.29	0.00	0.39	BRAF; MEK
1433766_at	C330023M02Rik	RIKEN cDNA C330023M02 gene	-6.30	0.00	0.66	BRAF; MEK
1422460_at	Mad2l1	MAD2 (mitotic arrest deficient. homolog)-like 1 (yeast)	-6.30	0.00	0.33	BRAF; MEK
1440344_at	NA	NA	-6.30	0.00	0.65	BRAF; MEK
1418114_at	Rbpj	recombination signal binding protein for immunoglobulin kappa J region	-6.30	0.00	0.51	BRAF; MEK
1416309_at	Nusap1	nucleolar and spindle associated protein 1	-6.31	0.00	0.34	BRAF; MEK
1429326_at	Cenpl	centromere protein L	-6.31	0.00	0.44	BRAF; MEK
1419644_at	Cstf2	cleavage stimulation factor. 3' pre-RNA subunit 2	-6.31	0.00	0.50	BRAF; MEK
1449491_at	Card10	caspase recruitment domain family. member 10	-6.31	0.00	0.41	BRAF; MEK
1423682_a_at	Cdca4	cell division cycle associated 4	-6.31	0.00	0.46	BRAF; MEK
1455834_x_at	Tacc3	transforming. acidic coiled-coil containing protein 3	-6.32	0.00	0.46	BRAF; MEK
1455529_at	Mex3a	mex3 homolog A (C. elegans)	-6.32	0.00	0.49	BRAF; MEK
1446196_at	Hmga2	high mobility group AT-hook 2	-6.32	0.00	0.28	BRAF; MEK
1434888_a_at	Matr3	matrin 3	-6.33	0.00	0.65	; MEK
1421612_a_at	H2afy2	H2A histone family. member Y2	-6.33	0.00	0.46	BRAF; MEK
1424877_a_at	Alad	aminolevulinate. delta-. dehydratase	-6.33	0.00	0.43	BRAF; MEK
1428908_at	Rbm25	RNA binding motif protein 25	-6.33	0.00	0.56	BRAF; MEK
1428480_at	Cdca8	cell division cycle associated 8	-6.34	0.00	0.34	BRAF; MEK
1451294_s_at	Snrpe	small nuclear ribonucleoprotein E	-6.34	0.00	0.68	BRAF; MEK
1428639_at	Lin9	lin-9 homolog (C. elegans)	-6.34	0.00	0.40	BRAF; MEK
1427983_at	Zfp280c	zinc finger protein 280c	-6.34	0.00	0.61	BRAF; MEK
1423774_a_at	Prc1	protein regulator of cytokinesis 1	-6.35	0.00	0.29	BRAF; MEK
1415839_a_at	Npm1	nucleophosmin 1	-6.35	0.00	0.77	; MEK
1435054_at	Eme1	essential meiotic endonuclease 1 homolog 1 (S. pombe)	-6.37	0.00	0.47	BRAF; MEK
1447363_s_at	Bub1b	budding uninhibited by benzimidazoles 1 homolog. beta (S. cerevisiae)	-6.37	0.00	0.22	BRAF; MEK
1420479_a_at	Nap1l1	nucleosome assembly protein 1-like 1	-6.37	0.00	0.61	
1450963_at	Hnrnpf	heterogeneous nuclear ribonucleoprotein F	-6.37	0.00	0.55	BRAF
1454955_at	Ipo7	importin 7	-6.38	0.00	0.61	n.r. ;
1448271_a_at	Ddx21	DEAD (Asp-Glu-Ala-Asp) box polypeptide 21	-6.38	0.00	0.49	BRAF
1434392_at	Usp34	ubiquitin specific peptidase 34	-6.38	0.00	0.71	; MEK
1417506_at	Gmnn	geminin	-6.38	0.00	0.28	BRAF; MEK
1437154_at	Cep170	centrosomal protein 170	-6.38	0.00	0.68	; MEK
1415849_s_at	Stmn1	stathmin 1	-6.39	0.00	0.30	BRAF; MEK
1424552_at	Casp8	caspase 8	-6.39	0.00	0.53	BRAF; MEK
1420616_at	Ash2l	ash2 (absent. small. or homeotic)-like (Drosophila)	-6.39	0.00	0.63	BRAF; MEK
1428104_at	Tpx2	TPX2. microtubule-associated protein homolog (Xenopus laevis)	-6.39	0.00	0.37	BRAF; MEK
1416684_at	Fbl	fibrillarin	-6.40	0.00	0.48	BRAF; MEK
1451137_a_at	Brd8	bromodomain containing 8	-6.40	0.00	0.54	BRAF; MEK
1448526_at	Kpnb1	karyopherin (importin) beta 1	-6.41	0.00	0.71	BRAF; MEK
1415973_at	Marcks	myristoylated alanine rich protein kinase C substrate	-6.41	0.00	0.58	BRAF; MEK
1428543_at	Ppat	phosphoribosyl pyrophosphate amidotransferase	-6.42	0.00	0.42	BRAF; MEK

Probeset ID	Symbol	Gene Name	d-value	q-value	R-fold	Expression reversed by
1415972_at	Marcks	myristoylated alanine rich protein kinase C substrate	-6.42	0.00	0.55	BRAF; MEK
1435005_at	Cenpe	centromere protein E	-6.42	0.00	0.26	BRAF; MEK
1453226_at	3000004C01Rik	RIKEN cDNA 3000004C01 gene	-6.42	0.00	0.31	BRAF; MEK
1428976_at	Tmpo	thymopoietin	-6.43	0.00	0.44	BRAF; MEK
1448465_at	Nipsnap1	4-nitrophenylphosphatase domain and non-neuronal SNAP25-like protein homolog 1 (C. elegans)	-6.43	0.00	0.63	BRAF; MEK
1435938_at	Ckap2l	cytoskeleton associated protein 2-like	-6.43	0.00	0.45	BRAF; MEK
1450496_a_at	2810433K01Rik	RIKEN cDNA 2810433K01 gene	-6.43	0.00	0.24	BRAF; MEK
1418334_at	Dbf4	DBF4 homolog (S. cerevisiae)	-6.43	0.00	0.35	BRAF; MEK
1455043_at	Tnpo1	transportin 1	-6.43	0.00	0.57	BRAF; n.r.
1416177_at	Rbmxrt	RNA binding motif protein. X chromosome retrogene	-6.44	0.00	0.53	BRAF; MEK
1417586_at	Timeless	timeless homolog (Drosophila)	-6.44	0.00	0.31	BRAF; MEK
1438221_at	D1Ertd53e	DNA segment. Chr 1. ERATO Doi 53. expressed	-6.44	0.00	0.55	n.r. ; MEK
1425483_at	Tox	thymocyte selection-associated high mobility group box	-6.44	0.00	0.30	
1436549_a_at	Hnrnpa1	heterogeneous nuclear ribonucleoprotein A1	-6.45	0.00	0.55	; MEK
1454814_s_at	AU021838	expressed sequence AU021838	-6.45	0.00	0.59	BRAF; MEK
1422540_at	Fbln1	fibulin 1	-6.46	0.00	0.48	BRAF; MEK
1424292_at	Depdc1a	DEP domain containing 1a	-6.46	0.00	0.22	BRAF; MEK
1419513_a_at	Ect2	ect2 oncogene	-6.46	0.00	0.24	BRAF; MEK
1417998_at	Ptges3	prostaglandin E synthase 3 (cytosolic)	-6.46	0.00	0.72	BRAF; MEK
1432538_a_at	Rfc3	replication factor C (activator 1) 3	-6.47	0.00	0.36	BRAF; MEK
1455305_x_at	Hnrnpa1	heterogeneous nuclear ribonucleoprotein A1	-6.47	0.00	0.54	; MEK
1436808_x_at	Mcm5	minichromosome maintenance deficient 5. cell division cycle 46 (S. cerevisiae)	-6.47	0.00	0.23	BRAF; MEK
1423772_x_at	Slc25a5	solute carrier family 25 (mitochondrial carrier. adenine nucleotide translocator). member 5	-6.47	0.00	0.71	BRAF; MEK
1417445_at	Ndc80	NDC80 homolog. kinetochore complex component (S. cerevisiae)	-6.47	0.00	0.27	BRAF; MEK
1433781_a_at	Cldn12	claudin 12	-6.47	0.00	0.48	BRAF; MEK
1416251_at	Mcm6	minichromosome maintenance deficient 6 (MIS5 homolog. S. pombe) (S. cerevisiae)	-6.47	0.00	0.24	BRAF; MEK
1421936_at	Dapp1	dual adaptor for phosphotyrosine and 3-phosphoinositides 1	-6.48	0.00	0.44	; MEK
1453002_at	Sox11	SRY-box containing gene 11	-6.48	0.00	0.45	BRAF; MEK
1456475_s_at	Prkar2b	protein kinase. cAMP dependent regulatory. type II beta	-6.49	0.00	0.19	BRAF; MEK
1434403_at	Spred2	sprouty-related. EVH1 domain containing 2	-6.49	0.00	0.47	BRAF; MEK
1419360_a_at	Ss18	synovial sarcoma translocation. Chromosome 18	-6.50	0.00	0.71	; MEK
1458440_at	Specc1	sperm antigen with calponin homology and coiled-coil domains 1	-6.50	0.00	0.50	
1437945_x_at	Nap1l1	nucleosome assembly protein 1-like 1	-6.51	0.00	0.68	BRAF; MEK
1426476_at	Rasa1	RAS p21 protein activator 1	-6.51	0.00	0.55	
1426386_at	Rpl7l1	ribosomal protein L7-like 1	-6.51	0.00	0.60	BRAF; MEK
1416068_at	Kars	lysyl-tRNA synthetase	-6.51	0.00	0.75	BRAF; MEK
1415945_at	Mcm5	minichromosome maintenance deficient 5. cell division cycle 46 (S. cerevisiae)	-6.51	0.00	0.21	BRAF; MEK
1449171_at	Ttk	Ttk protein kinase	-6.51	0.00	0.19	BRAF; MEK
1436953_at	Wipf1	WAS/WASL interacting protein family. member 1	-6.51	0.00	0.70	BRAF; MEK
1426351_at	Hspd1	heat shock protein 1 (chaperonin)	-6.52	0.00	0.63	; MEK
1455730_at	Dlgap5	discs. large (Drosophila) homolog-associated protein 5	-6.52	0.00	0.34	BRAF; MEK
1417262_at	Ptgs2	prostaglandin-endoperoxide synthase 2	-6.52	0.00	0.19	
1416969_at	Gtse1	G two S phase expressed protein 1	-6.53	0.00	0.51	BRAF
1428907_at	Rbm25	RNA binding motif protein 25	-6.53	0.00	0.60	BRAF; MEK
1438673_at	Slc4a7	solute carrier family 4. sodium bicarbonate cotransporter. member 7	-6.54	0.00	0.53	
1425416_s_at	Psrc1	proline/serine-rich coiled-coil 1	-6.54	0.00	0.54	BRAF; n.r.
1417458_s_at	Cks2	CDC28 protein kinase regulatory subunit 2	-6.54	0.00	0.43	BRAF; MEK
1428481_s_at	Cdca8	cell division cycle associated 8	-6.55	0.00	0.31	BRAF; MEK
1424991_s_at	Tyms	thymidylate synthase	-6.55	0.00	0.37	BRAF; MEK
1434541_x_at	Khdrbs1	KH domain containing. RNA binding. signal transduction associated 1	-6.55	0.00	0.65	BRAF; MEK
1434767_at	C79407	expressed sequence C79407	-6.55	0.00	0.26	BRAF; MEK
1417947_at	Pcna	proliferating cell nuclear antigen	-6.55	0.00	0.44 *	BRAF; MEK
1434789_at	Depdc1b	DEP domain containing 1B	-6.56	0.00	0.32	BRAF; MEK

Probeset ID	Symbol	Gene Name	d-value	q-value	R-fold		Expression reversed by
1452291_at	Centd1	centaurin. delta 1	-6.56	0.00	0.42		BRAF; MEK
1442595_at	NA	NA	-6.56	0.00	0.34		BRAF
1418480_at	Ppbp	pro-platelet basic protein	-6.56	0.00	0.08		; MEK
1416779_at	Sdpr	serum deprivation response	-6.56	0.00	0.70		n.r. ; n.r.
1418027_at	Exo1	exonuclease 1	-6.56	0.00	0.29		BRAF
1428593_at	1700029F09Rik	RIKEN cDNA 1700029F09 gene	-6.56	0.00	0.46		BRAF; MEK
1417910_at	Ccna2	cyclin A2	-6.57	0.00	0.26	*	BRAF; MEK
1449217_at	Casp8ap2	caspase 8 associated protein 2	-6.58	0.00	0.43		; MEK
1425837_a_at	Ccrn4l	CCR4 carbon catabolite repression 4-like (S. cerevisiae)	-6.58	0.00	0.53		; MEK
1452168_x_at	Gspt1	G1 to S phase transition 1	-6.59	0.00	0.75		BRAF; n.r.
1436847_s_at	Cdca8	cell division cycle associated 8	-6.61	0.00	0.29		BRAF; MEK
1420592_a_at	Anp32e	acidic (leucine-rich) nuclear phosphoprotein 32 family. member E	-6.61	0.00	0.46		BRAF; MEK
1429739_a_at	Patz1	POZ (BTB) and AT hook containing zinc finger 1	-6.62	0.00	0.49		BRAF; MEK
1429724_at	Prpf4	PRP4 pre-mRNA processing factor 4 homolog (yeast)	-6.62	0.00	0.64		BRAF; MEK
1450677_at	Chek1	checkpoint kinase 1 homolog (S. pombe)	-6.62	0.00	0.28		BRAF; MEK
1423127_at	Impa1	inositol (myo)-1(or 4)-monophosphatase 1	-6.62	0.00	0.78		BRAF; MEK
1438664_at	Prkar2b	protein kinase. cAMP dependent regulatory. type II beta	-6.62	0.00	0.20		BRAF; MEK
1426736_at	Gspt1	G1 to S phase transition 1	-6.63	0.00	0.76		BRAF; n.r.
1433685_a_at	6430706D22Rik	RIKEN cDNA 6430706D22 gene	-6.64	0.00	0.42		BRAF; MEK
1419058_at	Polr1e	polymerase (RNA) I polypeptide E	-6.64	0.00	0.62		BRAF; MEK
1422286_a_at	Tgif1	TGFB-induced factor homeobox 1	-6.64	0.00	0.46		BRAF; MEK
1418152_at	Nsbp1	nucleosome binding protein 1	-6.65	0.00	0.27		BRAF; MEK
1421052_a_at	Sms	spermine synthase	-6.65	0.00	0.37		BRAF; MEK
1436708_x_at	Mcm4	minichromosome maintenance deficient 4 homolog (S. cerevisiae)	-6.65	0.00	0.26		BRAF; MEK
1428232_at	Cpsf6	cleavage and polyadenylation specific factor 6	-6.67	0.00	0.68		BRAF
1424128_x_at	Aurkb	aurora kinase B	-6.67	0.00	0.23		BRAF; MEK
1416118_at	Trim59	tripartite motif-containing 59	-6.67	0.00	0.48		BRAF; MEK
1424205_at	Smarca5	SWI/SNF related. matrix associated. actin dependent regulator of chromatin. subfamily a. member 5	-6.67	0.00	0.57		BRAF; MEK
1452635_x_at	Josd3	Josephin domain containing 3	-6.67	0.00	0.48		BRAF; MEK
1452881_at	Gins2	GINS complex subunit 2 (Psf2 homolog)	-6.68	0.00	0.44		BRAF; MEK
1418540_a_at	Ptpre	protein tyrosine phosphatase. receptor type. E	-6.68	0.00	0.43		BRAF; MEK
1420478_at	Nap1l1	nucleosome assembly protein 1-like 1	-6.68	0.00	0.67		; MEK
1416030_a_at	Mcm7	minichromosome maintenance deficient 7 (S. cerevisiae)	-6.68	0.00	0.27		BRAF; MEK
1450048_a_at	Idh2	isocitrate dehydrogenase 2 (NADP+). mitochondrial	-6.69	0.00	0.33		BRAF; MEK
1419172_at	Dhfr	dihydrofolate reductase	-6.69	0.00	0.35		BRAF; MEK
1449060_at	Kif2c	kinesin family member 2C	-6.69	0.00	0.25		BRAF; MEK
1449585_at	Il1rap	interleukin 1 receptor accessory protein	-6.69	0.00	0.54		; MEK
1452189_at	Wdr82	WD repeat domain containing 82	-6.70	0.00	0.71		BRAF; MEK
1439269_x_at	Mcm7	minichromosome maintenance deficient 7 (S. cerevisiae)	-6.70	0.00	0.29		BRAF; MEK
1426652_at	Mcm3	minichromosome maintenance deficient 3 (S. cerevisiae)	-6.70	0.00	0.28		BRAF; MEK
1438458_a_at	Sfpq	splicing factor proline/glutamine rich (polypyrimidine tract binding protein associated)	-6.71	0.00	0.48		BRAF; MEK
1455038_at	ENSMUSG00000074630	predicted gene. ENSMUSG00000074630	-6.71	0.00	0.24		BRAF; MEK
1452659_at	Dek	DEK oncogene (DNA binding)	-6.71	0.00	0.43		BRAF; MEK
1427541_x_at	Hmmr	hyaluronan mediated motility receptor (RHAMM)	-6.71	0.00	0.32		BRAF; MEK
1425142_a_at	Hnrnpd	heterogeneous nuclear ribonucleoprotein D	-6.72	0.00	0.38		BRAF; MEK
1450156_a_at	Hmmr	hyaluronan mediated motility receptor (RHAMM)	-6.72	0.00	0.30		BRAF; MEK
1416939_at	Ppa1	pyrophosphatase (inorganic) 1	-6.72	0.00	0.63		BRAF; MEK
1416488_at	Ccng2	cyclin G2	-6.72	0.00	0.50		BRAF; MEK
1444257_at	Prr11	proline rich 11	-6.72	0.00	0.39		BRAF; MEK
1416031_s_at	Mcm7	minichromosome maintenance deficient 7 (S. cerevisiae)	-6.72	0.00	0.30		BRAF; MEK
1416212_at	Magoh	mago-nashi homolog. proliferation-associated (Drosophila)	-6.72	0.00	0.65		BRAF; MEK
1434788_at	D930050A07Rik	RIKEN cDNA D930050A07 gene	-6.72	0.00	0.48		BRAF; MEK
1451184_at	Hnrnpa3	heterogeneous nuclear ribonucleoprotein A3	-6.73	0.00	0.50		BRAF; MEK
1449010_at	Hspa4l	heat shock protein 4 like	-6.73	0.00	0.49		BRAF; MEK

Probeset ID	*Symbol*	*Gene Name*	*d-value*	*q-value*	*R-fold*	*Expression reversed by*
1448957_at	Rbpj	recombination signal binding protein for immunoglobulin kappa J region	-6.74	0.00	0.50	BRAF; MEK
1439093_at	NA	NA	-6.74	0.00	0.31	BRAF; MEK
1437910_at	Tmem39b	transmembrane protein 39b	-6.75	0.00	0.71	BRAF; MEK
1416685_s_at	Fbl	fibrillarin	-6.75	0.00	0.53	BRAF; MEK
1420476_a_at	Nap1l1	nucleosome assembly protein 1-like 1	-6.75	0.00	0.57	BRAF; MEK
1429734_at	4632434I11Rik	RIKEN cDNA 4632434I11 gene	-6.75	0.00	0.36	BRAF; n.r.
1416961_at	Bub1b	budding uninhibited by benzimidazoles 1 homolog. beta (S. cerevisiae)	-6.76	0.00	0.25	BRAF; MEK
1447547_at	Ltbp1	latent transforming growth factor beta binding protein 1	-6.76	0.00	0.41	; MEK
1421731_a_at	Fen1	flap structure specific endonuclease 1	-6.77	0.00	0.40	BRAF; MEK
1419184_a_at	Fhl2	four and a half LIM domains 2	-6.77	0.00	0.49	BRAF; MEK
1450781_at	Hmga2	high mobility group AT-hook 2	-6.77	0.00	0.23	BRAF; MEK
1449298_a_at	Pde1a	phosphodiesterase 1A. calmodulin-dependent	-6.77	0.00	0.20	BRAF; MEK
1432187_at	Nup43	nucleoporin 43	-6.77	0.00	0.52	BRAF; MEK
1437100_x_at	Pim3	proviral integration site 3	-6.78	0.00	0.48	BRAF; MEK
1428233_at	Cpsf6	cleavage and polyadenylation specific factor 6	-6.78	0.00	0.54	BRAF; MEK
1429478_at	6720463M24Rik	RIKEN cDNA 6720463M24 gene	-6.78	0.00	0.28	BRAF; MEK
1419943_s_at	Ccnb1	cyclin B1	-6.78	0.00	0.29 *	BRAF; MEK
1451928_a_at	Rad18	RAD18 homolog (S. cerevisiae)	-6.78	0.00	0.35	BRAF
1424630_a_at	Brca1	breast cancer 1	-6.79	0.00	0.32	BRAF; MEK
1418026_at	Exo1	exonuclease 1	-6.79	0.00	0.22	BRAF; MEK
1454694_a_at	Top2a	topoisomerase (DNA) II alpha	-6.79	0.00	0.22	BRAF; MEK
1438015_at	Dkc1	dyskeratosis congenita 1. dyskerin homolog (human)	-6.80	0.00	0.39	BRAF; MEK
1419270_a_at	Dut	deoxyuridine triphosphatase	-6.80	0.00	0.32	BRAF; MEK
1424942_a_at	Myc	myelocytomatosis oncogene	-6.80	0.00	0.41 *	BRAF; MEK
1422814_at	Aspm	asp (abnormal spindle)-like. microcephaly associated (Drosophila)	-6.80	0.00	0.23	BRAF; MEK
1452241_at	Topbp1	topoisomerase (DNA) II binding protein 1	-6.81	0.00	0.39	BRAF; MEK
1421297_a_at	Cacna1c	calcium channel. voltage-dependent. L type. alpha 1C subunit	-6.82	0.00	0.43	BRAF; MEK
1417719_at	Sap30	sin3 associated polypeptide	-6.84	0.00	0.38	BRAF; MEK
1429171_a_at	Ncapg	on-SMC condensin I complex. subunit G	-6.84	0.00	0.19	BRAF; MEK
1437643_at	Cenpj	centromere protein J	-6.84	0.00	0.34	BRAF; MEK
1451021_a_at	Klf5	Kruppel-like factor 5	-6.86	0.00	0.21	BRAF; MEK
1459679_s_at	Myo1b	myosin IB	-6.86	0.00	0.33	BRAF; MEK
1456523_at	100039239	predicted gene. 100039239	-6.86	0.00	0.27	BRAF
1426865_a_at	Ncam1	neural cell adhesion molecule 1	-6.86	0.00	0.35	BRAF; MEK
1452947_at	Gprc5c	G protein-coupled receptor. family C. group 5. member C	-6.87	0.00	0.46	
1424046_at	Bub1	budding uninhibited by benzimidazoles 1 homolog (S. cerevisiae)	-6.87	0.00	0.21	BRAF; MEK
1416575_at	Cdc45l	cell division cycle 45 homolog (S. cerevisiae)-like	-6.88	0.00	0.34	BRAF; MEK
1416043_at	Nasp	nuclear autoantigenic sperm protein (histone-binding)	-6.90	0.00	0.30	
1451411_at	Gprc5b	G protein-coupled receptor. family C. group 5. member B	-6.91	0.00	0.62	BRAF; MEK
1427105_at	Cenpn	centromere protein N	-6.91	0.00	0.53	BRAF; MEK
1422864_at	Runx1	runt related transcription factor 1	-6.91	0.00	0.46	BRAF; MEK
1434748_at	Ckap2	cytoskeleton associated protein 2	-6.91	0.00	0.33	BRAF; MEK
1453314_x_at	2610039C10Rik	RIKEN cDNA 2610039C10 gene	-6.93	0.00	0.49	BRAF; MEK
1437186_at	BC055324	cDNA sequence BC055324	-6.93	0.00	0.38	BRAF; MEK
1438320_s_at	Mcm7	minichromosome maintenance deficient 7 (S. cerevisiae)	-6.94	0.00	0.26	BRAF; MEK
1432188_s_at	Nup43	nucleoporin 43	-6.94	0.00	0.51	BRAF; MEK
1422482_at	Ruvbl2	RuvB-like protein 2	-6.94	0.00	0.53	BRAF; MEK
1423312_at	Tpbg	trophoblast glycoprotein	-6.95	0.00	0.51	BRAF; MEK
1416664_at	Cdc20	cell division cycle 20 homolog (S. cerevisiae)	-6.95	0.00	0.28	BRAF; MEK
1448113_at	Stmn1	stathmin 1	-6.95	0.00	0.35	BRAF; MEK
1443381_at	Etv4	ets variant gene 4 (E1A enhancer binding protein. E1AF)	-6.95	0.00	0.63	BRAF; MEK
1423700_at	Rfc3	replication factor C (activator 1) 3	-6.95	0.00	0.39	BRAF; MEK
1433782_at	Cldn12	claudin 12	-6.96	0.00	0.49	BRAF; MEK
1424436_at	Gart	phosphoribosylglycinamide formyltransferase	-6.96	0.00	0.51	BRAF; MEK
1455304_at	Unc13c	unc-13 homolog C (C. elegans)	-6.96	0.00	0.32	BRAF; MEK

Probeset ID	Symbol	Gene Name	d-value	q-value	R-fold	Expression reversed by
1424971_at	Ccdc99	coiled-coil domain containing 99	-6.96	0.00	0.29	BRAF; MEK
1433663_s_at	AU014645	expressed sequence AU014645	-6.97	0.00	0.70	BRAF; MEK
1423813_at	Kif22	kinesin family member 22	-6.97	0.00	0.29	BRAF; MEK
1427269_at	Sfrs11	splicing factor. arginine/serine-rich 11	-6.97	0.00	0.58	BRAF; MEK
1423477_at	Zic1	zinc finger protein of the cerebellum 1	-6.98	0.00	0.55	BRAF; MEK
1416065_a_at	Ankrd10	ankyrin repeat domain 10	-6.99	0.00	0.51	BRAF; MEK
1423884_at	Cirh1a	cirrhosis. autosomal recessive 1A (human)	-6.99	0.00	0.73	BRAF; MEK
1417939_at	Rad51ap1	RAD51 associated protein 1	-6.99	0.00	0.37	BRAF
1420028_s_at	Mcm3	minichromosome maintenance deficient 3 (S. cerevisiae)	-6.99	0.00	0.27	BRAF; MEK
1450129_a_at	Socs6	suppressor of cytokine signaling 6	-7.00	0.00	0.66	BRAF; MEK
1427161_at	Cenpf	centromere protein F	-7.00	0.00	0.22	BRAF; MEK
1418827_at	Thex1	three prime histone mRNA exonuclease 1	-7.00	0.00	0.49	BRAF; MEK
1417930_at	Nab2	Ngfi-A binding protein 2	-7.01	0.00	0.45	BRAF; MEK
1434400_at	Tgif2	TGFB-induced factor homeobox 2	-7.02	0.00	0.44	BRAF; MEK
1416288_at	Dnaja1	DnaJ (Hsp40) homolog. subfamily A. member 1	-7.02	0.00	0.64	; MEK
1452314_at	Kif11	kinesin family member 11	-7.03	0.00	0.23	BRAF; MEK
1429294_at	Trip13	thyroid hormone receptor interactor 13	-7.05	0.00	0.29	BRAF; MEK
1423920_at	Ncaph	non-SMC condensin I complex. subunit H	-7.05	0.00	0.35	BRAF; MEK
1428968_at	Cep57	centrosomal protein 57	-7.06	0.00	0.64	BRAF; MEK
1437716_x_at	Kif22	kinesin family member 22	-7.07	0.00	0.33	BRAF; MEK
1455682_at	Abl2	v-abl Abelson murine leukemia viral oncogene homolog 2 (arg. Abelson-related gene)	-7.07	0.00	0.48	BRAF; MEK
1418369_at	Prim1	DNA primase. p49 subunit	-7.09	0.00	0.27	BRAF; MEK
1457044_at	4732474O15Rik	RIKEN cDNA 4732474O15 gene	-7.09	0.00	0.41	
1436942_at	A930035D04Rik	RIKEN cDNA A930035D04 gene	-7.10	0.00	0.47	BRAF; MEK
1427321_s_at	Cxadr	coxsackievirus and adenovirus receptor	-7.10	0.00	0.42	BRAF; MEK
1416258_at	Tk1	thymidine kinase 1	-7.10	0.00	0.23	BRAF; MEK
1448635_at	Smc2	structural maintenance of chromosomes 2	-7.11	0.00	0.24	BRAF; MEK
1432013_a_at	2610016C23Rik	RIKEN cDNA 2610016C23 gene	-7.11	0.00	0.47	BRAF; MEK
1415857_at	Emb	embigin	-7.12	0.00	0.28	n.r. ;
1433696_at	Hn1l	hematological and neurological expressed 1-like	-7.12	0.00	0.46	BRAF; MEK
1430139_at	Hells	helicase. lymphoid specific	-7.13	0.00	0.48	BRAF; MEK
1428069_at	Cdca7	cell division cycle associated 7	-7.13	0.00	0.27	BRAF; MEK
1451770_s_at	Dhx9	DEAH (Asp-Glu-Ala-His) box polypeptide 9	-7.14	0.00	0.58	BRAF; MEK
1452240_at	Brunol4	bruno-like 4. RNA binding protein (Drosophila)	-7.15	0.00	0.45	BRAF; MEK
1428713_s_at	Gins2	GINS complex subunit 2 (Psf2 homolog)	-7.16	0.00	0.49	BRAF; MEK
1428304_at	Esco2	establishment of cohesion 1 homolog 2 (S. cerevisiae)	-7.17	0.00	0.24	BRAF; MEK
1450986_at	Nol5	nucleolar protein 5	-7.17	0.00	0.36	BRAF; MEK
1417019_a_at	Cdc6	cell division cycle 6 homolog (S. cerevisiae)	-7.17	0.00	0.20	BRAF; MEK
1452151_at	Gramd4	GRAM domain containing 4	-7.17	0.00	0.51	BRAF; MEK
1454011_a_at	Rpa2	replication protein A2	-7.17	0.00	0.42	BRAF; MEK
1440831_at	Bach1	BTB and CNC homology 1	-7.17	0.00	0.70	; MEK
1453490_at	Sass6	spindle assembly 6 homolog (C. elegans)	-7.18	0.00	0.60	BRAF; MEK
1453064_at	Etaa1	Ewing's tumor-associated antigen 1	-7.18	0.00	0.37	BRAF; MEK
1416073_a_at	Pcnt	pericentrin (kendrin)	-7.18	0.00	0.54	BRAF; MEK
1457404_at	Nfkbiz	nuclear factor of kappa light polypeptide gene enhancer in B-cells inhibitor. zeta	-7.18	0.00	0.24	BRAF; MEK
1426471_at	Zfp52	zinc finger protein 52	-7.19	0.00	0.41	; MEK
1433751_at	Slc39a10	solute carrier family 39 (zinc transporter). member 10	-7.19	0.00	0.40	BRAF; MEK
1425179_at	Shmt1	serine hydroxymethyltransferase 1 (soluble)	-7.20	0.00	0.33	BRAF; MEK
1434225_at	Swap70	SWA-70 protein	-7.20	0.00	0.61	
1427911_at	Tmem173	transmembrane protein 173	-7.21	0.00	0.51	BRAF; MEK
1422935_x_at	Cbx1	chromobox homolog 1 (Drosophila HP1 beta)	-7.21	0.00	0.62	BRAF; MEK
1423531_a_at	Hnrnpa1	heterogeneous nuclear ribonucleoprotein A1	-7.21	0.00	0.34	BRAF; MEK
1452036_a_at	Tmpo	thymopoietin	-7.22	0.00	0.29	BRAF; MEK
1419574_at	Zfp292	zinc finger protein 292	-7.24	0.00	0.57	; MEK
1459546_s_at	Enpp1	ectonucleotide pyrophosphatase/phosphodiesterase 1	-7.24	0.00	0.63	BRAF; MEK

Probeset ID	*Symbol*	*Gene Name*	*d-value*	*q-value*	*R-fold*	*Expression reversed by*
1441997_at	Zfp184	zinc finger protein 184 (Kruppel-like)	-7.24	0.00	0.56	BRAF; MEK
1451128_s_at	Kif22	kinesin family member 22	-7.24	0.00	0.26	BRAF; MEK
1456326_at	Gm784	gene model 784. (NCBI)	-7.24	0.00	0.25	BRAF; MEK
1431921_a_at	Stag1	stromal antigen 1	-7.25	0.00	0.61	BRAF; MEK
1449061_a_at	Prim1	DNA primase. p49 subunit	-7.25	0.00	0.25	BRAF; MEK
1425815_a_at	Hmmr	hyaluronan mediated motility receptor (RHAMM)	-7.26	0.00	0.31	BRAF; MEK
1455488_at	6230416J20Rik	RIKEN cDNA 6230416J20 gene	-7.26	0.00	0.31	BRAF; MEK
1448943_at	Nrp1	neuropilin 1	-7.26	0.00	0.32	BRAF; MEK
1436309_at	Neto2	neuropilin (NRP) and tolloid (TLL)-like 2	-7.26	0.00	0.39	BRAF; MEK
1423675_at	Usp1	ubiquitin specific peptdiase 1	-7.28	0.00	0.37	BRAF; MEK
1417454_at	Cul4b	cullin 4B	-7.31	0.00	0.58	; MEK
1458385_at	Hspa4l	heat shock protein 4 like	-7.31	0.00	0.43	; MEK
1453722_s_at	LOC100048559	similar to splicing factor. arginine/serine-rich 1 (splicing factor 2. alternate splicing factor)	-7.31	0.00	0.50	BRAF; MEK
1424278_a_at	Birc5	baculoviral IAP repeat-containing 5	-7.32	0.00	0.25	BRAF; MEK
1448205_at	Ccnb1-rs1	cyclin B1. related sequence 1	-7.33	0.00	0.29	BRAF; MEK
1439394_x_at	Cdc20	cell division cycle 20 homolog (S. cerevisiae)	-7.33	0.00	0.42	BRAF; MEK
1440227_at	Slc5a3	solute carrier family 5 (inositol transporters). member 3	-7.33	0.00	0.31	BRAF; MEK
1460353_at	Tmem48	transmembrane protein 48	-7.33	0.00	0.59	BRAF; MEK
1421819_a_at	Set	SET translocation	-7.34	0.00	0.44	BRAF; MEK
1427682_a_at	Egr2	early growth response 2	-7.34	0.00	0.22	BRAF; MEK
1434607_at	Ddx52	DEAD (Asp-Glu-Ala-Asp) box polypeptide 52	-7.34	0.00	0.67	BRAF; MEK
1429295_s_at	Trip13	thyroid hormone receptor interactor 13	-7.35	0.00	0.23	BRAF; MEK
1450780_s_at	Hmga2	high mobility group AT-hook 2	-7.35	0.00	0.31	BRAF; MEK
1427971_at	Cdc73	cell division cycle 73. Paf1/RNA polymerase II complex component. homolog (S. cerevisiae)	-7.36	0.00	0.70	
1426854_a_at	LOC100047898	similar to protein phosphatase 2A inhibitor-2 I-2PP2A	-7.36	0.00	0.57	BRAF; MEK
1436512_at	Arl4c	ADP-ribosylation factor-like 4C	-7.36	0.00	0.32	BRAF; MEK
1436707_x_at	Ncaph	non-SMC condensin I complex. subunit H	-7.37	0.00	0.32	BRAF; MEK
1416558_at	Melk	maternal embryonic leucine zipper kinase	-7.38	0.00	0.23	BRAF; MEK
1422673_at	Prkd1	protein kinase D1	-7.38	0.00	0.52	BRAF; MEK
1418628_at	Khdrbs1	KH domain containing. RNA binding. signal transduction associated 1	-7.40	0.00	0.58	BRAF; MEK
1434437_x_at	Rrm2	ribonucleotide reductase M2	-7.44	0.00	0.29	BRAF; MEK
1451246_s_at	Aurkb	aurora kinase B	-7.44	0.00	0.28	BRAF; MEK
1415850_at	Rasa3	RAS p21 protein activator 3	-7.45	0.00	0.35	BRAF; MEK
1423310_at	Tpbg	trophoblast glycoprotein	-7.45	0.00	0.32	BRAF; MEK
1429227_x_at	Nap1l1	nucleosome assembly protein 1-like 1	-7.45	0.00	0.60	BRAF; MEK
1437287_at	1110020G09Rik	RIKEN cDNA 1110020G09 gene	-7.45	0.00	0.49	BRAF; MEK
1428061_at	Hat1	histone aminotransferase 1	-7.46	0.00	0.36	BRAF; MEK
1418539_a_at	Ptpre	protein tyrosine phosphatase. receptor type. E	-7.46	0.00	0.39	BRAF; MEK
1434468_at	Otud4	OTU domain containing 4	-7.47	0.00	0.47	BRAF; MEK
1448989_a_at	Myo1b	myosin IB	-7.48	0.00	0.41	BRAF; MEK
1452598_at	Gins1	GINS complex subunit 1 (Psf1 homolog)	-7.48	0.00	0.27	BRAF; MEK
1429372_at	Sox11	SRY-box containing gene 11	-7.49	0.00	0.37	; MEK
1429527_a_at	Plscr1	phospholipid scramblase 1	-7.49	0.00	0.29	BRAF; MEK
1416214_at	Mcm4	minichromosome maintenance deficient 4 homolog (S. cerevisiae)	-7.49	0.00	0.28	BRAF; MEK
1442095_at	Asxl3	additional sex combs like 3 (Drosophila)	-7.50	0.00	0.38	BRAF; MEK
1449699_s_at	C330027C09Rik	RIKEN cDNA C330027C09 gene	-7.51	0.00	0.24	BRAF; MEK
1416698_a_at	Cks1b	CDC28 protein kinase 1b	-7.52	0.00	0.55	BRAF; MEK
1417821_at	D17H6S56E-5	DNA segment. Chr 17. human D6S56E 5	-7.53	0.00	0.20	BRAF; MEK
1426698_a_at	Hnrnpm	heterogeneous nuclear ribonucleoprotein M	-7.54	0.00	0.48	BRAF; MEK
1424113_at	Lamb1-1	laminin B1 subunit 1	-7.54	0.00	0.34	BRAF; MEK
1428176_at	S1pr2	sphingosine-1-phosphate receptor 2	-7.54	0.00	0.52	BRAF; MEK
1448931_at	F2rl1	coagulation factor II (thrombin) receptor-like 1	-7.54	0.00	0.31	BRAF; MEK
1422851_at	Hmga2	high mobility group AT-hook 2	-7.55	0.00	0.29	BRAF; MEK
1425718_a_at	Ivns1abp	influenza virus NS1A binding protein	-7.55	0.00	0.41	; MEK
1415971_at	Marcks	myristoylated alanine rich protein kinase C substrate	-7.57	0.00	0.58	BRAF; MEK

Probeset ID	Symbol	Gene Name	d-value	q-value	R-fold	Expression reversed by
1450004_at	Tslp	thymic stromal lymphopoietin	-7.58	0.00	0.29	; MEK
1435892_at	Asxl3	additional sex combs like 3 (Drosophila)	-7.59	0.00	0.43	BRAF; MEK
1429304_at	Ankrd10	ankyrin repeat domain 10	-7.59	0.00	0.53	BRAF; MEK
1426994_at	Phlpp	PH domain and leucine rich repeat protein phosphatase	-7.59	0.00	0.48	BRAF
1453106_a_at	Rnmt	RNA (guanine-7-) methyltransferase	-7.59	0.00	0.68	BRAF; MEK
1428262_s_at	Hnrnpa3	heterogeneous nuclear ribonucleoprotein A3	-7.60	0.00	0.63	; MEK
1418828_at	Thex1	three prime histone mRNA exonuclease 1	-7.60	0.00	0.37	BRAF; MEK
1455105_at	Ptpn12	protein tyrosine phosphatase. non-receptor type 12	-7.61	0.00	0.66	; MEK
1425241_a_at	Wsb1	WD repeat and SOCS box-containing 1	-7.61	0.00	0.53	BRAF; MEK
1426377_at	Zfp281	zinc finger protein 281	-7.63	0.00	0.57	; MEK
1424206_at	Smarca5	SWI/SNF related. matrix associated. actin dependent regulator of chromatin. subfamily a. member 5	-7.63	0.00	0.52	BRAF; MEK
1441788_s_at	Dkc1	dyskeratosis congenita 1. dyskerin homolog (human)	-7.63	0.00	0.29	BRAF; MEK
1426426_at	Mak16	MAK16 homolog (S. cerevisiae)	-7.64	0.00	0.66	BRAF
1435888_at	Egfr	epidermal growth factor receptor	-7.65	0.00	0.32	; MEK
1435143_at	Elk3	ELK3. member of ETS oncogene family	-7.65	0.00	0.58	BRAF; MEK
1423847_at	Ncapd2	non-SMC condensin I complex. subunit D2	-7.66	0.00	0.26	BRAF; MEK
1458374_at	C79407	expressed sequence C79407	-7.66	0.00	0.30	BRAF; MEK
1427683_at	Egr2	early growth response 2	-7.66	0.00	0.16	BRAF; MEK
1429665_at	6230416J20Rik	RIKEN cDNA 6230416J20 gene	-7.67	0.00	0.49	BRAF; MEK
1430640_a_at	Prkar2b	protein kinase. cAMP dependent regulatory. type II beta	-7.68	0.00	0.22	BRAF; MEK
1424251_a_at	Hnrpdl	heterogeneous nuclear ribonucleoprotein D-like	-7.68	0.00	0.58	BRAF; MEK
1455803_at	Slco4a1	solute carrier organic anion transporter family. member 4a1	-7.69	0.00	0.18	BRAF; MEK
1435753_a_at	Nucks1	nuclear casein kinase and cyclin-dependent kinase substrate 1	-7.69	0.00	0.56	; MEK
1422430_at	Fignl1	fidgetin-like 1	-7.69	0.00	0.27	BRAF; MEK
1416145_at	Dhx15	DEAH (Asp-Glu-Ala-His) box polypeptide 15	-7.71	0.00	0.65	BRAF; MEK
1415697_at	G3bp2	GTPase activating protein (SH3 domain) binding protein 2	-7.72	0.00	0.55	BRAF; MEK
1449799_s_at	Pkp2	plakophilin 2	-7.75	0.00	0.40	; n.r.
1448990_a_at	Myo1b	myosin IB	-7.75	0.00	0.36	BRAF; MEK
1423714_at	Asf1b	ASF1 anti-silencing function 1 homolog B (S. cerevisiae)	-7.76	0.00	0.39	BRAF; MEK
1437495_at	Mbtps2	membrane-bound transcription factor peptidase. site 2	-7.76	0.00	0.42	BRAF; MEK
1420916_at	Prpf40a	PRP40 pre-mRNA processing factor 40 homolog A (yeast)	-7.77	0.00	0.65	; MEK
1423401_at	Etv6	ets variant gene 6 (TEL oncogene)	-7.78	0.00	0.50	BRAF; n.r.
1451105_at	Vash2	vasohibin 2	-7.79	0.00	0.30	BRAF; MEK
1424144_at	Cdt1	chromatin licensing and DNA replication factor 1	-7.79	0.00	0.21	BRAF; MEK
1448466_at	Cdca5	cell division cycle associated 5	-7.81	0.00	0.33	BRAF; MEK
1416155_at	Hmgb3	high mobility group box 3	-7.81	0.00	0.33	BRAF; MEK
1433580_at	Nup54	nucleoporin 54	-7.81	0.00	0.65	BRAF; MEK
1429368_at	Lrig3	leucine-rich repeats and immunoglobulin-like domains 3	-7.81	0.00	0.70	n.r. ; MEK
1423232_at	Etv4	ets variant gene 4 (E1A enhancer binding protein. E1AF)	-7.81	0.00	0.31	BRAF; MEK
1418253_a_at	Hspa4l	heat shock protein 4 like	-7.84	0.00	0.43	BRAF; MEK
1426830_a_at	Ahcyl1	S-adenosylhomocysteine hydrolase-like 1	-7.84	0.00	0.69	n.r. ; MEK
1450886_at	Gsg2	germ cell-specific gene 2	-7.84	0.00	0.25	BRAF; MEK
1430088_at	Zfp619	zinc finger protein 619	-7.85	0.00	0.71	
1423521_at	Lmnb1	lamin B1	-7.87	0.00	0.38	BRAF; MEK
1418281_at	Rad51	RAD51 homolog (S. cerevisiae)	-7.89	0.00	0.28	BRAF; MEK
1448441_at	Cks1b	CDC28 protein kinase 1b	-7.89	0.00	0.43	BRAF; MEK
1455655_a_at	Tardbp	TAR DNA binding protein	-7.90	0.00	0.54	
1455580_at	Usp6nl	USP6 N-terminal like	-7.90	0.00	0.67	BRAF; MEK
1439695_a_at	Kif20b	kinesin family member 20B	-7.90	0.00	0.18	BRAF; MEK
1434025_at	NA	NA	-7.92	0.00	0.16	BRAF; MEK
1415775_at	Rbbp7	retinoblastoma binding protein 7	-7.93	0.00	0.62	BRAF; MEK
1456130_at	LOC553091	hypothetical LOC553091	-7.94	0.00	0.33	BRAF; MEK
1415852_at	Impdh2	inosine 5'-phosphate dehydrogenase 2	-7.95	0.00	0.55	BRAF; MEK
1416280_at	Uba2	ubiquitin-like modifier activating enzyme 2	-7.95	0.00	0.65	BRAF; MEK
1423683_at	Cdca4	cell division cycle associated 4	-7.96	0.00	0.49	BRAF; MEK

Probeset ID	Symbol	Gene Name	d-value	q-value	R-fold	Expression reversed by
1439377_x_at	Cdc20	cell division cycle 20 homolog (S. cerevisiae)	-7.97	0.00	0.27	BRAF; MEK
1451415_at	1810011O10Rik	RIKEN cDNA 1810011O10 gene	-7.98	0.00	0.18	BRAF; MEK
1448182_a_at	Cd24a	CD24a antigen	-7.99	0.00	0.29	BRAF; MEK
1450053_at	Kif2a	kinesin family member 2A	-8.01	0.00	0.52	BRAF; MEK
1426363_x_at	H2afy2	H2A histone family. member Y2	-8.02	0.00	0.53	BRAF; MEK
1453422_a_at	1110020G09Rik	RIKEN cDNA 1110020G09 gene	-8.03	0.00	0.51	BRAF; MEK
1429051_s_at	Sox11	SRY-box containing gene 11	-8.03	0.00	0.38	BRAF; MEK
1427192_a_at	Brd8	bromodomain containing 8	-8.04	0.00	0.58	BRAF; MEK
1416802_a_at	Cdca5	cell division cycle associated 5	-8.05	0.00	0.26	BRAF; MEK
1428483_a_at	2610039C10Rik	RIKEN cDNA 2610039C10 gene	-8.06	0.00	0.47	BRAF; MEK
1418674_at	Osmr	oncostatin M receptor	-8.07	0.00	0.25	BRAF; MEK
1451323_at	Zfp7	zinc finger protein 7	-8.07	0.00	0.32	BRAF; MEK
1434880_at	Etv6	ets variant gene 6 (TEL oncogene)	-8.08	0.00	0.55	BRAF; MEK
1415860_at	Kpna2	karyopherin (importin) alpha 2	-8.09	0.00	0.57	BRAF; MEK
1416129_at	Errfi1	ERBB receptor feedback inhibitor 1	-8.09	0.00	0.49	BRAF; MEK
1452242_at	Cep55	centrosomal protein 55	-8.10	0.00	0.23	BRAF; MEK
1448314_at	Cdc2a	cell division cycle 2 homolog A (S. pombe)	-8.10	0.00	0.32 *	BRAF; MEK
1418678_at	Has2	hyaluronan synthase 2	-8.11	0.00	0.06	BRAF; MEK
1438368_a_at	Matr3	matrin 3	-8.15	0.00	0.61	; MEK
1423684_at	Hnrnpk	heterogeneous nuclear ribonucleoprotein K	-8.16	0.00	0.72	BRAF
1423324_at	Pnn	pinin	-8.16	0.00	0.53	; MEK
1428168_at	Mpzl1	myelin protein zero-like 1	-8.17	0.00	0.62	BRAF; MEK
1449169_at	Has2	hyaluronan synthase 2	-8.18	0.00	0.08	BRAF; MEK
1448728_a_at	Nfkbiz	nuclear factor of kappa light polypeptide gene enhancer in B-cells inhibitor. zeta	-8.20	0.00	0.38	BRAF; MEK
1424143_a_at	Cdt1	chromatin licensing and DNA replication factor 1	-8.20	0.00	0.23	BRAF; MEK
1448476_at	Nap1l4	nucleosome assembly protein 1-like 4	-8.21	0.00	0.73	BRAF; MEK
1417541_at	Hells	helicase. lymphoid specific	-8.26	0.00	0.20	BRAF; MEK
1438277_at	E130308A19Rik	RIKEN cDNA E130308A19 gene	-8.28	0.00	0.68	; MEK
1428100_at	Sfrs1	splicing factor. arginine/serine-rich 1 (ASF/SF2)	-8.28	0.00	0.52	BRAF; MEK
1423796_at	Sfpq	splicing factor proline/glutamine rich (polypyrimidine tract binding protein associated)	-8.29	0.00	0.54	BRAF; MEK
1434809_at	Arhgap28	Rho GTPase activating protein 28	-8.29	0.00	0.27	BRAF; MEK
1417618_at	Itih2	inter-alpha trypsin inhibitor. heavy chain 2	-8.30	0.00	0.37	BRAF; MEK
1427450_x_at	Myo1b	myosin IB	-8.30	0.00	0.40	BRAF; MEK
1448199_at	Ankrd10	ankyrin repeat domain 10	-8.30	0.00	0.49	BRAF; MEK
1419645_at	Cstf2	cleavage stimulation factor. 3' pre-RNA subunit 2	-8.31	0.00	0.50	BRAF; MEK
1433567_at	Gmps	guanine monphosphate synthetase	-8.32	0.00	0.57	BRAF; MEK
1422849_a_at	Pabpn1	poly? binding protein. nuclear 1	-8.33	0.00	0.61	BRAF; MEK
1423520_at	Lmnb1	lamin B1	-8.37	0.00	0.22	BRAF; MEK
1423474_at	Top1	topoisomerase (DNA) I	-8.39	0.00	0.67	BRAF; MEK
1423369_at	Fmr1	fragile X mental retardation syndrome 1 homolog	-8.41	0.00	0.36	; MEK
1423723_s_at	Tardbp	TAR DNA binding protein	-8.44	0.00	0.46	BRAF; MEK
1447585_s_at	Slc5a3	solute carrier family 5 (inositol transporters). member 3	-8.49	0.00	0.41	BRAF; MEK
1437502_x_at	Cd24a	CD24a antigen	-8.51	0.00	0.29	BRAF; MEK
1417323_at	Psrc1	proline/serine-rich coiled-coil 1	-8.53	0.00	0.49	BRAF; n.r.
1439040_at	Cenpe	centromere protein E	-8.57	0.00	0.27	BRAF; MEK
1449039_a_at	Hnrpdl	heterogeneous nuclear ribonucleoprotein D-like	-8.60	0.00	0.51	BRAF; MEK
1429499_at	Fbxo5	F-box protein 5	-8.61	0.00	0.15	BRAF; MEK
1428142_at	Etv5	ets variant gene 5	-8.63	0.00	0.36	BRAF; MEK
1416433_at	Rpa2	replication protein A2	-8.66	0.00	0.41	BRAF; MEK
1440007_at	D930003E18Rik	RIKEN cDNA D930003E18 gene	-8.71	0.00	0.32	
1439436_x_at	Incenp	inner centromere protein	-8.72	0.00	0.30	BRAF; MEK
1423982_at	Fusip1	FUS interacting protein (serine-arginine rich) 1	-8.73	0.00	0.52	; MEK
1438434_at	Arhgap11a	Rho GTPase activating protein 11A	-8.77	0.00	0.31	BRAF; MEK
1436387_at	C330006P03Rik	RIKEN cDNA C330006P03 gene	-8.79	0.00	0.41	BRAF; MEK
1422243_at	Fgf7	fibroblast growth factor 7	-8.81	0.00	0.17	BRAF; MEK

Probeset ID	Symbol	Gene Name	d-value	q-value	R-fold	Expression reversed by
1448791_at	Snx5	sorting nexin 5	-8.82	0.00	0.62	BRAF; MEK
1450950_at	Smc3	structural maintenace of chromosomes 3	-8.85	0.00	0.51	BRAF; MEK
1417646_a_at	Snx5	sorting nexin 5	-8.89	0.00	0.57	BRAF; MEK
1416950_at	Tnfaip8	tumor necrosis factor. alpha-induced protein 8	-8.92	0.00	0.22	BRAF; MEK
1417822_at	D17H6S56E-5	DNA segment. Chr 17. human D6S56E 5	-8.93	0.00	0.24	BRAF; MEK
1453125_at	Sox11	SRY-box containing gene 11	-8.93	0.00	0.32	BRAF; MEK
1433807_at	6720463M24Rik	RIKEN cDNA 6720463M24 gene	-8.94	0.00	0.35	BRAF; MEK
1449839_at	Casp3	caspase 3	-8.96	0.00	0.49	BRAF; MEK
1423758_at	G3bp2	GTPase activating protein (SH3 domain) binding protein 2	-8.97	0.00	0.66	BRAF; MEK
1416042_s_at	Nasp	nuclear autoantigenic sperm protein (histone-binding)	-9.01	0.00	0.28	BRAF; MEK
1450021_at	Ubqln2	ubiquilin 2	-9.01	0.00	0.57	BRAF; MEK
1416299_at	Shcbp1	Shc SH2-domain binding protein 1	-9.04	0.00	0.23	BRAF; MEK
1423455_at	ENSMUSG00000068659	predicted gene. ENSMUSG00000068659	-9.08	0.00	0.57	BRAF; MEK
1427129_a_at	Hnrnpr	heterogeneous nuclear ribonucleoprotein R	-9.08	0.00	0.64	BRAF; MEK
1437313_x_at	Hmgb2	high mobility group box 2	-9.09	0.00	0.21	BRAF; MEK
1421534_at	LOC14210	hypothetical LOC14210	-9.10	0.00	0.31	BRAF; MEK
1455990_at	Kif23	kinesin family member 23	-9.11	0.00	0.25	BRAF; MEK
1434339_at	Fnbp1l	formin binding protein 1-like	-9.16	0.00	0.36	BRAF; MEK
1456531_x_at	Prpf19	PRP19/PSO4 pre-mRNA processing factor 19 homolog (S. cerevisiae)	-9.20	0.00	0.59	BRAF; MEK
1434240_at	4632434I11Rik	RIKEN cDNA 4632434I11 gene	-9.31	0.00	0.37	BRAF
1428248_at	Nfx1	nuclear transcription factor. X-box binding 1	-9.33	0.00	0.66	BRAF; MEK
1424107_at	Kif18a	kinesin family member 18A	-9.34	0.00	0.33	BRAF; MEK
1448226_at	Rrm2	ribonucleotide reductase M2	-9.42	0.00	0.28	BRAF; MEK
1424181_at	Sept6	septin 6	-9.44	0.00	0.39	BRAF; MEK
1435306_a_at	Kif11	kinesin family member 11	-9.48	0.00	0.24	BRAF; MEK
1422016_a_at	Cenph	centromere protein H	-9.51	0.00	0.28	BRAF; MEK
1426473_at	Dnajc9	DnaJ (Hsp40) homolog. subfamily C. member 9	-9.55	0.00	0.38	BRAF; MEK
1415856_at	Emb	embigin	-9.72	0.00	0.25	n.r. ; MEK
1423811_at	Sf3a3	splicing factor 3a. subunit 3	-9.76	0.00	0.62	BRAF; MEK
1453366_at	Tdrkh	tudor and KH domain containing protein	-9.76	0.00	0.20	BRAF; MEK
1435484_at	Slc5a3	solute carrier family 5 (inositol transporters). member 3	-9.84	0.00	0.24	BRAF; MEK
1417483_at	Nfkbiz	nuclear factor of kappa light polypeptide gene enhancer in B-cells inhibitor. zeta	-9.84	0.00	0.21	BRAF; MEK
1453748_a_at	Kif23	kinesin family member 23	-9.89	0.00	0.22	BRAF; MEK
1460168_at	Slbp	stem-loop binding protein	-10.05	0.00	0.40	BRAF; MEK
1434258_s_at	Phactr4	phosphatase and actin regulator 4	-10.06	0.00	0.55	BRAF; MEK
1438016_at	Dkc1	dyskeratosis congenita 1. dyskerin homolog (human)	-10.07	0.00	0.33	BRAF; MEK
1454659_at	Dctd	dCMP deaminase	-10.16	0.00	0.34	BRAF; MEK
1435460_at	Prkg2	protein kinase. cGMP-dependent. type II	-10.21	0.00	0.16	BRAF; MEK
1423241_a_at	Tfdp1	transcription factor Dp 1	-10.22	0.00	0.62	BRAF; MEK
1452534_a_at	Hmgb2	high mobility group box 2	-10.42	0.00	0.16	BRAF; MEK
1438160_x_at	Slco4a1	solute carrier organic anion transporter family. member 4a1	-10.44	0.00	0.11	BRAF; MEK
1423919_at	BC023882	cDNA sequence BC023882	-10.47	0.00	0.41	BRAF; MEK
1438405_at	Fgf7	fibroblast growth factor 7	-10.53	0.00	0.15	BRAF; MEK
1417065_at	Egr1	early growth response 1	-10.54	0.00	0.09	BRAF; MEK
1419276_at	Enpp1	ectonucleotide pyrophosphatase/phosphodiesterase 1	-10.74	0.00	0.30	; MEK
1424932_at	Egfr	epidermal growth factor receptor	-10.87	0.00	0.37	
1447621_s_at	Tmem173	transmembrane protein 173	-10.97	0.00	0.32	BRAF; MEK
1454788_at	Arl4c	ADP-ribosylation factor-like 4C	-11.02	0.00	0.34	BRAF; MEK
1434660_at	Alkbh1	alkB. alkylation repair homolog 1 (E. coli)	-11.02	0.00	0.63	BRAF; MEK
1442368_at	Kctd12b	potassium channel tetramerisation domain containing 12b	-11.03	0.00	0.13	
1424114_s_at	Lamb1-1	laminin B1 subunit 1	-11.11	0.00	0.38	BRAF; MEK
1437122_at	Bcl2	B-cell leukemia/lymphoma 2	-11.27	0.00	0.55	BRAF; MEK
1435162_at	Prkg2	protein kinase. cGMP-dependent. type II	-11.69	0.00	0.08	BRAF; MEK
1452661_at	Tfrc	transferrin receptor	-11.84	0.00	0.19	BRAF; MEK
1436329_at	Egr3	early growth response 3	-12.14	0.00	0.14	BRAF; MEK

Probeset ID	*Symbol*	*Gene Name*	*d-value*	*q-value*	*R-fold*	*Expression reversed by*
1417155_at	Mycn	v-myc myelocytomatosis viral related oncogene. neuroblastoma derived (avian)	-12.54	0.00	0.17	BRAF; MEK
1440355_at	Kctd12b	potassium channel tetramerisation domain containing 12b	-12.92	0.00	0.16	
1453282_at	Cxadr	coxsackievirus and adenovirus receptor	-13.46	0.00	0.21	BRAF; MEK
1448558_a_at	Pla2g4a	phospholipase A2. group IVA (cytosolic. calcium-dependent)	-13.65	0.00	0.29	; MEK
1416041_at	Sgk1	serum/glucocorticoid regulated kinase 1	-13.93	0.00	0.22	BRAF; MEK
1422562_at	Rrad	Ras-related associated with diabetes	-14.39	0.00	0.28	BRAF; MEK
1415834_at	Dusp6	dual specificity phosphatase 6	-16.40	0.00	0.09 *	BRAF; MEK
