# Supplementary material for: Reversible, interrelated mRNA and miRNA expression patterns in the transcriptome of Rasless fibroblasts: functional and mechanistic implications
Source: BMC Genomics. 2013 Oct 25;14:731. doi: 10.1186/1471-2164-14-731 (PMC4007593; doi:10.1186/1471-2164-14-731)
Supplement: Additional file 2: Table S2 — Functional annotation of the downregulated differentially expressed genes of Rasless MEFs. The GeneCodis functional annotation tool was used on the list of downregulated genes included in Additional file 1: Table S1. Statistically significant associations of particular gene subsets to specific Gene Ontology (GO) functional categories designated as Biological Processes (section S2-BP), KEGG signaling pathways (section S2-KEGG), transcription factors (section S2-TF) and miRNAs prediction (section S2-miRNAs) are presented in this table. [file 1471-2164-14-731-S2.pdf]

Table S2. Functional annotation of the downregulated, differentially expressed genes of Rasless MEFs.

The GeneCodis (Gene Annotation Co-occurrence Discovery) functional annotation tool (http://genecodis.dacya.ucm.es) was used to identify statistically significant functional associations linking particular gene subsets contained within the list of repressed loci occurring in Rasless MEFs (Table S1, Additional file 1, FDR=0.01) to specific cellular functionalities, including particular Biological Processes (section S2-BP), Transcriptional Factors (section S2-TF), Signaling Pathways (Section S2-KEGG) or miRNAs (Section S2-miRNA).

The column labelled "*Functional Category*" identifies the specific functional GO terms recognized in each case for the corresponding groups of loci listed under the column labelled "*Genes*". The column labelled "*Number of Genes*" indicates the specific number of genes annotated to the indicated functionality, out of the total number (in parenthesis) of genes recognized by GeneCodis in the list of repressed genes of Rasless cells. The column labeled "*Corrected p-value*" refers to the statistical significance of the functional associations identified, and contains p-values calculated cases using the Hypergeometric Distribution and subsequently corrected by implementing the False Discovery Rate method of Benjamini and Hochberg [113].

In section S2-BP (pages 2-15), the column "*Functional Category*" assigns common colors to groups of related GO numbers and descriptions falling under the same, high level, general functional categories (DNA and RNA processing, Cell cycle, Cytoskeleton-regulated processes, Transport, Cell death, etc). In section S2-KEGG (pages 16-17), the column "*KEGG Pathway*" identifies KEGG number and denomination of signaling pathways potentially affected by the corresponding group of repressed loci listed in each case. In section S2-TF (pages 18-27), the "*Transcription Factors*" column identifies specific transcription factors that may account for repression of the corresponding groups of loci listed in each case. Finally, in section S2-miRNA (pages 28-53), the*"miRNA name*" column identifies specific mouse miRNAs that, according to current literature and databases, may contribute to the patterns of mRNA repression described for Rasless cells in Table S1.

Table S2-BP. Functional annotation to GO BIOLOGICAL PROCESSES of the downregulated, differentially expressed genes of Rasless MEFs

Functional category	*Number of genes*	*Corrected p-value*	*Genes repressed in Rasless cells (from Table S1)*
**DNA AND RNA PROCESSING**			
**GO:0016070: RNA metabolic process**	287(1215)	4,19E-80	Crebzf, Wdr3, Hmga2, Ell2, Rbbp7, Ruvbl1, Gli2, Tfdp1, Gtf2h2, U2af1, Cbx3, Rbmxrt, Kin, Esf1, Pabpn1, Asxl3, Myc, Lsm2, Elf2, Mcm2, Snrpd1, Chaf1b, Rnmt, Zfp57, Nola1, Pou2f1, Sf3a3, Gli3, Gpbp1, Srpk1, Narg1, Slbp, Fbl, Litaf, Etv4, 2210018M11Rik, Prpf19, Sfrs7, Prpf4b, Prim2, Mycn, Ilf3, Runx1, Mlf1ip, Egr1, Ebna1bp2, Taf5, Tbx3, Sip1, Arid4b, Uhrf1, Rnaseh2a, Cpsf2, Dmap1, Ppwd1, Elp2, Ecd, Utp6, 3930401K13Rik, Hat1, Vps36, Sfpq, Atrx, Bach1, Baz1a, Rnf2, Hnrnpm, Utp11l, Tyw3, Mphosph10, Satb2, Runx2, Ets2, Tcerg1, Ppil1, Smndc1, Bnc2, Mcm4, Trim27, 2510012J08Rik, Fhl2, Fosl1, Cand1, Tcf4, Mrpl1, Sertad1, Asf1b, Taf5l, Actl6a, Tfam, Mcm5, Egr2, Top2a, Nfx1, Dnmt1, Prpf4, Ddx46, Ezh2, Cenpk, Gemin6, Cpsf4, Asf1a, Nob1, Psrc1, Prpf38b, Trit1, Eftud2, Aebp2, Gemin8, Atad2, Rbl1, Dis3, Dbr1, Whsc1, Exosc8, Isy1, Mbtps2, Trim24, Zcchc8, Cnot6, Utp15, Snapc3, Ebf2, Hdac2, Trim28, Smarca5, Six4, Nono, Sfrs3, Sap30, Cpsf6, Prpf40a, Dimt1, E2f7, Ptbp2, Nudt21, Phf6, Tardbp, Rnps1, Ctbp2, Nab2, Pspc1, Sfrs4, Smarcc1, Snrpb, Rcl1, Mkx, Cdca7l, Trub1, Zfp280c, Phf17, Hnrnpk, Hnrpdl, Kars, Foxm1, 1110005A23Rik, Egr3, Zik1, Snrpb2, Cdca7, U2af2, Mcm3, Snrpa1, Ruvbl2, Wdr57, Etv5, Hnrnpc, Prpf38a, Psip1, Rbbp4, Ifi205, Tgif1, Magoh, Ilf2, Cebpz, Gmeb1, Eid1, Zfp422, Ddx20, Sf3a1, Imp3, Lsm8, 2410016O06Rik, Dlx1, Ets1, Ddx51, Sf1, Ssrp1, Exosc2, Sin3a, Rbm14, Med13, Timeless, 2700050L05Rik, Fusip1, Rbm9, Sfrs10, Hnrnpa2b1, Pprc1, Hltf, Dkc1, Hells, Nsun2, Med4, Suv39h1, Sltm, Brd8, Sub1, Med14, Cugbp1, Mcm7, Larp7, Prim1, Med13l, Zfp532, Prpf31, Rbm17, Eif4a3, Trmt6, Fus, Nrf1, Nfkbiz, Arhgap22, Ahctf1, Khsrp, Zfp184, Rnf12, Polr1e, Junb, Zfp36l2, Nsbp1, Zfp451, Sox4, Nr2f2, Klf5, Khdrbs1, Hnrnpf, Th1l, Zfp770, Elk3, Tcfe2a, Elavl1, Foxp1, Cdc40, Hnrnpa1, Mybl2, Pnn, Tgif2, Dhx15, Emg1, Ell, Bclaf1, 5730453I16Rik, Paip1, Syncrip, Suz12, 2610101N10Rik, Casp8ap2, Rbpj, Ash2l, Mybbp1a, Lcorl, Lif, Whsc2, Brunol4, Ppih, Etv6, Tead2, Cdc5l, Mcm6, Gata2, Ctcf, Hmga1, Fubp1, Prmt5, Cstf2, Sox11, Sfrs1, Hnrnpd, Ddx39, Snw1, Cbx2, Npm1, Suv39h2, Sirt1, Rest, E2f8

Functional category	*Number of genes*	*Corrected p-value*	*Genes repressed in Rasless cells (from Table S1)*
GO:0006259: DNA metabolic process	115(1215)	1,46E-71	Neil3, Rbbp7, Ruvbl1, Apex1, Gtf2h2, Kin, Myc, Ccne2, Mcm2, Lin9, Chaf1b, Rad54l, Smc3, 2210018M11Rik, Prpf19, Gins4, Prim2, Pola1, Casp3, Hmgn1, Dscc1, Terf1, Fanca, Recql4, Msh6, Rad51ap1, Pttg1, Uhrf1, Blm, Dbf4, Sfpq, Atrx, Fancd2, Rrm1, Trip13, Mcm4, Rpa2, Dtl, Mcm5, Top2a, Fancm, Dnmt1, Asf1a, Top1, Clspn, Psmc3ip, Cdc45l, Rfc2, Brca1, Gins1, Orc1l, Rad21, Exo1, Nono, Rad51, Pold3, Nasp, Parp1, Rrm2, Lig1, Gins3, Rfc4, Rad18, Pole2, Mcm3, Pold1, Ruvbl2, Gmnn, Tipin, Aifm1, Rad51c, Rbbp4, Topbp1, Smc5, Mbd4, Fancb, Ssrp1, Orc6l, Mre11a, Fen1, Gins2, Dffb, Hus1, Hells, Gen1, Mcm7, Rfc5, Prim1, Mcm10, Chek1, Apex2, Mnd1, Fancc, Smc6, Setx, Rfc3, Orc2l, Tinf2, Foxp1, Msh2, Ung, Nudt1, Trpc2, Usp1, Swap70, Tnks2, Pcna, Pola2, Cdt1, Eme1, Esco2, Mcm6, Tk1, Ctcf, Cdc6
GO:0034961: cellular biopolymer biosynthetic process	272(1215)	9,03E-65	Crebzf, Bcl10, Hmga2, Ell2, Rbbp7, Ruvbl1, Gli2, Tfdp1, Gtf2h2, Cbx3, Kin, Esf1, Asxl3, Igf2bp1, Gtf3c2, Myc, Ccne2, Elf2, Etf1, Rps9, Mcm2, Lin9, Pum2, Chaf1b, Zfp57, Pou2f1, Topors, Gli3, Gpbp1, Hbs1l, Narg1, Litaf, Etv4, 2210018M11Rik, Gins4, Prim2, Mycn, Ilf3, Runx1, Pola1, Mlf1ip, Egr1, Dscc1, Terf1, Taf5, Tbx3, Arid4b, Msh6, Uhrf1, Dmap1, Elp2, Rpo1_1, Ecd, Zcchc11, 3930401K13Rik, Blm, Hat1, Vps36, Dbf4, Sfpq, Mrpl19, Atrx, Bach1, Rrm1, Baz1a, Rnf2, Satb2, Runx2, Ets2, Tcerg1, Bnc2, Mcm4, Rpa2, Trim27, Fhl2, Dtl, Fosl1, Cand1, Rpl41, Tcf4, Mrpl1, Sertad1, Asf1b, Taf5l, Actl6a, Tfam, Mcm5, Egr2, Top2a, Nfx1, Dnmt1, Ezh2, Cenpk, Cdk2, Asf1a, Top1, Psrc1, Cdc73, Clspn, Patz1, Aebp2, Cdc45l, Atad2, Rps13, Rbl1, Ptrh2, Rfc2, Brca1, Tgfbr3, Whsc1, Mbtps2, Trim24, Cnot6, Snapc3, Ebf2, Pogz, Hdac2, Trim28, Smarca5, Gins1, Orc1l, Six4, Nono, Sap30, Pold3, E2f7, Eif4h, Phf6, Tardbp, Ctbp2, Nab2, Rpl3, Pspc1, Nasp, Smarcc1, Rrm2, Mkx, Cdca7l, Lig1, Zfp280c, Phf17, Hnrpdl, Kars, Foxm1, Gins3, 1110005A23Rik, Egr3, Zik1, Rfc4, Cdca7, Pole2, Mcm3, Pold1, Ruvbl2, Eif5, Gmnn, Tipin, Etv5, Psip1, Rbbp4, Ifi205, Tgif1, Ilf2, Cebpz, Mrps6, Gmeb1, Eid1, Rpl7l1, Zfp422, Ddx20, 2410016O06Rik, Dlx1, Ets1, Sf1, Denr, Tmpo, Ssrp1, Orc6l, Fen1, Sin3a, Rbm14, Med13, Timeless, 2700050L05Rik, Gins2, Fusip1, Pprc1, Eef1g, Hus1, Hltf, Hells, Med4, Suv39h1, Sltm, Brd8, Sub1, Med14, Mcm7, Rfc5, Prim1, Mcm10, Polr2b, Med13l, Zfp532, Trmt6, Fus, Nrf1, Nfkbiz, Arhgap22, Ahctf1, Khsrp, Zfp184, Twistnb, Rnf12, Polr1e, Junb, Nsbp1, Zfp451, Ccna2, Sox4, Nr2f2, Rfc3, Orc2l, Klf5, Khdrbs1, Th1l, Zfp770, Elk3, Rpo12, Tcfe2a, Foxp1, Msh2, Eif2s1, Mybl2, Pnn, Tgif2, Ell, Bclaf1, Paip1, Suz12, Casp8ap2, Rbpj, Ash2l, Myef2, Mybbp1a, Gspt1, Pcna, Pola2, Lcorl, Lif, Whsc2, Cdt1, Etv6, Tead2, Cdc5l, Mcm6, Ripk2, Gata2, Tk1, Ctcf, Cdc6, Hmga1, Fubp1, Prmt5, Sox11, Hnrnpd, Snw1, Cbx2, Suv39h2, Sirt1, Rest, E2f8
GO:0006396: RNA processing	103(1215)	5,86E-58	Wdr3, U2af1, Rbmxrt, Kin, Pabpn1, Lsm2, Snrpd1, Rnmt, Nola1, Sf3a3, Srpk1, Slbp, Fbl, Prpf19, Sfrs7, Prpf4b, Ebna1bp2, Sip1, Cpsf2, Ppwd1, Utp6, Sfpq, Hnrnpm, Utp11l, Tyw3, Mphosph10, Ppil1, Smndc1, 2510012J08Rik, Mrpl1, Prpf4, Ddx46, Gemin6, Cpsf4, Nob1, Prpf38b, Trit1, Eftud2, Gemin8, Dis3, Dbr1, Exosc8, Isy1, Zcchc8, Utp15, Nono, Sfrs3, Cpsf6, Prpf40a, Dimt1, Ptbp2, Nudt21, Tardbp, Rnps1, Sfrs4, Snrpb, Rcl1, Trub1, Hnrnpk, Snrpb2, U2af2, Snrpa1, Wdr57, Hnrnpc, Prpf38a, Magoh, Ddx20, Sf3a1, Imp3, Lsm8, Ddx51, Sf1, Exosc2, Fusip1, Rbm9, Sfrs10, Hnrnpa2b1, Dkc1, Nsun2, Cugbp1, Larp7, Prpf31, Rbm17, Eif4a3, Trmt6, Khsrp, Hnrnpf, Cdc40, Hnrnpa1, Pnn, Dhx15, Emg1, 5730453I16Rik, Syncrip, 2610101N10Rik, Brunol4, Ppih, Cdc5l, Cstf2, Sfrs1, Ddx39, Snw1, Npm1
GO:0006397: mRNA processing	77(1215)	4,20E-48	U2af1, Rbmxrt, Kin, Pabpn1, Lsm2, Snrpd1, Rnmt, Sf3a3, Srpk1, Slbp, Prpf19, Sfrs7, Prpf4b, Sip1, Cpsf2, Ppwd1, Sfpq, Hnrnpm, Ppil1, Smndc1, 2510012J08Rik, Prpf4, Ddx46, Gemin6, Cpsf4, Prpf38b, Eftud2, Gemin8, Dbr1, Isy1, Zcchc8, Nono, Sfrs3, Cpsf6, Prpf40a, Ptbp2, Nudt21, Tardbp, Rnps1, Sfrs4, Snrpb, Hnrnpk, Snrpb2, U2af2, Snrpa1, Wdr57, Hnrnpc, Prpf38a, Magoh, Ddx20, Sf3a1, Lsm8, Sf1, Fusip1, Rbm9, Sfrs10, Hnrnpa2b1, Cugbp1, Prpf31, Rbm17, Eif4a3, Khsrp, Hnrnpf, Cdc40, Hnrnpa1, Pnn, Dhx15, 5730453I16Rik, Syncrip, Brunol4, Ppih, Cdc5l, Cstf2, Sfrs1, Ddx39, Snw1, Npm1
GO:0016071: mRNA metabolic process	81(1215)	9,15E-48	U2af1, Rbmxrt, Kin, Pabpn1, Lsm2, Snrpd1, Rnmt, Sf3a3, Srpk1, Slbp, Prpf19, Sfrs7, Prpf4b, Sip1, Cpsf2, Ppwd1, Sfpq, Hnrnpm, Ppil1, Smndc1, 2510012J08Rik, Prpf4, Ddx46, Gemin6, Cpsf4, Prpf38b, Eftud2, Gemin8, Dbr1, Isy1, Zcchc8, Nono, Sfrs3, Cpsf6, Prpf40a, Ptbp2, Nudt21, Tardbp, Rnps1, Sfrs4, Snrpb, Hnrnpk, Hnrpdl, Snrpb2, U2af2, Snrpa1, Wdr57, Hnrnpc, Prpf38a, Magoh, Ddx20, Sf3a1, Lsm8, Sf1, Fusip1, Rbm9, Sfrs10, Hnrnpa2b1, Cugbp1, Prpf31, Rbm17, Eif4a3, Khsrp, Zfp36l2, Hnrnpf, Elavl1, Cdc40, Hnrnpa1, Pnn, Dhx15, 5730453I16Rik, Syncrip, Brunol4, Ppih, Cdc5l, Cstf2, Sfrs1, Hnrnpd, Ddx39, Snw1, Npm1
GO:0019219: regulation of nucleobase, nucleoside, nucleotide and nucleic acid metabolic process	210(1215)	1,79E-45	Crebzf, Bcl10, Hmga2, Ell2, Rbbp7, Ruvbl1, Gli2, Tfdp1, Gtf2h2, Cbx3, Esf1, Asxl3, Myc, Elf2, Mcm2, Chaf1b, Zfp57, Pou2f1, Topors, Gli3, Gpbp1, Srpk1, Narg1, Litaf, Etv4, 2210018M11Rik, Mycn, Ilf3, Runx1, Mlf1ip, Egr1, Terf1, Taf5, Tbx3, Arid4b, Msh6, Uhrf1, Dmap1, Elp2, Ecd, Zcchc11, 3930401K13Rik, Blm, Hat1, Vps36, Sfpq, Atrx, Bach1, Baz1a, Rnf2, Satb2, Runx2, Ets2, Tcerg1, Bnc2, Mcm4, Trim27, Fhl2, Fosl1, Cand1, Tcf4, Sertad1, Asf1b, Taf5l, Actl6a, Tfam, Mcm5, Egr2, Top2a, Nfx1, Dnmt1, Ezh2, Cenpk, Cdk2, Asf1a, Psrc1, Clspn, Patz1, Aebp2, Atad2, Rbl1, Tgfbr3, Whsc1, Mbtps2, Trim24, Cnot6, Snapc3, Ebf2, Pogz, Hdac2, Trim28, Smarca5, Six4, Nono, Sap30, E2f7, Phf6, Tardbp, Ctbp2, Nab2, Pspc1, Smarcc1, Mkx, Cdca7l, Zfp280c, Phf17, Hnrpdl, Foxm1, 1110005A23Rik, Egr3, Zik1, Cdca7, Mcm3, Ruvbl2, Gmnn, Tipin, Etv5, Psip1, Rbbp4, Ifi205, Tgif1, Ilf2, Cebpz, Gmeb1, Eid1, Zfp422, Ddx20, 2410016O06Rik, Dlx1, Ets1, Sf1, Tmpo, Ssrp1, Sin3a, Rbm14, Med13, Timeless, 2700050L05Rik, Fusip1, Pprc1, Hus1, Hltf, Hells, Med4, Suv39h1, Sltm, Brd8, Sub1, Med14, Mcm7, Med13l, Zfp532, Fus, Nrf1, Nfkbiz, Arhgap22, Ahctf1, Khsrp, Zfp184, Rnf12, Junb, Zfp36l2, Nsbp1, Zfp451, Ccna2, Sox4, Nr2f2, Klf5, Khdrbs1, Th1l, Zfp770, Elk3, Tcfe2a, Elavl1, Foxp1, Msh2, Mybl2, Pnn, Tgif2, Ell, Bclaf1, Suz12, Casp8ap2, Rbpj, Ash2l, Mybbp1a, Pcna, Lcorl, Lif, Whsc2, Cdt1, Etv6, Tead2, Cdc5l, Mcm6, Ripk2, Gata2, Ctcf, Hmga1, Fubp1, Prmt5, Sox11, Hnrnpd, Snw1, Cbx2, Npm1, Suv39h2, Sirt1, Rest, E2f8
GO:0008380: RNA splicing	67(1215)	2,61E-45	U2af1, Rbmxrt, Lsm2, Snrpd1, Sf3a3, Srpk1, Prpf19, Sfrs7, Prpf4b, Sip1, Ppwd1, Sfpq, Hnrnpm, Ppil1, Smndc1, 2510012J08Rik, Prpf4, Ddx46, Gemin6, Prpf38b, Eftud2, Gemin8, Dbr1, Isy1, Zcchc8, Nono, Sfrs3, Prpf40a, Ptbp2, Tardbp, Rnps1, Sfrs4, Snrpb, Hnrnpk, Snrpb2, U2af2, Snrpa1, Wdr57, Hnrnpc, Prpf38a, Magoh, Ddx20, Sf3a1, Lsm8, Sf1, Fusip1, Rbm9, Sfrs10, Hnrnpa2b1, Cugbp1, Prpf31, Rbm17, Eif4a3, Khsrp, Hnrnpf, Cdc40, Hnrnpa1, Pnn, Dhx15, Syncrip, Brunol4, Ppih, Cdc5l, Sfrs1, Ddx39, Snw1, Npm1

Functional category	*Number of genes*	*Corrected p-value*	*Genes repressed in Rasless cells (from Table S1)*
**GO:0010468: regulation of gene expression**	214(1215)	9,89E-45	Crebzf, Bcl10, Hmga2, Ell2, Rbbp7, Ruvbl1, Gli2, Tfdp1, Gtf2h2, Cbx3, H2afy, Alkbh1, Esf1, Asxl3, Igf2bp1, Myc, Elf2, Mcm2, Pum2, Chaf1b, Zfp57, Pou2f1, Topors, Gli3, Gpbp1, Srpk1, Narg1, Litaf, Etv4, 2210018M11Rik, Mycn, Ilf3, Runx1, Mlf1ip, Egr1, Terf1, Taf5, Tbx3, Arid4b, Uhrf1, Dmap1, Elp2, Ecd, Zcchc11, 3930401K13Rik, Hat1, Vps36, Sfpq, Atrx, Bach1, H2afy2, Baz1a, Rnf2, Satb2, Runx2, Ets2, Tcerg1, Bnc2, Mcm4, Trim27, Fhl2, Fosl1, Cand1, Tcf4, Sertad1, Asf1b, Taf5l, Actl6a, Tfam, Mcm5, Egr2, Top2a, Nfx1, Dnmt1, Ezh2, Cenpk, Cdk2, Asf1a, Psrc1, Patz1, Aebp2, Atad2, Rbl1, Brca1, Tgfbr3, Whsc1, Mbtps2, Trim24, Cnot6, Snapc3, Ebf2, Pogz, Hdac2, Trim28, Smarca5, Six4, Nono, Sap30, E2f7, Phf6, Tardbp, Ctbp2, Nab2, Pspc1, Smarcc1, Mkx, Cdca7l, Zfp280c, Phf17, Hnrpdl, Foxm1, 1110005A23Rik, Egr3, Zik1, Cdca7, Mcm3, Ruvbl2, Etv5, Psip1, Rbbp4, Ifi205, Tgif1, Ilf2, Cebpz, Gmeb1, Eid1, Zfp422, Ddx20, 2410016O06Rik, Dlx1, Mbd4, Ets1, Sf1, Tmpo, Ssrp1, Sin3a, Rbm14, Med13, Timeless, 2700050L05Rik, Fusip1, Pprc1, Hltf, Hells, Med4, Suv39h1, Sltm, Brd8, Sub1, Med14, Mcm7, Med13l, Zfp532, Trmt6, Fus, Nrf1, Nfkbiz, Arhgap22, Ahctf1, Khsrp, Zfp184, Rnf12, Junb, Zfp36l2, Nsbp1, Zfp451, Ccna2, Sox4, Nr2f2, Klf5, Khdrbs1, Th1l, Zfp770, Elk3, Tcfe2a, Elavl1, Foxp1, Eif2s1, Mybl2, Pnn, Tgif2, Ell, Bclaf1, Skp2, Paip1, Suz12, Casp8ap2, Rbpj, Ash2l, Mybbp1a, Lcorl, Lif, Whsc2, Etv6, Tead2, Cdc5l, Mcm6, Ripk2, Gata2, Ctcf, Hmga1, Fubp1, Prmt5, Sox11, Hnrnpd, Snw1, Cbx2, Bcl2, Npm1, Suv39h2, Sirt1, Thex1, Rest, E2f8
**GO:0010556: regulation of macromolecule biosynthetic process**	209(1215)	8,03E-43	Crebzf, Bcl10, Hmga2, Ell2, Rbbp7, Ruvbl1, Gli2, Tfdp1, Gtf2h2, Cbx3, Esf1, Asxl3, Igf2bp1, Myc, Elf2, Mcm2, Pum2, Chaf1b, Zfp57, Pou2f1, Topors, Gli3, Gpbp1, Narg1, Litaf, Etv4, 2210018M11Rik, Mycn, Ilf3, Runx1, Mlf1ip, Egr1, Terf1, Taf5, Tbx3, Arid4b, Uhrf1, Dmap1, Elp2, Ecd, Zcchc11, 3930401K13Rik, Hat1, Vps36, Sfpq, Atrx, Bach1, Baz1a, Rnf2, Satb2, Runx2, Ets2, Tcerg1, Bnc2, Mcm4, Trim27, Fhl2, Fosl1, Cand1, Tcf4, Sertad1, Asf1b, Taf5l, Actl6a, Tfam, Mcm5, Egr2, Top2a, Nfx1, Dnmt1, Ezh2, Cenpk, Cdk2, Asf1a, Psrc1, Clspn, Patz1, Aebp2, Atad2, Rbl1, Tgfbr3, Whsc1, Mbtps2, Trim24, Cnot6, Snapc3, Ebf2, Pogz, Hdac2, Trim28, Smarca5, Six4, Nono, Sap30, E2f7, Phf6, Tardbp, Ctbp2, Nab2, Pspc1, Smarcc1, Mkx, Cdca7l, Zfp280c, Phf17, Hnrpdl, Foxm1, 1110005A23Rik, Egr3, Zik1, Cdca7, Mcm3, Ruvbl2, Gmnn, Tipin, Etv5, Psip1, Rbbp4, Ifi205, Tgif1, Ilf2, Cebpz, Gmeb1, Eid1, Zfp422, Ddx20, 2410016O06Rik, Dlx1, Ets1, Sf1, Tmpo, Ssrp1, Sin3a, Rbm14, Med13, Timeless, 2700050L05Rik, Fusip1, Pprc1, Hus1, Hltf, Hells, Med4, Suv39h1, Sltm, Brd8, Sub1, Med14, Mcm7, Med13l, Zfp532, Trmt6, Fus, Nrf1, Nfkbiz, Arhgap22, Ahctf1, Khsrp, Zfp184, Rnf12, Junb, Nsbp1, Zfp451, Ccna2, Sox4, Nr2f2, Klf5, Khdrbs1, Th1l, Zfp770, Elk3, Tcfe2a, Foxp1, Eif2s1, Mybl2, Pnn, Tgif2, Ell, Bclaf1, Paip1, Suz12, Casp8ap2, Rbpj, Ash2l, Mybbp1a, Pcna, Lcorl, Lif, Whsc2, Cdt1, Etv6, Tead2, Cdc5l, Mcm6, Ripk2, Gata2, Ctcf, Hmga1, Fubp1, Prmt5, Sox11, Hnrnpd, Snw1, Cbx2, Bcl2, Suv39h2, Sirt1, Rest, E2f8
**GO:0031326: regulation of cellular biosynthetic process**	210(1215)	1,91E-42	Crebzf, Bcl10, Hmga2, Ell2, Rbbp7, Ruvbl1, Gli2, Tfdp1, Gtf2h2, Cbx3, Esf1, Asxl3, Igf2bp1, Myc, Elf2, Mcm2, Pum2, Chaf1b, Zfp57, Pou2f1, Topors, Gli3, Gpbp1, Narg1, Litaf, Etv4, 2210018M11Rik, Mycn, Ilf3, Runx1, Mlf1ip, Egr1, Terf1, Taf5, Tbx3, Arid4b, Uhrf1, Dmap1, Elp2, Ecd, Zcchc11, 3930401K13Rik, Hat1, Vps36, Sfpq, Atrx, Bach1, Baz1a, Rnf2, Satb2, Runx2, Ets2, Tcerg1, Bnc2, Mcm4, Trim27, Fhl2, Fosl1, Cand1, Tcf4, Sertad1, Asf1b, Taf5l, Actl6a, Tfam, Mcm5, Egr2, Top2a, Nfx1, Dnmt1, Ezh2, Cenpk, Cdk2, Asf1a, Psrc1, Clspn, Patz1, Aebp2, Atad2, Rbl1, Tgfbr3, Whsc1, Mbtps2, Trim24, Cnot6, Snapc3, Ebf2, Pogz, Hdac2, Trim28, Smarca5, Six4, Nono, Sap30, E2f7, Phf6, Tardbp, Ctbp2, Nab2, Pspc1, Smarcc1, Mkx, Cdca7l, Zfp280c, Phf17, Hnrpdl, Foxm1, 1110005A23Rik, Egr3, Zik1, Cdca7, Mcm3, Ruvbl2, Gmnn, Tipin, Etv5, Psip1, Hsp90aa1, Rbbp4, Ifi205, Tgif1, Ilf2, Cebpz, Gmeb1, Eid1, Zfp422, Ddx20, 2410016O06Rik, Dlx1, Ets1, Sf1, Tmpo, Ssrp1, Sin3a, Rbm14, Med13, Timeless, 2700050L05Rik, Fusip1, Pprc1, Hus1, Hltf, Hells, Med4, Suv39h1, Sltm, Brd8, Sub1, Med14, Mcm7, Med13l, Zfp532, Trmt6, Fus, Nrf1, Nfkbiz, Arhgap22, Ahctf1, Khsrp, Zfp184, Rnf12, Junb, Nsbp1, Zfp451, Ccna2, Sox4, Nr2f2, Klf5, Khdrbs1, Th1l, Zfp770, Elk3, Tcfe2a, Foxp1, Eif2s1, Mybl2, Pnn, Tgif2, Ell, Bclaf1, Paip1, Suz12, Casp8ap2, Rbpj, Ash2l, Mybbp1a, Pcna, Lcorl, Lif, Whsc2, Cdt1, Etv6, Tead2, Cdc5l, Mcm6, Ripk2, Gata2, Ctcf, Hmga1, Fubp1, Prmt5, Sox11, Hnrnpd, Snw1, Cbx2, Bcl2, Suv39h2, Sirt1, Rest, E2f8
**GO:0006260: DNA replication**	56(1215)	7,79E-42	Rbbp7, Kin, Ccne2, Mcm2, Lin9, Chaf1b, Gins4, Prim2, Pola1, Dscc1, Terf1, Msh6, Blm, Dbf4, Rrm1, Mcm4, Rpa2, Dtl, Mcm5, Top1, Clspn, Cdc45l, Rfc2, Brca1, Gins1, Orc1l, Pold3, Nasp, Rrm2, Lig1, Gins3, Rfc4, Pole2, Mcm3, Pold1, Gmnn, Tipin, Rbbp4, Ssrp1, Orc6l, Fen1, Gins2, Hus1, Mcm7, Rfc5, Prim1, Mcm10, Rfc3, Orc2l, Msh2, Pcna, Pola2, Cdt1, Mcm6, Tk1, Cdc6
**GO:0045449: regulation of transcription**	197(1215)	2,79E-40	Crebzf, Bcl10, Hmga2, Ell2, Rbbp7, Ruvbl1, Gli2, Tfdp1, Gtf2h2, Cbx3, Esf1, Asxl3, Myc, Elf2, Mcm2, Chaf1b, Zfp57, Pou2f1, Topors, Gli3, Gpbp1, Narg1, Litaf, Etv4, 2210018M11Rik, Mycn, Ilf3, Runx1, Mlf1ip, Egr1, Terf1, Taf5, Tbx3, Arid4b, Uhrf1, Dmap1, Elp2, Ecd, Zcchc11, 3930401K13Rik, Hat1, Vps36, Sfpq, Atrx, Bach1, Baz1a, Rnf2, Satb2, Runx2, Ets2, Tcerg1, Bnc2, Mcm4, Trim27, Fhl2, Fosl1, Cand1, Tcf4, Sertad1, Asf1b, Taf5l, Actl6a, Tfam, Mcm5, Egr2, Top2a, Nfx1, Dnmt1, Ezh2, Cenpk, Cdk2, Asf1a, Psrc1, Patz1, Aebp2, Atad2, Rbl1, Tgfbr3, Whsc1, Mbtps2, Trim24, Cnot6, Snapc3, Ebf2, Pogz, Hdac2, Trim28, Smarca5, Six4, Nono, Sap30, E2f7, Phf6, Tardbp, Ctbp2, Nab2, Pspc1, Smarcc1, Mkx, Cdca7l, Zfp280c, Phf17, Hnrpdl, Foxm1, 1110005A23Rik, Egr3, Zik1, Cdca7, Mcm3, Ruvbl2, Etv5, Psip1, Rbbp4, Ifi205, Tgif1, Ilf2, Cebpz, Gmeb1, Eid1, Zfp422, Ddx20, 2410016O06Rik, Dlx1, Ets1, Sf1, Tmpo, Ssrp1, Sin3a, Rbm14, Med13, Timeless, 2700050L05Rik, Fusip1, Pprc1, Hltf, Hells, Med4, Suv39h1, Sltm, Brd8, Sub1, Med14, Mcm7, Med13l, Zfp532, Fus, Nrf1, Nfkbiz, Arhgap22, Ahctf1, Khsrp, Zfp184, Rnf12, Junb, Nsbp1, Zfp451, Ccna2, Sox4, Nr2f2, Klf5, Khdrbs1, Th1l, Zfp770, Elk3, Tcfe2a, Foxp1, Mybl2, Pnn, Tgif2, Ell, Bclaf1, Suz12, Casp8ap2, Rbpj, Ash2l, Mybbp1a, Lcorl, Lif, Whsc2, Etv6, Tead2, Cdc5l, Mcm6, Ripk2, Gata2, Ctcf, Hmga1, Fubp1, Prmt5, Sox11, Hnrnpd, Snw1, Cbx2, Suv39h2, Sirt1, Rest, E2f8

Functional category	*Number of genes*	*Corrected p-value*	*Genes repressed in Rasless cells (from Table S1)*
**GO:0051252: regulation of RNA metabolic process**	190(1215)	9,28E-39	Crebzf, Hmga2, Ell2, Rbbp7, Ruvbl1, Gli2, Tfdp1, Gtf2h2, Cbx3, Esf1, Asxl3, Myc, Elf2, Mcm2, Chaf1b, Zfp57, Pou2f1, Gli3, Gpbp1, Srpk1, Narg1, Litaf, Etv4, 2210018M11Rik, Mycn, Ilf3, Runx1, Mlf1ip, Egr1, Taf5, Tbx3, Arid4b, Uhrf1, Dmap1, Elp2, Ecd, 3930401K13Rik, Hat1, Vps36, Sfpq, Atrx, Bach1, Baz1a, Rnf2, Satb2, Runx2, Ets2, Tcerg1, Bnc2, Mcm4, Trim27, Fhl2, Fosl1, Cand1, Tcf4, Sertad1, Asf1b, Taf5l, Actl6a, Tfam, Mcm5, Egr2, Top2a, Nfx1, Dnmt1, Ezh2, Cenpk, Asf1a, Psrc1, Aebp2, Atad2, Rbl1, Whsc1, Mbtps2, Trim24, Cnot6, Snapc3, Ebf2, Hdac2, Trim28, Smarca5, Six4, Nono, Sap30, E2f7, Phf6, Tardbp, Ctbp2, Nab2, Pspc1, Smarcc1, Mkx, Cdca7l, Zfp280c, Phf17, Hnrpdl, Foxm1, 1110005A23Rik, Egr3, Zik1, Cdca7, Mcm3, Ruvbl2, Etv5, Psip1, Rbbp4, Ifi205, Tgif1, Ilf2, Cebpz, Gmeb1, Eid1, Zfp422, Ddx20, 2410016O06Rik, Dlx1, Ets1, Sf1, Ssrp1, Sin3a, Rbm14, Med13, Timeless, 2700050L05Rik, Fusip1, Pprc1, Hltf, Hells, Med4, Suv39h1, Sltm, Brd8, Sub1, Med14, Mcm7, Med13l, Zfp532, Fus, Nrf1, Nfkbiz, Arhgap22, Ahctf1, Khsrp, Zfp184, Rnf12, Junb, Zfp36l2, Nsbp1, Zfp451, Sox4, Nr2f2, Klf5, Khdrbs1, Th1l, Zfp770, Elk3, Tcfe2a, Elavl1, Foxp1, Mybl2, Pnn, Tgif2, Ell, Bclaf1, Suz12, Casp8ap2, Rbpj, Ash2l, Mybbp1a, Lcorl, Lif, Whsc2, Etv6, Tead2, Cdc5l, Mcm6, Gata2, Ctcf, Hmga1, Fubp1, Prmt5, Sox11, Hnrnpd, Snw1, Cbx2, Npm1, Suv39h2, Sirt1, Rest, E2f8
**GO:0032774: RNA biosynthetic process**	188(1215)	1,73E-37	Crebzf, Hmga2, Ell2, Rbbp7, Ruvbl1, Gli2, Tfdp1, Gtf2h2, Cbx3, Esf1, Asxl3, Myc, Elf2, Mcm2, Chaf1b, Zfp57, Pou2f1, Gli3, Gpbp1, Narg1, Litaf, Etv4, 2210018M11Rik, Prim2, Mycn, Ilf3, Runx1, Mlf1ip, Egr1, Taf5, Tbx3, Arid4b, Uhrf1, Dmap1, Elp2, Ecd, 3930401K13Rik, Hat1, Vps36, Sfpq, Atrx, Bach1, Baz1a, Rnf2, Satb2, Runx2, Ets2, Tcerg1, Bnc2, Mcm4, Trim27, Fhl2, Fosl1, Cand1, Tcf4, Sertad1, Asf1b, Taf5l, Actl6a, Tfam, Mcm5, Egr2, Top2a, Nfx1, Dnmt1, Ezh2, Cenpk, Asf1a, Psrc1, Aebp2, Atad2, Rbl1, Whsc1, Mbtps2, Trim24, Cnot6, Snapc3, Ebf2, Hdac2, Trim28, Smarca5, Six4, Nono, Sap30, E2f7, Phf6, Tardbp, Ctbp2, Nab2, Pspc1, Smarcc1, Mkx, Cdca7l, Zfp280c, Phf17, Hnrpdl, Foxm1, 1110005A23Rik, Egr3, Zik1, Cdca7, Mcm3, Ruvbl2, Etv5, Psip1, Rbbp4, Ifi205, Tgif1, Ilf2, Cebpz, Gmeb1, Eid1, Zfp422, Ddx20, 2410016O06Rik, Dlx1, Ets1, Sf1, Ssrp1, Sin3a, Rbm14, Med13, Timeless, 2700050L05Rik, Pprc1, Hltf, Hells, Med4, Suv39h1, Sltm, Brd8, Sub1, Med14, Mcm7, Prim1, Med13l, Zfp532, Fus, Nrf1, Nfkbiz, Arhgap22, Ahctf1, Khsrp, Zfp184, Rnf12, Polr1e, Junb, Nsbp1, Zfp451, Sox4, Nr2f2, Klf5, Khdrbs1, Th1l, Zfp770, Elk3, Tcfe2a, Foxp1, Mybl2, Pnn, Tgif2, Ell, Bclaf1, Suz12, Casp8ap2, Rbpj, Ash2l, Mybbp1a, Lcorl, Lif, Whsc2, Etv6, Tead2, Cdc5l, Mcm6, Gata2, Ctcf, Hmga1, Fubp1, Prmt5, Sox11, Hnrnpd, Snw1, Cbx2, Suv39h2, Sirt1, Rest, E2f8
**GO:0006355: regulation of transcription, DNA-dependent**	185(1215)	1,39E-36	Crebzf, Hmga2, Ell2, Rbbp7, Ruvbl1, Gli2, Tfdp1, Gtf2h2, Cbx3, Esf1, Asxl3, Myc, Elf2, Mcm2, Chaf1b, Zfp57, Pou2f1, Gli3, Gpbp1, Narg1, Litaf, Etv4, 2210018M11Rik, Mycn, Ilf3, Runx1, Mlf1ip, Egr1, Taf5, Tbx3, Arid4b, Uhrf1, Dmap1, Elp2, Ecd, 3930401K13Rik, Hat1, Vps36, Sfpq, Atrx, Bach1, Baz1a, Rnf2, Satb2, Runx2, Ets2, Tcerg1, Bnc2, Mcm4, Trim27, Fhl2, Fosl1, Cand1, Tcf4, Sertad1, Asf1b, Taf5l, Actl6a, Tfam, Mcm5, Egr2, Top2a, Nfx1, Dnmt1, Ezh2, Cenpk, Asf1a, Psrc1, Aebp2, Atad2, Rbl1, Whsc1, Mbtps2, Trim24, Cnot6, Snapc3, Ebf2, Hdac2, Trim28, Smarca5, Six4, Nono, Sap30, E2f7, Phf6, Tardbp, Ctbp2, Nab2, Pspc1, Smarcc1, Mkx, Cdca7l, Zfp280c, Phf17, Hnrpdl, Foxm1, 1110005A23Rik, Egr3, Zik1, Cdca7, Mcm3, Ruvbl2, Etv5, Psip1, Rbbp4, Ifi205, Tgif1, Ilf2, Cebpz, Gmeb1, Eid1, Zfp422, Ddx20, 2410016O06Rik, Dlx1, Ets1, Sf1, Ssrp1, Sin3a, Rbm14, Med13, Timeless, 2700050L05Rik, Pprc1, Hltf, Hells, Med4, Suv39h1, Sltm, Brd8, Sub1, Med14, Mcm7, Med13l, Zfp532, Fus, Nrf1, Nfkbiz, Arhgap22, Ahctf1, Khsrp, Zfp184, Rnf12, Junb, Nsbp1, Zfp451, Sox4, Nr2f2, Klf5, Khdrbs1, Th1l, Zfp770, Elk3, Tcfe2a, Foxp1, Mybl2, Pnn, Tgif2, Ell, Bclaf1, Suz12, Casp8ap2, Rbpj, Ash2l, Mybbp1a, Lcorl, Lif, Whsc2, Etv6, Tead2, Cdc5l, Mcm6, Gata2, Ctcf, Hmga1, Fubp1, Prmt5, Sox11, Hnrnpd, Snw1, Cbx2, Suv39h2, Sirt1, Rest, E2f8
**GO:0006351: transcription, DNA-dependent**	186(1215)	1,40E-36	Crebzf, Hmga2, Ell2, Rbbp7, Ruvbl1, Gli2, Tfdp1, Gtf2h2, Cbx3, Esf1, Asxl3, Myc, Elf2, Mcm2, Chaf1b, Zfp57, Pou2f1, Gli3, Gpbp1, Narg1, Litaf, Etv4, 2210018M11Rik, Mycn, Ilf3, Runx1, Mlf1ip, Egr1, Taf5, Tbx3, Arid4b, Uhrf1, Dmap1, Elp2, Ecd, 3930401K13Rik, Hat1, Vps36, Sfpq, Atrx, Bach1, Baz1a, Rnf2, Satb2, Runx2, Ets2, Tcerg1, Bnc2, Mcm4, Trim27, Fhl2, Fosl1, Cand1, Tcf4, Sertad1, Asf1b, Taf5l, Actl6a, Tfam, Mcm5, Egr2, Top2a, Nfx1, Dnmt1, Ezh2, Cenpk, Asf1a, Psrc1, Aebp2, Atad2, Rbl1, Whsc1, Mbtps2, Trim24, Cnot6, Snapc3, Ebf2, Hdac2, Trim28, Smarca5, Six4, Nono, Sap30, E2f7, Phf6, Tardbp, Ctbp2, Nab2, Pspc1, Smarcc1, Mkx, Cdca7l, Zfp280c, Phf17, Hnrpdl, Foxm1, 1110005A23Rik, Egr3, Zik1, Cdca7, Mcm3, Ruvbl2, Etv5, Psip1, Rbbp4, Ifi205, Tgif1, Ilf2, Cebpz, Gmeb1, Eid1, Zfp422, Ddx20, 2410016O06Rik, Dlx1, Ets1, Sf1, Ssrp1, Sin3a, Rbm14, Med13, Timeless, 2700050L05Rik, Pprc1, Hltf, Hells, Med4, Suv39h1, Sltm, Brd8, Sub1, Med14, Mcm7, Med13l, Zfp532, Fus, Nrf1, Nfkbiz, Arhgap22, Ahctf1, Khsrp, Zfp184, Rnf12, Polr1e, Junb, Nsbp1, Zfp451, Sox4, Nr2f2, Klf5, Khdrbs1, Th1l, Zfp770, Elk3, Tcfe2a, Foxp1, Mybl2, Pnn, Tgif2, Ell, Bclaf1, Suz12, Casp8ap2, Rbpj, Ash2l, Mybbp1a, Lcorl, Lif, Whsc2, Etv6, Tead2, Cdc5l, Mcm6, Gata2, Ctcf, Hmga1, Fubp1, Prmt5, Sox11, Hnrnpd, Snw1, Cbx2, Suv39h2, Sirt1, Rest, E2f8
**GO:0045934: negative regulation of nucleobase, nucleoside, nucleotide and nucleic acid metabolic process**	48(1215)	5,23E-15	Rbbp7, Gli2, Zfp57, Pou2f1, Gli3, Egr1, Tbx3, Msh6, Dmap1, Blm, Hat1, Rnf2, Satb2, Trim27, Tcf4, Nfx1, Dnmt1, Clspn, Patz1, Rbl1, Trim28, Sap30, Ctbp2, Nab2, Gmnn, Tipin, Tgif1, Eid1, Ddx20, Dlx1, Sin3a, Fusip1, Hus1, Hells, Suv39h1, Nr2f2, Khdrbs1, Th1l, Foxp1, Msh2, Bclaf1, Suz12, Mybbp1a, Cdt1, Cbx2, Npm1, Sirt1, Rest
**GO:0006310: DNA recombination**	25(1215)	1,43E-14	Ruvbl1, Kin, Rad54l, Recql4, Msh6, Rad51ap1, Blm, Trip13, Psmc3ip, Exo1, Nono, Rad51, Lig1, Ruvbl2, Rad51c, Topbp1, Smc5, Hus1, Mnd1, Smc6, Foxp1, Msh2, Ung, Swap70, Eme1
**GO:0010605: negative regulation of macromolecule metabolic process**	53(1215)	5,73E-13	Rbbp7, Gli2, Myc, Zfp57, Pou2f1, Gli3, Egr1, Tbx3, Msh6, Dmap1, Blm, Hat1, Rnf2, Satb2, Trim27, Stmn1, Tcf4, Nfx1, Dnmt1, Clspn, Patz1, Rbl1, Trim28, Sap30, Ctbp2, Nab2, Gmnn, Tipin, Tgif1, Eid1, Ddx20, Dlx1, Sin3a, Fusip1, Hus1, Hells, Suv39h1, Nr2f2, Khdrbs1, Th1l, Foxp1, Msh2, Bclaf1, Skp2, Suz12, Rdx, Mybbp1a, Cdt1, Cbx2, Npm1, Sirt1, Thex1, Rest
**GO:0006270: DNA replication initiation**	10(1215)	1,41E-11	Ccne2, Mcm2, Mcm4, Mcm5, Clspn, Cdc45l, Mcm3, Mcm7, Cdt1, Mcm6

Functional category	*Number of genes*	*Corrected p-value*	*Genes repressed in Rasless cells (from Table S1)*
**GO:0031324: negative regulation of cellular metabolic process**	50(1215)	2,09E-11	Rbbp7, Gli2, Zfp57, Pou2f1, Gli3, Egr1, Tbx3, Msh6, Dmap1, Blm, Hat1, Rnf2, Satb2, Trim27, Stmn1, Tcf4, Nfx1, Dnmt1, Clspn, Patz1, Rbl1, Trim28, Sap30, Ctbp2, Nab2, Gmnn, Tipin, Tgif1, Eid1, Ddx20, Dlx1, Sin3a, Fusip1, Hus1, Hells, Suv39h1, Nr2f2, Khdrbs1, Th1l, Foxp1, Msh2, Bclaf1, Suz12, Rdx, Mybbp1a, Cdt1, Cbx2, Npm1, Sirt1, Rest
**GO:0010558: negative regulation of macromolecule biosynthetic process**	43(1215)	2,69E-11	Rbbp7, Gli2, Zfp57, Pou2f1, Gli3, Egr1, Tbx3, Dmap1, Hat1, Rnf2, Satb2, Trim27, Tcf4, Nfx1, Dnmt1, Clspn, Patz1, Rbl1, Trim28, Sap30, Ctbp2, Nab2, Gmnn, Tipin, Tgif1, Eid1, Ddx20, Dlx1, Sin3a, Hus1, Hells, Suv39h1, Nr2f2, Khdrbs1, Th1l, Foxp1, Bclaf1, Suz12, Mybbp1a, Cdt1, Cbx2, Sirt1, Rest
**GO:0006364: rRNA processing**	19(1215)	4,07E-11	Wdr3, Nola1, Fbl, Ebna1bp2, Utp6, Utp11l, Mphosph10, Nob1, Dis3, Exosc8, Utp15, Dimt1, Rcl1, Imp3, Ddx51, Exosc2, Dkc1, Eif4a3, Emg1
**GO:0031327: negative regulation of cellular biosynthetic process**	43(1215)	5,35E-11	Rbbp7, Gli2, Zfp57, Pou2f1, Gli3, Egr1, Tbx3, Dmap1, Hat1, Rnf2, Satb2, Trim27, Tcf4, Nfx1, Dnmt1, Clspn, Patz1, Rbl1, Trim28, Sap30, Ctbp2, Nab2, Gmnn, Tipin, Tgif1, Eid1, Ddx20, Dlx1, Sin3a, Hus1, Hells, Suv39h1, Nr2f2, Khdrbs1, Th1l, Foxp1, Bclaf1, Suz12, Mybbp1a, Cdt1, Cbx2, Sirt1, Rest
**GO:0010629: negative regulation of gene expression**	41(1215)	6,50E-11	Rbbp7, Gli2, Myc, Zfp57, Pou2f1, Gli3, Egr1, Tbx3, Dmap1, Hat1, Rnf2, Satb2, Trim27, Tcf4, Nfx1, Dnmt1, Patz1, Rbl1, Trim28, Sap30, Ctbp2, Nab2, Tgif1, Eid1, Ddx20, Dlx1, Sin3a, Hells, Suv39h1, Nr2f2, Khdrbs1, Th1l, Foxp1, Bclaf1, Skp2, Suz12, Mybbp1a, Cbx2, Sirt1, Thex1, Rest
**GO:0009890: negative regulation of biosynthetic process**	43(1215)	6,76E-11	Rbbp7, Gli2, Zfp57, Pou2f1, Gli3, Egr1, Tbx3, Dmap1, Hat1, Rnf2, Satb2, Trim27, Tcf4, Nfx1, Dnmt1, Clspn, Patz1, Rbl1, Trim28, Sap30, Ctbp2, Nab2, Gmnn, Tipin, Tgif1, Eid1, Ddx20, Dlx1, Sin3a, Hus1, Hells, Suv39h1, Nr2f2, Khdrbs1, Th1l, Foxp1, Bclaf1, Suz12, Mybbp1a, Cdt1, Cbx2, Sirt1, Rest
**GO:0034470: ncRNA processing**	24(1215)	3,70E-10	Wdr3, Nola1, Fbl, Ebna1bp2, Utp6, Utp11l, Tyw3, Mphosph10, Nob1, Trit1, Dis3, Exosc8, Utp15, Dimt1, Rcl1, Trub1, Imp3, Ddx51, Exosc2, Dkc1, Nsun2, Eif4a3, Trmt6, Emg1
**GO:0016481: negative regulation of transcription**	38(1215)	7,67E-10	Rbbp7, Gli2, Zfp57, Pou2f1, Gli3, Egr1, Tbx3, Dmap1, Hat1, Rnf2, Satb2, Trim27, Tcf4, Nfx1, Dnmt1, Patz1, Rbl1, Trim28, Sap30, Ctbp2, Nab2, Tgif1, Eid1, Ddx20, Dlx1, Sin3a, Hells, Suv39h1, Nr2f2, Khdrbs1, Th1l, Foxp1, Bclaf1, Suz12, Mybbp1a, Cbx2, Sirt1, Rest
**GO:0034660: ncRNA metabolic process**	26(1215)	7,02E-09	Wdr3, Nola1, Fbl, Ebna1bp2, Utp6, Utp11l, Tyw3, Mphosph10, Nob1, Trit1, Dis3, Exosc8, Utp15, Dimt1, Rcl1, Trub1, Kars, Imp3, Ddx51, Exosc2, Dkc1, Nsun2, Eif4a3, Trmt6, Khsrp, Emg1
**GO:0051253: negative regulation of RNA metabolic process**	32(1215)	1,99E-08	Rbbp7, Gli2, Zfp57, Egr1, Tbx3, Dmap1, Hat1, Rnf2, Satb2, Trim27, Tcf4, Nfx1, Dnmt1, Rbl1, Trim28, Sap30, Ctbp2, Tgif1, Eid1, Ddx20, Dlx1, Sin3a, Fusip1, Hells, Suv39h1, Nr2f2, Foxp1, Suz12, Cbx2, Npm1, Sirt1, Rest
**GO:0045892: negative regulation of transcription, DNA-dependent**	30(1215)	2,36E-07	Rbbp7, Gli2, Zfp57, Egr1, Tbx3, Dmap1, Hat1, Rnf2, Satb2, Trim27, Tcf4, Nfx1, Dnmt1, Rbl1, Trim28, Sap30, Ctbp2, Tgif1, Eid1, Ddx20, Dlx1, Sin3a, Hells, Suv39h1, Nr2f2, Foxp1, Suz12, Cbx2, Sirt1, Rest
**GO:0040029: regulation of gene expression, epigenetic**	11(1215)	6,38E-07	H2afy, Hat1, H2afy2, Trim27, Dnmt1, Brca1, Mbd4, Hells, Suv39h1, Ctcf, Sirt1
**GO:0000245: spliceosome assembly**	7(1215)	6,85E-07	Sip1, Smndc1, Gemin6, Ptbp2, Fusip1, Cugbp1, Brunol4
**GO:0051053: negative regulation of DNA metabolic process**	8(1215)	1,58E-06	Msh6, Blm, Clspn, Gmnn, Tipin, Hus1, Msh2, Cdt1
**GO:0006275: regulation of DNA replication**	6(1215)	4,04E-05	Clspn, Gmnn, Tipin, Hus1, Pcna, Cdt1
**GO:0051052: regulation of DNA metabolic process**	9(1215)	0,000100778	Msh6, Blm, Clspn, Gmnn, Tipin, Hus1, Msh2, Pcna, Cdt1
**GO:0045941: positive regulation of transcription**	33(1215)	0,00013076	Gli2, Tfdp1, Myc, Pou2f1, Topors, Gpbp1, Etv4, Tbx3, Satb2, Runx2, Sertad1, Egr2, Top2a, Cenpk, Cdk2, Psrc1, Ebf2, Trim28, Six4, Foxm1, Etv5, Ets1, Med13, Med14, Fus, Ccna2, Sox4, Klf5, Lif, Tead2, Gata2, Sox11, Snw1
**GO:0008156: negative regulation of DNA replication**	5(1215)	0,000137054	Clspn, Gmnn, Tipin, Hus1, Cdt1
**GO:0043414: biopolymer methylation**	10(1215)	0,000137702	Ilf3, Dnmt1, Ezh2, Mbd4, Hells, Suv39h1, Suz12, Ctcf, Prmt5, Suv39h2
**GO:0010628: positive regulation of gene expression**	33(1215)	0,000156879	Gli2, Tfdp1, Myc, Pou2f1, Topors, Gpbp1, Etv4, Tbx3, Satb2, Runx2, Sertad1, Egr2, Top2a, Cenpk, Cdk2, Psrc1, Ebf2, Trim28, Six4, Foxm1, Etv5, Ets1, Med13, Med14, Fus, Ccna2, Sox4, Klf5, Lif, Tead2, Gata2, Sox11, Snw1
**GO:0045935: positive regulation of nucleobase, nucleoside, nucleotide and nucleic acid metabolic process**	33(1215)	0,00029428	Gli2, Tfdp1, Myc, Pou2f1, Topors, Gpbp1, Etv4, Tbx3, Satb2, Runx2, Sertad1, Egr2, Top2a, Cenpk, Cdk2, Psrc1, Ebf2, Trim28, Six4, Foxm1, Etv5, Ets1, Med13, Med14, Fus, Ccna2, Sox4, Klf5, Lif, Tead2, Gata2, Sox11, Snw1
**GO:0006376: mRNA splice site selection**	4(1215)	0,000405426	Ptbp2, Fusip1, Cugbp1, Brunol4

Functional category	*Number of genes*	*Corrected p-value*	*Genes repressed in Rasless cells (from Table S1)*
**GO:0006753: nucleoside phosphate metabolic process**	22(1215)	0,000476357	Impdh2, Prps1, 3930401K13Rik, Rrm1, Dctd, Dhfr, Dtymk, Gart, Adsl, Nme4, Rrm2, Nampt, Nme1, Tyms, Mthfd1, Dhodh, Umps, Msh2, Nudt1, Nt5c3l, Adss, Gmps
**GO:0031328: positive regulation of cellular biosynthetic process**	34(1215)	0,000637651	Gli2, Tfdp1, Myc, Pou2f1, Topors, Gpbp1, Etv4, Tbx3, Satb2, Runx2, Sertad1, Egr2, Top2a, Cenpk, Cdk2, Psrc1, Ebf2, Trim28, Six4, Foxm1, Etv5, Hsp90aa1, Ets1, Med13, Med14, Fus, Ccna2, Sox4, Klf5, Lif, Tead2, Gata2, Sox11, Snw1
**GO:0009891: positive regulation of biosynthetic process**	34(1215)	0,000670178	Gli2, Tfdp1, Myc, Pou2f1, Topors, Gpbp1, Etv4, Tbx3, Satb2, Runx2, Sertad1, Egr2, Top2a, Cenpk, Cdk2, Psrc1, Ebf2, Trim28, Six4, Foxm1, Etv5, Hsp90aa1, Ets1, Med13, Med14, Fus, Ccna2, Sox4, Klf5, Lif, Tead2, Gata2, Sox11, Snw1
**GO:0010557: positive regulation of macromolecule biosynthetic process**	33(1215)	0,000679675	Gli2, Tfdp1, Myc, Pou2f1, Topors, Gpbp1, Etv4, Tbx3, Satb2, Runx2, Sertad1, Egr2, Top2a, Cenpk, Cdk2, Psrc1, Ebf2, Trim28, Six4, Foxm1, Etv5, Ets1, Med13, Med14, Fus, Ccna2, Sox4, Klf5, Lif, Tead2, Gata2, Sox11, Snw1
**GO:0032392: DNA geometric change**	4(1215)	0,000831256	Mcm2, Mcm4, Mcm7, Mcm6
**GO:0031325: positive regulation of cellular metabolic process**	37(1215)	0,00092186	Gli2, Tfdp1, Myc, Pou2f1, Topors, Gpbp1, Etv4, Tbx3, Cd24a, Ghr, Satb2, Runx2, Sertad1, Egr2, Top2a, Cenpk, Cdk2, Psrc1, Ebf2, Trim28, Six4, Foxm1, Etv5, Hsp90aa1, Ets1, Med13, Med14, Fus, Ccna2, Sox4, Klf5, Lif, Tead2, Gata2, Sox11, Snw1, Bcl2
**GO:0010604: positive regulation of macromolecule metabolic process**	36(1215)	0,00117545	Gli2, Tfdp1, Myc, Pou2f1, Topors, Gpbp1, Etv4, Tbx3, Cd24a, Ghr, Satb2, Runx2, Sertad1, Egr2, Top2a, Cenpk, Cdk2, Psrc1, Ebf2, Trim28, Six4, Foxm1, Etv5, Ets1, Med13, Med14, Fus, Ccna2, Sox4, Klf5, Lif, Tead2, Gata2, Sox11, Snw1, Bcl2
**GO:0050684: regulation of mRNA processing**	3(1215)	0,00225694	Srpk1, Fusip1, Npm1
**GO:0045814: negative regulation of gene expression, epigenetic**	5(1215)	0,00427099	Hat1, Trim27, Hells, Suv39h1, Sirt1
**GO:0046112: nucleobase biosynthetic process**	3(1215)	0,00472822	Gart, Dhodh, Umps
**GO:0006206: pyrimidine base metabolic process**	3(1215)	0,00472822	Mapk1, Dhodh, Umps
**GO:0006304: DNA modification**	4(1215)	0,00500234	Dnmt1, Mbd4, Hells, Ctcf
**GO:0009112: nucleobase metabolic process**	4(1215)	0,00711648	Gart, Mapk1, Dhodh, Umps
**GO:0016458: gene silencing**	6(1215)	0,00826841	Hat1, Dnmt1, Hells, Suv39h1, Sirt1, Thex1
**GO:0045893: positive regulation of transcription, DNA-dependent**	25(1215)	0,0090893	Gli2, Tfdp1, Myc, Pou2f1, Gpbp1, Tbx3, Satb2, Runx2, Egr2, Top2a, Cenpk, Psrc1, Trim28, Six4, Foxm1, Ets1, Med13, Med14, Fus, Sox4, Lif, Tead2, Gata2, Sox11, Snw1
**GO:0051254: positive regulation of RNA metabolic process**	25(1215)	0,0093486	Gli2, Tfdp1, Myc, Pou2f1, Gpbp1, Tbx3, Satb2, Runx2, Egr2, Top2a, Cenpk, Psrc1, Trim28, Six4, Foxm1, Ets1, Med13, Med14, Fus, Sox4, Lif, Tead2, Gata2, Sox11, Snw1
**GO:0050686: negative regulation of mRNA processing**	2(1215)	0,010557	Fusip1, Npm1
**GO:0033119: negative regulation of RNA splicing**	2(1215)	0,010557	Fusip1, Npm1
**GO:0010216: maintenance of DNA methylation**	2(1215)	0,010557	Hells, Ctcf
**GO:0044030: regulation of DNA methylation**	2(1215)	0,010557	Hells, Ctcf
**GO:0000244: assembly of spliceosomal tri-snRNP**	2(1215)	0,010557	Lsm2, Prpf31
**GO:0034404: nucleobase, nucleoside and nucleotide biosynthetic process**	3(1215)	0,0123272	Gart, Dhodh, Umps
**GO:0045910: negative regulation of DNA recombination**	3(1215)	0,0172894	Msh6, Blm, Msh2
**GO:0006308: DNA catabolic process**	4(1215)	0,0173992	Myc, Casp3, Aifm1, Dffb

Functional category	Number of genes	Corrected p-value	Genes repressed in Rasless cells (from Table S1)
GO:0043484: regulation of RNA splicing	2(1215)	0,0256688	Fusip1, Npm1
GO:0006808: regulation of nitrogen utilization	2(1215)	0,0256688	Nmral1, Bcl2
GO:0006287: base-excision repair, gap-filling	2(1215)	0,0256688	Pold1, Pcna
GO:0009108: coenzyme biosynthetic process	7(1215)	0,0332636	Pdss1, Gphn, Pank1, Nampt, Mthfd1, Acly, Pank4
GO:0006269: DNA replication, synthesis of RNA primer	2(1215)	0,0437819	Prim2, Prim1
GO:0045005: maintenance of fidelity during DNA-dependent DNA replication	3(1215)	0,0482499	Msh6, Pold1, Msh2
**METABOLITE PROCESSING (PROTEIN, CARBOHYDRATE AND LIPID PROCESSING)**			
GO:0044267: cellular protein metabolic process	186(1215)	4,24E-18	Bcl10, Csnk1g3, Map4k5, Mcpt8, Wsb1, Igf2bp1, Ubqln2, Herc5, Ctsw, Myc, Etf1, Rps9, Uba2, Pum2, Ppif, Topors, Dnaja2, Hbs1l, Srpk1, Cct8, Prpf19, Prpf4b, Ilf3, Bub1b, Metap2, Cdc25c, Abl2, Casp3, BC057552, Pak1, Ttk, Metap1, Vrk3, Nek2, Riok2, Uhrf1, Cd24a, Dusp4, Ppwd1, Melk, Fbxo32, Fbxo5, Hat1, Mrpl19, Ghr, Sbk1, Rnf2, Adamts7, Abi1, Ppil1, Ttll4, Stmn1, Dtl, Pkmyt1, Cand1, Rpl41, Pigf, Mrpl1, Sumo2, Cdc25a, Cdc2a, Ube2t, Nfx1, Prkd1, Ezh2, Cdk2, Dusp6, Ccnb1, Socs5, Psrc1, Stt3b, Arpc5, Prkar2b, Rps13, Ptpre, Pdk3, Ptrh2, Socs3, Brcc3, B3galnt1, Mbtps2, Tpp2, Cct3, Hdac2, Cdc7, Prkg2, Hspa8, Lrrk1, Eif4h, Usp37, Ctsh, Espl1, Actr3, Dnaja1, Rpl3, Parp1, Plk4, Casp2, Nat13, Mapk8, Kars, 1110005A23Rik, Rad18, Pctk2, Ptk7, Eif5, Mapk1, Nek6, Asph, Hsp90aa1, Pkn3, Ptpn2, Gsg2, Uchl5, Mrps6, Vrk1, Rpl7l1, Fkbp5, Ptpn12, Socs6, Cit, Sf3a1, Plk1, Bub1, Denr, Vbp1, Aurka, Ube2c, Cacybp, Cdca3, Ube2e3, Erap1, Eef1g, Hus1, Cct7, Csnk1a1, Cct2, Ppp1cc, Pdgfb, Suv39h1, Hspe1, Chek1, Stk17b, Trmt6, Pim3, Usp34, Usp14, Pbk, Khsrp, Rnf12, Egfr, Sox4, Nedd4l, B230120H23Rik, Hspd1, 6030408C04Rik, Ppid, Wee1, Eif2s1, Cct5, Trib1, Usp1, Skp2, Paip1, Suz12, Tll1, Sgk1, Mast4, Chuk, Rdx, Gspt1, Usp3, Yme1l1, Casp6, Lif, Socs4, Ppih, Casp8, Ripk2, Aurkb, Cdc20, Prmt5, Bcl2, Suv39h2, Mastl, Sirt1
GO:0006464: protein modification process	99(1215)	6,05E-11	Bcl10, Csnk1g3, Map4k5, Ubqln2, Herc5, Myc, Uba2, Srpk1, Prpf19, Prpf4b, Ilf3, Bub1b, Cdc25c, Abl2, Pak1, Ttk, Vrk3, Nek2, Riok2, Uhrf1, Cd24a, Dusp4, Melk, Hat1, Ghr, Sbk1, Rnf2, Abi1, Ttll4, Pkmyt1, Pigf, Sumo2, Cdc25a, Cdc2a, Ube2t, Prkd1, Ezh2, Cdk2, Dusp6, Ccnb1, Stt3b, Prkar2b, Ptpre, Pdk3, Socs3, B3galnt1, Hdac2, Cdc7, Prkg2, Lrrk1, Parp1, Plk4, Nat13, Mapk8, Pctk2, Ptk7, Mapk1, Nek6, Asph, Pkn3, Ptpn2, Gsg2, Vrk1, Ptpn12, Cit, Sf3a1, Plk1, Bub1, Aurka, Ube2c, Ube2e3, Hus1, Csnk1a1, Ppp1cc, Pdgfb, Suv39h1, Chek1, Stk17b, Pim3, Usp14, Pbk, Egfr, Nedd4l, B230120H23Rik, 6030408C04Rik, Wee1, Trib1, Suz12, Sgk1, Mast4, Chuk, Lif, Ripk2, Aurkb, Prmt5, Bcl2, Suv39h2, Mastl, Sirt1
GO:0016310: phosphorylation	65(1215)	8,04E-09	Bcl10, Csnk1g3, Map4k5, Srpk1, Prpf4b, Bub1b, Abl2, Pak1, Ttk, Vrk3, Nek2, Riok2, Cd24a, Melk, Ghr, Sbk1, Abi1, Pkmyt1, Ipmk, Cdc2a, Pik3c2a, Prkd1, Cdk2, Ccnb1, Prkar2b, Ptpre, Pdk3, Socs3, Cdc7, Prkg2, Lrrk1, Plk4, Mapk8, Pctk2, Ptk7, Mapk1, Nek6, Pkn3, Gsg2, Vrk1, Cit, Plk1, Bub1, Aurka, Hus1, Csnk1a1, Pdgfb, Chek1, Stk17b, Pim3, Pbk, Egfr, B230120H23Rik, Wee1, Msh2, Trib1, Sgk1, Fxn, Mast4, Chuk, Lif, Ripk2, Aurkb, Bcl2, Mastl
GO:0006457: protein folding	17(1215)	2,58E-05	Ppif, Dnaja2, Cct8, Ppwd1, Ppil1, Cct3, Hspa8, Dnaja1, Hsp90aa1, Fkbp5, Vbp1, Cct7, Cct2, Hspe1, Ppid, Cct5, Ppih
GO:0043632: modification-dependent macromolecule catabolic process	34(1215)	0,00212399	Wsb1, Herc5, Uba2, Topors, BC057552, Uhrf1, Fbxo32, Fbxo5, Rnf2, Dtl, Cand1, Sumo2, Ube2t, Nfx1, Socs5, Socs3, Brcc3, Usp37, Rad18, Uchl5, Socs6, Ube2c, Cacybp, Cdca3, Ube2e3, Usp34, Usp14, Rnf12, Nedd4l, Usp1, Skp2, Usp3, Socs4, Cdc20
GO:0030163: protein catabolic process	36(1215)	0,00223634	Wsb1, Herc5, Uba2, Topors, BC057552, Uhrf1, Fbxo32, Fbxo5, Rnf2, Xpo1, Dtl, Cand1, Sumo2, Ube2t, Nfx1, Socs5, Socs3, Brcc3, Usp37, Rad18, Uchl5, Socs6, Ube2c, Cacybp, Cdca3, Ube2e3, Usp34, Usp14, Rnf12, Nedd4l, Usp1, Skp2, Usp3, Yme1l1, Socs4, Cdc20
GO:0034962: cellular biopolymer catabolic process	34(1215)	0,00283434	Wsb1, Herc5, Uba2, Topors, BC057552, Uhrf1, Fbxo32, Fbxo5, Rnf2, Dtl, Cand1, Sumo2, Ube2t, Nfx1, Socs5, Socs3, Brcc3, Usp37, Rad18, Uchl5, Socs6, Ube2c, Cacybp, Cdca3, Ube2e3, Usp34, Usp14, Rnf12, Nedd4l, Usp1, Skp2, Usp3, Socs4, Cdc20
GO:0044257: cellular protein catabolic process	34(1215)	0,00283434	Wsb1, Herc5, Uba2, Topors, BC057552, Uhrf1, Fbxo32, Fbxo5, Rnf2, Dtl, Cand1, Sumo2, Ube2t, Nfx1, Socs5, Socs3, Brcc3, Usp37, Rad18, Uchl5, Socs6, Ube2c, Cacybp, Cdca3, Ube2e3, Usp34, Usp14, Rnf12, Nedd4l, Usp1, Skp2, Usp3, Socs4, Cdc20
GO:0051246: regulation of protein metabolic process	24(1215)	0,00492162	Igf2bp1, Pum2, Cd24a, Ghr, Xpo1, Stmn1, Ube2t, Psrc1, Arpc5, Prkar2b, Socs3, Prkg2, Actr3, 1110005A23Rik, Ube2c, Ube2e3, Pdgfb, Trmt6, Egfr, Eif2s1, Paip1, Rdx, Lif, Bcl2
GO:0032268: regulation of cellular protein metabolic process	20(1215)	0,00942216	Igf2bp1, Pum2, Cd24a, Ghr, Stmn1, Psrc1, Arpc5, Prkar2b, Socs3, Prkg2, Actr3, 1110005A23Rik, Pdgfb, Trmt6, Egfr, Eif2s1, Paip1, Rdx, Lif, Bcl2
GO:0006412: translation	24(1215)	0,00946441	Igf2bp1, Etf1, Rps9, Pum2, Hbs1l, Mrpl19, Rpl41, Mrpl1, Rps13, Ptrh2, Eif4h, Rpl3, Kars, 1110005A23Rik, Eif5, Mrps6, Rpl7l1, Denr, Eef1g, Trmt6, Khsrp, Eif2s1, Paip1, Gspt1

Functional category	*Number of genes*	*Corrected p-value*	*Genes repressed in Rasless cells (from Table S1)*
**GO:0010608: posttranscriptional regulation of gene expression**	11(1215)	0,0126468	Igf2bp1, Pum2, 1110005A23Rik, Trmt6, Zfp36l2, Sox4, Elavl1, Eif2s1, Paip1, Hnrnpd, Bcl2
**GO:0016311: dephosphorylation**	12(1215)	0,0137697	Synj2, Cdc25c, Dusp4, Cdc25a, Dusp6, Ptpre, Ptpn2, Mtmr4, Ptpn12, Ppp1cc, Mtm1, Bcl2
**GO:0006020: inositol metabolic process**	4(1215)	0,0139146	Ipmk, Slc5a3, Impa1, Impa2
**GO:0006508: proteolysis**	50(1215)	0,0265613	Mcpt8, Wsb1, Herc5, Ctsw, Uba2, Topors, Metap2, Casp3, BC057552, Metap1, Uhrf1, Fbxo32, Fbxo5, Rnf2, Adamts7, Dtl, Cand1, Sumo2, Ube2t, Nfx1, Socs5, Socs3, Brcc3, Mbtps2, Tpp2, Usp37, Ctsh, Espl1, Casp2, Rad18, Uchl5, Socs6, Ube2c, Cacybp, Cdca3, Ube2e3, Erap1, Usp34, Usp14, Rnf12, Nedd4l, Usp1, Skp2, Tll1, Usp3, Yme1l1, Casp6, Socs4, Casp8, Cdc20
**CELL CYCLE (MITOTIC AND MEIOTIC DIVISION)**			
**GO:0000279: M phase**	98(1215)	2,52E-68	Hmga2, Mki67, Ruvbl1, Kif11, Cdc27, Bub3, Ncapg2, Smc3, Cep55, Bub1b, Tubb5, Cdc25c, Pcnt, Terf1, Fanca, Nek2, Rcc1, Pttg1, 6720463M24Rik, Fbxo5, Nusap1, Fancd2, Ndc80, 1110001A07Rik, Cdca2, Trip13, Stmn1, Cdca5, Smc2, Cdc25a, Spag5, Ncaph2, Cdc2a, Sgol1, Ccnb2, Cdk2, Ccnb1, Psmc3ip, Cd2ap, Ncapd3, Rad21, Exo1, Rad51, Akap8, Smc4, Nde1, Kif20b, Spc24, Espl1, Ranbp1, 2810433K01Rik, Spc25, Aspm, Tpx2, Zwilch, Tipin, Nek6, Rad51c, Dsn1, Nsl1, Ccnf, Topbp1, Cit, Plk1, Bub1, Ncaph, Ube2c, Mre11a, Cdca3, Timeless, Anln, Hells, Cenpj, Nuf2, Mad2l1, Sgol2, Incenp, Mnd1, Cenph, Ccna2, Wee1, Rcc2, Tacc3, Zw10, Nudc, Lif, Kntc1, Stag1, Cdca8, Cks2, Cdc6, Aurkb, Birc5, Ncapd2, Cdc20, Ercc6l, Ccng2, Suv39h2
**GO:0007067: mitosis**	75(1215)	1,49E-57	Hmga2, Ruvbl1, Kif11, Cdc27, Bub3, Ncapg2, Smc3, Cep55, Bub1b, Cdc25c, Terf1, Nek2, Rcc1, Pttg1, 6720463M24Rik, Fbxo5, Nusap1, Ndc80, 1110001A07Rik, Cdca2, Cdca5, Smc2, Cdc25a, Spag5, Ncaph2, Cdc2a, Sgol1, Ccnb2, Cdk2, Ccnb1, Cd2ap, Ncapd3, Rad21, Akap8, Smc4, Nde1, Kif20b, Spc24, 2810433K01Rik, Spc25, Aspm, Zwilch, Tipin, Nek6, Dsn1, Nsl1, Ccnf, Cit, Plk1, Bub1, Ncaph, Ube2c, Cdca3, Timeless, Anln, Hells, Nuf2, Mad2l1, Incenp, Cenph, Ccna2, Wee1, Rcc2, Zw10, Nudc, Kntc1, Stag1, Cdca8, Cdc6, Aurkb, Birc5, Ncapd2, Cdc20, Ercc6l, Ccng2
**GO:0000087: M phase of mitotic cell cycle**	75(1215)	2,18E-57	Hmga2, Ruvbl1, Kif11, Cdc27, Bub3, Ncapg2, Smc3, Cep55, Bub1b, Cdc25c, Terf1, Nek2, Rcc1, Pttg1, 6720463M24Rik, Fbxo5, Nusap1, Ndc80, 1110001A07Rik, Cdca2, Cdca5, Smc2, Cdc25a, Spag5, Ncaph2, Cdc2a, Sgol1, Ccnb2, Cdk2, Ccnb1, Cd2ap, Ncapd3, Rad21, Akap8, Smc4, Nde1, Kif20b, Spc24, 2810433K01Rik, Spc25, Aspm, Zwilch, Tipin, Nek6, Dsn1, Nsl1, Ccnf, Cit, Plk1, Bub1, Ncaph, Ube2c, Cdca3, Timeless, Anln, Hells, Nuf2, Mad2l1, Incenp, Cenph, Ccna2, Wee1, Rcc2, Zw10, Nudc, Kntc1, Stag1, Cdca8, Cdc6, Aurkb, Birc5, Ncapd2, Cdc20, Ercc6l, Ccng2
**GO:0000819: sister chromatid segregation**	13(1215)	7,10E-15	Bub3, Ncapg2, Nek2, Nusap1, Smc2, Ncaph2, Ncapd3, Akap8, Smc4, Cit, Ncaph, Mad2l1, Ncapd2
**GO:0000070: mitotic sister chromatid segregation**	13(1215)	7,10E-15	Bub3, Ncapg2, Nek2, Nusap1, Smc2, Ncaph2, Ncapd3, Akap8, Smc4, Cit, Ncaph, Mad2l1, Ncapd2
**GO:0007126: meiosis**	21(1215)	2,09E-10	Mki67, Smc3, Fanca, Nek2, Pttg1, Fbxo5, Fancd2, Trip13, Sgol1, Psmc3ip, Exo1, Rad51, Espl1, Rad51c, Topbp1, Mre11a, Sgol2, Mnd1, Lif, Cks2, Suv39h2
**GO:0051327: M phase of meiotic cell cycle**	21(1215)	2,09E-10	Mki67, Smc3, Fanca, Nek2, Pttg1, Fbxo5, Fancd2, Trip13, Sgol1, Psmc3ip, Exo1, Rad51, Espl1, Rad51c, Topbp1, Mre11a, Sgol2, Mnd1, Lif, Cks2, Suv39h2
**GO:0007076: mitotic chromosome condensation**	9(1215)	8,57E-10	Ncapg2, Nusap1, Smc2, Ncaph2, Ncapd3, Akap8, Smc4, Ncaph, Ncapd2
**GO:0007051: spindle organization**	9(1215)	3,37E-08	Tubb5, Pcnt, Fbxo5, Stmn1, Espl1, Ranbp1, Tpx2, Cenpj, Tacc3
**GO:0010564: regulation of cell cycle process**	10(1215)	2,68E-05	Recql4, Fbxo5, Nusap1, Dbf4, Xpo1, Ranbp1, Mad2l1, Lif, Cdt1, Npm1
**GO:0007127: meiosis I**	9(1215)	2,84E-05	Pttg1, Fbxo5, Fancd2, Trip13, Rad51, Espl1, Rad51c, Topbp1, Cks2
**GO:0045132: meiotic chromosome segregation**	5(1215)	0,00024626	Pttg1, Sgol1, Espl1, Rad51c, Sgol2
**GO:0051298: centrosome duplication**	4(1215)	0,00040542 6	Xpo1, Brca1, Nde1, Npm1
**GO:0000075: cell cycle checkpoint**	7(1215)	0,00083036 4	Cdc2a, Clspn, Hus1, Chek1, Mad2l1, Msh2, Cdt1
**GO:0031570: DNA integrity checkpoint**	6(1215)	0,00090347 1	Cdc2a, Clspn, Hus1, Chek1, Msh2, Cdt1
**GO:0007131: reciprocal meiotic recombination**	4(1215)	0,00143294	Trip13, Rad51, Rad51c, Topbp1

Functional category	*Number of genes*	*Corrected p-value*	*Genes repressed in Rasless cells (from Table S1)*
**GO:0046605: regulation of centrosome cycle**	3(1215)	0,00225694	Xpo1, Ranbp1, Npm1
**GO:0007091: mitotic metaphase/anaphase transition**	4(1215)	0,00357849	Cdc27, Bub1b, Cit, Mad2l1
**GO:0045786: negative regulation of cell cycle**	11(1215)	0,00437747	Bcl10, Lin9, Bub1b, Casp3, Cdc73, Rbl1, Brca1, Gmnn, Mad2l1, Ctcf, Bcl2
**GO:0051325: interphase**	7(1215)	0,00708704	Tfdp1, Dbf4, Rbbp8, Chek1, Skp2, Cdt1, Bcl2
**GO:0051329: interphase of mitotic cell cycle**	7(1215)	0,00708704	Tfdp1, Dbf4, Rbbp8, Chek1, Skp2, Cdt1, Bcl2
**GO:0051225: spindle assembly**	3(1215)	0,00841623	Tubb5, Fbxo5, Tpx2
**GO:0007346: regulation of mitotic cell cycle**	8(1215)	0,00897067	Fbxo5, Nusap1, Dbf4, Cdc2a, Hus1, Mad2l1, Cdt1, Bcl2
**GO:0007100: mitotic centrosome separation**	2(1215)	0,010557	Kif11, Ranbp1
**GO:0051299: centrosome separation**	2(1215)	0,010557	Kif11, Ranbp1
**GO:0007062: sister chromatid cohesion**	3(1215)	0,0123272	Recql4, Rad51c, Sgol2
**GO:0051320: S phase**	3(1215)	0,0172894	Tfdp1, Dbf4, Cdt1
**GO:0000084: S phase of mitotic cell cycle**	3(1215)	0,0172894	Tfdp1, Dbf4, Cdt1
**GO:0010824: regulation of centrosome duplication**	2(1215)	0,0256688	Xpo1, Npm1
**GO:0045143: homologous chromosome segregation**	2(1215)	0,0256688	Pttg1, Espl1
**GO:0007140: male meiosis**	4(1215)	0,0263621	Fanca, Trip13, Rad51c, Suv39h2
**GO:0030953: spindle astral microtubule organization**	2(1215)	0,0437819	Cenpj, Tacc3
**GO:0000089: mitotic metaphase**	2(1215)	0,0437819	Fbxo5, Ndc80
**DNA DAMAGE RESPONSE**			
**GO:0006281: DNA repair**	55(1215)	6,54E-31	Neil3, Apex1, Gtf2h2, Kin, Chaf1b, Rad54l, Smc3, 2210018M11Rik, Prpf19, Hmgn1, Fanca, Msh6, Rad51ap1, Pttg1, Uhrf1, Blm, Sfpq, Atrx, Fancd2, Trip13, Fancm, Asf1a, Clspn, Brca1, Rad21, Exo1, Nono, Rad51, Parp1, Lig1, Rad18, Pold1, Ruvbl2, Topbp1, Smc5, Mbd4, Fancb, Ssrp1, Mre11a, Fen1, Hus1, Gen1, Chek1, Apex2, Fancc, Smc6, Setx, Msh2, Ung, Nudt1, Trpc2, Usp1, Pcna, Eme1, Esco2
**GO:0009411: response to UV**	7(1215)	0,00100671	Casp3, Hmgn1, Msh6, Mapk8, Hus1, Msh2, Bcl2
**CYTOSKELETON-REGULATED PROCESSES**			
**GO:0006325: establishment or maintenance of chromatin architecture**	50(1215)	1,94E-19	Rbbp7, Ruvbl1, Cbx3, H2afy, Mcm2, 2210018M11Rik, Chd1, Hmgn1, Arid4b, Dmap1, Hat1, H2afy2, Baz1a, Rnf2, Satb2, Cbx1, Cbx5, Asf1b, Set, Actl6a, Setdb2, Ezh2, Hist1h2ae, Nap1l1, Asf1a, H2afz, Aebp2, Rbl1, Whsc1, Smarce1, Hdac2, Smarca5, H2afv, Cenpa, Nasp, Smarcc1, Nap1l4, Ruvbl2, Rbbp4, Gsg2, 2410016O06Rik, Hltf, Hells, Suv39h1, Brd8, Suz12, Prmt5, Cbx2, Suv39h2, Sirt1
**GO:0034622: cellular macromolecular complex assembly**	41(1215)	6,43E-13	H2afy, Lsm2, Mcm2, Tnpo1, Tubb5, Pak1, Sip1, Fbxo5, H2afy2, Xpo1, Smndc1, Tuba1c, Stmn1, Asf1b, Set, Tfam, Hist1h2ae, Gemin6, Nap1l1, Asf1a, Psrc1, H2afz, Arpc5, Xpo7, Ptbp2, H2afv, Cenpa, Actr3, Nap1l4, Ipo7, Eif5, Denr, Fusip1, Hells, Tube1, Cugbp1, Prpf31, Trmt6, Eif2s1, Kpnb1, Brunol4
**GO:0030261: chromosome condensation**	10(1215)	1,60E-10	Ncapg2, Nusap1, Smc2, Ncaph2, Top2a, Ncapd3, Akap8, Smc4, Ncaph, Ncapd2
**GO:0000226: microtubule cytoskeleton organization**	19(1215)	3,84E-09	Kif11, Tubb5, Pcnt, Fbxo5, Nusap1, Xpo1, Stmn1, Psrc1, Brca1, Cenpe, Nde1, Espl1, Ranbp1, Tpx2, Cenpj, Nuf2, Tacc3, Birc5, Npm1
**GO:0006461: protein complex assembly**	21(1215)	5,55E-06	Bcl10, Tnpo1, Tubb5, Pak1, Ctnna1, Fbxo5, Pdss1, Rrm1, Xpo1, Tuba1c, Stmn1, Tfam, Psrc1, Arpc5, Xpo7, Actr3, Rrm2, Ipo7, 2410015N17Rik, Tube1, Kpnb1

Functional category	*Number of genes*	*Corrected p-value*	*Genes repressed in Rasless cells (from Table S1)*
GO:0051297: centrosome organization	6(1215)	1,06E-05	Kif11, Xpo1, Brca1, Nde1, Ranbp1, Npm1
GO:0006334: nucleosome assembly	12(1215)	0,00105232	H2afy, Mcm2, H2afy2, Asf1b, Set, Hist1h2ae, Nap1l1, Asf1a, H2afz, H2afv, Cenpa, Nap1l4
GO:0046785: microtubule polymerization	3(1215)	0,00472822	Fbxo5, Stmn1, Psrc1
GO:0032200: telomere organization	5(1215)	0,00524048	Terf1, Blm, Parp1, Tinf2, Tnks2
GO:0000723: telomere maintenance	5(1215)	0,00524048	Terf1, Blm, Parp1, Tinf2, Tnks2
GO:0033043: regulation of organelle organization	10(1215)	0,0165829	Recql4, Xpo1, Stmn1, Psrc1, Arpc5, Actr3, Ranbp1, Tacc3, Rdx, Npm1
GO:0051493: regulation of cytoskeleton organization	9(1215)	0,0236597	Xpo1, Stmn1, Psrc1, Arpc5, Actr3, Ranbp1, Tacc3, Rdx, Npm1
GO:0031113: regulation of microtubule polymerization	2(1215)	0,0437819	Stmn1, Psrc1
GO:0007018: microtubule-based movement	14(1215)	5,05E-05	Kif11, Tubb5, Kif21a, Kif20a, Kif4, Tuba1c, Kif22, Kif2a, Nde1, Kif20b, Kif2c, Ktn1, Tube1, Kif18a
**TRANSPORT (METABOLITES, IONS AND VESICLES)**			
GO:0050658: RNA transport	22(1215)	2,66E-17	Nup93, Nupl1, Pom121, Nup37, Fmr1, Nxf1, Nup43, Xpo1, Tmem48, Nup155, Xpo7, Magoh, Nup133, Fusip1, Khsrp, Khdrbs1, G3bp2, Hnrnpa1, Nup88, Nxt1, Nup54, Nup107
GO:0050657: nucleic acid transport	22(1215)	2,66E-17	Nup93,Nupl1,Pom121,Nup37,Fmr1,Nxf1,Nup43,Xpo1,Tmem48,Nup155,Xpo7,Magoh,Nup133,Fusip1,Khsrp, Khdrbs1,G3bp2,Hnrnpa1,Nup88,Nxt1,Nup54,Nup107
GO:0051028: mRNA transport	21(1215)	1,79E-16	Nup93, Nupl1, Pom121, Nup37, Fmr1, Nxf1, Nup43, Xpo1, Tmem48, Nup155, Xpo7, Magoh, Nup133, Fusip1, Khsrp, Khdrbs1, G3bp2, Hnrnpa1, Nup88, Nxt1, Nup54, Nup107
GO:0006913: nucleocytoplasmic transport	21(1215)	2,23E-09	Nup50, Gli3, Tnpo1, Nxf1, Ran, Xpo1, Nutf2, Nup155, Xpo7, Cep57, Ipo7, Nup133, Fusip1, Kpna2, Khdrbs1, Tacc3, Nxt1, Kpnb1, Mybbp1a, Pola2, Npm1
GO:0051169: nuclear transport	21(1215)	2,68E-09	Nup50, Gli3, Tnpo1, Nxf1, Ran, Xpo1, Nutf2, Nup155, Xpo7, Cep57, Ipo7, Nup133, Fusip1, Kpna2, Khdrbs1, Tacc3, Nxt1, Kpnb1, Mybbp1a, Pola2, Npm1
GO:0065002: intracellular protein transmembrane transport	13(1215)	4,27E-06	Nup93, Nupl1, Pom121, Nup37, Nup43, Tmem48, Nup155, Xpo7, Nup133, Nup88, Nup54, Nup107, Timm8a1
GO:0006606: protein import into nucleus	12(1215)	5,70E-05	Gli3, Tnpo1, Ran, Xpo1, Nutf2, Xpo7, Cep57, Ipo7, Kpna2, Tacc3, Kpnb1, Pola2
GO:0017038: protein import	13(1215)	5,89E-05	Gli3, Tnpo1, Ran, Xpo1, Nutf2, Xpo7, Cep57, Ipo7, Kpna2, Tacc3, Kpnb1, Pola2, Timm8a1
GO:0030705: cytoskeleton-dependent intracellular transport	15(1215)	8,62E-05	Kif11, Tubb5, Kif21a, Kif20a, Kif4, Tuba1c, Wipf1, Kif22, Kif2a, Nde1, Kif20b, Kif2c, Ktn1, Tube1, Kif18a
GO:0006886: intracellular protein transport	28(1215)	0,000181735	Nup93, Nupl1, Pom121, Gli3, Nup37, Tnpo1, Cd24a, Nup43, Ran, Xpo1, Tmem48, Nutf2, Nup155, Xpo7, Cep57, Ipo7, Nup133, Kpna2, Ap1s3, Tacc3, Nup88, Nxt1, Kpnb1, Pcna, Pola2, Nup54, Nup107, Timm8a1
GO:0006405: RNA export from nucleus	5(1215)	0,000654883	Nxf1, Nup133, Fusip1, Khdrbs1, Nxt1
GO:0006605: protein targeting	14(1215)	0,00307334	Gli3, Tnpo1, Ran, Xpo1, Nutf2, Xpo7, Cep57, Ipo7, Kpna2, Tacc3, Nxt1, Kpnb1, Pola2, Timm8a1
GO:0015031: protein transport	39(1215)	0,0130066	Nup93, Nupl1, Pom121, Gli3, Nup37, Tnpo1, Snx5, Cd24a, Nup43, Kif20a, Ran, Vps36, Xpo1, Tmem48, Nutf2, Snx7, Nup155, Xpo7, Arf6, Cep57, Nasp, Ipo7, Nup133, Sdad1, Rab8b, Kpna2, Rab15, Ap1s3, Tacc3, Zw10, Nup88, Nxt1, Kpnb1, Pcna, Pola2, Kif18a, Nup54, Nup107, Timm8a1
GO:0006406: mRNA export from nucleus	3(1215)	0,0247957	Nxf1, Nup133, Fusip1
**CELL DEATH**			

Functional category	Number of genes	Corrected p-value	Genes repressed in Rasless cells (from Table S1)
GO:0006915: apoptotic process	61(1215)	6,45E-08	Bcl10, Tfdp1, Rasa1, Alkbh1, Myc, Qars, Unc5c, Topors, Gli3, Litaf, Bub1b, Casp3, Bcl2l11, Gramd4, Ctnna1, 1810011O10Rik, Msh6, Cd24a, H2-K1, Itgav, Smndc1, Tial1, Sh3kbp1, Tnfaip8, Trp53bp2, Brca1, Arf6, Rad21, Casp2, Ckap2, Phf17, Mapk8, Nek6, Aifm1, Ddx20, Dlx1, Mbd4, Dffb, Card10, Hells, Sltm, Stk17b, Spp1, 4632434I11Rik, Api5, 6030408C04Rik, Msh2, Bclaf1, Tmem173, Skp2, Sgk1, Siva1, Casp8ap2, Casp6, Phlpp, Casp8, Ripk2, Birc5, Sgms1, Bcl2, Sirt1
GO:0042981: regulation of apoptosis	35(1215)	0,000918262	Bcl10, Rasa1, Alkbh1, Myc, Qars, Gli3, Casp3, Bcl2l11, Ctnna1, Msh6, Cd24a, H2-K1, Brca1, Casp2, Mapk8, Ddx20, Dlx1, Mbd4, Card10, Hells, Spp1, Api5, 6030408C04Rik, Msh2, Bclaf1, Skp2, Siva1, Casp8ap2, Casp6, Casp8, Ripk2, Birc5, Sgms1, Bcl2, Sirt1
GO:0030262: apoptotic nuclear changes	5(1215)	0,000938818	Myc, Casp3, Cd24a, Aifm1, Dffb
GO:0043067: regulation of programmed cell death	35(1215)	0,00115892	Bcl10, Rasa1, Alkbh1, Myc, Qars, Gli3, Casp3, Bcl2l11, Ctnna1, Msh6, Cd24a, H2K1, Brca1, Casp2, Mapk8, Ddx20, Dlx1, Mbd4, Card10, Hells, Spp1, Api5, 6030408C04Rik, Msh2, Bclaf1, Skp2, Siva1, Casp8ap2, Casp6, Casp8, Ripk2, Birc5, Sgms1, Bcl2, Sirt1
GO:0006309: DNA fragmentation during apoptosis	4(1215)	0,00357849	Myc, Casp3, Aifm1, Dffb
GO:0008637: apoptotic mitochondrial changes	5(1215)	0,00971074	Myc, Casp3, Cd24a, Aifm1, Bcl2
GO:0008634: negative regulation of survival gene product expression	2(1215)	0,010557	Myc, Skp2
GO:0043066: negative regulation of apoptosis	17(1215)	0,0108044	Rasa1, Myc, Qars, Casp3, Ctnna1, Casp2, Mapk8, Dlx1, Hells, Spp1, Api5, 6030408C04Rik, Msh2, Skp2, Birc5, Sgms1, Bcl2
GO:0043069: negative regulation of programmed cell death	17(1215)	0,012757	Rasa1, Myc, Qars, Casp3, Ctnna1, Casp2, Mapk8, Dlx1, Hells, Spp1, Api5, 6030408C04Rik, Msh2, Skp2, Birc5, Sgms1, Bcl2
GO:0043065: positive regulation of apoptosis	17(1215)	0,0132173	Bcl10, Myc, Casp3, Bcl2l11, Msh6, Cd24a, H2-K1, Brca1, Casp2, Mapk8, Ddx20, Mbd4, Bclaf1, Siva1, Casp8ap2, Casp6, Ripk2
GO:0043068: positive regulation of programmed cell death	17(1215)	0,0143433	Bcl10, Myc, Casp3, Bcl2l11, Msh6, Cd24a, H2-K1, Brca1, Casp2, Mapk8, Ddx20, Mbd4, Bclaf1, Siva1, Casp8ap2, Casp6, Ripk2
GO:0051402: neuron apoptosis	8(1215)	0,0256458	Rasa1, Alkbh1, Casp3, Aifm1, Dlx1, Msh2, Birc5, Bcl2
GO:0012502: induction of programmed cell death	13(1215)	0,0310716	Bcl10, Myc, Casp3, Msh6, Cd24a, H2-K1, Brca1, Casp2, Mapk8, Ddx20, Mbd4, Casp8ap2, Casp6
GO:0006917: induction of apoptosis	13(1215)	0,0310716	Bcl10, Myc, Casp3, Msh6, Cd24a, H2-K1, Brca1, Casp2, Mapk8, Ddx20, Mbd4, Casp8ap2, Casp6
GO:0070230: positive regulation of lymphocyte apoptosis	2(1215)	0,0437819	Myc, Cd24a
GO:0006916: anti-apoptosis	8(1215)	0,0441314	Myc, Qars, Hells, Spp1, Api5, Skp2, Birc5, Bcl2
DEVELOPMENTAL PROCESS			
GO:0043009: chordate embryonic development	36(1215)	5,46E-07	Bcl10, Gli2, Alkbh1, Nup50, Amd1, Flvcr1, Gli3, Vangl2, Ncapg2, Prpf19, Runx1, Bcl2l11, Tbx3, Satb2, Abi1, Ipmk, Ccnb2, Ccnb1, Brca1, Tgfbr3, Dab2, Gins1, Six4, Nasp, Ptk7, Tgif1, Dlx1, Rbbp8, Junb, 6030408C04Rik, Msh2, Ell, Casp8, Gata2, Sfrs1, Fzd3
GO:0009887: organ morphogenesis	50(1215)	3,57E-06	Gli2, Rasa1, Alkbh1, Myc, Pou2f1, Gli3, Vangl2, Narg1, Etv4, Runx1, Hmgn1, Bcl2l11, Tbx3, Recql4, Itgav, Satb2, Runx2, Prkar2b, Arf6, Tgfbr3, Tnnt2, Six4, Nab2, Smarcc1, Foxm1, Gmnn, Mapk1, Etv5, Ccnf, Zfp422, Dlx1, Erap1, Vegfc, Nrp1, Spry2, Egfl7, Arhgap22, Gjc1, Junb, Csf1, Nr2f2, Klf5, Elk3, Rbpj, Chuk, Lif, Casp8, Gata2, Bcl2, Fzd3
GO:0048534: hemopoietic or lymphoid organ development	28(1215)	0,000127534	Flvcr1, Runx1, Timp1, Bcl2l11, Egr1, Hmgb3, Cd24a, Blm, Ncaph2, Ccnb2, Socs5, Patz1, Tgfbr3, Six4, Plscr1, Hsp90aa1, Hells, Csf1, Sox4, Foxp1, Tacc3, Msh2, Rbpj, Chuk, Lif, Casp8, Sfxn1, Bcl2
GO:0001701: in utero embryonic development	21(1215)	0,00015669	Alkbh1, Amd1, Flvcr1, Ncapg2, Prpf19, Runx1, Bcl2l11, Tbx3, Ccnb2, Ccnb1, Tgfbr3, Dab2, Gins1, Nasp, Rbbp8, Junb, 6030408C04Rik, Msh2, Ell, Gata2, Sfrs1
GO:0045596: negative regulation of cell differentiation	18(1215)	0,000359221	Gli3, Prpf19, Hmgb3, Ctnna1, Cd24a, Ezh2, Socs5, Enpp1, Tgfbr3, Aspm, Mapk1, Cit, Dlx1, Nrp1, Rif1, Rbpj, Lif, Sirt1

Functional category	Number of genes	Corrected p-value	Genes repressed in Rasless cells (from Table S1)
GO:0016331: morphogenesis of embryonic epithelium	11(1215)	0,000500469	Bcl10, Gli2, Nup50, Vangl2, Ipmk, Scrib, Ptk7, Tgif1, Vegfc, Casp8, Fzd3
GO:0030097: hemopoiesis	25(1215)	0,00050089	Flvcr1, Runx1, Timp1, Egr1, Hmgb3, Cd24a, Blm, Ncaph2, Socs5, Patz1, Tgfbr3, Plscr1, Hsp90aa1, Hells, Csf1, Sox4, Foxp1, Tacc3, Msh2, Rbpj, Chuk, Lif, Casp8, Sfxn1, Bcl2
GO:0002521: leukocyte differentiation	18(1215)	0,00107533	Egr1, Hmgb3, Cd24a, Blm, Ncaph2, Socs5, Patz1, Hsp90aa1, Hells, Csf1, Sox4, Foxp1, Msh2, Rbpj, Chuk, Lif, Casp8, Bcl2
GO:0021915: neural tube development	10(1215)	0,00208215	Bcl10, Gli2, Nup50, Gli3, Vangl2, Ipmk, Ptk7, Tgif1, Casp8, Fzd3
GO:0001841: neural tube formation	8(1215)	0,00236787	Bcl10, Nup50, Vangl2, Ipmk, Ptk7, Tgif1, Casp8, Fzd3
GO:0042476: odontogenesis	8(1215)	0,00276026	Gli2, Gli3, Bcl2l11, Runx2, Zfp422, Dlx1, Csf1, Chuk
GO:0048514: blood vessel morphogenesis	18(1215)	0,00332274	Rasa1, Narg1, Runx1, Itgav, Foxm1, Erap1, Vegfc, Nrp1, Egfl7, Arhgap22, Gjc1, Junb, Nr2f2, Klf5, Elk3, Rbpj, Casp8, Gata2
GO:0001838: embryonic epithelial tube formation	8(1215)	0,00354989	Bcl10, Nup50, Vangl2, Ipmk, Ptk7, Tgif1, Casp8, Fzd3
GO:0001568: blood vessel development	20(1215)	0,00366604	Rasa1, Narg1, Runx1, Tbx3, Itgav, Tgfbr3, Foxm1, Erap1, Vegfc, Nrp1, Egfl7, Arhgap22, Gjc1, Junb, Nr2f2, Klf5, Elk3, Rbpj, Casp8, Gata2
GO:0030098: lymphocyte differentiation	14(1215)	0,00404807	Egr1, Hmgb3, Cd24a, Blm, Ncaph2, Socs5, Patz1, Hsp90aa1, Hells, Sox4, Foxp1, Msh2, Rbpj, Bcl2
GO:0001944: vasculature development	20(1215)	0,00425115	Rasa1, Narg1, Runx1, Tbx3, Itgav, Tgfbr3, Foxm1, Erap1, Vegfc, Nrp1, Egfl7, Arhgap22, Gjc1, Junb, Nr2f2, Klf5, Elk3, Rbpj, Casp8, Gata2
GO:0042475: odontogenesis of dentine-containing tooth	7(1215)	0,00708704	Gli2, Gli3, Bcl2l11, Runx2, Dlx1, Csf1, Chuk
GO:0007405: neuroblast proliferation	6(1215)	0,00714006	Ctnna1, Cd24a, Racgap1, Nde1, Aspm, Vegfc
GO:0016447: somatic recombination of immunoglobulin gene segments	6(1215)	0,00826841	Msh6, Exo1, Foxp1, Msh2, Ung, Swap70
GO:0007507: heart development	17(1215)	0,0102853	Gli2, Gli3, Vangl2, Casp3, Tbx3, Trp53bp2, Tgfbr3, Tnnt2, Pkp2, Nrp1, Gjc1, Cxadr, Sox4, Foxp1, Nrp2, Rbpj, Casp8
GO:0016445: somatic diversification of immunoglobulins	6(1215)	0,0108172	Msh6, Exo1, Foxp1, Msh2, Ung, Swap70
GO:0007281: germ cell development	10(1215)	0,0116951	Fbxo5, Trip13, Tial1, Cep57, Strbp, Cugbp1, Fancc, Msh2, Pebp1, Bcl2
GO:0060348: bone development	13(1215)	0,0126357	Gli2, Ptger4, Satb2, Runx2, Fhl2, Egr2, Enpp1, Nab2, Ptgs2, Mapk8, Spp1, Csf1, Bcl2
GO:0010721: negative regulation of cell development	6(1215)	0,0132927	Ctnna1, Cd24a, Tgfbr3, Cit, Dlx1, Nrp1
GO:0000902: cell morphogenesis	29(1215)	0,0134862	Gli2, Alkbh1, Vangl2, Etv4, Epb4.1, Pcnt, Pak1, Ctnna1, Cd24a, Jub, Ect2, Stmn1, Egr2, Ank3, Tgfbr3, Dab2, Trim28, Nck2, Ptk7, Cit, Slit2, Pdgfb, Nrp1, Klf5, Plxnb1, Rbpj, Rdx, Bcl2, Llgl1
GO:0007517: muscle development	16(1215)	0,0169252	Luc7l, Pak1, Gphn, Ezh2, Tgfbr3, Tnnt2, Six4, Cenpf, Mkx, Gjc1, Cxadr, Nr2f2, Foxp1, Lif, Bcl2, Sirt1
GO:0002200: somatic diversification of immune receptors	6(1215)	0,0170466	Msh6, Exo1, Foxp1, Msh2, Ung, Swap70
GO:0001525: angiogenesis	13(1215)	0,0179739	Narg1, Runx1, Itgav, Erap1, Vegfc, Nrp1, Egfl7, Arhgap22, Klf5, Elk3, Rbpj, Casp8, Gata2
GO:0048754: branching morphogenesis of a tube	8(1215)	0,0256458	Gli2, Gli3, Mycn, Tbx3, Timeless, Nrp1, Spry2, Bcl2
GO:0008584: male gonad development	5(1215)	0,0263281	Bcl2l11, Fanca, Sf1, Msh2, Bcl2
GO:0014706: striated muscle development	13(1215)	0,0266548	Luc7l, Pak1, Gphn, Ezh2, Tnnt2, Six4, Cenpf, Gjc1, Cxadr, Nr2f2, Foxp1, Bcl2, Sirt1

Functional category	*Number of genes*	*Corrected p-value*	*Genes repressed in Rasless cells (from Table S1)*
GO:0045766: positive regulation of angiogenesis	3(1215)	0,0312471	Runx1, Erap1, Gata2
GO:0001833: inner cell mass cell proliferation	3(1215)	0,0312471	Ncapg2, Prpf19, Gins1
GO:0007417: central nervous system development	21(1215)	0,0348952	Gli2, Unc5c, Gli3, Prpf19, Fmr1, Lrp8, Runx1, Tbx3, Atrx, Egr2, Trp53bp2, Nde1, Aspm, Dlx1, Zic1, Nr2f2, Tacc3, Rbpj, Gata2, Bcl2, Llgl1
GO:0048538: thymus development	4(1215)	0,0351876	Bcl2l11, Ccnb2, Six4, Bcl2
GO:0060284: regulation of cell development	11(1215)	0,0352321	Gli2, Prpf19, Ctnna1, Cd24a, Tgfbr3, Aspm, Cit, Dlx1, Nrp1, Plxnb1, Bcl2
GO:0022008: neurogenesis	29(1215)	0,0354277	Gli2, Alkbh1, Gli3, Etv4, Prpf19, Runx1, Pak1, Ctnna1, Cd24a, Satb2, Stmn1, Egr2, Ank3, Racgap1, Nde1, Nab2, Aspm, Cit, Dlx1, Slit2, Vegfc, Nrp1, Nr2f2, Tacc3, Plxnb1, Rbpj, Lif, Gata2, Bcl2
GO:0050767: regulation of neurogenesis	10(1215)	0,0382593	Gli2, Prpf19, Ctnna1, Cd24a, Aspm, Cit, Dlx1, Nrp1, Plxnb1, Bcl2
GO:0001738: morphogenesis of a polarized epithelium	3(1215)	0,0398206	Vangl2, Ptk7, Fzd3
GO:0030278: regulation of ossification	6(1215)	0,0423857	Ptger4, Runx2, Egr2, Enpp1, Csf1, Bcl2
GO:0001843: neural tube closure	5(1215)	0,0425756	Bcl10, Vangl2, Ptk7, Tgif1, Fzd3
GO:0060323: head morphogenesis	2(1215)	0,0437819	Flvcr1, Asph
GO:0030879: mammary gland development	5(1215)	0,0462557	Gli2, Gli3, Bcl2l11, Tbx3, Nme1
GO:0050768: negative regulation of neurogenesis	5(1215)	0,0462557	Ctnna1, Cd24a, Cit, Dlx1, Nrp1
GO:0007420: brain development	17(1215)	0,0472104	Gli2, Unc5c, Gli3, Lrp8, Tbx3, Atrx, Egr2, Nde1, Aspm, Dlx1, Zic1, Nr2f2, Tacc3, Rbpj, Gata2, Bcl2, Llgl1
SIGNALING			
GO:0007242: intracellular signaling cascade	68(1215)	2,92E-05	Bcl10, Wsb1, Ptger4, Myc, Srpk1, Rrad, Centd1, Casp3, Depdc1a, Msh6, Cd24a, Elp2, Rasa3, Spred1, Ran, Itgav, Ect2, Stmn1, Errfi1, Depdc1b, Cdc2a, Pik3c2a, Prkd1, Cdgap, Net1, Ncam1, Socs5, Socs3, Brca1, Racgap1, Arf6, Lrrk1, Dnaja1, Mapk8, Slc9a3r1, Arl4c, Mapk1, Aifm1, Tgif1, Gsg2, Socs6, Cit, Tiam2, Mbd4, Dffb, Hus1, Rab8b, Card10, Cenpj, Unc13c, Chek1, Usp6nl, Csf1, B230120H23Rik, Rab15, G3bp2, Dusp9, Msh2, Spred2, Casp8ap2, Chuk, Lif, Socs4, Hrbl, Casp8, Rapgef2, Bcl2, Npm1
GO:0042770: DNA damage response, signal transduction	8(1215)	0,00107629	Msh6, Cdc2a, Brca1, Mbd4, Hus1, Chek1, Msh2, Npm1
GO:0021776: smoothened signaling pathway involved in spinal cord motor neuron cell fate specification	2(1215)	0,010557	Gli2, Gli3
GO:0021775: smoothened signaling pathway involved in ventral spinal cord interneuron specification	2(1215)	0,010557	Gli2, Gli3
GO:0007224: smoothened signaling pathway	5(1215)	0,0340137	Gli2, Gli3, Ctnna1, Runx2, Zic1
GO:0009966: regulation of signal transduction	28(1215)	0,0450002	Bcl10, Centd1, Ctnna1, Cd24a, Elp2, Zcchc11, Rasa3, Spred1, Ect2, Runx2, Net1, Ncam1, Socs5, Socs3, Tgif1, Socs6, Tiam2, Cenpj, Zic1, Spry2, Usp6nl, Csf1, Sox4, Spred2, Lif, Socs4, Hrbl, Npm1
CELL PROLIFERATION AND GROWTH			
GO:0008284: positive regulation of cell proliferation	23(1215)	0,000182512	Gli2, Fgf7, Myc, Lifr, Mycn, Recql4, Cd24a, Blm, Runx2, Tial1, Cdca7l, Foxm1, Tgif1, Vegfc, Pdgfb, Egfr, Csf1, Foxp1, Suz12, Rbpj, Marcksl1, Lif, Bcl2
GO:0008285: negative regulation of cell proliferation	13(1215)	0,0466595	Gli3, Casp3, Ctnna1, Cd24a, Tgfbr3, Ifitm3, E2f7, Asph, Tgif1, Cxadr, Cdkn2c, Rbpj, Bcl2

Functional category	*Number of genes*	*Corrected p-value*	*Genes repressed in Rasless cells (from Table S1)*
**GO:0001558: regulation of cell growth**	14(1215)	0,00010982	Hmga2, Ruvbl1, Dmap1, Crim1, Sertad1, Actl6a, Socs5, Psrc1, Socs3, Socs6, Ube2e3, Brd8, Socs4, Bcl2
**CELL ADHESION**			
**GO:0022408: negative regulation of cell-cell adhesion**	4(1215)	0,00928665	Cd24a, Tgfbr3, Trim28, Rbpj
**GO:0022407: regulation of cell-cell adhesion**	4(1215)	0,0263621	Cd24a, Tgfbr3, Trim28, Rbpj
**GO:0060232: delamination**	3(1215)	0,0398206	Tgfbr3, Trim28, Rbpj
**PROTEIN LOCATION**			
**GO:0032507: maintenance of protein location in cell**	4(1215)	0,0173992	Cenpe, Tnrc6a, Nuf2, Tacc3
**GO:0034453: microtubule anchoring**	2(1215)	0,0256688	Cenpe, Nuf2
**GO:0008608: attachment of spindle microtubules to kinetochore**	2(1215)	0,0256688	Cenpe, Nuf2
**OTHERS**			
GO:0016446: somatic hypermutation of immunoglobulin genes	4(1215)	0,00236948	Msh6, Exo1, Msh2, Ung
GO:0001776: leukocyte homeostasis	6(1215)	0,0260607	Casp3, Bcl2l11, Cd24a, Ccnb2, Fancc, Bcl2

Table S2-KEGG. Functional annotation to KEGG PATHWAYS of the downregulated, differentially expressed genes of Rasless MEFs

KEGG Pathway	*Number of genes*	*Corrected p-value*	*Genes repressed in Rasless cells (from Table S1)*
**(KEGG) 03040 :Spliceosome**	43(1239)	1,24E-28	U2af1, Magoh, Prpf4, Sf3a1, Snrpb2, Isy1, Hnrnpc, Snrpd1, Hnrnpa3, Hnrnpk, U2af2, Snrpb, Prpf19, Snrpg, Tcerg1, Hnrnpm, Smndc1, Ppih, Hnrnpu, 2610101N10Rik, Thoc2, Hspa8, Dhx15, Rbm25, Eif4a3, Prpf40a, Snrpe, Cdc40, Snrpa1, Hnrnpa1, Sf3a3, Prpf38b, Rbmxrt, Ddx46, Rbm17, Prpf3, Prpf31, Snw1, Ppil1, Lsm2, Eftud2, Prpf38a, Cdc5l
**(KEGG) 04110 :Cell cycle**	40(1239)	1,52E-26	Cdkn2c, Cdc20, Mad2l1, Bub1, Smc3, Ccne2, Stag1, Mcm4, Ccna2, Rad21, Rbl1, Pttg1, Mcm2, Espl1, Tfdp1, Cdc7, Cdc25a, Chek1, Mcm3, Mcm6, Ccnb1, Skp2, Pkmyt1, Cdc27, Mcm7, Plk1, Hdac2, Mcm5, Myc, Pcna, Bub1b, Cdc6, Cdk2, Cdc25c, Bub3, Wee1, Ccne1, Ttk, Ccnb2, Dbf4
**(KEGG) 03030 :DNA replication**	24(1239)	6,31E-26	Prim1, Pold1, Lig1, Mcm4, Pole2, Rpa2, Mcm2, Pold3, Rfc2, Rfc4, Rfc3, Dna2, Mcm3, Rfc5, Mcm6, Pole, Mcm7, Mcm5, Pcna, Prim2, Pola2, Pola1, Fen1, Rnaseh2a
**(KEGG) 03013 :RNA transport**	39(1239)	6,57E-21	Magoh, Nup50, Strap, Nup133, Pop1, Nup155, Clns1a, Nup93, Rnps1, Prmt5, Nup54, Eif2s1, Nup43, Nup88, Rangap1, Eif5, Gemin6, Nup37, Seh1l, Paip1, Gemin4, Sip1, Ddx20, Thoc2, Xpo1, Sumo2, Pnn, Kpnb1, Nup107, Nxt1, Tacc3, Eif4a3, Upf3b, Ran, Nxf1, Pom121, Nupl1, Tmem48, Gemin8
**(KEGG) 03430 :Mismatch repair**	14(1239)	1,99E-14	Pold1, Pms2, Lig1, Msh2, Exo1, Rpa2, Pold3, Rfc2, Rfc4, Rfc3, Rfc5, Msh6, Pcna, Msh3
**(KEGG) 03008 :Ribosome biogenesis in eukaryotes**	23(1239)	2,62E-14	Mphosph10, Pwp2, Fbl, Utp6, Wdr75, Wdr36, Pop1, Riok2, Imp3, Cirh1a, Rcl1, Emg1, Wdr43, Xpo1, Dkc1, Utp15, Utp18, Nxt1, Nob1, Gnl3, Ran, Nxf1, Wdr3
**(KEGG) 00240 :Pyrimidine metabolism**	24(1239)	2,38E-13	Dctd, Tk1, Prim1, Pold1, Polr1e, Nme4, Rrm2, Pole2, Tyms, Pold3, Dut, Nme1, Umps, Pnpt1, Dck, Polr3b, Pole, Rrm1, Dtymk, Polr2b, Prim2, Pola2, Dhodh, Pola1
**(KEGG) 03410 :Base excision repair**	14(1239)	3,71E-11	Pold1, Apex2, Lig1, Mbd4, Pole2, Pold3, Apex1, Neil3, Pole, Ung, Parp1, Pcna, Fen1, Hmgb1
**(KEGG) 00230 :Purine metabolism**	27(1239)	2,17E-10	Prim1, Pold1, Polr1e, Nme4, Rrm2, Adsl, Ppat, Pole2, Pold3, Impdh2, Nme1, Pde1a, Adss, Pnpt1, Dck, Polr3b, Pole, Rrm1, Pgm1, Enpp1, Polr2b, Prim2, Pola2, Gmps, Pola1, Gart, Prps1
**(KEGG) 03015 :mRNA surveillance pathway**	18(1239)	1,05E-08	Cstf2, Magoh, Nudt21, Rnps1, Cpsf6, Cpsf4, Pnn, Etf1, Nxt1, Cpsf2, Rnmt, Eif4a3, Upf3b, Nxf1, Pcf11, Gspt1, Pabpn1, Hbs1l
**(KEGG) 03420 :Nucleotide excision repair**	13(1239)	1,56E-08	Pold1, Lig1, Pole2, Cul4b, Rpa2, Pold3, Rfc2, Rfc4, Rfc3, Rfc5, Pole, Pcna, Gtf2h2
**(KEGG) 04114 :Oocyte meiosis**	19(1239)	4,44E-08	Cdc20, Mad2l1, Bub1, Smc3, Ccne2, Mapk1, Pttg1, Aurka, Espl1, Sgol1, Fbxo5, Pkmyt1, Cdc27, Plk1, Ppp1cc, Cdk2, Cdc25c, Ccne1, Ccnb2
**(KEGG) 05200 :Pathways in cancer**	32(1239)	7,57E-07	Pdgfb, Ccne2, Cks1b, Mapk1, Vegfc, Hsp90aa1, Ptgs2, Casp8, Msh2, Gli2, Ctnna1, Birc5, Egfr, Chuk, Ctbp2, Fgf7, Rad51, Skp2, Runx1, Tpm3, Casp3, Itgav, Hdac2, Mapk8, Msh6, Myc, Gli3, Cdk2, Fzd3, Msh3, Ccne1, Bcl2
**(KEGG) 03440 :Homologous recombination**	9(1239)	1,88E-06	Pold1, Rad54l, Rpa2, Pold3, Rad51, Eme1, Mre11a, Rad51c, Blm
**(KEGG) 04914 :Progesterone-mediated oocyte maturation**	14(1239)	1,26E-05	Mad2l1, Bub1, Mapk1, Hsp90aa1, Ccna2, Cdc25a, Ccnb1, Pkmyt1, Cdc27, Plk1, Mapk8, Cdk2, Cdc25c, Ccnb2
**(KEGG) 04115 :p53 signaling pathway**	11(1239)	0,000154189	Ccne2, Casp8, Rrm2, Ccng2, Chek1, Ccnb1, Casp3, Cdk2, Ccne1, Gtse1, Ccnb2
**(KEGG) 03018 :RNA degradation**	11(1239)	0,000255786	Cnot6, Dhx36, Eno3, Dcp1a, Pnpt1, Dis3, Patl1, Exosc2, Exosc8, Lsm2, Hspd1
**(KEGG) 04320 :Dorso-ventral axis formation**	6(1239)	0,00067387	Notch1, Mapk1, Ets1, Etv6, Egfr, Ets2
**(KEGG) 04141 :Protein processing in endoplasmic reticulum**	16(1239)	0,00108902	Ckap4, Hsp90aa1, Mbtps2, Ssr4, Stt3b, Eif2s1, Ube2e3, Dnaja2, Sec63, Hspa4l, Pdia6, Hspa8, Mapk8, Ubqln2, Dnaja1, Bcl2

KEGG Pathway	*Number of genes*	*Corrected p-value*	*Genes repressed in Rasless cells (from Table S1)*
**(KEGG) 00270 :Cysteine and methionine metabolism**	7(1239)	0,00127375	Ahcy, Mtap, Amd1, Mat2a, Sms, Dnmt1, Ahcyl1
**(KEGG) 05210 :Colorectal cancer**	9(1239)	0,00213011	Mapk1, Msh2, Birc5, Casp3, Mapk8, Msh6, Myc, Msh3, Bcl2
**(KEGG) 04010 :MAPK signaling pathway**	21(1239)	0,00217084	Pdgfb, Mapk1, Cd14, Dusp9, Stmn1, Rasa1, Dusp5, Egfr, Chuk, Cacna1c, Fgf7, Hspa8, Pla2g4a, Casp3, Rapgef2, B230120H23Rik, Mapk8, Dusp6, Myc, Dusp4, Pak1
**(KEGG) 00670 :One carbon pool by folate**	5(1239)	0,00262784	Mthfd1, Tyms, Shmt1, Gart, Dhfr
**(KEGG) 05222 :Small cell lung cancer**	10(1239)	0,00428299	Ccne2, Cks1b, Ptgs2, Chuk, Skp2, Itgav, Myc, Cdk2, Ccne1, Bcl2
**(KEGG) 04630 :Jak-STAT signaling pathway**	13(1239)	0,0149375	Lifr, Socs4, Ghr, Osmr, Tslp, Lif, Socs5, Spred1, Spry2, Il13ra1, Myc, Spred2, Socs3
**(KEGG) 04210 :Apoptosis**	9(1239)	0,0155185	Aifm1, Casp8, Prkar2b, Dffb, Chuk, Il1rap, Casp3, Bcl2, Casp6
**(KEGG) 05215 :Prostate cancer**	9(1239)	0,0204867	Pdgfb, Ccne2, Mapk1, Hsp90aa1, Egfr, Chuk, Cdk2, Ccne1, Bcl2
**(KEGG) 05020 :Prion diseases**	5(1239)	0,0372889	Notch1, Ncam1, Mapk1, Stip1, Egr1
**(KEGG) 04330 :Notch signaling pathway**	6(1239)	0,0375309	Rbpj, Notch1, Kat2a, Ctbp2, Hdac2, Snw1
**(KEGG) 04120 :Ubiquitin mediated proteolysis**	11(1239)	0,0383463	Cdc20, Prpf19, Ube2e3, Cul4b, Skp2, Uba2, Brca1, Cdc27, Nedd4l, Ube2c, Socs3

Table S2-TF. Functional annotation to "TRANSCRIPTION FACTORS" of the downregulated, differentially expressed genes of Rasless MEFs

Transcription Factor	*Number of genes*	*Corrected p-value*	*Genes repressed in Rasless cells (from Table S1)*
**V$E2F1_Q6**	64(1239)	9,63E-50	Syncrip, Suv39h1, H2afz, Pold1, Stag1, Fancd2, Fancc, Snrpd1, Nolc1, Klf5, Arhgap11a, Rrm2, Mcm4, Msh2, Ctcf, Stmn1, Pole2, Rbl1, Nasp, Mcm2, Pold3, Usp37, Ilf3, Cdc25a, Cdt1, Casp8ap2, Cbx3, Soat1, Mcm3, Mcm6, Atad2, Topbp1, Dck, Fbxo5, Rasal2, Kpnb1, Pkmyt1, Clspn, Zcchc8, Gmnn, E2f7, Mcm7, Rbbp4, Hmga1, H2afv, Ung, Ezh2, Prpf4b, Myc, Ipo7, Tmpo, Pcna, Cdc6, Gprc5b, Gspt1, Cdca7, Slc9a5, Dnajc9, Ranbp1, Prps1, Dnmt1, Fkbp5, Ncl, Cdc5l
**V$SP1_Q6**	216(1239)	1,80E-49	Taf5,Cdkn2c,Gphn,Cct3,Zmym1,Cenph,Hyal2,Sf3a1,Notch1,Syncrip,Ncam1,Trib1,Rpl3,Pdgfb,Suv39h1,Ahcy,Top2a,H2afz,Arpc5,Fbl,Cks1b,Slc38a1,Usp1,Trim25,Slc11a2,G3bp2,Set,Acly,Stag1,Alad,Sox4,Khdrbs1,Zbtb12,Junb,Ell2,Nolc1,Klf5,Ptpn2,Cct2,Melk,Mtmr4,Sertad1,Lig1,Dlg3,Hnrpdl,Fmr1,Egr2,Slc43a1,Ssr4,Elovl6,Cbx5,Ctcf,Txnrd1,Exo1,Sox12,Cbfb,Smarce1,Trim28,Axl,Sgol2,Idh3a,Osmr,Eno3,Nutf2,Tbx3,Mat2a,Stmn1,Rasa1,Sin3a,Pole2,Cit,Dusp5,Fus,Anln,Nasp,Ppih,Pcm1,Usp37,Ing5,Rbm12,Rpl12,Sec63,Cnnm4,Nab2,Tead2,Eif5,Khsrp,Shmt1,Zfp36l2,Rbbp7,Uchl5,Ilf3,Pogz,Cdc25a,Cdca3,Denr,Rbbp8,Pou2f1,Ssb,Nr2f2,Rcl1,Hspe1,Hat1,Top1,Ccng2,Supt16h,Matr3,Chtf18,Egr3,Fmnl3,Slc9a3r1,Casp8ap2,Ptk7,Topors,Zic1,Pank4,Adss,Plk4,Etv5,Elk3,Pnpt1,Hspa8,Tdrkh,Xpo1,Smarca5,Nusap1,Ptpn12,Sumo2,Il13ra1,Hn1,Safb,Eef1g,Rasal2,Timeless,Cd2ap,Kpnb1,Nxt1,Rrm1,Sdc1,Brca1,Zcchc8,Cyp26b1,Usp3,Mcm7,Sh3kbp1,Rbbp4,Plk1,Sap30,Hmga1,Slc25a5,Net1,Dhx9,Upf3b,H2afv,Sox11,Fosl1,Pprc1,Cct7,Ddx10,Rnf138,Col7a1,Ezh2,Timp1,Cdc40,Myc,Pkn3,Pcf11,Ipo7,Tcf4,Has2,Mrpl1,Polr2b,Marcks,Npm1,Rnf2,Dpf1,Tmpo,Jub,Oip5,Bzw2,Swap70,Kif2c,Cdk2,Blm,Ubqln2,Cdca7,Dnaja1,Nrf1,Slc9a5,Ell,Scrib,Hmgn1,Msh3,Psip1,Ranbp1,Nrp2,Wee1,Prpf3,Pafah1b3,Tpp2,Ivns1abp,Fkbp5,Pak1,Ccne1,Six4,Lsm2,Hspd1,Dhfr,Ktn1,Spred2,Etv4,Slbp,Ythdf2,Akap8,Tpbg,Prc1
**V$E2F4DP1_01**	62(1239)	4,08E-46	Syncrip, Suv39h1, H2afz, Pold1, Stag1, Fancd2, Fancc, Snrpd1, Nolc1, Arhgap11a, Rrm2, Lig1, Mcm4, Msh2, Mat2a, Stmn1, Pole2, Rbl1, Nasp, Mcm2, Pold3, Usp37, Rbbp7, Ilf3, Cdc25a, Casp8ap2, Cbx3, Soat1, Mcm3, Mcm6, Atad2, Topbp1, Dck, Fbxo5, Rasal2, Pkmyt1, Clspn, Zcchc8, Gmnn, E2f7, Mcm7, Hmga1, H2afv, Ung, Ezh2, Prpf4b, Myc, Ipo7, Tmpo, Pcna, Cdc6, Gprc5b, Gspt1, Cdca7, Slc9a5, Dnajc9, Ranbp1, Prps1, Dnmt1, Fkbp5, Ncl, Cdc5l
**V$E2F_Q6**	60(1239)	2,97E-45	H2afz, Slc38a1, Pold1, Stag1, Fancd2, Snrpd1, Nolc1, Arhgap11a, Rrm2, Mcm4, Msh2, Cbx5, Ctcf, Stmn1, Pole2, Rbl1, Nasp, Mcm2, Pold3, Usp37, Ilf3, Cdc25a, Cdt1, Casp8ap2, Soat1, Mcm3, Mcm6, Atad2, Topbp1, Dck, Rad51, Fbxo5, Rasal2, Kpnb1, Pkmyt1, Clspn, Zcchc8, Gmnn, E2f7, Mcm7, Ung, Ezh2, Prpf4b, Ipo7, Tmpo, Pcna, Pola2, Cdc6, Ppp1cc, Gprc5b, Gspt1, Cdca7, Slc9a5, Dnajc9, Ranbp1, Nrp2, Wee1, Prps1, Dnmt1, Ncl
**V$E2F_Q4**	60(1239)	3,74E-45	H2afz, Chd2, Slc38a1, Pold1, Stag1, Fancd2, Snrpd1, Nolc1, Arhgap11a, Rrm2, Mcm4, Msh2, Cbx5, Ctcf, Stmn1, Pole2, Rbl1, Nasp, Mcm2, Pold3, Usp37, Ilf3, Cdc25a, Cdt1, Casp8ap2, Soat1, Mcm3, Mcm6, Atad2, Topbp1, Dck, Rad51, Fbxo5, Rasal2, Kpnb1, Pkmyt1, Clspn, Zcchc8, Gmnn, E2f7, Mcm7, Ung, Ezh2, Ipo7, Tmpo, Pcna, Pola2, Cdc6, Ppp1cc, Gprc5b, Gspt1, Cdca7, Slc9a5, Dnajc9, Ranbp1, Nrp2, Wee1, Prps1, Dnmt1, Ncl
**V$E2F_02**	60(1239)	5,71E-45	Syncrip, Suv39h1, H2afz, Pold1, Stag1, Fancd2, Fancc, Snrpd1, Nolc1, Arhgap11a, Rrm2, Dlg3, Mcm4, Msh2, Stmn1, Pole2, Rbl1, Nasp, Mcm2, Pold3, Usp37, Ilf3, Cdc25a, Casp8ap2, Cbx3, Soat1, Mcm3, Mcm6, Atad2, Topbp1, Dck, Fbxo5, Rasal2, Pkmyt1, Clspn, Zcchc8, Gmnn, E2f7, Mcm7, Hmga1, H2afv, Ung, Ezh2, Prpf4b, Myc, Ipo7, Tmpo, Pcna, Cdc6, Gprc5b, Gspt1, Cdca7, Slc9a5, Dnajc9, Ranbp1, Prps1, Dnmt1, Fkbp5, Ncl, Cdc5l
**V$E2F1DP1_01**	60(1239)	5,71E-45	Syncrip, Suv39h1, H2afz, Pold1, Stag1, Fancd2, Fancc, Snrpd1, Nolc1, Arhgap11a, Rrm2, Dlg3, Mcm4, Msh2, Stmn1, Pole2, Rbl1, Nasp, Mcm2, Pold3, Usp37, Ilf3, Cdc25a, Casp8ap2, Cbx3, Soat1, Mcm3, Mcm6, Atad2, Topbp1, Dck, Fbxo5, Rasal2, Pkmyt1, Clspn, Zcchc8, Gmnn, E2f7, Mcm7, Hmga1, H2afv, Ung, Ezh2, Prpf4b, Myc, Ipo7, Tmpo, Pcna, Cdc6, Gprc5b, Gspt1, Cdca7, Slc9a5, Dnajc9, Ranbp1, Prps1, Dnmt1, Fkbp5, Ncl, Cdc5l
**V$E2F4DP2_01**	60(1239)	5,71E-45	Syncrip, Suv39h1, H2afz, Pold1, Stag1, Fancd2, Fancc, Snrpd1, Nolc1, Arhgap11a, Rrm2, Dlg3, Mcm4, Msh2, Stmn1, Pole2, Rbl1, Nasp, Mcm2, Pold3, Usp37, Ilf3, Cdc25a, Casp8ap2, Cbx3, Soat1, Mcm3, Mcm6, Atad2, Topbp1, Dck, Fbxo5, Rasal2, Pkmyt1, Clspn, Zcchc8, Gmnn, E2f7, Mcm7, Hmga1, H2afv, Ung, Ezh2, Prpf4b, Myc, Ipo7, Tmpo, Pcna, Cdc6, Gprc5b, Gspt1, Cdca7, Slc9a5, Dnajc9, Ranbp1, Prps1, Dnmt1, Fkbp5, Ncl, Cdc5l
**V$E2F1DP2_01**	60(1239)	7,90E-45	Syncrip, Suv39h1, H2afz, Pold1, Stag1, Fancd2, Fancc, Snrpd1, Nolc1, Arhgap11a, Rrm2, Dlg3, Mcm4, Msh2, Stmn1, Pole2, Rbl1, Nasp, Mcm2, Pold3, Usp37, Ilf3, Cdc25a, Casp8ap2, Cbx3, Soat1, Mcm3, Mcm6, Atad2, Topbp1, Dck, Fbxo5, Rasal2, Pkmyt1, Clspn, Zcchc8, Gmnn, E2f7, Mcm7, Hmga1, H2afv, Ung, Ezh2, Prpf4b, Myc, Ipo7, Tmpo, Pcna, Cdc6, Gprc5b, Gspt1, Cdca7, Slc9a5, Dnajc9, Ranbp1, Prps1, Dnmt1, Fkbp5, Ncl, Cdc5l
**V$E2F1DP1RB_01**	59(1239)	3,48E-44	H2afz, Slc38a1, Pold1, Snrpd1, Nolc1, Arhgap11a, Rrm2, Lig1, Msh2, Cbx5, Ctcf, Mat2a, Stmn1, Pole2, Nasp, Mcm2, Pold3, Usp37, Impdh2, Rbbp7, Ilf3, Cdc25a, Cdt1, Casp8ap2, Soat1, Mcm3, Mcm6, Atad2, Topbp1, Dck, Rad51, Fbxo5, Rasal2, Kpnb1, Pkmyt1, Clspn, Zcchc8, Gmnn, E2f7, Mcm7, Ung, Atrx, Ezh2, Ipo7, Tmpo, Pcna, Pola2, Cdc6, Ppp1cc, Gprc5b, Gspt1, Cdca7, Slc9a5, Dnajc9, Nrp2, Wee1, Prps1, Dnmt1, Ncl
**V$E2F_Q4_01**	59(1239)	4,17E-43	Suv39h1, H2afz, Prim1, Slc38a1, Stag1, Fancc, Klf5, Melk, Arhgap11a, Mcm4, Cbx5, Ctcf, Nutf2, Tbx3, Mat2a, Stmn1, Pole2, Nasp, Mcm2, Impdh2, Cdc25a, Top1, Casp2, Casp8ap2, Plk4, Mcm3, Mcm6, Atad2, Topbp1, Dck, Rad51, Fbxo5, Kntc1, Kpnb1, Pkmyt1, Zcchc8, Gmnn, E2f7, Mcm7, Rbbp4, Hmga1, Ung, Pprc1, Ezh2, Myc, Ipo7, Pcna, Pola2, Cdc6, Gprc5b, Cdca7, Ranbp1, Nrp2, Anp32e, Wee1, Dnmt1, Ncl, Etv4, Slbp
**V$LEF1_Q2**	194(1239)	1,38E-41	Abi1,Lifr,Ckap4,Ankrd28,Ank3,Sf1,Hyal2,Notch1,Uhrf1,Rbm14,Mmd,Syncrip,Trib1,Crlf1,Kif20a,Brd8,Elavl1,Chd2,Ccne2,Llgl1,Wdr5,Slc38a1,Set,Stag1,Ptbp2,Rcor1,Ets1,Sema4b,Khdrbs1,Casp8,Zbtb12,Etv6,Eps8,Cct2,Ddx31,Map4k5,Dlg3,Prkar2b,Hnrpdl,Fmr1,Ssr4,Slc43a3,Nipsnap1,Gli2,Xpo7,Ctcf,Rhobtb3,Txnrd1,Phc2,Cbfb,Smarce1,Glrx,Csnk1g3,Baz1a,Crim1,Tcerg1,Rest,Bach1,Tbx3,Mat2a,Stmn1,Syne2,Ube2e3,Myef2,Sfpq,Lmnb1,H2afy2,Kif21a,Fus,Tgif2,Rpa2,Pcm1,Foxp1,Dnaja2,Sec63,Fbln1,Tead2,Gata2,Eif5,Socs5,Ilf3,Cdc25a,Acsl3,Cdca3,Actr3,Chd1,Nr2f2,Slc5a3,Tial1,Luc7l,Top1,Ccng2,Gprc5c,Iqgap3,Pde1a,Sdpr,Paip1,Egr3,Asph,Mpzl1,Casp2,Spry2,Ptk7,Cnn3,Tes,Zic1,Adss,Cbx3,Etv5,Nfyb,Apex1,Prdx4,Elk3,Dlx1,Pank1,Pla2g4a,Plscr1,Nusap1,Ptpn12,Nav3,Il13ra1,Hn1,Pnn,Eef1g,Runx1,Tpm3,Rasal2,Rab15,Cenpf,Kpnb1,Nup107,Ebf2,Card10,Rrm1,Rnmt,Sdc1,Rab8b,Usp3,Mcm7,Sap30,Hmga1,Hdac2,Dhx9,Enpp1,Sox11,Nedd4l,Nxf1,Rnf138,Atrx,Ezh2,Tnks2,Pkn3,Pcf11,Tcf4,Has2,Mrpl1,Marcks,Wdr3,Rnf2,Dpf1,H2afy,Ankrd10,Pcna,Jub,Oip5,Nono,Bzw2,Mpp6,Lrp8,Bcl2l11,Dusp4,Dyrk3,Cdc25c,Ucp2,Gprc5b,Dnaja1,Klhdc2,Psip1,Nrp2,Hmga2,Ivns1abp,Fkbp5,Pak1,Ss18,Bnc2,Hmgb1,Six4,Fbxo32,Ktn1,Etv4,Ythdf2,Usp34,Smarcc1,Ahcyl1
**V$E2F1_Q6_01**	58(1239)	6,61E-41	Syncrip, Suv39h1, Stag1, Fancc, Nolc1, Klf5, Arhgap11a, Amd1, Mcm4, Msh2, Cbx5, Ctcf, Stmn1, Sin3a, Pole2, Rbl1, Nasp, Mcm2, Pold3, Usp37, Espl1, Ilf3, Cdc25a, Pou2f1, Casp2, Casp8ap2, Mcm3, Mcm6, Atad2, Topbp1, Rad51, Fbxo5, Pkmyt1, Clspn, Gmnn, Mcm7, Enpp1, Ung, Pprc1, Ezh2, Myc, Ipo7, Tmpo, Pcna, Pola2, Gprc5b, Gspt1, Cdca7, Slc9a5, Dnajc9, Ranbp1, Hmga2, Wee1, Prps1, Dnmt1, Fkbp5, Ncl, Cdc5l

Transcription Factor	*Number of genes*	*Corrected p-value*	*Genes repressed in Rasless cells (from Table S1)*
**V$E2F_Q6_01**	55(1239)	5,00E-39	Suv39h1, H2afz, Prim1, Pold1, Stag1, Klf5, Melk, Arhgap11a, Rrm2, Lig1, Mcm4, Cbx5, Ctcf, Nutf2, Tbx3, Mat2a, Stmn1, Pole2, Rbl1, Nasp, Mcm2, Cdc25a, Casp2, Casp8ap2, Plk4, Mcm3, Mcm6, Atad2, Topbp1, Dck, Rad51, Fbxo5, Kpnb1, Pkmyt1, Zcchc8, Gmnn, E2f7, Mcm7, Rbbp4, Dusp6, Ung, Ezh2, Myc, Ipo7, Pcna, Cdc6, Gprc5b, Cdca7, Ranbp1, Nrp2, Anp32e, Wee1, Dnmt1, Ncl, Etv4
**V$E2F_03**	55(1239)	2,20E-38	Suv39h1, H2afz, Prim1, Pold1, Stag1, Klf5, Melk, Lig1, Mcm4, Cbx5, Ctcf, Nutf2, Tbx3, Mat2a, Mrpl18, Stmn1, Eif2s1, Rbl1, Fus, Nasp, Rpa2, Mcm2, Cdc25a, Top1, Casp8ap2, Plk4, Mcm3, Mcm6, Atad2, Topbp1, Dck, Rad51, Fbxo5, Kntc1, Pkmyt1, Zcchc8, Gmnn, E2f7, Mcm7, Rbbp4, Ung, Pprc1, Ezh2, Myc, Ipo7, Pola2, Cdc6, Cdca7, Dnajc9, Ranbp1, Anp32e, Prps1, Dnmt1, Fkbp5, Slbp
**V$E2F1_Q4_01**	54(1239)	1,49E-37	Suv39h1, H2afz, Slc38a1, Usp1, Stag1, Fancc, Nup155, Klf5, Melk, Arhgap11a, Mcm4, Cbx5, Ctcf, Mat2a, Stmn1, Sin3a, Pole2, Nasp, Mcm2, Impdh2, Cdc25a, Top1, Egr3, Casp2, Casp8ap2, Mcm3, Mcm6, Atad2, Topbp1, Dck, Rad51, Fbxo5, Kpnb1, Pkmyt1, Zcchc8, Gmnn, Mcm7, Hmga1, Ung, Pprc1, Atrx, Ezh2, Myc, Ipo7, Pcna, Pola2, Cdc6, Cdca7, Ranbp1, Nrp2, Wee1, Prps1, Dnmt1, Ncl
**V$NFY_Q6_01**	115(1239)	1,91E-37	Gphn, Cct3, Ank3, Sf1, Ltbp1, Uhrf1, Rbm14, Mmd, Ncam1, Bub1, Trib1, Kif20a, Brd8, Suv39h1, Top2a, Arpc5, Cks1b, Mapk1, Usp1, Slc11a2, G3bp2, Nek2, Stag1, Sox4, Junb, Ell2, Nup93, Mtmr4, Sertad1, Arhgap11a, Dlg3, Mcm4, Msh2, Pim3, Ssr4, Cbx5, Sgol2, Mbd4, Terf1, Tcerg1, H2afx, Tbx3, Stmn1, Cit, Nasp, Nde1, Rpa2, Nup88, Ing5, Cdh10, Espl1, Khsrp, Cdc25a, Nup37, Asf1b, Ccng2, Casp2, Spry2, Stip1, Slc9a3r1, Cnn3, Tes, Etv5, Racgap1, Dgcr8, Slc4a7, Tdrkh, Dlx1, Troap, Xpo1, Pnn, Runx1, Etf1, Cenpf, Kpnb1, Rps9, Brca1, Clspn, Tacc3, Cyp26b1, Rab8b, Mcm7, Rbbp4, Plk1, Hmga1, Tpx2, Ly75, Cct7, Ddx10, Pcf11, Ube2c, Marcks, Spc25, Hmgb2, Mre11a, Lrp8, Epc2, Cdc25c, Hmga2, Wee1, Gart, Prpf31, Pabpn1, Tpp2, Kif23, Ivns1abp, Fkbp5, Rnaseh2a, Cks2, Erh, Ppil1, Spred2, Etv4, Mrpl50, Akap8
**V$E2F_Q3**	52(1239)	2,20E-36	H2afz, Slc38a1, Tfrc, Usp1, Klf5, Arhgap11a, Lig1, Mcm4, Cbx5, Ctcf, Tbx3, Mat2a, Mrpl18, Stmn1, Pole2, Nasp, Mcm2, Impdh2, Shmt1, Rbbp7, Cdc25a, Casp8ap2, Plk4, Mcm3, Mcm6, Atad2, Topbp1, Dck, Rad51, Fbxo5, Kpnb1, Pkmyt1, Zcchc8, Gmnn, E2f7, Mcm7, Rbbp4, Ung, Atrx, Ezh2, Ipo7, Pcna, Pola2, Cdc6, Ppp1cc, Cdca7, Ranbp1, Nrp2, Wee1, Prps1, Dnmt1, Ncl
**V$E2F1_Q3**	53(1239)	5,36E-36	H2afz, Tfrc, Usp1, Pold1, Snrpd1, Nolc1, Klf5, Arhgap11a, Rrm2, Msh2, Nutf2, Tbx3, Mrpl18, Stmn1, Pole2, Rbl1, Nasp, Mcm2, Pold3, Usp37, Shmt1, Ilf3, Cdc25a, Casp8ap2, Plk4, Soat1, Mcm3, Mcm6, Atad2, Topbp1, Dck, Fbxo5, Rasal2, Pkmyt1, Clspn, Zcchc8, Gmnn, E2f7, Mcm7, Rbbp4, Ezh2, Ipo7, Tmpo, Pcna, Cdc6, Gprc5b, Gspt1, Cdca7, Slc9a5, Dnajc9, Prps1, Dnmt1, Ncl
**V$E2F_Q3_01**	53(1239)	5,36E-36	Suv39h1, H2afz, Slc38a1, Usp1, Stag1, Fancc, Nup155, Klf5, Melk, Arhgap11a, Mcm4, Cbx5, Ctcf, Tbx3, Stmn1, Sin3a, Pole2, Nasp, Mcm2, Cdc25a, Top1, Egr3, Casp2, Casp8ap2, Mcm3, Mcm6, Atad2, Topbp1, Dck, Rad51, Fbxo5, Kpnb1, Pkmyt1, Zcchc8, Gmnn, E2f7, Mcm7, Hmga1, Ung, Pprc1, Ezh2, Myc, Ipo7, Pcna, Pola2, Cdc6, Cdca7, Ranbp1, Nrp2, Wee1, Prps1, Dnmt1, Ncl
**V$E2F1_Q4**	48(1239)	1,07E-29	Trib1, Lyar, Prim1, Slc38a1, Stag1, Melk, Hnrnpa3, Mcm4, Cbx5, Ctcf, Trim28, Stmn1, Sin3a, Pole2, Cit, Nasp, Rpa2, Lif, Zfp36l2, Dbr1, Chtf18, Cbx3, Dgcr8, Rad51, Fbxo5, Skp2, Kntc1, Kpnb1, Pkmyt1, Clspn, Zcchc8, E2f7, Sh3kbp1, Ung, Ezh2, Myc, Pcna, Pola2, Cdc6, Ppp1cc, Ranbp1, Nrp2, Wee1, Hmgb1, Erh, Spred2, Etv4, Slbp
**V$MYC_Q2**	93(1239)	1,14E-28	Cdkn2c, Hyal2, Notch1, Syncrip, Trib1, Nfx1, Lyar, H2afz, Arpc5, Fbl, Tfrc, Set, Acly, Mthfd1, Mtap, Nek6, Pop1, Hnrnpa3, Amd1, Hnrpdl, Fmr1, Slc43a1, Cbx5, U2af2, Rhobtb3, Sox12, Eno3, Tcerg1, Ppat, Mat2a, Stmn1, Lmnb1, Tgif2, Ddx18, Mcm2, Shmt1, Rbbp7, Sgol1, Socs5, Ilf3, Cirh1a, Rcl1, Snx5, Hspe1, Ctbp2, Tial1, Top1, Supt16h, Gprc5c, Runx2, Gemin4, Topors, Rpl30, Zic1, Adss, Suclg2, Tll1, Apex1, Dlx1, Xpo1, Pla2g4a, Rrs1, Ebna1bp2, Sdc1, Ewsr1, Rbbp4, Hmga1, Net1, Arf6, Sirt1, Fosl1, Pprc1, Rpl13a, Ipo7, Tcf4, Ube2c, Npm1, Eme1, Nudc, Lrp8, Dusp4, Eef1e1, Slc9a5, Ranbp1, Wee1, Prps1, Ivns1abp, Fen1, Fkbp5, Satb2, Ncl, Hspd1, Usp34
**V$NRF1_Q6**	82(1239)	7,69E-27	Abi1, Mad2l1, Cct3, Sf1, Rbm14, Hmmr, Arpc5, Cks1b, Set, Stag1, Pms2, Socs4, Ptpn2, Mcm4, Hnrpdl, Fmr1, Dffb, Pim3, Ssr4, Cbx5, Ccna2, Ctcf, U2af2, Cbfb, Trim28, Rad21, Rasa1, Eif2s1, Ccnf, Fus, Usp37, Rbm12, Cpsf4, Rbbp7, Ilf3, Rbbp8, Pou2f1, Hspe1, Tial1, Top1, Casp2, Gemin4, Topors, Sip1, Plk4, Ttll4, Prdx4, Bclaf1, Smarca5, Safb, Fbxo5, Nup107, Ewsr1, Gmnn, Kin, Dhx9, Pspc1, Arf6, Dusp6, Wdhd1, Tnks2, Polr2b, Npm1, Pcna, Pola2, Gtf3c2, Exosc2, Dnaja1, Slc9a5, Srpk1, Gart, Exosc8, Prpf3, Dnmt1, Cks2, Pigf, Bcl7a, Lsm2, Hspd1, Cct5, Cdc5l, Akap8
**V$FOXO4_01**	131(1239)	3,54E-24	Taf5, Gpr126, Cdkn2c, Ckap4, Ankrd28, Ank3, Hyal2, Ncam1, Trib1, Crlf1, Kif20a, Brd8, Pdgfb, Chd2, Llgl1, G3bp2, Ppil5, Tnpo1, Asxl1, Stag1, Rcor1, Ets1, Sema4b, Ell2, Klf5, Map4k5, Prkg2, Egr2, Dffb, Ghr, Prx, Elovl6, Xpo7, Cbfb, Phf6, Glrx, Csnk1g3, Crim1, Rest, Ppat, Rad21, Tbx3, Nup54, Nrp1, Mat2a, Stmn1, Eif2s1, Fus, Tgif2, Nasp, Whsc1, Foxp1, Cdh10, Impdh2, Tead2, Gata2, Lif, Stoml2, Ilf3, Cdc25a, Chd1, Pik3c2a, Pou2f1, Nr2f2, Rcl1, Tial1, Top1, Fgf7, Ccng2, Gprc5c, Runx2, Pde1a, Fmnl3, Mpzl1, Slc9a3r1, Zic1, Adss, Egr1, Etv5, Prdx4, Slc4a7, Dlx1, Ptpn12, Nav3, Il13ra1, Nap1l1, Runx1, Ebf2, Usp3, Mcm7, Rbbp4, Sap30, Mapk8, H2afv, Dusp6, Ung, Rnf138, Tnks2, Pkn3, Pcf11, Tcf4, Has2, Mrpl1, Marcks, H2afy, Pcna, Ets2, Hmgb2, Mpp6, Bcl2l11, Dusp4, Dyrk3, Arid4b, Cdk2, Cdc25c, Blm, Colec12, Gnb4, Klhdc2, Bub3, Nrp2, Hmga2, Pafah1b3, Fkbp5, Bnc2, Pigf, Satb2, Bcl2, Bcl7a, Fbxo32, Etv4
**V$MAZ_Q6**	137(1239)	4,42E-22	Gphn,Sf1,Notch1,Mycn,Mmd,Syncrip,Ncam1,Trib1,Pdgfb,Suv39h1,H2afz,Chd2,Arpc5,Ccne2,Cks1b,Slc38a1,Trim25,G3bp2,Set,Tnpo1,Stag1,Alad,Sox4,Junb,Sertad1,Hnrnpa3,Dlg3,Prkar2b,Hnrpdl,Dusp9,Fmr1,Egr2,Elovl6,Dapp1,Ctcf,Phc2,Sox12,Phf6,Nutf2,Baz1a,Rest,Rad21,Tbx3,Nrp1,Cx3cl1,Syne2,Sin3a,Lmnb1,Fus,Tgif2,Smndc1,Tnnt2,Rpl12,Sec63,Nab2,Tead2,Aebp2,Khsrp,Lif,Rbbp7,Uchl5,Ilf3,Pogz,Cdca3,Denr,Actr3,Chd1,Pou2f1,Nr2f2,Hspe1,Tial1,Luc7l,Hat1,Fgf7,Ccng2,Supt16h,Gprc5c,Matr3,Egr3,Mpzl1,Stip1,Slc9a3r1,Casp8ap2,Ptk7,Cnn3,Egr1,Cbx3,Etv5,Elk3,Hspa8,Dlx1,Troap,Xpo1,Pank1,Dhx15,Galnt4,Topbp1,Pnn,Eef1g,Tpm3,Etf1,Kpnb1,Rnmt,Sdc1,Ewsr1,Hmga1,Arf6,Sox11,Dusp6,Fosl1,Pprc1,Rnf138,Cdc40,Myc,Pkn3,Pcf11,Tcf4,Snrpa1,Marcks,Rnf2,Dusp4,Gspt1,Dnaja1,Nrf1,Psip1,Hmga2,Gart,Dnmt1,Ivns1abp,Bnc2,Ccne1,Six4,Hspd1,Spred2,Etv4,Smarcc1,Tpbg
**V$YY1_Q6**	51(1239)	5,70E-20	Sf3a1, Tardbp, Chd2, Ssr4, Sfxn1, Cbx5, Ctcf, U2af2, Rad21, Mat2a, Syne2, Sfpq, Lmnb1, Fus, Hmgb3, Nasp, Whsc2, Foxp1, Rbm12, Rif1, Aebp2, Eif5, Usp14, Ilf3, Cirh1a, Pou2f1, Snx5, Tial1, Racgap1, Wsb1, Elk3, Hspa8, Ier2, Sumo2, Hn1, Safb, Kntc1, Rrm1, Cpsf2, Upf3b, Pspc1, Atrx, Tnks2, Pcf11, Tcf4, Mrpl1, Epc2, Ppp1cc, Smyd5, Csnk1a1, Akap8
**V$E2F1_Q3_01**	39(1239)	7,43E-20	Syncrip, Ncam1, Prim1, Stag1, Zbtb12, Junb, Ssr4, Stmn1, Rbl1, Impdh2, Eif5, Zfp36l2, Cdc25a, Nr2f2, Egr3, Plk4, Elk3, Atad2, Topbp1, Dck, Kpnb1, Gmnn, Mcm7, Dusp6, Fosl1, Ezh2, Myc, Marcks, Nono, Lrp8, Ppp1cc, Gspt1, Dnajc9, Ranbp1, Wee1, Dnmt1, Satb2, Ythdf2, Tpbg
**V$MYCMAX_01**	38(1239)	2,06E-19	Syncrip, Trib1, Lyar, Tfrc, Asxl1, Mthfd1, Hnrpdl, Slc43a1, Cbx5, U2af2, Eno3, Tcerg1, Ppat, Mat2a, Stmn1, Ilf3, Rcl1, Snx5, Top1, Gprc5c, Runx2, Suclg2, Prdx4, Sdc1, Ewsr1, Hmga1, Pprc1, Ipo7, Npm1, Nudc, Lrp8, Ranbp1, Prps1, Ivns1abp, Satb2, Ncl, Etv4, Usp34

Transcription Factor	*Number of genes*	*Corrected p-value*	*Genes repressed in Rasless cells (from Table S1)*
**V$ELK1_02**	79(1239)	7,59E-18	Sf3a1, Elavl1, Cks1b, Stag1, Fancd2, Alad, Mtap, Nup155, Ptpn2, Mtmr4, Sertad1, Dhx36, Taf5l, Rnps1, Fmr1, Egr2, U2af2, Snrpb, Nutf2, Eif2s1, Ube2e3, Tomm70a, Nasp, Chuk, Sms, Dnaja2, Mastl, Rfc2, Pdap1, Stoml2, Pogz, Vrk3, Nup37, Cdca3, Hat1, Supt16h, Rfc4, Dbr1, Plk4, Egr1, Elk3, Ier2, Dhx15, Skp2, Uba2, Tpm3, Rrs1, Yme1l1, Smu1, Ebna1bp2, Nup107, Rnmt, Usp3, Mcm7, Rbbp4, Hmga1, Slc25a5, Tpx2, Snrpe, Cct7, Tnks2, Pkn3, Rnf2, Sf3a3, Eme1, Nudc, Bzw2, Hirip3, Gtf3c2, Blm, Gspt1, Dnaja1, Gart, Metap2, Dnmt1, Erh, Ppil1, Spred2, Ythdf2
**V$E2F_01**	21(1239)	1,02E-16	Fancc, Ptbp2, Nolc1, Amd1, Mcm4, H2afx, Nasp, Mcm2, Khsrp, Casp2, Mcm3, Rfc5, Topbp1, E2f7, Myc, Pcna, Ranbp1, Wee1, Prps1, Dnmt1, Bnc2
**V$ETS2_B**	80(1239)	3,50E-16	Taf5, Lifr, Sf1, Hyal2, Trib1, Pdgfb, Chd2, Cks1b, Trim25, Strbp, Ets1, Rasa3, Cldn12, Casp8, Junb, Etv6, Amd1, Slc43a1, Dapp1, Ctcf, Tcerg1, Rest, Nrp1, Ube2e3, Tgif2, Nasp, Egfl7, Foxp1, Dnaja2, Cnnm4, Pdap1, Lif, Rbbp7, Ruvbl1, Actr3, Tial1, Ppif, Sdpr, Egr3, Slc9a3r1, Ptk7, Egr1, Etv5, Tll1, Elk3, Dhx15, Dck, Safb, Tpm3, Etf1, Ctsw, Cd2ap, Kpnb1, Clspn, Usp3, Pgm1, Sh3kbp1, Rbbp4, Hmga1, Slc25a5, Cxadr, Arf6, Enpp1, Adamts7, Fosl1, Pprc1, Rnf138, Timp1, Tnks2, Pkn3, Ipo7, Bub1b, Bzw2, Dusp4, Epc2, Psip1, Tpp2, Myct1, Dnmt1, Ss18
**V$MYCMAX_B**	35(1239)	5,29E-16	Trib1, Pdgfb, Sox4, Eps8, Hnrpdl, Elovl6, Sox12, Nutf2, Stmn1, Tgif2, Rbbp7, Ilf3, Rcl1, Snx5, Egr3, Ptk7, Hn1, Etf1, Kpnb1, Ewsr1, Hmga1, Slc25a5, Arf6, Ipo7, Npm1, Jub, Bzw2, Lrp8, Ranbp1, Wee1, Ivns1abp, Ncl, Socs3, Cct5, Ythdf2
**V$GABP_B**	60(1239)	1,55E-14	Taf5, Hyal2, Sf3a1, Syncrip, Ncam1, Suv39h1, Cks1b, Tfrc, Cldn12, Socs4, Junb, Nup155, Ptpn2, Mtmr4, Amd1, Taf5l, Rnps1, Fmr1, Egr2, Nufip1, Ssr4, Elovl6, Dapp1, U2af2, Cbfb, Phf6, Sgol2, Eif2s1, Dusp5, Tgif2, Nasp, Chuk, Rpa2, Foxp1, Pogz, Trip13, Hat1, Nme1, Supt16h, Rfc4, Spry2, Slc9a3r1, Etv5, Elk3, Hspa8, Bclaf1, Ptpn12, Etf1, Smu1, Ebna1bp2, Rnmt, Hmga1, Cct7, Wdhd1, Hirip3, Ranbp1, Dnmt1, Pigf, Erh, Cdc5l
**V$SOX9_B1**	46(1239)	7,23E-14	Ank3, Hyal2, Ncam1, Top2a, Elavl1, G3bp2, Tnpo1, Ets1, Sox4, Rrm2, Elovl6, Phf6, Stmn1, Snapc3, Nasp, Dnaja2, Top1, Cnn3, Zic1, Dlx1, Pank1, Smarca5, Ptpn12, Hn1, Tpm3, Zcchc8, Cdc27, Rbbp4, Dusp6, Ilf2, Tcf4, Marcks, Bcl2l11, Slit2, Cdk2, Cdca7, Hmgn1, Psip1, Ranbp1, Wee1, Myct1, Fen1, Pak1, Bnc2, Hmgb1, Erh
**V$USF_C**	33(1239)	4,11E-13	Hyal2, Lyar, Fbl, Set, Mthfd1, Eno3, Ppat, Mat2a, Stmn1, Socs5, Snx5, Hspe1, Tial1, Gprc5c, Runx2, Suclg2, Tll1, Pla2g4a, Sdc1, Sh3kbp1, Arf6, Sirt1, Rpl13a, Npm1, Nudc, Lrp8, Exosc2, Eef1e1, Slc9a5, Wee1, Ivns1abp, Satb2, Etv4
**V$MYCMAX_02**	32(1239)	4,56E-13	Cdkn2c, Notch1, Syncrip, H2afz, Fbl, Mthfd1, Nolc1, Hnrpdl, Cbx5, Sox12, Bach1, Mat2a, Tgif2, Cirh1a, Rcl1, Top1, Runx2, Umps, Ptk7, Adss, Dlx1, Xpo1, Galnt4, Arf6, Pprc1, Tcf4, Npm1, Cdk2, Ivns1abp, Fkbp5, Satb2, Usp34
**V$E12_Q6**	119(1239)	1,32E-12	Gpr126, Cdkn2c, Ank3, Ltbp1, Hyal2, Syncrip, Ncam1, Trib1, Crlf1, Neto2, Pdgfb, H2afz, Chd2, Tfrc, Usp1, G3bp2, Acly, Asxl1, Stag1, Sema4b, Sox4, F2rl1, Eps8, Mtmr4, Amd1, Dusp9, Slc43a1, Gli2, Phc2, Sox12, Axl, Phf6, Lrig1, Eno3, Crim1, Syne2, Sin3a, Rbl1, Dusp5, Tgif2, Pcm1, Sms, Usp37, Nup88, Rangap1, Tead2, Gata2, Lif, Rbbp7, Uchl5, Socs5, Nck2, Asf1b, Pou2f1, Spred1, Slc5a3, Fgf7, Sdpr, Spry2, Slc9a3r1, Plk4, Elk3, Igsf3, Tdrkh, Acaa2, Il13ra1, Hn1, Skp2, Runx1, Tpm3, Rasal2, Cd2ap, Sdc1, Mcm7, Hmga1, Slc25a5, Hdac2, Cxadr, Arf6, Sirt1, Dusp6, Nedd4l, Fosl1, Ezh2, Tnks2, Myc, H2afy, Abl2, Jub, Ets2, Bzw2, Swap70, Dusp4, Slit2, Cdk2, Colec12, Gprc5b, Nrf1, Ell, Lbr, Psip1, Nrp2, Srpk1, Pafah1b3, Pabpn1, Ivns1abp, Dock5, Fkbp5, Pak1, Bnc2, Bcl2, Fbxo32, Ktn1, Spred2, Etv4, Socs3, Mrpl50, Ythdf2, Ahcyl1
**V$EGR1_01**	31(1239)	1,67E-12	Cdkn2c, Gphn, Sf1, Hyal2, Trib1, Pdgfb, Usp1, Sertad1, Hnrpdl, Ctcf, Sox12, Tbx3, Fus, Cnnm4, Nab2, Socs5, Spred1, Matr3, Egr3, Adss, Egr1, Smarca5, Runx1, Sh3kbp1, Hmga1, Ell, Hmgn1, Prpf3, Smyd5, Etv4, Tpbg
**V$NFMUE1_Q6**	29(1239)	2,76E-12	Sf1, Chd2, Stag1, Ptbp2, Cbx5, Rest, Sfpq, Hmgb3, Nasp, Rpa2, Rbm12, Rif1, Eif5, Pou2f1, Suv39h2, Rpl30, Bclaf1, Dhx15, Cpsf2, Pprc1, Tnks2, Pcf11, Tcf4, Ppp1cc, Prps1, Pabpn1, Smyd5, Erh, Ccne1
**V$NMYC_01**	31(1239)	2,88E-12	Hyal2, Fbl, Mthfd1, Slc43a1, Elovl6, U2af2, Sox12, Ppat, Lmnb1, Socs5, Rcl1, Hspe1, Ctbp2, Tial1, Top1, Runx2, Adss, Prdx4, Dlx1, Pla2g4a, Sh3kbp1, Arf6, Rpl13a, Npm1, Dusp4, Slc9a5, Wee1, Fkbp5, Bcl2, Hspd1, Usp34
**V$HSF_Q6**	32(1239)	4,18E-12	Gphn, Cct3, Ank3, Ltbp1, Cks1b, Ahsa1, Phf6, Nutf2, Mat2a, Ube2e3, Sin3a, Lmnb1, H2afy2, Cct8, Foxp1, Rangap1, Hspe1, Luc7l, Egr3, Stip1, Gemin4, Cbx3, Ppid, Tll1, Hspa8, Xpo1, Cct7, Lrp8, Lrrk1, Dnaja1, Six4, Hspd1
**V$NFAT_Q4_01**	98(1239)	5,28E-12	Ankrd28, Ltbp1, Hyal2, Syncrip, Pdgfb, Tinf2, Chd2, Impa2, Fbl, Cks1b, Trim25, Vegfc, Unc5c, Tnpo1, Acly, Stag1, Rcor1, Ets1, Nup155, Klf5, Amd1, Fmr1, Egr2, Slc43a1, Mrps6, Exo1, Smarce1, Phf6, Vrk1, Rest, Tbx3, Stmn1, Syne2, Sfpq, Kif21a, Tgif2, Pdk3, Whsc1, Foxp1, Nab2, Aebp2, Lif, Stoml2, Rbbp7, Pogz, Spred1, Slc5a3, Fgf7, Supt16h, Iqgap3, Asph, Spry2, Stip1, Slc9a3r1, Ptk7, Zic1, Etv5, Tll1, Racgap1, Elk3, Igsf3, Dlx1, Xpo1, Nav3, Skp2, Runx1, Tpm3, Rasal2, Yme1l1, Ebna1bp2, Ebf2, Cyp26b1, Rab8b, Sh3kbp1, Enpp1, Sox11, Nedd4l, Pcf11, Tcf4, Has2, Jub, Plxnb1, Ets2, Nono, Bzw2, Spp1, Colec12, Dnaja1, Psip1, Gpsm2, Hmga2, Anp32e, Bnc2, Pigf, Satb2, Ncl, Trit1, Cdc5l
**V$E4F1_Q6**	51(1239)	1,35E-11	Sf1, Ltbp1, Prpf4, Trib1, Tfrc, G3bp2, Stag1, Ddx51, Junb, Ell2, Nolc1, Dhx36, Amd1, Egr2, Nufip1, Ssr4, Ctcf, Smarce1, Dusp5, Chuk, Sms, Sec63, Nab2, Mastl, Rbbp7, Rbbp8, Rfc4, Egr3, Umps, Plk4, Egr1, Cbx3, Soat1, Wsb1, Smarca5, Etf1, Yme1l1, Cd2ap, Cxadr, Rpl13a, Tnks2, Lrp8, Bcl2l11, Dusp4, Rad51c, Ubqln2, Dnaja1, Slc9a5, Dnajc9, Hmgb1, Satb2
**V$EGR2_01**	25(1239)	2,20E-11	Cdkn2c, Syncrip, Pdgfb, Sox4, Klf5, Sertad1, Hnrpdl, Cx3cl1, Sin3a, H2afy2, Fus, Usp37, Nab2, Mastl, Egr3, Egr1, Smarca5, Sumo2, Runx1, Yme1l1, Nup107, Sirt1, Colec12, Smyd5, Etv4
**V$AP4_Q5**	82(1239)	2,93E-11	Cdkn2c, Ltbp1, Hyal2, Notch1, Mycn, Pdgfb, H2afz, Ccne2, Usp1, Set, Rrad, Ets1, Sox4, Klf5, Mtmr4, Hnrpdl, Egr2, Eif4enif1, Ghr, Mrps6, Slc43a3, Idh2, Sox12, Slc25a24, Eno3, Nutf2, Bach1, Ppat, Tbx3, Eif2s1, Tgif2, Foxp1, Tnnt2, Tead2, Pdap1, Stoml2, Rbbp7, Mybl2, Cdca3, Denr, Spred1, Luc7l, Fgf7, Supt16h, Pde1a, Sdpr, Ptk7, Suv39h2, Egr1, Etv5, Pvrl3, Dlx1, Smarca5, Nav3, Sumo2, Hn1, Fbxo5, Runx1, Tpm3, Etf1, Ebna1bp2, Usp3, Nedd4l, Col7a1, Myc, Pcf11, Tcf4, Dusp4, Lrrk1, Cdk2, Colec12, Dnaja1, Ell, Klhdc2, Psip1, Nrp2, Hmga2, Bnc2, Erh, Bcl7a, Ythdf2, Trit1
**V$FREAC2_01**	61(1239)	3,33E-11	Lifr, Ankrd28, Bub1, Trib1, Kif20a, Brd8, Pdgfb, Chd2, Ets1, Junb, Map4k5, Taf5l, Fmr1, Elovl6, Dapp1, Xpo7, Ctcf, Sox12, Axl, Csnk1g3, Rad21, Sin3a, Kif21a, Impdh2, Stoml2, Zfp36l2, Chd1, Pik3c2a, Pou2f1, Rcl1, Gprc5c, Pde1a, Etv5, Elk3, Dlx1, Pank1, Runx1, Nfrkb, Cd2ap, Usp3, Mcm7, Slc25a5, Myc, Pcf11, Tcf4, Marcks, Mpp6, Dusp4, Colec12, Klhdc2, Bub3, Wee1, Tpp2, Metap2, Fen1, Dock5, Pak1, Bnc2, Satb2, Fhl2, Usp34
**V$MAX_01**	28(1239)	1,28E-10	Notch1, Syncrip, Nfx1, Tfrc, Mthfd1, Cbx5, Tcerg1, Mat2a, Stmn1, Ilf3, Rcl1, Snx5, Top1, Supt16h, Runx2, Topors, Adss, Suclg2, Xpo1, Hmga1, Sirt1, Pprc1, Tcf4, Npm1, Eme1, Nudc, Prps1, Fen1
**V$PAX3_B**	25(1239)	2,05E-10	Ltbp1, Trib1, Junb, Dhx36, Egr2, Ctcf, Smarce1, Baz1a, Tbx3, Nab2, Mastl, Rbbp8, Supt16h, Egr3, Egr1, Soat1, Wsb1, Xpo1, Smarca5, Yme1l1, Ipo7, Bzw2, Lrp8, Rad51c, Hmgn1
**V$EGR_Q6**	29(1239)	2,79E-10	Gphn, Hyal2, Pdgfb, Arpc5, Unc5c, Sertad1, Egr2, Prx, Ctcf, Tgif2, Sec63, Cnnm4, Nr2f2, Matr3, Adss, Egr1, Smarca5, Il13ra1, Rasal2, Kpnb1, Sh3kbp1, Hmga1, Pprc1, Bcl2l11, Slc9a5, Anp32e, Prpf3, Six4, Ythdf2

Transcription Factor	*Number of genes*	*Corrected p-value*	*Genes repressed in Rasless cells (from Table S1)*
**V$MYCMAX_03**	27(1239)	3,36E-10	Cdkn2c, Syncrip, Trib1, Nfx1, Arpc5, Hnrnpa3, Fmr1, Cbx5, Mat2a, Stmn1, Tgif2, Mcm2, Shmt1, Socs5, Ilf3, Snx5, Topors, Zic1, Adss, Xpo1, Rbbp4, Sirt1, Pprc1, Rpl13a, Npm1, Eme1, Prps1
**V$CEBPB_02**	30(1239)	5,36E-10	Ckap4, Syncrip, Trib1, Pdgfb, H2afz, Chd2, Acly, Nek6, Etv6, Klf5, Baz1a, Stmn1, Foxp1, Pdap1, Spred1, Top1, Sdpr, Dlx1, Pla2g4a, Atad2, Ptpn12, Rasal2, Arf6, Pcf11, Mpp6, Dyrk3, Nrp2, Srpk1, Bnc2, Ruvbl2
**V$NF1_Q6**	56(1239)	9,88E-10	Cdkn2c, Lifr, Ckap4, Ankrd28, Syncrip, Ncam1, G3bp2, Rrad, Stag1, Sox4, Mtap, Map4k5, Dlg3, Egr2, Eif4enif1, Ghr, Idh2, Eno3, Mat2a, Tslp, Kif21a, Tgif2, Cdh10, Pou2f1, Slc5a3, Supt16h, Pde1a, Sdpr, Slc9a3r1, Ptk7, Etv5, Nfyb, Dlx1, Nav3, Runx1, Tpm3, Itih2, Ebf2, Rab8b, Rbbp4, Pum2, Nxf1, Tcf4, Plxnb1, Bzw2, Dusp4, Exosc2, Lrrk1, Colec12, Ranbp1, Nrp2, Hmga2, Pabpn1, Ivns1abp, Bnc2, Etv4
**V$WHN_B**	26(1239)	2,20E-09	Taf5, Prpf4, Trib1, H2afz, Arpc5, Zbtb12, Junb, Nolc1, Mtmr4, Amd1, Elovl6, Ctcf, Tbx3, Uchl5, Ssb, Hspe1, Top1, Supt16h, Egr3, Spry2, Gjc1, Cxadr, Arid4b, Hspa14, Hspd1, Ythdf2
**V$HSF1_01**	37(1239)	2,23E-09	Cct3, Ankrd28, Trib1, Neto2, H2afz, Chd2, Fancc, Nek6, Cct2, Eif4enif1, Mrps6, Dapp1, Phf6, Baz1a, Mat2a, Cct8, Cdh10, Eif5, Socs5, Chd1, Hspe1, Elf2, Egr3, Stip1, Cbx3, Ppid, Elk3, Hspa8, Xpo1, Kntc1, Pkn3, Dnaja1, Nrf1, Bnc2, Six4, Hspd1, Usp34
**V$MYOD_Q6**	58(1239)	7,34E-09	Gphn, Hyal2, Notch1, Ncam1, Trib1, Crlf1, Pdgfb, H2afz, Ccne2, Rrad, Mum1, Hnrpdl, Fmr1, Egr2, Eif4enif1, Mrps6, Slc43a3, Idh2, Xpo7, Eno3, Tgif2, Pcm1, Nup88, Cdh10, Tead2, Gata2, Pdap1, Rbbp7, Pou2f1, Nr2f2, Supt16h, Pde1a, Tdrkh, Xpo1, Smarca5, Hn1, Runx1, Etf1, Cd2ap, Hmga1, Slc25a5, Nedd4l, Col7a1, Ezh2, Tnks2, Myc, Pcf11, Abl2, Dusp4, Slit2, Nrf1, Psip1, Myct1, Bnc2, Bcl7a, Fbxo32, Ythdf2, Trit1
**V$ERR1_Q2**	61(1239)	1,38E-08	Lifr, Ankrd28, Ank3, Metap1, Elavl1, Chd2, Tfrc, Nek2, Asxl1, Fancd2, Sox4, Apex2, Socs4, Etv6, Map4k5, Prkg2, Pim3, Ssr4, Nipsnap1, Sox12, Phf6, Idh3a, Eno3, Tcerg1, Nrp1, Cx3cl1, Stmn1, Tomm70a, Hmgb3, Usp37, Dnaja2, Fbln1, Pgk1, Hspe1, Fgf7, Pde1a, Slc9a3r1, Dgcr8, Dlx1, Pank1, Atad2, Smarca5, Eef1g, Tpm3, Usp3, Kin, Slc25a5, Arf6, Rnf2, Bub1b, Bzw2, Mpp6, Epc2, Ppp1cc, Psip1, Ranbp1, Bnc2, Ccne1, Hspd1, Ythdf2, Ahcyl1
**V$TFIIA_Q6**	26(1239)	2,18E-08	Ckap4, Hyal2, Syncrip, Trib1, Elavl1, H2afz, Unc5c, Ppil5, Etv6, Trim28, Axl, Nup54, Eif5, Luc7l, Egr1, Ier2, Gjc1, Mcm7, Rbbp4, Pcf11, Prps1, Pabpn1, Myct1, Fhl2, Bcl7a, Ktn1
**V$HIF1_Q3**	23(1239)	2,21E-08	Ltbp1, Hyal2, Pdgfb, Tfrc, Lig1, Elovl6, Stmn1, Pdk3, Usp37, Pgk1, Nr2f2, Snx5, Tial1, Tll1, Prdx4, Hmga1, Pprc1, Lrp8, Bcl2l11, Nrf1, Bcl2, Ruvbl2, Ktn1
**V$ZF5_01**	24(1239)	3,45E-08	Elavl1, Junb, Ctcf, Phc2, Nutf2, Stmn1, Aebp2, Nr2f2, Spred1, Gprc5c, Mpzl1, Sumo2, Kpnb1, Rbbp4, Fosl1, Jub, Lrp8, Kif2c, Ppp1cc, Tpp2, Pak1, Ccne1, Bcl7a, Ythdf2
**V$USF_01**	24(1239)	3,81E-08	Nfx1, Arpc5, Tfrc, Set, Hnrnpa3, Fmr1, U2af2, Stmn1, Mcm2, Sgol1, Ilf3, Snx5, Topors, Apex1, Xpo1, Rbbp4, Sirt1, Rpl13a, Npm1, Eme1, Nudc, Ranbp1, Prps1, Fen1
**V$HOXA4_Q2**	25(1239)	5,37E-08	Ltbp1, Syncrip, Apex2, Etv6, Klf5, Amd1, Phc2, H2afx, Nup54, Nrp1, Syne2, Dnaja2, Stoml2, Zfp36l2, Pogz, Actr3, Pou2f1, Cnn3, Zic1, Elk3, Dlx1, Dusp6, Nxf1, Slc9a5, Hmga2
**V$NFY_01**	23(1239)	1,11E-07	Ncam1, Nek2, Junb, Dlg3, Sgol2, Stmn1, Cit, Ing5, Zfp36l2, Cdc25a, Cnn3, Etv5, Racgap1, Dlx1, Xpo1, Tacc3, Plk1, Dusp6, Pcf11, Lrp8, Hmga2, Kif23, Cks2
**V$EGR3_01**	13(1239)	1,11E-07	Cdkn2c, Pdgfb, Klf5, Hnrpdl, Fus, Mastl, Egr3, Egr1, Yme1l1, Sirt1, Ranbp1, Smyd5, Etv4
**V$NFY_Q6**	23(1239)	1,34E-07	Bub1, Suv39h1, Top2a, Nek2, Sertad1, Dlg3, Msh2, Cit, Nup37, Ctbp2, Spry2, Racgap1, Slc4a7, Dlx1, Cenpf, Atrx, Hmgb2, Lrp8, Hmga2, Gart, Cks2, Six4, Ttk
**V$PAX4_03**	68(1239)	1,47E-07	Abi1, Hyal2, Syncrip, Crlf1, Pdgfb, Cks1b, Rrad, Stag1, Klf5, Ddx31, Dlg3, Prkar2b, Eif4enif1, Elovl6, Ccna2, Xpo7, Ctcf, Cbfb, Axl, Slc25a24, Crim1, H2afx, Tbx3, Stmn1, Rasa1, Rbl1, Dusp5, Pdk3, Cdca2, Nab2, Impdh2, Tead2, Lif, Pogz, Pou2f1, Luc7l, Supt16h, Egr3, Fmnl3, Spry2, Ptk7, Cbx3, Etv5, Elk3, Igsf3, Dlx1, Dhx15, Ptpn12, Fbxo5, Eef1g, Kntc1, Tpm3, Rasal2, Kpnb1, Cyp26b1, Hmga1, Nedd4l, Pcf11, Tcf4, Dpf1, H2afy, Jub, Gnb4, Gspt1, Slc9a5, Pak1, Six4, Ythdf2
**V$ZIC3_01**	24(1239)	1,79E-07	Mycn, Syncrip, Fancd2, Etv6, Ghr, Prx, Phf6, Foxp1, Rbm12, Pou2f1, Gprc5c, Gemin4, Ptk7, Nfyb, Safb, Nfrkb, Rasal2, Cyp26b1, Pprc1, Col7a1, Has2, Lrp8, Six4, Litaf
**V$ALPHACP1_01**	23(1239)	1,99E-07	Sf1, Usp1, Nek2, Sertad1, Dlg3, Cdh10, Cdca3, Fgf7, Ccng2, Spry2, Xpo1, Pank1, Ebf2, Dusp6, Ilf2, Pcf11, Ube2c, Pcna, Mre11a, Lrp8, Gart, Aspm, Cks2
**V$SREBP1_01**	35(1239)	2,10E-07	Pdgfb, Chd2, Arpc5, Set, Stag1, Hnrnpa3, Dlg3, Fmr1, Ctcf, U2af2, Stmn1, Eif2s1, Smndc1, Mcm2, Pcm1, Foxp1, Ilf3, Supt16h, Topors, Apex1, Dlx1, Xpo1, Rbbp4, Sirt1, Atrx, Has2, H2afy, Eme1, Swap70, Cdk2, Blm, Fen1, Bcl7a, Fbxo32, Ahcyl1
**V$PAX_Q6**	24(1239)	2,32E-07	Taf5, Trib1, Crlf1, Metap1, Chd2, Etv6, Amd1, Dlg3, Mrps6, Rcl1, Luc7l, Egr3, Spry2, Stip1, Ptk7, Etv5, Ppid, Runx1, Tpm3, Ube2c, Eme1, Lrrk1, Gnb4, Nrp2
**V$NFY_C**	22(1239)	2,61E-07	Uhrf1, Rbm14, Ncam1, Kif20a, Brd8, Dlg3, Cbx5, Baz1a, Tcerg1, Rpa2, Espl1, Cdc25a, Etv5, Racgap1, Ube2c, Spc25, Lrp8, Tpp2, Kif23, Cks2, Satb2, Mrpl50
**V$TEL2_Q6**	21(1239)	2,78E-07	Taf5, Hyal2, Chd2, Cks1b, Casp8, Junb, Amd1, Rnps1, Dapp1, U2af2, Snrpb, Tgif2, Nasp, Slc9a3r1, Ctsw, Usp3, Hmga1, Slc25a5, Hirip3, Erh, Ppil1
**V$ARNT_01**	23(1239)	2,82E-07	Syncrip, Pdgfb, Cbx5, Tcerg1, Ppat, Stmn1, Shmt1, Ilf3, Rcl1, Snx5, Adss, Xpo1, Arf6, Sirt1, Pprc1, Rpl13a, Npm1, Eme1, Nudc, Prps1, Pabpn1, Hspd1, Etv4
**V$NGFIC_01**	22(1239)	3,87E-07	Cdkn2c, Hyal2, Syncrip, Trib1, Pdgfb, G3bp2, Sertad1, Hnrpdl, Cx3cl1, Sin3a, Fus, Cnnm4, Matr3, Egr3, Adss, Egr1, Sumo2, Etf1, Sh3kbp1, Slc25a5, Prpf3, Smyd5
**V$CEBP_Q2_01**	24(1239)	4,76E-07	Syncrip, Trib1, Chd2, Etv6, Klf5, Crim1, Stmn1, Egfr, Foxp1, Spred1, Sdpr, Etv5, Dlx1, Pla2g4a, Atad2, Ebf2, Arf6, Mpp6, Dyrk3, Nrp2, Wee1, Bnc2, Ruvbl2, Usp34
**V$USF_Q6**	22(1239)	5,18E-07	Trib1, Nfx1, Dusp9, Egr2, Tcerg1, Stmn1, Sgol1, Socs5, Hspe1, Ctbp2, Supt16h, Prdx4, Ewsr1, Arf6, Sirt1, Ipo7, Npm1, Eme1, Hmgn1, Ranbp1, Wee1, Prps1
**V$SRF_Q4**	24(1239)	5,60E-07	Chd2, G3bp2, Junb, Taf5l, Egr2, H2afx, Cx3cl1, Dusp5, Foxp1, Rbbp7, Nr2f2, Sdpr, Egr3, Asph, Egr1, Ier2, Tpm3, Dusp6, Atrx, Tcf4, Ube2c, Anp32e, Bnc2, Myo1b
**V$GFI1_01**	33(1239)	6,30E-07	Ncam1, Ets1, Sox4, Mtmr4, Phc2, Crim1, Stmn1, Rangap1, Lif, Pogz, Pou2f1, Nr2f2, Top1, Fgf7, Ccng2, Sdpr, Gemin4, Zic1, Pvrl3, Dgcr8, Slc4a7, Pank1, Ebf2, Hmga1, Mapk8, Atrx, Tcf4, Colec12, Cdca7, Nrp2, Bnc2, Fbxo32, Tpbg
**V$YY1_01**	22(1239)	8,31E-07	Syncrip, Chd2, Stag1, Map4k5, Dlg3, Egr2, Ctcf, Phf6, Fus, Cdh10, Matr3, Egr3, Dlx1, Tpm3, Rab8b, Cxadr, Pcf11, Tcf4, Plxnb1, Hmgb2, Gtf3c2, Bnc2
**V$STAT1_02**	21(1239)	8,47E-07	Cks1b, Set, Sema4b, Sox4, Tcerg1, Nasp, Dnaja2, Pogz, Hspe1, Cbx3, Dlx1, Bclaf1, Skp2, Rab8b, Pcf11, Jub, Gspt1, Pabpn1, Hspd1, Csnk1a1, Mrpl50

Transcription Factor	*Number of genes*	*Corrected p-value*	*Genes repressed in Rasless cells (from Table S1)*
**V$AHRARNT_01**	16(1239)	1,08E-06	Pdgfb, H2afz, Cks1b, Usp1, Sox4, Ghr, Rbbp7, Egr3, Soat1, Wsb1, Elk3, Runx1, Cyp26b1, Pprc1, Tnks2, Jub
**V$SPZ1_01**	22(1239)	1,09E-06	Hyal2, Syncrip, Snrpd1, Xpo7, Ctcf, Rbm12, Nab2, Ilf3, Pou2f1, Nr2f2, Xpo1, Sumo2, Tpm3, Kpnb1, Hmga1, Pcf11, Kcnk2, Ranbp1, Bcl7a, Six4, Ythdf2, Smarcc1
**V$SRF_C**	21(1239)	1,12E-06	Ltbp1, Syncrip, G3bp2, Junb, Egr2, Dusp5, Foxp1, Rbbp7, Actr3, Nr2f2, Top1, Matr3, Egr3, Egr1, Ier2, Tpm3, Rrm1, Dusp6, Fosl1, Fhl2, Myo1b
**V$HNF4_DR1_Q3**	22(1239)	1,28E-06	Ckap4, Asxl1, Zbtb12, Mtmr4, Eno3, Tomm70a, Usp37, Dnaja2, Fbln1, Shmt1, Hspe1, Egr3, Timeless, Net1, Gtf3c2, Gprc5b, Pabpn1, Fkbp5, Ss18, Smyd5, Hspd1, Ahcyl1
**V$AREB6_04**	22(1239)	1,39E-06	Cdkn2c, Ltbp1, Sox4, Junb, Klf5, Elovl6, Gemin4, Xpo1, Nav3, Itih2, Ebf2, Arf6, H2afv, Sox11, Dusp6, Pcf11, Tcf4, Swap70, Gnb4, Gart, Chst1, Hmgb1
**V$TEF1_Q6**	33(1239)	1,50E-06	Cdkn2c, Lifr, Ank3, Rbm14, Crlf1, Cks1b, Asxl1, Dlg3, Egr2, Prx, Rest, Sin3a, Tgif2, Tnnt2, Pogz, Acsl3, Cdca3, Spred1, Tial1, Gprc5c, Pde1a, Slc9a3r1, Cnn3, Zic1, Rasal2, Hmga1, Rnf2, Jub, Plxnb1, Hmgb2, Swap70, Cdk2, Six4
**V$YY1_02**	20(1239)	1,51E-06	Cdkn2c, Ptbp2, Ctcf, Rad21, Nasp, Rif1, Pou2f1, Rpl30, Wsb1, Hspa8, Ier2, Dhx15, Upf3b, Tnks2, Pcf11, Tcf4, Mrpl1, Gtf3c2, Smyd5, Erh
**V$GATA1_01**	21(1239)	1,58E-06	Ank3, Syncrip, Crlf1, Suv39h1, Acly, Msh2, Ctcf, Sox12, Nab2, Aebp2, Pou2f1, Supt16h, Ptk7, Zic1, Plk4, Dhx15, Rasal2, Kpnb1, Cdc27, Bnc2, Hspa14
**V$USF2_Q6**	21(1239)	1,72E-06	Cdkn2c, Arpc5, Slc43a1, Cbx5, Stmn1, Ctbp2, Tial1, Adss, Pla2g4a, Ebna1bp2, Rbbp4, Net1, Sirt1, Fosl1, Pprc1, Ipo7, Nudc, Lrp8, Wee1, Ivns1abp, Fen1
**V$ARNT_02**	21(1239)	1,86E-06	Cdkn2c, Notch1, Syncrip, Nfx1, H2afz, Arpc5, Slc43a1, Cbx5, U2af2, Stmn1, Tgif2, Smndc1, Topors, Zic1, Apex1, Rbbp4, Hmga1, Sirt1, Eme1, Fen1, Ncl
**V$SP1_Q4_01**	21(1239)	1,86E-06	Hyal2, Ptpn2, Hnrpdl, Sox12, Smarce1, Trim28, Stmn1, Fus, Nasp, Tead2, Rbbp7, Cdc25a, Ptk7, Smarca5, Timeless, Cyp26b1, Sh3kbp1, Tcf4, Jub, Swap70, Slbp
**V$NERF_Q2**	21(1239)	2,23E-06	Hyal2, Chd2, Tfrc, Junb, Slc43a1, Nrp1, Rasa1, Tgif2, Rbbp7, Uchl5, Cdca3, Actr3, Egr3, Topors, Elk3, Hn1, Hmga1, Fosl1, Ranbp1, Tpp2, Spred2
**V$HNF4ALPHA_Q6**	22(1239)	2,67E-06	Pdgfb, Llgl1, Zbtb12, Nek6, Nutf2, Schip1, Nrp1, Usp37, Dnaja2, Stoml2, Nr2f2, Tial1, Hat1, Supt16h, Timeless, Fosl1, Prpf4b, Gtf3c2, Gart, Pabpn1, Fkbp5, Smyd5
**V$TATA_01**	63(1239)	2,73E-06	Cdkn2c, Ank3, Pdgfb, Top2a, H2afz, Chd2, Ppil5, Rcor1, Sema4b, Junb, Prkg2, Egr2, Mbtps2, Ctcf, Schip1, Bach1, Tbx3, Mat2a, Tslp, Cx3cl1, Syne2, Hist1h2ae, Nasp, Egfl7, Whsc1, Foxp1, Rbm12, Impdh2, Lif, Zfp36l2, Actr3, Pou2f1, Nr2f2, Snx5, Luc7l, Runx2, Ankrd32, Tes, Etv5, Dlx1, Pank1, Nav3, Ppbp, Hn1, Tpm3, Rasal2, Ebf2, Cyp26b1, Pum2, Mapk8, Arf6, Myc, Pcf11, Tcf4, Tmpo, Bzw2, Slc9a5, Nrp2, Myct1, Ect2, Bnc2, Satb2, Fhl2
**V$CEBP_Q2**	20(1239)	3,08E-06	Pdgfb, Suv39h1, Chd2, Stag1, Osmr, Foxp1, Pdap1, Nr2f2, Spred1, Pla2g4a, Atad2, Ptpn12, Skp2, Rasal2, Kin, Arf6, Tcf4, Mpp6, Nrp2, Ruvbl2
**V$PU1_Q6**	36(1239)	4,09E-06	Abi1, Ankrd28, Ncam1, Csf1, Chd2, Etv6, Hnrnpa3, Elovl6, Rest, Nrp1, Cx3cl1, Pold3, Sms, Lif, Trip13, Pou2f1, Ctbp2, Luc7l, Slc9a3r1, Ptk7, Tll1, Pla2g4a, Galnt4, Tpm3, Etf1, Timeless, Rab8b, Pprc1, Cct7, Tmpo, Ppp1cc, Psip1, Bnc2, Bcl2, Spred2, Cdc5l
**V$CREBP1CJUN_01**	21(1239)	4,46E-06	Ltbp1, Syncrip, Ncam1, Elavl1, Slc38a1, G3bp2, Stag1, Ddx51, Ell2, Etv6, Dhx36, Ccna2, Cenpe, Tgif2, Umps, Suv39h2, Egr1, Yme1l1, Cd2ap, Rad51c, Gnb4
**V$COUP_DR1_Q6**	20(1239)	5,32E-06	Cks1b, Slc11a2, Asxl1, Apex2, Zbtb12, Eno3, Rest, Tomm70a, Usp37, Dnaja2, Fbln1, Stoml2, Nr2f2, Hspe1, Clspn, Arf6, Ss18, Smyd5, Hspd1, Ahcyl1
**V$HNF3ALPHA_Q6**	19(1239)	5,67E-06	Abi1, Trib1, Chd2, Taf5l, Egr2, Foxp1, Stoml2, Pogz, Pik3c2a, Luc7l, Nav3, Ebf2, Pum2, H2afv, Dusp6, H2afy, Bub3, Pafah1b3, Tpp2
**V$SRF_Q6**	22(1239)	5,86E-06	Syncrip, G3bp2, Junb, Egr2, H2afx, Cx3cl1, Sin3a, Dusp5, Foxp1, Rbbp7, Actr3, Nr2f2, Sdpr, Matr3, Egr3, Asph, Tes, Egr1, Acaa2, Tpm3, Rrm1, Fosl1
**V$LEF1_Q6**	22(1239)	5,86E-06	Lifr, Ankrd28, Uhrf1, Syncrip, Chd2, Khdrbs1, Etv6, Baz1a, Tgif2, Mpzl1, Elk3, Ebf2, Sdc1, Dhx9, Sox11, Lrp8, Nrp2, Wee1, Ivns1abp, Pak1, Six4, Smarcc1
**V$MEIS1_01**	45(1239)	6,15E-06	Taf5, Cdkn2c, Gphn, Ckap4, Ncam1, H2afz, Chd2, Arpc5, Tnpo1, Ets1, Sox4, Klf5, Eif4enif1, Xpo7, Phc2, Vrk1, Baz1a, Nrp1, Tgif2, Spred1, Tial1, Top1, Vil1, Pde1a, Elk3, Xpo1, Nav3, Fbxo5, Runx1, Nfrkb, Sirt1, Cct7, Nxf1, Col7a1, Myc, Pcf11, Dpf1, Abl2, Gnb4, Nrf1, Psip1, Pabpn1, Dnmt1, Ss18, Bnc2
**V$SP1_Q6_01**	19(1239)	6,62E-06	Klf5, Ptpn2, Smarce1, Trim28, Idh3a, Stmn1, Cnnm4, Tead2, Shmt1, Suv39h2, Adss, Nfyb, Timeless, Cyp26b1, Net1, Tcf4, Jub, Ell, Ktn1
**V$HIF1_Q5**	20(1239)	7,94E-06	Ltbp1, Hyal2, Psmd5, Pdgfb, Lig1, Elovl6, Stmn1, Pdk3, Snx5, Tial1, Prdx4, Kpnb1, Hmga1, Lrp8, Bcl2l11, Nrf1, Slc9a5, Wee1, Bcl2, Ktn1
**V$CREB_Q4**	20(1239)	8,60E-06	Ltbp1, Trib1, Elavl1, Slc38a1, G3bp2, Fancd2, Ddx51, Socs4, Dhx36, Ctcf, Tgif2, Rbbp8, Egr3, Plk4, Cbx3, Cd2ap, Wdhd1, Ubqln2, Dnajc9, Pak1
**V$AP2_Q6**	20(1239)	9,31E-06	Mmd, Crlf1, Pdgfb, Ctcf, Sox12, Trim28, Rasa1, Pold3, Cnnm4, Tead2, Gata2, Cdc25a, Pou2f1, Spry2, Rasal2, Sdc1, Sirt1, Nrf1, Exosc8, Bcl2
**V$FOX_Q2**	19(1239)	9,32E-06	Ncam1, Junb, Prkar2b, Egr2, Ctcf, Crim1, Cdh10, Stoml2, Pogz, Rpl7a, Pik3c2a, Gprc5c, Ebf2, Cyp26b1, Mcm7, H2afv, H2afy, Swap70, Tpp2
**V$AHR_Q5**	18(1239)	1,09E-05	Sf1, H2afz, Set, Stag1, Hnrpdl, Usp37, Rbbp7, Pou2f1, Nr2f2, Supt16h, Asph, Prdx4, Runx1, Tnks2, Jub, Bzw2, Nrf1, Slc9a5
**V$ETS1_B**	20(1239)	1,09E-05	Hyal2, Pdgfb, Junb, Etv6, Amd1, Elovl6, Tgif2, Foxp1, Lif, Ruvbl1, Spred1, Luc7l, Nme1, Slc9a3r1, Elk3, Etf1, Hmga1, Fosl1, Tpp2, Spred2
**V$AREB6_01**	46(1239)	1,25E-05	Cdkn2c, Sf1, Notch1, Ncam1, Trib1, Crlf1, Pdgfb, Slc38a1, G3bp2, Rrad, Klf5, Ptpn2, Egr2, Sox12, Nup54, Nrp1, Tgif2, Foxp1, Gata2, Eif5, Stoml2, Socs5, Gprc5c, Egr3, Spry2, Ptk7, Zic1, Etv5, Il13ra1, Hn1, Rbmx2, Rrm1, Cyp26b1, Rab8b, Sh3kbp1, Nedd4l, Tnks2, Has2, Bzw2, Ell, Bub3, Hmga2, Chst1, Pafah1b3, Six4, Etv4
**V$HNF3_Q6**	43(1239)	1,27E-05	Abi1, Ltbp1, Ncam1, Slc29a1, Asxl1, Sox4, Nek6, Junb, Klf5, Egr2, Prx, Ctcf, Cbfb, Ppat, Rad21, Syne2, Egfl7, Foxp1, Cdh10, Stoml2, Pogz, Cdc25a, Pik3c2a, Nr2f2, Luc7l, Gprc5c, Gemin4, Slc9a3r1, Zic1, Tll1, Nav3, Ebf2, Rnmt, Brca1, Cyp26b1, Pum2, H2afv, Dusp6, Tcf4, H2afy, Bzw2, Ell, Pafah1b3
**V$OCT1_02**	19(1239)	1,29E-05	Cdkn2c, Ank3, Ltbp1, Ahcy, Arpc5, Rest, Foxp1, Pou2f1, Tial1, Pde1a, Zic1, Dlx1, Runx1, Tcf4, Rnf2, Hmgb2, Bub3, Aspm, Bnc2
**V$OCT1_05**	20(1239)	1,35E-05	Ptpn2, Egr2, Rhobtb3, Cnnm4, Pou2f1, Pank4, Nfyb, Elk3, Dlx1, Topbp1, Enpp1, Dusp6, Cdk2, Gprc5b, Cdca7, Nrp2, Bnc2, Hmgb1, Satb2, Bcl2

Transcription Factor	*Number of genes*	*Corrected p-value*	*Genes repressed in Rasless cells (from Table S1)*
**V$RORA1_01**	20(1239)	1,45E-05	Chd2, Rcor1, Socs4, Eno3, Nrp1, Cx3cl1, Nr2f2, Hspe1, Asph, Slc4a7, Tpm3, Arf6, Eme1, Bzw2, Mpp6, Psip1, Chst1, Ivns1abp, Hspd1, Ythdf2
**V$PAX6_01**	12(1239)	1,56E-05	Taf5, H2afz, Sox4, Rhobtb3, Baz1a, Stoml2, Slc5a3, Sdpr, Nfyb, Rrm1, Hmga1, Tcf4
**V$STAT1_03**	19(1239)	1,62E-05	Sema4b, Sox4, Elovl6, Tcerg1, Sin3a, Dnaja2, Hspe1, Cbx3, Dlx1, Bclaf1, Skp2, Brca1, Rab8b, Pcf11, Jub, Gspt1, Pabpn1, Hspd1, Csnk1a1
**V$FOXD3_01**	18(1239)	1,77E-05	Ckap4, Ankrd28, Junb, Egr2, Csnk1g3, Crim1, H2afx, Nrp1, Stoml2, Pogz, Pik3c2a, Etv5, Runx1, Cyp26b1, Cxadr, Has2, Marcks, Bnc2
**V$HFH8_01**	18(1239)	1,77E-05	Ankrd28, Asxl1, Egr2, Xpo7, Yes1, Rad21, Kif21a, Impdh2, Pogz, Chd1, Pou2f1, Etv5, Runx1, Cyp26b1, Usp3, Marcks, Bub3, Fhl2
**V$USF_02**	20(1239)	1,79E-05	Cdkn2c, Crlf1, Nfx1, Mthfd1, Fmr1, Cbx5, Tcerg1, Mat2a, Ilf3, Umps, Topors, Xpo1, Runx1, Sirt1, Npm1, H2afy, Eme1, Nudc, Prps1, Fen1
**V$NFAT_Q6**	19(1239)	2,03E-05	Ankrd28, Rbm14, Pdgfb, Ghr, Phf6, Foxp1, Lif, Sgol1, Nr2f2, Tial1, Zic1, Etv5, Tll1, Elk3, Safb, Arf6, Marcks, Anp32e, Bnc2
**V$ATF_01**	20(1239)	2,07E-05	Trib1, Elavl1, Ddx51, Dhx36, Egr2, Ssr4, Ccna2, Tgif2, Top1, Egr3, Etf1, Yme1l1, Cd2ap, Marcks, Dusp4, Cdk2, Gnb4, Gart, Pak1, Spred2
**V$AP1_C**	53(1239)	2,17E-05	Cdkn2c, Ankrd28, Ank3, Trib1, Slc38a1, Trim25, Asxl1, Nek6, Klf5, Sertad1, Map4k5, Prx, Gli2, Osmr, Slc25a24, Eno3, Bach1, Ube2e3, Nasp, Pdap1, Stoml2, Rbbp7, Acsl3, Spred1, Tial1, Luc7l, Nme1, Vil1, Asph, Etv5, Tll1, Elk3, Igsf3, Dlx1, Runx1, Nfrkb, Ebf2, Ewsr1, Usp3, Hmga1, Fosl1, Col7a1, Tnks2, Pkn3, Ube2c, Ell, Bub3, Nrp2, Chst1, Fkbp5, Csnk1a1, Etv4, Tpbg
**V$CMYB_01**	19(1239)	2,18E-05	Rbm14, Pdgfb, Lyar, Snrpd1, Klf5, Hnrnpa3, Stmn1, Aebp2, Pogz, Egr3, Dlx1, Tpm3, Kpnb1, Cdc27, Sh3kbp1, Arf6, Ranbp1, Ruvbl2, Etv4
**V$PAX4_04**	18(1239)	2,22E-05	Asxl1, Sox4, Foxp1, Cdh10, Impdh2, Stoml2, Pik3c2a, Slc5a3, Supt16h, Pde1a, Sdpr, Timeless, Ebf2, Nedd4l, Tcf4, Marcks, Dusp4, Six4
**V$ETS_Q4**	19(1239)	2,31E-05	Hyal2, Pdgfb, Chd2, Strbp, Junb, Amd1, Tcerg1, Nasp, Foxp1, Pdap1, Lif, Actr3, Slc9a3r1, Etv5, Elk3, Etf1, Usp3, Hmga1, Tpp2
**V$CETS1P54_01**	18(1239)	2,39E-05	Chd2, Cks1b, Fmr1, Ctcf, U2af2, Snrpb, Tomm70a, Nasp, Egfl7, Dnaja2, Stoml2, Spry2, Ebna1bp2, Tnks2, Ipo7, Gspt1, Dnmt1, Ppil1
**V$BRN2_01**	19(1239)	2,65E-05	Ctnna1, Cbfb, Baz1a, Anln, Impdh2, Iqgap3, Cnn3, Etv5, Dlx1, Polr3b, Rab8b, Arf6, Dusp6, Fosl1, Pcf11, Tcf4, Ell, Myo1b, Etv4
**V$CREB_Q3**	19(1239)	2,65E-05	Cdkn2c, Ncam1, Chd2, Nolc1, Nup155, Rnps1, Nufip1, Usp14, Pogz, Hspe1, Spry2, Etv5, Rrm1, Gtf3c2, Arid4b, Prpf31, Pak1, Hspd1, Spred2
**V$MYB_Q5_01**	19(1239)	2,65E-05	Gphn, Ankrd28, Ltbp1, Kif20a, Brd8, G3bp2, Rasa3, Klf5, Xpo7, Rhobtb3, Shmt1, Cdca3, Xpo1, Pgm1, Gtf3c2, Cdk2, Hmga2, Six4, Etv4
**V$CEBP_C**	17(1239)	2,79E-05	Nfx1, Chd2, Sox4, Klf5, Osmr, Crim1, Foxp1, Pik3c2a, Luc7l, Zic1, Dlx1, Pla2g4a, Ptpn12, Phactr4, Etf1, Rrm1, Epc2
**V$ATF3_Q6**	33(1239)	2,81E-05	Ltbp1, Trib1, Slc38a1, G3bp2, Rrad, Ddx51, Ell2, Dhx36, Prx, Ccna2, Schip1, Cenpe, Ch25h, Tgif2, Egfr, Vrk3, Nck2, Top1, Iqgap3, Umps, Suv39h2, Cnn3, Tes, Runx1, Tpm3, Yme1l1, Cd2ap, Jub, Arid4b, Rad51c, Gnb4, Pak1, Hbs1l
**V$SP1_01**	19(1239)	2,81E-05	Hyal2, Syncrip, Acly, Ssr4, Stmn1, Sec63, Pou2f1, Nr2f2, Kpnb1, Cyp26b1, Rbbp4, Hmga1, Pprc1, Pcf11, Bzw2, Pak1, Ccne1, Bcl2, Six4
**V$HMGIY_Q6**	19(1239)	3,01E-05	Taf5, Ankrd28, Ncam1, Ptbp2, Klf5, Osmr, Stmn1, Pold3, Foxp1, Lif, Pou2f1, Tial1, Stip1, Zic1, Rasal2, Rrm1, Cdc27, Dusp6, Nedd4l
**V$CEBPA_01**	19(1239)	3,01E-05	Notch1, Syncrip, Trib1, Pdgfb, Osmr, Crim1, Egfr, Nr2f2, Spred1, Dlx1, Pla2g4a, Rasal2, Rbbp4, Pum2, Arf6, Sox11, Nrp2, Ruvbl2, Usp34
**V$POU1F1_Q6**	18(1239)	3,16E-05	Ank3, Uhrf1, Sox4, Dusp9, Asf1a, Crim1, Nrp1, H2afy2, Stoml2, Pou2f1, Tial1, Zic1, Dlx1, Runx1, Ebf2, Pcf11, Bzw2, Bnc2
**V$SRF_Q5_01**	19(1239)	3,19E-05	Chd2, G3bp2, Junb, Taf5l, Egr2, H2afx, Dusp5, Foxp1, Rbbp7, Actr3, Nr2f2, Fgf7, Sdpr, Egr3, Egr1, Ier2, Tpm3, Arf6, Bnc2
**V$ATF1_Q6**	19(1239)	3,19E-05	Trib1, Slc38a1, Ell2, Rnps1, Cenpe, Tgif2, Foxp1, Top1, Pde1a, Suv39h2, Cnn3, Cd2ap, Sox11, Dyrk3, Gnb4, Nrf1, Ivns1abp, Pak1, Spred2
**V$FAC1_01**	17(1239)	3,48E-05	Junb, Eps8, Map4k5, Cbfb, Foxp1, Nr2f2, Slc5a3, Ccng2, Zic1, Nav3, Runx1, Brca1, Has2, Nrp2, Six4, Csnk1a1, Usp34
**V$COUP_01**	19(1239)	3,66E-05	Pdgfb, Cks1b, Pold1, Slc11a2, Asxl1, Nek6, Mtmr4, Nrp1, Tomm70a, Usp37, Dnaja2, Fbln1, Stoml2, Nr2f2, Timeless, Net1, Pabpn1, Fkbp5, Smyd5
**V$CREB_Q2**	19(1239)	3,66E-05	Ltbp1, Trib1, Elavl1, Slc38a1, Ddx51, Dhx36, Ctcf, Tgif2, Sms, Pdap1, Rbbp8, Egr3, Plk4, Egr1, Cbx3, Etf1, Cd2ap, Ubqln2, Dnajc9
**V$DR1_Q3**	18(1239)	4,54E-05	Ckap4, Asxl1, Zbtb12, Rest, Tomm70a, Usp37, Dnaja2, Fbln1, Stoml2, Nr2f2, Hspe1, Timeless, Net1, Gtf3c2, Fkbp5, Ss18, Smyd5, Hspd1
**V$RP58_01**	18(1239)	4,54E-05	Ltbp1, Pdgfb, Klf5, Amd1, Dlg3, Kif21a, Foxp1, Fgf7, Fmnl3, Ptk7, Tll1, Runx1, Pprc1, Col7a1, Colec12, Arhgap22, Gpsm2, Spred2
**V$VDR_Q3**	18(1239)	4,54E-05	Ncam1, Chd2, Asxl1, Prx, Xpo7, Ctcf, Sin3a, Sec63, Lif, Pogz, Pou2f1, Rcl1, Ptk7, Xpo1, Smarca5, Sh3kbp1, Cct7, Jub
**V$PITX2_Q2**	38(1239)	4,69E-05	Lifr, Ankrd28, Ltbp1, Trib1, Sox4, Klf5, Prx, Slc43a3, Gli2, Ctcf, Phc2, Nup54, Dusp5, Khsrp, Pou2f1, Slc5a3, Ctbp2, Luc7l, Supt16h, Pde1a, Spry2, Igsf3, Slc4a7, Acaa2, Nav3, Runx1, Rasal2, Cd2ap, Arf6, Dusp6, Tcf4, Bzw2, Chst1, Bnc2, Hmgb1, Bcl2, Ktn1, Myo1b
**V$PTF1BETA_Q6**	18(1239)	4,82E-05	Ank3, Chd2, Ptbp2, Sox4, Etv6, Ptpn2, Mtmr4, Ctcf, Rest, Rasa1, Hmgb3, Rif1, Lif, Stoml2, Zic1, Xpo1, Gspt1, Bcl2
**V$OSF2_Q6**	19(1239)	4,84E-05	Ncam1, Stag1, Mtmr4, Amd1, Phf6, Csnk1g3, Sin3a, Rpa2, Pou2f1, Supt16h, Gprc5c, Runx2, Zic1, Runx1, Etf1, Sirt1, Tcf4, H2afy, Akap8
**V$USF_Q6_01**	17(1239)	5,07E-05	Trib1, Nfx1, Hnrpdl, Slc43a1, Schip1, Stmn1, Tgif2, Socs5, Ilf3, Tll1, Apex1, Ewsr1, Sirt1, Ipo7, Npm1, Ranbp1, Wee1
**V$GATA4_Q3**	18(1239)	7,66E-05	Lifr, Ankrd28, Sf1, Ets1, Amd1, Smarce1, Nrp1, Egfl7, Lif, Nr2f2, Egr3, Zic1, Rasal2, Arf6, Nedd4l, Pcf11, Ect2, Bnc2

Transcription Factor	*Number of genes*	*Corrected p-value*	*Genes repressed in Rasless cells (from Table S1)*
**V$POU6F1_01**	18(1239)	8,22E-05	Ncam1, Chd2, Sox4, Nek6, Baz1a, Foxp1, Stoml2, Pou2f1, Pank4, Etv5, Tll1, Dlx1, Nfrkb, Marcks, Mpp6, Epc2, Bnc2, Fbxo32
**V$MAF_Q6**	19(1239)	8,39E-05	Hyal2, Syncrip, Chd2, Unc5c, Clns1a, Map4k5, Elovl6, Hmgb3, Supt16h, Slc9a3r1, Ttc3, Timeless, Kpnb1, Hmga1, Has2, Bnc2, Six4, Cdc5l, Tpbg
**V$CEBPB_01**	19(1239)	8,39E-05	Syncrip, Trib1, Chd2, Rcor1, Klf5, Crim1, Foxp1, Pdap1, Zic1, Prdx4, Dlx1, Acaa2, Pla2g4a, Etf1, Pcf11, Marcks, Nrp2, Srpk1, Ruvbl2
**V$RSRFC4_Q2**	29(1239)	8,48E-05	Nek2, Rrad, Rcor1, Amd1, Taf5l, Egr2, Prx, Rhobtb3, Eno3, Mat2a, Foxp1, Lif, Stoml2, Luc7l, Asph, Pank1, Tpm3, Cyp26b1, Tcf4, Has2, Bzw2, Epc2, Gnb4, Slc9a5, Nrp2, Tpp2, Bnc2, Satb2, Ktn1
**V$PBX1_01**	18(1239)	8,63E-05	Ltbp1, Chd2, Junb, Mrps6, Slc43a3, Schip1, Stmn1, Dusp5, Lif, Supt16h, Gemin4, Zic1, Dlx1, Ebf2, Pgm1, Nedd4l, Swap70, Bnc2
**V$FOXJ2_02**	17(1239)	9,39E-05	Lifr, Ltbp1, Chd2, Ghr, Phf6, Crim1, Foxp1, Cdh10, Fgf7, Pvrl3, Ttc3, Arf6, Tcf4, Has2, Nrf1, Ect2, Satb2
**V$MYB_Q3**	17(1239)	0,000109332	Taf5, Gphn, Ankrd28, Ltbp1, Rasa3, Klf5, Rhobtb3, Foxp1, Pdap1, Shmt1, Cdca3, Xpo1, Gtf3c2, Cdk2, Nrf1, Hmga2, Etv4
**V$SOX5_01**	18(1239)	0,00011422	Ankrd28, Ank3, Ncam1, G3bp2, Nrp1, Dusp5, Nasp, Tial1, Fmnl3, Dlx1, Rbbp4, Dusp6, Tnks2, Tcf4, Exosc2, Pak1, Bnc2, Satb2
**V$MTF1_Q4**	18(1239)	0,00012134	Mtmr4, Lig1, Xpo7, U2af2, Khsrp, Rbbp7, Cdc25a, Pou2f1, Tial1, Egr1, Elk3, Rasal2, Hmga1, H2afv, Tnks2, Marcks, Bzw2, Nrf1
**V$HLF_01**	18(1239)	0,00012134	Cdkn2c, Chd2, Hnrnpa3, Xpo7, Crim1, Egfr, Foxp1, Cdh10, Dnaja2, Pdap1, Pogz, Nr2f2, Spred1, Top1, Dlx1, Atad2, Mpp6, Etv4
**V$ER_Q6_01**	18(1239)	0,000149685	Cdkn2c, Lifr, Chd2, Asxl1, Apex2, Cldn12, Stmn1, Dnaja2, Zfp36l2, Spred1, Pde1a, Zic1, Dgcr8, Slc4a7, Tpm3, Rnmt, Sdc1, Slc25a5
**V$HFH4_01**	24(1239)	0,000165142	Ncam1, Asxl1, Egr2, Prx, Elovl6, Crim1, Schip1, Rad21, Tbx3, Sin3a, Cdh10, Pogz, Pik3c2a, Nr2f2, Luc7l, Iqgap3, Nav3, Runx1, Ebf2, Cyp26b1, H2afv, Tcf4, H2afy, Bub3
**V$HP1SITEFACTOR_Q6**	17(1239)	0,000166373	Ankrd28, Syncrip, Chd2, Rcor1, Sox4, Casp8, Ctcf, Axl, Schip1, Pou2f1, Luc7l, Zic1, Dlx1, Xpo1, Pum2, H2afy, Bnc2
**V$FOXJ2_01**	15(1239)	0,000182507	Ncam1, Bub1, Egr2, Rest, Ch25h, Cdh10, Stoml2, Pogz, Pik3c2a, Luc7l, Runx2, Cyp26b1, Pcf11, Bub3, Fen1
**V$MEF2_02**	38(1239)	0,000188089	Lifr, Ankrd28, Ncam1, Chd2, Arpc5, Nek2, Rcor1, Ets1, Sox4, Cldn12, Junb, Klf5, Map4k5, Prkar2b, Ctnna1, Foxp1, Tnnt2, Nab2, Top1, Asph, Tll1, Pank1, Rrs1, Rasal2, Cdc27, Enpp1, Has2, Spc25, Bcl2l11, Gnb4, Slc9a5, Nrp2, Tpp2, Bnc2, Snx7, Ktn1, Ssrp1, Tpbg
**V$FOXO1_02**	17(1239)	0,000189242	Lifr, Ankrd28, Kif20a, Brd8, Chd2, Asxl1, Map4k5, Xpo7, Csnk1g3, Rad21, Sin3a, Pik3c2a, Pou2f1, Etv5, Ddx46, Pak1, Satb2
**V$MYOD_01**	17(1239)	0,000216082	Hyal2, Crlf1, H2afz, Eif4enif1, Sox12, Bach1, Tnnt2, Rangap1, Slc5a3, Luc7l, Ptk7, Egr1, Usp3, Pcf11, Psip1, Srpk1, Ktn1
**V$TEF_Q6**	17(1239)	0,000216082	Sox4, Amd1, Egr2, Ctcf, Crim1, Nrp1, Foxp1, Spred1, Fgf7, Spry2, Etv5, Ebf2, Pum2, Pcf11, Gprc5b, Bub3, Bnc2
**V$TCF4_Q5**	17(1239)	0,000230693	Lifr, Ankrd28, Chd2, Map4k5, Pcm1, Sdpr, Zic1, Elk3, Dlx1, Pank1, Plscr1, Ebf2, Has2, Ucp2, Nrp2, Six4, Usp34
**V$ZID_01**	16(1239)	0,000258344	Ltbp1, Chd2, Apex2, Map4k5, Ctcf, Foxp1, Cdc25a, Supt16h, Zic1, Adss, Elk3, Enpp1, Pcf11, Tcf4, Marcks, Hmgb1
**V$CREB_02**	17(1239)	0,000262495	Chd2, Ddx51, Ell2, Dhx36, Sox12, Snrpg, Cenpe, Sms, Rbbp8, Umps, Plk4, Smarca5, Cd2ap, Ivns1abp, Pak1, Hmgb1, Spred2
**V$AML_Q6**	23(1239)	0,000273591	Lifr, Ltbp1, Trib1, Nek6, Mtmr4, Amd1, Phf6, Nutf2, Rbl1, Cnnm4, Lif, Pou2f1, Luc7l, Supt16h, Runx2, Nav3, Runx1, Etf1, Kpnb1, Sirt1, Pcf11, H2afy, Bnc2
**V$SRY_02**	17(1239)	0,00027812	G3bp2, Nrp1, Dusp5, Gata2, Chd1, Tial1, Ebf2, Mcm7, Rbbp4, Dusp6, Pcf11, Tcf4, H2afy, Mpp6, Pak1, Bnc2, Six4
**V$CDC5_01**	18(1239)	0,000292664	Cdkn2c, Chd2, Ppil5, Rcor1, Nek6, Egr2, Csnk1g3, Nrp1, Rpa2, Foxp1, Sec63, Supt16h, Rasal2, Sdc1, Hmga1, Atrx, Gli3, Prps1
**V$PAX4_02**	17(1239)	0,000292839	Chd2, Slc38a1, Sox4, Etv6, Foxp1, Zfp36l2, Pogz, Sdpr, Zic1, Elk3, Dlx1, Ebf2, Tcf4, Has2, Marcks, Bzw2, Mpp6
**V$FOXO1_01**	17(1239)	0,000292839	Ankrd28, Asxl1, Elovl6, Csnk1g3, H2afx, Impdh2, Chd1, Ccng2, Gprc5c, Etv5, Dlx1, Runx1, Usp3, Mcm7, H2afv, Klhdc2, Bub3
**V$CREB_01**	17(1239)	0,000310012	Ltbp1, Elavl1, Slc38a1, G3bp2, Ddx51, Ell2, Dhx36, Ccna2, Cenpe, Tgif2, Umps, Suv39h2, Plk4, Cd2ap, Rad51c, Gnb4, Pak1
**V$CEBP_Q3**	17(1239)	0,000310012	Syncrip, Ncam1, Pdgfb, Suv39h1, Junb, Nup155, Syne2, Nr2f2, Spred1, Etv5, Pla2g4a, Pum2, Mpp6, Dyrk3, Ss18, Ruvbl2, Usp34
**V$AP4_Q6_01**	17(1239)	0,000328044	Hyal2, Notch1, Mycn, Pdgfb, Ccne2, Eif4enif1, Sox12, Bach1, Ppat, Tnnt2, Sumo2, Hn1, Myc, Psip1, Bnc2, Ythdf2, Trit1
**V$SMAD_Q6**	17(1239)	0,000328044	Ltbp1, Pdgfb, Cks1b, Prx, Cbx5, Crim1, Nrp1, Stmn1, Dusp5, Rbm12, Runx2, Elk3, Rrm1, Sh3kbp1, Jub, Anp32e, Bcl2
**V$E2F_Q2**	13(1239)	0,000332521	Usp1, Stmn1, Sfpq, Pogz, Pou2f1, Mpzl1, Suv39h2, Tpm3, Rbbp4, Rnf2, Lrp8, Anp32e, Ythdf2
**V$STAT5A_03**	17(1239)	0,000346971	Mns1, Etv6, Amd1, Vrk1, Stmn1, Pole2, Spred1, Tial1, Ptk7, Sumo2, Tpm3, Ebf2, Cxadr, Nedd4l, Tcf4, Marcks, Gnb4
**V$HFH3_01**	15(1239)	0,000360869	Ncam1, Egr2, Prx, Crim1, Rest, Rad21, Cdh10, Stoml2, Pik3c2a, Gprc5c, Runx2, Etv5, Nav3, Cyp26b1, Bub3
**V$GR_Q6_01**	17(1239)	0,000366829	Gpr126, Ank3, Syncrip, Pdgfb, Ets1, Khdrbs1, Etv6, Schip1, Bach1, Stmn1, Whsc1, Tead2, Pdap1, Sdc1, Nrp2, Fen1, Bcl7a
**V$ZIC1_01**	17(1239)	0,000389785	Notch1, Pdgfb, Unc5c, Fancd2, Etv6, Ghr, Tgif2, Rbm12, Aebp2, Pou2f1, Supt16h, Gemin4, Ptk7, Rrm1, Cyp26b1, Col7a1, Lrp8
**V$STAT6_01**	17(1239)	0,000441811	Trib1, Slc38a1, Etv6, Amd1, Vrk1, Stmn1, Pole2, Spred1, Tial1, Stip1, Ptk7, Sumo2, Cxadr, Nedd4l, Tcf4, Marcks, Gnb4
**V$CREBP1_Q2**	16(1239)	0,000446132	Ltbp1, Trib1, Elavl1, Ddx51, Ell2, Dhx36, Egr2, Tgif2, Rbbp8, Egr3, Suv39h2, Egr1, Cbx3, Yme1l1, Cd2ap, Ubqln2
**V$COMP1_01**	11(1239)	0,000467343	Ncam1, Phf6, Ilf3, Slc5a3, Zic1, Nfyb, Dlx1, Nav3, Ebf2, Gnb4, Hmgb1
**V$NRF2_01**	15(1239)	0,000469357	Arpc5, Cks1b, Mtmr4, Rnps1, Fmr1, Egr2, Phf6, Baz1a, Eif2s1, Tomm70a, Chuk, Tnks2, Gspt1, Dnmt1, Bcl2
**V$TST1_01**	17(1239)	0,000491701	Ankrd28, Stag1, Nek6, Klf5, Egr2, Ccnf, Lif, Nr2f2, Slc5a3, Pde1a, Zic1, Rad18, Cxadr, Atrx, Has2, Hmga2, Bnc2
**V$DR3_Q4**	11(1239)	0,00050819	Hyal2, Rbm14, Pdgfb, Chd2, Asxl1, Pou2f1, Xpo1, Ttc3, Kpnb1, Cdc27, Tmpo

Transcription Factor	*Number of genes*	*Corrected p-value*	*Genes repressed in Rasless cells (from Table S1)*
**V$MMEF2_Q6**	18(1239)	0,000555856	Rcor1, Junb, Prx, Sin3a, Tomm70a, Dnaja2, Pou2f1, Luc7l, Runx2, Asph, Topbp1, Sdc1, Cyp26b1, Has2, Bzw2, Gnb4, Nrp2, Bnc2
**V$MSX1_01**	14(1239)	0,000558492	Sox4, Ell2, Cbx5, Foxp1, Cirh1a, Pou2f1, Spry2, Zic1, Etv5, Dlx1, Ebf2, Nxf1, H2afy, Spag5
**V$OCT1_04**	16(1239)	0,000562709	Ank3, Ncam1, Ahcy, Chd2, Arpc5, Tpd52, Foxp1, Stoml2, Pou2f1, Pde1a, Zic1, Dlx1, Topbp1, Runx1, Arf6, Bnc2
**V$HNF6_Q6**	16(1239)	0,000594803	Abi1, Ankrd28, Chd2, Sox4, Mat2a, Cdh10, Stoml2, Pogz, Pou2f1, Zic1, Cbx3, Runx1, Nup107, Ebf2, Bnc2, Etv4
**V$ZIC2_01**	16(1239)	0,000594803	Mycn, Syncrip, Fancd2, Etv6, Ghr, Prx, Sox12, Phf6, Sfpq, Foxp1, Rbm12, Ptk7, Cyp26b1, Col7a1, Lrp8, Lrrk1
**V$SF1_Q6**	20(1239)	0,00068937	Ankrd28, Chd2, Tfrc, Fancd2, Socs4, Idh3a, Cx3cl1, Hmgb3, Dnaja2, Hspe1, Pank1, Sumo2, Safb, Kin, Spc24, Ipo7, Bub1b, Ranbp1, Ccne1, Hspd1
**V$PPAR_DR1_Q2**	16(1239)	0,00071124	Mmd, Asxl1, Zbtb12, Eno3, Rest, Tomm70a, Usp37, Dnaja2, Stoml2, Nr2f2, Rcl1, Slc25a5, Net1, Mrpl1, Ss18, Smyd5
**V$FREAC3_01**	16(1239)	0,00071124	Ankrd28, Ppil5, Map4k5, Egr2, Xpo7, Pogz, Pou2f1, Tial1, Nav3, Mcm7, Pcf11, Mpp6, Bub3, Pak1, Bnc2, Etv4
**V$MAZR_01**	15(1239)	0,00072366	Sf1, Junb, Hnrpdl, Cbfb, Rbl1, Fus, Aebp2, Pogz, Etv5, Etf1, Cdc27, Hmga1, Ezh2, Pcf11, Gart
**V$TCF11MAFG_01**	23(1239)	0,000725145	Pdgfb, Pop1, Mtmr4, Dlg3, Txnrd1, Bach1, Tomm70a, Foxp1, Eif5, Rbbp7, Usp14, Spred1, Tial1, Sdpr, Tll1, Sumo2, Il13ra1, Tpm3, Cd2ap, Fosl1, Ran, Anp32e, Usp34
**V$AP1_Q4**	17(1239)	0,00079951	Lifr, Ankrd28, Trib1, Nek6, Bach1, Pdap1, Stoml2, Rbbp7, Tial1, Zic1, Etv5, Elk3, Nfrkb, Usp3, Pkn3, Ube2c, Slc9a5
**V$NKX22_01**	13(1239)	0,000800016	Gpr126, Cdkn2c, Notch1, Egr2, Schip1, Foxp1, Aebp2, Rbbp7, Sdpr, Etv5, Sh3kbp1, Cct7, Nrp2
**V$FOXO4_02**	16(1239)	0,000837975	Kif20a, Brd8, Chd2, Asxl1, Klf5, Elovl6, Xpo7, Etv5, Runx1, Usp3, Mcm7, Has2, Mpp6, Bub3, Satb2, Bcl7a
**V$GATA1_05**	17(1239)	0,000839726	Gpr126, Klf5, Map4k5, Egr2, Ctcf, Syne2, Sin3a, Tgif2, Egfl7, Nr2f2, Sdpr, Zic1, Ptpn12, Nfrkb, Pum2, Myct1, Ect2
**V$CACBINDINGPROTEIN_Q6**	15(1239)	0,00091614	Ncam1, Pdgfb, Pou2f1, Supt16h, Dlx1, Eef1g, Kntc1, Kpnb1, Sdc1, Nedd4l, Dpf1, Gnb4, Pabpn1, Bcl7a, Six4
**V$AFP1_Q6**	16(1239)	0,000934094	Ankrd28, Sox4, Mtmr4, Egfr, Foxp1, Sdpr, Zic1, Dlx1, Cyp26b1, Pum2, Dusp6, Myc, Has2, Ect2, Satb2, Usp34
**V$ATF4_Q2**	16(1239)	0,000983118	Ltbp1, Trib1, Chd2, Ddx51, Dhx36, Ctcf, Cenpe, Pdap1, Usp14, Rbbp8, Cbx3, Xpo1, Hn1, Ubqln2, Pak1, Cdc5l
**V$TAXCREB_01**	11(1239)	0,000983825	Asxl1, Ctcf, Usp14, Plk4, Smarca5, Cd2ap, Slc25a5, Pcf11, Marcks, Gspt1, Pak1
**V$STAT6_02**	16(1239)	0,00103427	Gphn, Ankrd28, Trim25, Sema4b, Slc43a1, Ctcf, Adsl, Egfl7, Dnaja2, Luc7l, Tll1, Cd2ap, Hmga1, Ly75, Rnf138, Pkn3
**V$STAT_01**	16(1239)	0,00103427	Ank3, Trim25, Set, Asxl1, Nek6, Tcerg1, Nrp1, Rasa1, Asph, Etv5, Bclaf1, Plscr1, Sdc1, Polr2b, Mpp6, Dusp4
**V$OCT1_Q6**	16(1239)	0,00115967	Ank3, Egr2, Sox12, Lif, Socs5, Nfyb, Dlx1, Ebf2, Dusp6, Tcf4, Cdk2, Gprc5b, Cdca7, Nrp2, Satb2, Bcl2
**V$DR4_Q2**	15(1239)	0,00121212	Pdgfb, Amd1, Cx3cl1, Tgif2, Foxp1, Acsl3, Ctbp2, Ptk7, Dlx1, Ebf2, Pum2, Arid4b, Gart, Bnc2, Hmgb1
**V$ELF1_Q6**	15(1239)	0,00121212	Ankrd28, Sf1, Ncam1, Csf1, Dapp1, Ctcf, Baz1a, Nrp1, Nasp, Dnaja2, Rcl1, Supt16h, Marcks, Six4, Spred2
**V$SRF_01**	7(1239)	0,0012719	Junb, Egr2, Actr3, Egr3, Egr1, Ier2, Fosl1
**V$PAX2_02**	16(1239)	0,00127344	Cdc20, Nup54, Egfr, Eif5, Etv5, Elk3, Xpo1, Galnt4, Rasal2, Sdc1, Rpl13a, Tnks2, Tcf4, Marcks, Mpp6, Dusp4
**V$HSF2_01**	15(1239)	0,0012775	Cct3, Trib1, Pdgfb, Nek6, Mat2a, Cct8, Eif5, Egr3, Ppid, Hspa8, Kntc1, Ewsr1, Bnc2, Six4, Hspd1
**V$GATA1_02**	15(1239)	0,00133336	Lifr, Suv39h1, Chd2, Egr2, Ctcf, Schip1, Sh3kbp1, Sox11, Jub, Ccrn4l, Nrp2, Ect2, Bnc2, Satb2, Ccne1
**V$LYF1_01**	16(1239)	0,00133682	Impa2, Asxl1, Nek6, Hnrnpa3, Nrp1, Syne2, Tead2, Tes, Pank1, Timeless, Cdc27, Dusp6, Cdc40, Tcf4, Mpp6, Bnc2
**V$CEBP_01**	16(1239)	0,00133682	Ltbp1, Acly, Nup155, Egr2, Rest, Syne2, Foxp1, Stoml2, Top1, Supt16h, Sumo2, Atrx, Kcnk2, Epc2, Bnc2, Ddx52
**V$FREAC7_01**	13(1239)	0,00140857	Junb, Csnk1g3, Ch25h, Stoml2, Pogz, Pik3c2a, Pou2f1, Runx2, Etv5, Elk3, Nfrkb, Bub3, Bnc2
**V$P300_01**	15(1239)	0,00149133	Syncrip, Cks1b, Ptbp2, Junb, Crim1, Foxp1, Ctbp2, Nme1, Egr3, Zic1, Dlx1, Sumo2, Pum2, Arf6, Six4
**V$SRY_01**	13(1239)	0,00149547	Cdkn2c, Ckap4, Ank3, Ncam1, Egr2, Dffb, Stmn1, Pik3c2a, Nav3, Dusp6, Tnks2, Pcf11, Arid4b
**V$GR_01**	13(1239)	0,00159386	Syncrip, G3bp2, Ets1, Khdrbs1, Klf5, Eif4enif1, Dnaja2, Tead2, Rbbp7, Sdc1, Pum2, Ppp1cc, Fen1
**V$PAX4_01**	16(1239)	0,00163161	Taf5, Syncrip, Pdgfb, Elavl1, Dlg3, Acsl3, Ctbp2, Spry2, Etv5, Xpo1, Kpnb1, Arf6, Pcf11, Gtf3c2, Psip1, Ranbp1
**V$TAL1ALPHAE47_01**	15(1239)	0,00164958	Ltbp1, Pdgfb, Acly, Stag1, Ell2, Sox12, Foxp1, Fgf7, Pde1a, Fmnl3, Nav3, E2f7, Sh3kbp1, Swap70, Arhgap22
**V$AR_Q6**	14(1239)	0,00173645	Syncrip, H2afz, Sin3a, Lif, Actr3, Tial1, Supt16h, Skp2, Tpm3, Pum2, Pcf11, Lrrk1, Srpk1, Bnc2
**V$BACH1_01**	16(1239)	0,00179004	Ankrd28, Trib1, Nek6, Sertad1, Prx, Ube2e3, Rbbp7, Tial1, Luc7l, Tll1, Elk3, Hmga1, Col7a1, Ube2c, Ell, Chst1
**V$CRX_Q4**	16(1239)	0,00179004	Cdkn2c, Lifr, Ltbp1, Chd2, Khsrp, Zfp36l2, Ctbp2, Fgf7, Etv5, Igsf3, Acaa2, Jub, Ets2, Bnc2, Satb2, Usp34
**V$PPARA_02**	10(1239)	0,00187589	Trib1, Pdgfb, Chd2, Prx, Pogz, Supt16h, Smarca5, Kpnb1, Arf6, Pak1
**V$IK1_01**	16(1239)	0,00208719	Cdkn2c, Lifr, Ltbp1, Nek6, Eif4enif1, Foxp1, Stoml2, Top1, Tes, Dlx1, Il13ra1, Polr3b, Rrm1, Brca1, Polr2b, Hmga2
**V$POU3F2_01**	9(1239)	0,00210775	Chd2, Sox4, Phf6, Csnk1g3, Baz1a, Nrp1, Cdh10, Supt16h, Bnc2
**V$FXR_Q3**	9(1239)	0,00210775	Trib1, Egr2, Stoml2, Pde1a, Zic1, Brca1, Kin, Has2, Swap70
**V$FOXM1_01**	15(1239)	0,00237541	Cdkn2c, Syncrip, Ncam1, Khdrbs1, Amd1, Ctcf, Crim1, Stmn1, Cdh10, Ptk7, Dlx1, Ebf2, Cdc25c, Anp32e, Bnc2
**V$BACH2_01**	16(1239)	0,00240334	Trib1, Nup155, Sertad1, Prx, Bach1, Ube2e3, Sms, Usp14, Tial1, Luc7l, Usp3, Col7a1, Tnks2, Pkn3, Ell, Chst1
**V$IK2_01**	16(1239)	0,00252063	Ank3, Ltbp1, Ets1, Tead2, Slc5a3, Supt16h, Stip1, Tes, Dlx1, Ptpn12, Polr3b, Kpnb1, Pcf11, Nrp2, Hmga2, Bcl2
**V$LXR_Q3**	7(1239)	0,00252205	Amd1, Ctcf, Acsl3, Spry2, Has2, Tmpo, Etv4
**V$HNF1_C**	14(1239)	0,00252263	Ncam1, Ptgs2, Yes1, Nrp1, Pou2f1, Fgf7, Supt16h, Egr1, Dlx1, Pla2g4a, Tcf4, Marcks, Epc2, Bub3
**V$ISRE_01**	14(1239)	0,00265046	Cdkn2c, Slc11a2, Unc5c, Etv6, Fmr1, Egr2, Dapp1, Rangap1, Pgk1, Ripk2, Top1, Sdpr, Etv5, Gnb4
**V$CHOP_01**	14(1239)	0,00265046	Gphn, Ankrd28, Syncrip, Trib1, Sox4, Phf6, Stmn1, Socs5, Supt16h, Adss, Etf1, Hmgb2, Bcl2l11, Hmgb1
**V$AP2_Q3**	15(1239)	0,0027162	Sf1, Mycn, Pdgfb, Ctcf, Sox12, Tbx3, Mat2a, Cpsf4, Acsl3, Zic1, Etf1, Rasal2, Gprc5b, Nrf1, Etv4
**V$FOXO3_01**	14(1239)	0,00277184	Kif20a, Brd8, Chd2, Junb, Egr2, Elovl6, Sox12, Impdh2, Etv5, Mcm7, Has2, Mpp6, Bub3, Bcl7a
**V$AHR_01**	7(1239)	0,00278015	Ncam1, Prx, Tgif2, Supt16h, Egr1, Kpnb1, Nrf1
**V$PXR_Q2**	15(1239)	0,00281864	Gphn, Chd2, Cks1b, Egr2, Nrp1, Cdh10, Supt16h, Etv5, Atad2, Itih2, Usp3, Ipo7, Cdk2, Bnc2, Etv4
**V$PAX8_01**	5(1239)	0,00281982	Taf5, Trib1, Supt16h, Pum2, Ahcyl1
**V$CDX2_Q5**	15(1239)	0,00296047	Stag1, Sox4, Mbtps2, Xpo7, Nrp1, Foxp1, Pdap1, Luc7l, Elk3, Dlx1, Nav3, Pcf11, Ube2c, Hmga2, Bnc2
**V$AP2_Q6_01**	14(1239)	0,00304037	Pdgfb, Dlg3, Ctcf, Trim28, Rad21, Stmn1, Cnnm4, Cpsf4, Nr2f2, Spred1, Egr3, Ptk7, Dgcr8, Nrf1
**V$TATA_C**	17(1239)	0,00304763	Ltbp1, Prpf4, Metap1, Chd2, Prkar2b, Bach1, H2afx, Tbx3, Foxp1, Sdpr, Pank1, Nav3, Topbp1, Rab15, Tcf4, Has2, Myct1
**V$NFKB_Q6**	15(1239)	0,00308279	Arpc5, Slc11a2, Fancc, Ptgs2, Elovl6, Tslp, Sin3a, Nr2f2, Tial1, Top1, Stip1, Xpo1, Runx1, Pafah1b3, Bnc2
**V$NKX61_01**	14(1239)	0,00318864	Chd2, Sox4, Rhobtb3, Cdh10, Stoml2, Rbbp8, Fgf7, Sdpr, Dusp6, Tcf4, Ube2c, Marcks, Ets2, Satb2

Transcription Factor	*Number of genes*	*Corrected p-value*	*Genes repressed in Rasless cells (from Table S1)*
**V$HNF4_01**	15(1239)	0,00322212	Asxl1, Ptbp2, Nek6, Nipsnap1, Tomm70a, Dnaja2, Hspe1, Timeless, Slc25a5, Net1, Pabpn1, Fkbp5, Ss18, Smyd5, Hspd1
**V$CEBPDELTA_Q6**	14(1239)	0,00353055	Notch1, Trib1, H2afz, Chd2, Apex2, Egfr, Foxp1, Pdap1, Sdpr, Pla2g4a, Marcks, Gtf3c2, Arid4b, Ddx52
**V$CREB_Q2_01**	13(1239)	0,00360991	Ltbp1, Syncrip, Trib1, Ddx51, Dhx36, Cenpe, Tgif2, Gprc5c, Plk4, Egr1, Cd2ap, Pak1, Spred2
**V$TCF1P_Q6**	14(1239)	0,00369747	Cdkn2c, Trib1, Etv6, Tbx3, Rangap1, Nab2, Uchl5, Cenpf, Gmnn, Plk1, Arf6, Pola2, Nrf1, Tpp2
**V$CACCCBINDINGFACTOR_Q6**	15(1239)	0,00369968	Crlf1, G3bp2, Tial1, Spry2, Slc9a3r1, Etv5, Dlx1, Xpo1, Sdc1, Cdc27, Pcf11, Pabpn1, Smyd5, Six4, Ythdf2
**V$ER_Q6_02**	15(1239)	0,0038306	Elavl1, Chd2, Cldn12, Ssr4, Sox12, Idh3a, Schip1, Stmn1, Hmgb3, Stoml2, Tial1, Elk3, Slc4a7, Plxnb1, Bzw2
**V$ELK1_01**	14(1239)	0,00384021	Taf5, Lifr, Prpf4, Cks1b, Sox4, Nup155, U2af2, Phc2, Egfl7, Hat1, Dbr1, Elk3, Ebna1bp2, Enpp1
**V$ZF5_B**	14(1239)	0,00384021	Cdkn2c, Gphn, Rbm14, Syncrip, Pdgfb, Junb, Mtmr4, Fmr1, Mat2a, Stmn1, H2afy2, Etf1, Marcks, Bcl7a
**V$TITF1_Q3**	14(1239)	0,00384021	Ptbp2, Egfr, Foxp1, Impdh2, Aebp2, Ilf3, Pogz, Pou2f1, Tial1, Ptk7, Tcf4, Dyrk3, Bnc2, Bcl2
**V$ETF_Q6**	8(1239)	0,00405441	Uchl5, Cdc7, Pou2f1, Suv39h2, Rasal2, Rnf2, Lrp8, Ythdf2
**V$TCF11_01**	13(1239)	0,00415092	Chd2, Txnrd1, Nup54, Nrp1, Ube2e3, Pdap1, Pou2f1, Nme1, Supt16h, Ebf2, Ran, Satb2, Fhl2
**V$TAL1BETAITF2_01**	14(1239)	0,00420322	Ltbp1, Pdgfb, Acly, Stag1, Ell2, Sox12, Foxp1, Pde1a, Fmnl3, Nap1l1, E2f7, Sh3kbp1, Swap70, Arhgap22
**V$AP1_01**	15(1239)	0,00437766	Ankrd28, Trib1, Nek6, Sertad1, Bach1, Rbbp7, Spred1, Tial1, Luc7l, Tll1, Dlx1, Ewsr1, Nedd4l, Fosl1, Col7a1
**V$LXR_DR4_Q3**	7(1239)	0,00439432	Etv6, Ctcf, Foxp1, Acsl3, Ptk7, Pum2, Etv4
**V$VDR_Q6**	14(1239)	0,00439457	Syncrip, Rpl3, Pold1, Asxl1, Xpo1, Kpnb1, Ebf2, Slc25a5, Fosl1, Pcf11, Tmpo, Arhgap22, Six4, Trit1
**V$DBP_Q6**	14(1239)	0,0045927	Syncrip, Trib1, Slc29a1, Mns1, Sox4, Map4k5, Slc5a3, Luc7l, Ebf2, Pum2, Dusp6, H2afy, Gpsm2, Chst1
**V$HEN1_01**	12(1239)	0,00492111	Hyal2, Pdgfb, Dusp9, Eno3, Tbx3, Sin3a, Foxp1, Ptk7, Smarca5, Tpm3, Cdk2, Ythdf2
**V$NFKB_C**	15(1239)	0,00496642	Gphn, Arpc5, Ptgs2, Egr2, Slc43a1, Mrps6, Phf6, Eno3, Tslp, Sin3a, Top1, Egr3, Rad18, Dnaja1, Ect2
**V$HFH1_01**	14(1239)	0,00502875	Ankrd28, Ncam1, Taf5l, Egr2, Csnk1g3, Rad21, Nrp1, Impdh2, Chd1, Pik3c2a, Etv5, Cyp26b1, Bub3, Satb2
**V$STAT3_02**	10(1239)	0,0053947	Gphn, Ltbp1, Ncam1, Set, Egr3, Egr1, Kpnb1, Sdc1, Wee1, Bcl7a
**V$CIZ_01**	13(1239)	0,0055422	Cdkn2c, Ckap4, Ncam1, Pdgfb, Chd2, Phf6, Tgif2, Nr2f2, Zic1, Igsf3, Tcf4, Tmpo, Bnc2
**V$E4BP4_01**	16(1239)	0,00567894	Unc5c, Elovl6, Xpo7, Asf1a, Crim1, Cx3cl1, Dnaja2, Pgk1, Pogz, Spred1, Hspe1, Etv5, Mpp6, Wee1, Hspd1, Etv4
**V$CHX10_01**	33(1239)	0,00571779	Cdkn2c, H2afz, Chd2, Etv6, Amd1, U2af2, Yes1, Baz1a, Bach1, Nrp1, Syne2, Rpa2, Cdh10, Socs5, Pogz, Pou2f1, Ccng2, Gprc5c, Pde1a, Cnn3, Zic1, Suclg2, Tll1, Dlx1, Il13ra1, Marcks, Kcnk2, Spag5, Ets2, Slc9a5, Fkbp5, Bnc2, Myo1b
**V$STAT5B_01**	18(1239)	0,00573283	Ank3, Trim25, Asxl1, Nek6, Prkar2b, Nrp1, Lif, Asph, Etv5, Bclaf1, Plscr1, Ppbp, Sdc1, Polr2b, Jub, Mpp6, Dusp4, Ell
**V$ATF6_01**	9(1239)	0,00575333	Syncrip, Stag1, Etv6, Egr2, Top1, Egr3, Egr1, Tnks2, Dusp4
**V$TTF1_Q6**	14(1239)	0,00627325	Ptbp2, Klf5, Lif, Ccng2, Ptk7, Zic1, Dlx1, Ezh2, Myc, Tcf4, Wdr3, Plxnb1, Bnc2, Erh
**V$AREB6_02**	14(1239)	0,00644073	Trib1, Usp1, Stag1, Sox4, Mtmr4, Ctcf, Usp37, Uchl5, Sdpr, Il13ra1, Bzw2, Nrf1, Nrp2, Pabpn1
**V$POU3F2_02**	14(1239)	0,00644073	Asxl1, Mtmr4, Phc2, Dusp5, Foxp1, Pik3c2a, Pou2f1, Top1, Fgf7, Supt16h, Pank4, Dlx1, Cxadr, Kcnk2
**V$MEF2_Q6_01**	14(1239)	0,00644073	Rcor1, Prx, Foxp1, Tnnt2, Lif, Ctbp2, Asph, Bzw2, Gnb4, Slc9a5, Tpp2, Bnc2, Ktn1, Tpbg
**V$GR_Q6**	14(1239)	0,00644073	Lifr, Syncrip, Ncam1, Ppil5, Klf5, Prx, Asf1a, Schip1, Dlx1, Sdc1, Mcm7, Bnc2, Hmgb1, Ruvbl2
**V$S8_01**	14(1239)	0,00644073	Asf1a, Phc2, Nrp1, Pole2, Foxp1, Socs5, Pogz, Gprc5c, Cnn3, Dlx1, Ebf2, Mapk8, Has2, Kcnk2
**V$E47_01**	14(1239)	0,00644073	Neto2, Pdgfb, Sema4b, Amd1, Dusp9, Eif4enif1, Sox12, Rbl1, Foxp1, Rangap1, Sdpr, Plk4, Runx1, Psip1
**V$E2A_Q2**	13(1239)	0,00652749	Notch1, Neto2, Pdgfb, Eif4enif1, Eno3, Ptk7, Hn1, Runx1, Hmga1, Slit2, Gprc5b, Psip1, Bcl7a
**V$HEN1_02**	11(1239)	0,00667136	Hyal2, Pdgfb, Ccne2, Eno3, Tbx3, Ptk7, Etv5, Smarca5, Myc, Tcf4, Cdk2
**V$NKX25_01**	9(1239)	0,00676822	Klf5, Tpd52, Ctcf, Egfr, Luc7l, Dlx1, Tcf4, Bnc2, Bcl2
**V$STAT5A_02**	9(1239)	0,00676822	Syncrip, Chd2, Asxl1, Foxp1, Tial1, Gprc5c, Runx1, Sdc1, Mpp6
**V$OCT_C**	21(1239)	0,00680569	Ank3, Asxl1, Egr2, Phc2, Phf6, Rest, Stmn1, Foxp1, Cdh10, Cnnm4, Pou2f1, Top1, Pank4, Nfyb, Dlx1, Tcf4, Nono, Bzw2, Cdk2, Gprc5b, Bcl2
**V$AP2GAMMA_01**	13(1239)	0,00681753	Pdgfb, Hnrpdl, Ctcf, Phc2, Trim28, Eif2s1, Tead2, Gprc5c, Ptk7, Lrp8, Six4, Etv4, Ythdf2
**V$LHX3_01**	16(1239)	0,00691752	H2afz, Junb, Rhobtb3, Baz1a, Nrp1, Rpa2, Cdh10, Vil1, Suclg2, Ebf2, Has2, Marcks, Kcnk2, Nono, Fkbp5, Bnc2
**V$IRF_Q6**	17(1239)	0,00699799	Cdkn2c, Slc11a2, Asxl1, Etv6, Fmr1, Dapp1, Nrp1, Syne2, Lif, Top1, Etv5, Nav3, Gjc1, Edil3, Psip1, Chst1, Satb2
**V$PIT1_Q6**	13(1239)	0,00701845	Chd2, Sox4, Junb, Sox12, Baz1a, Nrp1, Pou2f1, Fgf7, Etv5, Dusp6, Pcf11, Marcks, Spag5
**V$GCM_Q2**	13(1239)	0,00701845	Taf5, Cdkn2c, Pdgfb, Ell2, Klf5, Cx3cl1, Dusp5, Tnnt2, Pank1, Kntc1, Ets2, Mpp6, Ythdf2
**V$CDP_01**	7(1239)	0,00707804	Chd2, Phc2, Baz1a, Pou2f1, Egr1, Ebf2, Myo1b
**V$PEA3_Q6**	13(1239)	0,00729939	Taf5, Cdkn2c, Ank3, Sf1, Ncam1, Slc43a1, Nrp1, Dusp5, Ctbp2, Tial1, Ctsw, Ipo7, Fen1
**V$OCT1_01**	14(1239)	0,00777689	Ank3, Asxl1, Stag1, Egr2, Phc2, Foxp1, Pou2f1, Nr2f2, Nfyb, Dlx1, Dusp6, Tcf4, Ppp1cc, Cdk2
**V$IPF1_Q4**	13(1239)	0,00799429	Syncrip, Sox4, Egr2, Elovl6, Nrp1, Nr2f2, Zic1, Ebf2, Pgm1, Hmgb2, Nono, Mpp6, Epc2
**V$TFIII_Q6**	12(1239)	0,00895518	Sf1, Mmd, Strap, Nutf2, Sin3a, Nab2, Pou2f1, Nr2f2, Xpo1, Kpnb1, Six4, Spred2
**V$RREB1_01**	12(1239)	0,00938585	Rbl1, Rbm12, Tead2, Supt16h, Egr3, Fmnl3, Dlx1, Runx1, Kpnb1, Pcf11, Pabpn1, Ahcyl1
**V$AP1_Q4_01**	14(1239)	0,00959652	Ankrd28, Map4k5, Prx, Bach1, Ube2e3, Rbbp7, Tial1, Etv5, Tll1, Elk3, Usp3, Fosl1, Col7a1, Chst1
**V$IK3_01**	12(1239)	0,00979904	Lifr, Ank3, Etv6, Tgif2, Egr3, Egr1, Xpo1, Pcf11, Polr2b, Hmga2, Bnc2, Usp34
**V$COREBINDINGFACTOR_Q6**	14(1239)	0,00995685	Ank3, Ltbp1, Phf6, Vrk1, Nutf2, Lif, Luc7l, Supt16h, Runx2, Nav3, Runx1, Etf1, Enpp1, Epc2
**V$ICSBP_Q6**	13(1239)	0,0113735	Cdkn2c, Syncrip, Slc11a2, Rcor1, Fmr1, Dapp1, Phf6, Syne2, Ripk2, Top1, Ly75, Chst1, Satb2
**V$GATA_C**	20(1239)	0,011533	Lifr, Map4k5, Egr2, Fgf7, Supt16h, Slc9a3r1, Zic1, Soat1, Nfrkb, Tpm3, Arf6, Dusp6, Rpl13a, Pcf11, Ccrn4l, Lrig3, Nrp2, Myct1, Fhl2, Litaf
**V$GATA6_01**	13(1239)	0,0118136	Etv6, Ctcf, Phc2, Tgif2, Foxp1, Nr2f2, Tial1, Runx2, Etv5, Pum2, Ccrn4l, Myct1, Ect2
**V$AHRARNT_02**	3(1239)	0,012165	Supt16h, Egr1, Nrf1
**V$TGIF_01**	12(1239)	0,0134229	Taf5, Cdkn2c, Gphn, Sox4, Ctcf, Tgif2, Cdh10, Impdh2, Aebp2, Spred1, Col7a1, Abl2
**V$CEBPGAMMA_Q6**	13(1239)	0,0139253	Slc38a1, Ppil5, Smarce1, Csnk1g3, Eif5, Cdca3, Ctbp2, Ptk7, Dlx1, Nav3, Tmpo, Nrp2, Metap2
**V$CREB_Q4_01**	11(1239)	0,0166421	Ltbp1, Trib1, Ddx51, Dhx36, Egr2, Cenpe, Suv39h2, Plk4, Cd2ap, Pak1, Spred2

Transcription Factor	*Number of genes*	*Corrected p-value*	*Genes repressed in Rasless cells (from Table S1)*
**V$GRE_C**	8(1239)	0,0166514	Cdkn2c, Metap1, Ppil5, Tial1, Luc7l, Egr1, Mcm7, Myct1
**V$NKX25_02**	13(1239)	0,016994	Ank3, Ltbp1, Notch1, Dlg3, Anln, Impdh2, Cacna1c, Pou2f1, Fgf7, Pvrl3, Dlx1, Has2, Hmga2
**V$PPARA_01**	4(1239)	0,0170138	Klf5, Tomm70a, Dnaja2, Ss18
**V$RORA2_01**	9(1239)	0,0170578	Rcor1, Apex2, Taf5l, Rest, Acsl3, Asph, Slc4a7, Marcks, Ivns1abp
**V$OCT1_Q5_01**	13(1239)	0,0182478	Tpd52, Egr2, Phf6, Foxp1, Cdh10, Cnnm4, Nfyb, Dlx1, Arf6, Dusp6, Cdk2, Gprc5b, Bcl2
**V$OLF1_01**	21(1239)	0,0182979	Crlf1, Ell2, Etv6, Dlg3, Ghr, Smarce1, Axl, H2afy2, Pdap1, Egr1, Elk3, Dlx1, Gjc1, Upf3b, Pprc1, Ipo7, Dpf1, Lrp8, Gtf3c2, Dusp4, Etv4
**V$CDPCR3HD_01**	12(1239)	0,0185754	Ppil5, Hnrnpa3, Dlg3, Tbx3, Aebp2, Pde1a, Egr3, Cbx3, Galnt4, Timeless, Dpf1, Gart
**V$IRF7_01**	13(1239)	0,0188085	Taf5, Sox4, Dapp1, Nrp1, Rbl1, Hmgb3, Lif, Dlx1, Gjc1, Arf6, Pkn3, Bnc2, Ythdf2
**V$AR_03**	5(1239)	0,0198069	Syncrip, Khdrbs1, Map4k5, Sdc1, Smyd5
**V$EFC_Q6**	13(1239)	0,0202192	Arpc5, Nek2, Ddx31, Sin3a, Pde1a, Ebna1bp2, Ebf2, Rrm1, Cyp26b1, Ets2, Nono, Nudc, Bnc2
**V$PPARG_01**	4(1239)	0,0213562	Asxl1, Usp37, Dnaja2, Smyd5
**V$AR_01**	8(1239)	0,0216225	Lifr, Ell2, Slc43a1, Stoml2, Luc7l, Etv5, Pank1, Slc9a5
**V$OCT1_03**	11(1239)	0,0229635	Cdkn2c, Lifr, Chd2, Amd1, Nrp1, Elk3, Dlx1, Tcf4, Jub, Bub3, Nrp2
**V$FXR_IR1_Q6**	7(1239)	0,0232135	Ltbp1, Eif5, Ebf2, Brca1, Arf6, Hmgb2, Nono
**V$NRF2_Q4**	12(1239)	0,0232691	Prx, Txnrd1, Bach1, Tomm70a, Rbbp7, Usp14, Tial1, Zic1, Etv5, Tll1, Ran, Pkn3
**V$PR_02**	8(1239)	0,0239158	Syncrip, Pdgfb, Ets1, Khdrbs1, Dlg3, Eif4enif1, Phc2, Sdc1
**V$EVI1_06**	3(1239)	0,0245472	Map4k5, Pcf11, Tcf4
**V$PR_01**	8(1239)	0,0251202	Syncrip, Pdgfb, Ets1, Khdrbs1, Dlg3, Dnaja2, Sdc1, Ppp1cc
**V$CDP_02**	7(1239)	0,025978	Ptgs2, Tbx3, Lif, Tcf4, Marcks, Cdca7, Etv4
**V$AP1_Q2**	13(1239)	0,0264736	Ankrd28, Map4k5, Pdap1, Stoml2, Zic1, Etv5, Elk3, Usp3, Pkn3, Ube2c, Slc9a5, Bnc2, Six4
**V$T3R_Q6**	12(1239)	0,0266394	Ppil5, Junb, Mtmr4, Elovl6, Tbx3, Stoml2, Dlx1, Usp3, Mcm7, Bzw2, Ranbp1, Bnc2
**V$PBX1_02**	8(1239)	0,0275619	Khdrbs1, Mrps6, Slc43a3, Lif, Egr3, Zic1, Ebf2, Pabpn1
**V$SP1_Q2_01**	12(1239)	0,0275944	Hyal2, Acly, Ptpn2, Trim28, Tbx3, Nr2f2, Etv5, Timeless, Pprc1, Jub, Pak1, Six4
**V$HAND1E47_01**	12(1239)	0,029576	Stag1, Klf5, Stmn1, Dusp5, Cdh10, Pou2f1, Nr2f2, Ebf2, Pcf11, Has2, Tmpo, Cdk2
**V$AMEF2_Q6**	12(1239)	0,0306032	Chd2, Eno3, Cdh10, Dnaja2, Ctbp2, Runx2, Sdc1, Cyp26b1, Has2, Bzw2, Nrp2, Bnc2
**V$RSRFC4_01**	12(1239)	0,0315595	Eno3, Foxp1, Lif, Stoml2, Luc7l, Pank1, Cyp26b1, Has2, Bzw2, Slc9a5, Tpp2, Ktn1
**V$AP1_Q6**	12(1239)	0,0315595	Trib1, Nek6, Pdap1, Rbbp7, Tial1, Etv5, Tll1, Elk3, Nfrkb, Pkn3, Ube2c, Slc9a5
**V$RFX1_01**	11(1239)	0,0317343	F2rl1, Cx3cl1, Nab2, Stoml2, Zfp36l2, Hat1, Rfc5, Zcchc8, Npm1, Dusp4, Bnc2
**V$TAXCREB_02**	3(1239)	0,0320884	Crlf1, Sertad1, Nab2
**V$AP1_Q6_01**	12(1239)	0,0324382	Ank3, Map4k5, Pdap1, Rbbp7, Tial1, Vil1, Tll1, Elk3, Nfrkb, Pkn3, Chst1, Csnk1a1
**V$MZF1_02**	11(1239)	0,0326986	Chd2, G3bp2, Asxl1, Foxp1, Supt16h, Egr3, Cbx3, Xpo1, Ewsr1, Pcf11, Gart
**V$TAL1BETAE47_01**	11(1239)	0,0337806	Ltbp1, Acly, Stag1, Ell2, Foxp1, Fgf7, Pde1a, Fmnl3, Sh3kbp1, Swap70, Arhgap22
**V$MYB_Q6**	11(1239)	0,0337806	Ankrd28, Ltbp1, Snrpd1, Klf5, Dlg3, Cdca3, Dlx1, Sdc1, Gtf3c2, Cdk2, Tpp2
**V$NFKB_Q6_01**	13(1239)	0,0347187	Gphn, Mrps6, Eno3, Tslp, Sin3a, Nr2f2, Top1, Tpm3, Cdc27, Sh3kbp1, Dusp6, Plxnb1, Bnc2
**V$OCT1_06**	12(1239)	0,0355802	Ank3, Chd2, Rcor1, Map4k5, Baz1a, Rest, Syne2, Rpa2, Foxp1, Nfyb, Dlx1, Bcl2
**V$MYOD_Q6_01**	11(1239)	0,0361189	Hyal2, Egr2, Stmn1, Ppif, Acaa2, Hn1, Runx1, Hmga1, Psip1, Bcl7a, Fbxo32
**V$EVI1_05**	9(1239)	0,0372665	Chd2, Nek6, Nrp1, Stoml2, Luc7l, Cyp26b1, Sox11, Myct1, Satb2
**V$PAX5_01**	8(1239)	0,0375253	Asxl1, Lig1, Dlx1, Cdc27, Arf6, Cdc40, Prpf4b, Nrf1
**V$SMAD3_Q6**	11(1239)	0,0385609	Ltbp1, Chd2, Mtmr4, Tgif2, Foxp1, Nr2f2, Top1, Fgf7, Jub, Bzw2, Hmgb1
**V$GATA3_01**	11(1239)	0,0398787	Syncrip, Crlf1, Klf5, Rhobtb3, Sox12, Pou2f1, Sh3kbp1, Jub, Bnc2, Satb2, Spred2
**V$NKX62_Q2**	11(1239)	0,0427268	Cdkn2c, Chd2, Ets1, Sdpr, Zic1, Dlx1, Xpo1, Marcks, Bnc2, Myo1b, Etv4
**V$LBP1_Q6**	10(1239)	0,0433828	Cdkn2c, Egr2, Tbx3, Tnnt2, Supt16h, Pcf11, Hmga2, Bcl7a, Ythdf2, Trit1
**V$OCT1_B**	12(1239)	0,0437495	Notch1, Arpc5, Stag1, Egr2, Foxp1, Cnnm4, Nfyb, Dusp6, Cdk2, Gprc5b, Satb2, Bcl2
**V$MYOGENIN_Q6**	11(1239)	0,0437595	Cdkn2c, Rrad, Axl, Eno3, Elk3, Igsf3, Sumo2, Nedd4l, Pcf11, Hmga2, Erh
**V$AML1_01**	11(1239)	0,0437595	Ank3, Egr2, Vrk1, Tnnt2, Pou2f1, Supt16h, Nav3, Nfrkb, Rasal2, Sirt1, Pcf11
**V$AML1_Q6**	11(1239)	0,0437595	Ank3, Egr2, Vrk1, Tnnt2, Pou2f1, Supt16h, Nav3, Nfrkb, Rasal2, Sirt1, Pcf11
**V$CART1_01**	10(1239)	0,0461092	Cdkn2c, Chd2, Ppil5, Sox4, Stoml2, Pogz, Pou2f1, Pvrl3, Pcf11, Bnc2
**V$EVI1_04**	11(1239)	0,0465177	Cdkn2c, Ppil5, Rcor1, Klf5, Eif5, Stoml2, Pogz, Nr2f2, Zic1, Has2, Bub3
**V$AP4_01**	11(1239)	0,0495214	Ltbp1, Hyal2, Ell2, Nutf2, Sin3a, Foxp1, Nab2, Dlx1, Nav3, Sox11, Dpf1
**V$TBP_01**	11(1239)	0,0495214	Rcor1, Sema4b, Egr2, Ctcf, Schip1, Mat2a, Luc7l, Pank1, Cyp26b1, Bnc2, Fhl2
**V$ATF_B**	9(1239)	0,0498357	Ltbp1, Elavl1, Ccna2, Suv39h2, Cd2ap, Rad51c, Gnb4, Pak1, Spred2

Table S2-miRNA. Functional annotation to "miRNA" of the downregulated, differentially expressed genes of Rasless MEFs

miRNA name	*Number of Genes*	*Corrected p-value*	*miRNA targets among genes repressed in Rasless cells (Table S1)*
**mmu-miR-590-3p**	84(1239)	1,36E-21	Etaa1,Ncaph,Gemin4,Timeless,Pcm1,Pop1,Cenpc1,Ercc6l,Whsc1,D19Bwg1357e,Ifi205,Fhl2,Pole2,Setdb2,Cep192,Cenpj,Riok2,Msh2,Tiam2,Lrp8,BC055324,Rrm2,Fbxo5,Luc7l,Klhdc2,Msh3,Usp1,Cacybp,Ccdc99,Pcna,Zmym1,Smek1,Cep152,4930547N16Rik,Casc5,Parp12,Gtf2h2,Depdc1a,Nup107,Traip,Ipo7,Top2a,Snrpd1,Snrpg,Cep70,Nme4,2610301G19Rik,Kin,Tpx2,Rbbp8,Nab2,Ints5,Nup88,Mpp6,Ankrd28,Rbmx2,Mphosph10,Esco2,Mastl,Ruvbl2,Ociad2,Plscr1,Cnot6,Ell2,Nup133,Pum2,Akap8,Fancd2,Rcl1,Cenpk,Ppwd1,Atrx,Pdss1,Thoc2,Utp11l,Nol11,Smu1,Hells,Ppa1,Cebpz,Ltbp1,Cdc73,Ift74,Gtse1
**mmu-miR-340-5p**	76(1239)	1,45E-21	Ncaph,Timm8a1,Tmem39b,H2afv,Prim2,Dhx9,Ktn1,Fhl2,Prpf40a,Fus,Lrig3,Sass6,2610101N10Rik,Ddx27,Cep192,Ruvbl1,Nrf1,Mad2l1,Cep68,Ahctf1,Ttll4,Sltm,Tmem173,Rnps1,Luc7l,Klhdc2,Nudt1,Zfp184,Sh3kbp1,Pdk3,Thsd7a,Cep152,Pcnt,Cul4b,Ccng2,Ptpn2,2210018M11Rik,Depdc1a,Ssb,Palld,Rad54l,Pttg1,Bub1b,Pprc1,Sumo2,Rfc5,Zwilch,Pvrl3,Fzd3,Kif2c,Zfp619,Gphn,Mphosph10,Nde1,Eif4enif1,Actl6a,Cdc40,Tnks2,Ilf3,Mastl,Actr3,Spp1,C79407,D16Ertd472e,Fancd2,Ppwd1,Utp11l,Sf3a3,Whsc2,Hells,Pou2f1,Arf6,Gtse1,Siva1,Kif23,Hnrpdl
**mmu-let-7b***	84(1239)	1,86E-21	Tnfaip8,Tgif2,Prim2,Dhx9,Ktn1,Depdc1b,Nol9,Elf2,Pole2,Plk1,Trim59,Sass6,Pspc1,Cep192,Pnn,Dnmt1,Tpd52,Rbbp7,Aspm,Marcks,Ets2,Nuf2,Tiam2,Ahctf1,Hspd1,Zw10,Acaa2,Nfyb,BC055324,Pim3,Mcm3,Luc7l,Dbr1,Klhdc2,Cand1,Agxt2l2,Aftph,Ash2l,Pik3c2a,Cd2ap,Pde1a,Slc25a5,Ccnb2,Abcb1b,Casc5,Pcnt,2310057M21Rik,Ptpn12,Ewsr1,Nasp,Wipf1,Ssb,Espl1,Pttg1,Ubqln2,Rasa1,Bub1b,Hmgb2,Ctsh,Hat1,Mpp6,Rsf1,Iws1,Ube2t,Sip1,Nudt21,Fgf7,Casp8ap2,Patz1,Kif22,BC016423,Nsmce4a,Suv39h2,Mycn,Yes1,Cep55,Cnot6,Ddx10,Esf1,Apex1,Tmpo,Ift74,Zcchc11,Smc4
**mmu-miR-467e**	84(1239)	2,57E-20	Snx5,Nedd4l,Prim2,Prkar2b,Pwp2,Rnmt,Ktn1,Hspa8,Depdc1b,Plk4,Ogfrl1,Hmmr,Cenpq,Mrps22,1110020G09Rik,Bub1,Cep192,Pnn,Ccdc45,Cdc27,Aspm,Mup4,E2f8,Zc3h15,Yme1l1,Wsb1,Msh6,Mrps6,Dhx36,Acaa2,Cacybp,Ccdc99,Eef1e1,Pik3c2a,Zmym1,Pla2g4a,Trip13,Ccnb2,BC031781,Thsd7a,Eif4a3,Casc5,Crlf1,Casp2,Zfp217,Hnrpll,Pnpt1,Depdc1a,Ewsr1,Acsl3,Prpf38a,Usp6nl,Slco4a1,Myo1b,Wdr75,Nupl1,Uchl5,Bub1b,Itgav,2810055F11Rik,Nme4,Psmd14,Fzd3,Sox4,Cenpf,Ttc3,Pbk,Eif5,Cacna1c,Bnc2,Cep55,Rpl13a,Pdss1,Thoc2,Cnn3,Me2,Zfp281,Gpsm2,Nsl1,Cebpz,Rbm12,Ltbp1,Gtse1,2810417H13Rik
**mmu-miR-208b**	74(1239)	6,36E-20	6430527G18Rik,Timm8a1,Phf6,Marcksl1,Dhx9,Gmnn,Ktn1,Txnrd1,Lrig3,Tes,U90926,1110020G09Rik,Cep192,Elp2,Cdc27,Mad2l1,Tox,Sltm,Mtf2,Tubb5,Fen1,Nfyb,Cenph,Aftph,Ccdc99,Stip1,Emg1,Slc25a5,Ccnb2,Psmc3ip,D1Bwg0212e,Zfp217,Ube2c,Spc25,Shcbp1,Polr2b,Sgms1,Prpf4b,Ptpn12,Ssb,Trim25,Asf1b,Card10,Csnk1a1,Kpna2,Rbm25,F630043A04Rik,Mbd4,Tpx2,Ppbp,Ube2t,3110003A17Rik,2410042D21Rik,Synj2,Mastl,Usp37,Foxp1,Pkmyt1,5730559C18Rik,Plscr1,Ddx10,Fbxo32,Dcbld1,Rcl1,Cenpk,Cnn3,Sf3a3,Me2,Zfp281,Mrpl18,Ecd,Rrs1,Eps8,Pogz
**mmu-miR-381**	73(1239)	2,59E-19	Tnfaip8,Sap30,H2afv,Upf3b,Nol9,Mum1,Nup37,Snapc3,Gemin6,Tes,Elavl1,Kif11,Ube2e3,Xpo7,Egr2,Zc3h15,Vrk3,Ahctf1,Hspd1,Tubb5,Lcorl,Rnps1,Dbr1,Klhdc2,Etf1,Tll1,Hspa14,Usp1,Agxt2l2,Pik3c2a,Cd2ap,Pde1a,Ccnb2,Cep152,Ccnb1,Snrpa1,Pcnt,Tial1,Prpf38a,Nasp,Wipf1,Tube1,Asf1b,Snw1,Smc3,Rasa1,Prpf3,Cep70,Nme4,Arid4b,2610301G19Rik,Kin,Ube2t,Fzd3,3110003A17Rik,Clspn,Tipin,Patz1,Kif22,Actl6a,Lmnb2,Cep55,Fignl1,Ppwd1,Vbp1,Smu1,Apex1,Dyrk3,4632434I11Rik,Egfl7,Rfc4,Gtse1,Mrpl18
**mmu-miR-466d-3p**	96(1239)	2,63E-19	Etaa1,Asph,Rbm26,Cenpc1,Dgcr8,Birc5,Prpf40a,Mum1,Mybbp1a,Zfp52,Pole2,Dsn1,Rangap1,Lrig3,Fubp1,Aurkb,1110020G09Rik,Bub1,Ythdf2,Elp2,Cenpj,Ccdc45,Aurka,Tcerg1,Riok2,Aspm,6720463M24Rik,Sltm,Schip1,Mtf2,Lcorl,Mcm3,Nfatc2ip,Tll1,Hspa14,Asxl3,Lin9,Eme1,Brd8,Gjc1,Ccnb2,Chuk,Abcb1b,Runx2,Mbtps2,Npn2,Mpzl1,Rad51c,Set,Dusp6,Satb2,Gtf2h2,Depdc1a,Ewsr1,Zfp54,Nupl1,Pttg1,Rasa1,Cdc7,Rbm25,Npm1,Ckap2,Itih2,Nme4,2610002M06Rik,Gli3,2610301G19Rik,Tpx2,Ube2t,Casp8ap2,Kif2c,Sox4,Il1rap,2410042D21Rik,Gnl3,Topbp1,Nsmce4a,Bnc2,Cep55,Spp1,Syne2,Slc43a3,Ddx10,Lmnb1,Fmnl3,Lrrc8c,Thoc2,Ddx39,AI848100,1700025G04Rik,Pcf11,Dyrk3,Cenpi,2010204K13Rik,Slc4a7,Kif23
**mmu-let-7c-1***	84(1239)	3,17E-18	Ahsa1,Asph,Prim2,Cenpc1,Ktn1,Nol9,Taf5,Zfp606,Plk1,Trim59,Pank1,Med4,Pspc1,Cep192,Ddx46,Dnmt1,Egr2,Marcks,Ets2,Als2cr12,Ahctf1,Hspd1,Zw10,Slit2,Pim3,Mcm3,Pgk1,Fnbp1l,Dbr1,Cand1,Rpl30,Setx,1700029F09Rik,Slc25a5,Ccnb2,Abcb1b,Psmc3ip,Cep152,B230120H23Rik,Casc5,Scrib,Pcnt,Rmi1,Depdc1a,Ewsr1,Nasp,Wipf1,Cbx3,Ran,Nusap1,Wdr75,Nfkbiz,Ubqln2,Uchl5,Hmgb2,Snrpd1,Neil3,Psmd14,Cenpa,Hat1,Pigf,Ube2t,Nudt21,Fgf7,3110003A17Rik,Limd2,Gphn,Kif22,Ddx31,G3bp1,Nsmce4a,Mcm2,Yes1,Ociad2,Plscr1,Cnot6,Ddx10,Pold1,Dcbld1,Dtymk,Ppa1,Ltbp1,Ift74,Smc4
**mmu-miR-466f-3p**	92(1239)	2,94E-17	Sap30,Rbm26,Diap3,Ranbp1,Gmnn,Depdc1b,Mum1,Plk4,Mybbp1a,Ogfrl1,Ap1s3,Rangap1,Fubp1,Aurkb,Kif11,Ythdf2,Noc4l,Cep192,Cenpj,Aurka,Xpo7,Aspm,Als2cr12,Nol10,Tmem48,Sltm,Mtf2,Api5,Lcorl,Rnps1,BC055324,Mcm3,Nfatc2ip,Cenph,Klhdc2,Tll1,Hspa14,Asxl3,Smarce1,Denr,Usp34,Cbx1,Gjc1,Ccnb2,Chst1,Mbtps2,Npn2,Lig1,Arl6ip6,Dusp6,Shcbp1,Gtf2h2,Casp8,Lrrk1,Ptpn12,Trim25,Asxl1,Pttg1,Cdc7,Bub1b,Nfx1,Itih2,Syt17,Gli3,Paip1,Egr1,Ctsh,Mkx,Cdca3,Il1rap,Topbp1,Synj2,Pabpn1,Suv39h2,Foxp1,Plekha5,Bnc2,Cep55,Slc43a3,Lmnb1,Ell2,Akap8,Rfc2,Mast4,Thoc2,1700025G04Rik,Gmeb1,Pcf11,Tmpo,Cenpi,Cdc73,Mrpl18
**mmu-miR-495**	72(1239)	6,15E-17	Zfp292,Ercc6l,Ankrd32,Rnmt,Dhx9,Steap1,Plk4,Has2,Ppih,Fubp1,2610101N10Rik,Cep192,Ddx46,Elp2,Pnn,Dnmt1,Rbbp7,Nrf1,Cdc5l,Ahctf1,Ythdc1,Mrps6,BC055324,Mcm3,Snrpb,Etf1,Cand1,Pcna,Larp7,Lbr,Supt16h,Prpf38b,Dusp6,Ube2c,Polr2b,Lrrk1,Nup107,Rad54l,Pttg1,Snw1,Cdc7,Ckap2,Snrpd1,2810055F11Rik,Dbf4,Ptk7,Hn1,Unc13c,Rad18,Psmd14,Nab2,Ube2t,Mphosph10,Polr3b,Eif5,Cct8,Ddx10,Rrad,Ell2,6330503K22Rik,Fancd2,Snrpb2,Sf3a3,Tnfaip6,Incenp,Hells,Nup43,E130308A19Rik,Tmpo,Rbpj,Socs4,Pogz
**mmu-miR-132**	80(1239)	6,39E-17	Tnfaip8,Arhgap22,Prim2,Upf3b,Dctd,Cenpo,Setdb2,Pspc1,Pnn,Dnmt1,Pgm1,Pdia6,Pom121,Aspm,Msh2,Tmem176b,Msh6,Mrps6,Sltm,Mtf2,Slit2,Tgif1,Dusp9,Snrpb,Hdac2,Usp10,Myc,Glul,Larp7,Cbx1,Gjc1,Scrib,Kif21a,Baz1a,Pcnt,Hbs1l,Ccng2,Mtap,Ptpn12,Stoml2,Snw1,Nfkbiz,Rasa1,Cdc7,Pprc1,Hn1,Melk,Psmd14,Ptcd3,Pvrl3,Nudt21,Prps1,Socs6,Sox4,Eif4enif1,Topbp1,Esco2,Actl6a,Ddx20,Dapp1,Mcm2,H2afz,Bnc2,Rrad,Ly75,Col7a1,Nob1,Ppwd1,Trub1,Rpl13a,Bcl2l11,Incenp,4632434I11Rik,Cebpz,E130308A19Rik,Rbm12,Ecd,Vash2,Zcchc11,Smc4
**mmu-miR-30b**	79(1239)	6,64E-17	Etaa1,Chd1,2810046L04Rik,Shmt1,6430527G18Rik,Sap30,Diap3,Trim24,Vegfc,Rnmt,Dhx9,Fhl2,Plk4,Wdr82,Zfp52,Cenpq,Prdx4,Ppih,Mapk8,Ddx46,Dnmt1,Aspm,Ank3,Tiam2,Ythdc1,Nol10,Msh6,Utp18,Nfatc2ip,Dbr1,Tll1,Hspa14,Rpl30,Stip1,Chst1,Runx2,Arl6ip6,Scrib,Kntc1,Smc5,Hbs1l,Gtf2h2,Rad54l,Tube1,Slco4a1,Wdr75,Nupl1,Csnk1a1,Rasa1,Cdc7,Bub1b,Cdca4,Arid4b,Zcchc2,Ube2t,Racgap1,Limd2,Casp6,Actl6a,Cct8,Tnrc6a,Cnot6,Rrad,Spc24,Ell2,Nolc1,Stmn1,Gprc5c,Fancd2,Dcbld1,2610027L16Rik,Pdss1,Tex10,4632434I11Rik,Cdca7,Ppa1,Terf1,Mrpl18,Ahcyl1
**mmu-miR-130b**	78(1239)	6,71E-17	Sub1,1110012J17Rik,Rcc2,Tnpo1,Prkar2b,Rbmxrt,Caprin1,Gmnn,Depdc1b,Eef1g,Vps36,Spred2,Snx7,Fosl1,Cenpq,Idh3a,Emb,Ythdf2,Ddx46,Mup4,Parp1,Zc3h15,Tiam2,Kif18a,Suv39h1,Apex2,Hdac2,Aebp2,Ttyh3,Cdca2,Zfp184,Enpp1,Ccdc99,Ncapg2,Pde1a,Gins1,Ncapd2,Chst1,Abcb1b,Cep152,Satb2,2310057M21Rik,Rpl12,Acsl3,Sf3a1,Fbl,Slco4a1,Smndc1,Kpna2,Smc3,Rpa2,Cep70,Nme4,2610301G19Rik,Nab2,Hat1,Chd2,Ptcd3,Dut,Limd2,Gemin8,Tipin,Slco1a5,Mastl,Rbm14,Cct8,Tnrc6a,Ssrp1,B3galnt1,Abl2,D16Ertd472e,Cep170,Pcf11,Tnfaip6,Khdrbs1,C330027C09Rik,Ivns1abp,Slc4a7

miRNA name	Number of Genes	Corrected p-value	miRNA targets among genes repressed in Rasless cells (Table S1)
mmu-miR-20b	78(1239)	7,42E-17	Nedd4l,Diap3,Dffb,Zfp292,Gmnn,Dkc1,Mum1,Tes,Fubp1,2610101N10Rik,1110020G09Rik,Dnmt1,Ube2e3,Rbbp7,Msh2,Egr3,D2Wsu81e,Rest,Tiam2,Ahctf1,Nt5dc2,Prim1,Cdk2,Mcm3,Sfpq,Rrm2,Rad21,Cep57,Lin9,Hmgb3,Pde1a,Abcb1b,Casc5,Rad51c,Scrib,Zfp217,Idh2,Tslp,Ccng2,Gtf2h2,Casp8,Lrrk1,2210018M11Rik,Ewsr1,Metap1,Zfp54,Usp3,Kpna2,Rasa1,Bub1b,Top2a,Rpa2,Wee1,Arid4b,Gli3,Aifm1,Nup88,Mpp6,Iws1,Fzd3,Lrig1,Bbs12,Mcm7,Actl6a,Ptrh2,Tnks2,Mastl,Ddx20,Osmr,Mycn,Cep55,Rfc2,Rsrc2,Thoc2,4632434I11Rik,Cdca7,Ezh2,Cebpz
mmu-miR-208	68(1239)	7,48E-17	Timm8a1,Pop1,H2afv,Marcksl1,Gmnn,Ktn1,Depdc1b,Lrig3,Tes,U90926,Emb,1110020G09Rik,Cep192,Elp2,Cdc27,Tox,Sltm,Mtf2,Tubb5,Cdk2,Fen1,Pgk1,Cenph,Aftph,Ccdc99,Emg1,Slc25a5,Ctnna1,Psmc3ip,D1Bwg0212e,Zfp217,Spc25,Shcbp1,Rbbp4,Polr2b,Sgms1,Ptpn12,Ssb,Trim25,Asf1b,Csnk1a1,Kpna2,Ckap2,F630043A04Rik,Mbd4,Tpx2,Ppbp,Ube2t,Mphosph10,Ss18,Patz1,Wdr73,Synj2,Mastl,Usp37,Foxp1,Pkmyt1,Ddx10,Fbxo32,Dcbld1,Rcl1,Cnn3,Sf3a3,Mrpl18,Ecd,Rrs1,Eps8,Pogz
mmu-miR-7a*	64(1239)	7,58E-17	6430527G18Rik,2700050L05Rik,Zfp292,Fhl2,Steap1,Wdr3,1110020G09Rik,Cep192,Exosc8,Dnmt1,Myef2,Tiam2,Zw10,Dhx36,Tubb5,Cdk2,Snrpb,Sfpq,Msh3,Zic1,Eef1e1,Pla2g4a,Zmynd19,2700029M09Rik,Casc5,Polr2b,Cul4b,Prpf4b,2210018M11Rik,Sf3a1,Trim25,Fbl,Hmgb1,Zfp7,Nupl1,Kpna2,Bub1b,Hspa4l,2810055F11Rik,Syt17,Kin,Mpp6,Trp53bp2,Dut,Xpo1,Nutf2,Nsmce4a,Ilf3,Umps,Cnot6,Rrad,C79407,Dcbld1,Mrpl50,Rsrc2,Cenpk,Psmd5,Thoc2,Sf3a3,Nsl1,Rbm12,Gtse1,2010204K13Rik,Ecd
mmu-let-7a*	74(1239)	1,31E-16	Tgif2,Prim2,Dhx9,Amd1,Ktn1,Nol9,Elf2,Pole2,Plk1,Sass6,Med4,Pspc1,Cep192,Ddx46,Dnmt1,Rbbp7,Aspm,Marcks,Ets2,Nuf2,Ahctf1,Hspd1,Zw10,Utp18,Acaa2,Ect2,BC055324,Pim3,Rad21,Luc7l,Klhdc2,Pik3c2a,Emg1,Pde1a,Slc25a5,Ccnb2,Casc5,Kif21a,Pcnt,Rpl12,Ptpn12,Ewsr1,Nasp,Wipf1,Espl1,Ubqln2,Rasa1,Hmgb2,Nme4,Arid4b,Ctsh,Hat1,Mpp6,Rsf1,Ube2t,Sip1,Casp8ap2,Slc38a1,Patz1,Kif22,Nsmce4a,Mycn,Yes1,Cep55,Cnot6,Ddx10,Pum2,Psmd5,Incenp,Tmpo,Ift74,Top1,Zcchc11,Smc4
mmu-let-7f*	76(1239)	1,35E-16	2810046L04Rik,Tnfaip8,Prim2,Abi1,Dhx9,Ktn1,Nol9,Zfp606,Pole2,Plk1,Ripk2,Sass6,2610101N10Rik,Pspc1,Cep192,Rbbp7,Aspm,Marcks,Nuf2,Ahctf1,Hspd1,Zw10,Acaa2,Ect2,Nfyb,BC055324,Pim3,Luc7l,Dbr1,Klhdc2,Cd2ap,Emg1,Pde1a,Slc25a5,Ccnb2,Abcb1b,Casc5,Kif21a,Pcnt,Ccng2,Ptpn12,Ewsr1,Nasp,Wipf1,Nusap1,Espl1,Ubqln2,Rasa1,Hmgb2,Itgav,Arid4b,Ctsh,Hat1,Mpp6,Ptplad1,Ube2t,Sip1,Nudt21,Fgf7,Casp8ap2,Slc38a1,Kif22,G3bp1,Nsmce4a,Suv39h2,Mycn,Yes1,Cnot6,Nufip1,Ddx10,Esf1,Incenp,Cdc73,Ift74,Zcchc11,Smc4
mmu-miR-466b-3-3p	85(1239)	1,51E-16	Etaa1,Asph,Rbm26,Cenpc1,Birc5,Gmnn,Prpf40a,Mum1,Zfp52,Pole2,Dsn1,Rangap1,Lrig3,Fubp1,Cenpo,Aurkb,1110020G09Rik,Bub1,Ythdf2,Elp2,Cenpj,Ccdc45,Aurka,Tcerg1,Riok2,Aspm,6720463M24Rik,Sltm,Lcorl,Mcm3,Tll1,Asxl3,Lin9,Eme1,Cbx1,Brd8,Ccnb2,Chuk,Abcb1b,Mbtps2,Npn2,Mpzl1,Rad51c,Set,Dusp6,Shcbp1,Satb2,Gtf2h2,Depdc1a,Ewsr1,Zfp54,Nupl1,Pttg1,Rasa1,Cdc7,Rbm25,Npm1,Ckap2,Nfx1,Itih2,2610002M06Rik,Gli3,Tpx2,Ube2t,Casp8ap2,Kif2c,2410042D21Rik,Gnl3,Topbp1,Nsmce4a,Pabpn1,Cdc6,Bnc2,Spp1,Syne2,Slc43a3,Ddx10,Lmnb1,Lrrc8c,Thoc2,Pcf11,Dyrk3,Cenpi,2010204K13Rik,Mrpl18
mmu-miR-363	78(1239)	1,99E-16	Fmr1,Rbm26,H2afv,Birc5,Ankrd32,Dhx9,Fhl2,Ets1,Taf5,Snx7,Has2,Dcp1a,Cenpo,Ncl,Bub3,Aurka,Cdc27,Mup4,Nrf1,E2f8,Ahctf1,Cit,2510012J08Rik,Rhobtb3,Lrp8,Tgif1,Nfatc2ip,Cenph,Asxl3,Ppil1,Ccdc99,Rpl30,Setx,Larp7,Smek1,B230120H23Rik,Prpf38b,Set,Arl4c,Kif21a,Cul4b,Rsbn1,Ccng2,Casp8,Mtap,Rpl12,Acsl3,Hyal2,Lyar,Myo1b,Kpna2,E330009J07Rik,Bub1b,Hspa4l,Mbd4,Soat1,Kif22,Gnl3,BC016423,Gas2l3,Prc1,Cep55,Ssrp1,Syne2,Rrad,Fancd2,Dcbld1,Dtymk,Ppwd1,Thoc2,Ptger4,Cdca8,Incenp,Ppa1,Cdh10,Dock5,Mrpl18,Ecd
mmu-miR-142-5p	67(1239)	2,46E-16	Etaa1,Zfp275,Ercc6l,Skp2,Dhx9,Ktn1,Lrig3,Atad2,Sass6,Mnd1,Dnmt1,Nrf1,Ilf2,Ahctf1,Msh6,Mrps6,Schip1,Hdac2,Usp10,Ccdc99,Ung,Pkn3,Ccnb2,Zc3h8,Imp3,Ptpn2,Vrk1,Lrrk1,Depdc1a,Metap1,Zfp451,Tube1,Rbm25,Bub1b,Pif1,Ckap2,Snrpd1,Paip1,Melk,Mtm1,Hat1,Rsf1,Pvrl3,Pbk,U2af2,Ilf3,Mastl,Usp37,Ncapg,Tpp2,Mycn,Cnot6,Akap8,Pafah1b3,C79407,Gprc5c,Tacc3,Thoc2,AI848100,Twistnb,Zfp281,Khdrbs1,Terf1,Tpbg,Hnrpdl,Zcchc11,Pogz
mmu-miR-292-5p	77(1239)	3,78E-16	Shmt1,Cdca7l,Tmem39b,Arhgap22,Pcm1,Prim2,Upf3b,Depdc1b,Cks1b,Zfp334,Tnnt2,Phactr4,Rangap1,Paxip1,Setdb2,Cep192,Riok2,Ddx52,Myef2,Ank3,Cep68,Tiam2,Lcorl,Hdac2,Tuba1c,Luc7l,Tfdp1,Agxt2l2,BC027072,Ash2l,Rrm1,Glul,Cct2,Ncapg2,Gins1,Cbx1,Prpf38b,Ccrn4l,Set,Gsg2,Imp3,Gtf2h2,Prpf4b,Prpf38a,Csnk1a1,Usp3,Nfkbiz,Smc3,Dimt1,Npm1,4930579G24Rik,Tead2,2810055F11Rik,Mbd4,Snrpg,2810025M15Rik,Rsf1,Iws1,Axl,Pvrl3,Clspn,Zbtb12,Wdr73,Sertad1,Gas2l3,Cacna1c,Mycn,Tnrc6a,Bnc2,Gprc5c,Alkbh1,Ptbp2,1110004E09Rik,Tex10,Ezh2,Wdhd1,Cirh1a
mmu-miR-20a	77(1239)	3,78E-16	Nedd4l,2810046L04Rik,Diap3,Dffb,Zfp292,Gmnn,Pole2,Has2,Lrig3,Atad2,Fubp1,1110020G09Rik,Dnmt1,Ube2e3,Rbbp7,Egr3,Rest,Tiam2,Ahctf1,2510012J08Rik,Slc11a2,Utp18,Prim1,Cdk2,Brca1,Mcm3,Sfpq,Rad21,Armc8,Cenph,Lin9,Ccnb2,Abcb1b,Casc5,Rad51c,Scrib,Idh2,Tslp,Hnrpll,Ccng2,Gtf2h2,Casp8,Lrrk1,2210018M11Rik,Ewsr1,Metap1,Zfp54,Usp3,Kpna2,Klhl23,Bub1b,Top2a,Rpa2,Wee1,Hn1,Arid4b,Gli3,Aifm1,Tpx2,Nup88,Mpp6,Iws1,Fzd3,Bbs12,Mcm7,Actl6a,Tnks2,Mastl,Osmr,Mycn,Ociad2,Cep55,Rsrc2,Thoc2,4632434I11Rik,Ezh2,Cebpz
mmu-miR-323-3p	75(1239)	6,59E-16	Fmr1,6430527G18Rik,Dffb,Sirt1,Marcksl1,Rnmt,Dhx9,Upf3b,Prpf19,Ddx46,Rdx,Ube2e3,Il13ra1,6720463M24Rik,E2f8,Myef2,Parp1,Vrk3,Tiam2,Nudt1,Cand1,Aftph,Enpp1,Cep152,Gpbp1,Rad51c,Scrib,Ncapd3,Zfp217,Cul4b,Vangl2,Gnb4,Gtf2h2,Ptpn2,Ewsr1,Smc3,E330009J07Rik,Mbd4,Snrpg,Cep70,Nt5c3l,Rad18,Psmd14,Net1,Chd2,Igsf3,Ptcd3,Fzd3,Ints7,Nde1,Patz1,Ttc3,Synj2,Cdc40,Ilf3,Foxp1,Eif5,Cacna1c,Tnrc6a,Mboat2,Ell2,Th1l,Bcl2l11,Nol11,Smu1,H2afx,Zfp281,Hells,Ezh2,Tmpo,Fancc,Cdt1,Mrpl18,Rbpj,Hnrpdl
mmu-miR-17	78(1239)	1,08E-15	Nedd4l,2810046L04Rik,Diap3,Zfp292,Mum1,Pole2,Has2,Dsn1,1110020G09Rik,Dnmt1,Pdia6,Rbbp7,Msh2,Egr3,Rest,Tiam2,Ahctf1,Nt5dc2,2510012J08Rik,Slc11a2,Tmem48,Tmem173,Cdk2,Brca1,Mcm3,Sfpq,Rad21,Armc8,Cenph,Cep57,Lin9,Hmgb3,Eme1,Ccnb2,Abcb1b,Cep152,Casc5,Rad51c,Scrib,E130303B06Rik,Zfp217,Idh2,Tslp,Ccng2,Gtf2h2,Casp8,Lrrk1,2210018M11Rik,Ewsr1,Metap1,Zfp54,Usp3,Kpna2,Bub1b,Top2a,Wee1,Nme4,Arid4b,Gli3,Aifm1,Nup88,Mpp6,Iws1,Fzd3,Bbs12,Mcm7,Actl6a,Tnks2,Mastl,Ddx20,Osmr,Mycn,Cep55,Rsrc2,Thoc2,4632434I11Rik,Ezh2,Cebpz
mmu-miR-466a-3p	86(1239)	1,09E-15	Etaa1,Asph,Rbm26,Cenpc1,Birc5,Mum1,Mybbp1a,Zfp52,Pole2,Dsn1,Ccne1,Rangap1,Lrig3,Fubp1,Cenpo,Aurkb,1110020G09Rik,Bub1,Elp2,Cenpj,Ccdc45,Aurka,Aspm,Sltm,Schip1,Lcorl,Mcm3,Tll1,Hspa14,Asxl3,Lin9,Eme1,Brd8,Ccnb2,Chuk,Abcb1b,Mbtps2,Npn2,Mpzl1,Rad51c,Set,Dusp6,Satb2,Gtf2h2,Depdc1a,Ewsr1,Zfp54,Asf1b,Nupl1,Pttg1,Rasa1,Cdc7,Rbm25,Npm1,Ckap2,Itih2,2610002M06Rik,Gli3,Tpx2,Ube2t,Casp8ap2,Kif2c,Sox4,Il1rap,2410042D21Rik,Gnl3,Topbp1,Nsmce4a,Bnc2,Cep55,Spp1,Syne2,Slc43a3,Ddx10,Lmnb1,Lrrc8c,Thoc2,Ddx39,AI848100,1700025G04Rik,Pcf11,Dyrk3,Cenpi,2010204K13Rik,Mrpl18,Kif23
mmu-miR-20a*	62(1239)	1,57E-15	Tgif2,Pcm1,Rbm26,Dnaja2,Birc5,Fhl2,Zfp606,Tnnt2,Ogfrl1,Spag5,Smpdl3b,Ccne1,Fubp1,Strbp,Wdr3,Kif11,Dnmt1,Ptpre,Mad2l1,Ahctf1,Casp3,Snrpb,Zic1,Tll1,Pcna,Sh3kbp1,Larp7,Trip13,1700029F09Rik,2700029M09Rik,2310057M21Rik,H2afy,Hmgb1,E330009J07Rik,Npm1,Neil3,Tk1,Wee1,Snrpg,Rfc5,Mkx,Hat1,Chd2,Ankrd28,Pvrl3,Fgf7,3110003A17Rik,Dut,Slc38a1,Patz1,Ttc3,Tpm3,Nsmce4a,Ilf3,Usp37,Nav3,Stmn1,Fancd2,E330016A19Rik,Gart,Ezh2,Wdhd1
mmu-miR-221	68(1239)	1,64E-15	Shmt1,Pcm1,Dffb,Prim2,Dgcr8,Mcm5,Dhx9,Hspa8,Zfp606,Spag5,Mki67,Zfp760,Las1l,Noc4l,Cep192,Dnmt1,Msh2,Tmem176b,Nrf1,Nfyb,BC055324,Snrpb,Rrm2,Pgk1,Cep57,Exo1,Ccdc99,Eef1e1,Sh3kbp1,Zfp532,Pde1a,Ccnb2,Gpbp1,Zc3h8,Recql4,Rad54l,Ran,Asxl1,Pttg1,Smc3,Cdca4,Ckap2,Tk1,Adsl,Cep70,Trit1,3110003A17Rik,Ghr,Gnl3,Pbk,Nsmce4a,Cdc40,Osmr,Pkmyt1,Pum2,Stmn1,Abl2,Fancd2,Nob1,Arhgap19,Fignl1,2610027L16Rik,Lifr,Cdca8,Tpbg,2810408A11Rik,Socs4,Pogz

miRNA name	*Number of Genes*	*Corrected p-value*	*miRNA targets among genes repressed in Rasless cells (Table S1)*
**mmu-miR-200c**	77(1239)	1,69E-15	Chd1,2810046L04Rik,Diap3,Prkar2b,Abi1,Rnmt,Skp2,Gmnn,Depdc1b,Zfp334,Has2,2810008M24Rik,Ripk2,Fubp1,Cep192,Tcerg1,Riok2,Ndc80,Aspm,Msh2,Xkr5,Ruvbl1,Ahctf1,Ythdc1,Mcm4,Schip1,Mtf2,Tuba1c,Fbxo5,Psip1,Lin9,Ctbp2,Zfp532,Pla2g4a,Usp34,Matr3,U2af1,Usp14,Mapk1,Hn1l,Smc5,Idh2,Parp12,Ccng2,Ewsr1,Acsl3,Ssb,Cbx3,Ran,Usp6nl,Ptges3,Tardbp,Cdc7,Hmgb2,4930579G24Rik,Neil3,Arid4b,Ckap4,Anln,Smc2,Kcnk2,Asf1a,Strap,Pold1,B3galnt1,Gprc5c,Rsrc2,Fignl1,Cep170,Rpl13a,Atrx,Pdss1,Thoc2,Pcf11,E130308A19Rik,Top1,Eif2s1
**mmu-miR-30c**	74(1239)	2,40E-15	Etaa1,Chd1,2810046L04Rik,6430527G18Rik,Sap30,Diap3,Trim24,Vegfc,Rnmt,Dhx9,Fhl2,Wdr82,Cenpq,Ppih,Cpsf6,Dnmt1,Aspm,Msh2,Ank3,Tiam2,Ythdc1,Msh6,Mrps6,Nfyb,BC055324,Dbr1,Hspa14,Cand1,Rpl30,Stip1,Chst1,Arl6ip6,Kntc1,Hbs1l,Gtf2h2,Sbno1,Stag1,Rad54l,Nusap1,Slco4a1,Wdr75,Nupl1,Rasa1,Rbm25,Bub1b,Cdca4,Dbf4,Arid4b,Ctsh,Zcchc2,Ube2t,Limd2,Cenpf,Actl6a,U2af2,Synj2,Tnrc6a,Cnot6,Rrad,Spc24,Lmnb1,Nolc1,Stmn1,Abl2,Gprc5c,Fancd2,Dcbld1,2610027L16Rik,Tex10,Zfp281,Ppa1,Terf1,Mrpl18,Ahcyl1
**mmu-miR-374**	62(1239)	2,52E-15	Nono,Fmr1,Vegfc,Dhx9,Ktn1,Plk4,Nup37,Acly,Ddx46,Tpd52,Mad2l1,Nuf2,Ythdc1,Ttll4,Prim1,Hdac2,Dbr1,Tfdp1,Sgol1,Slc25a5,Kif20a,Psmc3ip,Rad51c,Ccrn4l,Shcbp1,2310057M21Rik,Ccng2,Atad5,Depdc1a,Ptpn12,Wipf1,Nup107,Zfp451,Cobll1,Tardbp,Kpna2,2810055F11Rik,Neil3,Itih2,Dbf4,Paip1,Hat1,Fgf7,Ints7,Trp53bp2,Smc2,Nsmce4a,Suv39h2,Foxp1,Cep55,Esf1,Akap8,Ppwd1,Atrx,Pdss1,Zfp281,Hells,Cdca7,Fancb,Ltbp1,Top1,Rbpj
**mmu-miR-144**	70(1239)	5,97E-15	Nedd4l,Ncaph,Timm8a1,Pwp2,Dhx9,Kif2a,Nup37,Has2,Pank1,Med4,1110020G09Rik,Cep192,Elp2,Dnmt1,Ube2e3,Ddx52,Rbbp7,Marcks,E2f8,Ppat,Dis3,Kif18a,Nol10,Mcm4,Utp18,Sin3a,Hdac2,Cct2,Zfp36l2,Pla2g4a,Gins1,Bzw2,Arl6ip6,Ncapd3,Kif21a,Shcbp1,Idh2,Hnrpll,Polr2b,Cul4b,Ptpn12,Ssb,Hmgb1,Larp4,Smc3,4930579G24Rik,Nr2f2,2810055F11Rik,Paip1,Ctsh,Hat1,Rsf1,Wdr55,Ghr,Gnl3,Nsmce4a,Usp37,Nup155,Plscr1,Cnot6,Ddx10,Nav3,Esf1,Cenpk,Cep170,Rpl13a,Phlda1,Me2,Gtse1,Zcchc11
**mmu-miR-290-5p**	73(1239)	7,77E-15	Hsp90aa1,Cdca7l,Arhgap22,Rcc2,H2afv,Ell,Mcm5,Upf3b,Depdc1b,F2rl1,Bclaf1,Spag5,Atad2,Setdb2,Cep192,Riok2,Ddx52,Pdia6,Il13ra1,Nrf1,Myef2,Ank3,Cep68,Tiam2,Mcm3,Hdac2,Rad21,Luc7l,Tfdp1,Tll1,Agxt2l2,Matr3,2610039C10Rik,Cbx1,Brd8,Ccrn4l,Set,Gsg2,2310057M21Rik,Imp3,Gtf2h2,Prpf4b,Stoml2,Smc3,Dimt1,Npm1,Hspa4l,2810055F11Rik,Rpa2,2810025M15Rik,Rsf1,Axl,Pvrl3,Racgap1,Cenpf,Nde1,Hmgn1,Zbtb12,Ahcy,Sertad1,Ilf3,Cacna1c,Mycn,Tnrc6a,Nav3,Gprc5c,Fancd2,Lrrc8c,Ptbp2,1110004E09Rik,Tex10,Pcf11,Cirh1a
**mmu-miR-291b-3p**	78(1239)	7,94E-15	Nedd4l,Lrrc45,1110012J17Rik,Pcm1,Diap3,Dffb,Mtmr4,D19Bwg1357e,Gmnn,Elf2,Vps36,Snx7,Ctsw,Ppih,Tes,Fubp1,Bub3,Ube2e3,Mup4,6720463M24Rik,Msh2,Tiam2,Ifitm3,Fbxo5,Armc8,Etf1,Myc,Zfp184,Glul,Setx,B230120H23Rik,E130303B06Rik,Pcnt,Shcbp1,Hnrpll,Hbs1l,Casp8,Pola1,2210018M11Rik,Ssb,Zfp451,Ankrd57,Zfp54,2810474O19Rik,Wdr75,Nupl1,Usp3,Ubqln2,Uchl5,Topors,Pprc1,Cep70,Nt5c3l,Mtm1,Net1,Ptcd3,Zfp619,Apitd1,Mcm7,Actl6a,2410016O06Rik,Slco1a5,Mastl,Anp32b,Tnrc6a,Spp1,Syne2,Mcm6,Lrrc8c,Mrpl50,Ppwd1,Cdca8,Smu1,Incenp,Hells,H2afy2,Cks2,Kif23
**mmu-miR-429**	71(1239)	8,14E-15	Nedd4l,2810046L04Rik,Prkar2b,Errfi1,Birc5,Rnmt,D19Bwg1357e,Gmnn,Nsun2,Spred2,Has2,2810008M24Rik,Fubp1,Cenpo,Cep192,Ddx46,Tcerg1,Riok2,Ndc80,Mup4,Ruvbl1,Zc3h15,Ahctf1,Mcm4,Schip1,Zfp770,Slit2,Lcorl,Fbxo5,Klhdc2,Cep57,Lin9,Cdc25a,Hmgb3,Zfp532,Pla2g4a,2610039C10Rik,U2af1,Casp2,Idh2,Suz12,Ccng2,Ssb,Usp6nl,Larp4,Cdc7,Tead2,Pprc1,Top2a,Unc5c,Arid4b,Ckap4,Anln,Smc2,Kcnk2,Asf1a,Mycn,Mboat2,Actr3,Pold1,B3galnt1,Mcm6,Gprc5c,Rcl1,Rsrc2,Fignl1,Rpl13a,Mast4,Snrpb2,Thoc2,E130308A19Rik
**mmu-miR-34b-3p**	69(1239)	9,27E-15	Lrrc45,Prim2,Cenpc1,Prkar2b,Marcksl1,Dhx9,Nol9,Kif2a,Plk4,Mki67,Pank1,Tcerg1,Riok2,Ddx52,Msh2,Zc3h15,Ttll4,Sltm,Taf5l,Tmem173,Mthfd1,Tuba1c,Rad21,Armc8,Usp1,Cand1,Ppil1,Rpl30,Pcna,Pde1a,Psmc3ip,Cep152,U2af1,Casc5,Lig1,Scrib,Snrpa1,Pola1,Prpf4b,H2-K1,Fbl,Traip,Ipo7,Chaf1b,Pif1,Snrpd1,Dbf4,Arid4b,Psmd14,Smchd1,Cdca3,Clspn,Syncrip,Apitd1,Ahcy,Pold3,Anp32b,Fmnl3,B3galnt1,Cenpk,Ppwd1,Ddx39,Khdrbs1,Cdc73,Ahcyl1,Dnajc9,Kif23,Dmap1,Pogz
**mmu-miR-212**	76(1239)	1,05E-14	Tnfaip8,Arhgap22,Prim2,Dnaja2,Upf3b,Elf2,Ccne1,Cenpo,Setdb2,Pspc1,Pnn,Pgm1,Pdia6,Pom121,Aspm,Msh2,Tmem176b,Vrk3,Msh6,Utp18,Sltm,Mtf2,Dusp9,Snrpb,Myc,Glul,Ung,Setx,Larp7,Cbx1,Gjc1,B230120H23Rik,Scrib,Kntc1,Smc5,Kif21a,Baz1a,Pcnt,Rps9,Recql4,Ptpn12,Spry2,Snw1,Nfkbiz,E330009J07Rik,Cdc7,Pprc1,Top2a,Hn1,Pvrl3,Socs6,Sox4,Eif4enif1,Topbp1,Pbk,U2af2,Rbm14,Ddx20,Lmnb2,Dapp1,Mcm2,H2afz,Bnc2,Rrad,Ly75,Col7a1,Nob1,Ppwd1,Rpl13a,Bcl2l11,Twistnb,Incenp,Cebpz,E130308A19Rik,Ecd,Zcchc11
**mmu-miR-148a**	75(1239)	1,18E-14	Chd1,Rcc2,Prkar2b,Pwp2,Fancm,Whsc1,Depdc1b,Snx7,Ctsw,Plk1,Dnmt1,Ube2e3,Egr2,Parp1,Nuf2,Lrp8,Myc,Cand1,Sms,Cdca2,Chuk,Lbr,Psmc3ip,Cep152,Rad51c,Prpf38b,Ncapd3,Zc3h8,Baz1a,Hbs1l,Sgms1,Imp3,Ptpn2,H2-K1,Ppp1cc,2210018M11Rik,Ewsr1,Trim25,Ankrd57,Slco4a1,Nupl1,Usp3,Ccna2,Uchl5,Phf17,Snrpd1,Snrpg,Nme4,Nmral1,Gli3,Ctsh,Psmd14,Plxnb1,Ptcd3,Jub,Dut,Gemin8,Tipin,Lrig1,Tinf2,Tbx3,Gas2l3,Dapp1,Tnrc6a,Ssrp1,Syne2,B3galnt1,Abl2,Litaf,Whsc2,C330027C09Rik,4632434I11Rik,Myct1,Tmpo,Dnajc9
**mmu-miR-148b**	73(1239)	2,01E-14	Chd1,Rcc2,Prkar2b,Pwp2,Ercc6l,Whsc1,D19Bwg1357e,Depdc1b,Snx7,Ctsw,Plk1,Dnmt1,Ube2e3,Egr2,Nrf1,Parp1,Nuf2,Lrp8,Acaa2,Hdac2,Myc,Cand1,Cdca2,Chuk,Lbr,Psmc3ip,Cep152,Rad51c,Ncapd3,Zc3h8,Zfp217,Hbs1l,Sgms1,Imp3,Ptpn2,H2-K1,Ppp1cc,Ewsr1,Trim25,Ankrd57,Slco4a1,Usp3,Kpna2,Ccna2,Uchl5,Phf17,Snrpd1,Snrpg,Nme4,Nmral1,Gli3,Ctsh,Psmd14,Plxnb1,Ptcd3,Axl,Dut,Nde1,Tbx3,Topbp1,Stt3b,Gas2l3,Dapp1,Mycn,Tnrc6a,Syne2,B3galnt1,6330503K22Rik,Abl2,Whsc2,Myct1,Tmpo,Dnajc9
**mmu-miR-30a**	72(1239)	2,41E-14	Etaa1,Chd1,2810046L04Rik,6430527G18Rik,Sap30,Diap3,Prim2,Trim24,Vegfc,Rnmt,Dhx9,Cenpq,Prdx4,Ppih,Atad2,Las1l,Ddx46,Aspm,Msh2,Mad2l1,Tox,Ythdc1,Msh6,Nfyb,Dbr1,Hspa14,Cand1,Rpl30,Pla2g4a,Chst1,Arl6ip6,Ccnf,Kntc1,Smc5,Hbs1l,Imp3,Gtf2h2,Rad54l,Slco4a1,Wdr75,Rasa1,Cdc7,Cdca4,2810055F11Rik,Neil3,Arid4b,Zcchc2,Psmd14,Ube2t,Limd2,Actl6a,Cct8,Tnrc6a,Cnot6,Rrad,Abl2,Ly75,Fancd2,Dcbld1,Mre11a,Cenpk,Trub1,2610027L16Rik,Snrpb2,Tex10,Zfp281,4632434I11Rik,Ppa1,Terf1,Mrpl18,Ahcyl1,Eftud2
**mmu-miR-301b**	70(1239)	3,53E-14	Snx5,Nedd4l,Lrrc45,Diap3,Vps36,Snx7,Has2,Wdr3,Ddx46,5730590G19Rik,Mup4,Tiam2,Gspt1,Kif18a,Sfpq,Hdac2,Myc,Csf1,Cdca2,Zfp184,Enpp1,Ccdc99,Ncapg2,Pde1a,Gins1,Matr3,Ncapd2,Chst1,Ccrn4l,Snrpa1,Hbs1l,Gnb4,Rpl12,Fbl,Slco4a1,Ptges3,Smndc1,Wdr75,Nupl1,Ncam1,Usp3,Nap1l1,Hspa4l,Nme4,Arid4b,Kpnb1,Nab2,Hat1,Chd2,Mpp6,Smchd1,Fgf7,Nde1,Apitd1,Slco1a5,Rbm14,Ddx20,Prc1,Anp32b,Tnrc6a,B3galnt1,Mcm6,Lrrc8c,Fignl1,Atrx,Vbp1,Khdrbs1,Ivns1abp,Kif23,Hnrpdl
**mmu-miR-568**	62(1239)	3,97E-14	H2afv,Dhx9,D19Bwg1357e,Depdc1b,Mum1,Zfp52,Fosl1,Pole2,Plk1,Lrig3,1110020G09Rik,Cep192,Cenpj,Dnmt1,Ube2e3,Ddx52,6720463M24Rik,E2f8,Ahctf1,Hspd1,Wsb1,Dhx36,Snrpb,Cenph,Klhdc2,Stip1,Pla2g4a,Ccnb2,Ccnb1,Scrib,Baz1a,Shcbp1,Cul4b,Hbs1l,2310057M21Rik,Pola1,Pnpt1,Depdc1a,Nupl1,Pttg1,Rbm25,Tead2,Nfx1,Snrpd1,Dbf4,2610002M06Rik,Egr1,Chd2,Mpp6,Ube2t,Cenpe,Eif4enif1,Topbp1,Actl6a,Eif5,Bnc2,Plscr1,Cnot6,Dcbld1,Hirip3,Cenpk,Cenpi
**mmu-miR-93**	71(1239)	4,77E-14	Nedd4l,Pcm1,Diap3,Zfp292,Tnpo1,Gmnn,Vps36,Dsn1,2610101N10Rik,1110020G09Rik,Dnmt1,Rbbp7,Egr3,D2Wsu81e,Rest,Vrk3,Tiam2,Mcm3,Rrm2,Cep57,Lin9,Setx,Pde1a,Gpbp1,2700029M09Rik,Casc5,Arl6ip6,Rad51c,Scrib,Idh2,Ccng2,Gtf2h2,Casp8,Lrrk1,2210018M11Rik,Metap1,Zfp54,Ipo7,Kpna2,Rasa1,Klhl23,Rpa2,Wee1,Hn1,Runx1,Aifm1,Mtm1,Nup88,Mpp6,Iws1,Pvrl3,Fzd3,Socs6,Lrig1,Bbs12,Mcm7,Fbln1,Actl6a,Tnks2,Mastl,Ddx20,Mycn,Tnrc6a,Spp1,Ddx10,Rfpl4,4632434I11Rik,Cdca7,Cebpz,Rbl1,Gtse1

miRNA name	*Number of Genes*	*Corrected p-value*	*miRNA targets among genes repressed in Rasless cells (Table S1)*
**mmu-miR-703**	64(1239)	4,98E-14	Nono,Sap30,Prim2,Tnpo1,Prkar2b,Dgcr8,Ranbp1,Pwp2,Dhx9,D19Bwg1357e,Kif2a,Gemin6,Prpf19,Rangap1,Fubp1,2610101N10Rik,Srpk1,Pnn,Dnmt1,Parp1,Ctcf,Zw10,Mrps6,Slit2,Brca1,Sms,Lin9,Ctbp2,Smc5,Pcnt,Rpl12,Ewsr1,Tial1,Ssb,Traip,2810474O19Rik,Nupl1,Cdc7,Bub1b,Cdca4,2810055F11Rik,Dbf4,Cep70,Trit1,Cenpa,2810025M15Rik,Ube2t,Pvrl3,Cct3,Fgfr1op,Tpm3,Bnc2,Cep55,Plscr1,Ddx39,Ptger4,Phlda1,Incenp,4632434I11Rik,Ivns1abp,Ppa1,Uhrf1,Kif23,Wdr36
**mmu-miR-465a-5p**	67(1239)	5,19E-14	Sap30,Pcm1,Diap3,Zfp292,Prx,Ifi205,Pak1,Kif2a,Plk4,Spag5,Has2,2810008M24Rik,Las1l,Elavl1,Cep192,Ddx46,Cenpj,Ccdc45,Dnmt1,Pgm1,Msh2,Blm,Por,Cct7,Ddx18,Fbxo5,Msh3,Usp1,Exo1,Eef1e1,Dusp4,Pcna,Cdc25a,Trip13,Kif20a,Crlf1,Ube2c,Casp8,Sbno1,Kpna2,Smc3,Hmgb2,Rfc5,Mtm1,Psmd14,Ints5,Pigf,Casp8ap2,Dus3l,Actl6a,Mastl,Strap,Cep55,Cnot6,B3galnt1,Tacc3,Cenpk,Twistnb,Rad51,Gart,Terf1,2010204K13Rik,Dnajc9,Ecd,Eif2s1,Socs4,Smc4
**mmu-miR-302b***	63(1239)	5,27E-14	6430527G18Rik,Prim2,Prkar2b,Rbmxrt,D19Bwg1357e,Gmnn,Zfp52,Ogfrl1,Mki67,Prpf19,Paxip1,Fubp1,Pspc1,Elp2,Ccdc45,Aurka,Pgm1,Ruvbl1,Zc3h15,Polr1e,Dis3,Gspt1,Ppil5,Zw10,Blm,Klhdc2,Cand1,Ctbp2,Larp7,Ncapg2,Ctnna1,Abcb1b,Scrib,Cul4b,Gnb4,Stk17b,Lyar,Palld,2810474O19Rik,Chek1,Zfp53,Ccna2,Klhl23,Ckap2,Pank4,Rbbp8,Pvrl3,Dut,Zbtb12,Pbk,Impa1,Bnc2,Ociad2,Nufip1,Ell2,Trub1,Tnfaip6,Ccdc18,4632434I11Rik,Slc4a7,Hnrpdl,Eif2s1,1810011O10Rik
**mmu-miR-101a**	74(1239)	5,36E-14	Chd1,Ncaph,Cdca7l,Tmem39b,Zfp292,Tnpo1,Prkar2b,Ranbp1,Rbmxrt,Mcm5,Rnmt,Ktn1,Bclaf1,Has2,Noc4l,Ddx46,Pgm1,Rbbp7,6720463M24Rik,Msh2,Hspe1,Ythdc1,Sltm,Junb,Aebp2,Rrm1,Cct2,Cd2ap,Zfp36l2,Gjc1,U2af1,Bzw2,Zfp217,Spc25,Ccng2,Gtf2h2,Ptpn2,Metap1,Chek1,Larp4,Tardbp,Kpna2,Nfx1,Top2a,Prpf3,Rad18,Hat1,Rsf1,Ptcd3,Dut,Abhd10,Ghr,Neto2,Wdr73,Nsmce4a,Tnks2,Foxp1,Prc1,Mycn,Cnot6,Lmnb1,Stmn1,Bhlhb9,Dcbld1,Zcchc8,Rsrc2,Atrx,Etv4,Phlda1,Me2,Rad51,Ezh2,Cebpz,Ecd
**mmu-miR-200b**	73(1239)	5,85E-14	Chd1,2810046L04Rik,Prkar2b,Birc5,Abi1,Skp2,D19Bwg1357e,Gmnn,Nsun2,Spred2,Ripk2,Fubp1,Cenpo,Setdb2,Cep192,Tcerg1,Riok2,Ndc80,Ruvbl1,Nrf1,Tiam2,Ahctf1,Ythdc1,Mcm4,Schip1,Zfp770,Slit2,Tuba1c,Fbxo5,Lin9,Ctbp2,Dusp4,Zfp532,Pla2g4a,Matr3,U2af1,Ccnb1,Idh2,Suz12,Ccng2,Ssb,Tube1,Usp6nl,Asxl1,Cdc7,Pprc1,2810055F11Rik,Neil3,Arid4b,Anln,Smc2,Kcnk2,Ghr,Strap,Mycn,Mboat2,Pold1,B3galnt1,Gprc5c,Rcl1,Arhgap19,Rsrc2,Fignl1,Cep170,Rpl13a,Atrx,Psmd5,Thoc2,Pcf11,Hells,Ezh2,E130308A19Rik,Socs4
**mmu-miR-544**	63(1239)	6,22E-14	Lrrc45,Sap30,Arhgap22,Ccne1,Sass6,Nipsnap1,2010002N04Rik,Mapk8,Strbp,Bub3,Rdx,6720463M24Rik,Tiam2,Pdap1,Prim1,Taf5l,Mthfd1,Lsm2,Pgk1,Sms,BC027072,Ash2l,Exo1,Emg1,Pla2g4a,Ccnb2,Gpbp1,2700029M09Rik,E130303B06Rik,Zc3h8,Snrpa1,Recql4,Gpr126,Cul4b,Gtf2h2,H2afy,Acsl3,Zfp53,Pttg1,Rasa1,Dimt1,Gins4,Mns1,Snrpg,Pigf,Axl,Sfxn1,Prr11,Pvrl3,Dut,Socs6,Clspn,Patz1,Rps13,Foxp1,H2afz,Bnc2,Cnot6,Rrad,Gprc5c,Sf3a3,Apex1,H2afy2
**mmu-miR-181c**	69(1239)	7,62E-14	Tgif2,1110012J17Rik,Prkar2b,Rnmt,D19Bwg1357e,Fhl2,Cks1b,Plk4,Aurkb,Pspc1,Ythdf2,Noc4l,Elp2,Tcerg1,Pdia6,Bcl10,6720463M24Rik,Nrf1,Vrk3,Wsb1,Fxn,Schip1,Pdap1,BC055324,Nfatc2ip,Armc8,Msh3,Ccdc99,Pcna,Pla2g4a,Mbtps2,Gpbp1,Usp14,4930547N16Rik,Ccnf,Snrpa1,Troap,Gtf2h2,H2-K1,Crebzf,Ppp1cc,Rad54l,Cdc7,Hmgb2,Nfx1,Rpa2,Melk,Psmd14,Racgap1,Fgf7,3110003A17Rik,Actl6a,Wdr73,Cdc40,Foxp1,Dapp1,E2f7,Col7a1,1110004E09Rik,Erh,Phlda1,Me2,Incenp,Gpsm2,Ppa1,Cenpi,Nxt1,Rbpj,Smc4
**mmu-miR-33***	51(1239)	8,11E-14	Pcm1,Rcc2,H2afv,Gmnn,Steap1,Snx7,Plk1,Cenpq,Prdx4,1110020G09Rik,Pdia6,Aspm,Msh2,Kif18a,Wsb1,Blm,Cct7,Dbr1,Pcna,Pla2g4a,Thsd7a,Cep152,B230120H23Rik,Sgms1,Lrrk1,Rpl12,Ewsr1,Metap1,Cobll1,Espl1,Asxl1,Pttg1,Pprc1,Top2a,Dbf4,Rbbp8,Zwilch,3110003A17Rik,Dut,Zbtb12,2410016O06Rik,Tnks2,Usp37,Rif1,Nup133,Ly75,Whsc2,Smu1,Ppa1,Tpbg,Slc4a7
**mmu-miR-106a**	71(1239)	8,87E-14	Nedd4l,Diap3,Zfp292,Gmnn,Taf5,Vps36,Mum1,Elk3,Dsn1,2610101N10Rik,Wdr3,1110020G09Rik,Dnmt1,Rbbp7,Egr3,Rest,Ahctf1,Slc11a2,Mcm3,Sfpq,Rrm2,Rad21,Armc8,Cep57,Pde1a,Abcb1b,2700029M09Rik,Casc5,Rad51c,Scrib,Idh2,Tslp,Ccng2,Gtf2h2,Casp8,2210018M11Rik,Ewsr1,Metap1,Zfp54,Ipo7,Usp3,Kpna2,Rasa1,Bub1b,Top2a,Rpa2,Wee1,Aifm1,Nup88,Mpp6,Iws1,Fzd3,Racgap1,Ints7,Lrig1,Bbs12,Mcm7,Actl6a,Tnks2,Mastl,Ddx20,Osmr,Mycn,Cep55,Ddx10,Rsrc2,Stil,Ppwd1,Thoc2,Ezh2,Cebpz
**mmu-miR-297a***	54(1239)	1,18E-13	Etaa1,Sap30,Asph,Rbm26,Cenpc1,Birc5,Mum1,Rangap1,Fubp1,Aurkb,Bub1,Aurka,Aspm,Lcorl,Tll1,Asxl3,Lin9,Usp34,Eme1,Gjc1,Ccnb2,Chuk,Mbtps2,Set,Dusp6,Satb2,Gtf2h2,Depdc1a,Zfp54,Nupl1,Pttg1,Rbm25,Npm1,Ckap2,Itih2,Ube2t,Casp8ap2,3110003A17Rik,2410042D21Rik,Gnl3,Topbp1,Nsmce4a,Bnc2,Cep55,Slc43a3,Ddx10,Lmnb1,Alkbh1,Lrrc8c,Thoc2,AI848100,1700025G04Rik,Pcf11,Cenpi
**mmu-miR-590-5p**	67(1239)	1,37E-13	Etaa1,6430527G18Rik,Asph,Pcm1,2700050L05Rik,Skp2,Elf2,Vps36,Plk1,Ccne1,Pspc1,Bub3,Bcl10,D2Wsu81e,E2f8,Nrp1,Dhx36,Dbr1,Hspa14,Usp1,Myc,Nudt1,Cacybp,Eef1e1,Pcna,Zmym1,Cd2ap,Pde1a,Gins1,Ctnna1,Cep152,B230120H23Rik,Crlf1,Snrpa1,Satb2,Casp8,Pnpt1,Depdc1a,Fanca,Spry2,Larp4,Snw1,Smc3,Itgav,Mbd4,Wee1,Cep70,Mtm1,2810025M15Rik,Ptcd3,Pvrl3,Cct3,Tinf2,Foxp1,H2afz,Ddx10,Mcm6,Zcchc8,Trub1,Atrx,1110004E09Rik,Twistnb,Rad51,Gart,Ccdc18,Tmpo,Pogz
**mmu-miR-875-3p**	63(1239)	1,39E-13	Sap30,1110012J17Rik,Impa2,Skp2,Elf2,Nup37,Paxip1,Kif4,Mnd1,Srpk1,Ddx46,Tcerg1,Pgm1,Riok2,Aspm,Kif18a,Impdh2,Lcorl,Hdac2,Fnbp1l,Dbr1,Hspa14,Cand1,Serbp1,Trim28,Bzw2,Frat2,Ewsr1,Tube1,Cbx3,2810474O19Rik,Smndc1,Card10,Usp3,Hmgb2,Nfx1,Dbf4,Arid4b,Zwilch,Cenpa,Nab2,Ints5,Pvrl3,3110003A17Rik,Rps13,2410016O06Rik,Cdc40,Ddx20,Impa1,Plscr1,Cnot6,Nav3,6330503K22Rik,Cenpk,Cep170,Ppwd1,Pdss1,Phlda1,Hells,Pou2f1,Kif23,Socs4,Cirh1a
**mmu-miR-224**	68(1239)	2,33E-13	Ahsa1,Asph,Prim2,Prkar2b,Impa2,Dhx9,Fhl2,Depdc1b,F2rl1,Ogfrl1,Dctd,Rangap1,Sass6,Ddx27,Rpl7a,Tcerg1,6720463M24Rik,Ank3,Dis3,2510012J08Rik,Epc2,Nfrkb,Nfatc2ip,Pcna,Sh3kbp1,Pkn3,Chuk,Mbtps2,Casc5,Kif21a,Hbs1l,Fbl,Espl1,Zfp53,Usp3,Ier2,Bub1b,Itgav,Nme4,2610002M06Rik,Mtm1,Sip1,Pvrl3,Dut,Cenpe,Zfp619,Cct3,Plekhh2,Dus3l,Ttc3,Apitd1,Polr3b,Tnks2,Ddx20,Eif5,Ruvbl2,Ociad2,Ssrp1,Slc43a1,Syne2,Col7a1,Smu1,C330027C09Rik,Egfl7,H2afy2,Arf6,2010204K13Rik,Eftud2
**mmu-miR-30e**	71(1239)	2,47E-13	Etaa1,Sap30,Diap3,Prim2,Trim24,Vegfc,Dhx9,Cenpq,Prdx4,Ppih,Atad2,Rapgef2,Las1l,Ddx46,Aspm,Mad2l1,Tox,Msh6,Nfyb,BC055324,Dbr1,Hspa14,Cand1,Rpl30,Pla2g4a,Chst1,Arl6ip6,Ccnf,Scrib,Kntc1,Smc5,Gtf2h2,Sbk1,Rad54l,Tube1,Slco4a1,Wdr75,Rasa1,Rbm25,Cdca4,Mmd,2810055F11Rik,Neil3,Dbf4,Unc5c,Arid4b,Zcchc2,Psmd14,Ube2t,Limd2,Cct3,Abhd10,Actl6a,Cct8,Tnrc6a,Cnot6,Rrad,Abl2,Fancd2,Dcbld1,Cenpk,Trub1,Rpl13a,2610027L16Rik,Tex10,Zfp281,4632434I11Rik,Ppa1,Terf1,Mrpl18,Ahcyl1
**mmu-miR-291a-3p**	71(1239)	3,13E-13	Etaa1,Prpf31,Caprin1,4930422G04Rik,Ankrd32,Skp2,Gmnn,Spred2,Bclaf1,Mum1,Ctsw,Cenpq,Kif4,1110020G09Rik,Dnmt1,Msh2,Egr3,Rest,Vrk3,Tox,Ahctf1,Schip1,Nfrkb,Zic1,Rpl30,Larp7,Abcb1b,Cep152,Casp2,Scrib,E130303B06Rik,Zfp217,Idh2,Tslp,Ccng2,Gtf2h2,Lrrk1,Ankrd57,Asf1b,Tyms,Kpna2,Snw1,Colec12,Rpa2,Wee1,Hn1,Runx1,Aifm1,Nup88,Sip1,Casp8ap2,Wdr55,Dut,Bbs12,Gnl3,Asf1a,Synj2,Foxp1,Ddx20,Cep55,Rsrc2,Mast4,Pdss1,Thoc2,Tnfaip6,Ccdc18,4632434I11Rik,Pou2f1,Cebpz,Gtse1,Slc4a7
**mmu-miR-301a**	68(1239)	3,30E-13	Nedd4l,Lrrc45,Diap3,Depdc1b,Vps36,Snx7,Has2,Wdr3,Ddx46,Mup4,Ptpre,Tiam2,Gspt1,Epc2,Sfpq,Hdac2,Myc,Cdca2,Zfp184,Enpp1,Ccdc99,Nek6,Ncapg2,Pde1a,Gins1,Ncapd2,Chst1,Ccrn4l,Snrpa1,Hbs1l,Rpl12,Ewsr1,Fbl,Slco4a1,Ptges3,Smndc1,Wdr75,Nupl1,Usp3,Nap1l1,Hspa4l,Snrpd1,Nme4,Cenpa,Hat1,Chd2,Ptcd3,Smchd1,Fgf7,Dut,Apitd1,Slco1a5,Rbm14,Ddx20,Tnrc6a,Cnot6,Nav3,B3galnt1,Mcm6,Lrrc8c,Fignl1,Ppwd1,Atrx,Vbp1,Khdrbs1,Ccdc18,Cks2,Kif23

miRNA name	*Number of Genes*	*Corrected p-value*	*miRNA targets among genes repressed in Rasless cells (Table S1)*
**mmu-miR-721**	68(1239)	3,47E-13	1110012J17Rik,Pcm1,Diap3,Rcc2,2700050L05Rik,Prkar2b,Mtmr4,Caprin1,Dhx9,Depdc1b,Taf5,Has2,Emb,Ythdf2,Cep192,Ddx46,Mup4,Tiam2,Kif18a,Fen1,Hdac2,Tuba1c,Ttyh3,Armc8,Cenph,Zfp184,Enpp1,Ccdc99,Cd2ap,Ncapg2,Pde1a,Gins1,Matr3,Ncapd2,Dtl,Chst1,Abcb1b,Hbs1l,2310057M21Rik,Sgms1,Imp3,Ssb,Slco4a1,Nap1l1,Ubqln2,Rpa2,Nme4,Hat1,Chd2,Ptcd3,Jub,Dut,Socs6,Limd2,Actl6a,Slco1a5,Rbm14,Cct8,Tnrc6a,Ssrp1,B3galnt1,Lrrc8c,Cep170,Pcf11,Tnfaip6,Khdrbs1,Cks2,Dnajc9
**mmu-miR-598**	64(1239)	3,70E-13	Shmt1,Zfp292,Prim2,Birc5,Cks1b,F2rl1,Snx7,Mybbp1a,Has2,Ripk2,Nipsnap1,Rapgef2,2010002N04Rik,Pdgfb,1110020G09Rik,Ddx46,Nrf1,Ank3,Impdh2,Taf5l,Gins2,Asxl3,Agxt2l2,Sms,Ppil1,Pla2g4a,Usp34,Slc25a5,Cep152,Casc5,Set,Ncapd3,Zfp217,Recql4,Cul4b,Rad54l,Slbp,Ncam1,Tead2,Gli2,Neil3,Ptk7,Snrpg,Trit1,Pkp2,Psmd14,Chd2,Pigf,Cdca3,Pvrl3,Fgf7,Ints7,Sox4,Kif22,Nutf2,Wdr73,Sertad1,Bnc2,Nav3,Pum2,Thoc2,1700054N08Rik,H2afy2,Dmap1
**mmu-miR-465c-5p**	65(1239)	5,92E-13	Sap30,Pcm1,Zfp292,Ifi205,Spred2,Pak1,Kif2a,Plk4,Spag5,Has2,2810008M24Rik,Las1l,Cep192,Ddx46,Cenpj,Ccdc45,Dnmt1,Pgm1,Riok2,Msh2,Cdc5l,Dis3,Blm,Por,Ddx18,Msh3,Usp1,Agxt2l2,Eef1e1,Pcna,Cd2ap,Kif20a,Crlf1,Casp8,Pola1,Prpf4b,Fanca,Cobll1,Smc3,Gins4,Hmgb2,2810055F11Rik,Psmd14,Ints5,Pigf,Casp8ap2,Prkg2,Actl6a,Mastl,Cep55,Cnot6,Tacc3,Pola2,Rad51,Gart,Egfl7,Ppa1,Nsl1,Terf1,2010204K13Rik,Dnajc9,Ecd,Eif2s1,Socs4,Smc4
**mmu-miR-338-5p**	64(1239)	6,69E-13	Pop1,Prkar2b,Abi1,Dhx9,Ktn1,Cks1b,Prdx4,Ccne1,2610101N10Rik,Strbp,Ddx46,Rpl7a,Dnmt1,Riok2,Ets2,D2Wsu81e,Nuf2,Kif18a,Casp3,Mtf2,Blm,Cacybp,Cep57,Lin9,Pcna,Cd2ap,Hmgb3,Gins1,Slc25a5,Chst1,Abcb1b,Dhodh,Cul4b,Hbs1l,Ptpn2,Ptpn12,Nxf1,Cobll1,Spry2,Pttg1,Tardbp,Pif1,Gli2,Pank4,Magoh,Fgf7,Fgfr1op,Esco2,Nsmce4a,Mycn,Yes1,Ssrp1,Ddx10,Mcm6,Rfpl4,Col7a1,Zcchc8,Fignl1,Cenpk,Atrx,Psmd5,Pou2f1,Ift74,Rrs1
**mmu-miR-322***	57(1239)	6,72E-13	Nedd4l,Khsrp,Ktn1,Fosl1,Spag5,Smpdl3b,Aurkb,1110020G09Rik,Ccdc45,Tiam2,Cldn12,Usp34,Eme1,Cep152,Mbtps2,Gsg2,Cul4b,Wipf1,Dhx15,Traip,Gins4,Hspa4l,Unc5c,Chd2,Ptcd3,Sip1,Cenpf,Mphosph10,Patz1,Gnl3,Topbp1,Stt3b,G3bp1,Ddx20,Tpp2,Strap,Mycn,Tnrc6a,Yes1,Syne2,Nav3,Spc24,Esf1,Pold1,Mcm6,C79407,Hirip3,Ppwd1,Vbp1,AI848100,Smu1,Zfp281,4632434I11Rik,Ppa1,Rrs1,Wdr36,Hnrpdl
**mmu-miR-465b-5p**	64(1239)	6,94E-13	Sap30,Pcm1,Diap3,Zfp292,Rbmxrt,Ifi205,Kif2a,Plk4,2810008M24Rik,Ripk2,Cep192,Ddx46,Elp2,Cenpj,Ccdc45,Pgm1,Riok2,Msh2,Cdc5l,Dis3,Blm,Por,Ddx18,Msh3,Usp1,Agxt2l2,Exo1,Eef1e1,Pcna,Kif20a,Crlf1,Casp8,Pola1,Prpf4b,Fanca,Nup107,Smc3,Gins4,Hmgb2,2810055F11Rik,Psmd14,Ints5,Pigf,Cdca3,Casp8ap2,Actl6a,Mastl,Hltf,Cep55,Tacc3,Cenpk,Pola2,Twistnb,Gart,Ppa1,Nsl1,Terf1,2010204K13Rik,Dnajc9,Ecd,Kif23,Eif2s1,Socs4,Smc4
**mmu-miR-29b**	72(1239)	7,65E-13	2810046L04Rik,Ranbp1,Prpf40a,Nol9,Elf2,Mki67,Plk1,Prdx4,Ccne1,Rapgef2,Las1l,Aurkb,Mapk8,Pdgfb,6720463M24Rik,Cep68,Nol10,Ttll4,Tmem48,Blm,Tubb5,Tmem173,Cdk2,Sfpq,Bach1,Cenph,Sms,Smarce1,Ung,Usp34,1700029F09Rik,Cbx1,Abcb1b,Eif4a3,Lig1,Scrib,Idh2,Otud4,Hbs1l,2310057M21Rik,Nasp,Nxf1,Zfp54,Espl1,E330009J07Rik,Ccna2,Cdc7,Cdca4,Dbf4,Prpf3,Kin,Psmd14,Hat1,Cdca3,Sertad1,Impa1,Pkmyt1,Mycn,Nav3,Lmnb1,Pafah1b3,Dcbld1,Col7a1,Zcchc8,Rsrc2,Bcl2l11,Etv4,H2afx,Rad51,Fancc,Cdh10,Smarcc1
**mmu-let-7i***	65(1239)	7,68E-13	Snx5,Ahsa1,Tmem39b,Dffb,Prx,Prpf40a,Cks1b,Spred2,Zfp606,Crim1,Etv6,Mki67,Ripk2,2010002N04Rik,Ythdf2,Dnmt1,Bcl10,Rbbp7,Aspm,D2Wsu81e,Tox,Fxn,Sin3a,Fen1,Mcm3,Armc8,Tfdp1,Usp1,Rpl3,Ppil1,Setx,Zmynd19,1700029F09Rik,Cep152,B230120H23Rik,Npn2,Set,Ncapd3,Parp12,Ewsr1,Wipf1,Pttg1,Ier2,Rasa1,Ccna2,Hmgb2,Itgav,Tk1,Psmd14,Mpp6,Fgf7,3110003A17Rik,Polr3b,Suv39h2,Cnot6,Rrad,Specc1,Zcchc8,Nob1,Dtymk,Ppa1,Wdhd1,E130308A19Rik,Tmpo,Ltbp1
**mmu-miR-101b**	71(1239)	9,35E-13	Ncaph,Cdca7l,Tmem39b,Zfp292,Tnpo1,Prkar2b,Ranbp1,Rbmxrt,Rnmt,Ktn1,Cks1b,Bclaf1,Has2,Noc4l,Ddx46,Pgm1,Rbbp7,6720463M24Rik,Msh2,Hspe1,Sltm,Junb,Mcm3,Aebp2,Rrm1,Cct2,Zfp36l2,U2af1,Bzw2,Zfp217,Spc25,Baz1a,Ccng2,Gtf2h2,Ptpn2,Pola1,Metap1,Nasp,Larp4,Mns1,Nfx1,Top2a,Prpf3,Rad18,Ctsh,Hat1,Ptcd3,Pvrl3,Dut,Abhd10,Fgfr1op,Neto2,Wdr73,Nsmce4a,Tnks2,Prc1,Mycn,Yes1,Lmnb1,Stmn1,Bhlhb9,Dcbld1,Rsrc2,Atrx,Cnn3,Etv4,Phlda1,Me2,Rad51,Ezh2,Cebpz
**mmu-miR-293***	52(1239)	1,38E-12	Cdca7l,Pcm1,Depdc1b,Cks1b,Bclaf1,Atad2,Setdb2,Med4,Cep192,Ddx52,Tiam2,Mtf2,Hdac2,Luc7l,Armc8,Tfdp1,Agxt2l2,Matr3,Cbx1,Runx2,Gpbp1,Bzw2,Set,Rbbp4,Imp3,Gtf2h2,Prpf4b,Pnpt1,Csnk1g3,Csnk1a1,Smc3,Npm1,Hspa4l,2810055F11Rik,2810025M15Rik,Iws1,Pvrl3,Sox4,Zbtb12,Wdr73,Sertad1,Gas2l3,Cacna1c,Mycn,Tnrc6a,Spc24,Gprc5c,Lrrc8c,1110004E09Rik,Zfp281,Ezh2,Cirh1a
**mmu-miR-802**	60(1239)	1,44E-12	Asph,Tmem39b,Abi1,Ankrd32,Ktn1,Steap1,Snapc3,Acly,Idh3a,2610101N10Rik,Ythdf2,Rbbp7,Mad2l1,Tox,Dis3,Nrp1,Hspd1,Ccne2,Msh6,Mrps6,Tgif1,Lcorl,Cct7,Cct5,Cbfb,Cep57,Ctbp2,Matr3,2610039C10Rik,Usp14,Spc25,Pcnt,2310057M21Rik,Wipf1,Cobll1,Fbl,Ran,Nupl1,Usp3,Kpna2,Smc3,Cdca4,Paip1,Mkx,Dut,Topbp1,Pbk,Actl6a,Nsmce4a,Rif1,Cct8,Yes1,Ddx10,Hirip3,Vbp1,Cdca8,Khdrbs1,Hells,Nup43,Arf6
**mmu-miR-302a***	58(1239)	1,66E-12	Prim2,Tnpo1,Prkar2b,Vegfc,Gmnn,Snx7,Mki67,Cenpq,Atad2,Rpl7a,Ddx52,Tiam2,Mtf2,Hdac2,Armc8,Cbfb,Agxt2l2,Aftph,Sgol1,Larp7,Matr3,Abcb1b,Mpzl1,Scrib,2310057M21Rik,Imp3,Depdc1a,Prpf38a,Stk17b,Lyar,Smndc1,Nfkbiz,Tead2,Top2a,2810055F11Rik,Itih2,Paip1,Pkp2,2810025M15Rik,Anln,Syncrip,Abhd10,Zbtb12,Asf1a,Ints2,Mycn,Plscr1,Nav3,Fmnl3,Akap8,Specc1,Nob1,Mre11a,Thoc2,Cnn3,Pebp1,Eftud2,Slc4a7
**mmu-miR-494**	56(1239)	1,67E-12	Chd1,6430527G18Rik,Sap30,Ahsa1,Asph,Gemin4,Rcc2,H2afv,Ercc6l,Ankrd32,Dhx9,Ktn1,Cks1b,Rfwd3,Mum1,Ogfrl1,Spag5,Plk1,Dck,Sass6,Cenpo,Rapgef2,Las1l,Aurkb,Bub1,Ythdf2,Ythdc1,Tgif1,Dbr1,Hspa14,Lin9,Pcna,Ccnb2,Set,Ptpn12,Sf1,Tube1,Nusap1,Espl1,Usp3,Nfkbiz,Hmgb2,Arid4b,Rbbp8,Ube2t,Jub,Mphosph10,Fbln1,Eif5,Plscr1,Rrad,Col7a1,Zfp281,Khdrbs1,Siva1,Ecd
**mmu-miR-471**	60(1239)	1,71E-12	Etaa1,Tmem39b,Pop1,Dhx9,Gmnn,Pak1,Kif2a,Bclaf1,Atad2,Strbp,1110020G09Rik,Noc4l,Elp2,6720463M24Rik,Msh2,Vil1,Nrf1,Hspd1,Tmem48,Schip1,Cct5,Hspa14,Rcc1,Egfr,Pla2g4a,Abcb1b,Cep152,4930547N16Rik,Casc5,Ube2c,Baz1a,Pcnt,Pnpt1,2810474O19Rik,Kpna2,E330009J07Rik,Npm1,1110034A24Rik,Top2a,2810055F11Rik,Neil3,Cep70,Slc9a3r1,Paip1,Kin,Ppbp,Ckap4,Mpp6,Pvrl3,Anln,Pbk,Wdr73,Foxp1,Ncapg,Ddx10,Nob1,Lifr,Sf3a3,2010204K13Rik,Zcchc11
**mmu-miR-30d**	66(1239)	1,82E-12	Etaa1,Nedd4l,2810046L04Rik,Tnfaip8,Sap30,Diap3,Prim2,Trim24,Dhx9,Steap1,Wdr82,Cenpq,Ppih,Las1l,Ddx46,6720463M24Rik,Msh2,Mad2l1,Tox,Ythdc1,Epc2,Msh6,Rnf138,BC055324,Dbr1,Hspa14,Rpl30,Pla2g4a,Chst1,Ccnf,Scrib,Kntc1,Smc5,Hbs1l,Gtf2h2,Rad54l,Slco4a1,Wdr75,Ckap2l,Cdca4,Neil3,Dbf4,Zcchc2,Psmd14,Ube2t,Limd2,Rfc3,Actl6a,Cct8,Tnrc6a,Cnot6,Rrad,Spc24,Abl2,Fancd2,Dcbld1,Cenpk,Trub1,2610027L16Rik,Tex10,Zfp281,4632434I11Rik,Ppa1,Terf1,Mrpl18,Ahcyl1
**mmu-miR-743a**	64(1239)	2,01E-12	2810046L04Rik,Cdca7l,Zfp292,Impa2,Birc5,Prx,Dhx9,Ifi205,Ktn1,Fhl2,Eef1g,Nup37,Txnrd1,Atad2,U90926,Wdr3,Elp2,Pnn,Dnmt1,Nfrkb,Cct7,Sfpq,Ddx18,Cand1,Asxl3,Cep57,Emg1,BC031781,Cep152,Dhodh,Ncapd3,Ube2c,Pcnt,Pola1,Ppp1cc,Prpf38a,Ssb,Tube1,Ckap2,Top2a,Hn1,Rfc5,Cep70,2610002M06Rik,Nab2,Cenpn,Casp8ap2,Plekhh2,Gphn,Tpm3,Synj2,Nsmce4a,Ilf3,Plscr1,Fancd2,Rfc2,Ppwd1,Utp11l,Incenp,Ccdc18,Pou2f1,H2afy2,2010204K13Rik,Kif23
**mmu-miR-199a-3p**	64(1239)	2,12E-12	2810046L04Rik,Rbm26,Zfp292,Nup54,Dhx9,Ktn1,Prpf40a,Depdc1b,Steap1,Kif2a,Zfp52,Tnnt2,2810008M24Rik,Sass6,Rnf2,Wdr3,Ppat,Ahctf1,Hspd1,Mrps6,Fen1,Aebp2,Cct5,Nudt1,Agxt2l2,Egfr,Larp7,Matr3,Ncapd2,Pdk3,Dusp5,Ankrd10,Casc5,Snrpa1,Recql4,Hbs1l,Sbno1,Pnpt1,Mcm10,Fbl,Rbm25,Bub1b,Gtpbp10,Snrpd1,Prpf3,Unc13c,Arid4b,Kin,Net1,2810025M15Rik,Ptcd3,Pigf,Pvrl3,Trp53bp2,Patz1,Neto2,Actl6a,Trpc2,Bhlhb9,Hirip3,Pola2,Ezh2,Cdt1,Mrpl18

miRNA name	*Number of Genes*	*Corrected p-value*	*miRNA targets among genes repressed in Rasless cells (Table S1)*
**mmu-miR-376c**	60(1239)	2,43E-12	Nedd4l,Ncaph,Diap3,Prkar2b,Gmnn,Ktn1,Fhl2,Vps36,Mum1,Acly,Ppih,Noc4l,Ddx46,Cenpj,Riok2,Nuf2,Tox,Prim1,Snrpb,Pgk1,Luc7l,Usp34,Trip13,Cep152,Bzw2,Sgms1,Tial1,Ssb,Zfp451,Slco4a1,Kpna2,Rbm25,Pprc1,Wee1,Cep70,Nme4,Runx1,Aifm1,Ctsh,Rbbp8,Zcchc2,Ppbp,Hat1,Pigf,Sip1,Fzd3,Anln,Limd2,Dus3l,Ptrh2,Rrad,Lmnb1,Rfc2,Nob1,2610027L16Rik,Pcf11,Terf1,Cks2,Kif23,Dlx1
**mmu-miR-295**	60(1239)	2,43E-12	Ankrd32,Skp2,Gmnn,Ktn1,Mum1,Plk4,Ctsw,Cenpq,Dnmt1,Pdia6,Rbbp7,Egr3,Rest,Vrk3,Tiam2,Schip1,Lcorl,Rnps1,Ppid,Dbr1,Lin9,Rpl30,Cct2,Setx,Larp7,Scrib,E130303B06Rik,Idh2,Ccng2,Gtf2h2,Lrrk1,Asf1b,Ipo7,Zfp53,Kpna2,Snw1,Colec12,Neil3,Wee1,Hn1,2610301G19Rik,Aifm1,Mtm1,Nup88,Pvrl3,Dut,Synj2,Cep55,Nufip1,6330503K22Rik,Rfpl4,E330016A19Rik,Dtymk,Rsrc2,Pdss1,Thoc2,Hells,Ccdc18,4632434I11Rik,Fancc
**mmu-miR-694**	51(1239)	2,47E-12	Nedd4l,Rbm26,Gmnn,Nup37,Sass6,Cep192,Pnn,Pgm1,Bcl10,Aspm,Kif18a,Utp18,Ppid,Fnbp1l,Lin9,Trim28,Bzw2,Gtf2h2,Ewsr1,Hmgb1,Espl1,Usp3,Prpf3,Snrpg,Nt5c3l,Aifm1,Net1,Zwilch,Mpp6,Pvrl3,Fzd3,Lrig1,Patz1,Rps13,Tnks2,Ddx20,Tpp2,Cnot6,Zcchc8,Cenpk,Ppwd1,Pdss1,Gmeb1,Pcf11,Me2,Dyrk3,4632434I11Rik,Kif23,Hnrpdl,Socs4,Smc4
**mmu-miR-466g**	77(1239)	2,56E-12	Ncaph,Pcm1,H2afv,Ell,Mcm5,Birc5,Mum1,Plk4,Mybbp1a,Ap1s3,Cenpq,Rangap1,Paxip1,Cenpo,2610101N10Rik,Las1l,Aurkb,Ythdf2,Noc4l,Ddx46,Dnmt1,Aspm,Msh2,Tox,Ahctf1,Gspt1,Prim1,Mthfd1,Rnps1,BC055324,Hdac2,Pgk1,Cand1,Cacybp,Dusp4,Cd2ap,Zmynd19,Ncapd2,Ccnb2,Micall2,Abcb1b,Npn2,Set,Polr2b,Gtf2h2,H2afy,Ewsr1,Nasp,Stk17b,Usp3,Rpa2,Dbf4,Sumo2,Pkp2,Ube2t,Sip1,Lrig1,Actl6a,2410016O06Rik,Nup155,Cep55,Ssrp1,Rrad,Esf1,Mcm6,Abl2,D16Ertd472e,Cenpk,Nek2,Incenp,Zfp281,Cenpi,Kif23,Zcchc11,Tdrkh,Smc4,Cirh1a
**mmu-miR-19b**	64(1239)	2,76E-12	Ahsa1,Cenpc1,Birc5,Dhx9,Steap1,Mum1,Fosl1,Crim1,Prpf19,Pank1,1110020G09Rik,Cep192,Dnmt1,Nrf1,E2f8,Sltm,Tgif1,Apex2,Rnps1,Armc8,Gpd2,Aftph,Lin9,Enpp1,Ccdc99,Cbx1,Ccnb2,Chst1,Psmc3ip,Bzw2,Scrib,Zc3h8,Kif21a,Ppp1cc,Prpf38a,Nasp,Sf3a1,Smndc1,Itgav,Nt5c3l,Melk,Psmd14,Cenpa,Igsf3,Igf2bp1,Limd2,Gemin8,Lrig1,Abhd10,Actl6a,Slc39a10,Tnks2,Pold3,Lrrc40,Cnot6,Spc24,Ell2,Cenpk,Whsc2,Ivns1abp,Ppa1,Gtse1,Cks2,1810011O10Rik
**mmu-miR-191**	60(1239)	3,05E-12	Etaa1,Rbm26,Prkar2b,Dhx9,Ifi205,Depdc1b,Mum1,Las1l,Elavl1,Pspc1,Ddx46,Mad2l1,Cdc5l,Als2cr12,Nuf2,Slc11a2,Sltm,Tubb5,Lsm2,Tuba1c,Hmgb3,Slc19a1,Zfp532,Supt16h,Ankrd10,Bzw2,Ube2c,Kif21a,Gtf2h2,Sbk1,Mtap,Rpl12,Tial1,Wipf1,Palld,Nfkbiz,Snrpd1,Psmd14,Nup88,Dut,Patz1,Ilf3,Foxp1,Plscr1,Mcm6,Dcbld1,Ppwd1,Snrpb2,Lifr,Cdca8,Incenp,Zfp281,Myct1,Pou2f1,E130308A19Rik,Terf1,Gtse1,Mrpl18,Rbpj,Hnrpdl
**mmu-miR-291a-5p**	67(1239)	3,33E-12	Ncaph,Cdca7l,Sap30,Gemin4,Tmem39b,Pcm1,Cks1b,Glrx,Snx7,Fosl1,Plk1,Cenpq,Mrps22,Bub1,Cep192,Pnn,Rdx,Rbbp7,Ruvbl1,Nrf1,Nuf2,Nol10,Zw10,Sltm,Blm,Sin3a,Brca1,Ash2l,Rrm1,Sgol1,Rcc1,Hmga1,Trim28,Thsd7a,Casc5,Sbno1,Stag1,Rpl12,Prpf38a,Asf1b,Myo1b,Espl1,Nupl1,Ckap2l,E330009J07Rik,Pprc1,Nfx1,Rbbp8,Psmd14,Cdca3,Tinf2,Dus3l,Zrsr2,Tpm3,Nsmce4a,Foxp1,Osmr,Tnrc6a,Mcm2,Actr3,Slc43a3,Ddx10,Incenp,Cdc73,Gtse1,Tpbg,Slc4a7
**mmu-miR-29c**	71(1239)	4,02E-12	2810046L04Rik,Tnfaip8,Ranbp1,Pwp2,Prpf40a,Elf2,Mki67,Ccne1,Rapgef2,Las1l,Aurkb,Mapk8,Pdgfb,Noc4l,6720463M24Rik,Cep68,Nol10,Tmem48,Blm,Rnf138,Tubb5,Tmem173,Cdk2,Brca1,Cenph,Smarce1,Usp34,1700029F09Rik,Abcb1b,Eif4a3,Lig1,Frat2,Scrib,Set,Idh2,Hbs1l,2310057M21Rik,Nasp,Dhx15,Rad54l,Zfp54,Slco4a1,Espl1,E330009J07Rik,Ccna2,Cdc7,Cdca4,Dbf4,Prpf3,Kin,Psmd14,Hat1,Cdca3,2610318N02Rik,Sertad1,Pkmyt1,Mycn,Plscr1,Nav3,Lmnb1,Pafah1b3,Dcbld1,Col7a1,Zcchc8,Bcl2l11,Etv4,Rad51,Egfl7,Cdh10,Smarcc1,Kif23
**mmu-miR-464**	62(1239)	4,32E-12	Lrrc45,Asph,Timeless,Pcm1,Diap3,Whsc1,Ccbe1,Snx7,Smpdl3b,Pspc1,Bub3,Xkr5,Mad2l1,Polr1e,Kif18a,Mcm4,Mrps6,Nfyb,Mcm3,Hdac2,Luc7l,Rab15,Hspa14,Agxt2l2,Ctbp2,Dusp4,Pik3c2a,Ncapd2,U2af1,Npn2,Iqgap3,Shcbp1,Satb2,H2-K1,Lrrk1,2210018M11Rik,Ankrd57,2810474O19Rik,Espl1,Npm1,Topors,Pprc1,Melk,Net1,Ppbp,Nup88,Dut,Limd2,Patz1,Hmgn1,Xpo1,Zrsr2,Sertad1,Slc39a10,B3galnt1,Gprc5c,Ppwd1,Utp11l,Lifr,Ccdc18,Terf1,Tpbg
**mmu-miR-743b-3p**	61(1239)	6,89E-12	2810046L04Rik,Ell,Errfi1,Impa2,Prx,Dhx9,Ifi205,Ktn1,Cks1b,Nup37,Atad2,Pspc1,Dnmt1,Bcl10,Marcks,Cct7,Sfpq,Cenph,Cand1,Ung,Emg1,Pla2g4a,Ncapd2,1700029F09Rik,Ncapd3,Ube2c,Idh2,Pola1,Sbno1,Prpf4b,Ssb,Hmgb1,Rasa1,Top2a,Wee1,Hn1,2610002M06Rik,Rad18,Ppbp,Chd2,Ube2t,Casp8ap2,Trp53bp2,Dut,Abhd10,Patz1,Nucks1,Tpm3,Synj2,Nsmce4a,Ilf3,Slc43a3,Fancd2,Ppwd1,Utp11l,Incenp,Dyrk3,Pou2f1,H2afy2,2010204K13Rik,Pogz
**mmu-miR-216b**	64(1239)	7,11E-12	Nono,Asph,1110012J17Rik,Nup54,Trim24,Dgcr8,Fhl2,Depdc1b,Pak1,Ogfrl1,Smpdl3b,Scd1,Tes,2010002N04Rik,Zc3h15,Tiam2,Sgol2,Ttll4,Mrps6,Impdh2,Nfyb,Cand1,Rrm1,Exo1,Ctbp2,Pla2g4a,Set,Ncapd3,Suz12,2310057M21Rik,Sgms1,Gtf2h2,Stag1,Nusap1,Spred1,Zfp7,Larp4,Nfx1,Top2a,Cep70,Paip1,Hat1,Pigf,Smchd1,Wdr55,Trp53bp2,Lrig1,Patz1,Ddx31,Ptrh2,Ilf3,Cep55,Cnot6,Mcm6,Fancd2,E330016A19Rik,Zcchc8,Mre11a,Bcl2l11,Tnfaip6,Zfp281,Terf1,Tpbg,Eif2s1
**mmu-miR-19a**	64(1239)	9,58E-12	Nedd4l,Ahsa1,Cenpc1,Dgcr8,Dhx9,Steap1,Mum1,Fosl1,Crim1,Pank1,1110020G09Rik,Cep192,Aurka,Rbbp7,Marcks,Nrf1,E2f8,Hspd1,Tgif1,Apex2,Rnps1,Armc8,Gpd2,Aftph,Lin9,Zfp184,Enpp1,Ccdc99,Cbx1,Ccnb2,Psmc3ip,Bzw2,Scrib,Zc3h8,Zfp217,Kif21a,Imp3,Pola1,Ppp1cc,Rpl12,Prpf38a,Nasp,Sf3a1,Itgav,Unc5c,Rad18,Melk,Cenpa,Igf2bp1,Limd2,Lrig1,Abhd10,Actl6a,Slc39a10,Cnot6,Spc24,Ell2,Rsrc2,Cenpk,Whsc2,Ivns1abp,Ppa1,Gtse1,1810011O10Rik
**mmu-let-7d***	63(1239)	1,26E-11	Prim2,Mcm5,Impa2,Ktn1,Depdc1b,Nol9,Elf2,Pole2,Plk1,Ppih,Sass6,Tes,Pspc1,Cep192,Dnmt1,Rbbp7,Marcks,Nuf2,Ahctf1,Hspd1,Zw10,Acaa2,Pim3,Luc7l,Klhdc2,Zic1,Agxt2l2,Pik3c2a,Cd2ap,Zmynd19,Slc25a5,Ccnb2,Supt16h,Casc5,Bzw2,Set,Pcnt,Ptpn12,Ewsr1,Nasp,Wipf1,Ran,Spry2,Nfkbiz,Ncaph2,Rasa1,Hmgb2,Unc13c,Hat1,Sip1,Patz1,Kif22,BC016423,Nsmce4a,Suv39h2,Mycn,Yes1,Ddx10,Nav3,Esf1,Ift74,Wdr36,Smc4
**mmu-miR-451**	60(1239)	1,67E-11	Asph,Khsrp,Rbm26,Trim24,4930422G04Rik,Ankrd32,Rnmt,Dhx9,Prpf19,Pspc1,Ythdf2,Tcerg1,Msh6,Sltm,Tubb5,Tgif1,Sfpq,Aebp2,Msh3,Myc,Cand1,Asxl3,Eef1e1,Rpl30,Usp34,2610039C10Rik,Trim28,Mpzl1,Cul4b,Ppp1cc,Lrrk1,Ptpn12,Tube1,Chaf1b,Smc3,Npm1,Pif1,Hmgb2,Top2a,Unc13c,Ctsh,Mlf1ip,Sertad1,Synj2,Ilf3,Rbm14,Ddx20,Mboat2,Yes1,Cep55,Syne2,Nup133,Nolc1,B3galnt1,Rsrc2,Erh,E130308A19Rik,Arf6,Mrpl18,Rrs1
**mmu-miR-148a***	51(1239)	1,78E-11	Etaa1,Lrrc45,Tmem39b,Arhgap22,Socs5,Mum1,Wdr3,Riok2,Nrf1,Casp3,Prim1,BC055324,Lsm2,Cenph,Etf1,Cand1,Cbx1,Hnrpll,Cul4b,Satb2,2310057M21Rik,Sgms1,Imp3,Mcm10,Espl1,Usp3,Npm1,Nfx1,Prpf3,Ptcd3,3110003A17Rik,Gphn,Synj2,Ddx20,Cct8,Syne2,Rrad,Fancd2,E330016A19Rik,Cenpk,1110004E09Rik,Gmeb1,Tnfaip6,Khdrbs1,Egfl7,Pou2f1,Cdca7,H2afy2,Nxt1,Eftud2,Cirh1a
**mmu-miR-878-3p**	65(1239)	2,03E-11	Dffb,Zfp292,Dhx9,Zfp334,Pak1,Ctsw,Txnrd1,Swap70,Las1l,Rnf2,Wdr3,Elp2,Aurka,Rdx,Nuf2,Vrk3,Msh6,Zfp770,Mthfd1,Ebf2,Snrpb,Fnbp1l,Zfp184,Enpp1,Slc29a1,2610039C10Rik,Pdk3,Adss,Gpbp1,Kntc1,Recql4,Polr2b,Ppp1cc,Acsl3,Nxf1,Tube1,Hmgb1,Larp4,Card10,Kpna2,Npm1,Rfc5,Igsf3,Pvrl3,Wdr55,Clspn,Nde1,Patz1,Kif22,Nsmce4a,Plscr1,Cnot6,Syne2,Gprc5c,Rfc2,Tacc3,Ptbp2,Lifr,Cdca8,Tmpo,Uhrf1,Nxt1,Ahcyl1,Rbpj,Hnrpdl
**mmu-miR-300**	57(1239)	2,07E-11	Nedd4l,Sap30,H2afv,Fhl2,Nol9,Vps36,Gemin6,Atad2,Tes,1110020G09Rik,Bub3,Xkr5,Nuf2,Vrk3,Kif18a,Taf5l,Mcm3,Dbr1,Klhdc2,Etf1,Hspa14,Agxt2l2,Cd2ap,Zfp36l2,Ncapd2,Dtl,Cep152,Usp14,Snrpa1,Kif21a,Pcnt,Sbno1,Tial1,Nasp,Wipf1,Nfkbiz,Npm1,Hspa4l,Prpf3,Sumo2,Cep70,Nme4,Kin,Chd2,Ube2t,Fzd3,Cenpe,Kif22,Nudc,Spp1,Rcl1,Pcf11,Apex1,Egfl7,Cenpi,Arf6,Cdh10
**mmu-miR-106b**	63(1239)	2,82E-11	Nedd4l,Diap3,Gmnn,Dsn1,Atad2,Wdr3,Dnmt1,Pdia6,Rbbp7,Egr3,Rest,Ahctf1,Nt5dc2,Mcm3,Sfpq,Rrm2,Rad21,Dbr1,Cenph,Cep57,Lin9,Usp34,Casc5,Rad51c,Scrib,Idh2,Tslp,Ccng2,Gtf2h2,Casp8,2210018M11Rik,Ewsr1,Metap1,Tube1,Ipo7,Usp3,Kpna2,Bub1b,Wee1,Nme4,Gli3,Aifm1,Nup88,Iws1,Fzd3,Racgap1,Bbs12,Mcm7,Actl6a,Tnks2,Mastl,Ddx20,Osmr,Mycn,Ociad2,Cep55,Spp1,Ddx10,Rsrc2,Stil,Thoc2,Ezh2,Cebpz

miRNA name	*Number of Genes*	*Corrected p-value*	*miRNA targets among genes repressed in Rasless cells (Table S1)*
**mmu-let-7g***	65(1239)	3,15E-11	Snx5,Ncaph,Tmem39b,Fancm,Prx,Nsun2,Ets1,Nol9,Mybbp1a,Kif4,Cenpo,Ddx46,Dnmt1,Rdx,Pdia6,Egr2,Ank3,Nuf2,Slc11a2,Por,Mthfd1,Ect2,Nfyb,Pim3,Pgk1,Fnbp1l,Sox11,Dbr1,Emg1,Zmynd19,1700029F09Rik,Psmc3ip,Ncapd3,Pcnt,Idh2,Acsl3,Nasp,Pttg1,Colec12,Uchl5,Bub1b,Hmgb2,Snrpd1,Mbd4,Paip1,Melk,Pigf,Cenpn,3110003A17Rik,Limd2,Tipin,Kif22,Polr3b,Synj2,Nsmce4a,Foxp1,Pold3,Rrad,Nav3,Dcbld1,Dtymk,Pola2,Whsc2,Ppa1,E130308A19Rik
**mmu-miR-759**	57(1239)	3,19E-11	6430527G18Rik,Tmem39b,Diap3,Whsc1,D19Bwg1357e,Vps36,Tnnt2,Mki67,Plk1,Cenpq,Prdx4,Elavl1,Dnmt1,Riok2,Als2cr12,Socs3,Ttll4,Mthfd1,Tuba1c,Cenph,Adss,Cep152,Casc5,Prpf38b,Pcnt,Idh2,Ccng2,Gtf2h2,2210018M11Rik,Tial1,Lyar,Zfp451,Zfp54,Nupl1,Usp3,Kpna2,Pank4,Ppbp,Wdr55,Tinf2,Casp6,Synj2,Mastl,Foxp1,Ddx20,Tpp2,Cct8,H2afz,Spp1,Lmnb1,Stmn1,Mcm6,Cenpk,Utp11l,Tnfaip6,2010204K13Rik,Wdr36
**mmu-miR-467c**	67(1239)	3,20E-11	Nedd4l,Diap3,Prim2,Ankrd32,D19Bwg1357e,Vps36,Snx7,Zfp52,Tnnt2,Ctsw,Gemin6,Rangap1,Nap1l4,1110020G09Rik,Tcerg1,Aspm,Mup4,Msh6,Cpsf4,Acaa2,Junb,Hdac2,Armc8,Cenph,Klhdc2,Cep57,Zfp184,Ccdc99,Pik3c2a,Larp7,Eme1,Cep152,Zfp217,Kif21a,Shcbp1,Gtf2h2,Casp8,Crebzf,2210018M11Rik,Nxf1,Trim25,Zfp54,Wdr75,Usp3,Rasa1,Uchl5,Nme4,Mtm1,Ube2t,Cenpn,Slc38a1,Sox4,Cenpf,Ttc3,Apitd1,Nucks1,Mcm7,Actl6a,Mastl,Cx3cl1,Cacna1c,Slc43a1,Stil,Pcf11,Cenpi,Ltbp1,Cdt1
**mmu-miR-302c***	58(1239)	3,27E-11	6430527G18Rik,Prkar2b,Rbmxrt,Caprin1,D19Bwg1357e,Gmnn,Zfp52,Mki67,Hmmr,Prpf19,Ccne1,Atad2,Fubp1,Rpl7a,Elp2,Ccdc45,Aurka,Pgm1,Mad2l1,Zc3h15,Cit,Ccne2,Ppil5,Apex2,BC027072,Ctbp2,Larp7,Ncapg2,Ctnna1,Abcb1b,Casp2,Gnb4,Prpf4b,Lyar,Palld,Zfp7,2810474O19Rik,Zfp53,Ccna2,Ckap2,Snrpg,Nt5c3l,Rbbp8,Pvrl3,Ints2,Bnc2,Ell2,Dcbld1,Trub1,Nek2,Cnn3,Pola2,Pcf11,Tnfaip6,Ccdc18,4632434I11Rik,Hnrpdl,Eif2s1
**mmu-miR-425**	62(1239)	3,37E-11	Zfp292,Fhl2,Llgl1,Snx7,Dsn1,Ctsw,Prdx4,Wdr3,Noc4l,Cdc5l,Nuf2,Mrps6,Impdh2,Hdac2,Klhdc2,Zmym1,Smek1,Trip13,Slc29a1,2610039C10Rik,Zc3h8,Kif21a,Hbs1l,Sgms1,Lrrk1,Nasp,Fbl,Slco4a1,Cdca4,Phf17,Ckap2,Nfx1,Kin,Mtm1,Ptcd3,Ube2t,Sfxn1,Fzd3,Nudt21,Wdr55,Socs6,Plekhh2,Patz1,Kif22,Mlf1ip,Synj2,Slc39a10,Mastl,Ncapg,Ssrp1,Gprc5c,Rfc2,Ptbp2,Thoc2,Me2,Rad51,Nsl1,Cebpz,Mrpl18,Ahcyl1,Hnrpdl,Cirh1a
**mmu-miR-217**	59(1239)	4,24E-11	Nedd4l,Tgif2,Tmem39b,Pcm1,Diap3,Abi1,Depdc1b,Ets1,Ogfrl1,Spag5,Ppih,Dctd,Strbp,Wdr3,Dnmt1,Tpd52,Ube2e3,Ddx52,Ptpre,Ank3,Zc3h15,Ahctf1,Ythdc1,Lrp8,Snrpb,Aebp2,Tfdp1,Lin9,Pcna,Egfr,Matr3,Supt16h,Gpbp1,Ppp1cc,Rad54l,Hmgb1,2810474O19Rik,Nupl1,Usp3,Cdca4,Snrpg,Trit1,Zcchc2,Pigf,Axl,Pvrl3,Dut,Patz1,Neto2,Foxp1,Dtymk,2610027L16Rik,Thoc2,Sf3a3,Cdca8,Gart,Ezh2,E130308A19Rik,Lif
**mmu-miR-182**	62(1239)	4,24E-11	Fmr1,Nedd4l,6430527G18Rik,Asph,Prpf31,Diap3,Prim2,Ccbe1,Ktn1,Vps36,Kif2a,Txnrd1,Cenpq,Smpdl3b,Atad2,Aurkb,Cep192,Rpl7a,Ube2e3,Marcks,Ank3,Zw10,Schip1,Enpp1,Ppil1,Egfr,Trip13,Cbx1,Snrpa1,Pcnt,Lrrk1,Fanca,Tube1,Myo1b,Ckap2l,Rasa1,Npm1,Snrpd1,Tk1,Dbf4,Nme4,2610301G19Rik,Gemin8,Bbs12,Nudc,Nucks1,Topbp1,U2af2,2410016O06Rik,Foxp1,Specc1,Dtymk,Cep170,Ppwd1,Cnn3,Pola2,Cdca8,Twistnb,Hells,Smarcc1,Rrs1,Socs4
**mmu-miR-369-3p**	63(1239)	4,43E-11	Sirt1,Tnpo1,Prkar2b,Vegfc,Fhl2,Prpf40a,Snx7,Plk4,Spag5,Prdx4,1110020G09Rik,Ythdf2,Ddx46,Riok2,6720463M24Rik,Marcks,Nuf2,Wsb1,Prim1,Lcorl,Mcm3,Hdac2,Cd2ap,Pla2g4a,Trip13,Cbx1,Kif20a,Ccnb2,Runx2,Mbtps2,Usp14,Pcnt,Shcbp1,Ccng2,Sgms1,Ptpn12,Acsl3,Nup107,Cobll1,Fbl,Nap1l1,Cdc7,Phf17,2810055F11Rik,Ptk7,Trit1,Ptcd3,Ints7,Dut,Gemin8,Nsmce4a,Nup155,Yes1,E2f7,Ddx10,B3galnt1,Rfpl4,Fignl1,Ppwd1,Thoc2,Zfp281,Fancc,Zcchc11
**mmu-miR-410**	57(1239)	5,69E-11	Nono,Sub1,Prkar2b,Clns1a,Birc5,Abi1,Dhx9,Depdc1b,Nol9,Atad2,Rapgef2,Elp2,Nuf2,Cdca5,Zw10,Luc7l,Cdca2,Cd2ap,Zmynd19,Matr3,2310057M21Rik,Casp8,Nasp,Wipf1,Slbp,Hmgb1,Zfp54,Pttg1,Nr2f2,Ckap2,Paip1,Kin,Ctsh,Ppbp,Rbm17,Fgf7,Ints7,Dut,Mphosph10,Fgfr1op,U2af2,Cdc40,Foxp1,B3galnt1,C79407,Dcbld1,E330016A19Rik,Tacc3,Hirip3,Mast4,Rad51,Nxt1,Cdh10,Tpbg,Siva1,Kif23,Hnrpdl
**mmu-miR-130a**	65(1239)	6,06E-11	Nedd4l,1110012J17Rik,Diap3,Rcc2,Cenpc1,Prkar2b,Rbmxrt,Cks1b,Vps36,Spred2,Snx7,Cenpq,Idh3a,Ythdf2,Ddx46,Exosc8,5730590G19Rik,Mup4,Parp1,Zc3h15,Tiam2,Kif18a,Mcm4,Suv39h1,Hdac2,Zfp184,Enpp1,Ccdc99,Pde1a,Gins1,Ncapd2,Chst1,Abcb1b,Cep152,Otud4,2310057M21Rik,Rpl12,Zfp451,Fbl,Slco4a1,Wdr75,Rasa1,Rpa2,Nme4,2610301G19Rik,Rad18,Mtm1,Hat1,Ptplad1,Ptcd3,Ube2t,Dut,Limd2,Gemin8,Tipin,Slco1a5,Rbm14,Tnrc6a,B3galnt1,Pcf11,Khdrbs1,Arf6,Slc4a7,Kif23,Hnrpdl
**mmu-miR-883b-3p**	65(1239)	6,72E-11	Snx5,Lrrc45,Tmem39b,Depdc1b,Ets1,Snapc3,Mki67,Cenpq,Tes,Pank1,Paxip1,Nap1l4,Dnmt1,Tpd52,Msh2,Ruvbl1,Rest,Zc3h15,Ahctf1,Lrp8,Snrpb,Cct5,Luc7l,Klhdc2,Ash2l,Pcna,U2af1,Set,Hnrpll,Suz12,Dhx15,Cbx3,Ran,Spry2,Wdr75,Nupl1,Hspa4l,D10Wsu102e,Itih2,Arid4b,Egr1,Trit1,Tpx2,Mkx,Nab2,Ckap4,Nup88,Rsf1,Syncrip,Nudc,Gnl3,Pbk,Ilf3,Eif5,Plekha5,Ell2,Fbxo32,Nob1,Dtymk,Rasal2,Thoc2,Phlda1,Ppa1,Ezh2,Lif
**mmu-miR-20b***	54(1239)	7,60E-11	Asph,Tmem39b,Pcm1,Prim2,Prkar2b,Mcm5,Plk1,Tes,Emb,Pspc1,Srpk1,Exosc8,Aurka,Fkbp5,Tiam2,Ttll4,Mrps6,Prim1,Lsm2,Sox11,Armc8,Rab15,BC027072,Ctbp2,Hmgb3,Emg1,Larp7,Zmynd19,Cbx1,Pkn3,Recql4,2310057M21Rik,Gtf2h2,Ptpn2,H2afy,Nasp,Dhx15,Kars,Usp3,E330009J07Rik,Mns1,Rfc5,Melk,3110003A17Rik,Prkg2,Ptrh2,Foxp1,Nav3,Fancd2,2610027L16Rik,Sf3a3,Ccdc18,Nxt1,Smyd5
**mmu-miR-691**	58(1239)	8,24E-11	Nedd4l,Asph,Arhgap22,Pcm1,Caprin1,Skp2,D19Bwg1357e,Bclaf1,Rpl7a,Bcl10,Cep68,Nrp1,Mtf2,Dusp9,Fen1,Snrpb,Lsm2,Sfpq,Hdac2,Setx,Micall2,Gsg2,Rsbn1,Pnpt1,Fanca,Csnk1g3,Cobll1,Spry2,Tyms,Usp3,Hmga2,Itgav,Egr1,Pank4,Psmd14,Sfxn1,Ddx11,Kif2c,Rad51ap1,Slco1a5,Rbm14,Usp37,Nav3,Fancd2,Dcbld1,Rcl1,Mre11a,Ppwd1,Atrx,C330027C09Rik,Egfl7,Pou2f1,Ezh2,Cebpz,Top1,Eftud2,2810408A11Rik,Ecd
**mmu-miR-139-5p**	63(1239)	8,41E-11	Cdca7l,H2afv,Zfp292,Prim2,Birc5,Depdc1b,Acly,Ctsw,Plk1,Paxip1,Setdb2,Pspc1,Pdgfb,1110020G09Rik,Tpd52,Ppat,Ank3,Sgol2,Kif18a,Hspd1,2510012J08Rik,Ttll4,Slc11a2,Mrps6,Schip1,Fen1,Pgk1,Myc,Emg1,Cbx1,Ankrd10,Lig1,Ccnf,Kntc1,Spc25,Sgms1,H2-K1,Crebzf,2210018M11Rik,Ssb,Dhx15,Mns1,Unc13c,Pigf,Fzd3,Jub,Rbmx2,Cct3,Casp6,Kcnk2,Synj2,Ociad2,Ssrp1,B3galnt1,Fbxo32,Rpl13a,Nek2,Pola2,Gpsm2,Ppa1,Ezh2,Fancc,Ecd
**mmu-miR-487b**	59(1239)	8,76E-11	Snx5,2810046L04Rik,Trim24,Mcm5,Cpsf2,Upf3b,Hspa8,Prpf40a,Snx7,Mki67,Cep192,Pnn,Aspm,2510012J08Rik,Nfyb,Zic1,Asxl3,Enpp1,Ppil1,Usp34,Crlf1,Mpzl1,Pcnt,Cul4b,Hmgb1,Topors,Ckap2,Sumo2,G3bp2,Ebna1bp2,Cachd1,Nup88,Ube2t,Socs6,Smc2,Gnl3,Mlf1ip,Actl6a,G3bp1,Nsmce4a,Nup155,Anp32b,Plscr1,Nav3,Mcm6,Alkbh1,Rasal2,Rpl13a,Pola2,Whsc2,Zfp281,C330027C09Rik,Ezh2,Fancc,Cdc73,Cdh10,Top1,Mrpl18,Kif23
**mmu-miR-669c**	62(1239)	8,81E-11	Etaa1,Pop1,Prim2,D19Bwg1357e,Depdc1b,Elf2,Glrx,Kif2a,Mum1,Ripk2,Tes,Kif4,Fubp1,Ddx27,Exosc8,Pgm1,Aspm,Myef2,Gspt1,Nol10,Mcm4,Ppil5,Tmem48,D2Ertd750e,Mcm3,Cct5,Luc7l,Etf1,Myc,Agxt2l2,Ash2l,Dusp4,Rpl30,Zmym1,Pde1a,Cbx1,Chst1,Pnpt1,Prpf38a,Ssb,Kars,Spry2,Hmgb2,Neil3,Dbf4,Cep70,Arid4b,2610301G19Rik,Ctsh,Casp8ap2,Gphn,Cenpl,BC016423,Usp37,Ddx20,Dapp1,Tnrc6a,Plscr1,Dcbld1,Dyrk3,Tpbg,Tdrkh
**mmu-miR-190**	54(1239)	8,86E-11	Snx5,Sap30,Pop1,Diap3,Ifi205,Steap1,Snx7,Nup37,Pole2,Plk1,Smpdl3b,Atad2,Pgm1,Rbbp7,Mad2l1,Kif18a,Yme1l1,Wsb1,Msh6,Blm,Tmem173,Pla2g4a,Pde1a,Ncapd3,Shcbp1,Rsbn1,Hbs1l,Tial1,Nup107,Lyar,Zfp451,Chek1,Nfkbiz,Nfx1,Paip1,Pank4,Mtm1,Psmd14,Hat1,Cenpn,Gphn,Synj2,Slco1a5,Eif5,Tnrc6a,Bhlhb9,Ppwd1,Mast4,Pdss1,Hells,Dyrk3,Nsl1,Rbl1,Zcchc11
**mmu-miR-34c***	51(1239)	9,47E-11	Lrrc45,Cenpc1,Prkar2b,Marcksl1,Impa2,Dhx9,Kif2a,Plk4,Tnnt2,Pspc1,Ddx52,Bcl10,Msh2,Zc3h15,Ttll4,Sltm,Mtf2,Tmem173,Tuba1c,Armc8,Cand1,Rpl30,Psmc3ip,U2af1,Casc5,Lig1,Scrib,Smc5,Fbl,Ipo7,Chaf1b,Dbf4,Arid4b,Psmd14,Smchd1,Lrig1,Ghr,Ahcy,Actl6a,Ilf3,Eif5,Strap,B3galnt1,Rcl1,Cenpk,Ppwd1,Dnaja1,Ddx39,Rbpj,Dnajc9,Dmap1

miRNA name	*Number of Genes*	*Corrected p-value*	*miRNA targets among genes repressed in Rasless cells (Table S1)*
**mmu-miR-25**	65(1239)	1,03E-10	Rbm26,Sirt1,H2afv,Fhl2,Ets1,Zfp606,Has2,Cenpq,Dcp1a,Rpl7a,Ddx52,Mup4,6720463M24Rik,Egr2,Nrf1,Ank3,Ahctf1,Cit,Mtf2,Tgif1,BC055324,Nfatc2ip,Rad21,Eef1e1,Zmynd19,B230120H23Rik,2700029M09Rik,Arl4c,Pcnt,Tslp,Cul4b,Rsbn1,Mtap,Rpl12,Tube1,Nusap1,Zfp54,Hspa4l,Mmd,Mbd4,Prpf3,Sumo2,Mtm1,Pkp2,Anln,3110003A17Rik,Abhd10,Neto2,Polr3b,Gas2l3,Usp37,Eif5,Pold1,Mcm6,Fancd2,Dcbld1,Ppwd1,Atrx,Nek2,Pcf11,Ppa1,Cdh10,Dock5,Mrpl18,Rbpj
**mmu-miR-30a***	58(1239)	1,12E-10	Prpf31,Prim2,Prkar2b,Elf2,Acly,Spag5,Dsn1,Hmmr,Cenpq,Xpo7,Ank3,Vrk3,Lcorl,Lsm2,Luc7l,Etf1,Egfr,Emg1,Usp34,Gins1,Abcb1b,B230120H23Rik,Ncapd3,Smc5,Rps9,Troap,H2afy,2810474O19Rik,Kin,2810025M15Rik,Ints5,Rbm17,Axl,Clspn,Patz1,Nudc,Ahcy,Synj2,Mycn,Spp1,Syne2,Stmn1,Mcm6,Dcbld1,Rcl1,Lrrc8c,Ppwd1,AI848100,Cdca8,Gmeb1,Khdrbs1,C330027C09Rik,4632434I11Rik,Arf6,Cdc73,Gtse1,Mrpl18,Smc4
**mmu-miR-32**	63(1239)	1,24E-10	6430527G18Rik,Rbm26,Trim24,Fancm,Dhx9,Fhl2,Taf5,Plk4,Has2,Smpdl3b,Rangap1,Cenpo,Strbp,Dnmt1,Mup4,Marcks,Nrf1,Ahctf1,Mtf2,Tgif1,BC055324,Nfatc2ip,Rad21,Ppil1,Eef1e1,Rpl30,Hmgb3,Larp7,Chst1,B230120H23Rik,2700029M09Rik,Prpf38b,Cul4b,Rsbn1,Ccng2,Ewsr1,Acsl3,Sf1,Lyar,Zfp54,Myo1b,Nfkbiz,Mbd4,Zcchc2,Psmd14,Iws1,Usp37,Ncapg,Eif5,Ell2,Pold1,Fancd2,Dcbld1,Dtymk,Ppwd1,Rpl13a,Atrx,Pcf11,Rad51,Ppa1,Cdh10,Dock5,Tcf4
**mmu-miR-126-3p**	59(1239)	1,41E-10	Tmem39b,Pcm1,Prkar2b,Impa2,D19Bwg1357e,Gmnn,Depdc1b,Elf2,Smpdl3b,Lrig3,Atad2,Las1l,1110020G09Rik,Cep192,Aspm,Msh6,Casp3,Schip1,Suv39h1,Cct7,Pim3,Asxl3,Eef1e1,Pdk3,Bzw2,Zfp217,Idh2,Atad5,Stoml2,Dhx15,Tube1,Hspa4l,Ckap2,Pank4,Net1,Cachd1,Pvrl3,Ints7,Slc38a1,Cenpf,Ss18,Gnl3,Pbk,Ilf3,Cacna1c,Syne2,E2f7,B3galnt1,Rfc2,Cenpk,Pdss1,Rad51,Ezh2,Tmpo,Dock5,Tpbg,Kif23,Dmap1,Eps8
**mmu-miR-883a-3p**	64(1239)	1,58E-10	Snx5,Lrrc45,Phf6,Depdc1b,Ets1,Snapc3,Mki67,Cenpq,Tes,Pank1,Nap1l4,Pspc1,Ythdf2,Dnmt1,Aspm,Ruvbl1,E2f8,Rest,Cep68,Zc3h15,Ahctf1,Lrp8,Ect2,Snrpb,Cct5,Luc7l,Klhdc2,Pcna,Hmgb3,Trip13,Adss,U2af1,Set,Hnrpll,Ptpn2,Dhx15,Cbx3,Spry2,Nupl1,Cdc7,Itih2,Arid4b,Egr1,Tpx2,Mkx,Nab2,Nup88,Rsf1,Cenpf,Nudc,Ghr,Gnl3,Pbk,Eif5,Plekha5,Ell2,Fbxo32,Dtymk,Rasal2,Thoc2,Phlda1,Rad51,Incenp,Ezh2
**mmu-miR-181a**	60(1239)	1,66E-10	Tgif2,Rnmt,Fhl2,Cks1b,Mum1,Plk4,Ap1s3,Aurkb,Pspc1,Noc4l,Pdia6,Bcl10,Rbbp7,Nrf1,Vrk3,Wsb1,Fxn,Schip1,Pdap1,BC055324,Nfatc2ip,Ccdc99,Pcna,Pla2g4a,Gins1,Gpbp1,Npn2,Ccnf,Snrpa1,Troap,Gtf2h2,H2-K1,Crebzf,Rad54l,Nap1l1,Cdc7,Hmgb2,Nfx1,Rpa2,Melk,Psmd14,Ube2t,Actl6a,Wdr73,Polr3b,Cdc40,Ilf3,Foxp1,Dapp1,E2f7,Rfc2,Col7a1,Phlda1,Me2,Gpsm2,Ppa1,Cenpi,Uhrf1,Nxt1,Rbpj
**mmu-miR-376a**	61(1239)	1,84E-10	Etaa1,D19Bwg1357e,Ifi205,Cks1b,Tgfbr3,Pak1,Mum1,Lrig3,Fubp1,Ddx46,Elp2,Pgm1,Tmem176b,Mrps6,Slit2,Snrpb,Msh3,Ccdc99,Pcna,Psrc1,Ctnna1,Trim28,Polr2b,Lrrk1,Ssb,Lyar,Zfp451,Espl1,Nupl1,Kpna2,Ncaph2,Rbm25,4930579G24Rik,Itgav,2610002M06Rik,Aifm1,Cachd1,Chd2,Nup88,Pigf,Ube2t,Fgf7,Wdr55,Anln,Cct3,Gnl3,Cenpl,Usp37,Tpp2,5730559C18Rik,Plscr1,Ssrp1,Cnot6,Spp1,Gprc5c,D16Ertd472e,Rfc2,Ppwd1,Thoc2,Tnfaip6,2010204K13Rik
**mmu-miR-302a**	62(1239)	2,01E-10	Diap3,Ankrd32,Skp2,Ktn1,Llgl1,Bclaf1,Fosl1,Ctsw,Cenpq,2810008M24Rik,Atad2,Dnmt1,Ddx52,Egr3,Rest,Parp1,Vrk3,Mrps6,Schip1,Prim1,Rnps1,Ppid,Dbr1,Larp7,Cbx1,Cep152,E130303B06Rik,Idh2,Gtf2h2,Lrrk1,Nxf1,Asf1b,Pttg1,Kpna2,Snw1,Nfkbiz,Cdca4,Neil3,Rpa2,Wee1,Hn1,Arid4b,2610301G19Rik,Aifm1,Nup88,Ptcd3,Smchd1,Wdr55,Trp53bp2,Dut,Bbs12,Gnl3,Synj2,Cep55,Rsrc2,Pdss1,Thoc2,Hells,Ccdc18,4632434I11Rik,Pou2f1,Cdh10
**mmu-miR-448**	51(1239)	2,13E-10	Cenpc1,Prkar2b,Elf2,Steap1,Kif2a,Mki67,Cenpq,Aurkb,Ube2e3,Aspm,Myef2,Ank3,Zc3h15,Vrk3,Klhdc2,Tll1,Lin9,Rpl30,Sh3kbp1,Setx,Cul4b,Gtf2h2,Ptpn12,Spry2,Ipo7,Nupl1,Csnk1a1,Snw1,Ubqln2,Ckap2,Prpf3,Arid4b,Paip1,Tpx2,Pkp2,Ube2t,Casp8ap2,Gphn,Kcnk2,Gnl3,Topbp1,Nsmce4a,Usp37,Ddx20,Eif5,Tpp2,Ppwd1,Pdss1,Tnfaip6,Dyrk3,Eps8
**mmu-miR-26b**	59(1239)	2,15E-10	Fmr1,Diap3,Rnmt,Depdc1b,Hmmr,Ccne1,Mnd1,Rpl7a,Msh2,Xkr5,Mad2l1,Rest,Tox,Kif18a,Zw10,Sltm,Impdh2,Cct7,Pim3,Hmga1,2610039C10Rik,Cbx1,Pkn3,Cep152,Ube2c,Kif21a,Shcbp1,Elovl6,Cul4b,Hmgb1,Slco4a1,Usp3,Kpna2,Dimt1,Neil3,Psmd14,Net1,Zwilch,Mpp6,Ube2t,Cdca3,Smc2,Synj2,G3bp1,Usp37,Ncapg,Ints2,Cct8,Tnrc6a,Stmn1,Ppwd1,Vbp1,Hells,Ivns1abp,Ezh2,Tmpo,Terf1,Cks2,Hnrpdl
**mmu-miR-467d**	66(1239)	2,18E-10	Nedd4l,Lrrc45,Diap3,Mtmr4,D19Bwg1357e,Vps36,Snx7,Tnnt2,Ctsw,Gemin6,Rangap1,Nap1l4,1110020G09Rik,Cep192,Tcerg1,Aspm,Mup4,Msh6,Cpsf4,Acaa2,Ifitm3,Hdac2,Klhdc2,Zfp184,Ppil1,Ccdc99,Pik3c2a,Kif21a,Shcbp1,Hbs1l,Imp3,Casp8,2210018M11Rik,Ewsr1,Nxf1,Trim25,Zfp54,Wdr75,Usp3,Rasa1,Uchl5,Nme4,Mtm1,Sox4,Cenpf,Apitd1,Nucks1,Mcm7,Actl6a,2410016O06Rik,Slco1a5,Mastl,Cx3cl1,Cacna1c,Slc43a1,Syne2,Ddx10,Mcm6,Phlda1,Pcf11,Ccdc18,Gpsm2,Cdca7,Cenpi,Ltbp1,Cdt1
**mmu-miR-291b-5p**	64(1239)	2,44E-10	6430527G18Rik,Ncaph,Cdca7l,Sap30,Tmem39b,Pcm1,Cks1b,Glrx,Snx7,Nup37,Plk1,Cenpq,Prpf19,Mrps22,Bub1,Cep192,Pnn,Tcerg1,Rdx,Rbbp7,Egr2,Ruvbl1,Nrf1,Nol10,Socs3,Zw10,Sltm,Brca1,Ash2l,Rrm1,Sgol1,Rcc1,Cdc25a,Hmga1,Thsd7a,Casc5,Crlf1,Snrpa1,Prpf38a,Asf1b,Espl1,Nupl1,Ckap2l,E330009J07Rik,Pprc1,Nfx1,Rbbp8,Psmd14,Cdca3,Tinf2,Dus3l,Nsmce4a,Foxp1,Tnrc6a,Mcm2,Actr3,Slc43a3,Ddx10,Vbp1,Incenp,E130308A19Rik,Gtse1,Tpbg,Slc4a7
**mmu-miR-411**	57(1239)	2,81E-10	Sap30,1110012J17Rik,Arhgap22,Cenpc1,Trim24,Ercc6l,Mybbp1a,Ap1s3,Cenpq,Sass6,Nipsnap1,Elp2,Rdx,Hspd1,Mtf2,Acaa2,Tgif1,Cct7,Pim3,Mcm3,Rrm2,Nudt1,Asxl3,Ccnb2,Runx2,Cep152,Casp2,Kif21a,Pcnt,Depdc1a,Stoml2,Dhx15,Slco4a1,Wdr75,Gins4,Cdca4,Tk1,Tpx2,Psmd14,Ppbp,Abhd10,Casp6,Chtf18,Gnl3,Pbk,Actl6a,Dapp1,Yes1,Mre11a,Timp1,Lifr,Cdca8,Rad51,Incenp,Ppa1,Gtse1,Dnajc9
**mmu-miR-382***	49(1239)	2,88E-10	Etaa1,2810046L04Rik,Trim24,Rnmt,Spred2,Kif2a,Dsn1,Dctd,Prim1,BC055324,Klhdc2,Zfp184,Zik1,Pla2g4a,Smek1,Abcb1b,Npn2,Pcnt,Casp8,Metap1,Csnk1g3,Ankrd57,2810474O19Rik,E330009J07Rik,Rbm25,4930579G24Rik,Nt5c3l,Pkp2,Pvrl3,Eif4enif1,Patz1,Wdr73,Eif5,Tpp2,Dapp1,Strap,Plscr1,Slc43a3,Pum2,Rfpl4,Sdpr,Col7a1,Rcl1,Ddx39,Pebp1,Ivns1abp,Tmpo,Rbpj,Kif23
**mmu-miR-450b-5p**	60(1239)	2,90E-10	6430527G18Rik,Diap3,Ercc6l,Caprin1,Ifi205,Snx7,Nup37,Zfp52,Mapk8,1110020G09Rik,Ythdf2,Ddx46,Exosc8,Tcerg1,Riok2,6720463M24Rik,Fkbp5,Tiam2,Nrp1,Ahctf1,Gspt1,Prim1,BC055324,Pim3,Lsm2,Rrm1,Pcna,Emg1,Pde1a,E130303B06Rik,Pcnt,Tslp,Recql4,Rsbn1,Hbs1l,Sbno1,Lrrk1,E330009J07Rik,Itih2,Dbf4,Snrpg,Hat1,Pvrl3,3110003A17Rik,Socs6,Clspn,Ss18,Apitd1,Usp37,Eif5,Anp32b,Dcbld1,Cenpk,Nol11,Pcf11,Incenp,Dyrk3,Ezh2,Smyd5,Smc4
**mmu-miR-717**	58(1239)	3,28E-10	Prim2,Ell,Ankrd32,D19Bwg1357e,Mum1,Plk1,Rangap1,Idh3a,2010002N04Rik,Noc4l,Elp2,Tiam2,Acaa2,Hdac2,Tuba1c,Luc7l,Klhdc2,Usp34,Ncapg2,Ncapd2,Pdk3,Kif20a,Chst1,Spc25,Baz1a,Parp12,2310057M21Rik,Ppp1cc,Mtap,Wipf1,Usp3,Cdc7,Snrpg,Hn1,Mcpt8,2810025M15Rik,Ptplad1,Fzd3,Anln,Dus3l,Prmt5,Wdr73,Sertad1,Ilf3,Cdkn2c,Dapp1,Strap,Actr3,Tacc3,Cenpk,Ptbp2,Tex10,Nol11,Incenp,4632434I11Rik,Rbm12,Gtse1,Rbpj
**mmu-miR-92a**	63(1239)	3,92E-10	Gata2,Rbm26,Sirt1,H2afv,Birc5,Fhl2,Ets1,Has2,Dcp1a,Cdc27,Mup4,Nrf1,Rest,Zc3h15,Ahctf1,Cit,Tgif1,BC055324,Nfatc2ip,Rad21,Asxl3,Eef1e1,B230120H23Rik,2700029M09Rik,Arl4c,Pcnt,Cul4b,Rsbn1,Ccng2,Mtap,Rpl12,Ewsr1,Tube1,Nusap1,Zfp54,Myo1b,Mmd,Tk1,Mbd4,Mtm1,Sfxn1,Prps1,3110003A17Rik,Abhd10,Neto2,BC016423,Slco1a5,Foxp1,Eif5,Pum2,Fancd2,Dcbld1,Dtymk,Ppwd1,Atrx,Pcf11,Zfp281,Gpsm2,Ppa1,Nsl1,Cdh10,Dock5,Mrpl18
**mmu-miR-302b**	60(1239)	4,69E-10	Etaa1,Diap3,Ankrd32,Llgl1,Bclaf1,Cenpq,Atad2,Dnmt1,Riok2,Ddx52,Egr3,Rest,Parp1,Vrk3,Schip1,Prim1,Rnps1,Ppid,Dbr1,Trib1,Larp7,2610039C10Rik,Cbx1,Abcb1b,Cep152,Casp2,E130303B06Rik,Idh2,Tslp,Gtf2h2,Lrrk1,Nxf1,Asf1b,Pttg1,Kpna2,Snw1,Nfkbiz,Cdca4,Rpa2,Wee1,Hn1,Nmral1,Aifm1,Nup88,Smchd1,Wdr55,Trp53bp2,Dut,Tinf2,Bbs12,Synj2,Cep55,Rsrc2,Pdss1,Pcf11,Hells,C330027C09Rik,Ccdc18,4632434I11Rik,Pou2f1

miRNA name	*Number of Genes*	*Corrected p-value*	*miRNA targets among genes repressed in Rasless cells (Table S1)*
**mmu-miR-434-3p**	59(1239)	4,74E-10	Etaa1,Timeless,Mtmr4,Caprin1,Tcf19,Ucp2,2610101N10Rik,Ddx27,Ddx46,Tcerg1,E2f8,Cdc5l,Kif18a,Ttll4,Zw10,Ppid,BC055324,Ctbp2,Zmynd19,Scrib,Zc3h8,Ccng2,Atad5,Gtf2h2,Ptpn2,H2-K1,Rpl12,Metap1,Trim25,Dhx15,Cbx3,Pif1,Nr2f2,Rpa2,Arid4b,Sip1,Pvrl3,Eif4enif1,Tbx3,Nutf2,Tpm3,Synj2,Cacna1c,Syne2,B3galnt1,Mrpl1,Cd14,Gprc5c,Fancd2,Zcchc8,Thoc2,Cdca8,Gmps,Incenp,Ppa1,Ahcyl1,2810408A11Rik,Rrs1,Eif2s1
**mmu-miR-200a**	64(1239)	5,08E-10	6430527G18Rik,Timeless,Birc5,Abi1,Rnmt,Glrx,Arhgap11a,Fus,Has2,2610101N10Rik,Setdb2,Wdr3,Ythdf2,Ddx46,Tcerg1,Ube2e3,Aspm,Mup4,Ruvbl1,Fkbp5,Ctcf,Ythdc1,Ccne2,Cyp26b1,Schip1,Mtf2,Api5,Sfpq,Luc7l,Klhdc2,Rrm1,Ctbp2,Trip13,Cbx1,Kif20a,U2af1,Lig1,Dusp6,Baz1a,Idh2,Suz12,Vangl2,Ssb,Ptges3,Tead2,Mbd4,Unc5c,Arid4b,Kin,Net1,Dus3l,Tnks2,Ilf3,Mcm6,Arhgap19,Cep170,Rpl13a,Mast4,Psmd5,Bcl2l11,Tfrc,Top1,Mrpl18,Eps8
**mmu-miR-194**	59(1239)	5,52E-10	Tmem39b,Trim24,Marcksl1,Dhx9,Depdc1b,Cks1b,Steap1,Txnrd1,2610101N10Rik,Pspc1,Exosc8,Pnn,E2f8,Vrk3,Nrp1,Sltm,Schip1,Blm,Tgif1,Cldn12,Cct5,Luc7l,Ash2l,Egfr,Trip13,Dtl,Set,Hnrpll,Gtf2h2,Ptpn2,Fanca,Hyal2,Fbl,Bub1b,Top2a,Dbf4,Sumo2,Rsf1,Rbm17,Cenpn,Fzd3,Nudt21,Jub,Rbmx2,Cd24a,Limd2,Nudc,BC016423,Wdr73,Synj2,Cct8,Hirip3,Ppwd1,1700025G04Rik,Cdca8,Ppa1,Nsl1,E130308A19Rik,Dmap1
**mmu-miR-215**	57(1239)	5,61E-10	Cdca7l,2700050L05Rik,Mtmr4,Upf3b,Ets1,Ccne1,Sass6,2610101N10Rik,Setdb2,Wdr5,Pdgfb,Noc4l,Aurka,Ruvbl1,Zc3h15,Tox,Slc11a2,Dhx36,Luc7l,Tll1,Sms,Rrm1,Ccdc99,Ncapg2,Casc5,Smc5,Pnpt1,Depdc1a,Larp4,D10Wsu102e,Ckap2,1110034A24Rik,Top2a,Snrpg,Nme4,Nmral1,Egr1,Isg20l2,Gphn,Chtf18,Rif1,Cdc6,D16Ertd472e,Fancd2,E330016A19Rik,Ppwd1,Psmd5,Ddx39,Cdca8,Pcf11,Hells,Gart,Dyrk3,2010204K13Rik,Rbpj,Slc4a7,Pogz
**mmu-miR-294**	55(1239)	6,65E-10	Etaa1,Ncaph,Zfp292,Spred2,Kif2a,Bclaf1,Ctsw,Cenpq,Kif4,Elavl1,1110020G09Rik,Dnmt1,Pdia6,Egr3,Rest,Vrk3,Tmem173,Trib1,Larp7,Abcb1b,Psmc3ip,Cep152,E130303B06Rik,Ccng2,Gtf2h2,Lrrk1,Rpl12,Stk17b,Palld,Kpna2,Pif1,Cdca4,Rpa2,Wee1,Hn1,Runx1,Aifm1,Nup88,Iws1,Pvrl3,Wdr55,Bbs12,Asf1a,Cep55,Dtymk,Rsrc2,Mast4,Pdss1,Thoc2,Cnn3,Pebp1,Ccdc18,4632434I11Rik,Pou2f1,Cebpz
**mmu-miR-181d**	59(1239)	7,13E-10	Nedd4l,Tgif2,1110012J17Rik,Prkar2b,Rnmt,Amd1,Mum1,Phactr4,Rapgef2,Aurkb,Pspc1,1110020G09Rik,Ythdf2,Pdia6,Vrk3,Tox,Fxn,Schip1,Pdap1,Acaa2,Prim1,BC055324,Rad21,Ccdc99,Pcna,Pla2g4a,Gins1,Abcb1b,Mbtps2,Gpbp1,Npn2,Ccnf,Snrpa1,Hbs1l,Troap,Gtf2h2,H2-K1,Crebzf,Rpl12,Rbm25,Hmgb2,Nfx1,Rad18,Melk,Tpx2,Fgf7,4930427A07Rik,Actl6a,Cdc40,Foxp1,Mboat2,Ptbp2,1110004E09Rik,Cdca8,Phlda1,Me2,Ppa1,Nxt1,Rbpj
**mmu-miR-362-3p**	62(1239)	7,24E-10	Fmr1,Cdca7l,Asph,Pcm1,Dffb,Impa2,Whsc1,D19Bwg1357e,Eef1g,Arhgap11a,Gemin6,Rangap1,Ncl,Noc4l,Tpd52,D2Wsu81e,Ppat,Slc11a2,Cct7,Lsm2,Tuba1c,Cct5,Aftph,Cep57,Scarb1,Cbx1,Brd8,Lig1,Mpzl1,Rad51c,Kif21a,H2afy,Metap1,Zfp451,Tardbp,Kpna2,Colec12,Cdc7,Gins4,Syt17,Pkp2,Limd2,Rps13,Cdc40,Ociad2,Actr3,Cnot6,Slc43a1,Nufip1,Pold1,Rfc2,Cenpk,Ptbp2,Mast4,1110004E09Rik,Erh,Cdca8,Nol11,Cd300lb,Uhrf1,Tpbg,Hnrpdl
**mmu-miR-450a-5p**	57(1239)	7,68E-10	2810046L04Rik,Diap3,Prkar2b,Ercc6l,Upf3b,Glrx,Snx7,Cenpq,Ythdf2,Ddx46,Rpl7a,Pnn,Tcerg1,Riok2,Ndc80,Ruvbl1,Gspt1,Slit2,Msh3,Sh3kbp1,Gpbp1,Casc5,Peg12,Pcnt,Shcbp1,Tslp,Rpl12,Tial1,Hyal2,Ssb,Zfp451,Spred1,Spry2,Slco4a1,Top2a,Itih2,Cep70,Mtm1,Ankrd28,Pvrl3,Fzd3,Wdr55,3110003A17Rik,Clspn,Cct3,G3bp1,Slco1a5,Rasal2,Cenpk,Sf3a3,Rad51,Dyrk3,4632434I11Rik,Ezh2,Nsl1,Hnrpdl,Tcf4
**mmu-miR-137**	59(1239)	7,79E-10	Ercc6l,Vegfc,Birc5,Abi1,Dhx9,Ccbe1,Nol9,Arhgap11a,Plk4,Tnnt2,Pgm1,Bcl10,Parp1,Ahctf1,Ythdc1,Nt5dc2,Casp3,Mrps6,Acaa2,Cenph,Dusp4,Pcna,Smek1,Slc25a5,Cbx1,Ctnna1,Casc5,Ccnb1,Zfp217,Baz1a,Ptgs2,Cul4b,Satb2,Ccng2,Gtf2h2,Ptpn2,Mtap,Tube1,Spry2,Nupl1,Gins4,Npm1,Hn1,Cep70,Arid4b,Gli3,Nab2,Jub,Trp53bp2,Rps13,Slc25a24,Mastl,Suv39h2,Cep55,Mcm6,D16Ertd472e,E330016A19Rik,Ezh2,Mrpl18
**mmu-miR-29a**	66(1239)	8,04E-10	Ranbp1,Prpf40a,Elf2,Ucp2,Mki67,Plk1,Rapgef2,Las1l,Aurkb,Mapk8,Noc4l,6720463M24Rik,Cep68,Nol10,Tmem48,Blm,Tubb5,Tmem173,Cdk2,Brca1,Sfpq,Smarce1,Usp34,Abcb1b,Eif4a3,Lig1,Scrib,Set,Idh2,Hbs1l,2310057M21Rik,Nasp,Rad54l,Zfp54,Espl1,E330009J07Rik,Ccna2,Cdc7,Cdca4,Dbf4,Prpf3,Kin,Psmd14,Hat1,Cdca3,Sox4,2610318N02Rik,Sertad1,Pkmyt1,Mycn,Nav3,Lmnb1,Pafah1b3,Dcbld1,Col7a1,Zcchc8,Rpl13a,Bcl2l11,Etv4,H2afx,Rad51,Egfl7,Cdh10,Smarcc1,Kif23,Dmap1
**mmu-miR-411***	46(1239)	8,09E-10	Chd1,Tmem39b,Rbm26,Prkar2b,Depdc1b,Elf2,F2rl1,Arhgap11a,Ruvbl1,Mad2l1,D2Wsu81e,Cdc5l,Gspt1,Yme1l1,Sltm,Nfrkb,Ect2,Luc7l,Cdc25a,Zfp532,Serbp1,Sbno1,Fanca,Ewsr1,Nasp,Wipf1,Cdc7,Hspa4l,Itgav,Top2a,Sumo2,Ppbp,Pigf,Smchd1,Nde1,Eif4enif1,Usp37,Ncapg,B3galnt1,E330016A19Rik,Cenpk,Whsc2,Me2,E130308A19Rik,Nxt1,Dmap1
**mmu-miR-295***	43(1239)	8,14E-10	Nck2,Ifi205,Ktn1,1110020G09Rik,Dnmt1,Ddx52,Bcl10,Tiam2,Impdh2,Mcm3,Lsm2,Hdac2,Etf1,Agxt2l2,Aftph,Cdca2,Enpp1,Hmgb3,Slc19a1,Gins1,Cbx1,Psmc3ip,Ncapd3,Recql4,Gtf2h2,Prpf4b,Pnpt1,Acsl3,Zfp451,Snrpd1,2810055F11Rik,Neil3,Paip1,Fgf7,Slc38a1,Asf1a,Tnrc6a,Yes1,Plscr1,B3galnt1,Bhlhb9,Arhgap19,Cenpk
**mmu-miR-30e***	56(1239)	8,38E-10	Prpf31,2700050L05Rik,Prim2,Prkar2b,Acly,Spag5,Dsn1,Hmmr,Cenpq,Kif4,Xpo7,Msh2,Ank3,Vrk3,Lcorl,Lsm2,Luc7l,Etf1,Usp34,Gins1,Ccnb2,B230120H23Rik,Ncapd3,Rps9,Troap,H2afy,Ssb,2810474O19Rik,Kin,Mtm1,Ints5,Rbm17,Axl,Patz1,Nudc,Ahcy,Synj2,Cdc40,Mycn,Ociad2,Syne2,Ddx10,Stmn1,Mcm6,Rcl1,Lrrc8c,AI848100,Cdca8,Khdrbs1,C330027C09Rik,4632434I11Rik,Arf6,Cdc73,Gtse1,Mrpl18,Smc4
**mmu-miR-350**	58(1239)	9,58E-10	Asph,Rbm26,Trim24,Ercc6l,Ccbe1,Fhl2,Eef1g,Kif2a,Mum1,Fubp1,Las1l,Pspc1,Srpk1,Dnmt1,Ube2e3,Polr1e,Casp3,Schip1,Prim1,Ppid,Lsm2,Fbxo5,Armc8,Cenph,Msh3,Aftph,Exo1,D1Bwg0212e,Kif21a,Tslp,Gnb4,Rpl12,Ewsr1,Spry2,Sumo2,Snrpg,Rad18,Psmd14,Cenpa,Rsf1,Pvrl3,Slc38a1,Cenpf,Fgd3,Fbln1,Ilf3,Bnc2,Pum2,Stmn1,Mcm6,Arhgap19,Ppwd1,Incenp,C330027C09Rik,Ivns1abp,Tmpo,Ift74,2010204K13Rik
**mmu-miR-141***	47(1239)	1,19E-09	Cenpc1,Fhl2,Eef1g,Mum1,Elk3,Smpdl3b,Prdx4,Ppih,Ncl,Ank3,Impdh2,Ctbp2,Emg1,Setx,Pde1a,E130303B06Rik,Rps9,Recql4,Prpf4b,Nxf1,Ssb,Rad54l,Traip,Ptges3,Smndc1,Smc3,Gins4,F630043A04Rik,Nab2,Clspn,Cenpf,Xpo1,Ilf3,Cnot6,Syne2,Stmn1,C79407,Abl2,Dcbld1,Rfc2,Rpl13a,Ptger4,Apex1,Incenp,Uhrf1,2010204K13Rik,Smyd5
**mmu-miR-152**	61(1239)	1,26E-09	Chd1,Ncaph,Rcc2,Prim2,Whsc1,D19Bwg1357e,Depdc1b,Tcf19,Snx7,Ctsw,Plk1,Aurkb,Dnmt1,Egr2,Ank3,Nuf2,Vrk3,Ppil5,Lrp8,Mtf2,Acaa2,Hdac2,Cand1,Sh3kbp1,Pde1a,Ccnb2,Lbr,Psmc3ip,Rad51c,Zc3h8,Hbs1l,Sgms1,Imp3,Ptpn2,H2-K1,Wipf1,Trim25,Palld,Slco4a1,Usp3,Ccna2,Cdc7,Nfx1,Snrpd1,Nme4,Nmral1,Ctsh,Mtm1,Psmd14,Rsf1,Ptcd3,Sip1,Dut,Tinf2,Foxp1,Mycn,Tnrc6a,Fmnl3,B3galnt1,Dock5,Dnajc9
**mmu-miR-33**	57(1239)	1,33E-09	Gemin4,Arhgap22,Gmnn,Fhl2,Tnnt2,Smpdl3b,Fubp1,Strbp,Ddx27,Ddx46,Ddx52,E2f8,Tiam2,Ahctf1,Casp3,Dhx36,Lcorl,Pim3,Lsm2,Tll1,Myc,Cand1,Cdca2,Sgol1,Zfp184,Dtl,BC031781,Eif4a3,Mapk1,Snrpa1,Ptpn2,Stag1,Rpl12,Gins4,F630043A04Rik,Tk1,Cep70,Nt5c3l,Mpp6,Pvrl3,3110003A17Rik,Cct3,Tipin,Mphosph10,Tpm3,Eif5,Cacna1c,Dapp1,Strap,Umps,Ssrp1,Fancd2,Nol11,Zfp281,Ezh2,Gins3,1810011O10Rik
**mmu-miR-181b**	59(1239)	1,40E-09	Nedd4l,Tgif2,1110012J17Rik,Prkar2b,Rnmt,Mum1,Phactr4,Rapgef2,Aurkb,Pspc1,Ythdf2,Noc4l,Pdia6,Bcl10,Nrf1,Vrk3,Fxn,Schip1,Pdap1,Acaa2,Nfatc2ip,Msh3,Ccdc99,Pcna,Pla2g4a,Gins1,Gpbp1,Npn2,4930547N16Rik,Ccnf,Snrpa1,Hbs1l,Troap,Gtf2h2,H2-K1,Crebzf,Rbm25,Hmgb2,Nfx1,Rad18,Melk,Tpx2,Fgf7,Actl6a,Wdr73,Polr3b,Cdc40,Ilf3,Foxp1,Mboat2,Col7a1,Ptbp2,Cdca8,Phlda1,Me2,Gpsm2,Ppa1,Nxt1,Rbpj

miRNA name	*Number of Genes*	*Corrected p-value*	*miRNA targets among genes repressed in Rasless cells (Table S1)*
**mmu-miR-190b**	51(1239)	1,42E-09	Sap30,Pop1,Rbm26,Ifi205,Steap1,Snx7,Nup37,Has2,Smpdl3b,Atad2,Pgm1,Myef2,Kif18a,Yme1l1,Wsb1,Blm,Tmem173,Eef1e1,Egfr,Pde1a,Kif21a,Shcbp1,Tslp,Rsbn1,Hbs1l,Gtf2h2,Nup107,Lyar,Chek1,Nfx1,Pank4,Melk,Mtm1,Psmd14,Chd2,Smchd1,Cenpn,Pvrl3,Soat1,Synj2,Slco1a5,Cdkn2c,Eif5,Tnrc6a,Actr3,Fignl1,Ppwd1,Pdss1,Hells,Dyrk3,Rbl1
**mmu-miR-128a**	59(1239)	1,62E-09	Pcm1,Dffb,Trim24,Ercc6l,Vegfc,Upf3b,Amd1,Depdc1b,Nol9,Zfp52,Ctsw,Gemin6,Fubp1,Pspc1,1110020G09Rik,Dnmt1,Aurka,Riok2,Ube2e3,Msh2,2510012J08Rik,Fndc4,Rrm2,Cct5,Csf1,Pla2g4a,Pde1a,Casc5,Sgms1,Pnpt1,Nusap1,Ncam1,Bub1b,Cdca4,Nfx1,Mmd,Cep70,Kin,Melk,2810025M15Rik,Mpp6,Pigf,Pvrl3,Ppif,Dut,Cct3,Gnl3,Sertad1,Ruvbl2,Mcm2,Plscr1,B3galnt1,Fbxo32,Ddx39,Nek2,Ppa1,Ezh2,Cebpz,Pogz
**mmu-miR-92b**	62(1239)	1,67E-09	2810046L04Rik,Gemin4,Gata2,Khsrp,Rbm26,Sirt1,H2afv,Errfi1,Birc5,Fhl2,Has2,Dcp1a,Cdc27,Mup4,Nrf1,Ahctf1,Cit,Tgif1,BC055324,Nfatc2ip,Rad21,Asxl3,Eef1e1,Smek1,Usp14,B230120H23Rik,2700029M09Rik,Pcnt,Cul4b,Rsbn1,Ccng2,Mtap,Rpl12,Csnk1g3,Nusap1,Myo1b,Mmd,Tk1,Mbd4,Zcchc2,3110003A17Rik,Slc38a1,Abhd10,Neto2,BC016423,Slco1a5,Foxp1,Eif5,Cep55,Mcm6,Fancd2,Dcbld1,Dtymk,Ppwd1,Atrx,Phlda1,Pcf11,Gpsm2,Ppa1,Cdh10,Dock5,Mrpl18
**mmu-miR-376b**	54(1239)	1,89E-09	Etaa1,D19Bwg1357e,Ifi205,Cks1b,Dkc1,Snx7,Mum1,Gemin6,Dcp1a,Lrig3,Fubp1,Noc4l,Elp2,Pgm1,Snrpb,Msh3,Psrc1,Trip13,Ncapd2,Ctnna1,Trim28,Zc3h8,Polr2b,Ssb,Espl1,Nupl1,Kpna2,Nfkbiz,Rbm25,Itgav,Top2a,2610002M06Rik,Arid4b,Aifm1,Pigf,Sip1,Fgf7,Wdr55,Anln,Tpp2,5730559C18Rik,H2afz,Plscr1,Cnot6,Spp1,Pum2,Mcm6,Gprc5c,Rfc2,Ppwd1,Th1l,Tnfaip6,2010204K13Rik,Ecd
**mmu-miR-124**	62(1239)	1,92E-09	Snx5,Nedd4l,H2afv,Ercc6l,Kif2a,Mum1,Ripk2,Mnd1,Fubp1,Emb,Aurka,Bcl10,Tmem48,Lrp8,Por,Brca1,Cct7,Mcm3,Lsm2,Luc7l,Usp1,Cct2,Sh3kbp1,Psrc1,Ncapg2,Slc29a1,U2af1,Dhodh,D1Bwg0212e,Idh2,Imp3,H2afy,Suclg2,Pnpt1,Ptpn12,Ccna2,Gins4,Nme4,Rad18,Mtm1,Mpp6,Ppif,Abhd10,Kcnk2,Ddx31,Pbk,Ilf3,5730559C18Rik,Ddx10,Gprc5c,Rfc2,Tacc3,Arhgap19,Ptbp2,Thoc2,1700025G04Rik,Incenp,1700054N08Rik,Gart,Wdhd1,Dock5,2010204K13Rik
**mmu-miR-223**	54(1239)	1,98E-09	Sap30,Tmem39b,Prkar2b,Rnmt,Prx,Eef1g,Fus,Spag5,Sass6,Kif4,Tcerg1,Ndc80,E2f8,Nol10,Mcm4,Acaa2,Fndc4,Cdk2,Adamts7,Usp10,Cbfb,Myc,Usp34,Slc29a1,Gins1,Cbx1,Usp14,Rad51c,Arl4c,Spc25,Acsl3,Tial1,Hyal2,Nxf1,Asf1b,Rasa1,Bub1b,Arid4b,Mtm1,Pkp2,Cachd1,Ddx11,3110003A17Rik,Slc38a1,BC016423,Wdr73,Pum2,Stmn1,Ptbp2,Rpl13a,Pola2,Tnfaip6,Tpbg,Pogz
**mmu-miR-329**	56(1239)	2,01E-09	6430527G18Rik,Asph,Pcm1,H2afv,Impa2,D19Bwg1357e,Mum1,Plk1,Gemin6,Rangap1,Rapgef2,Ncl,Rbbp7,Cct7,Lsm2,Nup93,Tuba1c,Cct5,Gpd2,Asxl3,Aftph,Cep57,Ung,Cbx1,Brd8,Gpbp1,Peg12,Lig1,Ncapd3,Kif21a,Atad5,H2afy,Prpf4b,Metap1,Kpna2,Gins4,4930579G24Rik,Nmral1,Pkp2,Limd2,Ss18,Fbln1,Wdr73,Cdc40,Cnot6,E2f7,Akap8,Rfc2,Stil,Ptbp2,Mast4,Nek2,Cdca8,1700054N08Rik,Cdh10,Tpbg
**mmu-miR-467b**	64(1239)	2,27E-09	Mtmr4,Ankrd32,D19Bwg1357e,Plk4,Spag5,Cenpq,Atad2,Mrps22,1110020G09Rik,Ddx52,Mup4,Egr3,Vrk3,Prim1,Tubb5,Suv39h1,Hdac2,Dbr1,Cacybp,Zfp184,Ccdc99,Pik3c2a,Larp7,Cbx1,Thsd7a,Cep152,Casp2,Rad51c,Gtf2h2,Pola1,Nxf1,Trim25,Wdr75,Snw1,Uchl5,Pif1,Hmgb2,Itgav,Nr2f2,Rfc5,Aifm1,Hat1,Ube2t,Smchd1,3110003A17Rik,Sox4,Cenpf,Nucks1,Actl6a,Asf1a,2410016O06Rik,Cdc40,Mastl,Syne2,Stil,Mast4,Twistnb,Pcf11,Ccdc18,4632434I11Rik,Nsl1,Cenpi,Cdt1,AA408296
**mmu-miR-129-5p**	54(1239)	2,72E-09	6430527G18Rik,Lrrc45,Rcc2,Prkar2b,Trim24,Rbmxrt,Marcksl1,Ifi205,Spag5,1110020G09Rik,Exosc8,Rbbp7,Egr3,Fkbp5,2510012J08Rik,Fbxo5,BC027072,Rrm1,Setx,Pkn3,Abcb1b,Lig1,E130303B06Rik,Zc3h8,Pcnt,Recql4,Gtf2h2,Sbno1,Acsl3,Prpf38a,Hyal2,Rasa1,Itih2,Prpf3,Paip1,Egr1,2810025M15Rik,Igsf3,Mpp6,Fgf7,Cct3,Syne2,Bhlhb9,Sdpr,Tacc3,Zcchc8,Tnfaip6,Gart,Ppa1,Nxt1,Smyd5,Ecd,Tcf4,Smc4
**mmu-miR-878-5p**	55(1239)	2,96E-09	Timeless,Upf3b,Zfp52,Acly,Sass6,2610101N10Rik,Srpk1,Elp2,Pgm1,Tiam2,Cit,Epc2,Fbxo5,Enpp1,Psrc1,Pla2g4a,Gins1,Ncapd2,Cep152,Gpbp1,B230120H23Rik,Baz1a,Pcnt,Rpl12,Ssb,Larp4,Cdca4,Mns1,Nfx1,Snrpg,Rad18,Mpp6,Smchd1,Ints7,Casp8ap2,Bbs12,Esco2,Pbk,Stt3b,Synj2,Nsmce4a,Gas2l3,Mastl,Ddx20,Mboat2,Syne2,Stmn1,Thoc2,Gpsm2,Ppa1,Tmpo,Nxt1,Cdh10,Rbpj,Smarcc1
**mmu-miR-153**	56(1239)	3,37E-09	Ankrd50,Nedd4l,Prkar2b,Rbmxrt,Birc5,Dhx9,Taf5,Pole2,Has2,Pspc1,Kif11,Dnmt1,E2f8,Rest,Lrp8,Mtf2,Dhx36,Snrpb,Sfpq,Armc8,Cep57,Ctbp2,Rpl30,Pla2g4a,Larp7,Ncapg2,Zfp217,Shcbp1,Cul4b,Casp8,Zfp451,Chaf1b,Nupl1,Kpna2,Rasa1,Mns1,Pprc1,Cep70,Pkp2,Ckap4,Pigf,Limd2,Abhd10,Ptrh2,Slc39a10,Slco1a5,Rrad,Ppwd1,Map4k5,Sf3a3,Cdca8,Smu1,Hells,Myct1,E130308A19Rik,Ltbp1
**mmu-miR-467a***	44(1239)	3,41E-09	Etaa1,Prim2,Cenpc1,Mum1,Aurkb,1110020G09Rik,Aurka,Aspm,Utp18,Schip1,Glul,Chuk,Npn2,Set,Satb2,Gtf2h2,Sbno1,Depdc1a,Ewsr1,Ssb,Zfp54,Pttg1,Rbm25,Npm1,Hmgb2,Ckap2,Itih2,Paip1,Casp8ap2,Anln,Il1rap,Smc2,Topbp1,Nsmce4a,Cep55,Slc43a3,Ddx10,Fancd2,Rfc2,Col7a1,Thoc2,Pcf11,Cenpi,Kif23
**mmu-miR-384-5p**	52(1239)	3,42E-09	Etaa1,Chd1,Sap30,Diap3,Prim2,Errfi1,Rnmt,Dhx9,Bclaf1,Plk4,Cenpq,Ppih,Atad2,Mnd1,Emb,Noc4l,Cep192,Tcerg1,Msh2,Msh6,Ppid,Dbr1,Hspa14,Cand1,Cbx1,Usp14,Smc5,Sbno1,Wdr75,Cdca4,Dbf4,Unc5c,Zcchc2,Ube2t,Pvrl3,Nudt21,2410042D21Rik,Actl6a,Synj2,Cct8,Cnot6,Rrad,Esf1,Rfpl4,Dcbld1,Cenpk,2610027L16Rik,Tex10,4632434I11Rik,Gpsm2,Ppa1,Terf1
**mmu-miR-23a**	57(1239)	3,56E-09	Sirt1,Marcksl1,Ankrd32,Skp2,Ktn1,Elf2,Mum1,Snapc3,Dctd,Ddx46,Ddx52,Pdia6,Aspm,6720463M24Rik,Vrk3,Zw10,Cpsf4,Tgif1,Rnps1,BC055324,Snrpb,Fbxo5,Cenph,Nudt1,Cand1,Ccdc99,Dusp5,Gpbp1,H2-K1,Crebzf,Lrrk1,Rpl12,Dhx15,Espl1,Kpna2,Ncaph2,E330009J07Rik,Hmgb2,Nt5c3l,Pank4,Pigf,Ube2t,Ppif,3110003A17Rik,Zbtb12,Tpm3,Gas2l3,Mboat2,Dcbld1,Ppwd1,Trub1,Snrpb2,Gmps,H2afx,Zfp281,Tmpo,Mrpl18
**mmu-miR-292-3p**	61(1239)	3,81E-09	Nedd4l,Diap3,Ap1s3,Atad2,Mrps22,2010002N04Rik,1110020G09Rik,Ddx46,Ddx52,Egr2,Egr3,Ilf2,Vrk3,Gspt1,Nt5dc2,Mrps6,Sin3a,Tubb5,Mcm3,Sfpq,Rrm2,Nudt1,Ncapd2,Pdk3,Pkn3,Cep152,Dusp6,Ccng2,Gtf2h2,Ppp1cc,Depdc1a,Sf3a1,Spry2,Usp3,Pif1,Nr2f2,Snrpd1,Arid4b,Tpx2,Ints5,Cenpn,Sip1,3110003A17Rik,Fbln1,Cdc40,Suv39h2,Ddx20,Osmr,Tpp2,Plekha5,Mboat2,H2afz,Syne2,Mcm6,Dcbld1,Eif4h,Khdrbs1,Nup43,4632434I11Rik,Ezh2,Cenpi
**mmu-miR-150***	47(1239)	3,81E-09	Nck2,H2afv,Rbmxrt,Prx,Wdr43,Fosl1,Ogfrl1,Sass6,Tes,Pnn,Il13ra1,Por,Lsm2,Fbxo5,Cct5,Myc,Agxt2l2,Enpp1,Pcna,Emg1,Ncapd2,Cbx1,Micall2,Crlf1,Snrpa1,Depdc1a,Wipf1,Nusap1,Wdr75,Nupl1,Sema4b,Prpf3,Pank4,Limd2,Gphn,Eif4enif1,Gnl3,Zrsr2,Prc1,Ssrp1,Slc43a3,Ddx10,Rcl1,Th1l,Cnn3,Ezh2,Smyd5
**mmu-miR-377**	51(1239)	3,97E-09	Ranbp1,Prx,Depdc1b,Kif2a,Gemin6,Fubp1,Noc4l,Tcerg1,Egr2,Ahctf1,Kif18a,Sltm,Dhx36,Tmem173,Armc8,Hspa14,Sgol1,Ung,Pla2g4a,Cep152,Ncapd3,Casp8,Tial1,Espl1,Larp4,Snw1,Rasa1,Hmga2,Cdca4,Nt5c3l,Egr1,Trit1,Psmd14,Fzd3,Cenpe,Rad51ap1,Foxp1,Anp32b,Yes1,Pum2,Dcbld1,Rfc2,Rcl1,Whsc2,Pcf11,Egfl7,Tmpo,Cdt1,Mrpl18,Hnrpdl,Socs4
**mmu-miR-107**	56(1239)	4,22E-09	Chd1,Lrrc45,Prim2,Mtmr4,Rnmt,Fhl2,Taf5,Wdr43,Ccne1,Sass6,Mapk8,Ddx46,Aurka,Pdia6,Parp1,Tox,Gspt1,Slc11a2,Mrps6,Taf5l,Nfrkb,Nfyb,Cct5,Armc8,Exo1,Ctbp2,Cep152,Shcbp1,Cul4b,Mtap,Ssb,Nupl1,Ncaph2,Ptk7,Nmral1,Hat1,Axl,Wdr55,Tinf2,Patz1,Mcm7,Foxp1,Eif5,Tpp2,Slc43a1,Pafah1b3,Mcm6,Rfpl4,Psmd5,Vbp1,Bcl2l11,4632434I11Rik,Rbm12,Cdc73,Ahcyl1,Gins3
**mmu-miR-467e***	43(1239)	4,32E-09	Etaa1,Prim2,Cenpc1,Mum1,1110020G09Rik,Aurka,Aspm,Utp18,Schip1,Ppid,Glul,Eme1,Npn2,Set,Satb2,Gtf2h2,Depdc1a,Ewsr1,Ssb,Zfp54,Pttg1,Rbm25,Npm1,Ckap2,Itih2,Paip1,G3bp2,Anln,Mphosph10,Il1rap,Smc2,Topbp1,Nsmce4a,Slc43a3,Ddx10,Fancd2,Rfc2,Col7a1,Thoc2,Pcf11,Cenpi,2010204K13Rik,Kif23

miRNA name	*Number of Genes*	*Corrected p-value*	*miRNA targets among genes repressed in Rasless cells (Table S1)*
**mmu-miR-126-5p**	50(1239)	4,65E-09	Sap30,Asph,Rbm26,Cenpc1,Kif2a,Zfp52,Pole2,Lrig3,Atad2,Pank1,Las1l,Pdgfb,Nrf1,Fndc4,Taf5l,Fnbp1l,Dbr1,Setx,Eme1,Slc25a5,Usp14,Snrpa1,2310057M21Rik,Gtf2h2,Sbno1,H2-K1,Pnpt1,Tube1,Usp3,Kpna2,Nfkbiz,Npm1,Neil3,Paip1,Hat1,2810025M15Rik,Rsf1,Fgf7,Casp6,Tbx3,BC016423,Sertad1,Ncapg,Rif1,Strap,D16Ertd472e,Psmd5,Ccdc18,Terf1,Nxt1
**mmu-miR-452**	52(1239)	4,82E-09	Tnfaip8,Dffb,Ercc6l,Mcm5,Hspa8,Fus,Ppih,Kif11,1110020G09Rik,Elp2,Aspm,Ruvbl1,Nrp1,Mrps6,Myc,Cep57,Sgol1,Zfp184,Pcna,Usp34,Pde1a,1700029F09Rik,Dhodh,B230120H23Rik,Pcnt,Polr2b,Gtf2h2,Hyal2,Tube1,Nupl1,Snw1,E330009J07Rik,Bub1b,Top2a,2810055F11Rik,Pkp2,Pigf,Pvrl3,Tmem49,3110003A17Rik,Neto2,Bnc2,Actr3,Cnot6,Spp1,B3galnt1,Abl2,Nob1,AI848100,Dyrk3,Nxt1,Smc4
**mmu-miR-467b***	42(1239)	5,20E-09	Etaa1,Asph,Rbm26,Prim2,Cenpc1,Mum1,Aurka,Aspm,E2f8,Utp18,Schip1,Chuk,Npn2,Satb2,Gtf2h2,Depdc1a,Ewsr1,Acsl3,Ssb,Nupl1,Pttg1,Rbm25,Npm1,Hmgb2,Ckap2,Itih2,Paip1,Il1rap,Smc2,Topbp1,Nsmce4a,Cep55,Spp1,Slc43a3,Ddx10,Fancd2,Rfc2,Col7a1,Thoc2,Pcf11,Cenpi,Kif23
**mmu-miR-871**	50(1239)	5,76E-09	Nup50,Arhgap22,Pcm1,Ankrd32,Kif2a,Prpf19,Cpsf6,2610101N10Rik,Las1l,Cep192,Dnmt1,Pgm1,Riok2,6720463M24Rik,Msh2,Ahctf1,Dhx36,Agxt2l2,Denr,Gins1,1700029F09Rik,Dtl,U2af1,H2-K1,Nup107,Rad54l,Kpna2,Gins4,Rad18,Socs6,4933427D14Rik,Prkg2,Dus3l,Ttc3,Kcnk2,Sertad1,Actr3,Cnot6,Syne2,Ddx10,Trub1,1110004E09Rik,Pola2,Ezh2,Cenpi,Ecd,Kif23,Eif2s1,Tcf4,Socs4
**mmu-miR-383**	57(1239)	6,25E-09	Fmr1,Ncaph,Sap30,Pcm1,Pop1,Rbm26,H2afv,Dgcr8,D19Bwg1357e,Ifi205,Fhl2,Depdc1b,Cks1b,Glrx,Nup37,Ncl,Rbbp7,Tmem176b,Ank3,Klhdc2,Agxt2l2,Zfp184,Usp34,Slc29a1,Pdk3,Trim28,Adss,Runx2,Dhx15,Slbp,Nusap1,F630043A04Rik,Aifm1,Kpnb1,Rbbp8,Psmd14,Hat1,Ckap4,Pvrl3,Ints7,Dut,Gphn,Tipin,Nsmce4a,Cdkn2c,Ssrp1,Nav3,Tacc3,Cenpk,Pola2,Me2,Rad51,Arf6,Uhrf1,Gtse1,Ecd,Eps8
**mmu-miR-21**	50(1239)	6,39E-09	6430527G18Rik,Phf6,Vegfc,Birc5,Vps36,Ppih,Ccne1,Pspc1,Rest,Msh6,Impdh2,Rnps1,Dbr1,Armc8,Hspa14,Nudt1,Pcna,Denr,Psrc1,Cd2ap,Gins1,Ccrn4l,Ncapd3,Snrpa1,Satb2,Ptpn2,Pnpt1,Mtap,Rbm25,Npm1,Itgav,Neil3,Rpa2,Rbm17,Pvrl3,Tmem49,Rbmx2,Tipin,Mlf1ip,Esco2,Rif1,H2afz,Plscr1,Sdpr,Dtymk,AI848100,Pou2f1,E130308A19Rik,Sec63,Ecd
**mmu-miR-409-3p**	54(1239)	6,57E-09	Asph,Tmem39b,Rbm26,Diap3,Mcm5,Kif2a,Mybbp1a,Smpdl3b,2810008M24Rik,Aurkb,Cep192,Rpl7a,Pnn,Ilf2,Ctcf,Cit,Rhobtb3,Msh6,Schip1,Snrpb,Cand1,Zfp184,Gjc1,Gpbp1,Spc25,Pcnt,Pola1,Prpf4b,Ewsr1,Zfp54,Usp3,Gins4,Topors,Hmgb2,Tead2,Slc9a3r1,3110003A17Rik,Xpo1,Zrsr2,2410016O06Rik,Anp32b,Dcbld1,Zcchc8,Nob1,Ppwd1,Ddx39,Utp11l,Pou2f1,Ppa1,Nsl1,Ift74,Tpbg,Ecd,Hnrpdl
**mmu-miR-186**	50(1239)	7,49E-09	Tnfaip8,Rbm26,Trim24,Hspa8,Fus,Cenpo,Elp2,Exosc8,Pgm1,Cdc5l,Ank3,Ebf2,Usp10,Gpd2,Psrc1,Pla2g4a,Trip13,Matr3,Gpbp1,Snrpa1,Pcnt,Idh2,2310057M21Rik,Gtf2h2,Nasp,Ssb,Kpna2,Rbm25,Hmgb2,Ptk7,Aifm1,Trit1,Ckap4,Nup88,Ube2t,Fzd3,Rbmx2,Slc38a1,Zbtb12,Foxp1,Yes1,Bnc2,Cd14,Ppwd1,Pola2,Pcf11,Gtse1,Ahcyl1,Slc4a7,Cirh1a
**mmu-miR-685**	54(1239)	7,62E-09	Abi1,Kif2a,Acly,Mki67,Kif4,Las1l,Srpk1,Elp2,Bub3,Hspe1,Cep68,Hspd1,Mcm4,Ppil5,Schip1,Nfrkb,Ppid,Sgol1,Eme1,Ccnb2,Adss,Baz1a,Otud4,Cul4b,Ppp1cc,Sf3a1,Wipf1,Asf1b,Smndc1,Pttg1,Prpf3,Smchd1,Cdca3,Pvrl3,Prps1,Socs6,Fgd3,Patz1,Wdr73,Nsmce4a,H2afz,E2f7,Mrpl1,Pafah1b3,E330016A19Rik,Mre11a,Gmps,Rad51,Incenp,Zfp281,C330027C09Rik,Gart,Pou2f1,Eps8
**mmu-miR-488***	46(1239)	7,71E-09	Nedd4l,Timm8a1,Ets1,Mybbp1a,Rangap1,Ddx46,Pgm1,Ptpre,E2f8,Ilf2,Ttll4,Acaa2,Tmem173,Snrpb,Luc7l,Kif20a,Chst1,Thsd7a,Baz1a,Sgms1,Gtf2h2,Pnpt1,2210018M11Rik,Nxf1,Rad54l,2810474O19Rik,Sema4b,Npm1,Pprc1,Nfx1,Snrpd1,Fzd3,Wdr55,Rps13,Dus3l,U2af2,Ptrh2,Sertad1,Akap8,Mcm6,Bhlhb9,Mre11a,Thoc2,Nsl1,Rbpj,Wdr36
**mmu-miR-409-5p**	57(1239)	9,06E-09	Ncaph,Depdc1b,Rangap1,Atad2,1110020G09Rik,Noc4l,Cdc27,Riok2,Hspe1,Nrf1,Vrk3,Tuba1c,Sox11,Zic1,Agxt2l2,Zfp184,Enpp1,Eef1e1,Setx,2610039C10Rik,Dusp5,Kif20a,Pkn3,Gjc1,Trim28,Abcb1b,Zfp217,Trim25,Ankrd57,Nusap1,2810474O19Rik,Ipo7,Pprc1,Ptk7,Snrpg,Unc5c,Melk,Pole,Fzd3,Ints7,Fgd3,Prkg2,Nutf2,Nsmce4a,Spc24,Pold1,Pum2,Mcm6,Gprc5c,Tacc3,Psmd5,Thoc2,Pola2,Gpsm2,Nsl1,Ahcyl1,Kif23
**mmu-miR-10a***	46(1239)	9,16E-09	Rbm26,H2afv,Whsc1,Rnmt,Cks1b,Pole2,Spag5,Plk1,Strbp,Wdr3,Srpk1,Cenpj,Riok2,Aspm,Mcm4,Casp3,Luc7l,Dbr1,Tll1,Egfr,Pla2g4a,Larp7,Usp34,Eme1,D1Bwg0212e,Cul4b,Lrrk1,Hmga2,Nr2f2,Trp53bp2,Topbp1,U2af2,Cacna1c,Plscr1,Lmnb1,Akap8,Mcm6,Ppwd1,Atrx,1110004E09Rik,Lifr,Ezh2,Gtse1,Tpbg,Hnrpdl,Pogz
**mmu-miR-143**	54(1239)	9,21E-09	Ncaph,Timeless,Pcm1,Prpf19,Rangap1,Mnd1,Nipsnap1,Wdr3,Cdc27,Ube2e3,Ttll4,BC055324,Hdac2,Msh3,Agxt2l2,Rrm1,Ctbp2,Glul,Emg1,Eme1,Slc25a5,Casc5,Lig1,Zfp217,Hbs1l,Imp3,Hyal2,Kars,Spred1,Smndc1,Npm1,Top2a,Gli3,Sip1,Fgf7,Ppif,Apitd1,Tpm3,Synj2,Pold3,Nav3,Abl2,Fancd2,Rcl1,Rsrc2,Trub1,Ptbp2,Sf3a3,Cdca8,Me2,Wdhd1,Rfc4,Cdh10,Pogz
**mmu-miR-133a***	43(1239)	9,40E-09	Zfp292,Tnpo1,Prx,Gmnn,Zfp334,Vps36,Spred2,Sass6,Cdc27,Tcerg1,6720463M24Rik,Marcks,Ets2,Wsb1,Por,Fbxo5,Rrm1,Lin9,Slc25a5,Cbx1,Mpzl1,Parp12,Hyal2,Kars,Cbx3,Asf1b,Hmgb2,Nfx1,Unc13c,Melk,Net1,Patz1,Neto2,2410016O06Rik,Eif5,Whsc2,Zfp281,Hells,Ccdc18,Myct1,Ift74,Slc4a7,Eif2s1
**mmu-miR-302d**	58(1239)	9,76E-09	Etaa1,Ankrd32,Ktn1,Llgl1,Bclaf1,Plk4,Ctsw,Cenpq,Atad2,Dnmt1,Ddx52,Pdia6,Egr3,Vrk3,Tox,Mrps6,Prim1,Dbr1,Trib1,Larp7,Cbx1,Psmc3ip,Cep152,E130303B06Rik,Idh2,Gtf2h2,Lrrk1,Nxf1,Asf1b,Pttg1,Kpna2,Snw1,Cdca4,Neil3,Rpa2,Wee1,Hn1,Nmral1,Aifm1,Nup88,Ptcd3,Smchd1,Wdr55,Dut,Bbs12,Synj2,Foxp1,Cep55,E330016A19Rik,Rsrc2,Mast4,Pdss1,Hells,Ccdc18,4632434I11Rik,Pou2f1,Cdh10,2810408A11Rik
**mmu-miR-582-5p**	55(1239)	1,02E-08	Prim2,Trim24,Prx,D19Bwg1357e,Depdc1b,Kif2a,Dctd,Ccne1,Atad2,Ddx27,Exosc8,Aurka,Msh2,Tmem176b,Vrk3,Tox,Wsb1,2510012J08Rik,Zw10,Por,Mcm3,Fbxo5,Cct5,Usp34,Eme1,Dusp5,Pcnt,Gpr126,2310057M21Rik,Gtf2h2,Spry2,Neil3,Dbf4,Pank4,Ctsh,Psmd14,Ptcd3,Pvrl3,Eif4enif1,Hmgn1,Topbp1,Ncapg,Eif5,Tnrc6a,Rrad,Dcbld1,1110004E09Rik,Whsc2,Phc2,E130308A19Rik,Tmpo,Top1,Tpbg,Slc4a7,Hnrpdl
**mmu-miR-181a-1***	54(1239)	1,37E-08	Lrrc45,Khsrp,Pcm1,H2afv,Zfp292,Fancm,Whsc1,Ripk2,Fubp1,Nap1l4,Ddx27,Noc4l,Dnmt1,Ruvbl1,D2Wsu81e,Vrk3,Ttll4,Slc11a2,Acaa2,Ect2,Mcm3,Nup93,Ctbp2,Ncapg2,Dusp5,Thsd7a,Dhodh,Lig1,Suz12,Atad5,Prpf4b,Chaf1b,Asxl1,E330009J07Rik,Cdc7,Mmd,Pkp2,Hat1,Rbm17,Tmem49,3110003A17Rik,Lrig1,Tinf2,Nsmce4a,Cdc40,Ddx20,Bnc2,Slc43a3,Mcm6,Mre11a,2610027L16Rik,E130308A19Rik,Wdr36,Dmap1
**mmu-miR-770-5p**	55(1239)	1,44E-08	Nedd4l,Gemin4,Clns1a,Pwp2,Whsc1,Abi1,Ccbe1,Nol9,Ctsw,Smpdl3b,Nipsnap1,Noc4l,Nrf1,Mad2l1,D2Wsu81e,Tmem48,Sltm,Pim3,Smarce1,Enpp1,Ung,Runx2,Lig1,Hbs1l,Imp3,Ptpn2,Pola1,Suclg2,Depdc1a,Nasp,Stoml2,2810474O19Rik,Card10,Tardbp,E330009J07Rik,Mbd4,Prpf3,Nab2,Hat1,2810025M15Rik,Cenpf,Ss18,Eif4enif1,Smc2,Ttc3,Prmt5,Slco1a5,Ilf3,Cacna1c,Pkmyt1,Nav3,Khdrbs1,Egfl7,Cdh10,Ahcyl1
**mmu-miR-543**	52(1239)	1,46E-08	Ankrd50,2810046L04Rik,Sap30,Diap3,Prkar2b,Dhx9,Pak1,Wdr43,Sass6,Kif4,Pspc1,Strbp,1110020G09Rik,Ythdf2,Ddx46,Pdia6,Vrk3,Tox,Utp18,Schip1,Pdap1,Tgif1,Fnbp1l,Ctbp2,Pcna,Pla2g4a,Abcb1b,Mbtps2,Gpbp1,Prpf38b,Snrpa1,Imp3,2810474O19Rik,Npm1,Hmgb2,Neil3,Unc13c,Slc9a3r1,Aifm1,Ptcd3,Topbp1,Actl6a,Wdr73,Cdc40,E2f7,Pafah1b3,1110004E09Rik,Erh,Zfp281,Nsl1,Cenpi,Nxt1
**mmu-miR-338-3p**	58(1239)	1,55E-08	Skp2,Dhx9,Gmnn,Llgl1,Mki67,Cenpq,Gemin6,Aurkb,1110020G09Rik,Aurka,6720463M24Rik,Msh2,Mad2l1,D2Wsu81e,Fkbp5,Cep68,Vrk3,Nrp1,Sgol2,Hspd1,2510012J08Rik,Mcm4,Fxn,D2Ertd750e,Cct5,Msh3,Enpp1,Zmym1,Cep152,Eif4a3,Ssb,Tube1,Cbx3,Pif1,Ckap2,Pank4,Pkp2,Cachd1,Wdr55,Jub,Dus3l,Apitd1,Zrsr2,Tpm3,5730559C18Rik,Syne2,B3galnt1,Stmn1,Gmeb1,Me2,Incenp,1700054N08Rik,Nsl1,Fancc,Cenpi,Nxt1,2010204K13Rik,Eif2s1

miRNA name	***Number of Genes***	***Corrected p-value***	***miRNA targets among genes repressed in Rasless cells (Table S1)***
**mmu-miR-183***	41(1239)	1,58E-08	Tmem39b,Prkar2b,Prx,Dhx9,D19Bwg1357e,Depdc1b,Snx7,Las1l,1110020G09Rik,Ythdf2,Cenpj,Ube2e3,Aspm,Myef2,Sgol2,Sltm,Mthfd1,Nfyb,Aebp2,Abcb1b,Zc3h8,Spc25,Idh2,Ccng2,Gtf2h2,Pnpt1,Tial1,Zfp7,Rasa1,Bub1b,Mns1,2610301G19Rik,Mtm1,Igf2bp1,Anp32b,Pum2,Utp11l,Utp6,Incenp,Hells,Eps8
**mmu-miR-214**	59(1239)	1,58E-08	Cdca7l,Ahsa1,Arhgap22,Rcc2,Trim24,Dgcr8,Mum1,Acly,Dctd,Prpf19,1110020G09Rik,Tpd52,Pdia6,6720463M24Rik,Nrf1,Egr3,Ttll4,Cdca5,Tmem48,Mrps6,Cpsf4,Pdap1,Acaa2,Tubb5,Suv39h1,Cct7,Ppid,Ung,Ncapd2,Pkn3,Trim28,Psmc3ip,B230120H23Rik,Ccnf,Rasa1,Cdc7,Bub1b,Trit1,Plxnb1,Jub,Kif2c,Fgd3,Mphosph10,G3bp1,Ilf3,Mastl,Ch25h,Prc1,Dapp1,Plscr1,Mcm6,Dcbld1,Tacc3,Ddx39,Phc2,Gart,Rbl1,Slc4a7,Eps8
**mmu-miR-140***	52(1239)	1,67E-08	H2afv,D19Bwg1357e,Depdc1b,Nol9,Pak1,Ap1s3,Aurkb,Wdr5,Noc4l,Smc6,Pnn,Vil1,Mad2l1,Nrp1,Sltm,Schip1,Snrpb,Rab15,Psrc1,Zmym1,Slc19a1,Gins1,2610039C10Rik,Ncapd2,Trim28,Scrib,Ncapd3,Troap,Imp3,Rpl12,Spred1,Pttg1,Cdc7,Nr2f2,Ckap2,Mbd4,Wee1,Unc5c,Pank4,Trit1,2810025M15Rik,Slco1a5,Dapp1,Ociad2,Nav3,Gprc5c,Dcbld1,Trub1,Mast4,Rad51,Hnrpdl,1810011O10Rik
**mmu-miR-483**	54(1239)	1,70E-08	Tgif2,Timeless,Cenpc1,Prkar2b,Abi1,Prx,Gmnn,Nol9,9030617O03Rik,Mki67,Ppih,Atad2,Kif4,Las1l,Mup4,Egr3,Acaa2,Tubb5,Fen1,Snrpb,Gpd2,Ncapd2,Ccnf,Hn1l,Rad51c,Ube2c,Spc25,X99384,H2-K1,Ssb,Neil3,Itih2,Adsl,Wee1,Prpf3,Hn1,Melk,Ptcd3,Cenpn,Slc38a1,Wdr73,Sertad1,Pafah1b3,Rcl1,Tacc3,2610027L16Rik,Cnn3,Utp11l,Timp1,Incenp,2010204K13Rik,2810408A11Rik,Dlx1,Pogz
**mmu-miR-706**	61(1239)	1,79E-08	Thap2,Asph,Tmem39b,H2afv,Zfp292,Cenpc1,Trim24,Birc5,Kif2a,Atad2,Dck,Kl,Aspm,Vil1,Nrf1,Cdc5l,Nol10,Ppil5,Rrm2,Tuba1c,Trim27,Dhodh,Ankrd10,Six4,Rad51c,Crebzf,2810474O19Rik,Kpna2,Rbm25,Sema4b,Itgav,F630043A04Rik,Ebna1bp2,Melk,Igsf3,Sip1,Ints7,Isg20l2,Clspn,Patz1,Prkg2,Dus3l,Nucks1,Cenpl,Ddx20,Cacna1c,Zfp422,Rrad,Nup133,Akap8,Pafah1b3,Alkbh1,Stil,Tnfaip6,Incenp,C330027C09Rik,Ccdc18,Nsl1,Tfam,2010204K13Rik,Kif23
**mmu-miR-380-5p**	51(1239)	1,79E-08	Zfp292,Prkar2b,Marcksl1,D19Bwg1357e,Mki67,Ccne1,Rpl7a,Cenpj,Riok2,2510012J08Rik,Tuba1c,Myc,Sh3kbp1,Pdk3,Slc25a5,Ctnna1,Adss,Gtf2h2,Ppp1cc,Rpl12,Gins4,Mns1,Top2a,Tk1,Unc13c,Mtm1,2810025M15Rik,Pvrl3,Xpo1,Mybl2,Gas2l3,Eif5,Rrad,Nav3,Lmnb1,Ell2,Esf1,Trub1,Th1l,Psmd5,Thoc2,Pola2,Utp11l,Cd300lb,Hells,C330027C09Rik,Myct1,Ppa1,Cebpz,Cdh10,Dock5
**mmu-miR-142-3p**	53(1239)	1,84E-08	Pcm1,Prx,Gmnn,Tnnt2,Smpdl3b,Lrig3,Mnd1,Cenpj,Aspm,Msh2,Marcks,Ilf2,Rest,Ank3,Wsb1,Zw10,Msh6,Lcorl,Cenph,Rab15,Agxt2l2,Cdca2,Zfp184,Egfr,Emg1,Pla2g4a,Ctnna1,Zfp217,Idh2,Lrrk1,Ewsr1,Palld,Hmgb1,Hspa4l,Itgav,Eno3,Paip1,2810025M15Rik,3110003A17Rik,Trp53bp2,Patz1,Pbk,BC016423,Mastl,Ch25h,Usp37,Cct8,Rfpl4,Cxadr,Hirip3,AI848100,Zfp281,Tpbg
**mmu-miR-342-3p**	54(1239)	1,84E-08	Etaa1,Nck2,1110012J17Rik,Timeless,Prpf31,Trim24,Ranbp1,Rnmt,Depdc1b,Mum1,Ucp2,Plk1,Ppih,Ccne1,Kl,Fubp1,Aspm,Tmem48,Nudt1,Agxt2l2,Pkn3,Lig1,Arl4c,Casp8,Sf3a1,Stoml2,Kars,Zfp7,Spry2,Rasa1,Hmga2,Itgav,Rpa2,Nt5c3l,Pkp2,Psmd14,Nab2,Socs6,Plekhh2,Patz1,Tbx3,Ttc3,Nudc,Sertad1,Ilf3,Rfc2,Mrpl50,Whsc2,Pcf11,Myct1,Egfl7,Mrpl18,Dnajc9,AA408296
**mmu-miR-15a***	46(1239)	2,04E-08	Fmr1,Nedd4l,2810046L04Rik,Prx,Tes,Paxip1,Wdr3,Bub3,Mup4,Egr2,Tgif1,BC055324,Lsm2,Klhdc2,Agxt2l2,Enpp1,Slc29a1,Ncapd2,Pkn3,Ccnb2,Casc5,Ncapd3,Kif21a,Casp8,Vrk1,Stoml2,Zfp451,Dhx15,Fbl,E330009J07Rik,Cenpa,Hat1,Fgd3,Lrig1,Nsmce4a,Prc1,Plscr1,Slc43a1,E2f7,Rrad,Ell2,Hirip3,Ddx39,Gmeb1,Hells,Eftud2
**mmu-miR-155**	52(1239)	2,44E-08	Rbm26,Nup54,Prkar2b,4930422G04Rik,Gmnn,Elf2,Steap1,Pak1,1110020G09Rik,Cep192,Ccdc45,Pgm1,Mcm4,Snrpb,Etf1,Cand1,Lin9,Trip13,Cbx1,Pkn3,Zfp217,Rsbn1,Gtf2h2,Ptpn2,Stag1,Usp3,2810055F11Rik,Wee1,2610002M06Rik,Dek,Ankrd28,Fgf7,Ints7,Patz1,Ghr,2410016O06Rik,Foxp1,Mcm2,Bnc2,Cep55,Rfpl4,Ly75,Dcbld1,Bcl2l11,Me2,Ppa1,Rbl1,Rbm12,Cdc73,Terf1,Uhrf1,1810011O10Rik
**mmu-miR-216a**	55(1239)	2,56E-08	2810046L04Rik,Dffb,Ell,Trim24,Whsc1,Fhl2,Mybbp1a,Cenpq,Noc4l,Cenpj,Dnmt1,Ddx52,Parp1,Zc3h15,Mrps6,Schip1,Cct7,D2Ertd750e,Luc7l,Dbr1,Etf1,Gpd2,Smarce1,Larp7,Pde1a,Dusp5,Kif20a,Ctnna1,Adss,Prpf38b,Ncapd3,Baz1a,Sgms1,Cobll1,Nupl1,Hmgb2,Phf17,1110034A24Rik,Pprc1,Nmral1,Ppbp,Ptplad1,Wdr55,Rbmx2,Clspn,Dus3l,Ttc3,Prc1,Syne2,Dtymk,Bcl2l11,Sf3a3,Ltbp1,Tpbg,Kif23
**mmu-miR-124***	48(1239)	2,75E-08	Gmnn,Gemin6,Sass6,Pank1,Kif4,Setdb2,Strbp,Msh2,Nrf1,Sltm,Lcorl,Brca1,Larp7,Bzw2,Smc5,Pcnt,Tslp,H2afy,Stag1,Ewsr1,Tial1,Asf1b,Nupl1,Cdc7,Gins4,Neil3,Aifm1,Pkp2,Magoh,Rbm17,Fzd3,Dut,Cct3,Prmt5,Neto2,Wdr73,Prc1,Plekha5,Rfpl4,Nob1,Ddx39,Timp1,Gmeb1,C330027C09Rik,Myct1,Cdca7,Ppa1,Eftud2
**mmu-miR-709**	65(1239)	2,87E-08	Thap2,Asph,Arhgap22,Zfp292,Cenpc1,Mcm5,Eef1g,Ucp2,Atad2,Rapgef2,Noc4l,Riok2,Aspm,Nrf1,Nuf2,Sgol2,Nol10,Tubb5,Tuba1c,Luc7l,Larp7,Trim27,2610039C10Rik,Micall2,Chst1,Supt16h,Casc5,Rad51c,Pcnt,Gsg2,Tial1,Traip,2810474O19Rik,Ncaph2,Bub1b,1110034A24Rik,Nfx1,F630043A04Rik,Nt5c3l,Ebna1bp2,Mtm1,Nab2,Ptplad1,Cenpn,Sip1,Ints7,Isg20l2,Kif2c,Soat1,Patz1,Dus3l,Nucks1,Ilf3,Usp37,Ddx20,Akap8,Pafah1b3,Alkbh1,Trub1,Cnn3,Tnfaip6,Cdca7,Nsl1,E130308A19Rik,Kif23
**mmu-miR-300***	43(1239)	3,14E-08	Ahsa1,Impa2,Crim1,Cenpq,1110020G09Rik,Rpl7a,Dnmt1,Aspm,Mup4,Cdc5l,Impdh2,Ppid,Nfyb,Trim28,Lig1,Casp2,Pnpt1,Prpf38a,Palld,Nfkbiz,E330009J07Rik,Itgav,Wee1,Aifm1,Melk,Ctsh,Trit1,Tpx2,Cenpa,Ints5,Pvrl3,Wdr73,Tnks2,Ddx20,Lmnb1,Dtymk,Cep170,Ppwd1,4632434I11Rik,Pou2f1,Cdt1,Siva1,Ecd
**mmu-miR-653**	48(1239)	3,37E-08	Nono,Ercc6l,Rbmxrt,Nol9,Glrx,Kif2a,Snx7,Zfp52,Sass6,2610101N10Rik,Cep192,Pgm1,6720463M24Rik,Ahctf1,Schip1,Klhdc2,Msh3,Ssr4,Pla2g4a,Larp7,Usp34,Ncapd2,Bzw2,Dusp6,Baz1a,Pnpt1,Sf1,Spry2,Itgav,Itih2,Sumo2,Paip1,Rsf1,Pigf,Dut,Mphosph10,Topbp1,Pbk,Actr3,Rrad,Nav3,Lmnb1,C79407,Dcbld1,Lrrc8c,Zfp281,Gtse1,Rrs1
**mmu-miR-105**	54(1239)	3,38E-08	Nedd4l,Shmt1,Lrrc45,Cenpc1,Ankrd32,Dkc1,Ctsw,Cenpq,Mnd1,5730590G19Rik,Vrk3,Tox,Nol10,Ppil5,Taf5l,Rrm2,Tuba1c,Klhdc2,Ash2l,Cep152,Zfp217,Idh2,Casp8,Sf3a1,Ncam1,Csnk1a1,Usp3,Nfkbiz,Colec12,Cdca4,Neil3,Rpa2,Cep70,Pank4,Mtm1,Chd2,Cenpn,Cdca3,Igf2bp1,Dut,Tinf2,Hmgn1,Fbln1,Synj2,Spp1,Spc24,Rsrc2,Pcf11,Nup43,Ccdc18,4632434I11Rik,Pou2f1,Fancb,Top1
**mmu-miR-872***	40(1239)	3,91E-08	Gemin4,1110012J17Rik,Diap3,Dhx9,Spag5,Rest,Tox,Sltm,Blm,Dbr1,Rpl30,Zmym1,Emg1,Pla2g4a,Smek1,Bzw2,Sdad1,Zc3h8,Cul4b,Hbs1l,Hyal2,Ssb,Dhx15,Ncaph2,Bub1b,Npm1,Nfx1,Syt17,2610002M06Rik,Ube2t,4930427A07Rik,Topbp1,Sertad1,Slc43a3,B3galnt1,Cd14,Mast4,1700025G04Rik,Incenp,E130308A19Rik
**mmu-miR-193**	52(1239)	4,31E-08	Nck2,6430527G18Rik,Timm8a1,Tmem39b,Marcksl1,Impa2,Whsc1,Nsun2,Llgl1,Dkc1,Kif2a,Nrf1,Vrk3,Socs3,Zw10,Pdap1,Acaa2,Mthfd1,D2Ertd750e,Nup93,Tll1,Sgol1,Ccdc99,Cct2,2610039C10Rik,Trim28,Casc5,Zc3h8,Pcnt,Sbk1,Cbx3,Usp3,1110034A24Rik,Top2a,Tk1,Mcpt8,Exosc2,Clspn,Gemin8,Prkg2,Dus3l,Ilf3,Prc1,Rif1,Mycn,Bnc2,Cnot6,Rrad,Stmn1,Abl2,H2afx,Rbl1
**mmu-miR-34b-5p**	57(1239)	4,42E-08	Pcm1,Mtmr4,Gmnn,Ktn1,Prpf40a,Snx7,Tnnt2,Ctsw,Ythdf2,Exosc8,Mad2l1,Msh6,Acaa2,Tuba1c,Zik1,Slc29a1,Cep152,B230120H23Rik,Bzw2,Ncapd3,Ptpn12,Snw1,Eno3,Nme4,Nt5c3l,Paip1,Pigf,Igf2bp1,Ppif,Gemin8,Apitd1,Ahcy,Actl6a,Nsmce4a,Rbm14,Usp37,Foxp1,Anp32b,Ssrp1,Slc43a3,Nav3,Lmnb1,Ell2,Fmnl3,Rfc2,Litaf,Dtymk,Arhgap19,Ppwd1,Rpl13a,Timp1,Cdca8,Zfp281,Hells,Ccdc18,Ezh2,2810408A11Rik
**mmu-miR-294***	42(1239)	4,58E-08	Ktn1,Prpf40a,2610101N10Rik,Cep192,Als2cr12,Parp1,Sltm,Cpsf4,Rnps1,Snrpb,Cenph,Msh3,2700029M09Rik,Rad51c,Smc5,Snrpa1,Gtf2h2,Pola1,Acsl3,Nasp,Hyal2,Ptges3,Nfkbiz,Snrpd1,2810055F11Rik,Paip1,Mtm1,Hat1,Ptcd3,Axl,Limd2,Dus3l,Zbtb12,Cdc40,Foxp1,Anp32b,Ddx10,6330503K22Rik,Thoc2,Rad51,Zfp281,Myct1

miRNA name	*Number of Genes*	*Corrected p-value*	*miRNA targets among genes repressed in Rasless cells (Table S1)*
**mmu-miR-496**	45(1239)	4,83E-08	Amd1,Vps36,Hmmr,Dnmt1,Ddx52,Nrf1,E2f8,Tiam2,Ahctf1,Msh6,Dbr1,Klhdc2,Zfp36l2,Pla2g4a,Pkn3,Ctnna1,Psmc3ip,Cep152,Casc5,Ncapd3,Shcbp1,Cul4b,Cobll1,Spred1,Wdr75,Usp3,Gins4,2810055F11Rik,Neil3,Snrpg,Psmd14,Hat1,Clspn,Patz1,Esco2,Synj2,Cdkn2c,Suv39h2,Impa1,Anp32b,Mboat2,Nav3,E330016A19Rik,Etv4,Gtse1
**mmu-miR-421**	50(1239)	5,14E-08	Ahsa1,Asph,Gemin4,Dnaja2,Upf3b,Gmnn,Fhl2,Hmmr,Cenpq,Ccne1,Cep192,Tox,Wsb1,Sltm,Tubb5,Mthfd1,Rnps1,Mcm3,Cct5,Ung,Smek1,Ncapg2,Matr3,Dtl,Lig1,Casp2,Spc25,Cul4b,Ptpn2,Ppp1cc,Depdc1a,Wdr75,Nfkbiz,Snrpd1,Arid4b,Fzd3,Wdr55,Cenpf,Mphosph10,Synj2,Rbm14,Plekha5,Ddx10,Bhlhb9,Dcbld1,Tacc3,4632434I11Rik,Nxt1,Gtse1,Hnrpdl
**mmu-miR-196a**	57(1239)	5,46E-08	Etaa1,Rbm26,Rcc2,Elf2,Zfp606,Ccne1,Noc4l,Rdx,Rbbp7,Aspm,Myef2,Parp1,Socs3,Ttll4,Tmem48,Casp3,Schip1,Ebf2,Myc,Psrc1,Egfr,Eme1,Dusp5,Casc5,Scrib,Ncapd3,Rpl12,Nxf1,Lyar,Espl1,Ier2,Snw1,Ncaph2,Prpf3,Nme4,Nt5c3l,Paip1,Trp53bp2,Zfp619,Kif22,Actl6a,Wdr73,Tnks2,Cdkn2c,Prc1,Dapp1,Slc43a3,D16Ertd472e,Tacc3,Rasal2,Ptger4,Timp1,Ezh2,Mrpl18,Gins3,Cirh1a,Pogz
**mmu-miR-466e-5p**	63(1239)	6,10E-08	Prim2,Ell,Birc5,Ankrd32,Dhx9,Steap1,Mum1,Ctsw,Phactr4,Elavl1,Ddx27,Rbbp7,Aspm,E2f8,Ank3,Kif18a,Mtf2,Lcorl,Nfatc2ip,Cep57,Lin9,Zfp184,Rpl30,Pik3c2a,Pla2g4a,Usp34,1700029F09Rik,Gjc1,Kntc1,Ube2c,Kif21a,Gtf2h2,Sbk1,Nupl1,Nfx1,Neil3,Sumo2,Snrpg,Aifm1,Mtm1,Pvrl3,Casp6,Kcnk2,Mlf1ip,Tnks2,Ch25h,Usp37,Prc1,Bnc2,Cep55,Plscr1,Slc43a1,Rrad,Mcm6,Specc1,Cenpk,Tex10,Cdca8,Gmeb1,Tmpo,Ltbp1,Eps8,Tdrkh
**mmu-miR-136***	45(1239)	6,26E-08	Nck2,Tnfaip8,Lrrc45,Rbm26,Cbx5,Fhl2,Depdc1b,F2rl1,Prpf19,Ythdf2,Mad2l1,Zc3h15,Ttll4,Slc11a2,Zw10,Ddx18,Sox11,Nudt1,Cdca2,Cbx1,Thsd7a,Gpbp1,4930547N16Rik,Casc5,Crlf1,Kif21a,Cul4b,Hbs1l,Troap,Pttg1,Nfkbiz,Cachd1,Chd2,Magoh,Rsf1,Fgf7,Cct3,Tinf2,B3galnt1,Mre11a,Timp1,Khdrbs1,4632434I11Rik,Rbpj,Hnrpdl
**mmu-miR-682**	53(1239)	6,41E-08	Ahsa1,F2rl1,Kif2a,Ppih,Elp2,Exosc8,Bub3,Cpsf4,Nup93,Hdac2,Armc8,Etf1,Psip1,Nudt1,Lin9,Ctbp2,Cd2ap,Emg1,Pde1a,Trip13,2610039C10Rik,Pkn3,Dhodh,Scrib,E130303B06Rik,Recql4,Ccng2,Rad54l,Tube1,Mcm10,Asf1b,Ncam1,Usp3,Rfc5,Paip1,Pigf,Cenpn,Exosc2,Sertad1,Cacna1c,Plekha5,Ssrp1,Fancd2,Nob1,Trub1,Map4k5,Ddx39,Rad51,4632434I11Rik,Ppa1,Tpbg,Kif23,Smc4
**mmu-miR-222**	50(1239)	6,41E-08	Sap30,Pcm1,Dffb,Impa2,Dhx9,Upf3b,Hspa8,Tcf19,Zfp606,Las1l,Noc4l,Dnmt1,Ddx52,Tmem176b,Nrf1,Tox,Sgol2,Nfyb,Pgk1,Asxl3,BC027072,Ccdc99,Idh2,Recql4,Gtf2h2,Rad54l,Usp3,Gins4,Npm1,Cep70,Qars,Cdca3,Jub,Pbk,Nsmce4a,Pkmyt1,Stmn1,Abl2,Fancd2,Dtymk,Fignl1,Cenpk,Th1l,2610027L16Rik,Lifr,Cdca8,Ccdc18,Gtse1,Tpbg,Pogz
**mmu-miR-547**	50(1239)	6,41E-08	Pcm1,Rbm26,Prpf40a,Depdc1b,Lrig3,Tes,Ythdf2,Pnn,Ube2e3,Nuf2,Mtf2,Ppid,Zic1,Myc,Lin9,Emg1,Zmynd19,Pde1a,Ncapd2,Pdk3,Zfp217,Depdc1a,Prpf38a,Bcl7a,Nfkbiz,Rbm25,2810055F11Rik,Prpf3,Unc13c,Nme4,Runx1,Pank4,2810025M15Rik,Mpp6,Axl,Ube2t,Cenpn,Anln,Dus3l,Kcnk2,Gnl3,Sertad1,Cdc40,Cct8,6330503K22Rik,Bhlhb9,Trub1,Wdhd1,Wdr36,Eif2s1
**mmu-miR-1**	49(1239)	6,43E-08	Nedd4l,Prim2,Cenpc1,Ktn1,Kif2a,Fubp1,Kif11,Cep192,Elp2,Dnmt1,Pgm1,Aspm,Cdc5l,Myef2,Ctcf,Dis3,Hspd1,Zw10,Schip1,Exo1,Pla2g4a,Pde1a,Slc25a5,Adss,Hbs1l,Casp8,Wipf1,Dhx15,Nupl1,Rasa1,Ckap2,Snrpd1,Dbf4,Sumo2,Gli3,Pkp2,Pvrl3,3110003A17Rik,Jub,Cenpe,Ss18,Tpm3,Actl6a,Plscr1,Cnn3,Pcf11,Zfp281,Fancb,Cebpz
**mmu-miR-511**	47(1239)	6,49E-08	Prpf31,Rbm26,Clns1a,Ets1,Kif2a,Rapgef2,Noc4l,Aspm,Egr3,E2f8,Fen1,Cdca2,Exo1,Trip13,Eme1,2610039C10Rik,Dusp5,Abcb1b,Casc5,Peg12,Spc25,Recql4,Rpl12,Rad54l,Traip,Nr2f2,2810055F11Rik,2610301G19Rik,Tpx2,Pole,Ptplad1,Prmt5,Topbp1,Synj2,Anp32b,Akap8,Cd14,Ppwd1,Trub1,Mast4,Pola2,Pebp1,Gart,Tmpo,Rbpj,Kif23,Hnrpdl
**mmu-miR-193b**	53(1239)	7,19E-08	Timm8a1,Arhgap22,Zfp292,Marcksl1,Whsc1,Nsun2,Llgl1,Mum1,Nrf1,Egr3,Zw10,Pdap1,Acaa2,Mthfd1,Rnps1,D2Ertd750e,Nup93,Sgol1,Ccdc99,Cct2,2610039C10Rik,Trim28,Casc5,Zc3h8,Parp12,Sbk1,Ppp1cc,Usp3,Top2a,Tk1,Mcpt8,Rsf1,Ptcd3,Exosc2,Clspn,Gemin8,Il1rap,Prkg2,Dus3l,Bbs12,2410016O06Rik,Ilf3,Prc1,Rif1,Mycn,Bnc2,Cnot6,Rrad,Stmn1,Abl2,H2afx,Fancc,Rbpj
**mmu-miR-693-5p**	57(1239)	7,21E-08	Notch1,Gata2,Ranbp1,Tcf19,Paxip1,2610101N10Rik,Bcl10,Msh2,Fkbp5,Sltm,Pim3,BC027072,Exo1,Ccdc99,Eef1e1,Ncapd2,Ccnf,Frat2,Scrib,Kif21a,Prpf38a,Nasp,Wipf1,Lyar,Tube1,Fbl,Traip,Usp6nl,Spry2,Kpna2,Bub1b,Rfc5,Melk,Ctsh,Nup88,Slc38a1,Casp6,Apitd1,Nucks1,Zrsr2,Ahcy,G3bp1,Tpp2,Pkmyt1,5730559C18Rik,Slc43a1,Gprc5c,Rcl1,Tacc3,Ptbp2,Th1l,Ccdc18,Gtse1,2010204K13Rik,Hnrpdl,1810011O10Rik,Dlx1
**mmu-miR-467a**	60(1239)	7,61E-08	Diap3,Zfp292,Mtmr4,Mrps22,Strbp,1110020G09Rik,Ddx46,Ddx52,Egr3,D2Wsu81e,Dis3,Ahctf1,Nt5dc2,Tubb5,Suv39h1,Mcm3,Rrm2,Rad21,Armc8,Cdc25a,Pkn3,Thsd7a,Cep152,Lig1,Rad51c,Rpl12,Sf3a1,Nup107,Asf1b,Usp3,Uchl5,Pif1,Hmgb2,Itgav,Nr2f2,Snrpd1,Rfc5,Rad18,Hat1,Mpp6,Cenpn,3110003A17Rik,Cenpf,Fbln1,Actl6a,Cdc40,Suv39h2,Ddx20,Cep55,Syne2,E330016A19Rik,Mrpl50,Stil,Mast4,Bcl2l11,Twistnb,Pcf11,Gpsm2,Dnajc9,AA408296
**mmu-miR-466a-5p**	64(1239)	8,33E-08	Nck2,Prim2,Ell,Ankrd32,Dhx9,Steap1,Mum1,Ctsw,Phactr4,Elavl1,Ddx27,Aspm,E2f8,Kif18a,Msh6,Mtf2,Lcorl,Nfatc2ip,Cep57,Rpl30,Pik3c2a,Pla2g4a,Usp34,1700029F09Rik,Gjc1,Runx2,Rad51c,Kntc1,Ube2c,Kif21a,Gtf2h2,Sbk1,Nusap1,Neil3,Dbf4,Sumo2,Snrpg,Paip1,Mkx,Sip1,Wdr55,Casp6,Kcnk2,Mlf1ip,Topbp1,Ch25h,Usp37,Prc1,Bnc2,Cep55,Plscr1,Slc43a1,Rrad,Mcm6,Specc1,Cenpk,Tex10,Cdca8,Nol11,Gmeb1,Tmpo,Ltbp1,Eps8,Tdrkh
**mmu-miR-449a**	54(1239)	8,81E-08	Nedd4l,Tmem39b,Mcm5,Gmnn,Prpf40a,Tcf19,Taf5,Tnnt2,Ctsw,Sass6,Ccne2,Msh6,Acaa2,Mcm3,Nup93,Asxl3,Sgol1,Slc29a1,2610039C10Rik,Ankrd10,Bzw2,Iqgap3,Ncapd3,Tslp,Vrk1,Fanca,Snw1,Eno3,Nme4,Nt5c3l,2810025M15Rik,Pvrl3,Ppif,Kif2c,Patz1,Actl6a,Nsmce4a,Cdkn2c,Usp37,Foxp1,Mycn,Anp32b,Slc43a3,Rfc2,E330016A19Rik,Ppwd1,Rpl13a,Erh,Zfp281,Hells,Ift74,Slc4a7,Vash2,Cirh1a
**mmu-miR-302c**	54(1239)	9,18E-08	Thap2,1110012J17Rik,Prim2,Llgl1,Bclaf1,2810008M24Rik,Atad2,Ddx46,Dnmt1,Rdx,Riok2,Msh2,Egr3,Ttll4,Slc11a2,Tubb5,Larp7,Abcb1b,Cep152,E130303B06Rik,Idh2,Hbs1l,Gtf2h2,Lrrk1,Rpl12,Asf1b,Pttg1,Nfkbiz,Cdca4,Hspa4l,Rpa2,Hn1,Nmral1,Arid4b,Aifm1,Kin,Pkp2,Nup88,Wdr55,Dut,Cct3,Bbs12,Synj2,Foxp1,Cct8,Rfpl4,Pdss1,Tnfaip6,Pebp1,4632434I11Rik,Ppa1,Slc4a7,Kif23,1810011O10Rik
**mmu-miR-463**	50(1239)	9,22E-08	Cdca7l,Diap3,Clns1a,Mcm5,Dhx9,Ccne1,Noc4l,Ddx46,Dnmt1,Aspm,Myef2,Mrps6,Blm,Impdh2,Lcorl,Mthfd1,Pgk1,Fbxo5,Lin9,Hmgb3,Pde1a,Bzw2,Scrib,Pcnt,Polr2b,Gtf2h2,Prpf4b,Dhx15,Wdr75,Bub1b,Top2a,Unc5c,Cenpa,Prps1,Tmem49,Sox4,Bbs12,Actl6a,Cct8,Tacc3,Cenpk,Trub1,Eif4h,Bcl2l11,Rad51,Hells,Tmpo,Nxt1,Kif23,Cirh1a
**mmu-miR-26a**	53(1239)	9,29E-08	Diap3,Ranbp1,Rnmt,Depdc1b,Hmmr,Ccne1,Mnd1,Msh2,Rest,Tox,Kif18a,Sltm,Impdh2,Nfrkb,Cct7,Pim3,Hmga1,2610039C10Rik,Cbx1,Pkn3,Cep152,Shcbp1,Cul4b,Hmgb1,Chek1,Usp3,Kpna2,Dimt1,Neil3,Cep70,Psmd14,Net1,Ppbp,Ube2t,Cdca3,Synj2,G3bp1,Usp37,Ncapg,Cct8,Tnrc6a,Stmn1,Ppwd1,Vbp1,Phlda1,Hells,Ivns1abp,Pou2f1,Ezh2,Tmpo,Terf1,Cks2,Hnrpdl
**mmu-miR-719**	46(1239)	9,32E-08	Nedd4l,Ncaph,Rbm26,H2afv,Nol9,Kif2a,Dsn1,Noc4l,Msh2,Zw10,Mrps6,Snrpb,Lsm2,Pgk1,Fbxo5,Eef1e1,Pla2g4a,Pde1a,Rasa3,Scrib,Ncapd3,Zc3h8,Hbs1l,Rmi1,Acsl3,Stoml2,Tube1,Nupl1,Ckap2l,Pttg1,Sema4b,1110034A24Rik,Nt5c3l,Cachd1,Mpp6,Patz1,Sdpr,Rfc2,Arhgap19,Vbp1,Cdca8,Me2,Gart,Eftud2,Tdrkh,Pogz
**mmu-miR-546**	51(1239)	9,48E-08	Ncaph,Arhgap22,Gata2,Pop1,Prx,Fhl2,Cks1b,Mum1,Wdr43,Mybbp1a,Has2,Smpdl3b,Paxip1,Nap1l4,Cep192,Rpl7a,Vil1,Hspe1,Ank3,Kif18a,Hspd1,2510012J08Rik,Slc11a2,Msh6,Impdh2,Sfpq,Cenph,Pcna,Slc19a1,Zfp36l2,Eme1,Dhodh,Casc5,E130303B06Rik,Recql4,Zfp7,Slc9a3r1,Psmd14,Chd2,Slc38a1,Tbx3,Ssrp1,Slc43a3,Etv5,Ccdc18,Pou2f1,Mrpl18,Smyd5,Ecd,Kif23,Pogz

miRNA name	*Number of Genes*	*Corrected p-value*	*miRNA targets among genes repressed in Rasless cells (Table S1)*
**mmu-miR-463***	38(1239)	9,58E-08	Gemin4,Rcc2,Skp2,Zfp606,1110020G09Rik,Srpk1,Cep192,Aurka,Mup4,Zc3h15,Impdh2,Acaa2,Nup93,Aebp2,Sgol1,Zfp184,Egfr,Ankrd10,Ppp1cc,Ssb,Hmgb1,Chek1,Tardbp,Rbm25,Nfx1,Snrpg,Cep70,Prps1,Gas2l3,Tpp2,Nav3,Lrrc8c,Cenpk,Atrx,Thoc2,Cdca7,Eif2s1,Smc4
**mmu-miR-154***	43(1239)	1,30E-07	2810046L04Rik,Sirt1,Prkar2b,Trim24,Errfi1,Mcm5,Upf3b,Prpf40a,Zfp334,Gemin6,Elp2,Aspm,Dis3,2510012J08Rik,Fxn,Aftph,BC027072,Zmym1,Usp34,Chst1,Pcnt,Cul4b,Pttg1,Prpf3,Slc9a3r1,Slc38a1,Socs6,Gnl3,Mlf1ip,2410016O06Rik,Nsmce4a,Foxp1,Nup155,Anp32b,Nav3,Rfpl4,Lrrc8c,Whsc2,Zfp281,C330027C09Rik,Cdh10,Mrpl18,Kif23
**mmu-miR-466b-5p**	62(1239)	1,51E-07	Prim2,Ell,Birc5,Ankrd32,Dhx9,Steap1,Mum1,Ctsw,Phactr4,Elavl1,Ddx27,Rbbp7,Aspm,E2f8,Kif18a,Yme1l1,Mtf2,Lcorl,Nfatc2ip,Cep57,Zfp184,Rpl30,Pik3c2a,Cd2ap,Usp34,Brd8,Gjc1,Rad51c,Kntc1,Ube2c,Kif21a,Gtf2h2,Sbk1,Nusap1,Neil3,Syt17,Sumo2,Snrpg,Aifm1,Pvrl3,Casp6,Kcnk2,Mlf1ip,Tnks2,Ch25h,Usp37,Prc1,Bnc2,Cep55,Plscr1,Slc43a1,Rrad,Mcm6,Specc1,Col7a1,Cenpk,Tex10,Cdca8,Gmeb1,Tmpo,Ltbp1,Eps8
**mmu-miR-192**	49(1239)	1,54E-07	Cdca7l,Dhx9,Ets1,Sass6,Wdr5,Ruvbl1,Als2cr12,Ank3,Ctcf,Tox,Slc11a2,Cdk2,Luc7l,Tll1,Rrm1,Ccdc99,Zfp532,Casc5,Crlf1,Iqgap3,Rmi1,Depdc1a,Larp4,Tardbp,D10Wsu102e,Ckap2,1110034A24Rik,Top2a,Mmd,Snrpg,Egr1,Isg20l2,Tipin,Chtf18,Gas2l3,Rif1,Cdc6,Pum2,Fancd2,E330016A19Rik,Ppwd1,H2afx,Hells,Dyrk3,Nsl1,2010204K13Rik,Rbpj,Slc4a7,Pogz
**mmu-miR-652**	51(1239)	1,72E-07	Tgif2,Ktn1,Nol9,Llgl1,Elf2,Fosl1,Ctsw,Tes,Elavl1,Wdr3,Cdc27,Msh2,Vil1,Ilf2,Nuf2,Mrps6,Schip1,Tfdp1,Slc19a1,Zmynd19,Trim28,Mpzl1,Idh2,Nxf1,Zfp451,Kars,Spry2,Wdr75,Ier2,Ncaph2,Gins4,Itgav,Wee1,Hn1,Slc38a1,Exosc2,Patz1,Ttc3,Synj2,2410016O06Rik,Foxp1,Tnrc6a,Mcm2,Ssrp1,Thoc2,Bcl2l11,Gart,Egfl7,E130308A19Rik,Uhrf1,Eftud2
**mmu-miR-466f-5p**	66(1239)	1,86E-07	Cdca7l,Arhgap22,Prim2,Ankrd32,Depdc1b,Mum1,Wdr43,Zfp52,Ctsw,Hmmr,Phactr4,Lrig3,Aurkb,Noc4l,Cenpj,Aspm,E2f8,Gspt1,Mtf2,Acaa2,Lcorl,Ect2,Fnbp1l,Armc8,Cenph,Zic1,Pik3c2a,Pla2g4a,Matr3,Gjc1,Chst1,Usp14,Eif4a3,Prpf38b,Sbno1,Lrrk1,Stoml2,Palld,Zfp54,Nfx1,Snrpd1,Syt17,Snrpg,Arid4b,Ctsh,Tpx2,Ube2t,Cenpn,Wdr55,Casp6,Mlf1ip,Actl6a,Bnc2,Plscr1,Fmnl3,Mcm6,Specc1,Dcbld1,Pdss1,Cnn3,1700025G04Rik,Cdca8,Ltbp1,Cdh10,Tpbg,Zcchc11
**mmu-miR-23b**	52(1239)	1,92E-07	Marcksl1,Skp2,Ktn1,Prpf40a,Elf2,Mum1,Dctd,Ddx46,Ddx52,6720463M24Rik,Vrk3,Zw10,Cpsf4,Tgif1,Rnps1,BC055324,Snrpb,Fbxo5,Cenph,Nudt1,Dusp5,Gpbp1,H2-K1,Crebzf,Lrrk1,Rpl12,Dhx15,Espl1,Kpna2,E330009J07Rik,Hmgb2,Pank4,Psmd14,Hat1,Pigf,Ube2t,Tmem49,Ppif,3110003A17Rik,Zbtb12,Zrsr2,Tpm3,Mboat2,Dcbld1,Ppwd1,Snrpb2,Gmps,H2afx,Zfp281,Tmpo,Gtse1,Mrpl18
**mmu-miR-9***	44(1239)	2,01E-07	Timeless,Pcm1,Impa2,Birc5,Ktn1,Hspa8,Fhl2,Pak1,Mnd1,Dnmt1,Tpd52,Xpo7,Tiam2,Ttll4,Tmem48,Mcm3,Hdac2,Usp1,Cand1,Rrm1,Pik3c2a,Emg1,Micall2,Baz1a,Rps9,Depdc1a,Slbp,Nupl1,Rasa1,Rbm25,Gtpbp10,Cdca4,Top2a,Snrpd1,Pigf,Rfc3,Pbk,Actl6a,Nsmce4a,Fancd2,Ddx39,Apex1,Wdr36,Smc4
**mmu-miR-466h**	65(1239)	2,11E-07	Prim2,Dhx9,Steap1,Ctsw,Fubp1,Aurkb,Smc6,Xkr5,E2f8,Ank3,Msh6,Acaa2,Sin3a,BC055324,Snrpb,Nup93,Armc8,Cenph,Cdca2,Lin9,Pik3c2a,1700029F09Rik,Kif20a,Brd8,Gjc1,Dtl,Chst1,Npn2,Iqgap3,Kif21a,Lrrk1,Sf3a1,Zfp451,Nupl1,Uchl5,Syt17,Ptk7,Sumo2,Aifm1,Zwilch,Mpp6,Ankrd28,Cenpn,Prr11,Casp8ap2,Wdr55,3110003A17Rik,Usp37,Cct8,Bnc2,Slc43a1,Rrad,Abl2,Lrrc8c,Cep170,Trub1,Rpl13a,2610027L16Rik,Pdss1,Tex10,Cdca8,Twistnb,Ltbp1,Tpbg,Kif23
**mmu-miR-367**	54(1239)	2,13E-07	Cdca7l,Rbm26,Tnpo1,Ercc6l,Fancm,Dhx9,Fhl2,Has2,Smpdl3b,Ripk2,Pspc1,Mup4,Marcks,Nrf1,Zc3h15,Ahctf1,Ccne2,Mtf2,BC055324,Ppil1,Eef1e1,Larp7,B230120H23Rik,Cul4b,Rsbn1,Imp3,Mtap,Rpl12,Acsl3,Prpf38a,Lyar,Zfp54,Myo1b,Zcchc2,Cachd1,Ints7,3110003A17Rik,BC016423,Ch25h,Foxp1,Eif5,Prc1,Fancd2,Dcbld1,Ppwd1,Rpl13a,Lifr,Pcf11,Zfp281,Ppa1,Cdh10,Dock5,Mrpl18,Ecd
**mmu-miR-141**	52(1239)	2,16E-07	6430527G18Rik,Timeless,Birc5,Abi1,Rnmt,Prx,Glrx,Fus,Has2,2610101N10Rik,Las1l,Setdb2,Ythdf2,Ddx46,Tcerg1,Aspm,Mup4,Ruvbl1,Fkbp5,Ythdc1,Ttll4,Ccne2,Mtf2,Api5,Mthfd1,Sfpq,Luc7l,Klhdc2,Ctbp2,Cbx1,Ctnna1,Psmc3ip,U2af1,Usp14,Dusp6,Baz1a,Idh2,Ssb,Slco4a1,Tead2,Arid4b,Dus3l,Tnks2,Ilf3,Eif5,Mcm6,Specc1,D16Ertd472e,Arhgap19,Cep170,Mast4,Psmd5
**mmu-miR-707**	48(1239)	2,33E-07	Ncaph,Asph,Llgl1,Ppih,Lrig3,2610101N10Rik,Nipsnap1,Dnmt1,Tcerg1,6720463M24Rik,Vrk3,Mrps6,Dhx36,Tmem173,D2Ertd750e,Mcm3,Usp1,Slc19a1,Setx,Larp7,Usp34,Cbx1,Adss,Dhodh,Casc5,Shcbp1,Recql4,Troap,Espl1,Rbm17,Axl,Fzd3,3110003A17Rik,Cenpf,Cnnm4,Rps13,Apitd1,Cct8,Fancd2,Lrrc8c,Mast4,Ddx39,Bcl2l11,Utp11l,4632434I11Rik,Cebpz,Kif23,Vash2
**mmu-miR-690**	47(1239)	2,33E-07	Pop1,Zfp292,Prkar2b,Skp2,Trim59,Idh3a,Kif4,Fubp1,Kif11,1110020G09Rik,Srpk1,Noc4l,Aurka,Ube2e3,Tiam2,Mrps6,Acaa2,Mthfd1,Hdac2,Cand1,Ncapd2,Cbx1,Adss,Abcb1b,Scrib,Troap,Depdc1a,E330009J07Rik,Wee1,Pigf,Dut,Dus3l,Rfc3,Nsmce4a,Tnrc6a,5730559C18Rik,Bnc2,Cnot6,Syne2,6330503K22Rik,Nob1,Mre11a,Thoc2,Cdca8,Apex1,Gtse1,Eftud2
**mmu-miR-674***	40(1239)	2,40E-07	Impa2,Birc5,Tcf19,Ets1,Tnnt2,Prpf19,Rangap1,1110020G09Rik,Srpk1,Fkbp5,Mcm3,Pgk1,Armc8,Ncapg2,Dhodh,Kif21a,Fbl,Zfp54,Csnk1a1,Tead2,Tk1,Nt5c3l,Ctsh,Psmd14,Net1,Zwilch,Cachd1,Ckap4,Sfxn1,Wdr55,Wdr73,Dapp1,Anp32b,Nav3,Ell2,Rfc2,Ppa1,Cebpz,Dmap1,Pogz
**mmu-miR-103**	52(1239)	2,55E-07	Chd1,Prim2,Dnaja2,Rnmt,Fhl2,Taf5,Wdr43,Acly,Ccne1,Sass6,Mapk8,Cep192,Ddx46,Aurka,Parp1,Slc11a2,Mrps6,Nfrkb,Nfyb,Cct5,Armc8,Exo1,Ctbp2,Cep152,Shcbp1,Cul4b,Ssb,Nupl1,Pttg1,Ncaph2,Uchl5,Nmral1,Axl,Wdr55,Clspn,Patz1,Mcm7,Usp37,Foxp1,Eif5,Tpp2,Slc43a1,Pafah1b3,Rfpl4,Vbp1,Bcl2l11,4632434I11Rik,Fancc,Rbm12,Cdc73,Smyd5,Gins3
**mmu-miR-183**	53(1239)	2,68E-07	Tgif2,Ranbp1,Rbmxrt,Skp2,Gmnn,Fhl2,Ets1,Cks1b,Elf2,Kif2a,Wdr43,Nme1,Cpsf6,Emb,Cdc27,Cdc5l,Rnf138,Impdh2,Dhx36,Sin3a,Ash2l,Enpp1,Egfr,Zmynd19,Chuk,Ccnb1,Smc5,Idh2,Nasp,Zfp451,Spry2,Slco4a1,Wdr75,Kpna2,Rpa2,Hn1,Trp53bp2,Exosc2,Kcnk2,Wdr73,Ilf3,Ch25h,Foxp1,Strap,Ssrp1,Slc43a3,Pdss1,E130308A19Rik,Tmpo,Smyd5,Smarcc1,Kif23,Hnrpdl
**mmu-miR-330***	45(1239)	2,79E-07	Cdca7l,Rcc2,Cenpc1,Ankrd32,Cks1b,Smpdl3b,Fubp1,Dnmt1,Ruvbl1,Als2cr12,Parp1,Hspd1,Tmem48,Tubb5,Apex2,Mcm3,Luc7l,Msh3,Scarb1,Sh3kbp1,Hmga1,2610039C10Rik,Runx2,B230120H23Rik,2700029M09Rik,Frat2,Idh2,Bub1b,Kin,Tpx2,Fzd3,Mcm7,Pbk,Actl6a,Cdc40,Ssrp1,Cd14,Sf3a3,Apex1,4632434I11Rik,Ezh2,Cenpi,Gtse1,Kif23,Eps8
**mmu-miR-675-3p**	45(1239)	2,79E-07	Etaa1,Nono,Nedd4l,Lrrc45,Asph,Cenpc1,Prx,Dhx9,D19Bwg1357e,Glrx,Mum1,Ctsw,2610101N10Rik,U90926,Aurkb,Ythdf2,Cep192,Msh2,Myef2,Cep68,Gspt1,Msh6,Impdh2,Apex2,D2Ertd750e,Cep57,Stip1,Hn1l,Pcnt,Gtf2h2,Cdc7,Rpa2,Unc13c,Aifm1,Cachd1,Pvrl3,Smc2,Topbp1,Cdc40,Prc1,Bhlhb9,1700025G04Rik,Dyrk3,Rbm12,Hnrpdl
**mmu-miR-9**	51(1239)	2,82E-07	Lrrc45,Rcc2,Prim2,Ktn1,Depdc1b,Snx7,Smpdl3b,Lrig3,Cep192,6720463M24Rik,Ahctf1,Yme1l1,Wsb1,Lrp8,Prim1,Msh3,Rrm1,Ctbp2,Zmynd19,Gins1,Pdk3,Ctnna1,Dtl,Abcb1b,B230120H23Rik,Bzw2,Scrib,Kif21a,Tslp,Cul4b,Nxf1,Rad54l,Csnk1a1,Hspa4l,Neil3,Prpf3,Unc13c,Nt5c3l,Melk,Pkp2,Psmd14,Cdca3,Exosc2,Socs6,Smarca5,Cdc40,Gas2l3,Pdss1,Whsc2,4632434I11Rik,Mrpl18
**mmu-miR-22**	53(1239)	2,88E-07	Pcm1,Rcc2,Dffb,Ell,Caprin1,Whsc1,Upf3b,Ktn1,Eef1g,Prpf19,Strbp,Pdia6,Ptpre,Vrk3,Sgol2,Lrp8,Tomm70a,Pdap1,Sin3a,Nfyb,Klhdc2,Zfp184,Emg1,Gpbp1,Rbbp4,X99384,Sgms1,Fanca,Prpf38a,Slbp,F630043A04Rik,Arpc5,Rfc5,Net1,Mat2a,Apitd1,Zrsr2,Cdc40,Impa1,Prc1,Lmnb2,Pkmyt1,Umps,Anp32b,Bnc2,Syne2,Gprc5c,Th1l,2610027L16Rik,Ptger4,Cdca7,Uhrf1,Eftud2
**mmu-miR-384-3p**	44(1239)	3,03E-07	Phf6,Prim2,D19Bwg1357e,Cenpq,Sass6,Emb,Med4,Ythdf2,Ube2e3,6720463M24Rik,Ets2,2510012J08Rik,Msh6,Mtf2,Taf5l,Sfpq,Usp10,Zfp184,Zmym1,Cbx1,Mbtps2,Usp14,Suz12,Vangl2,Sbno1,Prpf38a,Zfp451,Rad54l,Mcm10,Phf17,Unc5c,Arid4b,Pank4,Rsf1,Tmem49,Zfp619,Ncapg,Cct8,Cep170,Ptbp2,H2afx,Ahcyl1,Kif23,Pogz

miRNA name	*Number of Genes*	*Corrected p-value*	*miRNA targets among genes repressed in Rasless cells (Table S1)*
**mmu-miR-743b-5p**	52(1239)	3,23E-07	Snx5,Pcm1,Ankrd32,Gmnn,Cks1b,Plk1,Hmmr,Smpdl3b,Prpf19,Setdb2,Cep192,Pgm1,Tpd52,Pom121,Mad2l1,Vrk3,Wsb1,Zw10,Nfatc2ip,Armc8,Exo1,Ccdc99,Cct2,Pla2g4a,Slc25a5,Dtl,Pcnt,Shcbp1,Tslp,Ccna2,Gins4,Mmd,Snrpg,Mcpt8,Melk,Pkp2,Rbm17,Prkg2,Strap,Tnrc6a,Bnc2,Th1l,Psmd5,Pola2,Nol11,Gmeb1,Smu1,H2afx,Eftud2,Kif23,Eif2s1,Pogz
**mmu-miR-883a-5p**	54(1239)	4,38E-07	Timeless,Fhl2,Kif2a,Ogfrl1,Cenpq,Dctd,Prpf19,U90926,Ddx27,Pgm1,E2f8,Vrk3,Ttll4,Tmem48,Impdh2,Cct5,Dbr1,Gpd2,Egfr,Snrpa1,Pcnt,Cul4b,Vrk1,Fanca,Kars,Ptges3,Cdc7,Bub1b,Tead2,Tk1,Itih2,Arpc5,Nab2,Cachd1,Isg20l2,Ttc3,Apitd1,Tpm3,Tnks2,Rbm14,Dapp1,Plekha5,Spp1,Spc24,Ell2,Fmnl3,Dtymk,Mre11a,Cnn3,Lifr,Phlda1,Ivns1abp,Nsl1,Wdr36
**mmu-miR-139-3p**	51(1239)	4,71E-07	Fmr1,Arhgap22,Rcc2,Mcm5,Whsc1,Skp2,Eef1g,Spred2,Mum1,Ctsw,Ppih,Dctd,Rangap1,Aurka,Sgol2,Nt5dc2,2510012J08Rik,Mrps6,Nup93,Cct5,Cenph,Rrm1,Ppil1,Dusp4,Slc19a1,2610039C10Rik,Abcb1b,Thsd7a,Casp2,Baz1a,H2-K1,Crebzf,Nxf1,Ssb,Dhx15,Myo1b,Gli3,Tpx2,Zwilch,Slc38a1,BC016423,Wdr73,Pold3,Psmd5,Etv4,Cdca8,Hells,Egfl7,Eftud2,2810408A11Rik,Ecd
**mmu-miR-154**	44(1239)	4,72E-07	Clns1a,Dhx9,D19Bwg1357e,Ccbe1,Nol9,Elk3,Ppih,Aurkb,Ythdf2,Aurka,D2Wsu81e,Msh6,Mrps6,Por,Tmem173,Nfrkb,Klhdc2,Myc,Lin9,Pcna,Stip1,Pde1a,Matr3,Pkn3,Casc5,Satb2,Casp8,Crebzf,Prpf38a,Kctd12b,Snw1,Rasa1,Cdc7,Cdca4,Snrpg,Rfc5,Nme4,Nup88,Tnks2,Nolc1,Rfpl4,Dtymk,Thoc2,Tpbg
**mmu-miR-146b**	50(1239)	4,85E-07	Gemin4,Ercc6l,2810008M24Rik,Ripk2,Nap1l4,Strbp,Cep192,Parp1,Ahctf1,Schip1,Blm,Cdk2,Cct7,Psrc1,Zmym1,Emg1,2610039C10Rik,Adss,Cep152,Rps9,Satb2,Imp3,H2-K1,Prpf38a,Zfp451,Dhx15,Slbp,Slco4a1,Card10,Usp3,Npm1,Slc9a3r1,Pkp2,Cachd1,Axl,Cdca3,Kif2c,Kif22,Mastl,Actr3,Ddx10,Pum2,Nob1,Dtymk,Rsrc2,Whsc2,Cdh10,Top1,Kif23,Tdrkh
**mmu-miR-449c**	52(1239)	4,90E-07	Chd1,Nedd4l,Tmem39b,Marcksl1,Gmnn,Prpf40a,Tcf19,Wdr43,Tnnt2,Ctsw,D2Wsu81e,Vrk3,2510012J08Rik,Acaa2,Mcm3,Nup93,Ssr4,Slc29a1,2610039C10Rik,Cep152,B230120H23Rik,Bzw2,Iqgap3,Ncapd3,Tslp,H2-K1,Fanca,Wipf1,Snw1,Eno3,Nme4,Nt5c3l,Arid4b,Axl,Fzd3,Gemin8,Patz1,Actl6a,Nsmce4a,Cdc40,Usp37,Ssrp1,Slc43a3,Fmnl3,Rfc2,E330016A19Rik,Ppwd1,Rpl13a,Zfp281,Ift74,Vash2,Cirh1a
**mmu-miR-290-3p**	54(1239)	5,75E-07	Rbm26,Diap3,Ap1s3,Mrps22,Nipsnap1,1110020G09Rik,Cep192,Ddx46,Egr2,Egr3,Ilf2,Nt5dc2,Mrps6,Sin3a,Tubb5,Mcm3,Rrm2,Rad21,Nudt1,Rpl30,Pkn3,Cep152,Ccrn4l,Zfp217,Ccng2,Gtf2h2,Depdc1a,Rpl12,Sf3a1,Pif1,Itgav,Nr2f2,Arid4b,Rad18,Ebna1bp2,Tpx2,Hat1,Mpp6,Sip1,3110003A17Rik,Cenpf,Lrig1,Xpo1,Apitd1,Ahcy,Cdc40,Ddx20,Tpp2,Plekha5,Mboat2,Fmnl3,Phc2,Khdrbs1,Nup43
**mmu-miR-27b**	50(1239)	5,98E-07	Pcm1,Dffb,Cenpc1,Ercc6l,Ccbe1,Depdc1b,Zfp52,Ctsw,Gemin6,Dnmt1,Aurka,Riok2,Msh2,Vrk3,Dusp9,Rrm2,Cct5,Dbr1,Nudt1,Chst1,Cep152,Rad51c,Polr2b,Satb2,Sgms1,Imp3,Nxf1,Nusap1,2810474O19Rik,Npm1,Nr2f2,Nfx1,Mmd,Kin,Pvrl3,Fzd3,Ppif,Dut,Kif2c,Cct3,Apitd1,Wdr73,Eif5,Ruvbl2,Plscr1,Rfpl4,Dcbld1,Ddx39,1700025G04Rik,Ezh2
**mmu-miR-135a**	51(1239)	5,99E-07	Asph,Zfp292,Elk3,9030617O03Rik,Tnnt2,Tes,Ddx27,Nrp1,Kif18a,2510012J08Rik,Blm,BC055324,Aebp2,Msh3,Smarce1,Exo1,Ppil1,Pla2g4a,Smek1,1700029F09Rik,Dusp5,Ccnb2,Dtl,Psmc3ip,Dhodh,Bzw2,Baz1a,Hbs1l,Nxf1,Zfp7,Rnaseh2a,Gli2,1110034A24Rik,Prpf3,Nt5c3l,Mtm1,Zfp619,Prkg2,Mastl,Eif5,Strap,Yes1,Trub1,2610027L16Rik,Ddx39,Sf3a3,Apex1,Ccdc18,Pou2f1,Ppa1,Cirh1a
**mmu-miR-881***	33(1239)	6,12E-07	Etaa1,Ahsa1,Impa2,Ifi205,Mybbp1a,Ppih,Fkbp5,Nuf2,Socs3,Sltm,BC055324,D2Ertd750e,Pgk1,Thsd7a,Spc25,Crebzf,Prpf38a,Spry2,Kpna2,Gins4,Itgav,Rfc5,Pigf,Ube2t,Xpo1,Prkg2,Cdkn2c,Fancd2,Dtymk,Cdca8,Incenp,Rbpj,Pogz
**mmu-miR-374***	39(1239)	6,15E-07	Gata2,Impa2,Dhx9,Prpf40a,Snx7,Zfp606,Dcp1a,Zfp57,Elp2,Nuf2,Ahctf1,Tmem48,Lrp8,Dhx36,Acaa2,Luc7l,Tll1,Hspa14,Pde1a,Scrib,2310057M21Rik,Nfx1,Mmd,Nt5c3l,Pkp2,Cenpe,Pbk,Rbm14,Eif5,Yes1,Plscr1,Dtymk,Fignl1,Tex10,Pola2,Fancb,Cebpz,Tdrkh,Cirh1a
**mmu-miR-361**	46(1239)	6,27E-07	Nck2,Tmem39b,Sirt1,Depdc1b,Plk4,Zfp606,Nup37,9030617O03Rik,Snapc3,Idh3a,Nipsnap1,1110020G09Rik,Riok2,Ruvbl1,2510012J08Rik,Lcorl,Snrpb,Usp10,1700029F09Rik,Psmc3ip,Cep152,Snrpa1,Npm1,Phf17,Dbf4,Kin,Trit1,Hat1,Cachd1,Pvrl3,Fzd3,3110003A17Rik,Nsmce4a,Strap,Cnot6,Specc1,Col7a1,Mast4,Vbp1,Phlda1,Incenp,Hells,4632434I11Rik,Fancb,Ltbp1,2010204K13Rik
**mmu-miR-27a**	49(1239)	6,34E-07	Pcm1,Dffb,Cenpc1,Ercc6l,Ccbe1,Depdc1b,Zfp52,Gemin6,Prpf19,Aurka,Riok2,Msh2,Vrk3,Dusp9,Rrm2,Cct5,Dbr1,Nudt1,Cep152,Polr2b,Satb2,Sgms1,Imp3,Nxf1,2810474O19Rik,Bub1b,Nr2f2,Nfx1,Mmd,Kin,Hat1,Ckap4,Pvrl3,Fzd3,Ppif,Dut,Kif2c,Cct3,Apitd1,Wdr73,Eif5,Ruvbl2,Plscr1,Rfpl4,Dcbld1,Arhgap19,Ddx39,1700025G04Rik,Ezh2
**mmu-miR-135b**	49(1239)	6,60E-07	Ncaph,Elk3,Tnnt2,Tes,Ddx27,Egr3,Nrp1,2510012J08Rik,Blm,BC055324,Aebp2,Msh3,Smarce1,Exo1,Ppil1,Eef1e1,Pla2g4a,Trip13,1700029F09Rik,Dusp5,Ccnb2,Psmc3ip,Dhodh,Bzw2,Crlf1,Baz1a,Recql4,Hbs1l,2210018M11Rik,Stoml2,Zfp7,Rnaseh2a,E330009J07Rik,Rbm25,1110034A24Rik,Prpf3,Arid4b,Mtm1,Smchd1,Mastl,Eif5,Strap,Trub1,2610027L16Rik,Ddx39,Sf3a3,Ccdc18,Ppa1,Rbl1
**mmu-miR-434-5p**	50(1239)	6,64E-07	Etaa1,Ankrd50,Ahsa1,Trim24,Cks1b,Ppih,Prpf19,2610101N10Rik,Bub3,Cit,Zw10,Aebp2,Cenph,Asxl3,Enpp1,Ctbp2,Eef1e1,Dusp4,Ung,Zmynd19,Slc29a1,Gins1,Brd8,Peg12,Zc3h8,Hyal2,Rbm25,4930579G24Rik,Kin,Chd2,2810025M15Rik,Igsf3,Ptplad1,Prps1,Tipin,Lrig1,Nudc,Sertad1,5730559C18Rik,Syne2,Lmnb1,Pum2,B3galnt1,Fancd2,Mrpl50,Cenpk,Rpl13a,Sf3a3,Incenp,Rbm12
**mmu-miR-21***	45(1239)	6,87E-07	Depdc1b,Plk1,Ppih,Ccne1,Pank1,Wdr3,Cep192,Msh2,E2f8,Ppat,Cdca5,Schip1,Mtf2,Lcorl,Luc7l,Cand1,Larp7,Ncapd2,Psmc3ip,Wipf1,Usp3,Top2a,Arpc5,Pigf,Fzd3,Wdr55,Jub,Rbmx2,Dut,Limd2,Mphosph10,Tbx3,Foxp1,Strap,Rrad,Stmn1,Dcbld1,Col7a1,Pcf11,4632434I11Rik,Fancc,Gtse1,Cdt1,Mrpl18,1810011O10Rik
**mmu-miR-28***	40(1239)	6,88E-07	Rbm26,Upf3b,Elf2,Pak1,Prpf19,2610101N10Rik,Aurka,Tiam2,Tgif1,Ect2,Zmym1,Egfr,Pde1a,Smc5,Acsl3,Kars,Spred1,E330009J07Rik,Bub1b,Psmd14,Cct3,Smc2,Gnl3,Pum2,Bhlhb9,C79407,Zcchc8,Fignl1,Cenpk,Ptbp2,Snrpb2,Cnn3,Pcf11,Zfp281,Ccdc18,Ppa1,Nsl1,Ltbp1,Arf6,Slc4a7
**mmu-miR-872**	46(1239)	7,74E-07	Rbmxrt,Mum1,1110020G09Rik,Aurka,Rdx,Nrp1,Wsb1,Sltm,Dbr1,Zic1,Zfp184,Pde1a,Micall2,Runx2,Ube2c,Pcnt,Gtf2h2,Pola1,H2-K1,Crebzf,Traip,Spry2,Ipo7,Snw1,Hmga2,Tead2,Psmd14,Axl,Nsmce4a,Cdc40,Rbm14,Ch25h,Ruvbl2,Yes1,Cnot6,Spc24,Mcm6,Fignl1,Cenpk,Trub1,Pdss1,Thoc2,Nsl1,Eftud2,Dnajc9,Rrs1
**mmu-miR-700**	47(1239)	7,87E-07	Impa2,Mum1,Mybbp1a,Ctsw,Ppih,Rangap1,U90926,Cdc5l,Nuf2,Cit,2510012J08Rik,Rnps1,Usp10,Luc7l,BC027072,Ctbp2,Dusp4,Ung,Eme1,2610039C10Rik,Cbx1,Gjc1,Ccnb2,Cep152,Sgms1,Crebzf,Ppp1cc,2210018M11Rik,Wipf1,Kars,Chaf1b,Phf17,Tk1,Cachd1,Casp8ap2,Jub,Slc38a1,Apitd1,Sertad1,Ilf3,Anp32b,Abl2,Tacc3,Hirip3,Egfl7,Smarcc1,Hnrpdl
**mmu-miR-320**	48(1239)	7,97E-07	Prpf31,Nup54,Impa2,Birc5,Ifi205,Snx7,Las1l,Zc3h15,Gspt1,Ttll4,Tmem48,Casp3,Tubb5,Brca1,Luc7l,Zic1,Rrm1,Exo1,Pcna,Zmynd19,Abcb1b,Bzw2,Crlf1,Mapk1,Ncapd3,Snrpa1,Depdc1a,Tial1,Prpf38a,Cobll1,Rad54l,Cbx3,Ccna2,Bub1b,Pif1,Nfx1,Top2a,Snrpd1,Pank4,Limd2,Kif22,Xpo1,Rfc3,Sertad1,Nsmce4a,Mastl,Zfp281,Ppa1
**mmu-miR-151-3p**	45(1239)	8,18E-07	1110012J17Rik,Impa2,Mum1,Kif4,Ccdc45,Mad2l1,Ctcf,Sgol2,Lrp8,Brca1,BC055324,Lsm2,Aebp2,Nfatc2ip,Sgol1,Pde1a,Cbx1,Spc25,Sf3a1,Dhx15,Asf1b,Itgav,Ckap2,1110034A24Rik,Unc13c,Pkp2,Axl,Pvrl3,Cenpe,Cct3,4933427D14Rik,Prmt5,Ddx20,Cct8,Cnot6,Spp1,Pum2,B3galnt1,Fancd2,Rfc2,Rcl1,2610027L16Rik,Bcl2l11,AI848100,Eftud2

miRNA name	Number of Genes	Corrected p-value	miRNA targets among genes repressed in Rasless cells (Table S1)
**mmu-miR-669a**	48(1239)	8,27E-07	Vegfc,Impa2,Dhx9,Ccbe1,Depdc1b,Elf2,Kif2a,Snx7,Atad2,Med4,Nuf2,Gspt1,Tmem48,Lrp8,Dhx36,Usp10,Luc7l,Nudt1,Lin9,Dusp4,Pde1a,Gins1,Brd8,Chst1,Npn2,Gtf2h2,Zfp451,Dhx15,Dimt1,Hspa4l,Syt17,Magoh,Nup88,Eif4enif1,Rfc3,Mlf1ip,Topbp1,Wdr73,Pum2,Specc1,Fbxo32,Lrrc8c,Cenpk,1700025G04Rik,Cdca8,Gtse1,Hnrpdl,Tdrkh
**mmu-miR-96**	52(1239)	8,31E-07	Tgif2,Gemin4,1110012J17Rik,Arhgap22,Ranbp1,Mtmr4,Gmnn,Zfp334,Wdr43,Zfp606,Dsn1,Tes,Dnmt1,Aurka,Cdc27,Pgm1,Sltm,Pdap1,Impdh2,Cenph,Zfp184,Sh3kbp1,Zmym1,Zmynd19,Pde1a,Ncapd2,Ctnna1,Abcb1b,Casc5,Sgms1,Nxf1,Stoml2,Smc3,Rbm25,Topors,Nfx1,2810055F11Rik,Unc13c,Nme4,Tpm3,Trpc2,Synj2,2410016O06Rik,Usp37,Ncapg,Cnn3,Gart,Cdca7,Rbm12,Nxt1,Wdr36,Eps8
**mmu-miR-188-3p**	52(1239)	8,63E-07	Timeless,Pcm1,Gmnn,Tcf19,Ets1,9030617O03Rik,Dsn1,Ctsw,Plk1,Ddx27,Cenpj,Aspm,Ilf2,Gspt1,Slc11a2,Cpsf4,Pdap1,Dusp9,BC055324,Zmynd19,Cbx1,Pkn3,Gpbp1,Ncapd3,Sbno1,Ptpn12,Wipf1,Kars,Card10,Syt17,Nmral1,Nt5c3l,Aifm1,Pank4,Nab2,Cachd1,Cenpn,Limd2,Zbtb12,Tpm3,Sertad1,Tnrc6a,5730559C18Rik,B3galnt1,Specc1,Dcbld1,Rfc2,Mre11a,Rasal2,Ptbp2,Ivns1abp,Tdrkh
**mmu-miR-34a**	56(1239)	8,80E-07	Nono,Nedd4l,Pcm1,Marcksl1,Gmnn,Taf5,Tnnt2,Ctsw,Smpdl3b,D2Wsu81e,Cep68,Fxn,Msh6,Acaa2,Tuba1c,Zfp532,Zmynd19,2610039C10Rik,B230120H23Rik,Bzw2,Iqgap3,Ccnf,Ncapd3,Tslp,Ptpn12,Gli2,Eno3,Nme4,Nt5c3l,Mtm1,2810025M15Rik,Pigf,Axl,Ppif,Actl6a,Wdr73,Nsmce4a,Ilf3,Mastl,Usp37,Slc43a3,Nav3,Ell2,Fbxo32,Rfc2,Dtymk,Arhgap19,Ppwd1,Rpl13a,Timp1,Cdca8,Zfp281,Gart,Egfl7,Vash2,Cirh1a
**mmu-miR-455**	52(1239)	9,31E-07	Sirt1,Prim2,Dnaja2,Birc5,Taf5,Ppih,Rangap1,Sass6,Wdr3,Ets2,2510012J08Rik,Cdca5,Mcm3,Snrpb,Lsm2,Hdac2,Ddx18,Dbr1,Armc8,Nudt1,Larp7,Slc29a1,2610039C10Rik,Ccnb2,Dhodh,Eif4a3,Snrpa1,Troap,Vrk1,H2-K1,Rpl12,Prpf38a,Gins4,Nr2f2,Ctsh,Ckap4,Lrig1,Anp32b,Mcm2,H2afz,C79407,Fancd2,Tacc3,Ppwd1,Trub1,H2afx,Incenp,4632434I11Rik,Uhrf1,Kif23,Rrs1,Dmap1
**mmu-miR-325**	58(1239)	9,65E-07	Ankrd50,Asph,Rnmt,Steap1,Vps36,Prdx4,Ccne1,Pspc1,1110020G09Rik,Mup4,6720463M24Rik,Impdh2,Ifitm3,Tubb5,Ppid,Usp1,Asxl3,Cacybp,Ssr4,Larp7,Eme1,Cep152,Usp14,Lig1,Pnpt1,Trim25,Dhx15,Cbx3,Nap1l1,Cdc7,Npm1,Pprc1,Snrpd1,Neil3,Mcpt8,Ctsh,Hat1,Magoh,Mpp6,Qars,Rbmx2,Clspn,Mphosph10,Mlf1ip,Ddx31,Synj2,Gas2l3,Mastl,Cct8,H2afz,Rrad,B3galnt1,Lrrc8c,1110004E09Rik,Cdca8,Pcf11,Incenp,4632434I11Rik
**mmu-miR-693-3p**	49(1239)	9,68E-07	Tgif2,Rbm26,Nup54,Ercc6l,Impa2,Ankrd32,Tnnt2,Atad2,Tpd52,Bcl10,Cep68,Zc3h15,Msh6,Casp3,Blm,Pim3,Tuba1c,Luc7l,Exo1,Pla2g4a,Pde1a,Supt16h,Rad51c,Gnb4,Wipf1,Cobll1,Dhx15,Chaf1b,Rnaseh2a,Kpna2,Hmgb2,Wee1,Psmd14,Nup88,3110003A17Rik,Ttc3,Rad51ap1,2410016O06Rik,Impa1,Yes1,Ssrp1,Cd14,Rfpl4,Th1l,Rpl13a,Cebpz,Rbm12,Mrpl18,Eps8
**mmu-miR-450b-3p**	48(1239)	9,69E-07	Lrrc45,Diap3,Rbmxrt,Dhx9,D19Bwg1357e,Snx7,Ppih,2010002N04Rik,Noc4l,Rbbp7,Aspm,Tmem176b,Ilf2,Fkbp5,Tox,Dis3,Gspt1,Zw10,Usp10,Cd2ap,Larp7,Zmynd19,Cep152,Rad51c,Baz1a,Hmga2,Hmgb2,Ckap2,Top2a,Prpf3,Cep70,Psmd14,Exosc2,Nudc,Slc25a24,Nsmce4a,Slco1a5,Ddx20,Prc1,Tpp2,C79407,Th1l,Psmd5,Pdss1,Sf3a3,Incenp,Ppa1,Rbm12
**mmu-miR-431***	42(1239)	1,10E-06	Dhx9,Elf2,Spred2,Mum1,Prpf19,Fubp1,Nipsnap1,Cep192,Ets2,Ank3,Nuf2,Ahctf1,Lrp8,Dhx36,Mcm3,Asxl3,Ncapd2,Ccnb2,Set,Ewsr1,Prpf38a,Nusap1,Espl1,Nme4,Prps1,Fgd3,Apitd1,Pbk,Synj2,Foxp1,Pold3,Anp32b,Dcbld1,Rfc2,Hirip3,Cenpk,Pola2,Whsc2,Incenp,Ppa1,Cebpz,Hnrpdl
**mmu-miR-31***	39(1239)	1,14E-06	Fmr1,Nol9,Zfp334,Rfwd3,Spag5,Tes,Aspm,Ilf2,Myef2,Fkbp5,Nuf2,Vrk3,BC055324,Hdac2,Bach1,Zmym1,Hmgb3,Pcnt,Rpl12,Hmgb2,Rpa2,Tpx2,Rbm17,Sfxn1,Nudt21,3110003A17Rik,Dut,Rif1,Nup155,Pkmyt1,Nolc1,Abl2,Fancd2,Arhgap19,Rsrc2,Stil,Mast4,Ddx39,Cdt1
**mmu-miR-200c***	42(1239)	1,14E-06	Ahsa1,Tmem39b,Trim24,Pwp2,Rbmxrt,Impa2,Caprin1,D19Bwg1357e,Depdc1b,Nol9,Gemin6,1110020G09Rik,Ddx46,6720463M24Rik,Mad2l1,Prim1,Mcm3,Hdac2,Dbr1,Ash2l,2610039C10Rik,Crlf1,Smc5,Crebzf,Depdc1a,Sf3a1,Palld,Asf1b,Pprc1,Psmd14,3110003A17Rik,Prmt5,U2af2,Wdr73,Cdc40,Plekha5,Anp32b,Lmnb1,Stmn1,1700054N08Rik,Smyd5,Eps8
**mmu-miR-532-5p**	47(1239)	1,17E-06	Rnmt,2810008M24Rik,Ripk2,Kif4,Riok2,Hspe1,Ptpre,Egr3,Lsm2,Tuba1c,Hspa14,Rpl30,Cd2ap,Larp7,Ncapg2,Ncapd2,2700029M09Rik,Mapk1,Recql4,Gtf2h2,Prpf4b,Stoml2,Rad54l,Nupl1,Rbm25,1110034A24Rik,2810055F11Rik,Slc9a3r1,Mpp6,Pvrl3,Jub,Kif2c,Mcm7,Neto2,Topbp1,Synj2,Akap8,Bhlhb9,Rfc2,Ppwd1,Trub1,Smu1,Khdrbs1,Smarcc1,Kif23,Hnrpdl,Socs4
**mmu-miR-24-1***	44(1239)	1,21E-06	Tgif2,Fancm,Smpdl3b,Atad2,Kl,1110020G09Rik,Fkbp5,Acaa2,Nup93,Glul,Zmym1,Hmgb3,Mapk1,Set,E130303B06Rik,Idh2,Gsg2,Edil3,Sgms1,H2afy,Nasp,Hyal2,Cobll1,Hspa4l,Cep70,Pkp2,Cachd1,Cenpn,Exosc2,Prmt5,Wdr73,Foxp1,Ddx20,Ncapg,Ddx10,Cd14,Rasal2,Incenp,Hells,Pebp1,Ccdc18,Ezh2,Fancc,Cenpi
**mmu-miR-375**	49(1239)	1,22E-06	Etaa1,Timm8a1,D19Bwg1357e,Plk4,Dctd,Sass6,Tcerg1,Rbbp7,Tmem176b,Nrp1,Lrp8,Ncapd2,Smc5,Zfp217,Arl4c,Kif21a,Pcnt,2210018M11Rik,Cbx3,Ptges3,Smndc1,Usp3,Ubqln2,Cdc7,Nr2f2,Pprc1,Tk1,Ptk7,Aifm1,Ctsh,Mtm1,Psmd14,Net1,Rbm17,Pvrl3,2610318N02Rik,Apitd1,Usp37,Slc43a1,Fancd2,Rfc2,Khdrbs1,Pebp1,Ppa1,Cdh10,Dock5,Tpbg,2810408A11Rik,Kif23
**mmu-miR-24-2***	44(1239)	1,26E-06	Nedd4l,Tgif2,Fancm,Plk1,Smpdl3b,Atad2,Med4,1110020G09Rik,Fkbp5,Zw10,Acaa2,Nup93,Glul,Zmym1,Set,E130303B06Rik,Idh2,Gsg2,Edil3,Sgms1,Nasp,Hyal2,Cobll1,Trim25,Hspa4l,Cep70,Arid4b,Pkp2,Cachd1,Cenpn,Prmt5,Wdr73,Foxp1,Ddx20,Syne2,Cd14,Rasal2,Incenp,Hells,Pebp1,Ccdc18,Ezh2,Fancc,Cenpi
**mmu-miR-486**	52(1239)	1,29E-06	Fmr1,Whsc1,Skp2,Acly,Rapgef2,Las1l,2010002N04Rik,Xpo7,Egr2,Ahctf1,Impdh2,BC055324,Mcm3,Fnbp1l,Luc7l,Armc8,Agxt2l2,Psrc1,Pla2g4a,Pde1a,Ncapd2,Psmc3ip,Atad5,Sf1,Stoml2,Palld,Tube1,Pttg1,Colec12,Hmga2,Ckap2,Top2a,Snrpd1,Unc5c,Trp53bp2,Rps13,4930427A07Rik,Mcm2,Cep55,Rrad,Stmn1,Specc1,Abl2,Fancd2,Dcbld1,Zcchc8,Ppwd1,Th1l,Pola2,E130308A19Rik,Kif23,Tcf4
**mmu-miR-676***	37(1239)	1,46E-06	Whsc1,Kif2a,Bclaf1,Dsn1,Ctsw,Ripk2,Wdr3,Vil1,E2f8,Rnps1,Ect2,Pgk1,Luc7l,Cand1,Aftph,Rpl30,Cct2,Pla2g4a,Lig1,Zc3h8,Nxf1,Stoml2,Palld,Colec12,Hmgb2,Hn1,2610301G19Rik,Synj2,Cdkn2c,Cct8,Spp1,Slc43a1,Sdpr,Mast4,Pola2,Khdrbs1,Nxt1
**mmu-miR-687**	45(1239)	1,46E-06	Ncaph,Tmem39b,Rbm26,Nup54,Whsc1,Kif2a,Tes,Nap1l4,Rpl7a,Cdc5l,Cdca5,Mrps6,Mtf2,Lcorl,Pgk1,Fnbp1l,Sox11,Slc29a1,Ncapd2,Cep152,B230120H23Rik,Pcnt,Satb2,Ptpn12,Hyal2,Ankrd57,Usp3,F630043A04Rik,Top2a,Rad18,Dut,Ttc3,Synj2,Slc43a1,Gprc5c,Bcl2l11,Cnn3,Gmeb1,Khdrbs1,Pebp1,Pou2f1,Nxt1,Cdh10,Smyd5,Slc4a7
**mmu-miR-450a-3p**	50(1239)	1,67E-06	Lrrc45,Ahsa1,Arhgap22,Trim24,Kif2a,Fosl1,Kif4,Bub1,Noc4l,Ddx46,Ndc80,Aspm,Vil1,Ptpre,Ilf2,Ank3,Fxn,Por,Mthfd1,Nudt1,Dusp5,Cep152,Rmi1,Zfp451,Slco4a1,Ncam1,E330009J07Rik,Gli2,Pprc1,Snrpg,Nme4,Pkp2,Psmd14,Cdca3,Wdr55,Actl6a,Synj2,Slco1a5,Ddx20,Pold1,Nob1,Pdss1,Sf3a3,Incenp,Ppa1,Nsl1,Cebpz,Ift74,Tpbg,Eif2s1
**mmu-miR-24**	48(1239)	1,68E-06	Nedd4l,Whsc1,Cks1b,Taf5,9030617O03Rik,Plk1,Prpf19,Med4,Elavl1,Ccdc45,Mup4,Cdc5l,Vrk3,Nol10,Ttll4,Tmem173,Nudt1,Ccdc99,Larp7,Pde1a,Dhodh,Lig1,Rad51c,Gtf2h2,Asf1b,Sema4b,Snrpg,Rfc5,Mcpt8,Cachd1,Ints7,Jub,Fgd3,Cct3,Mphosph10,Kcnk2,4930427A07Rik,Sertad1,2410016O06Rik,Cx3cl1,Slc43a1,Dcbld1,Tacc3,Pola2,H2afx,Pebp1,Eftud2,Smarcc1
**mmu-miR-369-5p**	48(1239)	1,68E-06	1110012J17Rik,Glrx,Zfp606,Ctsw,Sass6,Cpsf6,Fubp1,Wdr3,Tiam2,Dis3,Blm,D2Ertd750e,Cldn12,Cacybp,Smarce1,Eef1e1,Cct2,Setx,Pla2g4a,1700029F09Rik,Cbx1,Ccnb2,Gpbp1,Eif4a3,Lig1,Hnrpll,Hbs1l,Depdc1a,Stk17b,Ssb,Dhx15,Kars,Nupl1,Asxl1,Hspa4l,Syt17,Mat2a,Ptcd3,Ankrd28,Fzd3,Racgap1,Wdr55,Gphn,Synj2,Lmnb1,Gprc5c,4632434I11Rik,Cdh10

miRNA name	Number of Genes	Corrected p-value	miRNA targets among genes repressed in Rasless cells (Table S1)
mmu-miR-684	42(1239)	1,69E-06	6430527G18Rik,Prpf31,Fhl2,Ets1,Kif2a,1110020G09Rik,Egr3,Tiam2,Rad21,Klhdc2,Cdca2,Ccnb2,Bzw2,Ccnb1,Set,H2afy,Ssb,Lyar,E330009J07Rik,Pprc1,Rfc5,Zcchc2,Ankrd28,Zbtb12,Trpc2,Tnks2,Dapp1,Plscr1,Ssrp1,Specc1,Cd14,Rfc2,Mre11a,Ppwd1,Ptbp2,Pola2,Rad51,Dyrk3,Tmpo,Mrpl18,Hnrpdl,Zcchc11
mmu-miR-7a	48(1239)	1,74E-06	Gemin4,Prim2,Rbmxrt,Caprin1,Plk4,Sass6,Riok2,Xpo7,Ptpre,Parp1,Nt5dc2,Kif18a,Cdk2,Brca1,Msh3,Nek6,Psrc1,Pdk3,Cep152,Snrpa1,Ptgs2,Rps9,Cul4b,Tial1,Ssb,Asxl1,Gli3,Slc9a3r1,Rsf1,Dut,Cct3,Casp6,Dus3l,U2af2,Bnc2,Ssrp1,Nav3,Lmnb1,Stmn1,1110004E09Rik,Psmd5,Utp11l,Gmeb1,Hells,Pou2f1,Cdc73,Hnrpdl,Eif2s1
mmu-miR-297b-5p	44(1239)	1,83E-06	Gemin4,Prim2,Dhx9,Kif2a,Mum1,Nup37,Swap70,Ddx27,Kif18a,Msh6,Lcorl,Snrpb,Armc8,Pik3c2a,Cd2ap,Pla2g4a,Gjc1,Runx2,Mbtps2,Eif4a3,Kntc1,Baz1a,Idh2,Nupl1,Neil3,Dbf4,Unc5c,Paip1,Pkp2,Sip1,Casp8ap2,Eif4enif1,Casp6,Kcnk2,Nsmce4a,Usp37,Bnc2,Cep55,Slc43a1,Rrad,Cenpk,Lifr,Dyrk3,Ltbp1
mmu-miR-468	44(1239)	1,83E-06	H2afv,Dgcr8,Ranbp1,Impa2,Abi1,Llgl1,Cks1b,Elf2,Wdr43,Snapc3,Prdx4,Tpd52,Myef2,Cit,2510012J08Rik,Schip1,Ifitm3,Armc8,Cep57,Psrc1,Pde1a,Cep152,Baz1a,Hbs1l,Kpna2,Top2a,Syt17,Cep70,Psmd14,Cenpn,Slc38a1,Slc39a10,Usp37,Eif5,Slc43a3,E330016A19Rik,Cenpk,Rad51,Incenp,Hells,Egfl7,Ift74,Cdt1,AA408296
mmu-miR-488	44(1239)	1,83E-06	Ahsa1,Impa2,Dhx9,Fhl2,Depdc1b,Has2,Ndc80,Ets2,Egr3,Hspd1,Zw10,Msh6,Impdh2,Sin3a,Brca1,Mthfd1,Snrpb,Hdac2,Hspa14,Denr,Setx,Dhodh,B230120H23Rik,Prpf38a,Ankrd57,Asf1b,Ier2,Bub1b,Dbf4,Rad18,Trit1,Mat2a,Pvrl3,Foxp1,Cct8,Syne2,Rrad,B3galnt1,Zfp281,4632434I11Rik,Pou2f1,Ltbp1,Nxt1,Gtse1
mmu-miR-218	46(1239)	1,86E-06	Lrrc45,Prkar2b,Whsc1,Ktn1,Depdc1b,Smpdl3b,Kif4,U90926,Tyw3,Hspe1,Lrp8,Sltm,Acaa2,Slit2,Nup93,Asxl3,Slc29a1,Dusp5,Psmc3ip,D1Bwg0212e,Zc3h8,Parp12,Crebzf,Kpna2,Ncaph2,Ubqln2,Bub1b,2810055F11Rik,Hn1,Nmral1,Arid4b,Tpx2,Smchd1,Rfc3,Dapp1,Cct8,E2f7,Pum2,Cenpk,Th1l,Tnfaip6,Ccdc18,Cebpz,Nxt1,Ift74,2810408A11Rik
mmu-miR-509-3p	50(1239)	1,91E-06	Phf6,Prkar2b,Vegfc,Impa2,Abi1,Llgl1,Ccne1,Idh3a,Emb,Myef2,Ank3,Yme1l1,Fbxo5,Psrc1,Usp34,Zmynd19,Trip13,B230120H23Rik,Casc5,Pcnt,Crebzf,Csnk1g3,Cobll1,Dhx15,Asxl1,Neil3,Wee1,Tpx2,Psmd14,Mpp6,Iws1,Ptcd3,Sox4,Lrig1,Eif4enif1,Nucks1,Pbk,Synj2,Mboat2,Esf1,Sdpr,Ppwd1,Trub1,Th1l,Rpl13a,AI848100,Twistnb,Fancc,Slc4a7,Cirh1a
mmu-miR-714	50(1239)	2,06E-06	Rcc2,H2afv,Ell,Depdc1b,Cks1b,Tgfbr3,Tnnt2,Ogfrl1,Ctsw,Cbx2,Elp2,Tmem176b,Polr1e,2510012J08Rik,Zw10,Tubb5,Adamts7,Cct7,Dusp9,Fen1,D2Ertd750e,Pgk1,Zic1,Nudt1,Denr,Slc29a1,Micall2,Set,Ncapd3,Hbs1l,X99384,Ewsr1,Nup107,Lyar,Ckap2l,Sema4b,Gins4,2610301G19Rik,Zcchc2,Net1,Fgd3,Mybl2,Wdr73,Ssrp1,Rrad,Cd14,Rasal2,Bcl2l11,Sf3a3,Phlda1
mmu-miR-542-3p	49(1239)	2,17E-06	Gemin4,Pop1,Clns1a,Atad2,Ripk2,Med4,Noc4l,Aurka,Fkbp5,Sgol2,Ttll4,Msh6,Lcorl,Ppid,Cenph,Zfp184,Ccdc99,Pcna,Egfr,Rsbn1,H2afy,Tial1,Hyal2,Nxf1,Lyar,Hmgb1,Nusap1,Dbf4,Cep70,Unc5c,2810025M15Rik,Nup88,3110003A17Rik,Ahcy,Trpc2,Nsmce4a,Usp37,Plscr1,Cnot6,Fignl1,Cenpk,Ppwd1,Rad51,Ppa1,Nsl1,Nxt1,Ecd,Dmap1,Zcchc11
mmu-miR-293	47(1239)	2,28E-06	Nedd4l,Lrrc45,Asph,Fhl2,Depdc1b,Has2,Smpdl3b,Atad2,2010002N04Rik,Pspc1,Noc4l,Cep192,Impdh2,Nfyb,Luc7l,Msh3,Nudt1,Zmym1,Pde1a,Thsd7a,Crlf1,Ube2c,Spc25,Troap,Imp3,Cdc7,Uchl5,Adsl,Nme4,2610301G19Rik,Psmd14,2810025M15Rik,Pvrl3,Lrig1,Rad51ap1,Pbk,Tnks2,Ddx20,Tpp2,Rrad,Nav3,Pold1,Dcbld1,Rfc2,2610027L16Rik,Phlda1,H2afx
mmu-miR-654-3p	50(1239)	2,30E-06	Sap30,Asph,2700050L05Rik,Gmnn,Depdc1b,Elf2,Taf5,Atad2,Wdr5,Pnn,Ttll4,Msh6,Schip1,Tmem173,Rad21,Emg1,Larp7,Pde1a,Gins1,Kif20a,Npn2,Casc5,Set,Spc25,Sgms1,Wipf1,Nup107,Asf1b,Usp3,Gins4,Gli2,Unc13c,Tpx2,Rbmx2,Limd2,Nudc,Apitd1,Zrsr2,Gas2l3,Impa1,Cnot6,Rcl1,Arhgap19,Trub1,Bcl2l11,Pola2,Nol11,Me2,Cenpi,Vash2
mmu-miR-297c	45(1239)	2,36E-06	Prim2,Rnmt,Dhx9,Nup37,Aurkb,Ddx27,Tcerg1,Msh6,Mtf2,Rnf138,Lcorl,Snrpb,Armc8,Pik3c2a,Cd2ap,Pla2g4a,Brd8,Runx2,Mbtps2,Eif4a3,Rad51c,Prpf38b,Kntc1,Baz1a,Idh2,Cobll1,Neil3,Dbf4,Syt17,Paip1,Pkp2,Sip1,Wdr55,Eif4enif1,Casp6,Kcnk2,Nsmce4a,Usp37,Bnc2,Cep55,Slc43a1,Rrad,Cenpk,Lifr,Ltbp1
mmu-miR-680	48(1239)	2,44E-06	Ncaph,Trim24,Whsc1,Ifi205,Mum1,Fosl1,Elavl1,Pspc1,Elp2,5730590G19Rik,Xpo7,Cdc5l,Vrk3,Pdap1,Apex2,Brca1,BC055324,Cct5,Pcna,Sh3kbp1,Eme1,Micall2,Lig1,Set,Rsbn1,Troap,Sgms1,Ppp1cc,Hyal2,Sf1,Mcm10,Snw1,Pprc1,Tk1,Slc9a3r1,Plxnb1,Qars,Sox4,Fgd3,Casp6,Rfc3,Prmt5,Tnks2,Slc43a3,Stmn1,Fancd2,Arf6,Dock5
mmu-miR-710	46(1239)	2,45E-06	Rbmxrt,Impa2,Snx7,Mybbp1a,Sass6,Dnmt1,Cdc5l,Gspt1,2510012J08Rik,Larp7,Runx2,Scrib,Sf3a1,Nxf1,Mcm10,Nusap1,Kpna2,Cdc7,Nt5c3l,Melk,Mtm1,Tpx2,Pkp2,Igsf3,Nup88,Ddx11,Dut,Slc38a1,Ddx31,Pbk,Synj2,Impa1,Dapp1,Cnot6,Slc43a1,Zcchc8,Arhgap19,Sf3a3,Egfl7,Nsl1,Tmpo,Tpbg,Eftud2,Smarcc1,Kif23,Eif2s1
mmu-miR-470	43(1239)	2,50E-06	Lrrc45,Dhx9,Hspa8,Fosl1,Hmmr,Ppih,Srpk1,Ddx46,Mup4,Egr2,Ythdc1,Fxn,Mrps6,Sltm,Pdap1,Tgif1,Mthfd1,Pgk1,Tuba1c,Exo1,Enpp1,Sh3kbp1,Hmgb3,Eme1,Pcnt,Satb2,Sf1,Bcl7a,Kars,Traip,Espl1,Usp3,Top2a,Nt5c3l,Cct3,Nudc,Ddx31,Pbk,Mycn,Hirip3,Th1l,Mast4,Apex1
mmu-miR-805	44(1239)	2,75E-06	2810046L04Rik,Dgcr8,Rbmxrt,Kif2a,Ppih,Ccne1,Sass6,Las1l,Ythdf2,Cep192,Elp2,Tpd52,Mup4,6720463M24Rik,Hspd1,Wsb1,Zw10,Schip1,Tgif1,Junb,Msh3,Lin9,Cct2,Psrc1,Pdk3,Mbtps2,4930547N16Rik,Rad51c,Spc25,Larp4,Kpna2,Cdc7,Snrpd1,Dbf4,Melk,Ell2,Ly75,Dtymk,Stil,Ppwd1,Rpl13a,Utp11l,Pcf11,Rbm12
mmu-miR-503	48(1239)	2,84E-06	Trim24,Tnnt2,Spag5,Ccne1,Ythdc1,Zw10,Mrps6,Pdap1,Tubb5,Tmem173,Adamts7,Lcorl,Tll1,Gpd2,Ung,Larp7,Zmynd19,Psmc3ip,Zfp217,Pcnt,Sgms1,Wipf1,Spry2,Ipo7,Nupl1,Bub1b,Hspa4l,Cenpn,Pvrl3,Dut,Lrig1,Mphosph10,Ttc3,Prmt5,Ptrh2,Ilf3,Usp37,Ssrp1,Stmn1,Dcbld1,Rcl1,Mast4,Ddx39,Whsc2,Gart,Cenpi,Dnajc9,Eps8
mmu-let-7c	54(1239)	2,89E-06	Phf6,Rcc2,Dffb,Tnpo1,Prkar2b,Birc5,Ktn1,Pak1,Dsn1,Lrig3,Kif4,Noc4l,Bub3,Nuf2,Ttll4,Casp3,Prim1,Tubb5,Cct7,Fbxo5,Myc,Slc19a1,Trip13,Eme1,2610039C10Rik,Cep152,Dusp6,Pcnt,X99384,Sgms1,Gtf2h2,Lyar,Dhx15,Espl1,Ncaph2,Ckap2,Nme4,Qars,Prps1,Actl6a,Ncapg,Plscr1,Akap8,Fbxo32,D16Ertd472e,Nob1,1110004E09Rik,Ddx39,Sf3a3,Ezh2,Tpbg,Kif23,Zcchc11,Eps8
mmu-miR-146a	49(1239)	2,90E-06	Asph,Gemin4,Ercc6l,Fhl2,Pak1,Ripk2,Nap1l4,Strbp,Aspm,Parp1,Ahctf1,Tmem48,Schip1,Blm,Psrc1,Slc19a1,Emg1,Slc25a5,Adss,Cep152,Supt16h,Ccrn4l,Rps9,Satb2,H2-K1,Tial1,Prpf38a,Zfp451,Dhx15,Rad54l,Slbp,Card10,Usp3,Npm1,Pif1,Nt5c3l,Pkp2,Cachd1,Cdca3,Kif22,Actr3,Pum2,Abl2,Nob1,Dtymk,Rsrc2,Rasal2,Cdh10,Top1
mmu-miR-344	43(1239)	2,94E-06	Sap30,Hspa8,Cks1b,Nme1,Atad2,Sass6,Cit,Impdh2,Tubb5,Armc8,Agxt2l2,BC027072,Dusp4,Dhodh,Ccnf,Hn1l,Dusp6,Rpl12,Ssb,Usp6nl,Nfkbiz,Rasa1,Tead2,Nfx1,F630043A04Rik,2810025M15Rik,Racgap1,Dut,Rps13,Ilf3,H2afz,Ddx10,Bhlhb9,Specc1,Gprc5c,Tacc3,2610027L16Rik,Etv4,Egfl7,Nsl1,H2afy2,Rbpj,Smc4
mmu-miR-145	47(1239)	3,07E-06	Mcm5,Birc5,D19Bwg1357e,Fhl2,Tcf19,Zfp334,Smpdl3b,Las1l,Smc6,Pdia6,Myef2,Zw10,Pdap1,Fndc4,Sin3a,Pgk1,Myc,Dusp4,Cdc25a,Cep152,Rad51c,Ccrn4l,Kif21a,Pcnt,Rps9,Cul4b,H2afy,Nasp,Ankrd57,Bub1b,Arpc5,Nab2,Cenpn,3110003A17Rik,Jub,Topbp1,Wdr73,Ddx20,Prc1,Ruvbl2,Pold3,Yes1,Actr3,Gmeb1,Pou2f1,Rbm12,Nxt1
mmu-miR-196a*	38(1239)	3,08E-06	Nedd4l,Tnpo1,Errfi1,Marcksl1,Dsn1,Ccne1,Tes,Aspm,Ptpre,Egr3,Cdc5l,Aftph,Usp34,Pcnt,Wipf1,Nupl1,Pttg1,Ier2,Phf17,Adsl,Jub,Rbmx2,Dut,Zfp619,Mphosph10,Kcnk2,Neto2,Ilf3,Usp37,Mboat2,Ssrp1,Rrad,Fmnl3,Rfc2,Cdca8,Hells,Nxt1,Kif23
mmu-miR-880	43(1239)	3,18E-06	Khsrp,Ell,Amd1,Dcp1a,Dctd,Ddx52,Vrk3,Ahctf1,Slc11a2,Lrp8,Acaa2,Suv39h1,Snrpb,Fbxo5,Msh3,Cep57,Rpl3,Setx,Pde1a,2700029M09Rik,Lig1,Snrpa1,Rpl12,Hyal2,Trim25,2810474O19Rik,Snw1,Nfkbiz,Gins4,Hmgb2,Cep70,Tpx2,Limd2,Chtf18,Smarca5,Lmnb1,Rasal2,Hells,Pou2f1,Fancc,Ltbp1,2810408A11Rik,Pogz

miRNA name	Number of Genes	Corrected p-value	miRNA targets among genes repressed in Rasless cells (Table S1)
**mmu-miR-881**	41(1239)	3,21E-06	Gemin4,D19Bwg1357e,Wdr43,Plk4,Kif4,Myef2,Ythdc1,Kif18a,Tll1,Stip1,Pde1a,Kif20a,Abcb1b,Sbno1,Rad54l,Mcm10,Smc3,Npm1,Pif1,Eno3,Top2a,Neil3,Nt5c3l,Arid4b,Paip1,Ctsh,Net1,Pole,Pigf,Sip1,Kif22,Sertad1,Tpp2,Plekha5,Spp1,Spc24,Fbxo32,Abl2,Trub1,Map4k5,Pebp1
**mmu-miR-297a**	45(1239)	3,47E-06	Prim2,Tnpo1,Dhx9,Aurkb,Ddx27,Msh6,Mtf2,Rnf138,Lcorl,BC055324,Snrpb,Armc8,Rpl30,Pik3c2a,Cd2ap,Pla2g4a,Gjc1,Runx2,Mbtps2,Eif4a3,Rad51c,Kntc1,Baz1a,Idh2,Cobll1,Nupl1,Neil3,Dbf4,Paip1,Pkp2,Sip1,Eif4enif1,Casp6,Kcnk2,Cenpl,Nsmce4a,Usp37,Bnc2,Cep55,Slc43a1,Rrad,Cenpk,Ltbp1,Tpbg,Tdrkh
**mmu-miR-470***	40(1239)	3,73E-06	Ncaph,Cdca7l,Sap30,Cenpc1,Kif2a,Rdx,Riok2,Ube2e3,Marcks,Cdc5l,Nuf2,Hspd1,Casp3,Sltm,Cct7,Dusp9,Nudt1,Rcc1,Rpl30,Pcna,Psrc1,Serbp1,Cbx1,Eif4a3,H2-K1,Ewsr1,Acsl3,Ctsh,2810025M15Rik,Ptcd3,Cd24a,Slc38a1,Cenpe,Mlf1ip,Ptrh2,Gprc5c,Fancd2,Trub1,2610027L16Rik,Nxt1
**mmu-miR-337-5p**	46(1239)	3,73E-06	Sap30,Prim2,Cenpc1,Depdc1b,Nol9,Eef1g,Glrx,Plk1,Ccne1,Wdr3,Cep192,Cdc27,Ube2e3,Msh2,Alad,Aebp2,Sox11,Ncapd2,Pkn3,Psmc3ip,Mpzl1,Gsg2,Atad5,Wipf1,Kars,Nfkbiz,Syt17,Dut,Nutf2,2410016O06Rik,Ilf3,Rbm14,Foxp1,Plekha5,Cep55,Slc43a1,Syne2,Slc43a3,Rrad,Stmn1,C79407,Nob1,Rasal2,H2afx,Incenp,Kif23
**mmu-miR-196b**	52(1239)	3,74E-06	Etaa1,Rbm26,Rcc2,Hspa8,Elf2,Zfp606,Ccne1,Rdx,Rbbp7,Aspm,Myef2,Parp1,Ttll4,Tmem48,Casp3,Schip1,Ebf2,BC055324,Psrc1,Slc25a5,Dusp5,Casc5,Ncapd3,Rsbn1,Imp3,Rpl12,Nxf1,Espl1,Ier2,Snw1,Sumo2,Nme4,Nt5c3l,Paip1,Chd2,Trp53bp2,Zfp619,Kif22,Actl6a,Wdr73,Cdkn2c,Dapp1,Syne2,Slc43a3,Tacc3,Ptger4,Timp1,Ezh2,Mrpl18,Tpbg,Cirh1a,Pogz
**mmu-miR-669b**	49(1239)	3,85E-06	Nedd4l,Ncaph,Ahsa1,Cenpc1,Spred2,Kif2a,Plk4,Smpdl3b,Lrig3,Sass6,Aurkb,Ddx27,Exosc8,6720463M24Rik,E2f8,Myef2,Ank3,Tiam2,Msh3,Sgol1,B230120H23Rik,Casc5,Shcbp1,Gtf2h2,Snrpg,Mcpt8,Nme4,Mtm1,Psmd14,Zwilch,Ube2t,3110003A17Rik,Patz1,Actl6a,Ncapg,Tpp2,Cep55,Mcm6,Dcbld1,Lrrc8c,Nob1,Rasal2,Cenpk,2610027L16Rik,Pcf11,Me2,Pou2f1,Mrpl18,Tdrkh
**mmu-miR-98**	46(1239)	3,85E-06	Rcc2,Prkar2b,Birc5,Taf5,Plk4,Prdx4,Lrig3,Fubp1,Las1l,Noc4l,Rdx,Pgm1,Ttll4,Casp3,Tubb5,Brca1,Cct7,Rrm2,Myc,Eme1,Trim28,Lbr,Casc5,Pcnt,X99384,Gtf2h2,Ptpn12,Rad54l,Espl1,Ckap2,Neil3,Nme4,Qars,Dus3l,Actl6a,Akap8,Ly75,Nob1,1110004E09Rik,Ddx39,Sf3a3,Ezh2,Fancb,Nxt1,Kif23,Tcf4
**mmu-miR-134**	45(1239)	3,87E-06	H2afv,Hspa8,Wdr43,Ogfrl1,Idh3a,Elavl1,Xkr5,Egr3,Myef2,Als2cr12,2510012J08Rik,Armc8,Myc,Asxl3,Ppil1,Ssr4,Psrc1,Zmynd19,Slc25a5,Ctnna1,Cep152,Idh2,Tslp,Sgms1,2810055F11Rik,Prpf3,Rfc5,Casp8ap2,Cenpf,Il1rap,Patz1,Nutf2,Ilf3,Cct8,Anp32b,Bnc2,Syne2,Bhlhb9,Cenpk,Ppwd1,Pola2,Egfl7,Cdca7,Rfc4,Mrpl18
**mmu-miR-677**	44(1239)	4,03E-06	Phf6,Ankrd32,Fus,Sass6,1110020G09Rik,Cep192,Egr3,Cdc5l,Sgol2,Hspd1,Cct7,BC055324,Agxt2l2,Ctbp2,Ung,Gins1,Trim28,Dhodh,Zfp217,Recql4,Imp3,Rpl12,Stoml2,Ckap2l,Rbm17,Cenpn,Isg20l2,Cd24a,Fgfr1op,Fbln1,BC016423,Cdkn2c,Rif1,Dapp1,Spp1,Pafah1b3,Abl2,Dcbld1,Ppwd1,Vbp1,Lifr,Khdrbs1,Dyrk3,4632434I11Rik
**mmu-miR-125b***	43(1239)	4,37E-06	Tmem39b,Rbmxrt,Marcksl1,Tcf19,Wdr43,Smpdl3b,Ppih,Rangap1,Riok2,Cdc5l,Nuf2,Api5,Dhx36,Prim1,Nfyb,Tfdp1,Cd2ap,Iqgap3,Gnb4,Prpf4b,Slbp,Ier2,Bub1b,Arid4b,Slc9a3r1,Egr1,Nup88,Rbmx2,Socs6,Clspn,Prmt5,Polr3b,Sertad1,Cdkn2c,Ddx20,Osmr,Ruvbl2,Pum2,Stmn1,Gprc5c,Cnn3,Nsl1,Eif2s1
**mmu-let-7i**	54(1239)	4,41E-06	Etaa1,Arhgap22,Rcc2,Birc5,Ktn1,Pak1,Dsn1,Ctsw,Prdx4,Lrig3,Bub3,Rdx,Pgm1,Tpd52,Rbbp7,Aspm,Tmem176b,Gspt1,Ttll4,Casp3,Acaa2,Prim1,Tubb5,Rrm2,Luc7l,Myc,Cep152,Casc5,Bzw2,Idh2,Imp3,Pola1,Ptpn12,Dhx15,Espl1,Nme4,Mtm1,Qars,Pvrl3,Prps1,Actl6a,Slc43a3,Rrad,Spc24,Akap8,Nob1,1110004E09Rik,Egfl7,Ezh2,Fancb,E130308A19Rik,Fancc,Kif23,Tcf4
**mmu-miR-509-5p**	49(1239)	4,41E-06	Trim24,Dnaja2,Whsc1,Cks1b,Spag5,Setdb2,Pdia6,Als2cr12,Tmem173,Sox11,Msh3,Tfdp1,Rab15,Tll1,Usp1,Sms,Scarb1,Cep152,Supt16h,Peg12,Lig1,Mpzl1,Arl4c,Spc25,H2-K1,Rpl12,Hyal2,Slco4a1,Larp4,Hn1,Mcpt8,Qars,Cdca3,Exosc2,Slc25a24,Wdr73,Sertad1,Cacna1c,Dapp1,Ssrp1,Esf1,Rfc2,Fignl1,Rpl13a,Pola2,Dyrk3,Cdca7,Arf6,Kif23
**mmu-miR-489**	46(1239)	4,45E-06	Gmnn,Ets1,Llgl1,Snx7,Acly,Dsn1,Ctsw,Paxip1,Wdr3,1110020G09Rik,Cdc5l,Ank3,Nuf2,Sltm,Cct7,Hdac2,Klhdc2,Hspa14,Enpp1,Slc29a1,2610039C10Rik,Mrpl19,Sdad1,Polr2b,Sgms1,Hmgb1,Kin,Ints5,Pvrl3,Wdr55,Trp53bp2,Nde1,Patz1,Kif22,Smc2,Mastl,Eif5,Gprc5c,Rfc2,Ptbp2,Thoc2,Uhrf1,Cdh10,Mrpl18,Kif23,Hnrpdl
**mmu-miR-539**	40(1239)	4,53E-06	Etaa1,Ncaph,Ahsa1,Whsc1,Smpdl3b,Emb,Las1l,Strbp,1110020G09Rik,Dnmt1,Mup4,Kif18a,Ttll4,Brca1,Hdac2,Zfp184,Ncapd3,Snrpa1,H2-K1,2210018M11Rik,Tial1,Kars,Cachd1,Mpp6,Pigf,Pvrl3,Fgf7,3110003A17Rik,Cenpf,Patz1,Prkg2,Zbtb12,Ptrh2,Foxp1,Spp1,Slc43a3,Rasal2,Rpl13a,AI848100,Ezh2
**mmu-miR-455***	37(1239)	4,54E-06	Asph,Timeless,Rcc2,Prim2,Kif2a,Prpf19,Paxip1,E2f8,Zw10,Msh6,Tubb5,Por,Nup93,Nfatc2ip,Nudt1,Agxt2l2,Zmym1,Cbx1,Gtf2h2,Lrrk1,Ewsr1,Kars,Usp3,Nfkbiz,Neil3,Pkp2,Pvrl3,Casp6,Zrsr2,Fbln1,Ahcy,Synj2,Bnc2,Rcl1,Ppwd1,Lif,Socs4
**mmu-miR-654-5p**	49(1239)	4,55E-06	Asph,Rbm26,Rcc2,Trim24,Taf5,Wdr43,Sass6,Wdr3,Noc4l,Rdx,Tiam2,Mcm4,Sltm,Tuba1c,Nudt1,Ctbp2,Pdk3,Slc25a5,Ccnb2,Dhodh,Spc25,Casp8,Pola1,Crebzf,Wipf1,Dhx15,Syt17,Unc13c,Psmd14,Nab2,Chd2,Ddx11,Sip1,Cdca3,Pvrl3,Tinf2,Abhd10,2410016O06Rik,Mastl,Plekha5,Slc43a3,E2f7,Mrpl50,2610027L16Rik,Phc2,Ppa1,Fancb,2010204K13Rik,Zcchc11
**mmu-miR-129-3p**	47(1239)	4,56E-06	Tmem39b,Zfp292,Trim24,Ercc6l,Dhx9,D19Bwg1357e,Acly,Srpk1,Rpl7a,Egr3,Cpsf4,Nfatc2ip,Msh3,Nudt1,Cand1,Cacybp,Egfr,Slc29a1,Micall2,Abcb1b,Supt16h,B230120H23Rik,Mpzl1,Ccnf,Ccng2,Rpl12,Sf1,Nxf1,Stoml2,Rad54l,Dbf4,Rfc5,Gli3,Pigf,Jub,Patz1,Casp6,Nudc,Ddx31,Ly75,Etv5,Incenp,Dyrk3,Mrpl18,Eftud2,2810408A11Rik,Slc4a7
**mmu-miR-499**	44(1239)	4,64E-06	1110012J17Rik,Tmem39b,Pop1,Ercc6l,Amd1,2810008M24Rik,Trim59,Atad2,Aurkb,Dnmt1,Ube2e3,6720463M24Rik,Nuf2,Dis3,Fxn,Blm,Mthfd1,Rnps1,Sfpq,Rad21,Cep57,Zfp184,Enpp1,Pcna,Cd2ap,Snrpa1,Rpl12,Ewsr1,Prpf38a,Usp6nl,Zfp54,Mbd4,Tpx2,Pvrl3,Tipin,Mastl,H2afz,Slc43a1,2610027L16Rik,1110004E09Rik,Cnn3,Zfp281,Socs4,Pogz
**mmu-miR-879**	43(1239)	5,26E-06	Etaa1,Ahsa1,Arhgap22,Whsc1,Ccbe1,Ppih,Prpf19,Aurkb,1110020G09Rik,Noc4l,Riok2,Ube2e3,Ilf2,Ttll4,Mthfd1,Msh3,Myc,Gins1,2610039C10Rik,Kif20a,Pkn3,Dhodh,Eif4a3,Parp12,Rpl12,Spry2,Espl1,Hmgb2,Mbd4,Sumo2,Cep70,Nme4,Aifm1,Tpx2,Mphosph10,Spp1,Slc43a1,Gprc5c,Rfc2,Utp11l,Gtse1,Cdt1,Mrpl18
**mmu-miR-742**	46(1239)	5,34E-06	Cenpc1,Tnpo1,Birc5,Pak1,Plk4,Swap70,Ddx27,6720463M24Rik,Ruvbl1,Cdc5l,Fkbp5,Zc3h15,Hspa14,Cand1,Usp34,Pkn3,Lbr,Iqgap3,Gsg2,Hbs1l,Ptpn2,Tube1,Cdc7,Pprc1,Arid4b,Ctsh,Prr11,Dus3l,Bbs12,Esco2,Smarca5,Rbm14,Slc43a1,Nob1,1110004E09Rik,Bcl2l11,Twistnb,Hells,4632434I11Rik,Fancc,Cdt1,Rbpj,Siva1,Dnajc9,Slc4a7,Rrs1
**mmu-miR-335-5p**	48(1239)	5,76E-06	Sap30,Tmem39b,Pcm1,Prim2,Clns1a,Rbmxrt,Cpsf2,Gmnn,Ktn1,Kif2a,Plk4,Spag5,Kif4,Gspt1,2510012J08Rik,Rad21,Dbr1,Etf1,Cand1,Zfp184,Pcna,Zmym1,Trim28,Pcnt,Idh2,Gsg2,Prpf4b,Fanca,Tial1,Slbp,Ubqln2,Rasa1,Hmgb2,Pvrl3,Gemin8,Ddx31,Nsmce4a,Ilf3,Ssrp1,Bhlhb9,Tacc3,Ptbp2,Atrx,Bcl2l11,Tnfaip6,Cdh10,Rbpj,Wdr36
**mmu-miR-380-3p**	42(1239)	6,18E-06	Nedd4l,Asph,Gemin4,D19Bwg1357e,Zfp52,Txnrd1,Nap1l4,Cenpj,Pnn,Nrf1,Msh6,Schip1,Prim1,Dbr1,Cenph,Sh3kbp1,Zfp36l2,Slc25a5,Baz1a,Polr2b,2310057M21Rik,Edil3,Gtf2h2,Rpl12,Slco4a1,Nupl1,Colec12,Cdc7,Snrpd1,Hat1,Sip1,3110003A17Rik,Patz1,Casp6,Rad51ap1,Cep170,Th1l,Cnn3,Cd300lb,C330027C09Rik,4632434I11Rik,Tpbg
**mmu-miR-708**	42(1239)	6,41E-06	Shmt1,Whsc1,Spag5,Plk1,Ap1s3,Strbp,Vrk3,Snrpb,Pcna,Zmym1,Ung,Emg1,Eif4a3,Set,Ncapd3,Ube2c,Idh2,Hbs1l,Imp3,Ssb,Smc3,Hmgb2,Rpa2,Trit1,Ddx11,3110003A17Rik,Slc38a1,Ttc3,Ddx31,Yes1,Ssrp1,Fmnl3,Specc1,Gprc5c,Rfc2,Nob1,Trub1,Twistnb,Ccdc18,Mrpl18,Kif23,Cirh1a

miRNA name	Number of Genes	Corrected p-value	miRNA targets among genes repressed in Rasless cells (Table S1)
**mmu-miR-138***	38(1239)	6,42E-06	Asph,Noc4l,Ube2e3,Ilf2,Acaa2,Lcorl,Ppid,Fnbp1l,Rab15,Asxl3,Scarb1,Cct2,Hmgb3,Cbx1,Dusp5,Pkn3,Ppp1cc,Nusap1,Larp4,Nfkbiz,Hspa4l,F630043A04Rik,Tipin,Patz1,Pbk,U2af2,2410016O06Rik,Plekha5,Tacc3,Nob1,Fignl1,Etv4,Apex1,Incenp,Hells,Cdca7,2010204K13Rik,Mrpl18
**mmu-miR-715**	47(1239)	6,54E-06	6430527G18Rik,Tgif2,2700050L05Rik,Prkar2b,Whsc1,Ktn1,Tcf19,Cks1b,Pak1,Kif4,Noc4l,Xpo7,Msh2,Rest,Nrp1,Adamts7,Apex2,Hdac2,Klhdc2,Enpp1,Zmynd19,Brd8,Psmc3ip,Lig1,E130303B06Rik,Snrpa1,Kif21a,Cdc7,Kpnb1,Ctsh,Tpx2,Pkp2,Nab2,Fgd3,Cct3,Gphn,Nudc,Apitd1,Wdr73,Cacna1c,Nav3,Mcm6,Rcl1,Cenpk,Egfl7,Ppa1,Rbpj
**mmu-miR-29a***	44(1239)	6,58E-06	Pcm1,Impa2,Gemin6,Phactr4,Las1l,Cep192,Rdx,Pdia6,Ndc80,6720463M24Rik,Msh2,Ank3,Tmem48,Pgk1,Zfp184,Eef1e1,Setx,B230120H23Rik,Ptgs2,Pcnt,Gsg2,Nasp,Gins4,Hspa4l,Ckap2,Nfx1,Mcpt8,Pkp2,Psmd14,2810025M15Rik,Ptcd3,Fgf7,Igf2bp1,Trp53bp2,Actl6a,Synj2,Cdc40,Slco1a5,Spc24,Rpl13a,Ccdc18,Tmpo,Wdr36,Zcchc11
**mmu-miR-466c-5p**	58(1239)	6,75E-06	Prim2,Ell,Prx,Dhx9,Steap1,Mum1,Ctsw,Phactr4,Elavl1,Aspm,E2f8,Ank3,Kif18a,Yme1l1,Mtf2,Lcorl,Dusp9,Nfatc2ip,Cep57,Zfp184,Rpl30,Pik3c2a,Usp34,Gjc1,Mbtps2,Kntc1,Ube2c,Kif21a,Gtf2h2,Sbk1,Nusap1,Nfx1,Neil3,Sumo2,Snrpg,Aifm1,Mtm1,Cenpn,Wdr55,Casp6,Kcnk2,Mlf1ip,Polr3b,Tnks2,Ch25h,Usp37,Prc1,Bnc2,Cep55,Plscr1,Slc43a1,Rrad,Specc1,Cdca8,Gmeb1,Tmpo,Ltbp1,Eps8
**mmu-miR-876-3p**	48(1239)	6,83E-06	4930422G04Rik,Ktn1,Depdc1b,Kif2a,Mki67,Wdr3,Cep192,Tcerg1,Rest,Ahctf1,Mcm4,Ppid,Cenph,Tll1,Cand1,Agxt2l2,Denr,Dusp5,Gtf2h2,Ptpn2,Ewsr1,Myo1b,Pttg1,Gins4,Itgav,Phf17,Mmd,2810055F11Rik,Tk1,Nme4,Hat1,Pole,Iws1,3110003A17Rik,Esco2,Actl6a,Slc43a3,Nav3,Lmnb1,Tacc3,Cep170,Ppwd1,Me2,Hells,Cebpz,Dock5,Dnajc9,Ecd
**mmu-miR-34c**	49(1239)	7,41E-06	Pcm1,Mtmr4,Gmnn,Prpf40a,Tnnt2,Ctsw,Ythdf2,Noc4l,Exosc8,Casp3,Acaa2,Tuba1c,Slc29a1,Cep152,B230120H23Rik,Bzw2,Ncapd3,Ptpn12,Slco4a1,Snw1,Pif1,Eno3,Nme4,Nt5c3l,Paip1,Pigf,Igf2bp1,Ppif,Gemin8,Apitd1,Ahcy,Nsmce4a,Cdkn2c,Usp37,Foxp1,Ssrp1,Nav3,Lmnb1,Ell2,Fmnl3,Litaf,Dtymk,Arhgap19,Ppwd1,Rpl13a,Zfp281,Ccdc18,Ezh2,2810408A11Rik
**mmu-miR-27b***	41(1239)	7,47E-06	Asph,Rbm26,Ercc6l,Impa2,Als2cr12,Slc11a2,Cct7,Ebf2,Pgk1,Dbr1,Msh3,Pcna,Psrc1,Zmym1,1700029F09Rik,Gjc1,B230120H23Rik,Ube2c,Snrpa1,Spc25,Satb2,Rsbn1,Sgms1,Prpf38a,Gins4,Hspa4l,Syt17,Cdca3,Wdr55,Cct3,Eif4enif1,Xpo1,Nutf2,Nudc,Tnks2,Ch25h,Nav3,Fancd2,Nob1,Rpl13a,Tmpo
**mmu-miR-7b**	46(1239)	7,95E-06	Prim2,Rbmxrt,Snx7,Plk4,2610101N10Rik,Ptpre,Parp1,Nt5dc2,Slc11a2,Fndc4,Tmem173,Cdk2,Brca1,Msh3,Psrc1,Pdk3,Abcb1b,Cep152,Ncapd3,Snrpa1,Ptgs2,Rps9,Cul4b,Tial1,Asxl1,Rfc5,Gli3,Slc9a3r1,Tpx2,Rsf1,Cct3,Xpo1,Neto2,Topbp1,Fbln1,Pbk,Bnc2,Ssrp1,Nav3,2610027L16Rik,Psmd5,Utp11l,Hells,Pou2f1,Cdc73,Eif2s1
**mmu-miR-125b-3p**	51(1239)	7,97E-06	Nedd4l,Gata2,D19Bwg1357e,Gmnn,Mum1,Nipsnap1,Dnmt1,Aurka,Ahctf1,Impdh2,Por,Cdk2,Adamts7,Ppid,Msh3,Etf1,Tll1,Myc,Egfr,Pde1a,Cep152,Eif4a3,Kif21a,Troap,Ptpn2,Casp8,H2-K1,Crebzf,Prpf38a,Rnaseh2a,Gli2,Pprc1,Itih2,Trit1,Prmt5,Neto2,Wdr73,Dapp1,5730559C18Rik,Mcm2,B3galnt1,Mcm6,Cd14,Gprc5c,Fignl1,Mre11a,Rasal2,2610027L16Rik,Phc2,Tpbg,Eftud2
**mmu-let-7g**	51(1239)	8,21E-06	Phf6,Rcc2,Birc5,Ktn1,Elf2,Dsn1,Prdx4,Lrig3,Kif4,Bub3,Rdx,Pgm1,Riok2,Gspt1,Epc2,Ttll4,Casp3,Acaa2,Tubb5,Rrm2,Fbxo5,Myc,Slc19a1,Eme1,2610039C10Rik,Abcb1b,Cep152,Casc5,Bzw2,Polr2b,Imp3,Ptpn12,Espl1,Bub1b,Nme4,Mtm1,Qars,Pvrl3,Nudt21,Kif2c,Actl6a,Slc43a3,Spc24,Bhlhb9,D16Ertd472e,Nob1,1110004E09Rik,Ddx39,Ezh2,Kif23,Tcf4
**mmu-miR-10b***	41(1239)	8,70E-06	Impa2,Whsc1,Ppih,Rpl7a,Riok2,Ube2e3,Pom121,Parp1,Slc11a2,Casp3,Hdac2,Fbxo5,Dbr1,Zfp184,Larp7,Eme1,Gins1,Dhodh,Ccnf,Cul4b,Rsbn1,Sbno1,Lrrk1,Hmga2,Gli2,Apitd1,Kcnk2,U2af2,Nsmce4a,Ilf3,Ddx20,Mycn,Syne2,Lmnb1,Cenpk,Ppwd1,Mast4,1110004E09Rik,Phlda1,Arf6,Cirh1a
**mmu-miR-200b***	40(1239)	8,72E-06	Ahsa1,Phf6,Trim24,Whsc1,Tcf19,Gemin6,Swap70,Cep192,Cct7,Fen1,Ppid,Pgk1,Cenph,Cbfb,2610039C10Rik,Dusp5,Abcb1b,Set,Cul4b,Rmi1,Lrrk1,Mcm10,Pprc1,2610301G19Rik,Mpp6,Pvrl3,Exosc2,Slco1a5,Impa1,Lmnb1,Stmn1,Dcbld1,Rfc2,Cenpk,Egfl7,Cdh10,Cdt1,Smyd5,Eps8,Tdrkh
**mmu-miR-686**	42(1239)	8,72E-06	Phf6,Timeless,Rcc2,Prim2,Cbx2,Wdr3,D2Wsu81e,Fkbp5,Prim1,Brca1,Tuba1c,Usp34,Zmynd19,Eme1,Slc25a5,Imp3,Pnpt1,Rpl12,Ewsr1,Rbm25,Npm1,Cep70,Nmral1,Igsf3,Ints7,Isg20l2,3110003A17Rik,Trp53bp2,Limd2,Lrig1,Cnnm4,Apitd1,Kcnk2,Prmt5,Suv39h2,Mcm6,Hirip3,Ppwd1,Th1l,Phlda1,Incenp,Fancc
**mmu-miR-22***	40(1239)	9,45E-06	Tnpo1,Skp2,Nol9,Mybbp1a,2010002N04Rik,Ddx52,Mthfd1,Fen1,Ppid,Cdca2,Slc29a1,Gpbp1,Pcnt,Sgms1,Gtf2h2,H2-K1,Tial1,Cobll1,Kars,Larp4,Bub1b,Pprc1,Unc13c,Gli3,2610301G19Rik,Paip1,Chd2,Cenpn,Trp53bp2,Topbp1,Zrsr2,Wdr73,Syne2,Slc43a3,Incenp,Pou2f1,Nsl1,Tmpo,Cdc73,Eftud2
**mmu-miR-197**	43(1239)	1,01E-05	Nck2,Pcm1,Rbm26,2700050L05Rik,Nol9,Eef1g,Mki67,Prpf19,Ddx27,Ddx52,Xpo7,Ptpre,Msh6,Taf5l,Nfrkb,Rnps1,Scarb1,Cdc25a,Pde1a,Ctnna1,Chst1,Rad51c,Ptgs2,Pcnt,X99384,Pola1,Traip,E330009J07Rik,Gli2,Dbf4,Trit1,Exosc2,Sertad1,Anp32b,Cep55,Syne2,E330016A19Rik,Rasal2,Eif4h,Phlda1,Wdhd1,Terf1,Wdr36
**mmu-miR-485***	38(1239)	1,01E-05	Rbm26,Clns1a,Prx,Upf3b,Mum1,Plk4,Ctsw,Nrp1,Slc11a2,Fxn,Nfyb,Mcm3,Rab15,Ncapd3,Pcnt,2210018M11Rik,Rpl12,Kars,Espl1,Zfp53,Axl,Sip1,Pvrl3,Nudt21,Socs6,Gnl3,Nsmce4a,Cnot6,Nav3,Rfpl4,Dcbld1,Lrrc8c,Etv5,Pola2,Egfl7,Fancc,Kif23,Socs4
**mmu-miR-220**	53(1239)	1,01E-05	H2afv,Cenpc1,Tnpo1,D19Bwg1357e,Depdc1b,Nol9,Spred2,Acly,Ddx52,Xpo7,Nrf1,Mad2l1,Fkbp5,Nrp1,2510012J08Rik,Sltm,Taf5l,Enpp1,Gins1,Ncapd2,Ncapd3,Kif21a,Imp3,Ddx51,Depdc1a,Ipo7,Tardbp,Nap1l1,F630043A04Rik,Mbd4,Wee1,Unc5c,Hat1,Plxnb1,2810025M15Rik,Fzd3,Racgap1,Fgd3,Ss18,Eif4enif1,Trpc2,Slco1a5,Ilf3,Gprc5c,Dcbld1,Rasal2,Pola2,Nsl1,Cebpz,Slc4a7,Wdr36,1810011O10Rik,Pogz
**mmu-miR-206**	42(1239)	1,14E-05	Nedd4l,Asph,Prim2,Ktn1,Fubp1,Pspc1,Cep192,Elp2,Pgm1,Aspm,Ilf2,Cdc5l,Myef2,Hspd1,Nol10,Zw10,Pla2g4a,Pde1a,Slc25a5,Adss,Zfp217,Satb2,Casp8,Pola1,Dhx15,Rasa1,Snrpd1,Dbf4,Sumo2,Pvrl3,3110003A17Rik,Jub,Cenpe,Ss18,Tpm3,Actl6a,Plscr1,Rcl1,Cnn3,Cdca7,Fancb,Cebpz
**mmu-miR-708***	38(1239)	1,14E-05	Nono,Cenpc1,Mtmr4,Ktn1,Fus,Prpf19,Ddx27,Myef2,Casp3,Blm,Tgif1,Sfpq,Rpl30,Gins1,Ccnb2,Rps9,Satb2,H2afy,Fanca,Tial1,Pttg1,Usp3,E330009J07Rik,Bub1b,Pigf,Cct3,Nde1,Cenpl,Impa1,Dapp1,Cnot6,Bhlhb9,Cdca8,Whsc2,Incenp,Zfp281,Ccdc18,Smc4
**mmu-miR-202-3p**	45(1239)	1,16E-05	Snx5,Nedd4l,Ahsa1,Ercc6l,Zfp334,Kif2a,Dnmt1,Ddx52,Lcorl,Dusp9,Tuba1c,Fbxo5,Klhdc2,Pde1a,Gins1,2610039C10Rik,Pdk3,1700029F09Rik,Casc5,Ccrn4l,Spc25,Idh2,Cul4b,Pnpt1,Prpf38a,Sf3a1,Nusap1,Itgav,Nr2f2,2810055F11Rik,Nme4,Nt5c3l,Hat1,Pvrl3,3110003A17Rik,Abhd10,Actl6a,H2afz,Cep55,Ddx10,Abl2,Rcl1,Dtymk,Eif2s1,Socs4
**mmu-miR-704**	42(1239)	1,28E-05	Arhgap22,Cenpc1,Ifi205,Depdc1b,Nme1,Plk1,Smpdl3b,Dctd,Ccne1,Mnd1,Rpl7a,Vil1,Tmem176b,Gspt1,Mrps6,Cdca2,Setx,Pla2g4a,Larp7,Pde1a,1700029F09Rik,Zc3h8,Shcbp1,Ccng2,Sbno1,Tial1,Cbx3,Wdr75,Syt17,Mbd4,Pank4,Wdr55,Isg20l2,Rbmx2,Cnnm4,Tpp2,Rcl1,Dtymk,Arhgap19,Th1l,Ppa1,Rbpj
**mmu-miR-503***	33(1239)	1,32E-05	Caprin1,Kif2a,Tnnt2,Ccne1,Rdx,D2Wsu81e,E2f8,Tiam2,Impdh2,Taf5l,Lcorl,Lsm2,Klhdc2,Cep152,Shcbp1,Pnpt1,Ewsr1,Ubqln2,Gins4,2810055F11Rik,Tk1,Cep70,Nme4,Patz1,Tpm3,Foxp1,Nav3,Mcm6,Cep170,Atrx,Incenp,Gart,Ccdc18
**mmu-miR-341**	44(1239)	1,32E-05	Timeless,Rnmt,Depdc1b,Cks1b,Wdr82,Dctd,Egr3,Dis3,Nt5dc2,2510012J08Rik,Ppil5,Zw10,Nfrkb,Rnps1,BC055324,Fnbp1l,Cand1,Agxt2l2,Zmym1,Hmgb3,Setx,Lig1,Satb2,Nxf1,Nupl1,Ncam1,Gins4,Gli2,Snrpd1,Egr1,Pank4,Clspn,Tipin,Xpo1,Topbp1,Cdkn2c,Dapp1,Anp32b,Nav3,Specc1,Dtymk,Utp11l,Tmpo,Dnajc9

miRNA name	Number of Genes	Corrected p-value	miRNA targets among genes repressed in Rasless cells (Table S1)
**mmu-miR-127***	34(1239)	1,37E-05	Nck2,Mtmr4,Fosl1,Nrf1,Nrp1,Sltm,Impdh2,Mthfd1,Etf1,Zic1,Myc,Cand1,Pik3c2a,Hmgb3,Pde1a,Cbx1,Kif20a,Gpbp1,Scrib,Rad54l,E330009J07Rik,Sema4b,Hmgb2,Snrpd1,Rbbp8,Wdr73,2410016O06Rik,Mycn,Rpl13a,Pcf11,Khdrbs1,Ezh2,Uhrf1,Mrpl18
**mmu-miR-674**	46(1239)	1,40E-05	1110012J17Rik,Ranbp1,Socs5,Prx,Fhl2,Dkc1,Las1l,Aurkb,Wdr5,1110020G09Rik,Dnmt1,Ilf2,Adamts7,Ttyh3,Cdca2,Scarb1,Hmgb3,Cbx1,Psmc3ip,Usp14,Sdad1,Zc3h8,Cul4b,Parp12,Hbs1l,Crebzf,Fanca,Palld,Usp6nl,Hmga2,Sumo2,Psmd14,Hat1,Ptplad1,Cct3,Slco1a5,Ilf3,Cdkn2c,Plekha5,Spp1,Nol11,Pebp1,Gart,H2afy2,Gtse1,Kif23
**mmu-miR-592**	40(1239)	1,58E-05	Pcm1,Ctsw,Ccne1,Atad2,1110020G09Rik,Ythdf2,Rbbp7,Vrk3,Nrp1,Tmem48,Ppid,BC055324,Luc7l,Aftph,Enpp1,Setx,Pde1a,Chst1,Psmc3ip,Casc5,Cobll1,Dhx15,2810474O19Rik,E330009J07Rik,Nfx1,Mmd,Neil3,Magoh,Rbm17,Clspn,Fgd3,Sertad1,Synj2,Ch25h,Ddx20,Impa1,Spc24,Pola2,Gmeb1,Hnrpdl
**mmu-miR-99a**	45(1239)	1,66E-05	Sap30,Ahsa1,Arhgap22,Pak1,Plk1,Ythdf2,Noc4l,Rbbp7,Cdc5l,Mrps6,Impdh2,BC055324,Luc7l,Zic1,Ccdc99,Slc29a1,Eme1,2610039C10Rik,Prpf4,Chuk,Supt16h,Casc5,Arl6ip6,Hn1l,Ccng2,Stoml2,Trim25,Nupl1,1110034A24Rik,Anp32e,Cep70,Mkx,Igsf3,Fgf7,Anln,Bbs12,Apitd1,Smarca5,Wdr73,Osmr,Mcm2,Rrad,Fmnl3,Nol11,E130308A19Rik
**mmu-miR-582-3p**	43(1239)	1,82E-05	Ercc6l,Errfi1,Hspa8,Smpdl3b,Sass6,Emb,Ddx46,Pgm1,Utp15,Dis3,Ahctf1,2510012J08Rik,Schip1,Msh3,Rrm1,Smek1,Ncapg2,Lbr,Mbtps2,Ccnb1,Snrpa1,Gsg2,Acsl3,Kars,Usp3,1110054O05Rik,Neil3,Arid4b,Pkp2,Casp8ap2,Dut,Slc38a1,Gemin8,Topbp1,Eif5,Mboat2,Mcm6,Dtymk,Ptbp2,Ptger4,Khdrbs1,Slc4a7,Eps8
**mmu-miR-883b-5p**	50(1239)	1,88E-05	Shmt1,Lrrc45,Fhl2,Nup37,Ogfrl1,Txnrd1,Prpf19,Mnd1,Emb,Ddx27,Tpd52,Bcl10,Aspm,Vrk3,Yme1l1,Ttll4,Impdh2,Psip1,Agxt2l2,Rrm1,Ccdc99,Egfr,Hmgb3,Pkn3,Ccnb2,Cep152,Cul4b,Parp12,Prpf4b,2210018M11Rik,Cdc7,Bub1b,Itgav,Tk1,Psmd14,Cct3,Mphosph10,4933427D14Rik,Tnks2,Cdkn2c,Dapp1,Plekha5,Spp1,Ell2,Fmnl3,B3galnt1,Bhlhb9,Col7a1,Ppwd1,Cdh10
**mmu-miR-16***	36(1239)	2,10E-05	Etaa1,Ktn1,Fhl2,Steap1,Taf5,Kif2a,Ccne1,Sass6,Mnd1,Pgm1,E2f8,Cdc5l,Ythdc1,Kif18a,Tmem48,Utp18,Klhdc2,Pcna,Gins1,Abcb1b,Shcbp1,Polr2b,Suz12,Neil3,Nme4,Hat1,Zrsr2,Eif5,Tpp2,Esf1,Twistnb,Smu1,Gart,Ccdc18,Tmpo,Fancc
**mmu-miR-100**	43(1239)	2,11E-05	Ahsa1,Timm8a1,Arhgap22,Impa2,Birc5,Plk1,Noc4l,Tyw3,Mrps6,Impdh2,Usp10,Wdr77,Fnbp1l,Zic1,Slc29a1,Eme1,Prpf4,Chuk,Supt16h,Casc5,Set,Ccng2,Imp3,Stoml2,Trim25,Nupl1,Cep70,Mkx,Anln,Bbs12,Apitd1,Smarca5,Mcm2,Rrad,Spc24,Fmnl3,AI848100,Nol11,Smu1,Hells,E130308A19Rik,H2afy2,Pogz
**mmu-miR-574-3p**	47(1239)	2,20E-05	Ahsa1,Pcm1,Ell,Dnaja2,Mcm5,Cks1b,Plk1,Prdx4,Noc4l,Rbbp7,Msh2,Nrp1,Por,Sfpq,Wdr77,Hspa14,Cct2,Pkn3,Psmc3ip,Six4,Dusp6,2310057M21Rik,Gli2,Nfx1,Syt17,Hn1,Pkp2,Psmd14,Plxnb1,Apitd1,Mlf1ip,Ddx31,Wdr73,Sertad1,Rbm14,Usp37,Impa1,Lmnb2,Plekha5,Yes1,Ociad2,Actr3,Gprc5c,Dcbld1,Col7a1,Cep170,Arf6
**mmu-miR-698**	41(1239)	2,37E-05	2810046L04Rik,Tgif2,Ncaph,1110012J17Rik,Pop1,Rcc2,Crim1,Pole2,Ppih,U90926,Noc4l,Nrf1,Ttll4,Cdca5,Cct7,Lsm2,Lig1,Set,Cul4b,Gtf2h2,H2-K1,Ssb,Hmga2,Slc9a3r1,Ctsh,Exosc2,Cenpf,Tinf2,Prkg2,Nutf2,Wdr73,Cacna1c,Plekha5,2610027L16Rik,Cd300lb,Zfp281,Gpsm2,H2afy2,Smyd5,Eftud2,2810408A11Rik
**mmu-miR-744***	36(1239)	2,38E-05	Nck2,Wdr3,Rpl7a,Rdx,Vrk3,Ythdc1,Lrp8,Lsm2,Msh3,Cand1,Eef1e1,Slc25a5,Thsd7a,Psmc3ip,Crlf1,Ewsr1,Nasp,Cbx3,Colec12,1110034A24Rik,Cep70,Unc5c,Pvrl3,Tmem49,Cct3,Mybl2,BC016423,Trpc2,Sertad1,Nsmce4a,Pkmyt1,Thoc2,Ddx39,Etv4,Cdca8,Smyd5
**mmu-miR-345-3p**	42(1239)	2,39E-05	Arhgap22,Atad2,Ripk2,Sass6,Ddx27,Ptpre,Cdc5l,Hspd1,Slc11a2,Zw10,Lrp8,Pdap1,BC055324,Mcm3,Scarb1,Pla2g4a,Abcb1b,Casp2,Imp3,H2-K1,Depdc1a,Traip,Usp3,Itgav,Ptk7,Prpf3,Nt5c3l,Zfp619,Gnl3,Rbm14,Dapp1,Plekha5,Spp1,Esf1,Gprc5c,Fancd2,E330016A19Rik,Etv4,Cdca8,Incenp,Egfl7,Ppa1
**mmu-miR-453**	41(1239)	2,54E-05	Lrrc45,Whsc1,Llgl1,Mapk8,Noc4l,Cep192,Ddx52,Xkr5,Sin3a,Por,Fen1,Tuba1c,Sox11,Msh3,Agxt2l2,Usp34,Slc25a5,Cbx1,Gjc1,Runx2,Sgms1,Pola1,Bub1b,Hspa4l,Pprc1,2810055F11Rik,Melk,Tpx2,Trp53bp2,Cct3,Cnnm4,Tbx3,Apitd1,Neto2,Dapp1,Pkmyt1,5730559C18Rik,Rrad,Specc1,2610027L16Rik,Cdca7
**mmu-miR-696**	42(1239)	2,57E-05	Lrrc45,Arhgap22,Prkar2b,Pwp2,Llgl1,Hmmr,Gemin6,Dctd,Ruvbl1,Nrf1,Ank3,Nrp1,Acaa2,Por,Rnps1,Nup93,Usp10,Nfatc2ip,Armc8,Agxt2l2,Lin9,Stip1,Chst1,H2afy,Kars,Gli2,Nfx1,Arid4b,Tpx2,Cachd1,Mpp6,Cenpn,4933427D14Rik,Slc43a1,Bhlhb9,Rcl1,Psmd5,Pdss1,Ppa1,Ltbp1,Cdh10,Siva1
**mmu-miR-200a***	38(1239)	2,64E-05	Thap2,Ahsa1,Trim24,Whsc1,Cep192,Cct7,Aebp2,Dbr1,2610039C10Rik,Abcb1b,Set,Smc5,Rpl12,Sf3a1,Palld,Mcm10,Ubqln2,Pprc1,Pank4,Exosc2,Smc2,Slco1a5,Lmnb2,Lmnb1,Esf1,Stmn1,Specc1,Dcbld1,Rfc2,Cenpk,Map4k5,1700025G04Rik,Egfl7,Gpsm2,Cdh10,Cdt1,Smyd5,Eps8
**mmu-let-7b**	50(1239)	2,65E-05	Arhgap22,Rcc2,Dffb,Prkar2b,Birc5,Dsn1,Prdx4,Lrig3,Noc4l,Nuf2,Ttll4,Casp3,Prim1,Tubb5,Cct7,Myc,Slc19a1,Eme1,Cep152,Npn2,Bzw2,Iqgap3,Dusp6,Pcnt,Idh2,X99384,Sgms1,Dhx15,Espl1,Nme4,Pole,Qars,Pvrl3,Prps1,Kif2c,Soat1,Plscr1,Spc24,Akap8,Fbxo32,Nob1,1110004E09Rik,Ddx39,Sf3a3,Pcf11,Ezh2,Fancb,Kif23,Zcchc11,Eps8
**mmu-miR-28**	42(1239)	2,85E-05	Etaa1,Whsc1,Plk1,Cep192,Riok2,Als2cr12,Vrk3,Zw10,Snrpb,Luc7l,Emg1,Gins1,Set,Ncapd3,Gsg2,Satb2,Imp3,H2-K1,Ssb,Cdca4,Tk1,Mbd4,Trit1,Pigf,Pvrl3,Slc38a1,Abhd10,Ttc3,Nudc,Apitd1,Ddx31,Pbk,Nsmce4a,Yes1,Ssrp1,Specc1,Nob1,Trub1,Incenp,E130308A19Rik,Cdt1,Mrpl18
**mmu-miR-19a***	35(1239)	3,01E-05	Nck2,Lrrc45,Gemin4,Vegfc,Impa2,Caprin1,Skp2,Snx7,Zfp57,Pspc1,1110020G09Rik,Cdc5l,Wsb1,Dhx36,Sin3a,D2Ertd750e,Cenph,Sgol1,2610039C10Rik,Hbs1l,Nxf1,Lyar,2810055F11Rik,Nudc,Actl6a,Cdkn2c,Spp1,Pum2,Rasal2,2610027L16Rik,Gmeb1,Ppa1,Nsl1,Tmpo,Mrpl18
**mmu-miR-186***	35(1239)	3,13E-05	2810046L04Rik,Asph,Fus,Dctd,Sass6,Cenpo,Pgm1,2510012J08Rik,Mcm3,Cdca2,Egfr,Ung,2610039C10Rik,Scrib,Sgms1,Ptges3,D10Wsu102e,Itgav,Top2a,Arid4b,Slc9a3r1,Cachd1,Tinf2,Nudc,Mastl,Plscr1,Nudt14,Gprc5c,Hirip3,Cep170,Nek2,Gmeb1,Tmpo,H2afy2,Kif23
**mmu-miR-210**	44(1239)	3,53E-05	Rcc2,Prim2,Vegfc,Med4,Ddx27,Dnmt1,Xpo7,Xkr5,Msh6,Tomm70a,Blm,Adamts7,Snrpb,Tuba1c,Armc8,Enpp1,Ppil1,Ctbp2,Pik3c2a,Eif4a3,Zfp217,Imp3,Nupl1,Pif1,Gli2,Neil3,Sumo2,Aifm1,Cenpn,Slc38a1,Cct3,Rfc3,Tpm3,Lmnb2,Cct8,Slc43a1,Syne2,Trub1,2610027L16Rik,Tex10,Gmeb1,Ltbp1,Gtse1,Eps8
**mmu-miR-713**	42(1239)	3,54E-05	Lrrc45,2700050L05Rik,Mcm5,Impa2,Plk1,Fubp1,1110020G09Rik,Srpk1,Bcl10,Aspm,Xkr5,Ilf2,Adamts7,Brca1,Cdca2,Cbx1,2700029M09Rik,Casp2,Ccnf,Pola1,Prpf4b,E330009J07Rik,Itgav,Trit1,Psmd14,Ankrd28,Pvrl3,Trp53bp2,Gnl3,Nucks1,Tpm3,U2af2,2410016O06Rik,Ilf3,Zcchc8,Thoc2,Incenp,Gart,Egfl7,H2afy2,Fancc,2010204K13Rik
**mmu-miR-201**	42(1239)	3,79E-05	D19Bwg1357e,Taf5,Zfp606,Sass6,Kif4,Elp2,Cdc5l,Rnps1,BC055324,Mcm3,Lsm2,Etf1,Enpp1,Cdc25a,Ung,Trim28,Abcb1b,Dhodh,Frat2,Zfp217,Pcnt,Satb2,Zfp54,Cdc7,Hspa4l,2810055F11Rik,Adsl,Snrpg,Egr1,Nudt21,Fbln1,Trpc2,Sertad1,Mboat2,Yes1,Ddx10,Dcbld1,Rfc2,Bcl2l11,Gart,Tmpo,Slc4a7
**mmu-miR-16**	46(1239)	3,97E-05	Ncaph,Diap3,Depdc1b,Mum1,Spag5,Ccne1,Mnd1,Msh2,Ppat,Fkbp5,Ythdc1,Wsb1,Mrps6,Tubb5,Tuba1c,Hspa14,Ung,Ncapg2,Psmc3ip,Cep152,Kif21a,Shcbp1,Fbl,Asf1b,Ipo7,Smndc1,Card10,Ncaph2,Top2a,Wee1,Psmd14,Fzd3,Clspn,Rad51ap1,BC016423,Ptrh2,Mastl,Cep55,E330016A19Rik,Mast4,Vbp1,Ddx39,Rad51,Nxt1,Dnajc9,1810011O10Rik
**mmu-miR-299***	33(1239)	3,98E-05	Dhx9,Gmnn,Nup37,Setdb2,Ddx46,Riok2,Cdc5l,Als2cr12,Tmem48,BC055324,Myc,Eef1e1,Slc19a1,Pkn3,Pnpt1,Rpl12,Rad54l,Ckap2,Nt5c3l,Hat1,Abhd10,Dus3l,Mlf1ip,Wdr73,Mboat2,Cep55,2610027L16Rik,Erh,Pola2,Rad51,Ccdc18,Ecd,Dlx1

miRNA name	*Number of Genes*	*Corrected p-value*	*miRNA targets among genes repressed in Rasless cells (Table S1)*
**mmu-miR-758**	43(1239)	4,18E-05	Gata2,Cks1b,Cbx2,Utp15,Parp1,Rnf138,Taf5l,Myc,Sms,Cep57,Enpp1,Egfr,Pla2g4a,Lig1,Ncapd3,Zc3h8,Smc5,Pcnt,Cul4b,Parp12,Troap,H2-K1,Cdc7,Gins4,4930579G24Rik,Dbf4,Rfc5,Melk,Hat1,Gnl3,Ahcy,Prc1,Ssrp1,Lmnb1,Pum2,Litaf,Ppwd1,Thoc2,Erh,H2afy2,Cdt1,2810408A11Rik,Cirh1a
**mmu-miR-203**	38(1239)	4,18E-05	Nedd4l,Rbm26,Cenpc1,Nup54,Ercc6l,Skp2,Steap1,Kif2a,Prpf19,Tes,Mrps22,Pspc1,Myef2,Pdap1,Cct7,Dusp5,Scrib,Pola1,Crebzf,Ssb,Slco4a1,Csnk1a1,Snw1,Snrpd1,Gli3,Pigf,Fgf7,Neto2,Pbk,Actl6a,Synj2,Cdkn2c,Bhlhb9,Whsc2,H2afy2,2010204K13Rik,Dnajc9,Ecd
**mmu-miR-218-2***	39(1239)	4,23E-05	Nono,Chd1,Vegfc,Skp2,Cks1b,Pak1,Hmmr,Mnd1,Rpl7a,Aurka,Cdc27,Impdh2,Prim1,Hspa14,Ccdc99,Zmynd19,Cbx1,Abcb1b,Iqgap3,Cdca4,Nfx1,Dbf4,Prpf3,Nmral1,Ppbp,Cct3,Hmgn1,Trpc2,Yes1,Rrad,Specc1,Dcbld1,Tacc3,Ppwd1,Pola2,Timp1,Ccdc18,Slc4a7,Socs4
**mmu-miR-181a-2***	41(1239)	4,30E-05	Lrrc45,Prpf31,Rbm26,Dffb,H2afv,Nol9,Mki67,Prpf19,Nap1l4,Emb,Egr3,Ythdc1,Socs3,Tmem173,Zic1,Enpp1,Dusp4,Dusp5,Ctnna1,Ankrd10,Bzw2,Sbk1,Ppp1cc,Ran,Rpa2,Mpp6,Exosc2,Lrig1,Abhd10,Patz1,Neto2,4930427A07Rik,G3bp1,Cdc40,Cdkn2c,Actr3,Trub1,Snrpb2,Thoc2,Wdhd1,Ecd
**mmu-miR-140**	45(1239)	4,39E-05	Ncaph,Clns1a,Pwp2,Acly,Ctsw,Wdr3,Smc6,Tpd52,Egr2,Tmem176b,Hspd1,Mrps6,Tgif1,Fen1,Ppid,Usp10,Pgk1,Cenph,Cand1,Lin9,Slc19a1,Rasa3,Cep152,Mbtps2,Cul4b,Pprc1,Wee1,Nme4,Runx1,Nt5c3l,Chd2,Pole,Igsf3,2410042D21Rik,Nudc,Esco2,Dapp1,Cep55,Slc43a3,Lmnb1,Fmnl3,Bcl2l11,Cebpz,Ecd,Kif23
**mmu-miR-362-5p**	43(1239)	4,44E-05	Eef1g,Cks1b,Sass6,Ncl,Kif11,1110020G09Rik,Bub3,Ddx52,Parp1,Ythdc1,Usp34,Dhodh,Usp14,Crlf1,Zfp217,Hbs1l,Ptpn2,Pnpt1,Hyal2,Stoml2,Asf1b,Npm1,Tead2,Cep70,Runx1,Paip1,Zwilch,Ptcd3,Ube2t,Cct3,Xpo1,Nutf2,Ttc3,Nudc,Specc1,Pola2,Whsc2,Rad51,H2afy2,Terf1,Slc4a7,Zcchc11,Cirh1a
**mmu-miR-497**	45(1239)	4,51E-05	Tgif2,Depdc1b,Taf5,Pak1,Ccne1,Mnd1,Cep192,Msh2,Fkbp5,Cdca5,Mrps6,Cenph,Cdc25a,Ung,Cep152,Mpzl1,Sdad1,Shcbp1,Sgms1,Metap1,Nusap1,Zfp54,Uchl5,Sema4b,Cdca4,Wee1,Fzd3,Ppif,Limd2,Prmt5,Synj2,Ssrp1,Cnot6,Stmn1,Eif4h,Ddx39,1700025G04Rik,Pcf11,Pebp1,Ccdc18,4632434I11Rik,Nxt1,Gtse1,Ecd,Wdr36
**mmu-miR-324-3p**	49(1239)	4,52E-05	Fmr1,Nedd4l,Whsc1,D19Bwg1357e,Ccbe1,Hspa8,Nol9,Elf2,9030617O03Rik,Nipsnap1,Med4,Riok2,Xpo7,Msh2,Fxn,Impdh2,Nfrkb,Junb,Luc7l,Msh3,Cbfb,Enpp1,Stip1,Emg1,Pkn3,Zfp217,Sbk1,Hyal2,Asf1b,Csnk1a1,Cdca4,Mcpt8,Mkx,Ddx11,Clspn,Patz1,Ch25h,5730559C18Rik,Syne2,Fmnl3,Pafah1b3,Col7a1,Th1l,Eif4h,Rad51,Terf1,Lif,Mrpl18,1810011O10Rik
**mmu-miR-466d-5p**	56(1239)	4,64E-05	2810046L04Rik,Cdca7l,Dffb,Prim2,Impa2,Dhx9,Mum1,Ctsw,Phactr4,Ddx27,E2f8,Msh6,Mtf2,Lcorl,Armc8,Aftph,Lin9,Ctbp2,Pik3c2a,Cd2ap,Pla2g4a,Usp34,Brd8,Npn2,Kntc1,Zfp217,Kif21a,Pcnt,Gtf2h2,Sbk1,Lrrk1,Nupl1,Snw1,Pif1,Neil3,Syt17,Sumo2,Cenpn,Ddx11,Casp8ap2,Nde1,Casp6,Gnl3,Usp37,Prc1,Cct8,Bnc2,Plscr1,Slc43a1,Rrad,Spc24,Akap8,Cenpk,1700025G04Rik,Cdca8,Ltbp1
**mmu-miR-324-5p**	48(1239)	4,73E-05	Ncaph,Asph,Arhgap22,Impa2,D19Bwg1357e,Upf3b,Ogfrl1,Plk1,Ddx21,Ccne1,Cep192,Ptpre,Nol10,Apex2,Mcm3,Lsm2,Nfatc2ip,Luc7l,Glul,Slc19a1,Cep152,Rad51c,Set,Sf3a1,Sema4b,Top2a,Snrpd1,Unc5c,Gli3,Aifm1,Kin,Melk,Chd2,Pigf,Qars,Sox4,Gemin8,Zfp619,Mcm7,Synj2,Mycn,Ssrp1,Syne2,Rrad,Rsrc2,Nek2,Rab8b,Fancc
**mmu-let-7d**	48(1239)	5,03E-05	Tmem39b,Rcc2,Tnpo1,Prkar2b,Birc5,Ktn1,Dsn1,Mki67,Rangap1,Lrig3,Atad2,Noc4l,Ube2e3,Ttll4,Prim1,Tubb5,Myc,Eme1,2610039C10Rik,Cep152,Casc5,Bzw2,Dusp6,Pcnt,Sgms1,Gtf2h2,Espl1,Bub1b,1110034A24Rik,Nme4,Qars,Sip1,Prps1,Kif2c,Dus3l,Mcm7,Synj2,Plscr1,Akap8,D16Ertd472e,Nob1,1110004E09Rik,Ddx39,Sf3a3,Ezh2,Kif23,Hnrpdl,Zcchc11
**mmu-miR-325***	34(1239)	5,22E-05	Prim2,Prx,Amd1,Atad2,Sass6,Kif4,Tcerg1,Ilf2,Nrp1,Sgol2,Mrps6,Sltm,Acaa2,Tuba1c,Stip1,Zmym1,Pde1a,Matr3,1700029F09Rik,U2af1,Casp2,Zfp451,Kpna2,Pprc1,Snrpd1,Nme4,Cachd1,Trpc2,Ddx20,Mre11a,Ptbp2,Atrx,2610027L16Rik,Nsl1
**mmu-miR-764-5p**	46(1239)	5,38E-05	Etaa1,Mcm5,Ctsw,Ccne1,Msh2,D2Wsu81e,Zc3h15,Ttll4,Tmem48,Cpsf4,Junb,Armc8,Nudt1,Gpd2,Rrm1,Ppil1,Zmynd19,Cbx1,Lig1,Idh2,Tslp,H2afy,Ewsr1,Rnaseh2a,Nt5c3l,Pole,Trp53bp2,Slc38a1,Cct3,Kcnk2,Tpm3,Polr3b,Synj2,Usp37,Cnot6,Mcm6,Rsrc2,Ppwd1,Whsc2,Me2,Incenp,4632434I11Rik,Ezh2,Fancc,Ift74,Eftud2
**mmu-miR-876-5p**	40(1239)	5,61E-05	4930422G04Rik,Glrx,Zfp606,Nipsnap1,Bub3,Xkr5,Mad2l1,Dhx36,Ctbp2,Zmym1,Pde1a,Eme1,Rasa3,Usp14,Lig1,Sdad1,Ube2c,Gnb4,Ptpn2,Sf3a1,Itgav,Phf17,Snrpg,Mcpt8,Unc5c,Ctsh,Net1,Ints7,3110003A17Rik,Nutf2,Smc2,Chtf18,Nsmce4a,Cnot6,Lmnb1,Dtymk,Cep170,Hells,Arf6,Ecd
**mmu-miR-335-3p**	35(1239)	6,06E-05	Prpf31,Ifi205,Prdx4,Cpsf6,Kif4,Fubp1,Nipsnap1,Ddx52,Gspt1,Tmem48,Sltm,Cct7,Fbxo5,Zfp184,Eef1e1,Pde1a,Ccnb2,Gtf2h2,Vrk1,Depdc1a,Tial1,Lyar,Zfp451,Syt17,Pvrl3,3110003A17Rik,Tacc3,Nol11,Smu1,Cebpz,Cenpi,Cdc73,Eftud2,Wdr36,Pogz
**mmu-miR-574-5p**	44(1239)	6,08E-05	2810046L04Rik,Pcm1,Prim2,Prdx4,Phactr4,Fubp1,Bub1,Vil1,Mrps6,Acaa2,Dusp9,Nudt1,Pik3c2a,Pkn3,Gjc1,Chst1,Ccnb1,Kntc1,Pcnt,Lrrk1,Depdc1a,Tial1,Lyar,Nusap1,Slco4a1,Kpna2,Dimt1,Neil3,Sumo2,Nab2,Ptcd3,Qars,Prr11,Dus3l,Bnc2,Lmnb1,Cep170,Rpl13a,1110004E09Rik,Psmd5,Pdss1,Tex10,Cdca8,Ltbp1
**mmu-let-7a**	47(1239)	6,15E-05	Phf6,Rcc2,Birc5,Ktn1,Dsn1,Lrig3,Noc4l,Bub3,Nuf2,Ttll4,Casp3,Prim1,Tubb5,Cct7,Fbxo5,Myc,Slc19a1,Trip13,Eme1,2610039C10Rik,Cep152,Dusp6,Pcnt,X99384,Gtf2h2,Dhx15,Espl1,Ckap2,Nme4,Psmd14,Qars,Sip1,Pvrl3,Prps1,Mcm7,Actl6a,Akap8,D16Ertd472e,Nob1,1110004E09Rik,Ddx39,Sf3a3,Ezh2,Fancb,Kif23,Hnrpdl,Zcchc11
**mmu-miR-501-3p**	46(1239)	6,90E-05	Nono,Ahsa1,Timeless,Rcc2,Prkar2b,Impa2,Whsc1,D19Bwg1357e,Spred2,Tnnt2,Smpdl3b,Las1l,Cep192,Riok2,Ank3,Nol10,Por,Cct7,BC055324,Aftph,Gins1,Imp3,Ptpn2,Ppp1cc,Lrrk1,Rpl12,Palld,Hmgb1,Ptges3,Adsl,Casp8ap2,3110003A17Rik,Lrig1,Zbtb12,Prmt5,Wdr73,Cdkn2c,Mycn,Syne2,Cenpk,Ptbp2,Gmeb1,Incenp,Gart,H2afy2,Rrs1
**mmu-let-7e**	47(1239)	6,95E-05	Arhgap22,Rcc2,Dffb,Birc5,Ktn1,Eef1g,Dsn1,Rangap1,Lrig3,Noc4l,Ttll4,Prim1,Cct7,Myc,Slc19a1,Trip13,Eme1,Cep152,Casc5,Bzw2,Iqgap3,Dusp6,Pcnt,X99384,Dhx15,Espl1,Cdc7,Ckap2,1110034A24Rik,Nme4,Mtm1,Psmd14,Qars,Sip1,Nudt21,Patz1,Mcm7,Akap8,Bhlhb9,Nob1,1110004E09Rik,Ddx39,Sf3a3,Ezh2,Fancb,Nxt1,Kif23
**mmu-miR-699**	43(1239)	7,73E-05	Nedd4l,Prkar2b,Mcm5,Marcksl1,Gmnn,Ctsw,Nipsnap1,D2Wsu81e,Casp3,Acaa2,Nfrkb,Gins2,Asxl3,Cep152,Bzw2,Iqgap3,Ncapd3,Sgms1,Wipf1,Slco4a1,Gli2,Rpa2,Nme4,Hat1,Pole,Pvrl3,Gemin8,Chtf18,Nsmce4a,Usp37,Ssrp1,Fmnl3,Rfc2,Dtymk,Ppwd1,Rpl13a,Erh,Pola2,Pcf11,E130308A19Rik,Fancc,2810408A11Rik,Cirh1a
**mmu-miR-340-3p**	37(1239)	8,78E-05	Nck2,Nol9,Tgfbr3,Ctsw,Txnrd1,Prpf19,Swap70,1110020G09Rik,Ddx27,Rbbp7,Msh2,Xkr5,Ruvbl1,Mad2l1,Tgif1,Cct7,Rnps1,Fen1,Zfp184,Cd2ap,Trip13,Zc3h8,Sgms1,Nxf1,Trim25,Sumo2,Nmral1,Zwilch,Kif2c,Lrig1,Rfc2,Trub1,Khdrbs1,Dock5,Smyd5,Tcf4,Eps8
**mmu-miR-199a-5p**	47(1239)	8,88E-05	Nedd4l,2810046L04Rik,Asph,Pcm1,Sirt1,Prkar2b,Ktn1,Prpf40a,Ets1,Mum1,9030617O03Rik,Rest,Ppat,Slc11a2,Mrps6,Tmem173,Hdac2,Tuba1c,Cd2ap,Emg1,Pde1a,Ccnb2,Abcb1b,Smc5,Imp3,Nasp,Cbx3,Spry2,Snw1,Phf17,Tpx2,Rbbp8,Magoh,Rbm17,Dut,Ss18,Dus3l,Foxp1,Mcm6,Sdpr,E330016A19Rik,Rcl1,Ppwd1,Snrpb2,Timp1,Nsl1,Eif2s1
**mmu-miR-433***	33(1239)	9,52E-05	Hsp90aa1,Lrrc45,Dffb,Zfp52,Gemin6,Dcp1a,Kif11,1110020G09Rik,Msh2,Als2cr12,Parp1,Vrk3,Fxn,Klhdc2,Usp34,Kif20a,Snrpa1,Recql4,Traip,Ckap2,Snrpg,Chd2,2810025M15Rik,Clspn,Lrig1,Dus3l,Trpc2,Plekha5,Ociad2,Me2,Ppa1,E130308A19Rik,Tpbg
**mmu-miR-382**	39(1239)	0,000101142	Marcksl1,Impa2,Ets1,Lrig3,Ddx27,Riok2,Tmem176b,Nuf2,Hspd1,Myc,Larp7,Pde1a,Matr3,Thsd7a,Baz1a,Hbs1l,Atad5,Tial1,Prpf38a,Trim25,Rasa1,E330009J07Rik,Ckap2,Paip1,Tpx2,Psmd14,Chd2,Smchd1,Mastl,Plscr1,Dtymk,Hirip3,Cenpk,Utp11l,Pcf11,Dyrk3,Ppa1,Top1,1810011O10Rik

miRNA name	*Number of Genes*	*Corrected p-value*	*miRNA targets among genes repressed in Rasless cells (Table S1)*
**mmu-miR-322**	42(1239)	0,000132095	6430527G18Rik,Tgif2,Spag5,Has2,Dcp1a,Ccne1,Cep192,Utp15,Ilf2,Fkbp5,Ythdc1,Wsb1,Mrps6,Ppil1,Rpl30,Cdc25a,Ncapd2,Shcbp1,Rpl12,Acsl3,Wipf1,Nusap1,Spry2,Top2a,Psmd14,Trp53bp2,Slc38a1,Limd2,Clspn,Lrig1,Neto2,Rrad,Arhgap19,Vbp1,Ddx39,1700025G04Rik,Pcf11,Rad51,Pebp1,Fancc,Nxt1,Mrpl18
**mmu-miR-10b**	40(1239)	0,000136217	Gemin4,Birc5,Hspa8,Prpf19,5730590G19Rik,6720463M24Rik,Kif18a,Rnps1,Cand1,Sgol1,Scarb1,Smarce1,Ccdc99,Denr,Ncapd2,Eif4a3,Bzw2,Lrrk1,Traip,Tead2,Hn1,Igsf3,Tipin,Patz1,Dus3l,Apitd1,Zbtb12,Tpm3,Osmr,Specc1,Abl2,Pola2,Rad51,C330027C09Rik,Pou2f1,Rbm12,Arf6,Cdh10,Gtse1,Kif23
**mmu-miR-125b-5p**	49(1239)	0,000145505	Ahsa1,Gata2,Ccbe1,Tcf19,Sass6,Pdia6,Fkbp5,Tox,Impdh2,Taf5l,Lcorl,Snrpb,Cdca2,Ash2l,Ccdc99,Rpl30,Cct2,Troap,Ptpn2,Rpl12,Tube1,Kars,Csnk1a1,Usp3,Colec12,Itgav,Top2a,Tk1,Itih2,Racgap1,Ints7,Wdr55,Limd2,Cnnm4,Nudc,Wdr73,Synj2,Cdc40,Usp37,Dapp1,Fmnl3,Gprc5c,E330016A19Rik,Ppwd1,Snrpb2,Lifr,Phc2,Zfp281,Lif
**mmu-miR-874**	41(1239)	0,000147454	Nedd4l,Lrrc45,Arhgap22,H2afv,Ell,Birc5,Eef1g,Plk1,Prpf19,Setdb2,Srpk1,Xpo7,Pom121,Por,Luc7l,Etf1,Cand1,Agxt2l2,Setx,Ncapd2,Kif21a,Rpl12,Slbp,Slco4a1,Phf17,Prpf3,Rfc5,Slc9a3r1,Trit1,Clspn,Dus3l,Nudc,4930427A07Rik,Lmnb2,Tnrc6a,5730559C18Rik,Abl2,Gprc5c,Ddx39,Gpsm2,Cirh1a
**mmu-miR-697**	41(1239)	0,000152014	Nup54,Rnmt,Hspa8,Mnd1,Kif11,Pnn,Xpo7,Pdia6,Zc3h15,Por,Apex2,Pgk1,Nfatc2ip,Armc8,Tll1,Cand1,Sms,Chst1,Ccnb1,Set,Recql4,Stoml2,Tube1,Nusap1,Smndc1,Nr2f2,2610002M06Rik,Gli3,Ppbp,Ckap4,Pvrl3,Fgf7,Jub,Mybl2,Fancd2,Alkbh1,Rcl1,H2afy2,Nxt1,Ahcyl1,Dnajc9
**mmu-miR-125a-3p**	48(1239)	0,000154285	Lrrc45,Impa2,Kif2a,Has2,Aurka,Msh2,Egr3,Fkbp5,Nrp1,Hspd1,Brca1,Snrpb,Aebp2,Ttyh3,Fnbp1l,Rcc1,Stip1,Crlf1,Troap,Fbl,Slco4a1,Ncam1,Snw1,Bub1b,Nme4,Gli3,Aifm1,Ints7,Dut,Patz1,Bbs12,Ddx31,Neto2,Wdr73,Bnc2,Plscr1,Akap8,Ly75,Fancd2,Rcl1,Etv4,Khdrbs1,Cdca7,Ppa1,Nsl1,Smyd5,Eftud2,Smarcc1
**mmu-miR-378**	37(1239)	0,000159015	Ahsa1,Arhgap22,Prpf19,Las1l,Nrf1,Fxn,Msh6,Lrp8,Fen1,Mcm3,Aebp2,Tll1,Cand1,Scarb1,Pkn3,Trim28,Imp3,Hyal2,Stoml2,Trim25,Asf1b,Nme4,Ctsh,3110003A17Rik,Gemin8,Ahcy,Trpc2,Nup155,Spp1,Slc43a3,Stmn1,Gprc5c,Fancd2,Rcl1,Egfl7,Cdca7,Rrs1
**mmu-miR-296-3p**	47(1239)	0,000159089	Ahsa1,Pcm1,Mybbp1a,Spag5,Ctsw,Phactr4,Mnd1,2010002N04Rik,Ddx27,Rpl7a,Tmem176b,Acaa2,Fndc4,Tmem173,Apex2,Pim3,Myc,Exo1,Psmc3ip,Cep152,Iqgap3,Troap,H2-K1,Bub1b,Pprc1,Prpf3,Nme4,Ctsh,Cachd1,Cenpn,Fzd3,Patz1,Ilf3,Ruvbl2,Pold1,Pum2,Bhlhb9,Cd14,Gprc5c,Dcbld1,Lifr,Zfp281,Ccdc18,Arf6,Nxt1,Gtse1,Zcchc11
**mmu-miR-30b***	41(1239)	0,000160736	Lrrc45,Birc5,Fhl2,Elf2,Dkc1,Tnnt2,Spag5,Fubp1,Elp2,Mrps6,Lsm2,Luc7l,Eme1,Pdk3,Cbx1,Usp14,Spc25,Sgms1,Ppp1cc,Kars,Snrpg,Gli3,Aifm1,Cdca3,Fzd3,Cct3,Nutf2,Mlf1ip,Sertad1,Slco1a5,Syne2,Cd14,Rcl1,Th1l,2610027L16Rik,Ddx39,Pebp1,Ppa1,Nsl1,Terf1,Eftud2
**mmu-miR-122**	41(1239)	0,00016566	Nedd4l,Tmem39b,Diap3,Llgl1,Kif2a,Wdr43,9030617O03Rik,Phactr4,Idh3a,Fubp1,Ube2e3,Tox,Hspd1,Msh6,Ifitm3,Lcorl,Zic1,Nudt1,Agxt2l2,Crlf1,Ccnb1,Tial1,Lyar,Cobll1,Nusap1,Zfp54,Wdr75,Dbf4,Snrpg,Zcchc2,Qars,3110003A17Rik,Socs6,Tinf2,Ttc3,Usp37,Hirip3,Eif4h,Psmd5,Dock5,Tpbg
**mmu-miR-673-5p**	44(1239)	0,000168658	Prpf31,Whsc1,Rnmt,Prpf19,Ccne1,Med4,Wdr3,Srpk1,Dnmt1,Tpd52,Fkbp5,Vrk3,Suv39h1,Mcm3,Luc7l,Abcb1b,Casc5,Zfp217,Pcnt,Gnb4,Sbno1,2210018M11Rik,Ewsr1,Nupl1,Bub1b,Cdca4,Ctsh,Hat1,Cenpn,Fzd3,Ppif,Kif2c,Patz1,Trpc2,Anp32b,Tnrc6a,Esf1,Rcl1,Mast4,Thoc2,Bcl2l11,Pou2f1,Ppa1,Smyd5
**mmu-miR-692**	33(1239)	0,00017341	Arhgap22,Nup54,D19Bwg1357e,Ktn1,Wdr3,Prim1,Aftph,Rrm1,Pde1a,Scrib,Smc5,2310057M21Rik,Zfp451,Fbl,Ran,Wdr75,F630043A04Rik,Stt3b,Polr3b,Synj2,Usp37,Bnc2,Spp1,Syne2,Stmn1,1110004E09Rik,Thoc2,Gart,Terf1,Cks2,2810408A11Rik,Smarcc1,Zcchc11
**mmu-miR-17***	36(1239)	0,0001751	Asph,Depdc1b,Acly,Plk1,Idh3a,Tes,Emb,Srpk1,Lsm2,Armc8,Rab15,Emg1,Larp7,Zmynd19,Cbx1,Pkn3,Recql4,Hbs1l,Gtf2h2,Ptpn2,Tial1,Kars,Usp3,Ncaph2,E330009J07Rik,Cdc7,Mns1,Melk,Cenpf,Nav3,Rasal2,2610027L16Rik,Ccdc18,Pou2f1,Nxt1,Smyd5
**mmu-miR-449b**	43(1239)	0,000177646	Nedd4l,Notch1,Marcksl1,Gmnn,Prpf40a,Tcf19,Tnnt2,Ctsw,Nipsnap1,Cdc5l,Als2cr12,Gspt1,2510012J08Rik,Msh6,Casp3,Acaa2,Mcm3,Sgol1,Slc29a1,B230120H23Rik,Bzw2,Iqgap3,Sema4b,Pif1,Eno3,Nme4,Nt5c3l,Pole,Ppif,Gemin8,Actl6a,Nsmce4a,Usp37,Foxp1,Slc43a3,Rfc2,Arhgap19,Ppwd1,Rpl13a,Timp1,Zfp281,Ift74,Cirh1a
**mmu-miR-15a**	44(1239)	0,000183164	Ktn1,Depdc1b,Ccne1,Msh2,Mad2l1,Ppat,Fkbp5,Wsb1,Mrps6,Cenph,Cdc25a,Ung,Trip13,Cep152,Kif21a,Shcbp1,Metap1,Palld,Nusap1,Usp3,Ncaph2,Cdca4,Top2a,Psmd14,Hat1,Ppif,Limd2,Clspn,Lrig1,Neto2,Ptrh2,Ssrp1,Rrad,Stmn1,Specc1,Eif4h,Thoc2,Ddx39,Rad51,Hells,Ccdc18,Nxt1,Mrpl18,Ecd
**mmu-miR-323-5p**	44(1239)	0,000187503	6430527G18Rik,Arhgap22,Rnmt,Fosl1,Nipsnap1,Aurka,Myef2,Casp3,Cpsf4,Blm,Cand1,Rpl3,Slc19a1,Dusp5,Psmc3ip,Bzw2,Rad51c,E130303B06Rik,2310057M21Rik,H2afy,Nxf1,Cobll1,Slco4a1,Espl1,Ier2,Smc3,Gins4,Tead2,Rpa2,Nt5c3l,Pole,Prkg2,Trpc2,Gprc5c,Tacc3,Mast4,Nol11,Smu1,Rfc4,Dock5,Ecd,Eps8,Klf5,Dlx1
**mmu-miR-133a**	44(1239)	0,000187503	Tnfaip8,Prpf31,Rcc2,H2afv,Elf2,Lrig3,1110020G09Rik,Riok2,6720463M24Rik,Egr3,D2Wsu81e,2510012J08Rik,Ttll4,Acaa2,Prim1,Nudt1,Aftph,Ctbp2,B230120H23Rik,2700029M09Rik,Peg12,Rad54l,Chaf1b,Nmral1,Rad18,Ankrd28,Sfxn1,Pvrl3,Cct3,Chtf18,Slco1a5,Usp37,Gprc5c,Rcl1,Ptbp2,Th1l,Rpl13a,Ddx39,Whsc2,Phlda1,H2afx,Phc2,Cebpz,Lif
**mmu-miR-532-3p**	45(1239)	0,0001881	2810046L04Rik,Lrrc45,Timeless,Pwp2,Whsc1,Tnnt2,Plk1,Rangap1,Nap1l4,Pspc1,Mapk8,Ddx27,Xpo7,Aspm,Egr3,Gspt1,Cyp26b1,Mrps6,Cpsf4,Pdap1,Zfp184,Zmym1,Ung,Cbx1,Pkn3,Chst1,Gpbp1,2700029M09Rik,Scrib,Ncapd3,Sf1,Sf3a1,Wipf1,Dbf4,Nab2,Magoh,Rsf1,Limd2,Xpo1,Zbtb12,Mcm2,Slc43a1,Rfc2,Mre11a,Th1l
**mmu-miR-195**	41(1239)	0,000209681	Ktn1,Spag5,Has2,Ccne1,Emb,Msh2,Egr3,Mrps6,Tubb5,Tuba1c,Aftph,Rpl30,Adss,Psmc3ip,Cep152,Usp14,Kif21a,Shcbp1,Wipf1,Asf1b,Ipo7,Nupl1,Ncaph2,Cdca4,Top2a,Wee1,Psmd14,Pvrl3,Fzd3,Ppif,Clspn,Ptrh2,Ssrp1,Arhgap19,Mast4,Ddx39,Whsc2,Rad51,Pebp1,Dnajc9,1810011O10Rik
**mmu-let-7f**	44(1239)	0,000217082	Phf6,Rcc2,Prkar2b,Birc5,Ktn1,Dsn1,Mki67,Lrig3,Noc4l,Bub3,Ttll4,Casp3,Acaa2,Tubb5,Cct7,Cenph,Myc,2610039C10Rik,Cep152,Casc5,Dusp6,Gtf2h2,Dhx15,Espl1,Ckap2,1110034A24Rik,Nme4,Mtm1,Qars,Sip1,Pvrl3,Actl6a,Akap8,D16Ertd472e,Nob1,1110004E09Rik,Ddx39,Sf3a3,Ezh2,Fancb,Nxt1,Kif23,Hnrpdl,Zcchc11
**mmu-miR-701**	39(1239)	0,000225321	Ncaph,Mtmr4,Impa2,Birc5,Skp2,Nol9,Ppih,Trim59,Emb,6720463M24Rik,Dis3,Tmem173,Pim3,Cct5,Luc7l,Klhdc2,Cand1,Ash2l,Chst1,Thsd7a,Mbtps2,Cul4b,Lrrk1,Prpf38a,Tube1,Gtpbp10,Hmgb2,Tk1,Limd2,Tbx3,Synj2,Bnc2,Cd14,Cenpk,Atrx,Pdss1,Incenp,Gtse1,Rbpj
**mmu-miR-676**	38(1239)	0,000244067	Lrrc45,Rnmt,Has2,2010002N04Rik,Rpl7a,Sltm,Nudt1,Csf1,Dusp5,Pcnt,Satb2,Troap,Ptpn2,Sbno1,Fanca,Hyal2,Phf17,Pprc1,Rpa2,Ptk7,Rfc5,Racgap1,Prps1,Chtf18,Dapp1,Tnrc6a,Arhgap19,Ppwd1,Psmd5,1700025G04Rik,Phlda1,Cebpz,E130308A19Rik,Tmpo,Nxt1,Smyd5,Ecd,Hnrpdl
**mmu-miR-151-5p**	43(1239)	0,000244255	Asph,H2afv,Tnnt2,Plk1,Ppih,Cenpo,Cep192,D2Wsu81e,Fkbp5,Vrk3,Adamts7,Cct7,Snrpb,Sox11,Luc7l,Cenph,Denr,Gins1,Gjc1,Eif4a3,Idh2,Imp3,Fanca,Prpf38a,Metap1,Hyal2,Hspa4l,Trit1,Nab2,Rbm17,Dus3l,Actl6a,Usp37,Ociad2,Esf1,Specc1,Cd14,Fancd2,Rfc2,Nob1,E130308A19Rik,Cdt1,Tpbg
**mmu-miR-551b**	37(1239)	0,000273668	Khsrp,Caprin1,Tcf19,Tnnt2,Acly,Dsn1,Plk1,Ccne1,Pank1,Slc11a2,Aebp2,Stip1,Usp14,Crlf1,Cul4b,Palld,Pttg1,Mbd4,2810025M15Rik,Cenpn,Igf2bp1,Exosc2,Tipin,Cnnm4,Nudc,Chtf18,BC016423,Dapp1,B3galnt1,Arhgap19,Mast4,Pola2,Cdca8,Me2,Pou2f1,2810408A11Rik,Smc4

miRNA name	*Number of Genes*	*Corrected p-value*	*miRNA targets among genes repressed in Rasless cells (Table S1)*
**mmu-miR-343**	36(1239)	0,000296538	Nck2,Ranbp1,Eef1g,Txnrd1,Dcp1a,Prpf19,Rangap1,Cep192,Egr2,Nrp1,Junb,Mcm3,Lsm2,Alad,Fbxo5,Sox11,Msh3,Ppil1,Stip1,Cbx1,Ctnna1,Micall2,Ccnb1,Recql4,Cul4b,2210018M11Rik,Hyal2,Rad54l,Ctsh,Slc38a1,Zbtb12,Rbm14,Pold3,Rrad,Lmnb1,Nob1
**mmu-miR-365**	43(1239)	0,000307166	Hsp90aa1,Dffb,Ktn1,Prpf40a,Ets1,Zfp606,9030617O03Rik,Kif4,Cep192,Pgm1,D2Wsu81e,Ttll4,Zw10,Mrps6,Impdh2,BC055324,Brd8,Dtl,Crlf1,Ccrn4l,Ptpn2,Ppp1cc,Lrrk1,2210018M11Rik,Sf3a1,Snw1,Pprc1,Nme4,Mpp6,Cenpn,Igf2bp1,Soat1,Synj2,Ddx20,Tpp2,E2f7,Nav3,Col7a1,Rsrc2,Cep170,Vbp1,1700025G04Rik,Lifr
**mmu-miR-219**	41(1239)	0,000308353	Shmt1,Lrrc45,Prim2,Trim24,Impa2,Ktn1,Tnnt2,Ddx27,Cep192,Pgm1,Ddx52,Zc3h15,Nrp1,Prim1,Msh3,Asxl3,Cdca2,Emg1,Pla2g4a,Ncapd2,Micall2,Polr2b,Imp3,Sbno1,Ssb,Cdc7,Topors,Itgav,Ptk7,Rbm14,Foxp1,Bnc2,Slc43a3,Pum2,Rfpl4,Zcchc8,Thoc2,Apex1,Ezh2,Nsl1,Cdc73
**mmu-miR-199b***	35(1239)	0,000378307	2810046L04Rik,Sirt1,Ktn1,Ets1,Mum1,9030617O03Rik,Setdb2,Aurkb,Ppat,Ythdc1,Zw10,Mrps6,Dhx36,Tuba1c,Emg1,Setx,Pde1a,Abcb1b,Smc5,Nasp,Cbx3,Spry2,Snw1,Tpx2,Rbbp8,Ddx11,Dut,Ss18,Dus3l,Foxp1,Rcl1,Ppwd1,Timp1,Nsl1,Tmpo
**mmu-miR-138**	43(1239)	0,000385044	Arhgap22,Prkar2b,Gmnn,Fhl2,Nol9,Snx7,Mybbp1a,Kif11,Bcl10,Mad2l1,Sgol2,2510012J08Rik,Cpsf4,Sin3a,Por,Agxt2l2,Sh3kbp1,Zfp36l2,Gins1,Cbx1,Rad51c,Set,E130303B06Rik,Kntc1,Snrpa1,Pcnt,2310057M21Rik,Troap,Prpf4b,Fanca,Zfp7,Cdca4,Hspa4l,Hmgb2,Ckap2,Rad18,Mtm1,Cachd1,Trp53bp2,Fgd3,2410016O06Rik,Ilf3,Ezh2
**mmu-miR-31**	41(1239)	0,000389615	Impa2,Prx,Ktn1,Taf5,Vps36,Mki67,Kif4,Ddx27,Elp2,Snrpb,Scarb1,Ung,Eme1,1700029F09Rik,Cbx1,Supt16h,Polr2b,Pola1,Hyal2,Espl1,Ptcd3,Slc38a1,Kif2c,Rps13,Mcm7,Prmt5,Topbp1,Pbk,Wdr73,Ilf3,Pkmyt1,Mboat2,Akap8,Fancd2,Tacc3,2610027L16Rik,Map4k5,Cdca8,Cdc73,Rbpj,Cirh1a
**mmu-miR-465a-3p**	43(1239)	0,000394454	Ncaph,Pwp2,Errfi1,4930422G04Rik,D19Bwg1357e,Mki67,Elp2,Ddx52,Egr3,Cdc5l,Mrps6,Acaa2,Agxt2l2,Pde1a,Slc25a5,Snrpa1,Recql4,Ankrd57,Cbx3,Nusap1,Kpna2,Phf17,F630043A04Rik,Gli3,Pigf,Slc38a1,Patz1,Prkg2,Tnks2,Slco1a5,Foxp1,Cd14,Gprc5c,Fancd2,Pola2,Phlda1,Gart,Arf6,Terf1,Nxt1,Gtse1,Smarcc1,Eps8
**mmu-miR-298**	37(1239)	0,000397493	6430527G18Rik,Dffb,Prx,Fosl1,Ctsw,Ccne1,Rapgef2,Srpk1,Marcks,D2Wsu81e,Kif18a,Nol10,Ttll4,Impdh2,Lsm2,Tuba1c,Psrc1,Gsg2,Troap,Palld,Rad54l,2810474O19Rik,Pole,Ddx11,Sip1,Trpc2,Usp37,Ssrp1,Pafah1b3,Cd14,Rfc2,Etv4,Apex1,Incenp,Gtse1,Smyd5,Kif23
**mmu-miR-668**	39(1239)	0,000400232	Asph,Birc5,Elf2,Dsn1,Strbp,Mup4,Por,Mcm3,Csf1,Enpp1,Psrc1,Setx,Trib1,Pde1a,Matr3,Gpbp1,Peg12,Lig1,Lrrk1,Sf3a1,Zfp451,Zfp7,2810055F11Rik,Pvrl3,4930427A07Rik,Fbln1,U2af2,Ociad2,Nav3,Fancd2,Mre11a,Cdca8,Whsc2,Zfp281,Wdhd1,Cebpz,Fancc,AA408296,Zcchc11
**mmu-miR-218-1***	34(1239)	0,000405049	Vegfc,Tcf19,Cks1b,Spag5,Ppih,Nap1l4,Nipsnap1,Noc4l,Ccdc45,Impdh2,Hspa14,Pla2g4a,Smek1,Zmynd19,Pkn3,Abcb1b,Ube2c,Gtf2h2,Traip,Tk1,Ppbp,Cachd1,Sox4,Gemin8,Patz1,Foxp1,Pum2,Mcm6,Gprc5c,Mre11a,Ppwd1,Pou2f1,Arf6,Hnrpdl
**mmu-miR-671-5p**	44(1239)	0,000420598	Etaa1,Chd1,Tgif2,Ncaph,Tmem39b,Prpf31,Fhl2,Ctsw,Lrig3,Kl,Riok2,Pdia6,Xkr5,Cyp26b1,Nfrkb,Apex2,Brca1,Snrpb,Gpd2,Csf1,Ppil1,Slc19a1,Iqgap3,Troap,Lrrk1,Hmga2,Bub1b,Gins4,Eno3,Slc9a3r1,Zcchc2,Fgd3,Prc1,H2afz,Ssrp1,Pold1,Mcm6,Fbxo32,Pola2,Rad51,4632434I11Rik,Pou2f1,H2afy2,Slc4a7
**mmu-miR-351**	41(1239)	0,000432552	2810046L04Rik,Cdca7l,Tmem39b,Impa2,Ccbe1,Tcf19,Sass6,Aurka,Cpsf4,Impdh2,Fen1,Ash2l,Rpl30,Cdc25a,Rasa3,Scrib,Troap,Rpl12,Ewsr1,Asxl1,Hmgb2,Pprc1,Itih2,Ints7,Wdr55,Ppif,Limd2,Nudc,Wdr73,Synj2,Usp37,Cacna1c,Syne2,Gprc5c,Rsrc2,Ppwd1,Lifr,Etv4,Cdca8,Phc2,Kif23
**mmu-miR-505**	40(1239)	0,000446911	H2afv,Prim2,Dhx9,Upf3b,Kif4,Noc4l,Nrf1,Socs3,Tmem48,Sltm,Schip1,Lcorl,Mcm3,Luc7l,Ash2l,Ung,Smek1,Lig1,Hnrpll,Gsg2,Ptpn2,Sbno1,Dhx15,Smndc1,Pprc1,Unc13c,Arid4b,Wdr55,Abhd10,Cdc40,Mycn,Syne2,Cep170,Lifr,Me2,Dyrk3,Gtse1,Mrpl18,Ecd,Hnrpdl
**mmu-miR-15b***	33(1239)	0,000482158	Nup54,Rbmxrt,Whsc1,Ifi205,Kif2a,Sass6,Nipsnap1,Pgm1,Ddx52,Mup4,Tiam2,Utp18,Cdk2,Dbr1,Rpl30,Cul4b,Gtf2h2,Ewsr1,Hmga2,Dimt1,Gins4,Nfx1,Snrpd1,Pkp2,Hat1,Sip1,Pvrl3,Plscr1,Cd14,Psmd5,Thoc2,Cebpz,Cdc73
**mmu-miR-106b***	32(1239)	0,00050362	Arhgap22,2700050L05Rik,H2afv,Dgcr8,Rnmt,Fhl2,Txnrd1,Med4,Ube2e3,Aspm,Vil1,Zw10,Zmynd19,Crlf1,Hbs1l,Zfp451,Slco4a1,Myo1b,Colec12,Uchl5,Pprc1,Pank4,Anln,Cenpf,Fbln1,Wdr73,Sertad1,Ociad2,Ssrp1,Abl2,Gart,Ppa1
**mmu-miR-742***	29(1239)	0,000565771	Prdx4,Dctd,U90926,Cdc5l,Myef2,Cep68,Nuf2,Luc7l,Armc8,Sgol1,BC031781,4930547N16Rik,Crlf1,Set,Hmgb1,Espl1,E330009J07Rik,2810055F11Rik,Rpa2,Pkp2,Psmd14,Nab2,Lrig1,Wdr73,Gas2l3,Ilf3,Eif5,Rasal2,Slc4a7
**mmu-miR-18a**	43(1239)	0,000587408	Arhgap22,Pole2,Spag5,Dsn1,Egr3,D2Wsu81e,Ilf2,Ttll4,Cdk2,Cenph,Usp34,Rad51c,Troap,Gtf2h2,Ptpn2,2210018M11Rik,Fanca,Prpf38a,Nupl1,Kpna2,Ccna2,Wee1,Hn1,Nme4,Arid4b,Cenpa,Pigf,Anln,Mcm7,Neto2,Pbk,Ahcy,Actl6a,Mastl,Cdkn2c,Foxp1,Mycn,Ociad2,Cnot6,Stmn1,Cnn3,Ivns1abp,Rbm12
**mmu-miR-540-3p**	46(1239)	0,00061465	Ahsa1,D19Bwg1357e,Llgl1,Mum1,Mybbp1a,Mki67,Nap1l4,Aurkb,Zfp57,Pom121,Egr2,Tmem176b,Sltm,Blm,Dhx36,Pim3,Rrm2,Agxt2l2,Ccdc99,Egfr,Setx,Slc25a5,Scrib,Hbs1l,Prpf38a,Cbx3,Hmgb1,Nusap1,Ptcd3,Axl,Racgap1,Jub,Tpm3,Cdkn2c,Pkmyt1,Mboat2,Ociad2,Nav3,Rfc2,Tacc3,Pola2,Cdca8,Arf6,2010204K13Rik,Eftud2,Eps8
**mmu-miR-204**	37(1239)	0,000618892	Birc5,Ifi205,Ktn1,Taf5,Pak1,Prpf19,Cenpo,Mad2l1,Ilf2,Sltm,Sox11,Cdca2,Eef1e1,Zmynd19,Abcb1b,Rad54l,Zwilch,Cenpn,Exosc2,Patz1,Kcnk2,Synj2,Ssrp1,Slc43a1,Spc24,Pum2,Dcbld1,Zcchc8,2610027L16Rik,Thoc2,Fancc,Cdc73,Terf1,Gtse1,1810011O10Rik,Vash2,Pogz
**mmu-miR-490**	40(1239)	0,000642919	H2afv,Prkar2b,Pwp2,Gmnn,Mybbp1a,Spag5,Lrig3,Xkr5,Tyw3,Mrps6,Snrpb,Klhdc2,Ppil1,Crlf1,Rad51c,Gpr126,Hyal2,Pttg1,Kpna2,Colec12,Npm1,Tk1,Nt5c3l,Pkp2,Axl,Igf2bp1,Ppif,Dut,Cct3,Prkg2,Slco1a5,Rbm14,Syne2,Fancd2,Rasal2,Snrpb2,Incenp,Rbm12,Arf6,Rbpj
**mmu-miR-879***	29(1239)	0,000647676	Pcm1,Vps36,Sgol2,Casp3,Dusp9,Aebp2,Myc,Cand1,Ccdc99,Zmynd19,Cbx1,Peg12,Parp12,Ccng2,Gtf2h2,Rpl12,Rbm25,Gins4,Itgav,Top2a,Snrpg,Zwilch,Pigf,Tinf2,Polr3b,Mastl,Foxp1,Tacc3,Incenp
**mmu-miR-331-5p**	38(1239)	0,000670128	Shmt1,Nup54,Marcksl1,2810008M24Rik,Emb,Med4,Strbp,Cep192,Exosc8,Mrps6,Acaa2,Lsm2,Sgol1,Zfp184,Ppil1,Zmym1,Ctnna1,Adss,Casc5,Idh2,Sgms1,Prpf38a,Sumo2,3110003A17Rik,Rbmx2,Eif4enif1,Dus3l,4930427A07Rik,BC016423,Synj2,Gas2l3,Cct8,Slc43a3,Ddx10,Gprc5c,Ccdc18,4632434I11Rik,Cebpz
**mmu-miR-412**	35(1239)	0,000671058	Nck2,Impa2,D19Bwg1357e,Txnrd1,Rangap1,1110020G09Rik,Srpk1,Noc4l,E2f8,Nrp1,2510012J08Rik,Fxn,BC055324,Nup93,Scarb1,Dusp4,Pla2g4a,Ankrd10,Ccnf,Scrib,Smc5,Rps9,2310057M21Rik,Crebzf,Ncam1,Aifm1,Mpp6,Casp8ap2,Dapp1,Mcm2,Ddx39,Pcf11,Incenp,Kif23,Eps8
**mmu-miR-188-5p**	41(1239)	0,000684867	Lrrc45,Impa2,D19Bwg1357e,Eef1g,Plk1,Wdr3,Cit,Nol10,Ttll4,Mcm3,Lsm2,Nudt1,Rpl30,Ncapd2,Chuk,Tslp,Recql4,Ptpn2,Wipf1,Rad54l,Sema4b,Pprc1,2610301G19Rik,Kin,Melk,Axl,Rbmx2,Eif4enif1,Ptrh2,Synj2,Ilf3,Mboat2,Syne2,Akap8,Fancd2,Nob1,Rsrc2,Fignl1,Cdca8,Ccdc18,Tpbg
**mmu-miR-214***	30(1239)	0,000686236	Prpf31,Whsc1,Nol9,Kif2a,Wdr43,Ccne1,Msh2,Sgol2,Fxn,Cand1,Eme1,Ube2c,Palld,Ncaph2,Cdc7,Itgav,Axl,Zbtb12,Kcnk2,Chtf18,Cdc40,Slc43a1,Rrad,Nudt14,C79407,Rcl1,Erh,Etv4,Pou2f1,Rbpj
**mmu-miR-133b**	42(1239)	0,000690067	Tnfaip8,Prpf31,Rcc2,H2afv,Elf2,Lrig3,1110020G09Rik,Pgm1,Riok2,6720463M24Rik,D2Wsu81e,2510012J08Rik,Prim1,Nudt1,Aftph,Cep57,Ctbp2,B230120H23Rik,2700029M09Rik,Peg12,X99384,Rad54l,Chaf1b,Nmral1,Rad18,Sfxn1,Prr11,Pvrl3,Cct3,Chtf18,Slco1a5,Usp37,Pold1,Gprc5c,Rcl1,Ptbp2,Th1l,Rpl13a,Whsc2,Phlda1,Cebpz,Lif

miRNA name	*Number of Genes*	*Corrected p-value*	*miRNA targets among genes repressed in Rasless cells (Table S1)*
**mmu-miR-501-5p**	38(1239)	0,000745255	1110012J17Rik,Tmem39b,Prim2,Nup54,Prkar2b,Errfi1,Rbmxrt,Cpsf2,Cks1b,Kif2a,Fubp1,1110020G09Rik,Aspm,Tmem176b,2510012J08Rik,Tmem173,BC055324,Pgk1,Casc5,Imp3,Ptpn2,Metap1,Rad54l,Slbp,Hmgb1,Spry2,Chaf1b,Nr2f2,Gli3,Igf2bp1,Ddx31,Stt3b,BC016423,Synj2,Cdc40,Atrx,1700025G04Rik,H2afy2
**mmu-miR-29b***	38(1239)	0,000765562	Smpdl3b,Lrig3,Strbp,Cep192,Ddx46,Pdia6,Tox,Ttll4,Nfrkb,Brca1,D2Ertd750e,Rrm2,Pgk1,Agxt2l2,Slc29a1,Bzw2,Ncapd3,Rsbn1,Rpl12,Ewsr1,Stoml2,Slco4a1,Tardbp,Sema4b,Nr2f2,Eno3,2810055F11Rik,2810025M15Rik,Wdr55,Dut,Cct3,Dus3l,Sertad1,Mast4,Ddx39,Dyrk3,Rfc4,Wdr36
**mmu-miR-202-5p**	38(1239)	0,000809386	Etaa1,Errfi1,Rnmt,Zfp334,2610101N10Rik,Nipsnap1,Rpl7a,Egr2,Nrp1,Wsb1,Apex2,D2Ertd750e,Fnbp1l,Luc7l,Cenph,Cand1,Lbr,Mbtps2,Dusp6,Sgms1,Nusap1,Slco4a1,Klhl23,Hmgb2,Nr2f2,Snrpd1,Prpf3,Paip1,Ttc3,Apitd1,Ncapg,Ddx10,Mcm6,Abl2,Rasal2,Cep170,Hells,Wdr36
**mmu-miR-875-5p**	40(1239)	0,000810515	Taf5,Arhgap11a,Ctsw,Impdh2,Tgif1,Apex2,Nfyb,D2Ertd750e,Lsm2,Fbxo5,Hspa14,Pik3c2a,Zmym1,Cbx1,Crlf1,Set,Ncapd3,Spc25,Zfp54,Prpf3,Mcpt8,Gli3,Pkp2,Pvrl3,Nudt21,3110003A17Rik,Limd2,Abhd10,Mlf1ip,Metap2,Ddx20,Ncapg,Prc1,Plscr1,Rfc2,Cenpk,Cebpz,Fancc,Hnrpdl,Cirh1a
**mmu-miR-150**	38(1239)	0,000929707	6430527G18Rik,Tmem39b,Cenpc1,Ccbe1,Atad2,Elavl1,Egr2,Egr3,Ahctf1,Pdap1,Dhx36,Fen1,Etf1,Tll1,Nudt1,Rpl30,Cbx1,B230120H23Rik,Lig1,X99384,Imp3,Rpl12,Stoml2,Spry2,Nfx1,3110003A17Rik,Exosc2,Ghr,Zrsr2,2410016O06Rik,Dapp1,Anp32b,Gprc5c,Rsrc2,Stil,Incenp,Arf6,Dnajc9
**mmu-miR-484**	39(1239)	0,00102261	Ncaph,Arhgap22,Nup54,Dhx9,F2rl1,Plk1,Kif4,Aurka,Ddx52,Xpo7,Bcl10,Parp1,Por,Dusp9,Pim3,Exo1,Ppil1,Psrc1,Ncapd2,Micall2,Lig1,Scrib,2210018M11Rik,Nxf1,Asf1b,Ier2,E330009J07Rik,Rpa2,2610301G19Rik,Cachd1,Jub,Tinf2,Zbtb12,Sertad1,Ilf3,Cacna1c,Ociad2,Stil,Hirip3
**mmu-miR-15b**	42(1239)	0,00106925	Tgif2,Diap3,Errfi1,Depdc1b,Ccne1,Pspc1,Cep192,Msh2,Ppat,Wsb1,Cdca5,Mrps6,Cct5,Cenph,Exo1,Cdc25a,Cep152,Kif21a,Shcbp1,Metap1,Nusap1,Spry2,Ncaph2,Cdca4,Top2a,Wee1,Psmd14,Hat1,Ppif,Lrig1,Neto2,Ptrh2,Ssrp1,Rrad,Stmn1,Mast4,Ddx39,1700025G04Rik,Rad51,Khdrbs1,4632434I11Rik,Ecd
**mmu-miR-873**	40(1239)	0,00108495	Tgif2,Dffb,Kif2a,Mum1,Sass6,Strbp,Xpo7,Pdia6,Als2cr12,Nuf2,Ythdc1,2510012J08Rik,Fndc4,Por,Pgk1,Exo1,Enpp1,Matr3,Trim28,Dtl,Gsg2,Satb2,H2afy,Spred1,Pttg1,Cdc7,Bub1b,Neil3,Rfc5,Nt5c3l,Slc9a3r1,Kif2c,Mphosph10,Xpo1,Apitd1,Wdr73,Lmnb1,Dcbld1,Rpl13a,Etv4
**mmu-miR-184**	38(1239)	0,00115292	Ncaph,Cdca7l,Gemin4,Rbmxrt,Vegfc,Cenpj,Yme1l1,Cyp26b1,Pdap1,Dhx36,Apex2,Mthfd1,Snrpb,Fbxo5,Nudt1,Glul,Pkn3,Supt16h,Ube2c,Hbs1l,Tial1,Sf1,E330009J07Rik,Cdc7,Hn1,Zcchc2,Psmd14,Cenpn,Nudc,Cdkn2c,Tnrc6a,Slc43a3,Tacc3,Lifr,Phc2,Incenp,Cenpi,Dnajc9
**mmu-miR-327**	37(1239)	0,00116767	Nono,Prim2,Eef1g,Has2,Hmmr,Dctd,Aurka,Ddx52,Pdia6,6720463M24Rik,Ruvbl1,Nol10,Ttll4,Pdap1,Prim1,Sfpq,Pgk1,Sgol1,Larp7,Gins1,Zfp217,Pcnt,Sgms1,Sf3a1,Snrpd1,Nme4,Gli3,Hat1,Plxnb1,Cdca3,Mcm7,Lmnb1,Akap8,Ppwd1,Ezh2,Kif23,Eif2s1
**mmu-miR-671-3p**	42(1239)	0,00117544	Nedd4l,Lrrc45,Arhgap22,Prkar2b,Prx,Tcf19,Tnnt2,Plk1,Prpf19,Aurkb,Ttll4,Cdca5,Zw10,Pdap1,Cct7,Nudt1,Ccnf,Rad51c,Pcnt,Rps9,Cul4b,Imp3,Ddx51,Rpl12,Wipf1,Trim25,Ankrd57,Bub1b,Casp6,Zbtb12,Stt3b,Wdr73,Slc43a1,Fmnl3,Gprc5c,Cdca8,Pebp1,Ppa1,Ift74,Dock5,Smyd5,Eftud2
**mmu-miR-741**	32(1239)	0,00144426	Ncaph,D19Bwg1357e,Ifi205,Aurkb,Cenpj,Dnmt1,Rbbp7,Marcks,Ttll4,Acaa2,Brca1,BC055324,Myc,Agxt2l2,Sgol1,Rpl30,Sgms1,Prpf38a,Hyal2,Traip,E330009J07Rik,Ctsh,Pigf,Cdkn2c,Foxp1,Fancd2,Dtymk,Me2,4632434I11Rik,Rfc4,Eftud2,Smc4
**mmu-miR-376a***	33(1239)	0,00152292	Snx5,Tgif2,Sap30,Impa2,Nol9,Crim1,Pgm1,Riok2,Aspm,Ctcf,Pde1a,Eme1,Gsg2,Gtf2h2,Rpl12,Cobll1,Zfp7,Ncam1,E330009J07Rik,Unc5c,Pank4,Trit1,Tpx2,Fgf7,Stt3b,Ddx20,Tpp2,Plekha5,Spp1,Dtymk,Th1l,Nsl1,Rfc4
**mmu-miR-761**	38(1239)	0,00153692	Ahsa1,Fhl2,Eef1g,Kif2a,Cbx2,Egr3,Ttll4,Tmem48,Cpsf4,Acaa2,Tubb5,Sfpq,Cenph,Nudt1,Hmga1,Ung,Ncapd2,Pkn3,Psmc3ip,Crlf1,Cobll1,Rasa1,Sema4b,Phf17,Wee1,Hn1,Cenpe,Fgd3,Mphosph10,Foxp1,Dapp1,Mcm6,Dcbld1,Ddx39,Pola2,Gart,Ppa1,Dnajc9
**mmu-miR-10a**	37(1239)	0,00160729	Birc5,Hspa8,6720463M24Rik,Ppat,Kif18a,Rnps1,Cand1,Scarb1,Smarce1,Ccdc99,Ncapd2,Bzw2,Atad5,Lrrk1,Ddx51,Prpf38a,Traip,Tead2,Hn1,Igsf3,Sip1,Slc38a1,Tipin,Dus3l,Apitd1,Zbtb12,2410016O06Rik,Abl2,Pola2,C330027C09Rik,Ccdc18,Pou2f1,Arf6,Cdh10,Gtse1,Kif23,Eps8
**mmu-miR-27a***	34(1239)	0,00173114	Zfp52,Kif4,1110020G09Rik,Dnmt1,Ube2e3,Xkr5,Mcm4,Ttll4,Lsm2,Tll1,Pcna,Slc19a1,Gins1,Rad51c,Ssb,Rad54l,Gli2,Cep70,Nme4,Nt5c3l,Cdca3,Mphosph10,Patz1,Nutf2,Nudc,Tnks2,Eif5,Pafah1b3,Nob1,Pebp1,Ccdc18,Nxt1,Gtse1,Ahcyl1
**mmu-miR-146b***	29(1239)	0,00181783	Pwp2,Whsc1,Gmnn,Plk1,Ddx21,Rangap1,Fkbp5,Parp1,Zic1,Nudt1,Cdca2,Dtl,Lig1,Rad51c,Acsl3,Sf3a1,Itgav,Unc13c,Mcpt8,Ptcd3,Cdca3,Gemin8,Nudc,Pbk,Rfpl4,Ly75,Fancd2,Incenp,Fancc
**mmu-miR-376c***	29(1239)	0,00181783	Tgif2,Sap30,Timm8a1,Gemin4,Ankrd32,Ifi205,Depdc1b,Cenpq,Dis3,Kif18a,Hspd1,Pgk1,Fbxo5,Dbr1,Sgol1,Slc25a5,Gtf2h2,Rpl12,Cobll1,Snrpd1,Snrpg,Anln,3110003A17Rik,Usp37,Fbxo32,Cep170,Ppwd1,Pcf11,C330027C09Rik
**mmu-miR-130b***	26(1239)	0,00182053	Arhgap22,H2afv,Ranbp1,Impa2,Kif4,2010002N04Rik,1110020G09Rik,Cbx1,Pkn3,Cul4b,Rpl12,Wipf1,Rad54l,Nr2f2,Nmral1,Pkp2,Rps13,Sertad1,Nsmce4a,Pabpn1,Ssrp1,Spp1,Cenpk,Phlda1,Nsl1,Terf1
**mmu-miR-30c-2***	38(1239)	0,00192144	H2afv,Dhx9,Eef1g,Dkc1,Ctsw,Cenpq,1110020G09Rik,Ddx27,Pim3,Mcm3,Lsm2,Cand1,Rpl30,Slc25a5,Hyal2,Ier2,Cdc7,Gins4,Eno3,Pprc1,F630043A04Rik,Gli3,Trit1,Ckap4,Cdca3,Cct3,Dus3l,Nudc,Mcm7,Fbln1,Ahcy,Cdkn2c,Ociad2,Stmn1,Dcbld1,Rcl1,2610027L16Rik,Ddx39
**mmu-miR-763**	38(1239)	0,00207108	Nck2,Whsc1,Eef1g,Llgl1,Dkc1,Spag5,Ctsw,Sass6,Paxip1,Egr3,Lrp8,Fen1,Snrpb,Nup93,Pgk1,Ddx18,Cct5,Luc7l,Zmynd19,Slc29a1,Rps9,H2afy,Hyal2,Wipf1,Ncaph2,Rpa2,Hn1,Pole,Pvrl3,Apitd1,Bnc2,Pold1,Fancd2,Rfc2,Rcl1,Pou2f1,Ezh2,Eif2s1
**mmu-miR-801**	33(1239)	0,00217159	Prim2,Ercc6l,Fancm,Ankrd32,Depdc1b,Elf2,Tnnt2,Cenpq,Prpf19,Setdb2,Riok2,Tmem176b,D2Wsu81e,Tiam2,Gins1,Dtl,Satb2,Gtf2h2,Rpl12,Nupl1,Bub1b,Gins4,Top2a,Gli3,Ube2t,Ddx31,Plekha5,Dtymk,AI848100,Zfp281,Ezh2,Tmpo,Kif23
**mmu-miR-681**	37(1239)	0,00222667	Nck2,Gata2,Rcc2,Fhl2,Nol9,Llgl1,Wdr43,Ppih,Dnmt1,Xkr5,Egr3,Cdc5l,2510012J08Rik,Tgif1,Cct7,Fen1,Gins2,Eef1e1,Pkn3,Eif4a3,2700029M09Rik,Casc5,Ppp1cc,Depdc1a,Nxf1,Rad54l,Ncaph2,Sema4b,Ints5,Dus3l,Ddx31,Topbp1,2410016O06Rik,Plekha5,Ssrp1,Nob1,Cdca8
**mmu-miR-720**	34(1239)	0,00224855	Ncaph,Lrrc45,D19Bwg1357e,Nol9,Zfp334,Has2,1110020G09Rik,Dnmt1,6720463M24Rik,D2Wsu81e,Cep68,Eme1,Dusp5,B230120H23Rik,Lig1,Scrib,Pcnt,Rpl12,Palld,Spry2,Rbm25,Aifm1,Racgap1,Prmt5,Sertad1,Ncapg,Rfc2,E330016A19Rik,Cenpk,Cnn3,Cebpz,Fancc,Eftud2,Hnrpdl
**mmu-miR-193***	33(1239)	0,00254877	Pcm1,Whsc1,Hspa8,Nol9,Mybbp1a,Ctsw,Ets2,Fxn,Sltm,D2Ertd750e,Snrpb,Pgk1,Exo1,Emg1,Eif4a3,Casc5,Satb2,Rsbn1,Lrrk1,Fbl,Ckap2l,Cdc7,Top2a,Itih2,Slc38a1,Cdc40,Ddx20,Ssrp1,Cd14,Cenpk,Cnn3,Nxt1,Smyd5
**mmu-miR-99b**	36(1239)	0,00264801	Ahsa1,Arhgap22,Birc5,Pak1,Nap1l4,Ythdf2,Noc4l,6720463M24Rik,Mrps6,Denr,Hmga1,Slc29a1,Eme1,2610039C10Rik,Prpf4,Chuk,Supt16h,Ccnf,Ccng2,Mtap,Stoml2,Trim25,Nupl1,Card10,Top2a,Cep70,Igsf3,Apitd1,Smarca5,Rrad,Fmnl3,Col7a1,Cdca8,Nol11,Cdca7,E130308A19Rik

miRNA name	Number of Genes	Corrected p-value	miRNA targets among genes repressed in Rasless cells (Table S1)
**mmu-miR-718**	33(1239)	0,00268076	Lrrc45,Ahsa1,Clns1a,Whsc1,Skp2,D19Bwg1357e,Egr3,Ank3,Ttll4,Cpsf4,Impdh2,Adamts7,Nfyb,Mcm3,Zic1,Nudt1,Slc29a1,Pkn3,Psmc3ip,E130303B06Rik,Lrrk1,Ncaph2,Gins4,Unc13c,Aifm1,Magoh,Mpp6,Igf2bp1,Lrig1,Zbtb12,Actl6a,E2f7,1700054N08Rik
**mmu-miR-125a-5p**	42(1239)	0,00272057	Asph,Tmem39b,Impa2,Ccbe1,Sass6,Setdb2,Elavl1,Pdia6,Impdh2,Cdca2,Rasa3,Troap,Ewsr1,Stoml2,Zfp451,Tube1,Usp3,Hmgb2,Top2a,Itih2,Ptk7,Arid4b,Racgap1,Wdr55,Limd2,Cenpf,Nudc,Zbtb12,Wdr73,Synj2,Usp37,Dapp1,Syne2,Fbxo32,Gprc5c,Arhgap19,Ppwd1,AI848100,Cdca8,Phc2,Zfp281,Lif
**mmu-miR-147**	37(1239)	0,00276382	Skp2,Plk1,Fubp1,Setdb2,Gspt1,Ttll4,Pim3,Mcm3,Nup93,Usp10,Cand1,Gjc1,Usp14,Kif21a,Baz1a,Idh2,Nasp,Nusap1,Topors,Itgav,Ckap2,Rad18,Pank4,Hat1,Igf2bp1,Tinf2,Ilf3,Tnrc6a,E2f7,Arhgap19,Rpl13a,Cdca8,Cebpz,Fancc,Tpbg,Slc4a7,Vash2
**mmu-miR-678**	37(1239)	0,00282785	Sap30,Rcc2,Lrig3,Pank1,Kif4,1110020G09Rik,Mrps6,Sltm,Acaa2,Lsm2,Klhdc2,Agxt2l2,Zfp184,Emg1,Ncapd2,Eif4a3,Kif21a,Pcnt,Hbs1l,Imp3,H2-K1,Usp3,Sema4b,4930579G24Rik,Ctsh,Zwilch,Axl,Zrsr2,Ddx20,Mcm6,Fbxo32,Phlda1,Pebp1,Nxt1,Dock5,Eftud2,Slc4a7
**mmu-miR-483***	29(1239)	0,00288978	Nono,Tgif2,F2rl1,Mum1,Prpf19,Tes,Nap1l4,Rcc1,Psrc1,Setx,Cbx1,Lig1,Ier2,Snw1,Tead2,Nr2f2,Egr1,Trit1,Cachd1,Jub,Tbx3,Rad51ap1,U2af2,Ssrp1,Gprc5c,Me2,Gart,Egfl7,2810408A11Rik
**mmu-miR-683**	35(1239)	0,00321053	Socs5,Skp2,Prx,Dsn1,Srpk1,Rpl7a,Ilf2,Vrk3,Cbx1,Pkn3,Micall2,Gnb4,Rpl12,Palld,Rpa2,Slc9a3r1,Rbmx2,Limd2,Ss18,Prmt5,U2af2,Dapp1,Syne2,Dtymk,Hirip3,Rasal2,Cdca8,Phc2,Cebpz,Nxt1,Dock5,Rrs1,Eif2s1,1810011O10Rik,Dlx1
**mmu-miR-675-5p**	40(1239)	0,00347685	6430527G18Rik,Dhx9,Mum1,Etv6,Tnnt2,Fus,Cep192,Elp2,Ruvbl1,Egr3,Dis3,Tmem48,Dusp9,Lsm2,Usp10,Pgk1,Zfp36l2,Emg1,Bzw2,Crlf1,Hn1l,Sgms1,Imp3,Ptpn2,Mtap,Fanca,Sf3a1,Cbx3,Snw1,Nme4,Runx1,Nt5c3l,Clspn,Apitd1,Mcm7,Cdc40,Ilf3,Slc43a1,Etv5,Me2
**mmu-miR-379**	36(1239)	0,00352129	Impa2,Ccne1,Elp2,Ube2e3,Nrf1,Sgol2,Sin3a,Tmem173,Cct7,Pgk1,Tuba1c,Klhdc2,Trip13,Runx2,U2af1,Spc25,Crebzf,Acsl3,Slco4a1,Cdc7,Nme4,Unc5c,Mtm1,Tpx2,Zcchc2,Ppbp,Ints7,Xpo1,Prmt5,Tacc3,Tex10,Lifr,Tmpo,Arf6,Dnajc9,Cirh1a
**mmu-miR-485**	35(1239)	0,00371071	Ncaph,Mum1,Ctsw,Srpk1,Fxn,Ddx18,Nfatc2ip,Eme1,Eif4a3,Scrib,E130303B06Rik,Tial1,Hyal2,Stoml2,Bub1b,Topors,Pprc1,Prpf3,2610301G19Rik,Pkp2,Mpp6,Cenpn,Pvrl3,Racgap1,Gemin8,Usp37,Rif1,Pkmyt1,Plekha5,Tnrc6a,Actr3,Ssrp1,Mre11a,Th1l,Mrpl18
**mmu-miR-431**	35(1239)	0,00389142	Lrrc45,Gemin4,Dffb,Prim2,Impa2,Fhl2,Spred2,Crim1,Atad2,Ncl,Ythdf2,Dnmt1,Tcerg1,Mpzl1,Scrib,Recql4,Depdc1a,Fanca,Hmgb1,Ran,Chaf1b,Ckap2l,Mns1,Pank4,Psmd14,2810025M15Rik,Wdr55,Tpm3,Stt3b,Cacna1c,Dapp1,Litaf,Dtymk,Fancc,1810011O10Rik
**mmu-miR-299**	34(1239)	0,00410008	Pcm1,Rcc2,Dnaja2,Fus,Ctsw,Ppih,Ddx27,Cdca5,Taf5l,Tmem173,Lsm2,Luc7l,Nudt1,Asxl3,Rpl30,Pdk3,Crlf1,Smc5,Sgms1,Myo1b,Rasa1,Nt5c3l,Ctsh,Pole,Tinf2,Eif4enif1,Rrad,Zcchc8,Dtymk,Ppwd1,Khdrbs1,Ltbp1,Terf1,Tcf4
**mmu-miR-433**	34(1239)	0,00463125	Arhgap22,Pcm1,Gemin6,Nipsnap1,Ncl,Cenpj,Aspm,Vil1,Ilf2,Zw10,Msh6,Mcm3,Asxl3,Ung,Pdk3,Iqgap3,Prpf38b,Troap,Gtf2h2,Depdc1a,Ewsr1,Usp3,Tardbp,Syt17,Sfxn1,Pvrl3,Fgf7,Nutf2,Nsmce4a,Rfpl4,Utp11l,Gart,E130308A19Rik,Cirh1a
**mmu-miR-744**	39(1239)	0,00478341	Nck2,Nedd4l,Ahsa1,Asph,Khsrp,Whsc1,Tnnt2,Ctsw,Plk1,Rangap1,Swap70,Dnmt1,Tmem176b,Ptpre,Zc3h15,Cit,2510012J08Rik,Acaa2,Por,Adamts7,Junb,Nudt1,Agxt2l2,Micall2,Snrpa1,Sbno1,Traip,Cdca4,Ckap2,Rpa2,Nt5c3l,2610301G19Rik,U2af2,E2f7,Nudt14,C79407,Nob1,Fancc,Nxt1
**mmu-miR-136**	32(1239)	0,00527562	Asph,Zfp292,Tnpo1,Amd1,Mki67,Mnd1,Nipsnap1,1110020G09Rik,Rpl7a,Nuf2,Dis3,Nrp1,Msh6,Msh3,Asxl3,Cep57,Setx,Ctnna1,2700029M09Rik,Casc5,Ccng2,Rpl12,Hyal2,2810474O19Rik,Ncam1,Pprc1,Zbtb12,Ddx20,Yes1,Pou2f1,Slc4a7,Socs4
**mmu-miR-345-5p**	37(1239)	0,00537851	Arhgap22,Prpf19,Ank3,Slc11a2,Apex2,Mcm3,Snrpb,Nudt1,Scarb1,Slc29a1,Eme1,Bzw2,Crlf1,Ppp1cc,Wipf1,Spry2,Ier2,Bub1b,Nt5c3l,2610301G19Rik,Trit1,Igf2bp1,Tbx3,Chtf18,Prmt5,Ddx31,2410016O06Rik,Ssrp1,B3galnt1,Pafah1b3,Bhlhb9,Gprc5c,Col7a1,Eif4h,Ing5,Etv4,Fancc
**mmu-miR-26b***	28(1239)	0,00550666	Upf3b,Ddx27,Nrp1,Pdap1,Lsm2,Klhdc2,Myc,Nudt1,Cdca2,Slc25a5,Ccnb2,Usp14,Smc5,Pcnt,Sgms1,Itgav,Rfc5,Cenpf,Tinf2,Zbtb12,BC016423,Wdr73,Dapp1,Ociad2,Slc43a1,Spc24,Pou2f1,Nxt1
**mmu-miR-187**	34(1239)	0,00569729	Rbm26,Trim24,Dcp1a,1110020G09Rik,Pdap1,Lcorl,Nup93,Nfatc2ip,Etf1,Gins1,Ncapd3,Spc25,Hbs1l,Fanca,Tial1,Nasp,Wdr75,Phf17,Psmd14,Wdr55,Limd2,Zfp619,Mcm7,Prc1,Mycn,Nav3,Bcl2l11,Cdca8,H2afx,Pou2f1,Cebpz,Fancc,Arf6,Terf1
**mmu-miR-93***	31(1239)	0,00610769	Ercc6l,Whsc1,Depdc1b,Nol9,Mybbp1a,Aurkb,Ank3,Tmem48,Acaa2,Csf1,Sgol1,Mbtps2,Mpzl1,Lrrk1,Kars,Colec12,Bub1b,Zcchc2,Prr11,Slc38a1,Apitd1,Prmt5,Slc43a3,Specc1,Dtymk,Rsrc2,Trub1,Cdca8,Gmeb1,Cdca7,Eps8
**mmu-miR-18b**	38(1239)	0,00626237	Arhgap22,Diap3,Elf2,Spag5,2810008M24Rik,Egr3,D2Wsu81e,Por,Nudt1,Pde1a,Cbx1,Gtf2h2,Ptpn2,Casp8,2210018M11Rik,Fanca,Prpf38a,Nupl1,Rasa1,Ccna2,Tk1,Hn1,Arid4b,Cenpa,Pigf,Mcm7,Ahcy,Actl6a,Mastl,Cdkn2c,Foxp1,Pkmyt1,Ociad2,Spc24,Eif4h,Cnn3,Rbm12,Nxt1
**mmu-miR-679**	33(1239)	0,00644399	Lrrc45,Gata2,Pcm1,H2afv,Eef1g,Prpf19,Kif4,Nipsnap1,Parp1,Taf5l,Brca1,BC055324,Snrpb,Idh2,Satb2,Rpl12,Sf3a1,Usp6nl,Tyms,Card10,Gtpbp10,Neil3,Tpx2,Pkp2,Psmd14,2810025M15Rik,Pvrl3,Dut,Cct3,Cep170,1110004E09Rik,Nsl1,Klf5
**mmu-miR-339-5p**	36(1239)	0,00646342	Fmr1,Lrrc45,Gata2,Ell,Tcf19,Prpf19,1110020G09Rik,Xpo7,Dusp9,Msh3,Rrm1,Zmym1,Adss,Set,Ube2c,Fbl,E330009J07Rik,Cdc7,Phf17,Rfc5,Chd2,Prps1,Fgd3,Prkg2,Dus3l,Zbtb12,Sertad1,Cnot6,Slc43a1,Fmnl3,Pafah1b3,Gmeb1,Incenp,1700054N08Rik,Ltbp1,Gtse1
**mmu-miR-688**	34(1239)	0,00712087	Timeless,H2afv,Birc5,Prx,Dhx9,Mum1,Dcp1a,Cdc5l,Vrk3,Tubb5,Junb,BC055324,Pgk1,Ctbp2,Zmynd19,Slc29a1,Eme1,Ncapd2,Scrib,Ncapd3,2210018M11Rik,Sf3a1,Hat1,Pole,Mpp6,Slc43a1,Slc43a3,Gprc5c,Nob1,Myct1,E130308A19Rik,Tpbg,2810408A11Rik,Eps8
**mmu-miR-667**	36(1239)	0,00767968	Chd1,H2afv,Tnpo1,Ranbp1,Upf3b,Nsun2,Noc4l,Aurka,2510012J08Rik,Slc11a2,Rrm2,Cep57,Ppil1,Gjc1,Chst1,Set,Recql4,2210018M11Rik,Tead2,Phf17,Pprc1,Wee1,Nmral1,Nt5c3l,Tpx2,Igsf3,Gemin8,Prkg2,Dus3l,Dapp1,Mboat2,Gprc5c,Rfc2,Erh,Mrpl18,Hnrpdl
**mmu-miR-541**	31(1239)	0,00828323	Tcf19,Ctsw,Pgm1,Aspm,Vil1,Ruvbl1,Egr3,Vrk3,Slc11a2,Sltm,Lsm2,Ddx18,Tll1,Nudt1,Rcc1,2610039C10Rik,Lrrk1,Usp3,Sema4b,Mpp6,Rbm17,Qars,Mphosph10,Esco2,Tnks2,Plekha5,Fancd2,Gmeb1,Me2,Fancb,Eps8
**mmu-miR-337-3p**	34(1239)	0,00866691	Mtmr4,Nol9,Tnnt2,Nipsnap1,Wdr3,Cep192,Ube2e3,Ttll4,Msh6,Hdac2,Fnbp1l,Myc,Nudt1,Asxl3,Mbtps2,Spc25,Nxf1,Phf17,Pvrl3,3110003A17Rik,Rps13,Nutf2,Sertad1,2410016O06Rik,Foxp1,Plekha5,Tnrc6a,5730559C18Rik,Slc43a1,Syne2,Rpl13a,AI848100,Top1,Rbpj
**mmu-miR-804**	30(1239)	0,00917464	Nedd4l,Ahsa1,Pop1,Rbm26,Eef1g,Fosl1,Med4,Cep192,Mup4,Nuf2,Sltm,Luc7l,Rrm1,Ppil1,Psrc1,Gins1,Ppp1cc,Tead2,Snrpd1,Rpa2,Arid4b,Sip1,Fzd3,Bbs12,Polr3b,Plscr1,Esf1,Cd14,Rbm12,Kif23
**mmu-miR-145***	24(1239)	0,0101009	Spag5,Lrig3,Vil1,D2Wsu81e,Msh6,Mcm3,Lsm2,Tuba1c,Ppil1,Usp34,Pcnt,Hyal2,Nxf1,Rad54l,Ubqln2,Nt5c3l,Kif2c,Cct3,Slc43a3,Pdss1,Gmeb1,Phlda1,Cebpz,Ecd
**mmu-miR-331-3p**	33(1239)	0,0102391	Lrrc45,Tmem39b,Wdr43,Fosl1,Plk1,D2Wsu81e,Pdap1,Fndc4,Por,Cdca2,E130303B06Rik,Idh2,2210018M11Rik,Rpl12,Sf3a1,E330009J07Rik,Sema4b,Gli2,Rpa2,Nme4,Gli3,Igsf3,Cdca3,Exosc2,Sox4,Fgd3,Prmt5,Trpc2,Synj2,Rbm14,Pold3,Dapp1,Tacc3

miRNA name	*Number of Genes*	*Corrected p-value*	*miRNA targets among genes repressed in Rasless cells (Table S1)*
**mmu-miR-92a***	30(1239)	0,0107495	Ahsa1,Fhl2,Ruvbl1,Egr3,Por,Adamts7,Brca1,Sh3kbp1,Hmga1,2610039C10Rik,Psmc3ip,Pcnt,Rsbn1,Troap,Imp3,H2afy,Usp3,Cdca4,Tk1,Prpf3,Ints5,Patz1,Ociad2,Ssrp1,Ddx10,Tacc3,1110004E09Rik,Ddx39,Etv4,Gtse1
**mmu-miR-339-3p**	37(1239)	0,0110096	Lrrc45,Gemin4,Khsrp,Impa2,Ctsw,1110020G09Rik,Msh2,Ahctf1,Zw10,Tmem48,Mrps6,Pdap1,Brca1,Luc7l,Dusp4,Abcb1b,Gpbp1,Ankrd10,D1Bwg0212e,Casp2,Set,Ube2c,Nxf1,Unc5c,Zcchc2,Psmd14,Clspn,Patz1,4930427A07Rik,Pkmyt1,Fmnl3,Trub1,Cdca8,Pcf11,Cdca7,Rbpj,Eps8
**mmu-miR-101a***	27(1239)	0,0110715	Tmem39b,Cenpc1,Abi1,Rnmt,Prpf40a,Zfp334,Atad2,Ptpre,Mcm4,Cdca2,Cct2,Emg1,Gins1,Brd8,Runx2,E130303B06Rik,Prpf4b,Smndc1,Pttg1,Cdc7,Hmgb2,Anp32b,Actr3,Specc1,Lrrc8c,4632434I11Rik,Hnrpdl
**mmu-miR-211**	29(1239)	0,0136462	Prim2,Birc5,Caprin1,Ccbe1,Ktn1,Taf5,Prpf19,Nrf1,Slc11a2,Sltm,Cdca2,Eef1e1,Zmynd19,Abcb1b,Mbtps2,Rad54l,Egr1,Zwilch,Synj2,Ncapg,Ssrp1,Slc43a1,Spc24,Pum2,Zcchc8,2610027L16Rik,Phlda1,Cdc73,Pogz
**mmu-miR-500**	33(1239)	0,0139525	Nono,Asph,Timeless,Rcc2,Prkar2b,Impa2,Whsc1,D19Bwg1357e,Spred2,Tnnt2,Smpdl3b,Cep192,Riok2,Ank3,Cct7,BC055324,Gins1,Imp3,Ptpn2,Prpf4b,Rpl12,Ptges3,Adsl,Casp8ap2,3110003A17Rik,Lrig1,Zbtb12,Wdr73,Cdkn2c,Foxp1,Syne2,Gmeb1,H2afy2
**mmu-miR-672**	32(1239)	0,014562	1110012J17Rik,Pcm1,Dffb,Trim24,Mybbp1a,Dsn1,Trim59,1110020G09Rik,Cep192,Fxn,Casp3,Myc,Lin9,E130303B06Rik,Snrpa1,H2afy,Fanca,Kars,Gli2,Pprc1,Top2a,Neil3,Prpf3,Gli3,Ddx11,Kif2c,Cct3,2410016O06Rik,5730559C18Rik,Fancd2,Tacc3,Eftud2
**mmu-miR-205**	33(1239)	0,0147824	Etaa1,Khsrp,Birc5,Nme1,Txnrd1,Rapgef2,Ruvbl1,Cdc5l,Vrk3,Apex2,Msh3,Hspa14,Nudt1,4930547N16Rik,Satb2,Rpl12,Tial1,Hyal2,Tube1,Pkp2,Pvrl3,Trp53bp2,Fgd3,Wdr73,Polr3b,Sertad1,Synj2,Gprc5c,Th1l,Thoc2,Cdca8,Phc2,Top1
**mmu-miR-376b***	25(1239)	0,0161166	Tgif2,Sap30,Gemin4,Ankrd32,Ifi205,Setdb2,Dis3,Kif18a,Dhx36,Dbr1,Gtf2h2,Pnpt1,Rpl12,Cobll1,Slco4a1,Snrpd1,Neil3,Snrpg,Melk,Anln,3110003A17Rik,2410042D21Rik,Fbxo32,Ppwd1,Pcf11
**mmu-miR-666-3p**	35(1239)	0,0163462	Ncaph,Lrrc45,Asph,Dgcr8,Llgl1,Txnrd1,Ppih,Ripk2,Paxip1,Srpk1,Aurka,Ddx52,Mrps6,Pdap1,Armc8,2610039C10Rik,Cbx1,Runx2,Cul4b,Tial1,Wipf1,Trim25,Rbm25,Gli2,Nab2,Ankrd28,Tnrc6a,Cep55,Ssrp1,Nob1,2610027L16Rik,Eif4h,Ccdc18,Pou2f1,Smyd5
**mmu-miR-666-5p**	37(1239)	0,0195319	Whsc1,Steap1,Mum1,Tnnt2,Ddx52,6720463M24Rik,Fxn,Tmem48,Pdap1,Snrpb,Ash2l,Rrm1,Cd2ap,Slc29a1,Cbx1,Lbr,Psmc3ip,Gpbp1,U2af1,Pcnt,Sbno1,Lrrk1,Rpl12,Wipf1,E330009J07Rik,Hmga2,Slc9a3r1,Pank4,Pole,Apitd1,Pkmyt1,Slc43a1,E2f7,Nob1,Incenp,Wdhd1,Smc4
**mmu-miR-764-3p**	34(1239)	0,0199184	Ahsa1,Tmem39b,Birc5,Fhl2,Mum1,Ccne1,Setdb2,2010002N04Rik,Xkr5,Tmem176b,2510012J08Rik,Fndc4,Msh3,Larp7,Crlf1,Rad51c,Casp8,H2afy,Ewsr1,Card10,Tead2,Runx1,Ube2t,Racgap1,Wdr73,Polr3b,Prc1,Rfc2,Tacc3,Cdca8,Pcf11,4632434I11Rik,Pou2f1,Eftud2
**mmu-miR-882**	28(1239)	0,0199293	H2afv,Impa2,Plk1,Smpdl3b,Nipsnap1,Cdc27,6720463M24Rik,Tmem176b,Lrp8,Tubb5,Por,Cdca2,Itgav,Tk1,Ckap4,2810025M15Rik,Cdca3,Jub,Tinf2,Eif5,Dtymk,2610027L16Rik,Pola2,Apex1,Fancc,Cdh10,2010204K13Rik,Socs4
**mmu-miR-30c-1***	33(1239)	0,0204517	H2afv,Dhx9,Upf3b,Dkc1,Ctsw,Cenpq,Ppih,Nipsnap1,Ank3,Sgol2,Brca1,Pim3,Rpl30,Egfr,Eme1,Imp3,Ier2,Gins4,Eno3,Pprc1,F630043A04Rik,Gli3,Aifm1,Trit1,Cdca3,Nutf2,Nudc,Mcm7,Ahcy,Cdkn2c,Stmn1,2610027L16Rik,Ecd
**mmu-miR-370**	33(1239)	0,0208062	Nedd4l,Arhgap22,Pwp2,Birc5,Nol9,Cks1b,Fosl1,Prpf19,Setdb2,Pdap1,Emg1,Zmynd19,Cbx1,Idh2,Fanca,Prpf38a,Hyal2,Traip,Usp3,Anp32e,Rsf1,Fgf7,Igf2bp1,Trp53bp2,Apitd1,4930427A07Rik,Wdr73,Ssrp1,Fbxo32,Dtymk,Rpl13a,Ahcyl1,1810011O10Rik
**mmu-miR-127**	30(1239)	0,0222202	2810046L04Rik,Ahsa1,Timeless,Arhgap22,Nol9,Llgl1,Glrx,Taf5,Sass6,Aurkb,Ddx27,Ruvbl1,Egr3,Rhobtb3,Impdh2,Nup93,Rsbn1,Ankrd57,F630043A04Rik,Cachd1,Ttc3,Prmt5,Ddx31,Sertad1,Gprc5c,Rpl13a,2610027L16Rik,Pola2,Cdca8,Khdrbs1
**mmu-miR-423-5p**	32(1239)	0,026779	Nedd4l,Depdc1b,Spred2,Ctsw,2010002N04Rik,Ddx52,Aspm,Myef2,Nol10,2510012J08Rik,Ttll4,Ifitm3,Tubb5,Adamts7,Msh3,Hmga1,Slc29a1,Trim28,Eif4a3,Iqgap3,Ube2c,Idh2,Asxl1,Snw1,Patz1,Ilf3,Gprc5c,Pola2,Egfl7,Gtse1,Smyd5,Kif23
**mmu-miR-877**	28(1239)	0,0269296	Sap30,Timeless,Ankrd32,Eef1g,Ctsw,Prpf19,Wdr3,Zc3h15,Rnps1,Enpp1,Zmym1,Eif4a3,Mpzl1,Troap,Palld,Pprc1,Ints5,Cdca3,Exosc2,Cct3,Dus3l,Actl6a,Usp37,Dapp1,Plscr1,Cd14,Hirip3,Cenpk
**mmu-miR-191***	26(1239)	0,0308214	Gemin4,Arhgap22,H2afv,Ddx52,Ahctf1,Sgol2,Por,Fnbp1l,Smek1,2700029M09Rik,Imp3,2810474O19Rik,Ncam1,Nfkbiz,Cdc7,Hmgb2,Snrpd1,Mpp6,Cenpn,Zbtb12,Trpc2,Abl2,Fancd2,Bcl2l11,Smu1,Pou2f1
**mmu-miR-29c***	30(1239)	0,0311756	Wdr3,Riok2,6720463M24Rik,Vil1,Tiam2,Tox,Wsb1,Socs3,Nfrkb,Ppid,Lsm2,Zfp184,Enpp1,Ctbp2,Slc29a1,BC031781,Pcnt,Sbno1,2210018M11Rik,Chaf1b,Gtpbp10,Top2a,Ctsh,Mastl,Slc43a3,E2f7,Stmn1,Zcchc8,Ddx39,Pogz
**mmu-miR-135a***	27(1239)	0,0335964	Ctsw,Ddx21,Atad2,Ddx27,Aspm,Tmem176b,Vrk3,Por,Psrc1,Ung,Pde1a,Trim28,Cul4b,Pnpt1,Nasp,Pprc1,Paip1,Ctsh,Pole,Ddx31,2410016O06Rik,Rbm14,Cacna1c,2610027L16Rik,Gtse1,Tpbg,2810408A11Rik
**mmu-miR-425***	26(1239)	0,0345683	1110012J17Rik,Pcm1,Marcksl1,Zfp52,Ppih,Tmem176b,Hspd1,Impdh2,Junb,Psrc1,Cbx1,Wipf1,Cdca4,Gli2,Dbf4,Cct3,Mphosph10,Nudc,Prmt5,Syne2,Slc43a3,Rfc2,Cenpk,Sf3a3,Phc2,Myct1
**mmu-miR-542-5p**	32(1239)	0,0370743	1110012J17Rik,Rcc2,Ctsw,Kif4,Ptpre,Fkbp5,Ank3,Nuf2,Enpp1,Slc25a5,Micall2,Sbk1,Wipf1,Zfp54,Espl1,Cdc7,Top2a,Dbf4,Rsf1,Rbmx2,Trp53bp2,Mphosph10,Apitd1,Sertad1,Nsmce4a,Stmn1,Cenpk,Psmd5,Utp11l,Khdrbs1,Ift74,Ecd
**mmu-miR-326**	30(1239)	0,0411972	Gemin4,Prx,D19Bwg1357e,Mki67,Prpf19,Pdgfb,Ddx27,Ruvbl1,Nrp1,2510012J08Rik,Por,Dusp9,Klhdc2,Cand1,Cdca2,Gins1,Pif1,Gli2,Nme4,Nmral1,Ctsh,Fgd3,Sertad1,Cacna1c,Th1l,Mast4,Nsl1,Fancc,Eftud2,Slc4a7
**mmu-miR-712***	26(1239)	0,0441041	Notch1,Asph,Ktn1,Mum1,Wdr43,Ppih,Pank1,Nipsnap1,Mapk8,Riok2,Ruvbl1,Vrk3,Dhx36,Usp10,Exo1,Mpzl1,H2-K1,Ppp1cc,Rpl12,Nmral1,Smchd1,Eif4enif1,Rbm14,Ssrp1,Trub1,2610027L16Rik
**mmu-miR-203***	24(1239)	0,0444251	Snx5,2810046L04Rik,Ncaph,Lrrc45,Mybbp1a,Hmmr,Cep192,Ppid,Luc7l,Dbr1,Cenph,Rpl3,B230120H23Rik,Snrpa1,Idh2,Rsbn1,Ewsr1,Tk1,Pole,Igsf3,4933427D14Rik,Dus3l,Cdc40,Pola2
