# Supplementary material for: Reversible, interrelated mRNA and miRNA expression patterns in the transcriptome of Rasless fibroblasts: functional and mechanistic implications
Source: BMC Genomics. 2013 Oct 25;14:731. doi: 10.1186/1471-2164-14-731 (PMC4007593; doi:10.1186/1471-2164-14-731)
Supplement: Additional file 3: Table S3 — Functional annotation of the upregulated differentially expressed genes of Rasless MEFs. The GeneCodis functional annotation tool was used on the list of upregulated genes included in Additional file 1: Table S1. Statistical associations of particular gene subsets to specific Gene Ontology (GO) functional categories designated as Biological Processes (section S3-BP), KEGG signaling pathways (section S3-KEGG), transcription factors (section S3-TF) and miRNAs prediction (section S3-miRNAs) are presented in this table. [file 1471-2164-14-731-S3.pdf]

Table S3. **Functional annotation of the upregulated, differentially expressed genes of Rasless MEFs.** The GeneCodis (Gene Annotation Co-occurrence Discovery) functional annotation tool (http://genecodis.dacya.ucm.es) was used to identify statistically significant functional associations linking particular gene subsets contained within the list of induced loci occurring in Rasless MEFs (Table S1, Additional file 1, FDR=0.01) to specific cellular functionalities, including particular Biological Processes (section S3-BP), Signalling Pathways (Section S3-KEGG), Transcriptional Factors (section S3-TF) or miRNAs (section S3-miRNA).

The column labelled "*Functional Category*" identifies the specific functional GO terms recognized in each case for the corresponding groups of loci listed under the column labelled "*Genes*". The column labelled "*Number of Genes*" indicates the specific number of genes linked to the indicated functionality, out of the total number (in parenthesis) of genes recognized by GeneCodis in the list of induced genes of Rasless cells. The column labeled "*Corrected p-value*" refers to the statistical significance of the functional associations identified, and contains p-values calculated cases using the Hypergeometric Distribution and subsequently corrected by implementing the False Discovery Rate method of Benjamini and Hochberg [113].

In section S3-BP (pages 2-6), the column "*Functional category*" assigns common colors to groups of related GO numbers and descriptions falling under the same, high level, general functional categories (Metabolite processing, Signaling, Cell death, Transport, etc). In section S3-KEGG (pages 7-8), the column "*KEGG Pathway*" identifies KEGG number and denomination of signalling pathways potentially affected by the corresponding group of induced loci listed in each case. In section S3-TF (pages 9-15), the "*Transcription Factors*" column identifies specific transcription factors that may account for induction of the corresponding groups of loci listed in each chase. Finally, in section S3-miRNA (pages 16-40), the *"miRNA name*" column identifies specific mouse miRNAs that, according to current literature and databases, may contribute to the patterns of mRNA overexpression described for Rasless cells in Table S1.

Table S3-BP. Functional annotation to GO BIOLOGICAL PROCESSES of the induced, overexpressed genes of Rasless MEFs.

Functional category	*Number of genes*	*Corrected p-value*	*Genes induced in Rasless cells (from Table S1)*
TRANSPORT (METABOLITES, IONS AND VESICLES)			
GO:0006810: transport	119(776)	2,42E-29	Eif5a2,Crat,Ap3s1,Copg,Slc2a13,Slc6a7,Snx18,Ubl4,Crot,Trpc7,Slc39a3,Slc22a18,Rab3d,Gabarapl2,Kcnc2,Rab38,Glrb,Rab11fip5,Atp6v0e,Slc16a9,Napb,Kcnn1,Abcb6,Slc6a6,Stam2,Hdlbp,Trpv4,Ndufb2,Atp6v1e1,Vps39,Trappc6a,Dynlrb1,Slc35a2,F630110N24Rik,Copz2,Slc7a7,Abcd1,Tmco3,Qk,Slc31a2,Slc44a1,Stx8,AI317395,Ap3m2,Ero1l,Cacna1g,Myo6,Txnrd3,Atp2a3,Tpcn1,Ccdc64,Trappc2l,Ndel1,Nipa1,Tcn2,Ndufa10,Kpna1,Stard6,Slc38a7,Slc9a6,Slc35b1,Slc22a23,Grik2,Ergic3,Sec22c,Fabp3,Slc6a8,Slc35c2,Exoc3,Atg4a,2310046K01Rik,Vdac1,Slc22a4,S100a13,Snx10,Itpr2,Vps25,Dynlt3,Atg7,Rab2b,Dopey2,Pltp,Necap2,Slc26a11,Atp6v0b,Dirc2,Gabarap,Slc39a14,Tmed8,Slco3a1,Cyb5,Rab7l1,Slc1a4,Atp5s,Uqcrq,Slc25a39,Slc2a6,Vamp3,Snx32,Vps24,Chmp4c,Cog6,Clcn7,Cbara1,Tom1l2,Slc25a35,Slc41a2,Atp5e,Scn1b,Atp6v0a1,Tmem38a,Stxbp1,Lrsam1,Slc2a8,Yif1a,Vps29,Cacna1b,Fads3,Rab22a
GO:0015031: protein transport	41(776)	6,79E-11	Eif5a2, Ap3s1, Copg, Rab43, Snx18, Rab3d, Gabarapl2, Rab38, Rab11fip5, Rab40b, Napb, Stam2, Vps39, Copz2, Ap3m2, Myo6, Kpna1, Sec22c, Psen2, Exoc3, Atg4a, S100a13, Snx10, Vps25, Atg7, Rab2b, Dopey2, Necap2, Gabarap, Rab7l1, Vamp3, Snx32, Vps24, Chmp4c, Cog6, Tom1l2, Stxbp1, Lrsam1, Yif1a, Vps29, Rab22a

Functional category	*Number of genes*	*Corrected p-value*	*Genes induced in Rasless cells (from Table S1)*
**GO:0006811: ion transport**	39(776)	2,64E-08	Trpc7, Slc39a3, Slc22a18, Kcnc2, Glrb, Atp6v0e, Kcnn1, Trpv4, Atp6v1e1, Tmco3, Slc31a2, AI317395, Cacna1g, Atp2a3, Tpcn1, Nipa1, Tcn2, Slc38a7, Slc9a6, Slc22a23, Grik2, Slc6a8, Vdac1, Slc22a4, Itpr2, Wnk4, Slc26a11, Atp6v0b, Slc39a14, Slco3a1, Atp5s, Clcn7, Cbara1, Slc41a2, Atp5e, Scn1b, Atp6v0a1, Tmem38a, Cacna1b
**GO:0055085: transmembrane transport**	28(776)	4,62E-05	Eif5a2,Slc2a13,Trpc7,Slc39a3,Slc22a18,Kcnc2,Slc16a9,Slc6a6,Trpv4,F630110N24Rik,Slc7a7,Tmco3,Slc46a3,Slc44a1,AI317395,Tpcn1,Slc9a6,Slc35b1,Slc22a23,2310046K01Rik,Vdac1,Slc22a4,Itpr2,Slc26a11,Slc39a14,Clcn7,Slc2a8,Cacna1b
**GO:0016192: vesicle-mediated transport**	16(776)	0,000170089	Ap3s1,Copg,Napb,Trappc6a,Copz2,Stx8,Ap3m2,Trappc2l,Ergic3,Sec22c,Rab2b,Vamp3,Stx2,Stx7,Stxbp1,Yif1a
**GO:0006886: intracellular protein transport**	13(776)	0,00507565	Ap3s1,Copg,Ehd1,Napb,Stam2,Ap3m2,Kpna1,Grik2,Cdk5,Apba1,Stx2,Tom1l2,Stx7
**GO:0008333: endosome to lysosome transport**	5(776)	0,00696839	Stx8,Trak1,Mtm1,Vps24,Stx7
**GO:0006897: endocytosis**	10(776)	0,0146921	Mrc2,Snx18,Ehd1,Myo6,Ehd2,Necap2,Lrp4,App,Npc1,Rab22a
**GO:0045047: protein targeting to ER**	2(776)	0,0185842	Srp14,Srp54a
**GO:0032456: endocytic recycling**	3(776)	0,0190603	Ehd1,Ehd3,Ehd2
**GO:0008643: carbohydrate transport**	5(776)	0,0253772	Slc35a2,AI317395,Slc35b1,Slc2a6,Slc2a8
**GO:0006816: calcium ion transport**	9(776)	0,0303683	Trpc7,Trpv4,Cacna1g,Atp2a3,Tpcn1,Psen2,Itpr2,Cbara1,Cacna1b
**GO:0042308: negative regulation of protein import into nucleus**	2(776)	0,0323936	Pkia,Mdfic
**GO:0007340: acrosome reaction**	3(776)	0,0390576	Glrb,Trim36,Stx2
**GO:0015991: ATP hydrolysis coupled proton transport**	4(776)	0,0423888	Atp6v0e,Atp6v1e1,Atp6v0b,Atp6v0a1
**GO:0006887: exocytosis**	6(776)	0,0486208	Rab3d,Snapin,Scrn1,Cdk5,Exoc3,Stxbp1
**METABOLITE PROCESSING (PROTEIN, CARBOHYDRATE AND LIPID PROCESSING)**			
**GO:0008152: metabolic process**	55(776)	3,06E-08	Ugp2,Hadha,Fuca2,Crot,Dhrs3,Fahd2a,Papss2,Smpdl3a,Ctbs,Hexa,Aldh7a1,Adamts5,Prss36,Neu1,Atp9b,Nfs1,Acox1,Dusp3,Pld1,Trpt1,Lpcat2,Dhrs1,Camk1,Hexb,Ube2h,Atp2a3,Gstm2,Galns,Hsd3b3,Impdh1,Aldh6a1,Man2b1,Entpd5,Ppt2,Idua,Asah1,Aph1b,Rdh12,Naga,Atg7,Pde6d,Hgsnat,Srr,Adamts2,Ctsl,Galc,Glo1,Gstp1,Tyrp1,Acaa1a,Hadhb,Uchl1,Aph1a,Atp10d,Aldh3a2
**GO:0055114: oxidation-reduction process**	37(776)	5,75E-05	Sqrdl,Maob,Cat,Hadha,Dhrs3,Gpx4,Cyp27a1,Aldh7a1,Cyp4v3,Loxl1,Acox1,Maoa,Dhrs1,Alkbh6,Ero1l,Sepw1,Txnrd3,Suox,Gstk1,Hsd3b3,Impdh1,Aldh6a1,P4ha2,Rdh12,Srd5a3,Pdia5,Ypel5,Pcbd2,Cyb5,Bcmo1,Htatip2,Tyrp1,Cox6a2,Cyb5r3,Cox6b1,Fads3,Aldh3a2
**GO:0006749: glutathione metabolic process**	7(776)	0,000420833	Gpx4,Txnrd3,Gstm2,Gstk1,Glo1,Gstp1,Oplah
**GO:0033540: fatty acid beta-oxidation using acyl-CoA oxidase**	4(776)	0,000750702	Crat,Acox1,Abcd1,Acaa1a

Functional category	*Number of genes*	*Corrected p-value*	*Genes induced in Rasless cells (from Table S1)*
**GO:0006629: lipid metabolic process**	17(776)	0,000904153	Crat,Iah1,Plb1,Hadha,Samd8,Crot,Insig2,Hdlbp,Acox1,Asah1,Srd5a3,Plcb1,Abhd5,Acaa1a,Daglb,Hadhb,Fads3
**GO:0008654: phospholipid biosynthetic process**	8(776)	0,00106116	Pld1,Lpcat2,Hexb,Pcyt1a,Sh3glb1,Abhd5,Ptdss1,Chpt1
**GO:0006184: GTP catabolic process**	12(776)	0,00182145	Rras,Rhoq,Rragc,Rasl12,Arl2,Srp54a,Tuba8,Mras,Rhoj,Rit1,Tubb2a,Rab22a
**GO:0007220: Notch receptor processing**	3(776)	0,00693993	Psen2,Dner,Aph1a
**GO:0042326: negative regulation of phosphorylation**	5(776)	0,0103424	Bmp4,Pkia,Cdkn1a,Cdkn2b,1700021C14Rik
**GO:0006654: phosphatidic acid biosynthetic process**	3(776)	0,0106918	Pld1,Sh3glb1,Abhd5
**GO:0044255: cellular lipid metabolic process**	5(776)	0,0111373	Crat,Crot,Acox1,Abcd1,Acaa1a
**GO:0006631: fatty acid metabolic process**	8(776)	0,0194895	Crat,Hadha,Crot,Acox1,Abhd5,Cyb5,Acaa1a,Hadhb
**GO:0031293: membrane protein intracellular domain proteolysis**	3(776)	0,0323233	Aph1b,Psen2,Aph1a
**GO:0006663: platelet activating factor biosynthetic process**	2(776)	0,0323936	Lpcat2,Chpt1
**GO:0009313: oligosaccharide catabolic process**	2(776)	0,0323936	Ctbs,Hexb
**GO:0006689: ganglioside catabolic process**	2(776)	0,0323936	Hexa,Hexb
**GO:0051084: 'de novo' posttranslational protein folding**	2(776)	0,0323936	Entpd5,Sh3glb1
**GO:0005975: carbohydrate metabolic process**	11(776)	0,0324897	Fuca2,Ctbs,Hexa,Hexb,Man2b1,Idua,Naga,Galc,Glo1,Chid1,Chst12
**GO:0010951: negative regulation of endopeptidase activity**	7(776)	0,0328439	Serpinb6c,Renbp,Timp2,Serpinb9c,Serpinb9b,Serpinb1a,Reck
**GO:0006508: proteolysis**	19(776)	0,0343208	Prss29,Adamts5,Prss36,Scrn1,Bace1,Klk1b4,Ctso,Qpct,Nrip3,Htra3,Capn5,Atg4a,Ctsb,2010111I01Rik,Adamts2,Ctsl,Cpa6,Tpp1,Immp1l
**GO:0016310: phosphorylation**	27(776)	0,0350662	Pank2,Camk4,Papss2,Stk10,Stk39,Prkab2,Amhr2,Gsk3b,Ephb3,Mapk4,Guk1,Riok3,Cdk5,Ulk2,Akap4,Tollip,Grk4,Wnk4,Pkia,Ikbkg,Hck,Cdkn1a,Ckm,Cdkn2b,Camkk1,Dmpk,Nagk
**GO:0043086: negative regulation of catalytic activity**	7(776)	0,0354611	Anxa3,Timp2,Anxa1,Pde6d,Pkia,Cdkn1a,1700021C14Rik
**GO:0042787: protein ubiquitination involved in ubiquitin-dependent protein catabolic process**	5(776)	0,036621	Rnf144b,Lnx1,Wwp2,Hectd3,Os9
**GO:0043085: positive regulation of catalytic activity**	6(776)	0,0419269	Slc37a4,Adc,Timp2,Aph1b,Psen2,Aph1a
**GO:0006656: phosphatidylcholine biosynthetic process**	3(776)	0,0456366	Fabp3,Pcyt1a,Chpt1

Functional category	*Number of genes*	*Corrected p-value*	*Genes induced in Rasless cells (from Table S1)*
**GO:0006044: N-acetylglucosamine metabolic process**	3(776)	0,0456366	Renbp,Hexb,Nagk
**GO:0043433: negative regulation of sequence-specific DNA binding transcription factor activity**	5(776)	0,0466305	Thra,Prnp,Wwp2,Hr,Fzd6
**GO:0006657: CDP-choline pathway**	2(776)	0,0476976	Pcyt1a,Chpt1
**GO:0008612: peptidyl-lysine modification to hypusine**	2(776)	0,0476976	Eif5a2,Dhps
**GO:0071569: protein ufmylation**	2(776)	0,0476976	Ufc1,Ufm1
**GO:0019348: dolichol metabolic process**	2(776)	0,0476976	Srd5a3,Dpm2
**SIGNALING**			
**GO:0007264: small GTPase mediated signal transduction**	19(776)	1,73E-05	Rab43,Rab3d,Rab38,Rras,Rhoq,Rab40b,Klk1b4,Rasl12,Arl2,Nkiras1,Nkiras2,Rab2b,Rasl10b,Rab7l1,Arhgap1,Mras,Rhoj,Rit1,Rab22a
**GO:0043407: negative regulation of MAP kinase activity**	5(776)	0,0111373	Dab2ip,Gsk3b,Bmp4,Gstp1,Uchl1
**GO:0007219: Notch signaling pathway**	6(776)	0,0371148	Dtx2,Aph1b,Psen2,Dner,App,Aph1a
**GO:0007176: regulation of epidermal growth factor-activated receptor activity**	2(776)	0,0476976	Psen2,App
**CELL COMUNICATION**			
**GO:0007268: synaptic transmission**	12(776)	0,000187957	Snap91,Glrb,Snapin,Clstn1,Cacna1g,Myo6,Npy6r,Dlg2,Rapsn,Grik2,Vdac1,Apba1
**GO:0042552: myelination**	5(776)	0,0206488	Pmp22,Mal,Hexa,Qk,Hexb
**GO:0010807: regulation of synaptic vesicle priming**	2(776)	0,0323936	Napb,Stxbp1
**DEVELOPMENTAL PROCESS**			
**GO:0016322: neuron remodeling**	4(776)	0,000750702	Farp2,Cspg4,Ntn4,App
**GO:0031175: neuron projection development**	8(776)	0,00738065	Lamb2,Ccdc64,Ndel1,L1cam,Cdk5,Atg7,App,Ptprm
**GO:0016044: cellular membrane organization**	9(776)	0,0114223	Copg,Sgca,Stam2,Sgcb,Lpcat2,Vps25,Atg7,Vps24,Chmp4c
**GO:0007409: axonogenesis**	8(776)	0,0178423	Snap91,Gsk3b,Cdk5,Ulk2,App,Fzd8,Fzd6,Uchl1
**GO:0008360: regulation of cell shape**	7(776)	0,018157	Rhoq,Fn1,Cdc42ep2,S100a13,Tbccd1,Rhoj,Shroom3
**GO:0051124: synaptic growth at neuromuscular junction**	2(776)	0,0185842	Lrp4,App

Functional category	*Number of genes*	*Corrected p-value*	*Genes induced in Rasless cells (from Table S1)*
**GO:0043113: receptor clustering**	4(776)	0,0192507	Dlg2,Grik2,Cdk5,Lrp4
**GO:0032331: negative regulation of chondrocyte differentiation**	3(776)	0,0323233	Wnt9a,Bmp4,Ltbp3
**GO:0048642: negative regulation of skeletal muscle tissue development**	2(776)	0,0323936	Usp2,Tsc22d3
**GO:0001501: skeletal system development**	7(776)	0,0366988	Acp2,Hexa,Tgfb2,Hexb,Bmp4,Chrd,Ltbp3
**GO:0030198: extracellular matrix organization**	7(776)	0,042568	Fbln5,Fn1,Tgfb2,Ccdc80,App,Adamtsl4,Reck
**GO:0007412: axon target recognition**	2(776)	0,0476976	Stxbp1,Uchl1
**GO:0050873: brown fat cell differentiation**	4(776)	0,0481316	Rarres2,Ero1l,Aldh6a1,Nudt7
**CELL DEATH**			
**GO:0006916: anti-apoptosis**	13(776)	0,00131366	Birc7,Fn1,Gsk3b,Prnp,Psen2,Hbxip,Vnn1,Triap1,Sh3glb1,Cryab,Tsc22d3,Glo1,Mcl1
**GO:0043066: negative regulation of apoptotic process**	17(776)	0,00719886	Cat,Smad6,Tgfb2,Gsk3b,Prnp,Bmp4,Nme2,Anxa1,Adar,Psen2,Atg7,Cryab,Gstp1,Cdkn1a,Gas6,Igbp1,Mcl1
**GO:0043154: negative regulation of cysteine-type endopeptidase activity involved in apoptotic process**	6(776)	0,020777	Wnt9a,Smad6,Hbxip,Triap1,Cryab,Igbp1
**CYTOSKELETON-REGULATED PROCESSES**			
**GO:0007018: microtubule-based movement**	7(776)	0,0210754	Kif27,Kif3b,Dynlrb1,Kif1c,Tuba8,Kif3a,Tubb2a
**CELL CYCLE (MITOTIC AND MEIOTIC DIVISION)**			
**GO:0000086: G2/M transition of mitotic cell cycle**	4(776)	0,021455	Ccny,Plcb1,Cdkn1a,Cdkn2b
**GO:0016049: cell growth**	5(776)	0,0226594	Adra1b,Atp6v0e,Tgfb2,Entpd5,Nupr1
**OTHERS**			
**GO:0071363: cellular response to growth factor stimulus**	4(776)	0,032519	Ppp2r5b,Bmp4,Sparc,Gas6

Table S3-KEGG. Functional annotation to KEGG PATHWAYS of the induced, overexpressed genes of Rasless MEFs.

KEGG Pathway	*Number of genes*	*Corrected p-value*	*Genes induced in Rasless cells (from Table S1)*
**(KEGG) 04142 :Lysosome**	24(776)	1,74E-14	Hexa, Man2b1, Idua, Naga, Asah1, Npc1, Ctsl, Npc2, Acp2, Neu1, Hexb, Ppt2, Atp6v0b, Galc, Hgsnat, Scarb2, Ap3s1, Ap3m2, Atp6v0a1, Ctsb, Tpp1, Galns, Gnptg, Ctso
**(KEGG) 05010 :Alzheimer's disease**	17(776)	9,05E-06	Atp5e, Uqcrq, Cox6b1, Ndufb2, Aph1a, Gsk3b, Atp2a3, Cdk5, Psen2, Aph1b, Ndufb4, App, Bace1, Ndufa10, Itpr2, Plcb1, Cox6a2
**(KEGG) 04140 :Regulation of autophagy**	7(776)	0,000311192	Ulk2, Gabarapl2, Gabarap, Atg7, Atg12, Atg4a, Gabarapl1
**(KEGG) 04146 :Peroxisome**	10(776)	0,00036405	Acaa1a, Acox1, Gstk1, Cat, Crot, Pxmp4, Crat, Mpv17, Mlycd, Abcd1
**(KEGG) 00511 :Other glycan degradation**	5(776)	0,000708504	Hexa, Man2b1, Neu1, Hexb, Fuca2
**(KEGG) 00640 :Propanoate metabolism**	6(776)	0,00115798	Aldh6a1, Hadha, Mcee, Aldh3a2, Aldh7a1, Mlycd
**(KEGG) 00531 :Glycosaminoglycan degradation**	5(776)	0,00135779	Hexa, Idua, Hexb, Hgsnat, Galns
**(KEGG) 00280 :Valine, leucine and isoleucine degradation**	7(776)	0,00136813	Acaa1a, Aldh6a1, Hadhb, Hadha, Mcee, Aldh3a2, Aldh7a1
**(KEGG) 04144 :Endocytosis**	15(776)	0,00142523	Ehd1, Iqsec2, Ehd3, Rab11fip5, Tgfb2, Smad6, Stam2, Rab22a, Grk4, Vps25, Vps24, Pld1, Ehd2, Sh3glb1, Chmp4c
**(KEGG) 00190 :Oxidative phosphorylation**	11(776)	0,00187941	Atp5e, Uqcrq, Cox6b1, Ndufb2, Atp6v1e1, Atp6v0b, Ndufb4, Atp6v0a1, Ndufa10, Atp6v0e, Cox6a2
**(KEGG) 00380 :Tryptophan metabolism**	6(776)	0,0047997	Hadha, Maoa, Aldh3a2, Cat, Aldh7a1, Maob
**(KEGG) 00071 :Fatty acid metabolism**	6(776)	0,00634405	Acaa1a, Acox1, Hadhb, Hadha, Aldh3a2, Aldh7a1
**(KEGG) 00062 :Fatty acid elongation in mitochondria**	3(776)	0,00638668	Hadhb, Hadha, Ppt2
**(KEGG) 00520 :Amino sugar and nucleotide sugar metabolism**	6(776)	0,00683403	Hexa, Nagk, Cyb5r3, Renbp, Hexb, Ugp2
**(KEGG) 04145 :Phagosome**	11(776)	0,00767324	Stx7, Ctsl, Tuba8, Vamp3, Atp6v1e1, Atp6v0b, Atp6v0a1, Mrc2, Atp6v0e, Tubb2a, Tlr6
**(KEGG) 00260 :Glycine, serine and threonine metabolism**	5(776)	0,00789637	Srr, Maoa, Alas1, Aldh7a1, Maob
**(KEGG) 05146 :Amoebiasis**	9(776)	0,00813759	Serpinb1a, Tgfb2, Lama2, Serpinb6c, Serpinb9c, Serpinb9b, Lamb2, Plcb1, Fn1
**(KEGG) 04916 :Melanogenesis**	8(776)	0,0085614	Fzd8, Tyrp1, Creb3, Gsk3b, Adcy9, Wnt9a, Plcb1, Fzd6
**(KEGG) 05217 :Basal cell carcinoma**	6(776)	0,00891424	Fzd8, Bmp4, Gsk3b, Wnt9a, Hhip, Fzd6
**(KEGG) 00330 :Arginine and proline metabolism**	6(776)	0,00898224	Maoa, Aldh3a2, Adc, Aldh7a1, Maob, Ckm
**(KEGG) 00920 :Sulfur metabolism**	3(776)	0,00907299	Suox, Papss2, Chst12
**(KEGG) 00564 :Glycerophospholipid metabolism**	7(776)	0,0112858	Lpcat2, Plb1, Chpt1, Pld1, Ptdss1, Pcyt1a, Dgkg

KEGG Pathway	*Number of genes*	*Corrected p-value*	*Genes induced in Rasless cells (from Table S1)*
**(KEGG) 05016 :Huntington's disease**	11(776)	0,0115358	Atp5e, Uqcrq, Cox6b1, Ndufb2, Creb3, Vdac1, Ndufb4, Dnalc1, Ndufa10, Plcb1, Cox6a2
**(KEGG) 00620 :Pyruvate metabolism**	5(776)	0,0117742	Glo1, Acyp2, Acyp1, Aldh3a2, Aldh7a1
**(KEGG) 04360 :Axon guidance**	9(776)	0,0118808	L1cam, Arhgef12, Sema3f, Ephb3, Sema3c, Gsk3b, Cdk5, Sema3b, Ntn4
**(KEGG) 05012 :Parkinson's disease**	9(776)	0,0118808	Atp5e, Uqcrq, Cox6b1, Uchl1, Ndufb2, Vdac1, Ndufb4, Ndufa10, Cox6a2
**(KEGG) 05410 :Hypertrophic cardiomyopathy (HCM)**	7(776)	0,0119038	Itga9, Tgfb2, Lmna, Lama2, Sgcb, Sgca, Prkab2
**(KEGG) 00410 :beta-Alanine metabolism**	4(776)	0,0159053	Hadha, Aldh3a2, Aldh7a1, Mlycd
**(KEGG) 00340 :Histidine metabolism**	4(776)	0,0159053	Maoa, Aldh3a2, Aldh7a1, Maob
**(KEGG) 05414 :Dilated cardiomyopathy**	7(776)	0,0159599	Itga9, Tgfb2, Lmna, Adcy9, Lama2, Sgcb, Sgca
**(KEGG) 00604 :Glycosphingolipid biosynthesis - ganglio series**	3(776)	0,0188818	Hexa, St3gal5, Hexb
**(KEGG) 00603 :Glycosphingolipid biosynthesis - globo series**	3(776)	0,0188818	Hexa, Naga, Hexb
**(KEGG) 00565 :Ether lipid metabolism**	4(776)	0,0286588	Lpcat2, Plb1, Chpt1, Pld1
**(KEGG) 04130 :SNARE interactions in vesicular transport**	4(776)	0,0308896	Stx7, Vamp3, Stx2, Stx8
**(KEGG) 05323 :Rheumatoid arthritis**	6(776)	0,0360445	Ctsl, Tgfb2, Atp6v1e1, Atp6v0b, Atp6v0a1, Atp6v0e
**(KEGG) 00860 :Porphyrin and chlorophyll metabolism**	4(776)	0,0406406	Mmab, Alas1, Ugt2b36, Fech
**(KEGG) 05200 :Pathways in cancer**	14(776)	0,0407771	Fzd8, Bmp4, Cdkn1a, Tgfb2, Arnt, Gsk3b, Lama2, Wnt9a, Ikbkg, Hhip, Lamb2, Cdkn2b, Fzd6, Fn1
**(KEGG) 04350 :TGF-beta signaling pathway**	6(776)	0,0416433	Chrd, Bmp4, Tgfb2, Smad6, Amhr2, Cdkn2b
**(KEGG) 04540 :Gap junction**	6(776)	0,042932	Pdgfc, Tuba8, Adcy9, Itpr2, Plcb1, Tubb2a
**(KEGG) 00982 :Drug metabolism - cytochrome P450**	6(776)	0,0441799	Gstp1, Gstk1, Maoa, Gstm2, Ugt2b36, Maob
**(KEGG) 04010 :MAPK signaling pathway**	12(776)	0,0453099	Dusp8, Dusp22, Cacna1b, Tgfb2, Mras, Gadd45b, Ikbkg, Cacna1g, Jund, Rras, Map3k8, Dusp3
**(KEGG) 05215 :Prostate cancer**	6(776)	0,0467559	Pdgfc, Gstp1, Cdkn1a, Creb3, Gsk3b, Ikbkg
**(KEGG) 04020 :Calcium signaling pathway**	9(776)	0,0480743	Adra1b, Cacna1b, Atp2a3, Adcy9, Camk4, Cacna1g, Vdac1, Itpr2, Plcb1
**(KEGG) 03060 :Protein export**	3(776)	0,048273	Immp1l, Srp14, Srp54a

Table S3-TF. Functional annotation to "TRANSCRIPTION FACTORS" of the induced, overexpressed genes of Rasless MEFs.

Transcription Factor	*Number of genes*	*Corrected p-value*	*Genes induced in Rasless cells (from Table S1)*
V$SP1_Q6	109(776)	2,36E-16	Glo1, Hr, Cspg4, Npdc1, Zbtb4, Fzd8, Tbcd, Gstp1, Rab4b, Kcnn1, Srr, Klf2, Man2b1, Vps39, Gabarapl2, Bmp4, Hdlbp, Ttc15, Tom1, Hoxa1, Gabarap, Sirt2, Arhgap1, Cryab, Cdkn1a, Cacna1b, Habp4, Adamts2, Mtmr3, Samd8, Npc2, Smad6, Ephb3, Creb3, Apoa1bp, Ugp2, Camkk1, Gsk3b, Dnajc4, Gpx4, Tcta, Kif27, Atp6v0b, Sqrdl, Fbn2, Reck, Rragc, Rab22a, Maoa, Vps29, Slc39a3, Dbp, Nkiras2, Capn5, Impdh1, Nfe2l1, Mocs2, Ndel1, Lrsam1, Myadm, Srp14, Kif1c, Elmo2, Klf15, Ensa, Rab2b, Ing4, Cacna1g, Vps24, Dleu2, Stxbp1, Ap3s1, Mrps14, Sema3b, Amhr2, Crot, Mtss1, Arvcf, Stmn3, Jund, Aldh7a1, Slc2a6, Rras, Maob, Gpc4, Adamts5, Itpr2, Kif3a, Rab3d, Plcb1, Fbxl2, Pltp, Galns, Thra, Dusp3, Slc9a6, Gabarapl1, Tpcn1, Lrpap1, Abcd1, Clstn1, Abcb6, Cry2, Rab40b, Prkab2, Slco3a1, Pde6d, Cdc42ep2, Mrgprf
V$PAX4_03	60(776)	5,89E-11	Qpct, Stk10, Kremen1, Hr, Arhgef12, Dusp8, Pmp22, Trpc7, Ppp2r5b, Ank2, Man2b1, Chrd, Bmp4, S100a16, Rarres2, Apba1, Gabarap, Nrip3, Cdkn1a, Slc39a14, Smad6, Rbms2, Kctd9, Creb3, Rora, Add3, Dlg2, S100a1, Tcta, Pkia, Dab2ip, Mtus1, Nkiras2, Capn5, Slc37a4, Nfe2l1, Wnt9a, Gadd45b, Kif1c, Cacna1g, Ap3s1, Lamb2, Sema3b, Mtss1, Tob1, Gpc4, Adamts5, Rab3d, Pbxip1, Plcb1, Sparc, Usp2, Thra, Slc9a6, Prelp, Zdhhc8, Gpr133, Dmpk, Ntn4, Abcd1
V$E12_Q6	83(776)	4,23E-10	Hr, Arhgef12, Slc6a7, Zbtb4, Pmp22, Rab43, Fbxl20, Ank2, Ltbp3, Kcnc2, Ufc1, Agl, Nagk, Bmp4, Hdlbp, S100a16, Dhrs3, Adra1b, Hoxa1, Apba1, Gabarap, Nrip3, Repin1, Ssbp2, Ehd1, Fabp3, Ndrg4, Slc39a14, Sema3f, Cobl, Galnt2, Camkk1, Ppt2, Add3, Dlg2, Snx10, Atp6v0b, Pkia, Rragc, Rab22a, Mtus1, Dbp, Slc37a4, Wnt9a, Fuca2, Gadd45b, Psen2, Dtx2, Kif13b, Rasl10b, Serf2, C1qtnf1, Kif1c, Klf15, Cacna1g, Stxbp1, Hhip, Sema3b, Wwp2, Mtss1, Arvcf, App, Bace1, Itpr2, Pbxip1, Plcb1, Crat, Abtb2, Ckm, Usp2, Thra, Dusp3, Rhoq, Prelp, Cox6a2, Dmpk, Mpv17, Ntn4, Rasl12, Cdh7, Dgkg, Slco3a1, Mrgprf
V$AP4_Q5	59(776)	1,94E-09	L1cam, Pmp22, Ank2, Man2b1, Loxl1, Chrd, Lhfpl2, Ankrd12, Dhrs3, Hist1h3d, Apba1, Cryab, Mapk4, Rnf13, Renbp, Adamts2, Tuba8, Tgfb2, Arnt, Vamp3, Rapsn, Cpa6, Gsk3b, Dlg2, Esrrb, Napb, Cdk5, Lrba, Scrn1, Impdh1, Wnt9a, Rasl10b, Myadm, Serf2, C1qtnf1, Ube2h, Cacna1g, Dleu2, Lamb2, Mtss1, App, Map3k8, Ufm1, Tob1, Pbxip1, Abtb2, Ckm, Rit1, Slc1a4, Cox6a2, Dmpk, Lrch1, Mlycd, Rabac1, Slc35c2, Tollip, Rasl12, Tpcn1, Mrgprf
V$NF1_Q6	41(776)	3,37E-08	Wisp2, Hr, Arhgef12, Ppp2r5b, Loxl1, Slc35b1, Chrd, Ankrd12, Dhrs3, Jdp2, Cryab, Ssbp2, Fabp3, Abhd5, Rbms2, Camkk1, Sema3c, Rora, Lmna, Add3, Dlg2, Snx10, Atp2a3, Napb, Akap4, Kif13b, C1qtnf1, Ube2h, Tom1l2, Lancl1, Hhip, Ap3s1, Lamb2, Stac, Mtss1, Rras, Pbxip1, Plcb1, Rit1, Prelp, Abcb6
V$FOXO4_01	68(776)	3,97E-08	Dner, Kremen1, Satb1, Hr, Glrb, Dusp8, Rab43, Fbxl20, Kcnn1, Mfap5, Kcnc2, Ulk2, Agl, Gabarapl2, Bmp4, Zdhhc4, Dhrs3, Apba1, Gabarap, Ssbp2, Anxa1, Ehd1, Rnf13, Tgfb2, Smad6, Rbms2, Ndufb2, Ephb3, Rora, Dlg2, Nfs1, Pkia, Fbn2, Gpr39, Fstl1, Dbp, Atp10d, Dtx2, Gng5, Ube2h, Tom1l2, Ing4, Grik2, Hhip, Riok3, Atp6v0a1, Ezh1, App, Tob1, Pld1, Mrc2, Plcb1, Sh3glb1, Dirc2, Usp2, Thra, Rit1, Fn1, Rhoq, Ptprm, Gabarapl1, Ntn4, Clstn1, Znrf2, Cry2, Garnl3, Frmpd1, Mrgprf
V$MAZ_Q6	72(776)	1,50E-07	Satb1, Hr, Arhgef12, Slc6a7, Dusp8, Npdc1, Zbtb4, Fbxl20, Kcnc2, Gabarapl2, Chrd, Mkrn1, Bmp4, S100a16, Hoxa1, Arl2, Gabarap, Slamf9, Cdkn1a, Ssbp2, Tuba8, Tgfb2, Arnt, Selm, Rab38, Nkiras1, Galnt2, Ugp2, Rora, Lmna, Ppt2, Add3, Gsk3b, Dlg2, Dnajc4, Gpx4, Atp2a3, Tcta, Dab2ip, Mtus1, Dbp, Iscu, Impdh1, Gadd45b, Gdf15, Lrsam1, Myadm, Kif1c, Klf15, Ube2h, Ensa, Cacna1g, Ap3s1, Amhr2, Wwp2, Arvcf, Bace1, Rras, Map3k8, Tob1, Gpc4, Adamts5, Mrc2, Tbc1d14, Sparc, Usp2, Thra, Prelp, Dmpk, Clstn1, Dgkg, Cdc42ep2
V$AP1_C	45(776)	4,37E-07	Stk10, Cspg4, Pmp22, Dusp22, Gstp1, Ltbp3, Slc35b1, Fads3, Dhrs3, Suox, Nrip3, Cdkn1a, Cobl, Camkk1, Lmna, Cpa6, Snx10, Napb, Fstl1, Scrn1, Gadd45b, Dtx2, Lrsam1, Camk4, Sema3b, Atp6v0a1, Ecm1, App, Tob1, Rab3d, Pcsk6, Sparc, Usp2, Slc22a18, Rit1, Rhoq, Gabarapl1, Dmpk, Mpv17, Ntn4, Synpo, Abcd1, Abcb6, Lynx1, Mrgprf
V$ERR1_Q2	43(776)	8,54E-07	Dner, Satb1, Arhgef12, Dok4, Ltbp3, Kcnn1, Mfap5, Mal, Cdkn1a, Fabp3, Uchl1, Slc31a2, Sema3f, Ascc1, Creb3, Ugp2, Fbln5, Dlg2, Esrrb, Rab22a, Fstl1, Mtus1, Iscu, Nfe2l1, Slc2a13, Myadm, Nr2c1, Kif1c, Vdac1, Lamb2, App, Slc6a8, Abtb2, Usp2, Thra, Dusp3, Rhoq, Kif3b, Gabarapl1, Dscr3, Dmpk, Synpo, Slco3a1
V$PITX2_Q2	33(776)	2,30E-06	Hr, Pdgfc, Srr, Bmp4, Rpl17, Dhrs3, Hoxa1, Nrip3, Tyrp1, Tuba8, Arnt, Rbms2, Creb3, Rora, Ppt2, Add3, Gsk3b, Bbs5, Slc41a2, Slc35a2, Slc37a4, Impdh1, Gadd45b, Mtss1, Jund, App, Rras, Pbxip1, Ankrd29, Fzd6, Dusp3, Dmpk, Znrf2
V$MEIS1_01	34(776)	1,99E-05	Hr, Arhgef12, Cspg4, Ppp2r5b, Stard6, Ank2, Rhoj, Ankrd12, Dhrs3, Adra1b, Hoxa1, Apba1, Arhgap1, Anxa1, Mtmr3, Abhd5, Rora, Esrrb, Copz2, Elmo2, Ube2h, Ensa, Ap3s1, Riok3, Mtss1, Bace1, Pxmp4, Cdkn2b, P4ha2, Crat, Abtb2, Usp2, Thra, Mrgprf

Transcription Factor	*Number of genes*	*Corrected p-value*	*Genes induced in Rasless cells (from Table S1)*
V$NFAT_Q4_01	56(776)	3,42E-05	Ctbs, Arhgef12, Cspg4, Pmp22, Tbcd, Acyp2, Loxl1, Bmp4, Dhrs3, Hoxa1, Apba1, Gabarap, Jdp2, Repin1, Stx7, Cdkn1a, Ssbp2, Mapk4, Adamts2, Uchl1, Tgfb2, Abhd5, Rbms2, Rab38, Vamp3, Rora, Ppt2, Add3, Dlg2, Esrrb, Tcta, Fstl1, Slc35a2, Cables1, Gadd45b, Ube2h, Ensa, Stxbp1, Wwp2, Mtss1, Gdap1, Map3k8, Mrc2, Pcsk6, Pbxip1, Plcb1, Crat, Usp2, Rit1, Smpdl3a, Fn1, Prelp, Rasl12, Abcd1, Dgkg, Mrgprf
V$AP1_Q4_01	17(776)	3,42E-05	Ltbp3, Slc35b1, Dhrs3, Cdkn1a, Cobl, Camkk1, Snx10, Fstl1, Ecm1, Tob1, Rab3d, Usp2, Rit1, Mpv17, Ntn4, Synpo, Abcd1
V$LEF1_Q2	73(776)	4,07E-05	Stk10, Kremen1, Satb1, L1cam, Hr, Zbtb4, Pdgfc, Rab43, Aldh6a1, Ank2, Ltbp3, Slc35b1, Slc7a7, Mkrn1, Ankrd12, Gabarap, Jdp2, Nrip3, Ehd1, Sh3bgr, C1qtnf6, Uchl1, Tgfb2, Sema3f, Npc2, Smad6, Arnt, Ephb3, Rora, Add3, Gsk3b, Esrrb, Gstk1, Pkia, Dab2ip, Reck, Rab22a, Mtus1, Dbp, Nkiras2, Vnn1, Nfe2l1, Gdap2, Wnt9a, C1qtnf1, Elmo2, Ube2h, Ing4, Cacna1g, Anxa3, Grik2, Lamb2, Fech, Wwp2, Atp6v0a1, Stmn3, Jund, App, Bace1, Slc2a6, Slc16a9, Maob, Tob1, Gpc4, Adamts5, Kif3a, Usp2, Rit1, Rhoq, Trpv4, Gpr133, Abcd1, Pank2
V$BACH2_01	17(776)	4,17E-05	Fads3, Dhrs3, Cdkn1a, Camkk1, Lmna, Cpa6, Scrn1, Gadd45b, Dtx2, Ndel1, Rab3d, Rit1, Gabarapl1, Mpv17, Ntn4, Synpo, Abcd1
V$MTF1_Q4	16(776)	4,43E-05	Hr, Fbxl20, Chrd, Repin1, Dab2ip, Klf15, Ppp1r16a, Mtss1, Arvcf, Tob1, Gpc4, Mrc2, Kif3a, Ehd2, Abtb2, Dgkg
V$E4F1_Q6	28(776)	6,07E-05	Zbtb4, Ank2, Ufc1, Hoxa1, Arl2, Cdkn1a, Habp4, Tgfb2, Rbms2, Ndufb2, Maoa, Iscu, Atp10d, Ndel1, Nr2c1, Ube2h, Ensa, Ing4, Sema3b, Jund, Ndufa10, Mrc2, Fbxl2, Usp2, Thra, Hist1h4h, Ntn4, Cry2
V$SREBP1_01	23(776)	8,77E-05	Hexa, Arhgef12, Dusp8, Aldh6a1, Mmaa, S100a16, Tom1, Gabarap, Cbara1, Renbp, Acp2, Camkk1, Gsk3b, S100a1, Rragc, Rab22a, Nfe2l1, Fuca2, Cyp27a1, App, Dirc2, Dusp3, Tollip
V$OLF1_01	23(776)	9,09E-05	Hr, Pdgfc, Dusp22, Gas6, Fads3, Gabarapl2, Bmp4, Apba1, Repin1, Ephb3, Gsk3b, C1qtnf1, Rab2b, Sema3b, Mtss1, Itpr2, Ckm, Thra, Dusp3, Rhoq, Slc9a6, Dmpk, Frmpd1
V$AP1_01	16(776)	9,36E-05	Ppp2r5b, Gstp1, Dhrs3, Nrip3, Cdkn1a, Lmna, Cpa6, Snx10, Dtx2, Rab3d, Slc22a18, Rit1, Gabarapl1, Abcd1, Abcb6, Mrgprf
V$NFE2_01	18(776)	0,000136971	Gstp1, Dhrs3, Nrip3, Cdkn1a, Cobl, Camkk1, Cpa6, Snx10, Scrn1, Dtx2, Tob1, Rab3d, Slc22a18, Rit1, Gabarapl1, Synpo, Abcd1, Abcb6
V$FREAC2_01	33(776)	0,000162716	Hr, Npdc1, Pdgfc, Rab43, Ank2, Gabarapl2, Bmp4, Dhrs3, Hoxa1, Cdkn1a, Habp4, Asah1, C1qtnf6, Ephb3, Rora, Ppt2, Pkia, Dab2ip, Fstl1, Scrn1, Ube2h, Grik2, Hhip, Ezh1, Map3k8, Tob1, Pbxip1, Thra, Rhoq, Synpo, Clstn1, Pde6d, Mrgprf
V$HEB_Q6	15(776)	0,000173938	L1cam, Pmp22, Fbxl20, Dhrs3, Hist1h3d, Mapk4, Tgfb2, Dlg2, Nfe2l1, Ufm1, Ckm, Usp2, Slc1a4, Rasl12, Mrgprf
V$TCF11MAFG_01	20(776)	0,000178281	Satb1, Ero1l, Gstp1, Slc35b1, Hdlbp, Rnf13, Uchl1, Camkk1, Snx10, Atp2a3, Nkiras2, Dtx2, Grik2, Atp6v0a1, Tob1, Mrc2, Slc22a18, Gabarapl1, Pcyt1a, Abcb6
V$AP1_Q6	15(776)	0,000182228	Ltbp3, Dhrs3, Nrip3, Cdkn1a, Cobl, Camkk1, Lmna, Cpa6, Scrn1, Ecm1, Rab3d, Sparc, Rit1, Dmpk, Abcd1
V$TEF1_Q6	22(776)	0,000188792	Mfap5, Bmp4, Cryab, Ehd1, Slc31a2, Rapsn, Lmna, Add3, Dlg2, Fstl1, Myadm, Kif1c, Cacna1g, Lamb2, Mtss1, Jund, Rras, Pbxip1, Sparc, Usp2, Dmpk, Mrgprf
V$MEF2_02	29(776)	0,000266816	Stard6, Kcnn1, Myom2, Cryab, Cdkn1a, Vamp3, Lmna, Dlg2, Esrrb, Atp2a3, Lama2, Cat, Gadd45b, Timp2, Ndel1, Klf15, Sgcb, Cog6, Grik2, Lamb2, Stac, Gpc4, Ckm, Usp2, Rhoq, Camk1, Cox6a2, Dmpk, Slc35c2
V$ELK1_02	34(776)	0,000507657	Rab4b, Ufc1, Agl, Gabarapl2, Nagk, Mkrn1, Mtcp1, Acyp1, Sirt2, Arhgap1, Asb1, Rnf13, Galnt10, Samd8, Sema3f, Acp2, Tcta, Nfs1, Dab2ip, Mcl1, Mef2b, Nkiras2, Insig2, Srp14, Ing4, Bace1, Itgb1bp1, Rras, Ufm1, Rhoq, Dscr3, Pcyt1a, Mlycd, Slc35c2
V$ATF3_Q6	23(776)	0,000578038	Qpct, Zbtb4, Ppp2r5b, Rhoj, Adra1b, Cdkn1a, Ehd1, Sh3bgr, Ndufb2, Maoa, Ndel1, Ube2h, Ing4, Cacna1g, Hhip, Mtss1, Jund, Ndufa10, Pbxip1, Dusp3, Gabarapl1, Zdhhc8, Mrgprf
V$AREB6_02	14(776)	0,000580571	Slc6a7, Fads3, S100a16, Camkk1, Dlg2, S100a1, Rragc, Wnt9a, Wwp2, Crat, Usp2, Cox6a2, Dmpk, Dgkg

Transcription Factor	*Number of genes*	*Corrected p-value*	*Genes induced in Rasless cells (from Table S1)*
V$MYC_Q2	32(776)	0,0005886	Hexa, Aldh6a1, Fads3, Bmp4, Ankrd12, Tom1, Hoxa1, Arl2, Gabarap, Nrip3, Cbara1, Fabp3, Commd8, Slc31a2, Tgfb2, Sema3f, Camkk1, Gsk3b, Atp6v0b, Rragc, Mtus1, Gadd45b, Psen2, Camk4, Tom1l2, Hhip, Wwp2, Dirc2, Usp2, Mpv17, Znrf2, Abcb6
V$TCF1P_Q6	13(776)	0,00102772	Satb1, Dusp8, Zbtb4, Cdkn1a, Tuba8, Rbms2, Dlg2, Mtss1, App, Usp2, Slc9a6, Frmpd1, Mrgprf
V$ETS2_B	35(776)	0,00103851	Qpct, Stk10, C1qtnf5, Arhgef12, Rab43, Ltbp3, Dhps, Srr, Vps39, Rhoj, Hoxa1, Apba1, Cdkn1a, Ndrg4, C1qtnf6, Sema3f, Rbms2, Galnt2, Creb3, Rora, Atp2a3, Cdk5, Dab2ip, Fstl1, Impdh1, Wnt9a, Wwp2, Ecm1, Gpc4, Itpr2, Sparc, Fzd6, Slc35c2, Znrf2, Frmpd1
V$E4BP4_01	15(776)	0,00105635	Gabarapl2, Uchl1, Arnt, Atp2a3, Impdh1, Hist3h2a, Rasl10b, Ube2h, Ensa, Prnp, Tob1, Plcb1, Usp2, Slc35c2, Clstn1
V$ATF4_Q2	13(776)	0,00171272	Ppp2r5b, Nrip3, Selm, Gsk3b, Pkia, Maoa, Ube2h, Ecm1, Ndufa10, Dirc2, Usp2, Abcd1, Mrgprf
V$AP1_Q2_01	14(776)	0,00193938	Ltbp3, Cdkn1a, Cobl, Lmna, Cpa6, Snx10, Impdh1, Ndel1, Klf15, Sema3b, Rit1, Mpv17, Ntn4, Mrgprf
V$CP2_01	13(776)	0,00211979	Satb1, Hr, Pmp22, Ltbp3, Dhrs3, Atp2a3, Dab2ip, Dbp, Kif1c, Lamb2, Rhoq, Prelp, Pank2
V$BACH1_01	13(776)	0,00295799	Fads3, Cdkn1a, Camkk1, Lmna, Scrn1, Ecm1, Rab3d, Sparc, Gabarapl1, Mpv17, Ntn4, Abcd1, Mrgprf
V$GABP_B	25(776)	0,0031409	Stk10, Rab4b, Ufc1, Agl, Bmp4, Ttc15, Hoxa1, Mtcp1, Arhgap1, Slamf9, Galnt10, Acp2, Rbms2, Creb3, Tcta, Cdk5, Nkiras2, Iscu, Dtx2, Rab2b, Ing4, Nme2, Gpc4, Itpr2, Kif3b
V$FOXO1_02	12(776)	0,00314616	Hr, Dhrs3, Hoxa1, Cdkn1a, Ehd1, Rora, Fstl1, Ezh1, Sh2d4a, Thra, Clstn1, Mrgprf
V$NRF1_Q6	26(776)	0,0033216	Slc6a7, Npdc1, Zbtb4, Srr, Cacna1b, Ehd1, Akap13, Samd8, Abhd5, Tcta, Napb, Mef2b, Iscu, Mocs2, Nr2c1, Sgcb, Cog6, Snrpn, Vps24, Itgb1bp1, Cdkn2b, Kif3a, Stx8, Tollip, Abcb6, Kpna1
V$USF_01	12(776)	0,00343012	Aldh6a1, Bmp4, Ankrd12, Tom1, Hoxa1, Gabarap, Nrip3, Commd8, Slc31a2, Gsk3b, Tom1l2, Dirc2
V$MYOD_Q6	30(776)	0,00345492	Hr, Arhgef12, Ppp2r5b, Ank2, Chrd, Lhfpl2, Ehd1, Fabp3, Rnf13, Cobl, Galnt2, Rapsn, Camkk1, Gsk3b, Dlg2, Dab2ip, Rragc, Mtus1, Kif13b, Serf2, Adamts5, Plcb1, Crat, Ckm, Usp2, Dmpk, Ntn4, Mlycd, Rabac1, Tpcn1
V$IK2_01	13(776)	0,00359605	Pdgfc, Pmp22, Cryab, Sema3f, Rbms2, Vamp3, Rora, Napb, Pkia, Slc35a2, Gpc4, Usp2, Slco3a1
V$ETS1_B	12(776)	0,00393904	Stk10, Rab43, Vps39, Bmp4, Hoxa1, Apba1, Rbms2, Galnt2, Dab2ip, Impdh1, Kif1c, Itpr2
V$HEN1_02	10(776)	0,00401444	Ank2, Loxl1, Bmp4, Galnt10, Adamts2, Capn5, Impdh1, Rasl10b, Abtb2, Mrgprf
V$SOX5_01	12(776)	0,00426268	Ank2, Slc35b1, Dhrs3, Apba1, Ephb3, Dlg2, Fstl1, Hhip, Tob1, Gpc4, Mrc2, Plcb1
V$USF_02	12(776)	0,00532941	Dusp8, Aldh6a1, Ankrd12, Gabarap, Nrip3, Commd8, Slc31a2, Tgfb2, Vnn1, Tom1l2, Fzd6, Rhoq
V$ZIC3_01	12(776)	0,0063057	Fbxl20, Dhrs3, Cryab, Uchl1, Vamp3, Lmna, Fstl1, Mtus1, Impdh1, Sema3b, Thra, Mrgprf
V$NFY_Q6_01	32(776)	0,00652561	Fzd8, Aldh6a1, Rab4b, Bmp4, Ankrd12, Acyp1, Cacna1b, Sh3bgr, C1qtnf6, Smad6, Ugp2, Gsk3b, Dlg2, Tcta, Dbp, Atp10d, Cat, Hist3h2a, Ensa, Lancl1, Dleu2, Ap3m2, Pxmp4, Itpr2, Abtb2, Usp2, Rhoq, Cox6a2, Mlycd, Tpcn1, Pde6d, Pank2
V$NF1_Q6_01	12(776)	0,00746437	Fbxl20, Loxl1, Rhoj, Jdp2, Smad6, Lmna, Lgals9, Lamb2, Crot, Mtss1, Rras, Rhoq

Transcription Factor	*Number of genes*	*Corrected p-value*	*Genes induced in Rasless cells (from Table S1)*
V$AREB6_01	27(776)	0,00751332	Wisp2, Pdgfc, Rab43, Bmp4, Dhrs3, Hist1h3d, Gabarap, Repin1, Ndrg4, Uchl1, Tgfb2, Sema3f, Camkk1, Dlg2, Slc37a4, Kif1c, Rab2b, Cacna1g, Gpc4, Tbc1d14, Plcb1, Usp2, Thra, Fn1, Zdhhc8, Mpv17, Lynx1
V$ER_Q6	12(776)	0,00765758	Satb1, Pmp22, Slc35b1, Gabarapl2, Tuba8, Esrrb, Iscu, Myadm, Vps24, Gpc4, Ckm, Dusp3
V$SMAD3_Q6	11(776)	0,00844641	C1qtnf5, S100a16, Cdkn1a, Tgfb2, Smad6, Cdk5, Mrc2, Crat, Dusp3, Dmpk, Synpo
V$CMYB_01	11(776)	0,00869454	Hr, Aldh6a1, Tom1, Add3, Gsk3b, Slc39a3, Ube2h, Jund, Gpc4, Crat, Usp2
V$LHX3_01	13(776)	0,00877256	Jdp2, Stx7, Smad6, Sema3c, Rora, Add3, Rab22a, Slc37a4, Wnt9a, Rasl10b, Hhip, Mtss1, Tob1
V$SRF_Q6	12(776)	0,00877953	Agl, Rhoj, Hoxa1, Emilin2, Cacna1b, Myadm, Ckm, Prelp, Dmpk, Dgkg, Prkab2, Mrgprf
V$HIF1_Q5	11(776)	0,00889888	Slc6a7, Ero1l, Selm, Ephb3, Creb3, Rora, Ppt2, Slc37a4, Gadd45b, Abcb6, Cdc42ep2
V$EN1_01	7(776)	0,00903432	Ank2, Klf15, Ube2h, Atp9b, Rab2b, Gpc4, Plcb1
V$CREB_Q4_01	10(776)	0,00904348	Rbms2, Ndufb2, Maoa, Iscu, Ube2h, Hhip, Jund, Usp2, Rhoq, Mrgprf
V$SP1_01	11(776)	0,00928348	Srr, Nagk, Gabarap, Cryab, Mtmr3, Camkk1, Nfe2l1, Myadm, Kif1c, Mtss1, Clstn1
V$E2F1_Q3_01	11(776)	0,00928348	Satb1, Hr, Arhgef12, Aldh6a1, Vamp3, Reck, Elmo2, Fbxo9, Dleu2, Mrc2, Usp2
V$LFA1_Q6	11(776)	0,00956039	Rab4b, Gabarap, Npc2, Gsk3b, Kif1c, Cacna1g, Ppp1r16a, Bace1, Dmpk, Rasl12, Tpcn1
V$NRF2_01	10(776)	0,010015	Ufc1, Agl, Arhgap1, Acp2, Nkiras2, Insig2, Dtx2, Ing4, Wwp2, Rras
V$TATA_01	35(776)	0,0100572	Fzd8, Pdgfc, Srr, Klf2, Hdlbp, Cryab, Anxa1, Ehd1, Hist1h1e, Lyplal1, Uchl1, Tgfb2, Npc2, Fbln5, Sema3c, Rora, Tcta, Mtus1, Nkiras2, Slc37a4, Cables1, Gdf15, Ube2h, Stac, Ecm1, Tob1, Adamts5, Pltp, Ckm, Rit1, Fn1, Gpr133, Dmpk, Slc35c2, Slco3a1
V$CEBP_Q3	11(776)	0,0101357	Adamts2, Tgfb2, Arnt, Rora, Dlg2, Maoa, Hhip, Tob1, Gpc4, Pbxip1, Thra
V$ARNT_01	11(776)	0,0102785	Aldh6a1, Bmp4, Ankrd12, Hoxa1, Nrip3, Cbara1, Commd8, Tgfb2, Atp6v0b, Mtus1, Cry2
V$STAT5A_03	11(776)	0,0105823	Satb1, Tbcd, Dhrs3, C1qtnf6, Slc35a2, Fbxo9, Ube2h, Mtss1, Tob1, Gpc4, Fn1
V$EVI1_01	3(776)	0,0110184	Vamp3, Tob1, Crat
V$TCF11_01	10(776)	0,0111079	Jdp2, Rnf13, Dlg2, Rab22a, Slc41a2, Kif1c, Cdkn2b, Gpc4, Rit1, Mpv17
V$STAT6_01	11(776)	0,0118736	Satb1, Tbcd, Dhrs3, Mapk4, C1qtnf6, Slc35a2, Fbxo9, Mtss1, Tob1, Gpc4, Fn1
V$PXR_Q2	11(776)	0,0118736	Dhrs3, Smad6, Ugp2, Gsk3b, Fstl1, Scrn1, Cables1, Cacna1g, App, Slc6a6, Lrch1

Transcription Factor	*Number of genes*	*Corrected p-value*	*Genes induced in Rasless cells (from Table S1)*
V$PAX4_01	11(776)	0,0154196	Arhgef12, Rab43, Slc35b1, Suox, Hoxa1, Galnt10, Capn5, Iscu, Sema3b, Jund, Rras
V$ER_Q6_02	11(776)	0,0154196	Satb1, Gabarapl2, Hck, Cdkn1a, Creb3, Myadm, Vps24, Lamb2, Dusp3, Rhoq, Slco3a1
V$TFIIA_Q6	11(776)	0,0167056	Cspg4, Gabarapl2, Jdp2, Cryab, Lmna, Add3, Nkiras2, Anxa3, Sgca, Gpc4, Adamts5
V$AP1_Q4	11(776)	0,0167056	Ltbp3, Nrip3, Cdkn1a, Camkk1, Lmna, Esrrb, Scrn1, Dtx2, Usp2, Synpo, Abcb6
V$PTF1BETA_Q6	10(776)	0,0170972	Npdc1, Pmp22, Dusp22, Dhrs3, Apba1, Repin1, Smad6, Impdh1, Hhip, Thra
V$MZF1_01	10(776)	0,0170972	Hr, Fbxl20, Ank2, Hoxa1, Nrip3, Add3, Gsk3b, Ap3s1, Tob1, Prelp
V$GCM_Q2	10(776)	0,0170972	Ppp2r5b, Mfap5, Adra1b, Smad6, Add3, Fbxo9, Cacna1g, Pld1, Thra, Kpna1
V$ARP1_01	8(776)	0,0175343	C1qtnf5, Fbxl20, Ltbp3, Hoxa1, Dbp, Ensa, Cacna1g, Sparc
V$CREBP1_01	8(776)	0,0178288	Gabarapl2, Rbms2, Impdh1, Rasl10b, Prnp, Tob1, Usp2, Clstn1
V$GATA1_02	10(776)	0,0179157	Satb1, Bmp4, Apba1, Rora, Ube2h, Ing4, Ap3s1, Amhr2, Cry2, Cdc42ep2
V$USF2_Q6	10(776)	0,0182286	Fads3, Arl2, Nrip3, Cbara1, Fabp3, Tgfb2, Atp6v0b, Rragc, Psen2, Usp2
V$USF_Q6	10(776)	0,0187721	Hexa, Aldh6a1, Tom1, Hoxa1, Gabarap, Nrip3, Cbara1, Tom1l2, Dirc2, Mpv17
V$SOX9_B1	16(776)	0,0190617	Fbxl20, Mfap5, Apba1, Cdkn1a, Slc39a14, Fbln5, Dlg2, S100a1, Dab2ip, Fbn2, Kif1c, Mtss1, Maob, Adamts5, Tbc1d14, Fn1
V$TAL1ALPHAE47_01	10(776)	0,0198954	Scn1b, Gabarapl2, Hck, Hoxa1, Selm, Dtx2, Ap3s1, Riok3, Pbxip1, Plcb1
V$RFX1_02	10(776)	0,0204756	Ank2, Rpl17, Ehd1, Ppt2, Ap3m2, Pbxip1, Sparc, Dusp3, Camk1, Stx8
V$AR_01	7(776)	0,0206069	Pmp22, Cdkn1a, Rbms2, Dab2ip, Klf15, Abtb2, Usp2
V$EVI1_04	10(776)	0,021433	Pdgfc, Dhrs3, Cryab, Ankra2, Tgfb2, Vamp3, Mtus1, Cables1, Ap3m2, Tob1
V$CEBPA_01	10(776)	0,021433	Smad6, Arnt, Fbn2, Maoa, Fstl1, Hhip, Tob1, Gpc4, Thra, Rhoq
V$MMEF2_Q6	11(776)	0,0219037	Klf2, Dhrs3, Ehd1, Add3, Dlg2, Tcta, Insig2, Ckm, Usp2, Zdhhc8, Dmpk

Transcription Factor	*Number of genes*	*Corrected p-value*	*Genes induced in Rasless cells (from Table S1)*
V$ATF1_Q6	10(776)	0,0220471	Habp4, Ehd1, Smad6, Dlg2, Ube2h, Cacna1g, Hhip, Jund, Thra, Dusp3
V$STAT6_02	10(776)	0,0258523	Satb1, Vps39, Rhoj, Atp5e, Vamp3, Mtus1, Nkiras2, Usp2, Zdhhc8, Znrf2
V$PU1_Q6	18(776)	0,0260521	C1qtnf5, Hr, Tcn2, Fbxl20, Rhoj, Rpl17, Hoxa1, Slc39a14, Creb3, Rora, Add3, Atp2a3, Tcta, Impdh1, Rab2b, Cacna1g, Grik2, Itpr2
V$STAT_Q6	10(776)	0,0265547	Qpct, Agl, Mkrn1, Apba1, Add3, Nfs1, Slc41a2, Ube2h, Thra, Fn1
V$RSRFC4_Q2	16(776)	0,0269162	Kcnn1, Myom2, Cdkn1a, Dlg2, Atp2a3, Timp2, Elmo2, Stac, Gpc4, Ckm, Usp2, Rhoq, Gpr133, Slco3a1, Pde6d, Cdc42ep2
V$AP3_Q6	10(776)	0,0269897	Mmaa, Hdlbp, Npc2, Rora, Fstl1, Wwp2, Jund, Sparc, Fn1, Mpv17
V$RSRFC4_01	10(776)	0,0277151	Kcnn1, Ehd1, Dlg2, Atp2a3, Timp2, Stac, Gpc4, Ckm, Usp2, Slco3a1
V$AP2REP_01	8(776)	0,0277768	Zbtb4, Ltbp3, Rhoj, Cobl, Rora, Ube2h, Pcsk6, Frmpd1
V$AP1_Q6_01	10(776)	0,0281699	Nrip3, Lmna, Snx10, Scrn1, Tob1, Gabarapl1, Dmpk, Mpv17, Abcd1, Abcb6
V$ZID_01	9(776)	0,0287602	Hr, Gabarapl2, Chrd, Hist1h3d, C1qtnf6, Creb3, Iscu, Abtb2, Prelp
V$GATA2_01	6(776)	0,0289335	Rbms2, Ube2h, Grik2, Mpv17, Znrf2, Abcb6
V$P53_DECAMER_Q2	10(776)	0,0291108	Rab43, Cdkn1a, Anxa1, Add3, Dlg2, Dab2ip, Nkiras2, Nfe2l1, Abcd1, Slco3a1
V$EGR2_01	8(776)	0,0291922	Fbxl20, Chrd, Ephb3, Kif1c, Wwp2, Gpc4, Mrc2, Thra
V$AP2ALPHA_01	9(776)	0,0348934	Hr, Ppp2r5b, S100a16, Jund, Thra, Dmpk, Znrf2, Dgkg, Frmpd1
V$RP58_01	9(776)	0,0365516	Ank2, Ankrd12, Adamts2, Wnt9a, Dtx2, C1qtnf1, Cacna1g, Cdc42ep2, Mrgprf
V$TAL1BETAE47_01	9(776)	0,0365516	Scn1b, Gabarapl2, Hck, Selm, Wnt9a, Dtx2, Riok3, Pbxip1, Plcb1
V$MYB_Q6	9(776)	0,0365516	Ank2, Rab4b, Ssbp2, Rapsn, Tcta, Ube2h, Cacna1g, Fbxl2, Synpo
V$USF_C	10(776)	0,0375398	Fads3, Bmp4, Gabarap, Slc31a2, Psen2, Camk4, Hhip, Usp2, Znrf2, Abcb6
V$ELF1_Q6	9(776)	0,0375737	Dusp22, Ttc15, Rpl17, Creb3, Fbln5, Add3, Atp2a3, Myadm, Itpr2

Transcription Factor	*Number of genes*	*Corrected p-value*	*Genes induced in Rasless cells (from Table S1)*
V$HEN1_01	8(776)	0,0395718	Ank2, Loxl1, Bmp4, Capn5, Impdh1, Rasl10b, Abtb2, Mrgprf
V$OCT_C	14(776)	0,0429247	Arhgef12, Ephb3, Mtus1, Scrn1, Mras, Wnt9a, Hist3h2a, Jund, Rras, Sgca, Gpc4, Abtb2, Thra, Dgkg
V$ZIC2_01	9(776)	0,0433534	Hr, Dhrs3, Cryab, Rab22a, Fstl1, Mtus1, Impdh1, Sema3b, Usp2
V$MYCMAX_03	9(776)	0,0444984	Hexa, Aldh6a1, Tom1, Hoxa1, Gabarap, Nrip3, Cbara1, Uchl1, Tgfb2
V$AP1FJ_Q2	10(776)	0,0450397	Ltbp3, Nrip3, Camkk1, Lmna, Rab22a, Scrn1, Dtx2, Tob1, Usp2, Synpo
V$PAX3_B	8(776)	0,046049	Klf2, Cdkn1a, Habp4, Ndufb2, Fbln5, Ppt2, Ndel1, Stxbp1
V$PPARA_02	6(776)	0,0461552	Hr, Man2b1, Add3, Dlg2, Nkiras2, Kif1c
V$SREBP1_02	5(776)	0,0461704	Loxl1, Dhrs3, Kif1c, Jund, Ntn4
V$GATA1_04	9(776)	0,047245	Ank2, Pon3, Rora, Esrrb, Pkia, Slc35a2, Ube2h, Amhr2, Synpo
V$FAC1_01	8(776)	0,0480052	Fbxl20, Kcnc2, Gabarapl2, Gabarap, Mtmr3, Wnt9a, Klf15, Cacna1g
V$SRF_Q5_01	9(776)	0,0480555	Rhoj, Emilin2, Cacna1b, Myadm, Ube2h, Ckm, Dgkg, Prkab2, Mrgprf
V$AP4_01	9(776)	0,0480969	Ank2, Ssbp2, Mtmr3, Rapsn, Gsk3b, Rasl10b, Klf15, Gpc4, Usp2
V$CREB_01	9(776)	0,0480969	Rbms2, Ndufb2, Maoa, Ube2h, Ing4, Hhip, Jund, Ndufa10, Mrgprf
V$STAT4_01	9(776)	0,0480969	Satb1, Cspg4, Rhoj, Mtus1, Usp2, Fn1, Rhoq, Zdhhc8, Pde6d
V$YY1_Q6	13(776)	0,0482511	Zbtb4, Fzd8, Rab4b, Mtmr3, Arnt, Reck, Rab22a, Scrn1, Nfe2l1, Ensa, Ing4, Snrpn, Tbc1d14
V$P53_02	9(776)	0,0489325	Ppp2r5b, Repin1, Cdkn1a, Anxa1, Dab2ip, Gadd45b, Mrc2, Plcb1, Crat
V$COUP_01	9(776)	0,0489325	Satb1, Aldh6a1, Slc7a7, Dhrs3, Selm, Lmna, Dab2ip, Dusp3, Tpcn1
V$PR_Q2	9(776)	0,0497807	Tcn2, Ltbp3, Jdp2, Ssbp2, Wnt9a, Dtx2, Cacna1g, Wwp2, Znrf2
V$AFP1_Q6	9(776)	0,0497807	Agl, Hoxa1, Stx7, Sema3c, Rasl10b, Klf15, Ing4, Gpc4, Dmpk

Table S3-miRNA. Functional annotation to "miRNA" of the induced, overexpressed genes of Rasless MEFs.

miRNA name	*Number of genes*	*Corrected p-value*	*miRNA targets among genes induced in Rasless cells (from Table S1)*
**mmu-miR-762**	47(776)	2,85E-10	Ppp1r16a,Yif1b,Sgca,Stmn3,Slc6a7,Hspa12a,Rab4b,Trpc7,Slc22a18,Lrrc51,Clstn1,Ppp2r5b,Crybb3,Copz2,Renbp,Adcy9,Dhrs1,Ephb3,Gstm2,Ccdc64,Ndel1,Grina,Zbtb4,Pbxip1,Rapsn,P4ha2,Snta1,Ppt2,Ascc1,Dusp8,Ergic3,Lims2,Ing4,Atp6v0b,Def8,Klf2,Tanc2,Tex264,Dpm2,Fbn2,Mras,Dhps,Prelp,Cdkn2b,Fbxw4,Cacna1b,2810428I15Rik
**mmu-miR-770-3p**	44(776)	7,19E-10	Yif1b,Stmn3,Slc6a7,Ascc2,Tmem41a,Nt5m,Clstn1,Ppp2r5b,Slc35a2,Trpt1,Btf3l4,Dgkg,Ttc15,Dph1,Itgb1bp1,Cspg4,Bphl,P4ha2,Lims2,Deb1,Rdh12,Os9,Naga,Npdc1,Necap2,Gabarap,Ahnak,Mrpl55,Glo1,Slc41a3,Spata21,Kif13b,Ssbp2,Hck,BC046404,Itfg3,Dhps,Igbp1,Scn1b,Tbc1d9b,Cyb5r3,Slc2a8,Ltbp3,Zfp704
**mmu-miR-666-5p**	49(776)	1,44E-09	Mrc2,Zfand3,Sgca,Stmn3,Pef1,Ascc2,Adc,Birc7,S100a1,Trpc7,Alg14,Hexa,Atp6v0e,Emilin2,Cd81,Loxl1,1810020D17Rik,Anxa3,Skap2,Slc31a2,Gpr133,Slc44a1,Ttc15,Suox,Trappc2l,Gstk1,Galns,Ndufa10,Itgb1bp1,Arhgef12,Impdh1,Bphl,Snta1,Itpr2,Bbs5,Ctsb,Atp6v0b,Slc39a14,Sorbs3,Ptprm,Ndrg4,1810046J19Rik,Dnajc4,Tbc1d9b,Rabac1,Cyb5r3,Pcsk6,Ltbp3,Npc1
**mmu-miR-99a**	45(776)	1,71E-09	Ahnak2,Adra1b,Stmn3,Slc6a7,Alg14,Insig2,Cd81,Adamts5,Cd248,F630110N24Rik,Amhr2,1810020D17Rik,Tmco3,9430038I01Rik,Cdc42ep2,Sepw1,Hoxa1,Ndel1,Tcn2,Rora,Zbtb4,Nfe2l1,Impdh1,Serpinb9b,Ergic3,Mmaa,Fabp3,Adamtsl5,Zmat2,Tollip,Nol3,Plcb1,Ankrd29,Atp6v0b,App,Ahnak,Fzd8,Ptprm,2900026A02Rik,Tpp1,Igfbp2,Rhoj,Scn1b,Atp10d,Shroom3
**mmu-miR-423-5p**	44(776)	2,68E-09	1300014I06Rik,Dtx2,S100a1,Trpc7,Cyp27a1,Clstn1,AI593442,Upk3b,Ppp2r5b,Akap13,Sgcb,Clip1,Qk,Ndel1,Gdf15,Shank3,L1cam,Man2b1,Rapsn,P4ha2,Ascc1,Dusp8,Rell2,2310046K01Rik,Os9,Ing4,Mtcp1,Frmpd1,Atp6v0b,Gabarap,Sorbs3,Fzd8,Ssbp2,Tex264,Slc25a39,Mras,Cdkn1a,Dhps,1810046J19Rik,Ckm,Lmna,Limch1,Ltbp3,Shroom3
**mmu-miR-34c**	46(776)	5,73E-09	Ahnak2,Ifi35,Rpl17,Fbxo9,Nt5m,Hexa,Sh3bgr,Rras,Wnt9a,D16H22S680E,Stk39,Sync,Nudt13,Nfs1,Vps39,Slc31a2,9430038I01Rik,Ttc15,Ccdc64,Suox,Guk1,Galns,Arl2,Nfe2l1,Pbxip1,Bphl,Nrip3,Lims2,Glt8d1,Rell2,Mtcp1,Plcb1,Ankrd29,Acyp2,Tex264,Slc2a6,Ptprm,Dpm2,Itfg3,Mras,Nhlrc1,Wscd2,Chst12,Lmna,Dusp22,Zfp704
**mmu-miR-24**	43(776)	1,07E-08	1300014I06Rik,Ecm1,Rab4b,Gabarapl2,Hexa,Rras,Ehd1,Emilin2,Cd81,Kcnn1,Cyp4v3,Neu1,Iqsec2,Abcd1,BC027231,Appl2,Chrd,Rapsn,P4ha2,Dusp8,Lrrc57,Htra3,Slc35c2,S100a13,Itpr2,Os9,Naga,Necap2,Def8,Clip4,Tex264,Slc25a39,Mras,Clcn7,Scarb2,Tom1l2,Slc25a35,Atp5e,Fbxw4,Cpeb1,1810030N24Rik,Tmub2,Ltbp3
**mmu-miR-337-3p**	42(776)	1,31E-08	Zfand3,Maob,Sgca,Plb1,Pef1,Mal,Fbxo9,Papss2,Alg14,Sh3bgr,Elmo2,Wnt9a,Cd81,Abcb6,Acox1,Slc35a2,Tbcd,Adcy9,BC027231,Clip1,Ephb3,Myom2,Gstk1,Chrd,Cspg4,Mtap6,Snta1,Cables1,C330006K01Rik,Mlycd,Bbs5,BC089491,Slc39a14,Clip4,Nr2c1,Csrp2,Scarb2,Gas6,Slamf9,Fbxw4,Fads3,Nagk
**mmu-miR-504**	42(776)	1,41E-08	Ppp1r16a,Dtx2,Usp2,Cox4nb,Prss29,D16H22S680E,Kcnn1,Tgfb2,Adcy9,9830001H06Rik,Lmbr1,Camk1,Alkbh6,Myom2,Lgals9,Tpcn1,Arl2,Nipa1,Tcn2,Grina,Shank3,Epb4.1l4b,Idua,Rdh12,Adamtsl5,Nkiras2,Naga,Tollip,Gpc4,Ankrd29,0610037L13Rik,Atp6v0b,Zfand2b,Sirt2,Slc2a6,Ikbkg,BC046404,Igfbp2,Fbxw4,Limch1,Pcsk6,Aldh3a2
**mmu-miR-30a***	41(776)	3,88E-08	Sqrdl,Adc,Prss29,Nt5m,Cyp4v3,Cd248,Trappc6a,Dusp3,Calml4,Sepw1,Tob1,Ndufa10,Epb4.1l4b,Mtap6,P4ha2,Hist1h1e,Dusp8,Lrrc57,Iscu,Ccdc80,Slc22a4,Bbs5,Sned1,Nol3,Pde6d,Pltp,Srr,Slc26a11,Tanc2,Acyp2,Asb1,Ptprm,Mxra7,Vps24,Cpa6,BC004004,Adamtsl4,Gas6,Slamf9,1810030N24Rik,Cox6b1
**mmu-miR-122**	41(776)	4,22E-08	Dtx2,Stmn3,Adc,Mpv17,Sema3b,Nt5m,Sh3bgr,Atp6v0e,R74862,1810020D17Rik,Vps8,Dgkg,Lama2,Slc46a3,Rragc,2010003O02Rik,Ccdc64,Arl2,Gdf15,C1qtnf6,P4ha2,Serpinb9b,Cables1,Iscu,Psen2,Dopey2,Pde6d,Plcb1,Sh2d4a,Pxmp4,Ankrd29,Pdia5,Vps24,BC046404,Fbxl20,Htatip2,Arhgap1,Acyp1,Cpeb1,Ltbp3,Immp1l
**mmu-miR-18a**	44(776)	5,42E-08	Kif27,Lgr4,Cox4nb,Ascc2,Mpv17,Cyp27a1,Rras,Wnt9a,D16H22S680E,Inpp5a,AI593442,Smad6,Caml,Myadm,Tbcd,Akap13,Renbp,Adcy9,Skap2,Slc44a1,Fbxl2,Herpud2,Arhgef12,Slc35b1,2700078K21Rik,Serpinb9b,Sec22c,Iscu,Glt8d1,Rell2,Adamtsl5,Abtb2,Srp14,Ahnak,Cxcr7,Ikbkg,Ufc1,Cpa6,Cbara1,Acaa1a,Cpeb1,Ltbp3,Uchl1,Nagk
**mmu-miR-423-3p**	41(776)	7,44E-08	Dtx2,Commd8,Pef1,Prss29,Rab4b,Gpx4,Cyp27a1,Gabarapl2,Rras,Elmo2,Ehd1,Clstn1,Crybb3,F630110N24Rik,Qk,Dph1,Mapk4,Ndel1,Rora,Nme2,Htra3,Adar,Atg4a,Adamtsl5,Mustn1,Necap2,2010111I01Rik,Gabarap,Il17d,Kif13b,Atp5s,Slc25a39,Slc2a6,Vps24,Csrp2,Mras,Igfbp2,Gas6,Slc2a8,Ltbp3,Aldh3a2
**mmu-miR-133b**	43(776)	7,46E-08	Mrc2,Eif5a2,Ifi35,Sqrdl,Adc,Slc39a3,Rras,Clstn1,Slc35a2,F630110N24Rik,Akap13,Gramd3,Klk1b4,Txnrd3,Fbxl2,Appl2,Guk1,Arl2,Grina,Nfe2l1,Cspg4,Wwp2,Ehd3,Serpinb9b,Nrip3,Ttll7,Iscu,Rell2,Garnl3,Rdh12,Slc22a4,Atg7,Ahnak,Slc25a39,Slc2a6,Serf2,Dpm2,Csrp2,Scarb2,Gas6,Slamf9,Cacna1b,2810428I15Rik

miRNA name	Number of genes	Corrected p-value	miRNA targets among genes induced in Rasless cells (from Table S1)
**mmu-miR-671-5p**	44(776)	7,51E-08	Ppp1r16a,Sqrdl,Yif1b,Slc37a4,Plb1,Prss29,Magi3,Rras,Krt80,Nudt13,Atp9b,Atp6v1e1,Dynlrb1,Akap13,Clip1,Txnrd3,Zbtb4,Nfe2l1,Impdh1,Slc35b1,Rapsn,Bphl,Ascc1,Idua,Ehd3,Nrip3,Mfap5,Ly6c1,Ing4,Hscb,Ctsb,2010111I01Rik,Atp6v0b,Ikbkg,Hck,Cog6,Dhps,Tom1l2,Igfbp2,Atp6v0a1,Chst12,Pcsk6,Ltbp3,Shroom3
**mmu-miR-673-3p**	42(776)	7,84E-08	Dtx2,Kif27,Prss29,Sema3b,Hexa,5930434B04Rik,Mtap1a,Gadd45b,Loxl1,Trappc6a,Iqsec2,Crybb3,Renbp,Abcd1,Adcy9,Vps8,Sepw1,Cacna1g,Ephb3,Ccdc64,Gstk1,Hsd3b3,Wisp2,Ypel3,Shank3,Nfe2l1,Pbxip1,2700078K21Rik,Snta1,Pon3,Pltp,Necap2,Mrpl55,Fbn2,Mras,Scarb2,Rabac1,Uchl1,Yif1a,Cacna1b,Shroom3,Ankrd44
**mmu-miR-214**	44(776)	1,13E-07	Pank2,Mrc2,Ifi35,Rab43,Stmn3,Slc37a4,Snrpn,Arvcf,Rab4b,Lrrc51,Rras,Cd81,Atp6v1e1,Iqsec2,Dusp3,Gramd3,Skap2,Arl2,Tcn2,Itgb1bp1,Shank3,Pbxip1,Mtap6,Nudt7,Slc35c2,S100a13,Ly6c1,Sh2d4a,Ctsb,1300010F03Rik,Atp6v0b,BC029214,Uqcrq,Slc25a39,Cbara1,Dnajc4,Tbc1d14,Atp6v0a1,Rit1,Rabac1,Cyb5r3,Ltbp3,Fads3,2810428I15Rik
**mmu-miR-100**	39(776)	1,13E-07	Fech,Ahnak2,Adra1b,Stmn3,Alg14,Insig2,Rab40b,Znrf2,Adamts5,Smad6,Sync,F630110N24Rik,Amhr2,1810020D17Rik,Clip1,Tmco3,Mxra8,9430038I01Rik,Cdc42ep2,Sepw1,Hoxa1,Tcn2,Rora,Impdh1,Ergic3,Fabp3,Zmat2,Nol3,Plcb1,Atp6v0b,App,Ahnak,Clip4,Fzd8,Ptprm,Tpp1,Scn1b,Dusp22,Shroom3
**mmu-miR-125b-3p**	44(776)	1,13E-07	Mrc2,Iah1,Col14a1,Sgca,Dhrs3,Alg14,Sema3b,Nt5m,Trpv4,Lamb2,Vps39,Dusp3,Amhr2,Copz2,Clip1,Camk1,Ttc15,Ephb3,Myom2,Txnrd3,Tpcn1,Tcn2,Ndufa10,Itgb1bp1,Chrd,Zbtb4,Rapsn,Snta1,Lims2,Os9,S100a16,Sned1,Mustn1,Zfand2b,Asb1,Tex264,Slc25a39,Ptprm,Cdh7,Nudt18,Adamtsl4,Scarb2,Ltbp3,Mtus1
**mmu-miR-330**	40(776)	1,16E-07	Adc,Prss29,Sema3b,Hexa,Emilin2,Cd81,Ppapdc3,Smad6,Klf15,Iqsec2,Acox1,Copz2,Dab2ip,Tbcd,Rarres2,Clip1,Lgals9,Ccdc64,Idua,Ergic3,Tmem175,Mlycd,Itpr2,Ing4,Cryab,Tm9sf4,Zfand2b,Def8,Cyb5,Ptprm,Fbxl20,Gpr39,Acaa1a,Nhlrc1,Wscd2,Atp6v0a1,Rabac1,Lmna,Yif1a,Aph1a
**mmu-miR-30c-1***	40(776)	1,19E-07	Copg,Kif27,Ascc2,S100a1,Fahd2a,Smpdl3a,R74862,Clstn1,Prss36,Ppapdc3,Vps39,Copz2,Rarres2,Dhrs1,Tmco3,Arnt,Txnrd3,Arl2,Ypel3,Shank3,2700078K21Rik,Nupr1,Ly6c1,Ing4,Gdap2,Pde6d,Ctsb,Slc25a39,Ptprm,Itfg3,Csrp2,Mras,1810046J19Rik,Tom1l2,Slamf9,Atp6v0a1,1810030N24Rik,Wdr7,Cacna1b,1700094D03Rik
**mmu-miR-323-5p**	42(776)	1,23E-07	Ppp1r16a,Zfand3,Cat,Pef1,Tmem41a,Magi3,Rbms2,Habp4,Dscr3,Clstn1,Smad6,Tgfb2,F630110N24Rik,Copz2,Tbc1d22a,Tor3a,Pld1,Vps8,Anxa3,Sepw1,Myo6,Galns,Arl2,Tcn2,Impdh1,P4ha2,Ascc1,Dusp8,Fabp3,Garnl3,Mlycd,Pde4dip,Rab7l1,Asb1,Ufc1,Htatip2,Adamtsl4,Kif3a,Gas6,Uchl1,Cacna1b,2810428I15Rik
**mmu-miR-883b-5p**	44(776)	1,24E-07	Zfand3,Snap91,2310028O11Rik,Stmn3,Hspa12a,Gyg,Tlr6,Gabarapl2,Rab38,Cobl,Aldh7a1,Sh3bgr,Wnt9a,D16H22S680E,Fn1,Cyp4v3,Sync,Trpv4,Iqsec2,Crybb3,Amhr2,Myadm,Adcy9,1810020D17Rik,Vps8,Anxa3,Skap2,Ttc15,Slc9a6,Gp1bb,Cables1,Lims2,Glt8d1,2310046K01Rik,1300010F03Rik,BC029214,Asb1,Glipr1,Hck,Dpm2,Cdkn1a,Acaa1a,Zfp704,Tmem9b
**mmu-miR-693-3p**	40(776)	1,40E-07	Ppp1r16a,Rab4b,Elmo2,Ehd1,Emilin2,Clstn1,Nudt13,Nfs1,Creb3,Trappc6a,Dynlrb1,Akap13,Adcy9,BC027231,Skap2,Arl2,Dusp8,Deb1,Ppm1h,Ccdc80,Anxa1,Vdac1,Nkiras2,Pcyt1a,Ctsb,Hgsnat,Srp14,Isca2,Mrpl55,Tanc2,Ufc1,Itfg3,Scarb2,Fbxw4,Wdr7,Rabac1,Pcsk6,Zfp704,Nagk,Shroom3
**mmu-miR-666-3p**	41(776)	1,55E-07	Zfand3,Yif1b,Usp2,Adc,Dhrs3,Nt5m,Krt80,Inpp5a,Hdlbp,Trpv4,Nudt13,Slc35a2,F630110N24Rik,Armc8,Rragc,Arl2,Itgb1bp1,Arhgef12,Tom1,Impdh1,Snta1,St3gal5,Mocs2,Ttll7,Bbs5,Ing4,Nenf,Npdc1,Asb1,Sema3f,Hck,BC046404,Dpm2,Ckm,Gas6,1500011K16Rik,Fbxw4,Rabac1,Slc2a8,Pcsk6,Dmpk
**mmu-miR-30e***	39(776)	1,69E-07	Sqrdl,Adc,Prss29,Cyp4v3,Cd248,Dusp3,Calml4,Sepw1,Myom2,Tob1,Ndufa10,Mtap6,Idua,Dusp8,Lrrc57,Iscu,Deb1,Ccdc80,Lrch1,Bbs5,Sned1,Nol3,Pde6d,Pltp,Mtm1,Slc26a11,Acyp2,Asb1,Nr2c1,Ptprm,Mxra7,Vps24,Cpa6,BC004004,Adamtsl4,Gas6,Slamf9,1810030N24Rik,Cox6b1
**mmu-miR-676**	37(776)	1,88E-07	Zfand3,Pmp22,Cox4nb,Trpc7,Alg14,Lrrc51,Rras,Atp6v0e,Atp6v1e1,Cd248,F630110N24Rik,Dusp3,Copz2,Dgkg,Slc31a2,5830405N20Rik,Rasl12,Grina,Ypel3,L1cam,Rell2,2310046K01Rik,Nupr1,Naga,Vnn1,BC089491,Mustn1,Ankrd29,Slc41a3,Acyp1,Cog6,2900026A02Rik,Cdkn2b,Igbp1,Atp6v0a1,Stxbp1,1700094D03Rik
**mmu-miR-744**	42(776)	1,89E-07	Ahnak2,Adc,Rab4b,Lrrc51,Elmo2,Emilin2,Creb3,F630110N24Rik,Pigz,Sepw1,Cacna1g,Myo6,Ephb3,Gstm2,Arl2,Tcn2,Ndufa10,Itgb1bp1,Zbtb4,Pbxip1,2700078K21Rik,Snta1,Rdh12,Plcb1,4930523C07Rik,Pde4dip,Zfand2b,Uqcrq,Ptprm,1110034G24Rik,1810046J19Rik,Ckm,Fbxw4,Scn1b,Atp6v0a1,Camkk1,Rabac1,Pcsk6,Cox6b1,Uchl1,Mtus1,2810428I15Rik
**mmu-miR-449c**	41(776)	2,07E-07	Ifi35,Thra,Pmp22,Zdhhc4,Tcta,Magi3,Fbxo9,Nt5m,Hexa,Rras,Slc16a9,Nfs1,Vps39,Akap13,1110007C09Rik,Trpt1,Cry2,Ttc15,Pdgfc,Gstm2,Suox,Galns,Rora,Shank3,St3gal5,Mocs2,Mfap5,Fabp3,Rell2,1300010F03Rik,Def8,Acyp2,Tex264,Cog6,Mras,Tom1l2,Wscd2,Scn1b,Tubb2a,Zfp704,1700094D03Rik
**mmu-miR-145**	39(776)	2,23E-07	Snap91,Copg,Fahd2a,Nt5m,Fem1a,D16H22S680E,Nudt13,Loxl1,Nfs1,Vps39,Slc35a2,Lhfpl2,Myo6,Rora,Cspg4,Myeov2,Slc35b1,C1qtnf6,Nudt7,Lrrc57,Nrip3,Lims2,Pik3ip1,Ccdc80,1110003E01Rik,Rdh12,Slc22a4,Os9,Abtb2,Npdc1,Lrp4,Atp6v0b,BC046404,1810027O10Rik,Dpm2,Csrp2,Cpeb1,Mdfic,Lrsam1
**mmu-miR-764-3p**	40(776)	2,24E-07	Sqrdl,Yif1b,Copg,Plb1,Slc6a7,Prss29,Rras,Wnt9a,Scrn1,Atp6v1e1,Crybb3,Copz2,Renbp,Trpt1,Arnt,Txnrd3,2900010M23Rik,Tpcn1,Tob1,Snta1,2310046K01Rik,Ing4,Naga,S100a16,Gpc4,Lrp4,Atp6v0b,Sorbs3,Sirt2,Hck,1110034G24Rik,Cox6a2,Igfbp2,Scn1b,Tmub2,Chst12,Pcsk6,Uchl1,Dmpk,Shroom3

miRNA name	*Number of genes*	*Corrected p-value*	*miRNA targets among genes induced in Rasless cells (from Table S1)*
**mmu-miR-877**	35(776)	2,52E-07	Copg,Sgca,Tcta,Rab4b,Rab3d,Sh3bgr,Cd81,Caml,Iqsec2,Slc7a7,Adcy9,Pld1,Pigz,Ccny,AI317395,Mkrn1,Gstm2,Qpct,Tcn2,Slc35b1,Rapsn,Lims2,Pon3,Fabp3,Tollip,Tanc2,Slc41a3,Nr2c1,Vps24,Cbara1,Ckm,Slc25a35,Gas6,2610019F03Rik,Cox6b1
**mmu-miR-124**	43(776)	2,56E-07	Pank2,Mrc2,Ugp2,Dtx2,Ascc2,Rras,Dscr3,AI593442,Trpv4,Klf15,Tbcd,Abcd1,Adcy9,Trpt1,Dhrs1,Skap2,Slc31a2,Lgals9,Synpo,Suox,Trappc2l,Gstk1,Chrd,Hsd3b3,P4ha2,Snta1,2700078E11Rik,Grik2,Mlycd,Ing4,Mtm1,Grk4,Impact,Cyb5,Slc25a39,Ptprm,Sema3f,Hck,BC046404,Htatip2,Itfg3,Tbc1d14,Cacna1b
**mmu-miR-99b**	38(776)	2,62E-07	Maob,Slc6a7,Alg14,Cd81,Znrf2,Abcb6,Kif1c,F630110N24Rik,Amhr2,Tmco3,Cdc42ep2,Hoxa1,Atp2a3,Tcn2,Wisp2,Rora,Zbtb4,Impdh1,Ppt2,Serpinb9b,Pik3ip1,Zmat2,Sned1,Nol3,Ankrd29,Pde4dip,Atp6v0b,App,Ahnak,Tmed8,Fzd8,Ptprm,Ckm,Tpp1,Igfbp2,Scn1b,Mtus1,Shroom3
**mmu-miR-34b-5p**	42(776)	2,68E-07	Dym,Ecm1,Rpl17,Fbxo9,Nt5m,Hexa,Sh3bgr,Rras,Sync,Nudt13,Nfs1,Vps39,9430038I01Rik,Ttc15,Tpcn1,Trim36,Suox,Arl2,Nfe2l1,Pbxip1,Bphl,Ppt2,Serpinb9c,Nrip3,Glt8d1,Rell2,Bbs5,Mtcp1,Plcb1,Ankrd29,Pde4dip,Acyp2,Tex264,Ptprm,Itfg3,Cog6,Mras,Acaa1a,Nhlrc1,Wscd2,Zfp704,1700094D03Rik
**mmu-miR-194**	40(776)	2,71E-07	Pank2,Kif27,Gyg,Fahd2a,Rab4b,Tlr6,Nt5m,Sh3bgr,Emilin2,Cd81,Sync,Atp6v1e1,Tbcd,Adcy9,Vps8,2010003O02Rik,Hsd3b3,Itga9,Stard6,Slc9a6,Bphl,Ppt2,Nudt7,Tmem175,Psen2,Adamtsl5,Os9,Dner,Isca2,BC029214,Glo1,Tbccd1,Nr2c1,Ufc1,1110034G24Rik,Kif3a,Hadhb,Fzd6,Rabac1,Uchl1
**mmu-miR-337-5p**	38(776)	3,12E-07	Ppp1r16a,Dym,Zfand3,Thra,Cat,Slc6a7,Tmem41a,Rab4b,Aldh7a1,Emilin2,Dscr3,Vps39,Ank2,Slc35a2,Tbcd,Skap2,Ndel1,Ndufa10,Slc9a6,Rapsn,P4ha2,Iscu,Ppm1h,Tmem154,Itpr2,Dopey2,Dner,Plcb1,BC029214,Abhd5,Pcbd2,Rab7l1,Slc25a39,Htatip2,Ckm,Gdap1,Tbc1d9b,1700021C14Rik
**mmu-miR-139-5p**	41(776)	3,27E-07	Ppp1r16a,Zfand3,Thra,Map3k8,Slc6a7,Adc,Dhrs3,Trpc7,Nt5m,Rab11fip5,Emilin2,Inpp5a,Neu1,Lmbr1,Guk1,Qpct,Impdh1,Myeov2,Sec22c,Tmem175,Rell2,Psen2,Hbxip,Dopey2,Tollip,Plcb1,Sh2d4a,Pxmp4,Gpc4,Rab7l1,Tbccd1,Uqcrq,Ssfa2,Fbn2,Acyp1,Cog6,1810030N24Rik,2610019F03Rik,Mdfic,Vps29,1700094D03Rik
**mmu-miR-693-5p**	42(776)	3,74E-07	Stmn3,Slc6a7,Rbms2,Abcb6,Prss36,Nfs1,Atp6v1e1,Trappc6a,Iqsec2,Slc35a2,Renbp,Pld1,Btf3l4,Lyplal1,Lyrm1,Ero1l,Sepw1,Ypel3,Shank3,P4ha2,Ppt2,Ascc1,Nudt7,Nrip3,Fabp3,Mlycd,Vdac1,Dner,Ctsb,0610037L13Rik,Srp14,Slc41a3,Cyb5,Tmbim1,Glipr1,1810046J19Rik,Scarb2,Stx7,Pcsk6,Ltbp3,Uchl1,Aldh3a2
**mmu-miR-31**	39(776)	3,84E-07	Dtx2,Pef1,Ascc2,Tmem41a,Rras,Abcb6,Cyp4v3,Loxl1,Vps39,Slc35a2,Lyrm1,Dhrs1,Myom2,Herpud2,Lnx1,Ypel3,2700078K21Rik,Mtap6,Wwp2,Ppt2,Serpinb9c,Nrip3,Zdhhc8,Nkiras2,Atg7,Triap1,Grk4,Ahnak,Rab7l1,Asb1,Vps24,Trp53inp2,Cdh7,1110034G24Rik,Igbp1,Atp6v0a1,Tbc1d9b,Atp10d,Shroom3
**mmu-miR-17**	43(776)	3,84E-07	Map3k8,Adc,D16H22S680E,Smad6,Caml,Loxl1,Creb3,Armc8,Dok4,Mapk4,Rora,Pbxip1,Nudt7,Idua,St3gal5,Dusp8,2700078E11Rik,Serpinb9b,Mfap5,Htra3,Adamtsl5,Bbs5,Ing4,Ntn4,Dbp,Mmab,App,Ahnak,Tmed8,Clip4,Synpo2,Nr2c1,Chmp4c,Ufc1,Fbxl20,Cbara1,Acaa1a,Nhlrc1,Uchl1,Zfp704,Atp10d,Nagk,Ankrd44
**mmu-miR-742**	38(776)	4,11E-07	Gtf2h5,Sqrdl,Map3k8,Col14a1,Prss29,Crot,Coq10a,Fbxo9,Fem1a,Sh3bgr,Atp6v0e,Wnt9a,Smad6,Cyp4v3,Tmem64,Nfs1,Lyplal1,Ankra2,Zbtb4,Stard6,C1qtnf6,P4ha2,Fabp3,Gdap2,5530400B01Rik,Pde6d,Atp6v0b,Def8,Slco3a1,Uqcrq,Ikbkg,BC046404,Mras,Nudt18,Wscd2,Cyb5r3,Lmna,Ltbp3
**mmu-miR-105**	39(776)	4,86E-07	Gtf2h5,Sgca,Kif27,Mpv17,Lactb2,Alg14,Elmo2,Napb,Kcnn1,Trpv4,Crybb3,Dab2ip,Myo6,Dok4,Tcn2,Ypel3,Pbxip1,C1qtnf6,Serpinb9b,Ulk2,Slc22a4,Os9,Akap4,Mtm1,Atp6v0b,Glo1,Rab7l1,Ptprm,Ikbkg,Chmp4c,Csrp2,Mras,Sh3tc2,Cdkn1a,Cox6a2,Hadhb,Cpeb1,Wdr7,Uchl1
**mmu-miR-29a**	44(776)	5,02E-07	Rnf13,Copg,Ccdc126,Slc37a4,Pmp22,Adc,Arvcf,Gpx4,Smpdl3a,Hexa,Sh3bgr,Habp4,Emilin2,Abcb6,Smad6,Trpv4,Tgfb2,Vps8,Ephb3,Myom2,Dok4,Tpcn1,Pdgfc,Chrd,Impdh1,Asah1,Mfap5,Ccdc80,9130011J15Rik,Ing4,Grk4,Sh3glb1,Atp6v0b,Abhd5,Klf2,Tanc2,Chmp4c,Sh3tc2,Dhps,9930013L23Rik,Gas6,Cpeb1,Tubb2a,Dusp22
**mmu-miR-152**	41(776)	5,36E-07	Dym,Snrpn,Gabarapl2,Hexa,Sh3bgr,Lamb2,Mtmr3,Sgcb,Rragc,5830405N20Rik,Gstm2,Suox,Galns,Grina,Ypel3,Pbxip1,Ascc1,Nudt7,Nrip3,Akap4,Dner,Mtm1,Gpc4,Ankrd29,Srp14,Pde4dip,Atp6v0b,Acyp2,Slc2a6,Cdh7,Mras,Sh3tc2,Stx7,Cdkn2b,Slamf9,Rhoj,1810030N24Rik,Zfp704,Dmpk,Tmem9b,1700094D03Rik
**mmu-miR-7a**	38(776)	5,54E-07	Bri3,Cat,Smpdl3a,Insig2,Lrrc51,Rab38,Sh3bgr,Atp6v1e1,Creb3,Tgfb2,Ank2,Pld1,Camk1,Sepw1,Tpcn1,Herpud2,Tcn2,Grina,Shank3,Nudt7,Slc6a8,Hbxip,Bbs5,Pcyt1a,Pltp,4930523C07Rik,Pxmp4,Atp6v0b,Slc25a39,Nr2c1,Cog6,Tpp1,Tmub2,Limch1,Uchl1,1700094D03Rik,Atp10d,Shroom3
**mmu-miR-181a-2***	36(776)	5,58E-07	Gtf2h5,Wdr47,Pmp22,Cox4nb,Tmem41a,Tmem135,Habp4,Ehd1,Fn1,Sync,F630110N24Rik,Pld1,Vps8,Qk,Calml4,Rragc,Gstm2,Galns,Nfe2l1,Riok3,Adar,Garnl3,Nkiras2,Triap1,2010111I01Rik,Isca2,Zfand2b,Tanc2,Uqcrq,Ptprm,Scarb2,Slc25a35,Tmem38a,Limch1,Aldh3a2,Shroom3
**mmu-miR-324-3p**	42(776)	5,79E-07	Mrc2,Stmn3,Hadha,Birc7,Klhl18,Hexa,Rbms2,Habp4,D16H22S680E,Rab40b,Loxl1,Kif1c,Slc7a7,Abcd1,Pld1,Cry2,Myom2,Tpcn1,Nfe2l1,Impdh1,Man2b1,Mtap6,Ascc1,Megf6,Cdk5,Rell2,Mlycd,S100a13,Ing4,S100a16,4930523C07Rik,Pxmp4,Slc41a3,Sema3f,Chmp4c,1110034G24Rik,Cox6a2,1810046J19Rik,9930013L23Rik,Igfbp2,Scn1b,Chpt1
**mmu-miR-92a***	34(776)	6,52E-07	Pank2,Ecm1,Adc,S100a1,Rab4b,Rras,Elmo2,Stam2,Clstn1,Slc44a1,Sepw1,Repin1,Gstm2,Ccdc64,Arl2,Tcn2,Grina,Shank3,Mfap5,Adar,Rell2,Os9,Ly6c1,Tollip,Pltp,Dbp,Atp6v0b,Ckm,Slamf9,Scn1b,Chst12,Slc2a8,Uchl

miRNA name	*Number of genes*	*Corrected p-value*	*miRNA targets among genes induced in Rasless cells (from Table S1)*
			1,Dmpk
**mmu-miR-125b***	35(776)	6,86E-07	Rab43,Cat,Smpdl3a,Slc22a18,Rab3d,Aldh7a1,Habp4,Krt80,Tgfb2,Arnt,Ttc15,Dok4,Tpcn1,Snta1,Cables1,Ttll7,Ccdc80,Glt8d1,Rell2,Slc22a4,Srp14,Slc26a11,1300010F03Rik,Cyb5,Tex264,Ptprm,Ikbkg,Trp53inp2,Cog6,Csrp2,1810046J19Rik,1500011K16Rik,Yif1a,Tmem9b,1700094D03Rik
**mmu-miR-680**	38(776)	7,06E-07	Ahnak2,Thra,Yif1b,Kif27,Adc,Sema3b,Nt5m,Sh3bgr,4833439L19Rik,Creb3,Cd248,Trappc6a,Slc35a2,Tmem87b,Sepw1,Ephb3,Gstm2,Ccdc64,Ndufa10,Dlg2,Wisp2,Zbtb4,Cspg4,L1cam,Pbxip1,Wwp2,P4ha2,Ppt2,Pik3ip1,Itpr2,Ly6c1,Mtcp1,Mustn1,Dbp,BC029214,Fzd8,1810046J19Rik,Mtss1
**mmu-miR-20a**	41(776)	7,38E-07	Ugp2,Kif27,Slc6a7,Adc,Crot,Smad6,Caml,Neu1,Creb3,Ank2,Armc8,2010003O02Rik,Rora,Pbxip1,Nudt7,Idua,St3gal5,Dusp8,2700078E11Rik,Serpinb9c,Serpinb9b,Mfap5,Htra3,Adamtsl5,Ing4,Ntn4,Srp14,App,Ahnak,Clip4,Rab7l1,Nr2c1,Chmp4c,Ufc1,Cbara1,Acaa1a,Mdfic,Uchl1,Zfp704,Nagk,Ankrd44
**mmu-miR-449a**	39(776)	8,95E-07	Tcta,Magi3,Fbxo9,Hexa,Rras,Mtap1a,Nudt13,Nfs1,Vps39,1110007C09Rik,Trpt1,Lyplal1,Ttc15,Suox,Galns,Arl2,Zbtb4,Tom1,Ppt2,St3gal5,Mocs2,Nrip3,Mfap5,Fabp3,Rell2,Mtcp1,4930523C07Rik,1300010F03Rik,Chid1,Acyp2,Asb1,Tex264,Cog6,Mras,Wscd2,Rhoj,Scn1b,Tubb2a,1700094D03Rik
**mmu-miR-689**	37(776)	9,68E-07	Ifi35,Thra,2310028O11Rik,Cd81,Kcnn1,Ppapdc3,Upk3b,Iqsec2,F630110N24Rik,Dusp3,Pigz,Grina,Arhgef12,Gp1bb,Ppt2,Pde6d,4930523C07Rik,Dbp,Ankrd29,Npdc1,Lrp4,Tm9sf4,Slc39a14,Sorbs3,Sirt2,Cyb5,Fzd8,Sema3f,Fbxl20,1110034G24Rik,BC004004,2900026A02Rik,Cdkn1a,Slamf9,Scn1b,Ltbp3,Dmpk
**mmu-miR-290-5p**	40(776)	9,80E-07	Crat,Zfand3,Commd8,Rab4b,Fem1a,Znrf2,Hist2h2bb,Hdlbp,Loxl1,Cd248,Slc35a2,Vps8,Btf3l4,Slc44a1,Tcn2,Zbtb4,L1cam,Man2b1,Asah1,Tmem154,Glt8d1,Dner,Plcb1,Pxmp4,Gpc4,Zfand2b,Gabarap,Mrpl55,Fzd8,Tuba8,Fbxl20,BC004004,Acyp1,Dhps,1810046J19Rik,1810030N24Rik,Slc2a8,Limch1,Shroom3,Ankrd44
**mmu-miR-133a**	39(776)	1,24E-06	Mrc2,Eif5a2,Sqrdl,Adc,Slc39a3,Rras,Clstn1,Ppapdc3,Slc35a2,F630110N24Rik,Akap13,Gramd3,Klk1b4,Txnrd3,Appl2,Guk1,Arl2,Grina,Cspg4,Ehd3,Serpinb9b,Nrip3,Ttll7,Iscu,Rell2,Garnl3,Rdh12,Slc22a4,Atg7,Ahnak,Slc2a6,Serf2,Dpm2,Csrp2,Scarb2,Gas6,Slamf9,Cacna1b,2810428I15Rik
**mmu-miR-106a**	40(776)	1,25E-06	Map3k8,Usp2,Adc,Smad6,Caml,Armc8,Myom2,2010003O02Rik,Herpud2,Ndel1,Rora,Stard6,Pbxip1,Nudt7,St3gal5,Dusp8,2700078E11Rik,Mmaa,Htra3,Ing4,Ntn4,Dbp,Galc,App,Ahnak,Clip4,Chmp4c,Ufc1,Fbxl20,Arhgap1,Cog6,Clcn7,Cbara1,Acaa1a,1500011K16Rik,Uchl1,Zfp704,Atp10d,Nagk,Ankrd44
**mmu-miR-717**	37(776)	1,39E-06	Fech,Thra,Tssc4,Stmn3,9030409G11Rik,Caml,Cd248,F630110N24Rik,Vps8,Skap2,Arnt,Slc31a2,Tpcn1,Gstm2,Guk1,Wisp2,Myeov2,Aldh6a1,P4ha2,Ehd3,Add3,Glt8d1,Tmem175,S100a16,Hgsnat,Npdc1,Mrpl55,Acyp2,Uqcrq,Sema3f,Mras,Scarb2,Rabac1,Ltbp3,Uchl1,Vps29,1700094D03Rik
**mmu-miR-188-5p**	38(776)	1,64E-06	1300014I06Rik,Ppp1r16a,Zfp78,Dtx2,Slc6a7,Tmem41a,Tlr6,Abcb6,Stam2,Loxl1,Cd248,Trappc6a,Slc7a7,Adcy9,Sgcb,Camk1,Gpr133,Cacna1g,Myo6,Suox,Arl2,Slc35b1,Lrrc57,Vdac1,Slc22a4,Itpr2,Hgsnat,Gabarap,Ahnak,Uqcrq,Cdh7,Itfg3,Ckm,Acaa1a,Afap1l2,Atp6v0a1,Limch1,Atp10d
**mmu-miR-883a-3p**	40(776)	1,76E-06	Ugp2,Snap91,Bri3,Sgca,Hadha,Adc,Tmem41a,Nt5m,Wnt9a,Rab40b,Fn1,Clstn1,Akap13,Renbp,Fstl1,BC027231,Anxa3,Skap2,Ttc15,Wisp2,Stard6,Impdh1,Mocs2,Iscu,Trak1,Naga,Ctsb,Adamts2,Atp6v0b,Sstr4,Sirt2,1110034G24Rik,Clcn7,1810046J19Rik,Acaa1a,Scarb2,Fbxw4,Rhoj,Hint3,Atp10d
**mmu-miR-302b**	38(776)	2,18E-06	Ifi35,Maob,Magi3,Glrb,Emilin2,Loxl1,Dhrs1,Ttc15,Rragc,2010003O02Rik,Dlg2,Nudt7,2700078E11Rik,Serpinb9b,9130011J15Rik,S100a13,Akap4,Triap1,Pxmp4,Impact,1810037I17Rik,Clip4,Rab7l1,Synpo2,Nr2c1,Ikbkg,Ufc1,BC004004,Csrp2,Mras,Sh3tc2,Cdkn1a,Tpp1,Wdr7,Uchl1,Zfp704,1700094D03Rik,Ankrd44
**mmu-miR-291a-3p**	40(776)	2,30E-06	Camk4,Magi3,Crot,Smad6,Loxl1,Crybb3,Dab2ip,Ttc15,Txnrd3,Dlg2,Arhgef12,Nudt7,St3gal5,2700078E11Rik,Serpinb9b,Ehd2,Glt8d1,9130011J15Rik,Ing4,Tollip,Impact,Lrp4,Ahnak,Clip4,Rab7l1,Uqcrq,Nr2c1,Ikbkg,Ufc1,Csrp2,Sh3tc2,Acaa1a,Nhlrc1,Wdr7,Uchl1,Zfp704,Immp1l,Atp10d,Mtus1,Ankrd44
**mmu-miR-224**	38(776)	2,36E-06	Rnf13,Adc,Fbxo9,Smpdl3a,Alg14,Slc22a18,Ehd1,Wnt9a,Dscr3,Kcnn1,Nfs1,Ank2,Mrgprf,Tmco3,Qk,Atp2a3,Nipa1,Ypel3,L1cam,Idua,St3gal5,Itpr2,Apba1,Plcb1,Mtm1,Gpc4,Atp6v0b,Rab7l1,Kif13b,Ikbkg,Hck,Ssfa2,Csrp2,Cdkn1a,Dusp22,Tmem9b,Atp10d,Aldh3a2
**mmu-miR-532-3p**	39(776)	2,36E-06	Ppp1r16a,Mrc2,Maob,Adra1b,Usp2,Kif27,Slc6a7,Dhrs3,Selm,Rras,Clstn1,Prss36,Upk3b,Mtmr3,Tgfb2,Crybb3,F630110N24Rik,Pld1,Tmem87b,Ptplad2,Arnt,Ankra2,Slc31a2,Cacna1g,Lgals9,Zbtb4,Slc9a6,St3gal5,Cdk5,Atg4a,Npdc1,App,Spata21,Hck,Acaa1a,Wscd2,Scn1b,Chst12,Aph1a
**mmu-miR-351**	37(776)	2,36E-06	Ppp1r16a,Pank2,Oit3,Stmn3,Plb1,Kif27,Cd81,Abcb6,Dusp3,Adcy9,1700009P17Rik,Slc46a3,Ypel3,Shank3,Rapsn,Glt8d1,Psen2,Mlycd,Ing4,Dopey2,Pxmp4,Ankrd29,Npdc1,Lrp4,BC029214,Mrpl55,Kif13b,Synpo2,Ikbkg,Hck,Itfg3,Sh3tc2,Slc25a35,Slamf9,Fbxw4,Ltbp3,Atp10d
**mmu-miR-665**	36(776)	2,89E-06	Stmn3,Slc6a7,Prss29,Rras,Dscr3,Krt80,Klf15,Slc35a2,Pld1,Sepw1,Cacna1g,Lgals9,Hsd3b3,Wisp2,Nfe2l1,Pbxip1,Snta1,Ascc1,Deb1,Adar,Akap4,Ahnak,Tex264,Htatip2,Itfg3,Arhgap1,Sparc,Mras,Cox6a2,Ckm,Mtss1,Scarb2,Tom1l2,Atp5e,Chst12,Slc2a8

miRNA name	Number of genes	Corrected p-value	miRNA targets among genes induced in Rasless cells (from Table S1)
**mmu-miR-125a-5p**	40(776)	2,96E-06	Ppp1r16a,Pank2,Plb1,Kif27,Kremen1,Rab38,Upk3b,Vps39,Mtmr3,Tgfb2,Amhr2,Akap13,Adcy9,Dgkg,Klk1b4,Dhrs1,Slc46a3,Gpr133,Grina,Shank3,Tom1,Rapsn,P4ha2,Capn5,Rell2,Vnn1,Zfand2b,Slc2a6,Ikbkg,Hck,Itfg3,Acyp1,Sh3tc2,Slc25a35,Fbxw4,Tmem38a,1700021C14Rik,Pcsk6,Ltbp3,Mtus1
**mmu-miR-345-5p**	37(776)	3,06E-06	Mrc2,Zfand3,Thra,Tssc4,Sgca,Kif27,Pef1,Slc6a7,Tcta,Magi3,Dhrs3,Atp6v1e1,Tor3a,Ptplad2,Mxra8,Ttc15,Galns,Farp2,Chrd,Shank3,4933439F18Rik,Nfe2l1,Cspg4,Snta1,Idua,Adar,S100a16,Plcb1,Tm9sf4,Nr2c1,Ufm1,Cbara1,9930013L23Rik,Slamf9,Scn1b,Cacna1b,Npc1
**mmu-miR-764-5p**	38(776)	3,06E-06	Ahnak2,Plb1,Ascc2,Hexa,Elmo2,Sync,Renbp,Armc8,Pigz,Slc46a3,Arnt,2900010M23Rik,Pdgfc,Trim36,Arl2,Tcn2,Snta1,Htra3,Tmem175,Slc22a4,S100a13,Akap4,Galc,1300010F03Rik,Pdia5,Clip4,Rab7l1,Hck,Chmp4c,Clcn7,Cox6a2,Tom1l2,Igfbp2,Tmub2,Limch1,Pcsk6,Uchl1,Atp10d
**mmu-miR-18b**	38(776)	3,18E-06	Ccdc126,Cox4nb,Ascc2,Cyp27a1,Rras,Wnt9a,D16H22S680E,Inpp5a,AI593442,Smad6,Myadm,Tbcd,Akap13,Slc7a7,Clip1,Fbxl2,Synpo,Herpud2,Arhgef12,Slc35b1,Dusp8,Glt8d1,Rell2,Adamtsl5,Ctsb,Abtb2,Necap2,Srp14,Ikbkg,Ufc1,1110034G24Rik,Cpa6,Itfg3,Acaa1a,Cpeb1,Uchl1,Yif1a,Nagk
**mmu-let-7d***	37(776)	3,19E-06	Pank2,Zfand3,Dtx2,Iah1,Col14a1,Sgca,Slc37a4,Sh3bgr,Abcb6,AI593442,Caml,Atp9b,Renbp,Slc7a7,Pld1,Btf3l4,Myom2,Synpo,Gdf15,Mtap6,Pon3,Fabp3,Psen2,Trak1,Plcb1,Ctsl,App,Tbccd1,Ssbp2,Sema3f,Ufc1,Igfbp2,Esrrb,Rit1,Wdr7,Dusp22,Zfp704
**mmu-miR-200a**	40(776)	3,19E-06	Ugp2,Lgr4,Gyg,Crot,Rab38,Fn1,Cyp4v3,Upk3b,Lamb2,Vps39,Akap13,Lyplal1,Lmbr1,Tmco3,Riok3,P4ha2,Ppt2,Ascc1,Nudt7,St3gal5,Lrrc57,2700078E11Rik,Nrip3,Ppm1h,Ccdc80,Slc35c2,Pltp,1300010F03Rik,Tanc2,Cyb5,6530401N04Rik,Asb1,Ssfa2,1810046J19Rik,Daglb,9930013L23Rik,Wdr7,1700094D03Rik,Npc1,Chpt1
**mmu-miR-96**	38(776)	3,20E-06	Ahnak2,Rpl17,Pef1,Magi3,Insig2,Elmo2,Abcb6,Neu1,Cd248,Tgfb2,Lyplal1,Anxa3,Myom2,Lnx1,Gp1bb,Ccdc80,Garnl3,Itpr2,Ntn4,Vnn1,Pltp,Ctsb,Ankrd29,Cryab,Tm9sf4,Tsc22d3,Zfand2b,Ahnak,Mrpl55,Cyb5,Slc2a6,Vps24,1110034G24Rik,Igbp1,Cpeb1,C1qtnf1,Hhip,Ankrd44
**mmu-miR-196a**	39(776)	3,25E-06	Pank2,Crat,Ahnak2,Dtx2,Col14a1,Mpv17,Trpc7,Habp4,Fn1,Atp6v1e1,Dynlrb1,Gng5,Lmbr1,Lama2,Alkbh6,Ttc15,2900010M23Rik,Suox,Arl2,Slc9a6,Myeov2,Bphl,Riok3,Dusp8,Ergic3,Cdk5,Deb1,Anxa1,Def8,Ahnak,Ugt2b36,Uqcrq,Glipr1,Cpa6,Csrp2,Mras,Tyrp1,1810046J19Rik,Hint3
**mmu-miR-29c**	41(776)	3,39E-06	Copg,Ccdc126,Slc37a4,Pmp22,Adc,Arvcf,Smpdl3a,Hexa,Sh3bgr,Habp4,Emilin2,Abcb6,Smad6,Trpv4,Tgfb2,Vps8,Ephb3,Dok4,Tpcn1,Pdgfc,Chrd,Impdh1,Ascc1,Mfap5,Ccdc80,Fabp3,9130011J15Rik,Ing4,Grk4,Sh3glb1,Adamts2,Abhd5,Klf2,Chmp4c,Sh3tc2,Dhps,9930013L23Rik,Gas6,Cpeb1,Tubb2a,Dusp22
**mmu-miR-466c-5p**	44(776)	3,65E-06	Adra1b,Copg,Usp2,Plb1,Ecm1,Snrpn,Coq10a,Fbxo9,Insig2,Nt5m,Elmo2,Habp4,Sync,Lamb2,Amhr2,Akap13,Slc46a3,Bmp4,Calml4,Lgals9,Suox,Wisp2,Zbtb4,Man2b1,2700078K21Rik,Nrip3,Hectd3,C330006K01Rik,Rdh12,Slc22a4,Ly6c1,Nol3,Plcb1,Mtm1,Gabarap,Il17d,Ssbp2,Tuba8,ORF63,Acyp1,Mras,Kif3a,Daglb,Tpp1
**mmu-miR-205**	35(776)	4,08E-06	Acp2,Tssc4,Rab43,Magi3,Gabarapl2,Adamts5,AI593442,Prss36,F630110N24Rik,Slc7a7,Abcd1,1700009P17Rik,Ttc15,Rora,C1qtnf6,Mtap6,Mocs2,Lrrc57,Mfap5,Lims2,Itpr2,Naga,BC089491,Nenf,Plcb1,Abtb2,Grk4,Mrpl55,4930579G22Rik,Fbn2,Mras,Ckm,Igfbp2,Camkk1,Tmem38a
**mmu-miR-93**	38(776)	4,35E-06	Thra,Kif27,Adc,Tmem41a,Atp6v0e,Smad6,Cyp4v3,Caml,Neu1,Loxl1,Myom2,2010003O02Rik,Pbxip1,Idua,St3gal5,Tmem154,Adamtsl5,Ntn4,Mtm1,Srp14,1300010F03Rik,Gabarap,Ahnak,Clip4,Synpo2,Chmp4c,Ufc1,Arhgap1,Csrp2,Cbara1,Cdkn1a,Acaa1a,Nhlrc1,1500011K16Rik,Uchl1,Zfp704,Nagk,Ankrd44
**mmu-miR-151-5p**	37(776)	4,37E-06	Thra,Dtx2,Col14a1,Usp2,Ecm1,S100a1,Rbms2,Slc16a9,Clstn1,Trpv4,Nudt13,Iqsec2,Dynlrb1,Gramd3,Rragc,Sepw1,Itgb1bp1,Cspg4,Snta1,Nudt7,Dusp8,Pon3,Fabp3,Serinc3,Ing4,Pde6d,Sorbs3,Clip4,Asb1,Plac9,Nr2c1,Vps24,Clcn7,Slamf9,Chst12,C1qtnf1,Zfp704
**mmu-miR-743a**	36(776)	4,80E-06	Iah1,Stmn3,Cat,Lgr4,Ascc2,Insig2,Sh3bgr,Iqsec2,Dynlrb1,Ankra2,Npy6r,Myom2,2010003O02Rik,Ctso,Qpct,Stard6,Alas1,P4ha2,Ascc1,Idua,St3gal5,Ulk2,Vnn1,Grk4,Slc26a11,1700016M24Rik,Tanc2,Uqcrq,Ikbkg,Vps24,Chmp4c,Mras,Cpeb1,Rabac1,Cox6b1,Zfp704
**mmu-miR-574-5p**	36(776)	5,07E-06	Dym,Yif1b,Copg,Plb1,Snrpn,Mpv17,Sh3bgr,D16H22S680E,Emilin2,Creb3,Iqsec2,Vps8,Dhrs1,Lama2,Calml4,Atp2a3,Tpcn1,Dlg2,Gdf15,Man2b1,Nrip3,Lrch1,Rdh12,Tollip,Sh2d4a,Pxmp4,Grk4,1300010F03Rik,Pdia5,Pcbd2,Hck,ORF63,Cox6a2,Kif3a,Lrsam1,Immp1l
**mmu-miR-466d-5p**	44(776)	6,54E-06	1300014I06Rik,Dym,Adra1b,Copg,Usp2,Plb1,Ecm1,Snrpn,Fbxo9,Alg14,Nt5m,Elmo2,Emilin2,Sync,Tbcd,1110007C09Rik,Armc8,Skap2,Bmp4,Calml4,Lgals9,Suox,Zbtb4,Man2b1,2700078K21Rik,Nrip3,Mmaa,Tmem175,C330006K01Rik,Adamtsl5,Ly6c1,Tollip,Plcb1,0610037L13Rik,Tsc22d3,Il17d,Ssbp2,Hck,Ufm1,Mras,Kif3a,Tpp1,Tbc1d9b,Mtus1
**mmu-miR-138**	37(776)	6,95E-06	Pank2,Zfp78,Kremen1,Gyg,Magi3,Gpx4,Fbxo9,Fem1a,Ehd1,Clstn1,Prss36,Crybb3,F630110N24Rik,Dgkg,9430038I01Rik,Cacna1g,Arl2,Man2b1,Fabp3,Slc6a8,Ing4,Tollip,Pxmp4,Mtm1,Synpo2,Tex264,Vps24,Tuba8,Csrp2,Cox6a2,Ckm,Igfbp2,Gas6,Wscd2,1700021C14Rik,Pcsk6,2810428I15Rik

miRNA name	*Number of genes*	*Corrected p-value*	*miRNA targets among genes induced in Rasless cells (from Table S1)*
**mmu-miR-7b**	35(776)	7,22E-06	Gtf2h5,Wdr47,Bri3,Cat,Rpl17,Pef1,Ascc2,Klhl18,Smpdl3a,Insig2,Lrrc51,Sh3bgr,Creb3,Tgfb2,Ank2,Pld1,Herpud2,Tcn2,Grina,Shank3,Nudt7,Slc6a8,Hbxip,Vdac1,Bbs5,Pltp,4930523C07Rik,Pxmp4,Atp6v0b,Slc25a39,Nr2c1,Sparc,Tmub2,Uchl1,Atp10d
**mmu-miR-449b**	36(776)	7,46E-06	Ahnak2,Col14a1,Tcta,Arvcf,Fbxo9,Hexa,Rras,D16H22S680E,Stk39,Sync,Nfs1,Vps39,Vps8,Slc31a2,Ttc15,Ccdc64,Suox,Arl2,Zbtb4,Tom1,Bphl,Serpinb9c,Nrip3,Lims2,Tmem175,Rell2,Mtcp1,Plcb1,Ankrd29,Pde4dip,Def8,Acyp2,Tex264,Cog6,Sh3tc2,Wscd2
**mmu-miR-148b**	38(776)	7,58E-06	Mcee,Plb1,Gabarapl2,Sh3bgr,Klf15,Ank2,Sgcb,5830405N20Rik,Myom2,Txnrd3,Grina,Ypel3,Pbxip1,Ascc1,Nudt7,Idua,Nrip3,Akap4,Gpc4,Ankrd29,Pde4dip,Acyp2,Il17d,Slc2a6,Cdh7,Mras,Sh3tc2,Ckm,Cdkn2b,Slamf9,Rhoj,1810030N24Rik,Tmem50b,Zfp704,Dmpk,Tmem9b,1700094D03Rik,3110002H16Rik
**mmu-miR-466h**	44(776)	9,86E-06	Yif1b,Copg,Usp2,Mcee,Plb1,Ecm1,Rpl17,Snrpn,Zdhhc4,Fbxo9,Alg14,Elmo2,Atp6v0e,Lamb2,D6Wsu163e,Crybb3,Akap13,Armc8,Anxa3,Slc46a3,Calml4,Tpcn1,Man2b1,2700078K21Rik,Nrip3,Hectd3,Tmem175,C330006K01Rik,Slc22a4,Itpr2,Ntn4,Vnn1,Sned1,Slc26a11,Pcbd2,Ssbp2,Serf2,Gpr39,Cox6a2,Tom1l2,Tpp1,Igbp1,Tbc1d9b,Cyb5r3
**mmu-miR-106b**	36(776)	1,09E-05	Adc,Crot,Smad6,Loxl1,Akap13,Myom2,2010003O02Rik,Rora,Stard6,Pbxip1,Nudt7,Idua,St3gal5,Dusp8,Serpinb9b,Bbs5,Ntn4,App,Ahnak,Clip4,Fzd8,Chmp4c,Ufc1,Arhgap1,Cog6,Csrp2,Clcn7,Cbara1,1810046J19Rik,Acaa1a,1500011K16Rik,Uchl1,Zfp704,Atp10d,Nagk,Ankrd44
**mmu-miR-714**	36(776)	1,09E-05	Stmn3,Plb1,Prss29,Nt5m,Ehd1,Emilin2,Ppapdc3,Lamb2,Iqsec2,Slc35a2,Dhrs1,Sepw1,Tpcn1,Gstm2,Ypel3,Zbtb4,Myeov2,C1qtnf6,Snta1,Slc35c2,Adamtsl5,Ly6c1,Bbs5,Ing4,Dbp,Necap2,Adamts2,Tex264,Slc25a39,Hr,Tuba8,Adamtsl4,Atp5e,Rabac1,1700094D03Rik,Aph1a
**mmu-miR-695**	34(776)	1,10E-05	Zfp78,S100a1,Rab4b,Sema3b,Slc22a18,Caml,Vps8,Lyplal1,Pigz,9430038I01Rik,Ap3m2,Dph1,Guk1,Itgb1bp1,Shank3,Impdh1,Slc35b1,Garnl3,9130011J15Rik,Rdh12,Plcb1,Pltp,Srr,Frmpd1,1300010F03Rik,Mrpl55,Il17d,1810027O10Rik,Adamtsl4,Mtss1,Rhoj,Atp6v0a1,Shroom3,Ankrd44
**mmu-miR-700**	33(776)	1,23E-05	Ap3s1,Yif1b,Usp2,Cyp27a1,Hexa,Atp6v0e,Cd81,Loxl1,Nfs1,Dusp3,Copz2,Renbp,Adcy9,Qk,Sepw1,Trappc2l,Grina,Myeov2,Ergic3,Iscu,Zdhhc8,Psen2,2310046K01Rik,Dbp,Gpc4,Npdc1,Rab7l1,Kif13b,Nhlrc1,2610019F03Rik,Agl,Uchl1,Dmpk
**mmu-miR-202-3p**	34(776)	1,27E-05	Dtx2,Adra1b,Slc37a4,S100a1,Tmem41a,Smpdl3a,Sh3bgr,Rras,Caml,Tgfb2,Dusp3,Tbcd,Vps8,Gramd3,Tmem87b,Gstm2,Hsd3b3,Tom1,P4ha2,Lims2,Atg7,Pde6d,Ctsb,Sstr4,Slc2a6,Hck,Tuba8,Htatip2,Cpa6,Arhgap1,1810046J19Rik,Tpp1,Slamf9,Uchl1
**mmu-miR-331-3p**	33(776)	1,37E-05	Pank2,Gtf2h5,Usp2,Sgca,Mal,Sema3b,Hexa,Ppp2r5b,F630110N24Rik,Adcy9,Mrgpre,Synpo,Ypel3,Shank3,Nfe2l1,Cspg4,Impdh1,L1cam,Rell2,S100a16,Pde6d,Hgsnat,Sirt2,Synpo2,Mxra7,Ikbkg,Gpr39,Sh3tc2,Cbara1,Camkk1,Uchl1,Zfp704,Yif1a
**mmu-miR-466f-5p**	44(776)	1,44E-05	Pank2,Zfand3,Adra1b,Copg,Usp2,Mcee,Mpv17,Fbxo9,Alg14,Insig2,Lrrc51,Elmo2,D16H22S680E,R74862,Lamb2,D6Wsu163e,Creb3,Amhr2,Akap13,Armc8,Slc46a3,5830405N20Rik,Lgals9,Ccdc64,2700078K21Rik,St3gal5,Nrip3,Deb1,Ly6c1,Ing4,Plcb1,Tsc22d3,Ssbp2,Hr,Serf2,Acyp1,2900026A02Rik,Dnajc4,Daglb,Tom1l2,Igfbp2,Lrsam1,Limch1,1700094D03Rik
**mmu-miR-20b**	37(776)	1,46E-05	Map3k8,Camk4,Adc,Smad6,Caml,Neu1,Creb3,Tbcd,Pbxip1,Nudt7,St3gal5,Dusp8,2700078E11Rik,Serpinb9c,Serpinb9b,Htra3,Adamtsl5,Ing4,Ntn4,Dbp,Srp14,1300010F03Rik,App,Ahnak,Clip4,Synpo2,Chmp4c,Ufc1,Arhgap1,Cbara1,Acaa1a,Mdfic,Uchl1,Zfp704,Atp10d,Nagk,Ankrd44
**mmu-let-7i**	39(776)	1,50E-05	Dtx2,Col14a1,Mcee,Slc37a4,Coq10a,Tlr6,Nt5m,Sh3bgr,Rras,Elmo2,R74862,Cyp4v3,Trappc6a,Gng5,Lyrm1,Lama2,Skap2,Ankra2,Itgb1bp1,Chrd,Lnx1,Slc35b1,Ascc1,Ttll7,Fabp3,Rell2,Abtb2,Mtm1,Gpc4,Srp14,Isca2,BC029214,App,Def8,1110034G24Rik,6330406I15Rik,Acaa1a,C1qtnf1,Vps29
**mmu-miR-532-5p**	33(776)	1,61E-05	Thra,Ap3s1,Adra1b,Rab43,Plb1,Arvcf,Sh3bgr,Elmo2,AI593442,Cyp4v3,Trappc6a,Rhbdd1,Cry2,1700009P17Rik,Trappc2l,2700078K21Rik,Slc35c2,Ly6c1,Ing4,Pde6d,Ctsb,Atp6v0b,Atg12,Sirt2,Spata21,Bcmo1,Atp5s,Ikbkg,Vps24,Slc41a2,1810030N24Rik,Yif1a,Atp10d
**mmu-miR-341**	33(776)	1,82E-05	Acp2,Yif1b,Usp2,Mcee,Kremen1,Magi3,Dhrs3,Selm,Elmo2,Wnt9a,Clstn1,Lamb2,Vps39,Tgfb2,Dab2ip,Vps8,Anxa3,Hexb,Riok3,P4ha2,Snta1,Ascc1,Garnl3,Mtcp1,Abtb2,Rab7l1,Asb1,Ptprm,Slc25a35,Rit1,C1qtnf5,Ltbp3,Aldh3a2
**mmu-miR-328**	32(776)	1,82E-05	Dym,Sgca,Rab43,Cobl,Ehd1,Cd81,Atp6v1e1,Trappc6a,Kif1c,F630110N24Rik,Clip1,Ephb3,Tcn2,Shank3,Nfe2l1,Cspg4,Rapsn,Nupr1,Vnn1,BC089491,Mtcp1,Dbp,Zfand2b,Spata21,Il17d,Sema3f,Gpr39,Tom1l2,9930013L23Rik,Cdkn2b,Gas6,Lmna
**mmu-miR-130a**	37(776)	1,84E-05	Dym,Dtx2,Map3k8,Ubl4,Sh3bgr,Habp4,Cd81,Stk39,Gadd45b,Atp9b,D6Wsu163e,Mrgprf,Sgcb,Ankra2,Myom2,Nipa1,Ensa,Slc9a6,Bbs5,Reep3,Akap4,Plcb1,Mtm1,Ankrd29,Srp14,Adamts2,Tanc2,Acyp2,Ptprm,Hadhb,Tpp1,9930013L23Rik,Tbc1d9b,Vps29,Tmem9b,Ankrd44,3110002H16Rik

miRNA name	*Number of genes*	*Corrected p-value*	*miRNA targets among genes induced in Rasless cells (from Table S1)*
**mmu-miR-691**	33(776)	1,89E-05	Oit3,Copg,Alg14,Hist2h2bb,Trpv4,Amhr2,Akap13,Vps8,Gramd3,1700009P17Rik,Anxa3,5830405N20Rik,Repin1,2010003O02Rik,Rora,Mocs2,Htra3,Deb1,Glt8d1,Psen2,Nkiras2,Vnn1,Sned1,Triap1,Pltp,Gabarap,Kif13b,Nr2c1,Ufc1,Hadhb,Slc2a8,Tubb2a,Ankrd44
**mmu-miR-467e**	36(776)	1,98E-05	Fech,Cat,Kif27,Commd8,Birc7,Zdhhc4,Gyg,Sh3bgr,Rras,Atp6v0e,Cd81,Dscr3,Sync,Creb3,Calml4,Ttc15,Repin1,Tpcn1,Grina,Farp2,Dlg2,Lnx1,Slc35b1,Nudt7,Serpinb9b,Grik2,Cdk5,Akap4,Dbp,Atg12,Ikbkg,4930579G22Rik,Stx7,Ankrd12,Uchl1,Lrpap1
**mmu-miR-30c-2***	34(776)	1,99E-05	Copg,Kif27,Ascc2,S100a1,Fahd2a,Smpdl3a,Clstn1,Prss36,Ppapdc3,Copz2,Rarres2,Clip1,Dhrs1,Tmco3,Arnt,Arl2,Man2b1,Ppt2,Ccdc80,Ly6c1,Pde6d,Plcb1,Ctsb,Dbp,Nr2c1,Mras,1810046J19Rik,Tom1l2,Slamf9,Atp6v0a1,1810030N24Rik,Lmna,Cacna1b,1700094D03Rik
**mmu-miR-346**	31(776)	2,04E-05	Dym,Tssc4,Adra1b,Sgca,Slc37a4,Adc,Fahd2a,Selm,Insig2,Sema3b,Hexa,Ank2,Slc35a2,F630110N24Rik,Dusp3,Trpt1,1700009P17Rik,Ephb3,Ndel1,Idua,Anxa1,Galnt2,S100a13,Nupr1,Trak1,Ctsb,Zfand2b,Cox6a2,Atp5e,Tmem38a,Aph1a
**mmu-miR-149**	33(776)	2,07E-05	Mrc2,Thra,Tssc4,Sgca,Mtap1a,Cd81,Gadd45b,Nudt13,Dab2ip,Akap13,Renbp,Vps8,Dok4,Rasl12,P4ha2,Adar,Slc35c2,Vdac1,S100a13,Itpr2,Nupr1,Pde4dip,Klf2,Il17d,Kif13b,Tex264,Slc25a39,Hck,1810027O10Rik,Gpr39,BC004004,Tom1l2,Lrsam1
**mmu-miR-153**	34(776)	2,11E-05	Ppp1r16a,Sgca,Plb1,Crot,Smpdl3a,Rbms2,Sh3bgr,D6Wsu163e,Dusp3,Dab2ip,Armc8,Gramd3,Slc46a3,Qk,Ankra2,Dlg2,Bphl,Idua,Lrrc57,Lrch1,Tmem175,Fabp3,Mtcp1,Grk4,Srp14,Atp6v0b,App,Uqcrq,Slc2a6,Chmp4c,Igbp1,Fzd6,Satb1,Ankrd44
**mmu-miR-486**	36(776)	2,15E-05	Birc7,Tmem41a,Higd1a,Elmo2,Abcb6,Sync,Vps39,Slc35a2,Amhr2,Fstl1,Armc8,Tmem87b,Ankra2,Tob1,Ndel1,Itga9,Tom1,C330006K01Rik,Atg4a,Rdh12,5530400B01Rik,Tollip,Abtb2,Gpc4,Slc26a11,1300010F03Rik,Atp6v0b,Tbccd1,Cdh7,Cog6,Cbara1,Igfbp2,Slamf9,1500011K16Rik,Chst12,1700094D03Rik
**mmu-miR-28**	32(776)	2,28E-05	Sgca,Stmn3,Kif27,Mal,Tmem41a,Rbms2,Elmo2,Lamb2,Iqsec2,Dynlrb1,Lmbr1,Clip1,Arnt,Mxra8,Sepw1,Qpct,Ndel1,Itgb1bp1,Ppt2,Nudt7,Cdk5,S100a16,Ahnak,Clip4,Rab7l1,Gstp1,1110034G24Rik,Mras,Fbxw4,Slc2a8,Pcsk6,Aldh3a2
**mmu-miR-25**	37(776)	2,29E-05	Ugp2,Sgca,Lgr4,Adamts5,Smad6,Serpinb6c,Slc35a2,Dgkg,Gramd3,Anxa3,Slc31a2,Ap3m2,Tob1,Herpud2,Riok3,Asah1,Mfap5,Ccdc80,Tmem154,Ing4,4930523C07Rik,Mtm1,2010111I01Rik,Sirt2,Klf2,Cxcr7,Hck,Dpm2,Fbn2,Cbara1,Dnajc4,Cdkn2b,Igbp1,1700021C14Rik,Mdfic,Dusp22,Atp10d
**mmu-miR-429**	35(776)	2,29E-05	Ugp2,Plb1,Atp6v0e,Krt80,Stam2,Fn1,Serpinb6c,Slc35a2,Akap13,Lmbr1,Tpcn1,Nipa1,Nudt7,Dusp8,Nrip3,Ccdc80,Tmem154,Ly6c1,Plcb1,Pltp,Ahnak,Rab7l1,6530401N04Rik,Slc25a39,Sema3f,Hck,Acyp1,Slamf9,1500011K16Rik,Reck,Tubb2a,Npc1,Mtus1,Ankrd44,Chpt1
**mmu-miR-200a***	29(776)	2,56E-05	Gtf2h5,Kif27,Magi3,Coq10a,Zhx1,Insig2,Fn1,Cyp4v3,Sgcb,Lyrm1,Tmco3,Repin1,Rab3gap2,Stard6,Man2b1,Ascc1,Nudt7,Tmem175,Exoc3,Tanc2,Spata21,Rab7l1,Usp53,Htatip2,Arhgap1,Tom1l2,Wdr7,Chst12,Atp10d
**mmu-miR-301b**	35(776)	2,67E-05	Dym,Ubl4,Sh3bgr,Habp4,Atp9b,Loxl1,D6Wsu163e,Adcy9,1810020D17Rik,Mrgprf,Sgcb,Ankra2,Ttc15,2010003O02Rik,Tob1,Dlg2,2310046K01Rik,Akap4,Hscb,Adamts2,1810037I17Rik,Pdia5,Sstr4,Acyp2,Ptprm,Chmp4c,Itfg3,Slamf9,1500011K16Rik,Cpeb1,Tbc1d9b,Vps29,Tmem9b,Ankrd44,3110002H16Rik
**mmu-miR-540-3p**	38(776)	2,70E-05	Ppp1r16a,Gtf2h5,Zfand3,Stmn3,Kif27,Ascc2,Adc,Dhrs3,Trpc7,Fn1,Crybb3,Copz2,Akap13,Pigz,Slc46a3,Camk1,Sepw1,Guk1,Tcn2,Ndufa10,Itgb1bp1,Impdh1,P4ha2,Megf6,Adamtsl5,Mlycd,Trak1,Naga,Pde6d,Slc26a11,1300010F03Rik,1700016M24Rik,Slco3a1,Slc25a39,Hck,Tuba8,Ufc1,Cdh7
**mmu-miR-187**	32(776)	2,72E-05	Stmn3,Slc37a4,Commd8,Ehd1,Emilin2,Nudt13,Upk3b,Loxl1,Slc35a2,F630110N24Rik,Dusp3,Akap13,Slc7a7,Gramd3,Ttc15,Qpct,Shank3,Myeov2,Mfap5,Atg7,BC089491,Slc39a14,Klf2,Ptprm,Hck,Cog6,Cox6a2,Ckm,Kif3a,Tom1l2,Gas6,Dusp22
**mmu-miR-412**	30(776)	2,72E-05	Hadha,Tcta,Selm,Alg14,Klf15,Akap13,Tmem87b,Clip1,Dhrs1,Ephb3,Tpcn1,Arl2,Itgb1bp1,Wisp2,Rora,Shank3,Nfe2l1,Ppm1h,Chid1,Kif13b,Slc25a39,Sema3f,Sh3tc2,Gas6,Fbxw4,Cpeb1,Cyb5r3,Slc2a8,Tmem9b,Aldh3a2
**mmu-miR-201**	32(776)	2,73E-05	Cyp27a1,Sh3bgr,Stam2,Dynlrb1,F630110N24Rik,Rarres2,Klk1b4,1700009P17Rik,Dhrs1,Myom2,Gstk1,Lnx1,Ascc1,Dusp8,Lrrc57,2700078E11Rik,Iscu,Ppm1h,Ulk2,Mlycd,Itpr2,Ctsl,Def8,Acyp2,Kif13b,Ptdss1,Fbxl20,Slc25a35,1810030N24Rik,1700021C14Rik,Hint3,Aldh3a2
**mmu-miR-711**	31(776)	2,74E-05	Rab4b,Tlr6,Rras,Napb,Clstn1,Loxl1,Cdc42ep2,Slc44a1,Gstm2,Grina,Zbtb4,Impdh1,Aldh6a1,Ppt2,Htra3,Adar,2310046K01Rik,Ing4,Slc26a11,Tm9sf4,Klf2,Tanc2,Rab7l1,Synpo2,Cox6a2,Ckm,Stx7,Slc25a35,Rabac1,Atp10d,Chpt1
**mmu-miR-22**	35(776)	2,77E-05	Thra,Tssc4,Commd8,Hspa12a,Camk4,Ascc2,Sema3b,Rab3d,Nudt13,Ppp2r5b,Dynlrb1,Ank2,Slc7a7,Vps8,Arhgef12,Nfe2l1,Cspg4,C1qtnf6,Nudt7,St3gal5,Lrrc57,Htra3,Ccdc80,Lrch1,S100a16,Abtb2,2010111I01Rik,Slc39a14,Zdhhc24,Ufc1,Htatip2,Gas6,Slc2a8,Tmem9b,Npc1

miRNA name	*Number of genes*	*Corrected p-value*	*miRNA targets among genes induced in Rasless cells (from Table S1)*
**mmu-miR-296-5p**	35(776)	2,79E-05	Mrc2,Ahnak2,Thra,Stmn3,Pef1,Rab4b,Clstn1,AI593442,Ppapdc3,Trpv4,Upk3b,Klf15,Neu1,Iqsec2,Kif1c,F630110N24Rik,Trpt1,Dhrs1,Dusp8,Adar,Ing4,Atg7,S100a16,BC089491,Zfand2b,Sirt2,Ikbkg,Sema3f,Dpm2,Htatip2,Stxbp1,Cyb5r3,Ltbp3,Cacna1b,Aph1a
**mmu-miR-301a**	35(776)	2,79E-05	Dym,Ubl4,Sh3bgr,Elmo2,Atp9b,Loxl1,D6Wsu163e,Adcy9,1810020D17Rik,Btf3l4,Sgcb,Ankra2,Ttc15,2010003O02Rik,Suox,Tob1,Dlg2,Akap4,Adamts2,1810037I17Rik,Pdia5,Sstr4,Acyp2,Ptprm,Chmp4c,Itfg3,Slamf9,1500011K16Rik,Cpeb1,Tbc1d9b,Agl,Vps29,Tmem9b,Ankrd44,3110002H16Rik
**mmu-miR-590-5p**	34(776)	2,81E-05	Fech,Ugp2,Kif27,Rras,Prss36,Adcy9,Gramd3,Lmbr1,2010003O02Rik,Farp2,Arhgef12,Gdf15,Slc9a6,2700078K21Rik,Bphl,Ascc1,Nudt7,Lrrc57,Anxa1,Bbs5,Hscb,Dbp,Mtm1,Ssbp2,Dnalc1,1110034G24Rik,Acyp1,Cbara1,Reck,1810030N24Rik,Rabac1,Satb1,Uchl1,Zfp704
**mmu-miR-542-3p**	34(776)	2,91E-05	Mrc2,Col14a1,Stmn3,Rab4b,Fbxo9,Rab38,Fem1a,Emilin2,Iqsec2,Tbcd,Slc7a7,Mxra8,Txnrd3,Tpcn1,Trim36,Arl2,Nudt7,Lrrc57,Tmem175,Adamtsl5,Grk4,Gabarap,Mrpl55,Pcbd2,Chchd6,Uqcrq,Ufc1,Fbxl20,Htatip2,Tom1l2,Rabac1,Tubb2a,Uchl1,Yif1a
**mmu-miR-141**	34(776)	2,93E-05	Ugp2,Snap91,Tssc4,Sgca,Gyg,Rab38,Fn1,Upk3b,Vps39,Akap13,Lyplal1,Tmco3,Calml4,Fbxl2,Pdgfc,Riok3,P4ha2,Ppt2,Nudt7,St3gal5,Lrrc57,Ccdc80,Slc35c2,Pltp,Tanc2,Cyb5,6530401N04Rik,1810046J19Rik,Hadhb,9930013L23Rik,Tbc1d14,Wdr7,1700094D03Rik,Chpt1
**mmu-miR-467c**	37(776)	2,95E-05	Kif27,Mpv17,Trpc7,BC024814,Selm,Cyp27a1,Aldh7a1,Cd81,Stam2,Smad6,Cyp4v3,Caml,D6Wsu163e,Pld1,Armc8,Skap2,Calml4,Myom2,Tpcn1,Dlg2,Ypel3,Pbxip1,C1qtnf6,Nudt7,Dusp8,Serpinb9b,Pde6d,Mtm1,Gpc4,Pde4dip,Mmab,Gabarap,4930579G22Rik,Stx7,1500011K16Rik,Mdfic,Nagk
**mmu-miR-485**	32(776)	2,96E-05	Zfand3,Dtx2,Copg,Trpc7,Smpdl3a,Cyp27a1,Gadd45b,Vps8,Tmem87b,9430038I01Rik,Sepw1,Txnrd3,Dok4,Tpcn1,Epb4.1l4b,C1qtnf6,P4ha2,Ppt2,St3gal5,Rell2,Itpr2,Naga,Sh2d4a,0610037L13Rik,Isca2,Atp6v0b,Tex264,Bcmo1,1110034G24Rik,Fbn2,Rabac1,Lmna
**mmu-miR-483***	27(776)	2,96E-05	Adra1b,Slc37a4,Slc6a7,Cox4nb,Alg14,Atp6v0e,Trpv4,Ppp2r5b,Crybb3,Gabarapl1,1700009P17Rik,Tmem87b,Tpcn1,Chrd,Nfe2l1,Cspg4,Impdh1,Slc22a4,Nkiras2,Nenf,Zfand2b,Fzd8,Sema3f,Serf2,Cdh7,Acaa1a,Igfbp2
**mmu-miR-431**	32(776)	3,05E-05	Ahnak2,Dym,Adra1b,Plb1,Ascc2,Dhrs3,Rab4b,Nt5m,Atp6v0e,Abcb6,Stam2,Gadd45b,Ppp2r5b,Vps8,Anxa3,Ppt2,St3gal5,Mocs2,Glt8d1,S100a13,Nupr1,BC089491,Ctsb,Grk4,BC029214,Sirt2,Pcbd2,Tbccd1,Gstp1,Sh3tc2,1500011K16Rik,Dmpk
**mmu-miR-325**	39(776)	3,22E-05	Oit3,Crat,Stmn3,Slc6a7,Fbxo9,Insig2,Hexa,Emilin2,Rab40b,Kcnn1,Trpv4,Lamb2,Trappc6a,Crybb3,Ttc15,5830405N20Rik,Apoa1bp,2010003O02Rik,Gstm2,Lnx1,Epb4.1l4b,P4ha2,Nudt7,Tmem154,Fabp3,Galnt10,Psen2,Slc22a4,Bbs5,Gabarap,Glipr1,Ufc1,1110034G24Rik,Cdkn1a,Cox6a2,Scarb2,Hadhb,Immp1l,3110002H16Rik
**mmu-miR-188-3p**	35(776)	3,24E-05	Ppp1r16a,Usp2,Kif27,Dhrs3,Rab4b,Fbxo9,Rras,Habp4,Wnt9a,Abcb6,Clstn1,Tmem64,Neu1,Atp6v1e1,Cd248,Mtmr3,Tgfb2,F630110N24Rik,Ankra2,Slc31a2,Sepw1,Ccdc64,Chrd,Cdk5,Glt8d1,Rell2,Slc35c2,Dbp,Npdc1,Atp6v0b,App,Slco3a1,Spata21,Csrp2,Atp5e
**mmu-miR-339-3p**	35(776)	3,38E-05	Pank2,Yif1b,Sgca,Stmn3,Adc,Tmem41a,Smpdl3a,Nt5m,Hexa,Kcnn1,Clstn1,Trpv4,Adcy9,Gramd3,Sepw1,Cacna1g,Myo6,2900010M23Rik,A430110N23Rik,Rora,Ascc1,Fabp3,Slc35c2,Ly6c1,Nol3,Mustn1,Atp6v0b,Gabarap,Asb1,Csrp2,Ckm,Afap1l2,Esrrb,Tubb2a,Ltbp3
**mmu-miR-101a**	37(776)	3,39E-05	Ppp1r16a,Oit3,Zfand3,Ap3s1,Dtx2,Map3k8,Sgca,Mcee,Trpc7,Rab38,Abcb6,Stam2,Ppapdc3,Caml,Rab3gap2,Ccdc64,Nipa1,Ndufa10,Mtap6,Ascc1,Mfap5,Mlycd,Ing4,Dopey2,Pxmp4,Srp14,Nr2c1,Serf2,1110034G24Rik,Htatip2,Clcn7,Acaa1a,Cdkn2b,Tbc1d14,Fzd6,Tubb2a,Ltbp3
**mmu-miR-302a**	35(776)	3,43E-05	Maob,Mpv17,Magi3,Rab38,Emilin2,Loxl1,Rragc,2010003O02Rik,Dlg2,Nudt7,2700078E11Rik,Glt8d1,9130011J15Rik,S100a13,Os9,Akap4,Triap1,Impact,Lrp4,1810037I17Rik,Clip4,Rab7l1,Synpo2,Nr2c1,Ikbkg,Chmp4c,BC004004,Csrp2,Sh3tc2,Cdkn1a,Nhlrc1,Wdr7,Uchl1,1700094D03Rik,Ankrd44
**mmu-miR-883b-3p**	36(776)	3,58E-05	Ugp2,Snap91,Hadha,Adc,Mpv17,Tmem41a,Rab3d,Nt5m,Rab40b,Fn1,Clstn1,AI593442,Renbp,Fstl1,Anxa3,Ttc15,Wisp2,Stard6,Mocs2,Iscu,Naga,Ctsb,Srp14,Adamts2,Atp6v0b,Sstr4,1110034G24Rik,Clcn7,1810046J19Rik,Kif3a,Acaa1a,Scarb2,Hadhb,Igbp1,Fbxw4,Oplah
**mmu-miR-150**	32(776)	4,21E-05	Ahnak2,Dym,Iah1,Adra1b,Copg,Tcta,Dhrs3,Sync,Neu1,Iqsec2,Dynlrb1,Tbcd,Lyrm1,Gabarapl1,Ptplad2,Cacna1g,Appl2,Rora,Cspg4,Adamtsl5,Bbs5,Ing4,BC089491,Tollip,Dbp,Gpc4,Npdc1,Spata21,Glipr1,Wscd2,C1qtnf1,1700094D03Rik
**mmu-miR-763**	33(776)	4,23E-05	Ascc2,Prss29,Smpdl3a,Rras,Cyp4v3,Hdlbp,Ppp2r5b,Cd248,Tbcd,Gramd3,Lmbr1,Sepw1,Arl2,Impdh1,St3gal5,Nkiras2,Akap4,S100a16,Nol3,Pde6d,Pltp,Dbp,Srp14,Tbccd1,Ptdss1,Vps24,1110034G24Rik,4930579G22Rik,Cdkn1a,Dnajc4,Rabac1,Oplah,2810428I15Rik
**mmu-miR-339-5p**	33(776)	4,56E-05	Ppp1r16a,Gyg,Prss29,Hexa,Clstn1,F630110N24Rik,2900010M23Rik,Trappc2l,Galns,Ndel1,Grina,Rora,Slc9a6,Nfe2l1,2700078K21Rik,9130011J15Rik,Naga,Mustn1,Pcyt1a,Abtb2,Necap2,Lrba,Zfand2b,Sirt2,Tmbim1,Slc2a6,BC004004,Mras,Adamtsl4,Scarb2,Igfbp2,Atp5e,Scn1b

miRNA name	Number of genes	Corrected p-value	miRNA targets among genes induced in Rasless cells (from Table S1)
**mmu-miR-9**	33(776)	4,56E-05	Ahnak2,Pmp22,Ascc2,Prss29,Cyp4v3,Cd248,Tbcd,1110007C09Rik,Pld1,Tmem87b,Pigz,Anxa3,Slc31a2,Guk1,Chrd,Hsd3b3,P4ha2,Pltp,Hgsnat,0610037L13Rik,1300010F03Rik,A930005H10Rik,Sorbs3,Ugt2b36,Ptdss1,Cdh7,Itfg3,1810046J19Rik,Slc25a35,Wscd2,Igbp1,Lmna,Mtus1
**mmu-miR-615-5p**	35(776)	4,63E-05	Pank2,Thra,Adra1b,Sgca,Stmn3,Ecm1,Rab4b,Cd81,Clstn1,Trpv4,Creb3,Cd248,Trappc6a,Ank2,Chrd,Ypel3,Shank3,Cspg4,Pbxip1,Ppt2,Dusp8,Cables1,Rell2,Mlycd,Ntn4,Ctsb,Npdc1,Adamts2,BC029214,Fzd8,Kif13b,Slc2a6,2900026A02Rik,Cox6b1,Shroom3
**mmu-miR-467d**	37(776)	4,69E-05	Trpc7,BC024814,Selm,Aldh7a1,Cd81,Stam2,Smad6,Cyp4v3,Caml,D6Wsu163e,Akap13,Calml4,Ndel1,Tcn2,Gdf15,Pbxip1,2700078K21Rik,C1qtnf6,Dusp8,Serpinb9b,Cables1,Pde6d,Mtm1,Gpc4,Srp14,1300010F03Rik,Pde4dip,Atp6v0b,Mmab,Gabarap,Rab7l1,ORF63,Stx7,1500011K16Rik,Rit1,Mdfic,Nagk
**mmu-miR-206**	30(776)	5,45E-05	Bri3,Snrpn,Smpdl3a,Sh3bgr,Mtap1a,Fn1,D6Wsu163e,Adcy9,Sgcb,Dgkg,Lmbr1,Hexb,2010003O02Rik,Zbtb4,Tppp,P4ha2,Lrch1,Atg7,Dopey2,Pde6d,Plcb1,Ankrd29,App,Def8,Mrpl55,Ptprm,Vps24,Dhps,Kif3a,Cpeb1
**mmu-miR-883a-5p**	35(776)	5,51E-05	1300014I06Rik,2310028O11Rik,Stmn3,Adc,Fahd2a,Gabarapl2,Rab38,Sh3bgr,Ank2,Anxa3,Skap2,Qk,Mxra8,AI317395,Ccdc64,Wisp2,Impdh1,Iscu,Anxa1,Glt8d1,Ctsb,Dbp,Srr,Srp14,1300010F03Rik,Mmab,Gabarap,6530401N04Rik,Cox6a2,Kif3a,Acaa1a,Oplah,Atp10d,Aldh3a2,Shroom3
**mmu-miR-10b***	29(776)	5,89E-05	2310028O11Rik,Sqrdl,Stmn3,Emilin2,Adamts5,Caml,Adcy9,Fstl1,Lmbr1,Tmem87b,Clip1,Arnt,Hexb,Cacna1g,Wwp2,Snta1,Serpinb9c,Pon3,Adamtsl5,4930523C07Rik,1810037I17Rik,Ahnak,Acyp1,Cbara1,Daglb,Slc25a35,Igbp1,1700094D03Rik,Immp1l
**mmu-miR-9***	28(776)	5,89E-05	Pank2,Iah1,Bri3,Caml,F630110N24Rik,Tbcd,Vps8,2010003O02Rik,Appl2,Herpud2,Dlg2,Hsd3b3,Slc9a6,P4ha2,Glt8d1,Psen2,Plcb1,Srp14,Ctsl,Ugt2b36,Cxcr7,Slc25a35,Igbp1,Uchl1,Cacna1b,1700094D03Rik,Immp1l,Npc1
**mmu-miR-148a**	36(776)	5,91E-05	Dym,Plb1,Gabarapl2,Sh3bgr,Klf15,Sgcb,Myom2,Txnrd3,Grina,Ypel3,Cgnl1,Pbxip1,Ascc1,Nudt7,Mocs2,Nrip3,Akap4,Gpc4,Ankrd29,Pde4dip,Acyp2,Il17d,Cdh7,Mras,Sh3tc2,Ckm,Tom1l2,Stx7,Slamf9,Rhoj,1810030N24Rik,Tmem50b,Zfp704,Tmem9b,1700094D03Rik,3110002H16Rik
**mmu-miR-218-2***	29(776)	6,00E-05	Pank2,Thra,Dhrs3,Alg14,Trappc6a,Adcy9,Myom2,Atp2a3,Tcn2,Ndufa10,Chrd,Zbtb4,Ascc1,Adar,9130011J15Rik,S100a13,Npdc1,2010111I01Rik,1300010F03Rik,App,Spata21,Chchd6,Acyp2,Kif13b,Asb1,Synpo2,Cxcr7,Rhoj,Hint3
**mmu-miR-705**	34(776)	6,03E-05	Ppp1r16a,Pank2,Thra,Yif1b,Sgca,S100a1,Fahd2a,Cyp27a1,D16H22S680E,Trpv4,Slc35a2,Slc7a7,Abcd1,Vps8,Pigz,Camk1,Sepw1,Gstm2,Arl2,Ndufa10,Itgb1bp1,Zbtb4,Lims2,Nupr1,Mustn1,Ctsb,Gabarap,Slc25a39,BC046404,Scn1b,Rabac1,Lmna,Pcsk6,Ltbp3
**mmu-miR-464**	32(776)	6,22E-05	Maob,Magi3,Crot,Gpx4,Lrrc51,Cobl,Emilin2,Abcb6,Adamts5,Caml,Atp6v1e1,Tbcd,Clip1,Cacna1g,Apoa1bp,Gdf15,Lrrc57,Tmem175,Naga,Hscb,Mtcp1,1300010F03Rik,Atp6v0b,Tm9sf4,Mmab,Chchd6,Cpa6,Tyrp1,Scarb2,Yif1a,Tmem9b,1700094D03Rik
**mmu-miR-125b-5p**	37(776)	6,69E-05	Ppp1r16a,Pank2,Col14a1,Plb1,Kif27,Kremen1,Ppapdc3,Neu1,Tgfb2,Akap13,Dgkg,Slc46a3,Gpr133,Grina,Rapsn,Capn5,Psen2,Mlycd,Triap1,Ankrd29,Pde4dip,Atp6v0b,Tbccd1,Slc2a6,Ikbkg,Sema3f,Hck,Ssfa2,Chmp4c,Itfg3,Acyp1,Sh3tc2,1810046J19Rik,Slc25a35,Fbxw4,Tmem38a,1700021C14Rik
**mmu-miR-140**	33(776)	6,73E-05	Ifi35,Rnf13,Pmp22,Crot,Trpc7,Rab38,Wnt9a,Akap13,Adcy9,Gng5,Anxa3,Arnt,Stx8,Rab3gap2,Tpcn1,2010003O02Rik,Herpud2,Chrd,Ypel3,Ascc1,Glt8d1,Ly6c1,Dopey2,Tollip,Mustn1,Sirt2,Ssbp2,Hr,1110034G24Rik,4930579G22Rik,Mras,Slc25a35,Cdkn2b
**mmu-miR-322**	32(776)	6,86E-05	Bri3,Tcta,Tmem41a,Rab4b,Lyrm1,Klk1b4,Skap2,Cdc42ep2,Synpo,Arl2,Ypel3,C1qtnf6,Rapsn,Nudt7,Lrrc57,Glt8d1,Rdh12,Mlycd,Ly6c1,S100a16,Dopey2,BC089491,Triap1,0610037L13Rik,Srp14,Frmpd1,Zfand2b,Slc25a39,Ptprm,1110034G24Rik,Atp5e,Rabac1
**mmu-miR-712**	29(776)	7,21E-05	Snap91,Sgca,Stmn3,Ascc2,Krt80,Abcb6,Prss36,Kif1c,F630110N24Rik,Armc8,Ttc15,Guk1,Arl2,Ypel3,Nfe2l1,Mtap6,Snta1,Lrrc57,Mfap5,Pik3ip1,Glt8d1,Rell2,Zfp560,Naga,Nol3,Sh2d4a,Ptprm,Cog6,Igfbp2
**mmu-miR-30b**	35(776)	7,30E-05	Ppp1r16a,Pank2,Fech,Snrpn,Tmem41a,Magi3,Arvcf,Rab3d,Rab40b,Vps39,Ank2,Vps8,Gramd3,Ankra2,5830405N20Rik,Hoxa1,Aldh6a1,P4ha2,Ccdc80,Anxa1,Tmem175,Psen2,Vdac1,Bbs5,Frmpd1,Atg12,Ahnak,Pcbd2,Fbxl20,Slc41a2,Afap1l2,Fbxw4,Wdr7,Chst12,Atp10d
**mmu-miR-668**	31(776)	7,33E-05	Commd8,Mal,Gpx4,Smpdl3a,Rab3d,Cd81,Ppp2r5b,Lyplal1,1700009P17Rik,Gdf15,Cspg4,2700078K21Rik,P4ha2,Ascc1,Idua,Iscu,Cdk5,Ehd2,1110003E01Rik,Nkiras2,Zfand2b,Slc41a3,Htatip2,Scarb2,Igfbp2,Slamf9,Rhoj,Slc2a8,Limch1,1700094D03Rik,Kctd21
**mmu-miR-302a***	29(776)	7,53E-05	Prss29,Crot,Lrrc51,Fem1a,Atp6v0e,AI593442,Ank2,Dusp3,Pld1,Vps8,Armc8,Pigz,Ap3m2,Myo6,Suox,Tcn2,Myeov2,Nudt7,9130011J15Rik,Hbxip,4930523C07Rik,Gpc4,Ankrd29,1810037I17Rik,BC046404,1810027O10Rik,Dhps,Lrpap1,Shroom3
**mmu-miR-199b***	28(776)	7,75E-05	Ppp1r16a,Gtf2h5,Sgca,Fahd2a,Fbxo9,Hexa,Sh3bgr,Sync,Pld1,Gng5,Pigz,Ndel1,Pik3ip1,Tmem175,Akap4,2010111I01Rik,Tbccd1,Slc2a6,BC004004,Sparc,Csrp2,Daglb,Tom1l2,Igfbp2,Gas6,Fbxw4,Atp6v0a1,Cyb5r3

miRNA name	Number of genes	Corrected p-value	miRNA targets among genes induced in Rasless cells (from Table S1)
**mmu-miR-137**	33(776)	7,79E-05	Dym,Ap3s1,Iah1,Map3k8,Lgr4,Glrb,Inpp5a,AI593442,Btf3l4,Gramd3,Slc46a3,Appl2,Herpud2,Itgb1bp1,Stard6,Dusp8,Ergic3,Rell2,Os9,Gpc4,Ankrd29,0610037L13Rik,Lrba,1300010F03Rik,Ahnak,Kif13b,Sema3f,Acyp1,Cox6a2,1810046J19Rik,Scn1b,Dusp22,Chpt1
**mmu-miR-499**	30(776)	8,24E-05	Pank2,Adc,Papss2,Alg14,Gabarapl2,Btf3l4,Skap2,Ankra2,Herpud2,Ndel1,Mtap6,Nudt7,Nrip3,Iscu,Garnl3,Ulk2,Slc22a4,Ntn4,Hscb,Pde6d,Triap1,Plcb1,Pltp,Hgsnat,Srp14,Sparc,Atp5e,Igbp1,Hhip,Rab22a
**mmu-miR-342-3p**	32(776)	8,64E-05	Dtx2,Adra1b,Plb1,Gpx4,Smpdl3a,Tlr6,Insig2,Abcb6,Stam2,Clstn1,Creb3,Kif1c,Lyplal1,Tmco3,Myom2,Zbtb4,Wwp2,Nudt7,Ttll7,Ly6c1,Zmat2,Ctsb,Dbp,Sirt2,Tanc2,Rab7l1,Fbn2,Acyp1,Ankrd12,Tubb2a,1700094D03Rik,Mtus1
**mmu-miR-125a-3p**	36(776)	8,67E-05	Mpv17,Rab4b,Atp6v0e,Inpp5a,Trpv4,F630110N24Rik,Akap13,Trpt1,Fstl1,Rarres2,Tmco3,Itgb1bp1,Tom1,Rapsn,Riok3,Nudt7,1110003E01Rik,Trak1,S100a16,Mtcp1,Tollip,Ankrd29,Isca2,Atp6v0b,Mmab,Asb1,Tex264,Slc2a6,BC046404,Adamtsl4,1810046J19Rik,Scarb2,Slamf9,Fbxw4,Hint3,Shroom3
**mmu-miR-19a**	33(776)	9,23E-05	Gtf2h5,2310028O11Rik,Snrpn,Mpv17,Sh3bgr,Wnt9a,Klf15,Btf3l4,Armc8,Skap2,Ankra2,Suox,Nipa1,Rora,Slc9a6,Impdh1,St3gal5,Glt8d1,Itpr2,Reep3,Ankrd29,Ahnak,Atp5s,Kif3a,Acaa1a,Hadhb,Igbp1,Scn1b,Chst12,Ltbp3,Zfp704,Vps29,Hhip
**mmu-miR-292-3p**	35(776)	9,24E-05	Ugp2,Usp2,Sgca,Plb1,Arvcf,Coq10a,Fahd2a,Fbxo9,Papss2,Tlr6,Insig2,Cobl,Hexa,Nfs1,Stx8,Ttc15,Apoa1bp,A430110N23Rik,Nudt7,St3gal5,Serpinb9b,Slc22a4,Vnn1,Slc26a11,Mmab,Ahnak,Ikbkg,Ufc1,Csrp2,Clcn7,Nudt18,Dhps,Ankrd12,Atp6v0a1,Tmem38a
**mmu-miR-16**	33(776)	0,000101747	Dym,Wdr47,Bri3,Sgca,Tcta,Tmem41a,Rab4b,Rab40b,Mtmr3,1110007C09Rik,Lyrm1,Fbxl2,Tpcn1,2010003O02Rik,Arl2,Ypel3,Shank3,Rapsn,Nudt7,St3gal5,Ly6c1,S100a16,Dopey2,Abtb2,Srp14,Frmpd1,Zfand2b,Ahnak,Nr2c1,Cdh7,1110034G24Rik,Itfg3,Rabac1
**mmu-miR-674**	32(776)	0,000102141	Ap3s1,Dtx2,Snap91,Lrrc51,Prss36,Atp6v1e1,Abcd1,Vps8,Mrgprf,Ankra2,Gstm2,Ccdc64,Hsd3b3,2700078K21Rik,Rapsn,P4ha2,2700078E11Rik,Deb1,Ccdc80,Ing4,Gdap2,Grk4,Necap2,Ctsl,Ahnak,Htatip2,Cdkn1a,Scarb2,Slc25a35,Igbp1,Tmem38a,Fads3
**mmu-miR-146b***	25(776)	0,000102592	Ahnak2,Mpv17,Fbxo9,Rras,Ehd1,Lamb2,Trappc6a,F630110N24Rik,Adcy9,Alkbh6,2010003O02Rik,Ccdc64,2700078K21Rik,C1qtnf6,Slc35c2,Ly6c1,Reep3,Atg7,1300010F03Rik,Zfand2b,Chchd6,1110034G24Rik,Slc25a35,Slamf9,Lmna
**mmu-miR-93***	28(776)	0,000103386	Dym,Tmem41a,Higd1a,Tbc1d10a,Vps39,Akap13,Adcy9,Btf3l4,Clip1,Slc31a2,9430038I01Rik,5830405N20Rik,Ccdc64,Epb4.1l4b,Dopey2,BC089491,Plcb1,Srr,1300010F03Rik,Dhps,6330406I15Rik,Cox6a2,1810046J19Rik,Acaa1a,Tom1l2,Mrps14,Oplah,1700094D03Rik
**mmu-let-7g***	34(776)	0,000107389	Zfand3,Ap3s1,Ccdc126,Rab43,Rpl17,Kif27,Ascc2,Selm,Gadd45b,Atp9b,Mtmr3,Amhr2,Rarres2,Mrgprf,Skap2,Cacna1g,2900010M23Rik,Chrd,Stard6,L1cam,Man2b1,Mtap6,Wwp2,Asah1,Plcb1,Gpc4,Atp6v0b,Ahnak,Sirt2,Ssbp2,Tuba8,Ufc1,Cdh7,Acyp1
**mmu-miR-365**	33(776)	0,000107761	Pank2,Snap91,Map3k8,Col14a1,Sgca,Kif27,Tmem41a,Hexa,Ehd1,Nudt13,Trappc6a,Vps8,Fstl1,Arnt,Cdc42ep2,Tom1,Idua,Ttll7,Megf6,Add3,Ehd2,1110003E01Rik,Mustn1,Abtb2,Mmab,Mrpl55,Plac9,Ptdss1,Mras,Fzd6,Tmem38a,Ankrd44,Chpt1
**mmu-let-7a**	34(776)	0,000108965	Ppp1r16a,Ifi35,Dtx2,Mcee,Cat,Tcta,Tmem41a,Alg14,Hexa,Sh3bgr,Rras,Elmo2,R74862,Cyp4v3,Trappc6a,Akap13,Gng5,Lama2,Camk1,Chrd,Lnx1,Slc35b1,Fabp3,Gdap2,Tollip,Isca2,App,Zdhhc24,Ikbkg,Sema3f,1110034G24Rik,6330406I15Rik,1810046J19Rik,Kif3a
**mmu-miR-673-5p**	33(776)	0,000109407	Ppp1r16a,Cox4nb,Dhrs3,Rab4b,Trpc7,Sema3b,Emilin2,R74862,Abcb6,Inpp5a,Loxl1,Cd248,Vps8,Sepw1,Ephb3,Trappc2l,Tcn2,Slc9a6,Ascc1,Nudt7,St3gal5,Ehd3,Ccdc80,Zdhhc8,Nol3,0610037L13Rik,Atp6v0b,Hck,Acyp1,Stxbp1,Cacna1b,Nagk,Shroom3
**mmu-miR-683**	30(776)	0,000114705	Map3k8,Hspa12a,Selm,Cyp27a1,Nt5m,Atp6v1e1,Slc7a7,Btf3l4,Mrgprf,Klk1b4,Hexb,Txnrd3,Arhgef12,Rora,Snta1,Ascc1,Slc22a4,Os9,Nupr1,Mtcp1,2010111I01Rik,Zfand2b,Zdhhc24,Ptprm,Mxra7,Vps24,Ufc1,Arhgap1,Kif3a,Gas6
**mmu-miR-681**	31(776)	0,000114737	Mrc2,Ahnak2,Tssc4,Bri3,Ascc2,Adc,Fahd2a,Smpdl3a,Sema3b,Nt5m,Elmo2,Krt80,Nudt13,F630110N24Rik,Myadm,Tbcd,Vps8,Lyplal1,Dhrs1,Slc31a2,Stx8,Myom2,Tpcn1,Synpo,Ing4,Klf2,Bcmo1,Slc25a39,Cox6a2,Hadhb,Ltbp3
**mmu-miR-801**	28(776)	0,000115869	Plb1,Lgr4,Slc22a18,Tbcd,Btf3l4,Dgkg,Slc46a3,2010003O02Rik,Arl2,Itgb1bp1,Shank3,C1qtnf6,Garnl3,Nupr1,Akap4,Zfand2b,Rab7l1,Cdh7,Ndrg4,Gpr39,Cox6a2,1810046J19Rik,Ckm,Acaa1a,Nhlrc1,Gas6,1810030N24Rik,Wdr7
**mmu-miR-294**	30(776)	0,000116059	Gtf2h5,Magi3,Crot,Smad6,Crybb3,Txnrd3,2010003O02Rik,Nudt7,2700078E11Rik,Serpinb9b,Glt8d1,9130011J15Rik,Ing4,Dner,Lrp4,Mmab,Zfand2b,Ahnak,Clip4,Rab7l1,Csrp2,Sh3tc2,Acaa1a,Nhlrc1,Daglb,Uchl1,Zfp704,1700094D03Rik,Atp10d,Ankrd44
**mmu-miR-453**	29(776)	0,00011651	Ahnak2,Stmn3,Rab4b,Alg14,Sema3b,Caml,Ank2,Tbcd,Slc7a7,Abcd1,Tmem87b,Camk1,Emp2,Tcn2,Cspg4,Impdh1,Snta1,Dusp8,Pon3,2310046K01Rik,Trak1,Triap1,Def8,Uqcrq,BC046404,Serf2,Igbp1,Tbc1d9b,Wdr7

miRNA name	Number of genes	Corrected p-value	miRNA targets among genes induced in Rasless cells (from Table S1)
**mmu-miR-223**	30(776)	0,000125873	Sgca,Mcee,Smpdl3a,Prss36,Cd248,Btf3l4,1700009P17Rik,Tmem87b,Tom1,Gp1bb,Dusp8,Tmem154,Lrch1,Tmem175,Mlycd,Slc22a4,Mtm1,Slc39a14,Usp53,Kif13b,Asb1,Tex264,Slc2a6,Slc25a35,Atp5e,Rhoj,Pcsk6,Ltbp3,Immp1l,Aldh3a2
**mmu-miR-466g**	39(776)	0,000125957	Ahnak2,Thra,Dtx2,2310028O11Rik,Adra1b,Tmem41a,Smpdl3a,Insig2,Rab3d,5930434B04Rik,D16H22S680E,Stam2,Sync,Creb3,Dynlrb1,Dusp3,Qk,Gstm2,Guk1,Zbtb4,Myeov2,2700078K21Rik,Glt8d1,Zdhhc8,Ly6c1,Zmat2,Trak1,Grk4,Lrba,1300010F03Rik,Ahnak,Slco3a1,Rab7l1,Cdh7,1110034G24Rik,Kif3a,Tom1l2,Uchl1,Mtus1
**mmu-miR-361**	29(776)	0,000132126	Snrpn,Adc,Mpv17,Magi3,Rab38,D6Wsu163e,1810020D17Rik,Vps8,Gramd3,Klk1b4,Dhrs1,Mxra8,AI317395,Myo6,Qpct,Stard6,Grik2,Iscu,Tollip,Ctsl,A930005H10Rik,Spata21,Acyp2,Ugt2b36,Slc1a4,Ptprm,Igbp1,Zfp704,1700094D03Rik
**mmu-miR-124***	28(776)	0,000137134	Iah1,Kif27,Crot,Smpdl3a,Insig2,Nt5m,Hexa,D16H22S680E,Lamb2,Cd248,Tgfb2,Vps8,Dgkg,Gpr133,Ttc15,Atp2a3,Itgb1bp1,Snta1,Vnn1,Acyp2,Usp53,Tmbim1,Chmp4c,Dhps,1810046J19Rik,Tom1l2,Slamf9,1700094D03Rik
**mmu-miR-761**	31(776)	0,000137197	Ifi35,Usp2,Zbtb7c,Rab4b,Cd81,Trpv4,Gramd3,Txnrd3,Trappc2l,Arl2,Tcn2,L1cam,Pbxip1,Mtap6,Mocs2,Ulk2,Psen2,Ly6c1,Slc26a11,1300010F03Rik,Atp6v0b,BC029214,Slc25a39,Cbara1,Dnajc4,Tbc1d14,Atp6v0a1,Rabac1,Lmna,Limch1,2810428I15Rik
**mmu-let-7f***	33(776)	0,000137808	Zfand3,Ap3s1,Map3k8,Bri3,Slc37a4,Lgr4,Ascc2,Cobl,Sh3bgr,Atp9b,Akap13,Btf3l4,1700009P17Rik,Skap2,Myom2,Lnx1,Gdf15,Snta1,Serpinb9c,Glt8d1,Fabp3,Plcb1,Ctsl,Klf2,Tbccd1,Ssbp2,Sema3f,Cox6a2,Scarb2,Wdr7,Dusp22,Zfp704,Cacna1b
**mmu-miR-291b-3p**	36(776)	0,000145605	Mcee,Hadha,Higd1a,Habp4,D16H22S680E,Smad6,Caml,Loxl1,D6Wsu163e,Iqsec2,Dynlrb1,Armc8,Clip1,Tmco3,Mxra8,Grina,L1cam,Rapsn,Nudt7,Idua,Dusp8,Adamtsl5,Nupr1,Pde6d,Mtm1,Srp14,App,Ahnak,Clip4,Rab7l1,Cog6,Atp5e,1500011K16Rik,Zfp704,Mtus1,Nagk
**mmu-miR-590-3p**	32(776)	0,000146754	Pank2,Zfand3,Lgr4,Ascc2,Cyp27a1,Alg14,Stk39,Skap2,Ankra2,5830405N20Rik,Tob1,Qpct,Lnx1,Mtap6,Bphl,Mocs2,Ccdc80,Glt8d1,Sema3c,Slc22a4,Bbs5,Vnn1,Dopey2,Srp14,Pdia5,Acyp2,Ugt2b36,Ssbp2,Cdh7,Csrp2,Igfbp2,1810030N24Rik
**mmu-miR-688**	30(776)	0,000147028	Zfand3,Thra,Usp2,Slc37a4,Lgr4,Atp6v0e,Abcb6,Inpp5a,F630110N24Rik,Gramd3,Anxa3,Skap2,Camk1,Ephb3,Trappc2l,Ypel3,Cgnl1,Snta1,Cables1,Pik3ip1,Bbs5,Hgsnat,Rab7l1,Tbccd1,Csrp2,Tyrp1,Kif3a,Acaa1a,Tom1l2,Rit1
**mmu-miR-221**	30(776)	0,000148805	Bri3,Sgca,Sema3b,Nt5m,Prss36,Trappc6a,Vps8,Lyplal1,Mrgprf,Skap2,Mrgpre,Gpr133,Alkbh6,Calml4,Dph1,Ctso,Zbtb4,Nudt7,Lims2,Ly6c1,Reep3,Ankrd29,Zfand2b,Synpo2,Cxcr7,Htatip2,Tbc1d9b,Chst12,Pcsk6,Oplah
**mmu-miR-501-5p**	30(776)	0,000148805	Acp2,Thra,Sgca,Ecm1,Ascc2,Tmem41a,Crot,Caml,Cd248,Slc35a2,Slc7a7,Grina,L1cam,Aldh6a1,Slc35b1,Megf6,Adar,Zfp560,Os9,Clip4,Tex264,Cpa6,Acaa1a,Igfbp2,Wscd2,Igbp1,Tbc1d9b,Mdfic,Limch1,Zfp704
**mmu-miR-470**	28(776)	0,000148876	Ugp2,Tssc4,Ecm1,Dhrs3,Fbxo9,Prss36,Cd248,Mtmr3,Crybb3,Gng5,Cry2,Ap3m2,Ndel1,Ndufa10,Man2b1,Snta1,Nrip3,Ccdc80,Garnl3,Nkiras2,Npdc1,Spata21,Synpo2,Tex264,Cdh7,Csrp2,Rabac1,1700013F07Rik
**mmu-miR-494**	27(776)	0,00014965	Map3k8,Commd8,Mal,Rab38,Cyp4v3,Slc7a7,Vps8,Camk1,Qk,Appl2,Grina,Ndufa10,Zbtb4,Slc9a6,P4ha2,Grik2,Htra3,Pik3ip1,Mtcp1,Cyb5,Acyp2,Adamtsl4,Cox6a2,Dusp22,Vps29,Cacna1b,Immp1l
**mmu-miR-743b-5p**	32(776)	0,000153945	Oit3,Copg,Stmn3,Plb1,Dscr3,Caml,Loxl1,Slc7a7,Vps8,Armc8,Ttc15,Myom2,Ccdc64,Qpct,Myeov2,Ppm1h,Gdap2,Atg7,Nol3,Pde6d,Slc26a11,Clip4,Usp53,BC046404,Adamtsl4,Daglb,Scarb2,9930013L23Rik,Slamf9,Lmna,Uchl1,Zfp704
**mmu-miR-216b**	32(776)	0,000160645	Gtf2h5,Iah1,Plb1,Pef1,Tmem41a,Gpx4,Vps39,Copz2,Dab2ip,Adcy9,Slc46a3,9430038I01Rik,Hoxa1,Txnrd3,2010003O02Rik,Gstm2,Qpct,Zbtb4,Mocs2,2310046K01Rik,Trak1,Sh2d4a,Hgsnat,Srr,Npdc1,Necap2,A930005H10Rik,Hr,Fbxl20,Gpr39,1500011K16Rik,Tbc1d9b
**mmu-miR-27a***	28(776)	0,000161062	Usp2,Sema3b,Rras,Copz2,Gstm2,Grina,Zbtb4,Snta1,Dusp8,Mfap5,Anxa1,Rell2,Srr,Zfand2b,Ahnak,Spata21,Tex264,Plac9,Hck,Trp53inp2,Cdh7,Mras,Tom1l2,1500011K16Rik,1700021C14Rik,Stxbp1,Rabac1,Shroom3
**mmu-miR-21**	28(776)	0,000163308	Gnptg,Yif1b,Kif27,Sh3bgr,Adcy9,Sgcb,Armc8,Lmbr1,Clip1,Tpcn1,2010003O02Rik,Ndel1,Farp2,Lnx1,Lrrc57,Akap4,Plcb1,Ctsl,Spata21,Il17d,1110034G24Rik,Sparc,Acyp1,Reck,Rabac1,Satb1,Uchl1,Zfp704
**mmu-miR-542-5p**	31(776)	0,000163734	Tlr6,Lrrc51,Abcb6,Hist2h2bb,Inpp5a,Hdlbp,Acox1,Ank2,Dusp3,Copz2,Akap13,Vps8,Clip1,Dph1,Tpcn1,Gstm2,Trappc2l,Rell2,Adamtsl5,Vdac1,Os9,Slc39a14,Mrpl55,Slc41a3,Rab7l1,Bcmo1,Csrp2,1810046J19Rik,Slamf9,Yif1a,Shroom3
**mmu-miR-299**	29(776)	0,000171661	Thra,Ap3s1,Klhl18,Trpv4,Creb3,Kif1c,Slc35a2,Gng5,Btf3l4,Lmbr1,Pigz,Dhrs1,Myom2,Lgals9,Alas1,Idua,Serpinb9c,Ergic3,Slc35c2,Ly6c1,Hgsnat,Npdc1,Def8,Tanc2,Tuba8,Mras,Ckm,Pcsk6,Ltbp3
**mmu-miR-29b**	35(776)	0,000172812	Copg,Ccdc126,Slc37a4,Pmp22,Smpdl3a,Hexa,Sh3bgr,Habp4,Emilin2,Abcb6,Smad6,Vps8,Anxa3,Myo6,Ephb3,Tpcn1,Pdgfc,Qpct,Chrd,Impdh1,Asah1,Mfap5,Ccdc80,Fabp3,Ing4,Adamts2,Klf2,Tanc2,Nr2c1,Chmp4c,Fbxl20,Dhps,9930013L23Rik,Tubb2a,Dusp22
**mmu-miR-877***	23(776)	0,000174793	Sgca,Rab4b,Sema3b,Sh3bgr,Elmo2,Clstn1,Prss36,Loxl1,Crybb3,F630110N24Rik,Myo6,Txnrd3,Snta1,Adar,Nupr1,BC089491,Nenf,Pcyt1a,Zfand2b,Pdia5,4930579G22Rik,Mras,Igfbp2

miRNA name	Number of genes	Corrected p-value	miRNA targets among genes induced in Rasless cells (from Table S1)
**mmu-miR-744***	25(776)	0,000183871	Rnf13,Tssc4,Map3k8,Rab43,Stmn3,Ecm1,Hspa12a,Magi3,Sema3b,Napb,Nudt13,Ppp2r5b,D6Wsu163e,Iqsec2,Slc7a7,Rarres2,Camk1,Calml4,Repin1,Grina,Ascc1,Zfp560,Nupr1,Akap4,1810046J19Rik
**mmu-miR-874**	30(776)	0,000185626	Pank2,Rab43,Slc6a7,Prss29,Rab3d,Hexa,Clstn1,Ppp2r5b,Abcd1,Tbc1d22a,Trpt1,Alkbh6,Repin1,Synpo,Trappc2l,Guk1,Galns,Zbtb4,2700078K21Rik,Ergic3,Cdk5,Tmem154,Pxmp4,Dbp,Klf2,Ikbkg,Sema3f,Wscd2,Tbc1d9b,Lmna
**mmu-miR-707**	29(776)	0,000187443	Cat,Commd8,Prss29,Trpc7,Fbxo9,Sh3bgr,Smad6,Cd248,Ank2,Slc35a2,Clip1,Tob1,Grina,Itga9,P4ha2,Ascc1,Cables1,Ccdc80,C330006K01Rik,Atg4a,Mlycd,Tollip,Mustn1,Pcbd2,Cxcr7,Ptprm,Sema3f,Ckm,Scarb2
**mmu-miR-715**	31(776)	0,000194854	Ppp1r16a,Thra,Dtx2,Adra1b,Ecm1,Nt5m,Wnt9a,Abcb6,Hist2h2bb,Atp6v1e1,Trappc6a,Ank2,F630110N24Rik,Btf3l4,BC027231,Skap2,Ly6c1,Npdc1,Ahnak,Klf2,Kif13b,Ssbp2,Slc25a39,Zdhhc24,Gpr39,Acaa1a,Igfbp2,1810030N24Rik,Oplah,Ltbp3,Uchl1
**mmu-miR-30b***	30(776)	0,00019849	Pank2,Gpx4,R74862,Dusp3,Sgcb,Dhrs1,Hexb,Ankra2,Gpr133,Alkbh6,Ero1l,Grina,Farp2,Nme2,Idua,Hist1h1e,Anxa1,Hbxip,Trak1,Ctsb,1300010F03Rik,Asb1,Nr2c1,Fbxl20,Fbn2,Adamtsl4,Ckm,Slc25a35,Igfbp2,Cacna1b
**mmu-let-7i***	31(776)	0,000203429	Lgr4,Ascc2,Dhrs3,Ehd1,Abcb6,Cyp4v3,Gadd45b,Upk3b,Atp6v1e1,Tgfb2,Renbp,Armc8,Gramd3,Ccny,Anxa3,Cacna1g,Grina,Gdf15,Snta1,Mocs2,Adamtsl5,Pxmp4,Gpc4,1300010F03Rik,Cog6,Cox6a2,Hadhb,Slamf9,Cpeb1,Wdr7,Lrsam1
**mmu-miR-130b**	33(776)	0,000204417	Dym,Dtx2,Map3k8,Ubl4,Sh3bgr,Elmo2,Habp4,Stk39,Gadd45b,Atp9b,D6Wsu163e,Btf3l4,Mrgprf,Sgcb,Ankra2,Myom2,Ndel1,Grina,Ensa,Vdac1,Akap4,Plcb1,Ankrd29,Adamts2,Tanc2,Chchd6,Acyp2,Chmp4c,Hadhb,Tpp1,Tmem9b,Ankrd44,3110002H16Rik
**mmu-miR-293**	30(776)	0,000205473	Stmn3,Birc7,Hexa,Dynlrb1,Crybb3,Rhbdd1,Tmem87b,Ankra2,Myom2,Lgals9,Ccdc64,Farp2,Epb4.1l4b,Impdh1,Mfap5,Glt8d1,Bbs5,Ing4,Srp14,Slc39a14,Asb1,Ssbp2,Ikbkg,Htatip2,Acaa1a,Tom1l2,Cpeb1,2610019F03Rik,Chst12,Yif1a
**mmu-miR-409-5p**	32(776)	0,000205734	Ahnak2,Dtx2,Sqrdl,Commd8,Prss29,Gpx4,Alg14,5930434B04Rik,Habp4,Rassf3,Nudt13,Creb3,Tgfb2,Ank2,Tbcd,1810020D17Rik,BC027231,Gpr133,Galns,Wisp2,Rora,Shank3,2700078K21Rik,P4ha2,Serpinb9c,Mfap5,1810037I17Rik,2900026A02Rik,Cdkn1a,6330406I15Rik,Wdr7,Pcsk6
**mmu-miR-191**	29(776)	0,000208278	1300014I06Rik,Stmn3,Plb1,Kremen1,Fbxo9,Atp9b,D6Wsu163e,Cd248,Vps39,Gramd3,Anxa3,Dlg2,Zbtb4,Slc9a6,Tom1,Ppt2,Glt8d1,Pxmp4,Ctsl,Pdia5,Rab7l1,Ssbp2,Ptprm,Kif3a,Slamf9,Tubb2a,Satb1,Uchl1,Chpt1
**mmu-miR-142-3p**	30(776)	0,000225564	Ahnak2,Alg14,Rras,Inpp5a,Atp6v1e1,Creb3,Dusp3,Abcd1,Tmem87b,Ttc15,Qpct,Dusp8,Lrrc57,Glt8d1,Adar,Bbs5,1300010F03Rik,Def8,Pdia5,Acyp2,Il17d,Fzd8,Nr2c1,Vps24,Cdh7,Htatip2,Acaa1a,Tbc1d9b,Cyb5r3,Hint3
**mmu-let-7b**	34(776)	0,000226018	Ppp1r16a,Dtx2,Col14a1,Mcee,Rab4b,Rras,R74862,Prss36,Cyp4v3,Trappc6a,Akap13,Vps8,Gng5,Pigz,Lama2,Bmp4,Tcn2,Itgb1bp1,Chrd,Lnx1,Slc35b1,Ppt2,Glt8d1,Fabp3,Os9,Ing4,Gdap2,2010111I01Rik,Isca2,BC029214,Sema3f,1110034G24Rik,1810046J19Rik,Slamf9
**mmu-miR-19b**	31(776)	0,00022638	Snrpn,Mpv17,Sh3bgr,Wnt9a,Atp6v1e1,Btf3l4,Armc8,Skap2,Ankra2,Mkrn1,Suox,Rora,Slc9a6,Impdh1,St3gal5,Glt8d1,Itpr2,Ankrd29,Ahnak,Atp5s,Ptprm,Itfg3,Kif3a,Hadhb,Igbp1,1500011K16Rik,Scn1b,Ltbp3,Vps29,Hhip,Atp10d
**mmu-miR-721**	32(776)	0,000227093	Dym,Dtx2,Rab43,Cd81,Stk39,Gadd45b,D6Wsu163e,Adcy9,Sgcb,Armc8,Ankra2,Myom2,Ndel1,Dlg2,Ensa,Mfap5,Htra3,2310046K01Rik,Akap4,Mtcp1,Acyp2,Tbccd1,Ptprm,Hadhb,Tpp1,Slamf9,1500011K16Rik,Cpeb1,Vps29,Tmem9b,Ankrd44,3110002H16Rik
**mmu-miR-27b***	27(776)	0,00023078	Bri3,Tlr6,Insig2,Sema3b,Cobl,AI593442,Trappc6a,2010003O02Rik,Bphl,Ascc1,Dusp8,Lrrc57,Iscu,Cdk5,Isca2,Zfand2b,Nr2c1,1110034G24Rik,Fbn2,Mras,Cbara1,Ckm,Scarb2,Igfbp2,Dusp22,Mtus1,3110002H16Rik
**mmu-miR-615-3p**	32(776)	0,000231823	Mrc2,Thra,Yif1b,Mpv17,Prss29,Rras,Smad6,Rassf3,Trpv4,Trappc6a,Ank2,Txnrd3,Tob1,Arl2,Ypel3,Shank3,Pbxip1,Ppt2,Lims2,Rell2,Bbs5,BC089491,Srp14,Atp6v0b,Zfand2b,Tanc2,Ptdss1,Vps24,Itfg3,Esrrb,C1qtnf1,Dmpk
**mmu-miR-101b**	34(776)	0,00025016	Ppp1r16a,Dym,Zfand3,Ap3s1,Dtx2,Map3k8,Sgca,Mcee,Trpc7,Smpdl3a,Rab38,Napb,Abcb6,Stam2,Ppapdc3,Ccdc64,Ndufa10,Mtap6,Ascc1,Mfap5,Mlycd,Ing4,Dopey2,Necap2,Srp14,Serf2,Htatip2,Ndrg4,Acaa1a,Cdkn2b,Tbc1d14,Fzd6,Atp6v0a1,Tubb2a
**mmu-miR-505**	30(776)	0,000264035	Fbxo9,Tmem135,Wnt9a,Cyp4v3,Vps39,Tgfb2,Rhbdd1,9430038I01Rik,Ap3m2,2900010M23Rik,4933439F18Rik,Lrrc57,Ulk2,Itpr2,Gdap2,Atg7,Plcb1,Gpc4,Ctsl,Atp6v0b,Atg12,Cyb5,Fzd8,Hck,1810027O10Rik,Mras,Tyrp1,Igfbp2,Fbxw4,Tubb2a
**mmu-miR-873**	31(776)	0,000267998	Hspa12a,Ascc2,Selm,Cyp27a1,Rab38,AI593442,Ppapdc3,Adcy9,Pld1,Arnt,Camk1,Sepw1,Myom2,Appl2,Nme2,Shank3,Slc9a6,P4ha2,Cables1,9130011J15Rik,S100a16,Mtcp1,Nol3,Tanc2,Tbccd1,Ssbp2,Hr,Ufc1,Hadhb,Uchl1,Dmpk
**mmu-let-7d**	33(776)	0,000284971	Ppp1r16a,Ifi35,Bri3,Mcee,Tmem41a,Rab4b,Alg14,Hexa,Sh3bgr,Rras,Elmo2,Atp6v0e,R74862,Cyp4v3,Trappc6a,Akap13,Gng5,Lmbr1,Camk1,Suox,Galns,Chrd,Slc35b1,Fabp3,Gdap2,Ugt2b36,Ikbkg,1110034G24Rik,1810046J19Rik,Kif3a,Slc25a35,Wscd2,Tubb2a

miRNA name	*Number of genes*	*Corrected p-value*	*miRNA targets among genes induced in Rasless cells (from Table S1)*
**mmu-miR-140***	29(776)	0,000289688	Usp2,Slc37a4,Higd1a,Adamts5,Serpinb6c,Sepw1,Trappc2l,Guk1,Ndel1,Ascc1,Ehd3,Glt8d1,Slc35c2,Exoc3,Mlycd,S100a16,4930523C07Rik,BC029214,Il17d,Tex264,Cxcr7,Fbxl20,Htatip2,1810046J19Rik,Stx7,Tbc1d14,Dusp22,Cacna1b,1700094D03Rik
**mmu-miR-182**	31(776)	0,000292629	Wdr47,Slc37a4,Ascc2,Gyg,S100a1,Papss2,Nt5m,Cobl,Mtap1a,Atp6v0e,Inpp5a,Nudt13,Copz2,Pld1,Tob1,Grina,Itgb1bp1,Lnx1,L1cam,Deb1,Tollip,Npdc1,Srp14,Galc,Rab7l1,Ugt2b36,6530401N04Rik,Asb1,Sema3f,Vps24,Slc2a8
**mmu-miR-872**	28(776)	0,000301517	Iah1,Sqrdl,Col14a1,Plb1,Gyg,Arvcf,Trpc7,Rab40b,Tgfb2,Serpinb6c,Slc7a7,BC027231,Ankra2,Cacna1g,Rasl12,Suox,Gstk1,Aldh6a1,Iscu,Ccdc80,4930523C07Rik,A930005H10Rik,Tbccd1,Nr2c1,Nhlrc1,1810030N24Rik,Wdr7,Hint3
**mmu-let-7c**	34(776)	0,000314159	Ppp1r16a,Dtx2,Mcee,Tmem41a,Rab4b,Alg14,Sh3bgr,Rras,R74862,Cyp4v3,Trappc6a,Akap13,Gng5,Lama2,Camk1,Galns,Tcn2,Chrd,Lnx1,Slc35b1,Ttll7,Fabp3,Ing4,Gdap2,Tollip,Isca2,BC029214,Tex264,Ikbkg,Sema3f,1110034G24Rik,6330406I15Rik,1810046J19Rik,Slamf9
**mmu-miR-677**	28(776)	0,000315566	Col14a1,Glrb,Hexa,Sh3bgr,Rras,Lyrm1,Dhrs1,Skap2,Slc46a3,Rragc,Guk1,Hsd3b3,Ascc1,Mfap5,Ccdc80,Glt8d1,Fabp3,Ulk2,Os9,Pde6d,Ahnak,Tanc2,Acyp2,Fbn2,Cog6,Slc25a35,Slamf9,Reck
**mmu-miR-708***	25(776)	0,000322409	1810058I24Rik,Gpx4,Tlr6,Abcb6,Stk39,Iqsec2,1110007C09Rik,Cacna1g,Serpinb9b,Glt8d1,Psen2,Trak1,Zfand2b,Slc39a14,Ssbp2,Cxcr7,Slc2a6,Tuba8,Cpeb1,Tbc1d9b,Tmem38a,Satb1,Uchl1,1700094D03Rik,Aldh3a2
**mmu-let-7g**	33(776)	0,000326447	Ppp1r16a,Dtx2,Mcee,Sh3bgr,Rras,R74862,Cyp4v3,Trappc6a,Akap13,Gng5,Skap2,Ankra2,Gstk1,Itgb1bp1,Chrd,Slc35b1,Ttll7,Fabp3,Rell2,Mtm1,Isca2,BC029214,App,Ahnak,Zdhhc24,Ptprm,1110034G24Rik,6330406I15Rik,Kif3a,Tom1l2,Tpp1,Wscd2,Vps29
**mmu-miR-34a**	34(776)	0,000361235	Fbxo9,Hexa,Rras,Nudt13,Nfs1,1700009P17Rik,Ttc15,Rab3gap2,Tpcn1,Suox,Nfe2l1,Pbxip1,Nrip3,Deb1,Rell2,Serinc3,Atg7,BC089491,Mtcp1,Mustn1,Triap1,Mtm1,Ankrd29,Pde4dip,Slco3a1,Acyp2,Tex264,Ikbkg,Nhlrc1,Wscd2,Rhoj,Scn1b,Lmna,1700094D03Rik
**mmu-miR-342-5p**	30(776)	0,000362152	Sgca,Hexa,D16H22S680E,Slc16a9,Tbcd,Armc8,Slc44a1,Ccdc64,L1cam,Lims2,Ccdc80,Lrch1,S100a13,Os9,Ing4,Pltp,Isca2,Gabarap,Slc2a6,Hr,Ikbkg,Tom1l2,Atp6v0a1,Tbc1d9b,Chst12,Tubb2a,Oplah,Dmpk,Fads3,Aph1a
**mmu-miR-503**	30(776)	0,000368143	Pank2,Ahnak2,Thra,Usp2,Sgca,Kremen1,Arvcf,Rab4b,Higd1a,Atp6v1e1,Lyrm1,Pigz,Arl2,C1qtnf6,Rapsn,Ppt2,Ttll7,2310046K01Rik,Mlycd,S100a16,Srp14,1300010F03Rik,BC029214,Zfand2b,Slc25a39,Cbara1,Ckm,Dnajc4,Rabac1,Dmpk
**mmu-miR-200b**	33(776)	0,000369487	Pank2,Ugp2,Wdr47,Plb1,Crot,Lrrc51,Atp6v0e,Krt80,Stam2,Fn1,Serpinb6c,Slc35a2,Akap13,Slc44a1,Nipa1,Aldh6a1,P4ha2,St3gal5,Dusp8,Nrip3,Ccdc80,Tmem154,Ly6c1,Slc25a39,Acyp1,Scarb2,Slamf9,1500011K16Rik,Reck,Wdr7,Tubb2a,Npc1,Ankrd44
**mmu-miR-217**	29(776)	0,000374805	Zfp78,Kctd9,Col14a1,Magi3,Fbln5,Glrb,Rbms2,Emilin2,Fn1,Atp9b,Lyplal1,Skap2,Slc46a3,Fndc3b,Ttc15,Grik2,Iscu,Tmem154,Sned1,Dbp,Adamts2,Slc26a11,Sstr4,Tanc2,Asb1,Uqcrq,Kif3a,Acaa1a,Fbxw4
**mmu-miR-671-3p**	32(776)	0,000376395	Ppp1r16a,Ugp2,Kremen1,Trpc7,Krt80,Nudt13,Vps39,Copz2,Renbp,Pigz,Clip1,Emp2,Gstk1,Zbtb4,Cspg4,C1qtnf6,Ttll7,Sned1,Sorbs3,Mrpl55,Klf2,Kif13b,6530401N04Rik,Tex264,BC004004,Dhps,Scarb2,Tom1l2,Slc25a35,Igfbp2,Igbp1,Zfp704
**mmu-miR-675-5p**	32(776)	0,000376395	Yif1b,Bri3,Sgca,Slc37a4,Hadha,Kremen1,Atp6v0e,Cd81,Clstn1,Trpv4,Upk3b,Crybb3,Dusp3,Renbp,Myo6,Guk1,Tcn2,Rapsn,Dusp8,Tmem175,Rell2,Dner,Pltp,Srp14,Atp6v0b,1700016M24Rik,Tex264,Cdkn1a,Dhps,Atp6v0a1,Cpeb1,Camkk1
**mmu-miR-654-3p**	31(776)	0,000381689	Zfand3,Iah1,Kctd9,Ccdc126,Rab43,Commd8,Arvcf,Alg14,5930434B04Rik,Prss36,Tbcd,Akap13,Pld1,Vps8,Btf3l4,Tmem87b,Mapk4,Arhgef12,Shank3,Grik2,Nrip3,Mfap5,Rell2,Bbs5,Nol3,Dbp,Isca2,Tanc2,Cox6a2,Prelp,1700021C14Rik
**mmu-miR-433**	28(776)	0,000393196	Tssc4,Stmn3,Magi3,Dhrs3,Smpdl3a,Napb,Adamts5,Sync,Upk3b,F630110N24Rik,Tbcd,Slc44a1,Galns,Herpud2,Arl2,Lnx1,Hbxip,Bbs5,Vnn1,Mustn1,Dbp,Ctsl,Slc39a14,Ahnak,Bcmo1,Mxra7,Cox6b1,Zfp704
**mmu-miR-701**	28(776)	0,000401996	Slc37a4,Plb1,Samd8,Cobl,Fem1a,R74862,Smad6,Qk,Mxra8,9430038I01Rik,Cacna1g,Synpo,Zbtb4,Rapsn,P4ha2,Idua,Mlycd,Itpr2,Dopey2,BC089491,Gpc4,Slc41a3,Ugt2b36,Glipr1,Fbxl20,1110034G24Rik,6330406I15Rik,Igfbp2
**mmu-miR-128a**	31(776)	0,000415365	Ppp1r16a,Dym,Hadha,Rras,Smad6,Klf15,Nfs1,Sgcb,Clip1,Skap2,Guk1,Ypel3,Ascc1,Nrip3,Rell2,Psen2,Mlycd,Akap4,Sh2d4a,Grk4,Ankrd29,Npdc1,Galc,Il17d,Fbxl20,Sh3tc2,Tyrp1,1810046J19Rik,1810030N24Rik,Chst12,1700094D03Rik
**mmu-miR-667**	30(776)	0,000443319	Dtx2,Tssc4,Plb1,Trpc7,Sh3bgr,Creb3,Vps39,Iqsec2,BC027231,Arl2,Zbtb4,Tom1,P4ha2,Nudt7,Lims2,Zmat2,Ing4,Triap1,Necap2,Abhd5,Rab7l1,Cxcr7,Sema3f,Slc25a35,Ankrd12,Igfbp2,Tbc1d9b,Tmem38a,Limch1,Oplah
**mmu-miR-770-5p**	30(776)	0,000452461	Rpl17,Adc,Gyg,Tmem41a,Abcb6,Stam2,Tbcd,Adcy9,1810020D17Rik,Klk1b4,Dhrs1,Slc31a2,Cdc42ep2,Myo6,Chrd,Itga9,Ascc1,Idua,Mocs2,Pik3ip1,Ccdc80,Ing4,Sned1,Atp6v0b,Kif13b,6530401N04Rik,Tex264,Ndrg4,Igbp1,Cpeb1

miRNA name	Number of genes	Corrected p-value	miRNA targets among genes induced in Rasless cells (from Table S1)
**mmu-miR-433***	23(776)	0,000467081	Col14a1,Rab43,Adc,Stam2,Skap2,Arnt,Dok4,Ccdc64,Galns,Rora,Serpinb9c,Serpinb9b,Deb1,Hbxip,Mlycd,Ing4,Tollip,Srr,Sparc,Sh3tc2,Cdkn2b,Gas6,Yif1a
**mmu-miR-298**	27(776)	0,000472277	Dtx2,Rab4b,Elmo2,Atp6v0e,Gadd45b,Creb3,Slc35a2,Pigz,Suox,Galns,Arhgef12,Fabp3,Ing4,Mustn1,Srp14,1300010F03Rik,Klf2,Kif13b,Mxra7,Fbxl20,Cdkn1a,1810046J19Rik,Tbc1d9b,Lmna,Tubb2a,Cox6b1,2810428I15Rik
**mmu-miR-713**	28(776)	0,000472577	Dtx2,2310028O11Rik,Ecm1,Rpl17,Commd8,Crot,Insig2,Lrrc51,Cd81,Prss36,Sync,Vps8,Mrgprf,Anxa3,Tpcn1,Arl2,Wisp2,Iscu,Ccdc80,Zmat2,Ankrd29,2010111I01Rik,Cryab,Lrba,Ctsl,Slc26a11,Hck,Sh3tc2
**mmu-miR-547**	28(776)	0,000480899	Tcta,Magi3,D16H22S680E,Cyp4v3,Cd248,Tbcd,Mrgprf,5830405N20Rik,Ccdc64,Suox,Zbtb4,Slc9a6,Cables1,Adar,Itpr2,Nol3,Pxmp4,0610037L13Rik,Mrpl55,Tex264,Nr2c1,Vamp3,Vps24,Cdh7,Gpr39,Tom1l2,Cacna1b,3110002H16Rik
**mmu-miR-465a-3p**	31(776)	0,000482604	Zfand3,Ccdc126,Slc37a4,Adc,9030409G11Rik,Tlr6,Nt5m,Kcnn1,Caml,D6Wsu163e,Vps39,Dusp3,Anxa3,Arnt,Myo6,Ypel3,Rora,Rapsn,Lrrc57,Grik2,Adamtsl5,Naga,Grk4,2010111I01Rik,Ptprm,Cdh7,Clcn7,Cox6a2,Rhoj,Ltbp3,Yif1a
**mmu-miR-207**	25(776)	0,000482645	Thra,Adra1b,Sgca,Sh3bgr,Gadd45b,F630110N24Rik,Skap2,Alkbh6,Lgals9,Chrd,Slc9a6,Nfe2l1,Cspg4,Snta1,Ehd3,Cables1,Rell2,Nupr1,BC089491,Nenf,Hgsnat,Zfand2b,Mras,2900026A02Rik,Igfbp2
**mmu-miR-218**	28(776)	0,000483348	Commd8,Lgr4,Mpv17,Crot,Glrb,Adamts5,Stam2,Slc44a1,Cacna1g,Pdgfc,Tob1,Herpud2,Hsd3b3,Slc9a6,Mocs2,Ehd3,Htra3,Nkiras2,Mtcp1,Zfand2b,Acyp2,Il17d,Ptprm,Csrp2,Cox6a2,Slc25a35,1810030N24Rik,Tmub2
**mmu-miR-215**	29(776)	0,00048354	Tssc4,Ubl4,Prss29,Fbxo9,Ppapdc3,Caml,Clip1,Slc31a2,AI317395,Hoxa1,Pdgfc,Epb4.1l4b,Snta1,Lrrc57,Garnl3,9130011J15Rik,Hbxip,Atg4a,Ly6c1,Ing4,Mrpl55,Pcbd2,Sparc,Cog6,Ankrd12,Tbc1d14,Slc2a8,Npc1,Shroom3
**mmu-miR-467a**	34(776)	0,00048374	Ugp2,Magi3,Coq10a,Alg14,Hexa,Sh3bgr,Stam2,Clstn1,Hdlbp,Trpv4,Armc8,Ttc15,Mkrn1,Farp2,Nudt7,Dusp8,Serpinb9b,Ttll7,Htra3,Slc22a4,Bbs5,Trak1,Ntn4,Vnn1,Dopey2,2010111I01Rik,Ikbkg,Ufc1,Cdkn1a,Tom1l2,1700021C14Rik,Oplah,Dusp22,1700094D03Rik
**mmu-miR-487b**	29(776)	0,000487702	Dtx2,Rnf13,Kif27,Zbtb7c,Ascc2,Cyp27a1,Cd81,Cyp4v3,Rhbdd1,Skap2,Mxra8,Hoxa1,Tob1,Qpct,Lnx1,Mocs2,Asah1,Anxa1,Slc6a8,Sema3c,Slc26a11,Pcbd2,Rab7l1,Tbccd1,Acyp1,Cog6,Adamtsl4,Ankrd12,Immp1l
**mmu-miR-199a-5p**	32(776)	0,000507975	Ppp1r16a,Tssc4,Sgca,Fbxo9,Hexa,Sh3bgr,Sync,Iqsec2,Pld1,Pigz,Ndel1,Pik3ip1,Tmem175,Akap4,Grk4,Necap2,Def8,Tanc2,Tbccd1,Slc2a6,BC004004,Sparc,Csrp2,Daglb,Tom1l2,9930013L23Rik,Igfbp2,Gas6,Fbxw4,Atp6v0a1,Chst12,Cyb5r3
**mmu-miR-324-5p**	32(776)	0,000517689	Thra,Copg,Sgca,Cox4nb,Ascc2,Mpv17,Tcta,Clstn1,Cd248,F630110N24Rik,Tbcd,Slc7a7,Abcd1,1700009P17Rik,Suox,Chrd,Tom1,Nfe2l1,Myeov2,Psen2,Mlycd,Vdac1,Nkiras2,Triap1,Plcb1,Galc,App,Slco3a1,Nr2c1,Chmp4c,Acaa1a,Slc2a8
**mmu-let-7a***	30(776)	0,0005211	Zfand3,Map3k8,Bri3,Slc37a4,Ascc2,Cobl,Sh3bgr,Caml,Atp9b,Btf3l4,Anxa3,Skap2,Myom2,Gdf15,Tom1,Snta1,St3gal5,Serpinb9c,Pon3,Plcb1,Ctsl,Klf2,Tbccd1,Ssbp2,Sema3f,Ufc1,Wdr7,Dusp22,Zfp704,Cacna1b
**mmu-miR-709**	36(776)	0,000539647	Acp2,Arvcf,Rab4b,1810058I24Rik,Nt5m,Hexa,Elmo2,Krt80,Nudt13,Trappc6a,Akap13,Vps8,Pigz,Cdc42ep2,Stx8,Tpcn1,Trim36,Suox,Itgb1bp1,Impdh1,2700078K21Rik,Rapsn,Mmaa,Pik3ip1,Adamtsl5,Os9,Ing4,Mtm1,1300010F03Rik,Ahnak,Hr,Vamp3,Mxra7,Sema3f,Slc25a35,Agl
**mmu-let-7b***	30(776)	0,00054026	Zfand3,Dtx2,Bri3,Slc37a4,Ascc2,Sh3bgr,Abcb6,AI593442,Atp9b,Akap13,Btf3l4,Anxa3,Skap2,Myom2,Gdf15,Snta1,Fabp3,9130011J15Rik,Plcb1,Ctsl,App,Klf2,Pcbd2,Tbccd1,Sema3f,Ufc1,Ckm,Wdr7,Dusp22,Zfp704
**mmu-miR-147**	29(776)	0,000541125	Yif1b,Copg,Pef1,Trpc7,Alg14,Elmo2,Atp6v0e,Emilin2,Dscr3,AI593442,Upk3b,Atp9b,Lamb2,Vps8,Gabarapl1,Anxa3,Synpo,Chrd,Serpinb9b,Nupr1,Nol3,Pde6d,Pltp,Npdc1,2010111I01Rik,Cox6a2,Gas6,Tbc1d9b,Cyb5r3
**mmu-miR-329**	29(776)	0,000548041	Ahnak2,Dtx2,Adra1b,Stmn3,Fuca2,Nt5m,Fem1a,Clstn1,Smad6,Ank2,Slc7a7,Pld1,Tmem87b,Ttc15,Atp2a3,Synpo,Rora,Mlycd,Slc22a4,Os9,Sned1,4930523C07Rik,Def8,Ahnak,Tbccd1,Acaa1a,Igfbp2,Dusp22,Mtus1
**mmu-miR-200c***	25(776)	0,000548321	Maob,Zhx1,Krt80,Ank2,Slc44a1,2900010M23Rik,Chrd,Cables1,Mmaa,Tmem175,Slc22a4,Ly6c1,Srp14,Tanc2,Gstp1,Htatip2,Sh3tc2,Acaa1a,Tom1l2,Cdkn2b,Wscd2,Atp6v0a1,Mdfic,Chst12,Uchl1
**mmu-miR-378***	25(776)	0,000559282	Thra,Tssc4,Usp2,Sgca,Atp6v0e,Crybb3,Vps8,Armc8,Camk1,Cdc42ep2,Calml4,Lgals9,Shank3,Ascc1,Lrrc57,Tmem154,Mtcp1,Srr,Spata21,Atp5s,Nr2c1,Chmp4c,Cdkn1a,9930013L23Rik,Zfp704
**mmu-miR-196b**	32(776)	0,000574965	Pank2,Crat,Dtx2,Col14a1,Plb1,Upk3b,Atp6v1e1,Dynlrb1,Vps8,Gng5,Lmbr1,Lama2,9430038I01Rik,Alkbh6,Ttc15,2900010M23Rik,Arl2,Slc9a6,Cspg4,Myeov2,Cdk5,Deb1,Anxa1,Pde4dip,Def8,Tex264,Uqcrq,Cpa6,ORF63,Csrp2,Mras,Tyrp1
**mmu-miR-876-3p**	30(776)	0,000596786	Rnf13,Snap91,Gnptg,Pmp22,Lgr4,Fuca2,Crot,Wnt9a,Gng5,Anxa3,Arnt,Alkbh6,Rab3gap2,Tpcn1,2010003O02Rik,Myeov2,Glt8d1,Adamtsl5,Ly6c1,Mustn1,Slco3a1,Pcbd2,Cyb5,Cpa6,Acyp1,Mras,Cdkn2b,Cyb5r3,Cox6b1,Immp1l
**mmu-miR-302d**	31(776)	0,000601944	Maob,Papss2,Ehd1,Emilin2,Loxl1,Crybb3,Dhrs1,Rragc,2010003O02Rik,Nudt7,2700078E11Rik,Serpinb9b,9130011J15Rik,Os9,Serinc3,Triap1,Pde4dip,1810037I17Rik,Clip4,Rab7l1,Nr2c1,Csrp2,Mras,Sh3tc2,Cdkn1a,Cpeb1,Wdr7,Uchl1,Zfp704,1700094D03Rik,Ankrd44

miRNA name	*Number of genes*	*Corrected p-value*	*miRNA targets among genes induced in Rasless cells (from Table S1)*
**mmu-miR-190**	26(776)	0,000602075	Mcee,Ctbs,Cd81,BC027231,Lama2,Rab3gap2,Grik2,Dopey2,Mtcp1,Tollip,Plcb1,Mtm1,Cryab,Isca2,Glo1,Cyb5,Ugt2b36,Cdh7,Acyp1,Cox6a2,Gas6,Chst12,Tmem50b,Hint3,Immp1l,Mtus1
**mmu-miR-129-3p**	29(776)	0,000604891	Kif27,Tbc1d10a,Sync,Nudt13,Clip1,Lgals9,Arl2,Tom1,St3gal5,Mocs2,Nrip3,Pik3ip1,Ppm1h,Tmem175,Atg7,Dopey2,BC089491,Cryab,Lrp4,Ahnak,Clip4,Ikbkg,Sema3f,Fbxl20,Cox6a2,Gas6,Rabac1,Fads3,Shroom3
**mmu-miR-759**	27(776)	0,000618404	Ugp2,Sgca,Stmn3,Camk4,Mpv17,Smpdl3a,Atp6v0e,Cd81,Smad6,Ank2,Crybb3,Copz2,Mrgprf,Gramd3,Lmbr1,Dhrs1,2010003O02Rik,Rasl12,St3gal5,Tmem175,Hbxip,Slc26a11,Gabarap,Cog6,Cox6a2,1500011K16Rik,Immp1l
**mmu-miR-1**	27(776)	0,000618404	Sh3bgr,Mtap1a,Abcb6,Fn1,Gadd45b,D6Wsu163e,Ank2,Btf3l4,Sgcb,Dgkg,Lmbr1,Hexb,2010003O02Rik,P4ha2,Lrch1,Dopey2,Pde6d,Ankrd29,App,Def8,Vps24,Fbn2,Dhps,Kif3a,Cpeb1,Hhip,Npc1
**mmu-miR-135a***	25(776)	0,000619559	Usp2,Stmn3,Slc37a4,Mpv17,S100a1,Lactb2,Rab3d,Amhr2,Slc46a3,Arnt,Bmp4,Gstm2,Arl2,Ppt2,Glt8d1,Os9,0610037L13Rik,Chchd6,Ptprm,Ufc1,Gpr39,Slc25a35,Igfbp2,Rabac1,Vps29
**mmu-miR-488***	24(776)	0,000620167	Sgca,Stmn3,Smpdl3a,Cyp27a1,Mrgprf,Galns,C1qtnf6,Dusp8,Nrip3,Ly6c1,Hgsnat,Mrpl55,Cyb5,Kif13b,Tex264,Serf2,Cog6,Csrp2,Cdkn1a,Cox6a2,Igfbp2,Wdr7,Ltbp3,1700094D03Rik
**mmu-miR-92b**	32(776)	0,000635514	Ugp2,Sgca,Rab43,Lgr4,Nt5m,Hexa,Adamts5,Smad6,Serpinb6c,Slc35a2,Dgkg,Gramd3,Anxa3,Slc31a2,Gstm2,Herpud2,Riok3,Asah1,Tmem154,Ing4,Gdap2,Mustn1,Sirt2,Klf2,Asb1,Fbn2,Dnajc4,Cdkn2b,Igbp1,Dusp22,Atp10d,Mcl1
**mmu-miR-30c**	31(776)	0,00064235	Ppp1r16a,Pank2,Fech,Tmem41a,Magi3,Vps39,Ank2,Vps8,Gramd3,Emp2,Ankra2,5830405N20Rik,Hoxa1,P4ha2,Ccdc80,Anxa1,Tmem175,Psen2,Vdac1,Bbs5,Ankrd29,Frmpd1,Atg12,Ahnak,Pcbd2,Fbxl20,Slc41a2,Afap1l2,Fbxw4,Wdr7,Chst12
**mmu-miR-380-3p**	26(776)	0,000663506	Oit3,Wdr47,Commd8,Alg14,Rbms2,Prss36,Dusp3,Slc7a7,Ankra2,Rab3gap2,Qpct,Nipa1,Alas1,Slc9a6,Cables1,Ing4,Frmpd1,Lrba,App,Cyb5,Il17d,Chmp4c,Cdh7,Clcn7,Gas6,Pcsk6
**mmu-miR-300***	23(776)	0,000664005	Adra1b,Adc,Mpv17,Ehd1,Ppapdc3,Atp9b,Slc35a2,Tbc1d22a,Gng5,2900010M23Rik,2010003O02Rik,Arl2,Nme2,Serpinb9c,Ccdc80,Psen2,Ing4,Tollip,4930523C07Rik,Cyb5,Acyp2,Acyp1,Cpeb1
**mmu-miR-708**	26(776)	0,000675953	Pank2,Thra,Dtx2,Stmn3,Kif27,Tmem41a,Lamb2,Copz2,Renbp,Lmbr1,Clip1,Sepw1,Ndel1,Itgb1bp1,Wwp2,Ppt2,Lrrc57,Cdk5,9130011J15Rik,4930523C07Rik,Srr,Def8,Slc39a14,Clip4,Fbxw4,Cacna1b
**mmu-miR-295***	21(776)	0,000693655	Fech,Copg,Fem1a,Napb,Slc6a6,Nudt13,Vps8,Arnt,Mkrn1,Mlycd,Pdia5,Gabarap,Mrpl55,Spata21,Csrp2,Mras,Dhps,Slc25a35,Cdkn2b,Igbp1,Aldh3a2
**mmu-miR-551b**	26(776)	0,000706696	Mrc2,Plb1,Hadha,Cox4nb,Prss29,Gpx4,Cd81,Trpv4,Dhrs1,9430038I01Rik,Gstm2,Arhgef12,Dusp8,Nkiras2,Ing4,S100a16,Nol3,Plcb1,Isca2,Klf2,Cog6,Rhoj,Yif1a,Atp10d,Aldh3a2,Ankrd44
**mmu-miR-18a***	23(776)	0,000713076	Pank2,Thra,Sgca,Elmo2,Ppp2r5b,Trappc6a,Iqsec2,Slc7a7,Clip1,Alkbh6,Bmp4,Tpcn1,Grina,Nfe2l1,Cdk5,Nupr1,Mtcp1,Mras,Igbp1,Tmem38a,Cyb5r3,Lmna,Yif1a
**mmu-miR-697**	28(776)	0,000730281	1300014I06Rik,Tssc4,Rpl17,Gpx4,Slc39a3,Lrrc51,Tbc1d10a,Habp4,D16H22S680E,Clstn1,Creb3,Tbcd,Akap13,Rhbdd1,Armc8,5830405N20Rik,Myo6,Repin1,Myom2,L1cam,2310046K01Rik,Mlycd,Slc22a4,Pde4dip,Ufc1,6330406I15Rik,Ltbp3,1700094D03Rik
**mmu-miR-107**	29(776)	0,000731742	Tmem41a,Rab40b,Hist2h2bb,Mtmr3,Slc35a2,Armc8,Cry2,Klk1b4,1700009P17Rik,Gstm2,Arl2,Ndufa10,Ypel3,Cspg4,Lrrc57,Fabp3,Mlycd,BC089491,0610037L13Rik,Acyp2,Asb1,Ptprm,Tuba8,Cbara1,Acaa1a,Mtss1,Afap1l2,Agl,Shroom3
**mmu-miR-21***	26(776)	0,000733255	Pank2,Ahnak2,Dym,Arvcf,Rras,Emilin2,Neu1,Tbcd,Skap2,Txnrd3,Ndufa10,Hsd3b3,Shank3,4933439F18Rik,Ppt2,Glt8d1,C330006K01Rik,Dopey2,Nol3,Ctsb,1300010F03Rik,BC029214,Ahnak,Slc25a39,Tom1l2,Tmub2
**mmu-miR-28***	23(776)	0,000743883	Mrc2,Stmn3,Cat,Kif27,Commd8,Lactb2,Gpx4,Ank2,Dhrs1,Tmco3,Arnt,Hsd3b3,Stard6,Iscu,Glt8d1,Ing4,Sned1,Slc2a6,Mxra7,BC046404,Adamtsl4,Tmem38a,Zfp704
**mmu-miR-489**	28(776)	0,000755427	Sh3bgr,Napb,Loxl1,Creb3,Amhr2,Adcy9,1700009P17Rik,Npy6r,Qpct,Tcn2,Ndufa10,Impdh1,Grik2,Cdk5,Deb1,Lrch1,1110003E01Rik,Trak1,Triap1,Srp14,Rab7l1,Il17d,Fbxl20,Cdh7,Nhlrc1,1810030N24Rik,Tmem38a,Dusp22
**mmu-miR-682**	29(776)	0,000755913	Dym,Tssc4,2310028O11Rik,Fbxo9,Sh3bgr,Armc8,Dhrs1,Skap2,Slc44a1,Npy6r,Arhgef12,Slc35c2,Psen2,Bbs5,Ing4,4930523C07Rik,Ctsb,2010111I01Rik,Atp6v0b,Tm9sf4,Kif13b,Asb1,Kif3a,Slc25a35,Wdr7,Limch1,Pcsk6,Cacna1b,Mtus1
**mmu-miR-183**	30(776)	0,000761104	Fech,Tssc4,Rpl17,Ascc2,Glrb,Npc2,Slc35a2,Gng5,Anxa3,Skap2,Ndufa10,Serpinb9b,Htra3,Deb1,C330006K01Rik,Garnl3,Psen2,Itpr2,Nkiras2,Mustn1,Pltp,Srp14,Sstr4,Tanc2,Slc41a3,Ptdss1,Hadhb,Prelp,Hint3,Chpt1
**mmu-miR-540-5p**	30(776)	0,000775872	Ppp1r16a,Sqrdl,Adra1b,Wnt9a,Upk3b,Klf15,Iqsec2,Acox1,Synpo,Guk1,Slc9a6,Nfe2l1,Pbxip1,Wwp2,Ascc1,Nudt7,Idua,Lims2,Ppm1h,Os9,Nkiras2,Ankrd29,1810037I17Rik,Mrpl55,Asb1,Ikbkg,Sh3tc2,Cyb5r3,Pcsk6,Mcl1
**mmu-miR-184**	28(776)	0,000789107	Col14a1,Zbtb7c,Tmem41a,Wnt9a,Iqsec2,Serpinb6c,Adcy9,BC027231,Slc46a3,Chrd,2700078K21Rik,Ascc1,St3gal5,Tmem175,Zfp560,Naga,Akap4,Dopey2,Ankrd29,Npdc1,BC029214,Slc39a14,Ckm,Atp6v0a1,Rabac1,Cox6b1,Uchl1,Dmpk

miRNA name	*Number of genes*	*Corrected p-value*	*miRNA targets among genes induced in Rasless cells (from Table S1)*
**mmu-miR-409-3p**	28(776)	0,000789107	Pank2,Ap3s1,Plb1,Hspa12a,Mpv17,Trpc7,Smpdl3a,Hdlbp,Ank2,F630110N24Rik,Vps8,Clip1,2010003O02Rik,Itga9,Stard6,L1cam,Mocs2,Pon3,Rdh12,Zfp560,Vnn1,Akap4,Sned1,Npdc1,Hadhb,Tpp1,Npc1,Shroom3
**mmu-miR-214***	22(776)	0,00079389	Slc37a4,Selm,Sema3b,Rhbdd1,Adcy9,BC027231,Sepw1,Repin1,Zbtb4,Bphl,Nudt7,Dusp8,Deb1,Nupr1,Naga,Ntn4,Tbccd1,Tuba8,Gpr39,Cox6a2,Atp5e,Chst12
**mmu-miR-488**	26(776)	0,0007948	Ascc2,Zdhhc4,Insig2,Sh3bgr,Sync,Caml,Slc35a2,Clip1,Slc44a1,Riok3,Nudt7,Dusp8,Nrip3,2310046K01Rik,Itpr2,Pcyt1a,A930005H10Rik,Sstr4,Cyb5,Glipr1,Fbxl20,Cpa6,Csrp2,1810046J19Rik,Cacna1b,Shroom3
**mmu-miR-34c***	24(776)	0,000796567	Ccdc126,Habp4,Fn1,Armc8,Myom2,Mkrn1,Qpct,Ndufa10,Lnx1,Slc9a6,Hist1h1e,Dusp8,Megf6,Deb1,Ly6c1,Trak1,Tollip,Pltp,Gpc4,Ahnak,Ypel5,Cdh7,1700021C14Rik,Dusp22
**mmu-miR-29a***	27(776)	0,000799097	Ap3s1,Lgr4,Birc7,Insig2,Cd81,Kcnn1,Vps39,1810020D17Rik,Anxa3,Camk1,Ndufa10,Bphl,Mocs2,Iscu,C330006K01Rik,Nupr1,Vnn1,Mtcp1,2010111I01Rik,Ahnak,Slco3a1,Usp53,Nr2c1,BC046404,Cdh7,Htatip2,Sh3tc2
**mmu-miR-546**	28(776)	0,000805365	Zfand3,Dtx2,Hspa12a,Ascc2,Prss29,Dhrs3,Nt5m,Hexa,Rras,Krt80,Klf15,Mrgprf,Pigz,Mxra8,Sepw1,Repin1,Grina,Wisp2,Isca2,Klf2,Kif13b,6530401N04Rik,Itfg3,Acyp1,Fbxw4,Tmub2,Uchl1,Vps29
**mmu-miR-685**	28(776)	0,000841677	Zfand3,Hadha,Rab4b,Abcb6,Adamts5,Nudt13,Slc7a7,Skap2,Mxra8,Slc31a2,Lgals9,Grina,Ndufa10,Arhgef12,Ppt2,Rdh12,Vdac1,Itpr2,Slc39a14,Mrpl55,Clip4,Asb1,Hck,Trp53inp2,Ckm,Kif3a,Tom1l2,Chpt1
**mmu-miR-139-3p**	29(776)	0,000872385	Zfand3,Slc37a4,Plb1,Ascc2,Mal,Emilin2,Vps8,Rarres2,Tmem87b,Sepw1,Atp2a3,Trappc2l,Myeov2,Mtap6,Wdr54,Adar,9130011J15Rik,Slc35c2,Psen2,Dopey2,Ssbp2,Uqcrq,Htatip2,Fbn2,Adamtsl4,Ckm,Afap1l2,Wscd2,Rabac1
**mmu-miR-302c***	27(776)	0,000878021	Rab43,Snrpn,Slc6a7,Tmem41a,Crot,Trpc7,Napb,Stam2,Vps8,Lama2,Ap3m2,Tob1,Slc9a6,Bphl,Mocs2,Cables1,Deb1,Tmem154,9130011J15Rik,Ing4,Triap1,Pde4dip,Slc2a6,1810027O10Rik,Atp6v0a1,Chst12,Oplah
**mmu-miR-192**	27(776)	0,000893381	Tssc4,Prss29,Gpx4,Fbxo9,Clstn1,Ppapdc3,Hdlbp,Atp6v1e1,Clip1,Ttc15,Hoxa1,Epb4.1l4b,Snta1,Lrrc57,Tmem154,Garnl3,9130011J15Rik,Atg4a,Abtb2,Ahnak,Mrpl55,Cog6,Ankrd12,Tbc1d14,Slc2a8,Npc1,Shroom3
**mmu-let-7f**	30(776)	0,000893957	Ppp1r16a,Dtx2,Mcee,Cat,Rab4b,Alg14,Hexa,Sh3bgr,Rras,Elmo2,R74862,Cyp4v3,Akap13,Gng5,Chrd,Lnx1,Slc35b1,Riok3,Ppt2,Fabp3,Gdap2,Mtm1,Isca2,App,Ugt2b36,Zdhhc24,Ikbkg,1110034G24Rik,6330406I15Rik,Kif3a
**mmu-miR-292-5p**	31(776)	0,000929655	Tssc4,Copg,Rpl17,Commd8,3830406C13Rik,Fem1a,Znrf2,Loxl1,Cd248,Crybb3,Vps8,Lhfpl2,Nipa1,Tcn2,Zbtb4,L1cam,P4ha2,Gpc4,Isca2,Gabarap,Mrpl55,Fzd8,Slc1a4,Uqcrq,Dhps,Slc2a8,Lmna,Limch1,Hint3,Shroom3,Ankrd44
**mmu-miR-330***	25(776)	0,000942361	Fbxo9,Atp9b,Cd248,Tbcd,Sepw1,Txnrd3,Mkrn1,Trappc2l,2700078K21Rik,Anxa1,Zdhhc8,Ing4,Plcb1,Ctsb,Srp14,Atg12,Sstr4,Asb1,Ptprm,Ikbkg,Cog6,Sh3tc2,Igbp1,Tbc1d9b,Yif1a
**mmu-miR-103**	29(776)	0,00095791	Tmem41a,Elmo2,Rab40b,Znrf2,Hist2h2bb,Armc8,Klk1b4,1700009P17Rik,Calml4,Ttc15,Gstm2,Cspg4,Lrrc57,Cables1,Fabp3,Mlycd,Dopey2,BC089491,0610037L13Rik,Pde4dip,Acyp2,Ptprm,Tuba8,Cbara1,Acaa1a,Mtss1,Agl,Dusp22,Shroom3
**mmu-miR-327**	27(776)	0,000993772	Ppp1r16a,Bri3,Ascc2,Sh3bgr,Rras,Cyp4v3,Sync,Cd248,Dgkg,Qk,Sepw1,Dok4,Tcn2,Ypel3,Gdf15,P4ha2,Adar,Naga,Mtcp1,Klf2,Slc41a3,Nr2c1,Ptprm,Ufc1,Ckm,Igbp1,Aldh3a2
**mmu-miR-455***	22(776)	0,00109843	S100a1,Trpc7,Gpx4,Rhbdd1,Myom2,Qpct,Herpud2,Farp2,Lnx1,Lims2,0610037L13Rik,Gabarap,Pcbd2,Tbccd1,Asb1,Sema3f,1810027O10Rik,1110034G24Rik,Slc25a35,Ankrd12,Wdr7,Zfp704
**mmu-miR-126-3p**	28(776)	0,00110112	Col14a1,Ccdc126,Crot,Smad6,Ankra2,Calml4,Myom2,Nipa1,Lnx1,A430110N23Rik,Stard6,Tom1,P4ha2,Mfap5,Lims2,Mlycd,Os9,Ing4,Mustn1,Pltp,Npdc1,Slc26a11,Cpa6,Gpr39,Tyrp1,Ankrd12,Chst12,Tubb2a
**mmu-miR-20b***	25(776)	0,0011058	Zfand3,Fbxo9,Nt5m,Sh3bgr,Caml,Vps8,Armc8,Zbtb4,Cspg4,Ehd3,Lrrc57,Tmem175,Bbs5,Ctsb,Atp6v0b,A930005H10Rik,Atp5s,Hck,Dpm2,Fbn2,Csrp2,Rabac1,Pcsk6,Vps29,Aldh3a2
**mmu-miR-699**	28(776)	0,00111904	Ifi35,2310028O11Rik,Ecm1,Fbxo9,Hexa,Sh3bgr,Rras,Nudt13,Nfs1,Trappc6a,F630110N24Rik,Tpcn1,Tom1,Pbxip1,Nrip3,Rell2,Ankrd29,Atp6v0b,Acyp2,Tex264,Slc2a6,Hr,Ptprm,Ndrg4,Cog6,Mras,Wscd2,Pcsk6
**mmu-miR-450a-3p**	29(776)	0,00112328	Iah1,Copg,Slc37a4,Plb1,Fbxo9,Adamts5,Caml,Atp9b,Loxl1,Creb3,Cd248,Copz2,Dab2ip,Lyrm1,Clip1,Sepw1,Apoa1bp,Shank3,P4ha2,Snta1,Adamtsl5,Mustn1,1300010F03Rik,Sirt2,Klf2,Sema3f,Igfbp2,Ltbp3,1700094D03Rik
**mmu-miR-541**	25(776)	0,0011507	Pank2,Slc37a4,Birc7,Prss36,Sync,Caml,Anxa3,Arnt,5830405N20Rik,Nme2,Mlycd,Sh2d4a,Adamts2,Zfand2b,Gabarap,Tanc2,Il17d,Uqcrq,Ptprm,Hck,1810046J19Rik,Scarb2,Igfbp2,Rhoj,Zfp704
**mmu-miR-15b**	30(776)	0,00119188	Dym,Bri3,Tmem41a,Rab4b,Skap2,Slc46a3,Arl2,Ypel3,Shank3,C1qtnf6,Rapsn,St3gal5,Lrrc57,Glt8d1,Mlycd,Ly6c1,S100a16,Dopey2,0610037L13Rik,Srp14,Frmpd1,Zfand2b,Def8,Asb1,Slc25a39,Nr2c1,Itfg3,Cdkn1a,Rabac1,Zfp704
**mmu-miR-202-5p**	27(776)	0,00119199	Rnf13,Tssc4,Bri3,Adra1b,Commd8,Pmp22,Smpdl3a,Higd1a,Aldh7a1,D16H22S680E,Ppapdc3,Neu1,Tgfb2,Copz2,Clip1,Slc44a1,Slc9a6,Ulk2,Atg7,Slc26a11,1300010F03Rik,Clip4,Tbccd1,Vps24,Fbxl20,Acaa1a,Tmub2
**mmu-miR-493**	27(776)	0,00119199	Sqrdl,Map3k8,Atp6v0e,Abcb6,Cd248,Pld1,Mrgprf,Clip1,Bmp4,Ypel3,Pbxip1,Hist1h1e,Dusp8,Capn5,Rdh12,Grk4,Necap2,Clip4,Usp53,Ptdss1,Ptprm,1110034G24Rik,Fbn2,Dnajc4,1810030N24Rik,Wdr7,Zfp704

miRNA name	*Number of genes*	*Corrected p-value*	*miRNA targets among genes induced in Rasless cells (from Table S1)*
**mmu-miR-679**	26(776)	0,00122364	Ppp1r16a,Rnf13,2310028O11Rik,Sgca,Plb1,Slc6a7,Pmp22,S100a1,Selm,Smpdl3a,Nt5m,Rbms2,Gpr133,Slc44a1,Rragc,Cacna1g,Rab3gap2,Herpud2,Grik2,Dopey2,Synpo2,Slc25a39,Ptprm,Sema3f,Dpm2,C1qtnf5
**mmu-miR-30a**	30(776)	0,00124605	Ppp1r16a,Fech,Tmem41a,Smpdl3a,Cyp4v3,Stk39,Ank2,Vps8,Gramd3,Pigz,Ankra2,5830405N20Rik,Dok4,P4ha2,Anxa1,Tmem175,Vdac1,Slc22a4,Frmpd1,1300010F03Rik,1810037I17Rik,Pcbd2,Ssfa2,Fbxl20,Slc41a2,Afap1l2,Fbxw4,Cpeb1,Chst12,Dusp22
**mmu-miR-33**	28(776)	0,00124837	Dtx2,Rab43,Kif27,Lgr4,Zdhhc4,Crot,Cd81,Adamts5,Dynlrb1,Anxa3,Slc46a3,9430038I01Rik,Mapk4,Herpud2,Lnx1,Aldh6a1,Bphl,Ing4,Gpc4,Slc39a14,Acyp2,Ugt2b36,Asb1,Atp5s,Fbn2,Sh3tc2,Cbara1,Hadhb
**mmu-miR-485***	23(776)	0,00125185	Adra1b,Kif27,Smpdl3a,Cyp27a1,Mtap1a,Smad6,Trpt1,Mxra8,Gstm2,L1cam,P4ha2,Deb1,Ly6c1,Ing4,Sh2d4a,Slc26a11,1300010F03Rik,Def8,Pcbd2,Rab7l1,Atp5s,Igfbp2,Atp10d
**mmu-miR-509-5p**	29(776)	0,00126382	Mrc2,Col14a1,Usp2,Mpv17,Prss29,Fbxo9,Cobl,Clstn1,Ppp2r5b,1700009P17Rik,9430038I01Rik,Pdgfc,Myeov2,2700078K21Rik,Snta1,St3gal5,Lrrc57,Rdh12,Mlycd,Ly6c1,Zmat2,Isca2,1810037I17Rik,Ptprm,Ssfa2,Ufc1,1810046J19Rik,Chst12,Satb1
**mmu-miR-302c**	29(776)	0,00126382	Maob,Nt5m,Emilin2,Cyp4v3,Loxl1,Nfs1,Skap2,Ttc15,Rragc,Arl2,Rora,Nudt7,2700078E11Rik,Serpinb9b,9130011J15Rik,Pxmp4,Impact,Atp6v0b,Zfand2b,Ahnak,Rab7l1,Synpo2,Atp5s,Ikbkg,Ufc1,Htatip2,Nhlrc1,Atp5e,Fbxw4
**mmu-miR-452**	26(776)	0,00126662	Plb1,Hadha,Insig2,Rab40b,Trappc6a,Pld1,Mrgprf,Skap2,Arnt,Ttc15,Myom2,Slc9a6,Bphl,Ascc1,Mlycd,Grk4,Hgsnat,Klf2,Pcbd2,Cyb5,Tex264,Htatip2,Fbn2,Tmub2,Uchl1,Hint3
**mmu-miR-500**	27(776)	0,0012739	Adra1b,Snrpn,Mpv17,Selm,Rab38,R74862,Smad6,Dusp3,1810020D17Rik,Sepw1,Galns,Grina,Ypel3,Sec22c,Deb1,Psen2,Nkiras2,Ing4,Npdc1,Dirc2,Asb1,Tex264,Arhgap1,Lynx1,Limch1,Hhip,Aldh3a2
**mmu-miR-145***	20(776)	0,0012962	Fech,Maob,Mpv17,Selm,Sema3b,Creb3,Vps39,Amhr2,Lgals9,Trim36,2010003O02Rik,Ppt2,Rell2,Mtcp1,Npdc1,Zfand2b,Nr2c1,Zfp704,Dmpk,Shroom3
**mmu-miR-34b-3p**	28(776)	0,00130518	Zfand3,Sqrdl,Ccdc126,Pef1,Dusp3,Bace1,Armc8,9430038I01Rik,Myom2,Mkrn1,Qpct,Ndufa10,Lnx1,Slc9a6,C1qtnf6,Dusp8,Deb1,Tmem175,Ly6c1,Tollip,Pltp,Gpc4,Necap2,Ypel5,6430548M08Rik,Atp6v0a1,1700021C14Rik,Dusp22
**mmu-miR-10a***	23(776)	0,00131503	Map3k8,Stmn3,Lgr4,Hspa12a,Atp6v0e,Hdlbp,Gramd3,Arnt,9430038I01Rik,Tmem140,Slc9a6,Mfap5,Pon3,Ly6c1,Zmat2,Pdia5,Ptprm,Cdh7,Cbara1,Fzd6,Atp6v0a1,1700094D03Rik,Immp1l
**mmu-miR-29c***	26(776)	0,00132107	Ifi35,Birc7,Tcta,Prss29,Tlr6,Higd1a,Nt5m,Hist2h2bb,Akap13,1810020D17Rik,Trpt1,Ttc15,Rora,Mtap6,Nudt7,Garnl3,Triap1,Slc39a14,4930579G22Rik,Gpr39,Nhlrc1,Slc25a35,Afap1l2,Slamf9,Camkk1,Satb1
**mmu-miR-200b***	24(776)	0,00132378	Gtf2h5,Zhx1,Smpdl3a,Dscr3,D6Wsu163e,Cd248,Sgcb,Tmco3,Rab3gap2,Galns,Ascc1,Nudt7,Tmem175,Exoc3,Slc22a4,Tanc2,Rab7l1,Il17d,Usp53,Cdkn2b,1500011K16Rik,Wdr7,Chst12,Atp10d
**mmu-miR-670**	28(776)	0,00132606	Ppp1r16a,Pank2,Plb1,Fbxo9,Rab38,Ppapdc3,Neu1,Ppp2r5b,D6Wsu163e,Adcy9,Klk1b4,Slc46a3,Dlg2,Rapsn,Psen2,Mlycd,Triap1,Pxmp4,Lrp4,Mrpl55,Sirt2,Mxra7,Chmp4c,Cdh7,Itfg3,Sh3tc2,Fbxw4,Atp10d
**mmu-miR-469**	25(776)	0,00135344	Thra,Sgca,Gpx4,Hexa,Sh3bgr,Gadd45b,Caml,Crybb3,Slc35a2,Grina,Cspg4,Impdh1,Slc35b1,Snta1,Adar,Ly6c1,Hgsnat,Zfand2b,Gabarap,4930579G22Rik,Cox6a2,Gas6,Igbp1,2810428I15Rik,Shroom3
**mmu-miR-467b**	32(776)	0,00135632	Ahnak2,Tmem41a,Coq10a,Aldh7a1,Sh3bgr,Cd81,Trpv4,Lmbr1,Ttc15,Txnrd3,Ndel1,Farp2,Pbxip1,C1qtnf6,Nudt7,Serpinb9b,Ttll7,Glt8d1,Trak1,Ntn4,Mmab,1810037I17Rik,Gabarap,Rab7l1,Csrp2,Mras,Cdkn1a,Tom1l2,Stx7,Wdr7,Oplah,Uchl1
**mmu-miR-574-3p**	29(776)	0,00136432	Acp2,Iah1,Adra1b,Rab43,Cox4nb,Ascc2,Nudt13,Loxl1,Tmco3,Ephb3,Trappc2l,Shank3,Riok3,Wwp2,Nudt7,Mocs2,Pik3ip1,Vdac1,Os9,Trak1,Nol3,Sh2d4a,App,Rab7l1,Acyp1,Cog6,Cyb5r3,Dusp22,Mtus1
**mmu-miR-199a-3p**	28(776)	0,00139056	Wdr47,2310028O11Rik,Sh3bgr,Fn1,Ank2,Amhr2,Tmem87b,Qk,Stx8,Trim36,Qpct,Chrd,Nme2,Hectd3,Glt8d1,Srr,Srp14,1300010F03Rik,Def8,Acyp2,Ugt2b36,Csrp2,Tom1l2,Fbxw4,Chst12,Cox6b1,Immp1l,Aldh3a2
**mmu-miR-696**	26(776)	0,00139515	1300014I06Rik,Pank2,Zfand3,2310028O11Rik,Usp2,Alg14,Fn1,Lamb2,D6Wsu163e,Dusp3,Tbcd,Pld1,Armc8,Gabarapl1,Slc46a3,5830405N20Rik,Pdgfc,Ccdc64,Nudt7,C330006K01Rik,Ly6c1,Slc26a11,Uchl1,1700094D03Rik,Mtus1,Chpt1
**mmu-miR-743b-3p**	27(776)	0,001434	Iah1,Stmn3,Cat,Lgr4,Ascc2,Insig2,Sh3bgr,Abcd1,Fndc3b,5830405N20Rik,Npy6r,Fbxl2,2010003O02Rik,Qpct,Stard6,Alas1,P4ha2,Asah1,Mtcp1,Grk4,1700016M24Rik,Tanc2,Vps24,Mras,Mrps14,Cpeb1,Cox6b1
**mmu-miR-758**	27(776)	0,001434	Copg,Plb1,Prss29,Cyp27a1,Wnt9a,Cyp4v3,Klf15,D6Wsu163e,Trappc6a,Tbcd,Pigz,Tob1,Ndel1,Tcn2,Slc35b1,Atg4a,Zfp560,Zmat2,Pxmp4,Srr,Tuba8,Ufc1,Fbxl20,Acaa1a,Cpeb1,Uchl1,Cacna1b
**mmu-miR-15a**	29(776)	0,00146331	Dym,Bri3,Tcta,Tmem41a,Rab4b,D16H22S680E,Rab40b,Mtmr3,Gpr133,Fbxl2,2010003O02Rik,Arl2,Ypel3,Shank3,Rapsn,Nudt7,Mlycd,Ly6c1,Sned1,Dopey2,Nol3,0610037L13Rik,Srp14,Frmpd1,Pde4dip,Zfand2b,Itfg3,Ckm,Rabac1
**mmu-miR-362-5p**	27(776)	0,00148162	Plb1,Hspa12a,Adc,Tmem41a,D16H22S680E,Cyp4v3,Prkab2,Copz2,Ndufb4,Ephb3,Mkrn1,Guk1,Ndel1,Chrd,Arhgef12,Gdf15,Psen2,Mlycd,Os9,Reep3,2010111I01Rik,2900026A02Rik,1810046J19Rik,Igbp1,Tbc1d9b,Tmub2,Ltbp3

miRNA name	*Number of genes*	*Corrected p-value*	*miRNA targets among genes induced in Rasless cells (from Table S1)*
**mmu-miR-466f-3p**	36(776)	0,00148428	Gtf2h5,Dtx2,2310028O11Rik,Map3k8,Kif27,Mpv17,Insig2,Rab3d,Dscr3,Stam2,Smad6,Acox1,Kif1c,Tmem87b,Anxa3,Tpcn1,Gstm2,Gdf15,Slc9a6,Entpd5,2700078K21Rik,Zmat2,Trak1,Nol3,Sh2d4a,Dbp,Isca2,Ctsl,Pde4dip,Slc41a3,Ssbp2,Cdh7,Adamtsl4,Tubb2a,Vps29,Mtus1
**mmu-miR-804**	24(776)	0,00148451	Dym,Iah1,Rab4b,9030409G11Rik,Nt5m,Ppapdc3,Copz2,Skap2,Suox,Guk1,Cables1,Deb1,Ing4,Pxmp4,Ahnak,Atp5s,Vps24,Ufc1,Cog6,Cpeb1,Limch1,Cox6b1,Yif1a,Mtus1
**mmu-miR-144**	28(776)	0,00151353	Ppp1r16a,Zfand3,Ugp2,Snap91,Bri3,Atp6v1e1,Anxa3,Tob1,Qpct,Lnx1,Mfap5,Htra3,Deb1,Glt8d1,Garnl3,Mlycd,Bbs5,S100a16,Abtb2,Srr,Srp14,Slco3a1,Cxcr7,Ptprm,Sh3tc2,Wdr7,Chst12,Yif1a
**mmu-miR-878-5p**	27(776)	0,00153991	Acp2,Stmn3,Slc37a4,Sh3bgr,Ppapdc3,Smad6,Cyp4v3,Nudt13,Caml,Prkab2,Dynlrb1,Ank2,Dusp3,Fstl1,Btf3l4,Skap2,Qk,Qpct,Myeov2,Man2b1,Ascc1,Dbp,Srp14,Ssfa2,Cdh7,Acyp1,Ltbp3
**mmu-miR-26a**	28(776)	0,00164008	2310028O11Rik,Sgca,Kif27,Adc,Fbxo9,Adcy9,Qk,Rab3gap2,Tob1,Nipa1,Arhgef12,Mocs2,Hectd3,Ccdc80,Nupr1,Ntn4,5530400B01Rik,Ctsl,BC029214,Ahnak,Ugt2b36,Kif13b,Tmbim1,Ptprm,Cpa6,Gpr39,Mras,Fzd6
**mmu-miR-702**	28(776)	0,00170158	Dtx2,Rab11fip5,Cd248,Kif1c,F630110N24Rik,1700009P17Rik,Clip1,Arnt,Tpcn1,Apoa1bp,Arhgef12,Rell2,Vdac1,Zmat2,Pde4dip,Tsc22d3,Slc1a4,Ikbkg,Hck,Gpr39,Mras,Cdkn1a,Cox6a2,Acaa1a,Igfbp2,Gas6,Slamf9,Stxbp1
**mmu-miR-222**	26(776)	0,00170215	Bri3,Stmn3,Sema3b,Prss36,Trappc6a,Slc7a7,Adcy9,Pld1,Vps8,Mrgprf,Skap2,Alkbh6,Txnrd3,Ypel3,Zbtb4,L1cam,C1qtnf6,Tmem175,Ulk2,Ankrd29,Zfand2b,Ahnak,Chmp4c,4930579G22Rik,Htatip2,Uchl1
**mmu-miR-592**	24(776)	0,00179626	Sqrdl,Adra1b,Ccdc126,Fahd2a,Smpdl3a,Cyp27a1,Insig2,Tbcd,Akap13,Pld1,Lmbr1,Hsd3b3,Rell2,Nkiras2,4930523C07Rik,Asb1,Tmbim1,Synpo2,Tyrp1,1810046J19Rik,Mtss1,Igfbp2,2610019F03Rik,1700094D03Rik
**mmu-miR-343**	24(776)	0,00179626	Adra1b,Sgca,Kif27,Gabarapl2,Nudt13,Myom2,Ccdc64,Ypel3,Slc9a6,Cspg4,Slc35b1,Snta1,Lrrc57,Itpr2,Ing4,Nol3,BC029214,Ptprm,Mras,1810046J19Rik,Cpeb1,Lmna,Cox6b1,Mtus1
**mmu-miR-15a***	23(776)	0,00180693	Plb1,Tlr6,Sema3b,Lrrc51,Ehd1,Mtap1a,Abcb6,Loxl1,Tcn2,Snta1,Glt8d1,C330006K01Rik,Slc22a4,Naga,Triap1,Pdia5,Rab7l1,Asb1,Sema3f,Ckm,Tmub2,Chst12,Tubb2a
**mmu-miR-704**	25(776)	0,00186189	Sgca,Cyp27a1,Abcb6,Trappc6a,Mtmr3,Slc7a7,Slc46a3,Myom2,Ccdc64,Herpud2,Farp2,Slc9a6,Ulk2,Ly6c1,Dopey2,Pxmp4,Ankrd29,Def8,Zdhhc24,Itfg3,Cog6,2900026A02Rik,Scarb2,Tbc1d14,Fads3
**mmu-miR-674***	21(776)	0,00193129	Sgca,Lrrc51,Elmo2,Nudt13,Armc8,Dhrs1,Trappc2l,Arl2,Arhgef12,Slc9a6,Tmem175,Os9,Atg7,Atp6v0b,Asb1,Nr2c1,Ptprm,Csrp2,1500011K16Rik,Mdfic,Shroom3
**mmu-miR-652**	27(776)	0,00194924	Zfand3,Dtx2,Usp2,Rpl17,Nt5m,Npc2,Nfs1,Ank2,Mkrn1,Rora,Cspg4,Myeov2,Ascc1,Deb1,Garnl3,Psen2,Ing4,Dopey2,Slc41a3,Hr,Mxra7,Acaa1a,Nhlrc1,Stx7,Tpp1,Cpeb1,Tbc1d9b
**mmu-miR-466e-5p**	33(776)	0,00204843	Adra1b,Copg,Mcee,Plb1,Ecm1,Snrpn,Zdhhc4,Fbxo9,Insig2,Nt5m,Elmo2,Habp4,D16H22S680E,Sync,Akap13,Slc46a3,Calml4,Lgals9,Suox,Wisp2,Zbtb4,Man2b1,2700078K21Rik,Nrip3,Rdh12,Slc22a4,Ly6c1,Plcb1,Mtm1,Npdc1,Il17d,Ssbp2,Tpp1
**mmu-miR-706**	31(776)	0,00207175	Samd8,Mpv17,Fbxo9,Elmo2,Krt80,Slc16a9,Akap13,Gng5,Skap2,Camk1,Calml4,Stx8,5830405N20Rik,Trim36,Suox,Galns,Dusp8,Glt8d1,Adar,Nkiras1,Garnl3,Ing4,Reep3,Gpc4,1300010F03Rik,Ahnak,6530401N04Rik,Vamp3,Mxra7,Fzd6,Agl
**mmu-miR-190b**	24(776)	0,00208139	Mcee,Kif27,Lactb2,Cyp27a1,Gadd45b,Vps8,Rarres2,Gabarapl1,BC027231,Lama2,Nfe2l1,Grik2,Dopey2,Mtcp1,Tollip,Mtm1,Glo1,Cyb5,Ugt2b36,Synpo2,Cdh7,Cox6a2,Gas6,Hint3
**mmu-miR-484**	27(776)	0,00209252	Thra,Commd8,Pef1,Adc,Emilin2,Upk3b,Creb3,Pld1,Dgkg,Gramd3,Mapk4,Chrd,Ascc1,Slc35c2,Naga,Srp14,Sirt2,Spata21,Asb1,Tex264,Ptprm,Scarb2,Slamf9,1500011K16Rik,Rabac1,Slc2a8,Aph1a
**mmu-miR-878-3p**	29(776)	0,002098	Copg,Ubl4,Zhx1,Glrb,Sh3bgr,Adamts5,Upk3b,Ank2,Adcy9,Pigz,Ephb3,Tcn2,Grina,Ndufa10,Serpinb9c,Grik2,Add3,Pik3ip1,Zdhhc8,Bbs5,Tollip,Hgsnat,Srp14,Dnalc1,Ptprm,Cdh7,Gdap1,Cdkn2b,Wscd2
**mmu-miR-382***	22(776)	0,00228555	Zfand3,Acp2,Sgca,Ecm1,Cox4nb,Ppapdc3,Nfs1,Creb3,Skap2,Ppt2,Idua,Mfap5,Pdia5,Tanc2,Cdh7,1110034G24Rik,1810046J19Rik,Kif3a,Nhlrc1,1700021C14Rik,Zfp704,Mtus1
**mmu-miR-654-5p**	28(776)	0,00238268	Ap3s1,Sqrdl,Fbxo9,Papss2,Zhx1,Nt5m,Adamts5,Upk3b,Serpinb6c,9430038I01Rik,Slc44a1,Myom2,Lrrc57,Psen2,Bbs5,Pcyt1a,Triap1,Cryab,Sorbs3,Il17d,Fzd8,Slc2a6,Vamp3,Ptprm,1110034G24Rik,Slamf9,Slc2a8,Tubb2a
**mmu-miR-136**	24(776)	0,00241054	Gtf2h5,Ecm1,Pef1,D16H22S680E,Trpv4,Nudt13,Caml,Klf15,Creb3,Cd248,Slc35b1,Tmem175,Adar,Itpr2,Serinc3,Dynlt3,Vnn1,Gpc4,Nr2c1,1110034G24Rik,Htatip2,9930013L23Rik,Igfbp2,Slc2a8
**mmu-miR-154***	22(776)	0,00243845	Crot,Cyp27a1,Vps8,Qpct,Herpud2,Lnx1,Zbtb4,Asah1,Rell2,Slc22a4,Gdap2,Dner,Sh2d4a,Pxmp4,Slc26a11,Pcbd2,Acyp1,Kif3a,Ankrd12,Limch1,Vps29,Immp1l
**mmu-miR-220**	31(776)	0,0024942	Fech,Usp2,Ascc2,Ubl4,Fuca2,Tlr6,Insig2,Adamts5,Kif1c,Adcy9,Lyplal1,Klk1b4,Ptplad2,Sepw1,L1cam,Ascc1,Idua,Pik3ip1,Ccdc80,Galnt2,Mlycd,Def8,Abhd5,Sstr4,Tbccd1,Tex264,Tuba8,Htatip2,1810030N24Rik,Cacna1b,1700094D03Rik
**mmu-miR-30d**	28(776)	0,0025029	Ppp1r16a,Fech,Tmem41a,Magi3,Smpdl3a,Stk39,Ank2,Vps8,Fstl1,Pigz,Ankra2,5830405N20Rik,Dok4,P4ha2,Anxa1,Tmem175,Psen2,Slc22a4,Ankrd29,Frmpd1,1300010F03Rik,1810037I17Rik,Pcbd2,Fbxl20,Afap1l2,Fbxw4,Mrps14,Chst12

miRNA name	*Number of genes*	*Corrected p-value*	*miRNA targets among genes induced in Rasless cells (from Table S1)*
**mmu-miR-582-5p**	27(776)	0,0025291	Pef1,Smpdl3a,Sema3b,Nt5m,Ctbs,Cd81,Gadd45b,Rarres2,Mxra8,Dph1,Tpcn1,Pdgfc,Myeov2,Dusp8,Pik3ip1,Dopey2,Srp14,Atp6v0b,Usp53,Ssbp2,Nr2c1,Glipr1,Slc2a6,Tom1l2,Stx7,Hint3,Mtus1
**mmu-miR-200c**	30(776)	0,00253671	Ppp1r16a,Ugp2,Dtx2,Plb1,Crot,Atp6v0e,Krt80,Stam2,Fn1,Serpinb6c,Slc35a2,Clip1,Qk,Slc44a1,Nipa1,Aldh6a1,Ppt2,Dusp8,Nrip3,Ccdc80,Tmem154,Ly6c1,Tanc2,Ssfa2,Acyp1,1810046J19Rik,Slamf9,1500011K16Rik,Tubb2a,Npc1
**mmu-miR-684**	23(776)	0,00256946	Wdr47,Adra1b,Ecm1,Samd8,Crot,Cyp27a1,Ehd1,Smad6,Creb3,Dgkg,Camk1,2010003O02Rik,Guk1,Ypel3,Slc35b1,Pxmp4,1300010F03Rik,Ptdss1,Hck,Vps24,BC046404,Dpm2,Rhoj
**mmu-miR-362-3p**	29(776)	0,00276426	Maob,Adra1b,Stmn3,Rab38,Clstn1,Cyp4v3,F630110N24Rik,Tmem87b,BC027231,Arnt,Emp2,Slurp1,Ttc15,Ap3m2,Rora,P4ha2,Mlycd,Os9,Trak1,Sned1,4930523C07Rik,Slc26a11,Def8,Ahnak,Acaa1a,Igfbp2,Tmub2,1700013F07Rik,Tmem9b
**mmu-miR-290-3p**	29(776)	0,00276426	Ugp2,Sgca,Plb1,Arvcf,Coq10a,Fahd2a,Fbxo9,Papss2,Hexa,Hist2h2bb,Clstn1,Ank2,Stx8,Apoa1bp,Rora,Nudt7,Ehd3,Serpinb9b,Megf6,Slc22a4,Ing4,Ntn4,Vnn1,Ahnak,Ikbkg,Ufc1,Itfg3,Csrp2,Yif1a
**mmu-miR-295**	25(776)	0,00276756	Lactb2,Coq10a,Loxl1,Stx8,Ttc15,Txnrd3,2010003O02Rik,Dlg2,Serpinb9b,Glt8d1,Triap1,Mtm1,1810037I17Rik,Def8,Clip4,Rab7l1,Nr2c1,Csrp2,Sh3tc2,Cdkn1a,Acaa1a,Wdr7,Uchl1,1700094D03Rik,Ankrd44
**mmu-miR-193b**	27(776)	0,00277079	Ugp2,Tssc4,Cyp27a1,5930434B04Rik,Ppapdc3,Acox1,Tgfb2,Slc7a7,Vps8,Lmbr1,1700009P17Rik,Ttc15,Dph1,Wisp2,Snta1,Lrrc57,Pik3ip1,Tmem175,Nkiras2,BC089491,Dbp,Srp14,Ypel5,Slc25a39,Tbc1d14,1700094D03Rik,Mtus1
**mmu-miR-350**	27(776)	0,00286154	Fech,Dtx2,Adra1b,Kif27,Cox4nb,Smpdl3a,Lyplal1,Armc8,Dhrs1,Gpr133,Cacna1g,Appl2,Tcn2,Dlg2,Nudt7,Nrip3,Ttll7,Tmem154,Zfp560,S100a16,Dner,Pcyt1a,Atp6v0b,Ahnak,Cdh7,Cox6a2,Aldh3a2
**mmu-miR-151-3p**	24(776)	0,00297249	Ppp1r16a,Ugp2,Tssc4,Col14a1,Gyg,Rab4b,Ppp2r5b,Sgcb,1700009P17Rik,Anxa3,Cacna1g,Rell2,Dopey2,Atp6v0b,Mrpl55,Mtss1,Gas6,Tmem38a,Tmem50b,Rabac1,Pcsk6,Tubb2a,Hint3,Zfp704
**mmu-miR-421**	25(776)	0,00297373	Pank2,Tmem41a,Lrrc51,Hdlbp,Akap13,Gramd3,Slc46a3,Slc31a2,Ttc15,Suox,Trappc2l,Gstk1,Stard6,Slc9a6,Lrrc57,Ly6c1,Dopey2,Atp6v0b,1700016M24Rik,Rab7l1,Cxcr7,Cyb5r3,Pcsk6,Tubb2a,Hint3
**mmu-miR-425***	22(776)	0,00298587	Pank2,Tssc4,Abcb6,Nudt13,Crybb3,Akap13,Clip1,Sepw1,Cacna1g,2900010M23Rik,Shank3,Impdh1,Anxa1,Adar,Garnl3,Ahnak,Chmp4c,9930013L23Rik,Slamf9,Atp10d,Aldh3a2,Shroom3
**mmu-miR-672**	25(776)	0,00301263	Thra,Copg,Gpx4,Insig2,Smad6,Caml,Klf15,Cd248,Copz2,Renbp,Vps8,Cry2,Qpct,Mocs2,Lrrc57,Htra3,Itpr2,Ing4,Naga,Vnn1,Mustn1,Npdc1,Tex264,Atp5e,Igbp1
**mmu-miR-501-3p**	28(776)	0,00330821	Thra,Adra1b,Slc37a4,Snrpn,Selm,R74862,Smad6,Dusp3,Tbc1d22a,1810020D17Rik,Sepw1,Cacna1g,Grina,Ypel3,Shank3,L1cam,Sec22c,Deb1,Pon3,Psen2,Nkiras2,Ing4,Plcb1,Galc,Asb1,Ssbp2,Tex264,Limch1
**mmu-miR-719**	23(776)	0,00335072	Sh3bgr,Gadd45b,Nudt13,Cd248,Trappc6a,Amhr2,Dab2ip,Tbcd,Adcy9,1700009P17Rik,Pigz,Clip1,Cacna1g,Myom2,Ndel1,Arhgef12,Ing4,Nenf,Mustn1,Zfand2b,Rabac1,Pcsk6,2810428I15Rik
**mmu-miR-376b**	25(776)	0,00339053	Pank2,Snrpn,Cox4nb,Tmem41a,BC024814,Smpdl3a,Nfs1,Akap13,Qpct,Stard6,Bphl,Ppt2,Cryab,Rab7l1,Acyp2,6530401N04Rik,Slc25a39,Ptdss1,Cdh7,Cog6,1810046J19Rik,Tom1l2,Wdr7,Atp10d,Mtus1
**mmu-miR-338-5p**	26(776)	0,00340901	2310028O11Rik,Maob,Mcee,Mpv17,Rab3d,Rarres2,Dgkg,Myom2,Tob1,Qpct,Lnx1,Rora,Mfap5,Tmem175,Bbs5,4930523C07Rik,Abtb2,0610037L13Rik,Srp14,Galc,Klf2,Acyp2,Il17d,6530401N04Rik,Zdhhc24,Tubb2a
**mmu-miR-741**	22(776)	0,00345527	Alg14,Rab38,Hexa,Rras,Nfs1,Hexb,Hoxa1,Ndel1,Zbtb4,Myeov2,Ccdc80,Garnl3,Sstr4,1700016M24Rik,Klf2,Tmbim1,Atp5s,Fbxl20,Cpa6,Scarb2,Zfp704,Shroom3
**mmu-miR-142-5p**	24(776)	0,00345968	Smpdl3a,Prss36,D6Wsu163e,Creb3,Camk1,Cacna1g,Herpud2,Aldh6a1,Psen2,Mtm1,Def8,Pdia5,Il17d,Dnalc1,Nr2c1,Cpa6,Mras,Nudt18,Dhps,Cox6a2,Scarb2,Igbp1,1500011K16Rik,Ltbp3
**mmu-miR-370**	26(776)	0,00352068	Mrc2,Map3k8,Plb1,Ascc2,Adc,Tmem41a,Clstn1,Trappc6a,Iqsec2,Rarres2,Epb4.1l4b,Pbxip1,Ascc1,Rell2,Adamtsl5,Mlycd,Slc22a4,S100a13,Akap4,BC089491,Def8,Gabarap,Il17d,Ptprm,Tom1l2,Cyb5r3
**mmu-miR-690**	24(776)	0,00359127	Zfand3,Iah1,Snap91,Cox4nb,Hexa,Smad6,Lamb2,Atp6v1e1,Copz2,Adcy9,Mkrn1,Hsd3b3,Naga,Dbp,Isca2,A930005H10Rik,Nr2c1,1810027O10Rik,Fbxl20,Htatip2,Gpr39,Igbp1,Tbc1d14,Hint3
**mmu-miR-210**	26(776)	0,00364517	Usp2,Mcee,Kif27,Ascc2,Trpc7,Selm,Higd1a,Adamts5,Vps8,Armc8,Skap2,Gpr133,5830405N20Rik,2900010M23Rik,Itgb1bp1,Lnx1,Rora,Alas1,Man2b1,Grik2,Iscu,Tmem175,Gpr39,Scn1b,Hhip,1700094D03Rik
**mmu-miR-710**	25(776)	0,00376646	Plb1,Magi3,Fahd2a,Tlr6,Rab40b,Vps39,Rarres2,Ero1l,5830405N20Rik,Ephb3,Dph1,Apoa1bp,Zbtb4,Nfe2l1,Hectd3,Os9,S100a16,Ctsb,Mtm1,Atp6v0b,Slc39a14,Vamp3,Fbn2,Slc25a35,Fads3
**mmu-miR-300**	24(776)	0,00378623	Ugp2,Slc37a4,Dscr3,Gadd45b,Caml,Creb3,Vps39,Adcy9,Ndufb4,Ankra2,Txnrd3,Hectd3,Ing4,Ntn4,Mtcp1,Npdc1,Tanc2,Slc25a39,BC046404,Tyrp1,Igbp1,Rhoj,Mdfic,Uchl1
**mmu-miR-466b-5p**	32(776)	0,00379443	Adra1b,Usp2,Mcee,Plb1,Ecm1,Snrpn,Zdhhc4,Fbxo9,Insig2,Nt5m,Elmo2,Habp4,Sync,Akap13,Slc46a3,Bmp4,Calml4,Lgals9,Suox,Lnx1,Wisp2,Zbtb4,Man2b1,2700078K21Rik,Nrip3,Rdh12,Slc22a4,Ly6c1,Plcb1,Npdc1,Il17d,Ssbp2
**mmu-miR-712***	22(776)	0,00387485	Slc6a7,Pmp22,Lgr4,Smpdl3a,Lrrc51,F630110N24Rik,Akap13,Cry2,Clip1,Arnt,Qk,Guk1,St3gal5,C330006K01Rik,Pltp,2010111I01Rik,Ahnak,Mrpl55,Klf2,Fbxl20,Gpr39,Fbxw4

miRNA name	*Number of genes*	*Corrected p-value*	*miRNA targets among genes induced in Rasless cells (from Table S1)*
**mmu-miR-138***	21(776)	0,00392176	Ppp1r16a,Stmn3,Pef1,Gyg,Inpp5a,Timp2,Rab3gap2,Stac,Arl2,Stard6,Alas1,1300010F03Rik,Vamp3,Ufc1,2900026A02Rik,Igbp1,Cyb5r3,Slc2a8,Pcsk6,Hint3,Atp10d
**mmu-miR-872***	19(776)	0,00393469	Dym,Bri3,Adc,Trpc7,Gabarapl2,Napb,Dgkg,Tmem87b,Anxa3,Lama2,Appl2,Tob1,Tom1,Tmem154,4930523C07Rik,Srp14,Ahnak,Tom1l2,Cpeb1
**mmu-miR-509-3p**	27(776)	0,0040183	Ahnak2,Acp2,Bri3,Kremen1,Insig2,Fn1,Lama2,Cacna1g,2900010M23Rik,Pdgfc,Herpud2,Hsd3b3,Stard6,Aldh6a1,Serpinb9b,Garnl3,Rdh12,Ly6c1,Bbs5,Atg7,Mtcp1,Pltp,Mmab,Cyb5,Ptprm,Tmem38a,3110002H16Rik
**mmu-miR-483**	26(776)	0,00401937	Pank2,Snap91,Smpdl3a,R74862,AI593442,Creb3,Vps39,Iqsec2,Slc35a2,Dhrs1,Camk1,Ap3m2,Myom2,Ypel3,Arhgef12,Zbtb4,Wdr54,Psen2,Plcb1,BC029214,Gstp1,Slc25a39,Mtss1,Pcsk6,Ltbp3,Cox6b1
**mmu-miR-431***	22(776)	0,00408897	Ppp1r16a,Ahnak2,Sqrdl,Sgca,Plb1,Hspa12a,Nudt13,Neu1,Atp6v1e1,Vps39,F630110N24Rik,Akap13,Arl2,Myeov2,Pbxip1,Deb1,1110003E01Rik,Ly6c1,Slc26a11,Cyb5,Dhps,Cacna1b
**mmu-miR-338-3p**	28(776)	0,00426268	Iah1,Tmem41a,Insig2,Hexa,Nudt13,Cd248,Slc35a2,1700009P17Rik,Cacna1g,Dok4,Qpct,P4ha2,Lrrc57,Mfap5,Iscu,Itpr2,Bbs5,Isca2,Tmbim1,Ssfa2,4930579G22Rik,Htatip2,Dhps,Acaa1a,1700021C14Rik,2610019F03Rik,Yif1a,Mtus1
**mmu-miR-466a-3p**	33(776)	0,00456545	Adra1b,Plb1,Kif27,Mpv17,D16H22S680E,Stam2,Smad6,Sync,Klf15,Creb3,Acox1,Kif1c,Qk,Cacna1g,Npy6r,Lnx1,Asah1,Mmaa,Pon3,Zmat2,Isca2,Ctsl,Pde4dip,Slc41a3,Ssbp2,Chmp4c,Cdh7,Cog6,Nhlrc1,Stx7,Igbp1,Mrps14,Mtus1
**mmu-miR-495**	25(776)	0,00484481	Dtx2,Adra1b,Prss29,Higd1a,Slc7a7,Fstl1,Lyplal1,Maoa,Arnt,Ankra2,Slc44a1,Ttc15,Stard6,4933439F18Rik,Vnn1,Dner,Gpc4,Tanc2,Slc25a39,Itfg3,Slamf9,1700013F07Rik,Hint3,Tmem9b,Aldh3a2
**mmu-miR-17***	22(776)	0,00503388	Slc37a4,Adc,Nt5m,Krt80,Caml,Armc8,Arhgef12,Zbtb4,Cspg4,Impdh1,Dusp8,Ehd3,Lrrc57,Iscu,Bbs5,Tollip,Ctsb,Pcbd2,Hck,Dpm2,1500011K16Rik,Pcsk6
**mmu-miR-31***	20(776)	0,00523736	Iah1,Commd8,Lgr4,Mpv17,Nt5m,Glrb,Dusp3,BC027231,Ttc15,Slc9a6,P4ha2,Vdac1,Ntn4,Mustn1,Uqcrq,Sema3f,BC046404,Mras,Limch1,Oplah
**mmu-miR-598**	25(776)	0,00540245	Sgca,Slc37a4,Cat,Crot,Sh3bgr,Ehd1,Cyp4v3,Vps39,Tgfb2,Gpr133,Fbxl2,Rora,Deb1,Dner,Sh2d4a,Lrba,Pde4dip,Zfand2b,Cyb5,6530401N04Rik,Fbn2,Csrp2,Wscd2,Zfp704,2810428I15Rik
**mmu-miR-92a**	28(776)	0,0054896	Ugp2,Sgca,Lgr4,Nt5m,Adamts5,Smad6,Serpinb6c,Slc35a2,Dgkg,Gramd3,Anxa3,Herpud2,Grina,Riok3,Asah1,Tmem154,Mustn1,Mtm1,Sirt2,Klf2,Tanc2,Asb1,Fbn2,Dnajc4,Igbp1,1700021C14Rik,Dusp22,1700094D03Rik
**mmu-miR-491**	25(776)	0,00549325	Ppp1r16a,Sh3bgr,Rras,4833439L19Rik,Nudt13,Mtmr3,Clip1,Sepw1,Man2b1,Ppt2,Ergic3,Sh2d4a,Npdc1,Cryab,Def8,Sorbs3,Glo1,Klf2,Cyb5,Tex264,Itfg3,Dnajc4,Igfbp2,Chst12,Hint3
**mmu-miR-692**	20(776)	0,00555044	Adc,Insig2,Smad6,Crybb3,Amhr2,Slc44a1,Trappc2l,Man2b1,Snta1,St3gal5,Tmem154,Slc22a4,Nupr1,Ing4,Ufc1,1810046J19Rik,Igfbp2,Gas6,1700094D03Rik,2810428I15Rik
**mmu-miR-703**	24(776)	0,00567033	Dtx2,Mal,Lactb2,Gabarapl2,Adamts5,Gng5,Tob1,Man2b1,2700078K21Rik,Nudt7,Ppm1h,Garnl3,Exoc3,Atg4a,Gpc4,Srp14,Cdh7,4930579G22Rik,Mtss1,Tom1l2,Ankrd12,Satb1,Dusp22,Zfp704
**mmu-miR-468**	23(776)	0,0056752	Thra,Kremen1,Smpdl3a,Mtmr3,Amhr2,Armc8,Pigz,Skap2,Trappc2l,Ndel1,Nudt7,Grik2,Iscu,Lims2,Tmem154,Psen2,Hbxip,Zfp560,Vnn1,Srp14,Htatip2,Camkk1,Mtus1
**mmu-miR-720**	23(776)	0,00574731	Ascc2,Adc,Ehd1,Nudt13,Ppp2r5b,D6Wsu163e,1810020D17Rik,Stac,Guk1,Arl2,Nfe2l1,Cspg4,Man2b1,Grik2,Adamtsl5,Ing4,BC089491,Nenf,Kif13b,Scarb2,Igbp1,Pcsk6,Zfp704
**mmu-miR-377**	23(776)	0,00574731	Gtf2h5,Thra,Gyg,Smpdl3a,Cobl,Cd81,Abcb6,Armc8,Hoxa1,Zbtb4,Nudt7,Mocs2,Ttll7,Ntn4,Dner,Sh2d4a,Dbp,Tanc2,Rab7l1,Uqcrq,Tyrp1,Wscd2,Mtus1
**mmu-miR-367**	27(776)	0,00590301	Ugp2,Gyg,Higd1a,Stk10,Stam2,Smad6,Nudt13,Upk3b,Serpinb6c,Slc35a2,Dgkg,Gramd3,Cry2,Clip1,Anxa3,Herpud2,Itgb1bp1,Iscu,Lrch1,Dopey2,Ctsb,Klf2,Tex264,Ptprm,Kif3a,Dnajc4,Igbp1
**mmu-miR-497**	26(776)	0,0059719	Ahnak2,Bri3,Tmem41a,Rab4b,Gpx4,Mtmr3,Gpr133,Arl2,Hsd3b3,Ypel3,Rapsn,Nudt7,St3gal5,Mlycd,Ly6c1,Sned1,0610037L13Rik,Srp14,Frmpd1,Zfand2b,Asb1,Ptprm,1110034G24Rik,Htatip2,Tmub2,Rabac1
**mmu-miR-135a**	26(776)	0,00606714	Usp2,Plb1,Snrpn,Glrb,Adamts5,Slc7a7,Tmem87b,Ttc15,Chrd,Itga9,L1cam,Mtap6,Snta1,Glt8d1,Ing4,Pltp,4930523C07Rik,Mtm1,Npdc1,Pdia5,Cyb5,Mras,Acaa1a,Slamf9,Lrsam1,Yif1a
**mmu-miR-16***	20(776)	0,00622136	Thra,Adra1b,Sgca,Sema3b,Dscr3,Caml,Anxa3,Ttc15,Repin1,Herpud2,Ndel1,Bbs5,Akap4,Abtb2,Chmp4c,Sh3tc2,Cox6a2,Ankrd12,Chst12,Oplah
**mmu-miR-208**	23(776)	0,00625867	Gnptg,Maob,Bri3,Stmn3,Snrpn,Smpdl3a,Insig2,Inpp5a,AI593442,Slc7a7,1810020D17Rik,Dhrs1,Ndel1,Itga9,Atg4a,Zfp560,App,1810037I17Rik,Gdap1,Nhlrc1,Igbp1,Satb1,Immp1l
**mmu-miR-382**	23(776)	0,00625867	Rab43,Plb1,Lactb2,Dhrs3,Insig2,Caml,Ppp2r5b,Ank2,Gstm2,Qpct,Gp1bb,P4ha2,Ccdc80,Anxa1,Adamtsl5,Bcmo1,Ssfa2,BC046404,Serf2,Slc25a35,Cdkn2b,Igbp1,1500011K16Rik
**mmu-miR-369-5p**	25(776)	0,00630393	Smpdl3a,D16H22S680E,AI593442,Cyp4v3,Tbcd,Adcy9,Lhfpl2,Qk,Hexb,Slc44a1,2010003O02Rik,Gstk1,Slc9a6,Lrrc57,Add3,Garnl3,Rdh12,Gabarap,6530401N04Rik,Ptprm,Vps24,Igbp1,Tubb2a,Cacna1b,Shroom3

miRNA name	*Number of genes*	*Corrected p-value*	*miRNA targets among genes induced in Rasless cells (from Table S1)*
**mmu-miR-326**	24(776)	0,00633584	Plb1,Pef1,Adc,Gpx4,Cd81,Smad6,Klf15,Iqsec2,Acox1,Copz2,Renbp,Slc7a7,Rarres2,Bmp4,Zbtb4,Nfe2l1,Mlycd,Ing4,Pcyt1a,Def8,Cyb5,Acaa1a,Lmna,Yif1a
**mmu-miR-450b-5p**	26(776)	0,00642845	Dym,Iah1,Bri3,Pmp22,Atp6v0e,Abcb6,Cyp4v3,Fbxl2,Ndel1,Lnx1,Ascc1,Asah1,Lrrc57,Htra3,Dopey2,Mtcp1,Tollip,Nol3,1300010F03Rik,BC029214,Mmab,Tuba8,Csrp2,Tom1l2,Cdkn2b,Dusp22
**mmu-miR-470***	21(776)	0,00650992	Dtx2,Copg,Tmem41a,Alg14,Hexa,Habp4,Sync,Upk3b,Caml,Atp6v1e1,Ank2,Tmem87b,Galns,St3gal5,Mfap5,Ctsb,Tanc2,Nr2c1,1110034G24Rik,1700021C14Rik,Hint3
**mmu-miR-208b**	23(776)	0,00655983	Gnptg,Stmn3,Commd8,Insig2,Inpp5a,AI593442,Slc7a7,1810020D17Rik,Tmem87b,Dhrs1,Anxa3,Ndel1,Atg4a,Zfp560,Serinc3,Reep3,Tm9sf4,1700016M24Rik,Gdap1,Nhlrc1,Igbp1,Wdr7,Immp1l
**mmu-miR-805**	23(776)	0,0066737	Gnptg,Mcee,Rab43,Pef1,Lactb2,Rab38,R74862,Fn1,Slc35a2,Lancl1,Ndufb4,Hexb,Slc44a1,Hoxa1,Lnx1,Slc9a6,Pltp,Adamts2,Acyp2,Cpa6,2900026A02Rik,Mtss1,Afap1l2
**mmu-miR-675-3p**	22(776)	0,00678613	Pank2,Rab43,Zhx1,Insig2,Sh3bgr,Atp6v0e,D16H22S680E,Kcnn1,Gng5,Ankra2,Txnrd3,2900010M23Rik,Ypel3,Pbxip1,Iscu,Adamtsl5,Sned1,Pltp,Atp6v0b,Htatip2,Cpeb1,Wdr7
**mmu-miR-136***	21(776)	0,00730721	Maob,Bri3,Slc37a4,Cyp27a1,AI593442,D6Wsu163e,Slc44a1,Rragc,Qpct,Cables1,Deb1,BC089491,Galc,Ahnak,Klf2,Cyb5,Tbccd1,Slc25a39,Ptdss1,Csrp2,Immp1l
**mmu-miR-490**	25(776)	0,00770402	Rab11fip5,D16H22S680E,Krt80,Akap13,1810020D17Rik,Dgkg,Gramd3,Alkbh6,Ttc15,Txnrd3,Fbxl2,Snta1,Ascc1,Nrip3,Ing4,Naga,Adamts2,Ptprm,Hck,Ckm,1500011K16Rik,Tmem38a,Tmem50b,Yif1a,Aldh3a2
**mmu-miR-193***	22(776)	0,00774833	Thra,Gnptg,Yif1b,Plb1,Ascc2,Dhrs3,Rras,Tmco3,Mocs2,Lims2,Rell2,Itpr2,Dopey2,Ahnak,Sorbs3,Sirt2,Cpa6,Sh3tc2,1810046J19Rik,Wdr7,Chst12,Rabac1
**mmu-miR-10a**	24(776)	0,0080649	Ppp1r16a,Pank2,Tmem41a,Higd1a,Abcb6,Neu1,Trpt1,Gng5,Slc44a1,Repin1,Epb4.1l4b,Slc35c2,Ulk2,Mlycd,Ly6c1,Nkiras2,Tollip,Pltp,Zfand2b,Pdia5,Kif13b,Hr,Dpm2,Scarb2
**mmu-miR-204**	23(776)	0,00813122	Gabarapl2,Sh3bgr,Abcb6,Lyrm1,Gramd3,Camk1,Rragc,Grina,Chrd,Snta1,Lrrc57,Serpinb9b,Ppm1h,Mtcp1,Grk4,Ahnak,Clip4,Vps24,1810027O10Rik,Slc25a35,Slc2a8,Cox6b1,Yif1a
**mmu-miR-653**	22(776)	0,00848331	Ppp1r16a,Dym,Cyp27a1,Lrrc51,Rab40b,Smad6,Ank2,Gng5,Btf3l4,Clip1,2010003O02Rik,Bphl,P4ha2,Zmat2,Pde6d,Pkia,Rab7l1,Glipr1,1110034G24Rik,Reck,1700013F07Rik,1700094D03Rik
**mmu-miR-879***	18(776)	0,00852567	Commd8,Adc,Gpx4,Elmo2,Serpinb6c,Copz2,Ube2h,Cspg4,Ccdc80,Anxa1,Srp14,Mrpl55,1110034G24Rik,Mras,Mdfic,Zfp704,Dmpk,Yif1a
**mmu-miR-369-3p**	26(776)	0,00857471	Gtf2h5,Rnf13,Gnptg,Mcee,Lactb2,Higd1a,Stk39,Trpv4,Slc44a1,Tpcn1,Ccdc64,Qpct,Herpud2,St3gal5,Tmem175,Ly6c1,Hscb,Mtcp1,Srp14,Lrba,Acyp2,Acyp1,Stx7,Afap1l2,Gas6,Tubb2a
**mmu-miR-294***	19(776)	0,00865608	Kctd9,Kif27,Camk4,Fbxo9,Insig2,AI593442,Mtmr3,Kif1c,Slc7a7,Skap2,Cdc42ep2,Plcb1,Mtm1,Mrpl55,Ssbp2,Mxra7,Vps24,Tom1l2,Mtus1
**mmu-miR-411***	19(776)	0,00882303	Col14a1,Stmn3,Coq10a,Sh3bgr,AI593442,Skap2,Qpct,Nudt7,Asah1,Gdap2,Sned1,Dirc2,Pcbd2,Cxcr7,Ptprm,Acyp1,Mtss1,1700021C14Rik,Rabac1
**mmu-miR-24-2***	22(776)	0,00888189	Iah1,Hspa12a,Tcta,Elmo2,Nfs1,Dynlrb1,Slc7a7,Dok4,Apoa1bp,Trappc2l,Qpct,Epb4.1l4b,Nudt7,2700078E11Rik,Glt8d1,Atg4a,Ly6c1,Zfand2b,Hck,Serf2,Mtss1,9930013L23Rik
**mmu-miR-345-3p**	23(776)	0,00894785	Zfand3,Rab43,Magi3,Rbms2,Habp4,Crybb3,Btf3l4,Ptplad2,Rragc,Ndel1,Idua,Exoc3,Mustn1,Slco3a1,Spata21,Bcmo1,Ckm,Slamf9,Tbc1d9b,Pcsk6,Dusp22,1700094D03Rik,Mtus1
**mmu-miR-466a-5p**	31(776)	0,00911679	Adra1b,Copg,Mcee,Plb1,Ecm1,Snrpn,Zdhhc4,Fbxo9,Insig2,Nt5m,Elmo2,Habp4,D16H22S680E,Sync,Akap13,Slc46a3,Bmp4,Calml4,Lgals9,Suox,Zbtb4,Man2b1,2700078K21Rik,Nrip3,Slc22a4,Ly6c1,Plcb1,Npdc1,Il17d,Ssbp2,Tpp1
**mmu-miR-376c**	23(776)	0,00939076	Rnf13,Map3k8,Kif27,Mal,Smpdl3a,Rab38,Hexa,AI593442,Trappc6a,Akap13,Sepw1,Cacna1g,Suox,Slc9a6,Bphl,P4ha2,Rab7l1,Ugt2b36,Tom1l2,Atp6v0a1,Stxbp1,Zfp704,Immp1l
**mmu-miR-410**	23(776)	0,00939076	Tmem41a,Fbxo9,Dscr3,AI593442,Upk3b,Slc35a2,Fstl1,Lyplal1,Clip1,Myom2,Qpct,Pbxip1,2700078E11Rik,Ppm1h,Vnn1,Plcb1,Sh3glb1,Pcbd2,Acyp2,Slc25a39,Reck,Rit1,Mtus1
**mmu-miR-212**	28(776)	0,00941251	Ppp1r16a,Bri3,Lgr4,Arvcf,Vps39,Adcy9,Dgkg,Qk,Slc31a2,9430038I01Rik,Appl2,Lnx1,Tmem140,Cables1,Deb1,4930523C07Rik,Abtb2,Hgsnat,Atp6v0b,1700016M24Rik,Asb1,Tex264,Nr2c1,Hck,Ufc1,Dpm2,1810030N24Rik,Vps29
**mmu-miR-293***	19(776)	0,00944881	Commd8,Fem1a,Hdlbp,Vps8,Btf3l4,Armc8,Skap2,Tcn2,Zbtb4,L1cam,Glt8d1,S100a16,Gpc4,Gabarap,Mrpl55,Dhps,1810030N24Rik,Slc2a8,Shroom3
**mmu-miR-380-5p**	23(776)	0,00945278	Zfand3,Bri3,Lgr4,Sh3bgr,D16H22S680E,Prss36,Akap13,Bmp4,Myeov2,P4ha2,Idua,Ergic3,Deb1,Pltp,Ctsb,Mtm1,Pcbd2,Usp53,Cpa6,Gpr39,Pcsk6,Cacna1b,Atp10d
**mmu-miR-322***	21(776)	0,0094646	Mrc2,Iah1,Plb1,Commd8,Kif3b,Fbxo9,Vps8,Pigz,Hexb,Ccdc64,Tob1,Dlg2,Slc9a6,Anxa1,Bbs5,Mtcp1,Hgsnat,Zfand2b,Pcbd2,Acyp2,Afap1l2
**mmu-miR-340-3p**	21(776)	0,0094646	Plb1,Commd8,Tmem41a,Nt5m,Hdlbp,Nudt13,Gramd3,9430038I01Rik,Sepw1,Ascc1,Tmem175,Itpr2,Os9,5530400B01Rik,Dopey2,Srp14,Slc2a6,Chmp4c,Gas6,Limch1,Aldh3a2

miRNA name	*Number of genes*	*Corrected p-value*	*miRNA targets among genes induced in Rasless cells (from Table S1)*
**mmu-miR-19a***	19(776)	0,00955994	Ppp1r16a,Zfand3,Sqrdl,Sgca,Mcee,Smpdl3a,Fem1a,Nudt13,Tmem87b,Suox,Gdf15,Nudt7,Lrrc57,Iscu,Fabp3,Ulk2,ORF63,Cog6,Dnajc4
**mmu-miR-434-3p**	25(776)	0,00973301	Ppp1r16a,Rpl17,Snrpn,Rab38,Sh3bgr,Habp4,AI593442,Nfs1,Dusp3,1810020D17Rik,Tob1,Gp1bb,Atg4a,Mlycd,Naga,Lrba,Tm9sf4,Ahnak,Slco3a1,Gpr39,1810046J19Rik,Atp5e,Cacna1b,1700094D03Rik,Chpt1
**mmu-miR-191***	20(776)	0,00976673	Ahnak2,Ap3s1,Copg,Rpl17,Hspa12a,Prss29,Tmem41a,Cyp4v3,Crybb3,Dhrs1,Cacna1g,Iscu,9130011J15Rik,Npdc1,Atp6v0b,Ptprm,Clcn7,Chst12,Slc2a8,Atp10d
**mmu-miR-455**	26(776)	0,0097789	Bri3,Adra1b,Gabarapl2,Cyp4v3,Hdlbp,Nudt13,Armc8,BC027231,Myom2,Guk1,Wisp2,Zdhhc8,Ly6c1,Sned1,Nol3,Atp6v0b,Gstp1,Asb1,Tex264,Htatip2,Mras,Kif3a,Scarb2,C1qtnf1,Mcl1,2810428I15Rik
**mmu-miR-30e**	27(776)	0,0099672	Ppp1r16a,Snrpn,Tmem41a,Cyp4v3,Trappc6a,Ank2,Vps8,Gramd3,Pigz,Ankra2,5830405N20Rik,Stac,P4ha2,Anxa1,Tmem175,Galnt2,Frmpd1,Ctsl,Atg12,Pcbd2,Ssfa2,Fbxl20,Slc41a2,Afap1l2,Fbxw4,Wdr7,Chst12
**mmu-miR-10b**	23(776)	0,0102089	Ppp1r16a,Pank2,Tmem41a,Higd1a,Elmo2,Abcb6,Trpt1,Slc44a1,Ccdc64,Galns,Rora,Epb4.1l4b,Slc35c2,Rdh12,Adamtsl5,Mlycd,Ly6c1,Nkiras2,Tollip,Slc26a11,Zfand2b,Pdia5,Kif13b
**mmu-miR-99b***	21(776)	0,0106372	Mrc2,Adra1b,Plb1,Gpx4,Wnt9a,Slc7a7,Gramd3,Arhgef12,Gdf15,Gp1bb,Ascc1,Nudt7,Anxa1,Adar,Ulk2,Rdh12,S100a16,Clip4,Gstp1,Scarb2,Slamf9
**mmu-miR-203***	19(776)	0,010656	Magi3,Trpc7,Rbms2,Nudt13,Dusp3,Rab3gap2,Arl2,Chrd,Arhgef12,Nfe2l1,Myeov2,Ascc1,Ppm1h,Adar,Mustn1,Sstr4,Mras,Dnajc4,Uchl1
**mmu-miR-466d-3p**	32(776)	0,0109815	Adra1b,Plb1,Kif27,Mpv17,D16H22S680E,Stam2,Smad6,Sync,Klf15,Creb3,Kif1c,Lmbr1,Qk,Cacna1g,Npy6r,Lnx1,Pon3,Zmat2,Isca2,Ctsl,Pde4dip,Slc41a3,Ssbp2,Chmp4c,Cdh7,Acyp1,Cog6,Nhlrc1,Stx7,Igbp1,Mrps14,Mtus1
**mmu-miR-216a**	25(776)	0,0111283	Fech,Bri3,Adra1b,Plb1,Tcta,Ubl4,Gpx4,Lrrc51,Atp6v0e,Nudt13,Caml,Tgfb2,Ptplad2,Chrd,Man2b1,Mocs2,Fabp3,1110003E01Rik,Exoc3,Sh2d4a,Srp14,Isca2,Mrpl55,9930013L23Rik,Hint3
**mmu-miR-181a-1***	24(776)	0,0117786	Zfand3,Cat,Fbxo9,Alg14,D16H22S680E,Rassf3,Trpv4,Vps8,Skap2,Ttc15,Sepw1,Mkrn1,Lnx1,Gp1bb,Riok3,Anxa1,Adar,Exoc3,Srp14,App,Cog6,Cpeb1,Limch1,Tubb2a
**mmu-miR-101a***	19(776)	0,0121683	2310028O11Rik,3830406C13Rik,Smpdl3a,Gabarapl2,Prss36,Alkbh6,Slc44a1,Calml4,AI317395,Farp2,Bphl,9130011J15Rik,Srp14,Pcbd2,Asb1,Ptprm,Hck,Fzd6,Tmem9b
**mmu-miR-296-3p**	27(776)	0,0122202	Dtx2,Mcee,Sema3b,Trpv4,Vps8,Trpt1,Dhrs1,Gstm2,Trappc2l,Galns,Ndufa10,Rapsn,Cables1,Deb1,Dopey2,Mustn1,Tm9sf4,Slc39a14,Tex264,Mxra7,BC046404,Tpp1,Fbxw4,Dmpk,Yif1a,1700094D03Rik,2810428I15Rik
**mmu-miR-383**	25(776)	0,0123801	Bri3,Ecm1,Gyg,Alg14,Emilin2,Kcnn1,Trpv4,D6Wsu163e,Acox1,Clip1,Myom2,Deb1,Gdap2,BC089491,Plcb1,Gabarap,Slc2a6,Ptprm,BC046404,Dpm2,Cpa6,Slc25a35,Wscd2,Igbp1,Wdr7
**mmu-miR-381**	22(776)	0,0123982	Slc37a4,Kif27,Pef1,Sh3bgr,Krt80,Caml,Vps39,Ankra2,Gpr133,Txnrd3,Nipa1,Wisp2,Tom1,Snta1,Serpinb9b,Slc6a8,Ntn4,Pxmp4,Ahnak,Tom1l2,Mdfic,Zfp704
**mmu-miR-106b***	19(776)	0,0125483	Dtx2,Ascc2,Arvcf,Rab40b,Dynlrb1,Cacna1g,Herpud2,Ndufa10,Ascc1,Mocs2,Ing4,Slc26a11,Atp6v0b,Chchd6,Slc2a6,Adamtsl4,Ckm,Acaa1a,Yif1a
**mmu-miR-376a**	25(776)	0,0127041	Col14a1,Tmem41a,BC024814,Insig2,Adamts5,Akap13,1110007C09Rik,Adcy9,Tob1,Qpct,Stard6,Bphl,Serpinb9c,Zmat2,Gabarap,Rab7l1,Atp5s,Ptdss1,Nr2c1,Cdh7,Cog6,Wdr7,Uchl1,Mtus1,Shroom3
**mmu-miR-29b***	23(776)	0,012934	Ahnak2,Neu1,Copz2,Gng5,Anxa3,Slc31a2,Alkbh6,Sepw1,Nme2,Bphl,Serpinb9b,Deb1,BC089491,Nol3,Pde6d,Slc39a14,Mrpl55,Sirt2,Kif13b,Csrp2,1700021C14Rik,1700094D03Rik,Npc1
**mmu-miR-539**	20(776)	0,0131228	Fech,Adra1b,Rab43,Crot,Smpdl3a,Atp6v0e,AI593442,Caml,D6Wsu163e,Ank2,Fstl1,Gstk1,Tob1,Lnx1,Ccdc80,Adar,Fabp3,Tanc2,Cpa6,Wdr7
**mmu-miR-33***	17(776)	0,0131271	Lgr4,Alg14,Sh3bgr,Rab40b,Creb3,1700009P17Rik,Arnt,Fbxl2,Dusp8,Lrrc57,Fabp3,Mlycd,Bbs5,Akap4,Nol3,Atp6v0b,Acyp2
**mmu-miR-335-3p**	19(776)	0,0131644	Ecm1,Hspa12a,Insig2,Fem1a,Ank2,Sgcb,Alkbh6,Ndufa10,Itgb1bp1,Lnx1,Tmem154,Tmem175,Dopey2,BC089491,Clip4,Rab7l1,Acyp2,Ugt2b36,Csrp2
**mmu-miR-26b**	24(776)	0,0133617	2310028O11Rik,Sgca,Kif27,Commd8,Fbxo9,Insig2,Emilin2,Adcy9,Qk,Tob1,Nipa1,St3gal5,Mocs2,Hectd3,Ccdc80,Ugt2b36,Kif13b,Tmbim1,Ptprm,Ssfa2,Cpa6,Gpr39,Mras,Fzd6
**mmu-miR-467a***	18(776)	0,0133633	Mcee,Plb1,Kif27,Mpv17,Glrb,Stam2,Smad6,Sync,Cacna1g,Npy6r,Slc9a6,Ccdc80,Pde4dip,Slc41a3,Ugt2b36,Ssbp2,Cdh7,Stx7
**mmu-miR-181b**	25(776)	0,0133717	Kif27,Kremen1,Ppapdc3,Atp9b,Ank2,Lyplal1,Rragc,Cacna1g,Ephb3,Repin1,Tcn2,Zbtb4,Slc9a6,Pbxip1,Ascc1,Slc35c2,Psen2,Ly6c1,Hck,1110034G24Rik,Gpr39,Kif3a,Cpeb1,Oplah,Uchl1
**mmu-miR-155**	23(776)	0,0138653	Bri3,Slc37a4,Prss29,Cyp4v3,Npy6r,Rora,L1cam,Mocs2,Asah1,Add3,Pik3ip1,Mlycd,Pde6d,Pkia,Ctsl,1300010F03Rik,Def8,Kif3a,Tbc1d9b,1810030N24Rik,Tubb2a,Satb1,Chpt1
**mmu-miR-718**	21(776)	0,014187	Dtx2,Cox4nb,Hspa12a,Ascc2,Cobl,Prss36,Smad6,Gadd45b,Trpv4,Tpcn1,Ndufa10,Gdf15,Cdk5,Psen2,Fzd8,Slc25a39,Sema3f,Cox6a2,Gas6,Pcsk6,1700094D03Rik

miRNA name	Number of genes	Corrected p-value	miRNA targets among genes induced in Rasless cells (from Table S1)
**mmu-miR-448**	20(776)	0,0141887	Kctd9,Mcee,Commd8,Adc,Fbxo9,Stam2,Ppapdc3,Klf15,Trappc6a,Ank2,Tmem87b,Ttc15,Tpcn1,Qpct,Ccdc80,Ly6c1,Slco3a1,Mtss1,Hint3,1700094D03Rik
**mmu-miR-126-5p**	21(776)	0,0144112	Iah1,Adamts5,Cyp4v3,Dynlrb1,Lmbr1,Clip1,Skap2,Qk,Myom2,Qpct,Lnx1,Myeov2,Bbs5,Il17d,Synpo2,Ssfa2,Serf2,Nhlrc1,Igbp1,Tubb2a,3110002H16Rik
**mmu-miR-451**	23(776)	0,0149442	Gnptg,Ccdc126,Nt5m,Sh3bgr,Ank2,Lmbr1,Ankra2,Dok4,Appl2,Galns,Dusp8,Nrip3,Mfap5,Glt8d1,Ulk2,Npdc1,Srp14,Adamts2,Pde4dip,Gabarap,Hck,Cpa6,Mtss1
**mmu-miR-325***	18(776)	0,0160907	Zfand3,Ifi35,Iah1,Copg,Commd8,Gabarapl2,Sh3bgr,Creb3,Gramd3,Ptplad2,Anxa3,Gstm2,Ly6c1,5530400B01Rik,Dirc2,1810037I17Rik,Slc41a3,Tmem9b
**mmu-miR-181a**	24(776)	0,0161691	Dym,Kremen1,Abcb6,Stam2,Ppapdc3,Nudt13,Atp9b,Ank2,Pld1,Lyrm1,Mkrn1,Zbtb4,Slc9a6,Pbxip1,Sec22c,Psen2,Ly6c1,Tanc2,Hck,1110034G24Rik,Gpr39,Cpeb1,Oplah,Uchl1
**mmu-miR-880**	21(776)	0,0164452	Ppp1r16a,Tssc4,Plb1,Pef1,Ascc2,Fbxo9,Smad6,Cyp4v3,Trpv4,Cd248,Vps8,Gabarapl1,Impdh1,Itpr2,Gpc4,Mrpl55,Acyp2,1110034G24Rik,1810030N24Rik,Slc2a8,Aldh3a2
**mmu-miR-466b-3-3p**	29(776)	0,0167822	Adra1b,Plb1,Kif27,Mpv17,D16H22S680E,Stam2,Smad6,Sync,Creb3,Acox1,Anxa3,Cacna1g,Npy6r,Lnx1,Mmaa,Pon3,Zmat2,Ctsl,Pde4dip,Slc41a3,Ssbp2,Chmp4c,Acyp1,Cog6,Nhlrc1,Stx7,Igbp1,Mrps14,Mtus1
**mmu-miR-127***	17(776)	0,0170008	Dtx2,Sgca,R74862,Fn1,Iqsec2,5830405N20Rik,Synpo,Zbtb4,Nfe2l1,Ccdc80,Pxmp4,Slco3a1,1110034G24Rik,Gpr39,Slamf9,Slc2a8,Oplah
**mmu-miR-22***	20(776)	0,0176067	Gtf2h5,Copg,Prss29,Rab4b,Cyp27a1,Nfs1,Vps8,Btf3l4,Tmem87b,Clip1,Hoxa1,Bphl,Mmaa,Tollip,Dbp,Cyb5,Asb1,Slc25a39,Sema3f,Hint3
**mmu-miR-27a**	23(776)	0,017612	Dym,Habp4,Fn1,AI593442,Vps39,Vps8,Skap2,Appl2,Guk1,Ypel3,Asah1,Deb1,2310046K01Rik,Akap4,Npdc1,Slc26a11,Rab7l1,Asb1,Fbxl20,Cog6,Csrp2,Mras,1700094D03Rik
**mmu-miR-135b**	23(776)	0,0178575	Usp2,Plb1,Snrpn,Glrb,Adamts5,Tmem87b,Ttc15,Mtap6,Snta1,Glt8d1,Ing4,Pltp,4930523C07Rik,Mtm1,Npdc1,Pdia5,Csrp2,Mras,Acaa1a,Slamf9,Lrsam1,Yif1a,Vps29
**mmu-miR-297b-5p**	21(776)	0,0179939	Mcee,Plb1,Cat,Kif27,Snrpn,Fbxo9,Insig2,Sync,Amhr2,Akap13,Armc8,5830405N20Rik,2010003O02Rik,Lnx1,Ypel3,Plcb1,Tsc22d3,Pcbd2,Ssbp2,Chmp4c,Mras
**mmu-miR-186***	18(776)	0,018051	Ahnak2,Zfand3,Stmn3,Arvcf,Cd81,Prss36,Tgfb2,Kif1c,Copz2,Slc7a7,Rell2,Lrba,Mrpl55,1810046J19Rik,Fbxw4,Atp6v0a1,Tmem38a,Pcsk6
**mmu-miR-134**	22(776)	0,0186116	Sqrdl,Sgca,Kremen1,Smpdl3a,Insig2,Habp4,Crybb3,Armc8,Pigz,Hsd3b3,Iscu,Hbxip,Nol3,2010111I01Rik,Mmab,Slc25a39,Ptprm,Mxra7,Ckm,Slc25a35,Cacna1b,Lrpap1
**mmu-miR-582-3p**	22(776)	0,0188767	1300014I06Rik,Dym,Tmem41a,Cyp27a1,Rab40b,Atp6v1e1,Ank2,Sgcb,Clip1,2010003O02Rik,Rasl12,Ccdc64,Bphl,Slc22a4,Vnn1,Dopey2,Ctsb,Tyrp1,Dhps,Tom1l2,1700094D03Rik,Mcl1
**mmu-miR-698**	21(776)	0,0197326	Pank2,Thra,Bri3,Ascc2,Fem1a,Kif1c,Gng5,Gramd3,Ube2h,Gstm2,Arl2,Itga9,Lrrc57,Grik2,Mfap5,Zfand2b,Ahnak,Sorbs3,Sh3tc2,Scarb2,Igfbp2
**mmu-miR-467e***	17(776)	0,0199031	Mcee,Plb1,Kif27,Glrb,Stam2,Smad6,Sync,Npy6r,Slc9a6,Ccdc80,Pon3,Pde4dip,Slc41a3,Ugt2b36,Ssbp2,Cdh7,Stx7
**mmu-miR-127**	21(776)	0,0203107	9030409G11Rik,Slc16a9,Nudt13,Renbp,Adcy9,Tmem87b,Sepw1,Txnrd3,Mapk4,Ndel1,Epb4.1l4b,Gp1bb,Tollip,Plcb1,Spata21,Slc25a39,Cpa6,Sh3tc2,Adamtsl4,Tmem9b,Atp10d
**mmu-miR-146b**	23(776)	0,0211847	Mpv17,Cyp27a1,Insig2,Vps8,Sgcb,Tmem87b,Slc44a1,Gstm2,Ccdc64,Bphl,C330006K01Rik,Garnl3,Bbs5,Gdap2,Mustn1,Ctsb,Gpc4,1300010F03Rik,Pde4dip,Tanc2,Tyrp1,Chst12,Dusp22
**mmu-miR-129-5p**	22(776)	0,0212332	Mcee,Plb1,Copz2,Akap13,Vps8,Lyrm1,Clip1,Anxa3,Calml4,Farp2,Lnx1,Cspg4,2700078K21Rik,Deb1,Os9,Nupr1,Mmab,Zfand2b,Ptprm,Tuba8,Cpa6,Shroom3
**mmu-miR-185**	19(776)	0,022003	Fahd2a,Elmo2,Nfs1,Iqsec2,Ank2,Hsd3b3,Wisp2,L1cam,P4ha2,Garnl3,9130011J15Rik,Ing4,Gabarap,Cyb5,Asb1,Ikbkg,Chst12,Rabac1,Shroom3
**mmu-miR-496**	19(776)	0,022003	Pank2,Bri3,Smpdl3a,Nudt13,Gng5,Sgcb,Ttc15,Gstm2,Qpct,Mocs2,Bbs5,Serinc3,0610037L13Rik,Adamts2,Isca2,Zfand2b,1810037I17Rik,Rab7l1,Ndrg4
**mmu-miR-181c**	24(776)	0,0221219	Kremen1,D16H22S680E,Stam2,Ppapdc3,Nudt13,Ank2,Pld1,Lyplal1,Armc8,Ephb3,Mkrn1,Guk1,Slc9a6,Pbxip1,Ascc1,Nudt7,Ly6c1,Tanc2,Hck,Cdh7,1110034G24Rik,Gpr39,Reck,Aldh3a2
**mmu-miR-218-1***	19(776)	0,0226819	Bri3,Plb1,Cat,Fbxo9,R74862,Fn1,Dusp3,Abcd1,Stx8,Sepw1,Myeov2,Serpinb9b,Ly6c1,Bbs5,4930523C07Rik,Hgsnat,Acyp2,Slc25a39,Hadhb
**mmu-miR-7a***	19(776)	0,0238279	Nt5m,Slc7a7,Anxa3,Hexb,Ttc15,Zbtb4,Gdf15,Bphl,P4ha2,Srp14,Atp6v0b,Ikbkg,Acyp1,Cdkn2b,Wdr7,Chst12,Limch1,Hint3,Chpt1
**mmu-miR-802**	21(776)	0,0246305	Snap91,Sqrdl,Kif27,Insig2,Znrf2,Cyp4v3,Nudt13,Nfs1,Atp6v1e1,Skap2,Nfe2l1,Nudt7,Ccdc80,Rdh12,Srr,Ikbkg,Chmp4c,Tyrp1,Stx7,Wdr7,Shroom3
**mmu-miR-98**	22(776)	0,0248195	Ifi35,Dtx2,Tcta,Tmem41a,Alg14,Tlr6,Habp4,Cyp4v3,Trappc6a,Gng5,Skap2,Rab3gap2,Chrd,Lnx1,Slc35b1,Riok3,St3gal5,Grik2,Fabp3,Gdap2,1110034G24Rik,Acaa1a

miRNA name	Number of genes	Corrected p-value	miRNA targets among genes induced in Rasless cells (from Table S1)
**mmu-miR-875-5p**	23(776)	0,0252754	Wdr47,Col14a1,Dhrs3,Rbms2,Klf15,D6Wsu163e,Mtmr3,Ank2,Slc7a7,Alkbh6,Calml4,Ppt2,Deb1,Slc22a4,Mustn1,Gabarap,Chid1,Clip4,Ufc1,Cdh7,Ckm,Pcsk6,Aldh3a2
**mmu-let-7c-1***	26(776)	0,0254624	Zfand3,Kif27,Zdhhc4,Crot,Papss2,Selm,Glrb,Rab40b,Lmbr1,Ccny,Skap2,Cacna1g,Myom2,Ndel1,Ypel3,Gdf15,Rapsn,Mocs2,Iscu,Gpc4,App,Klf2,Dpm2,Cog6,Ltbp3,Zfp704
**mmu-miR-471**	21(776)	0,0256027	Adc,Lactb2,Rab3d,Gabarapl2,Glrb,Atp9b,Pld1,Ankra2,Tpcn1,Lnx1,Slc9a6,Serpinb9b,Mmaa,Glt8d1,Fabp3,Sema3c,Dner,Atp5s,Cpa6,Slc25a35,Igbp1
**mmu-miR-450b-3p**	22(776)	0,0261501	Thra,Iah1,Plb1,Mpv17,Ubl4,Gng5,Pigz,Sepw1,Apoa1bp,Ndel1,Farp2,Lnx1,Ascc1,Nkiras2,Srp14,Klf2,Il17d,Glipr1,Stx7,Igfbp2,1700021C14Rik,1700094D03Rik
**mmu-miR-871**	20(776)	0,0268393	Thra,Sqrdl,Commd8,Lgr4,Birc7,Caml,Gng5,Ttc15,Myom2,Ccdc64,Qpct,Slc9a6,Epb4.1l4b,Impdh1,Nrip3,Ing4,Lrba,Il17d,Ssbp2,Scarb2
**mmu-miR-882**	19(776)	0,0272449	Plb1,Kif27,Abcb6,Clstn1,Loxl1,Pigz,Txnrd3,Hsd3b3,2700078K21Rik,Adar,9130011J15Rik,Ly6c1,App,Gabarap,Il17d,Tuba8,Sparc,Igbp1,Chst12
**mmu-miR-363**	25(776)	0,0274914	Sgca,Lgr4,Kif3b,Gyg,Nt5m,Znrf2,Smad6,Serpinb6c,Dab2ip,Gramd3,Anxa3,Tob1,Herpud2,Mocs2,Iscu,Snx10,Dynlt3,4930523C07Rik,Sirt2,Klf2,Cxcr7,Dnajc4,Cdkn2b,Rabac1,Dusp22
**mmu-miR-376c***	17(776)	0,0277347	Pmp22,Alg14,AI593442,Nfs1,1110007C09Rik,Chrd,Lnx1,P4ha2,Nudt7,Glt8d1,Pon3,Clip4,Csrp2,1810030N24Rik,Wdr7,Limch1,Ltbp3
**mmu-miR-425**	23(776)	0,0291462	Adra1b,Sh3bgr,Loxl1,Dynlrb1,Lyplal1,Repin1,Ndufa10,Rora,Impdh1,Cdk5,Deb1,Bbs5,Trak1,Plcb1,Mtm1,Hgsnat,Srp14,Acyp2,Acaa1a,Hadhb,Igbp1,Camkk1,Stxbp1
**mmu-miR-195**	22(776)	0,0295098	Bri3,Tmem41a,Rab4b,Napb,Mtmr3,Fbxl2,2010003O02Rik,Arl2,Ypel3,Shank3,Rapsn,P4ha2,Nudt7,Mlycd,Srp14,Frmpd1,Zfand2b,Fbxl20,Itfg3,Ckm,Slamf9,Rabac1
**mmu-miR-875-3p**	21(776)	0,0297991	Tlr6,Gabarapl2,Atp6v0e,Loxl1,Lyrm1,Anxa3,Skap2,Nudt7,Asah1,Rdh12,Bbs5,Atg7,Dner,Sirt2,Tanc2,Chchd6,Ufc1,Dnajc4,Lmna,Limch1,Chpt1
**mmu-miR-323-3p**	24(776)	0,030101	Commd8,Adc,Klhl18,D16H22S680E,Klf15,Vps8,Slc44a1,Guk1,Itgb1bp1,Myeov2,Ascc1,Idua,Hist1h1e,Naga,Ctsb,Lrba,Ctsl,Zdhhc24,Cdh7,Htatip2,BC004004,Mtss1,Tom1l2,Reck
**mmu-miR-181d**	23(776)	0,0305872	Kif27,Kremen1,Mtap1a,Emilin2,Ppapdc3,Atp9b,Ank2,Lyplal1,Rragc,Repin1,Zbtb4,Slc9a6,Pbxip1,Slc35c2,Psen2,Ly6c1,Hck,1110034G24Rik,Gpr39,Kif3a,Cpeb1,Oplah,Uchl1
**mmu-miR-32**	24(776)	0,0308078	Ugp2,Gyg,Tcta,Nt5m,Adamts5,AI593442,Smad6,Trappc6a,Serpinb6c,Slc35a2,Dgkg,Gramd3,Anxa3,Ankra2,Tob1,Herpud2,Dynlt3,Klf2,Hck,Dpm2,Kif3a,Dnajc4,Igbp1,Dusp22
**mmu-miR-678**	22(776)	0,0323097	Pank2,Copg,Hadha,Pef1,Nt5m,Gadd45b,Atp9b,Slc35a2,Myo6,Ephb3,Ndel1,Hbxip,Mtcp1,Nol3,Sorbs3,Asb1,Slc25a39,Dpm2,Htatip2,Mras,Rabac1,Nagk
**mmu-miR-291a-5p**	24(776)	0,0328236	Map3k8,Bri3,Adra1b,Ascc2,Prss29,Tlr6,Trpv4,Cd248,Tgfb2,Mrgprf,Clip1,Ttc15,Stard6,Nfe2l1,Ehd3,Anxa1,Dynlt3,Mrpl55,Cdh7,Cpa6,Ckm,Rabac1,Zfp704,Mtus1
**mmu-miR-154**	19(776)	0,0333326	Acp2,Ugp2,Tmem41a,Trpc7,Lrrc51,Stam2,Nudt13,Mtmr3,Slc35a2,1810020D17Rik,Lmbr1,Tcn2,Hsd3b3,C1qtnf6,Mocs2,Atg7,Sned1,Tollip,Ufc1
**mmu-miR-543**	21(776)	0,0340433	Maob,D16H22S680E,Fn1,Ppapdc3,Sync,Nfs1,Bace1,Slc7a7,Tbc1d22a,Lyplal1,Repin1,Myom2,Lrrc57,Grik2,Nenf,Klf2,Cpa6,Gpr39,Sh3tc2,Slc2a8,Lmna
**mmu-miR-463**	21(776)	0,0349692	Snrpn,Lgr4,Kremen1,Rbms2,Inpp5a,Cyp4v3,1110007C09Rik,Skap2,Gstm2,Ypel3,Myeov2,Idua,Anxa1,Fabp3,Psen2,Adamtsl5,Reep3,S100a16,Csrp2,Adamtsl4,Uchl1
**mmu-let-7e**	24(776)	0,0366643	Ppp1r16a,Mcee,Rras,Elmo2,R74862,Cyp4v3,Trappc6a,Akap13,Gng5,Skap2,Trappc2l,Itgb1bp1,Ppt2,St3gal5,Fabp3,Gdap2,Tollip,Mtm1,Isca2,Tex264,Zdhhc24,1110034G24Rik,1810046J19Rik,Wscd2
**mmu-miR-503***	15(776)	0,0411302	Ugp2,Sgca,Kif27,Sema3b,Slc7a7,Sgcb,Ttc15,Qpct,Herpud2,Zbtb4,Mfap5,Bbs5,Lrba,Tyrp1,Acaa1a
**mmu-miR-669a**	21(776)	0,0411516	Ahnak2,Zfand3,Copg,Mcee,Insig2,Higd1a,Adamts5,Fstl1,Bmp4,Rapsn,Rdh12,Ly6c1,Triap1,Ahnak,Sorbs3,Acyp2,Cpa6,Mras,Gas6,Zfp704,Vps29
**mmu-miR-511**	19(776)	0,0413243	Bri3,Sgca,Gpx4,Elmo2,Sync,Myom2,Fbxl2,Ccdc64,Cspg4,Ccdc80,Ing4,Tollip,Pde6d,App,Mrpl55,1810027O10Rik,Cdh7,Cpa6,1810046J19Rik
**mmu-miR-340-5p**	19(776)	0,0429193	Thra,AI593442,Hdlbp,F630110N24Rik,Gng5,Skap2,9430038I01Rik,Myo6,Hsd3b3,Alas1,Rapsn,Ly6c1,Triap1,Slc41a3,Il17d,Ugt2b36,Ssbp2,Nr2c1,Scarb2
**mmu-miR-376b***	16(776)	0,0429636	Commd8,Pmp22,Ascc2,AI593442,Nfs1,Akap13,1110007C09Rik,Gng5,Chrd,Nudt7,Glt8d1,Pon3,Clip4,Rab7l1,Wdr7,Ltbp3
**mmu-miR-197**	20(776)	0,0429656	1300014I06Rik,Adamts5,Dynlrb1,Pld1,Trpt1,Gramd3,Gpr133,Chrd,Man2b1,St3gal5,Pik3ip1,Ulk2,Hbxip,Lrp4,Vamp3,Gpr39,Scn1b,Cpeb1,1700021C14Rik,Ankrd44
**mmu-miR-379**	21(776)	0,0442156	Ap3s1,Dtx2,Maob,Ecm1,Hadha,Hist2h2bb,Cyp4v3,Ppp2r5b,Dgkg,Tmem87b,Idua,Mtm1,Frmpd1,Gabarap,Nr2c1,Slc2a6,Cpa6,Gpr39,9930013L23Rik,Cyb5r3,Tubb2a

miRNA name	*Number of genes*	*Corrected p-value*	*miRNA targets among genes induced in Rasless cells (from Table S1)*
**mmu-miR-24-1***	19(776)	0,0452867	Hspa12a,Ctbs,Nfs1,Dynlrb1,Vps8,Skap2,Apoa1bp,Nudt7,2700078E11Rik,Glt8d1,Tmem175,Ly6c1,Npdc1,Zfand2b,Serf2,1110034G24Rik,Csrp2,Mtss1,9930013L23Rik
**mmu-miR-193**	21(776)	0,045849	Slc6a7,Mpv17,Cyp27a1,Slc7a7,Vps8,Lmbr1,1700009P17Rik,Slc31a2,Ttc15,Wisp2,Snta1,Lrrc57,Pik3ip1,Tmem175,Nkiras2,Dbp,Srp14,Daglb,Tbc1d14,Tbc1d9b,1700094D03Rik
**mmu-miR-186**	19(776)	0,0484185	Plb1,Rpl17,Hspa12a,Smad6,Cd248,Ank2,5830405N20Rik,Qpct,Chrd,Sec22c,Mfap5,C330006K01Rik,Serinc3,Ntn4,Ankrd29,1300010F03Rik,Clip4,Csrp2,Aph1a
**mmu-miR-146a**	22(776)	0,0487916	Wdr47,Mpv17,Cyp27a1,Insig2,Adcy9,Vps8,Sgcb,Tmem87b,Tmco3,Gstm2,Bphl,Mocs2,Garnl3,Bbs5,Gdap2,Ctsb,Gpc4,1300010F03Rik,Pde4dip,Tanc2,Tyrp1,Dusp22
**mmu-miR-694**	16(776)	0,0497103	Hexa,Atp6v0e,Anxa3,Ankra2,Nudt7,Mfap5,Deb1,9130011J15Rik,Bbs5,Tanc2,Chchd6,Rab7l1,Cpa6,Cox6a2,Dnajc4,Chpt1
