# Supplementary material for: Reversible, interrelated mRNA and miRNA expression patterns in the transcriptome of Rasless fibroblasts: functional and mechanistic implications
Source: BMC Genomics. 2013 Oct 25;14:731. doi: 10.1186/1471-2164-14-731 (PMC4007593; doi:10.1186/1471-2164-14-731)
Supplement: Additional file 4: Table S4 — Differentially expressed genes of Rasless cells showing reversed, opposite transcriptional pattern in both BRAF- and MEK1-rescued MEFs. List of differentially expressed genes in Rasless MEFs (93 induced and 339 repressed) that show opposite expression pattern in the transcriptional profiles of both BRAF-rescued and MEK1-rescued MEFs (generated by SAM comparison to Rasless cells at FDR = 0.01). [file 1471-2164-14-731-S4.pdf]

Table S4. Differentially expressed genes of Rasless cells showing reversed, opposite transcriptional pattern in both BRAF- or MEK1-rescued MEFs (FDR=0.01)

List of 93 induced (red color) and 339 repressed (green color) genes of the transcriptome of Rasless cells (identified by SAM comparison between Control and Rasless cells, FDR=0.01) that are concurrently and respectively listed as repressed and induced loci in the transcriptional profiles of both BRAF-rescued and MEK1-rescued MEFs (generated by SAM comparison of Rasless cells with BRAF-rescued or MEK1-rescued MEFs at same FDR=0.01. Loci are identified by Affymetrix *Probeset ID*, *Gene Name* and *Symbol*, and listed according to degree of overexpression or repression, quantified by *d-value*.

d-value is a parameter measuring the statistical distance separating the calculated expression value of each gene probe set from the null hypothesis (no-change). *q-value* is the estimated FDR at the largest *p-value* for which the probeset would be statistically significant. *R-fold* is a measure of the fold change of a probeset in the collection of microarrays provided by the SAM algorithm. Red colored data denotes overexpression, whereas green color denotes transcriptional repression in Rasless MEFs.

Probeset ID	*Symbol*	*Gene Name*	*d-value*	*q-value*	*R-fold*
1436978_at	Wnt9a	wingless-type MMTV integration site 9A	14.33	0.00	4.05
1438266_at	Adamtsl5	ADAMTS-like 5	14.25	0.00	4.88
1420336_at	Adamtsl5	ADAMTS-like 5	6.33	0.00	2.55
1449082_at	Mfap5	microfibrillar associated protein 5	12.54	0.00	15.90
1418454_at	Mfap5	microfibrillar associated protein 5	9.76	0.00	21.53
1432558_a_at	Mal	myelin and lymphocyte protein. T-cell differentiation protein	10.89	0.00	3.66
1417275_at	Mal	myelin and lymphocyte protein. T-cell differentiation protein	10.14	0.00	21.04
1450512_at	Ntn4	netrin 4	9.90	0.00	4.04
1424057_at	Gdap2	ganglioside-induced differentiation-associated-protein 2	9.48	0.00	1.50
1422347_at	Npy6r	neuropeptide Y receptor Y6	9.25	0.00	9.01
1438086_at	Npy6r	neuropeptide Y receptor Y6	8.77	0.00	17.26
1417604_at	Camk1	calcium/calmodulin-dependent protein kinase I	9.05	0.00	2.66
1417605_s_at	Camk1	calcium/calmodulin-dependent protein kinase I	7.32	0.00	2.66
1432158_a_at	Trappc2	trafficking protein particle complex 2	8.81	0.00	1.75
1417105_at	Trappc2l	trafficking protein particle complex 2-like	5.26	0.00	1.66
1433916_at	Vamp3	vesicle-associated membrane protein 3	8.74	0.00	1.44
1456245_x_at	Vamp3	vesicle-associated membrane protein 3	6.80	0.00	1.45
1433693_x_at	Vamp3	vesicle-associated membrane protein 3	5.68	0.00	1.31
1436865_at	Slc26a11	solute carrier family 26. member 11	8.66	0.00	1.44
1424762_at	C1qtnf5	C1q and tumor necrosis factor related protein 5	8.57	0.00	2.30
1422157_a_at	Itgb1bp1	integrin beta 1 binding protein 1	8.31	0.00	1.69
1434558_at	Wdr47	WD repeat domain 47	8.13	0.00	1.79
1450409_a_at	4930570C03Rik	RIKEN cDNA 4930570C03 gene	8.08	0.00	2.53
1450410_a_at	4930570C03Rik	RIKEN cDNA 4930570C03 gene	6.82	0.00	2.25
1422596_at	Nkain4	Na+/K+ transporting ATPase interacting 4	8.07	0.00	11.90
1419979_s_at	Creb3	cAMP responsive element binding protein 3	7.86	0.00	1.98
1424741_s_at	Creb3	cAMP responsive element binding protein 3	4.70	0.01	1.90
1418089_at	Stx8	syntaxin 8	7.78	0.00	1.55
1418088_a_at	Stx8	syntaxin 8	5.72	0.00	1.54
1438550_x_at	Srr	serine racemase	7.67	0.00	1.54
1438549_a_at	Srr	serine racemase	6.24	0.00	1.72
1455045_at	Srr	serine racemase	6.17	0.00	2.18

Probeset ID	*Symbol*	*Gene Name*	*d-value*	*q-value*	*R-fold*
1419664_at	Srr	serine racemase	5.88	0.00	2.30
1435446_a_at	Chpt1	choline phosphotransferase 1	7.66	0.00	1.66
1430539_at	Mxra7	matrix-remodelling associated 7	7.61	0.00	2.13
1440975_at	Mxra7	matrix-remodelling associated 7	5.82	0.00	2.48
1453855_at	Mxra7	matrix-remodelling associated 7	5.01	0.00	1.92
1443502_at	Birc7	baculoviral IAP repeat-containing 7 (livin)	7.51	0.00	1.66
1417565_at	Abhd5	abhydrolase domain containing 5	7.45	0.00	2.22
1417566_at	Abhd5	abhydrolase domain containing 5	5.66	0.00	1.93
1439794_at	NA	NA	7.40	0.00	9.45
1419668_at	Sgcb	sarcoglycan. beta (dystrophin-associated glycoprotein)	7.39	0.00	2.15
1436678_at	Sgcb	sarcoglycan. beta (dystrophin-associated glycoprotein)	6.12	0.00	2.50
1419667_at	Sgcb	sarcoglycan. beta (dystrophin-associated glycoprotein)	5.73	0.00	1.66
1452646_at	Trp53inp2	transformation related protein 53 inducible nuclear protein 2	7.34	0.00	1.93
1419015_at	Wisp2	WNT1 inducible signaling pathway protein 2	7.25	0.00	26.74
1419315_at	Slamf9	SLAM family member 9	7.24	0.00	1.92
1452879_at	Synpo2	synaptopodin 2	7.23	0.00	2.57
1450828_at	Synpo2	synaptopodin 2	5.33	0.00	1.52
1441206_at	Synpo2	synaptopodin 2	4.32	0.01	1.72
1439409_x_at	Tyrp1	tyrosinase-related protein 1	7.16	0.00	1.42
1417384_at	Entpd5	ectonucleoside triphosphate diphosphohydrolase 5	7.15	0.00	2.36
1433763_at	Entpd5	ectonucleoside triphosphate diphosphohydrolase 5	5.51	0.00	1.60
1417383_at	Entpd5	ectonucleoside triphosphate diphosphohydrolase 5	5.14	0.00	1.45
1417382_at	Entpd5	ectonucleoside triphosphate diphosphohydrolase 5	4.48	0.01	1.45
1420540_a_at	Rit1	Ras-like without CAAX 1	7.14	0.00	1.68
1428710_at	Rit1	Ras-like without CAAX 1	4.64	0.01	1.61
1424408_at	Lims2	LIM and senescent cell antigen like domains 2	7.07	0.00	2.12
1438011_at	Pcyt1a	phosphate cytidylyltransferase 1. choline. alpha isoform	7.00	0.00	1.65
1421957_a_at	Pcyt1a	phosphate cytidylyltransferase 1. choline. alpha isoform	4.49	0.01	1.58
1428891_at	9130213B05Rik	RIKEN cDNA 9130213B05 gene	6.96	0.00	4.97
1424214_at	9130213B05Rik	RIKEN cDNA 9130213B05 gene	6.34	0.00	4.98
1449331_a_at	Rapsn	receptor-associated protein of the synapse	6.90	0.00	1.93
1416408_at	Acox1	acyl-Coenzyme A oxidase 1. palmitoyl	6.81	0.00	1.93
1416409_at	Acox1	acyl-Coenzyme A oxidase 1. palmitoyl	4.99	0.00	1.77
1448233_at	Prnp	prion protein	6.80	0.00	2.46
1416130_at	Prnp	prion protein	5.91	0.00	3.46
1417527_at	Ap3m2	adaptor-related protein complex 3. mu 2 subunit	6.77	0.00	2.03
1448751_at	Ap3m2	adaptor-related protein complex 3. mu 2 subunit	4.69	0.01	1.89
1447845_s_at	Vnn1	vanin 1	6.76	0.00	21.50
1418486_at	Vnn1	vanin 1	6.53	0.00	23.38
1423205_at	Tm9sf4	transmembrane 9 superfamily protein member 4	6.70	0.00	1.38
1423204_at	Tm9sf4	transmembrane 9 superfamily protein member 4	4.35	0.01	1.24
1435874_at	Prkab2	protein kinase. AMP-activated. beta 2 non-catalytic subunit	6.48	0.00	1.73
1435875_at	Prkab2	protein kinase. AMP-activated. beta 2 non-catalytic subunit	6.39	0.00	1.65
1423376_a_at	Dok4	docking protein 4	6.48	0.00	1.78
1418436_at	Stx7	syntaxin 7	6.43	0.00	1.36
1426801_at	08-sep	septin 8	6.42	0.00	2.05
1455594_at	Exoc3	exocyst complex component 3	6.28	0.00	2.06
1420772_a_at	Tsc22d3	TSC22 domain family. member 3	6.11	0.00	3.80
1425557_x_at	Tsc22d3	TSC22 domain family. member 3	5.38	0.00	1.49

Probeset ID	Symbol	Gene Name	d-value	q-value	R-fold
1425281_a_at	Tsc22d3	TSC22 domain family. member 3	5.19	0.00	3.24
1451362_at	Rab7l1	RAB7. member RAS oncogene family-like 1	6.04	0.00	1.88
1421088_at	Gpc4	glypican 4	6.01	0.00	1.76
1436514_at	Gpc4	glypican 4	4.94	0.00	1.70
1449263_at	Ufm1	ubiquitin-fold modifier 1	5.87	0.00	1.82
1418899_at	Ufm1	ubiquitin-fold modifier 1	4.47	0.01	1.36
1449455_at	Hck	hemopoietic cell kinase	5.86	0.00	2.67
1424726_at	Tmem150	transmembrane protein 150	5.83	0.00	1.88
1455781_at	BC027231	cDNA sequence BC027231	5.77	0.00	2.31
1435009_at	Slc9a6	solute carrier family 9 (sodium/hydrogen exchanger). member 6	5.58	0.00	1.61
1435008_at	Slc9a6	solute carrier family 9 (sodium/hydrogen exchanger). member 6	4.77	0.01	1.43
1453573_at	Hist2h3c1	histone cluster 2. H3c1	5.53	0.00	2.13
1442051_at	Hist2h3c1	histone cluster 2. H3c1	4.41	0.01	5.78
1434537_at	Slco3a1	solute carrier organic anion transporter family. member 3a1	5.53	0.00	2.45
1449929_at	Dynlt3	dynein light chain Tctex-type 3	5.53	0.00	1.65
1420614_at	Dynlt3	dynein light chain Tctex-type 3	4.76	0.01	1.82
1448867_at	Tmem9b	TMEM9 domain family. member B	5.52	0.00	1.59
1437149_at	Slc6a6	solute carrier family 6 (neurotransmitter transporter. taurine). member 6	5.51	0.00	1.81
1449751_at	Slc6a6	solute carrier family 6 (neurotransmitter transporter. taurine). member 6	4.98	0.00	1.62
1422118_at	Sync	syncoilin	5.46	0.00	1.98
1434548_at	Serinc3	serine incorporator 3	5.39	0.00	1.94
1425068_a_at	Tex264	testis expressed gene 264	5.36	0.00	2.09
1422654_at	Sgca	sarcoglycan. alpha (dystrophin-associated glycoprotein)	5.36	0.00	1.64
1445565_at	Hist1h1e	histone cluster 1. H1e	5.32	0.00	1.73
1440330_at	Hist1h1e	histone cluster 1. H1e	4.40	0.01	1.43
1452667_at	Rab2b	RAB2B. member RAS oncogene family	5.27	0.00	1.98
1428115_a_at	Rab2b	RAB2B. member RAS oncogene family	5.10	0.00	2.01
1452668_x_at	Rab2b	RAB2B. member RAS oncogene family	5.10	0.00	1.96
1434354_at	Maob	monoamine oxidase B	5.26	0.00	3.76
1424411_at	Tmem189	transmembrane protein 189	5.25	0.00	1.74
1428067_at	Rasl12	RAS-like. family 12	5.25	0.00	3.12
1431167_at	Dgkg	diacylglycerol kinase. gamma	5.23	0.00	2.09
1428014_at	Hist1h4h	histone cluster 1. H4h	5.21	0.00	3.59
1435714_x_at	Il17d	interleukin 17D	5.18	0.00	2.22
1426711_at	Tmco3	transmembrane and coiled-coil domains 3	5.10	0.00	1.80
1436325_at	Rora	RAR-related orphan receptor alpha	5.05	0.00	2.71
1434313_at	Ccdc126	coiled-coil domain containing 126	4.94	0.00	2.21
1415911_at	Impact	imprinted and ancient	4.94	0.00	2.10
1424186_at	Ccdc80	coiled-coil domain containing 80	4.88	0.00	3.80
1416654_at	Slc31a2	solute carrier family 31. member 2	4.82	0.00	1.99
1422186_s_at	Cyb5r3	cytochrome b5 reductase 3	4.81	0.00	1.57
1424654_at	Acp2	acid phosphatase 2. lysosomal	4.79	0.00	1.36
1436788_at	Acp2	acid phosphatase 2. lysosomal	4.61	0.01	1.57
1447556_x_at	1700094D03Rik	RIKEN cDNA 1700094D03 gene	4.75	0.01	1.26
1417632_at	Atp6v0a1	ATPase. H+ transporting. lysosomal V0 subunit A1	4.75	0.01	2.02
1428312_at	Lrrc57	leucine rich repeat containing 57	4.72	0.01	1.55
1417659_at	Vps29	vacuolar protein sorting 29 (S. pombe)	4.67	0.01	1.44
1417660_s_at	Vps29	vacuolar protein sorting 29 (S. pombe)	4.44	0.01	1.37
1419548_at	Kpna1	karyopherin (importin) alpha 1	4.66	0.01	1.28

Probeset ID	*Symbol*	*Gene Name*	*d-value*	*q-value*	*R-fold*
1460260_s_at	Kpna1	karyopherin (importin) alpha 1	4.47	0.01	1.27
1434162_at	2700078E11Rik	RIKEN cDNA 2700078E11 gene	4.65	0.01	1.44
1423549_at	Slc1a4	solute carrier family 1 (glutamate/neutral amino acid transporter). member 4	4.65	0.01	2.14
1452719_at	Zdhhc24	zinc finger. DHHC domain containing 24	4.63	0.01	2.29
1421063_s_at	Snurf	SNRPN upstream reading frame	4.62	0.01	1.83
1421824_at	Bace1	beta-site APP cleaving enzyme 1	4.51	0.01	1.66
1422541_at	Ptprm	protein tyrosine phosphatase. receptor type. M	4.50	0.01	1.64
1438442_at	5730470L24Rik	RIKEN cDNA 5730470L24 gene	4.42	0.01	1.74
1422594_at	5730470L24Rik	RIKEN cDNA 5730470L24 gene	4.32	0.01	1.45
1417981_at	Insig2	insulin induced gene 2	4.42	0.01	1.54
1452955_at	Gtf2h5	general transcription factor IIH. polypeptide 5	4.41	0.01	1.66
1454929_s_at	Safb	scaffold attachment factor B	-4.03	0.01	0.72
1454928_at	Safb	scaffold attachment factor B	-4.77	0.01	0.65
1417886_at	Ints5	integrator complex subunit 5	-4.04	0.01	0.75
1454875_a_at	Rbbp4	retinoblastoma binding protein 4	-4.04	0.01	0.62
1434892_x_at	Rbbp4	retinoblastoma binding protein 4	-6.08	0.00	0.64
1423882_at	Rfwd3	ring finger and WD repeat domain 3	-4.04	0.01	0.59
1417238_at	Ewsr1	Ewing sarcoma breakpoint region 1	-4.05	0.01	0.64
1443466_s_at	Polr3b	polymerase (RNA) III (DNA directed) polypeptide B	-4.05	0.01	0.79
1452107_s_at	NA	NA	-4.05	0.01	0.17
1419152_at	2810417H13Rik	RIKEN cDNA 2810417H13 gene	-4.06	0.01	0.61
1419153_at	2810417H13Rik	RIKEN cDNA 2810417H13 gene	-6.04	0.00	0.23
1437286_x_at	1110020G09Rik	RIKEN cDNA 1110020G09 gene	-4.08	0.01	0.82
1437285_at	1110020G09Rik	RIKEN cDNA 1110020G09 gene	-4.84	0.00	0.76
1437287_at	1110020G09Rik	RIKEN cDNA 1110020G09 gene	-7.45	0.00	0.49
1453422_a_at	1110020G09Rik	RIKEN cDNA 1110020G09 gene	-8.03	0.00	0.51
1436654_at	Gen1	Gen homolog 1. endonuclease (Drosophila)	-4.11	0.01	0.59
1458373_at	Gen1	Gen homolog 1. endonuclease (Drosophila)	-5.47	0.00	0.59
1415869_a_at	Trim28	tripartite motif-containing 28	-4.11	0.01	0.61
1456308_x_at	Trim28	tripartite motif-containing 28	-5.13	0.00	0.62
1448358_s_at	Snrpg	small nuclear ribonucleoprotein polypeptide G	-4.12	0.01	0.63
1433830_at	Hnrnpa2b1	heterogeneous nuclear ribonucleoprotein A2/B1	-4.12	0.01	0.42
1434047_x_at	Hnrnpa2b1	heterogeneous nuclear ribonucleoprotein A2/B1	-4.60	0.01	0.65
1433829_a_at	Hnrnpa2b1	heterogeneous nuclear ribonucleoprotein A2/B1	-5.38	0.00	0.69
1420365_a_at	Hnrnpa2b1	heterogeneous nuclear ribonucleoprotein A2/B1	-6.20	0.00	0.47
1429871_at	Hmmr	hyaluronan mediated motility receptor (RHAMM)	-4.12	0.01	0.59
1450157_a_at	Hmmr	hyaluronan mediated motility receptor (RHAMM)	-5.69	0.00	0.28
1427541_x_at	Hmmr	hyaluronan mediated motility receptor (RHAMM)	-6.71	0.00	0.32
1450156_a_at	Hmmr	hyaluronan mediated motility receptor (RHAMM)	-6.72	0.00	0.30
1425815_a_at	Hmmr	hyaluronan mediated motility receptor (RHAMM)	-7.26	0.00	0.31
1442454_at	Top2a	topoisomerase (DNA) II alpha	-4.12	0.01	0.44
1454694_a_at	Top2a	topoisomerase (DNA) II alpha	-6.79	0.00	0.22
1435077_at	Asxl1	additional sex combs like 1 (Drosophila)	-4.13	0.01	0.61
1419741_at	Supt16h	suppressor of Ty 16 homolog (S. cerevisiae)	-4.13	0.01	0.65
1452098_at	Chtf18	CTF18. chromosome transmission fidelity factor 18 homolog (S. cerevisiae)	-4.14	0.01	0.34
1427982_s_at	Syne2	synaptic nuclear envelope 2	-4.15	0.01	0.28
1448103_s_at	Nono	non-POU-domain-containing. octamer binding protein	-4.15	0.01	0.76
1415820_x_at	Nono	non-POU-domain-containing. octamer binding protein	-4.98	0.00	0.70
1416393_at	Emg1	EMG1 nucleolar protein homolog (S. cerevisiae)	-4.16	0.01	0.67

Probeset ID	*Symbol*	*Gene Name*	*d-value*	*q-value*	*R-fold*
1437032_x_at	Rbm14	RNA binding motif protein 14	-4.16	0.01	0.67
1456566_x_at	Rbm14	RNA binding motif protein 14	-4.34	0.01	0.59
1436979_x_at	Rbm14	RNA binding motif protein 14	-5.40	0.00	0.56
1433697_at	Patl1	protein associated with topoisomerase II homolog 1 (yeast)	-4.16	0.01	0.78
1455915_at	Galnt4	UDP-N-acetyl-alpha-D-galactosamine:polypeptide N-acetylgalactosaminyltransferase 4	-4.17	0.01	0.65
1423460_at	Gigyf1	GRB10 interacting GYF protein 1	-4.17	0.01	0.79
1427685_a_at	Synj2	synaptojanin 2	-4.17	0.01	0.38
1452344_at	Synj2	synaptojanin 2	-4.85	0.00	0.39
1434541_x_at	Khdrbs1	KH domain containing. RNA binding. signal transduction associated 1	-6.55	0.00	0.65
1418628_at	Khdrbs1	KH domain containing. RNA binding. signal transduction associated 1	-7.40	0.00	0.58
1437389_x_at	Khdrbs1	KH domain containing. RNA binding. signal transduction associated 1	-4.17	0.01	0.61
1418629_a_at	Khdrbs1	KH domain containing. RNA binding. signal transduction associated 1	-4.45	0.01	0.63
1438462_x_at	Khdrbs1	KH domain containing. RNA binding. signal transduction associated 1	-4.91	0.00	0.72
1423517_at	LOC100038887	hypothetical protein LOC100038887	-4.17	0.01	0.80
1455988_a_at	LOC100038887	hypothetical protein LOC100038887	-4.20	0.01	0.77
1459740_s_at	Ucp2	uncoupling protein 2 (mitochondrial. proton carrier)	-4.19	0.01	0.72
1456005_a_at	Bcl2l11	BCL2-like 11 (apoptosis facilitator)	-4.19	0.01	0.45
1435448_at	Bcl2l11	BCL2-like 11 (apoptosis facilitator)	-4.81	0.00	0.58
1435449_at	Bcl2l11	BCL2-like 11 (apoptosis facilitator)	-4.98	0.00	0.52
1452816_at	Mlf1ip	myeloid leukemia factor 1 interacting protein	-4.20	0.01	0.72
1428518_at	Mlf1ip	myeloid leukemia factor 1 interacting protein	-6.15	0.00	0.36
1456325_at	Cep68	centrosomal protein 68	-4.20	0.01	0.66
1441340_at	Cep68	centrosomal protein 68	-5.33	0.00	0.60
1417434_at	Gpd2	glycerol phosphate dehydrogenase 2. mitochondrial	-4.20	0.01	0.65
1428827_at	Whsc1	Wolf-Hirschhorn syndrome candidate 1 (human)	-4.20	0.01	0.36
1455228_at	Whsc1	Wolf-Hirschhorn syndrome candidate 1 (human)	-5.54	0.00	0.58
1416621_at	Llgl1	lethal giant larvae homolog 1 (Drosophila)	-4.23	0.01	0.57
1448778_at	Sfrs4	splicing factor. arginine/serine-rich 4 (SRp75)	-4.24	0.01	0.52
1446760_at	Trip13	thyroid hormone receptor interactor 13	-4.24	0.01	0.79
1429294_at	Trip13	thyroid hormone receptor interactor 13	-7.05	0.00	0.29
1429295_s_at	Trip13	thyroid hormone receptor interactor 13	-7.35	0.00	0.23
1439459_x_at	Acly	ATP citrate lyase	-4.25	0.01	0.81
1449254_at	Spp1	secreted phosphoprotein 1	-4.25	0.01	0.27
1418330_at	Ctcf	CCCTC-binding factor	-4.26	0.01	0.68
1422823_at	Eps8	epidermal growth factor receptor pathway substrate 8	-4.26	0.01	0.41
1425733_a_at	Eps8	epidermal growth factor receptor pathway substrate 8	-4.33	0.01	0.52
1422824_s_at	Eps8	epidermal growth factor receptor pathway substrate 8	-4.84	0.00	0.37
1454042_a_at	Srpk1	serine/arginine-rich protein specific kinase 1	-4.27	0.01	0.67
1448774_at	Stoml2	stomatin (Epb7.2)-like 2	-4.28	0.01	0.56
1431226_a_at	Fndc4	fibronectin type III domain containing 4	-4.29	0.01	0.39
1449012_s_at	Fndc4	fibronectin type III domain containing 4	-4.43	0.01	0.47
1418372_at	Adsl	adenylosuccinate lyase	-4.29	0.01	0.63
1423050_s_at	Hnrnpu	heterogeneous nuclear ribonucleoprotein U	-4.30	0.01	0.75
1423051_at	Hnrnpu	heterogeneous nuclear ribonucleoprotein U	-6.00	0.00	0.52
1437244_at	Gas2l3	growth arrest-specific 2 like 3	-4.31	0.01	0.42
1455980_a_at	Gas2l3	growth arrest-specific 2 like 3	-4.34	0.01	0.36
1453416_at	Gas2l3	growth arrest-specific 2 like 3	-4.36	0.01	0.41
1415917_at	Mthfd1	methylenetetrahydrofolate dehydrogenase (NADP+ dependent).	-4.31	0.01	0.57

Probeset ID	*Symbol*	*Gene Name*	*d-value*	*q-value*	*R-fold*
		methenyltetrahydrofolate cyclohydrolase. formyltetrahydrofolate synthase			
1415916_a_at	Mthfd1	methylenetetrahydrofolate dehydrogenase (NADP+ dependent). methenyltetrahydrofolate cyclohydrolase. formyltetrahydrofolate synthase	-4.70	0.01	0.58
1418822_a_at	Arf6	ADP-ribosylation factor 6	-4.32	0.01	0.75
1427064_a_at	Scrib	scribbled homolog (Drosophila)	-4.34	0.01	0.61
1451356_at	Anp32e	acidic (leucine-rich) nuclear phosphoprotein 32 family. member E	-4.35	0.01	0.67
1420592_a_at	Anp32e	acidic (leucine-rich) nuclear phosphoprotein 32 family. member E	-6.61	0.00	0.46
1453769_at	Ckap2l	cytoskeleton associated protein 2-like	-4.35	0.01	0.47
1435938_at	Ckap2l	cytoskeleton associated protein 2-like	-6.43	0.00	0.45
1416415_a_at	H2afz	H2A histone family. member Z	-4.35	0.01	0.63
1455609_at	Cit	citron	-4.35	0.01	0.40
1422922_at	Recql4	RecQ protein-like 4	-4.35	0.01	0.63
1449343_s_at	Sin3a	transcriptional regulator. SIN3A (yeast)	-4.36	0.01	0.65
1419102_at	Sin3a	transcriptional regulator. SIN3A (yeast)	-4.42	0.01	0.57
1419101_at	Sin3a	transcriptional regulator. SIN3A (yeast)	-4.62	0.01	0.64
1439421_x_at	Cbx3	chromobox homolog 3 (Drosophila HP1 gamma)	-4.37	0.01	0.59
1448504_a_at	Cbx3	chromobox homolog 3 (Drosophila HP1 gamma)	-4.67	0.01	0.57
1434650_at	Pogz	pogo transposable element with ZNF domain	-4.37	0.01	0.67
1457708_at	Mbd4	methyl-CpG binding domain protein 4	-4.38	0.01	0.65
1428546_at	Syncrip	synaptotagmin binding. cytoplasmic RNA interacting protein	-4.38	0.01	0.58
1426402_at	Syncrip	synaptotagmin binding. cytoplasmic RNA interacting protein	-5.59	0.00	0.65
1438601_at	Pkmyt1	protein kinase. membrane associated tyrosine/threonine 1	-4.39	0.01	0.86
1426783_at	Kat2a	K(lysine) acetyltransferase 2A	-4.40	0.01	0.68
1421237_at	Tmpo	thymopoietin	-4.41	0.01	0.50
1426349_s_at	Tmpo	thymopoietin	-5.91	0.00	0.32
1428976_at	Tmpo	thymopoietin	-6.43	0.00	0.44
1452036_a_at	Tmpo	thymopoietin	-7.22	0.00	0.29
1424895_at	Gpsm2	G-protein signalling modulator 2 (AGS3-like. C. elegans)	-4.41	0.01	0.52
1449293_a_at	Skp2	S-phase kinase-associated protein 2 (p45)	-4.42	0.01	0.44
1436000_a_at	Skp2	S-phase kinase-associated protein 2 (p45)	-5.13	0.00	0.36
1460247_a_at	Skp2	S-phase kinase-associated protein 2 (p45)	-5.19	0.00	0.38
1437033_a_at	Skp2	S-phase kinase-associated protein 2 (p45)	-5.92	0.00	0.37
1418969_at	Skp2	S-phase kinase-associated protein 2 (p45)	-6.19	0.00	0.39
1448016_at	Sass6	spindle assembly 6 homolog (C. elegans)	-4.44	0.01	0.54
1453490_at	Sass6	spindle assembly 6 homolog (C. elegans)	-7.18	0.00	0.60
1455760_at	Slc9a5	solute carrier family 9 (sodium/hydrogen exchanger). member 5	-4.45	0.01	0.53
1423303_at	Paxip1	PAX interacting (with transcription-activation domain) protein 1	-4.46	0.01	0.58
1438951_x_at	Nup54	nucleoporin 54	-4.47	0.01	0.73
1433580_at	Nup54	nucleoporin 54	-7.81	0.00	0.65
1429002_at	Snw1	SNW domain containing 1	-4.47	0.01	0.79
1428224_at	Hnrpdl	heterogeneous nuclear ribonucleoprotein D-like	-4.48	0.01	0.57
1456698_s_at	Hnrpdl	heterogeneous nuclear ribonucleoprotein D-like	-4.99	0.00	0.58
1420093_s_at	Hnrpdl	heterogeneous nuclear ribonucleoprotein D-like	-5.57	0.00	0.62
1424251_a_at	Hnrpdl	heterogeneous nuclear ribonucleoprotein D-like	-7.68	0.00	0.58
1449039_a_at	Hnrpdl	heterogeneous nuclear ribonucleoprotein D-like	-8.60	0.00	0.51
1427441_a_at	Suclg2	succinate-Coenzyme A ligase. GDP-forming. beta subunit	-4.48	0.01	0.58
1436816_at	Nup133	nucleoporin 133	-4.49	0.01	0.56
1451111_at	Nup133	nucleoporin 133	-4.84	0.00	0.55
1423787_at	Nup133	nucleoporin 133	-4.94	0.00	0.57

Probeset ID	*Symbol*	*Gene Name*	*d-value*	*q-value*	*R-fold*
1449333_at	Sf3a1	splicing factor 3a. subunit 1	-4.54	0.01	0.64
1425166_at	Rbl1	retinoblastoma-like 1 (p107)	-4.55	0.01	0.43
1424156_at	Rbl1	retinoblastoma-like 1 (p107)	-5.27	0.00	0.30
1424033_at	Sfrs7	splicing factor. arginine/serine-rich 7	-4.55	0.01	0.47
1424883_s_at	Sfrs7	splicing factor. arginine/serine-rich 7	-5.39	0.00	0.45
1426653_at	Mcm3	minichromosome maintenance deficient 3 (S. cerevisiae)	-4.56	0.01	0.31
1449705_x_at	Mcm3	minichromosome maintenance deficient 3 (S. cerevisiae)	-5.82	0.00	0.33
1426652_at	Mcm3	minichromosome maintenance deficient 3 (S. cerevisiae)	-6.70	0.00	0.28
1420028_s_at	Mcm3	minichromosome maintenance deficient 3 (S. cerevisiae)	-6.99	0.00	0.27
1454952_s_at	Ncapd3	non-SMC condensin II complex. subunit D3	-4.57	0.01	0.38
1448324_at	Rnps1	ribonucleic acid binding protein S1	-4.58	0.01	0.62
1435728_at	Tyw3	tRNA-yW synthesizing protein 3 homolog (S. cerevisiae)	-4.59	0.01	0.70
1425022_at	Usp3	ubiquitin specific peptidase 3	-4.59	0.01	0.73
1425023_at	Usp3	ubiquitin specific peptidase 3	-6.24	0.00	0.67
1415829_at	Lbr	lamin B receptor	-4.60	0.01	0.50
1444235_at	1700025G04Rik	RIKEN cDNA 1700025G04 gene	-4.61	0.01	0.67
1436431_at	1700025G04Rik	RIKEN cDNA 1700025G04 gene	-5.48	0.00	0.51
1423524_at	Mastl	microtubule associated serine/threonine kinase-like	-4.62	0.01	0.35
1423525_at	Mastl	microtubule associated serine/threonine kinase-like	-5.25	0.00	0.50
1439753_x_at	Six4	sine oculis-related homeobox 4 homolog (Drosophila)	-4.65	0.01	0.47
1456862_at	Six4	sine oculis-related homeobox 4 homolog (Drosophila)	-4.90	0.00	0.44
1425767_a_at	Six4	sine oculis-related homeobox 4 homolog (Drosophila)	-5.22	0.00	0.53
1448235_s_at	Hmgb1	high mobility group box 1	-4.66	0.01	0.41
1435324_x_at	Hmgb1	high mobility group box 1	-5.14	0.00	0.49
1425048_a_at	Hmgb1	high mobility group box 1	-5.42	0.00	0.57
1416176_at	Hmgb1	high mobility group box 1	-6.07	0.00	0.56
1423123_at	Rad54l	RAD54 like (S. cerevisiae)	-4.66	0.01	0.66
1450862_at	Rad54l	RAD54 like (S. cerevisiae)	-6.05	0.00	0.27
1419620_at	Pttg1	pituitary tumor-transforming 1	-4.66	0.01	0.71
1438390_s_at	Pttg1	pituitary tumor-transforming 1	-5.18	0.00	0.42
1424105_a_at	Pttg1	pituitary tumor-transforming 1	-5.28	0.00	0.36
1420937_at	Cpsf2	cleavage and polyadenylation specific factor 2	-4.66	0.01	0.71
1420936_s_at	Cpsf2	cleavage and polyadenylation specific factor 2	-4.94	0.00	0.67
1431089_at	Cpsf2	cleavage and polyadenylation specific factor 2	-5.60	0.00	0.48
1421354_at	Prkg2	protein kinase. cGMP-dependent. type II	-4.67	0.01	0.68
1435460_at	Prkg2	protein kinase. cGMP-dependent. type II	-10.21	0.00	0.16
1435162_at	Prkg2	protein kinase. cGMP-dependent. type II	-11.69	0.00	0.08
1436747_at	1110014K08Rik	RIKEN cDNA 1110014K08 gene	-4.68	0.01	0.60
1436030_at	Cachd1	cache domain containing 1	-4.68	0.01	0.73
1434602_at	Med13l	mediator complex subunit 13-like	-4.68	0.01	0.63
1438339_at	Fancd2	Fanconi anemia. complementation group D2	-4.69	0.01	0.51
1439091_at	Fancd2	Fanconi anemia. complementation group D2	-4.82	0.00	0.36
1438833_at	Casc5	cancer susceptibility candidate 5	-4.69	0.01	0.55
1430193_at	Casc5	cancer susceptibility candidate 5	-5.92	0.00	0.42
1416126_at	Rpo1-2	RNA polymerase 1-2	-4.70	0.01	0.61
1456730_x_at	Actl6a	actin-like 6A	-4.71	0.01	0.65
1416569_at	Actl6a	actin-like 6A	-4.74	0.01	0.63
1415851_a_at	Impdh2	inosine 5'-phosphate dehydrogenase 2	-4.72	0.01	0.57
1415852_at	Impdh2	inosine 5'-phosphate dehydrogenase 2	-7.95	0.00	0.55

Probeset ID	*Symbol*	*Gene Name*	*d-value*	*q-value*	*R-fold*
1448231_at	Fkbp5	FK506 binding protein 5	-4.73	0.01	0.45
1416125_at	Fkbp5	FK506 binding protein 5	-5.43	0.00	0.32
1459765_s_at	Sf1	splicing factor 1	-4.76	0.01	0.61
1416161_at	Rad21	RAD21 homolog (S. pombe)	-4.76	0.01	0.53
1436026_at	Zfp703	zinc finger protein 703	-4.77	0.01	0.77
1423431_a_at	Mybbp1a	MYB binding protein (P160) 1a	-4.77	0.01	0.67
1421934_at	Cbx5	chromobox homolog 5 (Drosophila HP1a)	-4.77	0.01	0.63
1435575_at	Kntc1	kinetochore associated 1	-4.78	0.01	0.32
1423674_at	Usp1	ubiquitin specific peptdiase 1	-4.78	0.01	0.45
1451080_at	Usp1	ubiquitin specific peptdiase 1	-6.28	0.00	0.38
1423675_at	Usp1	ubiquitin specific peptdiase 1	-7.28	0.00	0.37
1426580_at	Plk4	polo-like kinase 4 (Drosophila)	-4.81	0.00	0.36
1452115_a_at	Plk4	polo-like kinase 4 (Drosophila)	-5.30	0.00	0.31
1419838_s_at	Plk4	polo-like kinase 4 (Drosophila)	-5.52	0.00	0.28
1456055_x_at	Pold1	polymerase (DNA directed). delta 1. catalytic subunit	-4.81	0.00	0.41
1448187_at	Pold1	polymerase (DNA directed). delta 1. catalytic subunit	-4.84	0.00	0.45
1454978_at	Ttyh3	tweety homolog 3 (Drosophila)	-4.82	0.00	0.59
1415781_a_at	Sumo2	SMT3 suppressor of mif two 3 homolog 2 (yeast)	-4.82	0.00	0.82
1415782_at	Sumo2	SMT3 suppressor of mif two 3 homolog 2 (yeast)	-6.17	0.00	0.77
1434850_at	Iqgap3	IQ motif containing GTPase activating protein 3	-4.82	0.00	0.43
1429104_at	Limd2	LIM domain containing 2	-4.82	0.00	0.49
1447766_x_at	Limd2	LIM domain containing 2	-5.02	0.00	0.69
1456377_x_at	Limd2	LIM domain containing 2	-5.56	0.00	0.42
1428310_at	Larp7	La ribonucleoprotein domain family. member 7	-4.82	0.00	0.54
1429558_a_at	Larp7	La ribonucleoprotein domain family. member 7	-6.18	0.00	0.56
1427886_at	Pom121	nuclear pore membrane protein 121	-4.83	0.00	0.64
1436036_at	Whsc2	Wolf-Hirschhorn syndrome candidate 2 (human)	-4.83	0.00	0.52
1428532_at	Ints7	integrator complex subunit 7	-4.83	0.00	0.54
1433552_a_at	Polr2b	polymerase (RNA) II (DNA directed) polypeptide B	-4.85	0.00	0.68
1433669_at	Akap8	A kinase (PRKA) anchor protein 8	-4.85	0.00	0.65
1450983_at	Akap8	A kinase (PRKA) anchor protein 8	-5.47	0.00	0.48
1417940_s_at	Rad51ap1	RAD51 associated protein 1	-4.86	0.00	0.56
1417938_at	Rad51ap1	RAD51 associated protein 1	-5.42	0.00	0.32
1448899_s_at	Rad51ap1	RAD51 associated protein 1	-5.91	0.00	0.30
1417939_at	Rad51ap1	RAD51 associated protein 1	-6.99	0.00	0.37
1453053_at	2610036L11Rik	RIKEN cDNA 2610036L11 gene	-4.87	0.00	0.63
1429156_at	2610036L11Rik	RIKEN cDNA 2610036L11 gene	-4.92	0.00	0.48
1460429_at	Cdc5l	cell division cycle 5-like (S. pombe)	-4.88	0.00	0.62
1418640_at	Sirt1	sirtuin 1 (silent mating type information regulation 2. homolog) 1 (S. cerevisiae)	-4.88	0.00	0.64
1436872_at	Tacc3	transforming. acidic coiled-coil containing protein 3	-4.90	0.00	0.59
1417450_a_at	Tacc3	transforming. acidic coiled-coil containing protein 3	-6.21	0.00	0.29
1455834_x_at	Tacc3	transforming. acidic coiled-coil containing protein 3	-6.32	0.00	0.46
1439510_at	Sgol1	shugoshin-like 1 (S. pombe)	-4.91	0.00	0.29
1418919_at	Sgol1	shugoshin-like 1 (S. pombe)	-6.02	0.00	0.27
1450920_at	Ccnb2	cyclin B2	-4.92	0.00	0.34
1448165_at	Casp2	caspase 2	-4.93	0.00	0.58
1415810_at	Uhrf1	ubiquitin-like. containing PHD and RING finger domains. 1	-4.94	0.00	0.21
1415811_at	Uhrf1	ubiquitin-like. containing PHD and RING finger domains. 1	-5.92	0.00	0.23
1436118_at	Vangl2	vang-like 2 (van gogh. Drosophila)	-4.94	0.00	0.52

Probeset ID	***Symbol***	***Gene Name***	***d-value***	***q-value***	***R-fold***
1455592_at	Vangl2	vang-like 2 (van gogh. Drosophila)	-5.18	0.00	0.50
1455220_at	Frat2	frequently rearranged in advanced T-cell lymphomas 2	-4.94	0.00	0.62
1447623_s_at	Prkd1	protein kinase D1	-4.94	0.00	0.49
1422673_at	Prkd1	protein kinase D1	-7.38	0.00	0.52
1450874_at	Matr3	matrin 3	-4.96	0.00	0.75
1436796_at	Matr3	matrin 3	-5.18	0.00	0.53
1434888_a_at	Matr3	matrin 3	-6.33	0.00	0.65
1438368_a_at	Matr3	matrin 3	-8.15	0.00	0.61
1456652_at	Dtl	denticleless homolog (Drosophila)	-4.98	0.00	0.46
1434695_at	Dtl	denticleless homolog (Drosophila)	-5.53	0.00	0.25
1432179_x_at	2810433K01Rik	RIKEN cDNA 2810433K01 gene	-5.00	0.00	0.57
1450496_a_at	2810433K01Rik	RIKEN cDNA 2810433K01 gene	-6.43	0.00	0.24
1452315_at	Kif11	kinesin family member 11	-5.01	0.00	0.32
1452314_at	Kif11	kinesin family member 11	-7.03	0.00	0.23
1435306_a_at	Kif11	kinesin family member 11	-9.48	0.00	0.24
1460221_at	Ptges3	prostaglandin E synthase 3 (cytosolic)	-5.01	0.00	0.74
1417998_at	Ptges3	prostaglandin E synthase 3 (cytosolic)	-6.46	0.00	0.72
1436018_at	Mex3a	mex3 homolog A (C. elegans)	-5.02	0.00	0.65
1455529_at	Mex3a	mex3 homolog A (C. elegans)	-6.32	0.00	0.49
1450929_at	Zfp57	zinc finger protein 57	-5.03	0.00	0.57
1454801_at	Ankrd28	ankyrin repeat domain 28	-5.03	0.00	0.51
1434630_at	Ankrd28	ankyrin repeat domain 28	-6.07	0.00	0.46
1437414_at	Zfp217	zinc finger protein 217	-5.03	0.00	0.52
1456721_at	Thsd7a	thrombospondin. type I. domain containing 7A	-5.07	0.00	0.60
1453067_at	Apitd1	apoptosis-inducing. TAF9-like domain 1	-5.09	0.00	0.53
1424092_at	Epb4.1	erythrocyte protein band 4.1	-5.09	0.00	0.45
1424766_at	Ercc6l	excision repair cross-complementing rodent repair deficiency complementation group 6-like	-5.10	0.00	0.34
1451400_at	Gemin8	gem (nuclear organelle) associated protein 8	-5.11	0.00	0.60
1423755_at	Zcchc8	zinc finger. CCHC domain containing 8	-5.12	0.00	0.59
1451306_at	Cdca7l	cell division cycle associated 7 like	-5.13	0.00	0.41
1438161_s_at	Rfc4	replication factor C (activator 1) 4	-5.13	0.00	0.35
1424321_at	Rfc4	replication factor C (activator 1) 4	-5.58	0.00	0.40
1437187_at	E2f7	E2F transcription factor 7	-5.14	0.00	0.30
1435595_at	1810011O10Rik	RIKEN cDNA 1810011O10 gene	-5.16	0.00	0.42
1451415_at	1810011O10Rik	RIKEN cDNA 1810011O10 gene	-7.98	0.00	0.18
1428648_at	Cand1	cullin associated and neddylation disassociated 1	-5.17	0.00	0.77
1447787_x_at	Gjc1	gap junction protein. gamma 1	-5.20	0.00	0.46
1449094_at	Gjc1	gap junction protein. gamma 1	-6.29	0.00	0.43
1433960_at	Isg20l2	interferon stimulated exonuclease gene 20-like 2	-5.21	0.00	0.65
1416167_at	Prdx4	peroxiredoxin 4	-5.21	0.00	0.69
1436892_at	Spred2	sprouty-related. EVH1 domain containing 2	-5.21	0.00	0.46
1434403_at	Spred2	sprouty-related. EVH1 domain containing 2	-6.49	0.00	0.47
1456079_x_at	Apex1	apurinic/apyrimidinic endonuclease 1	-5.26	0.00	0.64
1416135_at	Apex1	apurinic/apyrimidinic endonuclease 1	-5.74	0.00	0.65
1433935_at	AU020206	expressed sequence AU020206	-5.28	0.00	0.31
1416034_at	Cd24a	CD24a antigen	-5.28	0.00	0.26
1448182_a_at	Cd24a	CD24a antigen	-7.99	0.00	0.29
1437502_x_at	Cd24a	CD24a antigen	-8.51	0.00	0.29

Probeset ID	*Symbol*	*Gene Name*	*d-value*	*q-value*	*R-fold*
1424208_at	Ptger4	prostaglandin E receptor 4 (subtype EP4)	-5.28	0.00	0.48
1417037_at	Orc6l	origin recognition complex. subunit 6-like (S. cerevisiae)	-5.30	0.00	0.56
1448224_at	Tfam	transcription factor A. mitochondrial	-5.30	0.00	0.60
1442280_at	D2Ertd750e	DNA segment. Chr 2. ERATO Doi 750. expressed	-5.31	0.00	0.40
1420081_s_at	D2Ertd750e	DNA segment. Chr 2. ERATO Doi 750. expressed	-5.50	0.00	0.35
1423463_a_at	D2Ertd750e	DNA segment. Chr 2. ERATO Doi 750. expressed	-6.28	0.00	0.34
1442134_at	Prr11	proline rich 11	-5.31	0.00	0.50
1444257_at	Prr11	proline rich 11	-6.72	0.00	0.39
1434079_s_at	Mcm2	minichromosome maintenance deficient 2 mitotin (S. cerevisiae)	-5.31	0.00	0.35
1448777_at	Mcm2	minichromosome maintenance deficient 2 mitotin (S. cerevisiae)	-5.56	0.00	0.29
1452459_at	Aspm	asp (abnormal spindle)-like. microcephaly associated (Drosophila)	-5.32	0.00	0.43
1422814_at	Aspm	asp (abnormal spindle)-like. microcephaly associated (Drosophila)	-6.80	0.00	0.23
1451845_a_at	Ptrh2	peptidyl-tRNA hydrolase 2	-5.32	0.00	0.66
1452917_at	Rfc5	replication factor C (activator 1) 5	-5.34	0.00	0.40
1452912_at	Dscc1	defective in sister chromatid cohesion 1 homolog (S. cerevisiae)	-5.37	0.00	0.23
1451119_a_at	Fbln1	fibulin 1	-5.38	0.00	0.38
1422540_at	Fbln1	fibulin 1	-6.46	0.00	0.48
1436723_at	Cenpi	centromere protein I	-5.39	0.00	0.31
1447877_x_at	Dnmt1	DNA methyltransferase (cytosine-5) 1	-5.39	0.00	0.31
1435122_x_at	Dnmt1	DNA methyltransferase (cytosine-5) 1	-5.44	0.00	0.30
1422946_a_at	Dnmt1	DNA methyltransferase (cytosine-5) 1	-5.79	0.00	0.30
1417511_at	Lyar	Ly1 antibody reactive clone	-5.43	0.00	0.47
1426790_at	Ssrp1	structure specific recognition protein 1	-5.45	0.00	0.50
1426789_s_at	Ssrp1	structure specific recognition protein 1	-5.50	0.00	0.55
1426788_a_at	Ssrp1	structure specific recognition protein 1	-6.15	0.00	0.54
1418121_at	Vrk3	vaccinia related kinase 3	-5.46	0.00	0.77
1427707_a_at	Stil	Scl/Tal1 interrupting locus	-5.46	0.00	0.36
1435254_at	Plxnb1	plexin B1	-5.46	0.00	0.45
1427959_at	Abhd10	abhydrolase domain containing 10	-5.47	0.00	0.52
1460240_a_at	Hnrnpc	heterogeneous nuclear ribonucleoprotein C	-5.47	0.00	0.77
1428052_a_at	Zmym1	zinc finger. MYM domain containing 1	-5.48	0.00	0.55
1426533_at	Nol5a	nucleolar protein 5A	-5.49	0.00	0.47
1455035_s_at	Nol5a	nucleolar protein 5A	-6.21	0.00	0.45
1428291_at	Exosc8	exosome component 8	-5.50	0.00	0.46
1448134_at	X99384	cDNA sequence X99384	-5.51	0.00	0.49
1442058_s_at	Psmc3ip	proteasome (prosome. macropain) 26S subunit. ATPase 3. interacting protein	-5.52	0.00	0.34
1425271_at	Psmc3ip	proteasome (prosome. macropain) 26S subunit. ATPase 3. interacting protein	-6.02	0.00	0.41
1429109_at	Msl2l1	male-specific lethal 2-like 1 (Drosophila)	-5.53	0.00	0.62
1416544_at	Ezh2	enhancer of zeste homolog 2 (Drosophila)	-5.53	0.00	0.29
1455495_at	Abl2	v-abl Abelson murine leukemia viral oncogene homolog 2 (arg. Abelson-related gene)	-5.53	0.00	0.53
1455682_at	Abl2	v-abl Abelson murine leukemia viral oncogene homolog 2 (arg. Abelson-related gene)	-7.07	0.00	0.48
1438852_x_at	Mcm6	minichromosome maintenance deficient 6 (MIS5 homolog. S. pombe) (S. cerevisiae)	-5.54	0.00	0.20
1416251_at	Mcm6	minichromosome maintenance deficient 6 (MIS5 homolog. S. pombe) (S. cerevisiae)	-6.47	0.00	0.24
1416641_at	Lig1	ligase I. DNA. ATP-dependent	-5.54	0.00	0.31
1427094_at	Pole2	polymerase (DNA directed). epsilon 2 (p59 subunit)	-5.54	0.00	0.26
1417586_at	Timeless	timeless homolog (Drosophila)	-6.44	0.00	0.31
1417587_at	Timeless	timeless homolog (Drosophila)	-5.55	0.00	0.32

Probeset ID	***Symbol***	***Gene Name***	***d-value***	***q-value***	***R-fold***
1426817_at	Mki67	antigen identified by monoclonal antibody Ki 67	-5.56	0.00	0.20
1428029_a_at	H2afv	H2A histone family. member V	-5.58	0.00	0.60
1427282_a_at	Fxn	frataxin	-5.58	0.00	0.53
1426825_at	Fmnl3	formin-like 3	-5.59	0.00	0.65
1447275_at	Bbs12	Bardet-Biedl syndrome 12 (human)	-5.61	0.00	0.52
1426751_s_at	Nup107	nucleoporin 107	-5.61	0.00	0.53
1417457_at	Cks2	CDC28 protein kinase regulatory subunit 2	-5.63	0.00	0.47
1417458_s_at	Cks2	CDC28 protein kinase regulatory subunit 2	-6.54	0.00	0.43
1434365_a_at	BC055324	cDNA sequence BC055324	-5.64	0.00	0.35
1437186_at	BC055324	cDNA sequence BC055324	-6.93	0.00	0.38
1456077_x_at	Cdc25c	cell division cycle 25 homolog C (S. pombe)	-5.66	0.00	0.41
1422252_a_at	Cdc25c	cell division cycle 25 homolog C (S. pombe)	-5.83	0.00	0.52
1426612_at	Tipin	timeless interacting protein	-5.66	0.00	0.49
1434495_at	Patz1	POZ (BTB) and AT hook containing zinc finger 1	-5.67	0.00	0.49
1429739_a_at	Patz1	POZ (BTB) and AT hook containing zinc finger 1	-6.62	0.00	0.49
1450842_a_at	Cenpa	centromere protein A	-5.67	0.00	0.31
1455523_at	Cstf2	cleavage stimulation factor. 3' pre-RNA subunit 2	-5.69	0.00	0.64
1419644_at	Cstf2	cleavage stimulation factor. 3' pre-RNA subunit 2	-6.31	0.00	0.50
1419645_at	Cstf2	cleavage stimulation factor. 3' pre-RNA subunit 2	-8.31	0.00	0.50
1453683_a_at	Cep55	centrosomal protein 55	-5.70	0.00	0.26
1452242_at	Cep55	centrosomal protein 55	-8.10	0.00	0.23
1459651_s_at	OTTMUSG0000 0026223	predicted gene. OTTMUSG00000026223	-5.72	0.00	0.68
1416757_at	Zwilch	Zwilch. kinetochore associated. homolog (Drosophila)	-5.75	0.00	0.33
1456227_x_at	Rbbp7	retinoblastoma binding protein 7	-5.76	0.00	0.65
1415775_at	Rbbp7	retinoblastoma binding protein 7	-7.93	0.00	0.62
1448519_at	Tead2	TEA domain family member 2	-5.77	0.00	0.40
1438009_at	Hist1h2ae	histone cluster 1. H2ae	-5.78	0.00	0.27
1416915_at	Msh6	mutS homolog 6 (E. coli)	-5.80	0.00	0.28
1423775_s_at	Prc1	protein regulator of cytokinesis 1	-5.81	0.00	0.30
1423774_a_at	Prc1	protein regulator of cytokinesis 1	-6.35	0.00	0.29
1434190_at	Sms	spermine synthase	-5.81	0.00	0.57
1421052_a_at	Sms	spermine synthase	-6.65	0.00	0.37
1416868_at	Cdkn2c	cyclin-dependent kinase inhibitor 2C (p18. inhibits CDK4)	-5.82	0.00	0.56
1424629_at	Brca1	breast cancer 1	-5.82	0.00	0.22
1451417_at	Brca1	breast cancer 1	-5.90	0.00	0.38
1424630_a_at	Brca1	breast cancer 1	-6.79	0.00	0.32
1417681_at	Nudt21	nudix (nucleoside diphosphate linked moiety X)-type motif 21	-5.82	0.00	0.64
1440299_at	E330016A19Rik	RIKEN cDNA E330016A19 gene	-5.83	0.00	0.54
1429268_at	2610318N02Rik	RIKEN cDNA 2610318N02 gene	-5.84	0.00	0.50
1418084_at	Nrp1	neuropilin 1	-5.86	0.00	0.29
1448944_at	Nrp1	neuropilin 1	-6.18	0.00	0.29
1448943_at	Nrp1	neuropilin 1	-7.26	0.00	0.32
1437611_x_at	Kif2c	kinesin family member 2C	-5.88	0.00	0.29
1449060_at	Kif2c	kinesin family member 2C	-6.69	0.00	0.25
1459861_s_at	OTTMUSG0000 0006609	predicted gene. OTTMUSG00000006609	-5.90	0.00	0.59
1416076_at	Ccnb1-rs1	cyclin B1. related sequence 1	-5.90	0.00	0.24
1448205_at	Ccnb1-rs1	cyclin B1. related sequence 1	-7.33	0.00	0.29

Probeset ID	*Symbol*	*Gene Name*	*d-value*	*q-value*	*R-fold*
1423416_at	Smarcc1	SWI/SNF related. matrix associated. actin dependent regulator of chromatin. subfamily c. member 1	-5.92	0.00	0.52
1449207_a_at	Kif20a	kinesin family member 20A	-5.92	0.00	0.26
1417911_at	Ccna2	cyclin A2	-5.93	0.00	0.25
1417910_at	Ccna2	cyclin A2	-6.57	0.00	0.26
1452040_a_at	Cdca3	cell division cycle associated 3	-5.93	0.00	0.31
1449348_at	Mpp6	membrane protein. palmitoylated 6 (MAGUK p55 subfamily member 6)	-5.94	0.00	0.34
1437580_s_at	Nek2	NIMA (never in mitosis gene a)-related expressed kinase 2	-5.96	0.00	0.31
1417299_at	Nek2	NIMA (never in mitosis gene a)-related expressed kinase 2	-6.29	0.00	0.39
1433862_at	Espl1	extra spindle poles-like 1 (S. cerevisiae)	-5.96	0.00	0.25
1428090_at	Ptcd3	pentatricopeptide repeat domain 3	-5.97	0.00	0.60
1426002_a_at	Cdc7	cell division cycle 7 (S. cerevisiae)	-5.99	0.00	0.28
1429172_a_at	Ncapg	on-SMC condensin I complex. subunit G	-6.02	0.00	0.26
1417926_at	Ncapg2	non-SMC condensin II complex. subunit G2	-6.03	0.00	0.20
1429171_a_at	Ncapg	on-SMC condensin I complex. subunit G	-6.84	0.00	0.19
1458447_at	Cenpf	centromere protein F	-6.02	0.00	0.65
1427161_at	Cenpf	centromere protein F	-7.00	0.00	0.22
1449708_s_at	Chek1	checkpoint kinase 1 homolog (S. pombe)	-6.04	0.00	0.30
1450677_at	Chek1	checkpoint kinase 1 homolog (S. pombe)	-6.62	0.00	0.28
1450692_at	Kif4	kinesin family member 4	-6.05	0.00	0.32
1438811_at	Dlgap5	discs. large (Drosophila) homolog-associated protein 5	-6.05	0.00	0.53
1455730_at	Dlgap5	discs. large (Drosophila) homolog-associated protein 5	-6.52	0.00	0.34
1448191_at	Plk1	polo-like kinase 1 (Drosophila)	-6.06	0.00	0.28
1418264_at	Cenpk	centromere protein K	-6.07	0.00	0.39
1423311_s_at	Tpbg	trophoblast glycoprotein	-6.09	0.00	0.34
1423092_at	Incenp	inner centromere protein	-6.11	0.00	0.33
1439436_x_at	Incenp	inner centromere protein	-8.72	0.00	0.30
1416410_at	Pafah1b3	platelet-activating factor acetylhydrolase. isoform 1b. alpha1 subunit	-6.14	0.00	0.36
1456280_at	Clspn	claspin homolog (Xenopus laevis)	-6.14	0.00	0.25
1455727_at	Zrsr2	zinc finger (CCCH type). RNA binding motif and serine/arginine rich 2	-6.16	0.00	0.64
1453181_x_at	Plscr1	phospholipid scramblase 1	-6.17	0.00	0.30
1429527_a_at	Plscr1	phospholipid scramblase 1	-7.49	0.00	0.29
1435114_at	Wdhd1	WD repeat and HMG-box DNA binding protein 1	-6.17	0.00	0.32
1418380_at	Terf1	telomeric repeat binding factor 1	-6.21	0.00	0.39
1415730_at	5730453I16Rik	RIKEN cDNA 5730453I16 gene	-6.21	0.00	0.75
1455218_at	6330503K22Rik	RIKEN cDNA 6330503K22 gene	-6.23	0.00	0.56
1460669_at	Ilf3	interleukin enhancer binding factor 3	-6.24	0.00	0.64
1422460_at	Mad2l1	MAD2 (mitotic arrest deficient. homolog)-like 1 (yeast)	-6.30	0.00	0.33
1429326_at	Cenpl	centromere protein L	-6.31	0.00	0.44
1446196_at	Hmga2	high mobility group AT-hook 2	-6.32	0.00	0.28
1450781_at	Hmga2	high mobility group AT-hook 2	-6.77	0.00	0.23
1450780_s_at	Hmga2	high mobility group AT-hook 2	-7.35	0.00	0.31
1422851_at	Hmga2	high mobility group AT-hook 2	-7.55	0.00	0.29
1428480_at	Cdca8	cell division cycle associated 8	-6.34	0.00	0.34
1428481_s_at	Cdca8	cell division cycle associated 8	-6.55	0.00	0.31
1436847_s_at	Cdca8	cell division cycle associated 8	-6.61	0.00	0.29
1447363_s_at	Bub1b	budding uninhibited by benzimidazoles 1 homolog. beta (S. cerevisiae)	-6.37	0.00	0.22
1416961_at	Bub1b	budding uninhibited by benzimidazoles 1 homolog. beta (S. cerevisiae)	-6.76	0.00	0.25
1448113_at	Stmn1	stathmin 1	-6.95	0.00	0.35

Probeset ID	*Symbol*	*Gene Name*	*d-value*	*q-value*	*R-fold*
1415849_s_at	Stmn1	stathmin 1	-6.39	0.00	0.30
1420616_at	Ash2l	ash2 (absent. small. or homeotic)-like (Drosophila)	-6.39	0.00	0.63
1448526_at	Kpnb1	karyopherin (importin) beta 1	-6.41	0.00	0.71
1435005_at	Cenpe	centromere protein E	-6.42	0.00	0.26
1439040_at	Cenpe	centromere protein E	-8.57	0.00	0.27
1418334_at	Dbf4	DBF4 homolog (S. cerevisiae)	-6.43	0.00	0.35
1416177_at	Rbmxrt	RNA binding motif protein. X chromosome retrogene	-6.44	0.00	0.53
1419513_a_at	Ect2	ect2 oncogene	-6.46	0.00	0.24
1436808_x_at	Mcm5	minichromosome maintenance deficient 5. cell division cycle 46 (S. cerevisiae)	-6.47	0.00	0.23
1415945_at	Mcm5	minichromosome maintenance deficient 5. cell division cycle 46 (S. cerevisiae)	-6.51	0.00	0.21
1417445_at	Ndc80	NDC80 homolog. kinetochore complex component (S. cerevisiae)	-6.47	0.00	0.27
1456475_s_at	Prkar2b	protein kinase. cAMP dependent regulatory. type II beta	-6.49	0.00	0.19
1438664_at	Prkar2b	protein kinase. cAMP dependent regulatory. type II beta	-6.62	0.00	0.20
1430640_a_at	Prkar2b	protein kinase. cAMP dependent regulatory. type II beta	-7.68	0.00	0.22
1449171_at	Ttk	Ttk protein kinase	-6.51	0.00	0.19
1434767_at	C79407	expressed sequence C79407	-6.55	0.00	0.26
1458374_at	C79407	expressed sequence C79407	-7.66	0.00	0.30
1434789_at	Depdc1b	DEP domain containing 1B	-6.56	0.00	0.32
1418027_at	Exo1	exonuclease 1	-6.56	0.00	0.29
1418026_at	Exo1	exonuclease 1	-6.79	0.00	0.22
1423127_at	Impa1	inositol (myo)-1(or 4)-monophosphatase 1	-6.62	0.00	0.78
1433685_a_at	6430706D22Rik	RIKEN cDNA 6430706D22 gene	-6.64	0.00	0.42
1436708_x_at	Mcm4	minichromosome maintenance deficient 4 homolog (S. cerevisiae)	-6.65	0.00	0.26
1416214_at	Mcm4	minichromosome maintenance deficient 4 homolog (S. cerevisiae)	-7.49	0.00	0.28
1428232_at	Cpsf6	cleavage and polyadenylation specific factor 6	-6.67	0.00	0.68
1428233_at	Cpsf6	cleavage and polyadenylation specific factor 6	-6.78	0.00	0.54
1424128_x_at	Aurkb	aurora kinase B	-6.67	0.00	0.23
1451246_s_at	Aurkb	aurora kinase B	-7.44	0.00	0.28
1416118_at	Trim59	tripartite motif-containing 59	-6.67	0.00	0.48
1418540_a_at	Ptpre	protein tyrosine phosphatase. receptor type. E	-6.68	0.00	0.43
1418539_a_at	Ptpre	protein tyrosine phosphatase. receptor type. E	-7.46	0.00	0.39
1416030_a_at	Mcm7	minichromosome maintenance deficient 7 (S. cerevisiae)	-6.68	0.00	0.27
1439269_x_at	Mcm7	minichromosome maintenance deficient 7 (S. cerevisiae)	-6.70	0.00	0.29
1416031_s_at	Mcm7	minichromosome maintenance deficient 7 (S. cerevisiae)	-6.72	0.00	0.30
1438320_s_at	Mcm7	minichromosome maintenance deficient 7 (S. cerevisiae)	-6.94	0.00	0.26
1450048_a_at	Idh2	isocitrate dehydrogenase 2 (NADP+). mitochondrial	-6.69	0.00	0.33
1425142_a_at	Hnrnpd	heterogeneous nuclear ribonucleoprotein D	-6.72	0.00	0.38
1416488_at	Ccng2	cyclin G2	-6.72	0.00	0.50
1432187_at	Nup43	nucleoporin 43	-6.77	0.00	0.52
1432188_s_at	Nup43	nucleoporin 43	-6.94	0.00	0.51
1438015_at	Dkc1	dyskeratosis congenita 1. dyskerin homolog (human)	-6.80	0.00	0.39
1441788_s_at	Dkc1	dyskeratosis congenita 1. dyskerin homolog (human)	-7.63	0.00	0.29
1438016_at	Dkc1	dyskeratosis congenita 1. dyskerin homolog (human)	-10.07	0.00	0.33
1419270_a_at	Dut	deoxyuridine triphosphatase	-6.80	0.00	0.32
1452241_at	Topbp1	topoisomerase (DNA) II binding protein 1	-6.81	0.00	0.39
1424046_at	Bub1	budding uninhibited by benzimidazoles 1 homolog (S. cerevisiae)	-6.87	0.00	0.21
1416043_at	Nasp	nuclear autoantigenic sperm protein (histone-binding)	-6.90	0.00	0.30
1416042_s_at	Nasp	nuclear autoantigenic sperm protein (histone-binding)	-9.01	0.00	0.28
1416664_at	Cdc20	cell division cycle 20 homolog (S. cerevisiae)	-6.95	0.00	0.28

Probeset ID	*Symbol*	*Gene Name*	*d-value*	*q-value*	*R-fold*
1439394_x_at	Cdc20	cell division cycle 20 homolog (S. cerevisiae)	-7.33	0.00	0.42
1439377_x_at	Cdc20	cell division cycle 20 homolog (S. cerevisiae)	-7.97	0.00	0.27
1443381_at	Etv4	ets variant gene 4 (E1A enhancer binding protein. E1AF)	-6.95	0.00	0.63
1423232_at	Etv4	ets variant gene 4 (E1A enhancer binding protein. E1AF)	-7.81	0.00	0.31
1424971_at	Ccdc99	coiled-coil domain containing 99	-6.96	0.00	0.29
1423813_at	Kif22	kinesin family member 22	-6.97	0.00	0.29
1437716_x_at	Kif22	kinesin family member 22	-7.07	0.00	0.33
1451128_s_at	Kif22	kinesin family member 22	-7.24	0.00	0.26
1416065_a_at	Ankrd10	ankyrin repeat domain 10	-6.99	0.00	0.51
1429304_at	Ankrd10	ankyrin repeat domain 10	-7.59	0.00	0.53
1448199_at	Ankrd10	ankyrin repeat domain 10	-8.30	0.00	0.49
1418827_at	Thex1	three prime histone mRNA exonuclease 1	-7.00	0.00	0.49
1418828_at	Thex1	three prime histone mRNA exonuclease 1	-7.60	0.00	0.37
1423920_at	Ncaph	non-SMC condensin I complex. subunit H	-7.05	0.00	0.35
1436707_x_at	Ncaph	non-SMC condensin I complex. subunit H	-7.37	0.00	0.32
1418369_at	Prim1	DNA primase. p49 subunit	-7.09	0.00	0.27
1449061_a_at	Prim1	DNA primase. p49 subunit	-7.25	0.00	0.25
1436942_at	A930035D04Rik	RIKEN cDNA A930035D04 gene	-7.10	0.00	0.47
1427321_s_at	Cxadr	coxsackievirus and adenovirus receptor	-7.10	0.00	0.42
1453282_at	Cxadr	coxsackievirus and adenovirus receptor	-13.46	0.00	0.21
1416258_at	Tk1	thymidine kinase 1	-7.10	0.00	0.23
1428069_at	Cdca7	cell division cycle associated 7	-7.13	0.00	0.27
1417019_a_at	Cdc6	cell division cycle 6 homolog (S. cerevisiae)	-7.17	0.00	0.20
1452151_at	Gramd4	GRAM domain containing 4	-7.17	0.00	0.51
1454011_a_at	Rpa2	replication protein A2	-7.17	0.00	0.42
1416433_at	Rpa2	replication protein A2	-8.66	0.00	0.41
1453064_at	Etaa1	Ewing's tumor-associated antigen 1	-7.18	0.00	0.37
1416073_a_at	Pcnt	pericentrin (kendrin)	-7.18	0.00	0.54
1427911_at	Tmem173	transmembrane protein 173	-7.21	0.00	0.51
1447621_s_at	Tmem173	transmembrane protein 173	-10.97	0.00	0.32
1456326_at	Gm784	gene model 784. (NCBI)	-7.24	0.00	0.25
1455488_at	6230416J20Rik	RIKEN cDNA 6230416J20 gene	-7.26	0.00	0.31
1429665_at	6230416J20Rik	RIKEN cDNA 6230416J20 gene	-7.67	0.00	0.49
1440227_at	Slc5a3	solute carrier family 5 (inositol transporters). member 3	-7.33	0.00	0.31
1447585_s_at	Slc5a3	solute carrier family 5 (inositol transporters). member 3	-8.49	0.00	0.41
1435484_at	Slc5a3	solute carrier family 5 (inositol transporters). member 3	-9.84	0.00	0.24
1436512_at	Arl4c	ADP-ribosylation factor-like 4C	-7.36	0.00	0.32
1454788_at	Arl4c	ADP-ribosylation factor-like 4C	-11.02	0.00	0.34
1416558_at	Melk	maternal embryonic leucine zipper kinase	-7.38	0.00	0.23
1428061_at	Hat1	histone aminotransferase 1	-7.46	0.00	0.36
1452598_at	Gins1	GINS complex subunit 1 (Psf1 homolog)	-7.48	0.00	0.27
1442095_at	Asxl3	additional sex combs like 3 (Drosophila)	-7.50	0.00	0.38
1435892_at	Asxl3	additional sex combs like 3 (Drosophila)	-7.59	0.00	0.43
1416698_a_at	Cks1b	CDC28 protein kinase 1b	-7.52	0.00	0.55
1448441_at	Cks1b	CDC28 protein kinase 1b	-7.89	0.00	0.43
1417821_at	D17H6S56E-5	DNA segment. Chr 17. human D6S56E 5	-7.53	0.00	0.20
1417822_at	D17H6S56E-5	DNA segment. Chr 17. human D6S56E 5	-8.93	0.00	0.24
1423847_at	Ncapd2	non-SMC condensin I complex. subunit D2	-7.66	0.00	0.26
1422430_at	Fignl1	fidgetin-like 1	-7.69	0.00	0.27

Probeset ID	*Symbol*	*Gene Name*	*d-value*	*q-value*	*R-fold*
1423714_at	Asf1b	ASF1 anti-silencing function 1 homolog B (S. cerevisiae)	-7.76	0.00	0.39
1451105_at	Vash2	vasohibin 2	-7.79	0.00	0.30
1424144_at	Cdt1	chromatin licensing and DNA replication factor 1	-7.79	0.00	0.21
1424143_a_at	Cdt1	chromatin licensing and DNA replication factor 1	-8.20	0.00	0.23
1416155_at	Hmgb3	high mobility group box 3	-7.81	0.00	0.33
1450886_at	Gsg2	germ cell-specific gene 2	-7.84	0.00	0.25
1418281_at	Rad51	RAD51 homolog (S. cerevisiae)	-7.89	0.00	0.28
1451323_at	Zfp7	zinc finger protein 7	-8.07	0.00	0.32
1448314_at	Cdc2a	cell division cycle 2 homolog A (S. pombe)	-8.10	0.00	0.32
1418678_at	Has2	hyaluronan synthase 2	-8.11	0.00	0.06
1449169_at	Has2	hyaluronan synthase 2	-8.18	0.00	0.08
1433567_at	Gmps	guanine monphosphate synthetase	-8.32	0.00	0.57
1438434_at	Arhgap11a	Rho GTPase activating protein 11A	-8.77	0.00	0.31
1422243_at	Fgf7	fibroblast growth factor 7	-8.81	0.00	0.17
1438405_at	Fgf7	fibroblast growth factor 7	-10.53	0.00	0.15
1449839_at	Casp3	caspase 3	-8.96	0.00	0.49
1423455_at	ENSMUSG00000 068659	predicted gene. ENSMUSG00000068659	-9.08	0.00	0.57
1437313_x_at	Hmgb2	high mobility group box 2	-9.09	0.00	0.21
1452534_a_at	Hmgb2	high mobility group box 2	-10.42	0.00	0.16
1421534_at	LOC14210	hypothetical LOC14210	-9.10	0.00	0.31
1455990_at	Kif23	kinesin family member 23	-9.11	0.00	0.25
1453748_a_at	Kif23	kinesin family member 23	-9.89	0.00	0.22
1456531_x_at	Prpf19	PRP19/PSO4 pre-mRNA processing factor 19 homolog (S. cerevisiae)	-9.20	0.00	0.59
1426473_at	Dnajc9	DnaJ (Hsp40) homolog. subfamily C. member 9	-9.55	0.00	0.38
1453366_at	Tdrkh	tudor and KH domain containing protein	-9.76	0.00	0.20
1460168_at	Slbp	stem-loop binding protein	-10.05	0.00	0.40
1434258_s_at	Phactr4	phosphatase and actin regulator 4	-10.06	0.00	0.55
1454659_at	Dctd	dCMP deaminase	-10.16	0.00	0.34
1434660_at	Alkbh1	alkB. alkylation repair homolog 1 (E. coli)	-11.02	0.00	0.63
1417155_at	Mycn	v-myc myelocytomatosis viral related oncogene. neuroblastoma derived (avian)	-12.54	0.00	0.17
1415834_at	Dusp6	dual specificity phosphatase 6	-16.40	0.00	0.09
