# Supplementary material for: Reversible, interrelated mRNA and miRNA expression patterns in the transcriptome of Rasless fibroblasts: functional and mechanistic implications
Source: BMC Genomics. 2013 Oct 25;14:731. doi: 10.1186/1471-2164-14-731 (PMC4007593; doi:10.1186/1471-2164-14-731)
Supplement: Additional file 5: Table S5 — Functional annotation of differentially expressed repressed and induced genes of Rasless MEFs whose transcriptional pattern is reversed in both BRAF- and MEK1-rescued MEFs. The GeneCodis functional annotation tool was used on the list of genes included in Additional file 4: Table S4. Section S5A shows the results for the repressed genes while Section S5B shows the results from the induced genes. [file 1471-2164-14-731-S5.pdf]

Table S5. **Functional annotation of differentially expressed, repressed (S5A) and induced (S5B) genes of Rasless MEFs whose transcriptional pattern is reversed in both BRAF- and MEK1-rescued MEFs.**

The GeneCodis (Gene Annotation Co-occurrence Discovery) functional annotation tool (http://genecodis.dacya.ucm.es) was used to identify statistically significant functional associations linking particular gene subsets contained within the list of Rasless repressed (S5A) or induced (S5B) loci included in Table S4 (Additional file 4, FDR=0.01) to specific Biological Processes from the *Gene Ontology* (GO) database. The column labelled "*Functional category*" identifies the specific functional GO term recognized in each case for the corresponding groups of loci listed under the column labelled "Genes". GO terms descriptions falling under the same, high level, general functional categories (Cell Cycle, DNA and RNA processing, Metabolite processing, DNA damage response, etc) are depicted with distinct, common colors. The column labelled "*Number of Genes*" indicates the specific number of genes linked to the indicated functionality, out of the total number (in parenthesis) of genes recognized by GeneCodis in the list of repressed genes of Rasless cells. The column labeled "*Corrected p-value*" refers to the statistical significance of the functional associations identified, and contains p-values calculated cases using the Hypergeometric Distribution and subsequently corrected by implementing the False Discovery Rate method of Benjamini and Hochberg [113].

S5A. Functional annotation of repressed genes from Rasless MEFs that are induced in both BRAF- and MEK1-rescued MEFs

Functional Category	*Number of Genes*	*Corrected p-value*	*Genes repressed in Table S4*
**CELL CYCLE (MITOTIC AND MEIOTIC DIVISION)**			
**GO:0007049: cell cycle**	73(314)	2,20E-65	Ncaph, Timeless, Ercc6l, Mcm5, Cks1b, Plk1, Sass6, Aurkb, Kif11, Bub1, Ndc80, Aspm, Mad2l1, Cdc5l, Dscc1, Cit, Mcm4, Brca1, Mcm3, Rad21, Dlgap5, Sgol1, Ccdc99, Ncapd2, Ccnb2, Cdc25c, Casc5, Lig1, Ncapd3, Kntc1, Rbbp4, Gsg2, Ccng2, Nasp, Chek1, Pttg1, Usp3, Ccna2, Cdc7, Hmga2, Bub1b, Dbf4, Zwilch, Cdca3, Cdc20, Kif2c, Clspn, Cenpe, Tipin, Chtf18, Mcm7, Mastl, Cdkn2c, Prc1, Cdc6, Pkmyt1, Mcm2, Cep55, E2f7, Mcm6, C79407, Fancd2, Nek2, Cdca8, Incenp, Khdrbs1, Nup43, Rbl1, Terf1, Uhrf1, Cdt1, Cks2, Pogz
**GO:0051301: cell division**	48(314)	3,06E-45	Ncaph, Timeless, Ercc6l, Mcm5, Cks1b, Plk1, Aurkb, Kif11, Bub1, Ndc80, Aspm, Mad2l1, Cit, Rad21, Sgol1, Ccdc99, Ncapd2, Ccnb2, Cdc25c, Casc5, Lig1, Ncapd3, Kntc1, Ccng2, Pttg1, Ccna2, Cdc7, Hmga2, Bub1b, Zwilch, Cdca3, Cdc20, Kif2c, Cenpe, Tipin, Mastl, Prc1, Cdc6, Cep55, C79407, Nek2, Cdca8, Incenp, Nup43, Cdca7, Terf1, Cks2, Pogz
**GO:0007067: mitosis**	40(314)	4,62E-40	Ncaph, Timeless, Ercc6l, Plk1, Aurkb, Kif11, Bub1, Ndc80, Aspm, Mad2l1, Cit, Rad21, Sgol1, Ccdc99, Ncapd2, Ccnb2, Cdc25c, Casc5, Ncapd3, Kntc1, Ccng2, Pttg1, Ccna2, Hmga2, Bub1b, Zwilch, Cdca3, Cdc20, Kif2c, Cenpe, Tipin, Mastl, Cdc6, Cep55, C79407, Nek2, Cdca8, Incenp, Nup43, Terf1
**GO:0007059: chromosome segregation**	16(314)	7,11E-18	Srpk1, Bub1, Ndc80, Ctcf, Brca1, Rad21, Sgol1, Casc5, Espl1, Pttg1, Top2a, Kif2c, Cenpf, Nek2, Incenp, Nup43
**GO:0033261: regulation of S phase**	5(314)	4,65E-08	Timeless, Slbp, Chek1, Cdc7, Tipin
**GO:0051726: regulation of cell cycle**	10(314)	9,42E-08	Skp2, Ccnb2, Dtl, Zfp703, Ccng2, Ccna2, Mastl, Tacc3, Fignl1, Rbl1
**GO:0007076: mitotic chromosome condensation**	5(314)	8,05E-07	Ncaph, Ncapd2, Ncapd3, Ncapg, Akap8
**GO:0008608: attachment of spindle microtubules to kinetochore**	4(314)	3,05E-06	Ndc80, Sgol1, Casc5, Cenpe
**GO:0000070: mitotic sister chromatid segregation**	4(314)	1,29E-05	Mad2l1, Cit, Espl1, Nek2
**GO:0000076: DNA replication checkpoint**	3(314)	7,90E-05	Clspn, Tipin, Cdt1
**GO:0000087: M phase of mitotic cell cycle**	3(314)	7,90E-05	Dlgap5, Cdc25c, Cenpf
**GO:0007131: reciprocal meiotic recombination**	4(314)	0,000185328	Msh6, Trip13, Topbp1, Rad51
**GO:0000086: G2/M transition of mitotic cell cycle**	4(314)	0,000429187	Skp2, Cit, Chek1, Mastl
**GO:0051298: centrosome duplication**	3(314)	0,000481823	Sass6, Brca1, 6330503K22Rik
**GO:0000132: establishment of mitotic**	3(314)	0,000727738	Ndc80, Ccdc99, Cenpa

Functional Category	*Number of Genes*	*Corrected p-value*	*Genes repressed in Table S4*
spindle orientation			
GO:0007094: mitotic cell cycle spindle assembly checkpoint	3(314)	0,00105825	Mad2l1, Cenpe, Terf1
GO:0090007: regulation of mitotic anaphase	2(314)	0,00134703	Plk1, Cdc6
GO:0051984: positive regulation of chromosome segregation	2(314)	0,00134703	Cenpe, Cdc6
GO:0007093: mitotic cell cycle checkpoint	3(314)	0,0013998	Mad2l1, Kntc1, Zwilch
GO:0007126: meiosis	5(314)	0,00332418	Mki67, Exo1, Psmc3ip, Nek2, Rad51
GO:0071173: spindle assembly checkpoint	2(314)	0,00337246	Bub1, Casc5
GO:0000910: cytokinesis	4(314)	0,00337984	Aurkb, Cit, Prc1, Incenp
GO:0007127: meiosis I	2(314)	0,00552327	Espl1, Cks2
GO:0051983: regulation of chromosome segregation	2(314)	0,00552327	Bub1, Kif2c
GO:0007091: mitotic metaphase/anaphase transition	2(314)	0,00552327	Cit, Bub1b
GO:0045143: homologous chromosome segregation	2(314)	0,00552327	Espl1, Pttg1
GO:0000089: mitotic metaphase	2(314)	0,00797998	Ndc80, Cdca8
GO:0007099: centriole replication	2(314)	0,0105499	Sass6, 6330503K22Rik
GO:0010389: regulation of G2/M transition of mitotic cell cycle	2(314)	0,0137492	Ccnb2, Cenpf
GO:0007051: spindle organization	2(314)	0,0163194	Kif11, Pcnt
GO:0007090: regulation of S phase of mitotic cell cycle	2(314)	0,0163194	Dbf4, Cdt1
GO:0007052: mitotic spindle organization	2(314)	0,0163194	Ndc80, Stmn1
GO:0045070: positive regulation of viral genome replication	2(314)	0,0163194	Srpk1, Top2a
GO:0000079: regulation of cyclin-dependent protein kinase activity	3(314)	0,0173269	Ccnb2, Ccna2, Cdkn2c
GO:0000084: S phase of mitotic cell cycle	2(314)	0,0234741	Pold1, Ezh2
GO:0000278: mitotic cell cycle	2(314)	0,0278102	Usp3, Cenpf
GO:0071777: positive regulation of cell cycle cytokinesis	1(314)	0,038985	Cdc6
GO:0051310: metaphase plate congression	1(314)	0,038985	Cenpf
GO:0033314: mitotic cell cycle DNA replication checkpoint	1(314)	0,038985	Clspn
GO:0045870: positive regulation of retroviral genome replication	1(314)	0,038985	Top2a
GO:0033260: DNA replication involved in S phase	1(314)	0,038985	Slbp
GO:0045841: negative regulation of mitotic metaphase/anaphase transition	1(314)	0,038985	Mad2l1
GO:0045769: negative regulation of asymmetric cell division	1(314)	0,038985	Aspm
GO:0007063: regulation of sister chromatid cohesion	1(314)	0,038985	Bub1
GO:0007100: mitotic centrosome separation	1(314)	0,038985	Kif11
GO:0090307: spindle assembly involved in mitosis	1(314)	0,038985	Kif11
GO:0046601: positive regulation of centriole replication	1(314)	0,038985	Plk4
GO:0000082: G1/S transition of mitotic cell cycle	3(314)	0,0400578	Skp2, Iqgap3, Cdkn2c
GO:0045736: negative regulation of	2(314)	0,0494391	Plk1, Casp3

Functional Category	***Number of Genes***	***Corrected p-value***	***Genes repressed in Table S4***
**cyclin-dependent protein kinase activity**			
**DNA AND RNA PROCESSING**			
**GO:0006260: DNA replication**	32(314)	7,49E-35	Mcm5, Pole2, Rbbp7, Dscc1, Mcm4, Prim1, Sin3a, Brca1, Mcm3, Gins1, Dtl, Supt16h, Cdc25c, Lig1, Rbbp4, Nasp, Chek1, Cdc7, Rpa2, Tk1, Dbf4, Rfc5, Clspn, Chtf18, Mcm7, Cdc6, Mcm2, Ssrp1, Pold1, Mcm6, Rfc4, Cdt1
**GO:0006355: regulation of transcription, DNA-dependent**	54(314)	3,08E-14	Nono, Cdca7l, Timeless, Sirt1, Whsc1, Mybbp1a, Kat2a, Paxip1, Med13l, Zfp57, Dnmt1, Rbbp7, Cdc5l, Ctcf, Sin3a, Hnrnpd, Cand1, Asxl3, Ash2l, Trim28, Supt16h, Six4, Zfp217, Zfp703, Rbbp4, Ewsr1, Sf1, Asf1b, Asxl1, Hmga2, Tead2, Top2a, Patz1, Mlf1ip, Actl6a, Ilf3, Mycn, Ssrp1, E2f7, C79407, Etv4, Whsc2, Apex1, Khdrbs1, Cdca7, Ezh2, Tmpo, Rbl1, Terf1, Uhrf1, Tfam, Smarcc1, Hnrpdl, Pogz
**GO:0008380: RNA splicing**	22(314)	4,38E-14	Nono, Rbmxrt, Cpsf2, Prpf19, Hnrnpc, Srpk1, Cdc5l, Hnrnpu, Rnps1, Hnrnpd, Cstf2, Polr2b, Sf1, Sf3a1, Snw1, Hnrnpa2b1, Snrpg, Nudt21, Gemin8, Syncrip, Zrsr2, Zcchc8
**GO:0006397: mRNA processing**	21(314)	8,88E-13	Nono, Rbmxrt, Cpsf2, Prpf19, Cpsf6, Hnrnpc, Srpk1, Cdc5l, Hnrnpu, Rnps1, Cstf2, Sf1, Sf3a1, Slbp, Snw1, Hnrnpa2b1, Nudt21, Gemin8, Syncrip, Zrsr2, Zcchc8
**GO:0006351: transcription, DNA-dependent**	49(314)	1,73E-12	Nono, Cdca7l, Timeless, Sirt1, Whsc1, Mybbp1a, Kat2a, Paxip1, Med13l, Zfp57, Dnmt1, Rbbp7, Cdc5l, Ctcf, Prim1, Sin3a, Hnrnpd, Cand1, Asxl3, Ash2l, Trim28, Supt16h, Rbbp4, Polr2b, Ewsr1, Sf1, Hmgb1, Asf1b, Asxl1, Hmga2, Hmgb2, Tead2, Mlf1ip, Actl6a, Polr3b, Ilf3, Ssrp1, E2f7, Etv4, Whsc2, Apex1, Khdrbs1, Cdca7, Ezh2, Rbl1, Uhrf1, Tfam, Smarcc1, Hnrpdl
**GO:0006310: DNA recombination**	11(314)	7,33E-11	Nono, Paxip1, Brca1, Exo1, Psmc3ip, Lig1, Recql4, Rpa2, Rad51ap1, Apex1, Rad51
**GO:0006270: DNA-dependent DNA replication initiation**	6(314)	1,10E-08	Mcm5, Mcm4, Mcm3, Mcm7, Mcm2, Mcm6
**GO:0045892: negative regulation of transcription, DNA-dependent**	17(314)	2,47E-08	Timeless, Sirt1, Cbx5, Mybbp1a, Dnmt1, Ctcf, Sin3a, Brca1, Trim28, Zfp703, Cbx3, Snw1, Hmgb2, Cenpf, Patz1, Ilf3, Khdrbs1
**GO:0006268: DNA unwinding involved in replication**	5(314)	4,65E-08	Mcm4, Mcm7, Mcm2, Mcm6, Rad51
**GO:0006164: purine nucleotide biosynthetic process**	4(314)	0,000185328	Impdh2, Mthfd1, Adsl, Gmps
**GO:0007004: telomere maintenance via telomerase**	3(314)	0,0013998	Dkc1, Ptges3, Terf1
**GO:0006261: DNA-dependent DNA replication**	3(314)	0,00222991	Pole2, Top2a, Pold1
**GO:0010216: maintenance of DNA methylation**	2(314)	0,00337246	Dnmt1, Ctcf
**GO:0006177: GMP biosynthetic process**	2(314)	0,00337246	Impdh2, Gmps
**GO:0006306: DNA methylation**	3(314)	0,00453625	Dnmt1, Ctcf, Mbd4
**GO:0009168: purine ribonucleoside monophosphate biosynthetic process**	3(314)	0,00453625	Impdh2, Adsl, Gmps
**GO:0050684: regulation of mRNA processing**	2(314)	0,00552327	Srpk1, Safb
**GO:0051571: positive regulation of histone H3-K4 methylation**	2(314)	0,00552327	Paxip1, Dnmt1
**GO:0000122: negative regulation of transcription from RNA polymerase II promoter**	9(314)	0,00778043	Sirt1, Whsc1, Zfp57, Rbbp7, Sin3a, Trim28, Hmgb1, Hmga2, Rbl1
**GO:0006398: histone mRNA 3'-end processing**	2(314)	0,00797998	Cpsf2, Slbp
**GO:0070934: CRD-mediated mRNA stabilization**	2(314)	0,00797998	Hnrnpu, Syncrip
**GO:0009048: dosage compensation, by inactivation of X chromosome**	2(314)	0,00797998	Ctcf, Brca1
**GO:0006396: RNA processing**	4(314)	0,00946223	Dkc1, Exosc8, Larp7, Sf3a1
**GO:0006266: DNA ligation**	2(314)	0,0105499	Lig1, Top2a
**GO:0006259: DNA metabolic process**	3(314)	0,0106837	Pttg1, Top2a, Rad51
**GO:0000398: nuclear mRNA splicing, via spliceosome**	3(314)	0,0154716	Sf3a1, Snw1, Zrsr2
**GO:0043488: regulation of mRNA**	2(314)	0,0202844	Hnrnpd, Apex1

Functional Category	*Number of Genes*	*Corrected p-value*	*Genes repressed in Table S4*
**stability**			
**GO:0045893: positive regulation of transcription, DNA-dependent**	9(314)	0,0234362	Ctcf, Trim28, Six4, Ccna2, Hmgb2, Ilf3, Etv4, Tfam, Smarcc1
**GO:0006349: regulation of gene expression by genetic imprinting**	2(314)	0,0278102	Zfp57, Ctcf
**GO:0016578: histone deubiquitination**	2(314)	0,0308515	Kat2a, Usp3
**GO:0016180: snRNA processing**	2(314)	0,0308515	Ints5, Ints7
**GO:0051106: positive regulation of DNA ligation**	1(314)	0,038985	Rad51
**GO:0006348: chromatin silencing at telomere**	1(314)	0,038985	Hat1
**GO:0009152: purine ribonucleotide biosynthetic process**	1(314)	0,038985	Adsl
**GO:0046104: thymidine metabolic process**	1(314)	0,038985	Tk1
**GO:0032075: positive regulation of nuclease activity**	1(314)	0,038985	Hmgb2
**GO:0000105: histidine biosynthetic process**	1(314)	0,038985	Mthfd1
**GO:0031060: regulation of histone methylation**	1(314)	0,038985	Ctcf
**GO:0000416: positive regulation of histone H3-K36 methylation**	1(314)	0,038985	Paxip1
**GO:0031937: positive regulation of chromatin silencing**	1(314)	0,038985	Sirt1
**GO:0006343: establishment of chromatin silencing**	1(314)	0,038985	Sirt1
**GO:0006378: mRNA polyadenylation**	2(314)	0,0395876	Cpsf6, Nudt21
**GO:0051568: histone H3-K4 methylation**	2(314)	0,0395876	Paxip1, Ash2l
**GO:0032259: methylation**	5(314)	0,0397787	Whsc1, Dnmt1, Tyw3, Emg1, Ezh2
**GO:0045944: positive regulation of transcription from RNA polymerase II promoter**	10(314)	0,0401959	Psmc3ip, Six4, Hmgb1, Snw1, Hmgb2, Tead2, Top2a, Rbm14, Cenpk, Smarcc1
**GO:0006364: rRNA processing**	4(314)	0,0419854	Sirt1, Dkc1, Exosc8, Emg1
**GO:0043388: positive regulation of DNA binding**	2(314)	0,0447025	Hmgb1, Hmgb2
**GO:0008156: negative regulation of DNA replication**	2(314)	0,0491857	Hmgb1, Terf1
**GO:0016575: histone deacetylation**	2(314)	0,0494391	Sirt1, Rbm14
**METABOLITE PROCESSING (PROTEIN, CARBOHYDRATE AND LIPID PROCESSING)**			
**GO:0006468: protein phosphorylation**	22(314)	1,36E-07	Prkar2b, Plk4, Plk1, Aurkb, Srpk1, Bub1, Ptpre, Vrk3, Cit, Trim28, Gsg2, Chek1, Cdc7, Prkd1, Bub1b, Melk, Prkg2, Mastl, Ttk, Pkmyt1, Abl2, Nek2
**GO:0016310: phosphorylation**	21(314)	1,46E-05	Skp2, Plk4, Plk1, Srpk1, Bub1, Cit, Gsg2, Chek1, Cdc7, Bub1b, Tk1, Dbf4, Melk, Mastl, Cdkn2c, Ttk, Pkmyt1, Akap8, Abl2, Nek2, Cks2
**GO:0009060: aerobic respiration**	2(314)	0,0105499	Fxn, Adsl
**GO:0046855: inositol phosphate dephosphorylation**	2(314)	0,0105499	Synj2, Impa1
**GO:0042325: regulation of phosphorylation**	3(314)	0,0131104	Prkar2b, Cd24a, Mcm7
**GO:0006020: inositol metabolic process**	2(314)	0,0137492	Slc5a3, Impa1
**GO:0051353: positive regulation of oxidoreductase activity**	2(314)	0,0163194	Fxn, Abl2
**GO:0010722: regulation of ferrochelatase activity**	1(314)	0,038985	Fxn
**GO:0016567: protein ubiquitination**	5(314)	0,0406218	Rfwd3, Plk1, Prpf19, Brca1, Cand1
**GO:0000209: protein polyubiquitination**	3(314)	0,0446759	Skp2, Prpf19, Dtl
**DNA DAMAGE RESPONSE**			
**GO:0006974: response to DNA damage stimulus**	36(314)	4,90E-29	Nono, Timeless, Sirt1, Rfwd3, Prpf19, Paxip1, Msh6, Casp3, Brca1, Rad21, Usp1, Ash2l, Exo1, Dtl, Supt16h, Lig1, Rad54l, Chek1, Pttg1, Usp3, Top2a,

Functional Category	***Number of Genes***	***Corrected p-value***	***Genes repressed in Table S4***
			Rpa2, Mbd4, Clspn, Tipin, Apitd1, Mcm7, Topbp1, Rad51ap1, Ssrp1, Fancd2, Alkbh1, Gen1, Apex1, Rad51, Uhrf1
**GO:0006281: DNA repair**	29(314)	9,92E-23	Nono, Rfwd3, Prpf19, Paxip1, Msh6, Brca1, Rad21, Usp1, Exo1, Supt16h, Lig1, Rad54l, Chek1, Pttg1, Usp3, Rpa2, Mbd4, Clspn, Kif22, Apitd1, Topbp1, Rad51ap1, Ssrp1, Fancd2, Alkbh1, Gen1, Apex1, Rad51, Uhrf1
**GO:0006297: nucleotide-excision repair, DNA gap filling**	6(314)	3,96E-07	Pole2, Lig1, Rpa2, Rfc5, Pold1, Rfc4
**GO:0031572: G2/M transition DNA damage checkpoint**	5(314)	4,65E-06	Plk1, Brca1, Dtl, Chek1, Clspn
**GO:0010212: response to ionizing radiation**	5(314)	0,000163094	Rfwd3, Paxip1, Brca1, Rad54l, Topbp1
**GO:0009411: response to UV**	5(314)	0,000232741	Msh6, Casp3, Usp1, Dtl, Tipin
**GO:0050918: positive chemotaxis**	4(314)	0,000429187	Scrib, Hmgb2, Fgf7, Alkbh1
**GO:0006288: base-excision repair, DNA ligation**	2(314)	0,00134703	Hmgb1, Hmgb2
**GO:0045739: positive regulation of DNA repair**	3(314)	0,00915821	Sirt1, Brca1, Apex1
**GO:0000724: double-strand break repair via homologous recombination**	3(314)	0,0141938	Brca1, Rad54l, Rad51
**GO:0043627: response to estrogen stimulus**	3(314)	0,0222209	Brca1, Ash2l, Cd24a
**GO:0070301: cellular response to hydrogen peroxide**	2(314)	0,0234741	Fxn, Apex1
**GO:0006298: mismatch repair**	2(314)	0,0308515	Msh6, Exo1
**GO:0048545: response to steroid hormone stimulus**	2(314)	0,0308515	Hmgb2, Spp1
**GO:0006302: double-strand break repair**	3(314)	0,0322914	Brca1, Trip13, Rad54l
**GO:0042060: wound healing**	3(314)	0,0370525	Scrib, Vangl2, Fgf7
**GO:0050831: male-specific defense response to bacterium**	1(314)	0,038985	Hmgb1
**GO:0006282: regulation of DNA repair**	1(314)	0,038985	Usp1
**GO:0070370: cellular heat acclimation**	1(314)	0,038985	Rbbp7
**GO:0000077: DNA damage checkpoint**	2(314)	0,0441243	Chek1, Tipin
**CYTOSKELETON-REGULATED PROCESSES**			
**GO:0016568: chromatin modification**	15(314)	4,81E-08	Whsc1, Dnmt1, Rbbp7, Ctcf, Ash2l, Rbbp4, Gsg2, Asf1b, Asxl1, Usp3, Hat1, Actl6a, Ezh2, Rbl1, Smarcc1
**GO:0030261: chromosome condensation**	4(314)	0,000230568	Ncaph, Ncapd2, Ncapd3, Top2a
**GO:0007018: microtubule-based movement**	6(314)	0,000627124	Kif4, Kif11, Kif20a, Kif2c, Cenpe, Kif22
**GO:0006334: nucleosome assembly**	5(314)	0,00140607	H2afv, Asf1b, Cenpa, Mcm2, H2afz
**GO:0051382: kinetochore assembly**	2(314)	0,00337246	Cenpa, Pogz
**GO:0006999: nuclear pore organization**	2(314)	0,00337246	Pom121, Nup133
**GO:0006338: chromatin remodeling**	4(314)	0,00475204	Rbbp7, Rbbp4, Actl6a, Smarcc1
**GO:0051262: protein tetramerization**	3(314)	0,00784636	Cpsf6, Adsl, Nudt21
**GO:0051276: chromosome organization**	3(314)	0,00806018	Rad54l, Pttg1, Cdca8
**GO:0007019: microtubule depolymerization**	2(314)	0,0105499	Kif2c, Stmn1
**GO:0031532: actin cytoskeleton reorganization**	3(314)	0,0131104	Hmgb1, Fgf7, Eps8
**GO:0000245: spliceosome assembly**	2(314)	0,0202844	Prpf19, Zrsr2
**GO:0033108: mitochondrial respiratory chain complex assembly**	1(314)	0,038985	Tfam
**GO:0043486: histone exchange**	1(314)	0,038985	Nasp
**GO:0030866: cortical actin cytoskeleton organization**	2(314)	0,0395876	Llgl1, Arf6
**CELL PROLIFERATION AND GROWTH**			

Functional Category	*Number of Genes*	*Corrected p-value*	*Genes repressed in Table S4*
**GO:0008283: cell proliferation**	9(314)	0,000185875	Prkar2b, Kat2a, Mki67, Scrib, Nasp, Ptges3, Mcm7, Tacc3, Uhrf1
**GO:0008284: positive regulation of cell proliferation**	11(314)	0,00202872	Cdca7l, Fxn, Ash2l, Zfp703, Recql4, Hmgb1, Cdc7, Fgf7, Cdc20, Tipin, Mycn
**GO:0033601: positive regulation of mammary gland epithelial cell proliferation**	2(314)	0,00797998	Iqgap3, Zfp703
**GO:0001833: inner cell mass cell proliferation**	2(314)	0,0308515	Prpf19, Gins1
**GO:0030889: negative regulation of B cell proliferation**	2(314)	0,0308515	Casp3, Cd24a
**GO:0046014: negative regulation of T cell homeostatic proliferation**	1(314)	0,038985	Cd24a
**GO:0042103: positive regulation of T cell homeostatic proliferation**	1(314)	0,038985	Cd24a
**TRANSPORT (METABOLITES, IONS AND VESICLES)**			
**GO:0051028: mRNA transport**	6(314)	0,000376719	Nup54, Pom121, Nup107, Slbp, Nup133, Nup43
**GO:0048488: synaptic vesicle endocytosis**	2(314)	0,0234741	Scrib, Cd24a
**GO:0032597: B cell receptor transport into membrane raft**	1(314)	0,038985	Cd24a
**GO:0032600: chemokine receptor transport out of membrane raft**	1(314)	0,038985	Cd24a
**GO:0046831: regulation of RNA export from nucleus**	1(314)	0,038985	Khdrbs1
**GO:0015031: protein transport**	10(314)	0,0447186	Nup54, Pom121, Kif20a, Nasp, Nup107, Kpnb1, Cenpf, Nup133, Nup43, Arf6
**DEVELOPMENTAL PROCESS**			
**GO:0001843: neural tube closure**	5(314)	0,00195095	Kat2a, Scrib, Vangl2, Tead2, Stil
**GO:0003149: membranous septum morphogenesis**	2(314)	0,00797998	Whsc1, Vangl2
**GO:0045665: negative regulation of neuron differentiation**	4(314)	0,00995345	Prpf19, Aspm, Cit, Cd24a
**GO:0048538: thymus development**	3(314)	0,0131104	Ccnb2, Six4, Bcl2l11
**GO:0007399: nervous system development**	8(314)	0,015502	Kat2a, Nrp1, Cit, Hmgb1, Hmgb2, Actl6a, Stmn1, Smarcc1
**GO:0016446: somatic hypermutation of immunoglobulin genes**	2(314)	0,0163194	Msh6, Exo1
**GO:0048699: generation of neurons**	2(314)	0,0163194	Cit, Six4
**GO:0001701: in utero embryonic development**	7(314)	0,0228412	Kat2a, Sin3a, Ccnb2, Pcnt, Alkbh1, Stil, Bcl2l11
**GO:0048339: paraxial mesoderm development**	2(314)	0,0234741	Tead2, Nup133
**GO:0030903: notochord development**	2(314)	0,0234741	Tead2, Stil
**GO:0007275: multicellular organismal development**	16(314)	0,027824	Timeless, Sirt1, Spred2, Zfp57, Nrp1, Cit, Six4, Scrib, Vangl2, Plxnb1, Cenpe, H2afz, Stmn1, Fancd2, Stil, Uhrf1
**GO:0008584: male gonad development**	3(314)	0,0325403	Hmgb2, Patz1, Bcl2l11
**GO:0060998: regulation of dendritic spine development**	1(314)	0,038985	Arf6
**GO:0051154: negative regulation of striated muscle cell differentiation**	1(314)	0,038985	Ezh2
**GO:0045605: negative regulation of epidermal cell differentiation**	1(314)	0,038985	Ezh2
**GO:0035148: tube formation**	1(314)	0,038985	Bcl2l11
**GO:0003402: planar cell polarity pathway involved in axis elongation**	1(314)	0,038985	Vangl2
**GO:0060420: regulation of heart growth**	1(314)	0,038985	Dusp6
**GO:0060717: chorion development**	1(314)	0,038985	Paxip1
**GO:0003290: atrial septum secundum morphogenesis**	1(314)	0,038985	Whsc1
**GO:0003289: atrial septum primum**	1(314)	0,038985	Whsc1

Functional Category	*Number of Genes*	*Corrected p-value*	*Genes repressed in Table S4*
**morphogenesis**			
**GO:0030324: lung development**	4(314)	0,039477	Timeless, Hmgb1, Fgf7, Mycn
**CELL DEATH**			
**GO:0008630: DNA damage response, signal transduction resulting in induction of apoptosis**	3(314)	0,00383482	Msh6, Brca1, Mbd4
**GO:0006915: apoptosis**	13(314)	0,00780888	Sirt1, Bub1, Casp3, Tmem173, Ect2, Rad21, Gramd4, Casp2, Pcnt, Bub1b, Bcl2l11, Arf6, 1810011O10Rik
**GO:0008624: induction of apoptosis by extracellular signals**	5(314)	0,00971646	Casp3, Ect2, Casp2, Cd24a, Bcl2l11
**GO:0043065: positive regulation of apoptosis**	7(314)	0,0152979	Casp3, Casp2, Scrib, Dusp6, Hmgb1, Top2a, Bcl2l11
**GO:0043525: positive regulation of neuron apoptosis**	3(314)	0,0280321	Casp3, Casp2, Bcl2l11
**GO:0010942: positive regulation of cell death**	2(314)	0,0447025	Hmgb1, Mycn
**GO:0043066: negative regulation of apoptosis**	7(314)	0,0449741	Mad2l1, Fxn, Casp3, Sin3a, Casp2, Stil, Fignl1
**SIGNALING**			
**GO:0070373: negative regulation of ERK1 and ERK2 cascade**	2(314)	0,0395876	Vrk3, Dusp6
**GO:0000188: inactivation of MAPK activity**	2(314)	0,0447025	Spred2, Dusp6
**OTHER**			
**GO:0034501: protein localization to kinetochore**	3(314)	0,000309679	Aurkb, Ccdc99, Bub1b
**GO:0043029: T cell homeostasis**	3(314)	0,00577017	Casp3, Ccnb2, Bcl2l11
**GO:0071168: protein localization to chromatin**	2(314)	0,00337246	Plk1, Rad21
**GO:0045190: isotype switching**	2(314)	0,0308515	Msh6, Exo1
**GO:0045830: positive regulation of isotype switching**	1(314)	0,038985	Paxip1
**GO:0001309: age-dependent telomere shortening**	1(314)	0,038985	Terf1
**GO:0032913: negative regulation of transforming growth factor-beta3 production**	1(314)	0,038985	Cd24a
**GO:0034107: negative regulation of erythrocyte clearance**	1(314)	0,038985	Cd24a
**GO:0034119: negative regulation of erythrocyte aggregation**	1(314)	0,038985	Cd24a
**GO:2000145: regulation of cell motility**	1(314)	0,038985	Brca1
**GO:0033003: regulation of mast cell activation**	1(314)	0,038985	Ptpre
**GO:0035090: maintenance of apical/basal cell polarity**	1(314)	0,038985	Llgl1
**GO:0001782: B cell homeostasis**	2(314)	0,0441243	Casp3, Bcl2l11

S5B. Functional annotation of induced genes from Rasless MEFs that are repressed in both BRAF- and MEK1-rescued MEFs

Functional Category	*Number of Genes*	*Corrected p-value*	*Genes upregulated in Table S4*
**TRANSPORT (METABOLITES, IONS AND VESICLES)**			
**GO:0006810: transport**	17(86)	4,83E-05	Slc6a6, Tmco3, Slc31a2, Stx8, Ap3m2, Kpna1, Slc9a6, Exoc3, Dynlt3, Rab2b, Slc26a11, Slco3a1, Rab7l1, Slc1a4, Vamp3, Atp6v0a1, Vps29
**GO:0016192: vesicle-mediated transport**	5(86)	0,00443869	Stx8, Ap3m2, Rab2b, Vamp3, Stx7
**GO:0015031: protein transport**	7(86)	0,00900847	Ap3m2, Kpna1, Exoc3, Rab2b, Rab7l1, Vamp3, Vps29
**GO:0006906: vesicle fusion**	2(86)	0,0136743	Stx8, Stx7
**GO:0006896: Golgi to vacuole transport**	1(86)	0,0202459	Vps29
**GO:0034589: hydroxyproline transport**	1(86)	0,0202459	Slc1a4
**GO:0015826: threonine transport**	1(86)	0,0202459	Slc1a4
**GO:0001762: beta-alanine transport**	1(86)	0,0202459	Slc6a6
**GO:0015734: taurine transport**	1(86)	0,0202459	Slc6a6
**GO:0008333: endosome to lysosome transport**	2(86)	0,0309299	Stx8, Stx7
**GO:0015811: L-cystine transport**	1(86)	0,0312005	Slc1a4
**GO:0006811: ion transport**	6(86)	0,0346205	Tmco3, Slc31a2, Slc9a6, Slc26a11, Slco3a1, Atp6v0a1
**GO:0015732: prostaglandin transport**	1(86)	0,0355682	Slco3a1
**GO:0015825: L-serine transport**	1(86)	0,0355682	Slc1a4
**GO:0006886: intracellular protein transport**	3(86)	0,0360536	Ap3m2, Kpna1, Stx7
**GO:0003333: amino acid transmembrane transport**	2(86)	0,0365263	Slc6a6, Slc1a4
**GO:0006865: amino acid transport**	2(86)	0,0368832	Slc6a6, Slc1a4
**GO:0015808: L-alanine transport**	1(86)	0,0403518	Slc1a4
**GO:0035524: proline transmembrane transport**	1(86)	0,0453434	Slc1a4
**GO:0035434: copper ion transmembrane transport**	1(86)	0,0453434	Slc31a2
**GO:0015824: proline transport**	1(86)	0,0486711	Slc1a4
**METABOLITE PROCESSING (PROTEIN, CARBOHYDRATE AND LIPID PROCESSING)**			
**GO:0006657: CDP-choline pathway**	2(86)	0,00377501	Pcyt1a, Chpt1
**GO:0008654: phospholipid biosynthetic process**	3(86)	0,0110616	Pcyt1a, Abhd5, Chpt1
**GO:0006656: phosphatidylcholine biosynthetic process**	2(86)	0,012102	Pcyt1a, Chpt1
**GO:0043438: acetoacetic acid metabolic process**	1(86)	0,0202459	Tyrp1
**GO:0070178: D-serine metabolic process**	1(86)	0,0202459	Srr
**GO:0070179: D-serine biosynthetic process**	1(86)	0,0202459	Srr
**GO:0009069: serine family amino acid metabolic process**	1(86)	0,0202459	Srr
**GO:0006583: melanin biosynthetic process from tyrosine**	1(86)	0,0312005	Tyrp1
**GO:0015939: pantothenate metabolic process**	1(86)	0,0312005	Vnn1
**GO:0045964: positive regulation of dopamine metabolic process**	1(86)	0,0312005	Maob
**GO:0001933: negative regulation of protein phosphorylation**	2(86)	0,0327589	Prnp, Impact

Functional Category	*Number of Genes*	*Corrected p-value*	*Genes upregulated in Table S4*
**GO:0006695: cholesterol biosynthetic process**	2(86)	0,0329598	Insig2, Cyb5r3
**GO:0016126: sterol biosynthetic process**	2(86)	0,0329598	Insig2, Cyb5r3
**GO:0006663: platelet activating factor biosynthetic process**	1(86)	0,0355682	Chpt1
**GO:0046068: cGMP metabolic process**	1(86)	0,0355682	Rora
**GO:0006582: melanin metabolic process**	1(86)	0,0355682	Tyrp1
**GO:0051084: 'de novo' posttranslational protein folding**	1(86)	0,0355682	Entpd5
**GO:0010894: negative regulation of steroid biosynthetic process**	1(86)	0,0403518	Insig2
**GO:0071569: protein ufmylation**	1(86)	0,0403518	Ufm1
**GO:0006563: L-serine metabolic process**	1(86)	0,0453434	Srr
**GO:0042866: pyruvate biosynthetic process**	1(86)	0,0453434	Srr
**GO:0006654: phosphatidic acid biosynthetic process**	1(86)	0,0486711	Abhd5
**GO:0050435: beta-amyloid metabolic process**	1(86)	0,0486711	Bace1
**GO:0010898: positive regulation of triglyceride catabolic process**	1(86)	0,0486711	Abhd5
**CELL DEATH**			
**GO:0006916: anti-apoptosis**	4(86)	0,0130928	Birc7, Prnp, Vnn1, Tsc22d3
**GO:0048102: autophagic cell death**	1(86)	0,0403518	Acp2
**CYTOSKELETON-REGULATED PROCESSES**			
**GO:0016584: nucleosome positioning**	1(86)	0,0486711	Hist1h1e
**GO:0045103: intermediate filament-based process**	1(86)	0,0355682	Sync
**DEVELOPMENTAL PROCESS**			
**GO:0060668: regulation of branching involved in salivary gland morphogenesis by extracellular matrix-epithelial cell signaling**	1(86)	0,0202459	Ntn4
**GO:0010842: retina layer formation**	1(86)	0,0355682	Ptprm
**GO:0048642: negative regulation of skeletal muscle tissue development**	1(86)	0,0355682	Tsc22d3
**GO:0060363: cranial suture morphogenesis**	1(86)	0,0453434	Insig2
**GO:0035117: embryonic arm morphogenesis**	1(86)	0,0453434	Wnt9a
**SIGNALING**			
**GO:0070885: negative regulation of calcineurin-NFAT signaling pathway**	1(86)	0,0202459	Prnp
**GO:0014066: regulation of phosphatidylinositol 3-kinase cascade**	1(86)	0,0312005	Entpd5
**GO:0007264: small GTPase mediated signal transduction**	4(86)	0,033574	Rasl12, Rab2b, Rab7l1, Rit1
**GO:0014063: negative regulation of serotonin secretion**	1(86)	0,0355682	Maob
**GO:0001921: positive regulation of**	1(86)	0,0486711	Vamp3

Functional Category	*Number of Genes*	*Corrected p-value*	*Genes upregulated in Table S4*
receptor recycling			
<u>CELL PROLIFERATION AND GROWTH</u>			
**GO:0046007: negative regulation of activated T cell proliferation**	1(86)	0,0403518	Prnp
<u>**OTHER**</u>			
**GO:0002544: chronic inflammatory response**	1(86)	0,0202459	Vnn1
**GO:0009628: response to abiotic stimulus**	1(86)	0,0202459	Pcyt1a
**GO:0030967: ER-nuclear sterol response pathway**	1(86)	0,0312005	Insig2
**GO:0006991: response to sterol depletion**	1(86)	0,0312005	Insig2
**GO:0043030: regulation of macrophage activation**	1(86)	0,0403518	Rora
**GO:0010891: negative regulation of sequestering of triglyceride**	1(86)	0,0403518	Abhd5
**GO:0002526: acute inflammatory response**	1(86)	0,0486711	Vnn1
