# Supplementary figures and images for: Reversible, interrelated mRNA and miRNA expression patterns in the transcriptome of Rasless fibroblasts: functional and mechanistic implications
Source: BMC Genomics. 2013 Oct 25;14:731. doi: 10.1186/1471-2164-14-731 (PMC4007593; doi:10.1186/1471-2164-14-731)

**A**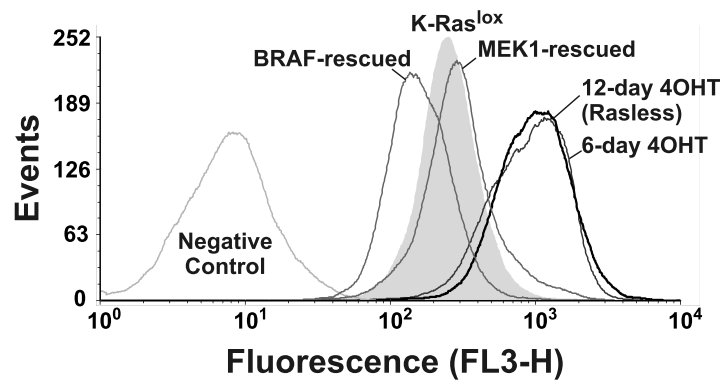**B**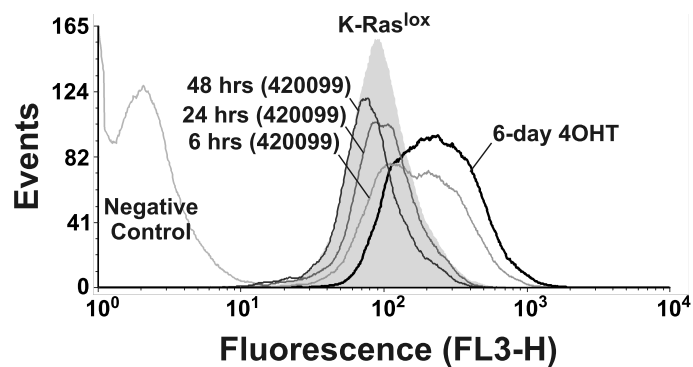**C**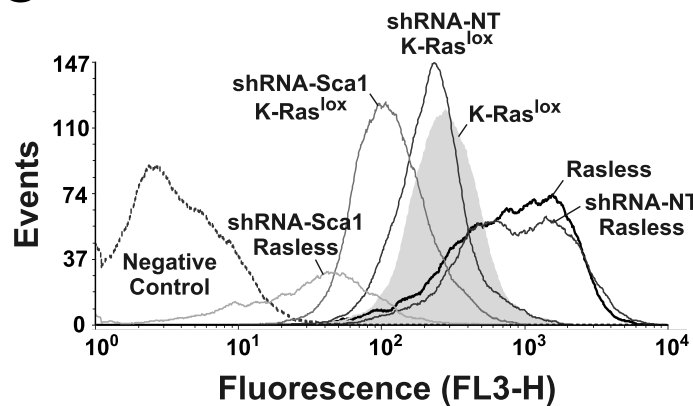**D**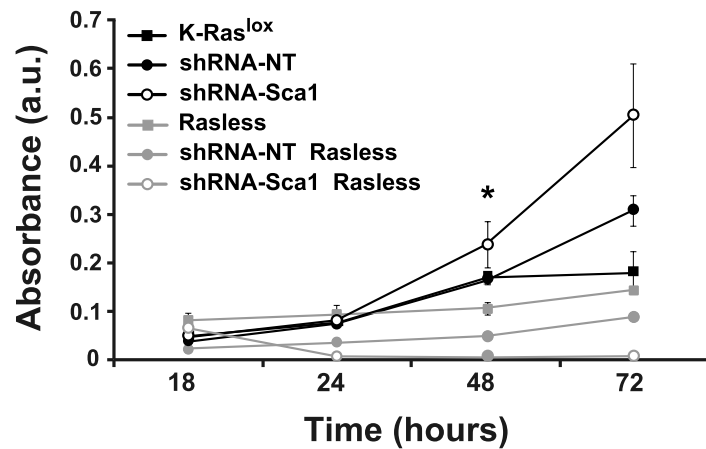**E**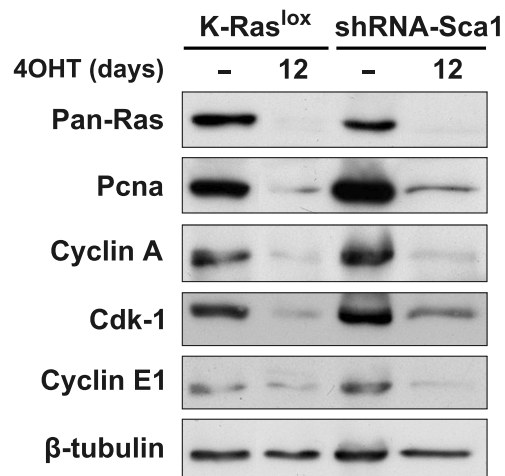

Supplement: Additional file 6: Figure S1 — Alterations of Sca1 expression in Rasless fibroblasts. (A) Flow cytometric analysis of Sca1 (Ly6A) protein expression using specific antibodies in K-Raslox MEFs before (solid grey profile) and after 6 days or 12 days of 4OHT treatment to render them Rasless, as well as in BRAF-rescued and MEK1-rescued MEFs. As a control, Sca1 protein expression in two constitutive double-knockout (H-Ras-/-; N-Ras-/-) MEF cell lines (A624-6 and A624-8) did not show any change after similar treatment with 4OHT for 9 or 16 days, indicating that increased Sca1 expression is not an off-target effect of 4OHT treatment (not shown). (B) Reduced Sca1 protein expression as a result of incubating 6-day 4OHT-treated K-Raslox MEFs with Jak inhibitor I (420099, Millipore) for the times indicated (6, 24 and 48 hours). K-Raslox MEFs treated with either DMSO or Jak inhibitor I showed a similar Sca1 expression to the control untreated K-Raslox MEFs (not shown). (C) Stable knockdown of Sca1 expression by specific shRNA-Sca1 constructs introduced into K-Raslox MEFs and Rasless cells (generated after 16- and 22-day 4OHT–treatment). As a control, stable integration of a non-targeting shRNA construct (shRNA-NT) did not cause any detectable changes in Sca1 expression in the same cell lines. (D) MTT proliferation assays of cultures of control K-Raslox and Rasless MEFs transduced with shRNA-NT and shRNA-Sca1 constructs. * p < 0.05 (shRNA-Sca1 vs K-Raslox). (E) Immunoblot assays of several cell cycle-related proteins in control, untreated K-Raslox MEFs and the same K-Raslox cells knocked down by means of a shRNA-Sca1 construct, before or after a 12-day 4OHT treatment to render them Rasless. [file 1471-2164-14-731-S6.pdf]

# A

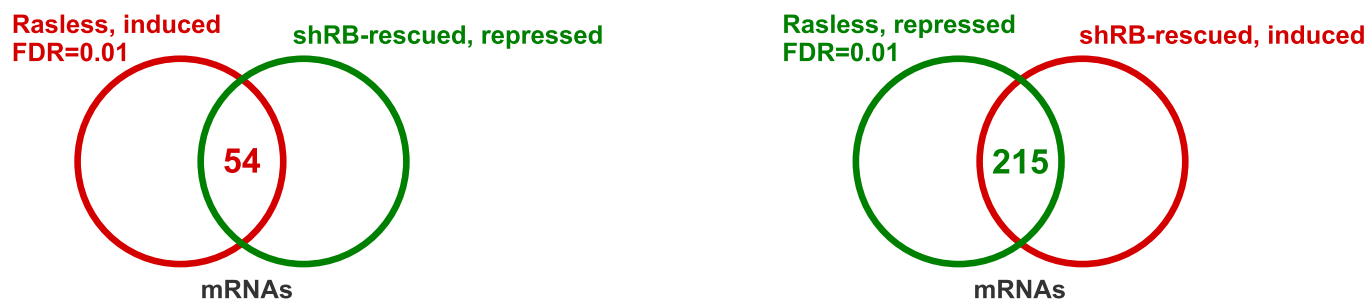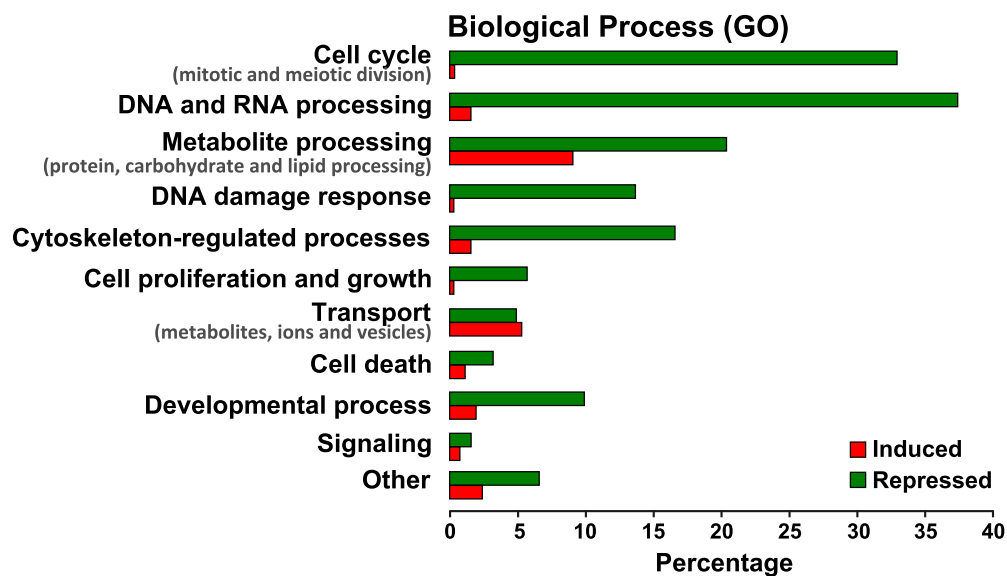

# B

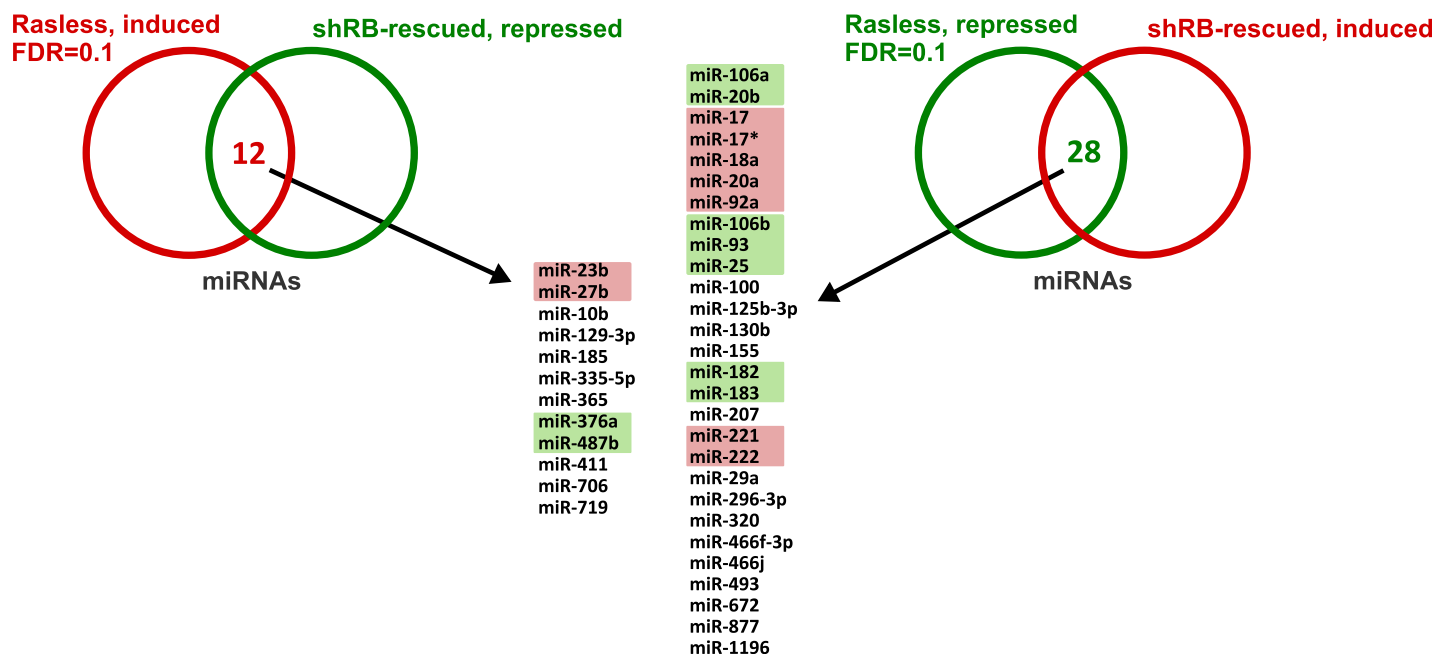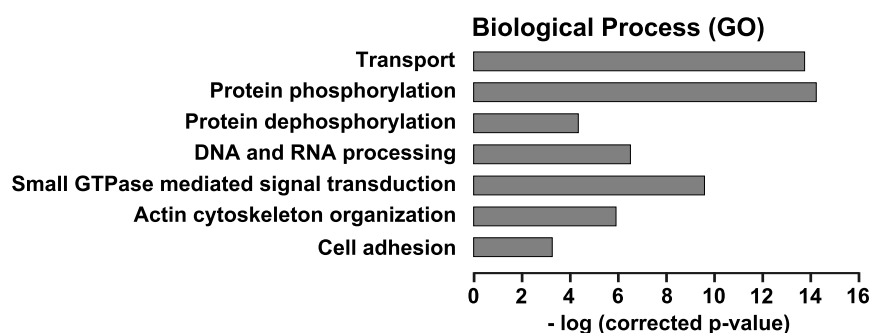

Supplement: Additional file 7: Figure S2 — Reversal of the mRNA and microRNA expression profiles of Rasless cells by RB silencing. (A) Differentially expressed mRNAs in Rasless MEFs showing the opposite pattern of expression in shRB-rescued cells. Venn diagrams showing numbers of shared, differentially expressed mRNAs that were simultaneously detected as induced (54 genes, left panel) or repressed (215 genes, right panel) in Rasless MEFs (pair-wise comparison with control MEFs, FDR = 0.01) and as repressed (left panel) or induced (right panel), respectively, in shRB-rescued MEFs (pair-wise comparisons with Rasless MEFs, FDR = 0.03); Diagrams generated using the Venny application. Red: transcriptional induction. Green: transcriptional repression. Histogram bars represent the functional enrichment of GO Biological Process categories linked to the list of induced (54) and repressed (215) genes identified in the upper Venn diagrams. The GeneCodis (Gene Annotation Co-occurrence Discovery) functional annotation tool was used to identify specific gene subsets within the list of 269 differentially expressed, induced or repressed genes that shared co-occurrent functional annotations linking them, with high statistical significance, to particular Biological Procesess. Green bars: repressed loci. Red bars: induced loci. (B) Differentially expressed microRNAs in Rasless MEFs showing the opposite pattern of expression in shRB-rescued cells. Venn diagrams showing the numbers of shared, differentially expressed miRNAs that were simultaneously detected as induced (12 miRNAs, left panel) or repressed (28 miRNAs, right panel) in Rasless MEFs (pair-wise comparison with control K-Raslox MEFs, FDR = 0.1) and as repressed (left panel) or induced (right panel), respectively, in shRB-rescued MEFs (pair-wise comparisons with Rasless MEFs, FDR ≤ 0,17); Diagrams generated using the Venny application software as indicated. Red: transcriptional induction. Green: transcriptional repression. Functional enrichment analysis of the list of 4 [file 1471-2164-14-731-S7.pdf]
